# Long-term blast control in high eating quality rice using multilines

**DOI:** 10.1038/s41598-022-19237-x

**Published:** 2022-09-01

**Authors:** Kouji Ishikawa, Tomohisa Kuroda, Takeshi Hori, Daisuke Iwata, Seijiro Matsuzawa, Jun Nakabayashi, Akira Sasaki, Taketo Ashizawa

**Affiliations:** 1 Niigata Agricultural Research Institute, Crop Research Center, Nagaoka, 940-0826 Japan; 2 Tokyo Medical and Dental University, Bunkyo-ku, 113-8510 Japan; 3 The Graduate University for Advanced Studies, Hayama, 240-0193 Japan; 4 National Agriculture and Food Research Organization (NARO), Tsukuba, 305-8666 Japan

**Keywords:** Ecology, Evolution, Microbiology

## Abstract

Combining genetic heterogeneity and crop homogeneity serves a dual purpose: disease control and maintaining harvest quality. Multilines, which consist of a genetically uniform mixture of plants, have the potential to suppress disease while maintaining eating quality, yet practical methods that facilitate commercial use over large geographical areas are lacking. Here, we describe effective rice multiline management based on seed mixture composition changes informed by monitoring virulent blast races in Niigata Prefecture, Japan. The most elite nonglutinous cultivar, Koshihikari, was converted into the multiline, Koshihikari BL (blast resistant lines) and planted on 94,000 ha in 2005. The most destructive rice disease, blast, was 79.4% and 81.8% less severe in leaves and panicles, respectively, during the 2005–2019 period compared to the year 2004. In addition, fungicidal application was reduced by two-thirds after the introduction of BL. Our results suggest that seed mixture diversification and rotation of resistant BL provides long-term disease control by avoiding virulent race evolution.

## Introduction

Blast caused by the fungus *Pyricularia oryzae* Cavara is the most devastating disease in rice (*Oryza sativa* L.). Rice is a staple cereal crop for over half the world’s population^1^. Blast infects rice seedlings in nurseries, and seedling blast leads to leaf blast in paddy fields. This results in panicle blast that causes the seed to become infected during maturation. The infected carrier seeds act as primary infection sources in its life cycle. *P*. *oryzae* avirulence in relation to race-specific rice resistance follows a gene-for-gene coevolution pathway^2–7^ between pathogenic variations and host genotypes. This arms race between rice resistance and the blast race can cause dramatic changes in newly emerged virulent races within a few years when monogenic (controlled by a single gene) resistant rice is introduced in paddy fields. This breakdown of resistance has been occurred in rice-blast interaction^6^.

Multilines^8–10^, which consist of a genetically uniform mixture of monogenic crops, are an environmentally friendly strategy for stable food production^11,12^. The heterogenic theory^8^ offers pathogen population dilution^11^ with fewer fungicidal applications for sustainable rice culture. However, breeding characteristically uniform rice lines and understanding blast population dynamics over large geographical areas are administrative burdens. The use of genetically heterogeneous genotypes or cultivar mixtures has been shown in commercial fields in cereals^13–16^. However, the mixture of heterogeneous seeds with identical characteristics equivalent to monogenic cultivars has not been introduced until now and is required to maintain eating quality^17–19^ for consumers and farmers under commercial crop production. To solve this problem, a multiline variety with a high eating quality, Koshihikari BL (KO-BL)^20^, was introduced in 2005 in Niigata Prefecture (Japan). In this study, we demonstrate that a 15-year history of composition change in KO-BL by monitoring of blast race changes. In addition, we believe that our simulation analysis of blast race dynamics in multiline varieties can support the determination of composition changes in KO-BL in the future.

## Results and discussion

The top-brand nonglutinous rice variety ‘Koshihikari’, which has a high palatability, is extremely susceptible to blast. Therefore, farmers apply fungicides over four times during the rice production season. As Koshihikari is sold by the Niigata brand, it has been traditionally viewed as having a high eating quality in Japan, and because of this, both farmers and consumers have requested that the multiline variety KO-BL be tested to determine if it is equivalent to Koshihikari before its introduction. Trials comparing Koshihikari and KO-BL were carried out in 2003 and 2004 in 594 and 622 fields covering 236 and 315 ha, respectively. These trials evaluated plant homogeneity, eating quality, and blast suppression using fewer fungicidal sprays. Following favorable results, in 2005, all Koshihikari were converted to KO-BL multiline variety covering an area of 94,000 ha. In addition, seed use and cultivation were restricted to the Niigata area to distinguish KO-BL from Koshihikari grown in other prefectures.

**Figure 1:**
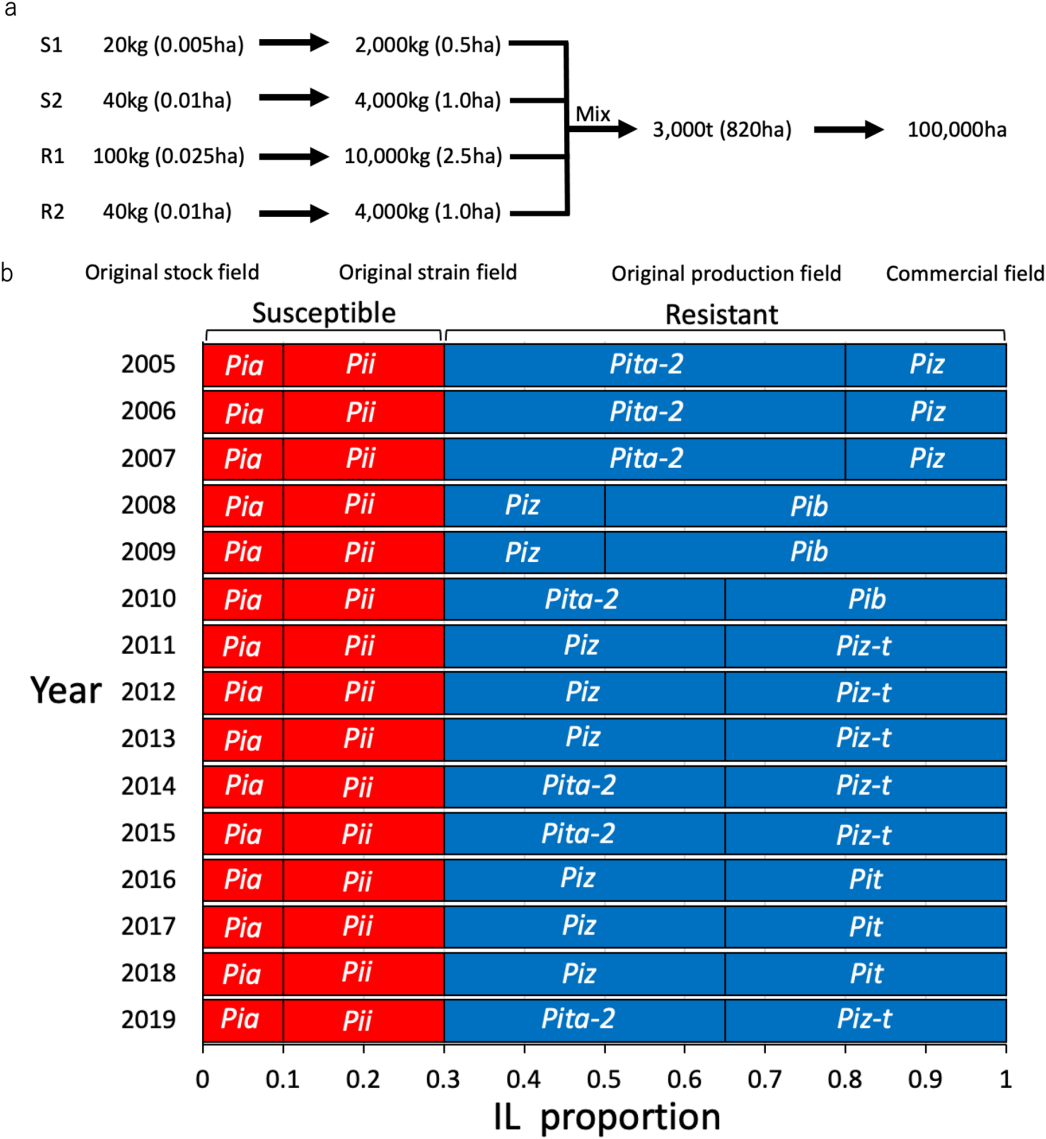
Representative seed production flow from original stock to commercial field and history of Koshihikari BL composition from 2005 to 2019 in Niigata Prefecture. (**a**) S1–S2, susceptible KO-BL; R1-R2, resistant KO-BL. Seeds obtained from original stock field at the Niigata Agricultural Research Institute. Seeds obtained from the original strain field and the original production field at both designated farmers’ fields. Commercial field (general farmers field) used for KO-BL production. Each field requires a year for seed production. (**b**) *Pia* and *Pii*, susceptible; *Pita-2, Piz, Pib, Piz-t*, and *Pit*, resistant. The proportion of susceptible KO-BLs and resistant KO-BLs was consistently 3:7 across years.

Seed production and mixture processes are managed with precision by each prefectural official member (Fig. 1a). Original isogenic lines (ILs) were separately produced from the original stock in the original strain fields by the Niigata prefectural government. Using a precise mixture machine, the mixture of four ILs was then blended by weight in 2000 kg volumes, all multiplied by ten (giving a total volume of 20 t). Original production fields and commercial fields all used blended seeds that had been authorized by seed production farmers and commercial farmers in the 2003 and 2004 trials. Thus, it takes two years for seed production at the original strain field followed by the original production field for the preparation of commercial fields; thus, the seed mixture composition needs to be determined at least two years before introduction. Susceptible and resistant (effective) ILs were mixed at a ratio of 3:7 from 2005 to 2019 (Fig. 1b, Supplementary Table S1). Susceptible ILs, possessing *Pia* and *Pii* genes, were always mixed at a ratio of 1:2, but the composition of resistant ILs, containing *Pita-2*, *Piz*, *Pib*, *Piz-t*, and *Pit* genes, was changed every two to three years to avoid the breakdown of resistance^6^. These changes were determined by annually monitoring blast race distributions.

**Figure 2:**
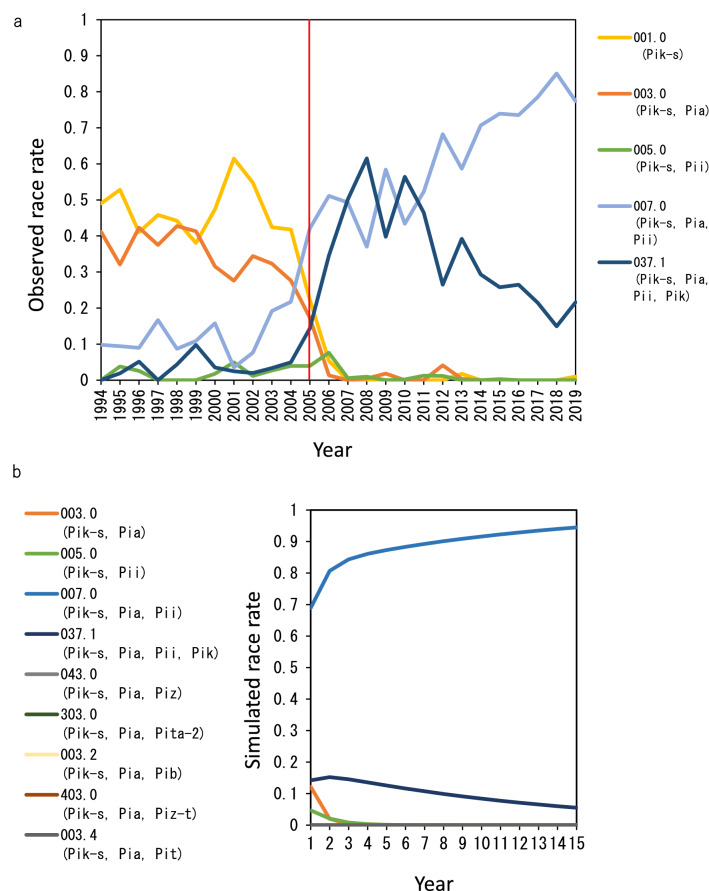
Blast race change during the 1994–2019 period in Niigata Prefecture and the worst-case simulation of blast race dynamics in KO-BL during the 2005–2019 (years 1–15) period. Races and virulences are shown in Table 1. (**a**) A red line indicates the year (2005) when KO-BL was introduced. Races 007.0 and 037.1 became dominant after the introduction. (**b**) Actual races and their rates in 2004 and annual KO-BL compositions from 2005 to 2019 were set in the simulation. Parameters set in the simulation were as follows: maximum lesion number in a year, 10,000,000; weather condition, 10 (favorable); virulent mutation rate, 10^–5^; overwintering probability, 0.01; number of simulated years, 15; and number of simulation trials, 1000. The 1000 trial results for the lesion number increase in each race were averaged in each year and transformed into rates to show race dynamics. All simulation results are shown in Supplementary Table 6 in Supplementary information 2. The races 007.0 and 037.1 were also dominant until year 15 (correspond to 2019). Both actual and simulated race dynamics showed no outbreaks of the resistant composition of KO-BL.

In Niigata Prefecture, the predominant 5 blast races distributed from 1994 to 2004 were 001.0 (virulent to Koshihikari [*Pik-s*]), 003.0 (virulent to *Pik-s* and *Pia*), 005.0 (virulent to *Pik-s* and *Pii*), 007.0 (virulent to *Pik-s*, *Pia*, and *Pii*), and 037.1 (virulent to *Pik-s*, *Pia*, *Pii*, and *Pik*) (Fig. 2a, Supplementary Table S2). Because all the 5 races were virulent to Koshihikari, which had been widely cultivated in Niigata area during the years, there were no drastic race changes. In addition, genetic variations in blast resistance indicated that Koshihikari also harbored the *Pish* gene, and that the *Pia*, *Pii*, and *Pik* genes were also dominant in the Hokuriku region, including Niigata Prefecture^21^. Virulent blast races against the resistance genes *Pish*, *Pia*, *Pii*, *Pi3*, *Pi5*(*t*), *Pik*, *Pik-s*, and *Pi19*(*t*) were dominantly distributed in Niigata Prefecture^22^. These reports confirmed that Koshihikari had been susceptible to dominant blast races before KO-BL introduction.

In the 2005 release year of KO-BL, the predominant blast races, 001.0 (virulent to *Pik-s*) and 003.0 (virulent to *Pik-s* and *Pia*), drastically decreased in distribution from 41.8% to 22.3% and 27.6% to 17.3%, respectively (Fig. 2a, Supplementary Table S2). Interestingly, races 001.0 and 003.0 rapidly decreased by 5.4% and 1.3% in 2006, respectively, even though especially *Pia*, which can be infected by the race 003.0, was used in the KO-BL composition. Because all ILs in the composition of KO-BL were resistant to race 001.0, and race 003.0 was only virulent to *Pia*, which made up 10% of the annual KO-BL composition (Table 1). In contrast, races 007.0 (virulent to *Pik-s*, *Pia*, and *Pii*) and 037.1 (virulent to *Pik-s*, *Pia*, *Pii*, and *Pik*) dominated from 2005 to 2019. The higher rate of race 007.0 detection was affected by 30% of the ILs composing the annual KO-BL were susceptible. The second highest rate of race 037.1 detection was affected by a number of factors: the high susceptibility of a minor cultivar that had *Pii* and *Pik*, the mosaic configuration of fields typical in Niigata, and the air-borne spread of race 037.1. To maintain consensus on KO-BL cultivation based on total blast suppression in Niigata, rarely detected races virulent to resistant ILs in commercial fields are strictly supervised by the prefectural government to avoid unnecessary confusion in Niigata residents.

**Table 1:**
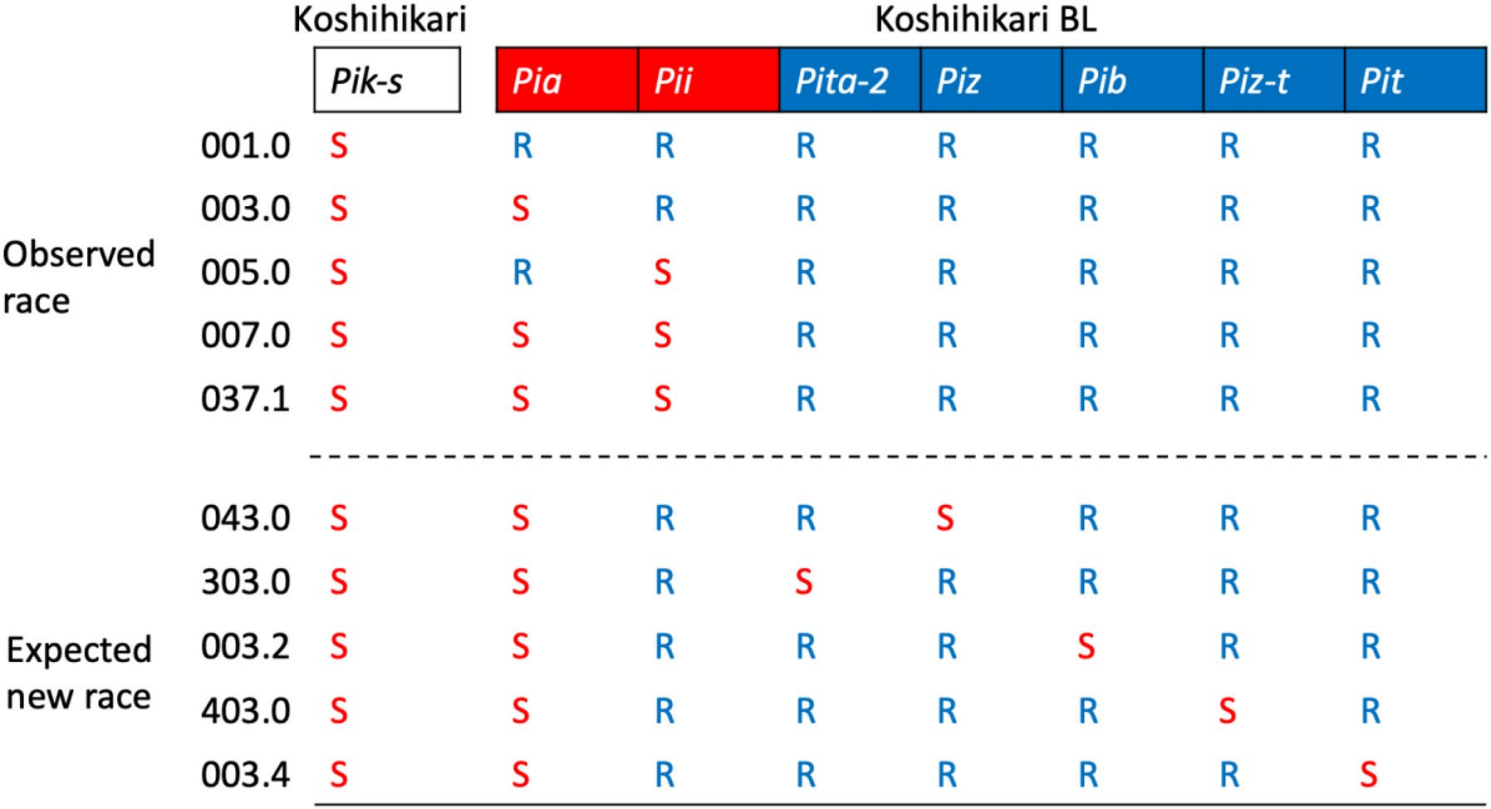
Susceptible or resistant reaction of Koshihikari and KO-BL against blast races.

^1^Koshihikari with *Pik-s* was susceptible (S) to observed race 001.0. *Pia*, *Pii*, *Pita-2*, *Piz*, *Pib*, *Piz-t* and *Pit* were resistant (R) to the race 001.0. *Pia* and *Pii* were part of the susceptible composition of KO-BL. *Pita-2*, *Piz*, *Pib*, *Piz-t* and *Pit* were part of the resistant composition of KO-BL against all observed races.

^2^The expected new races (043.0, 303.0, 003.2, 403.0 and 003.4) were virulent to respective resistant compositions of KO-BL(*Piz*, *Pita-2*, *Pib*, *Piz-t* and *Pit*)*.* All observed and expected new races were set for the simulation of race dynamics.

^3^Race 037.1 was virulent to *Pik* (not part of the composition of KO-BL).

In 2008, to mathematically support KO-BL composition changes, we developed a simulation software to estimate long-term blast race dynamics in multilines using a plant‒pathogen coevolution system^23^. The model calculated the persistence of resistant ILs to determine the optimal timing of changes to multiline variety compositions. To simulate race dynamics in KO-BL, we set five currently investigated races, 001.0 (virulent to *Pik-s*), 003.0 (virulent to *Pik-s* and *Pia*), 005.0 (virulent to *Pik-s* and *Pii*), 007.0 (virulent to *Pik-s*, *Pia*, and *Pii*), and 037.1 (virulent to *Pik-s*, *Pia*, *Pii*, and *Pik*), and their rates in 2004, as well as five emerging races, 043.0 (virulent to *Pik-s*, *Pia*, and *Piz*), 303.0 (virulent to *Pik-s*, *Pia*, and *Pita-2*), 003.2 (virulent to *Pik-s*, *Pia*, and *Pib*), 403.0 (virulent to *Pik-s*, *Pia*, and *Piz-t*), and 003.4 (virulent to *Pik-s*, *Pia*, and *Pit*) (see Fig. 2b, Supplementary Table S3) against five newly introduced respective resistant KO-BLs (see Fig. 1b, Supplementary Table S1) and the annual KO-BL compositions from 2005 to 2019. The worst case (severe epidemic) simulation result (Fig. 2b, Supplementary Tables S3 and S6) showed that race 007.0 (virulent to susceptible *Pik-s*, *Pia* and *Pii*) became the predominant race (77.4%), and race 037.1 (virulent to *Pik-s*, *Pia*, *Pii*, and *Pik*) remained at a low frequency (21.6%) until the fifteenth year (corresponding to 2019). In addition, super-race virulent to all KO-BLs did not emerge in this simulation. These suppression of outbreaks of newly emerged virulent races, including super-race on resistant KO-BL was apparently affected by 2–3 years of change in resistant KO-BL composition, and total suppression of blast occurrence decreasing the blast population. These results indicated that almost all the epidemics analyzed reflected actual race dynamics without affecting other minor races from other susceptible cultivars grown in Niigata, especially up to 2011. Thus, our decision support system provides an evaluation of KO-BL persistence and indicates the KO-BL composition changes needed for blast race population control in large areas. In addition, our simulation model may be useful for evaluating future KO-BL composition changes.

**Figure 3:**
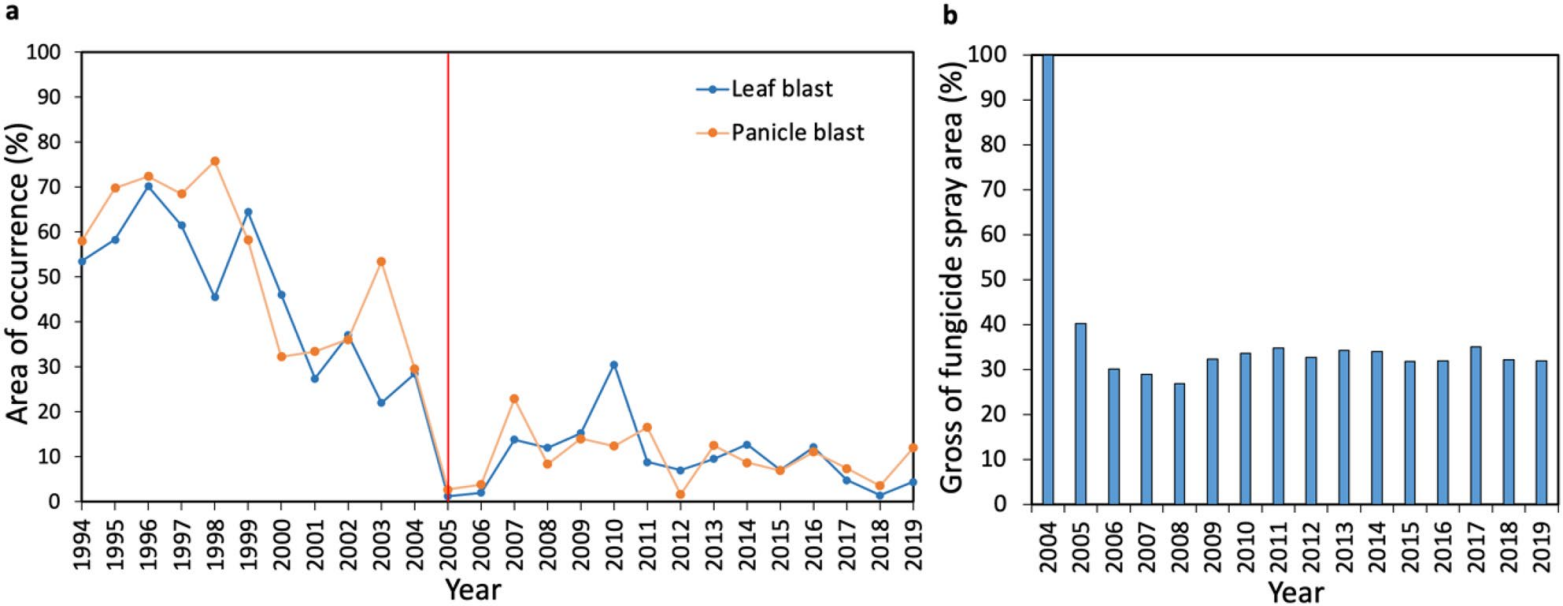
Leaf and panicle blast occurrence from 1994 to 2019 and blast control area from 2004 to 2019 in Niigata Prefecture. (**a**) A red line indicates the year (2005) when KO-BL was introduced. (**b**) Gross fungicide spray area decreased by approximately one-third during the 2005–2019 period compared with 2004.

Blast occurrence drastically decreased after 2005 (Fig. 3a, Supplementary Table S4). The average occurrence of leaf and panicle blast was 46.1% and 52.9% during the 1995–2004 period and 9.5% and 9.6% during the 2005–2019 period, respectively. This resulted in a blast suppression effect by 70% of the resistant composition in KO-BL. Current seed production fields are rarely contaminated with virulent races against resistant KO-BLs. This suggests that seed sanitation contributes to the suppression of virulent pathogen epidemics in multilines. In addition, induced resistance^24,25^ may have no effect on the practical use of multilines. Rice plants were found to induce a resistance response when inoculated with avirulent races of blast (those that stimulate protective responses to virulent race attacks). As the detection of several races in one area is rare and blast occurrence tends to be low, conditions that induce resistance in field situations do not occur. Fungicide applications to control blast in KO-BL and other minor cultivars decreased by approximately one-third during the 2005–2019 period compared with 2004 (Fig. 3b, Supplementary Table S5). Thus, the commercial scale use of crop diversity is clearly effective for the environmentally friendly control of airborne diseases.

The optimum long-term solution for pathogen population control using genetic diversity includes multilines. Blast occurrence in KO-BL introduced in Niigata, and the theoretical value of blast suppression in KO-BL tested at small scales, were reduced by approximately 10% compared to that of monoculture plots^26–28^. Thirty percent of susceptible ILs in KO-BL have the potential to improve compatible races with susceptible ILs and become predominant in large areas. This would contribute to the suppression of rapid increases in new virulent races emerging in the blast population. To maintain consensus on KO-BL cultivation based on total blast suppression in Niigata, rarely detected races virulent to resistant KO-BLs in commercial fields are strictly monitored by the prefectural government. Educating Niigata farmers ensures the long-term use of KO-BL. In fact, lower blast occurrence has been attributed to careful KO-BL cultivation and seed management.

The implementation of genetically diversified homogeneous seed mixtures, rotations with resistant KO-BL, restricted KO-BL cultivation, and pathogen monitoring allowed rice quality to be maintained, diseases to be suppressed, and environmentally sound agriculture to be economically viable in Niigata. Collaboration among prefectural officers, farmers, and consumers in Niigata has resulted in safer rice production with good agricultural practices (GAPs) that meet sustainable development goals (SDGs). In addition, DNA tests differentiate KO-BL from the original Koshihikari for buyers, thereby prohibiting illegal distribution. Multiline varieties have been used in small areas in two different prefectures. For example, in Miyagi pref., Sasanishiki BL consisted of *Pik*, *Pik-m*, and *Piz* at ratios of 4:3:3 and 3:3:4 in 1995 and 1996, respectively. This composition was changed to *Pik*, *Pik-m*, *Piz*, and *Piz-t* at a ratio of 1:1:4:4 from 1997 to 2007 to prevent an increase in race 037.1 (virulent to the BL: *Pik* and *Pik-m*). In addition, an equal mixture of seven BLs (*Pib*, *Pik*, *Pik-m*, *Piz*, *Piz-t*, *Pita*, and *Pita-2*) was cultivated in 300 ha areas (maximum 4000 ha) from 2008 to 2014 without any outbreaks observed. In Toyama pref., the Koshihikari Toyama BL, which consists of resistant ILs, *Pita-2*, *Pib*, and *Pik-p* at a ratio of 4:4:2, was cultivated in an area of 300 ha and required a 50% reduction in chemical inputs from 2003 up to the present. Our model also calculated a greater than 50-year persistence in terms of the small area effect in both prefectural cases. This result depends on an insufficient pathogen population increase in virulent mutations against resistant ILs (data not shown). In this way, the practical use of a multiline provides control without the need for as much fungicide with or without a periodic change in IL composition. Our results demonstrate that the management of crop and pathogen coevolution can control diseases at large scales and, thereby, contribute to global food security.

## Methods

### Rice plants

Rice (*Oryza sativa* L.) is a staple food crop for Japan and much of the world. To breed Koshihikari BL (KO-BL), a total of 7 isogenic lines, *Pit* on rice chromosome (chr.) 1, *Pib* on chr. 2, *Piz* and *Piz-t* on chr. 6, *Pii* on chr. 9, *Pia* on chr. 11 and *Pita-2* on chr. 12, were backcrossed five to seven times followed by a three-year line selection for KO-BL^20^. The KO-BL used in this study included 7 registered lines, *Pia*, *Pii*, *Pita-2, Piz, Pib, Piz-t*, and *Pit*, and protected by the Plant Variety Protection and Seed Act (Japanese Law) from the Ministry of Agriculture, Forestry and Fisheries as part of the Japanese Government. Seeds of the original stock field were obtained from paddy fields at the Niigata Agricultural Research Institute under the Niigata Prefectural Major Crop Seed Law. Seeds of the original strain field and original production field were produced by designated farmers under the same law. Rice of commercial fields were produced by general farmers restricted to Niigata Prefecture under the Agricultural Products Inspection Act (Japanese Law) by the Ministry of Agriculture, Forestry and Fisheries as part of the Japanese Government.

Blast-infected rice leaves and panicles were collected by Niigata prefectural officers under the Plant Protection Law from the Ministry of Agriculture, Forestry and Fisheries as part of the Japanese Government, and permissions were previously obtained from all farmers before sampling. The plant protection committees of all the Niigata municipalities were also authorized for these samplings.

### Race investigation

Our field tests were carried out in Niigata Prefecture (Japan), which has a wet season favorable for blast development during the rice growing season. To investigate blast race distributions, the Niigata area was divided into 5 km^2^ grid squares. If leaf and panicle blast lesions were found in each grid square, infected leaf and panicle samples were collected by Niigata prefectural officers. Officers from the plant protection committees of the various Niigata municipalities also collaborated in the blast lesion sampling. Plant pathological research was performed using the single spore isolation method. The pathogenicity of isolated *P*. *oryzae* was determined by rice seedling inoculation using the race differentiation method^29,30^. Briefly, seedlings of the 12 Japanese differential varieties (*Pik-s*, *Pia*, *Pii*, *Pik*, *Pik-m*, *Piz*, *Pita*, *Pita-2*, *Piz-t*, *Pik-p*, *Pib* and *Pit*) at the 4–5 leaf age were sprayed with 1 × 10^4^ spores/ml of the isolate and then moved to a humidic chamber (100% relative humidity) at 25 °C for 20 h. Inoculated plants were moved to a greenhouse at 25 °C for 1 week. Susceptible and resistant reactions were judged based on leaf blast lesion formation to determine the races of the isolates.

### Disease assessments

Before each cropping season started, a representative field from each 15 km^2^ grid square was selected to monitor disease and predict pest occurrence. After rice seedlings were planted in paddy fields, general epidemics, which initially started as a small number of leaf blast lesions, were observed in June. The subsequent development of leaf blasts (in July) and panicle blasts (in August and September) was evaluated as leaf blast lesion area per hill and diseased grains, respectively.

### KO-BL composition determination

Once a year, the Niigata prefectural committee assessed proposals for altering the resistant IL composition in KO-BL for the following two years using data on monitored race frequency and blast severity.

### Simulation model

The gene-for-gene system^23^ between rice genotypes and blast races was used to develop a simulation software. For the Niigata simulation, the parameters were set as follows: actual races and their rates investigated in 2004 (001.0 [0.423], 003.0 [0.245], 005.0 [0.038], 007.0 [0.196], 037.1 [0.049]), actual proportion of susceptible and resistant ILs used from 2005 to 2019 (Fig. 2b), mutation rate (10^–5^)^31^, overwintering probability (0.01), favorable weather condition (10), maximum lesion number (10,000,000), number of simulated years (15), number of simulation trials (1000), and variables (lesion number of each race). Races on each IL increased exponentially until reaching the maximum lesion number, i.e., 10,000,000 × 10 (100,000,000) under favorable weather conditions every year. Mutated races emerged at a 10^–5^ rate, and randomly selected races were overwintered, including mutated race from 10,000,000 × 10 × 0.01 (1,000,000) lesions. The 1000 trial results of the lesion number increase for each race were averaged in each year and transformed to the rates to show race dynamics.

### Ethics declarations

This study was performed in accordance with relevant rules, guidelines and regulations. There were no human subjects in this study.

## Supplementary Information

Supplementary Information 1.

Supplementary Information 2.

## Data Availability

All the data used in the present study are included in this manuscript and supplementary information files; however, we will provide data from the simulation results for combinations of races and rice resistance genes, and all the raw data used in this study from the corresponding author upon reasonable request to all interested scientists.
