# Supplementary material for: Long-term blast control in high eating quality rice using multilines
Source: Sci Rep. 2022 Sep 1;12:14880. doi: 10.1038/s41598-022-19237-x (PMC9436990; doi:10.1038/s41598-022-19237-x)
Supplement: Supplementary file 1 — Supplementary Information 1. [file 41598_2022_19237_MOESM1_ESM.docx]

**Long-term blast control in high eating quality rice using multilines**

Kouji Ishikawa^1^, Tomohisa Kuroda^1^, Takeshi Hori^1^, Daisuke Iwata^1^, Seijiro Matsuzawa^1^, Jun Nakabayashi^2^, Akira Sasaki^3^ & Taketo Ashizawa^4,*^

^1^ Niigata Agricultural Research Institute, Crop Research Center, Nagaoka 940-0826, Japan

^2^ Tokyo Medical and Dental University, Bunkyo-ku 113-8510, Japan

^3^ The Graduate University for Advanced Studies, Hayama 240-0193, Japan

^4^ National Agriculture and Food Research Organization (NARO), Tsukuba 305-8666, Japan

Corresponding.toketa@affrc.go.jp

Online raw data supplementary information 1

Supplementary Table 1. Same as Figure 1b of the raw data.

| Year/IL | *Pia* | *Pii* | *Pita-2* | *Piz* | *Pib* | *Piz-t* |
| --- | --- | --- | --- | --- | --- | --- |
| 2005 | 0.1 | 0.2 | 0.5 | 0.2 | 0 | 0 |
| 2006 | 0.1 | 0.2 | 0.5 | 0.2 | 0 | 0 |
| 2007 | 0.1 | 0.2 | 0.5 | 0.2 | 0 | 0 |
| 2008 | 0.1 | 0.2 | 0 | 0.2 | 0.5 | 0 |
| 2009 | 0.1 | 0.2 | 0 | 0.2 | 0.5 | 0 |
| 2010 | 0.1 | 0.2 | 0.35 | 0 | 0.35 | 0 |
| 2011 | 0.1 | 0.2 | 0 | 0.35 | 0 | 0.35 |
| 2012 | 0.1 | 0.2 | 0 | 0.35 | 0 | 0.35 |
| 2013 | 0.1 | 0.2 | 0. | 0.35 | 0 | 0.35 |
| 2014 | 0.1 | 0.2 | 0.35 | 0 | 0 | 0.35 |
| 2015 | 0.1 | 0.2 | 0.35 | 0 | 0 | 0.35 |
| 2016 | 0.1 | 0.2 | 0 | 0.35 | 0 | 0 |
| 2017 | 0.1 | 0.2 | 0 | 0.35 | 0 | 0 |
| 2018 | 0.1 | 0.2 | 0 | 0.35 | 0 | 0 |
| 2019 | 0.1 | 0.2 | 0.35 | 0 | 0 | 0.35 |

Supplementary Table 2. Same as Figure 2a of the raw data.

| Year/Race | 001.0 | 003.0 | 005.0 | 007.0 | 037.1 |
| --- | --- | --- | --- | --- | --- |
| 1994 | 0.490 | 0.412 | 0.000 | 0.098 | 0.000 |
| 1995 | 0.528 | 0.321 | 0.038 | 0.094 | 0.019 |
| 1996 | 0.410 | 0.423 | 0.026 | 0.090 | 0.051 |
| 1997 | 0.458 | 0.375 | 0.000 | 0.167 | 0.000 |
| 1998 | 0.442 | 0.428 | 0.000 | 0.087 | 0.043 |
| 1999 | 0.380 | 0.413 | 0.000 | 0.109 | 0.098 |
| 2000 | 0.474 | 0.316 | 0.018 | 0.158 | 0.035 |
| 2001 | 0.615 | 0.276 | 0.049 | 0.035 | 0.025 |
| 2002 | 0.548 | 0.344 | 0.012 | 0.076 | 0.020 |
| 2003 | 0.424 | 0.323 | 0.027 | 0.192 | 0.034 |
| 2004 | 0.418 | 0.276 | 0.039 | 0.217 | 0.049 |
| 2005 | 0.223 | 0.173 | 0.040 | 0.421 | 0.144 |
| 2006 | 0.054 | 0.013 | 0.076 | 0.511 | 0.345 |
| 2007 | 0.000 | 0.000 | 0.006 | 0.493 | 0.501 |
| 2008 | 0.000 | 0.005 | 0.010 | 0.370 | 0.615 |
| 2009 | 0.000 | 0.018 | 0.000 | 0.584 | 0.398 |
| 2010 | 0.000 | 0.000 | 0.002 | 0.434 | 0.564 |
| 2011 | 0.000 | 0.000 | 0.012 | 0.523 | 0.465 |
| 2012 | 0.000 | 0.041 | 0.012 | 0.682 | 0.265 |
| 2013 | 0.018 | 0.004 | 0.000 | 0.587 | 0.392 |
| 2014 | 0.000 | 0.000 | 0.000 | 0.707 | 0.293 |
| 2015 | 0.000 | 0.000 | 0.003 | 0.739 | 0.258 |
| 2016 | 0.000 | 0.000 | 0.000 | 0.735 | 0.265 |
| 2017 | 0.000 | 0.000 | 0.000 | 0.785 | 0.215 |
| 2018 | 0.000 | 0.000 | 0.000 | 0.851 | 0.149 |
| 2019 | 0.010 | 0.000 | 0.000 | 0.774 | 0.216 |

Supplementary Table 3. Same as Figure 2b of the summarized data. The raw data are in Supplementary Table 5 in Supplementary information 2.

| Year/Race | 003.0 | 005.0 | 007.0 | 037.1 | 043.0 | 303.0 | 003.2 | 403.0 | 003.4 |
| --- | --- | --- | --- | --- | --- | --- | --- | --- | --- |
| 1 | 0.122 | 0.047 | 0.689 | 0.142 | 0.000 | 0.000 | 0.000 | 0.000 | 0.000 |
| 2 | 0.020 | 0.021 | 0.807 | 0.152 | 0.000 | 0.000 | 0.000 | 0.000 | 0.000 |
| 3 | 0.003 | 0.008 | 0.843 | 0.145 | 0.000 | 0.000 | 0.000 | 0.000 | 0.000 |
| 4 | 0.000 | 0.003 | 0.861 | 0.136 | 0.000 | 0.000 | 0.000 | 0.000 | 0.000 |
| 5 | 0.000 | 0.001 | 0.873 | 0.126 | 0.000 | 0.000 | 0.000 | 0.000 | 0.000 |
| 6 | 0.000 | 0.000 | 0.883 | 0.116 | 0.000 | 0.000 | 0.000 | 0.000 | 0.000 |
| 7 | 0.000 | 0.000 | 0.892 | 0.107 | 0.000 | 0.000 | 0.000 | 0.000 | 0.000 |
| 8 | 0.000 | 0.000 | 0.901 | 0.099 | 0.000 | 0.000 | 0.000 | 0.000 | 0.000 |
| 9 | 0.000 | 0.000 | 0.909 | 0.091 | 0.000 | 0.000 | 0.000 | 0.000 | 0.000 |
| 10 | 0.000 | 0.000 | 0.916 | 0.084 | 0.000 | 0.000 | 0.000 | 0.000 | 0.000 |
| 11 | 0.000 | 0.000 | 0.923 | 0.077 | 0.000 | 0.000 | 0.000 | 0.000 | 0.000 |
| 12 | 0.000 | 0.000 | 0.929 | 0.071 | 0.000 | 0.000 | 0.000 | 0.000 | 0.000 |
| 13 | 0.000 | 0.000 | 0.935 | 0.065 | 0.000 | 0.000 | 0.000 | 0.000 | 0.000 |
| 14 | 0.000 | 0.000 | 0.940 | 0.060 | 0.000 | 0.000 | 0.000 | 0.000 | 0.000 |
| 15 | 0.000 | 0.000 | 0.945 | 0.055 | 0.000 | 0.000 | 0.000 | 0.000 | 0.000 |

Supplementary Table 4. Same as Figure 3a of the raw data.

| Year | Leaf blast (%) | Panicle blast (%) |
| --- | --- | --- |
| 1994 | 53.5 | 58 |
| 1995 | 58.3 | 69.8 |
| 1996 | 70.2 | 72.4 |
| 1997 | 61.5 | 68.5 |
| 1998 | 45.5 | 75.8 |
| 1999 | 64.5 | 58.2 |
| 2000 | 46.1 | 32.3 |
| 2001 | 27.4 | 33.4 |
| 2002 | 37.1 | 36.1 |
| 2003 | 22 | 53.4 |
| 2004 | 28.5 | 29.5 |
| 2005 | 1.2 | 2.7 |
| 2006 | 2 | 3.8 |
| 2007 | 13.8 | 22.9 |
| 2008 | 12 | 8.4 |
| 2009 | 15.2 | 14 |
| 2010 | 30.5 | 12.4 |
| 2011 | 8.8 | 16.5 |
| 2012 | 7 | 1.6 |
| 2013 | 9.5 | 12.5 |
| 2014 | 12.7 | 8.7 |
| 2015 | 7.1 | 6.9 |
| 2016 | 12.1 | 11.1 |
| 2017 | 4.8 | 7.4 |
| 2018 | 1.4 | 3.6 |
| 2019 | 4.4 | 12 |

Supplementary Table 5. Same as Figure 3b of the raw data.

| Year | Gross fungicide splay area (%) |
| --- | --- |
| 2004 | 100 |
| 2005 | 40.3 |
| 2006 | 30.1 |
| 2007 | 28.9 |
| 2008 | 26.9 |
| 2009 | 32.3 |
| 2010 | 33.6 |
| 2011 | 34.8 |
| 2012 | 32.7 |
| 2013 | 34.3 |
| 2014 | 34 |
| 2015 | 31.8 |
| 2016 | 31.9 |
| 2017 | 35.1 |
| 2018 | 32.2 |
| 2019 | 31.9 |
