# Supplementary material for: Long-term blast control in high eating quality rice using multilines
Source: Sci Rep. 2022 Sep 1;12:14880. doi: 10.1038/s41598-022-19237-x (PMC9436990; doi:10.1038/s41598-022-19237-x)
Supplement: Supplementary file 2 — Supplementary Information 2. [file 41598_2022_19237_MOESM2_ESM.docx]

**Long-term blast control in high eating quality rice using multilines**

Kouji Ishikawa^1^, Tomohisa Kuroda^1^, Takeshi Hori^1^, Daisuke Iwata^1^, Seijiro Matsuzawa^1^, Jun Nakabayashi^2^, Akira Sasaki^3^ & Taketo Ashizawa^4,*^

^1^ Niigata Agricultural Research Institute, Crop Research Center, Nagaoka 940-0826, Japan

^2^ Tokyo Medical and Dental University, Bunkyo-ku 113-8510, Japan

^3^ The Graduate University for Advanced Studies, Hayama 240-0193, Japan

^4^ National Agriculture and Food Research Organization (NARO), Tsukuba 305-8666, Japan

Corresponding.toketa@affrc.go.jp

Online raw data supplementary information 2

Supplementary Table 6. Same as Figure 2b of the raw data. Summarized data in Supplementary Table 3 in Supplementary information 1.

| Year 1 | Race |  |  |  |  |  |  |  |  |
| --- | --- | --- | --- | --- | --- | --- | --- | --- | --- |
| Trial | 003.0 | 005.0 | 007.0 | 037.1 | 043.0 | 303.0 | 003.2 | 403.0 | 003.4 |
| 1 | 0.120831 | 0.046772 | 0.688255 | 0.144142 | 0 | 0 | 0 | 0 | 0 |
| 2 | 0.122961 | 0.045431 | 0.688562 | 0.143045 | 0 | 0 | 0 | 0 | 0 |
| 3 | 0.124477 | 0.045784 | 0.688582 | 0.141157 | 0 | 0 | 0 | 0 | 0 |
| 4 | 0.121113 | 0.046417 | 0.69034 | 0.14212 | 0.00001 | 0 | 0 | 0 | 0 |
| 5 | 0.121513 | 0.046118 | 0.690222 | 0.142147 | 0 | 0 | 0 | 0 | 0 |
| 6 | 0.122342 | 0.046415 | 0.688146 | 0.143097 | 0 | 0 | 0 | 0 | 0 |
| 7 | 0.121911 | 0.047435 | 0.688541 | 0.142114 | 0 | 0 | 0 | 0 | 0 |
| 8 | 0.121256 | 0.046696 | 0.690503 | 0.141544 | 0 | 0 | 0 | 0 | 0 |
| 9 | 0.122911 | 0.046437 | 0.689063 | 0.141588 | 0 | 0 | 0 | 0 | 0 |
| 10 | 0.122784 | 0.046624 | 0.687954 | 0.142638 | 0 | 0 | 0 | 0 | 0 |
| 11 | 0.121638 | 0.04742 | 0.688492 | 0.14244 | 0.00001 | 0 | 0 | 0 | 0 |
| 12 | 0.12302 | 0.045406 | 0.688886 | 0.142688 | 0 | 0 | 0 | 0 | 0 |
| 13 | 0.121796 | 0.046989 | 0.690497 | 0.140718 | 0 | 0 | 0 | 0 | 0 |
| 14 | 0.122371 | 0.04679 | 0.687513 | 0.143317 | 0.00001 | 0 | 0 | 0 | 0 |
| 15 | 0.120915 | 0.046316 | 0.689377 | 0.143392 | 0 | 0 | 0 | 0 | 0 |
| 16 | 0.122755 | 0.047198 | 0.68624 | 0.143807 | 0 | 0 | 0 | 0 | 0 |
| 17 | 0.121869 | 0.046154 | 0.68966 | 0.142306 | 0.00001 | 0 | 0 | 0 | 0 |
| 18 | 0.120424 | 0.046319 | 0.690806 | 0.142451 | 0 | 0 | 0 | 0 | 0 |
| 19 | 0.122121 | 0.046727 | 0.686729 | 0.144422 | 0 | 0 | 0 | 0 | 0 |
| 20 | 0.120562 | 0.046774 | 0.688495 | 0.144169 | 0 | 0 | 0 | 0 | 0 |
| 21 | 0.121122 | 0.047109 | 0.691043 | 0.140726 | 0 | 0 | 0 | 0 | 0 |
| 22 | 0.120346 | 0.046618 | 0.688729 | 0.144298 | 0.00001 | 0 | 0 | 0 | 0 |
| 23 | 0.121519 | 0.04697 | 0.689418 | 0.142093 | 0 | 0 | 0 | 0 | 0 |
| 24 | 0.123086 | 0.047813 | 0.688791 | 0.14031 | 0 | 0 | 0 | 0 | 0 |
| 25 | 0.119536 | 0.046688 | 0.690886 | 0.14289 | 0 | 0 | 0 | 0 | 0 |
| 26 | 0.122395 | 0.047619 | 0.689948 | 0.140039 | 0 | 0 | 0 | 0 | 0 |
| 27 | 0.121217 | 0.046374 | 0.689436 | 0.142973 | 0 | 0 | 0 | 0 | 0 |
| 28 | 0.122057 | 0.047319 | 0.688316 | 0.142297 | 0.00001 | 0 | 0 | 0 | 0 |
| 29 | 0.121602 | 0.046768 | 0.689732 | 0.141897 | 0 | 0 | 0 | 0 | 0 |
| 30 | 0.121499 | 0.046786 | 0.688223 | 0.143491 | 0 | 0 | 0 | 0 | 0 |
| 31 | 0.121858 | 0.047058 | 0.690227 | 0.140856 | 0 | 0 | 0 | 0 | 0 |
| 32 | 0.119705 | 0.047459 | 0.689556 | 0.143279 | 0 | 0 | 0 | 0 | 0 |
| 33 | 0.120877 | 0.047753 | 0.68744 | 0.143931 | 0 | 0 | 0 | 0 | 0 |
| 34 | 0.12167 | 0.04607 | 0.689446 | 0.142804 | 0.00001 | 0 | 0 | 0 | 0 |
| 35 | 0.12215 | 0.046331 | 0.688368 | 0.143151 | 0 | 0 | 0 | 0 | 0 |
| 36 | 0.11983 | 0.04721 | 0.690203 | 0.142757 | 0 | 0 | 0 | 0 | 0 |
| 37 | 0.121898 | 0.048255 | 0.686544 | 0.143303 | 0 | 0 | 0 | 0 | 0 |
| 38 | 0.122268 | 0.045846 | 0.690818 | 0.141068 | 0 | 0 | 0 | 0 | 0 |
| 39 | 0.122079 | 0.047072 | 0.689271 | 0.141567 | 0.00001 | 0 | 0 | 0 | 0 |
| 40 | 0.120207 | 0.046438 | 0.69057 | 0.142785 | 0 | 0 | 0 | 0 | 0 |
| 41 | 0.121476 | 0.047845 | 0.688027 | 0.142641 | 0.00001 | 0 | 0 | 0 | 0 |
| 42 | 0.122687 | 0.046631 | 0.689994 | 0.140687 | 0 | 0 | 0 | 0 | 0 |
| 43 | 0.122587 | 0.048048 | 0.689501 | 0.139863 | 0 | 0 | 0 | 0 | 0 |
| 44 | 0.122296 | 0.046854 | 0.6884 | 0.14245 | 0 | 0 | 0 | 0 | 0 |
| 45 | 0.122481 | 0.047533 | 0.688739 | 0.141246 | 0 | 0 | 0 | 0 | 0 |
| 46 | 0.122303 | 0.047478 | 0.686862 | 0.143356 | 0 | 0 | 0 | 0 | 0 |
| 47 | 0.122301 | 0.047307 | 0.685938 | 0.144454 | 0 | 0 | 0 | 0 | 0 |
| 48 | 0.120875 | 0.045637 | 0.688872 | 0.144616 | 0 | 0 | 0 | 0 | 0 |
| 49 | 0.122154 | 0.046624 | 0.687251 | 0.143972 | 0 | 0 | 0 | 0 | 0 |
| 50 | 0.120061 | 0.046672 | 0.689144 | 0.144123 | 0 | 0 | 0 | 0 | 0 |
| 51 | 0.123794 | 0.046582 | 0.68816 | 0.141463 | 0 | 0 | 0 | 0 | 0 |
| 52 | 0.1222 | 0.04658 | 0.688672 | 0.142548 | 0 | 0 | 0 | 0 | 0 |
| 53 | 0.121647 | 0.047108 | 0.69212 | 0.139116 | 0.00001 | 0 | 0 | 0 | 0 |
| 54 | 0.120068 | 0.046609 | 0.691856 | 0.141467 | 0 | 0 | 0 | 0 | 0 |
| 55 | 0.121439 | 0.048101 | 0.689516 | 0.140944 | 0 | 0 | 0 | 0 | 0 |
| 56 | 0.121763 | 0.045996 | 0.688434 | 0.143808 | 0 | 0 | 0 | 0 | 0 |
| 57 | 0.120988 | 0.048024 | 0.689816 | 0.141173 | 0 | 0 | 0 | 0 | 0 |
| 58 | 0.122707 | 0.046821 | 0.689363 | 0.141108 | 0 | 0 | 0 | 0 | 0 |
| 59 | 0.122845 | 0.047721 | 0.6876 | 0.141835 | 0 | 0 | 0 | 0 | 0 |
| 60 | 0.122476 | 0.047068 | 0.688011 | 0.142445 | 0 | 0 | 0 | 0 | 0 |
| 61 | 0.122802 | 0.047147 | 0.688731 | 0.14132 | 0 | 0 | 0 | 0 | 0 |
| 62 | 0.121104 | 0.047407 | 0.690869 | 0.140621 | 0 | 0 | 0 | 0 | 0 |
| 63 | 0.123182 | 0.045935 | 0.690715 | 0.140168 | 0 | 0 | 0 | 0 | 0 |
| 64 | 0.121542 | 0.04759 | 0.689274 | 0.141594 | 0 | 0 | 0 | 0 | 0 |
| 65 | 0.123885 | 0.047002 | 0.684995 | 0.144119 | 0 | 0 | 0 | 0 | 0 |
| 66 | 0.122147 | 0.046421 | 0.690146 | 0.141287 | 0 | 0 | 0 | 0 | 0 |
| 67 | 0.12091 | 0.046725 | 0.689785 | 0.142579 | 0 | 0 | 0 | 0 | 0 |
| 68 | 0.123593 | 0.045982 | 0.689287 | 0.141129 | 0.00001 | 0 | 0 | 0 | 0 |
| 69 | 0.123103 | 0.046401 | 0.687113 | 0.143384 | 0 | 0 | 0 | 0 | 0 |
| 70 | 0.123513 | 0.046848 | 0.686173 | 0.143465 | 0 | 0 | 0 | 0 | 0 |
| 71 | 0.122519 | 0.047886 | 0.68647 | 0.143125 | 0 | 0 | 0 | 0 | 0 |
| 72 | 0.122481 | 0.04685 | 0.68971 | 0.140959 | 0 | 0 | 0 | 0 | 0 |
| 73 | 0.123691 | 0.046592 | 0.686856 | 0.142861 | 0 | 0 | 0 | 0 | 0 |
| 74 | 0.124175 | 0.046799 | 0.688005 | 0.14102 | 0 | 0 | 0 | 0 | 0 |
| 75 | 0.122438 | 0.046695 | 0.689071 | 0.141796 | 0 | 0 | 0 | 0 | 0 |
| 76 | 0.121737 | 0.048097 | 0.68897 | 0.141196 | 0 | 0 | 0 | 0 | 0 |
| 77 | 0.123375 | 0.04698 | 0.687119 | 0.142526 | 0 | 0 | 0 | 0 | 0 |
| 78 | 0.121191 | 0.047054 | 0.68962 | 0.142135 | 0 | 0 | 0 | 0 | 0 |
| 79 | 0.122562 | 0.045899 | 0.688557 | 0.142982 | 0 | 0 | 0 | 0 | 0 |
| 80 | 0.12199 | 0.046532 | 0.688272 | 0.143196 | 0.00001 | 0 | 0 | 0 | 0 |
| 81 | 0.12196 | 0.046955 | 0.688037 | 0.143047 | 0 | 0 | 0 | 0 | 0 |
| 82 | 0.122867 | 0.046513 | 0.688291 | 0.142329 | 0 | 0 | 0 | 0 | 0 |
| 83 | 0.121969 | 0.048732 | 0.689245 | 0.140054 | 0 | 0 | 0 | 0 | 0 |
| 84 | 0.122299 | 0.047613 | 0.690094 | 0.139993 | 0 | 0 | 0 | 0 | 0 |
| 85 | 0.120393 | 0.047511 | 0.688275 | 0.143821 | 0 | 0 | 0 | 0 | 0 |
| 86 | 0.121819 | 0.047378 | 0.690225 | 0.140578 | 0 | 0 | 0 | 0 | 0 |
| 87 | 0.122178 | 0.046954 | 0.687327 | 0.143541 | 0 | 0 | 0 | 0 | 0 |
| 88 | 0.122231 | 0.047521 | 0.687262 | 0.142987 | 0 | 0 | 0 | 0 | 0 |
| 89 | 0.125441 | 0.047414 | 0.685242 | 0.141903 | 0 | 0 | 0 | 0 | 0 |
| 90 | 0.120515 | 0.047026 | 0.690949 | 0.1415 | 0.00001 | 0 | 0 | 0 | 0 |
| 91 | 0.120781 | 0.046995 | 0.690508 | 0.141716 | 0 | 0 | 0 | 0 | 0 |
| 92 | 0.120917 | 0.048665 | 0.686934 | 0.143484 | 0 | 0 | 0 | 0 | 0 |
| 93 | 0.120257 | 0.047137 | 0.690358 | 0.142248 | 0 | 0 | 0 | 0 | 0 |
| 94 | 0.12196 | 0.047259 | 0.688596 | 0.142185 | 0 | 0 | 0 | 0 | 0 |
| 95 | 0.121207 | 0.04733 | 0.68879 | 0.142672 | 0 | 0 | 0 | 0 | 0 |
| 96 | 0.12034 | 0.04648 | 0.691573 | 0.141607 | 0 | 0 | 0 | 0 | 0 |
| 97 | 0.121943 | 0.046075 | 0.688685 | 0.143296 | 0 | 0 | 0 | 0 | 0 |
| 98 | 0.122005 | 0.047457 | 0.688096 | 0.142442 | 0 | 0 | 0 | 0 | 0 |
| 99 | 0.122674 | 0.046276 | 0.687014 | 0.144036 | 0 | 0 | 0 | 0 | 0 |
| 100 | 0.122496 | 0.047105 | 0.688654 | 0.141745 | 0 | 0 | 0 | 0 | 0 |
| 101 | 0.122338 | 0.047529 | 0.688206 | 0.141927 | 0 | 0 | 0 | 0 | 0 |
| 102 | 0.122961 | 0.046776 | 0.687028 | 0.143235 | 0 | 0 | 0 | 0 | 0 |
| 103 | 0.122594 | 0.048073 | 0.687813 | 0.14152 | 0 | 0 | 0 | 0 | 0 |
| 104 | 0.122348 | 0.046232 | 0.691265 | 0.140146 | 0.00001 | 0 | 0 | 0 | 0 |
| 105 | 0.122978 | 0.046527 | 0.68774 | 0.142755 | 0 | 0 | 0 | 0 | 0 |
| 106 | 0.121488 | 0.04615 | 0.687386 | 0.144975 | 0 | 0 | 0 | 0 | 0 |
| 107 | 0.122052 | 0.046048 | 0.688934 | 0.142955 | 0.00001 | 0 | 0 | 0 | 0 |
| 108 | 0.122741 | 0.047117 | 0.687025 | 0.143107 | 0.00001 | 0 | 0 | 0 | 0 |
| 109 | 0.122195 | 0.047294 | 0.687581 | 0.14293 | 0 | 0 | 0 | 0 | 0 |
| 110 | 0.122054 | 0.046111 | 0.690061 | 0.141774 | 0 | 0 | 0 | 0 | 0 |
| 111 | 0.123591 | 0.046433 | 0.688494 | 0.141482 | 0 | 0 | 0 | 0 | 0 |
| 112 | 0.123093 | 0.046897 | 0.688318 | 0.141692 | 0 | 0 | 0 | 0 | 0 |
| 113 | 0.122852 | 0.046824 | 0.688516 | 0.141808 | 0 | 0 | 0 | 0 | 0 |
| 114 | 0.123378 | 0.04641 | 0.688639 | 0.141573 | 0 | 0 | 0 | 0 | 0 |
| 115 | 0.121733 | 0.046189 | 0.691483 | 0.140595 | 0 | 0 | 0 | 0 | 0 |
| 116 | 0.122227 | 0.047936 | 0.687984 | 0.141853 | 0 | 0 | 0 | 0 | 0 |
| 117 | 0.120942 | 0.047033 | 0.68973 | 0.142295 | 0 | 0 | 0 | 0 | 0 |
| 118 | 0.121449 | 0.046429 | 0.691301 | 0.140821 | 0 | 0 | 0 | 0 | 0 |
| 119 | 0.122185 | 0.047013 | 0.686766 | 0.144037 | 0 | 0 | 0 | 0 | 0 |
| 120 | 0.122331 | 0.046564 | 0.690095 | 0.14101 | 0 | 0 | 0 | 0 | 0 |
| 121 | 0.119753 | 0.047776 | 0.690589 | 0.141873 | 0.00001 | 0 | 0 | 0 | 0 |
| 122 | 0.121636 | 0.045892 | 0.689962 | 0.14251 | 0 | 0 | 0 | 0 | 0 |
| 123 | 0.121428 | 0.046654 | 0.68947 | 0.142448 | 0 | 0 | 0 | 0 | 0 |
| 124 | 0.121119 | 0.04694 | 0.689728 | 0.142213 | 0 | 0 | 0 | 0 | 0 |
| 125 | 0.121519 | 0.046982 | 0.690945 | 0.140554 | 0 | 0 | 0 | 0 | 0 |
| 126 | 0.121648 | 0.046482 | 0.688038 | 0.143832 | 0 | 0 | 0 | 0 | 0 |
| 127 | 0.122267 | 0.046877 | 0.691159 | 0.139696 | 0 | 0 | 0 | 0 | 0 |
| 128 | 0.121651 | 0.046766 | 0.690966 | 0.140618 | 0 | 0 | 0 | 0 | 0 |
| 129 | 0.122056 | 0.046543 | 0.687373 | 0.144028 | 0 | 0 | 0 | 0 | 0 |
| 130 | 0.121442 | 0.047177 | 0.689128 | 0.142253 | 0 | 0 | 0 | 0 | 0 |
| 131 | 0.119946 | 0.04594 | 0.691849 | 0.142265 | 0 | 0 | 0 | 0 | 0 |
| 132 | 0.124338 | 0.047691 | 0.68488 | 0.143092 | 0 | 0 | 0 | 0 | 0 |
| 133 | 0.121605 | 0.047166 | 0.686442 | 0.144787 | 0 | 0 | 0 | 0 | 0 |
| 134 | 0.125276 | 0.046038 | 0.686505 | 0.142182 | 0 | 0 | 0 | 0 | 0 |
| 135 | 0.12312 | 0.045882 | 0.687877 | 0.14312 | 0 | 0 | 0 | 0 | 0 |
| 136 | 0.121888 | 0.047804 | 0.689974 | 0.140334 | 0 | 0 | 0 | 0 | 0 |
| 137 | 0.121418 | 0.047642 | 0.688528 | 0.142412 | 0 | 0 | 0 | 0 | 0 |
| 138 | 0.120153 | 0.045791 | 0.691078 | 0.142978 | 0 | 0 | 0 | 0 | 0 |
| 139 | 0.123187 | 0.046784 | 0.689014 | 0.141015 | 0 | 0 | 0 | 0 | 0 |
| 140 | 0.122357 | 0.046697 | 0.690164 | 0.140782 | 0 | 0 | 0 | 0 | 0 |
| 141 | 0.122869 | 0.046449 | 0.688373 | 0.142309 | 0 | 0 | 0 | 0 | 0 |
| 142 | 0.124299 | 0.047107 | 0.68499 | 0.143604 | 0 | 0 | 0 | 0 | 0 |
| 143 | 0.121942 | 0.046895 | 0.688956 | 0.142206 | 0 | 0 | 0 | 0 | 0 |
| 144 | 0.124164 | 0.046883 | 0.686699 | 0.142254 | 0 | 0 | 0 | 0 | 0 |
| 145 | 0.121717 | 0.04706 | 0.68956 | 0.141662 | 0 | 0 | 0 | 0 | 0 |
| 146 | 0.121755 | 0.046856 | 0.692652 | 0.138737 | 0 | 0 | 0 | 0 | 0 |
| 147 | 0.120693 | 0.046164 | 0.689417 | 0.143726 | 0 | 0 | 0 | 0 | 0 |
| 148 | 0.122689 | 0.046735 | 0.686396 | 0.14418 | 0 | 0 | 0 | 0 | 0 |
| 149 | 0.124162 | 0.045874 | 0.688135 | 0.141829 | 0 | 0 | 0 | 0 | 0 |
| 150 | 0.121523 | 0.046934 | 0.688456 | 0.143077 | 0.00001 | 0 | 0 | 0 | 0 |
| 151 | 0.121602 | 0.046787 | 0.687688 | 0.143924 | 0 | 0 | 0 | 0 | 0 |
| 152 | 0.120958 | 0.048152 | 0.687564 | 0.143326 | 0 | 0 | 0 | 0 | 0 |
| 153 | 0.123596 | 0.047341 | 0.687706 | 0.141356 | 0 | 0 | 0 | 0 | 0 |
| 154 | 0.12291 | 0.046608 | 0.688488 | 0.141995 | 0 | 0 | 0 | 0 | 0 |
| 155 | 0.120588 | 0.047609 | 0.68806 | 0.143744 | 0 | 0 | 0 | 0 | 0 |
| 156 | 0.121467 | 0.046447 | 0.68889 | 0.143196 | 0 | 0 | 0 | 0 | 0 |
| 157 | 0.122141 | 0.046655 | 0.690155 | 0.14105 | 0 | 0 | 0 | 0 | 0 |
| 158 | 0.122148 | 0.046496 | 0.691017 | 0.140339 | 0 | 0 | 0 | 0 | 0 |
| 159 | 0.121694 | 0.046739 | 0.689175 | 0.142391 | 0 | 0 | 0 | 0 | 0 |
| 160 | 0.122927 | 0.046283 | 0.691301 | 0.139478 | 0.00001 | 0 | 0 | 0 | 0 |
| 161 | 0.121844 | 0.046126 | 0.686557 | 0.145473 | 0 | 0 | 0 | 0 | 0 |
| 162 | 0.123724 | 0.046995 | 0.687731 | 0.14155 | 0 | 0 | 0 | 0 | 0 |
| 163 | 0.121883 | 0.047407 | 0.686975 | 0.143735 | 0 | 0 | 0 | 0 | 0 |
| 164 | 0.120694 | 0.047903 | 0.688044 | 0.143359 | 0 | 0 | 0 | 0 | 0 |
| 165 | 0.120325 | 0.047293 | 0.689457 | 0.142925 | 0 | 0 | 0 | 0 | 0 |
| 166 | 0.123877 | 0.046395 | 0.689638 | 0.14009 | 0 | 0 | 0 | 0 | 0 |
| 167 | 0.122688 | 0.048376 | 0.686799 | 0.142136 | 0 | 0 | 0 | 0 | 0 |
| 168 | 0.120819 | 0.047978 | 0.688634 | 0.142569 | 0 | 0 | 0 | 0 | 0 |
| 169 | 0.123087 | 0.046749 | 0.687116 | 0.143048 | 0 | 0 | 0 | 0 | 0 |
| 170 | 0.122415 | 0.047937 | 0.687266 | 0.142381 | 0 | 0 | 0 | 0 | 0 |
| 171 | 0.12282 | 0.04662 | 0.687438 | 0.143112 | 0.00001 | 0 | 0 | 0 | 0 |
| 172 | 0.122389 | 0.047253 | 0.68917 | 0.141188 | 0 | 0 | 0 | 0 | 0 |
| 173 | 0.122753 | 0.048822 | 0.686282 | 0.142143 | 0 | 0 | 0 | 0 | 0 |
| 174 | 0.123496 | 0.048348 | 0.684994 | 0.143162 | 0 | 0 | 0 | 0 | 0 |
| 175 | 0.122 | 0.046206 | 0.689985 | 0.141809 | 0 | 0 | 0 | 0 | 0 |
| 176 | 0.123734 | 0.046167 | 0.691347 | 0.138741 | 0.00001 | 0 | 0 | 0 | 0 |
| 177 | 0.12384 | 0.047141 | 0.686489 | 0.14253 | 0 | 0 | 0 | 0 | 0 |
| 178 | 0.120532 | 0.046317 | 0.691495 | 0.141656 | 0 | 0 | 0 | 0 | 0 |
| 179 | 0.122763 | 0.04757 | 0.687769 | 0.141899 | 0 | 0 | 0 | 0 | 0 |
| 180 | 0.122229 | 0.047344 | 0.688224 | 0.142203 | 0 | 0 | 0 | 0 | 0 |
| 181 | 0.122065 | 0.046325 | 0.690082 | 0.141528 | 0 | 0 | 0 | 0 | 0 |
| 182 | 0.121302 | 0.047042 | 0.689983 | 0.141674 | 0 | 0 | 0 | 0 | 0 |
| 183 | 0.122944 | 0.046856 | 0.687966 | 0.142234 | 0 | 0 | 0 | 0 | 0 |
| 184 | 0.121226 | 0.046122 | 0.690967 | 0.141685 | 0 | 0 | 0 | 0 | 0 |
| 185 | 0.12202 | 0.04687 | 0.687929 | 0.14318 | 0 | 0 | 0 | 0 | 0 |
| 186 | 0.12252 | 0.045586 | 0.689965 | 0.141919 | 0.00001 | 0 | 0 | 0 | 0 |
| 187 | 0.122333 | 0.045314 | 0.693804 | 0.138549 | 0 | 0 | 0 | 0 | 0 |
| 188 | 0.123475 | 0.046696 | 0.688121 | 0.141708 | 0 | 0 | 0 | 0 | 0 |
| 189 | 0.12221 | 0.047891 | 0.686666 | 0.143233 | 0 | 0 | 0 | 0 | 0 |
| 190 | 0.12268 | 0.046541 | 0.686861 | 0.143918 | 0 | 0 | 0 | 0 | 0 |
| 191 | 0.122729 | 0.047129 | 0.689345 | 0.140797 | 0 | 0 | 0 | 0 | 0 |
| 192 | 0.123893 | 0.046164 | 0.688571 | 0.141372 | 0 | 0 | 0 | 0 | 0 |
| 193 | 0.121885 | 0.046955 | 0.687955 | 0.143206 | 0 | 0 | 0 | 0 | 0 |
| 194 | 0.121949 | 0.04615 | 0.688497 | 0.143404 | 0 | 0 | 0 | 0 | 0 |
| 195 | 0.122634 | 0.047358 | 0.688663 | 0.141345 | 0 | 0 | 0 | 0 | 0 |
| 196 | 0.121137 | 0.046295 | 0.692114 | 0.140445 | 0.00001 | 0 | 0 | 0 | 0 |
| 197 | 0.120949 | 0.045973 | 0.692834 | 0.140244 | 0 | 0 | 0 | 0 | 0 |
| 198 | 0.123955 | 0.046133 | 0.688975 | 0.140927 | 0.00001 | 0 | 0 | 0 | 0 |
| 199 | 0.123181 | 0.046028 | 0.691066 | 0.139725 | 0 | 0 | 0 | 0 | 0 |
| 200 | 0.12204 | 0.047601 | 0.687548 | 0.142812 | 0 | 0 | 0 | 0 | 0 |
| 201 | 0.122831 | 0.047595 | 0.686831 | 0.142744 | 0 | 0 | 0 | 0 | 0 |
| 202 | 0.122247 | 0.047287 | 0.687799 | 0.142668 | 0 | 0 | 0 | 0 | 0 |
| 203 | 0.120385 | 0.046623 | 0.690375 | 0.142617 | 0 | 0 | 0 | 0 | 0 |
| 204 | 0.122275 | 0.048231 | 0.688775 | 0.140719 | 0 | 0 | 0 | 0 | 0 |
| 205 | 0.122808 | 0.045852 | 0.686546 | 0.144794 | 0 | 0 | 0 | 0 | 0 |
| 206 | 0.121512 | 0.046194 | 0.688339 | 0.143955 | 0 | 0 | 0 | 0 | 0 |
| 207 | 0.123597 | 0.047015 | 0.68762 | 0.141768 | 0 | 0 | 0 | 0 | 0 |
| 208 | 0.120289 | 0.046281 | 0.692654 | 0.140776 | 0 | 0 | 0 | 0 | 0 |
| 209 | 0.122808 | 0.047248 | 0.687161 | 0.142783 | 0 | 0 | 0 | 0 | 0 |
| 210 | 0.12155 | 0.047552 | 0.689298 | 0.1416 | 0 | 0 | 0 | 0 | 0 |
| 211 | 0.121088 | 0.047392 | 0.689787 | 0.141733 | 0 | 0 | 0 | 0 | 0 |
| 212 | 0.122733 | 0.046614 | 0.688486 | 0.142167 | 0 | 0 | 0 | 0 | 0 |
| 213 | 0.12281 | 0.047275 | 0.69109 | 0.138824 | 0 | 0 | 0 | 0 | 0 |
| 214 | 0.121433 | 0.047445 | 0.688447 | 0.142675 | 0 | 0 | 0 | 0 | 0 |
| 215 | 0.124301 | 0.047658 | 0.687397 | 0.140635 | 0.00001 | 0 | 0 | 0 | 0 |
| 216 | 0.123076 | 0.046561 | 0.689335 | 0.141028 | 0 | 0 | 0 | 0 | 0 |
| 217 | 0.122487 | 0.046199 | 0.689225 | 0.142089 | 0 | 0 | 0 | 0 | 0 |
| 218 | 0.121508 | 0.046781 | 0.688743 | 0.142968 | 0 | 0 | 0 | 0 | 0 |
| 219 | 0.121949 | 0.047688 | 0.690524 | 0.139839 | 0 | 0 | 0 | 0 | 0 |
| 220 | 0.121497 | 0.047054 | 0.689227 | 0.142222 | 0 | 0 | 0 | 0 | 0 |
| 221 | 0.122656 | 0.046025 | 0.687558 | 0.143761 | 0 | 0 | 0 | 0 | 0 |
| 222 | 0.122482 | 0.047497 | 0.690694 | 0.139328 | 0 | 0 | 0 | 0 | 0 |
| 223 | 0.121421 | 0.046735 | 0.689169 | 0.142675 | 0 | 0 | 0 | 0 | 0 |
| 224 | 0.123056 | 0.046136 | 0.687719 | 0.143089 | 0 | 0 | 0 | 0 | 0 |
| 225 | 0.12232 | 0.04812 | 0.690093 | 0.139467 | 0 | 0 | 0 | 0 | 0 |
| 226 | 0.121165 | 0.047647 | 0.689615 | 0.141573 | 0 | 0 | 0 | 0 | 0 |
| 227 | 0.120409 | 0.047545 | 0.689619 | 0.142427 | 0 | 0 | 0 | 0 | 0 |
| 228 | 0.120792 | 0.046456 | 0.690166 | 0.142586 | 0 | 0 | 0 | 0 | 0 |
| 229 | 0.121652 | 0.047213 | 0.689184 | 0.141951 | 0 | 0 | 0 | 0 | 0 |
| 230 | 0.120907 | 0.04647 | 0.690966 | 0.141658 | 0 | 0 | 0 | 0 | 0 |
| 231 | 0.122653 | 0.047142 | 0.690398 | 0.139807 | 0 | 0 | 0 | 0 | 0 |
| 232 | 0.122081 | 0.04643 | 0.689271 | 0.142208 | 0.00001 | 0 | 0 | 0 | 0 |
| 233 | 0.121547 | 0.047037 | 0.690747 | 0.14066 | 0.00001 | 0 | 0 | 0 | 0 |
| 234 | 0.120271 | 0.047253 | 0.692003 | 0.140474 | 0 | 0 | 0 | 0 | 0 |
| 235 | 0.122953 | 0.046551 | 0.689006 | 0.141481 | 0.00001 | 0 | 0 | 0 | 0 |
| 236 | 0.121294 | 0.046685 | 0.6878 | 0.144211 | 0.00001 | 0 | 0 | 0 | 0 |
| 237 | 0.123365 | 0.046939 | 0.687459 | 0.142237 | 0 | 0 | 0 | 0 | 0 |
| 238 | 0.123672 | 0.048272 | 0.6847 | 0.143356 | 0 | 0 | 0 | 0 | 0 |
| 239 | 0.123738 | 0.048009 | 0.683886 | 0.144366 | 0 | 0 | 0 | 0 | 0 |
| 240 | 0.123041 | 0.046148 | 0.688061 | 0.14275 | 0 | 0 | 0 | 0 | 0 |
| 241 | 0.121109 | 0.047164 | 0.690004 | 0.141723 | 0 | 0 | 0 | 0 | 0 |
| 242 | 0.122298 | 0.046381 | 0.688358 | 0.142953 | 0.00001 | 0 | 0 | 0 | 0 |
| 243 | 0.12117 | 0.045704 | 0.69205 | 0.141066 | 0.00001 | 0 | 0 | 0 | 0 |
| 244 | 0.121426 | 0.046675 | 0.690822 | 0.141076 | 0 | 0 | 0 | 0 | 0 |
| 245 | 0.122417 | 0.046825 | 0.689384 | 0.141364 | 0.00001 | 0 | 0 | 0 | 0 |
| 246 | 0.122624 | 0.046554 | 0.689009 | 0.141814 | 0 | 0 | 0 | 0 | 0 |
| 247 | 0.12205 | 0.046523 | 0.686708 | 0.14472 | 0 | 0 | 0 | 0 | 0 |
| 248 | 0.121311 | 0.046917 | 0.69014 | 0.141632 | 0 | 0 | 0 | 0 | 0 |
| 249 | 0.121257 | 0.04634 | 0.690001 | 0.142401 | 0 | 0 | 0 | 0 | 0 |
| 250 | 0.120918 | 0.047012 | 0.687721 | 0.144339 | 0.00001 | 0 | 0 | 0 | 0 |
| 251 | 0.122685 | 0.046745 | 0.688528 | 0.142042 | 0 | 0 | 0 | 0 | 0 |
| 252 | 0.120375 | 0.047277 | 0.690677 | 0.141671 | 0 | 0 | 0 | 0 | 0 |
| 253 | 0.122271 | 0.048601 | 0.684984 | 0.144144 | 0 | 0 | 0 | 0 | 0 |
| 254 | 0.121742 | 0.047019 | 0.687351 | 0.143889 | 0 | 0 | 0 | 0 | 0 |
| 255 | 0.122946 | 0.046779 | 0.688582 | 0.141693 | 0 | 0 | 0 | 0 | 0 |
| 256 | 0.123088 | 0.04712 | 0.687388 | 0.142393 | 0.00001 | 0 | 0 | 0 | 0 |
| 257 | 0.122148 | 0.047181 | 0.686979 | 0.143681 | 0.00001 | 0 | 0 | 0 | 0 |
| 258 | 0.121632 | 0.047281 | 0.688072 | 0.143015 | 0 | 0 | 0 | 0 | 0 |
| 259 | 0.121945 | 0.047163 | 0.689244 | 0.141638 | 0.00001 | 0 | 0 | 0 | 0 |
| 260 | 0.120842 | 0.0471 | 0.690027 | 0.142021 | 0.00001 | 0 | 0 | 0 | 0 |
| 261 | 0.121594 | 0.046317 | 0.69013 | 0.141959 | 0 | 0 | 0 | 0 | 0 |
| 262 | 0.123067 | 0.046261 | 0.689011 | 0.141661 | 0 | 0 | 0 | 0 | 0 |
| 263 | 0.122083 | 0.047011 | 0.68841 | 0.142485 | 0.00001 | 0 | 0 | 0 | 0 |
| 264 | 0.12235 | 0.047142 | 0.687821 | 0.142688 | 0 | 0 | 0 | 0 | 0 |
| 265 | 0.12108 | 0.047594 | 0.688315 | 0.143011 | 0 | 0 | 0 | 0 | 0 |
| 266 | 0.123028 | 0.047113 | 0.687885 | 0.141974 | 0 | 0 | 0 | 0 | 0 |
| 267 | 0.122604 | 0.047071 | 0.688653 | 0.141673 | 0 | 0 | 0 | 0 | 0 |
| 268 | 0.12311 | 0.047302 | 0.689821 | 0.139767 | 0 | 0 | 0 | 0 | 0 |
| 269 | 0.122276 | 0.046589 | 0.687797 | 0.143337 | 0 | 0 | 0 | 0 | 0 |
| 270 | 0.122115 | 0.047048 | 0.688831 | 0.142006 | 0 | 0 | 0 | 0 | 0 |
| 271 | 0.121504 | 0.047401 | 0.690237 | 0.140848 | 0.00001 | 0 | 0 | 0 | 0 |
| 272 | 0.121825 | 0.046783 | 0.69018 | 0.141212 | 0 | 0 | 0 | 0 | 0 |
| 273 | 0.122427 | 0.047276 | 0.688777 | 0.14152 | 0 | 0 | 0 | 0 | 0 |
| 274 | 0.120313 | 0.047053 | 0.691885 | 0.140748 | 0 | 0 | 0 | 0 | 0 |
| 275 | 0.118698 | 0.047867 | 0.690759 | 0.142676 | 0 | 0 | 0 | 0 | 0 |
| 276 | 0.122964 | 0.046222 | 0.688239 | 0.142575 | 0 | 0 | 0 | 0 | 0 |
| 277 | 0.120494 | 0.048151 | 0.687121 | 0.144233 | 0 | 0 | 0 | 0 | 0 |
| 278 | 0.122641 | 0.047137 | 0.687671 | 0.142551 | 0 | 0 | 0 | 0 | 0 |
| 279 | 0.121486 | 0.046772 | 0.688073 | 0.143668 | 0 | 0 | 0 | 0 | 0 |
| 280 | 0.123721 | 0.048796 | 0.687247 | 0.140236 | 0 | 0 | 0 | 0 | 0 |
| 281 | 0.1226 | 0.046701 | 0.687638 | 0.143061 | 0 | 0 | 0 | 0 | 0 |
| 282 | 0.123521 | 0.046218 | 0.687091 | 0.143169 | 0 | 0 | 0 | 0 | 0 |
| 283 | 0.12317 | 0.047362 | 0.68832 | 0.141148 | 0 | 0 | 0 | 0 | 0 |
| 284 | 0.122251 | 0.046967 | 0.689881 | 0.140902 | 0 | 0 | 0 | 0 | 0 |
| 285 | 0.123749 | 0.047168 | 0.685265 | 0.143818 | 0 | 0 | 0 | 0 | 0 |
| 286 | 0.120449 | 0.048142 | 0.689315 | 0.142094 | 0 | 0 | 0 | 0 | 0 |
| 287 | 0.120541 | 0.047021 | 0.690087 | 0.142352 | 0 | 0 | 0 | 0 | 0 |
| 288 | 0.121256 | 0.047677 | 0.689237 | 0.14183 | 0 | 0 | 0 | 0 | 0 |
| 289 | 0.121946 | 0.047434 | 0.687014 | 0.143605 | 0 | 0 | 0 | 0 | 0 |
| 290 | 0.123127 | 0.047432 | 0.687772 | 0.14167 | 0 | 0 | 0 | 0 | 0 |
| 291 | 0.123511 | 0.046477 | 0.688737 | 0.141274 | 0 | 0 | 0 | 0 | 0 |
| 292 | 0.121414 | 0.045991 | 0.692159 | 0.140435 | 0 | 0 | 0 | 0 | 0 |
| 293 | 0.122647 | 0.046625 | 0.685755 | 0.144974 | 0 | 0 | 0 | 0 | 0 |
| 294 | 0.120487 | 0.047845 | 0.688261 | 0.143406 | 0 | 0 | 0 | 0 | 0 |
| 295 | 0.119853 | 0.04678 | 0.689828 | 0.143539 | 0 | 0 | 0 | 0 | 0 |
| 296 | 0.121865 | 0.047123 | 0.687753 | 0.143259 | 0 | 0 | 0 | 0 | 0 |
| 297 | 0.121326 | 0.047736 | 0.688734 | 0.142203 | 0 | 0 | 0 | 0 | 0 |
| 298 | 0.120803 | 0.047691 | 0.690693 | 0.140813 | 0 | 0 | 0 | 0 | 0 |
| 299 | 0.122875 | 0.046639 | 0.686679 | 0.143797 | 0.00001 | 0 | 0 | 0 | 0 |
| 300 | 0.123178 | 0.046114 | 0.688541 | 0.142167 | 0 | 0 | 0 | 0 | 0 |
| 301 | 0.124593 | 0.047142 | 0.686097 | 0.142168 | 0 | 0 | 0 | 0 | 0 |
| 302 | 0.122859 | 0.04611 | 0.688473 | 0.142559 | 0 | 0 | 0 | 0 | 0 |
| 303 | 0.122311 | 0.04686 | 0.689021 | 0.141807 | 0 | 0 | 0 | 0 | 0 |
| 304 | 0.122579 | 0.047207 | 0.68648 | 0.143735 | 0 | 0 | 0 | 0 | 0 |
| 305 | 0.123976 | 0.046464 | 0.687832 | 0.141728 | 0 | 0 | 0 | 0 | 0 |
| 306 | 0.123062 | 0.046801 | 0.688629 | 0.141489 | 0.00002 | 0 | 0 | 0 | 0 |
| 307 | 0.123448 | 0.046632 | 0.687424 | 0.142496 | 0 | 0 | 0 | 0 | 0 |
| 308 | 0.12106 | 0.047168 | 0.691197 | 0.140574 | 0 | 0 | 0 | 0 | 0 |
| 309 | 0.12338 | 0.046199 | 0.689645 | 0.140766 | 0.00001 | 0 | 0 | 0 | 0 |
| 310 | 0.122468 | 0.0461 | 0.689551 | 0.141881 | 0 | 0 | 0 | 0 | 0 |
| 311 | 0.121932 | 0.04678 | 0.690161 | 0.141127 | 0 | 0 | 0 | 0 | 0 |
| 312 | 0.121955 | 0.046759 | 0.688484 | 0.142801 | 0 | 0 | 0 | 0 | 0 |
| 313 | 0.121876 | 0.04604 | 0.689892 | 0.142192 | 0 | 0 | 0 | 0 | 0 |
| 314 | 0.12155 | 0.045759 | 0.691032 | 0.141659 | 0 | 0 | 0 | 0 | 0 |
| 315 | 0.121896 | 0.04643 | 0.687804 | 0.14387 | 0 | 0 | 0 | 0 | 0 |
| 316 | 0.121673 | 0.047148 | 0.691255 | 0.139924 | 0 | 0 | 0 | 0 | 0 |
| 317 | 0.121223 | 0.047153 | 0.690343 | 0.141281 | 0 | 0 | 0 | 0 | 0 |
| 318 | 0.121511 | 0.045689 | 0.689406 | 0.143383 | 0.00001 | 0 | 0 | 0 | 0 |
| 319 | 0.121727 | 0.046525 | 0.687311 | 0.144437 | 0 | 0 | 0 | 0 | 0 |
| 320 | 0.120805 | 0.047768 | 0.690497 | 0.14093 | 0 | 0 | 0 | 0 | 0 |
| 321 | 0.121576 | 0.046473 | 0.689259 | 0.142691 | 0 | 0 | 0 | 0 | 0 |
| 322 | 0.121409 | 0.045971 | 0.691996 | 0.140624 | 0 | 0 | 0 | 0 | 0 |
| 323 | 0.121198 | 0.046766 | 0.690876 | 0.14116 | 0 | 0 | 0 | 0 | 0 |
| 324 | 0.123073 | 0.047041 | 0.689194 | 0.140693 | 0 | 0 | 0 | 0 | 0 |
| 325 | 0.122167 | 0.046941 | 0.690839 | 0.140053 | 0 | 0 | 0 | 0 | 0 |
| 326 | 0.122659 | 0.046038 | 0.689224 | 0.142079 | 0 | 0 | 0 | 0 | 0 |
| 327 | 0.123165 | 0.047213 | 0.688343 | 0.141279 | 0 | 0 | 0 | 0 | 0 |
| 328 | 0.122898 | 0.046872 | 0.689016 | 0.141215 | 0 | 0 | 0 | 0 | 0 |
| 329 | 0.123819 | 0.0466 | 0.687895 | 0.141686 | 0 | 0 | 0 | 0 | 0 |
| 330 | 0.122509 | 0.046306 | 0.689071 | 0.142113 | 0 | 0 | 0 | 0 | 0 |
| 331 | 0.122612 | 0.046273 | 0.6899 | 0.141215 | 0 | 0 | 0 | 0 | 0 |
| 332 | 0.122167 | 0.046887 | 0.689857 | 0.14109 | 0 | 0 | 0 | 0 | 0 |
| 333 | 0.121955 | 0.046068 | 0.690339 | 0.141638 | 0 | 0 | 0 | 0 | 0 |
| 334 | 0.122535 | 0.047495 | 0.6899 | 0.14007 | 0 | 0 | 0 | 0 | 0 |
| 335 | 0.121448 | 0.047269 | 0.689089 | 0.142194 | 0 | 0 | 0 | 0 | 0 |
| 336 | 0.120927 | 0.047033 | 0.689293 | 0.142748 | 0 | 0 | 0 | 0 | 0 |
| 337 | 0.122724 | 0.047749 | 0.686138 | 0.143389 | 0 | 0 | 0 | 0 | 0 |
| 338 | 0.120688 | 0.04579 | 0.693942 | 0.13957 | 0.00001 | 0 | 0 | 0 | 0 |
| 339 | 0.123196 | 0.048029 | 0.686689 | 0.142086 | 0 | 0 | 0 | 0 | 0 |
| 340 | 0.124039 | 0.048317 | 0.685949 | 0.141695 | 0 | 0 | 0 | 0 | 0 |
| 341 | 0.122557 | 0.046729 | 0.687435 | 0.143279 | 0 | 0 | 0 | 0 | 0 |
| 342 | 0.122978 | 0.045725 | 0.687761 | 0.143536 | 0 | 0 | 0 | 0 | 0 |
| 343 | 0.122442 | 0.046698 | 0.690536 | 0.140324 | 0 | 0 | 0 | 0 | 0 |
| 344 | 0.123939 | 0.046798 | 0.687192 | 0.142071 | 0 | 0 | 0 | 0 | 0 |
| 345 | 0.122188 | 0.046743 | 0.687233 | 0.143836 | 0 | 0 | 0 | 0 | 0 |
| 346 | 0.123419 | 0.047513 | 0.689798 | 0.13927 | 0 | 0 | 0 | 0 | 0 |
| 347 | 0.121628 | 0.046907 | 0.690394 | 0.141061 | 0.00001 | 0 | 0 | 0 | 0 |
| 348 | 0.123108 | 0.047159 | 0.687388 | 0.142345 | 0 | 0 | 0 | 0 | 0 |
| 349 | 0.120888 | 0.046322 | 0.689491 | 0.143299 | 0 | 0 | 0 | 0 | 0 |
| 350 | 0.121101 | 0.047429 | 0.690542 | 0.140927 | 0 | 0 | 0 | 0 | 0 |
| 351 | 0.122984 | 0.047201 | 0.688211 | 0.141604 | 0 | 0 | 0 | 0 | 0 |
| 352 | 0.121584 | 0.046591 | 0.691971 | 0.139854 | 0 | 0 | 0 | 0 | 0 |
| 353 | 0.121681 | 0.047036 | 0.689541 | 0.141742 | 0 | 0 | 0 | 0 | 0 |
| 354 | 0.123695 | 0.047656 | 0.685802 | 0.142847 | 0 | 0 | 0 | 0 | 0 |
| 355 | 0.120721 | 0.04651 | 0.690576 | 0.142192 | 0 | 0 | 0 | 0 | 0 |
| 356 | 0.121466 | 0.046415 | 0.689792 | 0.142328 | 0 | 0 | 0 | 0 | 0 |
| 357 | 0.122971 | 0.047554 | 0.687323 | 0.142152 | 0 | 0 | 0 | 0 | 0 |
| 358 | 0.120416 | 0.04741 | 0.691771 | 0.140403 | 0 | 0 | 0 | 0 | 0 |
| 359 | 0.121391 | 0.046791 | 0.690723 | 0.141095 | 0 | 0 | 0 | 0 | 0 |
| 360 | 0.121789 | 0.047514 | 0.691438 | 0.139259 | 0 | 0 | 0 | 0 | 0 |
| 361 | 0.120741 | 0.046846 | 0.690073 | 0.14234 | 0 | 0 | 0 | 0 | 0 |
| 362 | 0.122998 | 0.047627 | 0.689113 | 0.140262 | 0 | 0 | 0 | 0 | 0 |
| 363 | 0.121797 | 0.04609 | 0.689453 | 0.142661 | 0 | 0 | 0 | 0 | 0 |
| 364 | 0.121805 | 0.047167 | 0.688187 | 0.142841 | 0 | 0 | 0 | 0 | 0 |
| 365 | 0.123535 | 0.046787 | 0.687303 | 0.142375 | 0 | 0 | 0 | 0 | 0 |
| 366 | 0.122085 | 0.047627 | 0.688353 | 0.141935 | 0 | 0 | 0 | 0 | 0 |
| 367 | 0.121961 | 0.046211 | 0.688353 | 0.143474 | 0 | 0 | 0 | 0 | 0 |
| 368 | 0.121262 | 0.046167 | 0.692011 | 0.14056 | 0 | 0 | 0 | 0 | 0 |
| 369 | 0.121795 | 0.0468 | 0.689408 | 0.141996 | 0 | 0 | 0 | 0 | 0 |
| 370 | 0.122577 | 0.046737 | 0.68757 | 0.143116 | 0 | 0 | 0 | 0 | 0 |
| 371 | 0.121888 | 0.046947 | 0.688053 | 0.143091 | 0.00002 | 0 | 0 | 0 | 0 |
| 372 | 0.121926 | 0.048122 | 0.687023 | 0.142929 | 0 | 0 | 0 | 0 | 0 |
| 373 | 0.120331 | 0.046438 | 0.68975 | 0.143481 | 0 | 0 | 0 | 0 | 0 |
| 374 | 0.122185 | 0.048149 | 0.688562 | 0.141104 | 0 | 0 | 0 | 0 | 0 |
| 375 | 0.120395 | 0.04684 | 0.689208 | 0.143556 | 0 | 0 | 0 | 0 | 0 |
| 376 | 0.122077 | 0.048264 | 0.687693 | 0.141966 | 0 | 0 | 0 | 0 | 0 |
| 377 | 0.119899 | 0.047663 | 0.687997 | 0.144441 | 0 | 0 | 0 | 0 | 0 |
| 378 | 0.122013 | 0.046774 | 0.690912 | 0.140302 | 0 | 0 | 0 | 0 | 0 |
| 379 | 0.12237 | 0.047521 | 0.687138 | 0.142971 | 0 | 0 | 0 | 0 | 0 |
| 380 | 0.123772 | 0.046956 | 0.687319 | 0.141954 | 0 | 0 | 0 | 0 | 0 |
| 381 | 0.122629 | 0.047447 | 0.687534 | 0.142391 | 0 | 0 | 0 | 0 | 0 |
| 382 | 0.123293 | 0.044994 | 0.688603 | 0.14311 | 0 | 0 | 0 | 0 | 0 |
| 383 | 0.120443 | 0.046272 | 0.692262 | 0.141023 | 0 | 0 | 0 | 0 | 0 |
| 384 | 0.122999 | 0.047142 | 0.687362 | 0.142497 | 0 | 0 | 0 | 0 | 0 |
| 385 | 0.122094 | 0.046513 | 0.689096 | 0.142297 | 0 | 0 | 0 | 0 | 0 |
| 386 | 0.122655 | 0.047489 | 0.689057 | 0.1408 | 0 | 0 | 0 | 0 | 0 |
| 387 | 0.120496 | 0.048088 | 0.690057 | 0.141359 | 0 | 0 | 0 | 0 | 0 |
| 388 | 0.123905 | 0.047377 | 0.687945 | 0.140773 | 0 | 0 | 0 | 0 | 0 |
| 389 | 0.122714 | 0.04732 | 0.688395 | 0.14157 | 0 | 0 | 0 | 0 | 0 |
| 390 | 0.121147 | 0.047429 | 0.690673 | 0.14075 | 0 | 0 | 0 | 0 | 0 |
| 391 | 0.12196 | 0.047784 | 0.686883 | 0.143372 | 0 | 0 | 0 | 0 | 0 |
| 392 | 0.123878 | 0.047391 | 0.68827 | 0.140461 | 0 | 0 | 0 | 0 | 0 |
| 393 | 0.121408 | 0.046564 | 0.687497 | 0.144531 | 0 | 0 | 0 | 0 | 0 |
| 394 | 0.123373 | 0.046776 | 0.687406 | 0.142445 | 0 | 0 | 0 | 0 | 0 |
| 395 | 0.122188 | 0.046022 | 0.689626 | 0.142164 | 0 | 0 | 0 | 0 | 0 |
| 396 | 0.12181 | 0.046104 | 0.68889 | 0.143195 | 0 | 0 | 0 | 0 | 0 |
| 397 | 0.123612 | 0.047573 | 0.6884 | 0.140415 | 0 | 0 | 0 | 0 | 0 |
| 398 | 0.121899 | 0.046857 | 0.688436 | 0.142807 | 0 | 0 | 0 | 0 | 0 |
| 399 | 0.121795 | 0.046761 | 0.688282 | 0.143163 | 0 | 0 | 0 | 0 | 0 |
| 400 | 0.123712 | 0.047084 | 0.688121 | 0.141082 | 0 | 0 | 0 | 0 | 0 |
| 401 | 0.120591 | 0.045453 | 0.692039 | 0.141917 | 0 | 0 | 0 | 0 | 0 |
| 402 | 0.120587 | 0.046298 | 0.689719 | 0.143396 | 0 | 0 | 0 | 0 | 0 |
| 403 | 0.124032 | 0.047959 | 0.687787 | 0.140221 | 0 | 0 | 0 | 0 | 0 |
| 404 | 0.121955 | 0.046781 | 0.690872 | 0.140382 | 0.00001 | 0 | 0 | 0 | 0 |
| 405 | 0.124052 | 0.046739 | 0.687881 | 0.141328 | 0 | 0 | 0 | 0 | 0 |
| 406 | 0.121639 | 0.047724 | 0.68828 | 0.142357 | 0 | 0 | 0 | 0 | 0 |
| 407 | 0.122932 | 0.046529 | 0.688832 | 0.141708 | 0 | 0 | 0 | 0 | 0 |
| 408 | 0.121166 | 0.047197 | 0.688225 | 0.143412 | 0 | 0 | 0 | 0 | 0 |
| 409 | 0.122277 | 0.047637 | 0.688464 | 0.141622 | 0 | 0 | 0 | 0 | 0 |
| 410 | 0.122291 | 0.047491 | 0.688595 | 0.141623 | 0 | 0 | 0 | 0 | 0 |
| 411 | 0.122787 | 0.04649 | 0.688531 | 0.142192 | 0 | 0 | 0 | 0 | 0 |
| 412 | 0.122014 | 0.046697 | 0.688407 | 0.142873 | 0.00001 | 0 | 0 | 0 | 0 |
| 413 | 0.122315 | 0.046065 | 0.688596 | 0.143024 | 0 | 0 | 0 | 0 | 0 |
| 414 | 0.122609 | 0.047394 | 0.688098 | 0.1419 | 0 | 0 | 0 | 0 | 0 |
| 415 | 0.123326 | 0.046794 | 0.689007 | 0.140873 | 0 | 0 | 0 | 0 | 0 |
| 416 | 0.123849 | 0.046436 | 0.687611 | 0.142094 | 0.00001 | 0 | 0 | 0 | 0 |
| 417 | 0.121742 | 0.045841 | 0.690828 | 0.141589 | 0 | 0 | 0 | 0 | 0 |
| 418 | 0.120126 | 0.046274 | 0.693227 | 0.140373 | 0 | 0 | 0 | 0 | 0 |
| 419 | 0.122718 | 0.04713 | 0.688332 | 0.141819 | 0 | 0 | 0 | 0 | 0 |
| 420 | 0.121995 | 0.04745 | 0.687683 | 0.142873 | 0 | 0 | 0 | 0 | 0 |
| 421 | 0.121585 | 0.047858 | 0.688757 | 0.1418 | 0 | 0 | 0 | 0 | 0 |
| 422 | 0.122824 | 0.046858 | 0.688832 | 0.141487 | 0 | 0 | 0 | 0 | 0 |
| 423 | 0.121565 | 0.046977 | 0.689749 | 0.141709 | 0 | 0 | 0 | 0 | 0 |
| 424 | 0.122279 | 0.047385 | 0.68847 | 0.141855 | 0.00001 | 0 | 0 | 0 | 0 |
| 425 | 0.123883 | 0.04673 | 0.687167 | 0.14222 | 0 | 0 | 0 | 0 | 0 |
| 426 | 0.12167 | 0.047337 | 0.688069 | 0.142914 | 0.00001 | 0 | 0 | 0 | 0 |
| 427 | 0.121654 | 0.04824 | 0.689325 | 0.14078 | 0 | 0 | 0 | 0 | 0 |
| 428 | 0.120185 | 0.046125 | 0.693035 | 0.140655 | 0 | 0 | 0 | 0 | 0 |
| 429 | 0.122054 | 0.046881 | 0.689561 | 0.141504 | 0 | 0 | 0 | 0 | 0 |
| 430 | 0.121273 | 0.046863 | 0.68907 | 0.142794 | 0 | 0 | 0 | 0 | 0 |
| 431 | 0.120439 | 0.046571 | 0.69117 | 0.14182 | 0 | 0 | 0 | 0 | 0 |
| 432 | 0.120453 | 0.046862 | 0.688831 | 0.143854 | 0 | 0 | 0 | 0 | 0 |
| 433 | 0.122912 | 0.046351 | 0.688792 | 0.141945 | 0 | 0 | 0 | 0 | 0 |
| 434 | 0.121623 | 0.045263 | 0.691049 | 0.142065 | 0 | 0 | 0 | 0 | 0 |
| 435 | 0.1219 | 0.04684 | 0.69011 | 0.141149 | 0 | 0 | 0 | 0 | 0 |
| 436 | 0.121853 | 0.046177 | 0.689138 | 0.142832 | 0 | 0 | 0 | 0 | 0 |
| 437 | 0.123794 | 0.046588 | 0.687979 | 0.141639 | 0 | 0 | 0 | 0 | 0 |
| 438 | 0.122314 | 0.047906 | 0.68589 | 0.143889 | 0 | 0 | 0 | 0 | 0 |
| 439 | 0.122224 | 0.047423 | 0.687604 | 0.142748 | 0 | 0 | 0 | 0 | 0 |
| 440 | 0.121809 | 0.046623 | 0.690055 | 0.141513 | 0 | 0 | 0 | 0 | 0 |
| 441 | 0.121663 | 0.046463 | 0.690833 | 0.14104 | 0 | 0 | 0 | 0 | 0 |
| 442 | 0.122253 | 0.046986 | 0.688013 | 0.142748 | 0 | 0 | 0 | 0 | 0 |
| 443 | 0.123528 | 0.046576 | 0.686448 | 0.143449 | 0 | 0 | 0 | 0 | 0 |
| 444 | 0.122914 | 0.047605 | 0.687087 | 0.142394 | 0 | 0 | 0 | 0 | 0 |
| 445 | 0.12233 | 0.046048 | 0.688271 | 0.143351 | 0 | 0 | 0 | 0 | 0 |
| 446 | 0.122053 | 0.046735 | 0.687373 | 0.143838 | 0 | 0 | 0 | 0 | 0 |
| 447 | 0.120695 | 0.047409 | 0.688839 | 0.143047 | 0.00001 | 0 | 0 | 0 | 0 |
| 448 | 0.12264 | 0.048458 | 0.687391 | 0.141511 | 0 | 0 | 0 | 0 | 0 |
| 449 | 0.12269 | 0.047067 | 0.688072 | 0.142172 | 0 | 0 | 0 | 0 | 0 |
| 450 | 0.124434 | 0.045856 | 0.689556 | 0.140154 | 0 | 0 | 0 | 0 | 0 |
| 451 | 0.123048 | 0.047162 | 0.688295 | 0.141495 | 0 | 0 | 0 | 0 | 0 |
| 452 | 0.12347 | 0.046383 | 0.689426 | 0.140721 | 0 | 0 | 0 | 0 | 0 |
| 453 | 0.122769 | 0.047672 | 0.687779 | 0.14178 | 0 | 0 | 0 | 0 | 0 |
| 454 | 0.119996 | 0.045669 | 0.69187 | 0.142465 | 0 | 0 | 0 | 0 | 0 |
| 455 | 0.122212 | 0.046514 | 0.690501 | 0.140773 | 0 | 0 | 0 | 0 | 0 |
| 456 | 0.120702 | 0.047954 | 0.688262 | 0.143082 | 0 | 0 | 0 | 0 | 0 |
| 457 | 0.119952 | 0.046598 | 0.689678 | 0.143762 | 0.00001 | 0 | 0 | 0 | 0 |
| 458 | 0.122719 | 0.047499 | 0.689736 | 0.140046 | 0 | 0 | 0 | 0 | 0 |
| 459 | 0.120994 | 0.046983 | 0.687986 | 0.144036 | 0 | 0 | 0 | 0 | 0 |
| 460 | 0.121642 | 0.047928 | 0.685528 | 0.144902 | 0 | 0 | 0 | 0 | 0 |
| 461 | 0.120661 | 0.047194 | 0.690732 | 0.141413 | 0 | 0 | 0 | 0 | 0 |
| 462 | 0.122276 | 0.046734 | 0.690536 | 0.140454 | 0 | 0 | 0 | 0 | 0 |
| 463 | 0.120762 | 0.047258 | 0.689574 | 0.142405 | 0 | 0 | 0 | 0 | 0 |
| 464 | 0.121107 | 0.04762 | 0.690129 | 0.141134 | 0.00001 | 0 | 0 | 0 | 0 |
| 465 | 0.1217 | 0.046214 | 0.690468 | 0.141618 | 0 | 0 | 0 | 0 | 0 |
| 466 | 0.121186 | 0.047926 | 0.687149 | 0.143739 | 0 | 0 | 0 | 0 | 0 |
| 467 | 0.123379 | 0.04617 | 0.687203 | 0.143247 | 0 | 0 | 0 | 0 | 0 |
| 468 | 0.121139 | 0.046816 | 0.68917 | 0.142876 | 0 | 0 | 0 | 0 | 0 |
| 469 | 0.121219 | 0.047263 | 0.690048 | 0.14147 | 0 | 0 | 0 | 0 | 0 |
| 470 | 0.120696 | 0.046165 | 0.69249 | 0.14065 | 0 | 0 | 0 | 0 | 0 |
| 471 | 0.12356 | 0.047739 | 0.689033 | 0.139669 | 0 | 0 | 0 | 0 | 0 |
| 472 | 0.122816 | 0.047094 | 0.688969 | 0.141121 | 0 | 0 | 0 | 0 | 0 |
| 473 | 0.123176 | 0.047067 | 0.687812 | 0.141945 | 0 | 0 | 0 | 0 | 0 |
| 474 | 0.12183 | 0.046658 | 0.689773 | 0.141739 | 0 | 0 | 0 | 0 | 0 |
| 475 | 0.12226 | 0.046082 | 0.688416 | 0.143242 | 0 | 0 | 0 | 0 | 0 |
| 476 | 0.122615 | 0.04677 | 0.687829 | 0.142786 | 0 | 0 | 0 | 0 | 0 |
| 477 | 0.121595 | 0.047388 | 0.688901 | 0.142115 | 0 | 0 | 0 | 0 | 0 |
| 478 | 0.121413 | 0.046938 | 0.687558 | 0.144091 | 0 | 0 | 0 | 0 | 0 |
| 479 | 0.123456 | 0.047569 | 0.689203 | 0.139773 | 0 | 0 | 0 | 0 | 0 |
| 480 | 0.121879 | 0.046956 | 0.686358 | 0.144807 | 0 | 0 | 0 | 0 | 0 |
| 481 | 0.120712 | 0.047428 | 0.692412 | 0.139448 | 0 | 0 | 0 | 0 | 0 |
| 482 | 0.122289 | 0.047147 | 0.687924 | 0.14264 | 0 | 0 | 0 | 0 | 0 |
| 483 | 0.121786 | 0.047194 | 0.689519 | 0.141501 | 0 | 0 | 0 | 0 | 0 |
| 484 | 0.121741 | 0.047048 | 0.688627 | 0.142584 | 0 | 0 | 0 | 0 | 0 |
| 485 | 0.119178 | 0.047211 | 0.691052 | 0.142559 | 0 | 0 | 0 | 0 | 0 |
| 486 | 0.11998 | 0.047688 | 0.689608 | 0.142724 | 0 | 0 | 0 | 0 | 0 |
| 487 | 0.122084 | 0.047337 | 0.689025 | 0.141553 | 0 | 0 | 0 | 0 | 0 |
| 488 | 0.121556 | 0.048051 | 0.687267 | 0.143125 | 0 | 0 | 0 | 0 | 0 |
| 489 | 0.122125 | 0.046157 | 0.689434 | 0.142284 | 0 | 0 | 0 | 0 | 0 |
| 490 | 0.120734 | 0.04703 | 0.690117 | 0.142119 | 0 | 0 | 0 | 0 | 0 |
| 491 | 0.122377 | 0.046966 | 0.687507 | 0.14315 | 0 | 0 | 0 | 0 | 0 |
| 492 | 0.120848 | 0.04736 | 0.687339 | 0.144453 | 0 | 0 | 0 | 0 | 0 |
| 493 | 0.122875 | 0.046171 | 0.689737 | 0.141217 | 0 | 0 | 0 | 0 | 0 |
| 494 | 0.120409 | 0.047112 | 0.690065 | 0.142414 | 0 | 0 | 0 | 0 | 0 |
| 495 | 0.123332 | 0.047783 | 0.688097 | 0.140789 | 0 | 0 | 0 | 0 | 0 |
| 496 | 0.120756 | 0.046425 | 0.690197 | 0.142612 | 0.00001 | 0 | 0 | 0 | 0 |
| 497 | 0.122627 | 0.046791 | 0.689801 | 0.140771 | 0.00001 | 0 | 0 | 0 | 0 |
| 498 | 0.121973 | 0.04752 | 0.68881 | 0.141697 | 0 | 0 | 0 | 0 | 0 |
| 499 | 0.120738 | 0.046832 | 0.689671 | 0.142759 | 0 | 0 | 0 | 0 | 0 |
| 500 | 0.122374 | 0.04729 | 0.688024 | 0.142312 | 0 | 0 | 0 | 0 | 0 |
| 501 | 0.12346 | 0.047883 | 0.686936 | 0.141722 | 0 | 0 | 0 | 0 | 0 |
| 502 | 0.123955 | 0.046755 | 0.687621 | 0.141669 | 0 | 0 | 0 | 0 | 0 |
| 503 | 0.122119 | 0.046174 | 0.689028 | 0.142679 | 0 | 0 | 0 | 0 | 0 |
| 504 | 0.124956 | 0.048232 | 0.685276 | 0.141535 | 0 | 0 | 0 | 0 | 0 |
| 505 | 0.123928 | 0.046111 | 0.69073 | 0.139221 | 0.00001 | 0 | 0 | 0 | 0 |
| 506 | 0.122443 | 0.04699 | 0.689178 | 0.141389 | 0 | 0 | 0 | 0 | 0 |
| 507 | 0.121328 | 0.047383 | 0.688999 | 0.142289 | 0 | 0 | 0 | 0 | 0 |
| 508 | 0.121315 | 0.045608 | 0.691839 | 0.141238 | 0 | 0 | 0 | 0 | 0 |
| 509 | 0.122878 | 0.045707 | 0.691223 | 0.140192 | 0 | 0 | 0 | 0 | 0 |
| 510 | 0.122958 | 0.045787 | 0.6899 | 0.141354 | 0 | 0 | 0 | 0 | 0 |
| 511 | 0.122955 | 0.04723 | 0.685557 | 0.144257 | 0 | 0 | 0 | 0 | 0 |
| 512 | 0.120927 | 0.048819 | 0.687456 | 0.142798 | 0 | 0 | 0 | 0 | 0 |
| 513 | 0.123796 | 0.046289 | 0.688775 | 0.14114 | 0 | 0 | 0 | 0 | 0 |
| 514 | 0.122305 | 0.046347 | 0.689426 | 0.141922 | 0 | 0 | 0 | 0 | 0 |
| 515 | 0.122061 | 0.045539 | 0.690671 | 0.141728 | 0 | 0 | 0 | 0 | 0 |
| 516 | 0.122734 | 0.04652 | 0.688759 | 0.141988 | 0 | 0 | 0 | 0 | 0 |
| 517 | 0.122278 | 0.045151 | 0.690507 | 0.142065 | 0 | 0 | 0 | 0 | 0 |
| 518 | 0.121827 | 0.047569 | 0.687807 | 0.142787 | 0.00001 | 0 | 0 | 0 | 0 |
| 519 | 0.120818 | 0.047186 | 0.687706 | 0.144281 | 0.00001 | 0 | 0 | 0 | 0 |
| 520 | 0.121383 | 0.046115 | 0.688741 | 0.143751 | 0.00001 | 0 | 0 | 0 | 0 |
| 521 | 0.119858 | 0.04669 | 0.693182 | 0.14027 | 0 | 0 | 0 | 0 | 0 |
| 522 | 0.122572 | 0.045757 | 0.691942 | 0.139729 | 0 | 0 | 0 | 0 | 0 |
| 523 | 0.12222 | 0.046114 | 0.68959 | 0.142076 | 0 | 0 | 0 | 0 | 0 |
| 524 | 0.121947 | 0.046737 | 0.687321 | 0.143996 | 0 | 0 | 0 | 0 | 0 |
| 525 | 0.122239 | 0.04715 | 0.689659 | 0.140951 | 0 | 0 | 0 | 0 | 0 |
| 526 | 0.122956 | 0.046579 | 0.687973 | 0.142492 | 0 | 0 | 0 | 0 | 0 |
| 527 | 0.122448 | 0.047115 | 0.689012 | 0.141425 | 0 | 0 | 0 | 0 | 0 |
| 528 | 0.123662 | 0.047858 | 0.687284 | 0.141196 | 0 | 0 | 0 | 0 | 0 |
| 529 | 0.122557 | 0.047144 | 0.688377 | 0.141923 | 0 | 0 | 0 | 0 | 0 |
| 530 | 0.121061 | 0.047882 | 0.688107 | 0.14295 | 0 | 0 | 0 | 0 | 0 |
| 531 | 0.122498 | 0.046692 | 0.686852 | 0.143958 | 0 | 0 | 0 | 0 | 0 |
| 532 | 0.1223 | 0.048087 | 0.689553 | 0.14006 | 0 | 0 | 0 | 0 | 0 |
| 533 | 0.121842 | 0.047977 | 0.688943 | 0.141238 | 0 | 0 | 0 | 0 | 0 |
| 534 | 0.122128 | 0.047232 | 0.686553 | 0.144076 | 0.00001 | 0 | 0 | 0 | 0 |
| 535 | 0.12223 | 0.046532 | 0.68918 | 0.142048 | 0.00001 | 0 | 0 | 0 | 0 |
| 536 | 0.121257 | 0.047862 | 0.689135 | 0.141736 | 0.00001 | 0 | 0 | 0 | 0 |
| 537 | 0.12255 | 0.046136 | 0.689823 | 0.141491 | 0 | 0 | 0 | 0 | 0 |
| 538 | 0.121984 | 0.046477 | 0.688601 | 0.142927 | 0.00001 | 0 | 0 | 0 | 0 |
| 539 | 0.12171 | 0.046999 | 0.689048 | 0.142243 | 0 | 0 | 0 | 0 | 0 |
| 540 | 0.123807 | 0.046997 | 0.686793 | 0.142402 | 0 | 0 | 0 | 0 | 0 |
| 541 | 0.121628 | 0.045638 | 0.689511 | 0.143224 | 0 | 0 | 0 | 0 | 0 |
| 542 | 0.121011 | 0.046751 | 0.68946 | 0.142778 | 0 | 0 | 0 | 0 | 0 |
| 543 | 0.122755 | 0.046956 | 0.690771 | 0.139519 | 0 | 0 | 0 | 0 | 0 |
| 544 | 0.122475 | 0.046675 | 0.689996 | 0.140845 | 0.00001 | 0 | 0 | 0 | 0 |
| 545 | 0.124017 | 0.047657 | 0.686804 | 0.141513 | 0.00001 | 0 | 0 | 0 | 0 |
| 546 | 0.120173 | 0.047244 | 0.68905 | 0.143533 | 0 | 0 | 0 | 0 | 0 |
| 547 | 0.123425 | 0.047017 | 0.688746 | 0.140811 | 0 | 0 | 0 | 0 | 0 |
| 548 | 0.121403 | 0.046818 | 0.691095 | 0.140684 | 0 | 0 | 0 | 0 | 0 |
| 549 | 0.122546 | 0.047449 | 0.686842 | 0.143163 | 0 | 0 | 0 | 0 | 0 |
| 550 | 0.122082 | 0.046832 | 0.68998 | 0.141106 | 0 | 0 | 0 | 0 | 0 |
| 551 | 0.122242 | 0.048314 | 0.688522 | 0.140921 | 0 | 0 | 0 | 0 | 0 |
| 552 | 0.123278 | 0.047458 | 0.688584 | 0.14068 | 0 | 0 | 0 | 0 | 0 |
| 553 | 0.122606 | 0.046687 | 0.687345 | 0.143362 | 0 | 0 | 0 | 0 | 0 |
| 554 | 0.121173 | 0.046941 | 0.690305 | 0.141571 | 0.00001 | 0 | 0 | 0 | 0 |
| 555 | 0.11992 | 0.046769 | 0.692544 | 0.140767 | 0 | 0 | 0 | 0 | 0 |
| 556 | 0.121716 | 0.047185 | 0.689764 | 0.141335 | 0 | 0 | 0 | 0 | 0 |
| 557 | 0.123188 | 0.047516 | 0.688506 | 0.14079 | 0 | 0 | 0 | 0 | 0 |
| 558 | 0.123114 | 0.046308 | 0.687842 | 0.142735 | 0 | 0 | 0 | 0 | 0 |
| 559 | 0.120078 | 0.045642 | 0.691282 | 0.142998 | 0 | 0 | 0 | 0 | 0 |
| 560 | 0.121421 | 0.046763 | 0.690342 | 0.141474 | 0 | 0 | 0 | 0 | 0 |
| 561 | 0.12292 | 0.046796 | 0.685872 | 0.144412 | 0 | 0 | 0 | 0 | 0 |
| 562 | 0.120422 | 0.047335 | 0.688861 | 0.143381 | 0 | 0 | 0 | 0 | 0 |
| 563 | 0.121864 | 0.046832 | 0.688804 | 0.1425 | 0 | 0 | 0 | 0 | 0 |
| 564 | 0.122738 | 0.046274 | 0.690274 | 0.140715 | 0 | 0 | 0 | 0 | 0 |
| 565 | 0.123043 | 0.046242 | 0.687136 | 0.143579 | 0 | 0 | 0 | 0 | 0 |
| 566 | 0.121835 | 0.047862 | 0.686516 | 0.143786 | 0 | 0 | 0 | 0 | 0 |
| 567 | 0.121312 | 0.046806 | 0.690063 | 0.14181 | 0.00001 | 0 | 0 | 0 | 0 |
| 568 | 0.123144 | 0.046124 | 0.689677 | 0.141045 | 0.00001 | 0 | 0 | 0 | 0 |
| 569 | 0.120692 | 0.046325 | 0.691185 | 0.141788 | 0.00001 | 0 | 0 | 0 | 0 |
| 570 | 0.123279 | 0.046126 | 0.687062 | 0.143533 | 0 | 0 | 0 | 0 | 0 |
| 571 | 0.122068 | 0.046495 | 0.689459 | 0.141978 | 0 | 0 | 0 | 0 | 0 |
| 572 | 0.121217 | 0.045117 | 0.690601 | 0.143066 | 0 | 0 | 0 | 0 | 0 |
| 573 | 0.122841 | 0.046604 | 0.689707 | 0.140847 | 0 | 0 | 0 | 0 | 0 |
| 574 | 0.122365 | 0.046962 | 0.688628 | 0.142035 | 0.00001 | 0 | 0 | 0 | 0 |
| 575 | 0.120562 | 0.045992 | 0.691162 | 0.142284 | 0 | 0 | 0 | 0 | 0 |
| 576 | 0.121866 | 0.046164 | 0.691206 | 0.140764 | 0 | 0 | 0 | 0 | 0 |
| 577 | 0.122286 | 0.046709 | 0.687514 | 0.143491 | 0 | 0 | 0 | 0 | 0 |
| 578 | 0.122241 | 0.046642 | 0.689922 | 0.141195 | 0 | 0 | 0 | 0 | 0 |
| 579 | 0.122917 | 0.045317 | 0.690171 | 0.141586 | 0.00001 | 0 | 0 | 0 | 0 |
| 580 | 0.121835 | 0.04715 | 0.688741 | 0.142273 | 0 | 0 | 0 | 0 | 0 |
| 581 | 0.119084 | 0.047925 | 0.690443 | 0.142547 | 0 | 0 | 0 | 0 | 0 |
| 582 | 0.123135 | 0.047653 | 0.688397 | 0.140815 | 0 | 0 | 0 | 0 | 0 |
| 583 | 0.120834 | 0.046896 | 0.689596 | 0.142675 | 0 | 0 | 0 | 0 | 0 |
| 584 | 0.121971 | 0.047103 | 0.689258 | 0.141669 | 0 | 0 | 0 | 0 | 0 |
| 585 | 0.121255 | 0.0475 | 0.688255 | 0.14299 | 0 | 0 | 0 | 0 | 0 |
| 586 | 0.121315 | 0.047597 | 0.687276 | 0.143812 | 0 | 0 | 0 | 0 | 0 |
| 587 | 0.122579 | 0.045972 | 0.69052 | 0.140928 | 0 | 0 | 0 | 0 | 0 |
| 588 | 0.121264 | 0.047671 | 0.686468 | 0.144598 | 0 | 0 | 0 | 0 | 0 |
| 589 | 0.121019 | 0.046926 | 0.691096 | 0.140959 | 0 | 0 | 0 | 0 | 0 |
| 590 | 0.120295 | 0.046482 | 0.689085 | 0.144138 | 0 | 0 | 0 | 0 | 0 |
| 591 | 0.123512 | 0.045712 | 0.689299 | 0.141477 | 0 | 0 | 0 | 0 | 0 |
| 592 | 0.123861 | 0.046837 | 0.688551 | 0.140751 | 0 | 0 | 0 | 0 | 0 |
| 593 | 0.123223 | 0.045963 | 0.688908 | 0.141906 | 0 | 0 | 0 | 0 | 0 |
| 594 | 0.121187 | 0.045048 | 0.691097 | 0.142668 | 0 | 0 | 0 | 0 | 0 |
| 595 | 0.122466 | 0.046644 | 0.690126 | 0.140764 | 0 | 0 | 0 | 0 | 0 |
| 596 | 0.12204 | 0.046693 | 0.68996 | 0.141307 | 0 | 0 | 0 | 0 | 0 |
| 597 | 0.121455 | 0.047429 | 0.687225 | 0.143891 | 0 | 0 | 0 | 0 | 0 |
| 598 | 0.119343 | 0.048002 | 0.690341 | 0.142313 | 0 | 0 | 0 | 0 | 0 |
| 599 | 0.121602 | 0.046994 | 0.687867 | 0.143536 | 0 | 0 | 0 | 0 | 0 |
| 600 | 0.121968 | 0.047314 | 0.687627 | 0.143081 | 0.00001 | 0 | 0 | 0 | 0 |
| 601 | 0.121424 | 0.046569 | 0.689662 | 0.142345 | 0 | 0 | 0 | 0 | 0 |
| 602 | 0.122339 | 0.046795 | 0.687969 | 0.142897 | 0 | 0 | 0 | 0 | 0 |
| 603 | 0.121825 | 0.047769 | 0.687438 | 0.142967 | 0 | 0 | 0 | 0 | 0 |
| 604 | 0.124047 | 0.047696 | 0.687919 | 0.140338 | 0 | 0 | 0 | 0 | 0 |
| 605 | 0.122002 | 0.046207 | 0.689408 | 0.142383 | 0 | 0 | 0 | 0 | 0 |
| 606 | 0.122524 | 0.047442 | 0.687929 | 0.142106 | 0 | 0 | 0 | 0 | 0 |
| 607 | 0.12172 | 0.04887 | 0.687986 | 0.141424 | 0 | 0 | 0 | 0 | 0 |
| 608 | 0.120356 | 0.047442 | 0.689563 | 0.142638 | 0 | 0 | 0 | 0 | 0 |
| 609 | 0.12208 | 0.047092 | 0.689163 | 0.141666 | 0 | 0 | 0 | 0 | 0 |
| 610 | 0.121259 | 0.04745 | 0.690802 | 0.14049 | 0 | 0 | 0 | 0 | 0 |
| 611 | 0.122234 | 0.046968 | 0.688513 | 0.142285 | 0 | 0 | 0 | 0 | 0 |
| 612 | 0.123016 | 0.047038 | 0.686338 | 0.143608 | 0 | 0 | 0 | 0 | 0 |
| 613 | 0.12334 | 0.046275 | 0.687844 | 0.142531 | 0.00001 | 0 | 0 | 0 | 0 |
| 614 | 0.123295 | 0.04787 | 0.689391 | 0.139444 | 0 | 0 | 0 | 0 | 0 |
| 615 | 0.121498 | 0.046741 | 0.690948 | 0.140803 | 0.00001 | 0 | 0 | 0 | 0 |
| 616 | 0.120971 | 0.047701 | 0.689507 | 0.141821 | 0 | 0 | 0 | 0 | 0 |
| 617 | 0.122542 | 0.047587 | 0.688164 | 0.141707 | 0 | 0 | 0 | 0 | 0 |
| 618 | 0.123171 | 0.046734 | 0.687861 | 0.142235 | 0 | 0 | 0 | 0 | 0 |
| 619 | 0.119378 | 0.045883 | 0.691567 | 0.143173 | 0 | 0 | 0 | 0 | 0 |
| 620 | 0.121187 | 0.046399 | 0.690062 | 0.142352 | 0 | 0 | 0 | 0 | 0 |
| 621 | 0.124553 | 0.048078 | 0.686944 | 0.140426 | 0 | 0 | 0 | 0 | 0 |
| 622 | 0.122387 | 0.047115 | 0.686305 | 0.144194 | 0 | 0 | 0 | 0 | 0 |
| 623 | 0.120787 | 0.047029 | 0.691846 | 0.140338 | 0 | 0 | 0 | 0 | 0 |
| 624 | 0.121517 | 0.046571 | 0.689889 | 0.142023 | 0 | 0 | 0 | 0 | 0 |
| 625 | 0.122377 | 0.047288 | 0.689011 | 0.141314 | 0.00001 | 0 | 0 | 0 | 0 |
| 626 | 0.120814 | 0.047677 | 0.689925 | 0.141584 | 0 | 0 | 0 | 0 | 0 |
| 627 | 0.121813 | 0.046428 | 0.690548 | 0.141211 | 0 | 0 | 0 | 0 | 0 |
| 628 | 0.120361 | 0.04603 | 0.689625 | 0.143984 | 0 | 0 | 0 | 0 | 0 |
| 629 | 0.122179 | 0.047241 | 0.689298 | 0.141282 | 0 | 0 | 0 | 0 | 0 |
| 630 | 0.12086 | 0.046717 | 0.68893 | 0.143493 | 0 | 0 | 0 | 0 | 0 |
| 631 | 0.122232 | 0.047728 | 0.687105 | 0.142935 | 0 | 0 | 0 | 0 | 0 |
| 632 | 0.12442 | 0.047362 | 0.686689 | 0.141529 | 0 | 0 | 0 | 0 | 0 |
| 633 | 0.120612 | 0.046369 | 0.689052 | 0.143966 | 0 | 0 | 0 | 0 | 0 |
| 634 | 0.122618 | 0.047148 | 0.688598 | 0.141636 | 0 | 0 | 0 | 0 | 0 |
| 635 | 0.121562 | 0.046715 | 0.687999 | 0.143714 | 0.00001 | 0 | 0 | 0 | 0 |
| 636 | 0.124258 | 0.046963 | 0.686352 | 0.142427 | 0 | 0 | 0 | 0 | 0 |
| 637 | 0.121047 | 0.046027 | 0.690102 | 0.142824 | 0 | 0 | 0 | 0 | 0 |
| 638 | 0.122774 | 0.045879 | 0.689319 | 0.142028 | 0 | 0 | 0 | 0 | 0 |
| 639 | 0.122739 | 0.047054 | 0.686801 | 0.143407 | 0 | 0 | 0 | 0 | 0 |
| 640 | 0.12213 | 0.047504 | 0.686921 | 0.143445 | 0 | 0 | 0 | 0 | 0 |
| 641 | 0.122539 | 0.047298 | 0.689775 | 0.140388 | 0 | 0 | 0 | 0 | 0 |
| 642 | 0.122516 | 0.046388 | 0.688299 | 0.142797 | 0 | 0 | 0 | 0 | 0 |
| 643 | 0.121275 | 0.046799 | 0.690918 | 0.141008 | 0 | 0 | 0 | 0 | 0 |
| 644 | 0.121825 | 0.047234 | 0.68997 | 0.140971 | 0 | 0 | 0 | 0 | 0 |
| 645 | 0.120906 | 0.048846 | 0.687996 | 0.142242 | 0.00001 | 0 | 0 | 0 | 0 |
| 646 | 0.122121 | 0.046543 | 0.688955 | 0.142382 | 0 | 0 | 0 | 0 | 0 |
| 647 | 0.122673 | 0.046917 | 0.689999 | 0.140411 | 0 | 0 | 0 | 0 | 0 |
| 648 | 0.121835 | 0.047635 | 0.688186 | 0.142344 | 0 | 0 | 0 | 0 | 0 |
| 649 | 0.122235 | 0.047461 | 0.68842 | 0.141883 | 0 | 0 | 0 | 0 | 0 |
| 650 | 0.122725 | 0.046928 | 0.688632 | 0.141715 | 0 | 0 | 0 | 0 | 0 |
| 651 | 0.122619 | 0.047055 | 0.688869 | 0.141457 | 0 | 0 | 0 | 0 | 0 |
| 652 | 0.1217 | 0.047016 | 0.688559 | 0.142724 | 0 | 0 | 0 | 0 | 0 |
| 653 | 0.121192 | 0.045972 | 0.691124 | 0.141712 | 0 | 0 | 0 | 0 | 0 |
| 654 | 0.122215 | 0.047759 | 0.688865 | 0.141151 | 0.00001 | 0 | 0 | 0 | 0 |
| 655 | 0.1221 | 0.046388 | 0.688353 | 0.143159 | 0 | 0 | 0 | 0 | 0 |
| 656 | 0.122085 | 0.047281 | 0.68945 | 0.141184 | 0 | 0 | 0 | 0 | 0 |
| 657 | 0.1208 | 0.047071 | 0.692344 | 0.139786 | 0 | 0 | 0 | 0 | 0 |
| 658 | 0.121366 | 0.046071 | 0.688876 | 0.143688 | 0 | 0 | 0 | 0 | 0 |
| 659 | 0.120094 | 0.047155 | 0.688675 | 0.144075 | 0 | 0 | 0 | 0 | 0 |
| 660 | 0.122337 | 0.047193 | 0.688911 | 0.141559 | 0 | 0 | 0 | 0 | 0 |
| 661 | 0.122584 | 0.047257 | 0.687146 | 0.143013 | 0 | 0 | 0 | 0 | 0 |
| 662 | 0.121329 | 0.047342 | 0.688962 | 0.142357 | 0.00001 | 0 | 0 | 0 | 0 |
| 663 | 0.122202 | 0.045879 | 0.689147 | 0.142772 | 0 | 0 | 0 | 0 | 0 |
| 664 | 0.122732 | 0.046323 | 0.690383 | 0.140552 | 0.00001 | 0 | 0 | 0 | 0 |
| 665 | 0.123383 | 0.047248 | 0.685949 | 0.143421 | 0 | 0 | 0 | 0 | 0 |
| 666 | 0.120835 | 0.048206 | 0.688154 | 0.142804 | 0 | 0 | 0 | 0 | 0 |
| 667 | 0.123557 | 0.047394 | 0.687087 | 0.141962 | 0 | 0 | 0 | 0 | 0 |
| 668 | 0.121957 | 0.046012 | 0.688721 | 0.143311 | 0 | 0 | 0 | 0 | 0 |
| 669 | 0.120939 | 0.047121 | 0.687366 | 0.144574 | 0 | 0 | 0 | 0 | 0 |
| 670 | 0.12232 | 0.044973 | 0.689145 | 0.143552 | 0.00001 | 0 | 0 | 0 | 0 |
| 671 | 0.12044 | 0.045718 | 0.690933 | 0.142909 | 0 | 0 | 0 | 0 | 0 |
| 672 | 0.121061 | 0.046649 | 0.689685 | 0.142605 | 0 | 0 | 0 | 0 | 0 |
| 673 | 0.122581 | 0.047021 | 0.688747 | 0.141651 | 0 | 0 | 0 | 0 | 0 |
| 674 | 0.122876 | 0.047053 | 0.68766 | 0.142411 | 0 | 0 | 0 | 0 | 0 |
| 675 | 0.121776 | 0.04664 | 0.689615 | 0.14197 | 0 | 0 | 0 | 0 | 0 |
| 676 | 0.122974 | 0.047945 | 0.686117 | 0.142964 | 0 | 0 | 0 | 0 | 0 |
| 677 | 0.122999 | 0.045993 | 0.689457 | 0.141551 | 0 | 0 | 0 | 0 | 0 |
| 678 | 0.121884 | 0.045844 | 0.688678 | 0.143593 | 0 | 0 | 0 | 0 | 0 |
| 679 | 0.121255 | 0.047147 | 0.689252 | 0.142347 | 0 | 0 | 0 | 0 | 0 |
| 680 | 0.122527 | 0.047454 | 0.684883 | 0.145136 | 0 | 0 | 0 | 0 | 0 |
| 681 | 0.122194 | 0.045987 | 0.68796 | 0.143849 | 0.00001 | 0 | 0 | 0 | 0 |
| 682 | 0.122609 | 0.047036 | 0.690086 | 0.140269 | 0 | 0 | 0 | 0 | 0 |
| 683 | 0.121584 | 0.047458 | 0.688553 | 0.142405 | 0 | 0 | 0 | 0 | 0 |
| 684 | 0.120598 | 0.046493 | 0.690858 | 0.142052 | 0 | 0 | 0 | 0 | 0 |
| 685 | 0.122619 | 0.047262 | 0.690997 | 0.139122 | 0 | 0 | 0 | 0 | 0 |
| 686 | 0.122844 | 0.04667 | 0.686486 | 0.14398 | 0.00002 | 0 | 0 | 0 | 0 |
| 687 | 0.121808 | 0.047085 | 0.687399 | 0.143708 | 0 | 0 | 0 | 0 | 0 |
| 688 | 0.122112 | 0.047193 | 0.687635 | 0.14306 | 0 | 0 | 0 | 0 | 0 |
| 689 | 0.122986 | 0.047762 | 0.685423 | 0.143829 | 0 | 0 | 0 | 0 | 0 |
| 690 | 0.123309 | 0.04677 | 0.687172 | 0.142749 | 0 | 0 | 0 | 0 | 0 |
| 691 | 0.123347 | 0.047189 | 0.686903 | 0.14256 | 0 | 0 | 0 | 0 | 0 |
| 692 | 0.122802 | 0.046384 | 0.688355 | 0.142459 | 0 | 0 | 0 | 0 | 0 |
| 693 | 0.123089 | 0.046753 | 0.685905 | 0.144253 | 0 | 0 | 0 | 0 | 0 |
| 694 | 0.120424 | 0.047612 | 0.690338 | 0.141626 | 0 | 0 | 0 | 0 | 0 |
| 695 | 0.123082 | 0.046489 | 0.689108 | 0.141321 | 0 | 0 | 0 | 0 | 0 |
| 696 | 0.122833 | 0.047136 | 0.689219 | 0.140812 | 0 | 0 | 0 | 0 | 0 |
| 697 | 0.122886 | 0.046814 | 0.687327 | 0.142973 | 0 | 0 | 0 | 0 | 0 |
| 698 | 0.121198 | 0.047632 | 0.689448 | 0.141711 | 0.00001 | 0 | 0 | 0 | 0 |
| 699 | 0.120622 | 0.046429 | 0.691701 | 0.141248 | 0 | 0 | 0 | 0 | 0 |
| 700 | 0.122527 | 0.047541 | 0.686246 | 0.143686 | 0 | 0 | 0 | 0 | 0 |
| 701 | 0.121446 | 0.047012 | 0.690807 | 0.140735 | 0 | 0 | 0 | 0 | 0 |
| 702 | 0.123673 | 0.046629 | 0.689206 | 0.140492 | 0 | 0 | 0 | 0 | 0 |
| 703 | 0.121444 | 0.047084 | 0.688459 | 0.143013 | 0 | 0 | 0 | 0 | 0 |
| 704 | 0.121624 | 0.047001 | 0.68998 | 0.141395 | 0 | 0 | 0 | 0 | 0 |
| 705 | 0.121713 | 0.045534 | 0.688607 | 0.144146 | 0 | 0 | 0 | 0 | 0 |
| 706 | 0.122107 | 0.048478 | 0.688486 | 0.140929 | 0 | 0 | 0 | 0 | 0 |
| 707 | 0.122036 | 0.046433 | 0.689752 | 0.141779 | 0 | 0 | 0 | 0 | 0 |
| 708 | 0.119945 | 0.047634 | 0.690167 | 0.142253 | 0 | 0 | 0 | 0 | 0 |
| 709 | 0.12197 | 0.046392 | 0.688709 | 0.142929 | 0 | 0 | 0 | 0 | 0 |
| 710 | 0.121172 | 0.047006 | 0.688573 | 0.143249 | 0 | 0 | 0 | 0 | 0 |
| 711 | 0.122804 | 0.047784 | 0.686649 | 0.142763 | 0 | 0 | 0 | 0 | 0 |
| 712 | 0.121582 | 0.046876 | 0.690703 | 0.140839 | 0 | 0 | 0 | 0 | 0 |
| 713 | 0.120957 | 0.046455 | 0.6907 | 0.141888 | 0 | 0 | 0 | 0 | 0 |
| 714 | 0.123996 | 0.047584 | 0.686299 | 0.142121 | 0 | 0 | 0 | 0 | 0 |
| 715 | 0.121589 | 0.04639 | 0.688038 | 0.143983 | 0 | 0 | 0 | 0 | 0 |
| 716 | 0.121131 | 0.047864 | 0.68644 | 0.144565 | 0 | 0 | 0 | 0 | 0 |
| 717 | 0.123039 | 0.046695 | 0.689262 | 0.141004 | 0 | 0 | 0 | 0 | 0 |
| 718 | 0.120659 | 0.047159 | 0.691628 | 0.140554 | 0 | 0 | 0 | 0 | 0 |
| 719 | 0.12181 | 0.046839 | 0.692174 | 0.139177 | 0 | 0 | 0 | 0 | 0 |
| 720 | 0.122872 | 0.047386 | 0.685969 | 0.143763 | 0.00001 | 0 | 0 | 0 | 0 |
| 721 | 0.12028 | 0.046871 | 0.690452 | 0.142396 | 0 | 0 | 0 | 0 | 0 |
| 722 | 0.123892 | 0.047009 | 0.685892 | 0.143207 | 0 | 0 | 0 | 0 | 0 |
| 723 | 0.121523 | 0.046274 | 0.688385 | 0.143819 | 0 | 0 | 0 | 0 | 0 |
| 724 | 0.123153 | 0.047146 | 0.687914 | 0.141787 | 0 | 0 | 0 | 0 | 0 |
| 725 | 0.120643 | 0.046078 | 0.68993 | 0.143349 | 0 | 0 | 0 | 0 | 0 |
| 726 | 0.120918 | 0.046929 | 0.689712 | 0.142442 | 0 | 0 | 0 | 0 | 0 |
| 727 | 0.12153 | 0.047348 | 0.688605 | 0.142516 | 0 | 0 | 0 | 0 | 0 |
| 728 | 0.121874 | 0.04701 | 0.687932 | 0.143184 | 0 | 0 | 0 | 0 | 0 |
| 729 | 0.121796 | 0.047207 | 0.690894 | 0.140103 | 0 | 0 | 0 | 0 | 0 |
| 730 | 0.123897 | 0.046577 | 0.687835 | 0.14169 | 0 | 0 | 0 | 0 | 0 |
| 731 | 0.122497 | 0.046154 | 0.689526 | 0.141822 | 0 | 0 | 0 | 0 | 0 |
| 732 | 0.12241 | 0.047365 | 0.687797 | 0.142427 | 0 | 0 | 0 | 0 | 0 |
| 733 | 0.122214 | 0.046947 | 0.688464 | 0.142375 | 0 | 0 | 0 | 0 | 0 |
| 734 | 0.122236 | 0.047502 | 0.687144 | 0.143118 | 0 | 0 | 0 | 0 | 0 |
| 735 | 0.119609 | 0.047117 | 0.691263 | 0.142001 | 0.00001 | 0 | 0 | 0 | 0 |
| 736 | 0.12299 | 0.045762 | 0.689573 | 0.141676 | 0 | 0 | 0 | 0 | 0 |
| 737 | 0.122911 | 0.04671 | 0.688217 | 0.142161 | 0 | 0 | 0 | 0 | 0 |
| 738 | 0.122499 | 0.046734 | 0.688691 | 0.142076 | 0 | 0 | 0 | 0 | 0 |
| 739 | 0.12064 | 0.047161 | 0.690133 | 0.142066 | 0 | 0 | 0 | 0 | 0 |
| 740 | 0.121848 | 0.046283 | 0.690862 | 0.140997 | 0.00001 | 0 | 0 | 0 | 0 |
| 741 | 0.121445 | 0.04697 | 0.688941 | 0.142644 | 0 | 0 | 0 | 0 | 0 |
| 742 | 0.123049 | 0.047556 | 0.686917 | 0.142478 | 0 | 0 | 0 | 0 | 0 |
| 743 | 0.1203 | 0.045773 | 0.692387 | 0.14154 | 0 | 0 | 0 | 0 | 0 |
| 744 | 0.124257 | 0.04694 | 0.687348 | 0.141445 | 0.00001 | 0 | 0 | 0 | 0 |
| 745 | 0.123062 | 0.046653 | 0.687773 | 0.142501 | 0.00001 | 0 | 0 | 0 | 0 |
| 746 | 0.120614 | 0.046488 | 0.691186 | 0.141712 | 0 | 0 | 0 | 0 | 0 |
| 747 | 0.123817 | 0.047284 | 0.687457 | 0.141432 | 0.00001 | 0 | 0 | 0 | 0 |
| 748 | 0.122768 | 0.045978 | 0.690649 | 0.140605 | 0 | 0 | 0 | 0 | 0 |
| 749 | 0.12064 | 0.046345 | 0.692099 | 0.140916 | 0 | 0 | 0 | 0 | 0 |
| 750 | 0.123025 | 0.046715 | 0.690406 | 0.139854 | 0 | 0 | 0 | 0 | 0 |
| 751 | 0.120784 | 0.046779 | 0.69027 | 0.142167 | 0 | 0 | 0 | 0 | 0 |
| 752 | 0.122604 | 0.047768 | 0.687594 | 0.142035 | 0 | 0 | 0 | 0 | 0 |
| 753 | 0.121467 | 0.046975 | 0.690163 | 0.141395 | 0 | 0 | 0 | 0 | 0 |
| 754 | 0.122166 | 0.046678 | 0.690174 | 0.140983 | 0 | 0 | 0 | 0 | 0 |
| 755 | 0.122144 | 0.046708 | 0.688454 | 0.142694 | 0 | 0 | 0 | 0 | 0 |
| 756 | 0.122636 | 0.047599 | 0.687028 | 0.142737 | 0 | 0 | 0 | 0 | 0 |
| 757 | 0.122648 | 0.046808 | 0.687908 | 0.142636 | 0 | 0 | 0 | 0 | 0 |
| 758 | 0.121972 | 0.047889 | 0.688464 | 0.141675 | 0 | 0 | 0 | 0 | 0 |
| 759 | 0.123615 | 0.046413 | 0.687463 | 0.142509 | 0 | 0 | 0 | 0 | 0 |
| 760 | 0.122255 | 0.046833 | 0.686136 | 0.144775 | 0 | 0 | 0 | 0 | 0 |
| 761 | 0.12139 | 0.04767 | 0.686604 | 0.144336 | 0 | 0 | 0 | 0 | 0 |
| 762 | 0.123032 | 0.047608 | 0.686937 | 0.142424 | 0 | 0 | 0 | 0 | 0 |
| 763 | 0.121666 | 0.048152 | 0.688827 | 0.141355 | 0 | 0 | 0 | 0 | 0 |
| 764 | 0.120536 | 0.047522 | 0.689378 | 0.142565 | 0 | 0 | 0 | 0 | 0 |
| 765 | 0.122855 | 0.046349 | 0.687748 | 0.143049 | 0 | 0 | 0 | 0 | 0 |
| 766 | 0.12233 | 0.047771 | 0.689172 | 0.140727 | 0 | 0 | 0 | 0 | 0 |
| 767 | 0.122144 | 0.046968 | 0.689723 | 0.141165 | 0 | 0 | 0 | 0 | 0 |
| 768 | 0.123361 | 0.04574 | 0.688016 | 0.142883 | 0 | 0 | 0 | 0 | 0 |
| 769 | 0.122079 | 0.045568 | 0.689372 | 0.142981 | 0 | 0 | 0 | 0 | 0 |
| 770 | 0.123863 | 0.046046 | 0.688149 | 0.141942 | 0 | 0 | 0 | 0 | 0 |
| 771 | 0.123778 | 0.047303 | 0.685924 | 0.142985 | 0.00001 | 0 | 0 | 0 | 0 |
| 772 | 0.120959 | 0.048047 | 0.689533 | 0.141451 | 0.00001 | 0 | 0 | 0 | 0 |
| 773 | 0.121415 | 0.047585 | 0.690096 | 0.140904 | 0 | 0 | 0 | 0 | 0 |
| 774 | 0.121682 | 0.046535 | 0.689384 | 0.142398 | 0 | 0 | 0 | 0 | 0 |
| 775 | 0.122686 | 0.047026 | 0.687528 | 0.14276 | 0 | 0 | 0 | 0 | 0 |
| 776 | 0.120812 | 0.047687 | 0.6884 | 0.143101 | 0 | 0 | 0 | 0 | 0 |
| 777 | 0.122686 | 0.046734 | 0.688026 | 0.142554 | 0 | 0 | 0 | 0 | 0 |
| 778 | 0.121256 | 0.047151 | 0.689892 | 0.141692 | 0.00001 | 0 | 0 | 0 | 0 |
| 779 | 0.121151 | 0.04762 | 0.69119 | 0.140039 | 0 | 0 | 0 | 0 | 0 |
| 780 | 0.122252 | 0.046845 | 0.690337 | 0.140566 | 0 | 0 | 0 | 0 | 0 |
| 781 | 0.122258 | 0.047353 | 0.688012 | 0.142377 | 0 | 0 | 0 | 0 | 0 |
| 782 | 0.122235 | 0.046615 | 0.689931 | 0.141218 | 0 | 0 | 0 | 0 | 0 |
| 783 | 0.12155 | 0.047265 | 0.689683 | 0.141503 | 0 | 0 | 0 | 0 | 0 |
| 784 | 0.123188 | 0.046599 | 0.686051 | 0.144161 | 0 | 0 | 0 | 0 | 0 |
| 785 | 0.121167 | 0.045907 | 0.691099 | 0.141828 | 0 | 0 | 0 | 0 | 0 |
| 786 | 0.121905 | 0.046231 | 0.690059 | 0.141805 | 0 | 0 | 0 | 0 | 0 |
| 787 | 0.121573 | 0.046418 | 0.690784 | 0.141225 | 0 | 0 | 0 | 0 | 0 |
| 788 | 0.121071 | 0.046985 | 0.690373 | 0.141571 | 0 | 0 | 0 | 0 | 0 |
| 789 | 0.120858 | 0.047189 | 0.688997 | 0.142956 | 0 | 0 | 0 | 0 | 0 |
| 790 | 0.12024 | 0.047483 | 0.689767 | 0.14251 | 0 | 0 | 0 | 0 | 0 |
| 791 | 0.123279 | 0.047948 | 0.6873 | 0.141473 | 0 | 0 | 0 | 0 | 0 |
| 792 | 0.123845 | 0.047386 | 0.68787 | 0.140899 | 0 | 0 | 0 | 0 | 0 |
| 793 | 0.122706 | 0.046529 | 0.688983 | 0.141782 | 0 | 0 | 0 | 0 | 0 |
| 794 | 0.122677 | 0.046571 | 0.688518 | 0.142233 | 0 | 0 | 0 | 0 | 0 |
| 795 | 0.121459 | 0.046672 | 0.68758 | 0.144289 | 0 | 0 | 0 | 0 | 0 |
| 796 | 0.122879 | 0.046954 | 0.687284 | 0.142883 | 0 | 0 | 0 | 0 | 0 |
| 797 | 0.12262 | 0.046973 | 0.688117 | 0.142291 | 0 | 0 | 0 | 0 | 0 |
| 798 | 0.121599 | 0.045901 | 0.690486 | 0.142004 | 0.00001 | 0 | 0 | 0 | 0 |
| 799 | 0.119754 | 0.046023 | 0.692834 | 0.14139 | 0 | 0 | 0 | 0 | 0 |
| 800 | 0.121482 | 0.046687 | 0.687465 | 0.144366 | 0 | 0 | 0 | 0 | 0 |
| 801 | 0.122396 | 0.046654 | 0.690163 | 0.140787 | 0 | 0 | 0 | 0 | 0 |
| 802 | 0.119981 | 0.047444 | 0.689063 | 0.143513 | 0 | 0 | 0 | 0 | 0 |
| 803 | 0.120996 | 0.046668 | 0.692023 | 0.140313 | 0 | 0 | 0 | 0 | 0 |
| 804 | 0.119831 | 0.047271 | 0.690873 | 0.142024 | 0 | 0 | 0 | 0 | 0 |
| 805 | 0.123124 | 0.046402 | 0.690032 | 0.140442 | 0 | 0 | 0 | 0 | 0 |
| 806 | 0.121228 | 0.047119 | 0.690004 | 0.141639 | 0.00001 | 0 | 0 | 0 | 0 |
| 807 | 0.119873 | 0.048487 | 0.691466 | 0.140173 | 0 | 0 | 0 | 0 | 0 |
| 808 | 0.12274 | 0.046861 | 0.687152 | 0.143248 | 0 | 0 | 0 | 0 | 0 |
| 809 | 0.121718 | 0.047201 | 0.691241 | 0.13984 | 0 | 0 | 0 | 0 | 0 |
| 810 | 0.12115 | 0.046178 | 0.688378 | 0.144294 | 0 | 0 | 0 | 0 | 0 |
| 811 | 0.123028 | 0.048023 | 0.688792 | 0.140157 | 0 | 0 | 0 | 0 | 0 |
| 812 | 0.122054 | 0.047859 | 0.689497 | 0.14059 | 0 | 0 | 0 | 0 | 0 |
| 813 | 0.12236 | 0.047024 | 0.688618 | 0.141998 | 0 | 0 | 0 | 0 | 0 |
| 814 | 0.121463 | 0.047434 | 0.688642 | 0.142461 | 0 | 0 | 0 | 0 | 0 |
| 815 | 0.122788 | 0.046808 | 0.687248 | 0.143156 | 0 | 0 | 0 | 0 | 0 |
| 816 | 0.122778 | 0.046712 | 0.690243 | 0.140267 | 0 | 0 | 0 | 0 | 0 |
| 817 | 0.121803 | 0.046568 | 0.689357 | 0.142272 | 0 | 0 | 0 | 0 | 0 |
| 818 | 0.121227 | 0.046372 | 0.689289 | 0.143112 | 0 | 0 | 0 | 0 | 0 |
| 819 | 0.122213 | 0.046649 | 0.688131 | 0.143007 | 0 | 0 | 0 | 0 | 0 |
| 820 | 0.121657 | 0.045489 | 0.691098 | 0.141756 | 0 | 0 | 0 | 0 | 0 |
| 821 | 0.120517 | 0.046218 | 0.691533 | 0.141733 | 0 | 0 | 0 | 0 | 0 |
| 822 | 0.122162 | 0.047167 | 0.690267 | 0.140405 | 0 | 0 | 0 | 0 | 0 |
| 823 | 0.122776 | 0.047052 | 0.687547 | 0.142614 | 0.00001 | 0 | 0 | 0 | 0 |
| 824 | 0.12333 | 0.047243 | 0.689102 | 0.140325 | 0 | 0 | 0 | 0 | 0 |
| 825 | 0.122566 | 0.046911 | 0.68724 | 0.143283 | 0 | 0 | 0 | 0 | 0 |
| 826 | 0.122803 | 0.046342 | 0.689896 | 0.140959 | 0 | 0 | 0 | 0 | 0 |
| 827 | 0.120702 | 0.046362 | 0.691363 | 0.141573 | 0 | 0 | 0 | 0 | 0 |
| 828 | 0.122484 | 0.046665 | 0.690606 | 0.140244 | 0 | 0 | 0 | 0 | 0 |
| 829 | 0.120771 | 0.04699 | 0.692497 | 0.139741 | 0 | 0 | 0 | 0 | 0 |
| 830 | 0.120134 | 0.046679 | 0.690915 | 0.142272 | 0 | 0 | 0 | 0 | 0 |
| 831 | 0.122606 | 0.047563 | 0.687703 | 0.142127 | 0 | 0 | 0 | 0 | 0 |
| 832 | 0.120552 | 0.046739 | 0.690012 | 0.142697 | 0 | 0 | 0 | 0 | 0 |
| 833 | 0.121087 | 0.047332 | 0.689646 | 0.141935 | 0 | 0 | 0 | 0 | 0 |
| 834 | 0.120874 | 0.047247 | 0.689491 | 0.142379 | 0.00001 | 0 | 0 | 0 | 0 |
| 835 | 0.123517 | 0.047757 | 0.687295 | 0.141432 | 0 | 0 | 0 | 0 | 0 |
| 836 | 0.122391 | 0.048178 | 0.688657 | 0.140774 | 0 | 0 | 0 | 0 | 0 |
| 837 | 0.122301 | 0.045609 | 0.68942 | 0.14267 | 0 | 0 | 0 | 0 | 0 |
| 838 | 0.119792 | 0.046753 | 0.691953 | 0.141502 | 0 | 0 | 0 | 0 | 0 |
| 839 | 0.120815 | 0.047472 | 0.690414 | 0.141299 | 0 | 0 | 0 | 0 | 0 |
| 840 | 0.1212 | 0.046658 | 0.686009 | 0.146134 | 0 | 0 | 0 | 0 | 0 |
| 841 | 0.121938 | 0.047356 | 0.689046 | 0.14166 | 0 | 0 | 0 | 0 | 0 |
| 842 | 0.12514 | 0.045251 | 0.687205 | 0.142404 | 0 | 0 | 0 | 0 | 0 |
| 843 | 0.123049 | 0.046683 | 0.689357 | 0.140911 | 0 | 0 | 0 | 0 | 0 |
| 844 | 0.122287 | 0.046947 | 0.690473 | 0.140293 | 0 | 0 | 0 | 0 | 0 |
| 845 | 0.122045 | 0.046592 | 0.688215 | 0.143148 | 0 | 0 | 0 | 0 | 0 |
| 846 | 0.123819 | 0.045954 | 0.688048 | 0.142169 | 0.00001 | 0 | 0 | 0 | 0 |
| 847 | 0.123988 | 0.046363 | 0.688076 | 0.141573 | 0 | 0 | 0 | 0 | 0 |
| 848 | 0.120562 | 0.046993 | 0.690576 | 0.141869 | 0 | 0 | 0 | 0 | 0 |
| 849 | 0.122063 | 0.046748 | 0.689617 | 0.141562 | 0.00001 | 0 | 0 | 0 | 0 |
| 850 | 0.121262 | 0.046623 | 0.690069 | 0.142046 | 0 | 0 | 0 | 0 | 0 |
| 851 | 0.122358 | 0.0471 | 0.688009 | 0.142523 | 0.00001 | 0 | 0 | 0 | 0 |
| 852 | 0.124266 | 0.045921 | 0.686807 | 0.143006 | 0 | 0 | 0 | 0 | 0 |
| 853 | 0.123336 | 0.046656 | 0.686097 | 0.143911 | 0 | 0 | 0 | 0 | 0 |
| 854 | 0.122078 | 0.046026 | 0.689537 | 0.142358 | 0 | 0 | 0 | 0 | 0 |
| 855 | 0.121272 | 0.048135 | 0.688861 | 0.141732 | 0 | 0 | 0 | 0 | 0 |
| 856 | 0.124669 | 0.047496 | 0.68596 | 0.141875 | 0 | 0 | 0 | 0 | 0 |
| 857 | 0.124724 | 0.047165 | 0.686117 | 0.141994 | 0 | 0 | 0 | 0 | 0 |
| 858 | 0.121386 | 0.046999 | 0.689359 | 0.142256 | 0 | 0 | 0 | 0 | 0 |
| 859 | 0.121315 | 0.047264 | 0.690461 | 0.14096 | 0 | 0 | 0 | 0 | 0 |
| 860 | 0.120336 | 0.047146 | 0.689433 | 0.143085 | 0 | 0 | 0 | 0 | 0 |
| 861 | 0.122209 | 0.04711 | 0.688786 | 0.141896 | 0 | 0 | 0 | 0 | 0 |
| 862 | 0.123689 | 0.047446 | 0.685945 | 0.14292 | 0 | 0 | 0 | 0 | 0 |
| 863 | 0.121632 | 0.045868 | 0.689277 | 0.143224 | 0 | 0 | 0 | 0 | 0 |
| 864 | 0.120524 | 0.046697 | 0.69005 | 0.142729 | 0 | 0 | 0 | 0 | 0 |
| 865 | 0.12299 | 0.046327 | 0.688771 | 0.141912 | 0 | 0 | 0 | 0 | 0 |
| 866 | 0.12148 | 0.04613 | 0.689005 | 0.143385 | 0 | 0 | 0 | 0 | 0 |
| 867 | 0.124077 | 0.04587 | 0.686055 | 0.143998 | 0 | 0 | 0 | 0 | 0 |
| 868 | 0.120625 | 0.047956 | 0.688118 | 0.143301 | 0 | 0 | 0 | 0 | 0 |
| 869 | 0.12243 | 0.046615 | 0.687042 | 0.143914 | 0 | 0 | 0 | 0 | 0 |
| 870 | 0.120964 | 0.046636 | 0.690107 | 0.142293 | 0 | 0 | 0 | 0 | 0 |
| 871 | 0.12184 | 0.04716 | 0.68983 | 0.14117 | 0 | 0 | 0 | 0 | 0 |
| 872 | 0.121028 | 0.046657 | 0.688159 | 0.144156 | 0 | 0 | 0 | 0 | 0 |
| 873 | 0.122235 | 0.046911 | 0.688775 | 0.142079 | 0 | 0 | 0 | 0 | 0 |
| 874 | 0.122846 | 0.04645 | 0.689179 | 0.141526 | 0 | 0 | 0 | 0 | 0 |
| 875 | 0.122814 | 0.046148 | 0.68853 | 0.142508 | 0 | 0 | 0 | 0 | 0 |
| 876 | 0.121015 | 0.046433 | 0.68866 | 0.143891 | 0 | 0 | 0 | 0 | 0 |
| 877 | 0.122414 | 0.04616 | 0.689994 | 0.141432 | 0 | 0 | 0 | 0 | 0 |
| 878 | 0.12132 | 0.046514 | 0.689159 | 0.143006 | 0 | 0 | 0 | 0 | 0 |
| 879 | 0.122086 | 0.047325 | 0.687122 | 0.143467 | 0 | 0 | 0 | 0 | 0 |
| 880 | 0.122169 | 0.045957 | 0.693016 | 0.138859 | 0 | 0 | 0 | 0 | 0 |
| 881 | 0.120918 | 0.04718 | 0.687924 | 0.143978 | 0 | 0 | 0 | 0 | 0 |
| 882 | 0.123991 | 0.047096 | 0.685782 | 0.143122 | 0.00001 | 0 | 0 | 0 | 0 |
| 883 | 0.119699 | 0.046678 | 0.689402 | 0.144212 | 0.00001 | 0 | 0 | 0 | 0 |
| 884 | 0.12119 | 0.047781 | 0.688916 | 0.142114 | 0 | 0 | 0 | 0 | 0 |
| 885 | 0.12165 | 0.046374 | 0.69042 | 0.141556 | 0 | 0 | 0 | 0 | 0 |
| 886 | 0.123313 | 0.046948 | 0.687884 | 0.141855 | 0 | 0 | 0 | 0 | 0 |
| 887 | 0.122214 | 0.045505 | 0.691018 | 0.141263 | 0 | 0 | 0 | 0 | 0 |
| 888 | 0.12252 | 0.046597 | 0.687023 | 0.14386 | 0 | 0 | 0 | 0 | 0 |
| 889 | 0.123107 | 0.046096 | 0.687266 | 0.14353 | 0 | 0 | 0 | 0 | 0 |
| 890 | 0.123388 | 0.047083 | 0.688671 | 0.140858 | 0 | 0 | 0 | 0 | 0 |
| 891 | 0.124528 | 0.046315 | 0.685751 | 0.143407 | 0 | 0 | 0 | 0 | 0 |
| 892 | 0.122421 | 0.047115 | 0.68895 | 0.141515 | 0 | 0 | 0 | 0 | 0 |
| 893 | 0.123332 | 0.047766 | 0.686707 | 0.142195 | 0 | 0 | 0 | 0 | 0 |
| 894 | 0.122696 | 0.047165 | 0.690011 | 0.140127 | 0 | 0 | 0 | 0 | 0 |
| 895 | 0.123498 | 0.04649 | 0.689669 | 0.140343 | 0 | 0 | 0 | 0 | 0 |
| 896 | 0.124145 | 0.046689 | 0.688174 | 0.140992 | 0 | 0 | 0 | 0 | 0 |
| 897 | 0.121224 | 0.046624 | 0.688954 | 0.143198 | 0 | 0 | 0 | 0 | 0 |
| 898 | 0.120848 | 0.048048 | 0.688702 | 0.142402 | 0 | 0 | 0 | 0 | 0 |
| 899 | 0.121033 | 0.048207 | 0.689939 | 0.140822 | 0 | 0 | 0 | 0 | 0 |
| 900 | 0.123055 | 0.047071 | 0.688088 | 0.141785 | 0 | 0 | 0 | 0 | 0 |
| 901 | 0.122308 | 0.047522 | 0.687273 | 0.142897 | 0 | 0 | 0 | 0 | 0 |
| 902 | 0.122129 | 0.046187 | 0.689439 | 0.142234 | 0.00001 | 0 | 0 | 0 | 0 |
| 903 | 0.122087 | 0.047174 | 0.689535 | 0.141204 | 0 | 0 | 0 | 0 | 0 |
| 904 | 0.121931 | 0.046954 | 0.690154 | 0.140962 | 0 | 0 | 0 | 0 | 0 |
| 905 | 0.121274 | 0.046692 | 0.691059 | 0.140975 | 0 | 0 | 0 | 0 | 0 |
| 906 | 0.120883 | 0.047599 | 0.691251 | 0.140268 | 0 | 0 | 0 | 0 | 0 |
| 907 | 0.123792 | 0.046607 | 0.688019 | 0.141572 | 0.00001 | 0 | 0 | 0 | 0 |
| 908 | 0.121973 | 0.047048 | 0.689141 | 0.141837 | 0 | 0 | 0 | 0 | 0 |
| 909 | 0.124199 | 0.047479 | 0.685462 | 0.14286 | 0 | 0 | 0 | 0 | 0 |
| 910 | 0.121647 | 0.047894 | 0.686689 | 0.14377 | 0 | 0 | 0 | 0 | 0 |
| 911 | 0.12119 | 0.047558 | 0.690909 | 0.140343 | 0 | 0 | 0 | 0 | 0 |
| 912 | 0.121974 | 0.046719 | 0.688665 | 0.142641 | 0 | 0 | 0 | 0 | 0 |
| 913 | 0.121662 | 0.046597 | 0.691286 | 0.140456 | 0 | 0 | 0 | 0 | 0 |
| 914 | 0.122399 | 0.047066 | 0.690341 | 0.140194 | 0 | 0 | 0 | 0 | 0 |
| 915 | 0.122922 | 0.047419 | 0.687006 | 0.142654 | 0 | 0 | 0 | 0 | 0 |
| 916 | 0.123387 | 0.046745 | 0.687007 | 0.142861 | 0 | 0 | 0 | 0 | 0 |
| 917 | 0.122618 | 0.046769 | 0.68625 | 0.144363 | 0 | 0 | 0 | 0 | 0 |
| 918 | 0.122358 | 0.046449 | 0.689686 | 0.141507 | 0 | 0 | 0 | 0 | 0 |
| 919 | 0.122141 | 0.04752 | 0.688805 | 0.141534 | 0 | 0 | 0 | 0 | 0 |
| 920 | 0.120324 | 0.046394 | 0.689681 | 0.143601 | 0 | 0 | 0 | 0 | 0 |
| 921 | 0.123325 | 0.047401 | 0.686748 | 0.142525 | 0 | 0 | 0 | 0 | 0 |
| 922 | 0.121954 | 0.048229 | 0.687986 | 0.141832 | 0 | 0 | 0 | 0 | 0 |
| 923 | 0.122332 | 0.046824 | 0.690152 | 0.140692 | 0 | 0 | 0 | 0 | 0 |
| 924 | 0.12132 | 0.045966 | 0.688882 | 0.143832 | 0 | 0 | 0 | 0 | 0 |
| 925 | 0.121574 | 0.046545 | 0.690549 | 0.141321 | 0.00001 | 0 | 0 | 0 | 0 |
| 926 | 0.120368 | 0.048121 | 0.690021 | 0.14149 | 0 | 0 | 0 | 0 | 0 |
| 927 | 0.120908 | 0.046644 | 0.690899 | 0.141549 | 0 | 0 | 0 | 0 | 0 |
| 928 | 0.122016 | 0.046922 | 0.688959 | 0.142102 | 0 | 0 | 0 | 0 | 0 |
| 929 | 0.122129 | 0.047194 | 0.688401 | 0.142275 | 0 | 0 | 0 | 0 | 0 |
| 930 | 0.122363 | 0.047364 | 0.688898 | 0.141374 | 0 | 0 | 0 | 0 | 0 |
| 931 | 0.120855 | 0.047109 | 0.690369 | 0.141667 | 0 | 0 | 0 | 0 | 0 |
| 932 | 0.123528 | 0.047358 | 0.68675 | 0.142364 | 0 | 0 | 0 | 0 | 0 |
| 933 | 0.122502 | 0.047233 | 0.690115 | 0.14014 | 0.00001 | 0 | 0 | 0 | 0 |
| 934 | 0.122687 | 0.047463 | 0.68811 | 0.14174 | 0 | 0 | 0 | 0 | 0 |
| 935 | 0.122179 | 0.048156 | 0.6875 | 0.142165 | 0 | 0 | 0 | 0 | 0 |
| 936 | 0.123623 | 0.045924 | 0.688764 | 0.141689 | 0 | 0 | 0 | 0 | 0 |
| 937 | 0.122006 | 0.045862 | 0.690373 | 0.141758 | 0 | 0 | 0 | 0 | 0 |
| 938 | 0.121242 | 0.046768 | 0.689234 | 0.142746 | 0.00001 | 0 | 0 | 0 | 0 |
| 939 | 0.123626 | 0.046721 | 0.689221 | 0.140433 | 0 | 0 | 0 | 0 | 0 |
| 940 | 0.121452 | 0.046458 | 0.688251 | 0.143839 | 0 | 0 | 0 | 0 | 0 |
| 941 | 0.120493 | 0.0472 | 0.689258 | 0.143049 | 0 | 0 | 0 | 0 | 0 |
| 942 | 0.120981 | 0.047994 | 0.688278 | 0.142747 | 0 | 0 | 0 | 0 | 0 |
| 943 | 0.122515 | 0.047201 | 0.689667 | 0.140617 | 0 | 0 | 0 | 0 | 0 |
| 944 | 0.120552 | 0.048404 | 0.688481 | 0.142564 | 0 | 0 | 0 | 0 | 0 |
| 945 | 0.12237 | 0.04812 | 0.686089 | 0.143411 | 0.00001 | 0 | 0 | 0 | 0 |
| 946 | 0.120981 | 0.047121 | 0.690324 | 0.141574 | 0 | 0 | 0 | 0 | 0 |
| 947 | 0.120578 | 0.047528 | 0.69192 | 0.139974 | 0 | 0 | 0 | 0 | 0 |
| 948 | 0.121287 | 0.04785 | 0.687701 | 0.143162 | 0 | 0 | 0 | 0 | 0 |
| 949 | 0.123623 | 0.046469 | 0.688427 | 0.14148 | 0 | 0 | 0 | 0 | 0 |
| 950 | 0.12232 | 0.048399 | 0.688435 | 0.140845 | 0 | 0 | 0 | 0 | 0 |
| 951 | 0.121312 | 0.047823 | 0.691057 | 0.139809 | 0 | 0 | 0 | 0 | 0 |
| 952 | 0.120599 | 0.046946 | 0.691338 | 0.141118 | 0 | 0 | 0 | 0 | 0 |
| 953 | 0.122827 | 0.047129 | 0.687161 | 0.142873 | 0.00001 | 0 | 0 | 0 | 0 |
| 954 | 0.123431 | 0.045983 | 0.686647 | 0.143938 | 0 | 0 | 0 | 0 | 0 |
| 955 | 0.12267 | 0.046821 | 0.688617 | 0.141892 | 0 | 0 | 0 | 0 | 0 |
| 956 | 0.122167 | 0.046714 | 0.688849 | 0.14227 | 0 | 0 | 0 | 0 | 0 |
| 957 | 0.122606 | 0.047075 | 0.688552 | 0.141757 | 0.00001 | 0 | 0 | 0 | 0 |
| 958 | 0.121707 | 0.047596 | 0.689005 | 0.141692 | 0 | 0 | 0 | 0 | 0 |
| 959 | 0.122082 | 0.046997 | 0.685906 | 0.145015 | 0 | 0 | 0 | 0 | 0 |
| 960 | 0.122867 | 0.047358 | 0.687861 | 0.141914 | 0 | 0 | 0 | 0 | 0 |
| 961 | 0.120076 | 0.045869 | 0.691548 | 0.142497 | 0.00001 | 0 | 0 | 0 | 0 |
| 962 | 0.120839 | 0.047391 | 0.689817 | 0.141953 | 0 | 0 | 0 | 0 | 0 |
| 963 | 0.122752 | 0.046346 | 0.690315 | 0.140588 | 0 | 0 | 0 | 0 | 0 |
| 964 | 0.122788 | 0.046177 | 0.68984 | 0.141195 | 0 | 0 | 0 | 0 | 0 |
| 965 | 0.122099 | 0.045709 | 0.689839 | 0.142352 | 0 | 0 | 0 | 0 | 0 |
| 966 | 0.122364 | 0.045959 | 0.68946 | 0.142217 | 0 | 0 | 0 | 0 | 0 |
| 967 | 0.122327 | 0.046384 | 0.68911 | 0.142179 | 0 | 0 | 0 | 0 | 0 |
| 968 | 0.12247 | 0.046993 | 0.687977 | 0.14256 | 0 | 0 | 0 | 0 | 0 |
| 969 | 0.121109 | 0.047555 | 0.687316 | 0.144019 | 0 | 0 | 0 | 0 | 0 |
| 970 | 0.121928 | 0.04693 | 0.690981 | 0.140161 | 0 | 0 | 0 | 0 | 0 |
| 971 | 0.122521 | 0.046233 | 0.686927 | 0.144319 | 0 | 0 | 0 | 0 | 0 |
| 972 | 0.121751 | 0.047008 | 0.689425 | 0.141816 | 0 | 0 | 0 | 0 | 0 |
| 973 | 0.122431 | 0.047751 | 0.685802 | 0.144006 | 0.00001 | 0 | 0 | 0 | 0 |
| 974 | 0.123822 | 0.046422 | 0.687727 | 0.142028 | 0 | 0 | 0 | 0 | 0 |
| 975 | 0.122917 | 0.047257 | 0.688579 | 0.141247 | 0 | 0 | 0 | 0 | 0 |
| 976 | 0.120725 | 0.046771 | 0.688926 | 0.143578 | 0 | 0 | 0 | 0 | 0 |
| 977 | 0.120833 | 0.046455 | 0.690625 | 0.142087 | 0 | 0 | 0 | 0 | 0 |
| 978 | 0.122541 | 0.046331 | 0.689235 | 0.141894 | 0 | 0 | 0 | 0 | 0 |
| 979 | 0.123349 | 0.046524 | 0.68711 | 0.143017 | 0 | 0 | 0 | 0 | 0 |
| 980 | 0.12388 | 0.047215 | 0.685439 | 0.143456 | 0.00001 | 0 | 0 | 0 | 0 |
| 981 | 0.122437 | 0.046461 | 0.688861 | 0.142231 | 0.00001 | 0 | 0 | 0 | 0 |
| 982 | 0.121842 | 0.046192 | 0.690726 | 0.141241 | 0 | 0 | 0 | 0 | 0 |
| 983 | 0.121944 | 0.047291 | 0.688943 | 0.141822 | 0 | 0 | 0 | 0 | 0 |
| 984 | 0.121651 | 0.046025 | 0.68894 | 0.143384 | 0 | 0 | 0 | 0 | 0 |
| 985 | 0.121372 | 0.045892 | 0.690679 | 0.142057 | 0 | 0 | 0 | 0 | 0 |
| 986 | 0.121765 | 0.047442 | 0.689387 | 0.141406 | 0 | 0 | 0 | 0 | 0 |
| 987 | 0.122465 | 0.046159 | 0.689289 | 0.142088 | 0 | 0 | 0 | 0 | 0 |
| 988 | 0.123709 | 0.047729 | 0.685763 | 0.142799 | 0 | 0 | 0 | 0 | 0 |
| 989 | 0.123745 | 0.047373 | 0.686904 | 0.141968 | 0.00001 | 0 | 0 | 0 | 0 |
| 990 | 0.122615 | 0.047 | 0.689357 | 0.141019 | 0.00001 | 0 | 0 | 0 | 0 |
| 991 | 0.123886 | 0.047253 | 0.68769 | 0.141171 | 0 | 0 | 0 | 0 | 0 |
| 992 | 0.123887 | 0.047899 | 0.687411 | 0.140804 | 0 | 0 | 0 | 0 | 0 |
| 993 | 0.121711 | 0.047535 | 0.690116 | 0.140627 | 0.00001 | 0 | 0 | 0 | 0 |
| 994 | 0.122726 | 0.046498 | 0.689351 | 0.141425 | 0 | 0 | 0 | 0 | 0 |
| 995 | 0.122726 | 0.046495 | 0.689151 | 0.141629 | 0 | 0 | 0 | 0 | 0 |
| 996 | 0.12363 | 0.047181 | 0.687197 | 0.141993 | 0 | 0 | 0 | 0 | 0 |
| 997 | 0.1221 | 0.047023 | 0.68899 | 0.141887 | 0 | 0 | 0 | 0 | 0 |
| 998 | 0.121249 | 0.046935 | 0.688403 | 0.143413 | 0 | 0 | 0 | 0 | 0 |
| 999 | 0.123047 | 0.047668 | 0.688804 | 0.140471 | 0.00001 | 0 | 0 | 0 | 0 |
| 1000 | 0.122187 | 0.04738 | 0.687324 | 0.143108 | 0 | 0 | 0 | 0 | 0 |

| Year 2 | Race |  |  |  |  |  |  |  |  |
| --- | --- | --- | --- | --- | --- | --- | --- | --- | --- |
| Trial | 003.0 | 005.0 | 007.0 | 037.1 | 043.0 | 303.0 | 003.2 | 403.0 | 003.4 |
| 1 | 0.019782 | 0.020699 | 0.807723 | 0.151795 | 0 | 0 | 0 | 0 | 0 |
| 2 | 0.020268 | 0.021031 | 0.805384 | 0.153317 | 0 | 0 | 0 | 0 | 0 |
| 3 | 0.02076 | 0.020437 | 0.809193 | 0.149611 | 0 | 0 | 0 | 0 | 0 |
| 4 | 0.019846 | 0.021057 | 0.808321 | 0.150765 | 0 | 0 | 0 | 0 | 0 |
| 5 | 0.01989 | 0.020758 | 0.805804 | 0.153548 | 0 | 0 | 0 | 0 | 0 |
| 6 | 0.020307 | 0.020936 | 0.804814 | 0.153943 | 0 | 0 | 0 | 0 | 0 |
| 7 | 0.020178 | 0.021399 | 0.805082 | 0.153341 | 0 | 0 | 0 | 0 | 0 |
| 8 | 0.019393 | 0.02044 | 0.808172 | 0.151994 | 0 | 0 | 0 | 0 | 0 |
| 9 | 0.019541 | 0.020711 | 0.807848 | 0.151899 | 0 | 0 | 0 | 0 | 0 |
| 10 | 0.019668 | 0.020807 | 0.807111 | 0.152415 | 0 | 0 | 0 | 0 | 0 |
| 11 | 0.020278 | 0.020949 | 0.807612 | 0.151151 | 0 | 0 | 0 | 0 | 0 |
| 12 | 0.020016 | 0.020455 | 0.806487 | 0.153041 | 0 | 0 | 0 | 0 | 0 |
| 13 | 0.01949 | 0.02123 | 0.805906 | 0.153373 | 0 | 0 | 0 | 0 | 0 |
| 14 | 0.019848 | 0.021048 | 0.801788 | 0.157306 | 0 | 0 | 0 | 0 | 0 |
| 15 | 0.020124 | 0.020224 | 0.807615 | 0.152037 | 0 | 0 | 0 | 0 | 0 |
| 16 | 0.019934 | 0.021495 | 0.803048 | 0.155522 | 0 | 0 | 0 | 0 | 0 |
| 17 | 0.020014 | 0.020432 | 0.80729 | 0.152254 | 0 | 0 | 0 | 0 | 0 |
| 18 | 0.01981 | 0.020922 | 0.808102 | 0.151166 | 0 | 0 | 0 | 0 | 0 |
| 19 | 0.020023 | 0.021264 | 0.804757 | 0.153956 | 0 | 0 | 0 | 0 | 0 |
| 20 | 0.019767 | 0.020204 | 0.804937 | 0.155092 | 0 | 0 | 0 | 0 | 0 |
| 21 | 0.020131 | 0.020779 | 0.808443 | 0.150647 | 0 | 0 | 0 | 0 | 0 |
| 22 | 0.019264 | 0.02127 | 0.805615 | 0.153851 | 0 | 0 | 0 | 0 | 0 |
| 23 | 0.01939 | 0.020812 | 0.806949 | 0.152849 | 0 | 0 | 0 | 0 | 0 |
| 24 | 0.020091 | 0.021536 | 0.807318 | 0.151054 | 0 | 0 | 0 | 0 | 0 |
| 25 | 0.019904 | 0.020034 | 0.807792 | 0.15227 | 0 | 0 | 0 | 0 | 0 |
| 26 | 0.020391 | 0.02054 | 0.810537 | 0.148532 | 0 | 0 | 0 | 0 | 0 |
| 27 | 0.019198 | 0.020429 | 0.807389 | 0.152984 | 0 | 0 | 0 | 0 | 0 |
| 28 | 0.020047 | 0.021026 | 0.80621 | 0.152698 | 0 | 0 | 0 | 0 | 0 |
| 29 | 0.020303 | 0.021357 | 0.805243 | 0.153096 | 0 | 0 | 0 | 0 | 0 |
| 30 | 0.019916 | 0.021063 | 0.806353 | 0.152668 | 0 | 0 | 0 | 0 | 0 |
| 31 | 0.019149 | 0.020541 | 0.8075 | 0.152809 | 0 | 0 | 0 | 0 | 0 |
| 32 | 0.019928 | 0.021871 | 0.805219 | 0.152981 | 0 | 0 | 0 | 0 | 0 |
| 33 | 0.020424 | 0.020912 | 0.805622 | 0.153043 | 0 | 0 | 0 | 0 | 0 |
| 34 | 0.019914 | 0.020732 | 0.806983 | 0.152371 | 0 | 0 | 0 | 0 | 0 |
| 35 | 0.020273 | 0.019755 | 0.805067 | 0.154904 | 0 | 0 | 0 | 0 | 0 |
| 36 | 0.01955 | 0.020657 | 0.805873 | 0.15392 | 0 | 0 | 0 | 0 | 0 |
| 37 | 0.019753 | 0.021719 | 0.807232 | 0.151297 | 0 | 0 | 0 | 0 | 0 |
| 38 | 0.019719 | 0.020329 | 0.808998 | 0.150954 | 0 | 0 | 0 | 0 | 0 |
| 39 | 0.02031 | 0.020349 | 0.807144 | 0.152188 | 0 | 0 | 0 | 0 | 0 |
| 40 | 0.019371 | 0.020352 | 0.804441 | 0.155836 | 0 | 0 | 0 | 0 | 0 |
| 41 | 0.020067 | 0.021559 | 0.804991 | 0.153372 | 0 | 0 | 0 | 0 | 0 |
| 42 | 0.019132 | 0.021117 | 0.809881 | 0.149871 | 0 | 0 | 0 | 0 | 0 |
| 43 | 0.019866 | 0.021107 | 0.808251 | 0.150777 | 0 | 0 | 0 | 0 | 0 |
| 44 | 0.019248 | 0.021712 | 0.805711 | 0.153329 | 0 | 0 | 0 | 0 | 0 |
| 45 | 0.019934 | 0.021748 | 0.807617 | 0.150701 | 0 | 0 | 0 | 0 | 0 |
| 46 | 0.019468 | 0.021141 | 0.803975 | 0.155417 | 0 | 0 | 0 | 0 | 0 |
| 47 | 0.02023 | 0.020721 | 0.805674 | 0.153375 | 0 | 0 | 0 | 0 | 0 |
| 48 | 0.020204 | 0.020476 | 0.804663 | 0.154658 | 0 | 0 | 0 | 0 | 0 |
| 49 | 0.019918 | 0.020538 | 0.805847 | 0.153697 | 0 | 0 | 0 | 0 | 0 |
| 50 | 0.019305 | 0.020862 | 0.807742 | 0.152091 | 0 | 0 | 0 | 0 | 0 |
| 51 | 0.019769 | 0.02036 | 0.807391 | 0.152481 | 0 | 0 | 0 | 0 | 0 |
| 52 | 0.019848 | 0.021329 | 0.805289 | 0.153534 | 0 | 0 | 0 | 0 | 0 |
| 53 | 0.020668 | 0.020698 | 0.809633 | 0.148991 | 0 | 0 | 0 | 0 | 0 |
| 54 | 0.019131 | 0.020259 | 0.810452 | 0.150159 | 0 | 0 | 0 | 0 | 0 |
| 55 | 0.020443 | 0.021223 | 0.806332 | 0.152001 | 0 | 0 | 0 | 0 | 0 |
| 56 | 0.019477 | 0.020509 | 0.806898 | 0.153116 | 0 | 0 | 0 | 0 | 0 |
| 57 | 0.019888 | 0.021891 | 0.808138 | 0.150083 | 0 | 0 | 0 | 0 | 0 |
| 58 | 0.019266 | 0.021557 | 0.806299 | 0.152878 | 0 | 0 | 0 | 0 | 0 |
| 59 | 0.020156 | 0.021324 | 0.808005 | 0.150515 | 0 | 0 | 0 | 0 | 0 |
| 60 | 0.020583 | 0.020414 | 0.807674 | 0.151329 | 0 | 0 | 0 | 0 | 0 |
| 61 | 0.019249 | 0.021 | 0.808227 | 0.151525 | 0 | 0 | 0 | 0 | 0 |
| 62 | 0.020152 | 0.021042 | 0.808806 | 0.15 | 0 | 0 | 0 | 0 | 0 |
| 63 | 0.019589 | 0.020823 | 0.810555 | 0.149033 | 0 | 0 | 0 | 0 | 0 |
| 64 | 0.020107 | 0.021726 | 0.805893 | 0.152274 | 0 | 0 | 0 | 0 | 0 |
| 65 | 0.020505 | 0.020614 | 0.80484 | 0.154041 | 0 | 0 | 0 | 0 | 0 |
| 66 | 0.019627 | 0.020331 | 0.810249 | 0.149794 | 0 | 0 | 0 | 0 | 0 |
| 67 | 0.019859 | 0.020879 | 0.805788 | 0.153464 | 0 | 0 | 0 | 0 | 0 |
| 68 | 0.02 | 0.02057 | 0.80804 | 0.15138 | 0 | 0 | 0 | 0 | 0 |
| 69 | 0.020975 | 0.020485 | 0.803809 | 0.154731 | 0 | 0 | 0 | 0 | 0 |
| 70 | 0.020258 | 0.020387 | 0.806153 | 0.153203 | 0 | 0 | 0 | 0 | 0 |
| 71 | 0.019284 | 0.021204 | 0.805428 | 0.154074 | 0 | 0 | 0 | 0 | 0 |
| 72 | 0.019831 | 0.021246 | 0.80948 | 0.149444 | 0 | 0 | 0 | 0 | 0 |
| 73 | 0.020523 | 0.020613 | 0.805602 | 0.153263 | 0 | 0 | 0 | 0 | 0 |
| 74 | 0.018948 | 0.021341 | 0.806392 | 0.153319 | 0 | 0 | 0 | 0 | 0 |
| 75 | 0.020873 | 0.021711 | 0.804881 | 0.152535 | 0 | 0 | 0 | 0 | 0 |
| 76 | 0.019749 | 0.021822 | 0.807437 | 0.150991 | 0 | 0 | 0 | 0 | 0 |
| 77 | 0.01994 | 0.021552 | 0.805342 | 0.153167 | 0 | 0 | 0 | 0 | 0 |
| 78 | 0.019705 | 0.02119 | 0.81019 | 0.148916 | 0 | 0 | 0 | 0 | 0 |
| 79 | 0.020057 | 0.020257 | 0.806832 | 0.152855 | 0 | 0 | 0 | 0 | 0 |
| 80 | 0.019485 | 0.020418 | 0.806643 | 0.153444 | 0 | 0 | 0 | 0 | 0 |
| 81 | 0.019636 | 0.021258 | 0.803537 | 0.15557 | 0 | 0 | 0 | 0 | 0 |
| 82 | 0.019955 | 0.021527 | 0.807147 | 0.151371 | 0 | 0 | 0 | 0 | 0 |
| 83 | 0.020346 | 0.021297 | 0.809031 | 0.149326 | 0 | 0 | 0 | 0 | 0 |
| 84 | 0.019723 | 0.02114 | 0.809386 | 0.14975 | 0 | 0 | 0 | 0 | 0 |
| 85 | 0.019833 | 0.020858 | 0.803915 | 0.155393 | 0 | 0 | 0 | 0 | 0 |
| 86 | 0.019889 | 0.021358 | 0.808609 | 0.150144 | 0 | 0 | 0 | 0 | 0 |
| 87 | 0.019978 | 0.02071 | 0.805822 | 0.15349 | 0 | 0 | 0 | 0 | 0 |
| 88 | 0.018981 | 0.02138 | 0.806703 | 0.152927 | 0 | 0 | 0 | 0 | 0 |
| 89 | 0.020812 | 0.021624 | 0.803318 | 0.154246 | 0 | 0 | 0 | 0 | 0 |
| 90 | 0.019664 | 0.021087 | 0.806861 | 0.152358 | 0 | 0 | 0 | 0 | 0 |
| 91 | 0.019216 | 0.021045 | 0.807474 | 0.152265 | 0 | 0 | 0 | 0 | 0 |
| 92 | 0.019067 | 0.021497 | 0.804361 | 0.155075 | 0 | 0 | 0 | 0 | 0 |
| 93 | 0.019517 | 0.020697 | 0.808255 | 0.151532 | 0 | 0 | 0 | 0 | 0 |
| 94 | 0.020376 | 0.020934 | 0.807463 | 0.151227 | 0 | 0 | 0 | 0 | 0 |
| 95 | 0.01942 | 0.021277 | 0.805098 | 0.154204 | 0 | 0 | 0 | 0 | 0 |
| 96 | 0.019996 | 0.020526 | 0.808682 | 0.150796 | 0 | 0 | 0 | 0 | 0 |
| 97 | 0.01921 | 0.021287 | 0.80655 | 0.152954 | 0 | 0 | 0 | 0 | 0 |
| 98 | 0.02017 | 0.020663 | 0.805821 | 0.153345 | 0 | 0 | 0 | 0 | 0 |
| 99 | 0.019467 | 0.020321 | 0.807202 | 0.15301 | 0 | 0 | 0 | 0 | 0 |
| 100 | 0.020129 | 0.020069 | 0.807691 | 0.152111 | 0 | 0 | 0 | 0 | 0 |
| 101 | 0.01991 | 0.021491 | 0.807851 | 0.150748 | 0 | 0 | 0 | 0 | 0 |
| 102 | 0.019802 | 0.020653 | 0.806247 | 0.153299 | 0 | 0 | 0 | 0 | 0 |
| 103 | 0.020677 | 0.022293 | 0.802116 | 0.154915 | 0 | 0 | 0 | 0 | 0 |
| 104 | 0.020992 | 0.020612 | 0.809166 | 0.14919 | 0 | 0 | 0 | 0 | 0 |
| 105 | 0.019876 | 0.020833 | 0.80622 | 0.15307 | 0 | 0 | 0 | 0 | 0 |
| 106 | 0.019613 | 0.020882 | 0.805279 | 0.154226 | 0 | 0 | 0 | 0 | 0 |
| 107 | 0.019625 | 0.019725 | 0.808725 | 0.151905 | 0 | 0 | 0 | 0 | 0 |
| 108 | 0.019394 | 0.021306 | 0.805152 | 0.154128 | 0 | 0 | 0 | 0 | 0 |
| 109 | 0.019684 | 0.021078 | 0.807395 | 0.151843 | 0 | 0 | 0 | 0 | 0 |
| 110 | 0.020217 | 0.020046 | 0.810968 | 0.148769 | 0 | 0 | 0 | 0 | 0 |
| 111 | 0.019479 | 0.021437 | 0.807614 | 0.151471 | 0 | 0 | 0 | 0 | 0 |
| 112 | 0.019773 | 0.020557 | 0.804946 | 0.154725 | 0 | 0 | 0 | 0 | 0 |
| 113 | 0.019682 | 0.020973 | 0.808299 | 0.151046 | 0 | 0 | 0 | 0 | 0 |
| 114 | 0.020036 | 0.020416 | 0.807673 | 0.151875 | 0 | 0 | 0 | 0 | 0 |
| 115 | 0.019755 | 0.020015 | 0.808746 | 0.151485 | 0 | 0 | 0 | 0 | 0 |
| 116 | 0.020046 | 0.020725 | 0.808164 | 0.151066 | 0 | 0 | 0 | 0 | 0 |
| 117 | 0.018973 | 0.020749 | 0.808115 | 0.152164 | 0 | 0 | 0 | 0 | 0 |
| 118 | 0.019838 | 0.019928 | 0.81025 | 0.149984 | 0 | 0 | 0 | 0 | 0 |
| 119 | 0.019971 | 0.021009 | 0.805343 | 0.153677 | 0 | 0 | 0 | 0 | 0 |
| 120 | 0.020654 | 0.020694 | 0.80939 | 0.149263 | 0 | 0 | 0 | 0 | 0 |
| 121 | 0.018062 | 0.021817 | 0.80748 | 0.152631 | 0 | 0 | 0 | 0 | 0 |
| 122 | 0.019375 | 0.019966 | 0.808247 | 0.152412 | 0 | 0 | 0 | 0 | 0 |
| 123 | 0.020136 | 0.020447 | 0.808013 | 0.151404 | 0 | 0 | 0 | 0 | 0 |
| 124 | 0.020651 | 0.02109 | 0.807265 | 0.150994 | 0 | 0 | 0 | 0 | 0 |
| 125 | 0.019622 | 0.020862 | 0.809668 | 0.149848 | 0 | 0 | 0 | 0 | 0 |
| 126 | 0.019674 | 0.021024 | 0.806929 | 0.152372 | 0 | 0 | 0 | 0 | 0 |
| 127 | 0.019885 | 0.020363 | 0.810638 | 0.149115 | 0 | 0 | 0 | 0 | 0 |
| 128 | 0.019867 | 0.020358 | 0.808464 | 0.15131 | 0 | 0 | 0 | 0 | 0 |
| 129 | 0.019932 | 0.02023 | 0.807037 | 0.152802 | 0 | 0 | 0 | 0 | 0 |
| 130 | 0.020144 | 0.020893 | 0.806991 | 0.151972 | 0 | 0 | 0 | 0 | 0 |
| 131 | 0.019522 | 0.020278 | 0.810034 | 0.150166 | 0 | 0 | 0 | 0 | 0 |
| 132 | 0.020341 | 0.020634 | 0.804319 | 0.154706 | 0 | 0 | 0 | 0 | 0 |
| 133 | 0.020095 | 0.020475 | 0.804063 | 0.155367 | 0 | 0 | 0 | 0 | 0 |
| 134 | 0.020249 | 0.019682 | 0.807057 | 0.153011 | 0 | 0 | 0 | 0 | 0 |
| 135 | 0.02003 | 0.020399 | 0.804877 | 0.154695 | 0 | 0 | 0 | 0 | 0 |
| 136 | 0.019223 | 0.021489 | 0.809767 | 0.149521 | 0 | 0 | 0 | 0 | 0 |
| 137 | 0.020017 | 0.020676 | 0.807019 | 0.152288 | 0 | 0 | 0 | 0 | 0 |
| 138 | 0.019434 | 0.021081 | 0.804693 | 0.154792 | 0 | 0 | 0 | 0 | 0 |
| 139 | 0.020523 | 0.020383 | 0.807817 | 0.151277 | 0 | 0 | 0 | 0 | 0 |
| 140 | 0.020117 | 0.02126 | 0.806883 | 0.15174 | 0 | 0 | 0 | 0 | 0 |
| 141 | 0.020324 | 0.020952 | 0.804598 | 0.154126 | 0 | 0 | 0 | 0 | 0 |
| 142 | 0.020384 | 0.021627 | 0.804825 | 0.153164 | 0 | 0 | 0 | 0 | 0 |
| 143 | 0.020121 | 0.021265 | 0.805266 | 0.153348 | 0 | 0 | 0 | 0 | 0 |
| 144 | 0.020591 | 0.021139 | 0.805455 | 0.152815 | 0 | 0 | 0 | 0 | 0 |
| 145 | 0.020697 | 0.020667 | 0.808032 | 0.150603 | 0 | 0 | 0 | 0 | 0 |
| 146 | 0.019663 | 0.021436 | 0.811307 | 0.147595 | 0 | 0 | 0 | 0 | 0 |
| 147 | 0.020512 | 0.020872 | 0.805931 | 0.152685 | 0 | 0 | 0 | 0 | 0 |
| 148 | 0.02056 | 0.021062 | 0.803195 | 0.155183 | 0 | 0 | 0 | 0 | 0 |
| 149 | 0.02039 | 0.02045 | 0.806022 | 0.153139 | 0 | 0 | 0 | 0 | 0 |
| 150 | 0.020575 | 0.019735 | 0.806393 | 0.153277 | 0 | 0 | 0 | 0 | 0 |
| 151 | 0.019768 | 0.02038 | 0.805913 | 0.15394 | 0 | 0 | 0 | 0 | 0 |
| 152 | 0.019605 | 0.021563 | 0.804978 | 0.153855 | 0 | 0 | 0 | 0 | 0 |
| 153 | 0.020134 | 0.020545 | 0.808638 | 0.150682 | 0 | 0 | 0 | 0 | 0 |
| 154 | 0.019202 | 0.020151 | 0.807535 | 0.153113 | 0 | 0 | 0 | 0 | 0 |
| 155 | 0.019818 | 0.020558 | 0.804836 | 0.154788 | 0 | 0 | 0 | 0 | 0 |
| 156 | 0.019525 | 0.020806 | 0.807027 | 0.152642 | 0 | 0 | 0 | 0 | 0 |
| 157 | 0.020336 | 0.020657 | 0.807329 | 0.151677 | 0 | 0 | 0 | 0 | 0 |
| 158 | 0.020648 | 0.020398 | 0.807387 | 0.151557 | 0 | 0 | 0 | 0 | 0 |
| 159 | 0.019461 | 0.020354 | 0.807188 | 0.152997 | 0 | 0 | 0 | 0 | 0 |
| 160 | 0.020146 | 0.019956 | 0.812136 | 0.147763 | 0 | 0 | 0 | 0 | 0 |
| 161 | 0.020389 | 0.02015 | 0.804403 | 0.155058 | 0 | 0 | 0 | 0 | 0 |
| 162 | 0.020092 | 0.020564 | 0.805953 | 0.153391 | 0 | 0 | 0 | 0 | 0 |
| 163 | 0.020967 | 0.020538 | 0.803748 | 0.154747 | 0 | 0 | 0 | 0 | 0 |
| 164 | 0.019732 | 0.021603 | 0.805317 | 0.153348 | 0 | 0 | 0 | 0 | 0 |
| 165 | 0.018767 | 0.021381 | 0.80794 | 0.151912 | 0 | 0 | 0 | 0 | 0 |
| 166 | 0.020327 | 0.021064 | 0.808561 | 0.150048 | 0 | 0 | 0 | 0 | 0 |
| 167 | 0.01974 | 0.02161 | 0.806406 | 0.152244 | 0 | 0 | 0 | 0 | 0 |
| 168 | 0.019448 | 0.020595 | 0.808156 | 0.151802 | 0 | 0 | 0 | 0 | 0 |
| 169 | 0.020316 | 0.020845 | 0.804852 | 0.153987 | 0 | 0 | 0 | 0 | 0 |
| 170 | 0.019149 | 0.021984 | 0.805255 | 0.153611 | 0 | 0 | 0 | 0 | 0 |
| 171 | 0.019796 | 0.020837 | 0.808181 | 0.151187 | 0 | 0 | 0 | 0 | 0 |
| 172 | 0.020271 | 0.02091 | 0.805571 | 0.153247 | 0 | 0 | 0 | 0 | 0 |
| 173 | 0.019548 | 0.022289 | 0.804518 | 0.153645 | 0 | 0 | 0 | 0 | 0 |
| 174 | 0.020464 | 0.021916 | 0.803171 | 0.154449 | 0 | 0 | 0 | 0 | 0 |
| 175 | 0.019813 | 0.020424 | 0.807013 | 0.15275 | 0 | 0 | 0 | 0 | 0 |
| 176 | 0.02044 | 0.020261 | 0.811612 | 0.147657 | 0 | 0 | 0 | 0 | 0 |
| 177 | 0.020912 | 0.020315 | 0.806607 | 0.152165 | 0 | 0 | 0 | 0 | 0 |
| 178 | 0.019035 | 0.020859 | 0.808151 | 0.151956 | 0 | 0 | 0 | 0 | 0 |
| 179 | 0.020301 | 0.021353 | 0.806147 | 0.152199 | 0 | 0 | 0 | 0 | 0 |
| 180 | 0.019873 | 0.021206 | 0.806773 | 0.152149 | 0 | 0 | 0 | 0 | 0 |
| 181 | 0.019735 | 0.020815 | 0.809138 | 0.150312 | 0 | 0 | 0 | 0 | 0 |
| 182 | 0.019842 | 0.021376 | 0.807273 | 0.151509 | 0 | 0 | 0 | 0 | 0 |
| 183 | 0.02052 | 0.021511 | 0.806225 | 0.151744 | 0 | 0 | 0 | 0 | 0 |
| 184 | 0.019886 | 0.020333 | 0.80839 | 0.151391 | 0 | 0 | 0 | 0 | 0 |
| 185 | 0.019525 | 0.02075 | 0.805203 | 0.154522 | 0 | 0 | 0 | 0 | 0 |
| 186 | 0.020764 | 0.019619 | 0.808891 | 0.150726 | 0 | 0 | 0 | 0 | 0 |
| 187 | 0.019719 | 0.02026 | 0.812992 | 0.147029 | 0 | 0 | 0 | 0 | 0 |
| 188 | 0.020052 | 0.020072 | 0.808823 | 0.151052 | 0 | 0 | 0 | 0 | 0 |
| 189 | 0.019519 | 0.021359 | 0.80468 | 0.154442 | 0 | 0 | 0 | 0 | 0 |
| 190 | 0.020114 | 0.021372 | 0.805065 | 0.153448 | 0 | 0 | 0 | 0 | 0 |
| 191 | 0.020052 | 0.020915 | 0.806402 | 0.152632 | 0 | 0 | 0 | 0 | 0 |
| 192 | 0.020469 | 0.020459 | 0.807806 | 0.151267 | 0 | 0 | 0 | 0 | 0 |
| 193 | 0.019544 | 0.021567 | 0.804836 | 0.154053 | 0 | 0 | 0 | 0 | 0 |
| 194 | 0.01938 | 0.020788 | 0.80621 | 0.153622 | 0 | 0 | 0 | 0 | 0 |
| 195 | 0.020382 | 0.020852 | 0.806753 | 0.152012 | 0 | 0 | 0 | 0 | 0 |
| 196 | 0.019551 | 0.020539 | 0.807309 | 0.152602 | 0 | 0 | 0 | 0 | 0 |
| 197 | 0.019397 | 0.019538 | 0.81113 | 0.149935 | 0 | 0 | 0 | 0 | 0 |
| 198 | 0.020521 | 0.020511 | 0.807411 | 0.151547 | 0 | 0 | 0 | 0 | 0 |
| 199 | 0.019836 | 0.019806 | 0.81031 | 0.150049 | 0 | 0 | 0 | 0 | 0 |
| 200 | 0.019691 | 0.021945 | 0.8047 | 0.153664 | 0 | 0 | 0 | 0 | 0 |
| 201 | 0.021065 | 0.021235 | 0.805461 | 0.152239 | 0 | 0 | 0 | 0 | 0 |
| 202 | 0.021 | 0.021459 | 0.804759 | 0.152782 | 0 | 0 | 0 | 0 | 0 |
| 203 | 0.019139 | 0.020572 | 0.809171 | 0.151118 | 0 | 0 | 0 | 0 | 0 |
| 204 | 0.02041 | 0.021426 | 0.807175 | 0.150989 | 0 | 0 | 0 | 0 | 0 |
| 205 | 0.019763 | 0.020374 | 0.803514 | 0.156349 | 0 | 0 | 0 | 0 | 0 |
| 206 | 0.019872 | 0.02067 | 0.8068 | 0.152657 | 0 | 0 | 0 | 0 | 0 |
| 207 | 0.019866 | 0.02063 | 0.80745 | 0.152055 | 0 | 0 | 0 | 0 | 0 |
| 208 | 0.020211 | 0.019384 | 0.810858 | 0.149547 | 0 | 0 | 0 | 0 | 0 |
| 209 | 0.019393 | 0.02184 | 0.805558 | 0.153209 | 0 | 0 | 0 | 0 | 0 |
| 210 | 0.019803 | 0.020966 | 0.807103 | 0.152128 | 0 | 0 | 0 | 0 | 0 |
| 211 | 0.020293 | 0.021624 | 0.806119 | 0.151964 | 0 | 0 | 0 | 0 | 0 |
| 212 | 0.02003 | 0.02001 | 0.807565 | 0.152395 | 0 | 0 | 0 | 0 | 0 |
| 213 | 0.019841 | 0.021107 | 0.809645 | 0.149407 | 0 | 0 | 0 | 0 | 0 |
| 214 | 0.019161 | 0.021203 | 0.808415 | 0.15122 | 0 | 0 | 0 | 0 | 0 |
| 215 | 0.020316 | 0.021529 | 0.807274 | 0.150871 | 0 | 0 | 0 | 0 | 0 |
| 216 | 0.019961 | 0.020051 | 0.808343 | 0.151645 | 0 | 0 | 0 | 0 | 0 |
| 217 | 0.020145 | 0.020575 | 0.806967 | 0.152313 | 0 | 0 | 0 | 0 | 0 |
| 218 | 0.019455 | 0.02066 | 0.807654 | 0.152231 | 0 | 0 | 0 | 0 | 0 |
| 219 | 0.019568 | 0.021316 | 0.811069 | 0.148048 | 0 | 0 | 0 | 0 | 0 |
| 220 | 0.02043 | 0.02037 | 0.808156 | 0.151044 | 0 | 0 | 0 | 0 | 0 |
| 221 | 0.019618 | 0.020467 | 0.806537 | 0.153378 | 0 | 0 | 0 | 0 | 0 |
| 222 | 0.019412 | 0.02148 | 0.809375 | 0.149732 | 0 | 0 | 0 | 0 | 0 |
| 223 | 0.020786 | 0.020826 | 0.803473 | 0.154915 | 0 | 0 | 0 | 0 | 0 |
| 224 | 0.02068 | 0.02058 | 0.804336 | 0.154404 | 0 | 0 | 0 | 0 | 0 |
| 225 | 0.020311 | 0.021414 | 0.809361 | 0.148914 | 0 | 0 | 0 | 0 | 0 |
| 226 | 0.020371 | 0.020923 | 0.809959 | 0.148748 | 0 | 0 | 0 | 0 | 0 |
| 227 | 0.020125 | 0.020924 | 0.804928 | 0.154022 | 0 | 0 | 0 | 0 | 0 |
| 228 | 0.019767 | 0.020316 | 0.808226 | 0.151691 | 0 | 0 | 0 | 0 | 0 |
| 229 | 0.019353 | 0.020533 | 0.807977 | 0.152136 | 0 | 0 | 0 | 0 | 0 |
| 230 | 0.018516 | 0.020697 | 0.808835 | 0.151952 | 0 | 0 | 0 | 0 | 0 |
| 231 | 0.019851 | 0.020824 | 0.809731 | 0.149595 | 0 | 0 | 0 | 0 | 0 |
| 232 | 0.019695 | 0.021434 | 0.806402 | 0.152469 | 0 | 0 | 0 | 0 | 0 |
| 233 | 0.020007 | 0.020751 | 0.810515 | 0.148707 | 0 | 0 | 0 | 0 | 0 |
| 234 | 0.019195 | 0.0215 | 0.80957 | 0.149736 | 0 | 0 | 0 | 0 | 0 |
| 235 | 0.020405 | 0.020586 | 0.808936 | 0.150063 | 0 | 0 | 0 | 0 | 0 |
| 236 | 0.020465 | 0.020286 | 0.80433 | 0.154919 | 0 | 0 | 0 | 0 | 0 |
| 237 | 0.020398 | 0.020859 | 0.807482 | 0.15126 | 0 | 0 | 0 | 0 | 0 |
| 238 | 0.020132 | 0.021635 | 0.804159 | 0.154074 | 0 | 0 | 0 | 0 | 0 |
| 239 | 0.019913 | 0.020938 | 0.804285 | 0.154863 | 0 | 0 | 0 | 0 | 0 |
| 240 | 0.020287 | 0.020017 | 0.808329 | 0.151367 | 0 | 0 | 0 | 0 | 0 |
| 241 | 0.019881 | 0.020931 | 0.807568 | 0.151619 | 0 | 0 | 0 | 0 | 0 |
| 242 | 0.019583 | 0.020336 | 0.808046 | 0.152025 | 0 | 0 | 0 | 0 | 0 |
| 243 | 0.018984 | 0.020048 | 0.810352 | 0.150616 | 0 | 0 | 0 | 0 | 0 |
| 244 | 0.019839 | 0.020029 | 0.808345 | 0.151787 | 0 | 0 | 0 | 0 | 0 |
| 245 | 0.020261 | 0.020371 | 0.807043 | 0.152315 | 0 | 0 | 0 | 0 | 0 |
| 246 | 0.020087 | 0.021247 | 0.806545 | 0.15212 | 0 | 0 | 0 | 0 | 0 |
| 247 | 0.020527 | 0.020786 | 0.804377 | 0.15431 | 0 | 0 | 0 | 0 | 0 |
| 248 | 0.020081 | 0.019971 | 0.810959 | 0.148989 | 0 | 0 | 0 | 0 | 0 |
| 249 | 0.019404 | 0.020335 | 0.808866 | 0.151395 | 0 | 0 | 0 | 0 | 0 |
| 250 | 0.020392 | 0.021359 | 0.803191 | 0.155048 | 0 | 0 | 0 | 0 | 0 |
| 251 | 0.019271 | 0.021163 | 0.807646 | 0.151919 | 0 | 0 | 0 | 0 | 0 |
| 252 | 0.019801 | 0.021194 | 0.807291 | 0.151714 | 0 | 0 | 0 | 0 | 0 |
| 253 | 0.020451 | 0.021599 | 0.804683 | 0.153267 | 0 | 0 | 0 | 0 | 0 |
| 254 | 0.019982 | 0.021394 | 0.805589 | 0.153035 | 0 | 0 | 0 | 0 | 0 |
| 255 | 0.02001 | 0.021434 | 0.804189 | 0.154367 | 0 | 0 | 0 | 0 | 0 |
| 256 | 0.019968 | 0.020729 | 0.806016 | 0.153286 | 0 | 0 | 0 | 0 | 0 |
| 257 | 0.019848 | 0.020401 | 0.805291 | 0.154449 | 0 | 0 | 0 | 0 | 0 |
| 258 | 0.020378 | 0.021308 | 0.803635 | 0.154679 | 0 | 0 | 0 | 0 | 0 |
| 259 | 0.020032 | 0.020541 | 0.806621 | 0.152796 | 0 | 0 | 0 | 0 | 0 |
| 260 | 0.020012 | 0.021809 | 0.806932 | 0.151237 | 0 | 0 | 0 | 0 | 0 |
| 261 | 0.019335 | 0.020072 | 0.80817 | 0.152423 | 0 | 0 | 0 | 0 | 0 |
| 262 | 0.019969 | 0.020611 | 0.806819 | 0.152601 | 0 | 0 | 0 | 0 | 0 |
| 263 | 0.019837 | 0.02135 | 0.807161 | 0.151652 | 0 | 0 | 0 | 0 | 0 |
| 264 | 0.019064 | 0.020851 | 0.805267 | 0.154818 | 0 | 0 | 0 | 0 | 0 |
| 265 | 0.019992 | 0.021339 | 0.805852 | 0.152816 | 0 | 0 | 0 | 0 | 0 |
| 266 | 0.020548 | 0.020848 | 0.806641 | 0.151963 | 0 | 0 | 0 | 0 | 0 |
| 267 | 0.019854 | 0.019924 | 0.808633 | 0.151589 | 0 | 0 | 0 | 0 | 0 |
| 268 | 0.019898 | 0.02154 | 0.808142 | 0.15042 | 0 | 0 | 0 | 0 | 0 |
| 269 | 0.020291 | 0.020829 | 0.807229 | 0.151651 | 0 | 0 | 0 | 0 | 0 |
| 270 | 0.020398 | 0.020398 | 0.808868 | 0.150335 | 0 | 0 | 0 | 0 | 0 |
| 271 | 0.01991 | 0.020822 | 0.809114 | 0.150143 | 0 | 0 | 0 | 0 | 0 |
| 272 | 0.01882 | 0.020742 | 0.809642 | 0.150796 | 0 | 0 | 0 | 0 | 0 |
| 273 | 0.019828 | 0.021163 | 0.808148 | 0.15086 | 0 | 0 | 0 | 0 | 0 |
| 274 | 0.020601 | 0.020992 | 0.80975 | 0.148657 | 0 | 0 | 0 | 0 | 0 |
| 275 | 0.018998 | 0.021169 | 0.807398 | 0.152435 | 0 | 0 | 0 | 0 | 0 |
| 276 | 0.020166 | 0.020126 | 0.809412 | 0.150296 | 0 | 0 | 0 | 0 | 0 |
| 277 | 0.019433 | 0.022275 | 0.802994 | 0.155297 | 0 | 0 | 0 | 0 | 0 |
| 278 | 0.020765 | 0.021044 | 0.806868 | 0.151323 | 0 | 0 | 0 | 0 | 0 |
| 279 | 0.019588 | 0.021029 | 0.803577 | 0.155806 | 0 | 0 | 0 | 0 | 0 |
| 280 | 0.019714 | 0.021363 | 0.80775 | 0.151173 | 0 | 0 | 0 | 0 | 0 |
| 281 | 0.019537 | 0.021135 | 0.805529 | 0.153799 | 0 | 0 | 0 | 0 | 0 |
| 282 | 0.020097 | 0.020437 | 0.80463 | 0.154836 | 0 | 0 | 0 | 0 | 0 |
| 283 | 0.019706 | 0.021168 | 0.808986 | 0.15014 | 0 | 0 | 0 | 0 | 0 |
| 284 | 0.019801 | 0.020839 | 0.809357 | 0.150002 | 0 | 0 | 0 | 0 | 0 |
| 285 | 0.020494 | 0.020773 | 0.805569 | 0.153164 | 0 | 0 | 0 | 0 | 0 |
| 286 | 0.019593 | 0.021282 | 0.807099 | 0.152025 | 0 | 0 | 0 | 0 | 0 |
| 287 | 0.020034 | 0.021951 | 0.806181 | 0.151833 | 0 | 0 | 0 | 0 | 0 |
| 288 | 0.019688 | 0.021223 | 0.80688 | 0.152209 | 0 | 0 | 0 | 0 | 0 |
| 289 | 0.019745 | 0.020625 | 0.807403 | 0.152227 | 0 | 0 | 0 | 0 | 0 |
| 290 | 0.01969 | 0.020652 | 0.806135 | 0.153523 | 0 | 0 | 0 | 0 | 0 |
| 291 | 0.020551 | 0.020631 | 0.806856 | 0.151962 | 0 | 0 | 0 | 0 | 0 |
| 292 | 0.020068 | 0.019999 | 0.806793 | 0.15314 | 0 | 0 | 0 | 0 | 0 |
| 293 | 0.020543 | 0.021261 | 0.801282 | 0.156914 | 0 | 0 | 0 | 0 | 0 |
| 294 | 0.018997 | 0.020883 | 0.806017 | 0.154103 | 0 | 0 | 0 | 0 | 0 |
| 295 | 0.019785 | 0.019825 | 0.806001 | 0.154389 | 0 | 0 | 0 | 0 | 0 |
| 296 | 0.019229 | 0.021395 | 0.806587 | 0.152789 | 0 | 0 | 0 | 0 | 0 |
| 297 | 0.019543 | 0.020944 | 0.807131 | 0.152382 | 0 | 0 | 0 | 0 | 0 |
| 298 | 0.018846 | 0.022107 | 0.807082 | 0.151965 | 0 | 0 | 0 | 0 | 0 |
| 299 | 0.020553 | 0.020294 | 0.803818 | 0.155325 | 0 | 0 | 0 | 0 | 0 |
| 300 | 0.019427 | 0.020496 | 0.806231 | 0.153847 | 0 | 0 | 0 | 0 | 0 |
| 301 | 0.020422 | 0.02127 | 0.803398 | 0.154909 | 0 | 0 | 0 | 0 | 0 |
| 302 | 0.020586 | 0.020195 | 0.805947 | 0.153272 | 0 | 0 | 0 | 0 | 0 |
| 303 | 0.019847 | 0.020422 | 0.80874 | 0.15099 | 0 | 0 | 0 | 0 | 0 |
| 304 | 0.019271 | 0.021372 | 0.803308 | 0.156049 | 0 | 0 | 0 | 0 | 0 |
| 305 | 0.019773 | 0.019623 | 0.810506 | 0.150097 | 0 | 0 | 0 | 0 | 0 |
| 306 | 0.020151 | 0.02055 | 0.80908 | 0.150199 | 0 | 0 | 0 | 0 | 0 |
| 307 | 0.020299 | 0.021065 | 0.804707 | 0.153928 | 0 | 0 | 0 | 0 | 0 |
| 308 | 0.019802 | 0.020462 | 0.810634 | 0.149101 | 0 | 0 | 0 | 0 | 0 |
| 309 | 0.019378 | 0.020176 | 0.809662 | 0.150784 | 0 | 0 | 0 | 0 | 0 |
| 310 | 0.020404 | 0.020174 | 0.808789 | 0.150633 | 0 | 0 | 0 | 0 | 0 |
| 311 | 0.020538 | 0.021208 | 0.806139 | 0.152115 | 0 | 0 | 0 | 0 | 0 |
| 312 | 0.019812 | 0.020807 | 0.806279 | 0.153102 | 0 | 0 | 0 | 0 | 0 |
| 313 | 0.02059 | 0.019623 | 0.808282 | 0.151505 | 0 | 0 | 0 | 0 | 0 |
| 314 | 0.019607 | 0.020791 | 0.811294 | 0.148307 | 0 | 0 | 0 | 0 | 0 |
| 315 | 0.019997 | 0.02111 | 0.802962 | 0.155931 | 0 | 0 | 0 | 0 | 0 |
| 316 | 0.020672 | 0.021142 | 0.807196 | 0.15099 | 0 | 0 | 0 | 0 | 0 |
| 317 | 0.020024 | 0.020985 | 0.80833 | 0.150661 | 0 | 0 | 0 | 0 | 0 |
| 318 | 0.019744 | 0.020391 | 0.803615 | 0.156231 | 0 | 0 | 0 | 0 | 0 |
| 319 | 0.019682 | 0.021032 | 0.802882 | 0.156404 | 0 | 0 | 0 | 0 | 0 |
| 320 | 0.019367 | 0.020281 | 0.809203 | 0.151149 | 0 | 0 | 0 | 0 | 0 |
| 321 | 0.0203 | 0.019969 | 0.805714 | 0.154017 | 0 | 0 | 0 | 0 | 0 |
| 322 | 0.019684 | 0.020183 | 0.807763 | 0.15237 | 0 | 0 | 0 | 0 | 0 |
| 323 | 0.019454 | 0.020932 | 0.808441 | 0.151172 | 0 | 0 | 0 | 0 | 0 |
| 324 | 0.019644 | 0.020377 | 0.808159 | 0.15182 | 0 | 0 | 0 | 0 | 0 |
| 325 | 0.019901 | 0.02047 | 0.80958 | 0.150049 | 0 | 0 | 0 | 0 | 0 |
| 326 | 0.019262 | 0.0207 | 0.805415 | 0.154623 | 0 | 0 | 0 | 0 | 0 |
| 327 | 0.019763 | 0.020463 | 0.808363 | 0.151411 | 0 | 0 | 0 | 0 | 0 |
| 328 | 0.02012 | 0.021478 | 0.806915 | 0.151487 | 0 | 0 | 0 | 0 | 0 |
| 329 | 0.020751 | 0.020811 | 0.804665 | 0.153772 | 0 | 0 | 0 | 0 | 0 |
| 330 | 0.019937 | 0.020218 | 0.807269 | 0.152576 | 0 | 0 | 0 | 0 | 0 |
| 331 | 0.020269 | 0.020469 | 0.809471 | 0.149791 | 0 | 0 | 0 | 0 | 0 |
| 332 | 0.020671 | 0.02033 | 0.808468 | 0.150531 | 0 | 0 | 0 | 0 | 0 |
| 333 | 0.019851 | 0.02068 | 0.807863 | 0.151606 | 0 | 0 | 0 | 0 | 0 |
| 334 | 0.019897 | 0.020977 | 0.807815 | 0.151311 | 0 | 0 | 0 | 0 | 0 |
| 335 | 0.020057 | 0.021055 | 0.80621 | 0.152678 | 0 | 0 | 0 | 0 | 0 |
| 336 | 0.01925 | 0.021634 | 0.805933 | 0.153183 | 0 | 0 | 0 | 0 | 0 |
| 337 | 0.019941 | 0.020543 | 0.808315 | 0.1512 | 0 | 0 | 0 | 0 | 0 |
| 338 | 0.02016 | 0.020439 | 0.810112 | 0.149289 | 0 | 0 | 0 | 0 | 0 |
| 339 | 0.020048 | 0.020912 | 0.80573 | 0.153309 | 0 | 0 | 0 | 0 | 0 |
| 340 | 0.019703 | 0.021325 | 0.806908 | 0.152065 | 0 | 0 | 0 | 0 | 0 |
| 341 | 0.020049 | 0.020568 | 0.806554 | 0.152828 | 0 | 0 | 0 | 0 | 0 |
| 342 | 0.020666 | 0.020216 | 0.804051 | 0.155067 | 0 | 0 | 0 | 0 | 0 |
| 343 | 0.019072 | 0.020785 | 0.809546 | 0.150596 | 0 | 0 | 0 | 0 | 0 |
| 344 | 0.020094 | 0.019873 | 0.806672 | 0.153361 | 0 | 0 | 0 | 0 | 0 |
| 345 | 0.021299 | 0.020335 | 0.80426 | 0.154105 | 0 | 0 | 0 | 0 | 0 |
| 346 | 0.020464 | 0.020713 | 0.80803 | 0.150793 | 0 | 0 | 0 | 0 | 0 |
| 347 | 0.019996 | 0.020934 | 0.808086 | 0.150964 | 0 | 0 | 0 | 0 | 0 |
| 348 | 0.020183 | 0.020883 | 0.80637 | 0.152565 | 0 | 0 | 0 | 0 | 0 |
| 349 | 0.01933 | 0.0195 | 0.808018 | 0.153152 | 0 | 0 | 0 | 0 | 0 |
| 350 | 0.020222 | 0.021023 | 0.807967 | 0.150788 | 0 | 0 | 0 | 0 | 0 |
| 351 | 0.019664 | 0.020707 | 0.807579 | 0.15205 | 0 | 0 | 0 | 0 | 0 |
| 352 | 0.020139 | 0.020669 | 0.808502 | 0.15069 | 0 | 0 | 0 | 0 | 0 |
| 353 | 0.020572 | 0.020542 | 0.806573 | 0.152313 | 0 | 0 | 0 | 0 | 0 |
| 354 | 0.020523 | 0.021105 | 0.80379 | 0.154582 | 0 | 0 | 0 | 0 | 0 |
| 355 | 0.019826 | 0.020854 | 0.807894 | 0.151426 | 0 | 0 | 0 | 0 | 0 |
| 356 | 0.020181 | 0.020591 | 0.807394 | 0.151835 | 0 | 0 | 0 | 0 | 0 |
| 357 | 0.019977 | 0.020925 | 0.80467 | 0.154428 | 0 | 0 | 0 | 0 | 0 |
| 358 | 0.020121 | 0.021682 | 0.807293 | 0.150904 | 0 | 0 | 0 | 0 | 0 |
| 359 | 0.019566 | 0.021085 | 0.805726 | 0.153622 | 0 | 0 | 0 | 0 | 0 |
| 360 | 0.020018 | 0.020823 | 0.809806 | 0.149352 | 0 | 0 | 0 | 0 | 0 |
| 361 | 0.019271 | 0.02087 | 0.807083 | 0.152777 | 0 | 0 | 0 | 0 | 0 |
| 362 | 0.020842 | 0.020751 | 0.808118 | 0.150289 | 0 | 0 | 0 | 0 | 0 |
| 363 | 0.019966 | 0.019906 | 0.809954 | 0.150174 | 0 | 0 | 0 | 0 | 0 |
| 364 | 0.019089 | 0.021733 | 0.807368 | 0.151811 | 0 | 0 | 0 | 0 | 0 |
| 365 | 0.020743 | 0.021445 | 0.806375 | 0.151437 | 0 | 0 | 0 | 0 | 0 |
| 366 | 0.020497 | 0.02115 | 0.80541 | 0.152943 | 0 | 0 | 0 | 0 | 0 |
| 367 | 0.019729 | 0.021518 | 0.803948 | 0.154806 | 0 | 0 | 0 | 0 | 0 |
| 368 | 0.018769 | 0.020342 | 0.81153 | 0.149359 | 0 | 0 | 0 | 0 | 0 |
| 369 | 0.020349 | 0.020199 | 0.808205 | 0.151246 | 0 | 0 | 0 | 0 | 0 |
| 370 | 0.019177 | 0.02156 | 0.80681 | 0.152453 | 0 | 0 | 0 | 0 | 0 |
| 371 | 0.019904 | 0.020637 | 0.803517 | 0.155893 | 0 | 0 | 0 | 0 | 0 |
| 372 | 0.019973 | 0.021153 | 0.805034 | 0.153839 | 0 | 0 | 0 | 0 | 0 |
| 373 | 0.019593 | 0.020232 | 0.806753 | 0.153421 | 0 | 0 | 0 | 0 | 0 |
| 374 | 0.020721 | 0.020382 | 0.809342 | 0.149556 | 0 | 0 | 0 | 0 | 0 |
| 375 | 0.019845 | 0.020849 | 0.805352 | 0.153954 | 0 | 0 | 0 | 0 | 0 |
| 376 | 0.019994 | 0.021956 | 0.805142 | 0.152908 | 0 | 0 | 0 | 0 | 0 |
| 377 | 0.01995 | 0.021356 | 0.805726 | 0.152968 | 0 | 0 | 0 | 0 | 0 |
| 378 | 0.019704 | 0.020728 | 0.811786 | 0.147782 | 0 | 0 | 0 | 0 | 0 |
| 379 | 0.020665 | 0.021363 | 0.804664 | 0.153308 | 0 | 0 | 0 | 0 | 0 |
| 380 | 0.020606 | 0.021027 | 0.805543 | 0.152824 | 0 | 0 | 0 | 0 | 0 |
| 381 | 0.019709 | 0.021018 | 0.804246 | 0.155027 | 0 | 0 | 0 | 0 | 0 |
| 382 | 0.020385 | 0.019993 | 0.807151 | 0.152471 | 0 | 0 | 0 | 0 | 0 |
| 383 | 0.019267 | 0.02079 | 0.808246 | 0.151697 | 0 | 0 | 0 | 0 | 0 |
| 384 | 0.020009 | 0.021185 | 0.806244 | 0.152562 | 0 | 0 | 0 | 0 | 0 |
| 385 | 0.019605 | 0.020786 | 0.807994 | 0.151615 | 0 | 0 | 0 | 0 | 0 |
| 386 | 0.02004 | 0.020829 | 0.808004 | 0.151127 | 0 | 0 | 0 | 0 | 0 |
| 387 | 0.019141 | 0.021282 | 0.80639 | 0.153187 | 0 | 0 | 0 | 0 | 0 |
| 388 | 0.020761 | 0.020145 | 0.806913 | 0.152181 | 0 | 0 | 0 | 0 | 0 |
| 389 | 0.019489 | 0.021043 | 0.810543 | 0.148925 | 0 | 0 | 0 | 0 | 0 |
| 390 | 0.019558 | 0.021055 | 0.807234 | 0.152153 | 0 | 0 | 0 | 0 | 0 |
| 391 | 0.020106 | 0.021989 | 0.802396 | 0.155508 | 0 | 0 | 0 | 0 | 0 |
| 392 | 0.020025 | 0.02164 | 0.808219 | 0.150115 | 0 | 0 | 0 | 0 | 0 |
| 393 | 0.020824 | 0.021873 | 0.803901 | 0.153402 | 0 | 0 | 0 | 0 | 0 |
| 394 | 0.02005 | 0.020731 | 0.807038 | 0.152181 | 0 | 0 | 0 | 0 | 0 |
| 395 | 0.019842 | 0.020062 | 0.806216 | 0.15388 | 0 | 0 | 0 | 0 | 0 |
| 396 | 0.019618 | 0.020105 | 0.808701 | 0.151576 | 0 | 0 | 0 | 0 | 0 |
| 397 | 0.020851 | 0.021163 | 0.806704 | 0.151271 | 0 | 0 | 0 | 0 | 0 |
| 398 | 0.018964 | 0.020479 | 0.80804 | 0.152517 | 0 | 0 | 0 | 0 | 0 |
| 399 | 0.019971 | 0.021061 | 0.807222 | 0.151746 | 0 | 0 | 0 | 0 | 0 |
| 400 | 0.020482 | 0.020692 | 0.809839 | 0.148987 | 0 | 0 | 0 | 0 | 0 |
| 401 | 0.019925 | 0.020528 | 0.806452 | 0.153095 | 0 | 0 | 0 | 0 | 0 |
| 402 | 0.018882 | 0.019644 | 0.80837 | 0.153103 | 0 | 0 | 0 | 0 | 0 |
| 403 | 0.020122 | 0.022104 | 0.80736 | 0.150414 | 0 | 0 | 0 | 0 | 0 |
| 404 | 0.020132 | 0.02054 | 0.808377 | 0.150952 | 0 | 0 | 0 | 0 | 0 |
| 405 | 0.020601 | 0.020382 | 0.808567 | 0.15045 | 0 | 0 | 0 | 0 | 0 |
| 406 | 0.019778 | 0.021361 | 0.806716 | 0.152146 | 0 | 0 | 0 | 0 | 0 |
| 407 | 0.019008 | 0.021507 | 0.807802 | 0.151683 | 0 | 0 | 0 | 0 | 0 |
| 408 | 0.019602 | 0.019983 | 0.807199 | 0.153217 | 0 | 0 | 0 | 0 | 0 |
| 409 | 0.019562 | 0.021201 | 0.807849 | 0.151387 | 0 | 0 | 0 | 0 | 0 |
| 410 | 0.01948 | 0.020637 | 0.807319 | 0.152564 | 0 | 0 | 0 | 0 | 0 |
| 411 | 0.019992 | 0.021122 | 0.80699 | 0.151895 | 0 | 0 | 0 | 0 | 0 |
| 412 | 0.020136 | 0.020156 | 0.806641 | 0.153058 | 0 | 0 | 0 | 0 | 0 |
| 413 | 0.01991 | 0.020179 | 0.806857 | 0.153054 | 0 | 0 | 0 | 0 | 0 |
| 414 | 0.019842 | 0.021309 | 0.807081 | 0.151767 | 0 | 0 | 0 | 0 | 0 |
| 415 | 0.020243 | 0.020934 | 0.807851 | 0.150973 | 0 | 0 | 0 | 0 | 0 |
| 416 | 0.020798 | 0.021544 | 0.804777 | 0.152861 | 0 | 0 | 0 | 0 | 0 |
| 417 | 0.019221 | 0.020842 | 0.808469 | 0.151468 | 0 | 0 | 0 | 0 | 0 |
| 418 | 0.0187 | 0.021181 | 0.809171 | 0.150948 | 0 | 0 | 0 | 0 | 0 |
| 419 | 0.020157 | 0.020925 | 0.805639 | 0.153278 | 0 | 0 | 0 | 0 | 0 |
| 420 | 0.019561 | 0.0203 | 0.805909 | 0.15423 | 0 | 0 | 0 | 0 | 0 |
| 421 | 0.01984 | 0.02176 | 0.8064 | 0.151999 | 0 | 0 | 0 | 0 | 0 |
| 422 | 0.019222 | 0.021347 | 0.807605 | 0.151826 | 0 | 0 | 0 | 0 | 0 |
| 423 | 0.01987 | 0.021041 | 0.806903 | 0.152186 | 0 | 0 | 0 | 0 | 0 |
| 424 | 0.019775 | 0.022183 | 0.807468 | 0.150564 | 0 | 0 | 0 | 0 | 0 |
| 425 | 0.020603 | 0.01993 | 0.804936 | 0.154531 | 0 | 0 | 0 | 0 | 0 |
| 426 | 0.020828 | 0.020708 | 0.802734 | 0.15573 | 0 | 0 | 0 | 0 | 0 |
| 427 | 0.019562 | 0.021644 | 0.807494 | 0.1513 | 0 | 0 | 0 | 0 | 0 |
| 428 | 0.019823 | 0.020612 | 0.810164 | 0.1494 | 0 | 0 | 0 | 0 | 0 |
| 429 | 0.020558 | 0.020949 | 0.806441 | 0.152052 | 0 | 0 | 0 | 0 | 0 |
| 430 | 0.020108 | 0.021599 | 0.805535 | 0.152758 | 0 | 0 | 0 | 0 | 0 |
| 431 | 0.019536 | 0.020624 | 0.808755 | 0.151086 | 0 | 0 | 0 | 0 | 0 |
| 432 | 0.019249 | 0.020604 | 0.807771 | 0.152376 | 0 | 0 | 0 | 0 | 0 |
| 433 | 0.020727 | 0.021913 | 0.803669 | 0.153692 | 0 | 0 | 0 | 0 | 0 |
| 434 | 0.020368 | 0.020537 | 0.806842 | 0.152253 | 0 | 0 | 0 | 0 | 0 |
| 435 | 0.020168 | 0.021287 | 0.807963 | 0.150582 | 0 | 0 | 0 | 0 | 0 |
| 436 | 0.019877 | 0.021057 | 0.806577 | 0.152489 | 0 | 0 | 0 | 0 | 0 |
| 437 | 0.020363 | 0.020313 | 0.807378 | 0.151946 | 0 | 0 | 0 | 0 | 0 |
| 438 | 0.020202 | 0.021207 | 0.80485 | 0.153741 | 0 | 0 | 0 | 0 | 0 |
| 439 | 0.020718 | 0.020587 | 0.807234 | 0.151461 | 0 | 0 | 0 | 0 | 0 |
| 440 | 0.020187 | 0.020487 | 0.805734 | 0.153593 | 0 | 0 | 0 | 0 | 0 |
| 441 | 0.018479 | 0.021027 | 0.810274 | 0.15022 | 0 | 0 | 0 | 0 | 0 |
| 442 | 0.019876 | 0.020842 | 0.807032 | 0.152251 | 0 | 0 | 0 | 0 | 0 |
| 443 | 0.018811 | 0.020424 | 0.807061 | 0.153704 | 0 | 0 | 0 | 0 | 0 |
| 444 | 0.020484 | 0.021691 | 0.805172 | 0.152653 | 0 | 0 | 0 | 0 | 0 |
| 445 | 0.020121 | 0.02087 | 0.80397 | 0.155039 | 0 | 0 | 0 | 0 | 0 |
| 446 | 0.020188 | 0.020328 | 0.807121 | 0.152363 | 0 | 0 | 0 | 0 | 0 |
| 447 | 0.019597 | 0.021095 | 0.808708 | 0.150591 | 0 | 0 | 0 | 0 | 0 |
| 448 | 0.020909 | 0.02183 | 0.803917 | 0.153334 | 0 | 0 | 0 | 0 | 0 |
| 449 | 0.019443 | 0.02083 | 0.806085 | 0.153641 | 0 | 0 | 0 | 0 | 0 |
| 450 | 0.020562 | 0.021675 | 0.80615 | 0.151614 | 0 | 0 | 0 | 0 | 0 |
| 451 | 0.019979 | 0.020793 | 0.808235 | 0.150993 | 0 | 0 | 0 | 0 | 0 |
| 452 | 0.020454 | 0.020043 | 0.807065 | 0.152439 | 0 | 0 | 0 | 0 | 0 |
| 453 | 0.019842 | 0.020841 | 0.80876 | 0.150558 | 0 | 0 | 0 | 0 | 0 |
| 454 | 0.019847 | 0.020284 | 0.809553 | 0.150316 | 0 | 0 | 0 | 0 | 0 |
| 455 | 0.019743 | 0.021184 | 0.810443 | 0.14863 | 0 | 0 | 0 | 0 | 0 |
| 456 | 0.019153 | 0.020695 | 0.807703 | 0.152449 | 0 | 0 | 0 | 0 | 0 |
| 457 | 0.019834 | 0.021001 | 0.805885 | 0.153259 | 0 | 0 | 0 | 0 | 0 |
| 458 | 0.020312 | 0.020844 | 0.807604 | 0.15124 | 0 | 0 | 0 | 0 | 0 |
| 459 | 0.01963 | 0.020437 | 0.80497 | 0.154964 | 0 | 0 | 0 | 0 | 0 |
| 460 | 0.019073 | 0.021269 | 0.803621 | 0.156037 | 0 | 0 | 0 | 0 | 0 |
| 461 | 0.019331 | 0.020685 | 0.809546 | 0.150438 | 0 | 0 | 0 | 0 | 0 |
| 462 | 0.020148 | 0.020108 | 0.807911 | 0.151832 | 0 | 0 | 0 | 0 | 0 |
| 463 | 0.019587 | 0.021224 | 0.806143 | 0.153046 | 0 | 0 | 0 | 0 | 0 |
| 464 | 0.019779 | 0.021291 | 0.807856 | 0.151045 | 0 | 0 | 0 | 0 | 0 |
| 465 | 0.01998 | 0.020849 | 0.807948 | 0.151223 | 0 | 0 | 0 | 0 | 0 |
| 466 | 0.019464 | 0.022396 | 0.802814 | 0.155326 | 0 | 0 | 0 | 0 | 0 |
| 467 | 0.020397 | 0.020016 | 0.805408 | 0.154169 | 0 | 0 | 0 | 0 | 0 |
| 468 | 0.020173 | 0.02072 | 0.807763 | 0.151344 | 0 | 0 | 0 | 0 | 0 |
| 469 | 0.020269 | 0.020319 | 0.808644 | 0.150768 | 0 | 0 | 0 | 0 | 0 |
| 470 | 0.019919 | 0.020647 | 0.808366 | 0.151068 | 0 | 0 | 0 | 0 | 0 |
| 471 | 0.019979 | 0.021527 | 0.809444 | 0.149051 | 0 | 0 | 0 | 0 | 0 |
| 472 | 0.020127 | 0.020806 | 0.806449 | 0.152618 | 0 | 0 | 0 | 0 | 0 |
| 473 | 0.019568 | 0.021318 | 0.806511 | 0.152603 | 0 | 0 | 0 | 0 | 0 |
| 474 | 0.020293 | 0.020967 | 0.805297 | 0.153433 | 0 | 0 | 0 | 0 | 0 |
| 475 | 0.020368 | 0.020517 | 0.805726 | 0.153388 | 0 | 0 | 0 | 0 | 0 |
| 476 | 0.020397 | 0.021366 | 0.804635 | 0.153603 | 0 | 0 | 0 | 0 | 0 |
| 477 | 0.018868 | 0.020561 | 0.806936 | 0.153634 | 0 | 0 | 0 | 0 | 0 |
| 478 | 0.019591 | 0.021411 | 0.805324 | 0.153674 | 0 | 0 | 0 | 0 | 0 |
| 479 | 0.020072 | 0.0214 | 0.808081 | 0.150447 | 0 | 0 | 0 | 0 | 0 |
| 480 | 0.019637 | 0.020524 | 0.804023 | 0.155816 | 0 | 0 | 0 | 0 | 0 |
| 481 | 0.019225 | 0.021186 | 0.812811 | 0.146778 | 0 | 0 | 0 | 0 | 0 |
| 482 | 0.019732 | 0.020371 | 0.80715 | 0.152747 | 0 | 0 | 0 | 0 | 0 |
| 483 | 0.020279 | 0.020699 | 0.807805 | 0.151217 | 0 | 0 | 0 | 0 | 0 |
| 484 | 0.020676 | 0.020976 | 0.803827 | 0.154522 | 0 | 0 | 0 | 0 | 0 |
| 485 | 0.019343 | 0.020789 | 0.809525 | 0.150343 | 0 | 0 | 0 | 0 | 0 |
| 486 | 0.018867 | 0.020781 | 0.808839 | 0.151513 | 0 | 0 | 0 | 0 | 0 |
| 487 | 0.019597 | 0.020688 | 0.808972 | 0.150743 | 0 | 0 | 0 | 0 | 0 |
| 488 | 0.019353 | 0.020713 | 0.808654 | 0.15128 | 0 | 0 | 0 | 0 | 0 |
| 489 | 0.019911 | 0.020421 | 0.807523 | 0.152145 | 0 | 0 | 0 | 0 | 0 |
| 490 | 0.019459 | 0.021592 | 0.80642 | 0.152529 | 0 | 0 | 0 | 0 | 0 |
| 491 | 0.019414 | 0.021421 | 0.80563 | 0.153534 | 0 | 0 | 0 | 0 | 0 |
| 492 | 0.019792 | 0.021384 | 0.802951 | 0.155873 | 0 | 0 | 0 | 0 | 0 |
| 493 | 0.020485 | 0.020585 | 0.808394 | 0.150535 | 0 | 0 | 0 | 0 | 0 |
| 494 | 0.020359 | 0.021434 | 0.802574 | 0.155623 | 0 | 0 | 0 | 0 | 0 |
| 495 | 0.020764 | 0.021388 | 0.804775 | 0.153073 | 0 | 0 | 0 | 0 | 0 |
| 496 | 0.020433 | 0.020674 | 0.804317 | 0.154525 | 0 | 0 | 0 | 0 | 0 |
| 497 | 0.02031 | 0.02044 | 0.807968 | 0.151273 | 0 | 0 | 0 | 0 | 0 |
| 498 | 0.019435 | 0.020624 | 0.808036 | 0.151906 | 0 | 0 | 0 | 0 | 0 |
| 499 | 0.019503 | 0.021061 | 0.804618 | 0.154818 | 0 | 0 | 0 | 0 | 0 |
| 500 | 0.019974 | 0.021017 | 0.805722 | 0.153287 | 0 | 0 | 0 | 0 | 0 |
| 501 | 0.020008 | 0.021898 | 0.806259 | 0.151835 | 0 | 0 | 0 | 0 | 0 |
| 502 | 0.020102 | 0.020651 | 0.808332 | 0.150915 | 0 | 0 | 0 | 0 | 0 |
| 503 | 0.020246 | 0.020927 | 0.806455 | 0.152373 | 0 | 0 | 0 | 0 | 0 |
| 504 | 0.019638 | 0.021038 | 0.808474 | 0.15085 | 0 | 0 | 0 | 0 | 0 |
| 505 | 0.019787 | 0.020279 | 0.810272 | 0.149652 | 0 | 0 | 0 | 0 | 0 |
| 506 | 0.01969 | 0.0193 | 0.809254 | 0.151756 | 0 | 0 | 0 | 0 | 0 |
| 507 | 0.019318 | 0.020539 | 0.807351 | 0.152792 | 0 | 0 | 0 | 0 | 0 |
| 508 | 0.019919 | 0.020387 | 0.807496 | 0.152198 | 0 | 0 | 0 | 0 | 0 |
| 509 | 0.019204 | 0.020628 | 0.809694 | 0.150474 | 0 | 0 | 0 | 0 | 0 |
| 510 | 0.020444 | 0.020615 | 0.808288 | 0.150653 | 0 | 0 | 0 | 0 | 0 |
| 511 | 0.01989 | 0.021129 | 0.805864 | 0.153117 | 0 | 0 | 0 | 0 | 0 |
| 512 | 0.019689 | 0.021301 | 0.805887 | 0.153124 | 0 | 0 | 0 | 0 | 0 |
| 513 | 0.019985 | 0.021028 | 0.808485 | 0.150503 | 0 | 0 | 0 | 0 | 0 |
| 514 | 0.020101 | 0.020441 | 0.80662 | 0.152838 | 0 | 0 | 0 | 0 | 0 |
| 515 | 0.020036 | 0.019935 | 0.806867 | 0.153162 | 0 | 0 | 0 | 0 | 0 |
| 516 | 0.018781 | 0.020681 | 0.808173 | 0.152366 | 0 | 0 | 0 | 0 | 0 |
| 517 | 0.019843 | 0.020003 | 0.808255 | 0.151898 | 0 | 0 | 0 | 0 | 0 |
| 518 | 0.019765 | 0.021354 | 0.806107 | 0.152774 | 0 | 0 | 0 | 0 | 0 |
| 519 | 0.019449 | 0.020436 | 0.803844 | 0.156251 | 0 | 0 | 0 | 0 | 0 |
| 520 | 0.021131 | 0.020359 | 0.80428 | 0.15422 | 0 | 0 | 0 | 0 | 0 |
| 521 | 0.019805 | 0.020154 | 0.810358 | 0.149684 | 0 | 0 | 0 | 0 | 0 |
| 522 | 0.019936 | 0.020116 | 0.809846 | 0.150101 | 0 | 0 | 0 | 0 | 0 |
| 523 | 0.019751 | 0.020605 | 0.807163 | 0.152481 | 0 | 0 | 0 | 0 | 0 |
| 524 | 0.020708 | 0.020617 | 0.802327 | 0.156348 | 0 | 0 | 0 | 0 | 0 |
| 525 | 0.019491 | 0.020941 | 0.80793 | 0.151638 | 0 | 0 | 0 | 0 | 0 |
| 526 | 0.01921 | 0.020208 | 0.806853 | 0.153729 | 0 | 0 | 0 | 0 | 0 |
| 527 | 0.019212 | 0.020727 | 0.808054 | 0.152008 | 0 | 0 | 0 | 0 | 0 |
| 528 | 0.02112 | 0.020678 | 0.807661 | 0.150541 | 0 | 0 | 0 | 0 | 0 |
| 529 | 0.019701 | 0.020467 | 0.80775 | 0.152082 | 0 | 0 | 0 | 0 | 0 |
| 530 | 0.01925 | 0.021463 | 0.804401 | 0.154886 | 0 | 0 | 0 | 0 | 0 |
| 531 | 0.01912 | 0.021089 | 0.806047 | 0.153743 | 0 | 0 | 0 | 0 | 0 |
| 532 | 0.020466 | 0.020677 | 0.809113 | 0.149743 | 0 | 0 | 0 | 0 | 0 |
| 533 | 0.019852 | 0.020519 | 0.806122 | 0.153506 | 0 | 0 | 0 | 0 | 0 |
| 534 | 0.020516 | 0.020746 | 0.802627 | 0.156112 | 0 | 0 | 0 | 0 | 0 |
| 535 | 0.019283 | 0.02098 | 0.806694 | 0.153043 | 0 | 0 | 0 | 0 | 0 |
| 536 | 0.019353 | 0.021331 | 0.807022 | 0.152264 | 0 | 0 | 0 | 0 | 0 |
| 537 | 0.02005 | 0.01984 | 0.80918 | 0.150929 | 0 | 0 | 0 | 0 | 0 |
| 538 | 0.020379 | 0.020379 | 0.804306 | 0.154926 | 0 | 0 | 0 | 0 | 0 |
| 539 | 0.02039 | 0.021206 | 0.80673 | 0.151674 | 0 | 0 | 0 | 0 | 0 |
| 540 | 0.019969 | 0.021278 | 0.803792 | 0.15496 | 0 | 0 | 0 | 0 | 0 |
| 541 | 0.01994 | 0.020379 | 0.807581 | 0.1521 | 0 | 0 | 0 | 0 | 0 |
| 542 | 0.019563 | 0.021296 | 0.805948 | 0.153193 | 0 | 0 | 0 | 0 | 0 |
| 543 | 0.019656 | 0.021205 | 0.810583 | 0.148556 | 0 | 0 | 0 | 0 | 0 |
| 544 | 0.020689 | 0.020809 | 0.8068 | 0.151692 | 0 | 0 | 0 | 0 | 0 |
| 545 | 0.020927 | 0.021425 | 0.804574 | 0.153064 | 0 | 0 | 0 | 0 | 0 |
| 546 | 0.01995 | 0.021608 | 0.805448 | 0.152994 | 0 | 0 | 0 | 0 | 0 |
| 547 | 0.020923 | 0.020352 | 0.805927 | 0.152798 | 0 | 0 | 0 | 0 | 0 |
| 548 | 0.019779 | 0.021201 | 0.807789 | 0.151231 | 0 | 0 | 0 | 0 | 0 |
| 549 | 0.019568 | 0.021024 | 0.805288 | 0.15412 | 0 | 0 | 0 | 0 | 0 |
| 550 | 0.020618 | 0.020157 | 0.809394 | 0.149831 | 0 | 0 | 0 | 0 | 0 |
| 551 | 0.01913 | 0.021308 | 0.806673 | 0.152889 | 0 | 0 | 0 | 0 | 0 |
| 552 | 0.021237 | 0.020568 | 0.805776 | 0.152418 | 0 | 0 | 0 | 0 | 0 |
| 553 | 0.01994 | 0.020381 | 0.805679 | 0.154001 | 0 | 0 | 0 | 0 | 0 |
| 554 | 0.020111 | 0.020529 | 0.807322 | 0.152029 | 0 | 0 | 0 | 0 | 0 |
| 555 | 0.019868 | 0.019939 | 0.808267 | 0.151926 | 0 | 0 | 0 | 0 | 0 |
| 556 | 0.02006 | 0.020818 | 0.80817 | 0.150952 | 0 | 0 | 0 | 0 | 0 |
| 557 | 0.019683 | 0.022258 | 0.806562 | 0.151497 | 0 | 0 | 0 | 0 | 0 |
| 558 | 0.020225 | 0.020705 | 0.805 | 0.154071 | 0 | 0 | 0 | 0 | 0 |
| 559 | 0.019184 | 0.020116 | 0.807636 | 0.153064 | 0 | 0 | 0 | 0 | 0 |
| 560 | 0.020476 | 0.020163 | 0.808861 | 0.1505 | 0 | 0 | 0 | 0 | 0 |
| 561 | 0.020781 | 0.02113 | 0.804116 | 0.153973 | 0 | 0 | 0 | 0 | 0 |
| 562 | 0.019794 | 0.021282 | 0.806801 | 0.152122 | 0 | 0 | 0 | 0 | 0 |
| 563 | 0.019731 | 0.021151 | 0.805422 | 0.153696 | 0 | 0 | 0 | 0 | 0 |
| 564 | 0.019822 | 0.020934 | 0.809211 | 0.150033 | 0 | 0 | 0 | 0 | 0 |
| 565 | 0.020189 | 0.020448 | 0.80389 | 0.155473 | 0 | 0 | 0 | 0 | 0 |
| 566 | 0.019896 | 0.021497 | 0.803554 | 0.155053 | 0 | 0 | 0 | 0 | 0 |
| 567 | 0.019641 | 0.021152 | 0.807137 | 0.15207 | 0 | 0 | 0 | 0 | 0 |
| 568 | 0.020144 | 0.020901 | 0.808522 | 0.150423 | 0 | 0 | 0 | 0 | 0 |
| 569 | 0.019814 | 0.021389 | 0.807888 | 0.150909 | 0 | 0 | 0 | 0 | 0 |
| 570 | 0.020646 | 0.020777 | 0.80696 | 0.151617 | 0 | 0 | 0 | 0 | 0 |
| 571 | 0.020463 | 0.020373 | 0.806515 | 0.152649 | 0 | 0 | 0 | 0 | 0 |
| 572 | 0.019494 | 0.019912 | 0.806905 | 0.153689 | 0 | 0 | 0 | 0 | 0 |
| 573 | 0.02051 | 0.02007 | 0.806729 | 0.152692 | 0 | 0 | 0 | 0 | 0 |
| 574 | 0.019332 | 0.020736 | 0.806703 | 0.153209 | 0 | 0 | 0 | 0 | 0 |
| 575 | 0.019698 | 0.019509 | 0.808959 | 0.151834 | 0 | 0 | 0 | 0 | 0 |
| 576 | 0.020343 | 0.020033 | 0.808393 | 0.15123 | 0 | 0 | 0 | 0 | 0 |
| 577 | 0.020454 | 0.021579 | 0.80331 | 0.154657 | 0 | 0 | 0 | 0 | 0 |
| 578 | 0.020223 | 0.020632 | 0.80757 | 0.151574 | 0 | 0 | 0 | 0 | 0 |
| 579 | 0.019428 | 0.020208 | 0.806775 | 0.15359 | 0 | 0 | 0 | 0 | 0 |
| 580 | 0.019945 | 0.020567 | 0.807473 | 0.152015 | 0 | 0 | 0 | 0 | 0 |
| 581 | 0.018768 | 0.021152 | 0.807511 | 0.152569 | 0 | 0 | 0 | 0 | 0 |
| 582 | 0.020792 | 0.021001 | 0.806095 | 0.152112 | 0 | 0 | 0 | 0 | 0 |
| 583 | 0.019207 | 0.020278 | 0.806588 | 0.153927 | 0 | 0 | 0 | 0 | 0 |
| 584 | 0.019657 | 0.020506 | 0.807629 | 0.152208 | 0 | 0 | 0 | 0 | 0 |
| 585 | 0.020443 | 0.021348 | 0.806867 | 0.151343 | 0 | 0 | 0 | 0 | 0 |
| 586 | 0.02025 | 0.021502 | 0.804196 | 0.154051 | 0 | 0 | 0 | 0 | 0 |
| 587 | 0.020122 | 0.020052 | 0.808195 | 0.151631 | 0 | 0 | 0 | 0 | 0 |
| 588 | 0.019175 | 0.020989 | 0.807429 | 0.152406 | 0 | 0 | 0 | 0 | 0 |
| 589 | 0.019503 | 0.020321 | 0.809802 | 0.150374 | 0 | 0 | 0 | 0 | 0 |
| 590 | 0.018959 | 0.020052 | 0.804706 | 0.156284 | 0 | 0 | 0 | 0 | 0 |
| 591 | 0.020265 | 0.020065 | 0.807007 | 0.152663 | 0 | 0 | 0 | 0 | 0 |
| 592 | 0.020313 | 0.021372 | 0.807786 | 0.15053 | 0 | 0 | 0 | 0 | 0 |
| 593 | 0.020025 | 0.020425 | 0.808296 | 0.151253 | 0 | 0 | 0 | 0 | 0 |
| 594 | 0.019698 | 0.019558 | 0.809313 | 0.15143 | 0 | 0 | 0 | 0 | 0 |
| 595 | 0.019939 | 0.02109 | 0.807815 | 0.151156 | 0 | 0 | 0 | 0 | 0 |
| 596 | 0.019793 | 0.020517 | 0.80838 | 0.15131 | 0 | 0 | 0 | 0 | 0 |
| 597 | 0.020489 | 0.02088 | 0.805117 | 0.153514 | 0 | 0 | 0 | 0 | 0 |
| 598 | 0.019525 | 0.020526 | 0.811002 | 0.148948 | 0 | 0 | 0 | 0 | 0 |
| 599 | 0.019173 | 0.021257 | 0.807853 | 0.151718 | 0 | 0 | 0 | 0 | 0 |
| 600 | 0.020534 | 0.021188 | 0.805316 | 0.152942 | 0 | 0 | 0 | 0 | 0 |
| 601 | 0.019187 | 0.020758 | 0.807065 | 0.15299 | 0 | 0 | 0 | 0 | 0 |
| 602 | 0.020059 | 0.021083 | 0.808014 | 0.150844 | 0 | 0 | 0 | 0 | 0 |
| 603 | 0.020527 | 0.022023 | 0.806654 | 0.150796 | 0 | 0 | 0 | 0 | 0 |
| 604 | 0.020498 | 0.020778 | 0.806524 | 0.1522 | 0 | 0 | 0 | 0 | 0 |
| 605 | 0.01943 | 0.0206 | 0.807774 | 0.152197 | 0 | 0 | 0 | 0 | 0 |
| 606 | 0.018766 | 0.020515 | 0.808114 | 0.152606 | 0 | 0 | 0 | 0 | 0 |
| 607 | 0.020398 | 0.021757 | 0.807352 | 0.150493 | 0 | 0 | 0 | 0 | 0 |
| 608 | 0.020037 | 0.020803 | 0.804519 | 0.154641 | 0 | 0 | 0 | 0 | 0 |
| 609 | 0.02067 | 0.021011 | 0.807586 | 0.150732 | 0 | 0 | 0 | 0 | 0 |
| 610 | 0.020203 | 0.020032 | 0.80961 | 0.150155 | 0 | 0 | 0 | 0 | 0 |
| 611 | 0.019604 | 0.020581 | 0.809669 | 0.150146 | 0 | 0 | 0 | 0 | 0 |
| 612 | 0.021126 | 0.020441 | 0.804423 | 0.15401 | 0 | 0 | 0 | 0 | 0 |
| 613 | 0.020709 | 0.02083 | 0.80416 | 0.154271 | 0 | 0 | 0 | 0 | 0 |
| 614 | 0.019956 | 0.021216 | 0.809646 | 0.149182 | 0 | 0 | 0 | 0 | 0 |
| 615 | 0.019965 | 0.020535 | 0.808886 | 0.150605 | 0 | 0 | 0 | 0 | 0 |
| 616 | 0.019984 | 0.021511 | 0.805595 | 0.15291 | 0 | 0 | 0 | 0 | 0 |
| 617 | 0.020158 | 0.02198 | 0.806216 | 0.151646 | 0 | 0 | 0 | 0 | 0 |
| 618 | 0.020111 | 0.020871 | 0.806018 | 0.152999 | 0 | 0 | 0 | 0 | 0 |
| 619 | 0.018818 | 0.020683 | 0.80743 | 0.153069 | 0 | 0 | 0 | 0 | 0 |
| 620 | 0.019685 | 0.020638 | 0.807816 | 0.151861 | 0 | 0 | 0 | 0 | 0 |
| 621 | 0.02027 | 0.021126 | 0.807989 | 0.150615 | 0 | 0 | 0 | 0 | 0 |
| 622 | 0.01948 | 0.021003 | 0.805341 | 0.154176 | 0 | 0 | 0 | 0 | 0 |
| 623 | 0.019954 | 0.020655 | 0.808134 | 0.151257 | 0 | 0 | 0 | 0 | 0 |
| 624 | 0.020022 | 0.020884 | 0.806431 | 0.152663 | 0 | 0 | 0 | 0 | 0 |
| 625 | 0.019889 | 0.020531 | 0.808417 | 0.151152 | 0 | 0 | 0 | 0 | 0 |
| 626 | 0.019408 | 0.021397 | 0.807307 | 0.151888 | 0 | 0 | 0 | 0 | 0 |
| 627 | 0.020233 | 0.019993 | 0.807683 | 0.152091 | 0 | 0 | 0 | 0 | 0 |
| 628 | 0.019565 | 0.020673 | 0.807022 | 0.15274 | 0 | 0 | 0 | 0 | 0 |
| 629 | 0.019642 | 0.020081 | 0.809339 | 0.150939 | 0 | 0 | 0 | 0 | 0 |
| 630 | 0.019679 | 0.02044 | 0.805918 | 0.153963 | 0 | 0 | 0 | 0 | 0 |
| 631 | 0.019977 | 0.020966 | 0.803202 | 0.155854 | 0 | 0 | 0 | 0 | 0 |
| 632 | 0.02047 | 0.020909 | 0.808499 | 0.150122 | 0 | 0 | 0 | 0 | 0 |
| 633 | 0.019705 | 0.019174 | 0.807556 | 0.153565 | 0 | 0 | 0 | 0 | 0 |
| 634 | 0.019617 | 0.020715 | 0.806422 | 0.153246 | 0 | 0 | 0 | 0 | 0 |
| 635 | 0.020051 | 0.020404 | 0.807094 | 0.152421 | 0 | 0 | 0 | 0 | 0 |
| 636 | 0.020878 | 0.01983 | 0.806326 | 0.152966 | 0 | 0 | 0 | 0 | 0 |
| 637 | 0.019379 | 0.020338 | 0.807362 | 0.152922 | 0 | 0 | 0 | 0 | 0 |
| 638 | 0.019374 | 0.021125 | 0.807541 | 0.151959 | 0 | 0 | 0 | 0 | 0 |
| 639 | 0.019857 | 0.020927 | 0.804503 | 0.154713 | 0 | 0 | 0 | 0 | 0 |
| 640 | 0.020422 | 0.021145 | 0.802411 | 0.156022 | 0 | 0 | 0 | 0 | 0 |
| 641 | 0.021086 | 0.021156 | 0.805993 | 0.151765 | 0 | 0 | 0 | 0 | 0 |
| 642 | 0.019778 | 0.020667 | 0.807887 | 0.151667 | 0 | 0 | 0 | 0 | 0 |
| 643 | 0.019944 | 0.020659 | 0.807427 | 0.15197 | 0 | 0 | 0 | 0 | 0 |
| 644 | 0.019837 | 0.021811 | 0.808889 | 0.149462 | 0 | 0 | 0 | 0 | 0 |
| 645 | 0.019918 | 0.020978 | 0.80501 | 0.154073 | 0 | 0 | 0 | 0 | 0 |
| 646 | 0.019856 | 0.021034 | 0.806798 | 0.152313 | 0 | 0 | 0 | 0 | 0 |
| 647 | 0.020312 | 0.020731 | 0.807403 | 0.151555 | 0 | 0 | 0 | 0 | 0 |
| 648 | 0.019489 | 0.021291 | 0.805902 | 0.153319 | 0 | 0 | 0 | 0 | 0 |
| 649 | 0.019584 | 0.022032 | 0.806928 | 0.151456 | 0 | 0 | 0 | 0 | 0 |
| 650 | 0.020336 | 0.020804 | 0.806858 | 0.152003 | 0 | 0 | 0 | 0 | 0 |
| 651 | 0.02043 | 0.020781 | 0.807502 | 0.151287 | 0 | 0 | 0 | 0 | 0 |
| 652 | 0.020105 | 0.020918 | 0.805505 | 0.153472 | 0 | 0 | 0 | 0 | 0 |
| 653 | 0.019759 | 0.019216 | 0.81029 | 0.150735 | 0 | 0 | 0 | 0 | 0 |
| 654 | 0.020516 | 0.020916 | 0.805978 | 0.152581 | 0 | 0 | 0 | 0 | 0 |
| 655 | 0.019817 | 0.02099 | 0.804839 | 0.154354 | 0 | 0 | 0 | 0 | 0 |
| 656 | 0.019844 | 0.020851 | 0.808922 | 0.150382 | 0 | 0 | 0 | 0 | 0 |
| 657 | 0.019563 | 0.020742 | 0.80894 | 0.150756 | 0 | 0 | 0 | 0 | 0 |
| 658 | 0.020024 | 0.020273 | 0.806454 | 0.153249 | 0 | 0 | 0 | 0 | 0 |
| 659 | 0.019658 | 0.021125 | 0.80488 | 0.154338 | 0 | 0 | 0 | 0 | 0 |
| 660 | 0.020287 | 0.022256 | 0.806275 | 0.151182 | 0 | 0 | 0 | 0 | 0 |
| 661 | 0.019763 | 0.021963 | 0.804898 | 0.153375 | 0 | 0 | 0 | 0 | 0 |
| 662 | 0.019711 | 0.020885 | 0.807024 | 0.15236 | 0 | 0 | 0 | 0 | 0 |
| 663 | 0.0206 | 0.020209 | 0.805843 | 0.153348 | 0 | 0 | 0 | 0 | 0 |
| 664 | 0.019612 | 0.021694 | 0.809042 | 0.149633 | 0 | 0 | 0 | 0 | 0 |
| 665 | 0.020627 | 0.021117 | 0.803059 | 0.155197 | 0 | 0 | 0 | 0 | 0 |
| 666 | 0.019797 | 0.021222 | 0.806213 | 0.152768 | 0 | 0 | 0 | 0 | 0 |
| 667 | 0.019947 | 0.021011 | 0.808434 | 0.150608 | 0 | 0 | 0 | 0 | 0 |
| 668 | 0.020217 | 0.020686 | 0.804782 | 0.154315 | 0 | 0 | 0 | 0 | 0 |
| 669 | 0.019833 | 0.021575 | 0.804623 | 0.153969 | 0 | 0 | 0 | 0 | 0 |
| 670 | 0.020491 | 0.019632 | 0.805133 | 0.154735 | 0 | 0 | 0 | 0 | 0 |
| 671 | 0.019632 | 0.019622 | 0.807938 | 0.152808 | 0 | 0 | 0 | 0 | 0 |
| 672 | 0.01953 | 0.020783 | 0.804875 | 0.154812 | 0 | 0 | 0 | 0 | 0 |
| 673 | 0.02002 | 0.021353 | 0.806579 | 0.152047 | 0 | 0 | 0 | 0 | 0 |
| 674 | 0.020123 | 0.020303 | 0.806218 | 0.153356 | 0 | 0 | 0 | 0 | 0 |
| 675 | 0.019487 | 0.020055 | 0.807779 | 0.15268 | 0 | 0 | 0 | 0 | 0 |
| 676 | 0.01997 | 0.020996 | 0.807197 | 0.151837 | 0 | 0 | 0 | 0 | 0 |
| 677 | 0.020175 | 0.020205 | 0.807998 | 0.151622 | 0 | 0 | 0 | 0 | 0 |
| 678 | 0.020434 | 0.020384 | 0.805604 | 0.153579 | 0 | 0 | 0 | 0 | 0 |
| 679 | 0.020226 | 0.020316 | 0.807891 | 0.151567 | 0 | 0 | 0 | 0 | 0 |
| 680 | 0.020205 | 0.020786 | 0.803514 | 0.155495 | 0 | 0 | 0 | 0 | 0 |
| 681 | 0.019913 | 0.020653 | 0.80485 | 0.154554 | 0 | 0 | 0 | 0 | 0 |
| 682 | 0.01951 | 0.020474 | 0.807276 | 0.152739 | 0 | 0 | 0 | 0 | 0 |
| 683 | 0.019163 | 0.021869 | 0.807559 | 0.151409 | 0 | 0 | 0 | 0 | 0 |
| 684 | 0.019776 | 0.019606 | 0.809283 | 0.151335 | 0 | 0 | 0 | 0 | 0 |
| 685 | 0.020172 | 0.020877 | 0.809547 | 0.149403 | 0 | 0 | 0 | 0 | 0 |
| 686 | 0.019666 | 0.020186 | 0.805079 | 0.155009 | 0 | 0 | 0 | 0 | 0 |
| 687 | 0.020057 | 0.020962 | 0.806344 | 0.152638 | 0 | 0 | 0 | 0 | 0 |
| 688 | 0.019971 | 0.02028 | 0.806591 | 0.153158 | 0 | 0 | 0 | 0 | 0 |
| 689 | 0.019332 | 0.022566 | 0.803119 | 0.154984 | 0 | 0 | 0 | 0 | 0 |
| 690 | 0.019468 | 0.020855 | 0.807426 | 0.152251 | 0 | 0 | 0 | 0 | 0 |
| 691 | 0.020576 | 0.021106 | 0.806482 | 0.151837 | 0 | 0 | 0 | 0 | 0 |
| 692 | 0.019831 | 0.021055 | 0.806661 | 0.152453 | 0 | 0 | 0 | 0 | 0 |
| 693 | 0.020133 | 0.020233 | 0.802799 | 0.156834 | 0 | 0 | 0 | 0 | 0 |
| 694 | 0.020093 | 0.021054 | 0.806042 | 0.152811 | 0 | 0 | 0 | 0 | 0 |
| 695 | 0.020143 | 0.02049 | 0.80671 | 0.152656 | 0 | 0 | 0 | 0 | 0 |
| 696 | 0.02013 | 0.020509 | 0.810197 | 0.149165 | 0 | 0 | 0 | 0 | 0 |
| 697 | 0.020335 | 0.020984 | 0.805798 | 0.152884 | 0 | 0 | 0 | 0 | 0 |
| 698 | 0.019925 | 0.020885 | 0.807906 | 0.151284 | 0 | 0 | 0 | 0 | 0 |
| 699 | 0.019166 | 0.020375 | 0.809034 | 0.151425 | 0 | 0 | 0 | 0 | 0 |
| 700 | 0.019672 | 0.021358 | 0.807044 | 0.151926 | 0 | 0 | 0 | 0 | 0 |
| 701 | 0.019815 | 0.021778 | 0.809437 | 0.148971 | 0 | 0 | 0 | 0 | 0 |
| 702 | 0.0206 | 0.021019 | 0.806243 | 0.152138 | 0 | 0 | 0 | 0 | 0 |
| 703 | 0.0194 | 0.021055 | 0.808041 | 0.151504 | 0 | 0 | 0 | 0 | 0 |
| 704 | 0.020263 | 0.01983 | 0.80736 | 0.152548 | 0 | 0 | 0 | 0 | 0 |
| 705 | 0.019043 | 0.020194 | 0.808679 | 0.152084 | 0 | 0 | 0 | 0 | 0 |
| 706 | 0.019392 | 0.022069 | 0.806194 | 0.152344 | 0 | 0 | 0 | 0 | 0 |
| 707 | 0.020413 | 0.020777 | 0.806342 | 0.152468 | 0 | 0 | 0 | 0 | 0 |
| 708 | 0.019956 | 0.021624 | 0.806093 | 0.152326 | 0 | 0 | 0 | 0 | 0 |
| 709 | 0.019268 | 0.020255 | 0.81061 | 0.149867 | 0 | 0 | 0 | 0 | 0 |
| 710 | 0.019721 | 0.020501 | 0.808642 | 0.151136 | 0 | 0 | 0 | 0 | 0 |
| 711 | 0.019169 | 0.020887 | 0.806521 | 0.153423 | 0 | 0 | 0 | 0 | 0 |
| 712 | 0.01997 | 0.020734 | 0.810433 | 0.148863 | 0 | 0 | 0 | 0 | 0 |
| 713 | 0.019115 | 0.02067 | 0.806789 | 0.153417 | 0 | 0 | 0 | 0 | 0 |
| 714 | 0.019875 | 0.021219 | 0.80612 | 0.152786 | 0 | 0 | 0 | 0 | 0 |
| 715 | 0.020379 | 0.02041 | 0.804176 | 0.155035 | 0 | 0 | 0 | 0 | 0 |
| 716 | 0.019337 | 0.021657 | 0.803514 | 0.155492 | 0 | 0 | 0 | 0 | 0 |
| 717 | 0.020685 | 0.020604 | 0.806611 | 0.1521 | 0 | 0 | 0 | 0 | 0 |
| 718 | 0.019924 | 0.020671 | 0.809559 | 0.149846 | 0 | 0 | 0 | 0 | 0 |
| 719 | 0.020155 | 0.020988 | 0.807481 | 0.151376 | 0 | 0 | 0 | 0 | 0 |
| 720 | 0.020848 | 0.021308 | 0.802705 | 0.155109 | 0 | 0 | 0 | 0 | 0 |
| 721 | 0.019295 | 0.020895 | 0.804849 | 0.15496 | 0 | 0 | 0 | 0 | 0 |
| 722 | 0.020125 | 0.021597 | 0.803787 | 0.154492 | 0 | 0 | 0 | 0 | 0 |
| 723 | 0.020165 | 0.020615 | 0.805454 | 0.153765 | 0 | 0 | 0 | 0 | 0 |
| 724 | 0.02043 | 0.021586 | 0.805474 | 0.15251 | 0 | 0 | 0 | 0 | 0 |
| 725 | 0.01991 | 0.020731 | 0.806082 | 0.153276 | 0 | 0 | 0 | 0 | 0 |
| 726 | 0.019982 | 0.020949 | 0.807496 | 0.151572 | 0 | 0 | 0 | 0 | 0 |
| 727 | 0.019679 | 0.020789 | 0.806468 | 0.153064 | 0 | 0 | 0 | 0 | 0 |
| 728 | 0.019887 | 0.020007 | 0.80666 | 0.153445 | 0 | 0 | 0 | 0 | 0 |
| 729 | 0.019588 | 0.021186 | 0.806705 | 0.152521 | 0 | 0 | 0 | 0 | 0 |
| 730 | 0.019814 | 0.02101 | 0.80593 | 0.153246 | 0 | 0 | 0 | 0 | 0 |
| 731 | 0.019877 | 0.021087 | 0.807967 | 0.151069 | 0 | 0 | 0 | 0 | 0 |
| 732 | 0.019804 | 0.021977 | 0.805523 | 0.152696 | 0 | 0 | 0 | 0 | 0 |
| 733 | 0.019801 | 0.020733 | 0.808157 | 0.151309 | 0 | 0 | 0 | 0 | 0 |
| 734 | 0.019905 | 0.021003 | 0.80464 | 0.154452 | 0 | 0 | 0 | 0 | 0 |
| 735 | 0.019805 | 0.020751 | 0.808403 | 0.151031 | 0 | 0 | 0 | 0 | 0 |
| 736 | 0.019719 | 0.020893 | 0.808251 | 0.151137 | 0 | 0 | 0 | 0 | 0 |
| 737 | 0.019982 | 0.020891 | 0.807851 | 0.151275 | 0 | 0 | 0 | 0 | 0 |
| 738 | 0.019544 | 0.020593 | 0.807586 | 0.152277 | 0 | 0 | 0 | 0 | 0 |
| 739 | 0.019763 | 0.02112 | 0.805885 | 0.153232 | 0 | 0 | 0 | 0 | 0 |
| 740 | 0.020017 | 0.020675 | 0.810364 | 0.148934 | 0 | 0 | 0 | 0 | 0 |
| 741 | 0.020363 | 0.02074 | 0.80715 | 0.151747 | 0 | 0 | 0 | 0 | 0 |
| 742 | 0.020494 | 0.020855 | 0.806837 | 0.151814 | 0 | 0 | 0 | 0 | 0 |
| 743 | 0.020133 | 0.020823 | 0.806443 | 0.152601 | 0 | 0 | 0 | 0 | 0 |
| 744 | 0.020117 | 0.020578 | 0.808464 | 0.150811 | 0 | 0 | 0 | 0 | 0 |
| 745 | 0.020022 | 0.020523 | 0.810171 | 0.149254 | 0 | 0 | 0 | 0 | 0 |
| 746 | 0.020432 | 0.020412 | 0.805462 | 0.153693 | 0 | 0 | 0 | 0 | 0 |
| 747 | 0.020123 | 0.021598 | 0.804936 | 0.153333 | 0 | 0 | 0 | 0 | 0 |
| 748 | 0.02016 | 0.020711 | 0.809931 | 0.149199 | 0 | 0 | 0 | 0 | 0 |
| 749 | 0.019303 | 0.021309 | 0.808987 | 0.1504 | 0 | 0 | 0 | 0 | 0 |
| 750 | 0.020972 | 0.020393 | 0.809276 | 0.14936 | 0 | 0 | 0 | 0 | 0 |
| 751 | 0.019431 | 0.020914 | 0.808653 | 0.151002 | 0 | 0 | 0 | 0 | 0 |
| 752 | 0.01932 | 0.021076 | 0.80687 | 0.152734 | 0 | 0 | 0 | 0 | 0 |
| 753 | 0.019228 | 0.020038 | 0.809255 | 0.151478 | 0 | 0 | 0 | 0 | 0 |
| 754 | 0.02035 | 0.020873 | 0.806568 | 0.152209 | 0 | 0 | 0 | 0 | 0 |
| 755 | 0.01976 | 0.020975 | 0.806727 | 0.152538 | 0 | 0 | 0 | 0 | 0 |
| 756 | 0.020292 | 0.0215 | 0.804671 | 0.153536 | 0 | 0 | 0 | 0 | 0 |
| 757 | 0.019774 | 0.020176 | 0.80646 | 0.153589 | 0 | 0 | 0 | 0 | 0 |
| 758 | 0.019973 | 0.021203 | 0.805743 | 0.153081 | 0 | 0 | 0 | 0 | 0 |
| 759 | 0.019196 | 0.020219 | 0.807517 | 0.153068 | 0 | 0 | 0 | 0 | 0 |
| 760 | 0.019735 | 0.021119 | 0.804884 | 0.154262 | 0 | 0 | 0 | 0 | 0 |
| 761 | 0.019492 | 0.020789 | 0.804825 | 0.154895 | 0 | 0 | 0 | 0 | 0 |
| 762 | 0.020183 | 0.020503 | 0.80665 | 0.152664 | 0 | 0 | 0 | 0 | 0 |
| 763 | 0.020445 | 0.021986 | 0.808472 | 0.149097 | 0 | 0 | 0 | 0 | 0 |
| 764 | 0.020421 | 0.020461 | 0.805732 | 0.153386 | 0 | 0 | 0 | 0 | 0 |
| 765 | 0.020273 | 0.020574 | 0.80675 | 0.152403 | 0 | 0 | 0 | 0 | 0 |
| 766 | 0.020675 | 0.021325 | 0.80706 | 0.15094 | 0 | 0 | 0 | 0 | 0 |
| 767 | 0.020592 | 0.01989 | 0.809064 | 0.150454 | 0 | 0 | 0 | 0 | 0 |
| 768 | 0.020091 | 0.019982 | 0.807328 | 0.152598 | 0 | 0 | 0 | 0 | 0 |
| 769 | 0.019724 | 0.020585 | 0.809494 | 0.150197 | 0 | 0 | 0 | 0 | 0 |
| 770 | 0.020139 | 0.02022 | 0.806652 | 0.152989 | 0 | 0 | 0 | 0 | 0 |
| 771 | 0.019966 | 0.021068 | 0.807501 | 0.151445 | 0 | 0 | 0 | 0 | 0 |
| 772 | 0.019611 | 0.020638 | 0.80567 | 0.154072 | 0 | 0 | 0 | 0 | 0 |
| 773 | 0.019567 | 0.021395 | 0.806159 | 0.152879 | 0 | 0 | 0 | 0 | 0 |
| 774 | 0.019828 | 0.020667 | 0.806272 | 0.153233 | 0 | 0 | 0 | 0 | 0 |
| 775 | 0.019798 | 0.020719 | 0.807447 | 0.152037 | 0 | 0 | 0 | 0 | 0 |
| 776 | 0.02011 | 0.021258 | 0.8051 | 0.153532 | 0 | 0 | 0 | 0 | 0 |
| 777 | 0.01971 | 0.020752 | 0.807678 | 0.151861 | 0 | 0 | 0 | 0 | 0 |
| 778 | 0.019131 | 0.022229 | 0.807326 | 0.151314 | 0 | 0 | 0 | 0 | 0 |
| 779 | 0.019628 | 0.020823 | 0.810449 | 0.149099 | 0 | 0 | 0 | 0 | 0 |
| 780 | 0.019378 | 0.020343 | 0.808829 | 0.15145 | 0 | 0 | 0 | 0 | 0 |
| 781 | 0.019913 | 0.021202 | 0.804131 | 0.154754 | 0 | 0 | 0 | 0 | 0 |
| 782 | 0.020385 | 0.020475 | 0.808326 | 0.150813 | 0 | 0 | 0 | 0 | 0 |
| 783 | 0.020083 | 0.021441 | 0.80571 | 0.152765 | 0 | 0 | 0 | 0 | 0 |
| 784 | 0.019911 | 0.021086 | 0.803187 | 0.155816 | 0 | 0 | 0 | 0 | 0 |
| 785 | 0.018901 | 0.020166 | 0.807357 | 0.153576 | 0 | 0 | 0 | 0 | 0 |
| 786 | 0.019677 | 0.020115 | 0.806322 | 0.153885 | 0 | 0 | 0 | 0 | 0 |
| 787 | 0.020773 | 0.020843 | 0.809684 | 0.148699 | 0 | 0 | 0 | 0 | 0 |
| 788 | 0.019303 | 0.021138 | 0.807086 | 0.152473 | 0 | 0 | 0 | 0 | 0 |
| 789 | 0.02102 | 0.019897 | 0.804655 | 0.154428 | 0 | 0 | 0 | 0 | 0 |
| 790 | 0.019491 | 0.021672 | 0.807392 | 0.151445 | 0 | 0 | 0 | 0 | 0 |
| 791 | 0.019906 | 0.020994 | 0.807984 | 0.151116 | 0 | 0 | 0 | 0 | 0 |
| 792 | 0.020844 | 0.021287 | 0.806235 | 0.151635 | 0 | 0 | 0 | 0 | 0 |
| 793 | 0.019404 | 0.020854 | 0.808384 | 0.151359 | 0 | 0 | 0 | 0 | 0 |
| 794 | 0.020071 | 0.02074 | 0.807557 | 0.151632 | 0 | 0 | 0 | 0 | 0 |
| 795 | 0.019277 | 0.021079 | 0.805937 | 0.153707 | 0 | 0 | 0 | 0 | 0 |
| 796 | 0.020098 | 0.020746 | 0.807162 | 0.151993 | 0 | 0 | 0 | 0 | 0 |
| 797 | 0.02047 | 0.020859 | 0.806784 | 0.151886 | 0 | 0 | 0 | 0 | 0 |
| 798 | 0.019471 | 0.020008 | 0.808892 | 0.151619 | 0 | 0 | 0 | 0 | 0 |
| 799 | 0.019805 | 0.020564 | 0.808557 | 0.151074 | 0 | 0 | 0 | 0 | 0 |
| 800 | 0.020178 | 0.020679 | 0.80403 | 0.155114 | 0 | 0 | 0 | 0 | 0 |
| 801 | 0.020342 | 0.020412 | 0.810024 | 0.149212 | 0 | 0 | 0 | 0 | 0 |
| 802 | 0.020213 | 0.021562 | 0.804752 | 0.153473 | 0 | 0 | 0 | 0 | 0 |
| 803 | 0.020018 | 0.019988 | 0.810054 | 0.14994 | 0 | 0 | 0 | 0 | 0 |
| 804 | 0.020051 | 0.021515 | 0.804411 | 0.154023 | 0 | 0 | 0 | 0 | 0 |
| 805 | 0.019864 | 0.020405 | 0.809716 | 0.150015 | 0 | 0 | 0 | 0 | 0 |
| 806 | 0.020255 | 0.021193 | 0.805895 | 0.152658 | 0 | 0 | 0 | 0 | 0 |
| 807 | 0.020498 | 0.021551 | 0.807048 | 0.150904 | 0 | 0 | 0 | 0 | 0 |
| 808 | 0.019901 | 0.020688 | 0.805672 | 0.153738 | 0 | 0 | 0 | 0 | 0 |
| 809 | 0.020507 | 0.020327 | 0.806776 | 0.152389 | 0 | 0 | 0 | 0 | 0 |
| 810 | 0.019472 | 0.019991 | 0.804656 | 0.155882 | 0 | 0 | 0 | 0 | 0 |
| 811 | 0.020425 | 0.021209 | 0.807443 | 0.150923 | 0 | 0 | 0 | 0 | 0 |
| 812 | 0.019817 | 0.021505 | 0.811132 | 0.147547 | 0 | 0 | 0 | 0 | 0 |
| 813 | 0.019746 | 0.020736 | 0.808384 | 0.151134 | 0 | 0 | 0 | 0 | 0 |
| 814 | 0.020668 | 0.02096 | 0.806491 | 0.15188 | 0 | 0 | 0 | 0 | 0 |
| 815 | 0.019361 | 0.020081 | 0.80642 | 0.154138 | 0 | 0 | 0 | 0 | 0 |
| 816 | 0.019795 | 0.020306 | 0.809852 | 0.150047 | 0 | 0 | 0 | 0 | 0 |
| 817 | 0.019501 | 0.02096 | 0.808662 | 0.150878 | 0 | 0 | 0 | 0 | 0 |
| 818 | 0.020151 | 0.020948 | 0.806025 | 0.152876 | 0 | 0 | 0 | 0 | 0 |
| 819 | 0.020161 | 0.020549 | 0.803635 | 0.155655 | 0 | 0 | 0 | 0 | 0 |
| 820 | 0.020395 | 0.020805 | 0.809858 | 0.148942 | 0 | 0 | 0 | 0 | 0 |
| 821 | 0.019458 | 0.020697 | 0.810254 | 0.149591 | 0 | 0 | 0 | 0 | 0 |
| 822 | 0.019882 | 0.020992 | 0.808963 | 0.150164 | 0 | 0 | 0 | 0 | 0 |
| 823 | 0.019911 | 0.020142 | 0.805345 | 0.154602 | 0 | 0 | 0 | 0 | 0 |
| 824 | 0.02085 | 0.021292 | 0.806239 | 0.151618 | 0 | 0 | 0 | 0 | 0 |
| 825 | 0.019928 | 0.021306 | 0.805799 | 0.152967 | 0 | 0 | 0 | 0 | 0 |
| 826 | 0.019791 | 0.020344 | 0.807449 | 0.152416 | 0 | 0 | 0 | 0 | 0 |
| 827 | 0.019379 | 0.020778 | 0.807306 | 0.152537 | 0 | 0 | 0 | 0 | 0 |
| 828 | 0.020383 | 0.020003 | 0.809595 | 0.150019 | 0 | 0 | 0 | 0 | 0 |
| 829 | 0.020912 | 0.021063 | 0.808647 | 0.149377 | 0 | 0 | 0 | 0 | 0 |
| 830 | 0.019782 | 0.020962 | 0.807027 | 0.152219 | 0 | 0 | 0 | 0 | 0 |
| 831 | 0.019977 | 0.021137 | 0.806302 | 0.152584 | 0 | 0 | 0 | 0 | 0 |
| 832 | 0.019181 | 0.020818 | 0.808237 | 0.151763 | 0 | 0 | 0 | 0 | 0 |
| 833 | 0.019892 | 0.020588 | 0.806148 | 0.153372 | 0 | 0 | 0 | 0 | 0 |
| 834 | 0.020227 | 0.022027 | 0.805327 | 0.152399 | 0 | 0 | 0 | 0 | 0 |
| 835 | 0.019302 | 0.020339 | 0.806679 | 0.153681 | 0 | 0 | 0 | 0 | 0 |
| 836 | 0.020484 | 0.021466 | 0.806857 | 0.151192 | 0 | 0 | 0 | 0 | 0 |
| 837 | 0.020438 | 0.020909 | 0.808249 | 0.150403 | 0 | 0 | 0 | 0 | 0 |
| 838 | 0.019767 | 0.020669 | 0.809707 | 0.149847 | 0 | 0 | 0 | 0 | 0 |
| 839 | 0.019797 | 0.020457 | 0.807115 | 0.152631 | 0 | 0 | 0 | 0 | 0 |
| 840 | 0.020152 | 0.021173 | 0.800375 | 0.1583 | 0 | 0 | 0 | 0 | 0 |
| 841 | 0.019786 | 0.021181 | 0.808727 | 0.150305 | 0 | 0 | 0 | 0 | 0 |
| 842 | 0.020804 | 0.020083 | 0.804856 | 0.154257 | 0 | 0 | 0 | 0 | 0 |
| 843 | 0.020996 | 0.020966 | 0.807184 | 0.150854 | 0 | 0 | 0 | 0 | 0 |
| 844 | 0.019709 | 0.02129 | 0.8085 | 0.150501 | 0 | 0 | 0 | 0 | 0 |
| 845 | 0.019832 | 0.020639 | 0.80437 | 0.155159 | 0 | 0 | 0 | 0 | 0 |
| 846 | 0.020367 | 0.020206 | 0.80644 | 0.152977 | 0 | 0 | 0 | 0 | 0 |
| 847 | 0.020514 | 0.020404 | 0.807759 | 0.151324 | 0 | 0 | 0 | 0 | 0 |
| 848 | 0.019481 | 0.021063 | 0.806817 | 0.152638 | 0 | 0 | 0 | 0 | 0 |
| 849 | 0.020807 | 0.021616 | 0.806135 | 0.151422 | 0 | 0 | 0 | 0 | 0 |
| 850 | 0.019537 | 0.020958 | 0.804772 | 0.154733 | 0 | 0 | 0 | 0 | 0 |
| 851 | 0.020627 | 0.021442 | 0.806035 | 0.151866 | 0 | 0 | 0 | 0 | 0 |
| 852 | 0.020599 | 0.020818 | 0.805985 | 0.152599 | 0 | 0 | 0 | 0 | 0 |
| 853 | 0.020402 | 0.021323 | 0.803141 | 0.155134 | 0 | 0 | 0 | 0 | 0 |
| 854 | 0.019694 | 0.020075 | 0.806002 | 0.154228 | 0 | 0 | 0 | 0 | 0 |
| 855 | 0.018729 | 0.021962 | 0.806037 | 0.153273 | 0 | 0 | 0 | 0 | 0 |
| 856 | 0.021002 | 0.021381 | 0.804838 | 0.152778 | 0 | 0 | 0 | 0 | 0 |
| 857 | 0.021148 | 0.020919 | 0.804863 | 0.15307 | 0 | 0 | 0 | 0 | 0 |
| 858 | 0.020433 | 0.021611 | 0.806076 | 0.15188 | 0 | 0 | 0 | 0 | 0 |
| 859 | 0.019618 | 0.020871 | 0.809008 | 0.150503 | 0 | 0 | 0 | 0 | 0 |
| 860 | 0.019679 | 0.020888 | 0.804997 | 0.154436 | 0 | 0 | 0 | 0 | 0 |
| 861 | 0.01984 | 0.02146 | 0.808232 | 0.150458 | 0 | 0 | 0 | 0 | 0 |
| 862 | 0.020374 | 0.021117 | 0.805814 | 0.152696 | 0 | 0 | 0 | 0 | 0 |
| 863 | 0.020021 | 0.020745 | 0.805448 | 0.153785 | 0 | 0 | 0 | 0 | 0 |
| 864 | 0.019992 | 0.021091 | 0.806113 | 0.152804 | 0 | 0 | 0 | 0 | 0 |
| 865 | 0.019814 | 0.020669 | 0.808545 | 0.150971 | 0 | 0 | 0 | 0 | 0 |
| 866 | 0.019634 | 0.020332 | 0.806822 | 0.153212 | 0 | 0 | 0 | 0 | 0 |
| 867 | 0.020321 | 0.020502 | 0.801738 | 0.157438 | 0 | 0 | 0 | 0 | 0 |
| 868 | 0.01946 | 0.021331 | 0.803566 | 0.155632 | 0 | 0 | 0 | 0 | 0 |
| 869 | 0.020539 | 0.020187 | 0.804937 | 0.154328 | 0 | 0 | 0 | 0 | 0 |
| 870 | 0.019639 | 0.020552 | 0.806063 | 0.153746 | 0 | 0 | 0 | 0 | 0 |
| 871 | 0.020345 | 0.020951 | 0.81089 | 0.147814 | 0 | 0 | 0 | 0 | 0 |
| 872 | 0.019855 | 0.020206 | 0.804688 | 0.155251 | 0 | 0 | 0 | 0 | 0 |
| 873 | 0.019341 | 0.020728 | 0.808072 | 0.151859 | 0 | 0 | 0 | 0 | 0 |
| 874 | 0.019854 | 0.020265 | 0.807565 | 0.152316 | 0 | 0 | 0 | 0 | 0 |
| 875 | 0.020101 | 0.020578 | 0.807488 | 0.151832 | 0 | 0 | 0 | 0 | 0 |
| 876 | 0.019844 | 0.020424 | 0.807455 | 0.152277 | 0 | 0 | 0 | 0 | 0 |
| 877 | 0.019558 | 0.020669 | 0.808996 | 0.150777 | 0 | 0 | 0 | 0 | 0 |
| 878 | 0.01973 | 0.020269 | 0.807842 | 0.15216 | 0 | 0 | 0 | 0 | 0 |
| 879 | 0.020329 | 0.021653 | 0.805784 | 0.152235 | 0 | 0 | 0 | 0 | 0 |
| 880 | 0.019585 | 0.020569 | 0.811416 | 0.14842 | 0 | 0 | 0 | 0 | 0 |
| 881 | 0.018998 | 0.020897 | 0.803814 | 0.156291 | 0 | 0 | 0 | 0 | 0 |
| 882 | 0.019476 | 0.020707 | 0.806403 | 0.153394 | 0 | 0 | 0 | 0 | 0 |
| 883 | 0.020173 | 0.021165 | 0.804115 | 0.154528 | 0 | 0 | 0 | 0 | 0 |
| 884 | 0.020108 | 0.021716 | 0.805256 | 0.15292 | 0 | 0 | 0 | 0 | 0 |
| 885 | 0.019522 | 0.02038 | 0.808528 | 0.15157 | 0 | 0 | 0 | 0 | 0 |
| 886 | 0.019862 | 0.02157 | 0.804967 | 0.153601 | 0 | 0 | 0 | 0 | 0 |
| 887 | 0.020157 | 0.020326 | 0.808258 | 0.151259 | 0 | 0 | 0 | 0 | 0 |
| 888 | 0.020022 | 0.020494 | 0.803093 | 0.156391 | 0 | 0 | 0 | 0 | 0 |
| 889 | 0.019716 | 0.021221 | 0.80538 | 0.153683 | 0 | 0 | 0 | 0 | 0 |
| 890 | 0.019951 | 0.021162 | 0.808509 | 0.150378 | 0 | 0 | 0 | 0 | 0 |
| 891 | 0.020461 | 0.020112 | 0.806311 | 0.153116 | 0 | 0 | 0 | 0 | 0 |
| 892 | 0.02035 | 0.020709 | 0.809045 | 0.149896 | 0 | 0 | 0 | 0 | 0 |
| 893 | 0.020162 | 0.020991 | 0.805227 | 0.15362 | 0 | 0 | 0 | 0 | 0 |
| 894 | 0.019544 | 0.021103 | 0.808723 | 0.15063 | 0 | 0 | 0 | 0 | 0 |
| 895 | 0.019688 | 0.020245 | 0.811733 | 0.148333 | 0 | 0 | 0 | 0 | 0 |
| 896 | 0.019857 | 0.021124 | 0.809922 | 0.149096 | 0 | 0 | 0 | 0 | 0 |
| 897 | 0.020011 | 0.020479 | 0.806277 | 0.153233 | 0 | 0 | 0 | 0 | 0 |
| 898 | 0.020009 | 0.020955 | 0.805981 | 0.153055 | 0 | 0 | 0 | 0 | 0 |
| 899 | 0.019829 | 0.021271 | 0.807742 | 0.151158 | 0 | 0 | 0 | 0 | 0 |
| 900 | 0.019592 | 0.020914 | 0.808063 | 0.151431 | 0 | 0 | 0 | 0 | 0 |
| 901 | 0.019716 | 0.020981 | 0.808213 | 0.151089 | 0 | 0 | 0 | 0 | 0 |
| 902 | 0.020966 | 0.020357 | 0.806403 | 0.152244 | 0 | 0 | 0 | 0 | 0 |
| 903 | 0.019768 | 0.021388 | 0.808275 | 0.150569 | 0 | 0 | 0 | 0 | 0 |
| 904 | 0.020256 | 0.020156 | 0.808621 | 0.150967 | 0 | 0 | 0 | 0 | 0 |
| 905 | 0.019537 | 0.020975 | 0.807284 | 0.152204 | 0 | 0 | 0 | 0 | 0 |
| 906 | 0.019877 | 0.021242 | 0.809207 | 0.149674 | 0 | 0 | 0 | 0 | 0 |
| 907 | 0.019554 | 0.019993 | 0.808857 | 0.151595 | 0 | 0 | 0 | 0 | 0 |
| 908 | 0.020128 | 0.021871 | 0.806407 | 0.151594 | 0 | 0 | 0 | 0 | 0 |
| 909 | 0.020593 | 0.021422 | 0.80469 | 0.153295 | 0 | 0 | 0 | 0 | 0 |
| 910 | 0.019739 | 0.021103 | 0.804842 | 0.154317 | 0 | 0 | 0 | 0 | 0 |
| 911 | 0.019512 | 0.021014 | 0.810273 | 0.149202 | 0 | 0 | 0 | 0 | 0 |
| 912 | 0.019948 | 0.021151 | 0.804459 | 0.154442 | 0 | 0 | 0 | 0 | 0 |
| 913 | 0.020786 | 0.020135 | 0.809125 | 0.149954 | 0 | 0 | 0 | 0 | 0 |
| 914 | 0.019271 | 0.02095 | 0.811573 | 0.148206 | 0 | 0 | 0 | 0 | 0 |
| 915 | 0.019866 | 0.020075 | 0.804783 | 0.155277 | 0 | 0 | 0 | 0 | 0 |
| 916 | 0.019957 | 0.021184 | 0.806806 | 0.152053 | 0 | 0 | 0 | 0 | 0 |
| 917 | 0.019941 | 0.020121 | 0.804785 | 0.155153 | 0 | 0 | 0 | 0 | 0 |
| 918 | 0.019993 | 0.019745 | 0.810072 | 0.150189 | 0 | 0 | 0 | 0 | 0 |
| 919 | 0.019567 | 0.021017 | 0.805918 | 0.153498 | 0 | 0 | 0 | 0 | 0 |
| 920 | 0.020223 | 0.020233 | 0.80673 | 0.152815 | 0 | 0 | 0 | 0 | 0 |
| 921 | 0.020972 | 0.020317 | 0.805816 | 0.152895 | 0 | 0 | 0 | 0 | 0 |
| 922 | 0.019631 | 0.020525 | 0.810552 | 0.149292 | 0 | 0 | 0 | 0 | 0 |
| 923 | 0.019448 | 0.019989 | 0.810169 | 0.150395 | 0 | 0 | 0 | 0 | 0 |
| 924 | 0.020029 | 0.020297 | 0.80741 | 0.152265 | 0 | 0 | 0 | 0 | 0 |
| 925 | 0.019369 | 0.020941 | 0.807993 | 0.151686 | 0 | 0 | 0 | 0 | 0 |
| 926 | 0.019491 | 0.021776 | 0.807574 | 0.151159 | 0 | 0 | 0 | 0 | 0 |
| 927 | 0.019818 | 0.020453 | 0.808692 | 0.151037 | 0 | 0 | 0 | 0 | 0 |
| 928 | 0.01899 | 0.021561 | 0.805672 | 0.153777 | 0 | 0 | 0 | 0 | 0 |
| 929 | 0.020207 | 0.020678 | 0.80525 | 0.153865 | 0 | 0 | 0 | 0 | 0 |
| 930 | 0.020205 | 0.021036 | 0.808771 | 0.149987 | 0 | 0 | 0 | 0 | 0 |
| 931 | 0.01985 | 0.020728 | 0.808451 | 0.150971 | 0 | 0 | 0 | 0 | 0 |
| 932 | 0.01953 | 0.021263 | 0.804783 | 0.154424 | 0 | 0 | 0 | 0 | 0 |
| 933 | 0.020528 | 0.020808 | 0.81087 | 0.147784 | 0 | 0 | 0 | 0 | 0 |
| 934 | 0.019218 | 0.021321 | 0.806457 | 0.153004 | 0 | 0 | 0 | 0 | 0 |
| 935 | 0.01997 | 0.022079 | 0.806019 | 0.151933 | 0 | 0 | 0 | 0 | 0 |
| 936 | 0.020814 | 0.020083 | 0.806758 | 0.152345 | 0 | 0 | 0 | 0 | 0 |
| 937 | 0.01975 | 0.020233 | 0.810971 | 0.149046 | 0 | 0 | 0 | 0 | 0 |
| 938 | 0.020151 | 0.020761 | 0.805514 | 0.153564 | 0 | 0 | 0 | 0 | 0 |
| 939 | 0.021128 | 0.020919 | 0.810369 | 0.147585 | 0 | 0 | 0 | 0 | 0 |
| 940 | 0.019853 | 0.021277 | 0.804189 | 0.154681 | 0 | 0 | 0 | 0 | 0 |
| 941 | 0.019608 | 0.021433 | 0.805331 | 0.153628 | 0 | 0 | 0 | 0 | 0 |
| 942 | 0.019888 | 0.021623 | 0.806883 | 0.151607 | 0 | 0 | 0 | 0 | 0 |
| 943 | 0.019905 | 0.0209 | 0.810059 | 0.149136 | 0 | 0 | 0 | 0 | 0 |
| 944 | 0.019767 | 0.021753 | 0.804016 | 0.154465 | 0 | 0 | 0 | 0 | 0 |
| 945 | 0.020085 | 0.021935 | 0.801952 | 0.156027 | 0 | 0 | 0 | 0 | 0 |
| 946 | 0.019485 | 0.021678 | 0.808494 | 0.150343 | 0 | 0 | 0 | 0 | 0 |
| 947 | 0.020174 | 0.021001 | 0.80846 | 0.150365 | 0 | 0 | 0 | 0 | 0 |
| 948 | 0.019886 | 0.021527 | 0.804564 | 0.154023 | 0 | 0 | 0 | 0 | 0 |
| 949 | 0.019514 | 0.020618 | 0.810747 | 0.149122 | 0 | 0 | 0 | 0 | 0 |
| 950 | 0.020131 | 0.021732 | 0.806878 | 0.15126 | 0 | 0 | 0 | 0 | 0 |
| 951 | 0.018908 | 0.021098 | 0.809272 | 0.150723 | 0 | 0 | 0 | 0 | 0 |
| 952 | 0.01988 | 0.021415 | 0.808109 | 0.150596 | 0 | 0 | 0 | 0 | 0 |
| 953 | 0.019837 | 0.021397 | 0.808961 | 0.149806 | 0 | 0 | 0 | 0 | 0 |
| 954 | 0.019898 | 0.020948 | 0.805329 | 0.153825 | 0 | 0 | 0 | 0 | 0 |
| 955 | 0.020616 | 0.021455 | 0.804746 | 0.153182 | 0 | 0 | 0 | 0 | 0 |
| 956 | 0.02042 | 0.021032 | 0.805637 | 0.152911 | 0 | 0 | 0 | 0 | 0 |
| 957 | 0.020444 | 0.021631 | 0.80458 | 0.153346 | 0 | 0 | 0 | 0 | 0 |
| 958 | 0.019249 | 0.020962 | 0.807646 | 0.152133 | 0 | 0 | 0 | 0 | 0 |
| 959 | 0.019308 | 0.020585 | 0.805485 | 0.154622 | 0 | 0 | 0 | 0 | 0 |
| 960 | 0.019693 | 0.021066 | 0.807504 | 0.151738 | 0 | 0 | 0 | 0 | 0 |
| 961 | 0.020092 | 0.019942 | 0.808719 | 0.151227 | 0 | 0 | 0 | 0 | 0 |
| 962 | 0.019697 | 0.020829 | 0.808028 | 0.151446 | 0 | 0 | 0 | 0 | 0 |
| 963 | 0.020517 | 0.020168 | 0.807703 | 0.151612 | 0 | 0 | 0 | 0 | 0 |
| 964 | 0.020297 | 0.020416 | 0.807243 | 0.152044 | 0 | 0 | 0 | 0 | 0 |
| 965 | 0.020143 | 0.020482 | 0.805945 | 0.153429 | 0 | 0 | 0 | 0 | 0 |
| 966 | 0.019714 | 0.02084 | 0.80669 | 0.152757 | 0 | 0 | 0 | 0 | 0 |
| 967 | 0.019993 | 0.020932 | 0.806498 | 0.152577 | 0 | 0 | 0 | 0 | 0 |
| 968 | 0.020167 | 0.020779 | 0.806307 | 0.152747 | 0 | 0 | 0 | 0 | 0 |
| 969 | 0.019231 | 0.021272 | 0.806639 | 0.152859 | 0 | 0 | 0 | 0 | 0 |
| 970 | 0.019584 | 0.02076 | 0.808226 | 0.15143 | 0 | 0 | 0 | 0 | 0 |
| 971 | 0.02005 | 0.020629 | 0.806602 | 0.152719 | 0 | 0 | 0 | 0 | 0 |
| 972 | 0.020286 | 0.021127 | 0.806039 | 0.152548 | 0 | 0 | 0 | 0 | 0 |
| 973 | 0.020748 | 0.020957 | 0.807534 | 0.150752 | 0 | 0 | 0 | 0 | 0 |
| 974 | 0.019778 | 0.019698 | 0.808478 | 0.152047 | 0 | 0 | 0 | 0 | 0 |
| 975 | 0.019724 | 0.020368 | 0.808799 | 0.151109 | 0 | 0 | 0 | 0 | 0 |
| 976 | 0.020389 | 0.019852 | 0.806252 | 0.153507 | 0 | 0 | 0 | 0 | 0 |
| 977 | 0.019474 | 0.020532 | 0.810952 | 0.149041 | 0 | 0 | 0 | 0 | 0 |
| 978 | 0.020016 | 0.020509 | 0.808141 | 0.151333 | 0 | 0 | 0 | 0 | 0 |
| 979 | 0.019643 | 0.020182 | 0.808145 | 0.15203 | 0 | 0 | 0 | 0 | 0 |
| 980 | 0.020029 | 0.021038 | 0.804538 | 0.154394 | 0 | 0 | 0 | 0 | 0 |
| 981 | 0.020418 | 0.020418 | 0.80927 | 0.149875 | 0 | 0 | 0 | 0 | 0 |
| 982 | 0.019865 | 0.020672 | 0.808394 | 0.151068 | 0 | 0 | 0 | 0 | 0 |
| 983 | 0.019641 | 0.021206 | 0.805469 | 0.153684 | 0 | 0 | 0 | 0 | 0 |
| 984 | 0.019272 | 0.020543 | 0.808497 | 0.151687 | 0 | 0 | 0 | 0 | 0 |
| 985 | 0.02039 | 0.02043 | 0.805948 | 0.153232 | 0 | 0 | 0 | 0 | 0 |
| 986 | 0.020128 | 0.020835 | 0.80789 | 0.151147 | 0 | 0 | 0 | 0 | 0 |
| 987 | 0.020035 | 0.020928 | 0.80503 | 0.154007 | 0 | 0 | 0 | 0 | 0 |
| 988 | 0.019512 | 0.021148 | 0.805437 | 0.153902 | 0 | 0 | 0 | 0 | 0 |
| 989 | 0.020538 | 0.021667 | 0.804495 | 0.1533 | 0 | 0 | 0 | 0 | 0 |
| 990 | 0.0196 | 0.020948 | 0.808796 | 0.150646 | 0 | 0 | 0 | 0 | 0 |
| 991 | 0.019752 | 0.021425 | 0.807136 | 0.151687 | 0 | 0 | 0 | 0 | 0 |
| 992 | 0.020289 | 0.021787 | 0.806216 | 0.151708 | 0 | 0 | 0 | 0 | 0 |
| 993 | 0.019738 | 0.021389 | 0.807744 | 0.151129 | 0 | 0 | 0 | 0 | 0 |
| 994 | 0.020424 | 0.020874 | 0.809891 | 0.148811 | 0 | 0 | 0 | 0 | 0 |
| 995 | 0.019029 | 0.020111 | 0.807391 | 0.15347 | 0 | 0 | 0 | 0 | 0 |
| 996 | 0.019497 | 0.020242 | 0.80944 | 0.150821 | 0 | 0 | 0 | 0 | 0 |
| 997 | 0.019453 | 0.021217 | 0.806144 | 0.153187 | 0 | 0 | 0 | 0 | 0 |
| 998 | 0.019167 | 0.020141 | 0.808257 | 0.152434 | 0 | 0 | 0 | 0 | 0 |
| 999 | 0.019561 | 0.021052 | 0.808643 | 0.150724 | 0 | 0 | 0 | 0 | 0 |
| 1000 | 0.020089 | 0.02111 | 0.805251 | 0.153549 | 0 | 0 | 0 | 0 | 0 |

| Year 3 | Race |  |  |  |  |  |  |  |  |
| --- | --- | --- | --- | --- | --- | --- | --- | --- | --- |
| Trial | 003.0 | 005.0 | 007.0 | 037.1 | 043.0 | 303.0 | 003.2 | 403.0 | 003.4 |
| 1 | 0.002829 | 0.008537 | 0.844439 | 0.144194 | 0 | 0 | 0 | 0 | 0 |
| 2 | 0.003 | 0.0085 | 0.842138 | 0.146363 | 0 | 0 | 0 | 0 | 0 |
| 3 | 0.003086 | 0.007975 | 0.846462 | 0.142476 | 0 | 0 | 0 | 0 | 0 |
| 4 | 0.002838 | 0.008114 | 0.846915 | 0.142133 | 0 | 0 | 0 | 0 | 0 |
| 5 | 0.002973 | 0.008379 | 0.839992 | 0.148657 | 0 | 0 | 0 | 0 | 0 |
| 6 | 0.002962 | 0.008386 | 0.842611 | 0.146041 | 0 | 0 | 0 | 0 | 0 |
| 7 | 0.002744 | 0.008193 | 0.84086 | 0.148203 | 0 | 0 | 0 | 0 | 0 |
| 8 | 0.00273 | 0.007911 | 0.845315 | 0.144043 | 0 | 0 | 0 | 0 | 0 |
| 9 | 0.002829 | 0.008248 | 0.84432 | 0.144602 | 0 | 0 | 0 | 0 | 0 |
| 10 | 0.002854 | 0.007924 | 0.843782 | 0.14544 | 0 | 0 | 0 | 0 | 0 |
| 11 | 0.002898 | 0.008526 | 0.846351 | 0.142205 | 0 | 0 | 0 | 0 | 0 |
| 12 | 0.002904 | 0.007605 | 0.841298 | 0.148193 | 0 | 0 | 0 | 0 | 0 |
| 13 | 0.003246 | 0.008116 | 0.842439 | 0.146199 | 0 | 0 | 0 | 0 | 0 |
| 14 | 0.003083 | 0.008669 | 0.837456 | 0.150792 | 0 | 0 | 0 | 0 | 0 |
| 15 | 0.002769 | 0.008419 | 0.843448 | 0.145363 | 0 | 0 | 0 | 0 | 0 |
| 16 | 0.002985 | 0.008765 | 0.841741 | 0.146508 | 0 | 0 | 0 | 0 | 0 |
| 17 | 0.002723 | 0.008318 | 0.843843 | 0.145106 | 0 | 0 | 0 | 0 | 0 |
| 18 | 0.00325 | 0.007901 | 0.845391 | 0.143457 | 0 | 0 | 0 | 0 | 0 |
| 19 | 0.003052 | 0.008308 | 0.841129 | 0.147511 | 0 | 0 | 0 | 0 | 0 |
| 20 | 0.00266 | 0.007879 | 0.841924 | 0.147537 | 0 | 0 | 0 | 0 | 0 |
| 21 | 0.002996 | 0.00813 | 0.844381 | 0.144494 | 0 | 0 | 0 | 0 | 0 |
| 22 | 0.002947 | 0.008329 | 0.840197 | 0.148527 | 0 | 0 | 0 | 0 | 0 |
| 23 | 0.003145 | 0.008064 | 0.842175 | 0.146615 | 0 | 0 | 0 | 0 | 0 |
| 24 | 0.002623 | 0.008711 | 0.84221 | 0.146456 | 0 | 0 | 0 | 0 | 0 |
| 25 | 0.003299 | 0.007811 | 0.842026 | 0.146864 | 0 | 0 | 0 | 0 | 0 |
| 26 | 0.00298 | 0.008429 | 0.847234 | 0.141357 | 0 | 0 | 0 | 0 | 0 |
| 27 | 0.002663 | 0.00842 | 0.843028 | 0.145888 | 0 | 0 | 0 | 0 | 0 |
| 28 | 0.002745 | 0.008246 | 0.843343 | 0.145645 | 0 | 0 | 0 | 0 | 0 |
| 29 | 0.003014 | 0.00821 | 0.842807 | 0.14597 | 0 | 0 | 0 | 0 | 0 |
| 30 | 0.002904 | 0.008333 | 0.844179 | 0.144584 | 0 | 0 | 0 | 0 | 0 |
| 31 | 0.00258 | 0.007918 | 0.842819 | 0.146684 | 0 | 0 | 0 | 0 | 0 |
| 32 | 0.00313 | 0.00825 | 0.843376 | 0.145244 | 0 | 0 | 0 | 0 | 0 |
| 33 | 0.002897 | 0.008175 | 0.841964 | 0.146964 | 0 | 0 | 0 | 0 | 0 |
| 34 | 0.002738 | 0.008463 | 0.842395 | 0.146405 | 0 | 0 | 0 | 0 | 0 |
| 35 | 0.003169 | 0.007584 | 0.840203 | 0.149044 | 0 | 0 | 0 | 0 | 0 |
| 36 | 0.003067 | 0.008006 | 0.841985 | 0.146941 | 0 | 0 | 0 | 0 | 0 |
| 37 | 0.002829 | 0.009043 | 0.844142 | 0.143986 | 0 | 0 | 0 | 0 | 0 |
| 38 | 0.002956 | 0.008035 | 0.844165 | 0.144844 | 0 | 0 | 0 | 0 | 0 |
| 39 | 0.002934 | 0.00795 | 0.843769 | 0.145337 | 0 | 0 | 0 | 0 | 0 |
| 40 | 0.002599 | 0.007998 | 0.841287 | 0.148116 | 0 | 0 | 0 | 0 | 0 |
| 41 | 0.002773 | 0.008238 | 0.843758 | 0.145231 | 0 | 0 | 0 | 0 | 0 |
| 42 | 0.002701 | 0.009001 | 0.845697 | 0.142601 | 0 | 0 | 0 | 0 | 0 |
| 43 | 0.003068 | 0.007916 | 0.845572 | 0.143444 | 0 | 0 | 0 | 0 | 0 |
| 44 | 0.002913 | 0.008638 | 0.8428 | 0.145648 | 0 | 0 | 0 | 0 | 0 |
| 45 | 0.002961 | 0.008923 | 0.843341 | 0.144775 | 0 | 0 | 0 | 0 | 0 |
| 46 | 0.002728 | 0.008607 | 0.839583 | 0.149081 | 0 | 0 | 0 | 0 | 0 |
| 47 | 0.002888 | 0.007972 | 0.84179 | 0.147351 | 0 | 0 | 0 | 0 | 0 |
| 48 | 0.002969 | 0.008408 | 0.840088 | 0.148534 | 0 | 0 | 0 | 0 | 0 |
| 49 | 0.002919 | 0.008407 | 0.840212 | 0.148462 | 0 | 0 | 0 | 0 | 0 |
| 50 | 0.00296 | 0.008175 | 0.84346 | 0.145404 | 0 | 0 | 0 | 0 | 0 |
| 51 | 0.002877 | 0.008371 | 0.842722 | 0.146031 | 0 | 0 | 0 | 0 | 0 |
| 52 | 0.002877 | 0.008811 | 0.843872 | 0.14444 | 0 | 0 | 0 | 0 | 0 |
| 53 | 0.00297 | 0.007889 | 0.84671 | 0.142401 | 0 | 0 | 0 | 0 | 0 |
| 54 | 0.003139 | 0.007601 | 0.845424 | 0.143836 | 0 | 0 | 0 | 0 | 0 |
| 55 | 0.003031 | 0.008581 | 0.844589 | 0.143799 | 0 | 0 | 0 | 0 | 0 |
| 56 | 0.002631 | 0.007964 | 0.841394 | 0.148011 | 0 | 0 | 0 | 0 | 0 |
| 57 | 0.003058 | 0.008724 | 0.84325 | 0.144969 | 0 | 0 | 0 | 0 | 0 |
| 58 | 0.002962 | 0.008578 | 0.842187 | 0.146273 | 0 | 0 | 0 | 0 | 0 |
| 59 | 0.002728 | 0.008463 | 0.843305 | 0.145504 | 0 | 0 | 0 | 0 | 0 |
| 60 | 0.002932 | 0.007925 | 0.846399 | 0.142744 | 0 | 0 | 0 | 0 | 0 |
| 61 | 0.002891 | 0.00808 | 0.843563 | 0.145467 | 0 | 0 | 0 | 0 | 0 |
| 62 | 0.003104 | 0.008125 | 0.847214 | 0.141557 | 0 | 0 | 0 | 0 | 0 |
| 63 | 0.002933 | 0.008317 | 0.845658 | 0.143092 | 0 | 0 | 0 | 0 | 0 |
| 64 | 0.002915 | 0.009115 | 0.843334 | 0.144636 | 0 | 0 | 0 | 0 | 0 |
| 65 | 0.003024 | 0.007938 | 0.842847 | 0.14619 | 0 | 0 | 0 | 0 | 0 |
| 66 | 0.002725 | 0.007965 | 0.848946 | 0.140363 | 0 | 0 | 0 | 0 | 0 |
| 67 | 0.002964 | 0.008021 | 0.842731 | 0.146265 | 0 | 0 | 0 | 0 | 0 |
| 68 | 0.00307 | 0.008146 | 0.842284 | 0.1465 | 0 | 0 | 0 | 0 | 0 |
| 69 | 0.003055 | 0.008 | 0.84008 | 0.148866 | 0 | 0 | 0 | 0 | 0 |
| 70 | 0.002738 | 0.008223 | 0.844915 | 0.144125 | 0 | 0 | 0 | 0 | 0 |
| 71 | 0.002711 | 0.008752 | 0.842999 | 0.145499 | 0 | 0 | 0 | 0 | 0 |
| 72 | 0.003124 | 0.008475 | 0.846478 | 0.141923 | 0 | 0 | 0 | 0 | 0 |
| 73 | 0.002693 | 0.008189 | 0.842137 | 0.14698 | 0 | 0 | 0 | 0 | 0 |
| 74 | 0.002535 | 0.008215 | 0.845189 | 0.144061 | 0 | 0 | 0 | 0 | 0 |
| 75 | 0.003152 | 0.008769 | 0.842803 | 0.145277 | 0 | 0 | 0 | 0 | 0 |
| 76 | 0.002991 | 0.008942 | 0.84342 | 0.144648 | 0 | 0 | 0 | 0 | 0 |
| 77 | 0.002832 | 0.008695 | 0.843319 | 0.145155 | 0 | 0 | 0 | 0 | 0 |
| 78 | 0.002857 | 0.00847 | 0.846941 | 0.141732 | 0 | 0 | 0 | 0 | 0 |
| 79 | 0.00308 | 0.008038 | 0.846187 | 0.142694 | 0 | 0 | 0 | 0 | 0 |
| 80 | 0.002663 | 0.007919 | 0.842479 | 0.146939 | 0 | 0 | 0 | 0 | 0 |
| 81 | 0.002684 | 0.008154 | 0.840313 | 0.148849 | 0 | 0 | 0 | 0 | 0 |
| 82 | 0.002745 | 0.008155 | 0.843563 | 0.145537 | 0 | 0 | 0 | 0 | 0 |
| 83 | 0.003066 | 0.00845 | 0.846197 | 0.142286 | 0 | 0 | 0 | 0 | 0 |
| 84 | 0.002685 | 0.008205 | 0.846243 | 0.142867 | 0 | 0 | 0 | 0 | 0 |
| 85 | 0.0027 | 0.008493 | 0.841097 | 0.14771 | 0 | 0 | 0 | 0 | 0 |
| 86 | 0.002563 | 0.008308 | 0.849194 | 0.139935 | 0 | 0 | 0 | 0 | 0 |
| 87 | 0.002984 | 0.00813 | 0.841135 | 0.147752 | 0 | 0 | 0 | 0 | 0 |
| 88 | 0.002396 | 0.008555 | 0.844663 | 0.144366 | 0 | 0 | 0 | 0 | 0 |
| 89 | 0.002948 | 0.008277 | 0.842776 | 0.145999 | 0 | 0 | 0 | 0 | 0 |
| 90 | 0.002838 | 0.008123 | 0.843372 | 0.145638 | 0 | 0 | 0 | 0 | 0 |
| 91 | 0.002532 | 0.008207 | 0.841414 | 0.147847 | 0 | 0 | 0 | 0 | 0 |
| 92 | 0.002823 | 0.00821 | 0.839495 | 0.149472 | 0 | 0 | 0 | 0 | 0 |
| 93 | 0.002933 | 0.008396 | 0.84225 | 0.146421 | 0 | 0 | 0 | 0 | 0 |
| 94 | 0.002816 | 0.008166 | 0.843093 | 0.145925 | 0 | 0 | 0 | 0 | 0 |
| 95 | 0.00277 | 0.008289 | 0.840224 | 0.148718 | 0 | 0 | 0 | 0 | 0 |
| 96 | 0.003149 | 0.008574 | 0.843302 | 0.144975 | 0 | 0 | 0 | 0 | 0 |
| 97 | 0.002579 | 0.008667 | 0.840309 | 0.148445 | 0 | 0 | 0 | 0 | 0 |
| 98 | 0.002881 | 0.00818 | 0.841369 | 0.14757 | 0 | 0 | 0 | 0 | 0 |
| 99 | 0.002713 | 0.0085 | 0.841989 | 0.146798 | 0 | 0 | 0 | 0 | 0 |
| 100 | 0.003085 | 0.007858 | 0.845135 | 0.143922 | 0 | 0 | 0 | 0 | 0 |
| 101 | 0.003124 | 0.008355 | 0.845371 | 0.143149 | 0 | 0 | 0 | 0 | 0 |
| 102 | 0.003043 | 0.007743 | 0.844596 | 0.144618 | 0 | 0 | 0 | 0 | 0 |
| 103 | 0.00312 | 0.008279 | 0.840729 | 0.147873 | 0 | 0 | 0 | 0 | 0 |
| 104 | 0.002934 | 0.00809 | 0.845631 | 0.143325 | 0 | 0 | 0 | 0 | 0 |
| 105 | 0.002896 | 0.008627 | 0.842234 | 0.146242 | 0 | 0 | 0 | 0 | 0 |
| 106 | 0.002901 | 0.008209 | 0.842476 | 0.146414 | 0 | 0 | 0 | 0 | 0 |
| 107 | 0.002696 | 0.007719 | 0.842625 | 0.14692 | 0 | 0 | 0 | 0 | 0 |
| 108 | 0.003153 | 0.008685 | 0.839098 | 0.149045 | 0 | 0 | 0 | 0 | 0 |
| 109 | 0.002809 | 0.008089 | 0.845315 | 0.143786 | 0 | 0 | 0 | 0 | 0 |
| 110 | 0.003053 | 0.007553 | 0.847912 | 0.141482 | 0 | 0 | 0 | 0 | 0 |
| 111 | 0.002858 | 0.008613 | 0.844871 | 0.143658 | 0 | 0 | 0 | 0 | 0 |
| 112 | 0.002995 | 0.008051 | 0.841198 | 0.147756 | 0 | 0 | 0 | 0 | 0 |
| 113 | 0.002865 | 0.008254 | 0.843807 | 0.145074 | 0 | 0 | 0 | 0 | 0 |
| 114 | 0.002965 | 0.008076 | 0.845972 | 0.142987 | 0 | 0 | 0 | 0 | 0 |
| 115 | 0.003161 | 0.008017 | 0.848233 | 0.14059 | 0 | 0 | 0 | 0 | 0 |
| 116 | 0.002943 | 0.00854 | 0.845576 | 0.142941 | 0 | 0 | 0 | 0 | 0 |
| 117 | 0.002896 | 0.00845 | 0.844569 | 0.144084 | 0 | 0 | 0 | 0 | 0 |
| 118 | 0.00289 | 0.008321 | 0.844684 | 0.144104 | 0 | 0 | 0 | 0 | 0 |
| 119 | 0.002949 | 0.008376 | 0.841758 | 0.146918 | 0 | 0 | 0 | 0 | 0 |
| 120 | 0.003155 | 0.008338 | 0.845026 | 0.143481 | 0 | 0 | 0 | 0 | 0 |
| 121 | 0.002602 | 0.008278 | 0.845043 | 0.144067 | 0 | 0 | 0 | 0 | 0 |
| 122 | 0.002824 | 0.007654 | 0.845405 | 0.144117 | 0 | 0 | 0 | 0 | 0 |
| 123 | 0.002814 | 0.008052 | 0.84538 | 0.143753 | 0 | 0 | 0 | 0 | 0 |
| 124 | 0.002825 | 0.008376 | 0.845765 | 0.143033 | 0 | 0 | 0 | 0 | 0 |
| 125 | 0.002892 | 0.008367 | 0.844989 | 0.143752 | 0 | 0 | 0 | 0 | 0 |
| 126 | 0.002914 | 0.008232 | 0.842125 | 0.146729 | 0 | 0 | 0 | 0 | 0 |
| 127 | 0.002833 | 0.008498 | 0.845518 | 0.143151 | 0 | 0 | 0 | 0 | 0 |
| 128 | 0.002686 | 0.008227 | 0.845744 | 0.143343 | 0 | 0 | 0 | 0 | 0 |
| 129 | 0.002865 | 0.008073 | 0.842553 | 0.146509 | 0 | 0 | 0 | 0 | 0 |
| 130 | 0.002834 | 0.008122 | 0.844238 | 0.144806 | 0 | 0 | 0 | 0 | 0 |
| 131 | 0.002877 | 0.008244 | 0.84687 | 0.142008 | 0 | 0 | 0 | 0 | 0 |
| 132 | 0.003334 | 0.008175 | 0.842128 | 0.146363 | 0 | 0 | 0 | 0 | 0 |
| 133 | 0.00289 | 0.007799 | 0.842073 | 0.147238 | 0 | 0 | 0 | 0 | 0 |
| 134 | 0.002878 | 0.007953 | 0.845568 | 0.143601 | 0 | 0 | 0 | 0 | 0 |
| 135 | 0.002927 | 0.008203 | 0.839501 | 0.149369 | 0 | 0 | 0 | 0 | 0 |
| 136 | 0.003243 | 0.008413 | 0.844762 | 0.143581 | 0 | 0 | 0 | 0 | 0 |
| 137 | 0.002946 | 0.007904 | 0.8415 | 0.147649 | 0 | 0 | 0 | 0 | 0 |
| 138 | 0.002901 | 0.008613 | 0.840761 | 0.147725 | 0 | 0 | 0 | 0 | 0 |
| 139 | 0.00321 | 0.008327 | 0.844772 | 0.143691 | 0 | 0 | 0 | 0 | 0 |
| 140 | 0.002636 | 0.008434 | 0.842542 | 0.146388 | 0 | 0 | 0 | 0 | 0 |
| 141 | 0.002865 | 0.008236 | 0.840855 | 0.148045 | 0 | 0 | 0 | 0 | 0 |
| 142 | 0.002756 | 0.008359 | 0.841756 | 0.147129 | 0 | 0 | 0 | 0 | 0 |
| 143 | 0.003108 | 0.007992 | 0.843609 | 0.145291 | 0 | 0 | 0 | 0 | 0 |
| 144 | 0.002806 | 0.008618 | 0.843117 | 0.145458 | 0 | 0 | 0 | 0 | 0 |
| 145 | 0.003092 | 0.008235 | 0.844221 | 0.144452 | 0 | 0 | 0 | 0 | 0 |
| 146 | 0.002735 | 0.008605 | 0.847952 | 0.140708 | 0 | 0 | 0 | 0 | 0 |
| 147 | 0.003093 | 0.008332 | 0.844081 | 0.144494 | 0 | 0 | 0 | 0 | 0 |
| 148 | 0.003092 | 0.008477 | 0.841776 | 0.146655 | 0 | 0 | 0 | 0 | 0 |
| 149 | 0.003238 | 0.008364 | 0.843069 | 0.14533 | 0 | 0 | 0 | 0 | 0 |
| 150 | 0.003279 | 0.007761 | 0.843033 | 0.145927 | 0 | 0 | 0 | 0 | 0 |
| 151 | 0.002657 | 0.008212 | 0.841913 | 0.147217 | 0 | 0 | 0 | 0 | 0 |
| 152 | 0.002598 | 0.008839 | 0.840585 | 0.147978 | 0 | 0 | 0 | 0 | 0 |
| 153 | 0.003147 | 0.007283 | 0.846882 | 0.142689 | 0 | 0 | 0 | 0 | 0 |
| 154 | 0.002667 | 0.007549 | 0.845006 | 0.144779 | 0 | 0 | 0 | 0 | 0 |
| 155 | 0.003299 | 0.007609 | 0.841848 | 0.147244 | 0 | 0 | 0 | 0 | 0 |
| 156 | 0.002661 | 0.008383 | 0.841748 | 0.147207 | 0 | 0 | 0 | 0 | 0 |
| 157 | 0.003076 | 0.008329 | 0.84473 | 0.143866 | 0 | 0 | 0 | 0 | 0 |
| 158 | 0.003144 | 0.008036 | 0.845842 | 0.142978 | 0 | 0 | 0 | 0 | 0 |
| 159 | 0.002797 | 0.00816 | 0.840155 | 0.148888 | 0 | 0 | 0 | 0 | 0 |
| 160 | 0.002825 | 0.007782 | 0.850665 | 0.138728 | 0 | 0 | 0 | 0 | 0 |
| 161 | 0.002845 | 0.008028 | 0.840148 | 0.148978 | 0 | 0 | 0 | 0 | 0 |
| 162 | 0.003063 | 0.008352 | 0.840824 | 0.147761 | 0 | 0 | 0 | 0 | 0 |
| 163 | 0.003243 | 0.007833 | 0.841687 | 0.147238 | 0 | 0 | 0 | 0 | 0 |
| 164 | 0.002654 | 0.00883 | 0.842735 | 0.145782 | 0 | 0 | 0 | 0 | 0 |
| 165 | 0.002907 | 0.0087 | 0.842825 | 0.145569 | 0 | 0 | 0 | 0 | 0 |
| 166 | 0.002837 | 0.008066 | 0.843159 | 0.145938 | 0 | 0 | 0 | 0 | 0 |
| 167 | 0.003186 | 0.008259 | 0.84465 | 0.143906 | 0 | 0 | 0 | 0 | 0 |
| 168 | 0.002845 | 0.008246 | 0.843965 | 0.144944 | 0 | 0 | 0 | 0 | 0 |
| 169 | 0.00293 | 0.008476 | 0.841346 | 0.147249 | 0 | 0 | 0 | 0 | 0 |
| 170 | 0.002836 | 0.009227 | 0.841708 | 0.146229 | 0 | 0 | 0 | 0 | 0 |
| 171 | 0.002876 | 0.009019 | 0.844663 | 0.143442 | 0 | 0 | 0 | 0 | 0 |
| 172 | 0.00305 | 0.008395 | 0.841417 | 0.147139 | 0 | 0 | 0 | 0 | 0 |
| 173 | 0.002906 | 0.009178 | 0.842162 | 0.145755 | 0 | 0 | 0 | 0 | 0 |
| 174 | 0.002989 | 0.008967 | 0.840909 | 0.147134 | 0 | 0 | 0 | 0 | 0 |
| 175 | 0.00259 | 0.008391 | 0.840978 | 0.148041 | 0 | 0 | 0 | 0 | 0 |
| 176 | 0.003216 | 0.00794 | 0.847475 | 0.141329 | 0 | 0 | 0 | 0 | 0 |
| 177 | 0.002817 | 0.008252 | 0.842258 | 0.146673 | 0 | 0 | 0 | 0 | 0 |
| 178 | 0.002955 | 0.008744 | 0.842889 | 0.145412 | 0 | 0 | 0 | 0 | 0 |
| 179 | 0.003335 | 0.008458 | 0.840355 | 0.147853 | 0 | 0 | 0 | 0 | 0 |
| 180 | 0.002894 | 0.008544 | 0.84382 | 0.144742 | 0 | 0 | 0 | 0 | 0 |
| 181 | 0.002902 | 0.008366 | 0.84522 | 0.143512 | 0 | 0 | 0 | 0 | 0 |
| 182 | 0.002814 | 0.008403 | 0.844846 | 0.143937 | 0 | 0 | 0 | 0 | 0 |
| 183 | 0.002909 | 0.008417 | 0.843822 | 0.144852 | 0 | 0 | 0 | 0 | 0 |
| 184 | 0.002766 | 0.00821 | 0.846575 | 0.142449 | 0 | 0 | 0 | 0 | 0 |
| 185 | 0.002699 | 0.008057 | 0.842828 | 0.146416 | 0 | 0 | 0 | 0 | 0 |
| 186 | 0.003072 | 0.007956 | 0.845188 | 0.143784 | 0 | 0 | 0 | 0 | 0 |
| 187 | 0.002952 | 0.008127 | 0.850309 | 0.138612 | 0 | 0 | 0 | 0 | 0 |
| 188 | 0.002934 | 0.007804 | 0.843574 | 0.145688 | 0 | 0 | 0 | 0 | 0 |
| 189 | 0.002828 | 0.008565 | 0.841842 | 0.146765 | 0 | 0 | 0 | 0 | 0 |
| 190 | 0.002832 | 0.008006 | 0.842577 | 0.146586 | 0 | 0 | 0 | 0 | 0 |
| 191 | 0.002894 | 0.008101 | 0.842985 | 0.14602 | 0 | 0 | 0 | 0 | 0 |
| 192 | 0.003092 | 0.007769 | 0.843935 | 0.145205 | 0 | 0 | 0 | 0 | 0 |
| 193 | 0.003235 | 0.009183 | 0.840147 | 0.147435 | 0 | 0 | 0 | 0 | 0 |
| 194 | 0.002786 | 0.008098 | 0.842619 | 0.146497 | 0 | 0 | 0 | 0 | 0 |
| 195 | 0.002621 | 0.008383 | 0.839032 | 0.149964 | 0 | 0 | 0 | 0 | 0 |
| 196 | 0.002862 | 0.008316 | 0.842018 | 0.146804 | 0 | 0 | 0 | 0 | 0 |
| 197 | 0.002853 | 0.007798 | 0.846529 | 0.14282 | 0 | 0 | 0 | 0 | 0 |
| 198 | 0.002911 | 0.008185 | 0.844109 | 0.144776 | 0 | 0 | 0 | 0 | 0 |
| 199 | 0.002741 | 0.008023 | 0.847816 | 0.14142 | 0 | 0 | 0 | 0 | 0 |
| 200 | 0.003244 | 0.009042 | 0.839261 | 0.148453 | 0 | 0 | 0 | 0 | 0 |
| 201 | 0.003124 | 0.009283 | 0.841997 | 0.145597 | 0 | 0 | 0 | 0 | 0 |
| 202 | 0.003161 | 0.008172 | 0.844433 | 0.144233 | 0 | 0 | 0 | 0 | 0 |
| 203 | 0.003238 | 0.007939 | 0.846383 | 0.14244 | 0 | 0 | 0 | 0 | 0 |
| 204 | 0.002913 | 0.008729 | 0.844405 | 0.143953 | 0 | 0 | 0 | 0 | 0 |
| 205 | 0.002971 | 0.007962 | 0.8379 | 0.151167 | 0 | 0 | 0 | 0 | 0 |
| 206 | 0.003235 | 0.007978 | 0.842051 | 0.146736 | 0 | 0 | 0 | 0 | 0 |
| 207 | 0.002952 | 0.007978 | 0.843967 | 0.145104 | 0 | 0 | 0 | 0 | 0 |
| 208 | 0.002683 | 0.007751 | 0.847752 | 0.141814 | 0 | 0 | 0 | 0 | 0 |
| 209 | 0.002886 | 0.008437 | 0.842378 | 0.146299 | 0 | 0 | 0 | 0 | 0 |
| 210 | 0.002987 | 0.007956 | 0.843465 | 0.145592 | 0 | 0 | 0 | 0 | 0 |
| 211 | 0.002907 | 0.008952 | 0.843896 | 0.144245 | 0 | 0 | 0 | 0 | 0 |
| 212 | 0.00308 | 0.008599 | 0.84397 | 0.144351 | 0 | 0 | 0 | 0 | 0 |
| 213 | 0.002621 | 0.007665 | 0.846611 | 0.143103 | 0 | 0 | 0 | 0 | 0 |
| 214 | 0.002822 | 0.008295 | 0.843909 | 0.144974 | 0 | 0 | 0 | 0 | 0 |
| 215 | 0.003042 | 0.008339 | 0.844228 | 0.14438 | 0 | 0 | 0 | 0 | 0 |
| 216 | 0.002848 | 0.007833 | 0.846378 | 0.142941 | 0 | 0 | 0 | 0 | 0 |
| 217 | 0.003053 | 0.007447 | 0.845002 | 0.144499 | 0 | 0 | 0 | 0 | 0 |
| 218 | 0.002924 | 0.007341 | 0.844718 | 0.145017 | 0 | 0 | 0 | 0 | 0 |
| 219 | 0.002931 | 0.007842 | 0.847892 | 0.141335 | 0 | 0 | 0 | 0 | 0 |
| 220 | 0.003196 | 0.008156 | 0.843304 | 0.145343 | 0 | 0 | 0 | 0 | 0 |
| 221 | 0.002583 | 0.008288 | 0.842149 | 0.14698 | 0 | 0 | 0 | 0 | 0 |
| 222 | 0.002783 | 0.008168 | 0.845442 | 0.143606 | 0 | 0 | 0 | 0 | 0 |
| 223 | 0.003098 | 0.007869 | 0.840359 | 0.148674 | 0 | 0 | 0 | 0 | 0 |
| 224 | 0.002996 | 0.00844 | 0.841277 | 0.147287 | 0 | 0 | 0 | 0 | 0 |
| 225 | 0.002908 | 0.008009 | 0.847114 | 0.14197 | 0 | 0 | 0 | 0 | 0 |
| 226 | 0.002767 | 0.007919 | 0.846134 | 0.143179 | 0 | 0 | 0 | 0 | 0 |
| 227 | 0.002979 | 0.008321 | 0.840254 | 0.148446 | 0 | 0 | 0 | 0 | 0 |
| 228 | 0.002946 | 0.008118 | 0.846324 | 0.142613 | 0 | 0 | 0 | 0 | 0 |
| 229 | 0.003095 | 0.008334 | 0.845745 | 0.142826 | 0 | 0 | 0 | 0 | 0 |
| 230 | 0.00291 | 0.007973 | 0.841874 | 0.147243 | 0 | 0 | 0 | 0 | 0 |
| 231 | 0.002977 | 0.0082 | 0.845782 | 0.143041 | 0 | 0 | 0 | 0 | 0 |
| 232 | 0.002735 | 0.008637 | 0.844175 | 0.144453 | 0 | 0 | 0 | 0 | 0 |
| 233 | 0.003037 | 0.00832 | 0.842159 | 0.146474 | 0 | 0 | 0 | 0 | 0 |
| 234 | 0.002577 | 0.00843 | 0.844302 | 0.144691 | 0 | 0 | 0 | 0 | 0 |
| 235 | 0.002897 | 0.008549 | 0.846224 | 0.14231 | 0 | 0 | 0 | 0 | 0 |
| 236 | 0.002828 | 0.007964 | 0.839645 | 0.149562 | 0 | 0 | 0 | 0 | 0 |
| 237 | 0.003083 | 0.008271 | 0.841375 | 0.147271 | 0 | 0 | 0 | 0 | 0 |
| 238 | 0.003044 | 0.008724 | 0.839368 | 0.148865 | 0 | 0 | 0 | 0 | 0 |
| 239 | 0.002885 | 0.008765 | 0.842263 | 0.146087 | 0 | 0 | 0 | 0 | 0 |
| 240 | 0.002735 | 0.007956 | 0.845344 | 0.143964 | 0 | 0 | 0 | 0 | 0 |
| 241 | 0.003108 | 0.008059 | 0.845287 | 0.143546 | 0 | 0 | 0 | 0 | 0 |
| 242 | 0.00268 | 0.008784 | 0.842427 | 0.146098 | 0 | 0 | 0 | 0 | 0 |
| 243 | 0.002594 | 0.007771 | 0.84608 | 0.143555 | 0 | 0 | 0 | 0 | 0 |
| 244 | 0.003033 | 0.007297 | 0.845338 | 0.144333 | 0 | 0 | 0 | 0 | 0 |
| 245 | 0.003094 | 0.008374 | 0.843044 | 0.145478 | 0 | 0 | 0 | 0 | 0 |
| 246 | 0.003022 | 0.008675 | 0.841648 | 0.146656 | 0 | 0 | 0 | 0 | 0 |
| 247 | 0.003027 | 0.008005 | 0.841624 | 0.147344 | 0 | 0 | 0 | 0 | 0 |
| 248 | 0.003059 | 0.00819 | 0.845604 | 0.143148 | 0 | 0 | 0 | 0 | 0 |
| 249 | 0.003087 | 0.00786 | 0.844957 | 0.144096 | 0 | 0 | 0 | 0 | 0 |
| 250 | 0.002882 | 0.008575 | 0.838825 | 0.149709 | 0 | 0 | 0 | 0 | 0 |
| 251 | 0.002871 | 0.008453 | 0.843617 | 0.145059 | 0 | 0 | 0 | 0 | 0 |
| 252 | 0.002685 | 0.008217 | 0.844242 | 0.144856 | 0 | 0 | 0 | 0 | 0 |
| 253 | 0.003271 | 0.008461 | 0.843575 | 0.144694 | 0 | 0 | 0 | 0 | 0 |
| 254 | 0.003336 | 0.008683 | 0.842607 | 0.145375 | 0 | 0 | 0 | 0 | 0 |
| 255 | 0.003149 | 0.008274 | 0.840017 | 0.14856 | 0 | 0 | 0 | 0 | 0 |
| 256 | 0.003022 | 0.008485 | 0.839868 | 0.148625 | 0 | 0 | 0 | 0 | 0 |
| 257 | 0.003081 | 0.007762 | 0.840407 | 0.14874 | 0 | 0 | 0 | 0 | 0 |
| 258 | 0.003124 | 0.008544 | 0.839792 | 0.14854 | 0 | 0 | 0 | 0 | 0 |
| 259 | 0.002749 | 0.008478 | 0.84374 | 0.145033 | 0 | 0 | 0 | 0 | 0 |
| 260 | 0.003063 | 0.008306 | 0.844708 | 0.143922 | 0 | 0 | 0 | 0 | 0 |
| 261 | 0.002984 | 0.008124 | 0.845223 | 0.143668 | 0 | 0 | 0 | 0 | 0 |
| 262 | 0.002929 | 0.007901 | 0.843468 | 0.145701 | 0 | 0 | 0 | 0 | 0 |
| 263 | 0.002775 | 0.008354 | 0.845036 | 0.143835 | 0 | 0 | 0 | 0 | 0 |
| 264 | 0.002617 | 0.007982 | 0.83981 | 0.14959 | 0 | 0 | 0 | 0 | 0 |
| 265 | 0.003105 | 0.008924 | 0.841797 | 0.146174 | 0 | 0 | 0 | 0 | 0 |
| 266 | 0.002708 | 0.008362 | 0.843464 | 0.145466 | 0 | 0 | 0 | 0 | 0 |
| 267 | 0.002525 | 0.007797 | 0.843699 | 0.145978 | 0 | 0 | 0 | 0 | 0 |
| 268 | 0.002887 | 0.009156 | 0.843103 | 0.144854 | 0 | 0 | 0 | 0 | 0 |
| 269 | 0.003059 | 0.008232 | 0.844094 | 0.144615 | 0 | 0 | 0 | 0 | 0 |
| 270 | 0.002941 | 0.008473 | 0.845772 | 0.142814 | 0 | 0 | 0 | 0 | 0 |
| 271 | 0.003042 | 0.008576 | 0.845603 | 0.142759 | 0 | 0 | 0 | 0 | 0 |
| 272 | 0.002423 | 0.007727 | 0.844047 | 0.145803 | 0 | 0 | 0 | 0 | 0 |
| 273 | 0.002857 | 0.00831 | 0.844414 | 0.14442 | 0 | 0 | 0 | 0 | 0 |
| 274 | 0.002911 | 0.007991 | 0.848034 | 0.141063 | 0 | 0 | 0 | 0 | 0 |
| 275 | 0.002648 | 0.008513 | 0.843792 | 0.145047 | 0 | 0 | 0 | 0 | 0 |
| 276 | 0.002915 | 0.007457 | 0.845017 | 0.144611 | 0 | 0 | 0 | 0 | 0 |
| 277 | 0.002681 | 0.008943 | 0.838915 | 0.149461 | 0 | 0 | 0 | 0 | 0 |
| 278 | 0.002749 | 0.007997 | 0.843558 | 0.145695 | 0 | 0 | 0 | 0 | 0 |
| 279 | 0.003166 | 0.008335 | 0.840144 | 0.148356 | 0 | 0 | 0 | 0 | 0 |
| 280 | 0.00274 | 0.008141 | 0.845533 | 0.143586 | 0 | 0 | 0 | 0 | 0 |
| 281 | 0.002886 | 0.008448 | 0.842149 | 0.146518 | 0 | 0 | 0 | 0 | 0 |
| 282 | 0.002743 | 0.00852 | 0.843477 | 0.145259 | 0 | 0 | 0 | 0 | 0 |
| 283 | 0.002923 | 0.007776 | 0.846679 | 0.142621 | 0 | 0 | 0 | 0 | 0 |
| 284 | 0.002862 | 0.008387 | 0.844923 | 0.143828 | 0 | 0 | 0 | 0 | 0 |
| 285 | 0.003045 | 0.007973 | 0.841353 | 0.147629 | 0 | 0 | 0 | 0 | 0 |
| 286 | 0.002758 | 0.008704 | 0.844454 | 0.144085 | 0 | 0 | 0 | 0 | 0 |
| 287 | 0.002971 | 0.008721 | 0.845027 | 0.143282 | 0 | 0 | 0 | 0 | 0 |
| 288 | 0.00287 | 0.007941 | 0.84412 | 0.145069 | 0 | 0 | 0 | 0 | 0 |
| 289 | 0.00286 | 0.007988 | 0.843503 | 0.145649 | 0 | 0 | 0 | 0 | 0 |
| 290 | 0.002768 | 0.008174 | 0.840226 | 0.148832 | 0 | 0 | 0 | 0 | 0 |
| 291 | 0.003132 | 0.007826 | 0.845539 | 0.143503 | 0 | 0 | 0 | 0 | 0 |
| 292 | 0.00306 | 0.00784 | 0.844578 | 0.144521 | 0 | 0 | 0 | 0 | 0 |
| 293 | 0.00324 | 0.008646 | 0.838355 | 0.149759 | 0 | 0 | 0 | 0 | 0 |
| 294 | 0.002684 | 0.007794 | 0.843381 | 0.146141 | 0 | 0 | 0 | 0 | 0 |
| 295 | 0.003046 | 0.007551 | 0.841723 | 0.14768 | 0 | 0 | 0 | 0 | 0 |
| 296 | 0.003146 | 0.008239 | 0.842327 | 0.146288 | 0 | 0 | 0 | 0 | 0 |
| 297 | 0.002956 | 0.008206 | 0.843678 | 0.14516 | 0 | 0 | 0 | 0 | 0 |
| 298 | 0.003098 | 0.008833 | 0.842843 | 0.145226 | 0 | 0 | 0 | 0 | 0 |
| 299 | 0.00271 | 0.008019 | 0.838416 | 0.150845 | 0 | 0 | 0 | 0 | 0 |
| 300 | 0.002892 | 0.008015 | 0.841502 | 0.14759 | 0 | 0 | 0 | 0 | 0 |
| 301 | 0.002899 | 0.008166 | 0.842662 | 0.146273 | 0 | 0 | 0 | 0 | 0 |
| 302 | 0.002885 | 0.008028 | 0.843659 | 0.145428 | 0 | 0 | 0 | 0 | 0 |
| 303 | 0.002858 | 0.008215 | 0.84339 | 0.145537 | 0 | 0 | 0 | 0 | 0 |
| 304 | 0.002614 | 0.008478 | 0.840582 | 0.148326 | 0 | 0 | 0 | 0 | 0 |
| 305 | 0.003044 | 0.00752 | 0.846384 | 0.143052 | 0 | 0 | 0 | 0 | 0 |
| 306 | 0.002692 | 0.008376 | 0.844749 | 0.144163 | 0 | 0 | 0 | 0 | 0 |
| 307 | 0.003011 | 0.008418 | 0.841669 | 0.146902 | 0 | 0 | 0 | 0 | 0 |
| 308 | 0.003091 | 0.007687 | 0.847235 | 0.141987 | 0 | 0 | 0 | 0 | 0 |
| 309 | 0.002873 | 0.008248 | 0.845295 | 0.143585 | 0 | 0 | 0 | 0 | 0 |
| 310 | 0.003109 | 0.008291 | 0.845567 | 0.143032 | 0 | 0 | 0 | 0 | 0 |
| 311 | 0.003066 | 0.008015 | 0.844554 | 0.144366 | 0 | 0 | 0 | 0 | 0 |
| 312 | 0.003311 | 0.008297 | 0.841062 | 0.147329 | 0 | 0 | 0 | 0 | 0 |
| 313 | 0.003205 | 0.008132 | 0.84433 | 0.144334 | 0 | 0 | 0 | 0 | 0 |
| 314 | 0.002863 | 0.007861 | 0.846681 | 0.142595 | 0 | 0 | 0 | 0 | 0 |
| 315 | 0.003018 | 0.008323 | 0.840478 | 0.14818 | 0 | 0 | 0 | 0 | 0 |
| 316 | 0.002817 | 0.00862 | 0.844165 | 0.144398 | 0 | 0 | 0 | 0 | 0 |
| 317 | 0.002822 | 0.007944 | 0.846567 | 0.142667 | 0 | 0 | 0 | 0 | 0 |
| 318 | 0.002823 | 0.007939 | 0.839475 | 0.149713 | 0 | 0 | 0 | 0 | 0 |
| 319 | 0.002903 | 0.008521 | 0.838489 | 0.150087 | 0 | 0 | 0 | 0 | 0 |
| 320 | 0.003096 | 0.008287 | 0.844208 | 0.144409 | 0 | 0 | 0 | 0 | 0 |
| 321 | 0.003138 | 0.007846 | 0.840694 | 0.148321 | 0 | 0 | 0 | 0 | 0 |
| 322 | 0.003009 | 0.008036 | 0.843849 | 0.145106 | 0 | 0 | 0 | 0 | 0 |
| 323 | 0.002457 | 0.008321 | 0.843404 | 0.145817 | 0 | 0 | 0 | 0 | 0 |
| 324 | 0.002655 | 0.008014 | 0.843932 | 0.1454 | 0 | 0 | 0 | 0 | 0 |
| 325 | 0.003058 | 0.008108 | 0.846894 | 0.14194 | 0 | 0 | 0 | 0 | 0 |
| 326 | 0.002563 | 0.0077 | 0.843161 | 0.146575 | 0 | 0 | 0 | 0 | 0 |
| 327 | 0.00274 | 0.00786 | 0.844552 | 0.144849 | 0 | 0 | 0 | 0 | 0 |
| 328 | 0.003035 | 0.008804 | 0.843618 | 0.144543 | 0 | 0 | 0 | 0 | 0 |
| 329 | 0.003226 | 0.00805 | 0.842362 | 0.146363 | 0 | 0 | 0 | 0 | 0 |
| 330 | 0.002974 | 0.007945 | 0.842295 | 0.146786 | 0 | 0 | 0 | 0 | 0 |
| 331 | 0.003051 | 0.007953 | 0.845867 | 0.143129 | 0 | 0 | 0 | 0 | 0 |
| 332 | 0.003103 | 0.008493 | 0.843818 | 0.144586 | 0 | 0 | 0 | 0 | 0 |
| 333 | 0.002952 | 0.008263 | 0.842602 | 0.146183 | 0 | 0 | 0 | 0 | 0 |
| 334 | 0.002953 | 0.008225 | 0.844598 | 0.144224 | 0 | 0 | 0 | 0 | 0 |
| 335 | 0.002876 | 0.007969 | 0.842501 | 0.146653 | 0 | 0 | 0 | 0 | 0 |
| 336 | 0.002776 | 0.008427 | 0.842909 | 0.145889 | 0 | 0 | 0 | 0 | 0 |
| 337 | 0.003014 | 0.008195 | 0.844456 | 0.144335 | 0 | 0 | 0 | 0 | 0 |
| 338 | 0.002994 | 0.007822 | 0.847292 | 0.141892 | 0 | 0 | 0 | 0 | 0 |
| 339 | 0.002662 | 0.008577 | 0.842374 | 0.146386 | 0 | 0 | 0 | 0 | 0 |
| 340 | 0.002931 | 0.008674 | 0.841814 | 0.146582 | 0 | 0 | 0 | 0 | 0 |
| 341 | 0.003087 | 0.00852 | 0.842795 | 0.145598 | 0 | 0 | 0 | 0 | 0 |
| 342 | 0.002768 | 0.007744 | 0.840976 | 0.148513 | 0 | 0 | 0 | 0 | 0 |
| 343 | 0.00285 | 0.007821 | 0.844831 | 0.144497 | 0 | 0 | 0 | 0 | 0 |
| 344 | 0.002836 | 0.007555 | 0.84423 | 0.145379 | 0 | 0 | 0 | 0 | 0 |
| 345 | 0.003378 | 0.008275 | 0.843 | 0.145347 | 0 | 0 | 0 | 0 | 0 |
| 346 | 0.003069 | 0.008097 | 0.845929 | 0.142906 | 0 | 0 | 0 | 0 | 0 |
| 347 | 0.003113 | 0.008395 | 0.844644 | 0.143818 | 0 | 0 | 0 | 0 | 0 |
| 348 | 0.002848 | 0.008214 | 0.842565 | 0.146373 | 0 | 0 | 0 | 0 | 0 |
| 349 | 0.002809 | 0.007751 | 0.843683 | 0.145758 | 0 | 0 | 0 | 0 | 0 |
| 350 | 0.002725 | 0.008444 | 0.845686 | 0.143145 | 0 | 0 | 0 | 0 | 0 |
| 351 | 0.003288 | 0.008116 | 0.844374 | 0.144222 | 0 | 0 | 0 | 0 | 0 |
| 352 | 0.003001 | 0.008346 | 0.845813 | 0.14284 | 0 | 0 | 0 | 0 | 0 |
| 353 | 0.002779 | 0.008127 | 0.844287 | 0.144808 | 0 | 0 | 0 | 0 | 0 |
| 354 | 0.002981 | 0.007656 | 0.841646 | 0.147717 | 0 | 0 | 0 | 0 | 0 |
| 355 | 0.002716 | 0.008459 | 0.8449 | 0.143926 | 0 | 0 | 0 | 0 | 0 |
| 356 | 0.003133 | 0.008284 | 0.843095 | 0.145489 | 0 | 0 | 0 | 0 | 0 |
| 357 | 0.003272 | 0.008735 | 0.840706 | 0.147288 | 0 | 0 | 0 | 0 | 0 |
| 358 | 0.002841 | 0.008 | 0.845167 | 0.143993 | 0 | 0 | 0 | 0 | 0 |
| 359 | 0.002932 | 0.008538 | 0.841783 | 0.146747 | 0 | 0 | 0 | 0 | 0 |
| 360 | 0.002712 | 0.008325 | 0.843361 | 0.145602 | 0 | 0 | 0 | 0 | 0 |
| 361 | 0.002734 | 0.008801 | 0.840849 | 0.147616 | 0 | 0 | 0 | 0 | 0 |
| 362 | 0.00341 | 0.00816 | 0.845441 | 0.142989 | 0 | 0 | 0 | 0 | 0 |
| 363 | 0.003377 | 0.007915 | 0.844288 | 0.14442 | 0 | 0 | 0 | 0 | 0 |
| 364 | 0.002888 | 0.008865 | 0.844965 | 0.143281 | 0 | 0 | 0 | 0 | 0 |
| 365 | 0.003046 | 0.008129 | 0.843281 | 0.145545 | 0 | 0 | 0 | 0 | 0 |
| 366 | 0.003031 | 0.008277 | 0.842989 | 0.145703 | 0 | 0 | 0 | 0 | 0 |
| 367 | 0.003227 | 0.008443 | 0.840932 | 0.147398 | 0 | 0 | 0 | 0 | 0 |
| 368 | 0.002779 | 0.008116 | 0.845655 | 0.14345 | 0 | 0 | 0 | 0 | 0 |
| 369 | 0.003257 | 0.008232 | 0.841451 | 0.14706 | 0 | 0 | 0 | 0 | 0 |
| 370 | 0.003019 | 0.00826 | 0.841849 | 0.146871 | 0 | 0 | 0 | 0 | 0 |
| 371 | 0.002955 | 0.008344 | 0.840079 | 0.148562 | 0 | 0 | 0 | 0 | 0 |
| 372 | 0.002644 | 0.008242 | 0.840904 | 0.14821 | 0 | 0 | 0 | 0 | 0 |
| 373 | 0.00312 | 0.008246 | 0.842617 | 0.146017 | 0 | 0 | 0 | 0 | 0 |
| 374 | 0.002966 | 0.008035 | 0.846354 | 0.142645 | 0 | 0 | 0 | 0 | 0 |
| 375 | 0.002804 | 0.007819 | 0.843837 | 0.14554 | 0 | 0 | 0 | 0 | 0 |
| 376 | 0.003162 | 0.008302 | 0.842668 | 0.145868 | 0 | 0 | 0 | 0 | 0 |
| 377 | 0.002949 | 0.008575 | 0.841313 | 0.147163 | 0 | 0 | 0 | 0 | 0 |
| 378 | 0.00303 | 0.008368 | 0.847967 | 0.140636 | 0 | 0 | 0 | 0 | 0 |
| 379 | 0.003094 | 0.008009 | 0.844116 | 0.144781 | 0 | 0 | 0 | 0 | 0 |
| 380 | 0.002799 | 0.0078 | 0.843785 | 0.145615 | 0 | 0 | 0 | 0 | 0 |
| 381 | 0.002792 | 0.008078 | 0.842115 | 0.147015 | 0 | 0 | 0 | 0 | 0 |
| 382 | 0.003146 | 0.007776 | 0.841606 | 0.147471 | 0 | 0 | 0 | 0 | 0 |
| 383 | 0.002892 | 0.007842 | 0.845503 | 0.143764 | 0 | 0 | 0 | 0 | 0 |
| 384 | 0.002822 | 0.008237 | 0.840379 | 0.148562 | 0 | 0 | 0 | 0 | 0 |
| 385 | 0.002936 | 0.008218 | 0.845767 | 0.14308 | 0 | 0 | 0 | 0 | 0 |
| 386 | 0.002991 | 0.008501 | 0.843575 | 0.144933 | 0 | 0 | 0 | 0 | 0 |
| 387 | 0.002956 | 0.008268 | 0.842455 | 0.146322 | 0 | 0 | 0 | 0 | 0 |
| 388 | 0.002998 | 0.007831 | 0.843895 | 0.145276 | 0 | 0 | 0 | 0 | 0 |
| 389 | 0.003134 | 0.008428 | 0.846437 | 0.142002 | 0 | 0 | 0 | 0 | 0 |
| 390 | 0.002501 | 0.008504 | 0.844862 | 0.144132 | 0 | 0 | 0 | 0 | 0 |
| 391 | 0.002886 | 0.008699 | 0.838078 | 0.150337 | 0 | 0 | 0 | 0 | 0 |
| 392 | 0.002852 | 0.008716 | 0.843843 | 0.144589 | 0 | 0 | 0 | 0 | 0 |
| 393 | 0.002833 | 0.008659 | 0.841242 | 0.147267 | 0 | 0 | 0 | 0 | 0 |
| 394 | 0.002997 | 0.008392 | 0.843544 | 0.145067 | 0 | 0 | 0 | 0 | 0 |
| 395 | 0.002747 | 0.007545 | 0.843276 | 0.146432 | 0 | 0 | 0 | 0 | 0 |
| 396 | 0.003175 | 0.007683 | 0.845201 | 0.14394 | 0 | 0 | 0 | 0 | 0 |
| 397 | 0.003314 | 0.00844 | 0.845385 | 0.142851 | 0 | 0 | 0 | 0 | 0 |
| 398 | 0.002719 | 0.007648 | 0.846627 | 0.143007 | 0 | 0 | 0 | 0 | 0 |
| 399 | 0.003041 | 0.008361 | 0.842708 | 0.14589 | 0 | 0 | 0 | 0 | 0 |
| 400 | 0.003137 | 0.007891 | 0.845571 | 0.143401 | 0 | 0 | 0 | 0 | 0 |
| 401 | 0.003005 | 0.008224 | 0.842376 | 0.146395 | 0 | 0 | 0 | 0 | 0 |
| 402 | 0.002763 | 0.007877 | 0.844029 | 0.14533 | 0 | 0 | 0 | 0 | 0 |
| 403 | 0.002768 | 0.008492 | 0.84505 | 0.14369 | 0 | 0 | 0 | 0 | 0 |
| 404 | 0.002733 | 0.008957 | 0.844493 | 0.143816 | 0 | 0 | 0 | 0 | 0 |
| 405 | 0.003052 | 0.008166 | 0.846174 | 0.142608 | 0 | 0 | 0 | 0 | 0 |
| 406 | 0.002759 | 0.008617 | 0.841917 | 0.146707 | 0 | 0 | 0 | 0 | 0 |
| 407 | 0.002492 | 0.008901 | 0.843947 | 0.144661 | 0 | 0 | 0 | 0 | 0 |
| 408 | 0.002695 | 0.007664 | 0.841217 | 0.148425 | 0 | 0 | 0 | 0 | 0 |
| 409 | 0.002767 | 0.009328 | 0.84302 | 0.144884 | 0 | 0 | 0 | 0 | 0 |
| 410 | 0.002763 | 0.00825 | 0.843025 | 0.145962 | 0 | 0 | 0 | 0 | 0 |
| 411 | 0.003059 | 0.008758 | 0.84369 | 0.144493 | 0 | 0 | 0 | 0 | 0 |
| 412 | 0.002704 | 0.008073 | 0.842854 | 0.146369 | 0 | 0 | 0 | 0 | 0 |
| 413 | 0.00286 | 0.007869 | 0.842037 | 0.147234 | 0 | 0 | 0 | 0 | 0 |
| 414 | 0.002935 | 0.008605 | 0.844572 | 0.143888 | 0 | 0 | 0 | 0 | 0 |
| 415 | 0.002976 | 0.008082 | 0.845109 | 0.143833 | 0 | 0 | 0 | 0 | 0 |
| 416 | 0.002908 | 0.008692 | 0.842869 | 0.145501 | 0 | 0 | 0 | 0 | 0 |
| 417 | 0.002825 | 0.008025 | 0.842787 | 0.146363 | 0 | 0 | 0 | 0 | 0 |
| 418 | 0.003012 | 0.008026 | 0.845132 | 0.143831 | 0 | 0 | 0 | 0 | 0 |
| 419 | 0.003179 | 0.008485 | 0.840566 | 0.14777 | 0 | 0 | 0 | 0 | 0 |
| 420 | 0.002818 | 0.008425 | 0.841028 | 0.147729 | 0 | 0 | 0 | 0 | 0 |
| 421 | 0.002794 | 0.008602 | 0.843364 | 0.145239 | 0 | 0 | 0 | 0 | 0 |
| 422 | 0.002845 | 0.008786 | 0.843679 | 0.144689 | 0 | 0 | 0 | 0 | 0 |
| 423 | 0.002995 | 0.008204 | 0.843825 | 0.144976 | 0 | 0 | 0 | 0 | 0 |
| 424 | 0.002841 | 0.008853 | 0.846568 | 0.141728 | 0 | 0 | 0 | 0 | 0 |
| 425 | 0.003379 | 0.008291 | 0.840931 | 0.147399 | 0 | 0 | 0 | 0 | 0 |
| 426 | 0.003028 | 0.008774 | 0.839012 | 0.149186 | 0 | 0 | 0 | 0 | 0 |
| 427 | 0.002875 | 0.009088 | 0.843197 | 0.14484 | 0 | 0 | 0 | 0 | 0 |
| 428 | 0.002886 | 0.008417 | 0.847435 | 0.141262 | 0 | 0 | 0 | 0 | 0 |
| 429 | 0.002903 | 0.00851 | 0.843424 | 0.145163 | 0 | 0 | 0 | 0 | 0 |
| 430 | 0.002891 | 0.008472 | 0.84179 | 0.146848 | 0 | 0 | 0 | 0 | 0 |
| 431 | 0.003097 | 0.007927 | 0.84449 | 0.144486 | 0 | 0 | 0 | 0 | 0 |
| 432 | 0.002919 | 0.008077 | 0.842716 | 0.146289 | 0 | 0 | 0 | 0 | 0 |
| 433 | 0.003414 | 0.009035 | 0.842108 | 0.145443 | 0 | 0 | 0 | 0 | 0 |
| 434 | 0.003044 | 0.008221 | 0.842836 | 0.1459 | 0 | 0 | 0 | 0 | 0 |
| 435 | 0.003062 | 0.008457 | 0.84477 | 0.143712 | 0 | 0 | 0 | 0 | 0 |
| 436 | 0.003088 | 0.008112 | 0.841385 | 0.147415 | 0 | 0 | 0 | 0 | 0 |
| 437 | 0.003109 | 0.007834 | 0.840798 | 0.148259 | 0 | 0 | 0 | 0 | 0 |
| 438 | 0.003061 | 0.008705 | 0.842271 | 0.145963 | 0 | 0 | 0 | 0 | 0 |
| 439 | 0.003077 | 0.008276 | 0.843968 | 0.144679 | 0 | 0 | 0 | 0 | 0 |
| 440 | 0.003074 | 0.007845 | 0.843922 | 0.145159 | 0 | 0 | 0 | 0 | 0 |
| 441 | 0.002818 | 0.008564 | 0.845433 | 0.143185 | 0 | 0 | 0 | 0 | 0 |
| 442 | 0.002835 | 0.008035 | 0.842674 | 0.146456 | 0 | 0 | 0 | 0 | 0 |
| 443 | 0.002852 | 0.007779 | 0.843061 | 0.146308 | 0 | 0 | 0 | 0 | 0 |
| 444 | 0.003049 | 0.008527 | 0.843378 | 0.145046 | 0 | 0 | 0 | 0 | 0 |
| 445 | 0.002923 | 0.007822 | 0.840706 | 0.148549 | 0 | 0 | 0 | 0 | 0 |
| 446 | 0.00311 | 0.007939 | 0.842824 | 0.146127 | 0 | 0 | 0 | 0 | 0 |
| 447 | 0.003007 | 0.00834 | 0.842817 | 0.145836 | 0 | 0 | 0 | 0 | 0 |
| 448 | 0.003167 | 0.008739 | 0.840609 | 0.147486 | 0 | 0 | 0 | 0 | 0 |
| 449 | 0.002691 | 0.008173 | 0.843317 | 0.145819 | 0 | 0 | 0 | 0 | 0 |
| 450 | 0.003039 | 0.008587 | 0.842344 | 0.14603 | 0 | 0 | 0 | 0 | 0 |
| 451 | 0.003135 | 0.008046 | 0.846946 | 0.141873 | 0 | 0 | 0 | 0 | 0 |
| 452 | 0.003002 | 0.008277 | 0.841816 | 0.146906 | 0 | 0 | 0 | 0 | 0 |
| 453 | 0.00275 | 0.008181 | 0.84547 | 0.143599 | 0 | 0 | 0 | 0 | 0 |
| 454 | 0.002962 | 0.007439 | 0.846114 | 0.143485 | 0 | 0 | 0 | 0 | 0 |
| 455 | 0.00268 | 0.008549 | 0.847547 | 0.141223 | 0 | 0 | 0 | 0 | 0 |
| 456 | 0.002753 | 0.0082 | 0.842579 | 0.146468 | 0 | 0 | 0 | 0 | 0 |
| 457 | 0.002741 | 0.008541 | 0.842213 | 0.146496 | 0 | 0 | 0 | 0 | 0 |
| 458 | 0.002942 | 0.007949 | 0.845461 | 0.143649 | 0 | 0 | 0 | 0 | 0 |
| 459 | 0.002979 | 0.007618 | 0.842023 | 0.14738 | 0 | 0 | 0 | 0 | 0 |
| 460 | 0.002407 | 0.008475 | 0.841805 | 0.147313 | 0 | 0 | 0 | 0 | 0 |
| 461 | 0.002842 | 0.008945 | 0.844645 | 0.143568 | 0 | 0 | 0 | 0 | 0 |
| 462 | 0.002877 | 0.00742 | 0.846393 | 0.143309 | 0 | 0 | 0 | 0 | 0 |
| 463 | 0.00256 | 0.009031 | 0.840904 | 0.147505 | 0 | 0 | 0 | 0 | 0 |
| 464 | 0.003149 | 0.008812 | 0.844 | 0.144018 | 0 | 0 | 0 | 0 | 0 |
| 465 | 0.003082 | 0.008015 | 0.846351 | 0.142553 | 0 | 0 | 0 | 0 | 0 |
| 466 | 0.002845 | 0.009083 | 0.839355 | 0.148717 | 0 | 0 | 0 | 0 | 0 |
| 467 | 0.003092 | 0.007559 | 0.839879 | 0.14947 | 0 | 0 | 0 | 0 | 0 |
| 468 | 0.002831 | 0.008464 | 0.845075 | 0.14363 | 0 | 0 | 0 | 0 | 0 |
| 469 | 0.002881 | 0.007297 | 0.84586 | 0.143963 | 0 | 0 | 0 | 0 | 0 |
| 470 | 0.002884 | 0.007757 | 0.846444 | 0.142915 | 0 | 0 | 0 | 0 | 0 |
| 471 | 0.003042 | 0.008351 | 0.846338 | 0.142269 | 0 | 0 | 0 | 0 | 0 |
| 472 | 0.002747 | 0.007809 | 0.844239 | 0.145206 | 0 | 0 | 0 | 0 | 0 |
| 473 | 0.002784 | 0.008223 | 0.841476 | 0.147516 | 0 | 0 | 0 | 0 | 0 |
| 474 | 0.003168 | 0.008345 | 0.843906 | 0.14458 | 0 | 0 | 0 | 0 | 0 |
| 475 | 0.003123 | 0.008669 | 0.841699 | 0.146509 | 0 | 0 | 0 | 0 | 0 |
| 476 | 0.002975 | 0.008764 | 0.840571 | 0.14769 | 0 | 0 | 0 | 0 | 0 |
| 477 | 0.002514 | 0.008114 | 0.842965 | 0.146406 | 0 | 0 | 0 | 0 | 0 |
| 478 | 0.002633 | 0.008561 | 0.841644 | 0.147161 | 0 | 0 | 0 | 0 | 0 |
| 479 | 0.002876 | 0.00806 | 0.844218 | 0.144846 | 0 | 0 | 0 | 0 | 0 |
| 480 | 0.002737 | 0.00829 | 0.839336 | 0.149638 | 0 | 0 | 0 | 0 | 0 |
| 481 | 0.002814 | 0.008343 | 0.849667 | 0.139176 | 0 | 0 | 0 | 0 | 0 |
| 482 | 0.002795 | 0.008064 | 0.841197 | 0.147943 | 0 | 0 | 0 | 0 | 0 |
| 483 | 0.003168 | 0.008003 | 0.843813 | 0.145016 | 0 | 0 | 0 | 0 | 0 |
| 484 | 0.002963 | 0.008292 | 0.839038 | 0.149707 | 0 | 0 | 0 | 0 | 0 |
| 485 | 0.002943 | 0.00836 | 0.845787 | 0.14291 | 0 | 0 | 0 | 0 | 0 |
| 486 | 0.002654 | 0.008632 | 0.843891 | 0.144824 | 0 | 0 | 0 | 0 | 0 |
| 487 | 0.00263 | 0.007911 | 0.846653 | 0.142806 | 0 | 0 | 0 | 0 | 0 |
| 488 | 0.002978 | 0.008009 | 0.847491 | 0.141522 | 0 | 0 | 0 | 0 | 0 |
| 489 | 0.003008 | 0.008174 | 0.84593 | 0.142889 | 0 | 0 | 0 | 0 | 0 |
| 490 | 0.002894 | 0.008982 | 0.841813 | 0.146312 | 0 | 0 | 0 | 0 | 0 |
| 491 | 0.002749 | 0.008685 | 0.842072 | 0.146494 | 0 | 0 | 0 | 0 | 0 |
| 492 | 0.002961 | 0.008498 | 0.837655 | 0.150887 | 0 | 0 | 0 | 0 | 0 |
| 493 | 0.002939 | 0.008307 | 0.842152 | 0.146602 | 0 | 0 | 0 | 0 | 0 |
| 494 | 0.00293 | 0.008277 | 0.837633 | 0.15115 | 0 | 0 | 0 | 0 | 0 |
| 495 | 0.003228 | 0.008877 | 0.839223 | 0.148671 | 0 | 0 | 0 | 0 | 0 |
| 496 | 0.00305 | 0.008029 | 0.839978 | 0.148874 | 0 | 0 | 0 | 0 | 0 |
| 497 | 0.002792 | 0.007456 | 0.845182 | 0.14457 | 0 | 0 | 0 | 0 | 0 |
| 498 | 0.0027 | 0.00834 | 0.844032 | 0.144928 | 0 | 0 | 0 | 0 | 0 |
| 499 | 0.00278 | 0.008541 | 0.839536 | 0.149144 | 0 | 0 | 0 | 0 | 0 |
| 500 | 0.002917 | 0.007995 | 0.844364 | 0.144723 | 0 | 0 | 0 | 0 | 0 |
| 501 | 0.003008 | 0.008974 | 0.842621 | 0.145397 | 0 | 0 | 0 | 0 | 0 |
| 502 | 0.002753 | 0.008048 | 0.844901 | 0.144299 | 0 | 0 | 0 | 0 | 0 |
| 503 | 0.003153 | 0.007917 | 0.843272 | 0.145658 | 0 | 0 | 0 | 0 | 0 |
| 504 | 0.002847 | 0.007939 | 0.846111 | 0.143103 | 0 | 0 | 0 | 0 | 0 |
| 505 | 0.003012 | 0.00829 | 0.84406 | 0.144628 | 0 | 0 | 0 | 0 | 0 |
| 506 | 0.002832 | 0.007626 | 0.845419 | 0.144123 | 0 | 0 | 0 | 0 | 0 |
| 507 | 0.003077 | 0.008438 | 0.84363 | 0.144856 | 0 | 0 | 0 | 0 | 0 |
| 508 | 0.002713 | 0.007607 | 0.842656 | 0.147023 | 0 | 0 | 0 | 0 | 0 |
| 509 | 0.002662 | 0.008066 | 0.843846 | 0.145425 | 0 | 0 | 0 | 0 | 0 |
| 510 | 0.002757 | 0.008252 | 0.845412 | 0.143578 | 0 | 0 | 0 | 0 | 0 |
| 511 | 0.002921 | 0.008271 | 0.84422 | 0.144588 | 0 | 0 | 0 | 0 | 0 |
| 512 | 0.002951 | 0.008595 | 0.841961 | 0.146492 | 0 | 0 | 0 | 0 | 0 |
| 513 | 0.002869 | 0.008478 | 0.84501 | 0.143643 | 0 | 0 | 0 | 0 | 0 |
| 514 | 0.003038 | 0.008266 | 0.842446 | 0.14625 | 0 | 0 | 0 | 0 | 0 |
| 515 | 0.002979 | 0.008436 | 0.841503 | 0.147082 | 0 | 0 | 0 | 0 | 0 |
| 516 | 0.002546 | 0.008056 | 0.843647 | 0.14575 | 0 | 0 | 0 | 0 | 0 |
| 517 | 0.002894 | 0.007992 | 0.842582 | 0.146531 | 0 | 0 | 0 | 0 | 0 |
| 518 | 0.002699 | 0.008418 | 0.843708 | 0.145175 | 0 | 0 | 0 | 0 | 0 |
| 519 | 0.00293 | 0.008611 | 0.840078 | 0.148301 | 0 | 0 | 0 | 0 | 0 |
| 520 | 0.003237 | 0.008062 | 0.841863 | 0.146828 | 0 | 0 | 0 | 0 | 0 |
| 521 | 0.00298 | 0.008239 | 0.843133 | 0.145648 | 0 | 0 | 0 | 0 | 0 |
| 522 | 0.00316 | 0.00768 | 0.843509 | 0.145651 | 0 | 0 | 0 | 0 | 0 |
| 523 | 0.002865 | 0.008245 | 0.845246 | 0.143644 | 0 | 0 | 0 | 0 | 0 |
| 524 | 0.003081 | 0.007723 | 0.840982 | 0.148214 | 0 | 0 | 0 | 0 | 0 |
| 525 | 0.002701 | 0.008114 | 0.842244 | 0.146941 | 0 | 0 | 0 | 0 | 0 |
| 526 | 0.002906 | 0.007556 | 0.842618 | 0.14692 | 0 | 0 | 0 | 0 | 0 |
| 527 | 0.002842 | 0.008377 | 0.84355 | 0.145231 | 0 | 0 | 0 | 0 | 0 |
| 528 | 0.003131 | 0.008262 | 0.843667 | 0.14494 | 0 | 0 | 0 | 0 | 0 |
| 529 | 0.002747 | 0.00827 | 0.841739 | 0.147244 | 0 | 0 | 0 | 0 | 0 |
| 530 | 0.002863 | 0.00832 | 0.839901 | 0.148917 | 0 | 0 | 0 | 0 | 0 |
| 531 | 0.002803 | 0.008757 | 0.841547 | 0.146894 | 0 | 0 | 0 | 0 | 0 |
| 532 | 0.002892 | 0.008275 | 0.844846 | 0.143986 | 0 | 0 | 0 | 0 | 0 |
| 533 | 0.002669 | 0.007858 | 0.842733 | 0.146739 | 0 | 0 | 0 | 0 | 0 |
| 534 | 0.002866 | 0.008317 | 0.840062 | 0.148755 | 0 | 0 | 0 | 0 | 0 |
| 535 | 0.002493 | 0.008439 | 0.844719 | 0.144349 | 0 | 0 | 0 | 0 | 0 |
| 536 | 0.002813 | 0.008157 | 0.84446 | 0.14451 | 0 | 0 | 0 | 0 | 0 |
| 537 | 0.002617 | 0.008011 | 0.8453 | 0.144072 | 0 | 0 | 0 | 0 | 0 |
| 538 | 0.002798 | 0.007709 | 0.840597 | 0.148896 | 0 | 0 | 0 | 0 | 0 |
| 539 | 0.003061 | 0.008703 | 0.842277 | 0.145958 | 0 | 0 | 0 | 0 | 0 |
| 540 | 0.00302 | 0.008658 | 0.839425 | 0.148897 | 0 | 0 | 0 | 0 | 0 |
| 541 | 0.00274 | 0.00783 | 0.843492 | 0.145937 | 0 | 0 | 0 | 0 | 0 |
| 542 | 0.002766 | 0.008397 | 0.843278 | 0.14556 | 0 | 0 | 0 | 0 | 0 |
| 543 | 0.003 | 0.008497 | 0.846756 | 0.141747 | 0 | 0 | 0 | 0 | 0 |
| 544 | 0.002902 | 0.008215 | 0.843301 | 0.145563 | 0 | 0 | 0 | 0 | 0 |
| 545 | 0.002744 | 0.008858 | 0.840684 | 0.147714 | 0 | 0 | 0 | 0 | 0 |
| 546 | 0.002945 | 0.008655 | 0.842203 | 0.146196 | 0 | 0 | 0 | 0 | 0 |
| 547 | 0.003204 | 0.007981 | 0.842833 | 0.145983 | 0 | 0 | 0 | 0 | 0 |
| 548 | 0.002846 | 0.008739 | 0.845449 | 0.142966 | 0 | 0 | 0 | 0 | 0 |
| 549 | 0.003067 | 0.008741 | 0.839356 | 0.148836 | 0 | 0 | 0 | 0 | 0 |
| 550 | 0.003105 | 0.008022 | 0.844169 | 0.144704 | 0 | 0 | 0 | 0 | 0 |
| 551 | 0.002807 | 0.008562 | 0.843125 | 0.145506 | 0 | 0 | 0 | 0 | 0 |
| 552 | 0.003227 | 0.00854 | 0.842044 | 0.146189 | 0 | 0 | 0 | 0 | 0 |
| 553 | 0.002813 | 0.008417 | 0.840771 | 0.147999 | 0 | 0 | 0 | 0 | 0 |
| 554 | 0.002972 | 0.008445 | 0.844731 | 0.143852 | 0 | 0 | 0 | 0 | 0 |
| 555 | 0.002836 | 0.007763 | 0.842049 | 0.147352 | 0 | 0 | 0 | 0 | 0 |
| 556 | 0.002722 | 0.008605 | 0.847011 | 0.141662 | 0 | 0 | 0 | 0 | 0 |
| 557 | 0.002837 | 0.008871 | 0.844484 | 0.143808 | 0 | 0 | 0 | 0 | 0 |
| 558 | 0.003067 | 0.008012 | 0.84184 | 0.14708 | 0 | 0 | 0 | 0 | 0 |
| 559 | 0.003068 | 0.008274 | 0.842945 | 0.145714 | 0 | 0 | 0 | 0 | 0 |
| 560 | 0.002985 | 0.008245 | 0.843828 | 0.144942 | 0 | 0 | 0 | 0 | 0 |
| 561 | 0.003146 | 0.008856 | 0.841708 | 0.14629 | 0 | 0 | 0 | 0 | 0 |
| 562 | 0.002746 | 0.008407 | 0.843486 | 0.145361 | 0 | 0 | 0 | 0 | 0 |
| 563 | 0.003065 | 0.008237 | 0.841654 | 0.147044 | 0 | 0 | 0 | 0 | 0 |
| 564 | 0.002589 | 0.008274 | 0.845877 | 0.14326 | 0 | 0 | 0 | 0 | 0 |
| 565 | 0.002884 | 0.0081 | 0.841795 | 0.147221 | 0 | 0 | 0 | 0 | 0 |
| 566 | 0.003006 | 0.008516 | 0.838884 | 0.149594 | 0 | 0 | 0 | 0 | 0 |
| 567 | 0.002998 | 0.008455 | 0.842183 | 0.146365 | 0 | 0 | 0 | 0 | 0 |
| 568 | 0.002924 | 0.008026 | 0.845227 | 0.143823 | 0 | 0 | 0 | 0 | 0 |
| 569 | 0.002911 | 0.008414 | 0.845956 | 0.142719 | 0 | 0 | 0 | 0 | 0 |
| 570 | 0.002722 | 0.007925 | 0.842873 | 0.146481 | 0 | 0 | 0 | 0 | 0 |
| 571 | 0.003101 | 0.007833 | 0.843467 | 0.145598 | 0 | 0 | 0 | 0 | 0 |
| 572 | 0.002721 | 0.008052 | 0.842355 | 0.146872 | 0 | 0 | 0 | 0 | 0 |
| 573 | 0.00311 | 0.008299 | 0.843634 | 0.144957 | 0 | 0 | 0 | 0 | 0 |
| 574 | 0.002505 | 0.007766 | 0.845693 | 0.144015 | 0 | 0 | 0 | 0 | 0 |
| 575 | 0.002682 | 0.007786 | 0.843276 | 0.146255 | 0 | 0 | 0 | 0 | 0 |
| 576 | 0.003054 | 0.007521 | 0.844974 | 0.144452 | 0 | 0 | 0 | 0 | 0 |
| 577 | 0.00316 | 0.008523 | 0.838979 | 0.149338 | 0 | 0 | 0 | 0 | 0 |
| 578 | 0.003007 | 0.008078 | 0.842524 | 0.146392 | 0 | 0 | 0 | 0 | 0 |
| 579 | 0.002853 | 0.008106 | 0.841961 | 0.14708 | 0 | 0 | 0 | 0 | 0 |
| 580 | 0.002894 | 0.008451 | 0.844037 | 0.144618 | 0 | 0 | 0 | 0 | 0 |
| 581 | 0.002673 | 0.008402 | 0.842072 | 0.146853 | 0 | 0 | 0 | 0 | 0 |
| 582 | 0.003026 | 0.008395 | 0.84234 | 0.146239 | 0 | 0 | 0 | 0 | 0 |
| 583 | 0.002975 | 0.008077 | 0.8424 | 0.146548 | 0 | 0 | 0 | 0 | 0 |
| 584 | 0.002683 | 0.008248 | 0.84273 | 0.146338 | 0 | 0 | 0 | 0 | 0 |
| 585 | 0.003074 | 0.008365 | 0.84237 | 0.146191 | 0 | 0 | 0 | 0 | 0 |
| 586 | 0.002867 | 0.008391 | 0.840867 | 0.147874 | 0 | 0 | 0 | 0 | 0 |
| 587 | 0.002958 | 0.007331 | 0.844735 | 0.144976 | 0 | 0 | 0 | 0 | 0 |
| 588 | 0.002762 | 0.008205 | 0.844382 | 0.144651 | 0 | 0 | 0 | 0 | 0 |
| 589 | 0.002591 | 0.007843 | 0.846883 | 0.142683 | 0 | 0 | 0 | 0 | 0 |
| 590 | 0.002726 | 0.007967 | 0.83879 | 0.150517 | 0 | 0 | 0 | 0 | 0 |
| 591 | 0.002905 | 0.007757 | 0.843875 | 0.145463 | 0 | 0 | 0 | 0 | 0 |
| 592 | 0.002613 | 0.008748 | 0.843021 | 0.145617 | 0 | 0 | 0 | 0 | 0 |
| 593 | 0.002646 | 0.008008 | 0.845728 | 0.143617 | 0 | 0 | 0 | 0 | 0 |
| 594 | 0.002927 | 0.007764 | 0.844959 | 0.14435 | 0 | 0 | 0 | 0 | 0 |
| 595 | 0.002835 | 0.008655 | 0.844494 | 0.144015 | 0 | 0 | 0 | 0 | 0 |
| 596 | 0.002921 | 0.008125 | 0.846755 | 0.142199 | 0 | 0 | 0 | 0 | 0 |
| 597 | 0.002916 | 0.008408 | 0.840993 | 0.147683 | 0 | 0 | 0 | 0 | 0 |
| 598 | 0.002883 | 0.008158 | 0.84704 | 0.141919 | 0 | 0 | 0 | 0 | 0 |
| 599 | 0.00259 | 0.00788 | 0.844262 | 0.145267 | 0 | 0 | 0 | 0 | 0 |
| 600 | 0.003208 | 0.0081 | 0.840781 | 0.147891 | 0 | 0 | 0 | 0 | 0 |
| 601 | 0.002378 | 0.008728 | 0.841931 | 0.146964 | 0 | 0 | 0 | 0 | 0 |
| 602 | 0.002693 | 0.008359 | 0.844585 | 0.144363 | 0 | 0 | 0 | 0 | 0 |
| 603 | 0.003173 | 0.008349 | 0.843057 | 0.14542 | 0 | 0 | 0 | 0 | 0 |
| 604 | 0.00326 | 0.008695 | 0.843914 | 0.144131 | 0 | 0 | 0 | 0 | 0 |
| 605 | 0.002672 | 0.008596 | 0.845639 | 0.143093 | 0 | 0 | 0 | 0 | 0 |
| 606 | 0.002815 | 0.008223 | 0.842156 | 0.146806 | 0 | 0 | 0 | 0 | 0 |
| 607 | 0.002775 | 0.008415 | 0.846436 | 0.142374 | 0 | 0 | 0 | 0 | 0 |
| 608 | 0.003035 | 0.008247 | 0.840317 | 0.148401 | 0 | 0 | 0 | 0 | 0 |
| 609 | 0.00313 | 0.007978 | 0.846289 | 0.142603 | 0 | 0 | 0 | 0 | 0 |
| 610 | 0.002881 | 0.008045 | 0.844885 | 0.144189 | 0 | 0 | 0 | 0 | 0 |
| 611 | 0.002862 | 0.008248 | 0.845249 | 0.143641 | 0 | 0 | 0 | 0 | 0 |
| 612 | 0.003432 | 0.007616 | 0.840399 | 0.148554 | 0 | 0 | 0 | 0 | 0 |
| 613 | 0.002852 | 0.008354 | 0.841448 | 0.147326 | 0 | 0 | 0 | 0 | 0 |
| 614 | 0.003114 | 0.008509 | 0.849512 | 0.138864 | 0 | 0 | 0 | 0 | 0 |
| 615 | 0.003257 | 0.008082 | 0.845915 | 0.142726 | 0 | 0 | 0 | 0 | 0 |
| 616 | 0.003145 | 0.008223 | 0.842592 | 0.14604 | 0 | 0 | 0 | 0 | 0 |
| 617 | 0.00326 | 0.008415 | 0.844803 | 0.143521 | 0 | 0 | 0 | 0 | 0 |
| 618 | 0.002989 | 0.00815 | 0.842199 | 0.146663 | 0 | 0 | 0 | 0 | 0 |
| 619 | 0.002748 | 0.008225 | 0.843138 | 0.145889 | 0 | 0 | 0 | 0 | 0 |
| 620 | 0.002993 | 0.007932 | 0.845429 | 0.143645 | 0 | 0 | 0 | 0 | 0 |
| 621 | 0.003114 | 0.008006 | 0.845127 | 0.143753 | 0 | 0 | 0 | 0 | 0 |
| 622 | 0.003123 | 0.008288 | 0.841161 | 0.147429 | 0 | 0 | 0 | 0 | 0 |
| 623 | 0.002873 | 0.0082 | 0.844006 | 0.144921 | 0 | 0 | 0 | 0 | 0 |
| 624 | 0.002514 | 0.008718 | 0.843333 | 0.145435 | 0 | 0 | 0 | 0 | 0 |
| 625 | 0.003007 | 0.008076 | 0.844858 | 0.144018 | 0 | 0 | 0 | 0 | 0 |
| 626 | 0.002935 | 0.008344 | 0.842632 | 0.14609 | 0 | 0 | 0 | 0 | 0 |
| 627 | 0.00295 | 0.007897 | 0.843377 | 0.145776 | 0 | 0 | 0 | 0 | 0 |
| 628 | 0.002796 | 0.00828 | 0.843357 | 0.145567 | 0 | 0 | 0 | 0 | 0 |
| 629 | 0.003247 | 0.007757 | 0.844566 | 0.144431 | 0 | 0 | 0 | 0 | 0 |
| 630 | 0.003099 | 0.007874 | 0.840062 | 0.148964 | 0 | 0 | 0 | 0 | 0 |
| 631 | 0.002915 | 0.008226 | 0.84237 | 0.146488 | 0 | 0 | 0 | 0 | 0 |
| 632 | 0.00288 | 0.008368 | 0.846451 | 0.142301 | 0 | 0 | 0 | 0 | 0 |
| 633 | 0.002824 | 0.007225 | 0.84317 | 0.146781 | 0 | 0 | 0 | 0 | 0 |
| 634 | 0.002647 | 0.007812 | 0.843166 | 0.146374 | 0 | 0 | 0 | 0 | 0 |
| 635 | 0.003129 | 0.008115 | 0.843373 | 0.145373 | 0 | 0 | 0 | 0 | 0 |
| 636 | 0.003043 | 0.007552 | 0.842397 | 0.147007 | 0 | 0 | 0 | 0 | 0 |
| 637 | 0.002799 | 0.007886 | 0.842492 | 0.146824 | 0 | 0 | 0 | 0 | 0 |
| 638 | 0.002763 | 0.008178 | 0.84397 | 0.14509 | 0 | 0 | 0 | 0 | 0 |
| 639 | 0.003028 | 0.008302 | 0.839389 | 0.149281 | 0 | 0 | 0 | 0 | 0 |
| 640 | 0.002917 | 0.008114 | 0.839236 | 0.149733 | 0 | 0 | 0 | 0 | 0 |
| 641 | 0.002808 | 0.008223 | 0.843877 | 0.145092 | 0 | 0 | 0 | 0 | 0 |
| 642 | 0.003218 | 0.00823 | 0.843854 | 0.144697 | 0 | 0 | 0 | 0 | 0 |
| 643 | 0.002897 | 0.007868 | 0.842911 | 0.146324 | 0 | 0 | 0 | 0 | 0 |
| 644 | 0.002917 | 0.008232 | 0.848923 | 0.139929 | 0 | 0 | 0 | 0 | 0 |
| 645 | 0.002923 | 0.007788 | 0.843269 | 0.146009 | 0 | 0 | 0 | 0 | 0 |
| 646 | 0.002785 | 0.008183 | 0.841935 | 0.147098 | 0 | 0 | 0 | 0 | 0 |
| 647 | 0.002961 | 0.008312 | 0.844121 | 0.144606 | 0 | 0 | 0 | 0 | 0 |
| 648 | 0.003 | 0.009128 | 0.842973 | 0.144899 | 0 | 0 | 0 | 0 | 0 |
| 649 | 0.003 | 0.00862 | 0.843766 | 0.144614 | 0 | 0 | 0 | 0 | 0 |
| 650 | 0.003045 | 0.008186 | 0.845194 | 0.143576 | 0 | 0 | 0 | 0 | 0 |
| 651 | 0.003031 | 0.008213 | 0.843844 | 0.144911 | 0 | 0 | 0 | 0 | 0 |
| 652 | 0.002941 | 0.00821 | 0.843168 | 0.145682 | 0 | 0 | 0 | 0 | 0 |
| 653 | 0.003132 | 0.00794 | 0.845125 | 0.143802 | 0 | 0 | 0 | 0 | 0 |
| 654 | 0.003155 | 0.008443 | 0.841749 | 0.146643 | 0 | 0 | 0 | 0 | 0 |
| 655 | 0.002935 | 0.008373 | 0.840974 | 0.147718 | 0 | 0 | 0 | 0 | 0 |
| 656 | 0.002932 | 0.00819 | 0.847165 | 0.141713 | 0 | 0 | 0 | 0 | 0 |
| 657 | 0.002787 | 0.008402 | 0.845428 | 0.143383 | 0 | 0 | 0 | 0 | 0 |
| 658 | 0.002728 | 0.008335 | 0.840583 | 0.148354 | 0 | 0 | 0 | 0 | 0 |
| 659 | 0.00274 | 0.008971 | 0.842459 | 0.14583 | 0 | 0 | 0 | 0 | 0 |
| 660 | 0.003158 | 0.008519 | 0.846063 | 0.142261 | 0 | 0 | 0 | 0 | 0 |
| 661 | 0.002829 | 0.008636 | 0.842344 | 0.146191 | 0 | 0 | 0 | 0 | 0 |
| 662 | 0.002911 | 0.008642 | 0.842301 | 0.146147 | 0 | 0 | 0 | 0 | 0 |
| 663 | 0.002894 | 0.008593 | 0.843414 | 0.145099 | 0 | 0 | 0 | 0 | 0 |
| 664 | 0.002906 | 0.008467 | 0.847292 | 0.141326 | 0 | 0 | 0 | 0 | 0 |
| 665 | 0.002832 | 0.008187 | 0.8396 | 0.149381 | 0 | 0 | 0 | 0 | 0 |
| 666 | 0.002906 | 0.008048 | 0.843091 | 0.145955 | 0 | 0 | 0 | 0 | 0 |
| 667 | 0.002848 | 0.008424 | 0.84338 | 0.145348 | 0 | 0 | 0 | 0 | 0 |
| 668 | 0.002922 | 0.008075 | 0.840534 | 0.148469 | 0 | 0 | 0 | 0 | 0 |
| 669 | 0.00286 | 0.00873 | 0.840696 | 0.147714 | 0 | 0 | 0 | 0 | 0 |
| 670 | 0.002853 | 0.007506 | 0.841014 | 0.148626 | 0 | 0 | 0 | 0 | 0 |
| 671 | 0.002805 | 0.008326 | 0.84124 | 0.147629 | 0 | 0 | 0 | 0 | 0 |
| 672 | 0.002929 | 0.008578 | 0.839493 | 0.149 | 0 | 0 | 0 | 0 | 0 |
| 673 | 0.002799 | 0.008874 | 0.843964 | 0.144364 | 0 | 0 | 0 | 0 | 0 |
| 674 | 0.002871 | 0.008064 | 0.842945 | 0.14612 | 0 | 0 | 0 | 0 | 0 |
| 675 | 0.002916 | 0.008148 | 0.843325 | 0.14561 | 0 | 0 | 0 | 0 | 0 |
| 676 | 0.003033 | 0.008839 | 0.842807 | 0.145321 | 0 | 0 | 0 | 0 | 0 |
| 677 | 0.002813 | 0.007666 | 0.843603 | 0.145917 | 0 | 0 | 0 | 0 | 0 |
| 678 | 0.002861 | 0.007882 | 0.841874 | 0.147384 | 0 | 0 | 0 | 0 | 0 |
| 679 | 0.003212 | 0.008395 | 0.842233 | 0.14616 | 0 | 0 | 0 | 0 | 0 |
| 680 | 0.00276 | 0.008512 | 0.839041 | 0.149687 | 0 | 0 | 0 | 0 | 0 |
| 681 | 0.002838 | 0.008313 | 0.840226 | 0.148583 | 0 | 0 | 0 | 0 | 0 |
| 682 | 0.002917 | 0.007691 | 0.842883 | 0.146509 | 0 | 0 | 0 | 0 | 0 |
| 683 | 0.002722 | 0.008512 | 0.847074 | 0.141692 | 0 | 0 | 0 | 0 | 0 |
| 684 | 0.002759 | 0.00763 | 0.84437 | 0.145241 | 0 | 0 | 0 | 0 | 0 |
| 685 | 0.002842 | 0.008387 | 0.845969 | 0.142801 | 0 | 0 | 0 | 0 | 0 |
| 686 | 0.002575 | 0.007895 | 0.839064 | 0.150406 | 0 | 0 | 0 | 0 | 0 |
| 687 | 0.003007 | 0.00856 | 0.842347 | 0.146086 | 0 | 0 | 0 | 0 | 0 |
| 688 | 0.003086 | 0.007574 | 0.843522 | 0.145818 | 0 | 0 | 0 | 0 | 0 |
| 689 | 0.00258 | 0.008665 | 0.841953 | 0.146803 | 0 | 0 | 0 | 0 | 0 |
| 690 | 0.002734 | 0.008232 | 0.845573 | 0.143461 | 0 | 0 | 0 | 0 | 0 |
| 691 | 0.003068 | 0.00842 | 0.843758 | 0.144754 | 0 | 0 | 0 | 0 | 0 |
| 692 | 0.002669 | 0.008578 | 0.843328 | 0.145425 | 0 | 0 | 0 | 0 | 0 |
| 693 | 0.003256 | 0.008003 | 0.838746 | 0.149996 | 0 | 0 | 0 | 0 | 0 |
| 694 | 0.002735 | 0.00887 | 0.843221 | 0.145175 | 0 | 0 | 0 | 0 | 0 |
| 695 | 0.003007 | 0.007886 | 0.843632 | 0.145476 | 0 | 0 | 0 | 0 | 0 |
| 696 | 0.00309 | 0.00787 | 0.846846 | 0.142194 | 0 | 0 | 0 | 0 | 0 |
| 697 | 0.002955 | 0.008231 | 0.844479 | 0.144336 | 0 | 0 | 0 | 0 | 0 |
| 698 | 0.002911 | 0.008352 | 0.841581 | 0.147156 | 0 | 0 | 0 | 0 | 0 |
| 699 | 0.002874 | 0.008013 | 0.845276 | 0.143838 | 0 | 0 | 0 | 0 | 0 |
| 700 | 0.00288 | 0.008978 | 0.843181 | 0.144961 | 0 | 0 | 0 | 0 | 0 |
| 701 | 0.003077 | 0.008551 | 0.846358 | 0.142014 | 0 | 0 | 0 | 0 | 0 |
| 702 | 0.003393 | 0.008638 | 0.844914 | 0.143054 | 0 | 0 | 0 | 0 | 0 |
| 703 | 0.002781 | 0.008784 | 0.843876 | 0.144559 | 0 | 0 | 0 | 0 | 0 |
| 704 | 0.002998 | 0.007521 | 0.844299 | 0.145181 | 0 | 0 | 0 | 0 | 0 |
| 705 | 0.002657 | 0.00796 | 0.843377 | 0.146006 | 0 | 0 | 0 | 0 | 0 |
| 706 | 0.002966 | 0.008532 | 0.844433 | 0.144069 | 0 | 0 | 0 | 0 | 0 |
| 707 | 0.002604 | 0.00836 | 0.841806 | 0.14723 | 0 | 0 | 0 | 0 | 0 |
| 708 | 0.003072 | 0.008601 | 0.843563 | 0.144763 | 0 | 0 | 0 | 0 | 0 |
| 709 | 0.003139 | 0.008288 | 0.846594 | 0.141979 | 0 | 0 | 0 | 0 | 0 |
| 710 | 0.002712 | 0.008406 | 0.84217 | 0.146712 | 0 | 0 | 0 | 0 | 0 |
| 711 | 0.002596 | 0.008591 | 0.843841 | 0.144971 | 0 | 0 | 0 | 0 | 0 |
| 712 | 0.003081 | 0.008562 | 0.848221 | 0.140136 | 0 | 0 | 0 | 0 | 0 |
| 713 | 0.0027 | 0.00818 | 0.842651 | 0.146449 | 0 | 0 | 0 | 0 | 0 |
| 714 | 0.002627 | 0.008493 | 0.844121 | 0.144758 | 0 | 0 | 0 | 0 | 0 |
| 715 | 0.003135 | 0.008234 | 0.839724 | 0.148907 | 0 | 0 | 0 | 0 | 0 |
| 716 | 0.002854 | 0.008692 | 0.838375 | 0.150079 | 0 | 0 | 0 | 0 | 0 |
| 717 | 0.003091 | 0.007824 | 0.843458 | 0.145627 | 0 | 0 | 0 | 0 | 0 |
| 718 | 0.002909 | 0.008107 | 0.845305 | 0.14368 | 0 | 0 | 0 | 0 | 0 |
| 719 | 0.002794 | 0.007823 | 0.844529 | 0.144854 | 0 | 0 | 0 | 0 | 0 |
| 720 | 0.002896 | 0.008426 | 0.840194 | 0.148435 | 0 | 0 | 0 | 0 | 0 |
| 721 | 0.002764 | 0.008453 | 0.83968 | 0.149103 | 0 | 0 | 0 | 0 | 0 |
| 722 | 0.003053 | 0.00841 | 0.84185 | 0.146687 | 0 | 0 | 0 | 0 | 0 |
| 723 | 0.002966 | 0.008448 | 0.841591 | 0.146995 | 0 | 0 | 0 | 0 | 0 |
| 724 | 0.003007 | 0.008496 | 0.844391 | 0.144106 | 0 | 0 | 0 | 0 | 0 |
| 725 | 0.003191 | 0.008663 | 0.84004 | 0.148106 | 0 | 0 | 0 | 0 | 0 |
| 726 | 0.003016 | 0.007894 | 0.844948 | 0.144141 | 0 | 0 | 0 | 0 | 0 |
| 727 | 0.002785 | 0.008326 | 0.841638 | 0.14725 | 0 | 0 | 0 | 0 | 0 |
| 728 | 0.002861 | 0.008215 | 0.844853 | 0.14407 | 0 | 0 | 0 | 0 | 0 |
| 729 | 0.00279 | 0.008881 | 0.843221 | 0.145108 | 0 | 0 | 0 | 0 | 0 |
| 730 | 0.002965 | 0.008624 | 0.842097 | 0.146314 | 0 | 0 | 0 | 0 | 0 |
| 731 | 0.002794 | 0.008163 | 0.844008 | 0.145035 | 0 | 0 | 0 | 0 | 0 |
| 732 | 0.002707 | 0.008579 | 0.843605 | 0.145109 | 0 | 0 | 0 | 0 | 0 |
| 733 | 0.003136 | 0.008097 | 0.845092 | 0.143675 | 0 | 0 | 0 | 0 | 0 |
| 734 | 0.003055 | 0.008387 | 0.841036 | 0.147521 | 0 | 0 | 0 | 0 | 0 |
| 735 | 0.002973 | 0.008418 | 0.847113 | 0.141486 | 0 | 0 | 0 | 0 | 0 |
| 736 | 0.003143 | 0.008553 | 0.84358 | 0.144724 | 0 | 0 | 0 | 0 | 0 |
| 737 | 0.002712 | 0.008205 | 0.845791 | 0.143292 | 0 | 0 | 0 | 0 | 0 |
| 738 | 0.002769 | 0.007578 | 0.84198 | 0.147673 | 0 | 0 | 0 | 0 | 0 |
| 739 | 0.002668 | 0.007611 | 0.841397 | 0.148324 | 0 | 0 | 0 | 0 | 0 |
| 740 | 0.002844 | 0.008204 | 0.84777 | 0.141162 | 0 | 0 | 0 | 0 | 0 |
| 741 | 0.002858 | 0.008225 | 0.843424 | 0.145493 | 0 | 0 | 0 | 0 | 0 |
| 742 | 0.00273 | 0.008058 | 0.844656 | 0.144556 | 0 | 0 | 0 | 0 | 0 |
| 743 | 0.002978 | 0.008409 | 0.841815 | 0.146797 | 0 | 0 | 0 | 0 | 0 |
| 744 | 0.002991 | 0.008812 | 0.844286 | 0.14386 | 0 | 0 | 0 | 0 | 0 |
| 745 | 0.003085 | 0.008198 | 0.846246 | 0.142451 | 0 | 0 | 0 | 0 | 0 |
| 746 | 0.003023 | 0.007943 | 0.841854 | 0.14718 | 0 | 0 | 0 | 0 | 0 |
| 747 | 0.003002 | 0.008666 | 0.841182 | 0.147151 | 0 | 0 | 0 | 0 | 0 |
| 748 | 0.002935 | 0.007984 | 0.847042 | 0.142039 | 0 | 0 | 0 | 0 | 0 |
| 749 | 0.002941 | 0.00767 | 0.845883 | 0.143506 | 0 | 0 | 0 | 0 | 0 |
| 750 | 0.002974 | 0.00822 | 0.846506 | 0.142301 | 0 | 0 | 0 | 0 | 0 |
| 751 | 0.002784 | 0.008161 | 0.845825 | 0.14323 | 0 | 0 | 0 | 0 | 0 |
| 752 | 0.002928 | 0.008814 | 0.842107 | 0.146151 | 0 | 0 | 0 | 0 | 0 |
| 753 | 0.002664 | 0.007542 | 0.847655 | 0.142139 | 0 | 0 | 0 | 0 | 0 |
| 754 | 0.002868 | 0.008564 | 0.844651 | 0.143916 | 0 | 0 | 0 | 0 | 0 |
| 755 | 0.002728 | 0.008066 | 0.843461 | 0.145745 | 0 | 0 | 0 | 0 | 0 |
| 756 | 0.002937 | 0.008291 | 0.84112 | 0.147652 | 0 | 0 | 0 | 0 | 0 |
| 757 | 0.002696 | 0.008038 | 0.841645 | 0.147621 | 0 | 0 | 0 | 0 | 0 |
| 758 | 0.002883 | 0.008258 | 0.842626 | 0.146233 | 0 | 0 | 0 | 0 | 0 |
| 759 | 0.002904 | 0.00793 | 0.842246 | 0.14692 | 0 | 0 | 0 | 0 | 0 |
| 760 | 0.003004 | 0.008426 | 0.841863 | 0.146707 | 0 | 0 | 0 | 0 | 0 |
| 761 | 0.003157 | 0.008142 | 0.842061 | 0.14664 | 0 | 0 | 0 | 0 | 0 |
| 762 | 0.003019 | 0.007533 | 0.844163 | 0.145284 | 0 | 0 | 0 | 0 | 0 |
| 763 | 0.002992 | 0.009217 | 0.842786 | 0.145006 | 0 | 0 | 0 | 0 | 0 |
| 764 | 0.003234 | 0.00825 | 0.843335 | 0.145182 | 0 | 0 | 0 | 0 | 0 |
| 765 | 0.002954 | 0.008131 | 0.842097 | 0.146817 | 0 | 0 | 0 | 0 | 0 |
| 766 | 0.003216 | 0.008345 | 0.843534 | 0.144905 | 0 | 0 | 0 | 0 | 0 |
| 767 | 0.002939 | 0.00767 | 0.848102 | 0.141288 | 0 | 0 | 0 | 0 | 0 |
| 768 | 0.002824 | 0.008173 | 0.842899 | 0.146103 | 0 | 0 | 0 | 0 | 0 |
| 769 | 0.002913 | 0.007842 | 0.847812 | 0.141432 | 0 | 0 | 0 | 0 | 0 |
| 770 | 0.002938 | 0.007624 | 0.844706 | 0.144731 | 0 | 0 | 0 | 0 | 0 |
| 771 | 0.00285 | 0.008262 | 0.843367 | 0.145472 | 0 | 0 | 0 | 0 | 0 |
| 772 | 0.002803 | 0.008009 | 0.840803 | 0.148365 | 0 | 0 | 0 | 0 | 0 |
| 773 | 0.002818 | 0.008265 | 0.842035 | 0.146882 | 0 | 0 | 0 | 0 | 0 |
| 774 | 0.003009 | 0.008455 | 0.841951 | 0.146585 | 0 | 0 | 0 | 0 | 0 |
| 775 | 0.002674 | 0.008163 | 0.843246 | 0.145916 | 0 | 0 | 0 | 0 | 0 |
| 776 | 0.003011 | 0.008126 | 0.843625 | 0.145237 | 0 | 0 | 0 | 0 | 0 |
| 777 | 0.002682 | 0.008807 | 0.842207 | 0.146304 | 0 | 0 | 0 | 0 | 0 |
| 778 | 0.002905 | 0.008576 | 0.8452 | 0.143319 | 0 | 0 | 0 | 0 | 0 |
| 779 | 0.003023 | 0.007973 | 0.848306 | 0.140698 | 0 | 0 | 0 | 0 | 0 |
| 780 | 0.002843 | 0.007941 | 0.845747 | 0.143469 | 0 | 0 | 0 | 0 | 0 |
| 781 | 0.002972 | 0.008258 | 0.841199 | 0.14757 | 0 | 0 | 0 | 0 | 0 |
| 782 | 0.003021 | 0.008272 | 0.845366 | 0.143342 | 0 | 0 | 0 | 0 | 0 |
| 783 | 0.002997 | 0.008641 | 0.843924 | 0.144437 | 0 | 0 | 0 | 0 | 0 |
| 784 | 0.003033 | 0.008144 | 0.842303 | 0.146519 | 0 | 0 | 0 | 0 | 0 |
| 785 | 0.002664 | 0.007652 | 0.842636 | 0.147048 | 0 | 0 | 0 | 0 | 0 |
| 786 | 0.002773 | 0.007881 | 0.841773 | 0.147572 | 0 | 0 | 0 | 0 | 0 |
| 787 | 0.003144 | 0.008343 | 0.847382 | 0.141131 | 0 | 0 | 0 | 0 | 0 |
| 788 | 0.002666 | 0.008179 | 0.845438 | 0.143716 | 0 | 0 | 0 | 0 | 0 |
| 789 | 0.003117 | 0.008122 | 0.841301 | 0.14746 | 0 | 0 | 0 | 0 | 0 |
| 790 | 0.002582 | 0.008388 | 0.842849 | 0.146182 | 0 | 0 | 0 | 0 | 0 |
| 791 | 0.00266 | 0.008268 | 0.845565 | 0.143507 | 0 | 0 | 0 | 0 | 0 |
| 792 | 0.003565 | 0.008189 | 0.843226 | 0.14502 | 0 | 0 | 0 | 0 | 0 |
| 793 | 0.002895 | 0.008404 | 0.84222 | 0.146482 | 0 | 0 | 0 | 0 | 0 |
| 794 | 0.002917 | 0.00831 | 0.843969 | 0.144803 | 0 | 0 | 0 | 0 | 0 |
| 795 | 0.002778 | 0.008094 | 0.841364 | 0.147765 | 0 | 0 | 0 | 0 | 0 |
| 796 | 0.003043 | 0.008588 | 0.842137 | 0.146232 | 0 | 0 | 0 | 0 | 0 |
| 797 | 0.002931 | 0.008359 | 0.844519 | 0.144191 | 0 | 0 | 0 | 0 | 0 |
| 798 | 0.002639 | 0.007798 | 0.844082 | 0.145451 | 0 | 0 | 0 | 0 | 0 |
| 799 | 0.002754 | 0.008032 | 0.844376 | 0.144839 | 0 | 0 | 0 | 0 | 0 |
| 800 | 0.002916 | 0.00823 | 0.840781 | 0.148073 | 0 | 0 | 0 | 0 | 0 |
| 801 | 0.002761 | 0.007914 | 0.846875 | 0.14244 | 0 | 0 | 0 | 0 | 0 |
| 802 | 0.003057 | 0.00893 | 0.84105 | 0.146963 | 0 | 0 | 0 | 0 | 0 |
| 803 | 0.00281 | 0.00807 | 0.845745 | 0.143374 | 0 | 0 | 0 | 0 | 0 |
| 804 | 0.002925 | 0.008455 | 0.842054 | 0.146566 | 0 | 0 | 0 | 0 | 0 |
| 805 | 0.003085 | 0.007938 | 0.846768 | 0.14221 | 0 | 0 | 0 | 0 | 0 |
| 806 | 0.002832 | 0.00876 | 0.841873 | 0.146535 | 0 | 0 | 0 | 0 | 0 |
| 807 | 0.003168 | 0.008965 | 0.843271 | 0.144596 | 0 | 0 | 0 | 0 | 0 |
| 808 | 0.003203 | 0.007966 | 0.843316 | 0.145515 | 0 | 0 | 0 | 0 | 0 |
| 809 | 0.002829 | 0.008246 | 0.842631 | 0.146295 | 0 | 0 | 0 | 0 | 0 |
| 810 | 0.003111 | 0.008047 | 0.841031 | 0.147811 | 0 | 0 | 0 | 0 | 0 |
| 811 | 0.002739 | 0.008046 | 0.845753 | 0.143463 | 0 | 0 | 0 | 0 | 0 |
| 812 | 0.003357 | 0.008123 | 0.848208 | 0.140312 | 0 | 0 | 0 | 0 | 0 |
| 813 | 0.003057 | 0.008055 | 0.846622 | 0.142267 | 0 | 0 | 0 | 0 | 0 |
| 814 | 0.003203 | 0.008247 | 0.84343 | 0.14512 | 0 | 0 | 0 | 0 | 0 |
| 815 | 0.002829 | 0.007893 | 0.842211 | 0.147067 | 0 | 0 | 0 | 0 | 0 |
| 816 | 0.003171 | 0.007842 | 0.846393 | 0.142594 | 0 | 0 | 0 | 0 | 0 |
| 817 | 0.002485 | 0.008336 | 0.845031 | 0.144148 | 0 | 0 | 0 | 0 | 0 |
| 818 | 0.002957 | 0.008291 | 0.841636 | 0.147117 | 0 | 0 | 0 | 0 | 0 |
| 819 | 0.002587 | 0.008211 | 0.838497 | 0.150706 | 0 | 0 | 0 | 0 | 0 |
| 820 | 0.002634 | 0.008534 | 0.845449 | 0.143382 | 0 | 0 | 0 | 0 | 0 |
| 821 | 0.002894 | 0.008066 | 0.847561 | 0.141479 | 0 | 0 | 0 | 0 | 0 |
| 822 | 0.002871 | 0.008221 | 0.844524 | 0.144384 | 0 | 0 | 0 | 0 | 0 |
| 823 | 0.003333 | 0.008032 | 0.841979 | 0.146656 | 0 | 0 | 0 | 0 | 0 |
| 824 | 0.003109 | 0.008799 | 0.844511 | 0.143581 | 0 | 0 | 0 | 0 | 0 |
| 825 | 0.002667 | 0.008649 | 0.842257 | 0.146428 | 0 | 0 | 0 | 0 | 0 |
| 826 | 0.00282 | 0.007687 | 0.841824 | 0.147669 | 0 | 0 | 0 | 0 | 0 |
| 827 | 0.00288 | 0.0087 | 0.841278 | 0.147141 | 0 | 0 | 0 | 0 | 0 |
| 828 | 0.003175 | 0.007462 | 0.846317 | 0.143046 | 0 | 0 | 0 | 0 | 0 |
| 829 | 0.002942 | 0.008408 | 0.846554 | 0.142096 | 0 | 0 | 0 | 0 | 0 |
| 830 | 0.003001 | 0.008784 | 0.84449 | 0.143725 | 0 | 0 | 0 | 0 | 0 |
| 831 | 0.00264 | 0.008099 | 0.841896 | 0.147366 | 0 | 0 | 0 | 0 | 0 |
| 832 | 0.002917 | 0.008892 | 0.843744 | 0.144447 | 0 | 0 | 0 | 0 | 0 |
| 833 | 0.003056 | 0.008373 | 0.841614 | 0.146958 | 0 | 0 | 0 | 0 | 0 |
| 834 | 0.003124 | 0.008939 | 0.841772 | 0.146146 | 0 | 0 | 0 | 0 | 0 |
| 835 | 0.00281 | 0.007841 | 0.843288 | 0.14606 | 0 | 0 | 0 | 0 | 0 |
| 836 | 0.002807 | 0.008521 | 0.844873 | 0.143799 | 0 | 0 | 0 | 0 | 0 |
| 837 | 0.002978 | 0.008496 | 0.845909 | 0.142617 | 0 | 0 | 0 | 0 | 0 |
| 838 | 0.003045 | 0.008297 | 0.845092 | 0.143556 | 0 | 0 | 0 | 0 | 0 |
| 839 | 0.002809 | 0.008327 | 0.842032 | 0.146832 | 0 | 0 | 0 | 0 | 0 |
| 840 | 0.002902 | 0.008527 | 0.838554 | 0.150017 | 0 | 0 | 0 | 0 | 0 |
| 841 | 0.003076 | 0.008386 | 0.847102 | 0.141436 | 0 | 0 | 0 | 0 | 0 |
| 842 | 0.002735 | 0.008194 | 0.840692 | 0.148379 | 0 | 0 | 0 | 0 | 0 |
| 843 | 0.002964 | 0.008821 | 0.843336 | 0.144878 | 0 | 0 | 0 | 0 | 0 |
| 844 | 0.003085 | 0.009166 | 0.842867 | 0.144881 | 0 | 0 | 0 | 0 | 0 |
| 845 | 0.002874 | 0.008312 | 0.839145 | 0.14967 | 0 | 0 | 0 | 0 | 0 |
| 846 | 0.002746 | 0.008676 | 0.840813 | 0.147765 | 0 | 0 | 0 | 0 | 0 |
| 847 | 0.003226 | 0.008383 | 0.841826 | 0.146565 | 0 | 0 | 0 | 0 | 0 |
| 848 | 0.002797 | 0.00855 | 0.845123 | 0.143529 | 0 | 0 | 0 | 0 | 0 |
| 849 | 0.003397 | 0.008407 | 0.843922 | 0.144264 | 0 | 0 | 0 | 0 | 0 |
| 850 | 0.002989 | 0.008076 | 0.841457 | 0.147479 | 0 | 0 | 0 | 0 | 0 |
| 851 | 0.00315 | 0.008447 | 0.841478 | 0.146905 | 0 | 0 | 0 | 0 | 0 |
| 852 | 0.002933 | 0.00797 | 0.84273 | 0.146367 | 0 | 0 | 0 | 0 | 0 |
| 853 | 0.003089 | 0.008212 | 0.839822 | 0.148876 | 0 | 0 | 0 | 0 | 0 |
| 854 | 0.002791 | 0.008312 | 0.841092 | 0.147804 | 0 | 0 | 0 | 0 | 0 |
| 855 | 0.002492 | 0.008846 | 0.842378 | 0.146285 | 0 | 0 | 0 | 0 | 0 |
| 856 | 0.003171 | 0.008566 | 0.839251 | 0.149012 | 0 | 0 | 0 | 0 | 0 |
| 857 | 0.003229 | 0.008475 | 0.840075 | 0.148221 | 0 | 0 | 0 | 0 | 0 |
| 858 | 0.002902 | 0.008496 | 0.843132 | 0.14547 | 0 | 0 | 0 | 0 | 0 |
| 859 | 0.002966 | 0.008188 | 0.844999 | 0.143847 | 0 | 0 | 0 | 0 | 0 |
| 860 | 0.002834 | 0.008383 | 0.841601 | 0.147182 | 0 | 0 | 0 | 0 | 0 |
| 861 | 0.003036 | 0.007944 | 0.84572 | 0.143289 | 0 | 0 | 0 | 0 | 0 |
| 862 | 0.002799 | 0.007762 | 0.84411 | 0.145329 | 0 | 0 | 0 | 0 | 0 |
| 863 | 0.003249 | 0.008568 | 0.842072 | 0.146111 | 0 | 0 | 0 | 0 | 0 |
| 864 | 0.002894 | 0.008642 | 0.84188 | 0.146584 | 0 | 0 | 0 | 0 | 0 |
| 865 | 0.002821 | 0.008076 | 0.843662 | 0.145441 | 0 | 0 | 0 | 0 | 0 |
| 866 | 0.003205 | 0.008098 | 0.843866 | 0.144831 | 0 | 0 | 0 | 0 | 0 |
| 867 | 0.003091 | 0.008379 | 0.83815 | 0.15038 | 0 | 0 | 0 | 0 | 0 |
| 868 | 0.002846 | 0.008357 | 0.840418 | 0.148379 | 0 | 0 | 0 | 0 | 0 |
| 869 | 0.003035 | 0.008046 | 0.843229 | 0.14568 | 0 | 0 | 0 | 0 | 0 |
| 870 | 0.003056 | 0.007815 | 0.840101 | 0.149029 | 0 | 0 | 0 | 0 | 0 |
| 871 | 0.003237 | 0.008017 | 0.848215 | 0.140531 | 0 | 0 | 0 | 0 | 0 |
| 872 | 0.002968 | 0.008232 | 0.840409 | 0.148391 | 0 | 0 | 0 | 0 | 0 |
| 873 | 0.00295 | 0.008369 | 0.842639 | 0.146041 | 0 | 0 | 0 | 0 | 0 |
| 874 | 0.002959 | 0.007664 | 0.844734 | 0.144643 | 0 | 0 | 0 | 0 | 0 |
| 875 | 0.003083 | 0.008219 | 0.845239 | 0.143459 | 0 | 0 | 0 | 0 | 0 |
| 876 | 0.002842 | 0.008287 | 0.842814 | 0.146057 | 0 | 0 | 0 | 0 | 0 |
| 877 | 0.002576 | 0.008312 | 0.844655 | 0.144457 | 0 | 0 | 0 | 0 | 0 |
| 878 | 0.003141 | 0.008098 | 0.843763 | 0.144999 | 0 | 0 | 0 | 0 | 0 |
| 879 | 0.003165 | 0.008625 | 0.840539 | 0.147671 | 0 | 0 | 0 | 0 | 0 |
| 880 | 0.002896 | 0.007755 | 0.846507 | 0.142833 | 0 | 0 | 0 | 0 | 0 |
| 881 | 0.002863 | 0.007743 | 0.840149 | 0.149244 | 0 | 0 | 0 | 0 | 0 |
| 882 | 0.002626 | 0.008489 | 0.842487 | 0.146388 | 0 | 0 | 0 | 0 | 0 |
| 883 | 0.003041 | 0.008073 | 0.839727 | 0.149149 | 0 | 0 | 0 | 0 | 0 |
| 884 | 0.003078 | 0.008602 | 0.84115 | 0.147171 | 0 | 0 | 0 | 0 | 0 |
| 885 | 0.002457 | 0.00795 | 0.845369 | 0.144224 | 0 | 0 | 0 | 0 | 0 |
| 886 | 0.002665 | 0.008436 | 0.840996 | 0.147902 | 0 | 0 | 0 | 0 | 0 |
| 887 | 0.002699 | 0.007831 | 0.844849 | 0.14462 | 0 | 0 | 0 | 0 | 0 |
| 888 | 0.002861 | 0.008444 | 0.838615 | 0.15008 | 0 | 0 | 0 | 0 | 0 |
| 889 | 0.002917 | 0.008301 | 0.840104 | 0.148678 | 0 | 0 | 0 | 0 | 0 |
| 890 | 0.003286 | 0.008453 | 0.845418 | 0.142843 | 0 | 0 | 0 | 0 | 0 |
| 891 | 0.003085 | 0.008357 | 0.842833 | 0.145725 | 0 | 0 | 0 | 0 | 0 |
| 892 | 0.002859 | 0.008528 | 0.843622 | 0.144991 | 0 | 0 | 0 | 0 | 0 |
| 893 | 0.002979 | 0.008088 | 0.840618 | 0.148314 | 0 | 0 | 0 | 0 | 0 |
| 894 | 0.002917 | 0.008502 | 0.84379 | 0.144791 | 0 | 0 | 0 | 0 | 0 |
| 895 | 0.002772 | 0.008027 | 0.848061 | 0.14114 | 0 | 0 | 0 | 0 | 0 |
| 896 | 0.002642 | 0.008425 | 0.845428 | 0.143505 | 0 | 0 | 0 | 0 | 0 |
| 897 | 0.002816 | 0.008587 | 0.844159 | 0.144438 | 0 | 0 | 0 | 0 | 0 |
| 898 | 0.002638 | 0.007674 | 0.841658 | 0.14803 | 0 | 0 | 0 | 0 | 0 |
| 899 | 0.003024 | 0.008254 | 0.842143 | 0.14658 | 0 | 0 | 0 | 0 | 0 |
| 900 | 0.002824 | 0.009084 | 0.842359 | 0.145733 | 0 | 0 | 0 | 0 | 0 |
| 901 | 0.002972 | 0.008378 | 0.845151 | 0.143498 | 0 | 0 | 0 | 0 | 0 |
| 902 | 0.003167 | 0.007824 | 0.845885 | 0.143104 | 0 | 0 | 0 | 0 | 0 |
| 903 | 0.002683 | 0.008729 | 0.845671 | 0.142917 | 0 | 0 | 0 | 0 | 0 |
| 904 | 0.00288 | 0.007709 | 0.845139 | 0.144273 | 0 | 0 | 0 | 0 | 0 |
| 905 | 0.003048 | 0.008414 | 0.84366 | 0.144878 | 0 | 0 | 0 | 0 | 0 |
| 906 | 0.002449 | 0.008275 | 0.847462 | 0.141815 | 0 | 0 | 0 | 0 | 0 |
| 907 | 0.002918 | 0.008502 | 0.843729 | 0.144851 | 0 | 0 | 0 | 0 | 0 |
| 908 | 0.002547 | 0.00916 | 0.843883 | 0.14441 | 0 | 0 | 0 | 0 | 0 |
| 909 | 0.002569 | 0.008486 | 0.843788 | 0.145157 | 0 | 0 | 0 | 0 | 0 |
| 910 | 0.002826 | 0.008266 | 0.843072 | 0.145836 | 0 | 0 | 0 | 0 | 0 |
| 911 | 0.002914 | 0.008078 | 0.848353 | 0.140655 | 0 | 0 | 0 | 0 | 0 |
| 912 | 0.00292 | 0.008412 | 0.839116 | 0.149551 | 0 | 0 | 0 | 0 | 0 |
| 913 | 0.002959 | 0.00818 | 0.844967 | 0.143894 | 0 | 0 | 0 | 0 | 0 |
| 914 | 0.002808 | 0.008525 | 0.848543 | 0.140124 | 0 | 0 | 0 | 0 | 0 |
| 915 | 0.003224 | 0.008034 | 0.840301 | 0.148441 | 0 | 0 | 0 | 0 | 0 |
| 916 | 0.002763 | 0.008889 | 0.844218 | 0.14413 | 0 | 0 | 0 | 0 | 0 |
| 917 | 0.002721 | 0.007915 | 0.842401 | 0.146963 | 0 | 0 | 0 | 0 | 0 |
| 918 | 0.002783 | 0.007716 | 0.847362 | 0.14214 | 0 | 0 | 0 | 0 | 0 |
| 919 | 0.00252 | 0.00865 | 0.840839 | 0.147991 | 0 | 0 | 0 | 0 | 0 |
| 920 | 0.002537 | 0.008139 | 0.845316 | 0.144008 | 0 | 0 | 0 | 0 | 0 |
| 921 | 0.003097 | 0.007956 | 0.843185 | 0.145762 | 0 | 0 | 0 | 0 | 0 |
| 922 | 0.002814 | 0.007852 | 0.846762 | 0.142572 | 0 | 0 | 0 | 0 | 0 |
| 923 | 0.003031 | 0.007809 | 0.846457 | 0.142702 | 0 | 0 | 0 | 0 | 0 |
| 924 | 0.00305 | 0.00802 | 0.842789 | 0.146141 | 0 | 0 | 0 | 0 | 0 |
| 925 | 0.003 | 0.008002 | 0.844152 | 0.144836 | 0 | 0 | 0 | 0 | 0 |
| 926 | 0.003344 | 0.008668 | 0.845222 | 0.142766 | 0 | 0 | 0 | 0 | 0 |
| 927 | 0.003099 | 0.008409 | 0.844414 | 0.144078 | 0 | 0 | 0 | 0 | 0 |
| 928 | 0.002969 | 0.008062 | 0.842625 | 0.146343 | 0 | 0 | 0 | 0 | 0 |
| 929 | 0.002793 | 0.008029 | 0.839777 | 0.149401 | 0 | 0 | 0 | 0 | 0 |
| 930 | 0.002897 | 0.00888 | 0.845033 | 0.143191 | 0 | 0 | 0 | 0 | 0 |
| 931 | 0.003059 | 0.008199 | 0.845764 | 0.142978 | 0 | 0 | 0 | 0 | 0 |
| 932 | 0.002775 | 0.008596 | 0.840976 | 0.147653 | 0 | 0 | 0 | 0 | 0 |
| 933 | 0.003285 | 0.008227 | 0.846909 | 0.14158 | 0 | 0 | 0 | 0 | 0 |
| 934 | 0.002941 | 0.008505 | 0.842134 | 0.14642 | 0 | 0 | 0 | 0 | 0 |
| 935 | 0.003041 | 0.008752 | 0.843436 | 0.144771 | 0 | 0 | 0 | 0 | 0 |
| 936 | 0.002873 | 0.008346 | 0.843476 | 0.145305 | 0 | 0 | 0 | 0 | 0 |
| 937 | 0.002675 | 0.008064 | 0.848151 | 0.14111 | 0 | 0 | 0 | 0 | 0 |
| 938 | 0.002718 | 0.008144 | 0.845893 | 0.143224 | 0 | 0 | 0 | 0 | 0 |
| 939 | 0.002965 | 0.007798 | 0.848529 | 0.140708 | 0 | 0 | 0 | 0 | 0 |
| 940 | 0.003144 | 0.008129 | 0.84085 | 0.147877 | 0 | 0 | 0 | 0 | 0 |
| 941 | 0.002905 | 0.008445 | 0.842532 | 0.146118 | 0 | 0 | 0 | 0 | 0 |
| 942 | 0.003091 | 0.008648 | 0.843707 | 0.144554 | 0 | 0 | 0 | 0 | 0 |
| 943 | 0.002963 | 0.008388 | 0.846608 | 0.142041 | 0 | 0 | 0 | 0 | 0 |
| 944 | 0.002825 | 0.008416 | 0.840631 | 0.148128 | 0 | 0 | 0 | 0 | 0 |
| 945 | 0.00313 | 0.008991 | 0.839742 | 0.148136 | 0 | 0 | 0 | 0 | 0 |
| 946 | 0.002917 | 0.008364 | 0.846693 | 0.142025 | 0 | 0 | 0 | 0 | 0 |
| 947 | 0.003224 | 0.008367 | 0.84362 | 0.144789 | 0 | 0 | 0 | 0 | 0 |
| 948 | 0.003039 | 0.008155 | 0.843893 | 0.144912 | 0 | 0 | 0 | 0 | 0 |
| 949 | 0.002619 | 0.007729 | 0.850116 | 0.139536 | 0 | 0 | 0 | 0 | 0 |
| 950 | 0.002863 | 0.008439 | 0.844722 | 0.143975 | 0 | 0 | 0 | 0 | 0 |
| 951 | 0.002983 | 0.008027 | 0.84631 | 0.14268 | 0 | 0 | 0 | 0 | 0 |
| 952 | 0.003009 | 0.008747 | 0.846056 | 0.142188 | 0 | 0 | 0 | 0 | 0 |
| 953 | 0.003043 | 0.008545 | 0.844143 | 0.144269 | 0 | 0 | 0 | 0 | 0 |
| 954 | 0.002912 | 0.008585 | 0.841189 | 0.147314 | 0 | 0 | 0 | 0 | 0 |
| 955 | 0.003107 | 0.00853 | 0.844043 | 0.14432 | 0 | 0 | 0 | 0 | 0 |
| 956 | 0.002787 | 0.008501 | 0.841224 | 0.147488 | 0 | 0 | 0 | 0 | 0 |
| 957 | 0.002869 | 0.009197 | 0.841945 | 0.145989 | 0 | 0 | 0 | 0 | 0 |
| 958 | 0.002402 | 0.0083 | 0.846807 | 0.142461 | 0 | 0 | 0 | 0 | 0 |
| 959 | 0.002786 | 0.008259 | 0.840564 | 0.14839 | 0 | 0 | 0 | 0 | 0 |
| 960 | 0.00271 | 0.00813 | 0.844483 | 0.144677 | 0 | 0 | 0 | 0 | 0 |
| 961 | 0.002697 | 0.007712 | 0.84492 | 0.144651 | 0 | 0 | 0 | 0 | 0 |
| 962 | 0.003003 | 0.00836 | 0.844641 | 0.143996 | 0 | 0 | 0 | 0 | 0 |
| 963 | 0.003526 | 0.007922 | 0.842982 | 0.14557 | 0 | 0 | 0 | 0 | 0 |
| 964 | 0.002792 | 0.007887 | 0.844206 | 0.145115 | 0 | 0 | 0 | 0 | 0 |
| 965 | 0.00328 | 0.008165 | 0.844897 | 0.143658 | 0 | 0 | 0 | 0 | 0 |
| 966 | 0.003004 | 0.008152 | 0.843675 | 0.145169 | 0 | 0 | 0 | 0 | 0 |
| 967 | 0.002627 | 0.00869 | 0.842626 | 0.146056 | 0 | 0 | 0 | 0 | 0 |
| 968 | 0.003011 | 0.008039 | 0.84304 | 0.145911 | 0 | 0 | 0 | 0 | 0 |
| 969 | 0.00297 | 0.008361 | 0.844339 | 0.14433 | 0 | 0 | 0 | 0 | 0 |
| 970 | 0.003085 | 0.008585 | 0.845211 | 0.143119 | 0 | 0 | 0 | 0 | 0 |
| 971 | 0.003351 | 0.00877 | 0.844713 | 0.143167 | 0 | 0 | 0 | 0 | 0 |
| 972 | 0.00279 | 0.008351 | 0.841916 | 0.146943 | 0 | 0 | 0 | 0 | 0 |
| 973 | 0.003328 | 0.008385 | 0.84302 | 0.145257 | 0 | 0 | 0 | 0 | 0 |
| 974 | 0.002718 | 0.008293 | 0.843881 | 0.145108 | 0 | 0 | 0 | 0 | 0 |
| 975 | 0.003099 | 0.008117 | 0.845137 | 0.143646 | 0 | 0 | 0 | 0 | 0 |
| 976 | 0.002972 | 0.007684 | 0.844571 | 0.144774 | 0 | 0 | 0 | 0 | 0 |
| 977 | 0.002777 | 0.007938 | 0.845019 | 0.144267 | 0 | 0 | 0 | 0 | 0 |
| 978 | 0.002944 | 0.0075 | 0.845045 | 0.144511 | 0 | 0 | 0 | 0 | 0 |
| 979 | 0.002855 | 0.007871 | 0.843003 | 0.146271 | 0 | 0 | 0 | 0 | 0 |
| 980 | 0.002847 | 0.007834 | 0.842751 | 0.146568 | 0 | 0 | 0 | 0 | 0 |
| 981 | 0.002927 | 0.007941 | 0.844612 | 0.1445 | 0 | 0 | 0 | 0 | 0 |
| 982 | 0.002842 | 0.008035 | 0.844096 | 0.145027 | 0 | 0 | 0 | 0 | 0 |
| 983 | 0.002829 | 0.008358 | 0.840936 | 0.147877 | 0 | 0 | 0 | 0 | 0 |
| 984 | 0.00253 | 0.008684 | 0.842784 | 0.146002 | 0 | 0 | 0 | 0 | 0 |
| 985 | 0.003397 | 0.008056 | 0.843243 | 0.145305 | 0 | 0 | 0 | 0 | 0 |
| 986 | 0.003091 | 0.008252 | 0.845174 | 0.143483 | 0 | 0 | 0 | 0 | 0 |
| 987 | 0.002966 | 0.00773 | 0.844987 | 0.144317 | 0 | 0 | 0 | 0 | 0 |
| 988 | 0.002915 | 0.008795 | 0.84218 | 0.146109 | 0 | 0 | 0 | 0 | 0 |
| 989 | 0.00299 | 0.008317 | 0.842002 | 0.146691 | 0 | 0 | 0 | 0 | 0 |
| 990 | 0.003136 | 0.00831 | 0.844303 | 0.144211 | 0 | 0 | 0 | 0 | 0 |
| 991 | 0.003099 | 0.008689 | 0.842265 | 0.145946 | 0 | 0 | 0 | 0 | 0 |
| 992 | 0.003017 | 0.008972 | 0.843588 | 0.144422 | 0 | 0 | 0 | 0 | 0 |
| 993 | 0.003138 | 0.00843 | 0.846093 | 0.142339 | 0 | 0 | 0 | 0 | 0 |
| 994 | 0.003064 | 0.008221 | 0.846795 | 0.141921 | 0 | 0 | 0 | 0 | 0 |
| 995 | 0.002722 | 0.008045 | 0.844357 | 0.144877 | 0 | 0 | 0 | 0 | 0 |
| 996 | 0.002757 | 0.00795 | 0.84499 | 0.144302 | 0 | 0 | 0 | 0 | 0 |
| 997 | 0.002739 | 0.008337 | 0.840888 | 0.148036 | 0 | 0 | 0 | 0 | 0 |
| 998 | 0.002663 | 0.007372 | 0.845624 | 0.144341 | 0 | 0 | 0 | 0 | 0 |
| 999 | 0.002999 | 0.008115 | 0.845935 | 0.14294 | 0 | 0 | 0 | 0 | 0 |
| 1000 | 0.002956 | 0.00788 | 0.841371 | 0.147792 | 0 | 0 | 0 | 0 | 0 |

| Year 4 | Race |  |  |  |  |  |  |  |  |
| --- | --- | --- | --- | --- | --- | --- | --- | --- | --- |
| Trial | 003.0 | 005.0 | 007.0 | 037.1 | 043.0 | 303.0 | 003.2 | 403.0 | 003.4 |
| 1 | 0.00041 | 0.003686 | 0.860727 | 0.135178 | 0 | 0 | 0 | 0 | 0 |
| 2 | 0.000377 | 0.003354 | 0.85739 | 0.13888 | 0 | 0 | 0 | 0 | 0 |
| 3 | 0.000559 | 0.002937 | 0.862015 | 0.134488 | 0 | 0 | 0 | 0 | 0 |
| 4 | 0.000524 | 0.002951 | 0.863841 | 0.132684 | 0 | 0 | 0 | 0 | 0 |
| 5 | 0.000318 | 0.003471 | 0.856928 | 0.139282 | 0 | 0 | 0 | 0 | 0 |
| 6 | 0.00032 | 0.002876 | 0.862488 | 0.134317 | 0 | 0 | 0 | 0 | 0 |
| 7 | 0.000411 | 0.003286 | 0.860544 | 0.135759 | 0 | 0 | 0 | 0 | 0 |
| 8 | 0.000411 | 0.002795 | 0.862026 | 0.134767 | 0 | 0 | 0 | 0 | 0 |
| 9 | 0.00049 | 0.002788 | 0.862258 | 0.134465 | 0 | 0 | 0 | 0 | 0 |
| 10 | 0.000381 | 0.003068 | 0.862455 | 0.134096 | 0 | 0 | 0 | 0 | 0 |
| 11 | 0.00037 | 0.00352 | 0.862336 | 0.133764 | 0 | 0 | 0 | 0 | 0 |
| 12 | 0.00052 | 0.00315 | 0.860579 | 0.13575 | 0 | 0 | 0 | 0 | 0 |
| 13 | 0.000439 | 0.003573 | 0.858097 | 0.137892 | 0 | 0 | 0 | 0 | 0 |
| 14 | 0.00043 | 0.003589 | 0.853702 | 0.142279 | 0 | 0 | 0 | 0 | 0 |
| 15 | 0.000428 | 0.003201 | 0.858486 | 0.137885 | 0 | 0 | 0 | 0 | 0 |
| 16 | 0.00042 | 0.003357 | 0.861538 | 0.134686 | 0 | 0 | 0 | 0 | 0 |
| 17 | 0.000423 | 0.003385 | 0.861394 | 0.134788 | 0 | 0 | 0 | 0 | 0 |
| 18 | 0.000432 | 0.003095 | 0.861349 | 0.135124 | 0 | 0 | 0 | 0 | 0 |
| 19 | 0.000493 | 0.003148 | 0.859338 | 0.137021 | 0 | 0 | 0 | 0 | 0 |
| 20 | 0.000413 | 0.003213 | 0.860319 | 0.136055 | 0 | 0 | 0 | 0 | 0 |
| 21 | 0.00046 | 0.003093 | 0.860953 | 0.135493 | 0 | 0 | 0 | 0 | 0 |
| 22 | 0.000388 | 0.003464 | 0.856659 | 0.139489 | 0 | 0 | 0 | 0 | 0 |
| 23 | 0.000441 | 0.00332 | 0.859741 | 0.136498 | 0 | 0 | 0 | 0 | 0 |
| 24 | 0.00037 | 0.003253 | 0.859392 | 0.136985 | 0 | 0 | 0 | 0 | 0 |
| 25 | 0.000471 | 0.002766 | 0.861573 | 0.13519 | 0 | 0 | 0 | 0 | 0 |
| 26 | 0.0003 | 0.003408 | 0.86634 | 0.129953 | 0 | 0 | 0 | 0 | 0 |
| 27 | 0.000291 | 0.002981 | 0.860893 | 0.135835 | 0 | 0 | 0 | 0 | 0 |
| 28 | 0.000321 | 0.003118 | 0.860821 | 0.13571 | 0 | 0 | 0 | 0 | 0 |
| 29 | 0.00042 | 0.003298 | 0.859775 | 0.136507 | 0 | 0 | 0 | 0 | 0 |
| 30 | 0.000409 | 0.003542 | 0.861985 | 0.134063 | 0 | 0 | 0 | 0 | 0 |
| 31 | 0.00036 | 0.002874 | 0.860816 | 0.13595 | 0 | 0 | 0 | 0 | 0 |
| 32 | 0.000488 | 0.002977 | 0.861372 | 0.135162 | 0 | 0 | 0 | 0 | 0 |
| 33 | 0.000493 | 0.003059 | 0.859639 | 0.136809 | 0 | 0 | 0 | 0 | 0 |
| 34 | 0.000359 | 0.003294 | 0.860447 | 0.1359 | 0 | 0 | 0 | 0 | 0 |
| 35 | 0.000511 | 0.002877 | 0.855413 | 0.141199 | 0 | 0 | 0 | 0 | 0 |
| 36 | 0.000541 | 0.003105 | 0.85825 | 0.138104 | 0 | 0 | 0 | 0 | 0 |
| 37 | 0.000398 | 0.003531 | 0.862121 | 0.13395 | 0 | 0 | 0 | 0 | 0 |
| 38 | 0.0004 | 0.00305 | 0.861926 | 0.134623 | 0 | 0 | 0 | 0 | 0 |
| 39 | 0.000381 | 0.002969 | 0.859963 | 0.136677 | 0 | 0 | 0 | 0 | 0 |
| 40 | 0.000328 | 0.003122 | 0.858788 | 0.137763 | 0 | 0 | 0 | 0 | 0 |
| 41 | 0.000501 | 0.003233 | 0.862134 | 0.134132 | 0 | 0 | 0 | 0 | 0 |
| 42 | 0.000393 | 0.003638 | 0.864536 | 0.131433 | 0 | 0 | 0 | 0 | 0 |
| 43 | 0.000552 | 0.002961 | 0.861865 | 0.134622 | 0 | 0 | 0 | 0 | 0 |
| 44 | 0.000447 | 0.00305 | 0.860062 | 0.136441 | 0 | 0 | 0 | 0 | 0 |
| 45 | 0.000348 | 0.003603 | 0.859472 | 0.136577 | 0 | 0 | 0 | 0 | 0 |
| 46 | 0.000428 | 0.003163 | 0.859355 | 0.137054 | 0 | 0 | 0 | 0 | 0 |
| 47 | 0.000381 | 0.003177 | 0.861185 | 0.135257 | 0 | 0 | 0 | 0 | 0 |
| 48 | 0.000359 | 0.003163 | 0.858545 | 0.137933 | 0 | 0 | 0 | 0 | 0 |
| 49 | 0.000541 | 0.003134 | 0.856659 | 0.139666 | 0 | 0 | 0 | 0 | 0 |
| 50 | 0.000309 | 0.002715 | 0.860706 | 0.136269 | 0 | 0 | 0 | 0 | 0 |
| 51 | 0.00045 | 0.00335 | 0.858824 | 0.137376 | 0 | 0 | 0 | 0 | 0 |
| 52 | 0.000568 | 0.003009 | 0.860472 | 0.135952 | 0 | 0 | 0 | 0 | 0 |
| 53 | 0.000361 | 0.002961 | 0.864779 | 0.131868 | 0 | 0 | 0 | 0 | 0 |
| 54 | 0.000539 | 0.002932 | 0.861288 | 0.135242 | 0 | 0 | 0 | 0 | 0 |
| 55 | 0.000479 | 0.003434 | 0.863047 | 0.133041 | 0 | 0 | 0 | 0 | 0 |
| 56 | 0.000318 | 0.003221 | 0.857452 | 0.139008 | 0 | 0 | 0 | 0 | 0 |
| 57 | 0.000578 | 0.003398 | 0.86222 | 0.133804 | 0 | 0 | 0 | 0 | 0 |
| 58 | 0.00039 | 0.003393 | 0.859063 | 0.137154 | 0 | 0 | 0 | 0 | 0 |
| 59 | 0.000424 | 0.003168 | 0.860173 | 0.136236 | 0 | 0 | 0 | 0 | 0 |
| 60 | 0.000452 | 0.003084 | 0.861684 | 0.13478 | 0 | 0 | 0 | 0 | 0 |
| 61 | 0.00041 | 0.003441 | 0.86112 | 0.135029 | 0 | 0 | 0 | 0 | 0 |
| 62 | 0.000458 | 0.003176 | 0.863746 | 0.13262 | 0 | 0 | 0 | 0 | 0 |
| 63 | 0.000409 | 0.003315 | 0.86501 | 0.131266 | 0 | 0 | 0 | 0 | 0 |
| 64 | 0.00037 | 0.003446 | 0.861061 | 0.135123 | 0 | 0 | 0 | 0 | 0 |
| 65 | 0.000483 | 0.002895 | 0.860367 | 0.136255 | 0 | 0 | 0 | 0 | 0 |
| 66 | 0.000538 | 0.003038 | 0.864133 | 0.132292 | 0 | 0 | 0 | 0 | 0 |
| 67 | 0.000432 | 0.002884 | 0.859643 | 0.137031 | 0 | 0 | 0 | 0 | 0 |
| 68 | 0.000567 | 0.002765 | 0.861531 | 0.135136 | 0 | 0 | 0 | 0 | 0 |
| 69 | 0.000539 | 0.002796 | 0.859458 | 0.137206 | 0 | 0 | 0 | 0 | 0 |
| 70 | 0.000299 | 0.003303 | 0.861958 | 0.13444 | 0 | 0 | 0 | 0 | 0 |
| 71 | 0.000431 | 0.003616 | 0.860139 | 0.135774 | 0 | 0 | 0 | 0 | 0 |
| 72 | 0.000429 | 0.003571 | 0.864614 | 0.131385 | 0 | 0 | 0 | 0 | 0 |
| 73 | 0.000428 | 0.003236 | 0.860206 | 0.13613 | 0 | 0 | 0 | 0 | 0 |
| 74 | 0.000279 | 0.003146 | 0.86213 | 0.134445 | 0 | 0 | 0 | 0 | 0 |
| 75 | 0.000501 | 0.003907 | 0.859191 | 0.136401 | 0 | 0 | 0 | 0 | 0 |
| 76 | 0.000519 | 0.003566 | 0.86187 | 0.134045 | 0 | 0 | 0 | 0 | 0 |
| 77 | 0.000449 | 0.003286 | 0.861167 | 0.135098 | 0 | 0 | 0 | 0 | 0 |
| 78 | 0.000373 | 0.003506 | 0.862359 | 0.133762 | 0 | 0 | 0 | 0 | 0 |
| 79 | 0.00049 | 0.002981 | 0.864209 | 0.13232 | 0 | 0 | 0 | 0 | 0 |
| 80 | 0.000399 | 0.00283 | 0.862032 | 0.134739 | 0 | 0 | 0 | 0 | 0 |
| 81 | 0.000511 | 0.002968 | 0.857671 | 0.138849 | 0 | 0 | 0 | 0 | 0 |
| 82 | 0.000393 | 0.003231 | 0.861129 | 0.135247 | 0 | 0 | 0 | 0 | 0 |
| 83 | 0.000449 | 0.003385 | 0.862807 | 0.133359 | 0 | 0 | 0 | 0 | 0 |
| 84 | 0.0004 | 0.003428 | 0.862803 | 0.133369 | 0 | 0 | 0 | 0 | 0 |
| 85 | 0.000358 | 0.003258 | 0.859407 | 0.136978 | 0 | 0 | 0 | 0 | 0 |
| 86 | 0.000399 | 0.003086 | 0.866554 | 0.129961 | 0 | 0 | 0 | 0 | 0 |
| 87 | 0.000318 | 0.003671 | 0.857956 | 0.138055 | 0 | 0 | 0 | 0 | 0 |
| 88 | 0.000341 | 0.003059 | 0.863375 | 0.133195 | 0 | 0 | 0 | 0 | 0 |
| 89 | 0.000349 | 0.003185 | 0.860523 | 0.135942 | 0 | 0 | 0 | 0 | 0 |
| 90 | 0.00049 | 0.002838 | 0.862383 | 0.134259 | 0 | 0 | 0 | 0 | 0 |
| 91 | 0.000241 | 0.002899 | 0.859747 | 0.137113 | 0 | 0 | 0 | 0 | 0 |
| 92 | 0.00042 | 0.002958 | 0.856994 | 0.139628 | 0 | 0 | 0 | 0 | 0 |
| 93 | 0.000478 | 0.003215 | 0.859172 | 0.137135 | 0 | 0 | 0 | 0 | 0 |
| 94 | 0.000419 | 0.002924 | 0.861175 | 0.135481 | 0 | 0 | 0 | 0 | 0 |
| 95 | 0.000391 | 0.003432 | 0.855695 | 0.140482 | 0 | 0 | 0 | 0 | 0 |
| 96 | 0.000411 | 0.003282 | 0.859576 | 0.136731 | 0 | 0 | 0 | 0 | 0 |
| 97 | 0.00042 | 0.003367 | 0.857711 | 0.138503 | 0 | 0 | 0 | 0 | 0 |
| 98 | 0.000412 | 0.003139 | 0.859884 | 0.136565 | 0 | 0 | 0 | 0 | 0 |
| 99 | 0.00029 | 0.003208 | 0.859737 | 0.136764 | 0 | 0 | 0 | 0 | 0 |
| 100 | 0.000459 | 0.003125 | 0.863601 | 0.132814 | 0 | 0 | 0 | 0 | 0 |
| 101 | 0.000529 | 0.003423 | 0.862534 | 0.133514 | 0 | 0 | 0 | 0 | 0 |
| 102 | 0.000441 | 0.003064 | 0.862933 | 0.133562 | 0 | 0 | 0 | 0 | 0 |
| 103 | 0.00056 | 0.003173 | 0.857989 | 0.138278 | 0 | 0 | 0 | 0 | 0 |
| 104 | 0.00047 | 0.003343 | 0.863578 | 0.132599 | 0 | 0 | 0 | 0 | 0 |
| 105 | 0.000339 | 0.00329 | 0.861867 | 0.134505 | 0 | 0 | 0 | 0 | 0 |
| 106 | 0.0004 | 0.003513 | 0.85723 | 0.138856 | 0 | 0 | 0 | 0 | 0 |
| 107 | 0.000449 | 0.002964 | 0.859896 | 0.136632 | 0 | 0 | 0 | 0 | 0 |
| 108 | 0.00054 | 0.003209 | 0.858164 | 0.138067 | 0 | 0 | 0 | 0 | 0 |
| 109 | 0.000458 | 0.003396 | 0.862695 | 0.133451 | 0 | 0 | 0 | 0 | 0 |
| 110 | 0.000389 | 0.003013 | 0.863528 | 0.13307 | 0 | 0 | 0 | 0 | 0 |
| 111 | 0.000427 | 0.003393 | 0.861784 | 0.134396 | 0 | 0 | 0 | 0 | 0 |
| 112 | 0.000481 | 0.003017 | 0.857876 | 0.138626 | 0 | 0 | 0 | 0 | 0 |
| 113 | 0.00037 | 0.002843 | 0.862399 | 0.134387 | 0 | 0 | 0 | 0 | 0 |
| 114 | 0.00041 | 0.00336 | 0.862113 | 0.134116 | 0 | 0 | 0 | 0 | 0 |
| 115 | 0.000398 | 0.003037 | 0.867337 | 0.129228 | 0 | 0 | 0 | 0 | 0 |
| 116 | 0.00043 | 0.003512 | 0.863361 | 0.132697 | 0 | 0 | 0 | 0 | 0 |
| 117 | 0.00045 | 0.003127 | 0.860104 | 0.13632 | 0 | 0 | 0 | 0 | 0 |
| 118 | 0.000441 | 0.003235 | 0.864971 | 0.131354 | 0 | 0 | 0 | 0 | 0 |
| 119 | 0.000514 | 0.003091 | 0.859887 | 0.136508 | 0 | 0 | 0 | 0 | 0 |
| 120 | 0.000431 | 0.003236 | 0.860274 | 0.136059 | 0 | 0 | 0 | 0 | 0 |
| 121 | 0.000389 | 0.002993 | 0.863281 | 0.133337 | 0 | 0 | 0 | 0 | 0 |
| 122 | 0.000371 | 0.002911 | 0.863069 | 0.133649 | 0 | 0 | 0 | 0 | 0 |
| 123 | 0.000332 | 0.00299 | 0.861458 | 0.135219 | 0 | 0 | 0 | 0 | 0 |
| 124 | 0.00026 | 0.002906 | 0.865795 | 0.13104 | 0 | 0 | 0 | 0 | 0 |
| 125 | 0.000429 | 0.00327 | 0.860553 | 0.135748 | 0 | 0 | 0 | 0 | 0 |
| 126 | 0.000509 | 0.003154 | 0.859509 | 0.136828 | 0 | 0 | 0 | 0 | 0 |
| 127 | 0.000432 | 0.003197 | 0.863262 | 0.133109 | 0 | 0 | 0 | 0 | 0 |
| 128 | 0.000477 | 0.002914 | 0.862591 | 0.134018 | 0 | 0 | 0 | 0 | 0 |
| 129 | 0.00044 | 0.003321 | 0.857914 | 0.138325 | 0 | 0 | 0 | 0 | 0 |
| 130 | 0.000458 | 0.003057 | 0.862261 | 0.134224 | 0 | 0 | 0 | 0 | 0 |
| 131 | 0.000401 | 0.003597 | 0.864003 | 0.131999 | 0 | 0 | 0 | 0 | 0 |
| 132 | 0.000492 | 0.00319 | 0.860213 | 0.136105 | 0 | 0 | 0 | 0 | 0 |
| 133 | 0.000378 | 0.002986 | 0.859462 | 0.137173 | 0 | 0 | 0 | 0 | 0 |
| 134 | 0.000321 | 0.003222 | 0.862284 | 0.134173 | 0 | 0 | 0 | 0 | 0 |
| 135 | 0.00048 | 0.00328 | 0.857639 | 0.138601 | 0 | 0 | 0 | 0 | 0 |
| 136 | 0.000479 | 0.00316 | 0.862475 | 0.133886 | 0 | 0 | 0 | 0 | 0 |
| 137 | 0.000492 | 0.002913 | 0.858321 | 0.138274 | 0 | 0 | 0 | 0 | 0 |
| 138 | 0.000483 | 0.003391 | 0.857662 | 0.138464 | 0 | 0 | 0 | 0 | 0 |
| 139 | 0.000389 | 0.00328 | 0.864598 | 0.131734 | 0 | 0 | 0 | 0 | 0 |
| 140 | 0.000432 | 0.003334 | 0.8595 | 0.136735 | 0 | 0 | 0 | 0 | 0 |
| 141 | 0.000419 | 0.003229 | 0.859809 | 0.136543 | 0 | 0 | 0 | 0 | 0 |
| 142 | 0.000421 | 0.003129 | 0.858699 | 0.137752 | 0 | 0 | 0 | 0 | 0 |
| 143 | 0.000489 | 0.002911 | 0.859681 | 0.136919 | 0 | 0 | 0 | 0 | 0 |
| 144 | 0.000414 | 0.003514 | 0.859771 | 0.1363 | 0 | 0 | 0 | 0 | 0 |
| 145 | 0.000431 | 0.002974 | 0.862073 | 0.134522 | 0 | 0 | 0 | 0 | 0 |
| 146 | 0.000501 | 0.003169 | 0.866114 | 0.130215 | 0 | 0 | 0 | 0 | 0 |
| 147 | 0.000449 | 0.003216 | 0.860269 | 0.136065 | 0 | 0 | 0 | 0 | 0 |
| 148 | 0.000417 | 0.003076 | 0.858919 | 0.137588 | 0 | 0 | 0 | 0 | 0 |
| 149 | 0.000479 | 0.003076 | 0.86115 | 0.135295 | 0 | 0 | 0 | 0 | 0 |
| 150 | 0.00053 | 0.003213 | 0.858947 | 0.137309 | 0 | 0 | 0 | 0 | 0 |
| 151 | 0.000351 | 0.003233 | 0.860264 | 0.136152 | 0 | 0 | 0 | 0 | 0 |
| 152 | 0.00026 | 0.003465 | 0.856119 | 0.140156 | 0 | 0 | 0 | 0 | 0 |
| 153 | 0.000552 | 0.00269 | 0.864551 | 0.132207 | 0 | 0 | 0 | 0 | 0 |
| 154 | 0.000479 | 0.003183 | 0.859529 | 0.136808 | 0 | 0 | 0 | 0 | 0 |
| 155 | 0.000469 | 0.002975 | 0.857258 | 0.139297 | 0 | 0 | 0 | 0 | 0 |
| 156 | 0.00044 | 0.0034 | 0.859056 | 0.137104 | 0 | 0 | 0 | 0 | 0 |
| 157 | 0.000499 | 0.003247 | 0.862092 | 0.134162 | 0 | 0 | 0 | 0 | 0 |
| 158 | 0.000412 | 0.003027 | 0.861062 | 0.135499 | 0 | 0 | 0 | 0 | 0 |
| 159 | 0.000481 | 0.00321 | 0.856729 | 0.13958 | 0 | 0 | 0 | 0 | 0 |
| 160 | 0.000495 | 0.00314 | 0.866785 | 0.12958 | 0 | 0 | 0 | 0 | 0 |
| 161 | 0.0004 | 0.003129 | 0.857598 | 0.138872 | 0 | 0 | 0 | 0 | 0 |
| 162 | 0.000439 | 0.003439 | 0.859862 | 0.13626 | 0 | 0 | 0 | 0 | 0 |
| 163 | 0.00046 | 0.002842 | 0.859603 | 0.137094 | 0 | 0 | 0 | 0 | 0 |
| 164 | 0.000331 | 0.003255 | 0.859806 | 0.136608 | 0 | 0 | 0 | 0 | 0 |
| 165 | 0.000463 | 0.003762 | 0.860648 | 0.135127 | 0 | 0 | 0 | 0 | 0 |
| 166 | 0.000361 | 0.003387 | 0.858997 | 0.137255 | 0 | 0 | 0 | 0 | 0 |
| 167 | 0.000529 | 0.003024 | 0.861823 | 0.134624 | 0 | 0 | 0 | 0 | 0 |
| 168 | 0.00037 | 0.00342 | 0.860482 | 0.135728 | 0 | 0 | 0 | 0 | 0 |
| 169 | 0.000511 | 0.003218 | 0.859234 | 0.137036 | 0 | 0 | 0 | 0 | 0 |
| 170 | 0.00051 | 0.003677 | 0.859805 | 0.136008 | 0 | 0 | 0 | 0 | 0 |
| 171 | 0.00045 | 0.003202 | 0.863575 | 0.132773 | 0 | 0 | 0 | 0 | 0 |
| 172 | 0.000381 | 0.003306 | 0.860511 | 0.135802 | 0 | 0 | 0 | 0 | 0 |
| 173 | 0.000351 | 0.003769 | 0.860047 | 0.135834 | 0 | 0 | 0 | 0 | 0 |
| 174 | 0.000394 | 0.003427 | 0.857094 | 0.139085 | 0 | 0 | 0 | 0 | 0 |
| 175 | 0.00038 | 0.003149 | 0.859031 | 0.13744 | 0 | 0 | 0 | 0 | 0 |
| 176 | 0.00046 | 0.002913 | 0.865795 | 0.130802 | 0 | 0 | 0 | 0 | 0 |
| 177 | 0.000441 | 0.003335 | 0.859636 | 0.136588 | 0 | 0 | 0 | 0 | 0 |
| 178 | 0.000438 | 0.003416 | 0.859513 | 0.136632 | 0 | 0 | 0 | 0 | 0 |
| 179 | 0.000547 | 0.003284 | 0.857423 | 0.138746 | 0 | 0 | 0 | 0 | 0 |
| 180 | 0.00051 | 0.003129 | 0.861762 | 0.134598 | 0 | 0 | 0 | 0 | 0 |
| 181 | 0.000401 | 0.003196 | 0.862407 | 0.133996 | 0 | 0 | 0 | 0 | 0 |
| 182 | 0.00043 | 0.003488 | 0.862593 | 0.133489 | 0 | 0 | 0 | 0 | 0 |
| 183 | 0.000402 | 0.003226 | 0.860213 | 0.136159 | 0 | 0 | 0 | 0 | 0 |
| 184 | 0.000378 | 0.002868 | 0.865068 | 0.131685 | 0 | 0 | 0 | 0 | 0 |
| 185 | 0.000301 | 0.002917 | 0.861175 | 0.135608 | 0 | 0 | 0 | 0 | 0 |
| 186 | 0.000528 | 0.003071 | 0.860144 | 0.136256 | 0 | 0 | 0 | 0 | 0 |
| 187 | 0.000381 | 0.003171 | 0.866535 | 0.129912 | 0 | 0 | 0 | 0 | 0 |
| 188 | 0.00037 | 0.003058 | 0.859786 | 0.136787 | 0 | 0 | 0 | 0 | 0 |
| 189 | 0.000479 | 0.003242 | 0.859467 | 0.136812 | 0 | 0 | 0 | 0 | 0 |
| 190 | 0.000381 | 0.003005 | 0.8597 | 0.136914 | 0 | 0 | 0 | 0 | 0 |
| 191 | 0.000331 | 0.003225 | 0.859661 | 0.136783 | 0 | 0 | 0 | 0 | 0 |
| 192 | 0.000459 | 0.002796 | 0.861222 | 0.135523 | 0 | 0 | 0 | 0 | 0 |
| 193 | 0.00057 | 0.003418 | 0.85771 | 0.138303 | 0 | 0 | 0 | 0 | 0 |
| 194 | 0.000451 | 0.003036 | 0.85786 | 0.138653 | 0 | 0 | 0 | 0 | 0 |
| 195 | 0.000269 | 0.003283 | 0.857793 | 0.138654 | 0 | 0 | 0 | 0 | 0 |
| 196 | 0.00031 | 0.003338 | 0.859483 | 0.136869 | 0 | 0 | 0 | 0 | 0 |
| 197 | 0.00042 | 0.003044 | 0.863633 | 0.132903 | 0 | 0 | 0 | 0 | 0 |
| 198 | 0.000372 | 0.003243 | 0.860502 | 0.135862 | 0 | 0 | 0 | 0 | 0 |
| 199 | 0.000329 | 0.003383 | 0.862265 | 0.134023 | 0 | 0 | 0 | 0 | 0 |
| 200 | 0.00045 | 0.003698 | 0.856722 | 0.13913 | 0 | 0 | 0 | 0 | 0 |
| 201 | 0.000531 | 0.003808 | 0.860602 | 0.135059 | 0 | 0 | 0 | 0 | 0 |
| 202 | 0.000459 | 0.003266 | 0.863789 | 0.132485 | 0 | 0 | 0 | 0 | 0 |
| 203 | 0.000507 | 0.002735 | 0.863519 | 0.133239 | 0 | 0 | 0 | 0 | 0 |
| 204 | 0.00032 | 0.003692 | 0.858372 | 0.137616 | 0 | 0 | 0 | 0 | 0 |
| 205 | 0.000502 | 0.00306 | 0.854814 | 0.141625 | 0 | 0 | 0 | 0 | 0 |
| 206 | 0.000388 | 0.002839 | 0.857328 | 0.139445 | 0 | 0 | 0 | 0 | 0 |
| 207 | 0.00044 | 0.003292 | 0.861737 | 0.134531 | 0 | 0 | 0 | 0 | 0 |
| 208 | 0.000321 | 0.003281 | 0.865436 | 0.130962 | 0 | 0 | 0 | 0 | 0 |
| 209 | 0.00037 | 0.003192 | 0.859914 | 0.136524 | 0 | 0 | 0 | 0 | 0 |
| 210 | 0.000349 | 0.003007 | 0.860106 | 0.136538 | 0 | 0 | 0 | 0 | 0 |
| 211 | 0.00044 | 0.003279 | 0.860905 | 0.135376 | 0 | 0 | 0 | 0 | 0 |
| 212 | 0.000472 | 0.003134 | 0.860961 | 0.135434 | 0 | 0 | 0 | 0 | 0 |
| 213 | 0.000389 | 0.00289 | 0.862269 | 0.134453 | 0 | 0 | 0 | 0 | 0 |
| 214 | 0.000439 | 0.002944 | 0.861928 | 0.134689 | 0 | 0 | 0 | 0 | 0 |
| 215 | 0.000512 | 0.003172 | 0.860084 | 0.136212 | 0 | 0 | 0 | 0 | 0 |
| 216 | 0.00036 | 0.003216 | 0.863899 | 0.132526 | 0 | 0 | 0 | 0 | 0 |
| 217 | 0.000442 | 0.002952 | 0.863163 | 0.133443 | 0 | 0 | 0 | 0 | 0 |
| 218 | 0.00042 | 0.003038 | 0.859585 | 0.136957 | 0 | 0 | 0 | 0 | 0 |
| 219 | 0.000461 | 0.003199 | 0.864713 | 0.131626 | 0 | 0 | 0 | 0 | 0 |
| 220 | 0.000398 | 0.003204 | 0.861775 | 0.134623 | 0 | 0 | 0 | 0 | 0 |
| 221 | 0.000441 | 0.003135 | 0.859252 | 0.137173 | 0 | 0 | 0 | 0 | 0 |
| 222 | 0.000409 | 0.003195 | 0.861676 | 0.134719 | 0 | 0 | 0 | 0 | 0 |
| 223 | 0.000472 | 0.00303 | 0.858278 | 0.138221 | 0 | 0 | 0 | 0 | 0 |
| 224 | 0.000528 | 0.003424 | 0.859741 | 0.136307 | 0 | 0 | 0 | 0 | 0 |
| 225 | 0.000322 | 0.003067 | 0.864217 | 0.132394 | 0 | 0 | 0 | 0 | 0 |
| 226 | 0.000292 | 0.002857 | 0.865412 | 0.131439 | 0 | 0 | 0 | 0 | 0 |
| 227 | 0.000408 | 0.003017 | 0.858601 | 0.137974 | 0 | 0 | 0 | 0 | 0 |
| 228 | 0.000519 | 0.003081 | 0.862659 | 0.133741 | 0 | 0 | 0 | 0 | 0 |
| 229 | 0.000519 | 0.003162 | 0.863053 | 0.133267 | 0 | 0 | 0 | 0 | 0 |
| 230 | 0.000449 | 0.003121 | 0.858423 | 0.138007 | 0 | 0 | 0 | 0 | 0 |
| 231 | 0.000425 | 0.003073 | 0.862236 | 0.134266 | 0 | 0 | 0 | 0 | 0 |
| 232 | 0.00044 | 0.003258 | 0.861767 | 0.134535 | 0 | 0 | 0 | 0 | 0 |
| 233 | 0.000518 | 0.003197 | 0.860229 | 0.136047 | 0 | 0 | 0 | 0 | 0 |
| 234 | 0.000408 | 0.003227 | 0.860665 | 0.1357 | 0 | 0 | 0 | 0 | 0 |
| 235 | 0.00043 | 0.003369 | 0.865364 | 0.130827 | 0 | 0 | 0 | 0 | 0 |
| 236 | 0.000443 | 0.002828 | 0.85754 | 0.13919 | 0 | 0 | 0 | 0 | 0 |
| 237 | 0.00035 | 0.002987 | 0.859431 | 0.137232 | 0 | 0 | 0 | 0 | 0 |
| 238 | 0.000526 | 0.003472 | 0.8555 | 0.140502 | 0 | 0 | 0 | 0 | 0 |
| 239 | 0.00041 | 0.003209 | 0.859044 | 0.137337 | 0 | 0 | 0 | 0 | 0 |
| 240 | 0.000489 | 0.003054 | 0.862564 | 0.133893 | 0 | 0 | 0 | 0 | 0 |
| 241 | 0.000391 | 0.003079 | 0.863455 | 0.133075 | 0 | 0 | 0 | 0 | 0 |
| 242 | 0.00046 | 0.003317 | 0.859502 | 0.136711 | 0 | 0 | 0 | 0 | 0 |
| 243 | 0.0003 | 0.002825 | 0.864557 | 0.132318 | 0 | 0 | 0 | 0 | 0 |
| 244 | 0.000488 | 0.00297 | 0.860792 | 0.13575 | 0 | 0 | 0 | 0 | 0 |
| 245 | 0.000371 | 0.00337 | 0.86169 | 0.134558 | 0 | 0 | 0 | 0 | 0 |
| 246 | 0.000449 | 0.003371 | 0.861486 | 0.134694 | 0 | 0 | 0 | 0 | 0 |
| 247 | 0.00047 | 0.003101 | 0.857276 | 0.139153 | 0 | 0 | 0 | 0 | 0 |
| 248 | 0.000428 | 0.003435 | 0.863394 | 0.132743 | 0 | 0 | 0 | 0 | 0 |
| 249 | 0.000511 | 0.003346 | 0.861434 | 0.134709 | 0 | 0 | 0 | 0 | 0 |
| 250 | 0.000493 | 0.003504 | 0.856304 | 0.139699 | 0 | 0 | 0 | 0 | 0 |
| 251 | 0.000329 | 0.003583 | 0.8621 | 0.133987 | 0 | 0 | 0 | 0 | 0 |
| 252 | 0.00033 | 0.003222 | 0.861469 | 0.134979 | 0 | 0 | 0 | 0 | 0 |
| 253 | 0.00041 | 0.003392 | 0.859745 | 0.136453 | 0 | 0 | 0 | 0 | 0 |
| 254 | 0.000378 | 0.003141 | 0.861423 | 0.135059 | 0 | 0 | 0 | 0 | 0 |
| 255 | 0.000341 | 0.003336 | 0.858392 | 0.137932 | 0 | 0 | 0 | 0 | 0 |
| 256 | 0.000463 | 0.003611 | 0.855697 | 0.140229 | 0 | 0 | 0 | 0 | 0 |
| 257 | 0.00041 | 0.003221 | 0.858966 | 0.137382 | 0 | 0 | 0 | 0 | 0 |
| 258 | 0.000428 | 0.003237 | 0.858369 | 0.137965 | 0 | 0 | 0 | 0 | 0 |
| 259 | 0.000431 | 0.003365 | 0.861927 | 0.134277 | 0 | 0 | 0 | 0 | 0 |
| 260 | 0.000319 | 0.003154 | 0.861389 | 0.135137 | 0 | 0 | 0 | 0 | 0 |
| 261 | 0.000311 | 0.003259 | 0.862074 | 0.134356 | 0 | 0 | 0 | 0 | 0 |
| 262 | 0.000439 | 0.003145 | 0.858904 | 0.137511 | 0 | 0 | 0 | 0 | 0 |
| 263 | 0.000478 | 0.003011 | 0.863221 | 0.13329 | 0 | 0 | 0 | 0 | 0 |
| 264 | 0.00038 | 0.002787 | 0.857442 | 0.139391 | 0 | 0 | 0 | 0 | 0 |
| 265 | 0.000452 | 0.003598 | 0.860128 | 0.135821 | 0 | 0 | 0 | 0 | 0 |
| 266 | 0.000469 | 0.00339 | 0.863015 | 0.133127 | 0 | 0 | 0 | 0 | 0 |
| 267 | 0.000342 | 0.002989 | 0.859357 | 0.137313 | 0 | 0 | 0 | 0 | 0 |
| 268 | 0.00044 | 0.003563 | 0.860513 | 0.135484 | 0 | 0 | 0 | 0 | 0 |
| 269 | 0.000553 | 0.003096 | 0.861811 | 0.13454 | 0 | 0 | 0 | 0 | 0 |
| 270 | 0.000381 | 0.00309 | 0.862712 | 0.133816 | 0 | 0 | 0 | 0 | 0 |
| 271 | 0.000419 | 0.003489 | 0.862989 | 0.133083 | 0 | 0 | 0 | 0 | 0 |
| 272 | 0.000311 | 0.002966 | 0.859544 | 0.137179 | 0 | 0 | 0 | 0 | 0 |
| 273 | 0.000503 | 0.00326 | 0.861792 | 0.134445 | 0 | 0 | 0 | 0 | 0 |
| 274 | 0.000461 | 0.003346 | 0.865254 | 0.13094 | 0 | 0 | 0 | 0 | 0 |
| 275 | 0.000391 | 0.003219 | 0.860878 | 0.135512 | 0 | 0 | 0 | 0 | 0 |
| 276 | 0.000378 | 0.002684 | 0.860868 | 0.13607 | 0 | 0 | 0 | 0 | 0 |
| 277 | 0.00035 | 0.003805 | 0.856551 | 0.139294 | 0 | 0 | 0 | 0 | 0 |
| 278 | 0.000398 | 0.003347 | 0.861686 | 0.134569 | 0 | 0 | 0 | 0 | 0 |
| 279 | 0.000409 | 0.003169 | 0.858256 | 0.138166 | 0 | 0 | 0 | 0 | 0 |
| 280 | 0.000442 | 0.003262 | 0.860399 | 0.135897 | 0 | 0 | 0 | 0 | 0 |
| 281 | 0.00042 | 0.003347 | 0.858012 | 0.138221 | 0 | 0 | 0 | 0 | 0 |
| 282 | 0.000421 | 0.003316 | 0.861138 | 0.135125 | 0 | 0 | 0 | 0 | 0 |
| 283 | 0.000471 | 0.003316 | 0.863375 | 0.132838 | 0 | 0 | 0 | 0 | 0 |
| 284 | 0.00043 | 0.003159 | 0.86334 | 0.133071 | 0 | 0 | 0 | 0 | 0 |
| 285 | 0.000412 | 0.002883 | 0.859911 | 0.136795 | 0 | 0 | 0 | 0 | 0 |
| 286 | 0.000451 | 0.00321 | 0.863189 | 0.133149 | 0 | 0 | 0 | 0 | 0 |
| 287 | 0.000472 | 0.003497 | 0.862082 | 0.133948 | 0 | 0 | 0 | 0 | 0 |
| 288 | 0.0005 | 0.002889 | 0.861139 | 0.135473 | 0 | 0 | 0 | 0 | 0 |
| 289 | 0.000331 | 0.003032 | 0.862901 | 0.133736 | 0 | 0 | 0 | 0 | 0 |
| 290 | 0.000412 | 0.003132 | 0.859503 | 0.136953 | 0 | 0 | 0 | 0 | 0 |
| 291 | 0.000434 | 0.002978 | 0.861714 | 0.134874 | 0 | 0 | 0 | 0 | 0 |
| 292 | 0.000501 | 0.003254 | 0.85928 | 0.136966 | 0 | 0 | 0 | 0 | 0 |
| 293 | 0.000503 | 0.003668 | 0.854982 | 0.140847 | 0 | 0 | 0 | 0 | 0 |
| 294 | 0.000391 | 0.003137 | 0.859406 | 0.137066 | 0 | 0 | 0 | 0 | 0 |
| 295 | 0.000371 | 0.002614 | 0.859952 | 0.137064 | 0 | 0 | 0 | 0 | 0 |
| 296 | 0.000501 | 0.002995 | 0.858724 | 0.13778 | 0 | 0 | 0 | 0 | 0 |
| 297 | 0.000421 | 0.003381 | 0.858648 | 0.13755 | 0 | 0 | 0 | 0 | 0 |
| 298 | 0.00045 | 0.003207 | 0.862357 | 0.133987 | 0 | 0 | 0 | 0 | 0 |
| 299 | 0.000452 | 0.003112 | 0.854652 | 0.141784 | 0 | 0 | 0 | 0 | 0 |
| 300 | 0.000501 | 0.002914 | 0.857744 | 0.138842 | 0 | 0 | 0 | 0 | 0 |
| 301 | 0.000407 | 0.003131 | 0.861828 | 0.134634 | 0 | 0 | 0 | 0 | 0 |
| 302 | 0.000407 | 0.00274 | 0.86083 | 0.136023 | 0 | 0 | 0 | 0 | 0 |
| 303 | 0.000399 | 0.003241 | 0.860916 | 0.135444 | 0 | 0 | 0 | 0 | 0 |
| 304 | 0.000492 | 0.003314 | 0.858131 | 0.138063 | 0 | 0 | 0 | 0 | 0 |
| 305 | 0.00048 | 0.002797 | 0.865091 | 0.131632 | 0 | 0 | 0 | 0 | 0 |
| 306 | 0.00036 | 0.003336 | 0.861492 | 0.134803 | 0 | 0 | 0 | 0 | 0 |
| 307 | 0.000579 | 0.002996 | 0.858114 | 0.138311 | 0 | 0 | 0 | 0 | 0 |
| 308 | 0.000409 | 0.003086 | 0.86459 | 0.131915 | 0 | 0 | 0 | 0 | 0 |
| 309 | 0.000418 | 0.003364 | 0.862747 | 0.133471 | 0 | 0 | 0 | 0 | 0 |
| 310 | 0.000483 | 0.003338 | 0.862981 | 0.133198 | 0 | 0 | 0 | 0 | 0 |
| 311 | 0.000443 | 0.003451 | 0.859006 | 0.1371 | 0 | 0 | 0 | 0 | 0 |
| 312 | 0.000439 | 0.003295 | 0.858729 | 0.137536 | 0 | 0 | 0 | 0 | 0 |
| 313 | 0.000531 | 0.003176 | 0.86156 | 0.134733 | 0 | 0 | 0 | 0 | 0 |
| 314 | 0.00045 | 0.002689 | 0.86379 | 0.133071 | 0 | 0 | 0 | 0 | 0 |
| 315 | 0.00043 | 0.003263 | 0.858781 | 0.137526 | 0 | 0 | 0 | 0 | 0 |
| 316 | 0.0004 | 0.00348 | 0.860345 | 0.135775 | 0 | 0 | 0 | 0 | 0 |
| 317 | 0.000349 | 0.003132 | 0.862375 | 0.134144 | 0 | 0 | 0 | 0 | 0 |
| 318 | 0.000453 | 0.003052 | 0.854914 | 0.14155 | 0 | 0 | 0 | 0 | 0 |
| 319 | 0.000541 | 0.003386 | 0.855916 | 0.140156 | 0 | 0 | 0 | 0 | 0 |
| 320 | 0.000411 | 0.003168 | 0.862168 | 0.134253 | 0 | 0 | 0 | 0 | 0 |
| 321 | 0.00048 | 0.002828 | 0.85902 | 0.137673 | 0 | 0 | 0 | 0 | 0 |
| 322 | 0.00047 | 0.002961 | 0.859185 | 0.137384 | 0 | 0 | 0 | 0 | 0 |
| 323 | 0.00032 | 0.003118 | 0.861592 | 0.13497 | 0 | 0 | 0 | 0 | 0 |
| 324 | 0.000357 | 0.003001 | 0.862812 | 0.133831 | 0 | 0 | 0 | 0 | 0 |
| 325 | 0.00047 | 0.003053 | 0.863637 | 0.132839 | 0 | 0 | 0 | 0 | 0 |
| 326 | 0.000348 | 0.002817 | 0.861966 | 0.134869 | 0 | 0 | 0 | 0 | 0 |
| 327 | 0.000482 | 0.002801 | 0.86185 | 0.134867 | 0 | 0 | 0 | 0 | 0 |
| 328 | 0.000446 | 0.003446 | 0.862525 | 0.133584 | 0 | 0 | 0 | 0 | 0 |
| 329 | 0.00047 | 0.002958 | 0.860237 | 0.136335 | 0 | 0 | 0 | 0 | 0 |
| 330 | 0.000359 | 0.002995 | 0.858511 | 0.138135 | 0 | 0 | 0 | 0 | 0 |
| 331 | 0.000369 | 0.003135 | 0.862488 | 0.134008 | 0 | 0 | 0 | 0 | 0 |
| 332 | 0.000451 | 0.003117 | 0.86079 | 0.135642 | 0 | 0 | 0 | 0 | 0 |
| 333 | 0.000378 | 0.003136 | 0.858514 | 0.137971 | 0 | 0 | 0 | 0 | 0 |
| 334 | 0.000369 | 0.003364 | 0.861072 | 0.135194 | 0 | 0 | 0 | 0 | 0 |
| 335 | 0.000503 | 0.003222 | 0.858125 | 0.13815 | 0 | 0 | 0 | 0 | 0 |
| 336 | 0.00023 | 0.003326 | 0.860372 | 0.136071 | 0 | 0 | 0 | 0 | 0 |
| 337 | 0.000355 | 0.003077 | 0.861845 | 0.134723 | 0 | 0 | 0 | 0 | 0 |
| 338 | 0.000487 | 0.003068 | 0.864775 | 0.13167 | 0 | 0 | 0 | 0 | 0 |
| 339 | 0.00032 | 0.003328 | 0.861078 | 0.135275 | 0 | 0 | 0 | 0 | 0 |
| 340 | 0.000413 | 0.00307 | 0.859408 | 0.137109 | 0 | 0 | 0 | 0 | 0 |
| 341 | 0.00047 | 0.00341 | 0.85823 | 0.137889 | 0 | 0 | 0 | 0 | 0 |
| 342 | 0.000546 | 0.003205 | 0.857947 | 0.138303 | 0 | 0 | 0 | 0 | 0 |
| 343 | 0.000347 | 0.002866 | 0.861046 | 0.135742 | 0 | 0 | 0 | 0 | 0 |
| 344 | 0.000462 | 0.002921 | 0.859928 | 0.136689 | 0 | 0 | 0 | 0 | 0 |
| 345 | 0.000474 | 0.003218 | 0.859566 | 0.136742 | 0 | 0 | 0 | 0 | 0 |
| 346 | 0.00045 | 0.003117 | 0.864362 | 0.132071 | 0 | 0 | 0 | 0 | 0 |
| 347 | 0.000468 | 0.003119 | 0.86145 | 0.134953 | 0 | 0 | 0 | 0 | 0 |
| 348 | 0.00034 | 0.002913 | 0.86102 | 0.135727 | 0 | 0 | 0 | 0 | 0 |
| 349 | 0.000362 | 0.002986 | 0.860553 | 0.136099 | 0 | 0 | 0 | 0 | 0 |
| 350 | 0.000364 | 0.003124 | 0.862044 | 0.134468 | 0 | 0 | 0 | 0 | 0 |
| 351 | 0.000439 | 0.003345 | 0.861449 | 0.134767 | 0 | 0 | 0 | 0 | 0 |
| 352 | 0.000488 | 0.003485 | 0.864084 | 0.131943 | 0 | 0 | 0 | 0 | 0 |
| 353 | 0.00039 | 0.003118 | 0.862942 | 0.133551 | 0 | 0 | 0 | 0 | 0 |
| 354 | 0.000617 | 0.003312 | 0.86003 | 0.136041 | 0 | 0 | 0 | 0 | 0 |
| 355 | 0.000391 | 0.00331 | 0.862539 | 0.133759 | 0 | 0 | 0 | 0 | 0 |
| 356 | 0.000508 | 0.003125 | 0.86306 | 0.133307 | 0 | 0 | 0 | 0 | 0 |
| 357 | 0.000472 | 0.003471 | 0.856247 | 0.13981 | 0 | 0 | 0 | 0 | 0 |
| 358 | 0.00034 | 0.003207 | 0.8595 | 0.136953 | 0 | 0 | 0 | 0 | 0 |
| 359 | 0.000402 | 0.003293 | 0.858987 | 0.137318 | 0 | 0 | 0 | 0 | 0 |
| 360 | 0.000272 | 0.003304 | 0.860808 | 0.135616 | 0 | 0 | 0 | 0 | 0 |
| 361 | 0.000489 | 0.003215 | 0.859245 | 0.137051 | 0 | 0 | 0 | 0 | 0 |
| 362 | 0.00053 | 0.003532 | 0.861021 | 0.134916 | 0 | 0 | 0 | 0 | 0 |
| 363 | 0.000404 | 0.002996 | 0.861204 | 0.135396 | 0 | 0 | 0 | 0 | 0 |
| 364 | 0.00036 | 0.003648 | 0.862288 | 0.133704 | 0 | 0 | 0 | 0 | 0 |
| 365 | 0.000488 | 0.003175 | 0.859941 | 0.136396 | 0 | 0 | 0 | 0 | 0 |
| 366 | 0.000434 | 0.00294 | 0.860919 | 0.135707 | 0 | 0 | 0 | 0 | 0 |
| 367 | 0.000546 | 0.003533 | 0.858914 | 0.137008 | 0 | 0 | 0 | 0 | 0 |
| 368 | 0.000501 | 0.003143 | 0.863268 | 0.133088 | 0 | 0 | 0 | 0 | 0 |
| 369 | 0.000411 | 0.00332 | 0.85882 | 0.137449 | 0 | 0 | 0 | 0 | 0 |
| 370 | 0.000401 | 0.003377 | 0.859336 | 0.136886 | 0 | 0 | 0 | 0 | 0 |
| 371 | 0.000409 | 0.003184 | 0.857147 | 0.139149 | 0 | 0 | 0 | 0 | 0 |
| 372 | 0.000419 | 0.003032 | 0.857117 | 0.139432 | 0 | 0 | 0 | 0 | 0 |
| 373 | 0.000461 | 0.003005 | 0.859709 | 0.136825 | 0 | 0 | 0 | 0 | 0 |
| 374 | 0.000458 | 0.003026 | 0.863344 | 0.133172 | 0 | 0 | 0 | 0 | 0 |
| 375 | 0.000329 | 0.002992 | 0.860289 | 0.136389 | 0 | 0 | 0 | 0 | 0 |
| 376 | 0.000502 | 0.003023 | 0.859537 | 0.136937 | 0 | 0 | 0 | 0 | 0 |
| 377 | 0.000429 | 0.003236 | 0.856848 | 0.139488 | 0 | 0 | 0 | 0 | 0 |
| 378 | 0.000529 | 0.003072 | 0.862253 | 0.134147 | 0 | 0 | 0 | 0 | 0 |
| 379 | 0.000372 | 0.00312 | 0.863218 | 0.13329 | 0 | 0 | 0 | 0 | 0 |
| 380 | 0.000424 | 0.003038 | 0.860608 | 0.135931 | 0 | 0 | 0 | 0 | 0 |
| 381 | 0.000367 | 0.003547 | 0.85883 | 0.137256 | 0 | 0 | 0 | 0 | 0 |
| 382 | 0.000437 | 0.002951 | 0.860247 | 0.136365 | 0 | 0 | 0 | 0 | 0 |
| 383 | 0.000402 | 0.002827 | 0.8632 | 0.133571 | 0 | 0 | 0 | 0 | 0 |
| 384 | 0.000391 | 0.003168 | 0.856306 | 0.140134 | 0 | 0 | 0 | 0 | 0 |
| 385 | 0.000475 | 0.003348 | 0.861479 | 0.134697 | 0 | 0 | 0 | 0 | 0 |
| 386 | 0.00036 | 0.0033 | 0.860791 | 0.135549 | 0 | 0 | 0 | 0 | 0 |
| 387 | 0.000349 | 0.00297 | 0.86256 | 0.134121 | 0 | 0 | 0 | 0 | 0 |
| 388 | 0.00042 | 0.003018 | 0.861718 | 0.134844 | 0 | 0 | 0 | 0 | 0 |
| 389 | 0.000402 | 0.003498 | 0.864925 | 0.131175 | 0 | 0 | 0 | 0 | 0 |
| 390 | 0.00033 | 0.00323 | 0.860107 | 0.136334 | 0 | 0 | 0 | 0 | 0 |
| 391 | 0.000451 | 0.003076 | 0.852283 | 0.14419 | 0 | 0 | 0 | 0 | 0 |
| 392 | 0.000489 | 0.00334 | 0.858892 | 0.137279 | 0 | 0 | 0 | 0 | 0 |
| 393 | 0.000441 | 0.003248 | 0.8596 | 0.136711 | 0 | 0 | 0 | 0 | 0 |
| 394 | 0.00035 | 0.003262 | 0.860459 | 0.135929 | 0 | 0 | 0 | 0 | 0 |
| 395 | 0.000359 | 0.003 | 0.860412 | 0.13623 | 0 | 0 | 0 | 0 | 0 |
| 396 | 0.000361 | 0.002871 | 0.862798 | 0.13397 | 0 | 0 | 0 | 0 | 0 |
| 397 | 0.000526 | 0.003228 | 0.863528 | 0.132698 | 0 | 0 | 0 | 0 | 0 |
| 398 | 0.000521 | 0.003218 | 0.86264 | 0.133621 | 0 | 0 | 0 | 0 | 0 |
| 399 | 0.00057 | 0.003101 | 0.86035 | 0.135978 | 0 | 0 | 0 | 0 | 0 |
| 400 | 0.00043 | 0.002661 | 0.864249 | 0.13266 | 0 | 0 | 0 | 0 | 0 |
| 401 | 0.000397 | 0.003083 | 0.86088 | 0.13564 | 0 | 0 | 0 | 0 | 0 |
| 402 | 0.000469 | 0.002843 | 0.861639 | 0.13505 | 0 | 0 | 0 | 0 | 0 |
| 403 | 0.000462 | 0.003255 | 0.861992 | 0.134291 | 0 | 0 | 0 | 0 | 0 |
| 404 | 0.00042 | 0.003624 | 0.860845 | 0.135111 | 0 | 0 | 0 | 0 | 0 |
| 405 | 0.00045 | 0.003041 | 0.864804 | 0.131705 | 0 | 0 | 0 | 0 | 0 |
| 406 | 0.00041 | 0.0031 | 0.861552 | 0.134938 | 0 | 0 | 0 | 0 | 0 |
| 407 | 0.000321 | 0.003712 | 0.860889 | 0.135078 | 0 | 0 | 0 | 0 | 0 |
| 408 | 0.00043 | 0.002859 | 0.858438 | 0.138273 | 0 | 0 | 0 | 0 | 0 |
| 409 | 0.000309 | 0.003647 | 0.860488 | 0.135557 | 0 | 0 | 0 | 0 | 0 |
| 410 | 0.000502 | 0.003224 | 0.859998 | 0.136276 | 0 | 0 | 0 | 0 | 0 |
| 411 | 0.000489 | 0.003116 | 0.863275 | 0.133119 | 0 | 0 | 0 | 0 | 0 |
| 412 | 0.000429 | 0.003052 | 0.859963 | 0.136556 | 0 | 0 | 0 | 0 | 0 |
| 413 | 0.000381 | 0.003259 | 0.859736 | 0.136624 | 0 | 0 | 0 | 0 | 0 |
| 414 | 0.000442 | 0.003763 | 0.862422 | 0.133373 | 0 | 0 | 0 | 0 | 0 |
| 415 | 0.0004 | 0.002987 | 0.863071 | 0.133542 | 0 | 0 | 0 | 0 | 0 |
| 416 | 0.000398 | 0.003044 | 0.859896 | 0.136622 | 0 | 0 | 0 | 0 | 0 |
| 417 | 0.000479 | 0.003202 | 0.86134 | 0.134979 | 0 | 0 | 0 | 0 | 0 |
| 418 | 0.000403 | 0.00304 | 0.864017 | 0.13254 | 0 | 0 | 0 | 0 | 0 |
| 419 | 0.00045 | 0.00317 | 0.857353 | 0.139027 | 0 | 0 | 0 | 0 | 0 |
| 420 | 0.000482 | 0.003606 | 0.861197 | 0.134715 | 0 | 0 | 0 | 0 | 0 |
| 421 | 0.000409 | 0.003286 | 0.86029 | 0.136015 | 0 | 0 | 0 | 0 | 0 |
| 422 | 0.000372 | 0.003337 | 0.861013 | 0.135279 | 0 | 0 | 0 | 0 | 0 |
| 423 | 0.000507 | 0.003125 | 0.863395 | 0.132973 | 0 | 0 | 0 | 0 | 0 |
| 424 | 0.000428 | 0.003291 | 0.864716 | 0.131546 | 0 | 0 | 0 | 0 | 0 |
| 425 | 0.00044 | 0.003401 | 0.858718 | 0.137441 | 0 | 0 | 0 | 0 | 0 |
| 426 | 0.000448 | 0.003456 | 0.856934 | 0.139162 | 0 | 0 | 0 | 0 | 0 |
| 427 | 0.000361 | 0.003668 | 0.861034 | 0.134938 | 0 | 0 | 0 | 0 | 0 |
| 428 | 0.00039 | 0.003468 | 0.863874 | 0.132269 | 0 | 0 | 0 | 0 | 0 |
| 429 | 0.000449 | 0.003415 | 0.862802 | 0.133333 | 0 | 0 | 0 | 0 | 0 |
| 430 | 0.00032 | 0.00333 | 0.861414 | 0.134935 | 0 | 0 | 0 | 0 | 0 |
| 431 | 0.000451 | 0.002947 | 0.861788 | 0.134815 | 0 | 0 | 0 | 0 | 0 |
| 432 | 0.000551 | 0.002897 | 0.858117 | 0.138435 | 0 | 0 | 0 | 0 | 0 |
| 433 | 0.000589 | 0.003073 | 0.858688 | 0.137651 | 0 | 0 | 0 | 0 | 0 |
| 434 | 0.000499 | 0.003156 | 0.861723 | 0.134622 | 0 | 0 | 0 | 0 | 0 |
| 435 | 0.000368 | 0.003225 | 0.862152 | 0.134255 | 0 | 0 | 0 | 0 | 0 |
| 436 | 0.000437 | 0.003022 | 0.856944 | 0.139596 | 0 | 0 | 0 | 0 | 0 |
| 437 | 0.000609 | 0.002865 | 0.857565 | 0.138961 | 0 | 0 | 0 | 0 | 0 |
| 438 | 0.000512 | 0.003546 | 0.858285 | 0.137656 | 0 | 0 | 0 | 0 | 0 |
| 439 | 0.000319 | 0.003368 | 0.859379 | 0.136935 | 0 | 0 | 0 | 0 | 0 |
| 440 | 0.00044 | 0.002837 | 0.86028 | 0.136444 | 0 | 0 | 0 | 0 | 0 |
| 441 | 0.000457 | 0.003099 | 0.862402 | 0.134042 | 0 | 0 | 0 | 0 | 0 |
| 442 | 0.000442 | 0.002785 | 0.862167 | 0.134605 | 0 | 0 | 0 | 0 | 0 |
| 443 | 0.000384 | 0.003002 | 0.860661 | 0.135953 | 0 | 0 | 0 | 0 | 0 |
| 444 | 0.000459 | 0.003295 | 0.860442 | 0.135804 | 0 | 0 | 0 | 0 | 0 |
| 445 | 0.000389 | 0.003398 | 0.857008 | 0.139205 | 0 | 0 | 0 | 0 | 0 |
| 446 | 0.000489 | 0.002854 | 0.859307 | 0.137349 | 0 | 0 | 0 | 0 | 0 |
| 447 | 0.000558 | 0.003038 | 0.862053 | 0.134351 | 0 | 0 | 0 | 0 | 0 |
| 448 | 0.000571 | 0.003546 | 0.858518 | 0.137365 | 0 | 0 | 0 | 0 | 0 |
| 449 | 0.000339 | 0.003187 | 0.862128 | 0.134346 | 0 | 0 | 0 | 0 | 0 |
| 450 | 0.000519 | 0.003055 | 0.85999 | 0.136436 | 0 | 0 | 0 | 0 | 0 |
| 451 | 0.000471 | 0.002984 | 0.864016 | 0.13253 | 0 | 0 | 0 | 0 | 0 |
| 452 | 0.000369 | 0.003361 | 0.862229 | 0.134042 | 0 | 0 | 0 | 0 | 0 |
| 453 | 0.00041 | 0.003347 | 0.862701 | 0.133542 | 0 | 0 | 0 | 0 | 0 |
| 454 | 0.000291 | 0.003096 | 0.861198 | 0.135415 | 0 | 0 | 0 | 0 | 0 |
| 455 | 0.000483 | 0.003007 | 0.866145 | 0.130366 | 0 | 0 | 0 | 0 | 0 |
| 456 | 0.000339 | 0.00312 | 0.861117 | 0.135423 | 0 | 0 | 0 | 0 | 0 |
| 457 | 0.000388 | 0.003374 | 0.860744 | 0.135474 | 0 | 0 | 0 | 0 | 0 |
| 458 | 0.000371 | 0.003191 | 0.863275 | 0.133163 | 0 | 0 | 0 | 0 | 0 |
| 459 | 0.00035 | 0.002656 | 0.858236 | 0.138759 | 0 | 0 | 0 | 0 | 0 |
| 460 | 0.00045 | 0.003647 | 0.857687 | 0.138217 | 0 | 0 | 0 | 0 | 0 |
| 461 | 0.00037 | 0.003522 | 0.861596 | 0.134512 | 0 | 0 | 0 | 0 | 0 |
| 462 | 0.000331 | 0.002739 | 0.865481 | 0.131449 | 0 | 0 | 0 | 0 | 0 |
| 463 | 0.000359 | 0.003363 | 0.859511 | 0.136767 | 0 | 0 | 0 | 0 | 0 |
| 464 | 0.000373 | 0.003341 | 0.862207 | 0.134028 | 0 | 0 | 0 | 0 | 0 |
| 465 | 0.000564 | 0.002828 | 0.862211 | 0.134397 | 0 | 0 | 0 | 0 | 0 |
| 466 | 0.000511 | 0.003434 | 0.855777 | 0.140278 | 0 | 0 | 0 | 0 | 0 |
| 467 | 0.000399 | 0.002763 | 0.857613 | 0.139225 | 0 | 0 | 0 | 0 | 0 |
| 468 | 0.00033 | 0.003036 | 0.862234 | 0.134401 | 0 | 0 | 0 | 0 | 0 |
| 469 | 0.000413 | 0.002851 | 0.861914 | 0.134821 | 0 | 0 | 0 | 0 | 0 |
| 470 | 0.00045 | 0.00283 | 0.862472 | 0.134248 | 0 | 0 | 0 | 0 | 0 |
| 471 | 0.000428 | 0.003462 | 0.863476 | 0.132634 | 0 | 0 | 0 | 0 | 0 |
| 472 | 0.00032 | 0.002972 | 0.861076 | 0.135632 | 0 | 0 | 0 | 0 | 0 |
| 473 | 0.000331 | 0.00322 | 0.858888 | 0.137561 | 0 | 0 | 0 | 0 | 0 |
| 474 | 0.0005 | 0.003193 | 0.861629 | 0.134678 | 0 | 0 | 0 | 0 | 0 |
| 475 | 0.000371 | 0.003455 | 0.859943 | 0.136232 | 0 | 0 | 0 | 0 | 0 |
| 476 | 0.000498 | 0.003517 | 0.855989 | 0.139996 | 0 | 0 | 0 | 0 | 0 |
| 477 | 0.000382 | 0.002823 | 0.859974 | 0.136821 | 0 | 0 | 0 | 0 | 0 |
| 478 | 0.000331 | 0.003712 | 0.858291 | 0.137666 | 0 | 0 | 0 | 0 | 0 |
| 479 | 0.00048 | 0.002771 | 0.85915 | 0.137599 | 0 | 0 | 0 | 0 | 0 |
| 480 | 0.000496 | 0.002944 | 0.856143 | 0.140417 | 0 | 0 | 0 | 0 | 0 |
| 481 | 0.000342 | 0.003317 | 0.866695 | 0.129645 | 0 | 0 | 0 | 0 | 0 |
| 482 | 0.000459 | 0.002934 | 0.858406 | 0.138201 | 0 | 0 | 0 | 0 | 0 |
| 483 | 0.0004 | 0.003104 | 0.862192 | 0.134304 | 0 | 0 | 0 | 0 | 0 |
| 484 | 0.000411 | 0.003308 | 0.855871 | 0.14041 | 0 | 0 | 0 | 0 | 0 |
| 485 | 0.00041 | 0.003406 | 0.865765 | 0.130419 | 0 | 0 | 0 | 0 | 0 |
| 486 | 0.000348 | 0.003333 | 0.862121 | 0.134198 | 0 | 0 | 0 | 0 | 0 |
| 487 | 0.00032 | 0.00295 | 0.864891 | 0.131838 | 0 | 0 | 0 | 0 | 0 |
| 488 | 0.000411 | 0.003255 | 0.86285 | 0.133484 | 0 | 0 | 0 | 0 | 0 |
| 489 | 0.000489 | 0.003006 | 0.862653 | 0.133852 | 0 | 0 | 0 | 0 | 0 |
| 490 | 0.000427 | 0.003517 | 0.861272 | 0.134783 | 0 | 0 | 0 | 0 | 0 |
| 491 | 0.000452 | 0.003809 | 0.859223 | 0.136515 | 0 | 0 | 0 | 0 | 0 |
| 492 | 0.000369 | 0.003409 | 0.856837 | 0.139386 | 0 | 0 | 0 | 0 | 0 |
| 493 | 0.000419 | 0.002875 | 0.860236 | 0.136469 | 0 | 0 | 0 | 0 | 0 |
| 494 | 0.000349 | 0.003083 | 0.856021 | 0.140547 | 0 | 0 | 0 | 0 | 0 |
| 495 | 0.000508 | 0.003178 | 0.858862 | 0.137452 | 0 | 0 | 0 | 0 | 0 |
| 496 | 0.000446 | 0.003396 | 0.858791 | 0.137279 | 0 | 0 | 0 | 0 | 0 |
| 497 | 0.000458 | 0.002829 | 0.861565 | 0.135148 | 0 | 0 | 0 | 0 | 0 |
| 498 | 0.00034 | 0.003293 | 0.861821 | 0.134546 | 0 | 0 | 0 | 0 | 0 |
| 499 | 0.000339 | 0.003155 | 0.856284 | 0.140221 | 0 | 0 | 0 | 0 | 0 |
| 500 | 0.00037 | 0.003439 | 0.862425 | 0.133766 | 0 | 0 | 0 | 0 | 0 |
| 501 | 0.000358 | 0.003379 | 0.862914 | 0.133349 | 0 | 0 | 0 | 0 | 0 |
| 502 | 0.000489 | 0.003086 | 0.86286 | 0.133564 | 0 | 0 | 0 | 0 | 0 |
| 503 | 0.000396 | 0.003012 | 0.861673 | 0.134918 | 0 | 0 | 0 | 0 | 0 |
| 504 | 0.000491 | 0.003086 | 0.862404 | 0.134019 | 0 | 0 | 0 | 0 | 0 |
| 505 | 0.0005 | 0.003363 | 0.861013 | 0.135113 | 0 | 0 | 0 | 0 | 0 |
| 506 | 0.00044 | 0.002743 | 0.861373 | 0.135444 | 0 | 0 | 0 | 0 | 0 |
| 507 | 0.000492 | 0.003275 | 0.861285 | 0.134947 | 0 | 0 | 0 | 0 | 0 |
| 508 | 0.00044 | 0.003002 | 0.86046 | 0.136098 | 0 | 0 | 0 | 0 | 0 |
| 509 | 0.000381 | 0.003405 | 0.858996 | 0.137218 | 0 | 0 | 0 | 0 | 0 |
| 510 | 0.000511 | 0.003398 | 0.863533 | 0.132558 | 0 | 0 | 0 | 0 | 0 |
| 511 | 0.000421 | 0.003128 | 0.860956 | 0.135495 | 0 | 0 | 0 | 0 | 0 |
| 512 | 0.000419 | 0.003425 | 0.856671 | 0.139485 | 0 | 0 | 0 | 0 | 0 |
| 513 | 0.000451 | 0.003186 | 0.86227 | 0.134092 | 0 | 0 | 0 | 0 | 0 |
| 514 | 0.000439 | 0.003113 | 0.860486 | 0.135962 | 0 | 0 | 0 | 0 | 0 |
| 515 | 0.000363 | 0.003344 | 0.858645 | 0.137649 | 0 | 0 | 0 | 0 | 0 |
| 516 | 0.000411 | 0.003009 | 0.856376 | 0.140203 | 0 | 0 | 0 | 0 | 0 |
| 517 | 0.00034 | 0.002906 | 0.858939 | 0.137816 | 0 | 0 | 0 | 0 | 0 |
| 518 | 0.00042 | 0.003141 | 0.860356 | 0.136083 | 0 | 0 | 0 | 0 | 0 |
| 519 | 0.000458 | 0.003374 | 0.859581 | 0.136517 | 0 | 0 | 0 | 0 | 0 |
| 520 | 0.000551 | 0.003193 | 0.860367 | 0.135879 | 0 | 0 | 0 | 0 | 0 |
| 521 | 0.000469 | 0.003077 | 0.860793 | 0.135661 | 0 | 0 | 0 | 0 | 0 |
| 522 | 0.00052 | 0.002868 | 0.862593 | 0.134019 | 0 | 0 | 0 | 0 | 0 |
| 523 | 0.000409 | 0.003233 | 0.861627 | 0.134731 | 0 | 0 | 0 | 0 | 0 |
| 524 | 0.0006 | 0.003039 | 0.858512 | 0.137849 | 0 | 0 | 0 | 0 | 0 |
| 525 | 0.000397 | 0.002821 | 0.859395 | 0.137388 | 0 | 0 | 0 | 0 | 0 |
| 526 | 0.000452 | 0.002981 | 0.859571 | 0.136996 | 0 | 0 | 0 | 0 | 0 |
| 527 | 0.000429 | 0.003134 | 0.858466 | 0.137971 | 0 | 0 | 0 | 0 | 0 |
| 528 | 0.000448 | 0.003288 | 0.860859 | 0.135405 | 0 | 0 | 0 | 0 | 0 |
| 529 | 0.000381 | 0.003107 | 0.859815 | 0.136698 | 0 | 0 | 0 | 0 | 0 |
| 530 | 0.000469 | 0.003441 | 0.855423 | 0.140667 | 0 | 0 | 0 | 0 | 0 |
| 531 | 0.000469 | 0.002976 | 0.86024 | 0.136315 | 0 | 0 | 0 | 0 | 0 |
| 532 | 0.000378 | 0.002957 | 0.864777 | 0.131888 | 0 | 0 | 0 | 0 | 0 |
| 533 | 0.000358 | 0.003083 | 0.859658 | 0.136901 | 0 | 0 | 0 | 0 | 0 |
| 534 | 0.000459 | 0.003404 | 0.855906 | 0.14023 | 0 | 0 | 0 | 0 | 0 |
| 535 | 0.000389 | 0.002965 | 0.863331 | 0.133315 | 0 | 0 | 0 | 0 | 0 |
| 536 | 0.000348 | 0.002926 | 0.861654 | 0.135001 | 0 | 0 | 0 | 0 | 0 |
| 537 | 0.000381 | 0.003121 | 0.861253 | 0.135245 | 0 | 0 | 0 | 0 | 0 |
| 538 | 0.000389 | 0.002926 | 0.856548 | 0.140137 | 0 | 0 | 0 | 0 | 0 |
| 539 | 0.000599 | 0.003346 | 0.858922 | 0.137133 | 0 | 0 | 0 | 0 | 0 |
| 540 | 0.00042 | 0.003362 | 0.855761 | 0.140457 | 0 | 0 | 0 | 0 | 0 |
| 541 | 0.000359 | 0.002711 | 0.86149 | 0.13544 | 0 | 0 | 0 | 0 | 0 |
| 542 | 0.0006 | 0.00325 | 0.859091 | 0.137059 | 0 | 0 | 0 | 0 | 0 |
| 543 | 0.000377 | 0.003217 | 0.865261 | 0.131145 | 0 | 0 | 0 | 0 | 0 |
| 544 | 0.000508 | 0.003234 | 0.859378 | 0.13686 | 0 | 0 | 0 | 0 | 0 |
| 545 | 0.00042 | 0.003711 | 0.857283 | 0.138586 | 0 | 0 | 0 | 0 | 0 |
| 546 | 0.000391 | 0.003257 | 0.861357 | 0.134995 | 0 | 0 | 0 | 0 | 0 |
| 547 | 0.000479 | 0.002977 | 0.860595 | 0.135949 | 0 | 0 | 0 | 0 | 0 |
| 548 | 0.000421 | 0.003261 | 0.860937 | 0.135381 | 0 | 0 | 0 | 0 | 0 |
| 549 | 0.000383 | 0.003504 | 0.856467 | 0.139646 | 0 | 0 | 0 | 0 | 0 |
| 550 | 0.000417 | 0.002918 | 0.862402 | 0.134263 | 0 | 0 | 0 | 0 | 0 |
| 551 | 0.000382 | 0.003409 | 0.859584 | 0.136625 | 0 | 0 | 0 | 0 | 0 |
| 552 | 0.000541 | 0.003018 | 0.859304 | 0.137136 | 0 | 0 | 0 | 0 | 0 |
| 553 | 0.000381 | 0.003292 | 0.856251 | 0.140076 | 0 | 0 | 0 | 0 | 0 |
| 554 | 0.000552 | 0.003174 | 0.862512 | 0.133763 | 0 | 0 | 0 | 0 | 0 |
| 555 | 0.00049 | 0.003121 | 0.858418 | 0.137971 | 0 | 0 | 0 | 0 | 0 |
| 556 | 0.000389 | 0.003438 | 0.864378 | 0.131795 | 0 | 0 | 0 | 0 | 0 |
| 557 | 0.000379 | 0.003282 | 0.862417 | 0.133923 | 0 | 0 | 0 | 0 | 0 |
| 558 | 0.000371 | 0.003136 | 0.860157 | 0.136336 | 0 | 0 | 0 | 0 | 0 |
| 559 | 0.00049 | 0.00319 | 0.86329 | 0.13303 | 0 | 0 | 0 | 0 | 0 |
| 560 | 0.00042 | 0.003049 | 0.861163 | 0.135369 | 0 | 0 | 0 | 0 | 0 |
| 561 | 0.00048 | 0.003419 | 0.859288 | 0.136814 | 0 | 0 | 0 | 0 | 0 |
| 562 | 0.000329 | 0.003374 | 0.860259 | 0.136037 | 0 | 0 | 0 | 0 | 0 |
| 563 | 0.000562 | 0.002903 | 0.858361 | 0.138173 | 0 | 0 | 0 | 0 | 0 |
| 564 | 0.000401 | 0.003358 | 0.863701 | 0.132541 | 0 | 0 | 0 | 0 | 0 |
| 565 | 0.000521 | 0.003379 | 0.857076 | 0.139024 | 0 | 0 | 0 | 0 | 0 |
| 566 | 0.000482 | 0.003172 | 0.858358 | 0.137988 | 0 | 0 | 0 | 0 | 0 |
| 567 | 0.00037 | 0.003467 | 0.857809 | 0.138354 | 0 | 0 | 0 | 0 | 0 |
| 568 | 0.000468 | 0.003442 | 0.860865 | 0.135225 | 0 | 0 | 0 | 0 | 0 |
| 569 | 0.000458 | 0.003408 | 0.863933 | 0.132201 | 0 | 0 | 0 | 0 | 0 |
| 570 | 0.000468 | 0.0031 | 0.857899 | 0.138533 | 0 | 0 | 0 | 0 | 0 |
| 571 | 0.000411 | 0.003288 | 0.859901 | 0.1364 | 0 | 0 | 0 | 0 | 0 |
| 572 | 0.00046 | 0.003211 | 0.860131 | 0.136199 | 0 | 0 | 0 | 0 | 0 |
| 573 | 0.00051 | 0.003558 | 0.859557 | 0.136375 | 0 | 0 | 0 | 0 | 0 |
| 574 | 0.00042 | 0.00306 | 0.862212 | 0.134298 | 0 | 0 | 0 | 0 | 0 |
| 575 | 0.000371 | 0.00297 | 0.859489 | 0.13717 | 0 | 0 | 0 | 0 | 0 |
| 576 | 0.00047 | 0.002941 | 0.862164 | 0.134425 | 0 | 0 | 0 | 0 | 0 |
| 577 | 0.000453 | 0.003222 | 0.856277 | 0.140048 | 0 | 0 | 0 | 0 | 0 |
| 578 | 0.00032 | 0.003127 | 0.860112 | 0.136441 | 0 | 0 | 0 | 0 | 0 |
| 579 | 0.000461 | 0.003347 | 0.859483 | 0.136709 | 0 | 0 | 0 | 0 | 0 |
| 580 | 0.00033 | 0.003264 | 0.860896 | 0.135509 | 0 | 0 | 0 | 0 | 0 |
| 581 | 0.000299 | 0.00332 | 0.859793 | 0.136588 | 0 | 0 | 0 | 0 | 0 |
| 582 | 0.00042 | 0.003084 | 0.860856 | 0.13564 | 0 | 0 | 0 | 0 | 0 |
| 583 | 0.000349 | 0.003079 | 0.860204 | 0.136368 | 0 | 0 | 0 | 0 | 0 |
| 584 | 0.000437 | 0.00324 | 0.8558 | 0.140523 | 0 | 0 | 0 | 0 | 0 |
| 585 | 0.000449 | 0.002985 | 0.859418 | 0.137148 | 0 | 0 | 0 | 0 | 0 |
| 586 | 0.00055 | 0.003291 | 0.857909 | 0.138249 | 0 | 0 | 0 | 0 | 0 |
| 587 | 0.000481 | 0.002864 | 0.861614 | 0.135041 | 0 | 0 | 0 | 0 | 0 |
| 588 | 0.000317 | 0.003173 | 0.862582 | 0.133928 | 0 | 0 | 0 | 0 | 0 |
| 589 | 0.000331 | 0.002952 | 0.861789 | 0.134928 | 0 | 0 | 0 | 0 | 0 |
| 590 | 0.000321 | 0.002997 | 0.85588 | 0.140802 | 0 | 0 | 0 | 0 | 0 |
| 591 | 0.000419 | 0.003321 | 0.862364 | 0.133896 | 0 | 0 | 0 | 0 | 0 |
| 592 | 0.000439 | 0.003585 | 0.860872 | 0.135103 | 0 | 0 | 0 | 0 | 0 |
| 593 | 0.000377 | 0.002976 | 0.864061 | 0.132587 | 0 | 0 | 0 | 0 | 0 |
| 594 | 0.000461 | 0.002856 | 0.861235 | 0.135448 | 0 | 0 | 0 | 0 | 0 |
| 595 | 0.000514 | 0.003515 | 0.860627 | 0.135344 | 0 | 0 | 0 | 0 | 0 |
| 596 | 0.000362 | 0.003096 | 0.863749 | 0.132792 | 0 | 0 | 0 | 0 | 0 |
| 597 | 0.000429 | 0.003205 | 0.857953 | 0.138412 | 0 | 0 | 0 | 0 | 0 |
| 598 | 0.00049 | 0.002789 | 0.863173 | 0.133549 | 0 | 0 | 0 | 0 | 0 |
| 599 | 0.000441 | 0.002824 | 0.861697 | 0.135038 | 0 | 0 | 0 | 0 | 0 |
| 600 | 0.000441 | 0.003046 | 0.859163 | 0.137351 | 0 | 0 | 0 | 0 | 0 |
| 601 | 0.000369 | 0.003622 | 0.858124 | 0.137885 | 0 | 0 | 0 | 0 | 0 |
| 602 | 0.000397 | 0.003046 | 0.863186 | 0.13337 | 0 | 0 | 0 | 0 | 0 |
| 603 | 0.000489 | 0.003321 | 0.859716 | 0.136475 | 0 | 0 | 0 | 0 | 0 |
| 604 | 0.00043 | 0.003278 | 0.862077 | 0.134215 | 0 | 0 | 0 | 0 | 0 |
| 605 | 0.000379 | 0.003231 | 0.861278 | 0.135112 | 0 | 0 | 0 | 0 | 0 |
| 606 | 0.000488 | 0.003127 | 0.859823 | 0.136562 | 0 | 0 | 0 | 0 | 0 |
| 607 | 0.00046 | 0.003464 | 0.863375 | 0.132701 | 0 | 0 | 0 | 0 | 0 |
| 608 | 0.000571 | 0.003197 | 0.858191 | 0.138041 | 0 | 0 | 0 | 0 | 0 |
| 609 | 0.00044 | 0.00333 | 0.861783 | 0.134446 | 0 | 0 | 0 | 0 | 0 |
| 610 | 0.00042 | 0.003503 | 0.861627 | 0.13445 | 0 | 0 | 0 | 0 | 0 |
| 611 | 0.000449 | 0.003223 | 0.86195 | 0.134377 | 0 | 0 | 0 | 0 | 0 |
| 612 | 0.00051 | 0.002838 | 0.85878 | 0.137873 | 0 | 0 | 0 | 0 | 0 |
| 613 | 0.000585 | 0.003209 | 0.857758 | 0.138406 | 0 | 0 | 0 | 0 | 0 |
| 614 | 0.00043 | 0.003297 | 0.865607 | 0.130666 | 0 | 0 | 0 | 0 | 0 |
| 615 | 0.000452 | 0.003388 | 0.861175 | 0.134975 | 0 | 0 | 0 | 0 | 0 |
| 616 | 0.00044 | 0.003301 | 0.861537 | 0.134722 | 0 | 0 | 0 | 0 | 0 |
| 617 | 0.00041 | 0.003107 | 0.863047 | 0.133437 | 0 | 0 | 0 | 0 | 0 |
| 618 | 0.000519 | 0.003237 | 0.859251 | 0.136993 | 0 | 0 | 0 | 0 | 0 |
| 619 | 0.000381 | 0.003188 | 0.859886 | 0.136545 | 0 | 0 | 0 | 0 | 0 |
| 620 | 0.000537 | 0.00308 | 0.861169 | 0.135215 | 0 | 0 | 0 | 0 | 0 |
| 621 | 0.00047 | 0.00316 | 0.862029 | 0.134341 | 0 | 0 | 0 | 0 | 0 |
| 622 | 0.000481 | 0.003137 | 0.857836 | 0.138546 | 0 | 0 | 0 | 0 | 0 |
| 623 | 0.000391 | 0.003156 | 0.86164 | 0.134813 | 0 | 0 | 0 | 0 | 0 |
| 624 | 0.0004 | 0.003457 | 0.861242 | 0.134901 | 0 | 0 | 0 | 0 | 0 |
| 625 | 0.000329 | 0.00323 | 0.86332 | 0.133101 | 0 | 0 | 0 | 0 | 0 |
| 626 | 0.000341 | 0.003207 | 0.860063 | 0.13639 | 0 | 0 | 0 | 0 | 0 |
| 627 | 0.00039 | 0.003076 | 0.860158 | 0.136376 | 0 | 0 | 0 | 0 | 0 |
| 628 | 0.000359 | 0.003003 | 0.861768 | 0.134869 | 0 | 0 | 0 | 0 | 0 |
| 629 | 0.00035 | 0.003071 | 0.860058 | 0.136521 | 0 | 0 | 0 | 0 | 0 |
| 630 | 0.000534 | 0.0029 | 0.85626 | 0.140307 | 0 | 0 | 0 | 0 | 0 |
| 631 | 0.000419 | 0.003432 | 0.86004 | 0.136109 | 0 | 0 | 0 | 0 | 0 |
| 632 | 0.00042 | 0.003252 | 0.86259 | 0.133738 | 0 | 0 | 0 | 0 | 0 |
| 633 | 0.000609 | 0.002677 | 0.861136 | 0.135577 | 0 | 0 | 0 | 0 | 0 |
| 634 | 0.000471 | 0.002815 | 0.860381 | 0.136333 | 0 | 0 | 0 | 0 | 0 |
| 635 | 0.000392 | 0.003129 | 0.859646 | 0.136822 | 0 | 0 | 0 | 0 | 0 |
| 636 | 0.000528 | 0.003028 | 0.85935 | 0.137095 | 0 | 0 | 0 | 0 | 0 |
| 637 | 0.00035 | 0.002974 | 0.86019 | 0.136486 | 0 | 0 | 0 | 0 | 0 |
| 638 | 0.000452 | 0.003357 | 0.861002 | 0.135189 | 0 | 0 | 0 | 0 | 0 |
| 639 | 0.000451 | 0.003014 | 0.857379 | 0.139157 | 0 | 0 | 0 | 0 | 0 |
| 640 | 0.000431 | 0.002934 | 0.855619 | 0.141016 | 0 | 0 | 0 | 0 | 0 |
| 641 | 0.0005 | 0.003009 | 0.861526 | 0.134965 | 0 | 0 | 0 | 0 | 0 |
| 642 | 0.000389 | 0.003143 | 0.862211 | 0.134257 | 0 | 0 | 0 | 0 | 0 |
| 643 | 0.00047 | 0.003119 | 0.861033 | 0.135378 | 0 | 0 | 0 | 0 | 0 |
| 644 | 0.000501 | 0.002975 | 0.866826 | 0.129698 | 0 | 0 | 0 | 0 | 0 |
| 645 | 0.00046 | 0.003142 | 0.859463 | 0.136936 | 0 | 0 | 0 | 0 | 0 |
| 646 | 0.000503 | 0.003138 | 0.859096 | 0.137263 | 0 | 0 | 0 | 0 | 0 |
| 647 | 0.000449 | 0.002794 | 0.863423 | 0.133333 | 0 | 0 | 0 | 0 | 0 |
| 648 | 0.00052 | 0.003519 | 0.861156 | 0.134805 | 0 | 0 | 0 | 0 | 0 |
| 649 | 0.000528 | 0.003445 | 0.862771 | 0.133256 | 0 | 0 | 0 | 0 | 0 |
| 650 | 0.000544 | 0.003342 | 0.860989 | 0.135125 | 0 | 0 | 0 | 0 | 0 |
| 651 | 0.000409 | 0.002813 | 0.859854 | 0.136924 | 0 | 0 | 0 | 0 | 0 |
| 652 | 0.000512 | 0.003012 | 0.858837 | 0.137639 | 0 | 0 | 0 | 0 | 0 |
| 653 | 0.000512 | 0.003199 | 0.862523 | 0.133766 | 0 | 0 | 0 | 0 | 0 |
| 654 | 0.00037 | 0.003452 | 0.860146 | 0.136031 | 0 | 0 | 0 | 0 | 0 |
| 655 | 0.000421 | 0.003217 | 0.858775 | 0.137588 | 0 | 0 | 0 | 0 | 0 |
| 656 | 0.00039 | 0.003099 | 0.865575 | 0.130936 | 0 | 0 | 0 | 0 | 0 |
| 657 | 0.000491 | 0.003256 | 0.862555 | 0.133698 | 0 | 0 | 0 | 0 | 0 |
| 658 | 0.000372 | 0.002982 | 0.857702 | 0.138944 | 0 | 0 | 0 | 0 | 0 |
| 659 | 0.000352 | 0.003716 | 0.860126 | 0.135806 | 0 | 0 | 0 | 0 | 0 |
| 660 | 0.000529 | 0.003271 | 0.86366 | 0.13254 | 0 | 0 | 0 | 0 | 0 |
| 661 | 0.000399 | 0.003074 | 0.859792 | 0.136734 | 0 | 0 | 0 | 0 | 0 |
| 662 | 0.00041 | 0.003364 | 0.858364 | 0.137861 | 0 | 0 | 0 | 0 | 0 |
| 663 | 0.000461 | 0.003274 | 0.860101 | 0.136164 | 0 | 0 | 0 | 0 | 0 |
| 664 | 0.000318 | 0.003331 | 0.865141 | 0.131189 | 0 | 0 | 0 | 0 | 0 |
| 665 | 0.000297 | 0.003173 | 0.857044 | 0.139486 | 0 | 0 | 0 | 0 | 0 |
| 666 | 0.000418 | 0.003228 | 0.859542 | 0.136811 | 0 | 0 | 0 | 0 | 0 |
| 667 | 0.00052 | 0.003171 | 0.860561 | 0.135748 | 0 | 0 | 0 | 0 | 0 |
| 668 | 0.00029 | 0.00313 | 0.85688 | 0.1397 | 0 | 0 | 0 | 0 | 0 |
| 669 | 0.000398 | 0.00334 | 0.858004 | 0.138258 | 0 | 0 | 0 | 0 | 0 |
| 670 | 0.000391 | 0.002854 | 0.857685 | 0.139071 | 0 | 0 | 0 | 0 | 0 |
| 671 | 0.000489 | 0.003381 | 0.858055 | 0.138075 | 0 | 0 | 0 | 0 | 0 |
| 672 | 0.000468 | 0.003318 | 0.860819 | 0.135394 | 0 | 0 | 0 | 0 | 0 |
| 673 | 0.000459 | 0.003313 | 0.863503 | 0.132725 | 0 | 0 | 0 | 0 | 0 |
| 674 | 0.000555 | 0.002977 | 0.859354 | 0.137114 | 0 | 0 | 0 | 0 | 0 |
| 675 | 0.000429 | 0.003155 | 0.860872 | 0.135543 | 0 | 0 | 0 | 0 | 0 |
| 676 | 0.000459 | 0.003536 | 0.860219 | 0.135786 | 0 | 0 | 0 | 0 | 0 |
| 677 | 0.000419 | 0.002892 | 0.859814 | 0.136876 | 0 | 0 | 0 | 0 | 0 |
| 678 | 0.000429 | 0.00325 | 0.858175 | 0.138147 | 0 | 0 | 0 | 0 | 0 |
| 679 | 0.000448 | 0.002979 | 0.862068 | 0.134505 | 0 | 0 | 0 | 0 | 0 |
| 680 | 0.00042 | 0.003521 | 0.857897 | 0.138162 | 0 | 0 | 0 | 0 | 0 |
| 681 | 0.000312 | 0.003238 | 0.857697 | 0.138732 | 0 | 0 | 0 | 0 | 0 |
| 682 | 0.000401 | 0.002949 | 0.859513 | 0.137137 | 0 | 0 | 0 | 0 | 0 |
| 683 | 0.000519 | 0.003501 | 0.86333 | 0.13265 | 0 | 0 | 0 | 0 | 0 |
| 684 | 0.000373 | 0.002785 | 0.860542 | 0.136299 | 0 | 0 | 0 | 0 | 0 |
| 685 | 0.000349 | 0.003276 | 0.864291 | 0.132085 | 0 | 0 | 0 | 0 | 0 |
| 686 | 0.000378 | 0.003289 | 0.855881 | 0.140403 | 0 | 0 | 0 | 0 | 0 |
| 687 | 0.00044 | 0.003187 | 0.860184 | 0.136189 | 0 | 0 | 0 | 0 | 0 |
| 688 | 0.00048 | 0.00289 | 0.860587 | 0.136043 | 0 | 0 | 0 | 0 | 0 |
| 689 | 0.000478 | 0.003456 | 0.85774 | 0.138325 | 0 | 0 | 0 | 0 | 0 |
| 690 | 0.00037 | 0.002896 | 0.863545 | 0.13319 | 0 | 0 | 0 | 0 | 0 |
| 691 | 0.000484 | 0.003338 | 0.86003 | 0.136149 | 0 | 0 | 0 | 0 | 0 |
| 692 | 0.000389 | 0.003334 | 0.860661 | 0.135616 | 0 | 0 | 0 | 0 | 0 |
| 693 | 0.000541 | 0.003214 | 0.856478 | 0.139768 | 0 | 0 | 0 | 0 | 0 |
| 694 | 0.000421 | 0.003365 | 0.860189 | 0.136025 | 0 | 0 | 0 | 0 | 0 |
| 695 | 0.000381 | 0.002875 | 0.861799 | 0.134945 | 0 | 0 | 0 | 0 | 0 |
| 696 | 0.000451 | 0.002824 | 0.863945 | 0.132781 | 0 | 0 | 0 | 0 | 0 |
| 697 | 0.00048 | 0.003133 | 0.861706 | 0.134681 | 0 | 0 | 0 | 0 | 0 |
| 698 | 0.00048 | 0.003117 | 0.859953 | 0.13645 | 0 | 0 | 0 | 0 | 0 |
| 699 | 0.00038 | 0.00311 | 0.861659 | 0.134851 | 0 | 0 | 0 | 0 | 0 |
| 700 | 0.000399 | 0.003383 | 0.862416 | 0.133802 | 0 | 0 | 0 | 0 | 0 |
| 701 | 0.000391 | 0.003652 | 0.866069 | 0.129888 | 0 | 0 | 0 | 0 | 0 |
| 702 | 0.000382 | 0.003484 | 0.864429 | 0.131706 | 0 | 0 | 0 | 0 | 0 |
| 703 | 0.00048 | 0.003549 | 0.861388 | 0.134583 | 0 | 0 | 0 | 0 | 0 |
| 704 | 0.00036 | 0.00294 | 0.861242 | 0.135458 | 0 | 0 | 0 | 0 | 0 |
| 705 | 0.000281 | 0.003162 | 0.86018 | 0.136377 | 0 | 0 | 0 | 0 | 0 |
| 706 | 0.000429 | 0.003513 | 0.861506 | 0.134552 | 0 | 0 | 0 | 0 | 0 |
| 707 | 0.00034 | 0.003344 | 0.859072 | 0.137244 | 0 | 0 | 0 | 0 | 0 |
| 708 | 0.000373 | 0.003458 | 0.862433 | 0.133737 | 0 | 0 | 0 | 0 | 0 |
| 709 | 0.000602 | 0.003153 | 0.864622 | 0.131623 | 0 | 0 | 0 | 0 | 0 |
| 710 | 0.00041 | 0.00333 | 0.859706 | 0.136554 | 0 | 0 | 0 | 0 | 0 |
| 711 | 0.000302 | 0.00301 | 0.860367 | 0.136321 | 0 | 0 | 0 | 0 | 0 |
| 712 | 0.000451 | 0.0034 | 0.866307 | 0.129842 | 0 | 0 | 0 | 0 | 0 |
| 713 | 0.000299 | 0.002847 | 0.858311 | 0.138534 | 0 | 0 | 0 | 0 | 0 |
| 714 | 0.00045 | 0.003422 | 0.860156 | 0.135973 | 0 | 0 | 0 | 0 | 0 |
| 715 | 0.000359 | 0.003048 | 0.855744 | 0.140849 | 0 | 0 | 0 | 0 | 0 |
| 716 | 0.00042 | 0.003419 | 0.855418 | 0.140744 | 0 | 0 | 0 | 0 | 0 |
| 717 | 0.000393 | 0.003101 | 0.860227 | 0.136279 | 0 | 0 | 0 | 0 | 0 |
| 718 | 0.000398 | 0.003236 | 0.863453 | 0.132912 | 0 | 0 | 0 | 0 | 0 |
| 719 | 0.00033 | 0.002879 | 0.861726 | 0.135065 | 0 | 0 | 0 | 0 | 0 |
| 720 | 0.000378 | 0.003112 | 0.857752 | 0.138708 | 0 | 0 | 0 | 0 | 0 |
| 721 | 0.000383 | 0.003353 | 0.85652 | 0.139744 | 0 | 0 | 0 | 0 | 0 |
| 722 | 0.000361 | 0.003486 | 0.859666 | 0.136487 | 0 | 0 | 0 | 0 | 0 |
| 723 | 0.00045 | 0.003658 | 0.858815 | 0.137078 | 0 | 0 | 0 | 0 | 0 |
| 724 | 0.000451 | 0.003325 | 0.861101 | 0.135123 | 0 | 0 | 0 | 0 | 0 |
| 725 | 0.0005 | 0.003453 | 0.857629 | 0.138417 | 0 | 0 | 0 | 0 | 0 |
| 726 | 0.00044 | 0.002869 | 0.862881 | 0.13381 | 0 | 0 | 0 | 0 | 0 |
| 727 | 0.000351 | 0.003169 | 0.858394 | 0.138086 | 0 | 0 | 0 | 0 | 0 |
| 728 | 0.000522 | 0.003301 | 0.861651 | 0.134527 | 0 | 0 | 0 | 0 | 0 |
| 729 | 0.000427 | 0.003725 | 0.860271 | 0.135577 | 0 | 0 | 0 | 0 | 0 |
| 730 | 0.000368 | 0.003212 | 0.859979 | 0.136441 | 0 | 0 | 0 | 0 | 0 |
| 731 | 0.00035 | 0.003192 | 0.862369 | 0.134089 | 0 | 0 | 0 | 0 | 0 |
| 732 | 0.00045 | 0.003393 | 0.861361 | 0.134795 | 0 | 0 | 0 | 0 | 0 |
| 733 | 0.00052 | 0.00293 | 0.864682 | 0.131867 | 0 | 0 | 0 | 0 | 0 |
| 734 | 0.000579 | 0.003372 | 0.858408 | 0.137641 | 0 | 0 | 0 | 0 | 0 |
| 735 | 0.000398 | 0.003226 | 0.864474 | 0.131902 | 0 | 0 | 0 | 0 | 0 |
| 736 | 0.000459 | 0.003482 | 0.861436 | 0.134623 | 0 | 0 | 0 | 0 | 0 |
| 737 | 0.000422 | 0.002997 | 0.862288 | 0.134293 | 0 | 0 | 0 | 0 | 0 |
| 738 | 0.000457 | 0.002814 | 0.856609 | 0.14012 | 0 | 0 | 0 | 0 | 0 |
| 739 | 0.00033 | 0.003121 | 0.857626 | 0.138923 | 0 | 0 | 0 | 0 | 0 |
| 740 | 0.000409 | 0.003352 | 0.864394 | 0.131825 | 0 | 0 | 0 | 0 | 0 |
| 741 | 0.000319 | 0.003054 | 0.860364 | 0.136262 | 0 | 0 | 0 | 0 | 0 |
| 742 | 0.000399 | 0.003178 | 0.861875 | 0.134548 | 0 | 0 | 0 | 0 | 0 |
| 743 | 0.000568 | 0.00319 | 0.859209 | 0.137033 | 0 | 0 | 0 | 0 | 0 |
| 744 | 0.00039 | 0.00349 | 0.863538 | 0.132532 | 0 | 0 | 0 | 0 | 0 |
| 745 | 0.000421 | 0.003158 | 0.865022 | 0.131369 | 0 | 0 | 0 | 0 | 0 |
| 746 | 0.0004 | 0.003156 | 0.857923 | 0.138521 | 0 | 0 | 0 | 0 | 0 |
| 747 | 0.00046 | 0.003577 | 0.858049 | 0.137913 | 0 | 0 | 0 | 0 | 0 |
| 748 | 0.000399 | 0.002815 | 0.864095 | 0.13269 | 0 | 0 | 0 | 0 | 0 |
| 749 | 0.00034 | 0.00291 | 0.863121 | 0.133629 | 0 | 0 | 0 | 0 | 0 |
| 750 | 0.000439 | 0.003233 | 0.862679 | 0.133649 | 0 | 0 | 0 | 0 | 0 |
| 751 | 0.000511 | 0.002826 | 0.864148 | 0.132515 | 0 | 0 | 0 | 0 | 0 |
| 752 | 0.000471 | 0.003326 | 0.860816 | 0.135387 | 0 | 0 | 0 | 0 | 0 |
| 753 | 0.00033 | 0.002976 | 0.861921 | 0.134774 | 0 | 0 | 0 | 0 | 0 |
| 754 | 0.000412 | 0.003165 | 0.860395 | 0.136027 | 0 | 0 | 0 | 0 | 0 |
| 755 | 0.000527 | 0.003114 | 0.859906 | 0.136453 | 0 | 0 | 0 | 0 | 0 |
| 756 | 0.0005 | 0.0032 | 0.859734 | 0.136565 | 0 | 0 | 0 | 0 | 0 |
| 757 | 0.000414 | 0.003511 | 0.859614 | 0.136461 | 0 | 0 | 0 | 0 | 0 |
| 758 | 0.00031 | 0.003538 | 0.86025 | 0.135902 | 0 | 0 | 0 | 0 | 0 |
| 759 | 0.00041 | 0.003192 | 0.858449 | 0.137949 | 0 | 0 | 0 | 0 | 0 |
| 760 | 0.00033 | 0.003521 | 0.861212 | 0.134937 | 0 | 0 | 0 | 0 | 0 |
| 761 | 0.000443 | 0.003111 | 0.858341 | 0.138105 | 0 | 0 | 0 | 0 | 0 |
| 762 | 0.000371 | 0.002877 | 0.861434 | 0.135319 | 0 | 0 | 0 | 0 | 0 |
| 763 | 0.000509 | 0.003763 | 0.86116 | 0.134568 | 0 | 0 | 0 | 0 | 0 |
| 764 | 0.00055 | 0.003308 | 0.861734 | 0.134409 | 0 | 0 | 0 | 0 | 0 |
| 765 | 0.00043 | 0.003169 | 0.860079 | 0.136322 | 0 | 0 | 0 | 0 | 0 |
| 766 | 0.000509 | 0.002933 | 0.859685 | 0.136873 | 0 | 0 | 0 | 0 | 0 |
| 767 | 0.000481 | 0.002688 | 0.86467 | 0.132161 | 0 | 0 | 0 | 0 | 0 |
| 768 | 0.000488 | 0.003316 | 0.860529 | 0.135667 | 0 | 0 | 0 | 0 | 0 |
| 769 | 0.00035 | 0.003212 | 0.862304 | 0.134134 | 0 | 0 | 0 | 0 | 0 |
| 770 | 0.00042 | 0.003052 | 0.861512 | 0.135016 | 0 | 0 | 0 | 0 | 0 |
| 771 | 0.000412 | 0.003194 | 0.862264 | 0.134071 | 0 | 0 | 0 | 0 | 0 |
| 772 | 0.00057 | 0.002871 | 0.858784 | 0.137755 | 0 | 0 | 0 | 0 | 0 |
| 773 | 0.00039 | 0.003254 | 0.858115 | 0.13824 | 0 | 0 | 0 | 0 | 0 |
| 774 | 0.000541 | 0.003057 | 0.861886 | 0.134517 | 0 | 0 | 0 | 0 | 0 |
| 775 | 0.000369 | 0.003437 | 0.860657 | 0.135537 | 0 | 0 | 0 | 0 | 0 |
| 776 | 0.000398 | 0.003331 | 0.860182 | 0.136089 | 0 | 0 | 0 | 0 | 0 |
| 777 | 0.00044 | 0.003661 | 0.861583 | 0.134316 | 0 | 0 | 0 | 0 | 0 |
| 778 | 0.000468 | 0.003484 | 0.863277 | 0.132771 | 0 | 0 | 0 | 0 | 0 |
| 779 | 0.000379 | 0.003009 | 0.865522 | 0.13109 | 0 | 0 | 0 | 0 | 0 |
| 780 | 0.000397 | 0.003327 | 0.86162 | 0.134656 | 0 | 0 | 0 | 0 | 0 |
| 781 | 0.000449 | 0.00341 | 0.859164 | 0.136978 | 0 | 0 | 0 | 0 | 0 |
| 782 | 0.00046 | 0.003529 | 0.863477 | 0.132534 | 0 | 0 | 0 | 0 | 0 |
| 783 | 0.000448 | 0.003274 | 0.862417 | 0.133861 | 0 | 0 | 0 | 0 | 0 |
| 784 | 0.000521 | 0.003185 | 0.859405 | 0.136889 | 0 | 0 | 0 | 0 | 0 |
| 785 | 0.000339 | 0.002954 | 0.85961 | 0.137097 | 0 | 0 | 0 | 0 | 0 |
| 786 | 0.00042 | 0.002961 | 0.858626 | 0.137993 | 0 | 0 | 0 | 0 | 0 |
| 787 | 0.00049 | 0.00309 | 0.86441 | 0.13201 | 0 | 0 | 0 | 0 | 0 |
| 788 | 0.0003 | 0.003269 | 0.862181 | 0.13425 | 0 | 0 | 0 | 0 | 0 |
| 789 | 0.000451 | 0.002989 | 0.8586 | 0.13796 | 0 | 0 | 0 | 0 | 0 |
| 790 | 0.000329 | 0.003134 | 0.862587 | 0.13395 | 0 | 0 | 0 | 0 | 0 |
| 791 | 0.000389 | 0.003155 | 0.863108 | 0.133348 | 0 | 0 | 0 | 0 | 0 |
| 792 | 0.00043 | 0.003331 | 0.863096 | 0.133143 | 0 | 0 | 0 | 0 | 0 |
| 793 | 0.000432 | 0.002993 | 0.858443 | 0.138133 | 0 | 0 | 0 | 0 | 0 |
| 794 | 0.000503 | 0.00317 | 0.862322 | 0.134005 | 0 | 0 | 0 | 0 | 0 |
| 795 | 0.000432 | 0.003418 | 0.859084 | 0.137065 | 0 | 0 | 0 | 0 | 0 |
| 796 | 0.000373 | 0.003355 | 0.85973 | 0.136542 | 0 | 0 | 0 | 0 | 0 |
| 797 | 0.000512 | 0.003172 | 0.859788 | 0.136528 | 0 | 0 | 0 | 0 | 0 |
| 798 | 0.000452 | 0.003111 | 0.859857 | 0.136571 | 0 | 0 | 0 | 0 | 0 |
| 799 | 0.000472 | 0.003043 | 0.860336 | 0.136149 | 0 | 0 | 0 | 0 | 0 |
| 800 | 0.000512 | 0.003361 | 0.859178 | 0.13695 | 0 | 0 | 0 | 0 | 0 |
| 801 | 0.000409 | 0.003022 | 0.863198 | 0.133361 | 0 | 0 | 0 | 0 | 0 |
| 802 | 0.000491 | 0.003333 | 0.859804 | 0.136372 | 0 | 0 | 0 | 0 | 0 |
| 803 | 0.000538 | 0.00318 | 0.863877 | 0.132404 | 0 | 0 | 0 | 0 | 0 |
| 804 | 0.000447 | 0.003146 | 0.860998 | 0.13541 | 0 | 0 | 0 | 0 | 0 |
| 805 | 0.000321 | 0.002921 | 0.865455 | 0.131303 | 0 | 0 | 0 | 0 | 0 |
| 806 | 0.000502 | 0.003362 | 0.857712 | 0.138424 | 0 | 0 | 0 | 0 | 0 |
| 807 | 0.000462 | 0.003652 | 0.861715 | 0.134171 | 0 | 0 | 0 | 0 | 0 |
| 808 | 0.000482 | 0.00294 | 0.861002 | 0.135576 | 0 | 0 | 0 | 0 | 0 |
| 809 | 0.000381 | 0.002966 | 0.860808 | 0.135845 | 0 | 0 | 0 | 0 | 0 |
| 810 | 0.000512 | 0.003244 | 0.857529 | 0.138715 | 0 | 0 | 0 | 0 | 0 |
| 811 | 0.000432 | 0.003095 | 0.863383 | 0.13309 | 0 | 0 | 0 | 0 | 0 |
| 812 | 0.00038 | 0.002853 | 0.866169 | 0.130597 | 0 | 0 | 0 | 0 | 0 |
| 813 | 0.000563 | 0.003116 | 0.865302 | 0.131019 | 0 | 0 | 0 | 0 | 0 |
| 814 | 0.000451 | 0.003341 | 0.861142 | 0.135066 | 0 | 0 | 0 | 0 | 0 |
| 815 | 0.000478 | 0.002979 | 0.859109 | 0.137433 | 0 | 0 | 0 | 0 | 0 |
| 816 | 0.000498 | 0.00327 | 0.863177 | 0.133055 | 0 | 0 | 0 | 0 | 0 |
| 817 | 0.000401 | 0.003235 | 0.865949 | 0.130416 | 0 | 0 | 0 | 0 | 0 |
| 818 | 0.000368 | 0.003114 | 0.859263 | 0.137255 | 0 | 0 | 0 | 0 | 0 |
| 819 | 0.000459 | 0.002824 | 0.856332 | 0.140386 | 0 | 0 | 0 | 0 | 0 |
| 820 | 0.000431 | 0.003258 | 0.861816 | 0.134495 | 0 | 0 | 0 | 0 | 0 |
| 821 | 0.000419 | 0.003345 | 0.86598 | 0.130255 | 0 | 0 | 0 | 0 | 0 |
| 822 | 0.000331 | 0.003063 | 0.861836 | 0.13477 | 0 | 0 | 0 | 0 | 0 |
| 823 | 0.000442 | 0.003285 | 0.85921 | 0.137063 | 0 | 0 | 0 | 0 | 0 |
| 824 | 0.000513 | 0.003681 | 0.863817 | 0.131988 | 0 | 0 | 0 | 0 | 0 |
| 825 | 0.000281 | 0.003203 | 0.861422 | 0.135094 | 0 | 0 | 0 | 0 | 0 |
| 826 | 0.000348 | 0.002976 | 0.860779 | 0.135897 | 0 | 0 | 0 | 0 | 0 |
| 827 | 0.00049 | 0.003541 | 0.859282 | 0.136686 | 0 | 0 | 0 | 0 | 0 |
| 828 | 0.000512 | 0.003009 | 0.86327 | 0.13321 | 0 | 0 | 0 | 0 | 0 |
| 829 | 0.00042 | 0.003282 | 0.863809 | 0.132489 | 0 | 0 | 0 | 0 | 0 |
| 830 | 0.000379 | 0.003508 | 0.861544 | 0.134569 | 0 | 0 | 0 | 0 | 0 |
| 831 | 0.00039 | 0.003372 | 0.859214 | 0.137024 | 0 | 0 | 0 | 0 | 0 |
| 832 | 0.000477 | 0.00328 | 0.862666 | 0.133577 | 0 | 0 | 0 | 0 | 0 |
| 833 | 0.00029 | 0.003037 | 0.859124 | 0.13755 | 0 | 0 | 0 | 0 | 0 |
| 834 | 0.000392 | 0.003404 | 0.859744 | 0.136431 | 0 | 0 | 0 | 0 | 0 |
| 835 | 0.000472 | 0.003418 | 0.857908 | 0.138202 | 0 | 0 | 0 | 0 | 0 |
| 836 | 0.0005 | 0.003222 | 0.861786 | 0.134491 | 0 | 0 | 0 | 0 | 0 |
| 837 | 0.00036 | 0.003212 | 0.863219 | 0.133209 | 0 | 0 | 0 | 0 | 0 |
| 838 | 0.000429 | 0.003222 | 0.861717 | 0.134631 | 0 | 0 | 0 | 0 | 0 |
| 839 | 0.000372 | 0.003216 | 0.857186 | 0.139226 | 0 | 0 | 0 | 0 | 0 |
| 840 | 0.000563 | 0.003349 | 0.855784 | 0.140304 | 0 | 0 | 0 | 0 | 0 |
| 841 | 0.000401 | 0.003335 | 0.866687 | 0.129577 | 0 | 0 | 0 | 0 | 0 |
| 842 | 0.00042 | 0.003098 | 0.857488 | 0.138994 | 0 | 0 | 0 | 0 | 0 |
| 843 | 0.000448 | 0.003448 | 0.861512 | 0.134592 | 0 | 0 | 0 | 0 | 0 |
| 844 | 0.00034 | 0.00351 | 0.861748 | 0.134402 | 0 | 0 | 0 | 0 | 0 |
| 845 | 0.00045 | 0.003117 | 0.856642 | 0.139791 | 0 | 0 | 0 | 0 | 0 |
| 846 | 0.000489 | 0.003481 | 0.856859 | 0.139171 | 0 | 0 | 0 | 0 | 0 |
| 847 | 0.000369 | 0.0034 | 0.861046 | 0.135185 | 0 | 0 | 0 | 0 | 0 |
| 848 | 0.000532 | 0.003465 | 0.862562 | 0.133441 | 0 | 0 | 0 | 0 | 0 |
| 849 | 0.000509 | 0.003197 | 0.863463 | 0.132831 | 0 | 0 | 0 | 0 | 0 |
| 850 | 0.000389 | 0.003368 | 0.860088 | 0.136155 | 0 | 0 | 0 | 0 | 0 |
| 851 | 0.000382 | 0.003391 | 0.860698 | 0.135499 | 0 | 0 | 0 | 0 | 0 |
| 852 | 0.00038 | 0.002951 | 0.859876 | 0.136793 | 0 | 0 | 0 | 0 | 0 |
| 853 | 0.000534 | 0.003105 | 0.855487 | 0.140874 | 0 | 0 | 0 | 0 | 0 |
| 854 | 0.000448 | 0.003086 | 0.858347 | 0.138118 | 0 | 0 | 0 | 0 | 0 |
| 855 | 0.000399 | 0.003535 | 0.860093 | 0.135972 | 0 | 0 | 0 | 0 | 0 |
| 856 | 0.000513 | 0.00357 | 0.854682 | 0.141235 | 0 | 0 | 0 | 0 | 0 |
| 857 | 0.00031 | 0.00322 | 0.859021 | 0.137449 | 0 | 0 | 0 | 0 | 0 |
| 858 | 0.00045 | 0.003162 | 0.861743 | 0.134645 | 0 | 0 | 0 | 0 | 0 |
| 859 | 0.000399 | 0.003441 | 0.862314 | 0.133846 | 0 | 0 | 0 | 0 | 0 |
| 860 | 0.000482 | 0.003273 | 0.860514 | 0.135731 | 0 | 0 | 0 | 0 | 0 |
| 861 | 0.000409 | 0.003489 | 0.8614 | 0.134692 | 0 | 0 | 0 | 0 | 0 |
| 862 | 0.000309 | 0.003032 | 0.861449 | 0.13521 | 0 | 0 | 0 | 0 | 0 |
| 863 | 0.000429 | 0.003284 | 0.859819 | 0.136468 | 0 | 0 | 0 | 0 | 0 |
| 864 | 0.000441 | 0.003138 | 0.858098 | 0.138322 | 0 | 0 | 0 | 0 | 0 |
| 865 | 0.00041 | 0.00307 | 0.862757 | 0.133763 | 0 | 0 | 0 | 0 | 0 |
| 866 | 0.000461 | 0.003048 | 0.862137 | 0.134354 | 0 | 0 | 0 | 0 | 0 |
| 867 | 0.000391 | 0.003076 | 0.857893 | 0.13864 | 0 | 0 | 0 | 0 | 0 |
| 868 | 0.000399 | 0.00337 | 0.857345 | 0.138886 | 0 | 0 | 0 | 0 | 0 |
| 869 | 0.000349 | 0.002984 | 0.860268 | 0.136389 | 0 | 0 | 0 | 0 | 0 |
| 870 | 0.000541 | 0.002785 | 0.858474 | 0.138201 | 0 | 0 | 0 | 0 | 0 |
| 871 | 0.000433 | 0.002898 | 0.86492 | 0.131749 | 0 | 0 | 0 | 0 | 0 |
| 872 | 0.000392 | 0.003016 | 0.859294 | 0.137297 | 0 | 0 | 0 | 0 | 0 |
| 873 | 0.00044 | 0.003282 | 0.861646 | 0.134631 | 0 | 0 | 0 | 0 | 0 |
| 874 | 0.00056 | 0.002838 | 0.860462 | 0.13614 | 0 | 0 | 0 | 0 | 0 |
| 875 | 0.000412 | 0.003072 | 0.863727 | 0.132789 | 0 | 0 | 0 | 0 | 0 |
| 876 | 0.000418 | 0.003378 | 0.860674 | 0.13553 | 0 | 0 | 0 | 0 | 0 |
| 877 | 0.000424 | 0.003082 | 0.861769 | 0.134725 | 0 | 0 | 0 | 0 | 0 |
| 878 | 0.00053 | 0.003072 | 0.861707 | 0.13469 | 0 | 0 | 0 | 0 | 0 |
| 879 | 0.000431 | 0.003134 | 0.860285 | 0.13615 | 0 | 0 | 0 | 0 | 0 |
| 880 | 0.00039 | 0.002917 | 0.861179 | 0.135514 | 0 | 0 | 0 | 0 | 0 |
| 881 | 0.00038 | 0.00268 | 0.857339 | 0.139601 | 0 | 0 | 0 | 0 | 0 |
| 882 | 0.000411 | 0.003135 | 0.858434 | 0.13799 | 0 | 0 | 0 | 0 | 0 |
| 883 | 0.00046 | 0.003179 | 0.859001 | 0.137351 | 0 | 0 | 0 | 0 | 0 |
| 884 | 0.000429 | 0.00324 | 0.859305 | 0.137026 | 0 | 0 | 0 | 0 | 0 |
| 885 | 0.000359 | 0.003362 | 0.862529 | 0.133749 | 0 | 0 | 0 | 0 | 0 |
| 886 | 0.00046 | 0.003318 | 0.858926 | 0.137296 | 0 | 0 | 0 | 0 | 0 |
| 887 | 0.000379 | 0.003071 | 0.862075 | 0.134475 | 0 | 0 | 0 | 0 | 0 |
| 888 | 0.000401 | 0.003287 | 0.855996 | 0.140316 | 0 | 0 | 0 | 0 | 0 |
| 889 | 0.000419 | 0.003231 | 0.856559 | 0.139792 | 0 | 0 | 0 | 0 | 0 |
| 890 | 0.000461 | 0.003304 | 0.863012 | 0.133223 | 0 | 0 | 0 | 0 | 0 |
| 891 | 0.000459 | 0.003385 | 0.858729 | 0.137426 | 0 | 0 | 0 | 0 | 0 |
| 892 | 0.00036 | 0.0031 | 0.862272 | 0.134269 | 0 | 0 | 0 | 0 | 0 |
| 893 | 0.00046 | 0.00318 | 0.858857 | 0.137503 | 0 | 0 | 0 | 0 | 0 |
| 894 | 0.00026 | 0.0035 | 0.859724 | 0.136515 | 0 | 0 | 0 | 0 | 0 |
| 895 | 0.000469 | 0.003203 | 0.865114 | 0.131214 | 0 | 0 | 0 | 0 | 0 |
| 896 | 0.000461 | 0.003099 | 0.862631 | 0.133809 | 0 | 0 | 0 | 0 | 0 |
| 897 | 0.000382 | 0.003487 | 0.860994 | 0.135137 | 0 | 0 | 0 | 0 | 0 |
| 898 | 0.000369 | 0.002919 | 0.860193 | 0.136519 | 0 | 0 | 0 | 0 | 0 |
| 899 | 0.00054 | 0.003068 | 0.859465 | 0.136927 | 0 | 0 | 0 | 0 | 0 |
| 900 | 0.000441 | 0.003696 | 0.859017 | 0.136846 | 0 | 0 | 0 | 0 | 0 |
| 901 | 0.000379 | 0.0032 | 0.866316 | 0.130105 | 0 | 0 | 0 | 0 | 0 |
| 902 | 0.00046 | 0.002707 | 0.861968 | 0.134865 | 0 | 0 | 0 | 0 | 0 |
| 903 | 0.000431 | 0.003315 | 0.861625 | 0.134629 | 0 | 0 | 0 | 0 | 0 |
| 904 | 0.000361 | 0.003027 | 0.862948 | 0.133665 | 0 | 0 | 0 | 0 | 0 |
| 905 | 0.00045 | 0.003421 | 0.86185 | 0.134278 | 0 | 0 | 0 | 0 | 0 |
| 906 | 0.00028 | 0.003165 | 0.864239 | 0.132316 | 0 | 0 | 0 | 0 | 0 |
| 907 | 0.000537 | 0.00321 | 0.863925 | 0.132328 | 0 | 0 | 0 | 0 | 0 |
| 908 | 0.00041 | 0.003287 | 0.861341 | 0.134962 | 0 | 0 | 0 | 0 | 0 |
| 909 | 0.000427 | 0.003067 | 0.861079 | 0.135428 | 0 | 0 | 0 | 0 | 0 |
| 910 | 0.000469 | 0.00337 | 0.85878 | 0.137382 | 0 | 0 | 0 | 0 | 0 |
| 911 | 0.000527 | 0.00304 | 0.864998 | 0.131436 | 0 | 0 | 0 | 0 | 0 |
| 912 | 0.000501 | 0.003144 | 0.857064 | 0.139291 | 0 | 0 | 0 | 0 | 0 |
| 913 | 0.000511 | 0.003273 | 0.863098 | 0.133118 | 0 | 0 | 0 | 0 | 0 |
| 914 | 0.000361 | 0.003306 | 0.866066 | 0.130268 | 0 | 0 | 0 | 0 | 0 |
| 915 | 0.00041 | 0.002618 | 0.858679 | 0.138294 | 0 | 0 | 0 | 0 | 0 |
| 916 | 0.000299 | 0.003543 | 0.861059 | 0.1351 | 0 | 0 | 0 | 0 | 0 |
| 917 | 0.000417 | 0.00301 | 0.861024 | 0.135548 | 0 | 0 | 0 | 0 | 0 |
| 918 | 0.000509 | 0.002955 | 0.86339 | 0.133145 | 0 | 0 | 0 | 0 | 0 |
| 919 | 0.000408 | 0.003317 | 0.8576 | 0.138676 | 0 | 0 | 0 | 0 | 0 |
| 920 | 0.000402 | 0.003385 | 0.863827 | 0.132386 | 0 | 0 | 0 | 0 | 0 |
| 921 | 0.000479 | 0.002797 | 0.860157 | 0.136567 | 0 | 0 | 0 | 0 | 0 |
| 922 | 0.000419 | 0.003114 | 0.864468 | 0.131998 | 0 | 0 | 0 | 0 | 0 |
| 923 | 0.000512 | 0.00315 | 0.862786 | 0.133553 | 0 | 0 | 0 | 0 | 0 |
| 924 | 0.000379 | 0.003278 | 0.8587 | 0.137643 | 0 | 0 | 0 | 0 | 0 |
| 925 | 0.000427 | 0.003032 | 0.861912 | 0.134599 | 0 | 0 | 0 | 0 | 0 |
| 926 | 0.000452 | 0.003397 | 0.861085 | 0.135066 | 0 | 0 | 0 | 0 | 0 |
| 927 | 0.000509 | 0.003064 | 0.862 | 0.134427 | 0 | 0 | 0 | 0 | 0 |
| 928 | 0.000479 | 0.003175 | 0.858424 | 0.137922 | 0 | 0 | 0 | 0 | 0 |
| 929 | 0.00032 | 0.003267 | 0.856562 | 0.139851 | 0 | 0 | 0 | 0 | 0 |
| 930 | 0.000372 | 0.00389 | 0.862064 | 0.133674 | 0 | 0 | 0 | 0 | 0 |
| 931 | 0.00026 | 0.003155 | 0.864099 | 0.132485 | 0 | 0 | 0 | 0 | 0 |
| 932 | 0.00044 | 0.003209 | 0.860659 | 0.135693 | 0 | 0 | 0 | 0 | 0 |
| 933 | 0.000622 | 0.003238 | 0.864658 | 0.131482 | 0 | 0 | 0 | 0 | 0 |
| 934 | 0.000349 | 0.002977 | 0.858459 | 0.138215 | 0 | 0 | 0 | 0 | 0 |
| 935 | 0.000348 | 0.003576 | 0.861313 | 0.134764 | 0 | 0 | 0 | 0 | 0 |
| 936 | 0.000372 | 0.002862 | 0.861267 | 0.1355 | 0 | 0 | 0 | 0 | 0 |
| 937 | 0.00038 | 0.003214 | 0.865647 | 0.130758 | 0 | 0 | 0 | 0 | 0 |
| 938 | 0.000351 | 0.003201 | 0.862066 | 0.134371 | 0 | 0 | 0 | 0 | 0 |
| 939 | 0.000461 | 0.002837 | 0.865717 | 0.130985 | 0 | 0 | 0 | 0 | 0 |
| 940 | 0.000552 | 0.003425 | 0.855394 | 0.140629 | 0 | 0 | 0 | 0 | 0 |
| 941 | 0.00033 | 0.00343 | 0.860872 | 0.135368 | 0 | 0 | 0 | 0 | 0 |
| 942 | 0.000301 | 0.003454 | 0.862974 | 0.133271 | 0 | 0 | 0 | 0 | 0 |
| 943 | 0.000561 | 0.003017 | 0.867312 | 0.12911 | 0 | 0 | 0 | 0 | 0 |
| 944 | 0.00052 | 0.003287 | 0.859101 | 0.137092 | 0 | 0 | 0 | 0 | 0 |
| 945 | 0.00046 | 0.00346 | 0.85649 | 0.13959 | 0 | 0 | 0 | 0 | 0 |
| 946 | 0.00042 | 0.00297 | 0.863978 | 0.132632 | 0 | 0 | 0 | 0 | 0 |
| 947 | 0.000399 | 0.002925 | 0.859657 | 0.137018 | 0 | 0 | 0 | 0 | 0 |
| 948 | 0.00038 | 0.003119 | 0.860992 | 0.135509 | 0 | 0 | 0 | 0 | 0 |
| 949 | 0.000363 | 0.002804 | 0.866856 | 0.129977 | 0 | 0 | 0 | 0 | 0 |
| 950 | 0.00044 | 0.003113 | 0.862302 | 0.134145 | 0 | 0 | 0 | 0 | 0 |
| 951 | 0.000489 | 0.003323 | 0.862505 | 0.133683 | 0 | 0 | 0 | 0 | 0 |
| 952 | 0.00042 | 0.003237 | 0.862996 | 0.133348 | 0 | 0 | 0 | 0 | 0 |
| 953 | 0.000521 | 0.003145 | 0.860096 | 0.136239 | 0 | 0 | 0 | 0 | 0 |
| 954 | 0.00052 | 0.003221 | 0.858391 | 0.137868 | 0 | 0 | 0 | 0 | 0 |
| 955 | 0.000502 | 0.002768 | 0.861543 | 0.135187 | 0 | 0 | 0 | 0 | 0 |
| 956 | 0.000429 | 0.003456 | 0.860573 | 0.135542 | 0 | 0 | 0 | 0 | 0 |
| 957 | 0.000441 | 0.003715 | 0.859818 | 0.136027 | 0 | 0 | 0 | 0 | 0 |
| 958 | 0.000349 | 0.003202 | 0.864759 | 0.13167 | 0 | 0 | 0 | 0 | 0 |
| 959 | 0.000301 | 0.003044 | 0.857876 | 0.138778 | 0 | 0 | 0 | 0 | 0 |
| 960 | 0.000354 | 0.003111 | 0.860878 | 0.135657 | 0 | 0 | 0 | 0 | 0 |
| 961 | 0.00051 | 0.003018 | 0.861904 | 0.134558 | 0 | 0 | 0 | 0 | 0 |
| 962 | 0.000391 | 0.003217 | 0.862366 | 0.134026 | 0 | 0 | 0 | 0 | 0 |
| 963 | 0.00048 | 0.002869 | 0.861889 | 0.134763 | 0 | 0 | 0 | 0 | 0 |
| 964 | 0.000492 | 0.003114 | 0.861135 | 0.135259 | 0 | 0 | 0 | 0 | 0 |
| 965 | 0.00054 | 0.003099 | 0.860969 | 0.135392 | 0 | 0 | 0 | 0 | 0 |
| 966 | 0.000332 | 0.003187 | 0.862119 | 0.134362 | 0 | 0 | 0 | 0 | 0 |
| 967 | 0.000279 | 0.003283 | 0.860617 | 0.135821 | 0 | 0 | 0 | 0 | 0 |
| 968 | 0.000489 | 0.003165 | 0.859983 | 0.136363 | 0 | 0 | 0 | 0 | 0 |
| 969 | 0.000408 | 0.003261 | 0.862584 | 0.133748 | 0 | 0 | 0 | 0 | 0 |
| 970 | 0.000402 | 0.003689 | 0.859474 | 0.136435 | 0 | 0 | 0 | 0 | 0 |
| 971 | 0.000611 | 0.003645 | 0.862936 | 0.132809 | 0 | 0 | 0 | 0 | 0 |
| 972 | 0.00042 | 0.003436 | 0.857905 | 0.138239 | 0 | 0 | 0 | 0 | 0 |
| 973 | 0.000492 | 0.003271 | 0.860912 | 0.135315 | 0 | 0 | 0 | 0 | 0 |
| 974 | 0.00038 | 0.002893 | 0.860322 | 0.136404 | 0 | 0 | 0 | 0 | 0 |
| 975 | 0.000502 | 0.003225 | 0.861337 | 0.134936 | 0 | 0 | 0 | 0 | 0 |
| 976 | 0.000231 | 0.002806 | 0.862412 | 0.134551 | 0 | 0 | 0 | 0 | 0 |
| 977 | 0.000492 | 0.002923 | 0.860904 | 0.135681 | 0 | 0 | 0 | 0 | 0 |
| 978 | 0.00054 | 0.002849 | 0.863375 | 0.133236 | 0 | 0 | 0 | 0 | 0 |
| 979 | 0.00043 | 0.002741 | 0.860083 | 0.136746 | 0 | 0 | 0 | 0 | 0 |
| 980 | 0.000479 | 0.002961 | 0.860576 | 0.135984 | 0 | 0 | 0 | 0 | 0 |
| 981 | 0.000427 | 0.003179 | 0.863155 | 0.133238 | 0 | 0 | 0 | 0 | 0 |
| 982 | 0.00045 | 0.003272 | 0.859224 | 0.137053 | 0 | 0 | 0 | 0 | 0 |
| 983 | 0.00043 | 0.003327 | 0.858562 | 0.137682 | 0 | 0 | 0 | 0 | 0 |
| 984 | 0.000289 | 0.003474 | 0.860612 | 0.135626 | 0 | 0 | 0 | 0 | 0 |
| 985 | 0.000596 | 0.002868 | 0.86315 | 0.133386 | 0 | 0 | 0 | 0 | 0 |
| 986 | 0.00037 | 0.003008 | 0.863468 | 0.133154 | 0 | 0 | 0 | 0 | 0 |
| 987 | 0.000438 | 0.002855 | 0.862596 | 0.134111 | 0 | 0 | 0 | 0 | 0 |
| 988 | 0.00041 | 0.003559 | 0.858691 | 0.137339 | 0 | 0 | 0 | 0 | 0 |
| 989 | 0.00026 | 0.002905 | 0.859868 | 0.136968 | 0 | 0 | 0 | 0 | 0 |
| 990 | 0.000515 | 0.003219 | 0.86262 | 0.133626 | 0 | 0 | 0 | 0 | 0 |
| 991 | 0.000462 | 0.003794 | 0.861259 | 0.134485 | 0 | 0 | 0 | 0 | 0 |
| 992 | 0.000481 | 0.003395 | 0.862713 | 0.133411 | 0 | 0 | 0 | 0 | 0 |
| 993 | 0.000398 | 0.00317 | 0.86615 | 0.130282 | 0 | 0 | 0 | 0 | 0 |
| 994 | 0.00038 | 0.002998 | 0.863914 | 0.132708 | 0 | 0 | 0 | 0 | 0 |
| 995 | 0.000439 | 0.002944 | 0.862719 | 0.133897 | 0 | 0 | 0 | 0 | 0 |
| 996 | 0.00041 | 0.002878 | 0.865191 | 0.131521 | 0 | 0 | 0 | 0 | 0 |
| 997 | 0.00044 | 0.003019 | 0.858028 | 0.138514 | 0 | 0 | 0 | 0 | 0 |
| 998 | 0.000499 | 0.002955 | 0.862726 | 0.13382 | 0 | 0 | 0 | 0 | 0 |
| 999 | 0.000409 | 0.003149 | 0.863323 | 0.133099 | 0 | 0 | 0 | 0 | 0 |
| 1000 | 0.00035 | 0.002904 | 0.861053 | 0.135693 | 0 | 0 | 0 | 0 | 0 |

| Year 5 | Race |  |  |  |  |  |  |  |  |
| --- | --- | --- | --- | --- | --- | --- | --- | --- | --- |
| Trial | 003.0 | 005.0 | 007.0 | 037.1 | 043.0 | 303.0 | 003.2 | 403.0 | 003.4 |
| 1 | 0.0001 | 0.001533 | 0.873165 | 0.125203 | 0 | 0 | 0 | 0 | 0 |
| 2 | 0.00007 | 0.001452 | 0.86941 | 0.129069 | 0 | 0 | 0 | 0 | 0 |
| 3 | 0.000091 | 0.00105 | 0.871743 | 0.127116 | 0 | 0 | 0 | 0 | 0 |
| 4 | 0.00008 | 0.001181 | 0.876477 | 0.122262 | 0 | 0 | 0 | 0 | 0 |
| 5 | 0.00007 | 0.001324 | 0.869799 | 0.128806 | 0 | 0 | 0 | 0 | 0 |
| 6 | 0.00003 | 0.000983 | 0.874087 | 0.1249 | 0 | 0 | 0 | 0 | 0 |
| 7 | 0.00001 | 0.001282 | 0.873623 | 0.125085 | 0 | 0 | 0 | 0 | 0 |
| 8 | 0.000119 | 0.001026 | 0.873777 | 0.125078 | 0 | 0 | 0 | 0 | 0 |
| 9 | 0.00006 | 0.001109 | 0.87293 | 0.125901 | 0 | 0 | 0 | 0 | 0 |
| 10 | 0.00008 | 0.001281 | 0.874382 | 0.124257 | 0 | 0 | 0 | 0 | 0 |
| 11 | 0.00008 | 0.001379 | 0.87455 | 0.12398 | 0.00001 | 0 | 0 | 0 | 0 |
| 12 | 0.00008 | 0.001225 | 0.872074 | 0.126621 | 0 | 0 | 0 | 0 | 0 |
| 13 | 0.00008 | 0.001299 | 0.871205 | 0.127416 | 0 | 0 | 0 | 0 | 0 |
| 14 | 0.000101 | 0.001549 | 0.866044 | 0.132306 | 0 | 0 | 0 | 0 | 0 |
| 15 | 0.00007 | 0.001161 | 0.868589 | 0.13018 | 0 | 0 | 0 | 0 | 0 |
| 16 | 0.00006 | 0.001293 | 0.873632 | 0.125015 | 0 | 0 | 0 | 0 | 0 |
| 17 | 0.00009 | 0.001548 | 0.872454 | 0.125909 | 0 | 0 | 0 | 0 | 0 |
| 18 | 0.00017 | 0.00113 | 0.874119 | 0.124581 | 0 | 0 | 0 | 0 | 0 |
| 19 | 0.000099 | 0.001313 | 0.87096 | 0.127628 | 0 | 0 | 0 | 0 | 0 |
| 20 | 0.00007 | 0.001373 | 0.871454 | 0.127103 | 0 | 0 | 0 | 0 | 0 |
| 21 | 0.000079 | 0.001299 | 0.873368 | 0.125254 | 0 | 0 | 0 | 0 | 0 |
| 22 | 0.00004 | 0.001455 | 0.870773 | 0.127732 | 0 | 0 | 0 | 0 | 0 |
| 23 | 0.00004 | 0.001313 | 0.873538 | 0.125109 | 0 | 0 | 0 | 0 | 0 |
| 24 | 0.00003 | 0.001422 | 0.871405 | 0.127143 | 0 | 0 | 0 | 0 | 0 |
| 25 | 0.00008 | 0.000914 | 0.87364 | 0.125366 | 0 | 0 | 0 | 0 | 0 |
| 26 | 0.00005 | 0.001301 | 0.877415 | 0.121234 | 0 | 0 | 0 | 0 | 0 |
| 27 | 0.00005 | 0.001277 | 0.871634 | 0.127039 | 0 | 0 | 0 | 0 | 0 |
| 28 | 0.00002 | 0.001061 | 0.873371 | 0.125518 | 0.00003 | 0 | 0 | 0 | 0 |
| 29 | 0.00009 | 0.001138 | 0.872892 | 0.125881 | 0 | 0 | 0 | 0 | 0 |
| 30 | 0.00003 | 0.001548 | 0.874479 | 0.123943 | 0 | 0 | 0 | 0 | 0 |
| 31 | 0.000069 | 0.001169 | 0.873093 | 0.125669 | 0 | 0 | 0 | 0 | 0 |
| 32 | 0.00003 | 0.000973 | 0.87383 | 0.125168 | 0 | 0 | 0 | 0 | 0 |
| 33 | 0.0001 | 0.001234 | 0.872212 | 0.126454 | 0 | 0 | 0 | 0 | 0 |
| 34 | 0.00005 | 0.001334 | 0.872522 | 0.126094 | 0 | 0 | 0 | 0 | 0 |
| 35 | 0.00008 | 0.001111 | 0.865125 | 0.133684 | 0 | 0 | 0 | 0 | 0 |
| 36 | 0.00006 | 0.001176 | 0.873295 | 0.12547 | 0 | 0 | 0 | 0 | 0 |
| 37 | 0.00008 | 0.001191 | 0.875088 | 0.123642 | 0 | 0 | 0 | 0 | 0 |
| 38 | 0.00007 | 0.001188 | 0.873833 | 0.124909 | 0 | 0 | 0 | 0 | 0 |
| 39 | 0.00003 | 0.001174 | 0.871547 | 0.127209 | 0.00004 | 0 | 0 | 0 | 0 |
| 40 | 0.00009 | 0.0013 | 0.87079 | 0.127819 | 0 | 0 | 0 | 0 | 0 |
| 41 | 0.00008 | 0.00117 | 0.87504 | 0.123709 | 0 | 0 | 0 | 0 | 0 |
| 42 | 0.00009 | 0.001383 | 0.876428 | 0.1221 | 0 | 0 | 0 | 0 | 0 |
| 43 | 0.00006 | 0.001138 | 0.873678 | 0.125123 | 0 | 0 | 0 | 0 | 0 |
| 44 | 0.00009 | 0.001325 | 0.873118 | 0.125467 | 0 | 0 | 0 | 0 | 0 |
| 45 | 0.00006 | 0.00138 | 0.871558 | 0.127001 | 0 | 0 | 0 | 0 | 0 |
| 46 | 0.00011 | 0.001279 | 0.870281 | 0.12833 | 0 | 0 | 0 | 0 | 0 |
| 47 | 0.00008 | 0.001277 | 0.871057 | 0.127586 | 0 | 0 | 0 | 0 | 0 |
| 48 | 0.00003 | 0.00125 | 0.870371 | 0.128349 | 0 | 0 | 0 | 0 | 0 |
| 49 | 0.000101 | 0.001352 | 0.866261 | 0.132286 | 0 | 0 | 0 | 0 | 0 |
| 50 | 0.00006 | 0.000959 | 0.872515 | 0.126466 | 0 | 0 | 0 | 0 | 0 |
| 51 | 0.00007 | 0.001494 | 0.871138 | 0.127298 | 0 | 0 | 0 | 0 | 0 |
| 52 | 0.0001 | 0.000996 | 0.873866 | 0.125039 | 0 | 0 | 0 | 0 | 0 |
| 53 | 0.00004 | 0.001253 | 0.874529 | 0.124138 | 0.00004 | 0 | 0 | 0 | 0 |
| 54 | 0.000111 | 0.000996 | 0.873665 | 0.125228 | 0 | 0 | 0 | 0 | 0 |
| 55 | 0.00008 | 0.001261 | 0.875328 | 0.123331 | 0 | 0 | 0 | 0 | 0 |
| 56 | 0.00005 | 0.001441 | 0.870206 | 0.128302 | 0 | 0 | 0 | 0 | 0 |
| 57 | 0.00008 | 0.001152 | 0.874671 | 0.124097 | 0 | 0 | 0 | 0 | 0 |
| 58 | 0.00009 | 0.001402 | 0.871416 | 0.127092 | 0 | 0 | 0 | 0 | 0 |
| 59 | 0.00007 | 0.001157 | 0.872088 | 0.126685 | 0 | 0 | 0 | 0 | 0 |
| 60 | 0.00013 | 0.001151 | 0.873925 | 0.124794 | 0 | 0 | 0 | 0 | 0 |
| 61 | 0.00007 | 0.001436 | 0.874151 | 0.124342 | 0 | 0 | 0 | 0 | 0 |
| 62 | 0.00006 | 0.001108 | 0.874031 | 0.1248 | 0 | 0 | 0 | 0 | 0 |
| 63 | 0.00008 | 0.001138 | 0.875702 | 0.12308 | 0 | 0 | 0 | 0 | 0 |
| 64 | 0.00003 | 0.001359 | 0.872736 | 0.125874 | 0 | 0 | 0 | 0 | 0 |
| 65 | 0.00007 | 0.001243 | 0.872237 | 0.126451 | 0 | 0 | 0 | 0 | 0 |
| 66 | 0.00008 | 0.001333 | 0.877101 | 0.121486 | 0 | 0 | 0 | 0 | 0 |
| 67 | 0.00011 | 0.001357 | 0.873117 | 0.125416 | 0 | 0 | 0 | 0 | 0 |
| 68 | 0.0001 | 0.001161 | 0.872017 | 0.126722 | 0 | 0 | 0 | 0 | 0 |
| 69 | 0.00008 | 0.00087 | 0.872723 | 0.126326 | 0 | 0 | 0 | 0 | 0 |
| 70 | 0.00005 | 0.001447 | 0.87317 | 0.125333 | 0 | 0 | 0 | 0 | 0 |
| 71 | 0.00012 | 0.001562 | 0.871835 | 0.126403 | 0.00008 | 0 | 0 | 0 | 0 |
| 72 | 0.00001 | 0.001604 | 0.875964 | 0.122422 | 0 | 0 | 0 | 0 | 0 |
| 73 | 0.00008 | 0.001412 | 0.872584 | 0.125924 | 0 | 0 | 0 | 0 | 0 |
| 74 | 0.00006 | 0.001282 | 0.874959 | 0.123699 | 0 | 0 | 0 | 0 | 0 |
| 75 | 0.00009 | 0.001429 | 0.872201 | 0.12628 | 0 | 0 | 0 | 0 | 0 |
| 76 | 0.00007 | 0.001455 | 0.874624 | 0.123851 | 0 | 0 | 0 | 0 | 0 |
| 77 | 0.00005 | 0.001383 | 0.87456 | 0.124007 | 0 | 0 | 0 | 0 | 0 |
| 78 | 0.000061 | 0.001362 | 0.874306 | 0.124271 | 0 | 0 | 0 | 0 | 0 |
| 79 | 0.00003 | 0.001049 | 0.8758 | 0.123121 | 0 | 0 | 0 | 0 | 0 |
| 80 | 0.00007 | 0.001033 | 0.871901 | 0.126996 | 0 | 0 | 0 | 0 | 0 |
| 81 | 0.00005 | 0.001112 | 0.871238 | 0.1276 | 0 | 0 | 0 | 0 | 0 |
| 82 | 0.000089 | 0.001152 | 0.873435 | 0.125324 | 0 | 0 | 0 | 0 | 0 |
| 83 | 0.00003 | 0.000996 | 0.875865 | 0.123109 | 0 | 0 | 0 | 0 | 0 |
| 84 | 0.0001 | 0.001261 | 0.875943 | 0.122696 | 0 | 0 | 0 | 0 | 0 |
| 85 | 0.00007 | 0.001218 | 0.872526 | 0.126187 | 0 | 0 | 0 | 0 | 0 |
| 86 | 0.00006 | 0.001276 | 0.879128 | 0.119537 | 0 | 0 | 0 | 0 | 0 |
| 87 | 0.00004 | 0.001316 | 0.870204 | 0.128441 | 0 | 0 | 0 | 0 | 0 |
| 88 | 0.00005 | 0.001177 | 0.873883 | 0.124859 | 0.00003 | 0 | 0 | 0 | 0 |
| 89 | 0.00004 | 0.001314 | 0.872335 | 0.126311 | 0 | 0 | 0 | 0 | 0 |
| 90 | 0.00007 | 0.00112 | 0.87232 | 0.126471 | 0.00002 | 0 | 0 | 0 | 0 |
| 91 | 0.00005 | 0.001169 | 0.872525 | 0.126256 | 0 | 0 | 0 | 0 | 0 |
| 92 | 0.00008 | 0.001181 | 0.870692 | 0.128047 | 0 | 0 | 0 | 0 | 0 |
| 93 | 0.00007 | 0.001409 | 0.87241 | 0.126111 | 0 | 0 | 0 | 0 | 0 |
| 94 | 0.00005 | 0.001188 | 0.873143 | 0.125619 | 0 | 0 | 0 | 0 | 0 |
| 95 | 0.00005 | 0.001311 | 0.868936 | 0.129702 | 0 | 0 | 0 | 0 | 0 |
| 96 | 0.00009 | 0.00114 | 0.871036 | 0.127734 | 0 | 0 | 0 | 0 | 0 |
| 97 | 0.00007 | 0.001193 | 0.871239 | 0.127498 | 0 | 0 | 0 | 0 | 0 |
| 98 | 0.00006 | 0.001182 | 0.871571 | 0.127187 | 0 | 0 | 0 | 0 | 0 |
| 99 | 0.00005 | 0.00119 | 0.871028 | 0.127732 | 0 | 0 | 0 | 0 | 0 |
| 100 | 0.00009 | 0.001241 | 0.876199 | 0.12247 | 0 | 0 | 0 | 0 | 0 |
| 101 | 0.0001 | 0.001212 | 0.875245 | 0.123442 | 0 | 0 | 0 | 0 | 0 |
| 102 | 0.00007 | 0.001176 | 0.875823 | 0.122931 | 0 | 0 | 0 | 0 | 0 |
| 103 | 0.00013 | 0.00123 | 0.870208 | 0.128433 | 0 | 0 | 0 | 0 | 0 |
| 104 | 0.00008 | 0.001358 | 0.875672 | 0.12287 | 0.00002 | 0 | 0 | 0 | 0 |
| 105 | 0.00009 | 0.001355 | 0.873863 | 0.124691 | 0 | 0 | 0 | 0 | 0 |
| 106 | 0.00004 | 0.001438 | 0.869081 | 0.129441 | 0 | 0 | 0 | 0 | 0 |
| 107 | 0.00007 | 0.00097 | 0.873387 | 0.125454 | 0.00012 | 0 | 0 | 0 | 0 |
| 108 | 0.00005 | 0.001178 | 0.870523 | 0.12825 | 0 | 0 | 0 | 0 | 0 |
| 109 | 0.00007 | 0.001269 | 0.875112 | 0.123549 | 0 | 0 | 0 | 0 | 0 |
| 110 | 0.0001 | 0.00119 | 0.875459 | 0.123251 | 0 | 0 | 0 | 0 | 0 |
| 111 | 0.00009 | 0.001161 | 0.874965 | 0.123784 | 0 | 0 | 0 | 0 | 0 |
| 112 | 0.00008 | 0.001044 | 0.871496 | 0.127381 | 0 | 0 | 0 | 0 | 0 |
| 113 | 0.00008 | 0.000938 | 0.873553 | 0.125429 | 0 | 0 | 0 | 0 | 0 |
| 114 | 0.0001 | 0.00128 | 0.87598 | 0.12264 | 0 | 0 | 0 | 0 | 0 |
| 115 | 0.00007 | 0.001033 | 0.879311 | 0.119586 | 0 | 0 | 0 | 0 | 0 |
| 116 | 0.0001 | 0.001265 | 0.876081 | 0.122555 | 0 | 0 | 0 | 0 | 0 |
| 117 | 0.0001 | 0.001186 | 0.873996 | 0.124718 | 0 | 0 | 0 | 0 | 0 |
| 118 | 0.00004 | 0.001447 | 0.875387 | 0.123126 | 0 | 0 | 0 | 0 | 0 |
| 119 | 0.00008 | 0.001159 | 0.872941 | 0.12582 | 0 | 0 | 0 | 0 | 0 |
| 120 | 0.00004 | 0.001236 | 0.871812 | 0.126912 | 0 | 0 | 0 | 0 | 0 |
| 121 | 0.00004 | 0.00127 | 0.876022 | 0.122668 | 0 | 0 | 0 | 0 | 0 |
| 122 | 0.00006 | 0.001237 | 0.876324 | 0.122379 | 0 | 0 | 0 | 0 | 0 |
| 123 | 0.00003 | 0.00119 | 0.871778 | 0.127002 | 0 | 0 | 0 | 0 | 0 |
| 124 | 0.00004 | 0.00111 | 0.877858 | 0.120992 | 0 | 0 | 0 | 0 | 0 |
| 125 | 0.00008 | 0.001215 | 0.872893 | 0.125812 | 0 | 0 | 0 | 0 | 0 |
| 126 | 0.000109 | 0.00123 | 0.870127 | 0.128533 | 0 | 0 | 0 | 0 | 0 |
| 127 | 0.00006 | 0.001131 | 0.875826 | 0.122983 | 0 | 0 | 0 | 0 | 0 |
| 128 | 0.00006 | 0.000985 | 0.873556 | 0.125399 | 0 | 0 | 0 | 0 | 0 |
| 129 | 0.00004 | 0.001325 | 0.868392 | 0.130243 | 0 | 0 | 0 | 0 | 0 |
| 130 | 0.00006 | 0.001002 | 0.87367 | 0.125268 | 0 | 0 | 0 | 0 | 0 |
| 131 | 0.00006 | 0.001148 | 0.876464 | 0.122327 | 0 | 0 | 0 | 0 | 0 |
| 132 | 0.00009 | 0.00116 | 0.873779 | 0.124971 | 0 | 0 | 0 | 0 | 0 |
| 133 | 0.00003 | 0.00117 | 0.871318 | 0.127482 | 0 | 0 | 0 | 0 | 0 |
| 134 | 0.00005 | 0.001489 | 0.87472 | 0.123741 | 0 | 0 | 0 | 0 | 0 |
| 135 | 0.00006 | 0.001304 | 0.868316 | 0.13032 | 0 | 0 | 0 | 0 | 0 |
| 136 | 0.00006 | 0.001195 | 0.874961 | 0.123784 | 0 | 0 | 0 | 0 | 0 |
| 137 | 0.00011 | 0.001096 | 0.87198 | 0.126814 | 0 | 0 | 0 | 0 | 0 |
| 138 | 0.000101 | 0.00156 | 0.870106 | 0.128233 | 0 | 0 | 0 | 0 | 0 |
| 139 | 0.00008 | 0.001329 | 0.875144 | 0.123447 | 0 | 0 | 0 | 0 | 0 |
| 140 | 0.000101 | 0.001367 | 0.870722 | 0.12781 | 0 | 0 | 0 | 0 | 0 |
| 141 | 0.00006 | 0.001157 | 0.872709 | 0.126075 | 0 | 0 | 0 | 0 | 0 |
| 142 | 0.00006 | 0.001449 | 0.869205 | 0.129287 | 0 | 0 | 0 | 0 | 0 |
| 143 | 0.00007 | 0.001251 | 0.872727 | 0.125952 | 0 | 0 | 0 | 0 | 0 |
| 144 | 0.00004 | 0.001438 | 0.871763 | 0.126759 | 0 | 0 | 0 | 0 | 0 |
| 145 | 0.00007 | 0.001112 | 0.874536 | 0.124283 | 0 | 0 | 0 | 0 | 0 |
| 146 | 0.00008 | 0.001021 | 0.877441 | 0.121458 | 0 | 0 | 0 | 0 | 0 |
| 147 | 0.00005 | 0.001102 | 0.872292 | 0.126556 | 0 | 0 | 0 | 0 | 0 |
| 148 | 0.00007 | 0.001139 | 0.872066 | 0.126725 | 0 | 0 | 0 | 0 | 0 |
| 149 | 0.00008 | 0.00102 | 0.871824 | 0.127076 | 0 | 0 | 0 | 0 | 0 |
| 150 | 0.00009 | 0.001229 | 0.872542 | 0.126139 | 0 | 0 | 0 | 0 | 0 |
| 151 | 0.00006 | 0.001102 | 0.872548 | 0.12629 | 0 | 0 | 0 | 0 | 0 |
| 152 | 0.00004 | 0.001257 | 0.869724 | 0.128978 | 0 | 0 | 0 | 0 | 0 |
| 153 | 0.00004 | 0.001082 | 0.876415 | 0.122463 | 0 | 0 | 0 | 0 | 0 |
| 154 | 0.000059 | 0.001187 | 0.870415 | 0.128338 | 0 | 0 | 0 | 0 | 0 |
| 155 | 0.000101 | 0.001108 | 0.870297 | 0.128494 | 0 | 0 | 0 | 0 | 0 |
| 156 | 0.00008 | 0.001483 | 0.869585 | 0.128852 | 0 | 0 | 0 | 0 | 0 |
| 157 | 0.000111 | 0.001362 | 0.873194 | 0.125333 | 0 | 0 | 0 | 0 | 0 |
| 158 | 0.00009 | 0.001122 | 0.872338 | 0.12645 | 0 | 0 | 0 | 0 | 0 |
| 159 | 0.00008 | 0.001233 | 0.870371 | 0.128316 | 0 | 0 | 0 | 0 | 0 |
| 160 | 0.00011 | 0.001255 | 0.87826 | 0.120375 | 0 | 0 | 0 | 0 | 0 |
| 161 | 0.00006 | 0.00122 | 0.871522 | 0.127198 | 0 | 0 | 0 | 0 | 0 |
| 162 | 0.00008 | 0.001355 | 0.872416 | 0.126148 | 0 | 0 | 0 | 0 | 0 |
| 163 | 0.00005 | 0.001157 | 0.873553 | 0.125241 | 0 | 0 | 0 | 0 | 0 |
| 164 | 0.00005 | 0.001174 | 0.8724 | 0.126376 | 0 | 0 | 0 | 0 | 0 |
| 165 | 0.00001 | 0.001373 | 0.874716 | 0.1239 | 0 | 0 | 0 | 0 | 0 |
| 166 | 0.00008 | 0.001263 | 0.870516 | 0.128141 | 0 | 0 | 0 | 0 | 0 |
| 167 | 0.00012 | 0.001234 | 0.872755 | 0.12589 | 0 | 0 | 0 | 0 | 0 |
| 168 | 0.000071 | 0.001293 | 0.873465 | 0.125172 | 0 | 0 | 0 | 0 | 0 |
| 169 | 0.00015 | 0.001322 | 0.871047 | 0.12748 | 0 | 0 | 0 | 0 | 0 |
| 170 | 0.00007 | 0.001435 | 0.873247 | 0.125248 | 0 | 0 | 0 | 0 | 0 |
| 171 | 0.00009 | 0.001186 | 0.873151 | 0.125574 | 0 | 0 | 0 | 0 | 0 |
| 172 | 0.00006 | 0.001311 | 0.872049 | 0.12658 | 0 | 0 | 0 | 0 | 0 |
| 173 | 0.00004 | 0.001599 | 0.872531 | 0.12583 | 0 | 0 | 0 | 0 | 0 |
| 174 | 0.00003 | 0.001478 | 0.87012 | 0.128372 | 0 | 0 | 0 | 0 | 0 |
| 175 | 0.00006 | 0.001162 | 0.872761 | 0.126018 | 0 | 0 | 0 | 0 | 0 |
| 176 | 0.00009 | 0.001163 | 0.877817 | 0.120919 | 0.00001 | 0 | 0 | 0 | 0 |
| 177 | 0.00007 | 0.001281 | 0.870839 | 0.12781 | 0 | 0 | 0 | 0 | 0 |
| 178 | 0.00004 | 0.001336 | 0.87013 | 0.128494 | 0 | 0 | 0 | 0 | 0 |
| 179 | 0.00011 | 0.001416 | 0.868809 | 0.129664 | 0 | 0 | 0 | 0 | 0 |
| 180 | 0.0001 | 0.001311 | 0.87256 | 0.126029 | 0 | 0 | 0 | 0 | 0 |
| 181 | 0.00005 | 0.00123 | 0.874757 | 0.123963 | 0 | 0 | 0 | 0 | 0 |
| 182 | 0.00011 | 0.001409 | 0.875201 | 0.123281 | 0 | 0 | 0 | 0 | 0 |
| 183 | 0.00004 | 0.001121 | 0.871461 | 0.127378 | 0 | 0 | 0 | 0 | 0 |
| 184 | 0.000109 | 0.000975 | 0.877445 | 0.12147 | 0 | 0 | 0 | 0 | 0 |
| 185 | 0.00007 | 0.001168 | 0.873302 | 0.12546 | 0 | 0 | 0 | 0 | 0 |
| 186 | 0.00006 | 0.001188 | 0.873042 | 0.12571 | 0 | 0 | 0 | 0 | 0 |
| 187 | 0.00007 | 0.001354 | 0.878468 | 0.120108 | 0 | 0 | 0 | 0 | 0 |
| 188 | 0.00008 | 0.001077 | 0.872112 | 0.126731 | 0 | 0 | 0 | 0 | 0 |
| 189 | 0.000109 | 0.001184 | 0.870969 | 0.127738 | 0 | 0 | 0 | 0 | 0 |
| 190 | 0.00008 | 0.00103 | 0.874319 | 0.124571 | 0 | 0 | 0 | 0 | 0 |
| 191 | 0.00007 | 0.001429 | 0.872096 | 0.126405 | 0 | 0 | 0 | 0 | 0 |
| 192 | 0.00005 | 0.000967 | 0.873948 | 0.125034 | 0 | 0 | 0 | 0 | 0 |
| 193 | 0.00008 | 0.001301 | 0.871348 | 0.127271 | 0 | 0 | 0 | 0 | 0 |
| 194 | 0.00003 | 0.001124 | 0.870022 | 0.128824 | 0 | 0 | 0 | 0 | 0 |
| 195 | 0.00006 | 0.001148 | 0.870318 | 0.128475 | 0 | 0 | 0 | 0 | 0 |
| 196 | 0.00008 | 0.001388 | 0.870665 | 0.127867 | 0 | 0 | 0 | 0 | 0 |
| 197 | 0.000079 | 0.00131 | 0.874813 | 0.123798 | 0 | 0 | 0 | 0 | 0 |
| 198 | 0.00005 | 0.001392 | 0.871927 | 0.126591 | 0.00004 | 0 | 0 | 0 | 0 |
| 199 | 0.00008 | 0.001284 | 0.875409 | 0.123227 | 0 | 0 | 0 | 0 | 0 |
| 200 | 0.0001 | 0.001494 | 0.869195 | 0.129211 | 0 | 0 | 0 | 0 | 0 |
| 201 | 0.00009 | 0.001504 | 0.873329 | 0.125076 | 0 | 0 | 0 | 0 | 0 |
| 202 | 0.00009 | 0.001385 | 0.874621 | 0.123904 | 0 | 0 | 0 | 0 | 0 |
| 203 | 0.00007 | 0.001073 | 0.875009 | 0.123848 | 0 | 0 | 0 | 0 | 0 |
| 204 | 0.00003 | 0.001457 | 0.872989 | 0.125524 | 0 | 0 | 0 | 0 | 0 |
| 205 | 0.00008 | 0.001018 | 0.866265 | 0.132637 | 0 | 0 | 0 | 0 | 0 |
| 206 | 0.00005 | 0.001237 | 0.869351 | 0.129362 | 0 | 0 | 0 | 0 | 0 |
| 207 | 0.00012 | 0.00112 | 0.875878 | 0.122882 | 0 | 0 | 0 | 0 | 0 |
| 208 | 0.00005 | 0.001221 | 0.878231 | 0.120497 | 0 | 0 | 0 | 0 | 0 |
| 209 | 0.00004 | 0.001166 | 0.870147 | 0.128646 | 0 | 0 | 0 | 0 | 0 |
| 210 | 0.00007 | 0.001236 | 0.872504 | 0.126189 | 0 | 0 | 0 | 0 | 0 |
| 211 | 0.00005 | 0.001395 | 0.873292 | 0.125263 | 0 | 0 | 0 | 0 | 0 |
| 212 | 0.00008 | 0.001359 | 0.872207 | 0.126354 | 0 | 0 | 0 | 0 | 0 |
| 213 | 0.00006 | 0.00093 | 0.876514 | 0.122497 | 0 | 0 | 0 | 0 | 0 |
| 214 | 0.00003 | 0.001172 | 0.874777 | 0.124021 | 0 | 0 | 0 | 0 | 0 |
| 215 | 0.00007 | 0.001289 | 0.872634 | 0.126007 | 0 | 0 | 0 | 0 | 0 |
| 216 | 0.000091 | 0.001418 | 0.875277 | 0.123215 | 0 | 0 | 0 | 0 | 0 |
| 217 | 0.00011 | 0.001221 | 0.874209 | 0.124459 | 0 | 0 | 0 | 0 | 0 |
| 218 | 0.00005 | 0.001049 | 0.871636 | 0.127265 | 0 | 0 | 0 | 0 | 0 |
| 219 | 0.00008 | 0.000968 | 0.87946 | 0.119493 | 0 | 0 | 0 | 0 | 0 |
| 220 | 0.00006 | 0.001266 | 0.874473 | 0.124201 | 0 | 0 | 0 | 0 | 0 |
| 221 | 0.00005 | 0.001186 | 0.871273 | 0.127491 | 0 | 0 | 0 | 0 | 0 |
| 222 | 0.00004 | 0.001199 | 0.874056 | 0.124705 | 0 | 0 | 0 | 0 | 0 |
| 223 | 0.00005 | 0.001192 | 0.872569 | 0.126189 | 0 | 0 | 0 | 0 | 0 |
| 224 | 0.00005 | 0.001222 | 0.872099 | 0.126629 | 0 | 0 | 0 | 0 | 0 |
| 225 | 0.00007 | 0.001259 | 0.87633 | 0.122341 | 0 | 0 | 0 | 0 | 0 |
| 226 | 0.00003 | 0.000967 | 0.877602 | 0.121401 | 0 | 0 | 0 | 0 | 0 |
| 227 | 0.000071 | 0.00109 | 0.872127 | 0.126713 | 0 | 0 | 0 | 0 | 0 |
| 228 | 0.00008 | 0.001203 | 0.874451 | 0.124265 | 0 | 0 | 0 | 0 | 0 |
| 229 | 0.000099 | 0.001143 | 0.87504 | 0.123718 | 0 | 0 | 0 | 0 | 0 |
| 230 | 0.000079 | 0.001089 | 0.870639 | 0.128192 | 0 | 0 | 0 | 0 | 0 |
| 231 | 0.00006 | 0.001381 | 0.874413 | 0.124146 | 0 | 0 | 0 | 0 | 0 |
| 232 | 0.00005 | 0.001351 | 0.870994 | 0.127605 | 0 | 0 | 0 | 0 | 0 |
| 233 | 0.00006 | 0.001406 | 0.874155 | 0.124379 | 0 | 0 | 0 | 0 | 0 |
| 234 | 0.00007 | 0.001297 | 0.872574 | 0.12606 | 0 | 0 | 0 | 0 | 0 |
| 235 | 0.00008 | 0.001269 | 0.877826 | 0.120805 | 0.00002 | 0 | 0 | 0 | 0 |
| 236 | 0.00011 | 0.001102 | 0.869285 | 0.129503 | 0 | 0 | 0 | 0 | 0 |
| 237 | 0.00004 | 0.001298 | 0.872459 | 0.126203 | 0 | 0 | 0 | 0 | 0 |
| 238 | 0.00009 | 0.001482 | 0.868999 | 0.12943 | 0 | 0 | 0 | 0 | 0 |
| 239 | 0.00005 | 0.00106 | 0.869777 | 0.129113 | 0 | 0 | 0 | 0 | 0 |
| 240 | 0.00011 | 0.001105 | 0.874454 | 0.124332 | 0 | 0 | 0 | 0 | 0 |
| 241 | 0.00004 | 0.001075 | 0.876577 | 0.122308 | 0 | 0 | 0 | 0 | 0 |
| 242 | 0.00005 | 0.00129 | 0.872288 | 0.126351 | 0.00002 | 0 | 0 | 0 | 0 |
| 243 | 0.00005 | 0.000991 | 0.876375 | 0.122584 | 0 | 0 | 0 | 0 | 0 |
| 244 | 0.00008 | 0.001024 | 0.872725 | 0.126172 | 0 | 0 | 0 | 0 | 0 |
| 245 | 0.00003 | 0.001419 | 0.872466 | 0.126065 | 0.00002 | 0 | 0 | 0 | 0 |
| 246 | 0.00008 | 0.001216 | 0.8729 | 0.125804 | 0 | 0 | 0 | 0 | 0 |
| 247 | 0.000101 | 0.001282 | 0.868234 | 0.130383 | 0 | 0 | 0 | 0 | 0 |
| 248 | 0.00002 | 0.001557 | 0.875107 | 0.123316 | 0 | 0 | 0 | 0 | 0 |
| 249 | 0.00006 | 0.00128 | 0.8746 | 0.12406 | 0 | 0 | 0 | 0 | 0 |
| 250 | 0.00013 | 0.001451 | 0.869896 | 0.128523 | 0 | 0 | 0 | 0 | 0 |
| 251 | 0.00008 | 0.001366 | 0.872754 | 0.1258 | 0 | 0 | 0 | 0 | 0 |
| 252 | 0.00009 | 0.00128 | 0.874508 | 0.124123 | 0 | 0 | 0 | 0 | 0 |
| 253 | 0.0001 | 0.001203 | 0.87164 | 0.127057 | 0 | 0 | 0 | 0 | 0 |
| 254 | 0.00007 | 0.001372 | 0.872883 | 0.125675 | 0 | 0 | 0 | 0 | 0 |
| 255 | 0.00004 | 0.001349 | 0.872136 | 0.126475 | 0 | 0 | 0 | 0 | 0 |
| 256 | 0.00006 | 0.001244 | 0.867973 | 0.130723 | 0 | 0 | 0 | 0 | 0 |
| 257 | 0.00004 | 0.001153 | 0.871917 | 0.12688 | 0.00001 | 0 | 0 | 0 | 0 |
| 258 | 0.0001 | 0.001181 | 0.871307 | 0.127411 | 0 | 0 | 0 | 0 | 0 |
| 259 | 0.00002 | 0.001322 | 0.872925 | 0.125733 | 0 | 0 | 0 | 0 | 0 |
| 260 | 0.00007 | 0.001138 | 0.874199 | 0.124593 | 0 | 0 | 0 | 0 | 0 |
| 261 | 0.00003 | 0.001301 | 0.875281 | 0.123388 | 0 | 0 | 0 | 0 | 0 |
| 262 | 0.00005 | 0.001301 | 0.872451 | 0.126198 | 0 | 0 | 0 | 0 | 0 |
| 263 | 0.00015 | 0.001179 | 0.872383 | 0.126289 | 0 | 0 | 0 | 0 | 0 |
| 264 | 0.00005 | 0.000849 | 0.8713 | 0.127801 | 0 | 0 | 0 | 0 | 0 |
| 265 | 0.00004 | 0.001354 | 0.871025 | 0.127582 | 0 | 0 | 0 | 0 | 0 |
| 266 | 0.00011 | 0.00138 | 0.874483 | 0.124028 | 0 | 0 | 0 | 0 | 0 |
| 267 | 0.00006 | 0.00102 | 0.873441 | 0.125479 | 0 | 0 | 0 | 0 | 0 |
| 268 | 0.0001 | 0.001434 | 0.873019 | 0.125447 | 0 | 0 | 0 | 0 | 0 |
| 269 | 0.000121 | 0.001316 | 0.872411 | 0.126153 | 0 | 0 | 0 | 0 | 0 |
| 270 | 0.00006 | 0.001154 | 0.872304 | 0.126483 | 0 | 0 | 0 | 0 | 0 |
| 271 | 0.00005 | 0.001249 | 0.873322 | 0.12535 | 0.00003 | 0 | 0 | 0 | 0 |
| 272 | 0.00007 | 0.001072 | 0.87111 | 0.127748 | 0 | 0 | 0 | 0 | 0 |
| 273 | 0.0001 | 0.001175 | 0.87556 | 0.123164 | 0 | 0 | 0 | 0 | 0 |
| 274 | 0.00003 | 0.001336 | 0.875905 | 0.122729 | 0 | 0 | 0 | 0 | 0 |
| 275 | 0.00005 | 0.00132 | 0.873035 | 0.125595 | 0 | 0 | 0 | 0 | 0 |
| 276 | 0.00003 | 0.00106 | 0.874659 | 0.124251 | 0 | 0 | 0 | 0 | 0 |
| 277 | 0.00004 | 0.001542 | 0.868776 | 0.129641 | 0 | 0 | 0 | 0 | 0 |
| 278 | 0.00008 | 0.001283 | 0.872906 | 0.125731 | 0 | 0 | 0 | 0 | 0 |
| 279 | 0.00004 | 0.001124 | 0.870668 | 0.128168 | 0 | 0 | 0 | 0 | 0 |
| 280 | 0.00007 | 0.001411 | 0.871956 | 0.126563 | 0 | 0 | 0 | 0 | 0 |
| 281 | 0.00007 | 0.001287 | 0.870749 | 0.127894 | 0 | 0 | 0 | 0 | 0 |
| 282 | 0.00007 | 0.001175 | 0.87392 | 0.124836 | 0 | 0 | 0 | 0 | 0 |
| 283 | 0.00008 | 0.001322 | 0.87599 | 0.122608 | 0 | 0 | 0 | 0 | 0 |
| 284 | 0.00007 | 0.001104 | 0.875782 | 0.123044 | 0 | 0 | 0 | 0 | 0 |
| 285 | 0.00004 | 0.001112 | 0.870229 | 0.128619 | 0 | 0 | 0 | 0 | 0 |
| 286 | 0.00005 | 0.001222 | 0.876637 | 0.122091 | 0 | 0 | 0 | 0 | 0 |
| 287 | 0.00011 | 0.001481 | 0.875264 | 0.123145 | 0 | 0 | 0 | 0 | 0 |
| 288 | 0.00005 | 0.001072 | 0.873937 | 0.124941 | 0 | 0 | 0 | 0 | 0 |
| 289 | 0.00003 | 0.001392 | 0.873579 | 0.124999 | 0 | 0 | 0 | 0 | 0 |
| 290 | 0.00008 | 0.001434 | 0.870993 | 0.127493 | 0 | 0 | 0 | 0 | 0 |
| 291 | 0.00005 | 0.001075 | 0.874623 | 0.124252 | 0 | 0 | 0 | 0 | 0 |
| 292 | 0.00009 | 0.001059 | 0.872435 | 0.126416 | 0 | 0 | 0 | 0 | 0 |
| 293 | 0.00012 | 0.001541 | 0.868525 | 0.129814 | 0 | 0 | 0 | 0 | 0 |
| 294 | 0.00003 | 0.001371 | 0.870476 | 0.128123 | 0 | 0 | 0 | 0 | 0 |
| 295 | 0.00006 | 0.001028 | 0.872519 | 0.126393 | 0 | 0 | 0 | 0 | 0 |
| 296 | 0.00008 | 0.001166 | 0.86993 | 0.128823 | 0 | 0 | 0 | 0 | 0 |
| 297 | 0.00011 | 0.00117 | 0.870688 | 0.128032 | 0 | 0 | 0 | 0 | 0 |
| 298 | 0.000101 | 0.001228 | 0.874348 | 0.124323 | 0 | 0 | 0 | 0 | 0 |
| 299 | 0.00005 | 0.001262 | 0.867204 | 0.131484 | 0 | 0 | 0 | 0 | 0 |
| 300 | 0.00006 | 0.001183 | 0.86912 | 0.129636 | 0 | 0 | 0 | 0 | 0 |
| 301 | 0.00003 | 0.00114 | 0.873011 | 0.125819 | 0 | 0 | 0 | 0 | 0 |
| 302 | 0.00005 | 0.00104 | 0.874055 | 0.124855 | 0 | 0 | 0 | 0 | 0 |
| 303 | 0.00005 | 0.001283 | 0.871707 | 0.126959 | 0 | 0 | 0 | 0 | 0 |
| 304 | 0.00009 | 0.001278 | 0.869873 | 0.128759 | 0 | 0 | 0 | 0 | 0 |
| 305 | 0.00013 | 0.001096 | 0.875962 | 0.122812 | 0 | 0 | 0 | 0 | 0 |
| 306 | 0.00002 | 0.001467 | 0.873091 | 0.125413 | 0.00001 | 0 | 0 | 0 | 0 |
| 307 | 0.00008 | 0.001157 | 0.871952 | 0.126811 | 0 | 0 | 0 | 0 | 0 |
| 308 | 0.00007 | 0.001106 | 0.876252 | 0.122571 | 0 | 0 | 0 | 0 | 0 |
| 309 | 0.00006 | 0.001059 | 0.875629 | 0.123252 | 0 | 0 | 0 | 0 | 0 |
| 310 | 0.000201 | 0.001449 | 0.873639 | 0.124711 | 0 | 0 | 0 | 0 | 0 |
| 311 | 0.00005 | 0.001433 | 0.873116 | 0.125401 | 0 | 0 | 0 | 0 | 0 |
| 312 | 0.00008 | 0.001489 | 0.869553 | 0.128878 | 0 | 0 | 0 | 0 | 0 |
| 313 | 0.00012 | 0.001351 | 0.873479 | 0.12505 | 0 | 0 | 0 | 0 | 0 |
| 314 | 0.00015 | 0.001031 | 0.872861 | 0.125957 | 0 | 0 | 0 | 0 | 0 |
| 315 | 0.00008 | 0.00108 | 0.869102 | 0.129738 | 0 | 0 | 0 | 0 | 0 |
| 316 | 0.00009 | 0.001221 | 0.874165 | 0.124523 | 0 | 0 | 0 | 0 | 0 |
| 317 | 0.00003 | 0.001262 | 0.873314 | 0.125394 | 0 | 0 | 0 | 0 | 0 |
| 318 | 0.00004 | 0.001262 | 0.867017 | 0.131631 | 0.00005 | 0 | 0 | 0 | 0 |
| 319 | 0.0001 | 0.001424 | 0.870962 | 0.127515 | 0 | 0 | 0 | 0 | 0 |
| 320 | 0.000079 | 0.001389 | 0.873232 | 0.1253 | 0 | 0 | 0 | 0 | 0 |
| 321 | 0.0001 | 0.00132 | 0.870072 | 0.128507 | 0 | 0 | 0 | 0 | 0 |
| 322 | 0.00004 | 0.001134 | 0.873398 | 0.125428 | 0 | 0 | 0 | 0 | 0 |
| 323 | 0.00006 | 0.00102 | 0.871947 | 0.126973 | 0 | 0 | 0 | 0 | 0 |
| 324 | 0.00007 | 0.001079 | 0.873748 | 0.125104 | 0 | 0 | 0 | 0 | 0 |
| 325 | 0.000129 | 0.001174 | 0.875204 | 0.123492 | 0 | 0 | 0 | 0 | 0 |
| 326 | 0.00009 | 0.000911 | 0.873523 | 0.125477 | 0 | 0 | 0 | 0 | 0 |
| 327 | 0.00015 | 0.001029 | 0.873891 | 0.12493 | 0 | 0 | 0 | 0 | 0 |
| 328 | 0.0001 | 0.001375 | 0.875598 | 0.122928 | 0 | 0 | 0 | 0 | 0 |
| 329 | 0.00006 | 0.001272 | 0.873106 | 0.125562 | 0 | 0 | 0 | 0 | 0 |
| 330 | 0.00004 | 0.001369 | 0.870675 | 0.127916 | 0 | 0 | 0 | 0 | 0 |
| 331 | 0.00006 | 0.001062 | 0.874387 | 0.124492 | 0 | 0 | 0 | 0 | 0 |
| 332 | 0.00008 | 0.001224 | 0.872731 | 0.125965 | 0 | 0 | 0 | 0 | 0 |
| 333 | 0.00001 | 0.00134 | 0.870007 | 0.128643 | 0 | 0 | 0 | 0 | 0 |
| 334 | 0.00009 | 0.00136 | 0.874109 | 0.124441 | 0 | 0 | 0 | 0 | 0 |
| 335 | 0.00008 | 0.001258 | 0.87171 | 0.126953 | 0 | 0 | 0 | 0 | 0 |
| 336 | 0.00003 | 0.001212 | 0.87021 | 0.128548 | 0 | 0 | 0 | 0 | 0 |
| 337 | 0.00005 | 0.001164 | 0.874305 | 0.124481 | 0 | 0 | 0 | 0 | 0 |
| 338 | 0.00006 | 0.001242 | 0.876461 | 0.122236 | 0 | 0 | 0 | 0 | 0 |
| 339 | 0.00007 | 0.00132 | 0.87352 | 0.12509 | 0 | 0 | 0 | 0 | 0 |
| 340 | 0.00007 | 0.000936 | 0.87014 | 0.128854 | 0 | 0 | 0 | 0 | 0 |
| 341 | 0.00008 | 0.001322 | 0.869769 | 0.128829 | 0 | 0 | 0 | 0 | 0 |
| 342 | 0.00006 | 0.000937 | 0.869233 | 0.12977 | 0 | 0 | 0 | 0 | 0 |
| 343 | 0.000069 | 0.00094 | 0.872291 | 0.1267 | 0 | 0 | 0 | 0 | 0 |
| 344 | 0.00011 | 0.001001 | 0.872327 | 0.126562 | 0 | 0 | 0 | 0 | 0 |
| 345 | 0.00005 | 0.001336 | 0.872138 | 0.126476 | 0 | 0 | 0 | 0 | 0 |
| 346 | 0.00008 | 0.001084 | 0.875707 | 0.123129 | 0 | 0 | 0 | 0 | 0 |
| 347 | 0.00006 | 0.001292 | 0.873744 | 0.124904 | 0 | 0 | 0 | 0 | 0 |
| 348 | 0.00004 | 0.00117 | 0.874364 | 0.124426 | 0 | 0 | 0 | 0 | 0 |
| 349 | 0.00008 | 0.001326 | 0.871906 | 0.126688 | 0 | 0 | 0 | 0 | 0 |
| 350 | 0.00007 | 0.001247 | 0.873953 | 0.12473 | 0 | 0 | 0 | 0 | 0 |
| 351 | 0.00011 | 0.001391 | 0.872816 | 0.125683 | 0 | 0 | 0 | 0 | 0 |
| 352 | 0.000089 | 0.001312 | 0.875307 | 0.123291 | 0 | 0 | 0 | 0 | 0 |
| 353 | 0.00007 | 0.001371 | 0.876076 | 0.122483 | 0 | 0 | 0 | 0 | 0 |
| 354 | 0.00007 | 0.001116 | 0.872506 | 0.126307 | 0 | 0 | 0 | 0 | 0 |
| 355 | 0.00009 | 0.001155 | 0.875289 | 0.123466 | 0 | 0 | 0 | 0 | 0 |
| 356 | 0.00012 | 0.001061 | 0.87681 | 0.122009 | 0 | 0 | 0 | 0 | 0 |
| 357 | 0.00005 | 0.001382 | 0.869668 | 0.1289 | 0 | 0 | 0 | 0 | 0 |
| 358 | 0.00007 | 0.001235 | 0.874143 | 0.124552 | 0 | 0 | 0 | 0 | 0 |
| 359 | 0.000141 | 0.001266 | 0.871643 | 0.12695 | 0 | 0 | 0 | 0 | 0 |
| 360 | 0.00003 | 0.001211 | 0.872636 | 0.126123 | 0 | 0 | 0 | 0 | 0 |
| 361 | 0.000089 | 0.001113 | 0.872426 | 0.126372 | 0 | 0 | 0 | 0 | 0 |
| 362 | 0.00003 | 0.001385 | 0.87366 | 0.124924 | 0 | 0 | 0 | 0 | 0 |
| 363 | 0.00006 | 0.001195 | 0.873827 | 0.124918 | 0 | 0 | 0 | 0 | 0 |
| 364 | 0.00004 | 0.001316 | 0.875881 | 0.122763 | 0 | 0 | 0 | 0 | 0 |
| 365 | 0.00007 | 0.001236 | 0.872113 | 0.126581 | 0 | 0 | 0 | 0 | 0 |
| 366 | 0.00009 | 0.001001 | 0.873251 | 0.125658 | 0 | 0 | 0 | 0 | 0 |
| 367 | 0.0001 | 0.001365 | 0.870302 | 0.128233 | 0 | 0 | 0 | 0 | 0 |
| 368 | 0.00005 | 0.001357 | 0.875712 | 0.12288 | 0 | 0 | 0 | 0 | 0 |
| 369 | 0.00003 | 0.001108 | 0.87146 | 0.127402 | 0 | 0 | 0 | 0 | 0 |
| 370 | 0.00006 | 0.001139 | 0.871736 | 0.127064 | 0 | 0 | 0 | 0 | 0 |
| 371 | 0.00005 | 0.001189 | 0.871164 | 0.127507 | 0.00009 | 0 | 0 | 0 | 0 |
| 372 | 0.000091 | 0.001019 | 0.86786 | 0.13103 | 0 | 0 | 0 | 0 | 0 |
| 373 | 0.00007 | 0.001101 | 0.872341 | 0.126488 | 0 | 0 | 0 | 0 | 0 |
| 374 | 0.00005 | 0.001221 | 0.876345 | 0.122383 | 0 | 0 | 0 | 0 | 0 |
| 375 | 0.000111 | 0.001035 | 0.87227 | 0.126584 | 0 | 0 | 0 | 0 | 0 |
| 376 | 0.00006 | 0.001293 | 0.872506 | 0.12614 | 0 | 0 | 0 | 0 | 0 |
| 377 | 0.00004 | 0.001285 | 0.869479 | 0.129196 | 0 | 0 | 0 | 0 | 0 |
| 378 | 0.0001 | 0.001149 | 0.873243 | 0.125508 | 0 | 0 | 0 | 0 | 0 |
| 379 | 0.00005 | 0.001362 | 0.876849 | 0.12174 | 0 | 0 | 0 | 0 | 0 |
| 380 | 0.00005 | 0.001065 | 0.8719 | 0.126986 | 0 | 0 | 0 | 0 | 0 |
| 381 | 0.00006 | 0.001249 | 0.871818 | 0.126872 | 0 | 0 | 0 | 0 | 0 |
| 382 | 0.00006 | 0.001314 | 0.872116 | 0.12651 | 0 | 0 | 0 | 0 | 0 |
| 383 | 0.00003 | 0.001242 | 0.874093 | 0.124636 | 0 | 0 | 0 | 0 | 0 |
| 384 | 0.00006 | 0.001396 | 0.867239 | 0.131305 | 0 | 0 | 0 | 0 | 0 |
| 385 | 0.00007 | 0.001221 | 0.875101 | 0.123608 | 0 | 0 | 0 | 0 | 0 |
| 386 | 0.00006 | 0.001255 | 0.872635 | 0.12605 | 0 | 0 | 0 | 0 | 0 |
| 387 | 0.00004 | 0.00116 | 0.875309 | 0.123492 | 0 | 0 | 0 | 0 | 0 |
| 388 | 0.00007 | 0.001176 | 0.874761 | 0.123992 | 0 | 0 | 0 | 0 | 0 |
| 389 | 0.00004 | 0.001257 | 0.874477 | 0.124226 | 0 | 0 | 0 | 0 | 0 |
| 390 | 0.00004 | 0.001281 | 0.872149 | 0.126529 | 0 | 0 | 0 | 0 | 0 |
| 391 | 0.00007 | 0.001081 | 0.86464 | 0.134208 | 0 | 0 | 0 | 0 | 0 |
| 392 | 0.00011 | 0.001311 | 0.872418 | 0.126162 | 0 | 0 | 0 | 0 | 0 |
| 393 | 0.00005 | 0.001275 | 0.870529 | 0.128146 | 0 | 0 | 0 | 0 | 0 |
| 394 | 0.00003 | 0.001145 | 0.874537 | 0.124288 | 0 | 0 | 0 | 0 | 0 |
| 395 | 0.00002 | 0.001407 | 0.873169 | 0.125404 | 0 | 0 | 0 | 0 | 0 |
| 396 | 0.00007 | 0.001023 | 0.87509 | 0.123817 | 0 | 0 | 0 | 0 | 0 |
| 397 | 0.000121 | 0.001409 | 0.874297 | 0.124153 | 0.00002 | 0 | 0 | 0 | 0 |
| 398 | 0.000141 | 0.001271 | 0.873289 | 0.125299 | 0 | 0 | 0 | 0 | 0 |
| 399 | 0.00008 | 0.00116 | 0.872094 | 0.126666 | 0 | 0 | 0 | 0 | 0 |
| 400 | 0.00004 | 0.001133 | 0.877 | 0.121828 | 0 | 0 | 0 | 0 | 0 |
| 401 | 0.00009 | 0.001385 | 0.873708 | 0.124817 | 0 | 0 | 0 | 0 | 0 |
| 402 | 0.00007 | 0.001118 | 0.87243 | 0.126382 | 0 | 0 | 0 | 0 | 0 |
| 403 | 0.000139 | 0.001162 | 0.875125 | 0.123574 | 0 | 0 | 0 | 0 | 0 |
| 404 | 0.00004 | 0.001479 | 0.873682 | 0.124799 | 0 | 0 | 0 | 0 | 0 |
| 405 | 0.00007 | 0.001 | 0.877926 | 0.121004 | 0 | 0 | 0 | 0 | 0 |
| 406 | 0.000079 | 0.001181 | 0.872731 | 0.126009 | 0 | 0 | 0 | 0 | 0 |
| 407 | 0.00005 | 0.001522 | 0.873398 | 0.12503 | 0 | 0 | 0 | 0 | 0 |
| 408 | 0.0001 | 0.000971 | 0.871801 | 0.127128 | 0 | 0 | 0 | 0 | 0 |
| 409 | 0.00008 | 0.001393 | 0.871762 | 0.126764 | 0 | 0 | 0 | 0 | 0 |
| 410 | 0.00003 | 0.001021 | 0.872352 | 0.126597 | 0 | 0 | 0 | 0 | 0 |
| 411 | 0.00005 | 0.001077 | 0.876883 | 0.121989 | 0 | 0 | 0 | 0 | 0 |
| 412 | 0.00013 | 0.001282 | 0.87344 | 0.125148 | 0 | 0 | 0 | 0 | 0 |
| 413 | 0.00003 | 0.001304 | 0.870894 | 0.127773 | 0 | 0 | 0 | 0 | 0 |
| 414 | 0.00005 | 0.001447 | 0.8747 | 0.123803 | 0 | 0 | 0 | 0 | 0 |
| 415 | 0.00006 | 0.001329 | 0.874248 | 0.124363 | 0 | 0 | 0 | 0 | 0 |
| 416 | 0.00003 | 0.001302 | 0.87222 | 0.126418 | 0.00003 | 0 | 0 | 0 | 0 |
| 417 | 0.0001 | 0.001022 | 0.874474 | 0.124404 | 0 | 0 | 0 | 0 | 0 |
| 418 | 0.000149 | 0.001123 | 0.875734 | 0.122994 | 0 | 0 | 0 | 0 | 0 |
| 419 | 0.00009 | 0.001038 | 0.870177 | 0.128695 | 0 | 0 | 0 | 0 | 0 |
| 420 | 0.00003 | 0.001229 | 0.874505 | 0.124236 | 0 | 0 | 0 | 0 | 0 |
| 421 | 0.0001 | 0.001337 | 0.872353 | 0.126211 | 0 | 0 | 0 | 0 | 0 |
| 422 | 0.00003 | 0.001235 | 0.873434 | 0.125301 | 0 | 0 | 0 | 0 | 0 |
| 423 | 0.00005 | 0.001145 | 0.875056 | 0.12375 | 0 | 0 | 0 | 0 | 0 |
| 424 | 0.00006 | 0.001248 | 0.878709 | 0.119953 | 0.00003 | 0 | 0 | 0 | 0 |
| 425 | 0.00007 | 0.001396 | 0.871878 | 0.126656 | 0 | 0 | 0 | 0 | 0 |
| 426 | 0.00006 | 0.001248 | 0.869598 | 0.129094 | 0 | 0 | 0 | 0 | 0 |
| 427 | 0.00002 | 0.001356 | 0.874283 | 0.124341 | 0 | 0 | 0 | 0 | 0 |
| 428 | 0.00013 | 0.001441 | 0.876693 | 0.121736 | 0 | 0 | 0 | 0 | 0 |
| 429 | 0.00005 | 0.001147 | 0.875692 | 0.123112 | 0 | 0 | 0 | 0 | 0 |
| 430 | 0.00006 | 0.001312 | 0.875991 | 0.122637 | 0 | 0 | 0 | 0 | 0 |
| 431 | 0.00002 | 0.000959 | 0.873321 | 0.1257 | 0 | 0 | 0 | 0 | 0 |
| 432 | 0.000119 | 0.001182 | 0.869532 | 0.129167 | 0 | 0 | 0 | 0 | 0 |
| 433 | 0.000081 | 0.00116 | 0.871465 | 0.127294 | 0 | 0 | 0 | 0 | 0 |
| 434 | 0.00008 | 0.001327 | 0.873301 | 0.125292 | 0 | 0 | 0 | 0 | 0 |
| 435 | 0.00007 | 0.001348 | 0.87376 | 0.124823 | 0 | 0 | 0 | 0 | 0 |
| 436 | 0.00004 | 0.001003 | 0.868042 | 0.130916 | 0 | 0 | 0 | 0 | 0 |
| 437 | 0.00007 | 0.001007 | 0.872276 | 0.126647 | 0 | 0 | 0 | 0 | 0 |
| 438 | 0.00005 | 0.00148 | 0.871567 | 0.126903 | 0 | 0 | 0 | 0 | 0 |
| 439 | 0.00004 | 0.001173 | 0.87234 | 0.126447 | 0 | 0 | 0 | 0 | 0 |
| 440 | 0.00005 | 0.000944 | 0.872924 | 0.126083 | 0 | 0 | 0 | 0 | 0 |
| 441 | 0.00005 | 0.001046 | 0.87438 | 0.124524 | 0 | 0 | 0 | 0 | 0 |
| 442 | 0.00004 | 0.000985 | 0.871716 | 0.127259 | 0 | 0 | 0 | 0 | 0 |
| 443 | 0.00001 | 0.00123 | 0.872763 | 0.125997 | 0 | 0 | 0 | 0 | 0 |
| 444 | 0.00006 | 0.001426 | 0.873156 | 0.125358 | 0 | 0 | 0 | 0 | 0 |
| 445 | 0.00006 | 0.001295 | 0.86825 | 0.130395 | 0 | 0 | 0 | 0 | 0 |
| 446 | 0.000119 | 0.000933 | 0.872135 | 0.126813 | 0 | 0 | 0 | 0 | 0 |
| 447 | 0.00007 | 0.001169 | 0.875638 | 0.123122 | 0 | 0 | 0 | 0 | 0 |
| 448 | 0.00012 | 0.001443 | 0.870795 | 0.127643 | 0 | 0 | 0 | 0 | 0 |
| 449 | 0.00002 | 0.001219 | 0.875694 | 0.123067 | 0 | 0 | 0 | 0 | 0 |
| 450 | 0.000089 | 0.001102 | 0.872985 | 0.125823 | 0 | 0 | 0 | 0 | 0 |
| 451 | 0.00004 | 0.001315 | 0.876805 | 0.12184 | 0 | 0 | 0 | 0 | 0 |
| 452 | 0.00008 | 0.001213 | 0.873141 | 0.125566 | 0 | 0 | 0 | 0 | 0 |
| 453 | 0.00007 | 0.001366 | 0.874109 | 0.124455 | 0 | 0 | 0 | 0 | 0 |
| 454 | 0.00005 | 0.001137 | 0.874277 | 0.124536 | 0 | 0 | 0 | 0 | 0 |
| 455 | 0.00007 | 0.001192 | 0.879195 | 0.119543 | 0 | 0 | 0 | 0 | 0 |
| 456 | 0.00008 | 0.001276 | 0.873115 | 0.125529 | 0 | 0 | 0 | 0 | 0 |
| 457 | 0.00005 | 0.001225 | 0.870931 | 0.127754 | 0.00004 | 0 | 0 | 0 | 0 |
| 458 | 0.00005 | 0.001184 | 0.874674 | 0.124092 | 0 | 0 | 0 | 0 | 0 |
| 459 | 0.00001 | 0.001017 | 0.870185 | 0.128788 | 0 | 0 | 0 | 0 | 0 |
| 460 | 0.00002 | 0.001476 | 0.872264 | 0.126239 | 0 | 0 | 0 | 0 | 0 |
| 461 | 0.00008 | 0.001437 | 0.873166 | 0.125317 | 0 | 0 | 0 | 0 | 0 |
| 462 | 0.00004 | 0.001003 | 0.877442 | 0.121515 | 0 | 0 | 0 | 0 | 0 |
| 463 | 0.00007 | 0.001267 | 0.87141 | 0.127253 | 0 | 0 | 0 | 0 | 0 |
| 464 | 0.00004 | 0.001296 | 0.874284 | 0.124309 | 0.00007 | 0 | 0 | 0 | 0 |
| 465 | 0.00007 | 0.000905 | 0.873358 | 0.125667 | 0 | 0 | 0 | 0 | 0 |
| 466 | 0.00008 | 0.001143 | 0.870214 | 0.128563 | 0 | 0 | 0 | 0 | 0 |
| 467 | 0.00009 | 0.001031 | 0.868935 | 0.129944 | 0 | 0 | 0 | 0 | 0 |
| 468 | 0.00006 | 0.001091 | 0.873868 | 0.124981 | 0 | 0 | 0 | 0 | 0 |
| 469 | 0.00006 | 0.001186 | 0.874958 | 0.123797 | 0 | 0 | 0 | 0 | 0 |
| 470 | 0.00009 | 0.000977 | 0.875425 | 0.123508 | 0 | 0 | 0 | 0 | 0 |
| 471 | 0.00007 | 0.001345 | 0.874803 | 0.123782 | 0 | 0 | 0 | 0 | 0 |
| 472 | 0.00004 | 0.00115 | 0.87291 | 0.1259 | 0 | 0 | 0 | 0 | 0 |
| 473 | 0.00003 | 0.001279 | 0.870665 | 0.128026 | 0 | 0 | 0 | 0 | 0 |
| 474 | 0.00007 | 0.001133 | 0.873408 | 0.125389 | 0 | 0 | 0 | 0 | 0 |
| 475 | 0.00011 | 0.001133 | 0.871647 | 0.12711 | 0 | 0 | 0 | 0 | 0 |
| 476 | 0.00015 | 0.001336 | 0.867104 | 0.131411 | 0 | 0 | 0 | 0 | 0 |
| 477 | 0.00011 | 0.001111 | 0.87183 | 0.126949 | 0 | 0 | 0 | 0 | 0 |
| 478 | 0.00009 | 0.001156 | 0.870304 | 0.12845 | 0 | 0 | 0 | 0 | 0 |
| 479 | 0.00005 | 0.00099 | 0.869875 | 0.129084 | 0 | 0 | 0 | 0 | 0 |
| 480 | 0.00011 | 0.001411 | 0.868045 | 0.130433 | 0 | 0 | 0 | 0 | 0 |
| 481 | 0.00008 | 0.001174 | 0.879056 | 0.119689 | 0 | 0 | 0 | 0 | 0 |
| 482 | 0.00011 | 0.001147 | 0.869287 | 0.129456 | 0 | 0 | 0 | 0 | 0 |
| 483 | 0.00007 | 0.001136 | 0.873786 | 0.125007 | 0 | 0 | 0 | 0 | 0 |
| 484 | 0.00005 | 0.001324 | 0.867399 | 0.131227 | 0 | 0 | 0 | 0 | 0 |
| 485 | 0.00006 | 0.001425 | 0.878152 | 0.120363 | 0 | 0 | 0 | 0 | 0 |
| 486 | 0.00007 | 0.001285 | 0.875035 | 0.123609 | 0 | 0 | 0 | 0 | 0 |
| 487 | 0.00004 | 0.001191 | 0.877627 | 0.121142 | 0 | 0 | 0 | 0 | 0 |
| 488 | 0.00008 | 0.001102 | 0.875767 | 0.123051 | 0 | 0 | 0 | 0 | 0 |
| 489 | 0.00004 | 0.001231 | 0.875105 | 0.123624 | 0 | 0 | 0 | 0 | 0 |
| 490 | 0.000101 | 0.001468 | 0.873871 | 0.12456 | 0 | 0 | 0 | 0 | 0 |
| 491 | 0.00002 | 0.001436 | 0.871747 | 0.126797 | 0 | 0 | 0 | 0 | 0 |
| 492 | 0.00005 | 0.00123 | 0.868974 | 0.129746 | 0 | 0 | 0 | 0 | 0 |
| 493 | 0.00007 | 0.001227 | 0.870744 | 0.127958 | 0 | 0 | 0 | 0 | 0 |
| 494 | 0.00003 | 0.00101 | 0.87106 | 0.1279 | 0 | 0 | 0 | 0 | 0 |
| 495 | 0.00006 | 0.00127 | 0.873532 | 0.125138 | 0 | 0 | 0 | 0 | 0 |
| 496 | 0.000089 | 0.001322 | 0.869064 | 0.129445 | 0.00008 | 0 | 0 | 0 | 0 |
| 497 | 0.00008 | 0.000946 | 0.872803 | 0.126172 | 0 | 0 | 0 | 0 | 0 |
| 498 | 0.00009 | 0.001336 | 0.875577 | 0.122997 | 0 | 0 | 0 | 0 | 0 |
| 499 | 0.00005 | 0.001266 | 0.869224 | 0.12946 | 0 | 0 | 0 | 0 | 0 |
| 500 | 0.00008 | 0.001282 | 0.875976 | 0.122662 | 0 | 0 | 0 | 0 | 0 |
| 501 | 0.00006 | 0.00142 | 0.874617 | 0.123902 | 0 | 0 | 0 | 0 | 0 |
| 502 | 0.00009 | 0.00115 | 0.874926 | 0.123834 | 0 | 0 | 0 | 0 | 0 |
| 503 | 0.000079 | 0.001221 | 0.873605 | 0.125094 | 0 | 0 | 0 | 0 | 0 |
| 504 | 0.00006 | 0.001053 | 0.873884 | 0.125004 | 0 | 0 | 0 | 0 | 0 |
| 505 | 0.00008 | 0.001316 | 0.874697 | 0.123887 | 0.00002 | 0 | 0 | 0 | 0 |
| 506 | 0.00006 | 0.001047 | 0.874472 | 0.124422 | 0 | 0 | 0 | 0 | 0 |
| 507 | 0.000081 | 0.00114 | 0.873392 | 0.125387 | 0 | 0 | 0 | 0 | 0 |
| 508 | 0.00006 | 0.00118 | 0.872863 | 0.125897 | 0 | 0 | 0 | 0 | 0 |
| 509 | 0.00006 | 0.001108 | 0.873266 | 0.125566 | 0 | 0 | 0 | 0 | 0 |
| 510 | 0.00007 | 0.001468 | 0.875529 | 0.122932 | 0 | 0 | 0 | 0 | 0 |
| 511 | 0.00009 | 0.00112 | 0.872591 | 0.1262 | 0 | 0 | 0 | 0 | 0 |
| 512 | 0.00008 | 0.001276 | 0.868716 | 0.129929 | 0 | 0 | 0 | 0 | 0 |
| 513 | 0.00008 | 0.001203 | 0.874591 | 0.124125 | 0 | 0 | 0 | 0 | 0 |
| 514 | 0.00008 | 0.001149 | 0.873626 | 0.125145 | 0 | 0 | 0 | 0 | 0 |
| 515 | 0.00006 | 0.001267 | 0.871232 | 0.127441 | 0 | 0 | 0 | 0 | 0 |
| 516 | 0.0001 | 0.001285 | 0.86852 | 0.130096 | 0 | 0 | 0 | 0 | 0 |
| 517 | 0.00006 | 0.001341 | 0.870661 | 0.127938 | 0 | 0 | 0 | 0 | 0 |
| 518 | 0.00007 | 0.0012 | 0.873792 | 0.124937 | 0 | 0 | 0 | 0 | 0 |
| 519 | 0.00007 | 0.001154 | 0.87277 | 0.125905 | 0.0001 | 0 | 0 | 0 | 0 |
| 520 | 0.000091 | 0.00115 | 0.871405 | 0.127344 | 0.00001 | 0 | 0 | 0 | 0 |
| 521 | 0.00006 | 0.001285 | 0.872169 | 0.126486 | 0 | 0 | 0 | 0 | 0 |
| 522 | 0.00008 | 0.001167 | 0.87569 | 0.123063 | 0 | 0 | 0 | 0 | 0 |
| 523 | 0.000079 | 0.001032 | 0.873635 | 0.125254 | 0 | 0 | 0 | 0 | 0 |
| 524 | 0.000111 | 0.001146 | 0.870141 | 0.128601 | 0 | 0 | 0 | 0 | 0 |
| 525 | 0.0001 | 0.001051 | 0.870373 | 0.128475 | 0 | 0 | 0 | 0 | 0 |
| 526 | 0.00005 | 0.001253 | 0.870728 | 0.12797 | 0 | 0 | 0 | 0 | 0 |
| 527 | 0.00007 | 0.001076 | 0.872592 | 0.126262 | 0 | 0 | 0 | 0 | 0 |
| 528 | 0.00005 | 0.001197 | 0.871613 | 0.12714 | 0 | 0 | 0 | 0 | 0 |
| 529 | 0.00003 | 0.00098 | 0.871363 | 0.127627 | 0 | 0 | 0 | 0 | 0 |
| 530 | 0.00012 | 0.001337 | 0.867475 | 0.131069 | 0 | 0 | 0 | 0 | 0 |
| 531 | 0.00001 | 0.001157 | 0.873149 | 0.125683 | 0 | 0 | 0 | 0 | 0 |
| 532 | 0.00004 | 0.001162 | 0.877874 | 0.120924 | 0 | 0 | 0 | 0 | 0 |
| 533 | 0.00005 | 0.001147 | 0.871931 | 0.126873 | 0 | 0 | 0 | 0 | 0 |
| 534 | 0.00008 | 0.001318 | 0.86854 | 0.130062 | 0 | 0 | 0 | 0 | 0 |
| 535 | 0.00008 | 0.001339 | 0.876339 | 0.122242 | 0 | 0 | 0 | 0 | 0 |
| 536 | 0.00003 | 0.00098 | 0.875546 | 0.123344 | 0.0001 | 0 | 0 | 0 | 0 |
| 537 | 0.00008 | 0.001157 | 0.872191 | 0.126572 | 0 | 0 | 0 | 0 | 0 |
| 538 | 0.00006 | 0.000971 | 0.869168 | 0.1298 | 0 | 0 | 0 | 0 | 0 |
| 539 | 0.00012 | 0.001343 | 0.871532 | 0.127005 | 0 | 0 | 0 | 0 | 0 |
| 540 | 0.000141 | 0.001315 | 0.868274 | 0.13027 | 0 | 0 | 0 | 0 | 0 |
| 541 | 0.00006 | 0.001116 | 0.871874 | 0.12695 | 0 | 0 | 0 | 0 | 0 |
| 542 | 0.000081 | 0.001148 | 0.872667 | 0.126105 | 0 | 0 | 0 | 0 | 0 |
| 543 | 0.00005 | 0.00105 | 0.877047 | 0.121853 | 0 | 0 | 0 | 0 | 0 |
| 544 | 0.00009 | 0.00126 | 0.873278 | 0.125352 | 0.00002 | 0 | 0 | 0 | 0 |
| 545 | 0.00003 | 0.001489 | 0.869554 | 0.128927 | 0 | 0 | 0 | 0 | 0 |
| 546 | 0.00003 | 0.00136 | 0.87291 | 0.1257 | 0 | 0 | 0 | 0 | 0 |
| 547 | 0.000101 | 0.001131 | 0.872455 | 0.126314 | 0 | 0 | 0 | 0 | 0 |
| 548 | 0.00009 | 0.001326 | 0.873766 | 0.124818 | 0 | 0 | 0 | 0 | 0 |
| 549 | 0.00004 | 0.001223 | 0.869334 | 0.129403 | 0 | 0 | 0 | 0 | 0 |
| 550 | 0.00006 | 0.001105 | 0.874436 | 0.124399 | 0 | 0 | 0 | 0 | 0 |
| 551 | 0.00008 | 0.001347 | 0.873407 | 0.125166 | 0 | 0 | 0 | 0 | 0 |
| 552 | 0.00008 | 0.001338 | 0.870505 | 0.128077 | 0 | 0 | 0 | 0 | 0 |
| 553 | 0.00007 | 0.001251 | 0.86989 | 0.128789 | 0 | 0 | 0 | 0 | 0 |
| 554 | 0.00011 | 0.001257 | 0.874822 | 0.123811 | 0 | 0 | 0 | 0 | 0 |
| 555 | 0.0001 | 0.001208 | 0.871436 | 0.127257 | 0 | 0 | 0 | 0 | 0 |
| 556 | 0.00011 | 0.001445 | 0.876172 | 0.122274 | 0 | 0 | 0 | 0 | 0 |
| 557 | 0.00004 | 0.001356 | 0.874454 | 0.12415 | 0 | 0 | 0 | 0 | 0 |
| 558 | 0.00005 | 0.001128 | 0.872701 | 0.126121 | 0 | 0 | 0 | 0 | 0 |
| 559 | 0.00004 | 0.001351 | 0.873527 | 0.125083 | 0 | 0 | 0 | 0 | 0 |
| 560 | 0.00007 | 0.001247 | 0.874706 | 0.123977 | 0 | 0 | 0 | 0 | 0 |
| 561 | 0.00006 | 0.001384 | 0.870378 | 0.128178 | 0 | 0 | 0 | 0 | 0 |
| 562 | 0.00004 | 0.001518 | 0.871664 | 0.126779 | 0 | 0 | 0 | 0 | 0 |
| 563 | 0.00009 | 0.001265 | 0.870366 | 0.12828 | 0 | 0 | 0 | 0 | 0 |
| 564 | 0.00006 | 0.001456 | 0.875821 | 0.122663 | 0 | 0 | 0 | 0 | 0 |
| 565 | 0.00006 | 0.001282 | 0.869089 | 0.129569 | 0 | 0 | 0 | 0 | 0 |
| 566 | 0.00006 | 0.001164 | 0.872343 | 0.126433 | 0 | 0 | 0 | 0 | 0 |
| 567 | 0.00006 | 0.001399 | 0.871748 | 0.126794 | 0 | 0 | 0 | 0 | 0 |
| 568 | 0.00007 | 0.00129 | 0.872241 | 0.126399 | 0 | 0 | 0 | 0 | 0 |
| 569 | 0.00003 | 0.001294 | 0.877276 | 0.1214 | 0 | 0 | 0 | 0 | 0 |
| 570 | 0.000121 | 0.001256 | 0.868393 | 0.130231 | 0 | 0 | 0 | 0 | 0 |
| 571 | 0.00005 | 0.001466 | 0.874789 | 0.123695 | 0 | 0 | 0 | 0 | 0 |
| 572 | 0.00006 | 0.00125 | 0.872509 | 0.126181 | 0 | 0 | 0 | 0 | 0 |
| 573 | 0.00005 | 0.001408 | 0.873066 | 0.125477 | 0 | 0 | 0 | 0 | 0 |
| 574 | 0.00006 | 0.001319 | 0.873985 | 0.124627 | 0.00001 | 0 | 0 | 0 | 0 |
| 575 | 0.00011 | 0.001276 | 0.870384 | 0.12823 | 0 | 0 | 0 | 0 | 0 |
| 576 | 0.0001 | 0.001085 | 0.875152 | 0.123663 | 0 | 0 | 0 | 0 | 0 |
| 577 | 0.00006 | 0.001198 | 0.868873 | 0.129869 | 0 | 0 | 0 | 0 | 0 |
| 578 | 0.00006 | 0.001112 | 0.87387 | 0.124957 | 0 | 0 | 0 | 0 | 0 |
| 579 | 0.00012 | 0.001265 | 0.872919 | 0.125695 | 0 | 0 | 0 | 0 | 0 |
| 580 | 0.00005 | 0.001262 | 0.876938 | 0.12175 | 0 | 0 | 0 | 0 | 0 |
| 581 | 0.00005 | 0.001188 | 0.871778 | 0.126985 | 0 | 0 | 0 | 0 | 0 |
| 582 | 0.00007 | 0.001087 | 0.873827 | 0.125016 | 0 | 0 | 0 | 0 | 0 |
| 583 | 0.00008 | 0.001169 | 0.871291 | 0.12746 | 0 | 0 | 0 | 0 | 0 |
| 584 | 0.00005 | 0.001063 | 0.86618 | 0.132706 | 0 | 0 | 0 | 0 | 0 |
| 585 | 0.00005 | 0.001165 | 0.8718 | 0.126984 | 0 | 0 | 0 | 0 | 0 |
| 586 | 0.00012 | 0.001306 | 0.869368 | 0.129206 | 0 | 0 | 0 | 0 | 0 |
| 587 | 0.00007 | 0.001069 | 0.872317 | 0.126544 | 0 | 0 | 0 | 0 | 0 |
| 588 | 0.00006 | 0.001293 | 0.87595 | 0.122697 | 0 | 0 | 0 | 0 | 0 |
| 589 | 0.00007 | 0.001158 | 0.872964 | 0.125808 | 0 | 0 | 0 | 0 | 0 |
| 590 | 0.00005 | 0.001283 | 0.869152 | 0.129515 | 0 | 0 | 0 | 0 | 0 |
| 591 | 0.00006 | 0.001057 | 0.873398 | 0.125485 | 0 | 0 | 0 | 0 | 0 |
| 592 | 0.00004 | 0.00153 | 0.873696 | 0.124734 | 0 | 0 | 0 | 0 | 0 |
| 593 | 0.00005 | 0.000947 | 0.875725 | 0.123278 | 0 | 0 | 0 | 0 | 0 |
| 594 | 0.0001 | 0.00113 | 0.871427 | 0.127343 | 0 | 0 | 0 | 0 | 0 |
| 595 | 0.00008 | 0.001084 | 0.872909 | 0.125926 | 0 | 0 | 0 | 0 | 0 |
| 596 | 0.00009 | 0.001086 | 0.875199 | 0.123625 | 0 | 0 | 0 | 0 | 0 |
| 597 | 0.00007 | 0.00131 | 0.86909 | 0.12953 | 0 | 0 | 0 | 0 | 0 |
| 598 | 0.00003 | 0.001202 | 0.874852 | 0.123916 | 0 | 0 | 0 | 0 | 0 |
| 599 | 0.00004 | 0.00113 | 0.872567 | 0.126263 | 0 | 0 | 0 | 0 | 0 |
| 600 | 0.00007 | 0.001312 | 0.871171 | 0.127448 | 0 | 0 | 0 | 0 | 0 |
| 601 | 0.00012 | 0.001373 | 0.870893 | 0.127614 | 0 | 0 | 0 | 0 | 0 |
| 602 | 0.00009 | 0.001193 | 0.875233 | 0.123484 | 0 | 0 | 0 | 0 | 0 |
| 603 | 0.00006 | 0.001391 | 0.872608 | 0.125942 | 0 | 0 | 0 | 0 | 0 |
| 604 | 0.00007 | 0.001124 | 0.873852 | 0.124954 | 0 | 0 | 0 | 0 | 0 |
| 605 | 0.000059 | 0.001398 | 0.873699 | 0.124844 | 0 | 0 | 0 | 0 | 0 |
| 606 | 0.0001 | 0.00128 | 0.874385 | 0.124235 | 0 | 0 | 0 | 0 | 0 |
| 607 | 0.00008 | 0.001473 | 0.8757 | 0.122747 | 0 | 0 | 0 | 0 | 0 |
| 608 | 0.00011 | 0.001088 | 0.870121 | 0.128682 | 0 | 0 | 0 | 0 | 0 |
| 609 | 0.00009 | 0.001226 | 0.873541 | 0.125143 | 0 | 0 | 0 | 0 | 0 |
| 610 | 0.00005 | 0.001113 | 0.874394 | 0.124443 | 0 | 0 | 0 | 0 | 0 |
| 611 | 0.00005 | 0.00114 | 0.874375 | 0.124435 | 0 | 0 | 0 | 0 | 0 |
| 612 | 0.000081 | 0.00101 | 0.869485 | 0.129425 | 0 | 0 | 0 | 0 | 0 |
| 613 | 0.00005 | 0.001206 | 0.870533 | 0.12819 | 0.00002 | 0 | 0 | 0 | 0 |
| 614 | 0.00008 | 0.00126 | 0.877399 | 0.121261 | 0 | 0 | 0 | 0 | 0 |
| 615 | 0.000099 | 0.00122 | 0.874167 | 0.124504 | 0.00001 | 0 | 0 | 0 | 0 |
| 616 | 0.00009 | 0.001235 | 0.875402 | 0.123272 | 0 | 0 | 0 | 0 | 0 |
| 617 | 0.0001 | 0.001389 | 0.873677 | 0.124834 | 0 | 0 | 0 | 0 | 0 |
| 618 | 0.00008 | 0.001244 | 0.871922 | 0.126754 | 0 | 0 | 0 | 0 | 0 |
| 619 | 0.0001 | 0.001215 | 0.872415 | 0.126271 | 0 | 0 | 0 | 0 | 0 |
| 620 | 0.00013 | 0.001297 | 0.872638 | 0.125935 | 0 | 0 | 0 | 0 | 0 |
| 621 | 0.00005 | 0.001013 | 0.875242 | 0.123695 | 0 | 0 | 0 | 0 | 0 |
| 622 | 0.00007 | 0.001343 | 0.867977 | 0.13061 | 0 | 0 | 0 | 0 | 0 |
| 623 | 0.00007 | 0.001122 | 0.872951 | 0.125857 | 0 | 0 | 0 | 0 | 0 |
| 624 | 0.0001 | 0.001303 | 0.872165 | 0.126432 | 0 | 0 | 0 | 0 | 0 |
| 625 | 0.00003 | 0.001486 | 0.874132 | 0.124322 | 0.00003 | 0 | 0 | 0 | 0 |
| 626 | 0.00005 | 0.001272 | 0.870837 | 0.127841 | 0 | 0 | 0 | 0 | 0 |
| 627 | 0.00006 | 0.000901 | 0.873956 | 0.125083 | 0 | 0 | 0 | 0 | 0 |
| 628 | 0.00003 | 0.001084 | 0.872422 | 0.126464 | 0 | 0 | 0 | 0 | 0 |
| 629 | 0.00006 | 0.001055 | 0.872687 | 0.126199 | 0 | 0 | 0 | 0 | 0 |
| 630 | 0.00004 | 0.001088 | 0.870007 | 0.128865 | 0 | 0 | 0 | 0 | 0 |
| 631 | 0.00008 | 0.001134 | 0.872879 | 0.125908 | 0 | 0 | 0 | 0 | 0 |
| 632 | 0.000081 | 0.001281 | 0.874376 | 0.124263 | 0 | 0 | 0 | 0 | 0 |
| 633 | 0.0001 | 0.000999 | 0.874298 | 0.124603 | 0 | 0 | 0 | 0 | 0 |
| 634 | 0.000101 | 0.001189 | 0.873737 | 0.124974 | 0 | 0 | 0 | 0 | 0 |
| 635 | 0.00007 | 0.001172 | 0.871724 | 0.127034 | 0 | 0 | 0 | 0 | 0 |
| 636 | 0.000081 | 0.001039 | 0.87001 | 0.12887 | 0 | 0 | 0 | 0 | 0 |
| 637 | 0.00007 | 0.001186 | 0.872489 | 0.126255 | 0 | 0 | 0 | 0 | 0 |
| 638 | 0.00006 | 0.00126 | 0.872451 | 0.126229 | 0 | 0 | 0 | 0 | 0 |
| 639 | 0.00008 | 0.001211 | 0.870843 | 0.127865 | 0 | 0 | 0 | 0 | 0 |
| 640 | 0.00004 | 0.001048 | 0.867485 | 0.131427 | 0 | 0 | 0 | 0 | 0 |
| 641 | 0.00009 | 0.001039 | 0.872882 | 0.125989 | 0 | 0 | 0 | 0 | 0 |
| 642 | 0.00006 | 0.001128 | 0.874447 | 0.124365 | 0 | 0 | 0 | 0 | 0 |
| 643 | 0.00008 | 0.001064 | 0.873954 | 0.124902 | 0 | 0 | 0 | 0 | 0 |
| 644 | 0.00006 | 0.001197 | 0.87691 | 0.121833 | 0 | 0 | 0 | 0 | 0 |
| 645 | 0.00006 | 0.001035 | 0.872734 | 0.126171 | 0 | 0 | 0 | 0 | 0 |
| 646 | 0.00006 | 0.00145 | 0.869501 | 0.128989 | 0 | 0 | 0 | 0 | 0 |
| 647 | 0.00009 | 0.000951 | 0.875936 | 0.123024 | 0 | 0 | 0 | 0 | 0 |
| 648 | 0.000081 | 0.001229 | 0.873595 | 0.125096 | 0 | 0 | 0 | 0 | 0 |
| 649 | 0.00008 | 0.001279 | 0.874507 | 0.124135 | 0 | 0 | 0 | 0 | 0 |
| 650 | 0.00011 | 0.001172 | 0.872154 | 0.126564 | 0 | 0 | 0 | 0 | 0 |
| 651 | 0.00005 | 0.000943 | 0.872625 | 0.126382 | 0 | 0 | 0 | 0 | 0 |
| 652 | 0.00008 | 0.001186 | 0.871543 | 0.127191 | 0 | 0 | 0 | 0 | 0 |
| 653 | 0.00007 | 0.001274 | 0.87349 | 0.125166 | 0 | 0 | 0 | 0 | 0 |
| 654 | 0.00008 | 0.001214 | 0.874035 | 0.124671 | 0 | 0 | 0 | 0 | 0 |
| 655 | 0.00006 | 0.001304 | 0.869111 | 0.129525 | 0 | 0 | 0 | 0 | 0 |
| 656 | 0.00007 | 0.001081 | 0.878319 | 0.120529 | 0 | 0 | 0 | 0 | 0 |
| 657 | 0.00005 | 0.001089 | 0.873007 | 0.125854 | 0 | 0 | 0 | 0 | 0 |
| 658 | 0.00003 | 0.001312 | 0.870897 | 0.127761 | 0 | 0 | 0 | 0 | 0 |
| 659 | 0.00008 | 0.001436 | 0.872192 | 0.126292 | 0 | 0 | 0 | 0 | 0 |
| 660 | 0.00006 | 0.001317 | 0.876996 | 0.121627 | 0 | 0 | 0 | 0 | 0 |
| 661 | 0.000061 | 0.001135 | 0.871058 | 0.127746 | 0 | 0 | 0 | 0 | 0 |
| 662 | 0.00007 | 0.00139 | 0.869033 | 0.129507 | 0 | 0 | 0 | 0 | 0 |
| 663 | 0.00012 | 0.001049 | 0.872226 | 0.126606 | 0 | 0 | 0 | 0 | 0 |
| 664 | 0.00008 | 0.001381 | 0.876427 | 0.122092 | 0.00002 | 0 | 0 | 0 | 0 |
| 665 | 0.00008 | 0.001056 | 0.868749 | 0.130115 | 0 | 0 | 0 | 0 | 0 |
| 666 | 0.00008 | 0.001167 | 0.87218 | 0.126573 | 0 | 0 | 0 | 0 | 0 |
| 667 | 0.00007 | 0.001263 | 0.872418 | 0.126249 | 0 | 0 | 0 | 0 | 0 |
| 668 | 0.00007 | 0.001221 | 0.871007 | 0.127702 | 0 | 0 | 0 | 0 | 0 |
| 669 | 0.00008 | 0.001189 | 0.869642 | 0.12909 | 0 | 0 | 0 | 0 | 0 |
| 670 | 0.00006 | 0.001128 | 0.869671 | 0.129141 | 0 | 0 | 0 | 0 | 0 |
| 671 | 0.00009 | 0.001263 | 0.87195 | 0.126697 | 0 | 0 | 0 | 0 | 0 |
| 672 | 0.00006 | 0.001193 | 0.874107 | 0.12464 | 0 | 0 | 0 | 0 | 0 |
| 673 | 0.00008 | 0.001389 | 0.875242 | 0.123289 | 0 | 0 | 0 | 0 | 0 |
| 674 | 0.00011 | 0.001051 | 0.872908 | 0.125931 | 0 | 0 | 0 | 0 | 0 |
| 675 | 0.00002 | 0.001183 | 0.874194 | 0.124603 | 0 | 0 | 0 | 0 | 0 |
| 676 | 0.00009 | 0.001488 | 0.873492 | 0.12493 | 0 | 0 | 0 | 0 | 0 |
| 677 | 0.00006 | 0.00114 | 0.873201 | 0.125599 | 0 | 0 | 0 | 0 | 0 |
| 678 | 0.00007 | 0.001168 | 0.870163 | 0.128599 | 0 | 0 | 0 | 0 | 0 |
| 679 | 0.00008 | 0.001322 | 0.874308 | 0.12429 | 0 | 0 | 0 | 0 | 0 |
| 680 | 0.00004 | 0.001651 | 0.869873 | 0.128436 | 0 | 0 | 0 | 0 | 0 |
| 681 | 0.000131 | 0.001187 | 0.869178 | 0.129493 | 0.00001 | 0 | 0 | 0 | 0 |
| 682 | 0.00005 | 0.001047 | 0.871415 | 0.127488 | 0 | 0 | 0 | 0 | 0 |
| 683 | 0.00006 | 0.00117 | 0.874257 | 0.124514 | 0 | 0 | 0 | 0 | 0 |
| 684 | 0.000069 | 0.000953 | 0.8734 | 0.125577 | 0 | 0 | 0 | 0 | 0 |
| 685 | 0.00009 | 0.001125 | 0.876519 | 0.122266 | 0 | 0 | 0 | 0 | 0 |
| 686 | 0.0001 | 0.001248 | 0.86775 | 0.130872 | 0.00003 | 0 | 0 | 0 | 0 |
| 687 | 0.00007 | 0.001328 | 0.873387 | 0.125216 | 0 | 0 | 0 | 0 | 0 |
| 688 | 0.00006 | 0.001132 | 0.873261 | 0.125546 | 0 | 0 | 0 | 0 | 0 |
| 689 | 0.00005 | 0.001274 | 0.871985 | 0.126691 | 0 | 0 | 0 | 0 | 0 |
| 690 | 0.00006 | 0.001126 | 0.874912 | 0.123901 | 0 | 0 | 0 | 0 | 0 |
| 691 | 0.00004 | 0.001317 | 0.872113 | 0.126529 | 0 | 0 | 0 | 0 | 0 |
| 692 | 0.00008 | 0.001329 | 0.872772 | 0.125818 | 0 | 0 | 0 | 0 | 0 |
| 693 | 0.00011 | 0.001433 | 0.870072 | 0.128384 | 0 | 0 | 0 | 0 | 0 |
| 694 | 0.00002 | 0.001428 | 0.873752 | 0.1248 | 0 | 0 | 0 | 0 | 0 |
| 695 | 0.00006 | 0.000973 | 0.875336 | 0.12363 | 0 | 0 | 0 | 0 | 0 |
| 696 | 0.00004 | 0.001039 | 0.876267 | 0.122654 | 0 | 0 | 0 | 0 | 0 |
| 697 | 0.00008 | 0.001354 | 0.875525 | 0.12304 | 0 | 0 | 0 | 0 | 0 |
| 698 | 0.00013 | 0.001249 | 0.8723 | 0.126321 | 0 | 0 | 0 | 0 | 0 |
| 699 | 0.0001 | 0.001078 | 0.87446 | 0.124363 | 0 | 0 | 0 | 0 | 0 |
| 700 | 0.00008 | 0.001281 | 0.873786 | 0.124852 | 0 | 0 | 0 | 0 | 0 |
| 701 | 0.00007 | 0.001312 | 0.879965 | 0.118653 | 0 | 0 | 0 | 0 | 0 |
| 702 | 0.00013 | 0.001252 | 0.87802 | 0.120598 | 0 | 0 | 0 | 0 | 0 |
| 703 | 0.00004 | 0.001393 | 0.874522 | 0.124046 | 0 | 0 | 0 | 0 | 0 |
| 704 | 0.00006 | 0.001087 | 0.871876 | 0.126978 | 0 | 0 | 0 | 0 | 0 |
| 705 | 0.00005 | 0.00141 | 0.870908 | 0.127632 | 0 | 0 | 0 | 0 | 0 |
| 706 | 0.00005 | 0.001441 | 0.872038 | 0.126471 | 0 | 0 | 0 | 0 | 0 |
| 707 | 0.00004 | 0.001173 | 0.869834 | 0.128953 | 0 | 0 | 0 | 0 | 0 |
| 708 | 0.00005 | 0.001519 | 0.873285 | 0.125146 | 0 | 0 | 0 | 0 | 0 |
| 709 | 0.00008 | 0.001251 | 0.877646 | 0.121022 | 0 | 0 | 0 | 0 | 0 |
| 710 | 0.00004 | 0.001331 | 0.872467 | 0.126161 | 0 | 0 | 0 | 0 | 0 |
| 711 | 0.00005 | 0.001182 | 0.873965 | 0.124803 | 0 | 0 | 0 | 0 | 0 |
| 712 | 0.00006 | 0.001349 | 0.878707 | 0.119884 | 0 | 0 | 0 | 0 | 0 |
| 713 | 0.00007 | 0.000972 | 0.870471 | 0.128477 | 0.00001 | 0 | 0 | 0 | 0 |
| 714 | 0.00007 | 0.001167 | 0.872949 | 0.125814 | 0 | 0 | 0 | 0 | 0 |
| 715 | 0.00004 | 0.001022 | 0.867062 | 0.131876 | 0 | 0 | 0 | 0 | 0 |
| 716 | 0.00003 | 0.001344 | 0.867375 | 0.131251 | 0 | 0 | 0 | 0 | 0 |
| 717 | 0.000101 | 0.001348 | 0.871644 | 0.126908 | 0 | 0 | 0 | 0 | 0 |
| 718 | 0.00005 | 0.001417 | 0.877006 | 0.121527 | 0 | 0 | 0 | 0 | 0 |
| 719 | 0.00006 | 0.001122 | 0.87399 | 0.124829 | 0 | 0 | 0 | 0 | 0 |
| 720 | 0.00008 | 0.001065 | 0.869391 | 0.129414 | 0.00005 | 0 | 0 | 0 | 0 |
| 721 | 0.00007 | 0.001233 | 0.867299 | 0.131398 | 0 | 0 | 0 | 0 | 0 |
| 722 | 0.00003 | 0.001301 | 0.873114 | 0.125555 | 0 | 0 | 0 | 0 | 0 |
| 723 | 0.00011 | 0.001478 | 0.871048 | 0.127364 | 0 | 0 | 0 | 0 | 0 |
| 724 | 0.0001 | 0.001369 | 0.873827 | 0.124704 | 0 | 0 | 0 | 0 | 0 |
| 725 | 0.00008 | 0.00137 | 0.870357 | 0.128194 | 0 | 0 | 0 | 0 | 0 |
| 726 | 0.00009 | 0.001271 | 0.873738 | 0.124901 | 0 | 0 | 0 | 0 | 0 |
| 727 | 0.000101 | 0.001096 | 0.869986 | 0.128818 | 0 | 0 | 0 | 0 | 0 |
| 728 | 0.00006 | 0.00128 | 0.873372 | 0.125289 | 0 | 0 | 0 | 0 | 0 |
| 729 | 0.00002 | 0.001593 | 0.872376 | 0.126012 | 0 | 0 | 0 | 0 | 0 |
| 730 | 0.00009 | 0.001211 | 0.873814 | 0.124885 | 0 | 0 | 0 | 0 | 0 |
| 731 | 0.00007 | 0.001438 | 0.874556 | 0.123937 | 0 | 0 | 0 | 0 | 0 |
| 732 | 0.00005 | 0.001093 | 0.874925 | 0.123932 | 0 | 0 | 0 | 0 | 0 |
| 733 | 0.00009 | 0.001293 | 0.875009 | 0.123608 | 0 | 0 | 0 | 0 | 0 |
| 734 | 0.00009 | 0.001099 | 0.873385 | 0.125426 | 0 | 0 | 0 | 0 | 0 |
| 735 | 0.0001 | 0.001173 | 0.875817 | 0.12291 | 0 | 0 | 0 | 0 | 0 |
| 736 | 0.00008 | 0.001213 | 0.874806 | 0.123901 | 0 | 0 | 0 | 0 | 0 |
| 737 | 0.0001 | 0.001254 | 0.873835 | 0.124811 | 0 | 0 | 0 | 0 | 0 |
| 738 | 0.00008 | 0.001045 | 0.869696 | 0.129178 | 0 | 0 | 0 | 0 | 0 |
| 739 | 0.00004 | 0.001135 | 0.86996 | 0.128865 | 0 | 0 | 0 | 0 | 0 |
| 740 | 0.00004 | 0.001476 | 0.87605 | 0.122404 | 0.00003 | 0 | 0 | 0 | 0 |
| 741 | 0.00004 | 0.001245 | 0.87327 | 0.125445 | 0 | 0 | 0 | 0 | 0 |
| 742 | 0.00004 | 0.001168 | 0.874641 | 0.124152 | 0 | 0 | 0 | 0 | 0 |
| 743 | 0.000099 | 0.0013 | 0.871624 | 0.126977 | 0 | 0 | 0 | 0 | 0 |
| 744 | 0.0001 | 0.001505 | 0.876143 | 0.122213 | 0.00004 | 0 | 0 | 0 | 0 |
| 745 | 0.00007 | 0.001125 | 0.878094 | 0.120691 | 0.00002 | 0 | 0 | 0 | 0 |
| 746 | 0.00006 | 0.001079 | 0.870641 | 0.12822 | 0 | 0 | 0 | 0 | 0 |
| 747 | 0.00004 | 0.001214 | 0.870885 | 0.127861 | 0 | 0 | 0 | 0 | 0 |
| 748 | 0.00004 | 0.001229 | 0.876478 | 0.122253 | 0 | 0 | 0 | 0 | 0 |
| 749 | 0.00004 | 0.001048 | 0.87698 | 0.121933 | 0 | 0 | 0 | 0 | 0 |
| 750 | 0.00009 | 0.001073 | 0.874398 | 0.124438 | 0 | 0 | 0 | 0 | 0 |
| 751 | 0.00007 | 0.001138 | 0.876003 | 0.12279 | 0 | 0 | 0 | 0 | 0 |
| 752 | 0.0001 | 0.001316 | 0.872591 | 0.125993 | 0 | 0 | 0 | 0 | 0 |
| 753 | 0.00009 | 0.001065 | 0.875259 | 0.123586 | 0 | 0 | 0 | 0 | 0 |
| 754 | 0.00008 | 0.001236 | 0.871692 | 0.126991 | 0 | 0 | 0 | 0 | 0 |
| 755 | 0.00008 | 0.001365 | 0.870685 | 0.12787 | 0 | 0 | 0 | 0 | 0 |
| 756 | 0.00007 | 0.00104 | 0.871246 | 0.127644 | 0 | 0 | 0 | 0 | 0 |
| 757 | 0.00011 | 0.001315 | 0.87182 | 0.126755 | 0 | 0 | 0 | 0 | 0 |
| 758 | 0.00008 | 0.001404 | 0.870828 | 0.127688 | 0 | 0 | 0 | 0 | 0 |
| 759 | 0.00007 | 0.001162 | 0.871328 | 0.127441 | 0 | 0 | 0 | 0 | 0 |
| 760 | 0.00005 | 0.001414 | 0.873345 | 0.125191 | 0 | 0 | 0 | 0 | 0 |
| 761 | 0.00014 | 0.001159 | 0.871194 | 0.127508 | 0 | 0 | 0 | 0 | 0 |
| 762 | 0.0001 | 0.001186 | 0.873272 | 0.125442 | 0 | 0 | 0 | 0 | 0 |
| 763 | 0.00009 | 0.001513 | 0.873852 | 0.124546 | 0 | 0 | 0 | 0 | 0 |
| 764 | 0.000129 | 0.001083 | 0.875512 | 0.123277 | 0 | 0 | 0 | 0 | 0 |
| 765 | 0.00004 | 0.001352 | 0.8729 | 0.125707 | 0 | 0 | 0 | 0 | 0 |
| 766 | 0.00004 | 0.000979 | 0.869877 | 0.129104 | 0 | 0 | 0 | 0 | 0 |
| 767 | 0.00008 | 0.000881 | 0.874494 | 0.124544 | 0 | 0 | 0 | 0 | 0 |
| 768 | 0.00013 | 0.001298 | 0.87216 | 0.126413 | 0 | 0 | 0 | 0 | 0 |
| 769 | 0.00011 | 0.001316 | 0.87445 | 0.124124 | 0 | 0 | 0 | 0 | 0 |
| 770 | 0.00006 | 0.001181 | 0.872456 | 0.126303 | 0 | 0 | 0 | 0 | 0 |
| 771 | 0.00005 | 0.001232 | 0.873537 | 0.125101 | 0.00008 | 0 | 0 | 0 | 0 |
| 772 | 0.00013 | 0.001213 | 0.871775 | 0.126872 | 0.00001 | 0 | 0 | 0 | 0 |
| 773 | 0.00008 | 0.001253 | 0.870099 | 0.128568 | 0 | 0 | 0 | 0 | 0 |
| 774 | 0.000139 | 0.001184 | 0.87243 | 0.126247 | 0 | 0 | 0 | 0 | 0 |
| 775 | 0.000049 | 0.001471 | 0.873388 | 0.125091 | 0 | 0 | 0 | 0 | 0 |
| 776 | 0.00006 | 0.001364 | 0.871327 | 0.127248 | 0 | 0 | 0 | 0 | 0 |
| 777 | 0.0001 | 0.001296 | 0.874832 | 0.123772 | 0 | 0 | 0 | 0 | 0 |
| 778 | 0.0001 | 0.001293 | 0.876438 | 0.122169 | 0 | 0 | 0 | 0 | 0 |
| 779 | 0.00005 | 0.00118 | 0.877345 | 0.121425 | 0 | 0 | 0 | 0 | 0 |
| 780 | 0.00007 | 0.001248 | 0.872665 | 0.126017 | 0 | 0 | 0 | 0 | 0 |
| 781 | 0.00008 | 0.001177 | 0.871393 | 0.12735 | 0 | 0 | 0 | 0 | 0 |
| 782 | 0.00006 | 0.001294 | 0.876479 | 0.122167 | 0 | 0 | 0 | 0 | 0 |
| 783 | 0.00007 | 0.001385 | 0.872859 | 0.125685 | 0 | 0 | 0 | 0 | 0 |
| 784 | 0.0001 | 0.001278 | 0.872532 | 0.126091 | 0 | 0 | 0 | 0 | 0 |
| 785 | 0.00004 | 0.001155 | 0.87191 | 0.126894 | 0 | 0 | 0 | 0 | 0 |
| 786 | 0.00005 | 0.001356 | 0.870009 | 0.128585 | 0 | 0 | 0 | 0 | 0 |
| 787 | 0.00006 | 0.001243 | 0.87449 | 0.124207 | 0 | 0 | 0 | 0 | 0 |
| 788 | 0.00003 | 0.001543 | 0.875173 | 0.123254 | 0 | 0 | 0 | 0 | 0 |
| 789 | 0.00008 | 0.001159 | 0.870115 | 0.128646 | 0 | 0 | 0 | 0 | 0 |
| 790 | 0.00005 | 0.001089 | 0.87473 | 0.12413 | 0 | 0 | 0 | 0 | 0 |
| 791 | 0.00005 | 0.000991 | 0.87519 | 0.123769 | 0 | 0 | 0 | 0 | 0 |
| 792 | 0.00013 | 0.001232 | 0.875288 | 0.12335 | 0 | 0 | 0 | 0 | 0 |
| 793 | 0.00008 | 0.001287 | 0.87054 | 0.128093 | 0 | 0 | 0 | 0 | 0 |
| 794 | 0.00011 | 0.001265 | 0.875023 | 0.123602 | 0 | 0 | 0 | 0 | 0 |
| 795 | 0.0001 | 0.001226 | 0.869543 | 0.12913 | 0 | 0 | 0 | 0 | 0 |
| 796 | 0.00004 | 0.001191 | 0.873323 | 0.125446 | 0 | 0 | 0 | 0 | 0 |
| 797 | 0.00005 | 0.001519 | 0.871158 | 0.127273 | 0 | 0 | 0 | 0 | 0 |
| 798 | 0.00006 | 0.001121 | 0.871608 | 0.127202 | 0.00001 | 0 | 0 | 0 | 0 |
| 799 | 0.00009 | 0.001058 | 0.873068 | 0.125784 | 0 | 0 | 0 | 0 | 0 |
| 800 | 0.00008 | 0.001322 | 0.870304 | 0.128294 | 0 | 0 | 0 | 0 | 0 |
| 801 | 0.00007 | 0.001209 | 0.877394 | 0.121317 | 0.00001 | 0 | 0 | 0 | 0 |
| 802 | 0.00008 | 0.001356 | 0.873175 | 0.12539 | 0 | 0 | 0 | 0 | 0 |
| 803 | 0.00007 | 0.001239 | 0.876154 | 0.122537 | 0 | 0 | 0 | 0 | 0 |
| 804 | 0.00006 | 0.001137 | 0.872663 | 0.12614 | 0 | 0 | 0 | 0 | 0 |
| 805 | 0.00003 | 0.001186 | 0.876401 | 0.122383 | 0 | 0 | 0 | 0 | 0 |
| 806 | 0.00006 | 0.001419 | 0.87197 | 0.12655 | 0 | 0 | 0 | 0 | 0 |
| 807 | 0.00009 | 0.001434 | 0.873554 | 0.124922 | 0 | 0 | 0 | 0 | 0 |
| 808 | 0.00007 | 0.00109 | 0.874131 | 0.124709 | 0 | 0 | 0 | 0 | 0 |
| 809 | 0.00008 | 0.001055 | 0.87269 | 0.126175 | 0 | 0 | 0 | 0 | 0 |
| 810 | 0.00012 | 0.001178 | 0.870248 | 0.128454 | 0 | 0 | 0 | 0 | 0 |
| 811 | 0.00005 | 0.001157 | 0.875661 | 0.123132 | 0 | 0 | 0 | 0 | 0 |
| 812 | 0.00006 | 0.000956 | 0.878363 | 0.120621 | 0 | 0 | 0 | 0 | 0 |
| 813 | 0.00011 | 0.001107 | 0.876845 | 0.121938 | 0 | 0 | 0 | 0 | 0 |
| 814 | 0.00012 | 0.001484 | 0.872912 | 0.125484 | 0 | 0 | 0 | 0 | 0 |
| 815 | 0.00006 | 0.001135 | 0.870621 | 0.128184 | 0 | 0 | 0 | 0 | 0 |
| 816 | 0.00006 | 0.001111 | 0.87501 | 0.123819 | 0 | 0 | 0 | 0 | 0 |
| 817 | 0.00006 | 0.001234 | 0.877958 | 0.120748 | 0 | 0 | 0 | 0 | 0 |
| 818 | 0.00004 | 0.001056 | 0.870818 | 0.128086 | 0 | 0 | 0 | 0 | 0 |
| 819 | 0.00006 | 0.001077 | 0.869651 | 0.129212 | 0 | 0 | 0 | 0 | 0 |
| 820 | 0.00005 | 0.001031 | 0.87461 | 0.124309 | 0 | 0 | 0 | 0 | 0 |
| 821 | 0.00006 | 0.00135 | 0.876386 | 0.122204 | 0 | 0 | 0 | 0 | 0 |
| 822 | 0.000081 | 0.001301 | 0.87384 | 0.124778 | 0 | 0 | 0 | 0 | 0 |
| 823 | 0.000109 | 0.001342 | 0.872953 | 0.125596 | 0 | 0 | 0 | 0 | 0 |
| 824 | 0.00009 | 0.001414 | 0.877128 | 0.121368 | 0 | 0 | 0 | 0 | 0 |
| 825 | 0.00007 | 0.001376 | 0.874172 | 0.124383 | 0 | 0 | 0 | 0 | 0 |
| 826 | 0.000089 | 0.00119 | 0.87448 | 0.12424 | 0 | 0 | 0 | 0 | 0 |
| 827 | 0.00005 | 0.001571 | 0.872942 | 0.125437 | 0 | 0 | 0 | 0 | 0 |
| 828 | 0.00007 | 0.001026 | 0.875497 | 0.123407 | 0 | 0 | 0 | 0 | 0 |
| 829 | 0.0001 | 0.0013 | 0.874676 | 0.123923 | 0 | 0 | 0 | 0 | 0 |
| 830 | 0.00013 | 0.001134 | 0.874294 | 0.124442 | 0 | 0 | 0 | 0 | 0 |
| 831 | 0.00005 | 0.001247 | 0.870518 | 0.128185 | 0 | 0 | 0 | 0 | 0 |
| 832 | 0.00007 | 0.001229 | 0.87407 | 0.124632 | 0 | 0 | 0 | 0 | 0 |
| 833 | 0.00008 | 0.001414 | 0.872734 | 0.125773 | 0 | 0 | 0 | 0 | 0 |
| 834 | 0.00007 | 0.001332 | 0.873485 | 0.125093 | 0.00002 | 0 | 0 | 0 | 0 |
| 835 | 0.00004 | 0.001393 | 0.869253 | 0.129314 | 0 | 0 | 0 | 0 | 0 |
| 836 | 0.00009 | 0.001345 | 0.871948 | 0.126617 | 0 | 0 | 0 | 0 | 0 |
| 837 | 0.00008 | 0.001224 | 0.875658 | 0.123037 | 0 | 0 | 0 | 0 | 0 |
| 838 | 0.00005 | 0.001153 | 0.873786 | 0.125011 | 0 | 0 | 0 | 0 | 0 |
| 839 | 0.00006 | 0.001211 | 0.870863 | 0.127866 | 0 | 0 | 0 | 0 | 0 |
| 840 | 0.00008 | 0.001114 | 0.870841 | 0.127966 | 0 | 0 | 0 | 0 | 0 |
| 841 | 0.00011 | 0.001506 | 0.877829 | 0.120556 | 0 | 0 | 0 | 0 | 0 |
| 842 | 0.00005 | 0.000987 | 0.869398 | 0.129565 | 0 | 0 | 0 | 0 | 0 |
| 843 | 0.00004 | 0.001358 | 0.873661 | 0.124941 | 0 | 0 | 0 | 0 | 0 |
| 844 | 0.00009 | 0.001279 | 0.873047 | 0.125584 | 0 | 0 | 0 | 0 | 0 |
| 845 | 0.00004 | 0.001173 | 0.869963 | 0.128824 | 0 | 0 | 0 | 0 | 0 |
| 846 | 0.00008 | 0.001157 | 0.870971 | 0.127791 | 0 | 0 | 0 | 0 | 0 |
| 847 | 0.00006 | 0.001404 | 0.872565 | 0.12597 | 0 | 0 | 0 | 0 | 0 |
| 848 | 0.00009 | 0.001398 | 0.876642 | 0.12187 | 0 | 0 | 0 | 0 | 0 |
| 849 | 0.00004 | 0.00137 | 0.876372 | 0.122218 | 0 | 0 | 0 | 0 | 0 |
| 850 | 0.00002 | 0.001266 | 0.872955 | 0.12576 | 0 | 0 | 0 | 0 | 0 |
| 851 | 0.00005 | 0.001365 | 0.873084 | 0.125442 | 0.00006 | 0 | 0 | 0 | 0 |
| 852 | 0.00007 | 0.001331 | 0.871475 | 0.127124 | 0 | 0 | 0 | 0 | 0 |
| 853 | 0.00008 | 0.001204 | 0.869094 | 0.129622 | 0 | 0 | 0 | 0 | 0 |
| 854 | 0.00007 | 0.0011 | 0.870038 | 0.128792 | 0 | 0 | 0 | 0 | 0 |
| 855 | 0.00004 | 0.001437 | 0.87234 | 0.126183 | 0 | 0 | 0 | 0 | 0 |
| 856 | 0.00008 | 0.001481 | 0.868231 | 0.130208 | 0 | 0 | 0 | 0 | 0 |
| 857 | 0.00007 | 0.001194 | 0.869999 | 0.128737 | 0 | 0 | 0 | 0 | 0 |
| 858 | 0.00011 | 0.001289 | 0.87286 | 0.125742 | 0 | 0 | 0 | 0 | 0 |
| 859 | 0.00008 | 0.001522 | 0.87455 | 0.123849 | 0 | 0 | 0 | 0 | 0 |
| 860 | 0.00007 | 0.001232 | 0.87346 | 0.125238 | 0 | 0 | 0 | 0 | 0 |
| 861 | 0.00006 | 0.001386 | 0.875481 | 0.123063 | 0.00001 | 0 | 0 | 0 | 0 |
| 862 | 0.00003 | 0.00134 | 0.873634 | 0.124996 | 0 | 0 | 0 | 0 | 0 |
| 863 | 0.00006 | 0.001183 | 0.872801 | 0.125956 | 0 | 0 | 0 | 0 | 0 |
| 864 | 0.00008 | 0.00127 | 0.872137 | 0.126513 | 0 | 0 | 0 | 0 | 0 |
| 865 | 0.00002 | 0.001024 | 0.873619 | 0.125337 | 0 | 0 | 0 | 0 | 0 |
| 866 | 0.00004 | 0.001093 | 0.874068 | 0.124798 | 0 | 0 | 0 | 0 | 0 |
| 867 | 0.00006 | 0.001171 | 0.870685 | 0.128084 | 0 | 0 | 0 | 0 | 0 |
| 868 | 0.0001 | 0.001386 | 0.869361 | 0.129152 | 0 | 0 | 0 | 0 | 0 |
| 869 | 0.00007 | 0.001133 | 0.869898 | 0.128889 | 0.00001 | 0 | 0 | 0 | 0 |
| 870 | 0.00008 | 0.001094 | 0.871722 | 0.127104 | 0 | 0 | 0 | 0 | 0 |
| 871 | 0.00008 | 0.001045 | 0.877096 | 0.12178 | 0 | 0 | 0 | 0 | 0 |
| 872 | 0.000059 | 0.00105 | 0.871371 | 0.12752 | 0 | 0 | 0 | 0 | 0 |
| 873 | 0.00011 | 0.001267 | 0.87363 | 0.124994 | 0 | 0 | 0 | 0 | 0 |
| 874 | 0.00005 | 0.001091 | 0.872338 | 0.126521 | 0 | 0 | 0 | 0 | 0 |
| 875 | 0.00011 | 0.001091 | 0.874861 | 0.123938 | 0 | 0 | 0 | 0 | 0 |
| 876 | 0.00002 | 0.001432 | 0.874091 | 0.124457 | 0 | 0 | 0 | 0 | 0 |
| 877 | 0.00007 | 0.001178 | 0.874126 | 0.124627 | 0 | 0 | 0 | 0 | 0 |
| 878 | 0.00006 | 0.00119 | 0.87262 | 0.12613 | 0 | 0 | 0 | 0 | 0 |
| 879 | 0.000109 | 0.000995 | 0.870989 | 0.127907 | 0 | 0 | 0 | 0 | 0 |
| 880 | 0.00009 | 0.001089 | 0.874297 | 0.124524 | 0 | 0 | 0 | 0 | 0 |
| 881 | 0.00003 | 0.001029 | 0.870112 | 0.128829 | 0 | 0 | 0 | 0 | 0 |
| 882 | 0.00003 | 0.001239 | 0.869421 | 0.12928 | 0.00003 | 0 | 0 | 0 | 0 |
| 883 | 0.00007 | 0.001248 | 0.871336 | 0.127317 | 0.00003 | 0 | 0 | 0 | 0 |
| 884 | 0.00004 | 0.001138 | 0.871251 | 0.127571 | 0 | 0 | 0 | 0 | 0 |
| 885 | 0.00005 | 0.001235 | 0.875667 | 0.123047 | 0 | 0 | 0 | 0 | 0 |
| 886 | 0.00005 | 0.00116 | 0.872473 | 0.126317 | 0 | 0 | 0 | 0 | 0 |
| 887 | 0.00006 | 0.001346 | 0.873737 | 0.124857 | 0 | 0 | 0 | 0 | 0 |
| 888 | 0.00008 | 0.001118 | 0.867811 | 0.130991 | 0 | 0 | 0 | 0 | 0 |
| 889 | 0.00007 | 0.001154 | 0.868432 | 0.130345 | 0 | 0 | 0 | 0 | 0 |
| 890 | 0.00008 | 0.001342 | 0.875413 | 0.123164 | 0 | 0 | 0 | 0 | 0 |
| 891 | 0.00013 | 0.001164 | 0.870093 | 0.128613 | 0 | 0 | 0 | 0 | 0 |
| 892 | 0.00007 | 0.001176 | 0.873255 | 0.125499 | 0 | 0 | 0 | 0 | 0 |
| 893 | 0.0001 | 0.001434 | 0.872338 | 0.126129 | 0 | 0 | 0 | 0 | 0 |
| 894 | 0.00002 | 0.001353 | 0.869891 | 0.128736 | 0 | 0 | 0 | 0 | 0 |
| 895 | 0.00007 | 0.001278 | 0.87709 | 0.121562 | 0 | 0 | 0 | 0 | 0 |
| 896 | 0.00005 | 0.001178 | 0.875252 | 0.12352 | 0 | 0 | 0 | 0 | 0 |
| 897 | 0.00008 | 0.001257 | 0.874332 | 0.124332 | 0 | 0 | 0 | 0 | 0 |
| 898 | 0.00007 | 0.00083 | 0.873199 | 0.125901 | 0 | 0 | 0 | 0 | 0 |
| 899 | 0.00006 | 0.001067 | 0.873016 | 0.125857 | 0 | 0 | 0 | 0 | 0 |
| 900 | 0.00005 | 0.001516 | 0.871801 | 0.126633 | 0 | 0 | 0 | 0 | 0 |
| 901 | 0.00007 | 0.001318 | 0.879851 | 0.118761 | 0 | 0 | 0 | 0 | 0 |
| 902 | 0.00004 | 0.000867 | 0.87369 | 0.125402 | 0 | 0 | 0 | 0 | 0 |
| 903 | 0.00008 | 0.001128 | 0.873175 | 0.125617 | 0 | 0 | 0 | 0 | 0 |
| 904 | 0.00008 | 0.001269 | 0.875036 | 0.123615 | 0 | 0 | 0 | 0 | 0 |
| 905 | 0.00007 | 0.001142 | 0.874084 | 0.124705 | 0 | 0 | 0 | 0 | 0 |
| 906 | 0.00004 | 0.001105 | 0.876031 | 0.122824 | 0 | 0 | 0 | 0 | 0 |
| 907 | 0.00009 | 0.001135 | 0.87704 | 0.121734 | 0 | 0 | 0 | 0 | 0 |
| 908 | 0.00011 | 0.001474 | 0.873794 | 0.124621 | 0 | 0 | 0 | 0 | 0 |
| 909 | 0.00007 | 0.00108 | 0.873311 | 0.125539 | 0 | 0 | 0 | 0 | 0 |
| 910 | 0.00007 | 0.001249 | 0.871584 | 0.127097 | 0 | 0 | 0 | 0 | 0 |
| 911 | 0.00004 | 0.001137 | 0.875676 | 0.123147 | 0 | 0 | 0 | 0 | 0 |
| 912 | 0.00007 | 0.001103 | 0.871482 | 0.127345 | 0 | 0 | 0 | 0 | 0 |
| 913 | 0.00007 | 0.00129 | 0.876415 | 0.122226 | 0 | 0 | 0 | 0 | 0 |
| 914 | 0.00005 | 0.00137 | 0.876778 | 0.121802 | 0 | 0 | 0 | 0 | 0 |
| 915 | 0.00005 | 0.000962 | 0.871174 | 0.127813 | 0 | 0 | 0 | 0 | 0 |
| 916 | 0.00002 | 0.001411 | 0.873704 | 0.124865 | 0 | 0 | 0 | 0 | 0 |
| 917 | 0.00008 | 0.001197 | 0.873582 | 0.125141 | 0 | 0 | 0 | 0 | 0 |
| 918 | 0.0001 | 0.001053 | 0.874831 | 0.124016 | 0 | 0 | 0 | 0 | 0 |
| 919 | 0.00005 | 0.001393 | 0.868837 | 0.12972 | 0 | 0 | 0 | 0 | 0 |
| 920 | 0.0001 | 0.001148 | 0.877401 | 0.12135 | 0 | 0 | 0 | 0 | 0 |
| 921 | 0.00006 | 0.001177 | 0.872404 | 0.126359 | 0 | 0 | 0 | 0 | 0 |
| 922 | 0.000081 | 0.001097 | 0.876917 | 0.121906 | 0 | 0 | 0 | 0 | 0 |
| 923 | 0.00007 | 0.001146 | 0.874103 | 0.124681 | 0 | 0 | 0 | 0 | 0 |
| 924 | 0.00006 | 0.001176 | 0.87141 | 0.127353 | 0 | 0 | 0 | 0 | 0 |
| 925 | 0.00006 | 0.000999 | 0.872981 | 0.12595 | 0.00001 | 0 | 0 | 0 | 0 |
| 926 | 0.00016 | 0.001428 | 0.87285 | 0.125562 | 0 | 0 | 0 | 0 | 0 |
| 927 | 0.00008 | 0.00117 | 0.873222 | 0.125529 | 0 | 0 | 0 | 0 | 0 |
| 928 | 0.00009 | 0.001238 | 0.870252 | 0.12842 | 0 | 0 | 0 | 0 | 0 |
| 929 | 0.00008 | 0.001288 | 0.868196 | 0.130436 | 0 | 0 | 0 | 0 | 0 |
| 930 | 0.00003 | 0.001435 | 0.875276 | 0.123259 | 0 | 0 | 0 | 0 | 0 |
| 931 | 0.00003 | 0.001239 | 0.87676 | 0.121971 | 0 | 0 | 0 | 0 | 0 |
| 932 | 0.00009 | 0.001218 | 0.873117 | 0.125575 | 0 | 0 | 0 | 0 | 0 |
| 933 | 0.00008 | 0.001123 | 0.877917 | 0.120879 | 0 | 0 | 0 | 0 | 0 |
| 934 | 0.00002 | 0.001118 | 0.86994 | 0.128923 | 0 | 0 | 0 | 0 | 0 |
| 935 | 0.00003 | 0.00132 | 0.873646 | 0.125004 | 0 | 0 | 0 | 0 | 0 |
| 936 | 0.00009 | 0.001125 | 0.874327 | 0.124458 | 0 | 0 | 0 | 0 | 0 |
| 937 | 0.00008 | 0.001309 | 0.878053 | 0.120558 | 0 | 0 | 0 | 0 | 0 |
| 938 | 0.00004 | 0.001154 | 0.872865 | 0.125941 | 0 | 0 | 0 | 0 | 0 |
| 939 | 0.00006 | 0.001013 | 0.877844 | 0.121082 | 0 | 0 | 0 | 0 | 0 |
| 940 | 0.00005 | 0.001134 | 0.869335 | 0.129482 | 0 | 0 | 0 | 0 | 0 |
| 941 | 0.00004 | 0.001247 | 0.87345 | 0.125263 | 0 | 0 | 0 | 0 | 0 |
| 942 | 0.00005 | 0.001444 | 0.874936 | 0.12357 | 0 | 0 | 0 | 0 | 0 |
| 943 | 0.000099 | 0.001172 | 0.878552 | 0.120177 | 0 | 0 | 0 | 0 | 0 |
| 944 | 0.00006 | 0.001069 | 0.87008 | 0.128792 | 0 | 0 | 0 | 0 | 0 |
| 945 | 0.00007 | 0.001368 | 0.869069 | 0.129493 | 0 | 0 | 0 | 0 | 0 |
| 946 | 0.00007 | 0.00108 | 0.876513 | 0.122336 | 0 | 0 | 0 | 0 | 0 |
| 947 | 0.00003 | 0.001166 | 0.87268 | 0.126125 | 0 | 0 | 0 | 0 | 0 |
| 948 | 0.00007 | 0.001141 | 0.872964 | 0.125825 | 0 | 0 | 0 | 0 | 0 |
| 949 | 0.000051 | 0.001032 | 0.878337 | 0.120581 | 0 | 0 | 0 | 0 | 0 |
| 950 | 0.00005 | 0.001112 | 0.873691 | 0.125148 | 0 | 0 | 0 | 0 | 0 |
| 951 | 0.00008 | 0.001356 | 0.875656 | 0.122908 | 0 | 0 | 0 | 0 | 0 |
| 952 | 0.00007 | 0.001193 | 0.877025 | 0.121713 | 0 | 0 | 0 | 0 | 0 |
| 953 | 0.00005 | 0.001044 | 0.871862 | 0.127044 | 0 | 0 | 0 | 0 | 0 |
| 954 | 0.00014 | 0.001231 | 0.870455 | 0.128174 | 0 | 0 | 0 | 0 | 0 |
| 955 | 0.00007 | 0.001142 | 0.873263 | 0.125525 | 0 | 0 | 0 | 0 | 0 |
| 956 | 0.00013 | 0.001298 | 0.872891 | 0.125681 | 0 | 0 | 0 | 0 | 0 |
| 957 | 0.00005 | 0.001503 | 0.871034 | 0.127413 | 0 | 0 | 0 | 0 | 0 |
| 958 | 0.00008 | 0.001355 | 0.874994 | 0.123571 | 0 | 0 | 0 | 0 | 0 |
| 959 | 0.00005 | 0.001126 | 0.868917 | 0.129907 | 0 | 0 | 0 | 0 | 0 |
| 960 | 0.00006 | 0.001464 | 0.873366 | 0.125111 | 0 | 0 | 0 | 0 | 0 |
| 961 | 0.00014 | 0.001311 | 0.873059 | 0.12548 | 0.00001 | 0 | 0 | 0 | 0 |
| 962 | 0.00003 | 0.001322 | 0.874545 | 0.124103 | 0 | 0 | 0 | 0 | 0 |
| 963 | 0.00004 | 0.001039 | 0.873692 | 0.125229 | 0 | 0 | 0 | 0 | 0 |
| 964 | 0.00009 | 0.001206 | 0.874862 | 0.123841 | 0 | 0 | 0 | 0 | 0 |
| 965 | 0.00006 | 0.001248 | 0.875067 | 0.123625 | 0 | 0 | 0 | 0 | 0 |
| 966 | 0.00002 | 0.001316 | 0.876153 | 0.122511 | 0 | 0 | 0 | 0 | 0 |
| 967 | 0.00001 | 0.001274 | 0.873243 | 0.125473 | 0 | 0 | 0 | 0 | 0 |
| 968 | 0.00005 | 0.001388 | 0.872653 | 0.125908 | 0 | 0 | 0 | 0 | 0 |
| 969 | 0.00009 | 0.00118 | 0.874162 | 0.124567 | 0 | 0 | 0 | 0 | 0 |
| 970 | 0.00005 | 0.001582 | 0.870748 | 0.12762 | 0 | 0 | 0 | 0 | 0 |
| 971 | 0.00015 | 0.001359 | 0.877239 | 0.121251 | 0 | 0 | 0 | 0 | 0 |
| 972 | 0.000141 | 0.001362 | 0.869661 | 0.128836 | 0 | 0 | 0 | 0 | 0 |
| 973 | 0.00008 | 0.001265 | 0.871851 | 0.126794 | 0.00001 | 0 | 0 | 0 | 0 |
| 974 | 0.00005 | 0.0013 | 0.873471 | 0.125179 | 0 | 0 | 0 | 0 | 0 |
| 975 | 0.0001 | 0.001314 | 0.874016 | 0.124571 | 0 | 0 | 0 | 0 | 0 |
| 976 | 0.00007 | 0.001042 | 0.873194 | 0.125694 | 0 | 0 | 0 | 0 | 0 |
| 977 | 0.00005 | 0.000997 | 0.872152 | 0.1268 | 0 | 0 | 0 | 0 | 0 |
| 978 | 0.00006 | 0.001261 | 0.875205 | 0.123474 | 0 | 0 | 0 | 0 | 0 |
| 979 | 0.00008 | 0.00111 | 0.872366 | 0.126445 | 0 | 0 | 0 | 0 | 0 |
| 980 | 0.00007 | 0.001077 | 0.875481 | 0.123372 | 0 | 0 | 0 | 0 | 0 |
| 981 | 0.00005 | 0.001088 | 0.874305 | 0.124557 | 0 | 0 | 0 | 0 | 0 |
| 982 | 0.00005 | 0.001281 | 0.87031 | 0.128359 | 0 | 0 | 0 | 0 | 0 |
| 983 | 0.00011 | 0.001297 | 0.869931 | 0.128662 | 0 | 0 | 0 | 0 | 0 |
| 984 | 0.00004 | 0.00146 | 0.874172 | 0.124327 | 0 | 0 | 0 | 0 | 0 |
| 985 | 0.00008 | 0.000997 | 0.875429 | 0.123494 | 0 | 0 | 0 | 0 | 0 |
| 986 | 0.00006 | 0.001274 | 0.876129 | 0.122537 | 0 | 0 | 0 | 0 | 0 |
| 987 | 0.00004 | 0.001056 | 0.876227 | 0.122678 | 0 | 0 | 0 | 0 | 0 |
| 988 | 0.0001 | 0.001464 | 0.871511 | 0.126925 | 0 | 0 | 0 | 0 | 0 |
| 989 | 0.00005 | 0.001307 | 0.871063 | 0.12758 | 0 | 0 | 0 | 0 | 0 |
| 990 | 0.00006 | 0.001378 | 0.873171 | 0.125351 | 0.00004 | 0 | 0 | 0 | 0 |
| 991 | 0.00008 | 0.00158 | 0.874265 | 0.124075 | 0 | 0 | 0 | 0 | 0 |
| 992 | 0.00011 | 0.001371 | 0.874303 | 0.124215 | 0 | 0 | 0 | 0 | 0 |
| 993 | 0.00007 | 0.001259 | 0.878025 | 0.120646 | 0 | 0 | 0 | 0 | 0 |
| 994 | 0.00003 | 0.001006 | 0.876717 | 0.122246 | 0 | 0 | 0 | 0 | 0 |
| 995 | 0.000151 | 0.0011 | 0.873276 | 0.125473 | 0 | 0 | 0 | 0 | 0 |
| 996 | 0.00005 | 0.000961 | 0.877297 | 0.121692 | 0 | 0 | 0 | 0 | 0 |
| 997 | 0.00005 | 0.001153 | 0.870555 | 0.128242 | 0 | 0 | 0 | 0 | 0 |
| 998 | 0.0001 | 0.000888 | 0.874963 | 0.12405 | 0 | 0 | 0 | 0 | 0 |
| 999 | 0.00001 | 0.001122 | 0.875085 | 0.123784 | 0 | 0 | 0 | 0 | 0 |
| 1000 | 0.00012 | 0.000961 | 0.873301 | 0.125618 | 0 | 0 | 0 | 0 | 0 |

| Year 6 | Race |  |  |  |  |  |  |  |  |
| --- | --- | --- | --- | --- | --- | --- | --- | --- | --- |
| Trial | 003.0 | 005.0 | 007.0 | 037.1 | 043.0 | 303.0 | 003.2 | 403.0 | 003.4 |
| 1 | 0.00003 | 0.000631 | 0.88387 | 0.115469 | 0 | 0 | 0 | 0 | 0 |
| 2 | 0 | 0.00061 | 0.878622 | 0.120769 | 0 | 0 | 0 | 0 | 0 |
| 3 | 0.00001 | 0.000468 | 0.88307 | 0.116452 | 0 | 0 | 0 | 0 | 0 |
| 4 | 0.00003 | 0.00041 | 0.885641 | 0.113919 | 0 | 0 | 0 | 0 | 0 |
| 5 | 0.00003 | 0.000506 | 0.88078 | 0.118684 | 0 | 0 | 0 | 0 | 0 |
| 6 | 0.00003 | 0.000411 | 0.884758 | 0.114801 | 0 | 0 | 0 | 0 | 0 |
| 7 | 0.00001 | 0.000382 | 0.883089 | 0.116519 | 0 | 0 | 0 | 0 | 0 |
| 8 | 0.00003 | 0.00047 | 0.88495 | 0.11455 | 0 | 0 | 0 | 0 | 0 |
| 9 | 0.00004 | 0.000509 | 0.883363 | 0.116088 | 0 | 0 | 0 | 0 | 0 |
| 10 | 0 | 0.000443 | 0.882834 | 0.116723 | 0 | 0 | 0 | 0 | 0 |
| 11 | 0 | 0.00047 | 0.882497 | 0.117033 | 0 | 0 | 0 | 0 | 0 |
| 12 | 0.00004 | 0.000389 | 0.881519 | 0.118052 | 0 | 0 | 0 | 0 | 0 |
| 13 | 0.00002 | 0.000604 | 0.88153 | 0.117846 | 0 | 0 | 0 | 0 | 0 |
| 14 | 0.00001 | 0.000538 | 0.876986 | 0.122467 | 0 | 0 | 0 | 0 | 0 |
| 15 | 0.00003 | 0.00043 | 0.879853 | 0.119686 | 0 | 0 | 0 | 0 | 0 |
| 16 | 0.00004 | 0.000519 | 0.882638 | 0.116803 | 0 | 0 | 0 | 0 | 0 |
| 17 | 0.00002 | 0.000644 | 0.882663 | 0.116673 | 0 | 0 | 0 | 0 | 0 |
| 18 | 0.00005 | 0.000441 | 0.883537 | 0.115972 | 0 | 0 | 0 | 0 | 0 |
| 19 | 0.00004 | 0.000492 | 0.882413 | 0.117055 | 0 | 0 | 0 | 0 | 0 |
| 20 | 0.00002 | 0.000562 | 0.881116 | 0.118302 | 0 | 0 | 0 | 0 | 0 |
| 21 | 0.00004 | 0.000389 | 0.882862 | 0.11671 | 0 | 0 | 0 | 0 | 0 |
| 22 | 0.00002 | 0.00062 | 0.881718 | 0.117642 | 0 | 0 | 0 | 0 | 0 |
| 23 | 0.00002 | 0.000568 | 0.884224 | 0.115189 | 0 | 0 | 0 | 0 | 0 |
| 24 | 0.00001 | 0.000449 | 0.881989 | 0.117552 | 0 | 0 | 0 | 0 | 0 |
| 25 | 0.00001 | 0.000342 | 0.88457 | 0.115078 | 0 | 0 | 0 | 0 | 0 |
| 26 | 0 | 0.0005 | 0.888624 | 0.110876 | 0 | 0 | 0 | 0 | 0 |
| 27 | 0.00003 | 0.000609 | 0.881914 | 0.117447 | 0 | 0 | 0 | 0 | 0 |
| 28 | 0.00001 | 0.0004 | 0.883209 | 0.116351 | 0 | 0 | 0 | 0 | 0 |
| 29 | 0.00002 | 0.00039 | 0.884196 | 0.115395 | 0 | 0 | 0 | 0 | 0 |
| 30 | 0.00004 | 0.00066 | 0.884199 | 0.115101 | 0 | 0 | 0 | 0 | 0 |
| 31 | 0.00003 | 0.000402 | 0.885886 | 0.113682 | 0 | 0 | 0 | 0 | 0 |
| 32 | 0 | 0.000361 | 0.884444 | 0.115195 | 0 | 0 | 0 | 0 | 0 |
| 33 | 0.00003 | 0.000531 | 0.882776 | 0.116663 | 0 | 0 | 0 | 0 | 0 |
| 34 | 0.00006 | 0.00055 | 0.883401 | 0.115989 | 0 | 0 | 0 | 0 | 0 |
| 35 | 0.00003 | 0.000526 | 0.875289 | 0.124155 | 0 | 0 | 0 | 0 | 0 |
| 36 | 0.00003 | 0.000471 | 0.882689 | 0.116811 | 0 | 0 | 0 | 0 | 0 |
| 37 | 0.00005 | 0.000331 | 0.886297 | 0.113322 | 0 | 0 | 0 | 0 | 0 |
| 38 | 0.00001 | 0.000419 | 0.884876 | 0.114695 | 0 | 0 | 0 | 0 | 0 |
| 39 | 0.00002 | 0.000311 | 0.881531 | 0.118098 | 0 | 0 | 0 | 0 | 0 |
| 40 | 0.00001 | 0.000499 | 0.879841 | 0.119649 | 0 | 0 | 0 | 0 | 0 |
| 41 | 0.00006 | 0.000408 | 0.884276 | 0.115257 | 0 | 0 | 0 | 0 | 0 |
| 42 | 0.00002 | 0.0005 | 0.886058 | 0.113421 | 0 | 0 | 0 | 0 | 0 |
| 43 | 0.00001 | 0.000319 | 0.883388 | 0.116283 | 0 | 0 | 0 | 0 | 0 |
| 44 | 0.00001 | 0.00053 | 0.883253 | 0.116207 | 0 | 0 | 0 | 0 | 0 |
| 45 | 0.00002 | 0.00051 | 0.882201 | 0.11727 | 0 | 0 | 0 | 0 | 0 |
| 46 | 0.00001 | 0.000399 | 0.88037 | 0.119221 | 0 | 0 | 0 | 0 | 0 |
| 47 | 0.00003 | 0.000401 | 0.882314 | 0.117255 | 0 | 0 | 0 | 0 | 0 |
| 48 | 0.00001 | 0.000471 | 0.88274 | 0.116779 | 0 | 0 | 0 | 0 | 0 |
| 49 | 0.00004 | 0.000471 | 0.878421 | 0.121067 | 0 | 0 | 0 | 0 | 0 |
| 50 | 0.00004 | 0.000351 | 0.884247 | 0.115362 | 0 | 0 | 0 | 0 | 0 |
| 51 | 0 | 0.000592 | 0.882387 | 0.117022 | 0 | 0 | 0 | 0 | 0 |
| 52 | 0.00003 | 0.0005 | 0.883669 | 0.115801 | 0 | 0 | 0 | 0 | 0 |
| 53 | 0.00003 | 0.00047 | 0.883656 | 0.115814 | 0 | 0 | 0 | 0 | 0 |
| 54 | 0.00001 | 0.00047 | 0.883357 | 0.116163 | 0 | 0 | 0 | 0 | 0 |
| 55 | 0.00002 | 0.000499 | 0.884057 | 0.115424 | 0 | 0 | 0 | 0 | 0 |
| 56 | 0.00001 | 0.000532 | 0.88143 | 0.118028 | 0 | 0 | 0 | 0 | 0 |
| 57 | 0.00001 | 0.000538 | 0.884758 | 0.114694 | 0 | 0 | 0 | 0 | 0 |
| 58 | 0.00003 | 0.000511 | 0.882564 | 0.116895 | 0 | 0 | 0 | 0 | 0 |
| 59 | 0.00006 | 0.00041 | 0.883361 | 0.116168 | 0 | 0 | 0 | 0 | 0 |
| 60 | 0.00001 | 0.000471 | 0.88412 | 0.115399 | 0 | 0 | 0 | 0 | 0 |
| 61 | 0 | 0.000439 | 0.883806 | 0.115756 | 0 | 0 | 0 | 0 | 0 |
| 62 | 0.00001 | 0.000358 | 0.883708 | 0.115924 | 0 | 0 | 0 | 0 | 0 |
| 63 | 0.00003 | 0.00039 | 0.884224 | 0.115356 | 0 | 0 | 0 | 0 | 0 |
| 64 | 0.00001 | 0.00048 | 0.881067 | 0.118443 | 0 | 0 | 0 | 0 | 0 |
| 65 | 0.00002 | 0.000423 | 0.882652 | 0.116905 | 0 | 0 | 0 | 0 | 0 |
| 66 | 0.00002 | 0.000454 | 0.886025 | 0.113501 | 0 | 0 | 0 | 0 | 0 |
| 67 | 0.00003 | 0.000529 | 0.885625 | 0.113816 | 0 | 0 | 0 | 0 | 0 |
| 68 | 0.00003 | 0.000381 | 0.882395 | 0.117194 | 0 | 0 | 0 | 0 | 0 |
| 69 | 0.00003 | 0.000301 | 0.883099 | 0.11657 | 0 | 0 | 0 | 0 | 0 |
| 70 | 0.00005 | 0.000496 | 0.883759 | 0.115695 | 0 | 0 | 0 | 0 | 0 |
| 71 | 0.00002 | 0.00054 | 0.881732 | 0.117598 | 0 | 0 | 0 | 0 | 0 |
| 72 | 0 | 0.000531 | 0.886529 | 0.11294 | 0 | 0 | 0 | 0 | 0 |
| 73 | 0.00001 | 0.000671 | 0.881697 | 0.117622 | 0 | 0 | 0 | 0 | 0 |
| 74 | 0.00002 | 0.0005 | 0.885491 | 0.11399 | 0 | 0 | 0 | 0 | 0 |
| 75 | 0.00003 | 0.000525 | 0.881575 | 0.117869 | 0 | 0 | 0 | 0 | 0 |
| 76 | 0.00002 | 0.000572 | 0.885672 | 0.113736 | 0 | 0 | 0 | 0 | 0 |
| 77 | 0.00001 | 0.000524 | 0.883921 | 0.115544 | 0 | 0 | 0 | 0 | 0 |
| 78 | 0.00001 | 0.000451 | 0.882775 | 0.116764 | 0 | 0 | 0 | 0 | 0 |
| 79 | 0.00001 | 0.0003 | 0.885587 | 0.114103 | 0 | 0 | 0 | 0 | 0 |
| 80 | 0.00001 | 0.000452 | 0.88248 | 0.117058 | 0 | 0 | 0 | 0 | 0 |
| 81 | 0.00002 | 0.000371 | 0.883004 | 0.116604 | 0 | 0 | 0 | 0 | 0 |
| 82 | 0.00002 | 0.000535 | 0.882114 | 0.117331 | 0 | 0 | 0 | 0 | 0 |
| 83 | 0.00004 | 0.000449 | 0.887526 | 0.111984 | 0 | 0 | 0 | 0 | 0 |
| 84 | 0.00003 | 0.000501 | 0.886087 | 0.113382 | 0 | 0 | 0 | 0 | 0 |
| 85 | 0.00002 | 0.000401 | 0.883038 | 0.11654 | 0 | 0 | 0 | 0 | 0 |
| 86 | 0.00001 | 0.0004 | 0.890384 | 0.109205 | 0 | 0 | 0 | 0 | 0 |
| 87 | 0.00001 | 0.000581 | 0.881777 | 0.117632 | 0 | 0 | 0 | 0 | 0 |
| 88 | 0 | 0.0004 | 0.882885 | 0.116685 | 0 | 0 | 0 | 0 | 0 |
| 89 | 0.00001 | 0.000548 | 0.881616 | 0.117826 | 0 | 0 | 0 | 0 | 0 |
| 90 | 0.00002 | 0.000429 | 0.882761 | 0.11678 | 0 | 0 | 0 | 0 | 0 |
| 91 | 0.00003 | 0.000379 | 0.883695 | 0.115897 | 0 | 0 | 0 | 0 | 0 |
| 92 | 0.00002 | 0.000557 | 0.881097 | 0.118326 | 0 | 0 | 0 | 0 | 0 |
| 93 | 0.00001 | 0.000573 | 0.883729 | 0.115688 | 0 | 0 | 0 | 0 | 0 |
| 94 | 0.00004 | 0.000481 | 0.883626 | 0.115853 | 0 | 0 | 0 | 0 | 0 |
| 95 | 0.00002 | 0.000472 | 0.880224 | 0.119284 | 0 | 0 | 0 | 0 | 0 |
| 96 | 0.00003 | 0.00045 | 0.882242 | 0.117278 | 0 | 0 | 0 | 0 | 0 |
| 97 | 0.00003 | 0.000463 | 0.880332 | 0.119175 | 0 | 0 | 0 | 0 | 0 |
| 98 | 0.00002 | 0.000422 | 0.88161 | 0.117948 | 0 | 0 | 0 | 0 | 0 |
| 99 | 0.00001 | 0.000497 | 0.880368 | 0.119125 | 0 | 0 | 0 | 0 | 0 |
| 100 | 0.00003 | 0.000468 | 0.885309 | 0.114193 | 0 | 0 | 0 | 0 | 0 |
| 101 | 0.00005 | 0.000471 | 0.885381 | 0.114098 | 0 | 0 | 0 | 0 | 0 |
| 102 | 0.00002 | 0.00048 | 0.886358 | 0.113142 | 0 | 0 | 0 | 0 | 0 |
| 103 | 0.00002 | 0.000412 | 0.880315 | 0.119253 | 0 | 0 | 0 | 0 | 0 |
| 104 | 0 | 0.000405 | 0.884264 | 0.115321 | 0 | 0 | 0 | 0 | 0 |
| 105 | 0.00004 | 0.000581 | 0.884546 | 0.114834 | 0 | 0 | 0 | 0 | 0 |
| 106 | 0.00003 | 0.000461 | 0.880591 | 0.118917 | 0 | 0 | 0 | 0 | 0 |
| 107 | 0.00003 | 0.000389 | 0.881898 | 0.117503 | 0 | 0 | 0 | 0 | 0 |
| 108 | 0 | 0.000442 | 0.879901 | 0.119657 | 0 | 0 | 0 | 0 | 0 |
| 109 | 0 | 0.000547 | 0.884829 | 0.114624 | 0 | 0 | 0 | 0 | 0 |
| 110 | 0.00002 | 0.000492 | 0.88637 | 0.113118 | 0 | 0 | 0 | 0 | 0 |
| 111 | 0.00002 | 0.000389 | 0.886876 | 0.112714 | 0 | 0 | 0 | 0 | 0 |
| 112 | 0.00001 | 0.000399 | 0.881948 | 0.117643 | 0 | 0 | 0 | 0 | 0 |
| 113 | 0.00002 | 0.00031 | 0.88444 | 0.11523 | 0 | 0 | 0 | 0 | 0 |
| 114 | 0.00003 | 0.000351 | 0.884738 | 0.11488 | 0 | 0 | 0 | 0 | 0 |
| 115 | 0.00002 | 0.00036 | 0.889648 | 0.109971 | 0 | 0 | 0 | 0 | 0 |
| 116 | 0.00002 | 0.0006 | 0.885841 | 0.113539 | 0 | 0 | 0 | 0 | 0 |
| 117 | 0 | 0.000513 | 0.883014 | 0.116472 | 0 | 0 | 0 | 0 | 0 |
| 118 | 0.00001 | 0.000536 | 0.885091 | 0.114364 | 0 | 0 | 0 | 0 | 0 |
| 119 | 0.00002 | 0.00032 | 0.883955 | 0.115705 | 0 | 0 | 0 | 0 | 0 |
| 120 | 0.00001 | 0.000646 | 0.882141 | 0.117203 | 0 | 0 | 0 | 0 | 0 |
| 121 | 0.00002 | 0.000543 | 0.884768 | 0.114669 | 0 | 0 | 0 | 0 | 0 |
| 122 | 0.00001 | 0.000422 | 0.886584 | 0.112984 | 0 | 0 | 0 | 0 | 0 |
| 123 | 0.00002 | 0.000378 | 0.882939 | 0.116663 | 0 | 0 | 0 | 0 | 0 |
| 124 | 0.00002 | 0.000351 | 0.887671 | 0.111958 | 0 | 0 | 0 | 0 | 0 |
| 125 | 0.00002 | 0.000561 | 0.883178 | 0.116241 | 0 | 0 | 0 | 0 | 0 |
| 126 | 0.00002 | 0.00031 | 0.880713 | 0.118957 | 0 | 0 | 0 | 0 | 0 |
| 127 | 0 | 0.000449 | 0.885265 | 0.114286 | 0 | 0 | 0 | 0 | 0 |
| 128 | 0.00002 | 0.000502 | 0.883437 | 0.11604 | 0 | 0 | 0 | 0 | 0 |
| 129 | 0 | 0.000593 | 0.877054 | 0.122353 | 0 | 0 | 0 | 0 | 0 |
| 130 | 0.00002 | 0.000389 | 0.884939 | 0.114652 | 0 | 0 | 0 | 0 | 0 |
| 131 | 0 | 0.000412 | 0.886436 | 0.113152 | 0 | 0 | 0 | 0 | 0 |
| 132 | 0 | 0.000482 | 0.886709 | 0.112808 | 0 | 0 | 0 | 0 | 0 |
| 133 | 0.00002 | 0.00045 | 0.882136 | 0.117394 | 0 | 0 | 0 | 0 | 0 |
| 134 | 0 | 0.000583 | 0.883969 | 0.115449 | 0 | 0 | 0 | 0 | 0 |
| 135 | 0.00001 | 0.000528 | 0.879276 | 0.120186 | 0 | 0 | 0 | 0 | 0 |
| 136 | 0.00003 | 0.00046 | 0.886889 | 0.112621 | 0 | 0 | 0 | 0 | 0 |
| 137 | 0.00007 | 0.000362 | 0.882502 | 0.117066 | 0 | 0 | 0 | 0 | 0 |
| 138 | 0.00001 | 0.00054 | 0.879682 | 0.119768 | 0 | 0 | 0 | 0 | 0 |
| 139 | 0 | 0.000583 | 0.886227 | 0.113191 | 0 | 0 | 0 | 0 | 0 |
| 140 | 0.00002 | 0.000429 | 0.882051 | 0.1175 | 0 | 0 | 0 | 0 | 0 |
| 141 | 0.00001 | 0.000522 | 0.883995 | 0.115472 | 0 | 0 | 0 | 0 | 0 |
| 142 | 0.00003 | 0.000509 | 0.879399 | 0.120062 | 0 | 0 | 0 | 0 | 0 |
| 143 | 0.00002 | 0.000509 | 0.88087 | 0.1186 | 0 | 0 | 0 | 0 | 0 |
| 144 | 0.00001 | 0.000639 | 0.882713 | 0.116638 | 0 | 0 | 0 | 0 | 0 |
| 145 | 0.00001 | 0.00043 | 0.884262 | 0.115298 | 0 | 0 | 0 | 0 | 0 |
| 146 | 0.00002 | 0.00048 | 0.887949 | 0.111552 | 0 | 0 | 0 | 0 | 0 |
| 147 | 0.00002 | 0.000442 | 0.88143 | 0.118108 | 0 | 0 | 0 | 0 | 0 |
| 148 | 0.00005 | 0.000401 | 0.882817 | 0.116733 | 0 | 0 | 0 | 0 | 0 |
| 149 | 0.00004 | 0.000321 | 0.882089 | 0.117549 | 0 | 0 | 0 | 0 | 0 |
| 150 | 0.00004 | 0.000549 | 0.882532 | 0.116879 | 0 | 0 | 0 | 0 | 0 |
| 151 | 0.00002 | 0.000528 | 0.884144 | 0.115308 | 0 | 0 | 0 | 0 | 0 |
| 152 | 0.00001 | 0.000467 | 0.879581 | 0.119942 | 0 | 0 | 0 | 0 | 0 |
| 153 | 0.00001 | 0.00032 | 0.884779 | 0.114891 | 0 | 0 | 0 | 0 | 0 |
| 154 | 0.00003 | 0.000509 | 0.880187 | 0.119273 | 0 | 0 | 0 | 0 | 0 |
| 155 | 0.00007 | 0.000431 | 0.879227 | 0.120272 | 0 | 0 | 0 | 0 | 0 |
| 156 | 0.00002 | 0.000567 | 0.880205 | 0.119208 | 0 | 0 | 0 | 0 | 0 |
| 157 | 0.00003 | 0.000522 | 0.882345 | 0.117103 | 0 | 0 | 0 | 0 | 0 |
| 158 | 0.00002 | 0.00038 | 0.88392 | 0.115681 | 0 | 0 | 0 | 0 | 0 |
| 159 | 0.00004 | 0.000357 | 0.879898 | 0.119706 | 0 | 0 | 0 | 0 | 0 |
| 160 | 0.00002 | 0.000627 | 0.886922 | 0.112431 | 0 | 0 | 0 | 0 | 0 |
| 161 | 0.00002 | 0.000451 | 0.882625 | 0.116904 | 0 | 0 | 0 | 0 | 0 |
| 162 | 0.00001 | 0.000601 | 0.883522 | 0.115868 | 0 | 0 | 0 | 0 | 0 |
| 163 | 0.00001 | 0.000428 | 0.883689 | 0.115873 | 0 | 0 | 0 | 0 | 0 |
| 164 | 0 | 0.00051 | 0.881035 | 0.118455 | 0 | 0 | 0 | 0 | 0 |
| 165 | 0.00001 | 0.000544 | 0.886611 | 0.112835 | 0 | 0 | 0 | 0 | 0 |
| 166 | 0.00002 | 0.000497 | 0.881978 | 0.117504 | 0 | 0 | 0 | 0 | 0 |
| 167 | 0.00004 | 0.000483 | 0.88311 | 0.116367 | 0 | 0 | 0 | 0 | 0 |
| 168 | 0.00006 | 0.000589 | 0.882393 | 0.116958 | 0 | 0 | 0 | 0 | 0 |
| 169 | 0.00002 | 0.000559 | 0.880965 | 0.118456 | 0 | 0 | 0 | 0 | 0 |
| 170 | 0.00001 | 0.000489 | 0.882793 | 0.116708 | 0 | 0 | 0 | 0 | 0 |
| 171 | 0.00002 | 0.000508 | 0.883689 | 0.115783 | 0 | 0 | 0 | 0 | 0 |
| 172 | 0 | 0.00043 | 0.883522 | 0.116048 | 0 | 0 | 0 | 0 | 0 |
| 173 | 0.00002 | 0.000611 | 0.884206 | 0.115163 | 0 | 0 | 0 | 0 | 0 |
| 174 | 0.00001 | 0.0006 | 0.880108 | 0.119283 | 0 | 0 | 0 | 0 | 0 |
| 175 | 0.00005 | 0.00047 | 0.882139 | 0.11734 | 0 | 0 | 0 | 0 | 0 |
| 176 | 0.00003 | 0.000368 | 0.889573 | 0.110019 | 0 | 0 | 0 | 0 | 0 |
| 177 | 0.00003 | 0.000422 | 0.882028 | 0.117521 | 0 | 0 | 0 | 0 | 0 |
| 178 | 0.00001 | 0.000361 | 0.880561 | 0.119067 | 0 | 0 | 0 | 0 | 0 |
| 179 | 0.00002 | 0.000543 | 0.880807 | 0.11863 | 0 | 0 | 0 | 0 | 0 |
| 180 | 0.00001 | 0.000532 | 0.884912 | 0.114546 | 0 | 0 | 0 | 0 | 0 |
| 181 | 0.00002 | 0.000562 | 0.884178 | 0.11524 | 0 | 0 | 0 | 0 | 0 |
| 182 | 0.00001 | 0.00059 | 0.884594 | 0.114806 | 0 | 0 | 0 | 0 | 0 |
| 183 | 0.00004 | 0.000349 | 0.883154 | 0.116457 | 0 | 0 | 0 | 0 | 0 |
| 184 | 0.00003 | 0.000369 | 0.886882 | 0.112719 | 0 | 0 | 0 | 0 | 0 |
| 185 | 0.00002 | 0.0005 | 0.884896 | 0.114585 | 0 | 0 | 0 | 0 | 0 |
| 186 | 0.00001 | 0.00041 | 0.88244 | 0.11714 | 0 | 0 | 0 | 0 | 0 |
| 187 | 0 | 0.00057 | 0.890307 | 0.109123 | 0 | 0 | 0 | 0 | 0 |
| 188 | 0 | 0.000374 | 0.880584 | 0.119041 | 0 | 0 | 0 | 0 | 0 |
| 189 | 0.00003 | 0.00051 | 0.882455 | 0.117005 | 0 | 0 | 0 | 0 | 0 |
| 190 | 0.00001 | 0.00028 | 0.884453 | 0.115257 | 0 | 0 | 0 | 0 | 0 |
| 191 | 0.00003 | 0.000712 | 0.883006 | 0.116252 | 0 | 0 | 0 | 0 | 0 |
| 192 | 0 | 0.000529 | 0.882538 | 0.116934 | 0 | 0 | 0 | 0 | 0 |
| 193 | 0.00002 | 0.000459 | 0.881191 | 0.11833 | 0 | 0 | 0 | 0 | 0 |
| 194 | 0.00002 | 0.000369 | 0.879892 | 0.11972 | 0 | 0 | 0 | 0 | 0 |
| 195 | 0 | 0.00035 | 0.882769 | 0.116881 | 0 | 0 | 0 | 0 | 0 |
| 196 | 0 | 0.00052 | 0.880007 | 0.119473 | 0 | 0 | 0 | 0 | 0 |
| 197 | 0.00004 | 0.00043 | 0.886715 | 0.112815 | 0 | 0 | 0 | 0 | 0 |
| 198 | 0.00002 | 0.000609 | 0.881753 | 0.117528 | 0 | 0 | 0 | 0 | 0 |
| 199 | 0.00001 | 0.000661 | 0.884738 | 0.114591 | 0 | 0 | 0 | 0 | 0 |
| 200 | 0.00002 | 0.000591 | 0.881109 | 0.118279 | 0 | 0 | 0 | 0 | 0 |
| 201 | 0.00001 | 0.000631 | 0.883019 | 0.11634 | 0 | 0 | 0 | 0 | 0 |
| 202 | 0 | 0.00061 | 0.884299 | 0.115091 | 0 | 0 | 0 | 0 | 0 |
| 203 | 0 | 0.00042 | 0.885949 | 0.113632 | 0 | 0 | 0 | 0 | 0 |
| 204 | 0.00002 | 0.000487 | 0.883782 | 0.11571 | 0 | 0 | 0 | 0 | 0 |
| 205 | 0.00004 | 0.000332 | 0.878152 | 0.121476 | 0 | 0 | 0 | 0 | 0 |
| 206 | 0.00003 | 0.000421 | 0.879155 | 0.120394 | 0 | 0 | 0 | 0 | 0 |
| 207 | 0.00002 | 0.000473 | 0.886933 | 0.112574 | 0 | 0 | 0 | 0 | 0 |
| 208 | 0.00004 | 0.000518 | 0.886807 | 0.112636 | 0 | 0 | 0 | 0 | 0 |
| 209 | 0.00001 | 0.000451 | 0.88192 | 0.117618 | 0 | 0 | 0 | 0 | 0 |
| 210 | 0.00002 | 0.00051 | 0.883407 | 0.116063 | 0 | 0 | 0 | 0 | 0 |
| 211 | 0.00001 | 0.000468 | 0.882862 | 0.11666 | 0 | 0 | 0 | 0 | 0 |
| 212 | 0.00002 | 0.000502 | 0.881474 | 0.118004 | 0 | 0 | 0 | 0 | 0 |
| 213 | 0 | 0.000357 | 0.886141 | 0.113502 | 0 | 0 | 0 | 0 | 0 |
| 214 | 0.00003 | 0.00033 | 0.884795 | 0.114845 | 0 | 0 | 0 | 0 | 0 |
| 215 | 0.00002 | 0.000417 | 0.883379 | 0.116184 | 0 | 0 | 0 | 0 | 0 |
| 216 | 0.00005 | 0.000571 | 0.885356 | 0.114023 | 0 | 0 | 0 | 0 | 0 |
| 217 | 0.00004 | 0.000489 | 0.883444 | 0.116027 | 0 | 0 | 0 | 0 | 0 |
| 218 | 0.00001 | 0.000519 | 0.885373 | 0.114098 | 0 | 0 | 0 | 0 | 0 |
| 219 | 0 | 0.000549 | 0.889359 | 0.110092 | 0 | 0 | 0 | 0 | 0 |
| 220 | 0.00001 | 0.000553 | 0.882251 | 0.117186 | 0 | 0 | 0 | 0 | 0 |
| 221 | 0.00002 | 0.000514 | 0.883113 | 0.116353 | 0 | 0 | 0 | 0 | 0 |
| 222 | 0.00001 | 0.00038 | 0.883891 | 0.115719 | 0 | 0 | 0 | 0 | 0 |
| 223 | 0.00003 | 0.000439 | 0.883191 | 0.11634 | 0 | 0 | 0 | 0 | 0 |
| 224 | 0 | 0.000493 | 0.880267 | 0.119239 | 0 | 0 | 0 | 0 | 0 |
| 225 | 0.00003 | 0.00052 | 0.887339 | 0.112111 | 0 | 0 | 0 | 0 | 0 |
| 226 | 0 | 0.000349 | 0.886427 | 0.113224 | 0 | 0 | 0 | 0 | 0 |
| 227 | 0.00001 | 0.00042 | 0.882613 | 0.116957 | 0 | 0 | 0 | 0 | 0 |
| 228 | 0.00003 | 0.000542 | 0.885059 | 0.114368 | 0 | 0 | 0 | 0 | 0 |
| 229 | 0.00003 | 0.000382 | 0.885345 | 0.114243 | 0 | 0 | 0 | 0 | 0 |
| 230 | 0.00001 | 0.000428 | 0.880128 | 0.119434 | 0 | 0 | 0 | 0 | 0 |
| 231 | 0.00001 | 0.000744 | 0.884435 | 0.114811 | 0 | 0 | 0 | 0 | 0 |
| 232 | 0.00004 | 0.000494 | 0.88316 | 0.116305 | 0 | 0 | 0 | 0 | 0 |
| 233 | 0.00003 | 0.000545 | 0.885023 | 0.114403 | 0 | 0 | 0 | 0 | 0 |
| 234 | 0.00002 | 0.000402 | 0.882911 | 0.116667 | 0 | 0 | 0 | 0 | 0 |
| 235 | 0.00002 | 0.000491 | 0.887604 | 0.111844 | 0 | 0 | 0 | 0 | 0 |
| 236 | 0.00006 | 0.000438 | 0.881449 | 0.118053 | 0 | 0 | 0 | 0 | 0 |
| 237 | 0.00001 | 0.000338 | 0.883291 | 0.116361 | 0 | 0 | 0 | 0 | 0 |
| 238 | 0 | 0.000709 | 0.877591 | 0.1217 | 0 | 0 | 0 | 0 | 0 |
| 239 | 0.00003 | 0.000461 | 0.882233 | 0.117276 | 0 | 0 | 0 | 0 | 0 |
| 240 | 0.00003 | 0.00037 | 0.88455 | 0.11505 | 0 | 0 | 0 | 0 | 0 |
| 241 | 0.00002 | 0.00042 | 0.88676 | 0.1128 | 0 | 0 | 0 | 0 | 0 |
| 242 | 0.00002 | 0.000416 | 0.882254 | 0.1173 | 0 | 0 | 0 | 0 | 0 |
| 243 | 0.00002 | 0.00035 | 0.887279 | 0.112352 | 0 | 0 | 0 | 0 | 0 |
| 244 | 0 | 0.00037 | 0.88266 | 0.11697 | 0 | 0 | 0 | 0 | 0 |
| 245 | 0.00001 | 0.000741 | 0.882866 | 0.116314 | 0 | 0 | 0 | 0 | 0 |
| 246 | 0.00003 | 0.00048 | 0.881484 | 0.118006 | 0 | 0 | 0 | 0 | 0 |
| 247 | 0.00002 | 0.000614 | 0.878827 | 0.120539 | 0 | 0 | 0 | 0 | 0 |
| 248 | 0.00002 | 0.00064 | 0.884655 | 0.114686 | 0 | 0 | 0 | 0 | 0 |
| 249 | 0.00002 | 0.000509 | 0.884129 | 0.115342 | 0 | 0 | 0 | 0 | 0 |
| 250 | 0.00003 | 0.000692 | 0.879706 | 0.119572 | 0 | 0 | 0 | 0 | 0 |
| 251 | 0.00001 | 0.000547 | 0.883167 | 0.116276 | 0 | 0 | 0 | 0 | 0 |
| 252 | 0.00002 | 0.000291 | 0.885454 | 0.114236 | 0 | 0 | 0 | 0 | 0 |
| 253 | 0.00002 | 0.000451 | 0.882288 | 0.117241 | 0 | 0 | 0 | 0 | 0 |
| 254 | 0.00001 | 0.00058 | 0.881646 | 0.117765 | 0 | 0 | 0 | 0 | 0 |
| 255 | 0.00003 | 0.000498 | 0.883801 | 0.115672 | 0 | 0 | 0 | 0 | 0 |
| 256 | 0.00002 | 0.000509 | 0.879683 | 0.119788 | 0 | 0 | 0 | 0 | 0 |
| 257 | 0 | 0.000471 | 0.882966 | 0.116553 | 0 | 0 | 0 | 0 | 0 |
| 258 | 0.00002 | 0.00047 | 0.879769 | 0.119741 | 0 | 0 | 0 | 0 | 0 |
| 259 | 0 | 0.000458 | 0.882628 | 0.116914 | 0 | 0 | 0 | 0 | 0 |
| 260 | 0.00001 | 0.000427 | 0.884965 | 0.114598 | 0 | 0 | 0 | 0 | 0 |
| 261 | 0.00001 | 0.000521 | 0.886385 | 0.113084 | 0 | 0 | 0 | 0 | 0 |
| 262 | 0 | 0.00046 | 0.882224 | 0.117317 | 0 | 0 | 0 | 0 | 0 |
| 263 | 0.00002 | 0.000529 | 0.88321 | 0.116241 | 0 | 0 | 0 | 0 | 0 |
| 264 | 0.00001 | 0.000328 | 0.880219 | 0.119443 | 0 | 0 | 0 | 0 | 0 |
| 265 | 0 | 0.000458 | 0.881473 | 0.118069 | 0 | 0 | 0 | 0 | 0 |
| 266 | 0.00001 | 0.000569 | 0.884606 | 0.114815 | 0 | 0 | 0 | 0 | 0 |
| 267 | 0.00001 | 0.00035 | 0.882989 | 0.116651 | 0 | 0 | 0 | 0 | 0 |
| 268 | 0.00003 | 0.000529 | 0.883828 | 0.115613 | 0 | 0 | 0 | 0 | 0 |
| 269 | 0.00003 | 0.000418 | 0.884879 | 0.114673 | 0 | 0 | 0 | 0 | 0 |
| 270 | 0.00006 | 0.000419 | 0.88261 | 0.116911 | 0 | 0 | 0 | 0 | 0 |
| 271 | 0.00002 | 0.000488 | 0.883453 | 0.116019 | 0 | 0 | 0 | 0 | 0 |
| 272 | 0.00004 | 0.000464 | 0.881293 | 0.118203 | 0 | 0 | 0 | 0 | 0 |
| 273 | 0.00002 | 0.000409 | 0.885399 | 0.114172 | 0 | 0 | 0 | 0 | 0 |
| 274 | 0 | 0.000566 | 0.887664 | 0.111769 | 0 | 0 | 0 | 0 | 0 |
| 275 | 0.00005 | 0.000551 | 0.883742 | 0.115658 | 0 | 0 | 0 | 0 | 0 |
| 276 | 0.00004 | 0.000331 | 0.883826 | 0.115802 | 0 | 0 | 0 | 0 | 0 |
| 277 | 0.00002 | 0.00067 | 0.879686 | 0.119624 | 0 | 0 | 0 | 0 | 0 |
| 278 | 0.00001 | 0.000429 | 0.882256 | 0.117305 | 0 | 0 | 0 | 0 | 0 |
| 279 | 0.00003 | 0.000483 | 0.881756 | 0.117731 | 0 | 0 | 0 | 0 | 0 |
| 280 | 0.00004 | 0.00047 | 0.881792 | 0.117698 | 0 | 0 | 0 | 0 | 0 |
| 281 | 0.00002 | 0.000582 | 0.882294 | 0.117103 | 0 | 0 | 0 | 0 | 0 |
| 282 | 0.00001 | 0.00052 | 0.883356 | 0.116115 | 0 | 0 | 0 | 0 | 0 |
| 283 | 0.00001 | 0.000461 | 0.885306 | 0.114222 | 0 | 0 | 0 | 0 | 0 |
| 284 | 0.00003 | 0.000477 | 0.88589 | 0.113603 | 0 | 0 | 0 | 0 | 0 |
| 285 | 0.00002 | 0.00041 | 0.879846 | 0.119724 | 0 | 0 | 0 | 0 | 0 |
| 286 | 0.00002 | 0.00038 | 0.887059 | 0.112541 | 0 | 0 | 0 | 0 | 0 |
| 287 | 0.00005 | 0.000509 | 0.884236 | 0.115205 | 0 | 0 | 0 | 0 | 0 |
| 288 | 0.00002 | 0.000251 | 0.881666 | 0.118063 | 0 | 0 | 0 | 0 | 0 |
| 289 | 0 | 0.00052 | 0.885293 | 0.114187 | 0 | 0 | 0 | 0 | 0 |
| 290 | 0.00001 | 0.000618 | 0.879881 | 0.119491 | 0 | 0 | 0 | 0 | 0 |
| 291 | 0.00001 | 0.00039 | 0.882592 | 0.117009 | 0 | 0 | 0 | 0 | 0 |
| 292 | 0.00003 | 0.000389 | 0.883109 | 0.116472 | 0 | 0 | 0 | 0 | 0 |
| 293 | 0.00005 | 0.000612 | 0.876957 | 0.12238 | 0 | 0 | 0 | 0 | 0 |
| 294 | 0.00003 | 0.00064 | 0.880256 | 0.119075 | 0 | 0 | 0 | 0 | 0 |
| 295 | 0.00003 | 0.000381 | 0.884045 | 0.115544 | 0 | 0 | 0 | 0 | 0 |
| 296 | 0.00002 | 0.000463 | 0.881834 | 0.117683 | 0 | 0 | 0 | 0 | 0 |
| 297 | 0.00001 | 0.000489 | 0.88081 | 0.118691 | 0 | 0 | 0 | 0 | 0 |
| 298 | 0.00003 | 0.000476 | 0.882803 | 0.116691 | 0 | 0 | 0 | 0 | 0 |
| 299 | 0.00001 | 0.000507 | 0.87723 | 0.122253 | 0 | 0 | 0 | 0 | 0 |
| 300 | 0.00001 | 0.000512 | 0.881182 | 0.118296 | 0 | 0 | 0 | 0 | 0 |
| 301 | 0.00003 | 0.000588 | 0.883333 | 0.11605 | 0 | 0 | 0 | 0 | 0 |
| 302 | 0.00003 | 0.000419 | 0.882275 | 0.117275 | 0 | 0 | 0 | 0 | 0 |
| 303 | 0.00002 | 0.00049 | 0.882227 | 0.117263 | 0 | 0 | 0 | 0 | 0 |
| 304 | 0.00001 | 0.000477 | 0.880791 | 0.118722 | 0 | 0 | 0 | 0 | 0 |
| 305 | 0.00004 | 0.000422 | 0.885916 | 0.113622 | 0 | 0 | 0 | 0 | 0 |
| 306 | 0.00001 | 0.000617 | 0.884722 | 0.114651 | 0 | 0 | 0 | 0 | 0 |
| 307 | 0.00001 | 0.000491 | 0.88243 | 0.117069 | 0 | 0 | 0 | 0 | 0 |
| 308 | 0.00002 | 0.000521 | 0.886027 | 0.113432 | 0 | 0 | 0 | 0 | 0 |
| 309 | 0.00002 | 0.000456 | 0.885053 | 0.114471 | 0 | 0 | 0 | 0 | 0 |
| 310 | 0.00002 | 0.000421 | 0.883841 | 0.115718 | 0 | 0 | 0 | 0 | 0 |
| 311 | 0.00005 | 0.000491 | 0.882766 | 0.116692 | 0 | 0 | 0 | 0 | 0 |
| 312 | 0 | 0.00059 | 0.879415 | 0.119995 | 0 | 0 | 0 | 0 | 0 |
| 313 | 0.00002 | 0.00058 | 0.883289 | 0.11611 | 0 | 0 | 0 | 0 | 0 |
| 314 | 0.00001 | 0.000259 | 0.8826 | 0.117131 | 0 | 0 | 0 | 0 | 0 |
| 315 | 0 | 0.000529 | 0.879522 | 0.119949 | 0 | 0 | 0 | 0 | 0 |
| 316 | 0 | 0.000398 | 0.883294 | 0.116308 | 0 | 0 | 0 | 0 | 0 |
| 317 | 0 | 0.000479 | 0.883324 | 0.116197 | 0 | 0 | 0 | 0 | 0 |
| 318 | 0.00002 | 0.00039 | 0.877313 | 0.122217 | 0 | 0 | 0 | 0 | 0 |
| 319 | 0.00003 | 0.000429 | 0.880059 | 0.119482 | 0 | 0 | 0 | 0 | 0 |
| 320 | 0.00002 | 0.000531 | 0.883923 | 0.115526 | 0 | 0 | 0 | 0 | 0 |
| 321 | 0.00003 | 0.000538 | 0.880083 | 0.119349 | 0 | 0 | 0 | 0 | 0 |
| 322 | 0 | 0.000511 | 0.884421 | 0.115068 | 0 | 0 | 0 | 0 | 0 |
| 323 | 0.00004 | 0.000407 | 0.882365 | 0.117189 | 0 | 0 | 0 | 0 | 0 |
| 324 | 0 | 0.000426 | 0.884504 | 0.11507 | 0 | 0 | 0 | 0 | 0 |
| 325 | 0.00002 | 0.000499 | 0.884266 | 0.115215 | 0 | 0 | 0 | 0 | 0 |
| 326 | 0 | 0.000321 | 0.884522 | 0.115157 | 0 | 0 | 0 | 0 | 0 |
| 327 | 0.00004 | 0.000371 | 0.883758 | 0.115831 | 0 | 0 | 0 | 0 | 0 |
| 328 | 0.00001 | 0.000522 | 0.886051 | 0.113417 | 0 | 0 | 0 | 0 | 0 |
| 329 | 0.00001 | 0.000424 | 0.881608 | 0.117957 | 0 | 0 | 0 | 0 | 0 |
| 330 | 0 | 0.000442 | 0.882645 | 0.116913 | 0 | 0 | 0 | 0 | 0 |
| 331 | 0.00002 | 0.00043 | 0.882312 | 0.117239 | 0 | 0 | 0 | 0 | 0 |
| 332 | 0.00003 | 0.00046 | 0.881212 | 0.118298 | 0 | 0 | 0 | 0 | 0 |
| 333 | 0 | 0.000519 | 0.880353 | 0.119127 | 0 | 0 | 0 | 0 | 0 |
| 334 | 0.00002 | 0.000489 | 0.884212 | 0.115279 | 0 | 0 | 0 | 0 | 0 |
| 335 | 0.00003 | 0.00042 | 0.883127 | 0.116423 | 0 | 0 | 0 | 0 | 0 |
| 336 | 0.00001 | 0.000441 | 0.880114 | 0.119435 | 0 | 0 | 0 | 0 | 0 |
| 337 | 0.00001 | 0.000471 | 0.883941 | 0.115577 | 0 | 0 | 0 | 0 | 0 |
| 338 | 0.00003 | 0.000441 | 0.887796 | 0.111733 | 0 | 0 | 0 | 0 | 0 |
| 339 | 0.00001 | 0.000489 | 0.883688 | 0.115813 | 0 | 0 | 0 | 0 | 0 |
| 340 | 0.00003 | 0.000311 | 0.879517 | 0.120142 | 0 | 0 | 0 | 0 | 0 |
| 341 | 0.00001 | 0.000594 | 0.879791 | 0.119605 | 0 | 0 | 0 | 0 | 0 |
| 342 | 0.00002 | 0.000461 | 0.878582 | 0.120937 | 0 | 0 | 0 | 0 | 0 |
| 343 | 0 | 0.000383 | 0.881602 | 0.118016 | 0 | 0 | 0 | 0 | 0 |
| 344 | 0 | 0.000378 | 0.883133 | 0.116489 | 0 | 0 | 0 | 0 | 0 |
| 345 | 0 | 0.000601 | 0.884927 | 0.114472 | 0 | 0 | 0 | 0 | 0 |
| 346 | 0.00005 | 0.000508 | 0.887117 | 0.112325 | 0 | 0 | 0 | 0 | 0 |
| 347 | 0 | 0.000382 | 0.883384 | 0.116234 | 0 | 0 | 0 | 0 | 0 |
| 348 | 0.00001 | 0.000499 | 0.88433 | 0.115162 | 0 | 0 | 0 | 0 | 0 |
| 349 | 0 | 0.00055 | 0.882549 | 0.116901 | 0 | 0 | 0 | 0 | 0 |
| 350 | 0.00002 | 0.000439 | 0.883045 | 0.116496 | 0 | 0 | 0 | 0 | 0 |
| 351 | 0.00001 | 0.000578 | 0.882022 | 0.11739 | 0 | 0 | 0 | 0 | 0 |
| 352 | 0.00001 | 0.000441 | 0.884806 | 0.114743 | 0 | 0 | 0 | 0 | 0 |
| 353 | 0.00001 | 0.000582 | 0.8855 | 0.113908 | 0 | 0 | 0 | 0 | 0 |
| 354 | 0.00002 | 0.00042 | 0.881272 | 0.118287 | 0 | 0 | 0 | 0 | 0 |
| 355 | 0.00001 | 0.000441 | 0.887061 | 0.112488 | 0 | 0 | 0 | 0 | 0 |
| 356 | 0.00003 | 0.000368 | 0.886143 | 0.113459 | 0 | 0 | 0 | 0 | 0 |
| 357 | 0.00002 | 0.000578 | 0.878105 | 0.121297 | 0 | 0 | 0 | 0 | 0 |
| 358 | 0.00001 | 0.000437 | 0.884924 | 0.114629 | 0 | 0 | 0 | 0 | 0 |
| 359 | 0.00002 | 0.000491 | 0.882153 | 0.117336 | 0 | 0 | 0 | 0 | 0 |
| 360 | 0.00001 | 0.000379 | 0.883245 | 0.116365 | 0 | 0 | 0 | 0 | 0 |
| 361 | 0.00004 | 0.00036 | 0.881386 | 0.118215 | 0 | 0 | 0 | 0 | 0 |
| 362 | 0.00004 | 0.000583 | 0.883886 | 0.115491 | 0 | 0 | 0 | 0 | 0 |
| 363 | 0.00004 | 0.000458 | 0.882923 | 0.116579 | 0 | 0 | 0 | 0 | 0 |
| 364 | 0.00001 | 0.000542 | 0.886264 | 0.113184 | 0 | 0 | 0 | 0 | 0 |
| 365 | 0.00002 | 0.000541 | 0.883711 | 0.115728 | 0 | 0 | 0 | 0 | 0 |
| 366 | 0.00003 | 0.000311 | 0.88387 | 0.115789 | 0 | 0 | 0 | 0 | 0 |
| 367 | 0.00004 | 0.000471 | 0.88117 | 0.11832 | 0 | 0 | 0 | 0 | 0 |
| 368 | 0.00002 | 0.000548 | 0.884044 | 0.115388 | 0 | 0 | 0 | 0 | 0 |
| 369 | 0.00001 | 0.00038 | 0.881972 | 0.117639 | 0 | 0 | 0 | 0 | 0 |
| 370 | 0 | 0.0004 | 0.88225 | 0.11735 | 0 | 0 | 0 | 0 | 0 |
| 371 | 0 | 0.000598 | 0.882603 | 0.116719 | 0 | 0 | 0 | 0 | 0 |
| 372 | 0 | 0.000528 | 0.878538 | 0.120933 | 0 | 0 | 0 | 0 | 0 |
| 373 | 0.00006 | 0.00037 | 0.88129 | 0.118279 | 0 | 0 | 0 | 0 | 0 |
| 374 | 0.00003 | 0.000339 | 0.885323 | 0.114308 | 0 | 0 | 0 | 0 | 0 |
| 375 | 0.00003 | 0.00044 | 0.880897 | 0.118633 | 0 | 0 | 0 | 0 | 0 |
| 376 | 0.00002 | 0.00056 | 0.882564 | 0.116855 | 0 | 0 | 0 | 0 | 0 |
| 377 | 0.00001 | 0.000491 | 0.881129 | 0.11837 | 0 | 0 | 0 | 0 | 0 |
| 378 | 0.00001 | 0.000507 | 0.883957 | 0.115526 | 0 | 0 | 0 | 0 | 0 |
| 379 | 0.00003 | 0.000408 | 0.886357 | 0.113205 | 0 | 0 | 0 | 0 | 0 |
| 380 | 0.00002 | 0.00039 | 0.881267 | 0.118323 | 0 | 0 | 0 | 0 | 0 |
| 381 | 0.00001 | 0.000543 | 0.882178 | 0.117269 | 0 | 0 | 0 | 0 | 0 |
| 382 | 0.00003 | 0.00054 | 0.883156 | 0.116274 | 0 | 0 | 0 | 0 | 0 |
| 383 | 0 | 0.000518 | 0.884364 | 0.115119 | 0 | 0 | 0 | 0 | 0 |
| 384 | 0.00003 | 0.000481 | 0.878388 | 0.121101 | 0 | 0 | 0 | 0 | 0 |
| 385 | 0.00002 | 0.000469 | 0.885923 | 0.113588 | 0 | 0 | 0 | 0 | 0 |
| 386 | 0 | 0.000432 | 0.882222 | 0.117346 | 0 | 0 | 0 | 0 | 0 |
| 387 | 0.00003 | 0.000431 | 0.884411 | 0.115128 | 0 | 0 | 0 | 0 | 0 |
| 388 | 0.00001 | 0.00048 | 0.885395 | 0.114115 | 0 | 0 | 0 | 0 | 0 |
| 389 | 0.00002 | 0.000411 | 0.885973 | 0.113596 | 0 | 0 | 0 | 0 | 0 |
| 390 | 0.00004 | 0.000629 | 0.881919 | 0.117412 | 0 | 0 | 0 | 0 | 0 |
| 391 | 0 | 0.000451 | 0.875832 | 0.123717 | 0 | 0 | 0 | 0 | 0 |
| 392 | 0.00002 | 0.000491 | 0.885337 | 0.114152 | 0 | 0 | 0 | 0 | 0 |
| 393 | 0 | 0.000534 | 0.8821 | 0.117366 | 0 | 0 | 0 | 0 | 0 |
| 394 | 0 | 0.000411 | 0.885635 | 0.113954 | 0 | 0 | 0 | 0 | 0 |
| 395 | 0.00002 | 0.000459 | 0.885651 | 0.11387 | 0 | 0 | 0 | 0 | 0 |
| 396 | 0 | 0.000509 | 0.88548 | 0.114011 | 0 | 0 | 0 | 0 | 0 |
| 397 | 0.00004 | 0.000538 | 0.8847 | 0.114722 | 0 | 0 | 0 | 0 | 0 |
| 398 | 0.00001 | 0.000586 | 0.883859 | 0.115545 | 0 | 0 | 0 | 0 | 0 |
| 399 | 0.00003 | 0.000419 | 0.882717 | 0.116834 | 0 | 0 | 0 | 0 | 0 |
| 400 | 0.00001 | 0.000441 | 0.885941 | 0.113608 | 0 | 0 | 0 | 0 | 0 |
| 401 | 0.00004 | 0.000619 | 0.883555 | 0.115786 | 0 | 0 | 0 | 0 | 0 |
| 402 | 0.00003 | 0.000503 | 0.881999 | 0.117468 | 0 | 0 | 0 | 0 | 0 |
| 403 | 0.00001 | 0.00043 | 0.88481 | 0.11475 | 0 | 0 | 0 | 0 | 0 |
| 404 | 0.00001 | 0.000619 | 0.882771 | 0.116601 | 0 | 0 | 0 | 0 | 0 |
| 405 | 0.00002 | 0.000387 | 0.88796 | 0.111633 | 0 | 0 | 0 | 0 | 0 |
| 406 | 0.00004 | 0.000578 | 0.88327 | 0.116112 | 0 | 0 | 0 | 0 | 0 |
| 407 | 0.00003 | 0.000764 | 0.884813 | 0.114392 | 0 | 0 | 0 | 0 | 0 |
| 408 | 0.00002 | 0.000455 | 0.8816 | 0.117926 | 0 | 0 | 0 | 0 | 0 |
| 409 | 0.00004 | 0.00059 | 0.880555 | 0.118815 | 0 | 0 | 0 | 0 | 0 |
| 410 | 0.00002 | 0.000423 | 0.882821 | 0.116736 | 0 | 0 | 0 | 0 | 0 |
| 411 | 0 | 0.00037 | 0.885494 | 0.114136 | 0 | 0 | 0 | 0 | 0 |
| 412 | 0.00002 | 0.000529 | 0.881615 | 0.117837 | 0 | 0 | 0 | 0 | 0 |
| 413 | 0 | 0.0005 | 0.8816 | 0.1179 | 0 | 0 | 0 | 0 | 0 |
| 414 | 0.00004 | 0.000579 | 0.88481 | 0.114572 | 0 | 0 | 0 | 0 | 0 |
| 415 | 0.00003 | 0.00051 | 0.883488 | 0.115971 | 0 | 0 | 0 | 0 | 0 |
| 416 | 0.00001 | 0.000609 | 0.884772 | 0.11457 | 0 | 0 | 0 | 0 | 0 |
| 417 | 0 | 0.000391 | 0.883988 | 0.115621 | 0 | 0 | 0 | 0 | 0 |
| 418 | 0.00002 | 0.000519 | 0.887658 | 0.111804 | 0 | 0 | 0 | 0 | 0 |
| 419 | 0.00002 | 0.000378 | 0.881771 | 0.117831 | 0 | 0 | 0 | 0 | 0 |
| 420 | 0.00002 | 0.000498 | 0.887111 | 0.11237 | 0 | 0 | 0 | 0 | 0 |
| 421 | 0.00002 | 0.00061 | 0.881858 | 0.117511 | 0 | 0 | 0 | 0 | 0 |
| 422 | 0 | 0.000622 | 0.884202 | 0.115176 | 0 | 0 | 0 | 0 | 0 |
| 423 | 0.00002 | 0.000474 | 0.884545 | 0.11496 | 0 | 0 | 0 | 0 | 0 |
| 424 | 0.00003 | 0.0004 | 0.887404 | 0.112157 | 0 | 0 | 0 | 0 | 0 |
| 425 | 0 | 0.000559 | 0.881234 | 0.118206 | 0 | 0 | 0 | 0 | 0 |
| 426 | 0.00001 | 0.00044 | 0.878905 | 0.120646 | 0 | 0 | 0 | 0 | 0 |
| 427 | 0 | 0.00054 | 0.885487 | 0.113972 | 0 | 0 | 0 | 0 | 0 |
| 428 | 0.00001 | 0.000565 | 0.887891 | 0.111534 | 0 | 0 | 0 | 0 | 0 |
| 429 | 0.00002 | 0.000451 | 0.885789 | 0.113739 | 0 | 0 | 0 | 0 | 0 |
| 430 | 0.00003 | 0.000732 | 0.884498 | 0.114739 | 0 | 0 | 0 | 0 | 0 |
| 431 | 0.00003 | 0.000339 | 0.883523 | 0.116108 | 0 | 0 | 0 | 0 | 0 |
| 432 | 0.00004 | 0.000581 | 0.877477 | 0.121902 | 0 | 0 | 0 | 0 | 0 |
| 433 | 0.00003 | 0.00042 | 0.882184 | 0.117366 | 0 | 0 | 0 | 0 | 0 |
| 434 | 0 | 0.000659 | 0.884547 | 0.114794 | 0 | 0 | 0 | 0 | 0 |
| 435 | 0.00004 | 0.000541 | 0.882726 | 0.116693 | 0 | 0 | 0 | 0 | 0 |
| 436 | 0 | 0.000401 | 0.880284 | 0.119315 | 0 | 0 | 0 | 0 | 0 |
| 437 | 0 | 0.000441 | 0.883368 | 0.116191 | 0 | 0 | 0 | 0 | 0 |
| 438 | 0.00001 | 0.00068 | 0.882914 | 0.116395 | 0 | 0 | 0 | 0 | 0 |
| 439 | 0 | 0.000553 | 0.882578 | 0.116868 | 0 | 0 | 0 | 0 | 0 |
| 440 | 0.00001 | 0.00042 | 0.885538 | 0.114031 | 0 | 0 | 0 | 0 | 0 |
| 441 | 0 | 0.000378 | 0.883865 | 0.115757 | 0 | 0 | 0 | 0 | 0 |
| 442 | 0.00004 | 0.000332 | 0.881609 | 0.118018 | 0 | 0 | 0 | 0 | 0 |
| 443 | 0.00002 | 0.000509 | 0.884052 | 0.11542 | 0 | 0 | 0 | 0 | 0 |
| 444 | 0.00003 | 0.000651 | 0.881673 | 0.117646 | 0 | 0 | 0 | 0 | 0 |
| 445 | 0.00003 | 0.00045 | 0.878843 | 0.120677 | 0 | 0 | 0 | 0 | 0 |
| 446 | 0.00003 | 0.000373 | 0.882631 | 0.116966 | 0 | 0 | 0 | 0 | 0 |
| 447 | 0.00001 | 0.00038 | 0.886955 | 0.112655 | 0 | 0 | 0 | 0 | 0 |
| 448 | 0.00003 | 0.0007 | 0.882327 | 0.116943 | 0 | 0 | 0 | 0 | 0 |
| 449 | 0.00002 | 0.00043 | 0.885229 | 0.114321 | 0 | 0 | 0 | 0 | 0 |
| 450 | 0.00005 | 0.000489 | 0.882917 | 0.116544 | 0 | 0 | 0 | 0 | 0 |
| 451 | 0.00001 | 0.000569 | 0.88695 | 0.112471 | 0 | 0 | 0 | 0 | 0 |
| 452 | 0.00002 | 0.000449 | 0.883516 | 0.116016 | 0 | 0 | 0 | 0 | 0 |
| 453 | 0.00004 | 0.000547 | 0.883443 | 0.11597 | 0 | 0 | 0 | 0 | 0 |
| 454 | 0.00002 | 0.000449 | 0.884904 | 0.114627 | 0 | 0 | 0 | 0 | 0 |
| 455 | 0.00005 | 0.000467 | 0.888982 | 0.110501 | 0 | 0 | 0 | 0 | 0 |
| 456 | 0.00004 | 0.000488 | 0.883251 | 0.116221 | 0 | 0 | 0 | 0 | 0 |
| 457 | 0.00001 | 0.000428 | 0.880929 | 0.118563 | 0 | 0 | 0 | 0 | 0 |
| 458 | 0.00002 | 0.000451 | 0.884437 | 0.115092 | 0 | 0 | 0 | 0 | 0 |
| 459 | 0.00002 | 0.000331 | 0.879359 | 0.12029 | 0 | 0 | 0 | 0 | 0 |
| 460 | 0 | 0.00065 | 0.881421 | 0.117929 | 0 | 0 | 0 | 0 | 0 |
| 461 | 0.00002 | 0.00044 | 0.883216 | 0.116324 | 0 | 0 | 0 | 0 | 0 |
| 462 | 0 | 0.000419 | 0.888291 | 0.111291 | 0 | 0 | 0 | 0 | 0 |
| 463 | 0.00003 | 0.000436 | 0.882816 | 0.116718 | 0 | 0 | 0 | 0 | 0 |
| 464 | 0.00001 | 0.000586 | 0.883486 | 0.115798 | 0 | 0 | 0 | 0 | 0 |
| 465 | 0.00005 | 0.000319 | 0.8838 | 0.11583 | 0 | 0 | 0 | 0 | 0 |
| 466 | 0.00002 | 0.00042 | 0.880238 | 0.119322 | 0 | 0 | 0 | 0 | 0 |
| 467 | 0.00004 | 0.000291 | 0.878146 | 0.121523 | 0 | 0 | 0 | 0 | 0 |
| 468 | 0.00002 | 0.000491 | 0.883459 | 0.11603 | 0 | 0 | 0 | 0 | 0 |
| 469 | 0.00001 | 0.000475 | 0.885251 | 0.114264 | 0 | 0 | 0 | 0 | 0 |
| 470 | 0.00002 | 0.000351 | 0.885613 | 0.114016 | 0 | 0 | 0 | 0 | 0 |
| 471 | 0.00001 | 0.000539 | 0.885505 | 0.113946 | 0 | 0 | 0 | 0 | 0 |
| 472 | 0.00001 | 0.0004 | 0.883044 | 0.116546 | 0 | 0 | 0 | 0 | 0 |
| 473 | 0 | 0.000562 | 0.883732 | 0.115706 | 0 | 0 | 0 | 0 | 0 |
| 474 | 0 | 0.000439 | 0.883815 | 0.115746 | 0 | 0 | 0 | 0 | 0 |
| 475 | 0.00002 | 0.000408 | 0.883357 | 0.116215 | 0 | 0 | 0 | 0 | 0 |
| 476 | 0.00001 | 0.000499 | 0.877941 | 0.12155 | 0 | 0 | 0 | 0 | 0 |
| 477 | 0.00002 | 0.0004 | 0.883954 | 0.115626 | 0 | 0 | 0 | 0 | 0 |
| 478 | 0.00005 | 0.000461 | 0.881934 | 0.117555 | 0 | 0 | 0 | 0 | 0 |
| 479 | 0.00001 | 0.000371 | 0.880825 | 0.118794 | 0 | 0 | 0 | 0 | 0 |
| 480 | 0.00003 | 0.000562 | 0.879494 | 0.119914 | 0 | 0 | 0 | 0 | 0 |
| 481 | 0 | 0.000522 | 0.889367 | 0.110111 | 0 | 0 | 0 | 0 | 0 |
| 482 | 0 | 0.00046 | 0.879977 | 0.119563 | 0 | 0 | 0 | 0 | 0 |
| 483 | 0.00001 | 0.00037 | 0.883532 | 0.116088 | 0 | 0 | 0 | 0 | 0 |
| 484 | 0 | 0.000459 | 0.876838 | 0.122703 | 0 | 0 | 0 | 0 | 0 |
| 485 | 0 | 0.000527 | 0.889636 | 0.109836 | 0 | 0 | 0 | 0 | 0 |
| 486 | 0.00002 | 0.00049 | 0.884242 | 0.115247 | 0 | 0 | 0 | 0 | 0 |
| 487 | 0 | 0.000359 | 0.885806 | 0.113835 | 0 | 0 | 0 | 0 | 0 |
| 488 | 0.00002 | 0.00046 | 0.883972 | 0.115547 | 0 | 0 | 0 | 0 | 0 |
| 489 | 0.00001 | 0.000553 | 0.884785 | 0.114652 | 0 | 0 | 0 | 0 | 0 |
| 490 | 0.00002 | 0.000718 | 0.884423 | 0.11484 | 0 | 0 | 0 | 0 | 0 |
| 491 | 0 | 0.00052 | 0.882341 | 0.117139 | 0 | 0 | 0 | 0 | 0 |
| 492 | 0.00002 | 0.000453 | 0.879037 | 0.12049 | 0 | 0 | 0 | 0 | 0 |
| 493 | 0.00002 | 0.000472 | 0.882637 | 0.116871 | 0 | 0 | 0 | 0 | 0 |
| 494 | 0.00001 | 0.000462 | 0.880995 | 0.118533 | 0 | 0 | 0 | 0 | 0 |
| 495 | 0 | 0.000479 | 0.883744 | 0.115778 | 0 | 0 | 0 | 0 | 0 |
| 496 | 0.00001 | 0.000409 | 0.879719 | 0.119753 | 0 | 0 | 0 | 0 | 0 |
| 497 | 0 | 0.000502 | 0.882086 | 0.117411 | 0 | 0 | 0 | 0 | 0 |
| 498 | 0.00005 | 0.000503 | 0.887765 | 0.111681 | 0 | 0 | 0 | 0 | 0 |
| 499 | 0.00004 | 0.000482 | 0.881845 | 0.117633 | 0 | 0 | 0 | 0 | 0 |
| 500 | 0.00006 | 0.000458 | 0.885604 | 0.113878 | 0 | 0 | 0 | 0 | 0 |
| 501 | 0 | 0.000541 | 0.884947 | 0.114512 | 0 | 0 | 0 | 0 | 0 |
| 502 | 0.00001 | 0.000603 | 0.884625 | 0.114761 | 0 | 0 | 0 | 0 | 0 |
| 503 | 0.00002 | 0.000432 | 0.882918 | 0.11663 | 0 | 0 | 0 | 0 | 0 |
| 504 | 0 | 0.00031 | 0.88313 | 0.11656 | 0 | 0 | 0 | 0 | 0 |
| 505 | 0.00004 | 0.00051 | 0.883193 | 0.116236 | 0 | 0 | 0 | 0 | 0 |
| 506 | 0.00001 | 0.00034 | 0.883928 | 0.115722 | 0 | 0 | 0 | 0 | 0 |
| 507 | 0.00004 | 0.000483 | 0.882893 | 0.116583 | 0 | 0 | 0 | 0 | 0 |
| 508 | 0 | 0.000568 | 0.880718 | 0.118713 | 0 | 0 | 0 | 0 | 0 |
| 509 | 0 | 0.000449 | 0.883575 | 0.115975 | 0 | 0 | 0 | 0 | 0 |
| 510 | 0.00001 | 0.000517 | 0.885187 | 0.114286 | 0 | 0 | 0 | 0 | 0 |
| 511 | 0.00007 | 0.000311 | 0.88361 | 0.116009 | 0 | 0 | 0 | 0 | 0 |
| 512 | 0.00001 | 0.00043 | 0.879679 | 0.119881 | 0 | 0 | 0 | 0 | 0 |
| 513 | 0.00001 | 0.0005 | 0.883698 | 0.115792 | 0 | 0 | 0 | 0 | 0 |
| 514 | 0.00002 | 0.000341 | 0.883959 | 0.11568 | 0 | 0 | 0 | 0 | 0 |
| 515 | 0.00003 | 0.000419 | 0.883981 | 0.11557 | 0 | 0 | 0 | 0 | 0 |
| 516 | 0.00002 | 0.000478 | 0.877223 | 0.122279 | 0 | 0 | 0 | 0 | 0 |
| 517 | 0.00002 | 0.000581 | 0.879819 | 0.11958 | 0 | 0 | 0 | 0 | 0 |
| 518 | 0.00005 | 0.00049 | 0.884279 | 0.115181 | 0 | 0 | 0 | 0 | 0 |
| 519 | 0.00003 | 0.000422 | 0.881297 | 0.11812 | 0 | 0 | 0 | 0 | 0 |
| 520 | 0.00005 | 0.000391 | 0.883792 | 0.115747 | 0 | 0 | 0 | 0 | 0 |
| 521 | 0.00001 | 0.000529 | 0.881415 | 0.118046 | 0 | 0 | 0 | 0 | 0 |
| 522 | 0 | 0.00049 | 0.884225 | 0.115285 | 0 | 0 | 0 | 0 | 0 |
| 523 | 0.00001 | 0.000403 | 0.88329 | 0.116297 | 0 | 0 | 0 | 0 | 0 |
| 524 | 0.00004 | 0.000279 | 0.880875 | 0.118807 | 0 | 0 | 0 | 0 | 0 |
| 525 | 0.00001 | 0.000512 | 0.880229 | 0.119249 | 0 | 0 | 0 | 0 | 0 |
| 526 | 0.00004 | 0.000442 | 0.88095 | 0.118568 | 0 | 0 | 0 | 0 | 0 |
| 527 | 0 | 0.000389 | 0.883445 | 0.116166 | 0 | 0 | 0 | 0 | 0 |
| 528 | 0.00006 | 0.000429 | 0.881458 | 0.118052 | 0 | 0 | 0 | 0 | 0 |
| 529 | 0.00002 | 0.000511 | 0.880061 | 0.119408 | 0 | 0 | 0 | 0 | 0 |
| 530 | 0.00002 | 0.000609 | 0.877824 | 0.121547 | 0 | 0 | 0 | 0 | 0 |
| 531 | 0.00001 | 0.000553 | 0.883206 | 0.116231 | 0 | 0 | 0 | 0 | 0 |
| 532 | 0.00001 | 0.000359 | 0.886733 | 0.112898 | 0 | 0 | 0 | 0 | 0 |
| 533 | 0 | 0.000473 | 0.882052 | 0.117475 | 0 | 0 | 0 | 0 | 0 |
| 534 | 0.00003 | 0.000435 | 0.876313 | 0.123223 | 0 | 0 | 0 | 0 | 0 |
| 535 | 0 | 0.00059 | 0.886838 | 0.112572 | 0 | 0 | 0 | 0 | 0 |
| 536 | 0.00001 | 0.000369 | 0.886884 | 0.112637 | 0 | 0 | 0 | 0 | 0 |
| 537 | 0.00001 | 0.000318 | 0.884343 | 0.115328 | 0 | 0 | 0 | 0 | 0 |
| 538 | 0 | 0.00053 | 0.87923 | 0.120241 | 0 | 0 | 0 | 0 | 0 |
| 539 | 0.00003 | 0.000672 | 0.880764 | 0.118534 | 0 | 0 | 0 | 0 | 0 |
| 540 | 0.00002 | 0.000702 | 0.879483 | 0.119795 | 0 | 0 | 0 | 0 | 0 |
| 541 | 0.00001 | 0.000419 | 0.883598 | 0.115973 | 0 | 0 | 0 | 0 | 0 |
| 542 | 0.00003 | 0.000533 | 0.883245 | 0.116192 | 0 | 0 | 0 | 0 | 0 |
| 543 | 0.00002 | 0.000382 | 0.887275 | 0.112324 | 0 | 0 | 0 | 0 | 0 |
| 544 | 0 | 0.00049 | 0.886016 | 0.113465 | 0 | 0 | 0 | 0 | 0 |
| 545 | 0.00003 | 0.000528 | 0.878898 | 0.120544 | 0 | 0 | 0 | 0 | 0 |
| 546 | 0.00002 | 0.000581 | 0.884086 | 0.115313 | 0 | 0 | 0 | 0 | 0 |
| 547 | 0.00006 | 0.000429 | 0.882493 | 0.117018 | 0 | 0 | 0 | 0 | 0 |
| 548 | 0.00001 | 0.000487 | 0.884265 | 0.115238 | 0 | 0 | 0 | 0 | 0 |
| 549 | 0 | 0.000369 | 0.879753 | 0.119877 | 0 | 0 | 0 | 0 | 0 |
| 550 | 0.00004 | 0.000398 | 0.885295 | 0.114267 | 0 | 0 | 0 | 0 | 0 |
| 551 | 0.00005 | 0.000601 | 0.882904 | 0.116444 | 0 | 0 | 0 | 0 | 0 |
| 552 | 0.00002 | 0.000379 | 0.881763 | 0.117838 | 0 | 0 | 0 | 0 | 0 |
| 553 | 0.00003 | 0.000585 | 0.880644 | 0.118741 | 0 | 0 | 0 | 0 | 0 |
| 554 | 0.00003 | 0.000492 | 0.885108 | 0.11437 | 0 | 0 | 0 | 0 | 0 |
| 555 | 0.00002 | 0.00037 | 0.881069 | 0.118541 | 0 | 0 | 0 | 0 | 0 |
| 556 | 0 | 0.000399 | 0.885257 | 0.114344 | 0 | 0 | 0 | 0 | 0 |
| 557 | 0 | 0.000558 | 0.884015 | 0.115426 | 0 | 0 | 0 | 0 | 0 |
| 558 | 0.00002 | 0.000428 | 0.883595 | 0.115957 | 0 | 0 | 0 | 0 | 0 |
| 559 | 0 | 0.0005 | 0.883391 | 0.116109 | 0 | 0 | 0 | 0 | 0 |
| 560 | 0.00004 | 0.00039 | 0.883141 | 0.116429 | 0 | 0 | 0 | 0 | 0 |
| 561 | 0.00002 | 0.000509 | 0.881109 | 0.118362 | 0 | 0 | 0 | 0 | 0 |
| 562 | 0.00001 | 0.000403 | 0.880522 | 0.119065 | 0 | 0 | 0 | 0 | 0 |
| 563 | 0 | 0.000537 | 0.880987 | 0.118476 | 0 | 0 | 0 | 0 | 0 |
| 564 | 0.00002 | 0.000457 | 0.887776 | 0.111747 | 0 | 0 | 0 | 0 | 0 |
| 565 | 0.00001 | 0.000501 | 0.87811 | 0.121378 | 0 | 0 | 0 | 0 | 0 |
| 566 | 0.00003 | 0.000471 | 0.883654 | 0.115845 | 0 | 0 | 0 | 0 | 0 |
| 567 | 0.00002 | 0.000382 | 0.881717 | 0.117881 | 0 | 0 | 0 | 0 | 0 |
| 568 | 0.00002 | 0.000532 | 0.882518 | 0.11693 | 0 | 0 | 0 | 0 | 0 |
| 569 | 0.00004 | 0.000548 | 0.886302 | 0.113111 | 0 | 0 | 0 | 0 | 0 |
| 570 | 0.00004 | 0.000529 | 0.877992 | 0.121439 | 0 | 0 | 0 | 0 | 0 |
| 571 | 0 | 0.000601 | 0.884389 | 0.11501 | 0 | 0 | 0 | 0 | 0 |
| 572 | 0 | 0.000501 | 0.883181 | 0.116317 | 0 | 0 | 0 | 0 | 0 |
| 573 | 0.00003 | 0.000619 | 0.884198 | 0.115153 | 0 | 0 | 0 | 0 | 0 |
| 574 | 0 | 0.000487 | 0.883929 | 0.115574 | 0 | 0 | 0 | 0 | 0 |
| 575 | 0.00001 | 0.000428 | 0.881482 | 0.11808 | 0 | 0 | 0 | 0 | 0 |
| 576 | 0.00001 | 0.00034 | 0.88657 | 0.11308 | 0 | 0 | 0 | 0 | 0 |
| 577 | 0.00002 | 0.00045 | 0.879038 | 0.120493 | 0 | 0 | 0 | 0 | 0 |
| 578 | 0.00002 | 0.000453 | 0.883096 | 0.116431 | 0 | 0 | 0 | 0 | 0 |
| 579 | 0.00003 | 0.00047 | 0.882804 | 0.116696 | 0 | 0 | 0 | 0 | 0 |
| 580 | 0.00001 | 0.000631 | 0.886551 | 0.112807 | 0 | 0 | 0 | 0 | 0 |
| 581 | 0.00003 | 0.000479 | 0.88165 | 0.117841 | 0 | 0 | 0 | 0 | 0 |
| 582 | 0.00002 | 0.000411 | 0.883418 | 0.116152 | 0 | 0 | 0 | 0 | 0 |
| 583 | 0.00003 | 0.000519 | 0.879452 | 0.119999 | 0 | 0 | 0 | 0 | 0 |
| 584 | 0.00001 | 0.0003 | 0.879762 | 0.119928 | 0 | 0 | 0 | 0 | 0 |
| 585 | 0.00004 | 0.000428 | 0.88122 | 0.118311 | 0 | 0 | 0 | 0 | 0 |
| 586 | 0.00005 | 0.000562 | 0.881279 | 0.118109 | 0 | 0 | 0 | 0 | 0 |
| 587 | 0.00004 | 0.000379 | 0.882204 | 0.117377 | 0 | 0 | 0 | 0 | 0 |
| 588 | 0.00002 | 0.00049 | 0.885189 | 0.1143 | 0 | 0 | 0 | 0 | 0 |
| 589 | 0.00001 | 0.00045 | 0.883437 | 0.116103 | 0 | 0 | 0 | 0 | 0 |
| 590 | 0.00002 | 0.000494 | 0.878693 | 0.120794 | 0 | 0 | 0 | 0 | 0 |
| 591 | 0.00003 | 0.000359 | 0.882403 | 0.117208 | 0 | 0 | 0 | 0 | 0 |
| 592 | 0 | 0.00055 | 0.883522 | 0.115928 | 0 | 0 | 0 | 0 | 0 |
| 593 | 0 | 0.000321 | 0.887494 | 0.112184 | 0 | 0 | 0 | 0 | 0 |
| 594 | 0.00003 | 0.000381 | 0.881787 | 0.117802 | 0 | 0 | 0 | 0 | 0 |
| 595 | 0.00002 | 0.00049 | 0.882052 | 0.117438 | 0 | 0 | 0 | 0 | 0 |
| 596 | 0.00001 | 0.0004 | 0.884341 | 0.11525 | 0 | 0 | 0 | 0 | 0 |
| 597 | 0.00002 | 0.000439 | 0.880046 | 0.119494 | 0 | 0 | 0 | 0 | 0 |
| 598 | 0.000041 | 0.000507 | 0.883064 | 0.116388 | 0 | 0 | 0 | 0 | 0 |
| 599 | 0 | 0.000339 | 0.882588 | 0.117072 | 0 | 0 | 0 | 0 | 0 |
| 600 | 0.00003 | 0.000381 | 0.881548 | 0.118041 | 0 | 0 | 0 | 0 | 0 |
| 601 | 0.00004 | 0.000517 | 0.88168 | 0.117763 | 0 | 0 | 0 | 0 | 0 |
| 602 | 0 | 0.000432 | 0.885203 | 0.114365 | 0 | 0 | 0 | 0 | 0 |
| 603 | 0.00002 | 0.000549 | 0.882991 | 0.11644 | 0 | 0 | 0 | 0 | 0 |
| 604 | 0.00003 | 0.000531 | 0.883402 | 0.116037 | 0 | 0 | 0 | 0 | 0 |
| 605 | 0.00002 | 0.000518 | 0.883638 | 0.115825 | 0 | 0 | 0 | 0 | 0 |
| 606 | 0.00001 | 0.00055 | 0.886015 | 0.113425 | 0 | 0 | 0 | 0 | 0 |
| 607 | 0.00002 | 0.000658 | 0.886075 | 0.113247 | 0 | 0 | 0 | 0 | 0 |
| 608 | 0.00003 | 0.000408 | 0.880991 | 0.118571 | 0 | 0 | 0 | 0 | 0 |
| 609 | 0.00003 | 0.000392 | 0.885266 | 0.114312 | 0 | 0 | 0 | 0 | 0 |
| 610 | 0.00001 | 0.000351 | 0.884133 | 0.115506 | 0 | 0 | 0 | 0 | 0 |
| 611 | 0.00005 | 0.000492 | 0.883732 | 0.115725 | 0 | 0 | 0 | 0 | 0 |
| 612 | 0.00002 | 0.000361 | 0.879386 | 0.120233 | 0 | 0 | 0 | 0 | 0 |
| 613 | 0 | 0.000561 | 0.879306 | 0.120103 | 0 | 0 | 0 | 0 | 0 |
| 614 | 0.00002 | 0.0004 | 0.887657 | 0.111923 | 0 | 0 | 0 | 0 | 0 |
| 615 | 0.00002 | 0.00044 | 0.8827 | 0.11683 | 0 | 0 | 0 | 0 | 0 |
| 616 | 0 | 0.000502 | 0.885304 | 0.114194 | 0 | 0 | 0 | 0 | 0 |
| 617 | 0 | 0.000556 | 0.883248 | 0.116196 | 0 | 0 | 0 | 0 | 0 |
| 618 | 0.00002 | 0.000473 | 0.880585 | 0.118922 | 0 | 0 | 0 | 0 | 0 |
| 619 | 0.00004 | 0.000427 | 0.885213 | 0.11432 | 0 | 0 | 0 | 0 | 0 |
| 620 | 0.00003 | 0.000451 | 0.88264 | 0.116878 | 0 | 0 | 0 | 0 | 0 |
| 621 | 0 | 0.000299 | 0.885518 | 0.114183 | 0 | 0 | 0 | 0 | 0 |
| 622 | 0.00003 | 0.000578 | 0.87755 | 0.121841 | 0 | 0 | 0 | 0 | 0 |
| 623 | 0.00001 | 0.00041 | 0.882177 | 0.117403 | 0 | 0 | 0 | 0 | 0 |
| 624 | 0 | 0.000522 | 0.881619 | 0.117859 | 0 | 0 | 0 | 0 | 0 |
| 625 | 0.00003 | 0.000511 | 0.884539 | 0.11486 | 0 | 0 | 0 | 0 | 0 |
| 626 | 0.00005 | 0.000478 | 0.881901 | 0.117571 | 0 | 0 | 0 | 0 | 0 |
| 627 | 0.00002 | 0.000378 | 0.884088 | 0.115514 | 0 | 0 | 0 | 0 | 0 |
| 628 | 0.00001 | 0.000509 | 0.880184 | 0.119297 | 0 | 0 | 0 | 0 | 0 |
| 629 | 0.00004 | 0.00037 | 0.882751 | 0.116839 | 0 | 0 | 0 | 0 | 0 |
| 630 | 0.00003 | 0.00044 | 0.881301 | 0.118229 | 0 | 0 | 0 | 0 | 0 |
| 631 | 0.00002 | 0.00038 | 0.882619 | 0.116981 | 0 | 0 | 0 | 0 | 0 |
| 632 | 0.00001 | 0.00039 | 0.88353 | 0.11607 | 0 | 0 | 0 | 0 | 0 |
| 633 | 0 | 0.000313 | 0.882928 | 0.11676 | 0 | 0 | 0 | 0 | 0 |
| 634 | 0.00002 | 0.000503 | 0.881757 | 0.11772 | 0 | 0 | 0 | 0 | 0 |
| 635 | 0.00007 | 0.000558 | 0.883606 | 0.115766 | 0 | 0 | 0 | 0 | 0 |
| 636 | 0.00001 | 0.00035 | 0.880241 | 0.119399 | 0 | 0 | 0 | 0 | 0 |
| 637 | 0.00003 | 0.000369 | 0.88396 | 0.115642 | 0 | 0 | 0 | 0 | 0 |
| 638 | 0.00001 | 0.000638 | 0.881821 | 0.117531 | 0 | 0 | 0 | 0 | 0 |
| 639 | 0.00005 | 0.00043 | 0.879781 | 0.119739 | 0 | 0 | 0 | 0 | 0 |
| 640 | 0 | 0.00034 | 0.878242 | 0.121417 | 0 | 0 | 0 | 0 | 0 |
| 641 | 0.00005 | 0.000401 | 0.884082 | 0.115466 | 0 | 0 | 0 | 0 | 0 |
| 642 | 0.00001 | 0.000527 | 0.886527 | 0.112936 | 0 | 0 | 0 | 0 | 0 |
| 643 | 0.00003 | 0.00035 | 0.883909 | 0.11571 | 0 | 0 | 0 | 0 | 0 |
| 644 | 0.00001 | 0.00052 | 0.887222 | 0.112247 | 0 | 0 | 0 | 0 | 0 |
| 645 | 0 | 0.000381 | 0.884399 | 0.11522 | 0 | 0 | 0 | 0 | 0 |
| 646 | 0.00008 | 0.000538 | 0.879689 | 0.119693 | 0 | 0 | 0 | 0 | 0 |
| 647 | 0.00001 | 0.000429 | 0.884844 | 0.114718 | 0 | 0 | 0 | 0 | 0 |
| 648 | 0.00005 | 0.000461 | 0.885175 | 0.114314 | 0 | 0 | 0 | 0 | 0 |
| 649 | 0.00001 | 0.00056 | 0.884824 | 0.114606 | 0 | 0 | 0 | 0 | 0 |
| 650 | 0.00001 | 0.000524 | 0.881421 | 0.118045 | 0 | 0 | 0 | 0 | 0 |
| 651 | 0.00002 | 0.000327 | 0.884966 | 0.114688 | 0 | 0 | 0 | 0 | 0 |
| 652 | 0.00005 | 0.00037 | 0.882749 | 0.11683 | 0 | 0 | 0 | 0 | 0 |
| 653 | 0.00003 | 0.000507 | 0.884097 | 0.115366 | 0 | 0 | 0 | 0 | 0 |
| 654 | 0.00001 | 0.000381 | 0.884672 | 0.114937 | 0 | 0 | 0 | 0 | 0 |
| 655 | 0.00003 | 0.000541 | 0.876616 | 0.122813 | 0 | 0 | 0 | 0 | 0 |
| 656 | 0.00003 | 0.000351 | 0.88822 | 0.111399 | 0 | 0 | 0 | 0 | 0 |
| 657 | 0.00003 | 0.000392 | 0.883778 | 0.1158 | 0 | 0 | 0 | 0 | 0 |
| 658 | 0 | 0.00054 | 0.882261 | 0.117199 | 0 | 0 | 0 | 0 | 0 |
| 659 | 0 | 0.000633 | 0.882325 | 0.117042 | 0 | 0 | 0 | 0 | 0 |
| 660 | 0.00001 | 0.00051 | 0.887401 | 0.112078 | 0 | 0 | 0 | 0 | 0 |
| 661 | 0.00001 | 0.000478 | 0.882924 | 0.116587 | 0 | 0 | 0 | 0 | 0 |
| 662 | 0 | 0.000581 | 0.878356 | 0.121063 | 0 | 0 | 0 | 0 | 0 |
| 663 | 0.00003 | 0.000511 | 0.883778 | 0.11568 | 0 | 0 | 0 | 0 | 0 |
| 664 | 0.00002 | 0.000382 | 0.888223 | 0.111346 | 0 | 0 | 0 | 0 | 0 |
| 665 | 0.00002 | 0.000413 | 0.878908 | 0.120659 | 0 | 0 | 0 | 0 | 0 |
| 666 | 0 | 0.000402 | 0.881813 | 0.117785 | 0 | 0 | 0 | 0 | 0 |
| 667 | 0.00002 | 0.000481 | 0.882196 | 0.117304 | 0 | 0 | 0 | 0 | 0 |
| 668 | 0.00001 | 0.00053 | 0.881276 | 0.118183 | 0 | 0 | 0 | 0 | 0 |
| 669 | 0.00001 | 0.000543 | 0.881034 | 0.118413 | 0 | 0 | 0 | 0 | 0 |
| 670 | 0.00001 | 0.000431 | 0.880588 | 0.118971 | 0 | 0 | 0 | 0 | 0 |
| 671 | 0.00001 | 0.000478 | 0.881455 | 0.118057 | 0 | 0 | 0 | 0 | 0 |
| 672 | 0.00002 | 0.000358 | 0.881873 | 0.117748 | 0 | 0 | 0 | 0 | 0 |
| 673 | 0.00001 | 0.00065 | 0.883215 | 0.116125 | 0 | 0 | 0 | 0 | 0 |
| 674 | 0.00005 | 0.000443 | 0.88391 | 0.115596 | 0 | 0 | 0 | 0 | 0 |
| 675 | 0.00005 | 0.000432 | 0.883703 | 0.115815 | 0 | 0 | 0 | 0 | 0 |
| 676 | 0 | 0.000668 | 0.884633 | 0.114699 | 0 | 0 | 0 | 0 | 0 |
| 677 | 0.00001 | 0.000421 | 0.883963 | 0.115606 | 0 | 0 | 0 | 0 | 0 |
| 678 | 0.00002 | 0.00047 | 0.880142 | 0.119368 | 0 | 0 | 0 | 0 | 0 |
| 679 | 0.00001 | 0.000553 | 0.884502 | 0.114935 | 0 | 0 | 0 | 0 | 0 |
| 680 | 0 | 0.00061 | 0.880408 | 0.118982 | 0 | 0 | 0 | 0 | 0 |
| 681 | 0.00003 | 0.00048 | 0.880564 | 0.118926 | 0 | 0 | 0 | 0 | 0 |
| 682 | 0 | 0.000371 | 0.882263 | 0.117366 | 0 | 0 | 0 | 0 | 0 |
| 683 | 0.000079 | 0.000485 | 0.88479 | 0.114646 | 0 | 0 | 0 | 0 | 0 |
| 684 | 0.00001 | 0.000291 | 0.883481 | 0.116218 | 0 | 0 | 0 | 0 | 0 |
| 685 | 0.00002 | 0.000443 | 0.887854 | 0.111682 | 0 | 0 | 0 | 0 | 0 |
| 686 | 0.00003 | 0.000478 | 0.877692 | 0.12178 | 0 | 0 | 0 | 0 | 0 |
| 687 | 0.00002 | 0.00036 | 0.883541 | 0.116078 | 0 | 0 | 0 | 0 | 0 |
| 688 | 0.00003 | 0.00034 | 0.881992 | 0.117638 | 0 | 0 | 0 | 0 | 0 |
| 689 | 0.00001 | 0.000484 | 0.882631 | 0.116875 | 0 | 0 | 0 | 0 | 0 |
| 690 | 0.00001 | 0.00046 | 0.882607 | 0.116923 | 0 | 0 | 0 | 0 | 0 |
| 691 | 0.00001 | 0.00049 | 0.882502 | 0.116998 | 0 | 0 | 0 | 0 | 0 |
| 692 | 0 | 0.000523 | 0.88173 | 0.117747 | 0 | 0 | 0 | 0 | 0 |
| 693 | 0 | 0.000643 | 0.880888 | 0.118469 | 0 | 0 | 0 | 0 | 0 |
| 694 | 0 | 0.000538 | 0.885298 | 0.114164 | 0 | 0 | 0 | 0 | 0 |
| 695 | 0.00002 | 0.000411 | 0.884563 | 0.115005 | 0 | 0 | 0 | 0 | 0 |
| 696 | 0.00002 | 0.000499 | 0.887456 | 0.112025 | 0 | 0 | 0 | 0 | 0 |
| 697 | 0.00001 | 0.00047 | 0.885886 | 0.113634 | 0 | 0 | 0 | 0 | 0 |
| 698 | 0.00004 | 0.000511 | 0.882682 | 0.116766 | 0 | 0 | 0 | 0 | 0 |
| 699 | 0.00002 | 0.00045 | 0.883379 | 0.116151 | 0 | 0 | 0 | 0 | 0 |
| 700 | 0.00003 | 0.000501 | 0.88372 | 0.115749 | 0 | 0 | 0 | 0 | 0 |
| 701 | 0.00004 | 0.000499 | 0.891368 | 0.108093 | 0 | 0 | 0 | 0 | 0 |
| 702 | 0.00004 | 0.000511 | 0.887928 | 0.111521 | 0 | 0 | 0 | 0 | 0 |
| 703 | 0.00001 | 0.00037 | 0.883145 | 0.116475 | 0 | 0 | 0 | 0 | 0 |
| 704 | 0.00001 | 0.000419 | 0.882413 | 0.117158 | 0 | 0 | 0 | 0 | 0 |
| 705 | 0.00001 | 0.00052 | 0.879877 | 0.119593 | 0 | 0 | 0 | 0 | 0 |
| 706 | 0.00002 | 0.000522 | 0.881064 | 0.118394 | 0 | 0 | 0 | 0 | 0 |
| 707 | 0.00001 | 0.000702 | 0.87872 | 0.120567 | 0 | 0 | 0 | 0 | 0 |
| 708 | 0 | 0.000569 | 0.88269 | 0.116741 | 0 | 0 | 0 | 0 | 0 |
| 709 | 0.00001 | 0.00063 | 0.887529 | 0.111832 | 0 | 0 | 0 | 0 | 0 |
| 710 | 0.00002 | 0.000607 | 0.884149 | 0.115224 | 0 | 0 | 0 | 0 | 0 |
| 711 | 0.00001 | 0.000528 | 0.883823 | 0.115639 | 0 | 0 | 0 | 0 | 0 |
| 712 | 0 | 0.000501 | 0.889102 | 0.110397 | 0 | 0 | 0 | 0 | 0 |
| 713 | 0 | 0.000382 | 0.879337 | 0.120271 | 0 | 0 | 0 | 0 | 0 |
| 714 | 0.00001 | 0.000437 | 0.884245 | 0.115307 | 0 | 0 | 0 | 0 | 0 |
| 715 | 0.00002 | 0.00032 | 0.876497 | 0.123164 | 0 | 0 | 0 | 0 | 0 |
| 716 | 0 | 0.000532 | 0.876615 | 0.122853 | 0 | 0 | 0 | 0 | 0 |
| 717 | 0.00003 | 0.00058 | 0.882459 | 0.116931 | 0 | 0 | 0 | 0 | 0 |
| 718 | 0.00004 | 0.00063 | 0.887873 | 0.111457 | 0 | 0 | 0 | 0 | 0 |
| 719 | 0.00002 | 0.000422 | 0.885532 | 0.114026 | 0 | 0 | 0 | 0 | 0 |
| 720 | 0.00004 | 0.000389 | 0.879987 | 0.119524 | 0 | 0 | 0 | 0 | 0 |
| 721 | 0.00003 | 0.000462 | 0.879308 | 0.1202 | 0 | 0 | 0 | 0 | 0 |
| 722 | 0.00001 | 0.000581 | 0.884504 | 0.114905 | 0 | 0 | 0 | 0 | 0 |
| 723 | 0.00003 | 0.000488 | 0.883445 | 0.116038 | 0 | 0 | 0 | 0 | 0 |
| 724 | 0.00003 | 0.000439 | 0.884033 | 0.115498 | 0 | 0 | 0 | 0 | 0 |
| 725 | 0.00001 | 0.000451 | 0.880771 | 0.118768 | 0 | 0 | 0 | 0 | 0 |
| 726 | 0.00004 | 0.000611 | 0.885457 | 0.113892 | 0 | 0 | 0 | 0 | 0 |
| 727 | 0.00002 | 0.00039 | 0.879095 | 0.120496 | 0 | 0 | 0 | 0 | 0 |
| 728 | 0 | 0.000569 | 0.883629 | 0.115803 | 0 | 0 | 0 | 0 | 0 |
| 729 | 0.00004 | 0.000692 | 0.882542 | 0.116726 | 0 | 0 | 0 | 0 | 0 |
| 730 | 0.00002 | 0.000483 | 0.881296 | 0.118201 | 0 | 0 | 0 | 0 | 0 |
| 731 | 0.00003 | 0.000623 | 0.884736 | 0.114611 | 0 | 0 | 0 | 0 | 0 |
| 732 | 0.00001 | 0.000427 | 0.884466 | 0.115097 | 0 | 0 | 0 | 0 | 0 |
| 733 | 0.00001 | 0.000478 | 0.884795 | 0.114717 | 0 | 0 | 0 | 0 | 0 |
| 734 | 0.00001 | 0.000389 | 0.885065 | 0.114537 | 0 | 0 | 0 | 0 | 0 |
| 735 | 0.00002 | 0.000532 | 0.884209 | 0.115239 | 0 | 0 | 0 | 0 | 0 |
| 736 | 0.00002 | 0.000459 | 0.88513 | 0.114391 | 0 | 0 | 0 | 0 | 0 |
| 737 | 0.00001 | 0.000603 | 0.884171 | 0.115216 | 0 | 0 | 0 | 0 | 0 |
| 738 | 0.00001 | 0.000351 | 0.880564 | 0.119075 | 0 | 0 | 0 | 0 | 0 |
| 739 | 0.00003 | 0.000411 | 0.880774 | 0.118785 | 0 | 0 | 0 | 0 | 0 |
| 740 | 0 | 0.000561 | 0.885215 | 0.114224 | 0 | 0 | 0 | 0 | 0 |
| 741 | 0.00001 | 0.000462 | 0.884361 | 0.115167 | 0 | 0 | 0 | 0 | 0 |
| 742 | 0.00002 | 0.00035 | 0.884389 | 0.115241 | 0 | 0 | 0 | 0 | 0 |
| 743 | 0.00001 | 0.000502 | 0.882459 | 0.117029 | 0 | 0 | 0 | 0 | 0 |
| 744 | 0.00001 | 0.000679 | 0.886617 | 0.112654 | 0 | 0 | 0 | 0 | 0 |
| 745 | 0.00002 | 0.000473 | 0.887267 | 0.1122 | 0 | 0 | 0 | 0 | 0 |
| 746 | 0.00001 | 0.00052 | 0.879875 | 0.119595 | 0 | 0 | 0 | 0 | 0 |
| 747 | 0.00002 | 0.00039 | 0.881383 | 0.118208 | 0 | 0 | 0 | 0 | 0 |
| 748 | 0.00003 | 0.00058 | 0.885715 | 0.113676 | 0 | 0 | 0 | 0 | 0 |
| 749 | 0 | 0.000402 | 0.887216 | 0.112382 | 0 | 0 | 0 | 0 | 0 |
| 750 | 0.00002 | 0.000481 | 0.884483 | 0.115016 | 0 | 0 | 0 | 0 | 0 |
| 751 | 0.00001 | 0.000499 | 0.885673 | 0.113818 | 0 | 0 | 0 | 0 | 0 |
| 752 | 0.00002 | 0.000359 | 0.883234 | 0.116387 | 0 | 0 | 0 | 0 | 0 |
| 753 | 0.00004 | 0.000382 | 0.884868 | 0.114711 | 0 | 0 | 0 | 0 | 0 |
| 754 | 0.00001 | 0.000462 | 0.881315 | 0.118213 | 0 | 0 | 0 | 0 | 0 |
| 755 | 0.00001 | 0.000499 | 0.880281 | 0.11921 | 0 | 0 | 0 | 0 | 0 |
| 756 | 0.00003 | 0.00041 | 0.880678 | 0.118881 | 0 | 0 | 0 | 0 | 0 |
| 757 | 0.00002 | 0.000501 | 0.882089 | 0.11739 | 0 | 0 | 0 | 0 | 0 |
| 758 | 0.00005 | 0.000529 | 0.879577 | 0.119844 | 0 | 0 | 0 | 0 | 0 |
| 759 | 0.00003 | 0.00043 | 0.881845 | 0.117696 | 0 | 0 | 0 | 0 | 0 |
| 760 | 0.00002 | 0.000544 | 0.883258 | 0.116178 | 0 | 0 | 0 | 0 | 0 |
| 761 | 0.00004 | 0.00039 | 0.883176 | 0.116394 | 0 | 0 | 0 | 0 | 0 |
| 762 | 0.00002 | 0.000529 | 0.883456 | 0.115995 | 0 | 0 | 0 | 0 | 0 |
| 763 | 0.00002 | 0.000462 | 0.882581 | 0.116937 | 0 | 0 | 0 | 0 | 0 |
| 764 | 0.00002 | 0.000341 | 0.887313 | 0.112326 | 0 | 0 | 0 | 0 | 0 |
| 765 | 0.00001 | 0.000485 | 0.883574 | 0.115931 | 0 | 0 | 0 | 0 | 0 |
| 766 | 0.00002 | 0.00047 | 0.881041 | 0.11847 | 0 | 0 | 0 | 0 | 0 |
| 767 | 0.00002 | 0.000389 | 0.883856 | 0.115735 | 0 | 0 | 0 | 0 | 0 |
| 768 | 0.00002 | 0.000549 | 0.881909 | 0.117521 | 0 | 0 | 0 | 0 | 0 |
| 769 | 0.00001 | 0.000537 | 0.884499 | 0.114954 | 0 | 0 | 0 | 0 | 0 |
| 770 | 0.00002 | 0.000601 | 0.882833 | 0.116545 | 0 | 0 | 0 | 0 | 0 |
| 771 | 0 | 0.000389 | 0.882234 | 0.117297 | 0 | 0 | 0 | 0 | 0 |
| 772 | 0.00005 | 0.000402 | 0.881081 | 0.118456 | 0 | 0 | 0 | 0 | 0 |
| 773 | 0.00001 | 0.000639 | 0.879387 | 0.119963 | 0 | 0 | 0 | 0 | 0 |
| 774 | 0.00001 | 0.000419 | 0.882943 | 0.116628 | 0 | 0 | 0 | 0 | 0 |
| 775 | 0 | 0.000448 | 0.88469 | 0.114862 | 0 | 0 | 0 | 0 | 0 |
| 776 | 0.00003 | 0.000539 | 0.882527 | 0.116903 | 0 | 0 | 0 | 0 | 0 |
| 777 | 0.00002 | 0.00053 | 0.884343 | 0.115106 | 0 | 0 | 0 | 0 | 0 |
| 778 | 0.00001 | 0.000508 | 0.887126 | 0.112356 | 0 | 0 | 0 | 0 | 0 |
| 779 | 0.00003 | 0.000518 | 0.888036 | 0.111416 | 0 | 0 | 0 | 0 | 0 |
| 780 | 0.00001 | 0.000502 | 0.881696 | 0.117792 | 0 | 0 | 0 | 0 | 0 |
| 781 | 0.00003 | 0.000605 | 0.88238 | 0.116986 | 0 | 0 | 0 | 0 | 0 |
| 782 | 0.00002 | 0.000498 | 0.88671 | 0.112772 | 0 | 0 | 0 | 0 | 0 |
| 783 | 0.00002 | 0.000486 | 0.882367 | 0.117127 | 0 | 0 | 0 | 0 | 0 |
| 784 | 0.00003 | 0.000514 | 0.883237 | 0.116219 | 0 | 0 | 0 | 0 | 0 |
| 785 | 0.00001 | 0.00042 | 0.883941 | 0.115629 | 0 | 0 | 0 | 0 | 0 |
| 786 | 0 | 0.000531 | 0.880958 | 0.118511 | 0 | 0 | 0 | 0 | 0 |
| 787 | 0.00004 | 0.000419 | 0.882367 | 0.117174 | 0 | 0 | 0 | 0 | 0 |
| 788 | 0 | 0.000512 | 0.887028 | 0.11246 | 0 | 0 | 0 | 0 | 0 |
| 789 | 0.00001 | 0.000499 | 0.879546 | 0.119945 | 0 | 0 | 0 | 0 | 0 |
| 790 | 0.00001 | 0.0003 | 0.883892 | 0.115798 | 0 | 0 | 0 | 0 | 0 |
| 791 | 0.00001 | 0.000389 | 0.886223 | 0.113378 | 0 | 0 | 0 | 0 | 0 |
| 792 | 0.00003 | 0.000479 | 0.8842 | 0.11529 | 0 | 0 | 0 | 0 | 0 |
| 793 | 0.00003 | 0.000551 | 0.879336 | 0.120083 | 0 | 0 | 0 | 0 | 0 |
| 794 | 0.00002 | 0.000462 | 0.886759 | 0.112758 | 0 | 0 | 0 | 0 | 0 |
| 795 | 0.00004 | 0.00053 | 0.878679 | 0.120751 | 0 | 0 | 0 | 0 | 0 |
| 796 | 0.00005 | 0.00049 | 0.883832 | 0.115628 | 0 | 0 | 0 | 0 | 0 |
| 797 | 0 | 0.000432 | 0.881892 | 0.117676 | 0 | 0 | 0 | 0 | 0 |
| 798 | 0.00004 | 0.000427 | 0.882179 | 0.117334 | 0 | 0 | 0 | 0 | 0 |
| 799 | 0.00003 | 0.000551 | 0.884079 | 0.11534 | 0 | 0 | 0 | 0 | 0 |
| 800 | 0.00002 | 0.000431 | 0.879716 | 0.119833 | 0 | 0 | 0 | 0 | 0 |
| 801 | 0.00001 | 0.000478 | 0.888726 | 0.110776 | 0 | 0 | 0 | 0 | 0 |
| 802 | 0.00003 | 0.000481 | 0.882013 | 0.117476 | 0 | 0 | 0 | 0 | 0 |
| 803 | 0.00002 | 0.000528 | 0.886571 | 0.112882 | 0 | 0 | 0 | 0 | 0 |
| 804 | 0.00001 | 0.000509 | 0.882483 | 0.116998 | 0 | 0 | 0 | 0 | 0 |
| 805 | 0.00001 | 0.000467 | 0.886328 | 0.113195 | 0 | 0 | 0 | 0 | 0 |
| 806 | 0.00001 | 0.000481 | 0.882421 | 0.117089 | 0 | 0 | 0 | 0 | 0 |
| 807 | 0.00006 | 0.000571 | 0.884486 | 0.114883 | 0 | 0 | 0 | 0 | 0 |
| 808 | 0.00002 | 0.000414 | 0.881956 | 0.11761 | 0 | 0 | 0 | 0 | 0 |
| 809 | 0 | 0.000469 | 0.883467 | 0.116064 | 0 | 0 | 0 | 0 | 0 |
| 810 | 0.00002 | 0.000607 | 0.881239 | 0.118134 | 0 | 0 | 0 | 0 | 0 |
| 811 | 0.00003 | 0.000524 | 0.884092 | 0.115355 | 0 | 0 | 0 | 0 | 0 |
| 812 | 0.00001 | 0.000402 | 0.886939 | 0.11265 | 0 | 0 | 0 | 0 | 0 |
| 813 | 0.00001 | 0.00049 | 0.886402 | 0.113098 | 0 | 0 | 0 | 0 | 0 |
| 814 | 0.00002 | 0.000677 | 0.885898 | 0.113405 | 0 | 0 | 0 | 0 | 0 |
| 815 | 0 | 0.00018 | 0.882488 | 0.117332 | 0 | 0 | 0 | 0 | 0 |
| 816 | 0.00004 | 0.000441 | 0.885908 | 0.113611 | 0 | 0 | 0 | 0 | 0 |
| 817 | 0.00001 | 0.000498 | 0.88853 | 0.110962 | 0 | 0 | 0 | 0 | 0 |
| 818 | 0.00003 | 0.00049 | 0.880809 | 0.118672 | 0 | 0 | 0 | 0 | 0 |
| 819 | 0.00002 | 0.000338 | 0.880394 | 0.119248 | 0 | 0 | 0 | 0 | 0 |
| 820 | 0 | 0.000429 | 0.883684 | 0.115887 | 0 | 0 | 0 | 0 | 0 |
| 821 | 0.00001 | 0.000538 | 0.886834 | 0.112618 | 0 | 0 | 0 | 0 | 0 |
| 822 | 0.00004 | 0.00056 | 0.884018 | 0.115382 | 0 | 0 | 0 | 0 | 0 |
| 823 | 0.00004 | 0.000466 | 0.8825 | 0.116994 | 0 | 0 | 0 | 0 | 0 |
| 824 | 0.00001 | 0.000543 | 0.885945 | 0.113502 | 0 | 0 | 0 | 0 | 0 |
| 825 | 0 | 0.000461 | 0.884733 | 0.114805 | 0 | 0 | 0 | 0 | 0 |
| 826 | 0.00002 | 0.000359 | 0.885026 | 0.114595 | 0 | 0 | 0 | 0 | 0 |
| 827 | 0.00001 | 0.000629 | 0.882053 | 0.117308 | 0 | 0 | 0 | 0 | 0 |
| 828 | 0.00001 | 0.00033 | 0.884733 | 0.114927 | 0 | 0 | 0 | 0 | 0 |
| 829 | 0 | 0.00044 | 0.882428 | 0.117131 | 0 | 0 | 0 | 0 | 0 |
| 830 | 0.00002 | 0.000503 | 0.88425 | 0.115226 | 0 | 0 | 0 | 0 | 0 |
| 831 | 0.00004 | 0.00055 | 0.879651 | 0.119759 | 0 | 0 | 0 | 0 | 0 |
| 832 | 0.00001 | 0.000459 | 0.883141 | 0.11639 | 0 | 0 | 0 | 0 | 0 |
| 833 | 0.00003 | 0.000442 | 0.88237 | 0.117158 | 0 | 0 | 0 | 0 | 0 |
| 834 | 0.00003 | 0.0005 | 0.882854 | 0.116585 | 0 | 0 | 0 | 0 | 0 |
| 835 | 0.00003 | 0.000499 | 0.87931 | 0.120161 | 0 | 0 | 0 | 0 | 0 |
| 836 | 0.00003 | 0.00047 | 0.882124 | 0.117376 | 0 | 0 | 0 | 0 | 0 |
| 837 | 0.00001 | 0.00045 | 0.887694 | 0.111846 | 0 | 0 | 0 | 0 | 0 |
| 838 | 0.00002 | 0.000497 | 0.882109 | 0.117375 | 0 | 0 | 0 | 0 | 0 |
| 839 | 0 | 0.000371 | 0.882942 | 0.116687 | 0 | 0 | 0 | 0 | 0 |
| 840 | 0.00003 | 0.000358 | 0.883382 | 0.11623 | 0 | 0 | 0 | 0 | 0 |
| 841 | 0.00001 | 0.000622 | 0.887991 | 0.111377 | 0 | 0 | 0 | 0 | 0 |
| 842 | 0.00003 | 0.000299 | 0.880557 | 0.119114 | 0 | 0 | 0 | 0 | 0 |
| 843 | 0.00001 | 0.000575 | 0.884737 | 0.114679 | 0 | 0 | 0 | 0 | 0 |
| 844 | 0.00002 | 0.00045 | 0.884748 | 0.114782 | 0 | 0 | 0 | 0 | 0 |
| 845 | 0 | 0.00043 | 0.881237 | 0.118333 | 0 | 0 | 0 | 0 | 0 |
| 846 | 0.00001 | 0.000353 | 0.88007 | 0.119567 | 0 | 0 | 0 | 0 | 0 |
| 847 | 0.00002 | 0.000529 | 0.881748 | 0.117703 | 0 | 0 | 0 | 0 | 0 |
| 848 | 0.00001 | 0.000541 | 0.885535 | 0.113914 | 0 | 0 | 0 | 0 | 0 |
| 849 | 0.00001 | 0.000492 | 0.88687 | 0.112628 | 0 | 0 | 0 | 0 | 0 |
| 850 | 0.00002 | 0.000397 | 0.883326 | 0.116257 | 0 | 0 | 0 | 0 | 0 |
| 851 | 0 | 0.000522 | 0.882454 | 0.116924 | 0 | 0 | 0 | 0 | 0 |
| 852 | 0.00001 | 0.000411 | 0.881947 | 0.117632 | 0 | 0 | 0 | 0 | 0 |
| 853 | 0.00005 | 0.000351 | 0.880312 | 0.119286 | 0 | 0 | 0 | 0 | 0 |
| 854 | 0.00002 | 0.00037 | 0.880266 | 0.119344 | 0 | 0 | 0 | 0 | 0 |
| 855 | 0 | 0.000509 | 0.882065 | 0.117426 | 0 | 0 | 0 | 0 | 0 |
| 856 | 0.00001 | 0.000569 | 0.878983 | 0.120438 | 0 | 0 | 0 | 0 | 0 |
| 857 | 0.00004 | 0.0004 | 0.881396 | 0.118163 | 0 | 0 | 0 | 0 | 0 |
| 858 | 0.00003 | 0.000529 | 0.882791 | 0.11665 | 0 | 0 | 0 | 0 | 0 |
| 859 | 0.00004 | 0.000501 | 0.88458 | 0.114879 | 0 | 0 | 0 | 0 | 0 |
| 860 | 0.00001 | 0.000582 | 0.882838 | 0.11657 | 0 | 0 | 0 | 0 | 0 |
| 861 | 0.00001 | 0.000559 | 0.884767 | 0.114645 | 0 | 0 | 0 | 0 | 0 |
| 862 | 0 | 0.00045 | 0.883709 | 0.115841 | 0 | 0 | 0 | 0 | 0 |
| 863 | 0.00002 | 0.000658 | 0.882485 | 0.116837 | 0 | 0 | 0 | 0 | 0 |
| 864 | 0.00003 | 0.000533 | 0.882329 | 0.117107 | 0 | 0 | 0 | 0 | 0 |
| 865 | 0.00002 | 0.000391 | 0.884439 | 0.11515 | 0 | 0 | 0 | 0 | 0 |
| 866 | 0.00001 | 0.000342 | 0.884018 | 0.11563 | 0 | 0 | 0 | 0 | 0 |
| 867 | 0.00002 | 0.000479 | 0.881209 | 0.118292 | 0 | 0 | 0 | 0 | 0 |
| 868 | 0.00001 | 0.00045 | 0.877974 | 0.121566 | 0 | 0 | 0 | 0 | 0 |
| 869 | 0 | 0.000398 | 0.88061 | 0.118971 | 0 | 0 | 0 | 0 | 0 |
| 870 | 0.00001 | 0.000331 | 0.882487 | 0.117172 | 0 | 0 | 0 | 0 | 0 |
| 871 | 0.00003 | 0.000429 | 0.887782 | 0.111759 | 0 | 0 | 0 | 0 | 0 |
| 872 | 0.00002 | 0.000508 | 0.881752 | 0.11772 | 0 | 0 | 0 | 0 | 0 |
| 873 | 0.00004 | 0.00056 | 0.882857 | 0.116542 | 0 | 0 | 0 | 0 | 0 |
| 874 | 0.00002 | 0.000483 | 0.882223 | 0.117274 | 0 | 0 | 0 | 0 | 0 |
| 875 | 0.00005 | 0.000532 | 0.885738 | 0.11368 | 0 | 0 | 0 | 0 | 0 |
| 876 | 0 | 0.000567 | 0.883356 | 0.116076 | 0 | 0 | 0 | 0 | 0 |
| 877 | 0.00002 | 0.000452 | 0.884907 | 0.114621 | 0 | 0 | 0 | 0 | 0 |
| 878 | 0.00002 | 0.00052 | 0.88511 | 0.11435 | 0 | 0 | 0 | 0 | 0 |
| 879 | 0.00003 | 0.000418 | 0.882054 | 0.117498 | 0 | 0 | 0 | 0 | 0 |
| 880 | 0.00003 | 0.000399 | 0.884851 | 0.11472 | 0 | 0 | 0 | 0 | 0 |
| 881 | 0.00002 | 0.000412 | 0.880323 | 0.119245 | 0 | 0 | 0 | 0 | 0 |
| 882 | 0.00002 | 0.000602 | 0.880333 | 0.119035 | 0 | 0 | 0 | 0 | 0 |
| 883 | 0.00002 | 0.000489 | 0.882685 | 0.116797 | 0 | 0 | 0 | 0 | 0 |
| 884 | 0.00002 | 0.000489 | 0.879309 | 0.120183 | 0 | 0 | 0 | 0 | 0 |
| 885 | 0.00001 | 0.000482 | 0.886401 | 0.113106 | 0 | 0 | 0 | 0 | 0 |
| 886 | 0.00001 | 0.000495 | 0.883875 | 0.11562 | 0 | 0 | 0 | 0 | 0 |
| 887 | 0.00001 | 0.000462 | 0.883882 | 0.115646 | 0 | 0 | 0 | 0 | 0 |
| 888 | 0.00001 | 0.000471 | 0.878081 | 0.121439 | 0 | 0 | 0 | 0 | 0 |
| 889 | 0.00001 | 0.000517 | 0.876995 | 0.122478 | 0 | 0 | 0 | 0 | 0 |
| 890 | 0.00003 | 0.000488 | 0.885388 | 0.114094 | 0 | 0 | 0 | 0 | 0 |
| 891 | 0.00001 | 0.000529 | 0.880903 | 0.118558 | 0 | 0 | 0 | 0 | 0 |
| 892 | 0.00001 | 0.000418 | 0.882551 | 0.117022 | 0 | 0 | 0 | 0 | 0 |
| 893 | 0.00001 | 0.000528 | 0.883237 | 0.116226 | 0 | 0 | 0 | 0 | 0 |
| 894 | 0.00001 | 0.000543 | 0.882372 | 0.117075 | 0 | 0 | 0 | 0 | 0 |
| 895 | 0.00002 | 0.000471 | 0.888305 | 0.111204 | 0 | 0 | 0 | 0 | 0 |
| 896 | 0.00002 | 0.00049 | 0.885424 | 0.114066 | 0 | 0 | 0 | 0 | 0 |
| 897 | 0.00002 | 0.000449 | 0.884563 | 0.114968 | 0 | 0 | 0 | 0 | 0 |
| 898 | 0.00001 | 0.000251 | 0.88476 | 0.11498 | 0 | 0 | 0 | 0 | 0 |
| 899 | 0.00002 | 0.00049 | 0.88417 | 0.11532 | 0 | 0 | 0 | 0 | 0 |
| 900 | 0.00002 | 0.000545 | 0.882878 | 0.116558 | 0 | 0 | 0 | 0 | 0 |
| 901 | 0 | 0.00054 | 0.890134 | 0.109325 | 0 | 0 | 0 | 0 | 0 |
| 902 | 0.00002 | 0.000351 | 0.882972 | 0.116657 | 0 | 0 | 0 | 0 | 0 |
| 903 | 0.00001 | 0.000529 | 0.883332 | 0.116129 | 0 | 0 | 0 | 0 | 0 |
| 904 | 0.00002 | 0.000531 | 0.885891 | 0.113558 | 0 | 0 | 0 | 0 | 0 |
| 905 | 0.00004 | 0.000479 | 0.883976 | 0.115505 | 0 | 0 | 0 | 0 | 0 |
| 906 | 0 | 0.000431 | 0.884167 | 0.115402 | 0 | 0 | 0 | 0 | 0 |
| 907 | 0.00001 | 0.000442 | 0.886377 | 0.113172 | 0 | 0 | 0 | 0 | 0 |
| 908 | 0.00005 | 0.00059 | 0.886489 | 0.112871 | 0 | 0 | 0 | 0 | 0 |
| 909 | 0 | 0.00045 | 0.883775 | 0.115775 | 0 | 0 | 0 | 0 | 0 |
| 910 | 0.00003 | 0.000513 | 0.883025 | 0.116432 | 0 | 0 | 0 | 0 | 0 |
| 911 | 0.00001 | 0.000369 | 0.88641 | 0.11321 | 0 | 0 | 0 | 0 | 0 |
| 912 | 0.00002 | 0.000331 | 0.880811 | 0.118838 | 0 | 0 | 0 | 0 | 0 |
| 913 | 0.00001 | 0.00068 | 0.885273 | 0.114037 | 0 | 0 | 0 | 0 | 0 |
| 914 | 0.00006 | 0.000428 | 0.886283 | 0.113229 | 0 | 0 | 0 | 0 | 0 |
| 915 | 0.00002 | 0.00042 | 0.880523 | 0.119037 | 0 | 0 | 0 | 0 | 0 |
| 916 | 0.00001 | 0.000491 | 0.88344 | 0.116059 | 0 | 0 | 0 | 0 | 0 |
| 917 | 0.00003 | 0.000527 | 0.884152 | 0.115291 | 0 | 0 | 0 | 0 | 0 |
| 918 | 0 | 0.000399 | 0.885447 | 0.114154 | 0 | 0 | 0 | 0 | 0 |
| 919 | 0 | 0.000599 | 0.880286 | 0.119115 | 0 | 0 | 0 | 0 | 0 |
| 920 | 0.00002 | 0.000408 | 0.887493 | 0.11208 | 0 | 0 | 0 | 0 | 0 |
| 921 | 0.00004 | 0.000407 | 0.882928 | 0.116626 | 0 | 0 | 0 | 0 | 0 |
| 922 | 0.00005 | 0.000471 | 0.886021 | 0.113457 | 0 | 0 | 0 | 0 | 0 |
| 923 | 0.00002 | 0.000481 | 0.88406 | 0.115439 | 0 | 0 | 0 | 0 | 0 |
| 924 | 0.00003 | 0.000359 | 0.883423 | 0.116187 | 0 | 0 | 0 | 0 | 0 |
| 925 | 0.00002 | 0.000359 | 0.883268 | 0.116344 | 0 | 0 | 0 | 0 | 0 |
| 926 | 0.00005 | 0.000478 | 0.884181 | 0.115292 | 0 | 0 | 0 | 0 | 0 |
| 927 | 0.00001 | 0.000379 | 0.8823 | 0.117311 | 0 | 0 | 0 | 0 | 0 |
| 928 | 0.00001 | 0.00044 | 0.880238 | 0.119313 | 0 | 0 | 0 | 0 | 0 |
| 929 | 0 | 0.000443 | 0.87859 | 0.120967 | 0 | 0 | 0 | 0 | 0 |
| 930 | 0.00002 | 0.0005 | 0.886631 | 0.112849 | 0 | 0 | 0 | 0 | 0 |
| 931 | 0 | 0.000463 | 0.886995 | 0.112542 | 0 | 0 | 0 | 0 | 0 |
| 932 | 0 | 0.000441 | 0.883905 | 0.115653 | 0 | 0 | 0 | 0 | 0 |
| 933 | 0.00005 | 0.000418 | 0.888626 | 0.110906 | 0 | 0 | 0 | 0 | 0 |
| 934 | 0.00001 | 0.000419 | 0.880065 | 0.119506 | 0 | 0 | 0 | 0 | 0 |
| 935 | 0.00001 | 0.000617 | 0.88308 | 0.116293 | 0 | 0 | 0 | 0 | 0 |
| 936 | 0.00001 | 0.000537 | 0.885031 | 0.114421 | 0 | 0 | 0 | 0 | 0 |
| 937 | 0.00003 | 0.000522 | 0.888328 | 0.11112 | 0 | 0 | 0 | 0 | 0 |
| 938 | 0.00003 | 0.000409 | 0.881577 | 0.117984 | 0 | 0 | 0 | 0 | 0 |
| 939 | 0.00004 | 0.000442 | 0.886467 | 0.11305 | 0 | 0 | 0 | 0 | 0 |
| 940 | 0.00002 | 0.000418 | 0.880448 | 0.119114 | 0 | 0 | 0 | 0 | 0 |
| 941 | 0 | 0.00037 | 0.883299 | 0.11633 | 0 | 0 | 0 | 0 | 0 |
| 942 | 0.00003 | 0.00051 | 0.884863 | 0.114597 | 0 | 0 | 0 | 0 | 0 |
| 943 | 0.00003 | 0.000499 | 0.88731 | 0.112161 | 0 | 0 | 0 | 0 | 0 |
| 944 | 0.00002 | 0.00037 | 0.88127 | 0.11834 | 0 | 0 | 0 | 0 | 0 |
| 945 | 0 | 0.00043 | 0.878412 | 0.121158 | 0 | 0 | 0 | 0 | 0 |
| 946 | 0.00002 | 0.000353 | 0.885087 | 0.114541 | 0 | 0 | 0 | 0 | 0 |
| 947 | 0.00001 | 0.00042 | 0.880623 | 0.118947 | 0 | 0 | 0 | 0 | 0 |
| 948 | 0.00003 | 0.000469 | 0.884025 | 0.115476 | 0 | 0 | 0 | 0 | 0 |
| 949 | 0.00003 | 0.000399 | 0.888799 | 0.110772 | 0 | 0 | 0 | 0 | 0 |
| 950 | 0.00002 | 0.000378 | 0.885638 | 0.113964 | 0 | 0 | 0 | 0 | 0 |
| 951 | 0.00005 | 0.000612 | 0.886192 | 0.113145 | 0 | 0 | 0 | 0 | 0 |
| 952 | 0.00005 | 0.000438 | 0.885421 | 0.114091 | 0 | 0 | 0 | 0 | 0 |
| 953 | 0.00003 | 0.000451 | 0.882602 | 0.116916 | 0 | 0 | 0 | 0 | 0 |
| 954 | 0.00005 | 0.000429 | 0.88052 | 0.119001 | 0 | 0 | 0 | 0 | 0 |
| 955 | 0 | 0.000479 | 0.885802 | 0.113719 | 0 | 0 | 0 | 0 | 0 |
| 956 | 0.00003 | 0.000562 | 0.884233 | 0.115175 | 0 | 0 | 0 | 0 | 0 |
| 957 | 0 | 0.000641 | 0.881146 | 0.118213 | 0 | 0 | 0 | 0 | 0 |
| 958 | 0.00003 | 0.000449 | 0.885108 | 0.114414 | 0 | 0 | 0 | 0 | 0 |
| 959 | 0.00002 | 0.000441 | 0.878244 | 0.121294 | 0 | 0 | 0 | 0 | 0 |
| 960 | 0.00001 | 0.000629 | 0.883833 | 0.115528 | 0 | 0 | 0 | 0 | 0 |
| 961 | 0.00004 | 0.00056 | 0.882267 | 0.117123 | 0 | 0 | 0 | 0 | 0 |
| 962 | 0.00001 | 0.000466 | 0.884433 | 0.115091 | 0 | 0 | 0 | 0 | 0 |
| 963 | 0.00002 | 0.000421 | 0.884538 | 0.115021 | 0 | 0 | 0 | 0 | 0 |
| 964 | 0.00005 | 0.000452 | 0.886104 | 0.113393 | 0 | 0 | 0 | 0 | 0 |
| 965 | 0.00002 | 0.000538 | 0.886004 | 0.113438 | 0 | 0 | 0 | 0 | 0 |
| 966 | 0 | 0.000481 | 0.886942 | 0.112577 | 0 | 0 | 0 | 0 | 0 |
| 967 | 0.00001 | 0.000449 | 0.886269 | 0.113272 | 0 | 0 | 0 | 0 | 0 |
| 968 | 0.00004 | 0.000477 | 0.882785 | 0.116699 | 0 | 0 | 0 | 0 | 0 |
| 969 | 0.00005 | 0.000391 | 0.883383 | 0.116176 | 0 | 0 | 0 | 0 | 0 |
| 970 | 0.00001 | 0.000478 | 0.883291 | 0.116221 | 0 | 0 | 0 | 0 | 0 |
| 971 | 0.00003 | 0.000473 | 0.888744 | 0.110753 | 0 | 0 | 0 | 0 | 0 |
| 972 | 0.00001 | 0.000542 | 0.880561 | 0.118888 | 0 | 0 | 0 | 0 | 0 |
| 973 | 0.00002 | 0.00049 | 0.882355 | 0.117095 | 0 | 0 | 0 | 0 | 0 |
| 974 | 0.00001 | 0.000392 | 0.882157 | 0.117441 | 0 | 0 | 0 | 0 | 0 |
| 975 | 0.00002 | 0.000469 | 0.883825 | 0.115686 | 0 | 0 | 0 | 0 | 0 |
| 976 | 0.00005 | 0.000441 | 0.883192 | 0.116318 | 0 | 0 | 0 | 0 | 0 |
| 977 | 0.00002 | 0.00043 | 0.882318 | 0.117231 | 0 | 0 | 0 | 0 | 0 |
| 978 | 0.00002 | 0.000521 | 0.884194 | 0.115265 | 0 | 0 | 0 | 0 | 0 |
| 979 | 0 | 0.000389 | 0.883598 | 0.116014 | 0 | 0 | 0 | 0 | 0 |
| 980 | 0.00004 | 0.000392 | 0.886231 | 0.113336 | 0 | 0 | 0 | 0 | 0 |
| 981 | 0 | 0.000358 | 0.884574 | 0.115069 | 0 | 0 | 0 | 0 | 0 |
| 982 | 0 | 0.000477 | 0.881327 | 0.118197 | 0 | 0 | 0 | 0 | 0 |
| 983 | 0.00004 | 0.00049 | 0.880895 | 0.118575 | 0 | 0 | 0 | 0 | 0 |
| 984 | 0 | 0.000532 | 0.884937 | 0.114531 | 0 | 0 | 0 | 0 | 0 |
| 985 | 0.00002 | 0.00049 | 0.884741 | 0.114749 | 0 | 0 | 0 | 0 | 0 |
| 986 | 0 | 0.00053 | 0.886876 | 0.112595 | 0 | 0 | 0 | 0 | 0 |
| 987 | 0.00003 | 0.000372 | 0.884984 | 0.114614 | 0 | 0 | 0 | 0 | 0 |
| 988 | 0.00004 | 0.000575 | 0.88303 | 0.116355 | 0 | 0 | 0 | 0 | 0 |
| 989 | 0.00002 | 0.000411 | 0.878635 | 0.120934 | 0 | 0 | 0 | 0 | 0 |
| 990 | 0.00004 | 0.000539 | 0.882464 | 0.116907 | 0 | 0 | 0 | 0 | 0 |
| 991 | 0.00002 | 0.000599 | 0.885794 | 0.113587 | 0 | 0 | 0 | 0 | 0 |
| 992 | 0.00004 | 0.000472 | 0.884625 | 0.114863 | 0 | 0 | 0 | 0 | 0 |
| 993 | 0.00002 | 0.000469 | 0.887329 | 0.112182 | 0 | 0 | 0 | 0 | 0 |
| 994 | 0 | 0.00034 | 0.888025 | 0.111635 | 0 | 0 | 0 | 0 | 0 |
| 995 | 0.00003 | 0.000419 | 0.883616 | 0.115935 | 0 | 0 | 0 | 0 | 0 |
| 996 | 0.00001 | 0.000389 | 0.88757 | 0.112031 | 0 | 0 | 0 | 0 | 0 |
| 997 | 0.00001 | 0.000619 | 0.880041 | 0.11933 | 0 | 0 | 0 | 0 | 0 |
| 998 | 0.00003 | 0.000351 | 0.884629 | 0.114989 | 0 | 0 | 0 | 0 | 0 |
| 999 | 0 | 0.000444 | 0.885399 | 0.114157 | 0 | 0 | 0 | 0 | 0 |
| 1000 | 0.00003 | 0.00038 | 0.882136 | 0.117453 | 0 | 0 | 0 | 0 | 0 |

| Year 7 | Race |  |  |  |  |  |  |  |  |
| --- | --- | --- | --- | --- | --- | --- | --- | --- | --- |
| Trial | 003.0 | 005.0 | 007.0 | 037.1 | 043.0 | 303.0 | 003.2 | 403.0 | 003.4 |
| 1 | 0.00001 | 0.00033 | 0.893221 | 0.106439 | 0 | 0 | 0 | 0 | 0 |
| 2 | 0.00002 | 0.000221 | 0.888796 | 0.110963 | 0 | 0 | 0 | 0 | 0 |
| 3 | 0 | 0.00013 | 0.893624 | 0.106245 | 0 | 0 | 0 | 0 | 0 |
| 4 | 0.00001 | 0.000189 | 0.896161 | 0.10364 | 0 | 0 | 0 | 0 | 0 |
| 5 | 0.00002 | 0.000231 | 0.892097 | 0.107652 | 0 | 0 | 0 | 0 | 0 |
| 6 | 0.00001 | 0.000201 | 0.89322 | 0.106569 | 0 | 0 | 0 | 0 | 0 |
| 7 | 0.00001 | 0.00013 | 0.893975 | 0.105885 | 0 | 0 | 0 | 0 | 0 |
| 8 | 0.00001 | 0.000159 | 0.894605 | 0.105226 | 0 | 0 | 0 | 0 | 0 |
| 9 | 0.00001 | 0.00017 | 0.892784 | 0.107035 | 0 | 0 | 0 | 0 | 0 |
| 10 | 0 | 0.00022 | 0.891607 | 0.108173 | 0 | 0 | 0 | 0 | 0 |
| 11 | 0.00002 | 0.000141 | 0.894013 | 0.105826 | 0 | 0 | 0 | 0 | 0 |
| 12 | 0.00001 | 0.00016 | 0.891395 | 0.108435 | 0 | 0 | 0 | 0 | 0 |
| 13 | 0.00003 | 0.000299 | 0.890729 | 0.108942 | 0 | 0 | 0 | 0 | 0 |
| 14 | 0.00001 | 0.000247 | 0.887257 | 0.112486 | 0 | 0 | 0 | 0 | 0 |
| 15 | 0 | 0.00012 | 0.889333 | 0.110546 | 0 | 0 | 0 | 0 | 0 |
| 16 | 0.00001 | 0.000229 | 0.892378 | 0.107383 | 0 | 0 | 0 | 0 | 0 |
| 17 | 0.00001 | 0.000261 | 0.891915 | 0.107814 | 0 | 0 | 0 | 0 | 0 |
| 18 | 0.00001 | 0.000272 | 0.89231 | 0.107408 | 0 | 0 | 0 | 0 | 0 |
| 19 | 0.00001 | 0.000178 | 0.892942 | 0.10687 | 0 | 0 | 0 | 0 | 0 |
| 20 | 0.00003 | 0.000239 | 0.890066 | 0.109665 | 0 | 0 | 0 | 0 | 0 |
| 21 | 0.00004 | 0.00014 | 0.891592 | 0.108228 | 0 | 0 | 0 | 0 | 0 |
| 22 | 0 | 0.0003 | 0.889664 | 0.110036 | 0 | 0 | 0 | 0 | 0 |
| 23 | 0.00002 | 0.00029 | 0.893671 | 0.106018 | 0 | 0 | 0 | 0 | 0 |
| 24 | 0 | 0.000189 | 0.891766 | 0.108045 | 0 | 0 | 0 | 0 | 0 |
| 25 | 0.00002 | 0.0001 | 0.894919 | 0.104961 | 0 | 0 | 0 | 0 | 0 |
| 26 | 0 | 0.000208 | 0.89538 | 0.104412 | 0 | 0 | 0 | 0 | 0 |
| 27 | 0.00001 | 0.000269 | 0.892171 | 0.107551 | 0 | 0 | 0 | 0 | 0 |
| 28 | 0.00001 | 0.00015 | 0.893389 | 0.106441 | 0 | 0 | 0 | 0 | 0 |
| 29 | 0.00001 | 0.00014 | 0.893849 | 0.106 | 0 | 0 | 0 | 0 | 0 |
| 30 | 0 | 0.00023 | 0.893138 | 0.106632 | 0 | 0 | 0 | 0 | 0 |
| 31 | 0.00003 | 0.00008 | 0.894401 | 0.105489 | 0 | 0 | 0 | 0 | 0 |
| 32 | 0.00002 | 0.000168 | 0.894778 | 0.105034 | 0 | 0 | 0 | 0 | 0 |
| 33 | 0 | 0.00014 | 0.891962 | 0.107897 | 0 | 0 | 0 | 0 | 0 |
| 34 | 0.00003 | 0.000149 | 0.891795 | 0.108026 | 0 | 0 | 0 | 0 | 0 |
| 35 | 0.00001 | 0.000209 | 0.885892 | 0.11389 | 0 | 0 | 0 | 0 | 0 |
| 36 | 0.00001 | 0.000181 | 0.889158 | 0.110652 | 0 | 0 | 0 | 0 | 0 |
| 37 | 0.00001 | 0.0001 | 0.896345 | 0.103545 | 0 | 0 | 0 | 0 | 0 |
| 38 | 0 | 0.00025 | 0.894443 | 0.105307 | 0 | 0 | 0 | 0 | 0 |
| 39 | 0 | 0.00014 | 0.891248 | 0.108612 | 0 | 0 | 0 | 0 | 0 |
| 40 | 0.00001 | 0.00021 | 0.888307 | 0.111474 | 0 | 0 | 0 | 0 | 0 |
| 41 | 0 | 0.00018 | 0.8921 | 0.10772 | 0 | 0 | 0 | 0 | 0 |
| 42 | 0.00001 | 0.000251 | 0.894396 | 0.105343 | 0 | 0 | 0 | 0 | 0 |
| 43 | 0.00001 | 0.00007 | 0.892049 | 0.10787 | 0 | 0 | 0 | 0 | 0 |
| 44 | 0.00004 | 0.000279 | 0.892115 | 0.107566 | 0 | 0 | 0 | 0 | 0 |
| 45 | 0 | 0.00023 | 0.89033 | 0.109441 | 0 | 0 | 0 | 0 | 0 |
| 46 | 0.00002 | 0.00004 | 0.888461 | 0.111479 | 0 | 0 | 0 | 0 | 0 |
| 47 | 0.00002 | 0.00014 | 0.891149 | 0.10869 | 0 | 0 | 0 | 0 | 0 |
| 48 | 0 | 0.000169 | 0.892193 | 0.107638 | 0 | 0 | 0 | 0 | 0 |
| 49 | 0.00002 | 0.000219 | 0.889603 | 0.110157 | 0 | 0 | 0 | 0 | 0 |
| 50 | 0.00003 | 0.00017 | 0.895287 | 0.104513 | 0 | 0 | 0 | 0 | 0 |
| 51 | 0.00001 | 0.0002 | 0.890908 | 0.108883 | 0 | 0 | 0 | 0 | 0 |
| 52 | 0.00002 | 0.000139 | 0.894831 | 0.10501 | 0 | 0 | 0 | 0 | 0 |
| 53 | 0.00001 | 0.000159 | 0.893539 | 0.106293 | 0 | 0 | 0 | 0 | 0 |
| 54 | 0 | 0.00015 | 0.893372 | 0.106479 | 0 | 0 | 0 | 0 | 0 |
| 55 | 0.00002 | 0.000211 | 0.89397 | 0.105799 | 0 | 0 | 0 | 0 | 0 |
| 56 | 0.00002 | 0.000321 | 0.891876 | 0.107783 | 0 | 0 | 0 | 0 | 0 |
| 57 | 0.00002 | 0.000232 | 0.894021 | 0.105727 | 0 | 0 | 0 | 0 | 0 |
| 58 | 0.00004 | 0.000149 | 0.891324 | 0.108487 | 0 | 0 | 0 | 0 | 0 |
| 59 | 0.00001 | 0.000219 | 0.893631 | 0.106139 | 0 | 0 | 0 | 0 | 0 |
| 60 | 0.00001 | 0.00015 | 0.893321 | 0.106519 | 0 | 0 | 0 | 0 | 0 |
| 61 | 0.00001 | 0.00013 | 0.892428 | 0.107432 | 0 | 0 | 0 | 0 | 0 |
| 62 | 0 | 0.0001 | 0.892232 | 0.107668 | 0 | 0 | 0 | 0 | 0 |
| 63 | 0.00001 | 0.000101 | 0.891878 | 0.108011 | 0 | 0 | 0 | 0 | 0 |
| 64 | 0 | 0.000129 | 0.88953 | 0.110341 | 0 | 0 | 0 | 0 | 0 |
| 65 | 0.00003 | 0.0002 | 0.892197 | 0.107574 | 0 | 0 | 0 | 0 | 0 |
| 66 | 0.00001 | 0.000129 | 0.895737 | 0.104124 | 0 | 0 | 0 | 0 | 0 |
| 67 | 0.00005 | 0.00013 | 0.894199 | 0.105621 | 0 | 0 | 0 | 0 | 0 |
| 68 | 0 | 0.00016 | 0.8922 | 0.10764 | 0 | 0 | 0 | 0 | 0 |
| 69 | 0.00003 | 0.00009 | 0.891959 | 0.107921 | 0 | 0 | 0 | 0 | 0 |
| 70 | 0.00002 | 0.000209 | 0.89247 | 0.107301 | 0 | 0 | 0 | 0 | 0 |
| 71 | 0.00001 | 0.00027 | 0.889931 | 0.109769 | 0 | 0 | 0 | 0 | 0 |
| 72 | 0 | 0.000219 | 0.894982 | 0.104799 | 0 | 0 | 0 | 0 | 0 |
| 73 | 0 | 0.000271 | 0.891449 | 0.10828 | 0 | 0 | 0 | 0 | 0 |
| 74 | 0 | 0.00028 | 0.894832 | 0.104888 | 0 | 0 | 0 | 0 | 0 |
| 75 | 0.00002 | 0.000281 | 0.892531 | 0.107169 | 0 | 0 | 0 | 0 | 0 |
| 76 | 0 | 0.000249 | 0.893279 | 0.106471 | 0 | 0 | 0 | 0 | 0 |
| 77 | 0.00001 | 0.000191 | 0.892533 | 0.107267 | 0 | 0 | 0 | 0 | 0 |
| 78 | 0.00002 | 0.00016 | 0.892872 | 0.106949 | 0 | 0 | 0 | 0 | 0 |
| 79 | 0 | 0.00014 | 0.894526 | 0.105334 | 0 | 0 | 0 | 0 | 0 |
| 80 | 0.00002 | 0.00016 | 0.892476 | 0.107344 | 0 | 0 | 0 | 0 | 0 |
| 81 | 0.00002 | 0.00015 | 0.890122 | 0.109708 | 0 | 0 | 0 | 0 | 0 |
| 82 | 0 | 0.00017 | 0.889792 | 0.110038 | 0 | 0 | 0 | 0 | 0 |
| 83 | 0.00002 | 0.00018 | 0.897449 | 0.102351 | 0 | 0 | 0 | 0 | 0 |
| 84 | 0.00002 | 0.00016 | 0.896298 | 0.103522 | 0 | 0 | 0 | 0 | 0 |
| 85 | 0 | 0.00015 | 0.89229 | 0.10756 | 0 | 0 | 0 | 0 | 0 |
| 86 | 0 | 0.000139 | 0.900297 | 0.099564 | 0 | 0 | 0 | 0 | 0 |
| 87 | 0.00002 | 0.00023 | 0.891704 | 0.108046 | 0 | 0 | 0 | 0 | 0 |
| 88 | 0 | 0.00015 | 0.891324 | 0.108526 | 0 | 0 | 0 | 0 | 0 |
| 89 | 0.00001 | 0.000279 | 0.890297 | 0.109414 | 0 | 0 | 0 | 0 | 0 |
| 90 | 0 | 0.00012 | 0.891992 | 0.107888 | 0 | 0 | 0 | 0 | 0 |
| 91 | 0.00003 | 0.00017 | 0.892875 | 0.106924 | 0 | 0 | 0 | 0 | 0 |
| 92 | 0.00002 | 0.000209 | 0.889219 | 0.110552 | 0 | 0 | 0 | 0 | 0 |
| 93 | 0.00001 | 0.0002 | 0.894678 | 0.105112 | 0 | 0 | 0 | 0 | 0 |
| 94 | 0.00004 | 0.00021 | 0.895622 | 0.104128 | 0 | 0 | 0 | 0 | 0 |
| 95 | 0.00001 | 0.00021 | 0.891479 | 0.108301 | 0 | 0 | 0 | 0 | 0 |
| 96 | 0.00001 | 0.00021 | 0.892397 | 0.107383 | 0 | 0 | 0 | 0 | 0 |
| 97 | 0.00003 | 0.00016 | 0.889625 | 0.110185 | 0 | 0 | 0 | 0 | 0 |
| 98 | 0.00001 | 0.000191 | 0.891575 | 0.108224 | 0 | 0 | 0 | 0 | 0 |
| 99 | 0.00002 | 0.0002 | 0.887188 | 0.112592 | 0 | 0 | 0 | 0 | 0 |
| 100 | 0.00003 | 0.00013 | 0.895531 | 0.104309 | 0 | 0 | 0 | 0 | 0 |
| 101 | 0.00003 | 0.000169 | 0.894124 | 0.105676 | 0 | 0 | 0 | 0 | 0 |
| 102 | 0.00001 | 0.000212 | 0.895124 | 0.104654 | 0 | 0 | 0 | 0 | 0 |
| 103 | 0.00001 | 0.000189 | 0.891367 | 0.108434 | 0 | 0 | 0 | 0 | 0 |
| 104 | 0.00002 | 0.000239 | 0.894615 | 0.105126 | 0 | 0 | 0 | 0 | 0 |
| 105 | 0.00001 | 0.000261 | 0.892858 | 0.106871 | 0 | 0 | 0 | 0 | 0 |
| 106 | 0.00001 | 0.00018 | 0.890563 | 0.109247 | 0 | 0 | 0 | 0 | 0 |
| 107 | 0 | 0.000218 | 0.892492 | 0.10727 | 0 | 0 | 0 | 0 | 0 |
| 108 | 0.00001 | 0.000181 | 0.889356 | 0.110453 | 0 | 0 | 0 | 0 | 0 |
| 109 | 0 | 0.00024 | 0.89392 | 0.10584 | 0 | 0 | 0 | 0 | 0 |
| 110 | 0.00002 | 0.000121 | 0.89432 | 0.105539 | 0 | 0 | 0 | 0 | 0 |
| 111 | 0.00001 | 0.000152 | 0.896338 | 0.103501 | 0 | 0 | 0 | 0 | 0 |
| 112 | 0 | 0.000228 | 0.893442 | 0.106329 | 0 | 0 | 0 | 0 | 0 |
| 113 | 0.00002 | 0.00014 | 0.892103 | 0.107737 | 0 | 0 | 0 | 0 | 0 |
| 114 | 0.00003 | 0.000161 | 0.894981 | 0.104827 | 0 | 0 | 0 | 0 | 0 |
| 115 | 0.00004 | 0.000191 | 0.897666 | 0.102102 | 0 | 0 | 0 | 0 | 0 |
| 116 | 0.00001 | 0.000212 | 0.893681 | 0.106098 | 0 | 0 | 0 | 0 | 0 |
| 117 | 0.00003 | 0.000219 | 0.890938 | 0.108814 | 0 | 0 | 0 | 0 | 0 |
| 118 | 0.00003 | 0.000119 | 0.893777 | 0.106074 | 0 | 0 | 0 | 0 | 0 |
| 119 | 0.00002 | 0.00008 | 0.894178 | 0.105722 | 0 | 0 | 0 | 0 | 0 |
| 120 | 0.00002 | 0.00023 | 0.892563 | 0.107187 | 0 | 0 | 0 | 0 | 0 |
| 121 | 0.00001 | 0.000131 | 0.894108 | 0.105751 | 0 | 0 | 0 | 0 | 0 |
| 122 | 0.00001 | 0.0002 | 0.894483 | 0.105307 | 0 | 0 | 0 | 0 | 0 |
| 123 | 0.00001 | 0.00014 | 0.893121 | 0.106729 | 0 | 0 | 0 | 0 | 0 |
| 124 | 0.00002 | 0.00014 | 0.896213 | 0.103628 | 0 | 0 | 0 | 0 | 0 |
| 125 | 0.00002 | 0.000221 | 0.89261 | 0.107149 | 0 | 0 | 0 | 0 | 0 |
| 126 | 0.00002 | 0.00004 | 0.890799 | 0.109141 | 0 | 0 | 0 | 0 | 0 |
| 127 | 0 | 0.000151 | 0.893794 | 0.106055 | 0 | 0 | 0 | 0 | 0 |
| 128 | 0 | 0.00014 | 0.892388 | 0.107473 | 0 | 0 | 0 | 0 | 0 |
| 129 | 0.00001 | 0.000249 | 0.88962 | 0.110121 | 0 | 0 | 0 | 0 | 0 |
| 130 | 0.00001 | 0.000161 | 0.893233 | 0.106597 | 0 | 0 | 0 | 0 | 0 |
| 131 | 0.00001 | 0.00017 | 0.895064 | 0.104757 | 0 | 0 | 0 | 0 | 0 |
| 132 | 0 | 0.0002 | 0.895861 | 0.103938 | 0 | 0 | 0 | 0 | 0 |
| 133 | 0 | 0.00018 | 0.891772 | 0.108048 | 0 | 0 | 0 | 0 | 0 |
| 134 | 0.00001 | 0.000199 | 0.895493 | 0.104298 | 0 | 0 | 0 | 0 | 0 |
| 135 | 0 | 0.00015 | 0.888006 | 0.111844 | 0 | 0 | 0 | 0 | 0 |
| 136 | 0 | 0.00016 | 0.895781 | 0.104059 | 0 | 0 | 0 | 0 | 0 |
| 137 | 0.00003 | 0.00009 | 0.891815 | 0.108065 | 0 | 0 | 0 | 0 | 0 |
| 138 | 0 | 0.00016 | 0.887958 | 0.111882 | 0 | 0 | 0 | 0 | 0 |
| 139 | 0.00004 | 0.000199 | 0.895829 | 0.103932 | 0 | 0 | 0 | 0 | 0 |
| 140 | 0 | 0.00016 | 0.890684 | 0.109156 | 0 | 0 | 0 | 0 | 0 |
| 141 | 0.00002 | 0.000201 | 0.892926 | 0.106853 | 0 | 0 | 0 | 0 | 0 |
| 142 | 0.00003 | 0.00014 | 0.887687 | 0.112142 | 0 | 0 | 0 | 0 | 0 |
| 143 | 0.00002 | 0.000198 | 0.890727 | 0.109055 | 0 | 0 | 0 | 0 | 0 |
| 144 | 0.00001 | 0.000348 | 0.891511 | 0.108131 | 0 | 0 | 0 | 0 | 0 |
| 145 | 0.00003 | 0.000181 | 0.893821 | 0.105968 | 0 | 0 | 0 | 0 | 0 |
| 146 | 0.00001 | 0.000212 | 0.895673 | 0.104104 | 0 | 0 | 0 | 0 | 0 |
| 147 | 0.00001 | 0.00018 | 0.891343 | 0.108467 | 0 | 0 | 0 | 0 | 0 |
| 148 | 0.00003 | 0.000151 | 0.891833 | 0.107986 | 0 | 0 | 0 | 0 | 0 |
| 149 | 0.00004 | 0.00008 | 0.892785 | 0.107095 | 0 | 0 | 0 | 0 | 0 |
| 150 | 0.00001 | 0.000199 | 0.8921 | 0.107691 | 0 | 0 | 0 | 0 | 0 |
| 151 | 0.00001 | 0.000289 | 0.892272 | 0.107428 | 0 | 0 | 0 | 0 | 0 |
| 152 | 0.00002 | 0.00013 | 0.890076 | 0.109774 | 0 | 0 | 0 | 0 | 0 |
| 153 | 0.00002 | 0.000181 | 0.893876 | 0.105924 | 0 | 0 | 0 | 0 | 0 |
| 154 | 0.00002 | 0.000241 | 0.889443 | 0.110296 | 0 | 0 | 0 | 0 | 0 |
| 155 | 0.00001 | 0.00008 | 0.890771 | 0.109139 | 0 | 0 | 0 | 0 | 0 |
| 156 | 0.00001 | 0.000239 | 0.890427 | 0.109324 | 0 | 0 | 0 | 0 | 0 |
| 157 | 0.00004 | 0.00019 | 0.891387 | 0.108383 | 0 | 0 | 0 | 0 | 0 |
| 158 | 0.00002 | 0.00019 | 0.893195 | 0.106595 | 0 | 0 | 0 | 0 | 0 |
| 159 | 0.00002 | 0.000111 | 0.891127 | 0.108742 | 0 | 0 | 0 | 0 | 0 |
| 160 | 0.00001 | 0.00024 | 0.895512 | 0.104238 | 0 | 0 | 0 | 0 | 0 |
| 161 | 0.00001 | 0.00015 | 0.893769 | 0.106071 | 0 | 0 | 0 | 0 | 0 |
| 162 | 0.00001 | 0.00027 | 0.890487 | 0.109234 | 0 | 0 | 0 | 0 | 0 |
| 163 | 0.00001 | 0.000149 | 0.892763 | 0.107078 | 0 | 0 | 0 | 0 | 0 |
| 164 | 0.00001 | 0.000199 | 0.88864 | 0.111151 | 0 | 0 | 0 | 0 | 0 |
| 165 | 0 | 0.00012 | 0.89718 | 0.1027 | 0 | 0 | 0 | 0 | 0 |
| 166 | 0.00001 | 0.00015 | 0.891656 | 0.108184 | 0 | 0 | 0 | 0 | 0 |
| 167 | 0 | 0.000201 | 0.89166 | 0.10814 | 0 | 0 | 0 | 0 | 0 |
| 168 | 0.00003 | 0.000249 | 0.89205 | 0.107671 | 0 | 0 | 0 | 0 | 0 |
| 169 | 0.00001 | 0.00021 | 0.889807 | 0.109973 | 0 | 0 | 0 | 0 | 0 |
| 170 | 0.00001 | 0.000181 | 0.891496 | 0.108314 | 0 | 0 | 0 | 0 | 0 |
| 171 | 0.00001 | 0.00015 | 0.894286 | 0.105554 | 0 | 0 | 0 | 0 | 0 |
| 172 | 0 | 0.00017 | 0.891441 | 0.108389 | 0 | 0 | 0 | 0 | 0 |
| 173 | 0 | 0.00025 | 0.893887 | 0.105863 | 0 | 0 | 0 | 0 | 0 |
| 174 | 0 | 0.00015 | 0.890637 | 0.109213 | 0 | 0 | 0 | 0 | 0 |
| 175 | 0 | 0.000161 | 0.890782 | 0.109057 | 0 | 0 | 0 | 0 | 0 |
| 176 | 0.00001 | 0.000198 | 0.897983 | 0.101808 | 0 | 0 | 0 | 0 | 0 |
| 177 | 0 | 0.000171 | 0.892893 | 0.106936 | 0 | 0 | 0 | 0 | 0 |
| 178 | 0.00001 | 0.00014 | 0.890507 | 0.109343 | 0 | 0 | 0 | 0 | 0 |
| 179 | 0.00002 | 0.00025 | 0.890926 | 0.108804 | 0 | 0 | 0 | 0 | 0 |
| 180 | 0.00001 | 0.000208 | 0.893422 | 0.10636 | 0 | 0 | 0 | 0 | 0 |
| 181 | 0.00003 | 0.00011 | 0.892701 | 0.107159 | 0 | 0 | 0 | 0 | 0 |
| 182 | 0.00001 | 0.00025 | 0.892251 | 0.107489 | 0 | 0 | 0 | 0 | 0 |
| 183 | 0.00002 | 0.000119 | 0.892696 | 0.107164 | 0 | 0 | 0 | 0 | 0 |
| 184 | 0.00001 | 0.000159 | 0.89777 | 0.102061 | 0 | 0 | 0 | 0 | 0 |
| 185 | 0 | 0.00021 | 0.89535 | 0.104441 | 0 | 0 | 0 | 0 | 0 |
| 186 | 0 | 0.000212 | 0.891015 | 0.108773 | 0 | 0 | 0 | 0 | 0 |
| 187 | 0.00001 | 0.000159 | 0.899082 | 0.100748 | 0 | 0 | 0 | 0 | 0 |
| 188 | 0.00001 | 0.0002 | 0.89065 | 0.10914 | 0 | 0 | 0 | 0 | 0 |
| 189 | 0.00001 | 0.00019 | 0.892553 | 0.107248 | 0 | 0 | 0 | 0 | 0 |
| 190 | 0.00003 | 0.00014 | 0.893107 | 0.106722 | 0 | 0 | 0 | 0 | 0 |
| 191 | 0 | 0.000279 | 0.891848 | 0.107872 | 0 | 0 | 0 | 0 | 0 |
| 192 | 0.00001 | 0.00018 | 0.892005 | 0.107805 | 0 | 0 | 0 | 0 | 0 |
| 193 | 0.00001 | 0.00016 | 0.889602 | 0.110228 | 0 | 0 | 0 | 0 | 0 |
| 194 | 0.00002 | 0.00022 | 0.889597 | 0.110163 | 0 | 0 | 0 | 0 | 0 |
| 195 | 0.00002 | 0.0001 | 0.8914 | 0.108479 | 0 | 0 | 0 | 0 | 0 |
| 196 | 0.00004 | 0.000229 | 0.890423 | 0.109309 | 0 | 0 | 0 | 0 | 0 |
| 197 | 0.00004 | 0.000169 | 0.897523 | 0.102267 | 0 | 0 | 0 | 0 | 0 |
| 198 | 0.00001 | 0.000242 | 0.891152 | 0.108556 | 0 | 0 | 0 | 0 | 0 |
| 199 | 0 | 0.000231 | 0.895789 | 0.10398 | 0 | 0 | 0 | 0 | 0 |
| 200 | 0.00002 | 0.000211 | 0.890609 | 0.109159 | 0 | 0 | 0 | 0 | 0 |
| 201 | 0.00002 | 0.000249 | 0.89275 | 0.106981 | 0 | 0 | 0 | 0 | 0 |
| 202 | 0.00001 | 0.00029 | 0.893345 | 0.106355 | 0 | 0 | 0 | 0 | 0 |
| 203 | 0.00002 | 0.00011 | 0.896403 | 0.103468 | 0 | 0 | 0 | 0 | 0 |
| 204 | 0.00001 | 0.00012 | 0.893199 | 0.106671 | 0 | 0 | 0 | 0 | 0 |
| 205 | 0.00001 | 0.0001 | 0.887775 | 0.112115 | 0 | 0 | 0 | 0 | 0 |
| 206 | 0 | 0.00016 | 0.889986 | 0.109854 | 0 | 0 | 0 | 0 | 0 |
| 207 | 0.00001 | 0.00028 | 0.895853 | 0.103857 | 0 | 0 | 0 | 0 | 0 |
| 208 | 0.00002 | 0.00015 | 0.896127 | 0.103702 | 0 | 0 | 0 | 0 | 0 |
| 209 | 0.00003 | 0.00015 | 0.891595 | 0.108225 | 0 | 0 | 0 | 0 | 0 |
| 210 | 0.00002 | 0.00017 | 0.89257 | 0.10724 | 0 | 0 | 0 | 0 | 0 |
| 211 | 0.00001 | 0.000211 | 0.890846 | 0.108934 | 0 | 0 | 0 | 0 | 0 |
| 212 | 0.00004 | 0.00015 | 0.890938 | 0.108872 | 0 | 0 | 0 | 0 | 0 |
| 213 | 0.00001 | 0.000191 | 0.894618 | 0.105181 | 0 | 0 | 0 | 0 | 0 |
| 214 | 0.00004 | 0.00014 | 0.893758 | 0.106062 | 0 | 0 | 0 | 0 | 0 |
| 215 | 0.00002 | 0.00008 | 0.892714 | 0.107187 | 0 | 0 | 0 | 0 | 0 |
| 216 | 0.00001 | 0.00027 | 0.894701 | 0.10502 | 0 | 0 | 0 | 0 | 0 |
| 217 | 0.00001 | 0.00018 | 0.892422 | 0.107387 | 0 | 0 | 0 | 0 | 0 |
| 218 | 0 | 0.000109 | 0.894354 | 0.105537 | 0 | 0 | 0 | 0 | 0 |
| 219 | 0.00003 | 0.00019 | 0.899257 | 0.100523 | 0 | 0 | 0 | 0 | 0 |
| 220 | 0.00003 | 0.000241 | 0.893937 | 0.105792 | 0 | 0 | 0 | 0 | 0 |
| 221 | 0.00003 | 0.00022 | 0.891788 | 0.107962 | 0 | 0 | 0 | 0 | 0 |
| 222 | 0 | 0.00018 | 0.892313 | 0.107507 | 0 | 0 | 0 | 0 | 0 |
| 223 | 0.00001 | 0.00019 | 0.89258 | 0.10722 | 0 | 0 | 0 | 0 | 0 |
| 224 | 0.00001 | 0.00019 | 0.890533 | 0.109267 | 0 | 0 | 0 | 0 | 0 |
| 225 | 0.00001 | 0.000219 | 0.895843 | 0.103928 | 0 | 0 | 0 | 0 | 0 |
| 226 | 0 | 0.000071 | 0.894324 | 0.105605 | 0 | 0 | 0 | 0 | 0 |
| 227 | 0.00001 | 0.00024 | 0.892083 | 0.107668 | 0 | 0 | 0 | 0 | 0 |
| 228 | 0.00001 | 0.00019 | 0.894985 | 0.104815 | 0 | 0 | 0 | 0 | 0 |
| 229 | 0 | 0.00024 | 0.89426 | 0.1055 | 0 | 0 | 0 | 0 | 0 |
| 230 | 0 | 0.000121 | 0.889281 | 0.110598 | 0 | 0 | 0 | 0 | 0 |
| 231 | 0 | 0.000282 | 0.893066 | 0.106652 | 0 | 0 | 0 | 0 | 0 |
| 232 | 0.00001 | 0.000199 | 0.892245 | 0.107546 | 0 | 0 | 0 | 0 | 0 |
| 233 | 0.00002 | 0.000252 | 0.894615 | 0.105113 | 0 | 0 | 0 | 0 | 0 |
| 234 | 0.00002 | 0.00016 | 0.891324 | 0.108496 | 0 | 0 | 0 | 0 | 0 |
| 235 | 0 | 0.000167 | 0.89653 | 0.103303 | 0 | 0 | 0 | 0 | 0 |
| 236 | 0.00002 | 0.00009 | 0.891113 | 0.108777 | 0 | 0 | 0 | 0 | 0 |
| 237 | 0.00001 | 0.000121 | 0.893406 | 0.106463 | 0 | 0 | 0 | 0 | 0 |
| 238 | 0.00002 | 0.00024 | 0.888371 | 0.11137 | 0 | 0 | 0 | 0 | 0 |
| 239 | 0.00001 | 0.000131 | 0.889844 | 0.110015 | 0 | 0 | 0 | 0 | 0 |
| 240 | 0.00004 | 0.00016 | 0.893807 | 0.105993 | 0 | 0 | 0 | 0 | 0 |
| 241 | 0.00001 | 0.000161 | 0.894875 | 0.104953 | 0 | 0 | 0 | 0 | 0 |
| 242 | 0 | 0.00017 | 0.892187 | 0.107643 | 0 | 0 | 0 | 0 | 0 |
| 243 | 0 | 0.00009 | 0.897317 | 0.102593 | 0 | 0 | 0 | 0 | 0 |
| 244 | 0.00002 | 0.000169 | 0.891407 | 0.108403 | 0 | 0 | 0 | 0 | 0 |
| 245 | 0.00002 | 0.000271 | 0.890452 | 0.109246 | 0 | 0 | 0 | 0 | 0 |
| 246 | 0.00002 | 0.000211 | 0.889961 | 0.109809 | 0 | 0 | 0 | 0 | 0 |
| 247 | 0.00002 | 0.000279 | 0.887522 | 0.11218 | 0 | 0 | 0 | 0 | 0 |
| 248 | 0.00002 | 0.000249 | 0.894567 | 0.105164 | 0 | 0 | 0 | 0 | 0 |
| 249 | 0.00002 | 0.000241 | 0.894713 | 0.105026 | 0 | 0 | 0 | 0 | 0 |
| 250 | 0.00003 | 0.00021 | 0.888378 | 0.111383 | 0 | 0 | 0 | 0 | 0 |
| 251 | 0.00002 | 0.000199 | 0.89175 | 0.108031 | 0 | 0 | 0 | 0 | 0 |
| 252 | 0.00001 | 0.000099 | 0.894971 | 0.10492 | 0 | 0 | 0 | 0 | 0 |
| 253 | 0.00001 | 0.000201 | 0.892711 | 0.107078 | 0 | 0 | 0 | 0 | 0 |
| 254 | 0.00002 | 0.00021 | 0.890726 | 0.109045 | 0 | 0 | 0 | 0 | 0 |
| 255 | 0 | 0.000182 | 0.893326 | 0.106492 | 0 | 0 | 0 | 0 | 0 |
| 256 | 0 | 0.000229 | 0.890337 | 0.109434 | 0 | 0 | 0 | 0 | 0 |
| 257 | 0 | 0.00023 | 0.891461 | 0.108308 | 0 | 0 | 0 | 0 | 0 |
| 258 | 0.00002 | 0.000171 | 0.888462 | 0.111347 | 0 | 0 | 0 | 0 | 0 |
| 259 | 0.00001 | 0.000169 | 0.891769 | 0.108052 | 0 | 0 | 0 | 0 | 0 |
| 260 | 0 | 0.00022 | 0.894821 | 0.104959 | 0 | 0 | 0 | 0 | 0 |
| 261 | 0.00003 | 0.00012 | 0.895764 | 0.104086 | 0 | 0 | 0 | 0 | 0 |
| 262 | 0.00001 | 0.00021 | 0.891863 | 0.107917 | 0 | 0 | 0 | 0 | 0 |
| 263 | 0.00001 | 0.00015 | 0.891168 | 0.108672 | 0 | 0 | 0 | 0 | 0 |
| 264 | 0 | 0.00012 | 0.889445 | 0.110435 | 0 | 0 | 0 | 0 | 0 |
| 265 | 0.00001 | 0.00014 | 0.892074 | 0.107776 | 0 | 0 | 0 | 0 | 0 |
| 266 | 0.00003 | 0.000251 | 0.894594 | 0.105125 | 0 | 0 | 0 | 0 | 0 |
| 267 | 0.00001 | 0.00014 | 0.892633 | 0.107217 | 0 | 0 | 0 | 0 | 0 |
| 268 | 0.00001 | 0.00015 | 0.894528 | 0.105312 | 0 | 0 | 0 | 0 | 0 |
| 269 | 0.00003 | 0.0001 | 0.893497 | 0.106373 | 0 | 0 | 0 | 0 | 0 |
| 270 | 0.00003 | 0.000219 | 0.892437 | 0.107314 | 0 | 0 | 0 | 0 | 0 |
| 271 | 0.00002 | 0.000171 | 0.892114 | 0.107695 | 0 | 0 | 0 | 0 | 0 |
| 272 | 0 | 0.00014 | 0.890148 | 0.109712 | 0 | 0 | 0 | 0 | 0 |
| 273 | 0 | 0.00015 | 0.896488 | 0.103362 | 0 | 0 | 0 | 0 | 0 |
| 274 | 0.00001 | 0.00025 | 0.896259 | 0.103481 | 0 | 0 | 0 | 0 | 0 |
| 275 | 0 | 0.00016 | 0.893609 | 0.106231 | 0 | 0 | 0 | 0 | 0 |
| 276 | 0 | 0.00023 | 0.891922 | 0.107849 | 0 | 0 | 0 | 0 | 0 |
| 277 | 0.00001 | 0.000249 | 0.887589 | 0.112151 | 0 | 0 | 0 | 0 | 0 |
| 278 | 0 | 0.000191 | 0.891664 | 0.108146 | 0 | 0 | 0 | 0 | 0 |
| 279 | 0 | 0.000161 | 0.891164 | 0.108676 | 0 | 0 | 0 | 0 | 0 |
| 280 | 0 | 0.00014 | 0.889699 | 0.110161 | 0 | 0 | 0 | 0 | 0 |
| 281 | 0.00001 | 0.000199 | 0.890609 | 0.109182 | 0 | 0 | 0 | 0 | 0 |
| 282 | 0.00001 | 0.000251 | 0.89261 | 0.107129 | 0 | 0 | 0 | 0 | 0 |
| 283 | 0.00002 | 0.000211 | 0.895414 | 0.104355 | 0 | 0 | 0 | 0 | 0 |
| 284 | 0 | 0.00013 | 0.897025 | 0.102845 | 0 | 0 | 0 | 0 | 0 |
| 285 | 0.00003 | 0.00015 | 0.889215 | 0.110605 | 0 | 0 | 0 | 0 | 0 |
| 286 | 0 | 0.00017 | 0.896382 | 0.103448 | 0 | 0 | 0 | 0 | 0 |
| 287 | 0.00001 | 0.00019 | 0.892947 | 0.106853 | 0 | 0 | 0 | 0 | 0 |
| 288 | 0.00004 | 0.000169 | 0.8913 | 0.108492 | 0 | 0 | 0 | 0 | 0 |
| 289 | 0 | 0.000181 | 0.895231 | 0.104588 | 0 | 0 | 0 | 0 | 0 |
| 290 | 0 | 0.0002 | 0.888817 | 0.110984 | 0 | 0 | 0 | 0 | 0 |
| 291 | 0.00001 | 0.00014 | 0.891378 | 0.108472 | 0 | 0 | 0 | 0 | 0 |
| 292 | 0.00001 | 0.00013 | 0.891313 | 0.108547 | 0 | 0 | 0 | 0 | 0 |
| 293 | 0.00001 | 0.000201 | 0.886857 | 0.112932 | 0 | 0 | 0 | 0 | 0 |
| 294 | 0.00002 | 0.000269 | 0.890359 | 0.109353 | 0 | 0 | 0 | 0 | 0 |
| 295 | 0 | 0.00009 | 0.895457 | 0.104453 | 0 | 0 | 0 | 0 | 0 |
| 296 | 0.00005 | 0.000141 | 0.891246 | 0.108563 | 0 | 0 | 0 | 0 | 0 |
| 297 | 0.00001 | 0.000229 | 0.890519 | 0.109241 | 0 | 0 | 0 | 0 | 0 |
| 298 | 0.00001 | 0.0002 | 0.892187 | 0.107603 | 0 | 0 | 0 | 0 | 0 |
| 299 | 0 | 0.00016 | 0.885909 | 0.113931 | 0 | 0 | 0 | 0 | 0 |
| 300 | 0 | 0.000191 | 0.88986 | 0.109949 | 0 | 0 | 0 | 0 | 0 |
| 301 | 0.00001 | 0.00022 | 0.891452 | 0.108318 | 0 | 0 | 0 | 0 | 0 |
| 302 | 0 | 0.000109 | 0.891184 | 0.108707 | 0 | 0 | 0 | 0 | 0 |
| 303 | 0.00001 | 0.000209 | 0.889532 | 0.110249 | 0 | 0 | 0 | 0 | 0 |
| 304 | 0.00001 | 0.00015 | 0.890028 | 0.109813 | 0 | 0 | 0 | 0 | 0 |
| 305 | 0.00002 | 0.000229 | 0.89448 | 0.10527 | 0 | 0 | 0 | 0 | 0 |
| 306 | 0.00001 | 0.0003 | 0.894875 | 0.104815 | 0 | 0 | 0 | 0 | 0 |
| 307 | 0.00001 | 0.000267 | 0.893565 | 0.106158 | 0 | 0 | 0 | 0 | 0 |
| 308 | 0 | 0.000201 | 0.895122 | 0.104677 | 0 | 0 | 0 | 0 | 0 |
| 309 | 0.00002 | 0.00015 | 0.89396 | 0.10587 | 0 | 0 | 0 | 0 | 0 |
| 310 | 0.00002 | 0.000269 | 0.892778 | 0.106933 | 0 | 0 | 0 | 0 | 0 |
| 311 | 0 | 0.00017 | 0.890658 | 0.109171 | 0 | 0 | 0 | 0 | 0 |
| 312 | 0 | 0.00019 | 0.887545 | 0.112265 | 0 | 0 | 0 | 0 | 0 |
| 313 | 0.00001 | 0.00024 | 0.8924 | 0.10735 | 0 | 0 | 0 | 0 | 0 |
| 314 | 0.00002 | 0.00007 | 0.890995 | 0.108915 | 0 | 0 | 0 | 0 | 0 |
| 315 | 0.00001 | 0.00016 | 0.88904 | 0.11079 | 0 | 0 | 0 | 0 | 0 |
| 316 | 0.00002 | 0.00013 | 0.892889 | 0.10696 | 0 | 0 | 0 | 0 | 0 |
| 317 | 0.00001 | 0.00023 | 0.891406 | 0.108354 | 0 | 0 | 0 | 0 | 0 |
| 318 | 0.00001 | 0.00017 | 0.887212 | 0.112598 | 0 | 0 | 0 | 0 | 0 |
| 319 | 0.00002 | 0.00014 | 0.890038 | 0.109802 | 0 | 0 | 0 | 0 | 0 |
| 320 | 0.00002 | 0.00022 | 0.891812 | 0.107947 | 0 | 0 | 0 | 0 | 0 |
| 321 | 0.00002 | 0.00018 | 0.890533 | 0.109267 | 0 | 0 | 0 | 0 | 0 |
| 322 | 0.00001 | 0.00019 | 0.893458 | 0.106341 | 0 | 0 | 0 | 0 | 0 |
| 323 | 0.00002 | 0.000209 | 0.891952 | 0.107819 | 0 | 0 | 0 | 0 | 0 |
| 324 | 0.00001 | 0.00013 | 0.893039 | 0.106821 | 0 | 0 | 0 | 0 | 0 |
| 325 | 0.00003 | 0.00016 | 0.894286 | 0.105523 | 0 | 0 | 0 | 0 | 0 |
| 326 | 0.00001 | 0.000141 | 0.893344 | 0.106506 | 0 | 0 | 0 | 0 | 0 |
| 327 | 0.00001 | 0.000252 | 0.892816 | 0.106921 | 0 | 0 | 0 | 0 | 0 |
| 328 | 0.00002 | 0.000181 | 0.893956 | 0.105844 | 0 | 0 | 0 | 0 | 0 |
| 329 | 0.00001 | 0.000181 | 0.890476 | 0.109333 | 0 | 0 | 0 | 0 | 0 |
| 330 | 0 | 0.000159 | 0.892762 | 0.107079 | 0 | 0 | 0 | 0 | 0 |
| 331 | 0.00001 | 0.000181 | 0.892471 | 0.107338 | 0 | 0 | 0 | 0 | 0 |
| 332 | 0.00002 | 0.00017 | 0.889809 | 0.11 | 0 | 0 | 0 | 0 | 0 |
| 333 | 0.00001 | 0.000241 | 0.888016 | 0.111733 | 0 | 0 | 0 | 0 | 0 |
| 334 | 0.00001 | 0.00016 | 0.894153 | 0.105677 | 0 | 0 | 0 | 0 | 0 |
| 335 | 0.00001 | 0.00018 | 0.893632 | 0.106178 | 0 | 0 | 0 | 0 | 0 |
| 336 | 0.00004 | 0.000181 | 0.888207 | 0.111571 | 0 | 0 | 0 | 0 | 0 |
| 337 | 0.00001 | 0.000181 | 0.89444 | 0.105369 | 0 | 0 | 0 | 0 | 0 |
| 338 | 0.00003 | 0.000152 | 0.895519 | 0.104299 | 0 | 0 | 0 | 0 | 0 |
| 339 | 0 | 0.000161 | 0.893487 | 0.106352 | 0 | 0 | 0 | 0 | 0 |
| 340 | 0.00002 | 0.000169 | 0.889465 | 0.110346 | 0 | 0 | 0 | 0 | 0 |
| 341 | 0.00002 | 0.000199 | 0.888609 | 0.111172 | 0 | 0 | 0 | 0 | 0 |
| 342 | 0.00002 | 0.00013 | 0.887678 | 0.112172 | 0 | 0 | 0 | 0 | 0 |
| 343 | 0 | 0.000191 | 0.891859 | 0.107951 | 0 | 0 | 0 | 0 | 0 |
| 344 | 0 | 0.00011 | 0.893641 | 0.106249 | 0 | 0 | 0 | 0 | 0 |
| 345 | 0.00003 | 0.00024 | 0.895371 | 0.104359 | 0 | 0 | 0 | 0 | 0 |
| 346 | 0.00001 | 0.0002 | 0.897274 | 0.102516 | 0 | 0 | 0 | 0 | 0 |
| 347 | 0 | 0.0002 | 0.891271 | 0.108529 | 0 | 0 | 0 | 0 | 0 |
| 348 | 0 | 0.000159 | 0.892635 | 0.107206 | 0 | 0 | 0 | 0 | 0 |
| 349 | 0 | 0.0002 | 0.891564 | 0.108236 | 0 | 0 | 0 | 0 | 0 |
| 350 | 0.00001 | 0.000179 | 0.892804 | 0.107008 | 0 | 0 | 0 | 0 | 0 |
| 351 | 0 | 0.000249 | 0.88982 | 0.109931 | 0 | 0 | 0 | 0 | 0 |
| 352 | 0 | 0.00021 | 0.894094 | 0.105696 | 0 | 0 | 0 | 0 | 0 |
| 353 | 0.00002 | 0.00021 | 0.893541 | 0.106229 | 0 | 0 | 0 | 0 | 0 |
| 354 | 0.00001 | 0.00011 | 0.888638 | 0.111243 | 0 | 0 | 0 | 0 | 0 |
| 355 | 0.00002 | 0.000191 | 0.895317 | 0.104472 | 0 | 0 | 0 | 0 | 0 |
| 356 | 0.00002 | 0.0001 | 0.893935 | 0.105945 | 0 | 0 | 0 | 0 | 0 |
| 357 | 0.00002 | 0.000231 | 0.886363 | 0.113387 | 0 | 0 | 0 | 0 | 0 |
| 358 | 0.00001 | 0.000129 | 0.894479 | 0.105382 | 0 | 0 | 0 | 0 | 0 |
| 359 | 0.00002 | 0.00017 | 0.892499 | 0.107311 | 0 | 0 | 0 | 0 | 0 |
| 360 | 0.00001 | 0.000189 | 0.893739 | 0.106062 | 0 | 0 | 0 | 0 | 0 |
| 361 | 0.00002 | 0.00016 | 0.890151 | 0.109669 | 0 | 0 | 0 | 0 | 0 |
| 362 | 0.00003 | 0.000269 | 0.893555 | 0.106145 | 0 | 0 | 0 | 0 | 0 |
| 363 | 0 | 0.000189 | 0.891665 | 0.108146 | 0 | 0 | 0 | 0 | 0 |
| 364 | 0 | 0.000208 | 0.895746 | 0.104046 | 0 | 0 | 0 | 0 | 0 |
| 365 | 0.00003 | 0.000231 | 0.892155 | 0.107584 | 0 | 0 | 0 | 0 | 0 |
| 366 | 0.00007 | 0.00007 | 0.892739 | 0.107122 | 0 | 0 | 0 | 0 | 0 |
| 367 | 0.00004 | 0.00019 | 0.890207 | 0.109563 | 0 | 0 | 0 | 0 | 0 |
| 368 | 0.00002 | 0.000171 | 0.893709 | 0.1061 | 0 | 0 | 0 | 0 | 0 |
| 369 | 0 | 0.00021 | 0.890851 | 0.108939 | 0 | 0 | 0 | 0 | 0 |
| 370 | 0 | 0.000229 | 0.891236 | 0.108535 | 0 | 0 | 0 | 0 | 0 |
| 371 | 0.00004 | 0.00019 | 0.891102 | 0.108658 | 0 | 0 | 0 | 0 | 0 |
| 372 | 0 | 0.000259 | 0.887076 | 0.112665 | 0 | 0 | 0 | 0 | 0 |
| 373 | 0.00004 | 0.00018 | 0.889456 | 0.110325 | 0 | 0 | 0 | 0 | 0 |
| 374 | 0.00003 | 0.00012 | 0.894087 | 0.105762 | 0 | 0 | 0 | 0 | 0 |
| 375 | 0 | 0.00019 | 0.889266 | 0.110544 | 0 | 0 | 0 | 0 | 0 |
| 376 | 0.00002 | 0.000199 | 0.891968 | 0.107813 | 0 | 0 | 0 | 0 | 0 |
| 377 | 0.00001 | 0.00021 | 0.889584 | 0.110196 | 0 | 0 | 0 | 0 | 0 |
| 378 | 0.00001 | 0.0002 | 0.892984 | 0.106805 | 0 | 0 | 0 | 0 | 0 |
| 379 | 0.00003 | 0.00005 | 0.896059 | 0.10386 | 0 | 0 | 0 | 0 | 0 |
| 380 | 0 | 0.00012 | 0.889443 | 0.110437 | 0 | 0 | 0 | 0 | 0 |
| 381 | 0.00001 | 0.0002 | 0.892876 | 0.106914 | 0 | 0 | 0 | 0 | 0 |
| 382 | 0.00003 | 0.000159 | 0.891786 | 0.108025 | 0 | 0 | 0 | 0 | 0 |
| 383 | 0.00001 | 0.000201 | 0.892387 | 0.107402 | 0 | 0 | 0 | 0 | 0 |
| 384 | 0 | 0.000189 | 0.888007 | 0.111804 | 0 | 0 | 0 | 0 | 0 |
| 385 | 0.00002 | 0.00016 | 0.894713 | 0.105108 | 0 | 0 | 0 | 0 | 0 |
| 386 | 0.00001 | 0.00015 | 0.890879 | 0.108962 | 0 | 0 | 0 | 0 | 0 |
| 387 | 0.00001 | 0.000181 | 0.892349 | 0.10746 | 0 | 0 | 0 | 0 | 0 |
| 388 | 0.00002 | 0.000258 | 0.8963 | 0.103422 | 0 | 0 | 0 | 0 | 0 |
| 389 | 0 | 0.00019 | 0.893966 | 0.105844 | 0 | 0 | 0 | 0 | 0 |
| 390 | 0 | 0.000221 | 0.889975 | 0.109805 | 0 | 0 | 0 | 0 | 0 |
| 391 | 0.00001 | 0.000199 | 0.885135 | 0.114656 | 0 | 0 | 0 | 0 | 0 |
| 392 | 0 | 0.000232 | 0.894416 | 0.105352 | 0 | 0 | 0 | 0 | 0 |
| 393 | 0 | 0.000169 | 0.890759 | 0.109073 | 0 | 0 | 0 | 0 | 0 |
| 394 | 0.00001 | 0.00015 | 0.895249 | 0.104592 | 0 | 0 | 0 | 0 | 0 |
| 395 | 0 | 0.000119 | 0.89682 | 0.10306 | 0 | 0 | 0 | 0 | 0 |
| 396 | 0.00001 | 0.00016 | 0.894499 | 0.105331 | 0 | 0 | 0 | 0 | 0 |
| 397 | 0.00002 | 0.000239 | 0.892419 | 0.107322 | 0 | 0 | 0 | 0 | 0 |
| 398 | 0 | 0.0002 | 0.892241 | 0.107559 | 0 | 0 | 0 | 0 | 0 |
| 399 | 0.00001 | 0.0002 | 0.891442 | 0.108348 | 0 | 0 | 0 | 0 | 0 |
| 400 | 0 | 0.000171 | 0.894644 | 0.105185 | 0 | 0 | 0 | 0 | 0 |
| 401 | 0.00001 | 0.0002 | 0.890931 | 0.108858 | 0 | 0 | 0 | 0 | 0 |
| 402 | 0.00002 | 0.00019 | 0.89134 | 0.108449 | 0 | 0 | 0 | 0 | 0 |
| 403 | 0 | 0.00017 | 0.893841 | 0.105989 | 0 | 0 | 0 | 0 | 0 |
| 404 | 0.00001 | 0.000229 | 0.890774 | 0.108986 | 0 | 0 | 0 | 0 | 0 |
| 405 | 0.00002 | 0.000191 | 0.89487 | 0.104919 | 0 | 0 | 0 | 0 | 0 |
| 406 | 0 | 0.000199 | 0.891832 | 0.107968 | 0 | 0 | 0 | 0 | 0 |
| 407 | 0.00001 | 0.000379 | 0.892965 | 0.106646 | 0 | 0 | 0 | 0 | 0 |
| 408 | 0.00002 | 0.00025 | 0.89051 | 0.10922 | 0 | 0 | 0 | 0 | 0 |
| 409 | 0 | 0.000251 | 0.888855 | 0.110894 | 0 | 0 | 0 | 0 | 0 |
| 410 | 0 | 0.00014 | 0.889715 | 0.110145 | 0 | 0 | 0 | 0 | 0 |
| 411 | 0.00001 | 0.00015 | 0.892732 | 0.107108 | 0 | 0 | 0 | 0 | 0 |
| 412 | 0.00003 | 0.000211 | 0.890471 | 0.109288 | 0 | 0 | 0 | 0 | 0 |
| 413 | 0 | 0.00019 | 0.891157 | 0.108653 | 0 | 0 | 0 | 0 | 0 |
| 414 | 0.00003 | 0.00021 | 0.891679 | 0.108081 | 0 | 0 | 0 | 0 | 0 |
| 415 | 0.00002 | 0.000201 | 0.889869 | 0.109909 | 0 | 0 | 0 | 0 | 0 |
| 416 | 0.00002 | 0.000299 | 0.893331 | 0.10635 | 0 | 0 | 0 | 0 | 0 |
| 417 | 0 | 0.00015 | 0.892536 | 0.107313 | 0 | 0 | 0 | 0 | 0 |
| 418 | 0 | 0.000149 | 0.89561 | 0.104241 | 0 | 0 | 0 | 0 | 0 |
| 419 | 0.00001 | 0.000201 | 0.889872 | 0.109917 | 0 | 0 | 0 | 0 | 0 |
| 420 | 0.00003 | 0.00016 | 0.896742 | 0.103068 | 0 | 0 | 0 | 0 | 0 |
| 421 | 0 | 0.00018 | 0.891323 | 0.108497 | 0 | 0 | 0 | 0 | 0 |
| 422 | 0 | 0.000261 | 0.8934 | 0.10634 | 0 | 0 | 0 | 0 | 0 |
| 423 | 0 | 0.00016 | 0.895381 | 0.104459 | 0 | 0 | 0 | 0 | 0 |
| 424 | 0 | 0.000161 | 0.89491 | 0.10493 | 0 | 0 | 0 | 0 | 0 |
| 425 | 0.00001 | 0.00027 | 0.890002 | 0.109718 | 0 | 0 | 0 | 0 | 0 |
| 426 | 0 | 0.000212 | 0.888667 | 0.111121 | 0 | 0 | 0 | 0 | 0 |
| 427 | 0 | 0.00021 | 0.894552 | 0.105238 | 0 | 0 | 0 | 0 | 0 |
| 428 | 0.00001 | 0.00015 | 0.896926 | 0.102914 | 0 | 0 | 0 | 0 | 0 |
| 429 | 0.00002 | 0.000151 | 0.894593 | 0.105236 | 0 | 0 | 0 | 0 | 0 |
| 430 | 0 | 0.000359 | 0.893436 | 0.106205 | 0 | 0 | 0 | 0 | 0 |
| 431 | 0.00002 | 0.00013 | 0.893632 | 0.106218 | 0 | 0 | 0 | 0 | 0 |
| 432 | 0.00001 | 0.0002 | 0.886559 | 0.113231 | 0 | 0 | 0 | 0 | 0 |
| 433 | 0 | 0.00013 | 0.891652 | 0.108218 | 0 | 0 | 0 | 0 | 0 |
| 434 | 0.00001 | 0.00013 | 0.893777 | 0.106083 | 0 | 0 | 0 | 0 | 0 |
| 435 | 0.00002 | 0.000189 | 0.892542 | 0.10725 | 0 | 0 | 0 | 0 | 0 |
| 436 | 0.00001 | 0.00013 | 0.889884 | 0.109976 | 0 | 0 | 0 | 0 | 0 |
| 437 | 0 | 0.00017 | 0.892867 | 0.106963 | 0 | 0 | 0 | 0 | 0 |
| 438 | 0.00002 | 0.00026 | 0.892812 | 0.106908 | 0 | 0 | 0 | 0 | 0 |
| 439 | 0.00001 | 0.000189 | 0.892515 | 0.107286 | 0 | 0 | 0 | 0 | 0 |
| 440 | 0 | 0.000101 | 0.89229 | 0.10761 | 0 | 0 | 0 | 0 | 0 |
| 441 | 0.00002 | 0.00019 | 0.891122 | 0.108668 | 0 | 0 | 0 | 0 | 0 |
| 442 | 0.00001 | 0.000129 | 0.891577 | 0.108283 | 0 | 0 | 0 | 0 | 0 |
| 443 | 0.00004 | 0.000121 | 0.893576 | 0.106263 | 0 | 0 | 0 | 0 | 0 |
| 444 | 0.00003 | 0.00022 | 0.88929 | 0.110459 | 0 | 0 | 0 | 0 | 0 |
| 445 | 0 | 0.000221 | 0.889288 | 0.110492 | 0 | 0 | 0 | 0 | 0 |
| 446 | 0 | 0.00006 | 0.893061 | 0.106879 | 0 | 0 | 0 | 0 | 0 |
| 447 | 0 | 0.000141 | 0.895551 | 0.104308 | 0 | 0 | 0 | 0 | 0 |
| 448 | 0.00003 | 0.00037 | 0.891429 | 0.108172 | 0 | 0 | 0 | 0 | 0 |
| 449 | 0.00002 | 0.00014 | 0.894493 | 0.105347 | 0 | 0 | 0 | 0 | 0 |
| 450 | 0.00002 | 0.000131 | 0.891364 | 0.108485 | 0 | 0 | 0 | 0 | 0 |
| 451 | 0.00002 | 0.0002 | 0.896728 | 0.103052 | 0 | 0 | 0 | 0 | 0 |
| 452 | 0.00001 | 0.000169 | 0.891769 | 0.108052 | 0 | 0 | 0 | 0 | 0 |
| 453 | 0.00002 | 0.000199 | 0.8924 | 0.107381 | 0 | 0 | 0 | 0 | 0 |
| 454 | 0.00001 | 0.000139 | 0.894991 | 0.10486 | 0 | 0 | 0 | 0 | 0 |
| 455 | 0.00005 | 0.000099 | 0.898138 | 0.101713 | 0 | 0 | 0 | 0 | 0 |
| 456 | 0.00003 | 0.000161 | 0.891081 | 0.108728 | 0 | 0 | 0 | 0 | 0 |
| 457 | 0.00002 | 0.000229 | 0.890952 | 0.108798 | 0 | 0 | 0 | 0 | 0 |
| 458 | 0.00003 | 0.000121 | 0.893021 | 0.106828 | 0 | 0 | 0 | 0 | 0 |
| 459 | 0 | 0.0001 | 0.888075 | 0.111825 | 0 | 0 | 0 | 0 | 0 |
| 460 | 0.00003 | 0.000219 | 0.888399 | 0.111352 | 0 | 0 | 0 | 0 | 0 |
| 461 | 0.00002 | 0.00015 | 0.892274 | 0.107556 | 0 | 0 | 0 | 0 | 0 |
| 462 | 0.00002 | 0.00009 | 0.898683 | 0.101207 | 0 | 0 | 0 | 0 | 0 |
| 463 | 0.00001 | 0.00009 | 0.891455 | 0.108445 | 0 | 0 | 0 | 0 | 0 |
| 464 | 0 | 0.00013 | 0.893099 | 0.10674 | 0 | 0 | 0 | 0 | 0 |
| 465 | 0 | 0.00016 | 0.893501 | 0.106339 | 0 | 0 | 0 | 0 | 0 |
| 466 | 0 | 0.000149 | 0.888932 | 0.110919 | 0 | 0 | 0 | 0 | 0 |
| 467 | 0.00001 | 0.00007 | 0.887798 | 0.112122 | 0 | 0 | 0 | 0 | 0 |
| 468 | 0.00001 | 0.00019 | 0.893638 | 0.106162 | 0 | 0 | 0 | 0 | 0 |
| 469 | 0.00001 | 0.00012 | 0.892042 | 0.107828 | 0 | 0 | 0 | 0 | 0 |
| 470 | 0.00002 | 0.00012 | 0.894341 | 0.105518 | 0 | 0 | 0 | 0 | 0 |
| 471 | 0.00001 | 0.000161 | 0.894521 | 0.105308 | 0 | 0 | 0 | 0 | 0 |
| 472 | 0.00002 | 0.00012 | 0.89242 | 0.10744 | 0 | 0 | 0 | 0 | 0 |
| 473 | 0.00001 | 0.00018 | 0.891842 | 0.107968 | 0 | 0 | 0 | 0 | 0 |
| 474 | 0.00001 | 0.000139 | 0.891944 | 0.107907 | 0 | 0 | 0 | 0 | 0 |
| 475 | 0.00002 | 0.000131 | 0.891518 | 0.108331 | 0 | 0 | 0 | 0 | 0 |
| 476 | 0 | 0.000152 | 0.88654 | 0.113309 | 0 | 0 | 0 | 0 | 0 |
| 477 | 0.00001 | 0.00015 | 0.892252 | 0.107588 | 0 | 0 | 0 | 0 | 0 |
| 478 | 0.00002 | 0.000238 | 0.892328 | 0.107414 | 0 | 0 | 0 | 0 | 0 |
| 479 | 0.00002 | 0.00016 | 0.888787 | 0.111033 | 0 | 0 | 0 | 0 | 0 |
| 480 | 0 | 0.00026 | 0.888898 | 0.110842 | 0 | 0 | 0 | 0 | 0 |
| 481 | 0 | 0.000141 | 0.897994 | 0.101865 | 0 | 0 | 0 | 0 | 0 |
| 482 | 0 | 0.000129 | 0.888124 | 0.111747 | 0 | 0 | 0 | 0 | 0 |
| 483 | 0 | 0.00013 | 0.893438 | 0.106432 | 0 | 0 | 0 | 0 | 0 |
| 484 | 0.00001 | 0.00013 | 0.886363 | 0.113496 | 0 | 0 | 0 | 0 | 0 |
| 485 | 0.00001 | 0.000151 | 0.898129 | 0.101711 | 0 | 0 | 0 | 0 | 0 |
| 486 | 0.00001 | 0.00018 | 0.891535 | 0.108276 | 0 | 0 | 0 | 0 | 0 |
| 487 | 0.00001 | 0.000179 | 0.894593 | 0.105218 | 0 | 0 | 0 | 0 | 0 |
| 488 | 0 | 0.00018 | 0.892203 | 0.107617 | 0 | 0 | 0 | 0 | 0 |
| 489 | 0.00003 | 0.000218 | 0.895619 | 0.104133 | 0 | 0 | 0 | 0 | 0 |
| 490 | 0.00001 | 0.00025 | 0.895415 | 0.104326 | 0 | 0 | 0 | 0 | 0 |
| 491 | 0.00002 | 0.000199 | 0.891352 | 0.108429 | 0 | 0 | 0 | 0 | 0 |
| 492 | 0.00005 | 0.00018 | 0.888417 | 0.111353 | 0 | 0 | 0 | 0 | 0 |
| 493 | 0.00001 | 0.000291 | 0.890677 | 0.109022 | 0 | 0 | 0 | 0 | 0 |
| 494 | 0.00002 | 0.000199 | 0.892345 | 0.107436 | 0 | 0 | 0 | 0 | 0 |
| 495 | 0.00001 | 0.000181 | 0.892279 | 0.10753 | 0 | 0 | 0 | 0 | 0 |
| 496 | 0 | 0.00018 | 0.889759 | 0.110052 | 0 | 0 | 0 | 0 | 0 |
| 497 | 0.00003 | 0.000249 | 0.89152 | 0.108201 | 0 | 0 | 0 | 0 | 0 |
| 498 | 0.00002 | 0.000197 | 0.897469 | 0.102314 | 0 | 0 | 0 | 0 | 0 |
| 499 | 0.00001 | 0.00023 | 0.891197 | 0.108563 | 0 | 0 | 0 | 0 | 0 |
| 500 | 0.00002 | 0.00015 | 0.894615 | 0.105215 | 0 | 0 | 0 | 0 | 0 |
| 501 | 0.00001 | 0.00021 | 0.893057 | 0.106723 | 0 | 0 | 0 | 0 | 0 |
| 502 | 0 | 0.00029 | 0.892585 | 0.107125 | 0 | 0 | 0 | 0 | 0 |
| 503 | 0 | 0.00013 | 0.891541 | 0.108329 | 0 | 0 | 0 | 0 | 0 |
| 504 | 0 | 0.00012 | 0.894474 | 0.105406 | 0 | 0 | 0 | 0 | 0 |
| 505 | 0 | 0.0002 | 0.891046 | 0.108744 | 0 | 0 | 0 | 0 | 0 |
| 506 | 0 | 0.00017 | 0.893192 | 0.106638 | 0 | 0 | 0 | 0 | 0 |
| 507 | 0 | 0.00027 | 0.891987 | 0.107743 | 0 | 0 | 0 | 0 | 0 |
| 508 | 0.00001 | 0.000259 | 0.891701 | 0.10803 | 0 | 0 | 0 | 0 | 0 |
| 509 | 0.00001 | 0.00011 | 0.891761 | 0.108119 | 0 | 0 | 0 | 0 | 0 |
| 510 | 0.00001 | 0.000198 | 0.89272 | 0.107072 | 0 | 0 | 0 | 0 | 0 |
| 511 | 0.00002 | 0.00012 | 0.893409 | 0.106451 | 0 | 0 | 0 | 0 | 0 |
| 512 | 0.00001 | 0.00016 | 0.889204 | 0.110626 | 0 | 0 | 0 | 0 | 0 |
| 513 | 0.00001 | 0.00016 | 0.892554 | 0.107276 | 0 | 0 | 0 | 0 | 0 |
| 514 | 0.00001 | 0.00009 | 0.892123 | 0.107778 | 0 | 0 | 0 | 0 | 0 |
| 515 | 0.00002 | 0.00012 | 0.89525 | 0.104611 | 0 | 0 | 0 | 0 | 0 |
| 516 | 0.00001 | 0.00023 | 0.886699 | 0.113061 | 0 | 0 | 0 | 0 | 0 |
| 517 | 0.00002 | 0.00028 | 0.888802 | 0.110898 | 0 | 0 | 0 | 0 | 0 |
| 518 | 0.00001 | 0.000209 | 0.893754 | 0.106027 | 0 | 0 | 0 | 0 | 0 |
| 519 | 0.00001 | 0.00021 | 0.889327 | 0.110453 | 0 | 0 | 0 | 0 | 0 |
| 520 | 0.00003 | 0.0001 | 0.893795 | 0.106076 | 0 | 0 | 0 | 0 | 0 |
| 521 | 0 | 0.00022 | 0.890236 | 0.109544 | 0 | 0 | 0 | 0 | 0 |
| 522 | 0.00001 | 0.000219 | 0.894402 | 0.105369 | 0 | 0 | 0 | 0 | 0 |
| 523 | 0 | 0.000129 | 0.892618 | 0.107252 | 0 | 0 | 0 | 0 | 0 |
| 524 | 0.00001 | 0.00009 | 0.891255 | 0.108646 | 0 | 0 | 0 | 0 | 0 |
| 525 | 0.00001 | 0.000219 | 0.888244 | 0.111527 | 0 | 0 | 0 | 0 | 0 |
| 526 | 0.00001 | 0.00014 | 0.890752 | 0.109098 | 0 | 0 | 0 | 0 | 0 |
| 527 | 0.00003 | 0.000171 | 0.893367 | 0.106432 | 0 | 0 | 0 | 0 | 0 |
| 528 | 0.00003 | 0.00015 | 0.889132 | 0.110688 | 0 | 0 | 0 | 0 | 0 |
| 529 | 0 | 0.000181 | 0.888473 | 0.111346 | 0 | 0 | 0 | 0 | 0 |
| 530 | 0.00002 | 0.000251 | 0.888337 | 0.111392 | 0 | 0 | 0 | 0 | 0 |
| 531 | 0.00001 | 0.000201 | 0.891646 | 0.108144 | 0 | 0 | 0 | 0 | 0 |
| 532 | 0.00001 | 0.00015 | 0.897926 | 0.101914 | 0 | 0 | 0 | 0 | 0 |
| 533 | 0.00001 | 0.0002 | 0.890588 | 0.109202 | 0 | 0 | 0 | 0 | 0 |
| 534 | 0.00002 | 0.000222 | 0.887062 | 0.112696 | 0 | 0 | 0 | 0 | 0 |
| 535 | 0.00002 | 0.00025 | 0.896267 | 0.103464 | 0 | 0 | 0 | 0 | 0 |
| 536 | 0.00001 | 0.00012 | 0.89574 | 0.10413 | 0 | 0 | 0 | 0 | 0 |
| 537 | 0.00002 | 0.0001 | 0.891884 | 0.107996 | 0 | 0 | 0 | 0 | 0 |
| 538 | 0.00002 | 0.000249 | 0.888814 | 0.110917 | 0 | 0 | 0 | 0 | 0 |
| 539 | 0.00001 | 0.000301 | 0.888408 | 0.111281 | 0 | 0 | 0 | 0 | 0 |
| 540 | 0 | 0.000411 | 0.88877 | 0.110819 | 0 | 0 | 0 | 0 | 0 |
| 541 | 0.00003 | 0.00017 | 0.893264 | 0.106536 | 0 | 0 | 0 | 0 | 0 |
| 542 | 0.00002 | 0.0002 | 0.890688 | 0.109092 | 0 | 0 | 0 | 0 | 0 |
| 543 | 0.00001 | 0.00011 | 0.897001 | 0.102879 | 0 | 0 | 0 | 0 | 0 |
| 544 | 0.00001 | 0.00017 | 0.896396 | 0.103425 | 0 | 0 | 0 | 0 | 0 |
| 545 | 0.00004 | 0.00019 | 0.88713 | 0.11264 | 0 | 0 | 0 | 0 | 0 |
| 546 | 0.00001 | 0.0003 | 0.895906 | 0.103784 | 0 | 0 | 0 | 0 | 0 |
| 547 | 0.00003 | 0.00024 | 0.891681 | 0.108049 | 0 | 0 | 0 | 0 | 0 |
| 548 | 0.00003 | 0.00015 | 0.89369 | 0.10613 | 0 | 0 | 0 | 0 | 0 |
| 549 | 0.00003 | 0.00017 | 0.888278 | 0.111522 | 0 | 0 | 0 | 0 | 0 |
| 550 | 0.00001 | 0.000211 | 0.893275 | 0.106504 | 0 | 0 | 0 | 0 | 0 |
| 551 | 0.00005 | 0.000229 | 0.891834 | 0.107887 | 0 | 0 | 0 | 0 | 0 |
| 552 | 0 | 0.00012 | 0.890576 | 0.109304 | 0 | 0 | 0 | 0 | 0 |
| 553 | 0 | 0.000221 | 0.891238 | 0.108542 | 0 | 0 | 0 | 0 | 0 |
| 554 | 0.00004 | 0.000201 | 0.896164 | 0.103595 | 0 | 0 | 0 | 0 | 0 |
| 555 | 0.00001 | 0.00011 | 0.890931 | 0.108949 | 0 | 0 | 0 | 0 | 0 |
| 556 | 0.00001 | 0.000149 | 0.89636 | 0.10348 | 0 | 0 | 0 | 0 | 0 |
| 557 | 0.00001 | 0.0002 | 0.890982 | 0.108808 | 0 | 0 | 0 | 0 | 0 |
| 558 | 0 | 0.00015 | 0.891715 | 0.108135 | 0 | 0 | 0 | 0 | 0 |
| 559 | 0.00003 | 0.00013 | 0.892304 | 0.107536 | 0 | 0 | 0 | 0 | 0 |
| 560 | 0.00002 | 0.00017 | 0.893252 | 0.106558 | 0 | 0 | 0 | 0 | 0 |
| 561 | 0 | 0.00019 | 0.891052 | 0.108758 | 0 | 0 | 0 | 0 | 0 |
| 562 | 0.00003 | 0.00019 | 0.888004 | 0.111776 | 0 | 0 | 0 | 0 | 0 |
| 563 | 0.00003 | 0.00027 | 0.889367 | 0.110333 | 0 | 0 | 0 | 0 | 0 |
| 564 | 0 | 0.000181 | 0.895214 | 0.104605 | 0 | 0 | 0 | 0 | 0 |
| 565 | 0 | 0.000321 | 0.88736 | 0.11232 | 0 | 0 | 0 | 0 | 0 |
| 566 | 0.00001 | 0.00018 | 0.891789 | 0.108021 | 0 | 0 | 0 | 0 | 0 |
| 567 | 0 | 0.0001 | 0.890216 | 0.109684 | 0 | 0 | 0 | 0 | 0 |
| 568 | 0 | 0.000251 | 0.8922 | 0.10755 | 0 | 0 | 0 | 0 | 0 |
| 569 | 0.00002 | 0.00019 | 0.894729 | 0.105061 | 0 | 0 | 0 | 0 | 0 |
| 570 | 0.00006 | 0.000201 | 0.88759 | 0.112148 | 0 | 0 | 0 | 0 | 0 |
| 571 | 0.00001 | 0.00028 | 0.892386 | 0.107324 | 0 | 0 | 0 | 0 | 0 |
| 572 | 0.00004 | 0.000229 | 0.892897 | 0.106834 | 0 | 0 | 0 | 0 | 0 |
| 573 | 0.00001 | 0.000251 | 0.893314 | 0.106425 | 0 | 0 | 0 | 0 | 0 |
| 574 | 0 | 0.00018 | 0.893081 | 0.106739 | 0 | 0 | 0 | 0 | 0 |
| 575 | 0.00002 | 0.00013 | 0.892873 | 0.106977 | 0 | 0 | 0 | 0 | 0 |
| 576 | 0 | 0.00015 | 0.897122 | 0.102728 | 0 | 0 | 0 | 0 | 0 |
| 577 | 0.00001 | 0.00018 | 0.888578 | 0.111232 | 0 | 0 | 0 | 0 | 0 |
| 578 | 0.00002 | 0.00016 | 0.892379 | 0.10744 | 0 | 0 | 0 | 0 | 0 |
| 579 | 0 | 0.00018 | 0.890824 | 0.108996 | 0 | 0 | 0 | 0 | 0 |
| 580 | 0 | 0.00015 | 0.896082 | 0.103768 | 0 | 0 | 0 | 0 | 0 |
| 581 | 0.00001 | 0.00023 | 0.890481 | 0.109279 | 0 | 0 | 0 | 0 | 0 |
| 582 | 0 | 0.000161 | 0.891695 | 0.108144 | 0 | 0 | 0 | 0 | 0 |
| 583 | 0 | 0.00018 | 0.888577 | 0.111243 | 0 | 0 | 0 | 0 | 0 |
| 584 | 0 | 0.00009 | 0.88934 | 0.11057 | 0 | 0 | 0 | 0 | 0 |
| 585 | 0 | 0.000169 | 0.890396 | 0.109435 | 0 | 0 | 0 | 0 | 0 |
| 586 | 0.00005 | 0.00019 | 0.890505 | 0.109256 | 0 | 0 | 0 | 0 | 0 |
| 587 | 0.00002 | 0.00025 | 0.891864 | 0.107866 | 0 | 0 | 0 | 0 | 0 |
| 588 | 0.00002 | 0.000149 | 0.894094 | 0.105736 | 0 | 0 | 0 | 0 | 0 |
| 589 | 0 | 0.00011 | 0.892895 | 0.106994 | 0 | 0 | 0 | 0 | 0 |
| 590 | 0.00005 | 0.00014 | 0.889669 | 0.110141 | 0 | 0 | 0 | 0 | 0 |
| 591 | 0.00002 | 0.000192 | 0.890713 | 0.109075 | 0 | 0 | 0 | 0 | 0 |
| 592 | 0 | 0.000211 | 0.892611 | 0.107178 | 0 | 0 | 0 | 0 | 0 |
| 593 | 0.00001 | 0.00014 | 0.896957 | 0.102892 | 0 | 0 | 0 | 0 | 0 |
| 594 | 0.00002 | 0.000121 | 0.89178 | 0.108079 | 0 | 0 | 0 | 0 | 0 |
| 595 | 0.00001 | 0.000231 | 0.8926 | 0.10716 | 0 | 0 | 0 | 0 | 0 |
| 596 | 0.00001 | 0.000139 | 0.89374 | 0.106111 | 0 | 0 | 0 | 0 | 0 |
| 597 | 0.00003 | 0.00015 | 0.890209 | 0.109611 | 0 | 0 | 0 | 0 | 0 |
| 598 | 0.00002 | 0.00015 | 0.891956 | 0.107875 | 0 | 0 | 0 | 0 | 0 |
| 599 | 0 | 0.000091 | 0.893917 | 0.105993 | 0 | 0 | 0 | 0 | 0 |
| 600 | 0.00002 | 0.000159 | 0.890696 | 0.109124 | 0 | 0 | 0 | 0 | 0 |
| 601 | 0.00003 | 0.00017 | 0.892396 | 0.107404 | 0 | 0 | 0 | 0 | 0 |
| 602 | 0 | 0.00011 | 0.894952 | 0.104938 | 0 | 0 | 0 | 0 | 0 |
| 603 | 0.00001 | 0.00011 | 0.89187 | 0.10801 | 0 | 0 | 0 | 0 | 0 |
| 604 | 0 | 0.00019 | 0.892299 | 0.107512 | 0 | 0 | 0 | 0 | 0 |
| 605 | 0.00002 | 0.000189 | 0.892729 | 0.107062 | 0 | 0 | 0 | 0 | 0 |
| 606 | 0 | 0.000272 | 0.895413 | 0.104315 | 0 | 0 | 0 | 0 | 0 |
| 607 | 0.00003 | 0.000242 | 0.895887 | 0.10384 | 0 | 0 | 0 | 0 | 0 |
| 608 | 0.00001 | 0.000109 | 0.891388 | 0.108493 | 0 | 0 | 0 | 0 | 0 |
| 609 | 0.00003 | 0.000169 | 0.895696 | 0.104105 | 0 | 0 | 0 | 0 | 0 |
| 610 | 0.00002 | 0.000101 | 0.891151 | 0.108729 | 0 | 0 | 0 | 0 | 0 |
| 611 | 0.00002 | 0.00013 | 0.894184 | 0.105665 | 0 | 0 | 0 | 0 | 0 |
| 612 | 0.00002 | 0.000119 | 0.887124 | 0.112736 | 0 | 0 | 0 | 0 | 0 |
| 613 | 0.00001 | 0.00024 | 0.888376 | 0.111374 | 0 | 0 | 0 | 0 | 0 |
| 614 | 0.00002 | 0.00009 | 0.89732 | 0.102571 | 0 | 0 | 0 | 0 | 0 |
| 615 | 0 | 0.00015 | 0.893375 | 0.106475 | 0 | 0 | 0 | 0 | 0 |
| 616 | 0 | 0.000251 | 0.89291 | 0.106839 | 0 | 0 | 0 | 0 | 0 |
| 617 | 0.00001 | 0.00015 | 0.891894 | 0.107946 | 0 | 0 | 0 | 0 | 0 |
| 618 | 0 | 0.00009 | 0.888563 | 0.111347 | 0 | 0 | 0 | 0 | 0 |
| 619 | 0.00004 | 0.000179 | 0.894171 | 0.10561 | 0 | 0 | 0 | 0 | 0 |
| 620 | 0.00002 | 0.00016 | 0.890103 | 0.109718 | 0 | 0 | 0 | 0 | 0 |
| 621 | 0.00001 | 0.00004 | 0.894224 | 0.105726 | 0 | 0 | 0 | 0 | 0 |
| 622 | 0.00002 | 0.000232 | 0.887465 | 0.112282 | 0 | 0 | 0 | 0 | 0 |
| 623 | 0.00001 | 0.00015 | 0.89106 | 0.108781 | 0 | 0 | 0 | 0 | 0 |
| 624 | 0 | 0.000262 | 0.890832 | 0.108906 | 0 | 0 | 0 | 0 | 0 |
| 625 | 0 | 0.000252 | 0.893167 | 0.106571 | 0 | 0 | 0 | 0 | 0 |
| 626 | 0.00001 | 0.000229 | 0.889698 | 0.110063 | 0 | 0 | 0 | 0 | 0 |
| 627 | 0.00001 | 0.00008 | 0.892912 | 0.106998 | 0 | 0 | 0 | 0 | 0 |
| 628 | 0.00001 | 0.000299 | 0.891003 | 0.108688 | 0 | 0 | 0 | 0 | 0 |
| 629 | 0 | 0.000211 | 0.892361 | 0.107428 | 0 | 0 | 0 | 0 | 0 |
| 630 | 0.00002 | 0.000141 | 0.890081 | 0.109758 | 0 | 0 | 0 | 0 | 0 |
| 631 | 0 | 0.00014 | 0.891073 | 0.108787 | 0 | 0 | 0 | 0 | 0 |
| 632 | 0.00002 | 0.00007 | 0.891822 | 0.108088 | 0 | 0 | 0 | 0 | 0 |
| 633 | 0 | 0.00013 | 0.89247 | 0.1074 | 0 | 0 | 0 | 0 | 0 |
| 634 | 0.00003 | 0.00019 | 0.890697 | 0.109083 | 0 | 0 | 0 | 0 | 0 |
| 635 | 0.00003 | 0.000199 | 0.894601 | 0.105171 | 0 | 0 | 0 | 0 | 0 |
| 636 | 0.00001 | 0.000181 | 0.890196 | 0.109612 | 0 | 0 | 0 | 0 | 0 |
| 637 | 0 | 0.00009 | 0.892852 | 0.107058 | 0 | 0 | 0 | 0 | 0 |
| 638 | 0.00003 | 0.000211 | 0.89172 | 0.108039 | 0 | 0 | 0 | 0 | 0 |
| 639 | 0.00001 | 0.000131 | 0.889406 | 0.110454 | 0 | 0 | 0 | 0 | 0 |
| 640 | 0 | 0.00018 | 0.887579 | 0.112241 | 0 | 0 | 0 | 0 | 0 |
| 641 | 0.00002 | 0.000161 | 0.893719 | 0.1061 | 0 | 0 | 0 | 0 | 0 |
| 642 | 0.00001 | 0.00023 | 0.895362 | 0.104398 | 0 | 0 | 0 | 0 | 0 |
| 643 | 0.00002 | 0.00011 | 0.891616 | 0.108253 | 0 | 0 | 0 | 0 | 0 |
| 644 | 0 | 0.0002 | 0.895099 | 0.104701 | 0 | 0 | 0 | 0 | 0 |
| 645 | 0 | 0.00015 | 0.893591 | 0.106258 | 0 | 0 | 0 | 0 | 0 |
| 646 | 0.00002 | 0.00015 | 0.889007 | 0.110824 | 0 | 0 | 0 | 0 | 0 |
| 647 | 0 | 0.000169 | 0.893751 | 0.10608 | 0 | 0 | 0 | 0 | 0 |
| 648 | 0.00001 | 0.00023 | 0.892653 | 0.107107 | 0 | 0 | 0 | 0 | 0 |
| 649 | 0.00003 | 0.00026 | 0.893592 | 0.106118 | 0 | 0 | 0 | 0 | 0 |
| 650 | 0.00001 | 0.00023 | 0.890479 | 0.109281 | 0 | 0 | 0 | 0 | 0 |
| 651 | 0.00002 | 0.00009 | 0.892462 | 0.107429 | 0 | 0 | 0 | 0 | 0 |
| 652 | 0.00003 | 0.00016 | 0.891646 | 0.108163 | 0 | 0 | 0 | 0 | 0 |
| 653 | 0 | 0.000241 | 0.894267 | 0.105493 | 0 | 0 | 0 | 0 | 0 |
| 654 | 0.00001 | 0.0001 | 0.893718 | 0.106171 | 0 | 0 | 0 | 0 | 0 |
| 655 | 0.00001 | 0.000159 | 0.887509 | 0.112322 | 0 | 0 | 0 | 0 | 0 |
| 656 | 0.00001 | 0.000151 | 0.897515 | 0.102324 | 0 | 0 | 0 | 0 | 0 |
| 657 | 0.00002 | 0.00015 | 0.891858 | 0.107972 | 0 | 0 | 0 | 0 | 0 |
| 658 | 0.00002 | 0.000241 | 0.892056 | 0.107683 | 0 | 0 | 0 | 0 | 0 |
| 659 | 0.00001 | 0.00019 | 0.889311 | 0.110489 | 0 | 0 | 0 | 0 | 0 |
| 660 | 0.00002 | 0.00019 | 0.897322 | 0.102468 | 0 | 0 | 0 | 0 | 0 |
| 661 | 0 | 0.000199 | 0.893354 | 0.106447 | 0 | 0 | 0 | 0 | 0 |
| 662 | 0 | 0.000208 | 0.888606 | 0.111186 | 0 | 0 | 0 | 0 | 0 |
| 663 | 0.00003 | 0.00014 | 0.894637 | 0.105193 | 0 | 0 | 0 | 0 | 0 |
| 664 | 0.00001 | 0.00016 | 0.897856 | 0.101974 | 0 | 0 | 0 | 0 | 0 |
| 665 | 0.00001 | 0.00015 | 0.889237 | 0.110603 | 0 | 0 | 0 | 0 | 0 |
| 666 | 0 | 0.000181 | 0.890546 | 0.109273 | 0 | 0 | 0 | 0 | 0 |
| 667 | 0.00002 | 0.00016 | 0.888502 | 0.111318 | 0 | 0 | 0 | 0 | 0 |
| 668 | 0.00001 | 0.000171 | 0.888722 | 0.111098 | 0 | 0 | 0 | 0 | 0 |
| 669 | 0.00003 | 0.000159 | 0.8906 | 0.109211 | 0 | 0 | 0 | 0 | 0 |
| 670 | 0 | 0.000161 | 0.889096 | 0.110743 | 0 | 0 | 0 | 0 | 0 |
| 671 | 0.00002 | 0.000241 | 0.891774 | 0.107966 | 0 | 0 | 0 | 0 | 0 |
| 672 | 0 | 0.00014 | 0.891675 | 0.108185 | 0 | 0 | 0 | 0 | 0 |
| 673 | 0.00002 | 0.00017 | 0.892326 | 0.107484 | 0 | 0 | 0 | 0 | 0 |
| 674 | 0.00001 | 0.00017 | 0.891544 | 0.108276 | 0 | 0 | 0 | 0 | 0 |
| 675 | 0.00002 | 0.000161 | 0.892877 | 0.106942 | 0 | 0 | 0 | 0 | 0 |
| 676 | 0 | 0.00031 | 0.89448 | 0.10521 | 0 | 0 | 0 | 0 | 0 |
| 677 | 0.00004 | 0.00016 | 0.893025 | 0.106775 | 0 | 0 | 0 | 0 | 0 |
| 678 | 0 | 0.00021 | 0.889453 | 0.110337 | 0 | 0 | 0 | 0 | 0 |
| 679 | 0 | 0.00014 | 0.894632 | 0.105228 | 0 | 0 | 0 | 0 | 0 |
| 680 | 0 | 0.000211 | 0.889787 | 0.110002 | 0 | 0 | 0 | 0 | 0 |
| 681 | 0 | 0.00019 | 0.888497 | 0.111313 | 0 | 0 | 0 | 0 | 0 |
| 682 | 0.00005 | 0.00012 | 0.891188 | 0.108642 | 0 | 0 | 0 | 0 | 0 |
| 683 | 0.00002 | 0.00026 | 0.893703 | 0.106017 | 0 | 0 | 0 | 0 | 0 |
| 684 | 0.00001 | 0.00012 | 0.891569 | 0.108302 | 0 | 0 | 0 | 0 | 0 |
| 685 | 0 | 0.000081 | 0.895213 | 0.104707 | 0 | 0 | 0 | 0 | 0 |
| 686 | 0.00002 | 0.000191 | 0.886589 | 0.1132 | 0 | 0 | 0 | 0 | 0 |
| 687 | 0 | 0.00013 | 0.891608 | 0.108262 | 0 | 0 | 0 | 0 | 0 |
| 688 | 0.00001 | 0.00009 | 0.891174 | 0.108726 | 0 | 0 | 0 | 0 | 0 |
| 689 | 0 | 0.00017 | 0.890018 | 0.109812 | 0 | 0 | 0 | 0 | 0 |
| 690 | 0 | 0.000159 | 0.891962 | 0.107878 | 0 | 0 | 0 | 0 | 0 |
| 691 | 0.00001 | 0.000278 | 0.893642 | 0.10607 | 0 | 0 | 0 | 0 | 0 |
| 692 | 0.00001 | 0.000209 | 0.891302 | 0.108479 | 0 | 0 | 0 | 0 | 0 |
| 693 | 0.00002 | 0.000239 | 0.889924 | 0.109817 | 0 | 0 | 0 | 0 | 0 |
| 694 | 0.00001 | 0.00019 | 0.894459 | 0.105342 | 0 | 0 | 0 | 0 | 0 |
| 695 | 0 | 0.00011 | 0.893626 | 0.106265 | 0 | 0 | 0 | 0 | 0 |
| 696 | 0.00001 | 0.000259 | 0.895606 | 0.104125 | 0 | 0 | 0 | 0 | 0 |
| 697 | 0.00001 | 0.0002 | 0.894614 | 0.105176 | 0 | 0 | 0 | 0 | 0 |
| 698 | 0.00002 | 0.00011 | 0.892067 | 0.107803 | 0 | 0 | 0 | 0 | 0 |
| 699 | 0 | 0.000218 | 0.892476 | 0.107306 | 0 | 0 | 0 | 0 | 0 |
| 700 | 0.00001 | 0.000311 | 0.890719 | 0.10896 | 0 | 0 | 0 | 0 | 0 |
| 701 | 0 | 0.000231 | 0.900424 | 0.099345 | 0 | 0 | 0 | 0 | 0 |
| 702 | 0.00003 | 0.000233 | 0.89614 | 0.103597 | 0 | 0 | 0 | 0 | 0 |
| 703 | 0.00003 | 0.00016 | 0.891072 | 0.108738 | 0 | 0 | 0 | 0 | 0 |
| 704 | 0.00001 | 0.00018 | 0.892895 | 0.106915 | 0 | 0 | 0 | 0 | 0 |
| 705 | 0.00002 | 0.00018 | 0.890817 | 0.108984 | 0 | 0 | 0 | 0 | 0 |
| 706 | 0 | 0.000131 | 0.891858 | 0.108011 | 0 | 0 | 0 | 0 | 0 |
| 707 | 0 | 0.000289 | 0.888126 | 0.111585 | 0 | 0 | 0 | 0 | 0 |
| 708 | 0 | 0.00022 | 0.891129 | 0.108652 | 0 | 0 | 0 | 0 | 0 |
| 709 | 0.00002 | 0.000199 | 0.896192 | 0.10359 | 0 | 0 | 0 | 0 | 0 |
| 710 | 0 | 0.000389 | 0.893319 | 0.106292 | 0 | 0 | 0 | 0 | 0 |
| 711 | 0 | 0.000191 | 0.893843 | 0.105967 | 0 | 0 | 0 | 0 | 0 |
| 712 | 0.00002 | 0.000169 | 0.898149 | 0.101662 | 0 | 0 | 0 | 0 | 0 |
| 713 | 0.00002 | 0.00013 | 0.889348 | 0.110502 | 0 | 0 | 0 | 0 | 0 |
| 714 | 0.00001 | 0.000209 | 0.894196 | 0.105585 | 0 | 0 | 0 | 0 | 0 |
| 715 | 0 | 0.000131 | 0.885261 | 0.114608 | 0 | 0 | 0 | 0 | 0 |
| 716 | 0.00003 | 0.00017 | 0.886725 | 0.113075 | 0 | 0 | 0 | 0 | 0 |
| 717 | 0.00002 | 0.000298 | 0.892637 | 0.107045 | 0 | 0 | 0 | 0 | 0 |
| 718 | 0 | 0.000222 | 0.896243 | 0.103535 | 0 | 0 | 0 | 0 | 0 |
| 719 | 0.00002 | 0.00018 | 0.895997 | 0.103803 | 0 | 0 | 0 | 0 | 0 |
| 720 | 0 | 0.00013 | 0.889657 | 0.110212 | 0 | 0 | 0 | 0 | 0 |
| 721 | 0.00003 | 0.00014 | 0.88995 | 0.109879 | 0 | 0 | 0 | 0 | 0 |
| 722 | 0.00001 | 0.000199 | 0.893877 | 0.105913 | 0 | 0 | 0 | 0 | 0 |
| 723 | 0.00002 | 0.000179 | 0.891613 | 0.108188 | 0 | 0 | 0 | 0 | 0 |
| 724 | 0.00001 | 0.00018 | 0.893334 | 0.106476 | 0 | 0 | 0 | 0 | 0 |
| 725 | 0 | 0.00013 | 0.890795 | 0.109075 | 0 | 0 | 0 | 0 | 0 |
| 726 | 0.00001 | 0.000241 | 0.893755 | 0.105994 | 0 | 0 | 0 | 0 | 0 |
| 727 | 0 | 0.00011 | 0.888934 | 0.110955 | 0 | 0 | 0 | 0 | 0 |
| 728 | 0 | 0.000349 | 0.891795 | 0.107856 | 0 | 0 | 0 | 0 | 0 |
| 729 | 0.00002 | 0.00028 | 0.891528 | 0.108173 | 0 | 0 | 0 | 0 | 0 |
| 730 | 0.00001 | 0.000201 | 0.889949 | 0.10984 | 0 | 0 | 0 | 0 | 0 |
| 731 | 0.00001 | 0.00024 | 0.892834 | 0.106916 | 0 | 0 | 0 | 0 | 0 |
| 732 | 0.00002 | 0.000121 | 0.894434 | 0.105425 | 0 | 0 | 0 | 0 | 0 |
| 733 | 0.00001 | 0.000178 | 0.893392 | 0.10642 | 0 | 0 | 0 | 0 | 0 |
| 734 | 0.00001 | 0.000201 | 0.892622 | 0.107167 | 0 | 0 | 0 | 0 | 0 |
| 735 | 0 | 0.000261 | 0.894836 | 0.104903 | 0 | 0 | 0 | 0 | 0 |
| 736 | 0 | 0.00015 | 0.895437 | 0.104413 | 0 | 0 | 0 | 0 | 0 |
| 737 | 0 | 0.000239 | 0.89252 | 0.107241 | 0 | 0 | 0 | 0 | 0 |
| 738 | 0.00003 | 0.0001 | 0.890785 | 0.109085 | 0 | 0 | 0 | 0 | 0 |
| 739 | 0 | 0.00014 | 0.890198 | 0.109661 | 0 | 0 | 0 | 0 | 0 |
| 740 | 0.00002 | 0.000239 | 0.895178 | 0.104563 | 0 | 0 | 0 | 0 | 0 |
| 741 | 0.00002 | 0.000119 | 0.892551 | 0.10731 | 0 | 0 | 0 | 0 | 0 |
| 742 | 0.00003 | 0.000121 | 0.893445 | 0.106404 | 0 | 0 | 0 | 0 | 0 |
| 743 | 0.00002 | 0.000151 | 0.893445 | 0.106384 | 0 | 0 | 0 | 0 | 0 |
| 744 | 0.00004 | 0.00023 | 0.89535 | 0.10436 | 0 | 0 | 0 | 0 | 0 |
| 745 | 0.00002 | 0.000162 | 0.894563 | 0.105255 | 0 | 0 | 0 | 0 | 0 |
| 746 | 0.00001 | 0.000219 | 0.887841 | 0.11193 | 0 | 0 | 0 | 0 | 0 |
| 747 | 0.00001 | 0.00013 | 0.890979 | 0.108881 | 0 | 0 | 0 | 0 | 0 |
| 748 | 0.00001 | 0.000151 | 0.894492 | 0.105347 | 0 | 0 | 0 | 0 | 0 |
| 749 | 0.00001 | 0.000259 | 0.897817 | 0.101913 | 0 | 0 | 0 | 0 | 0 |
| 750 | 0 | 0.00017 | 0.893945 | 0.105885 | 0 | 0 | 0 | 0 | 0 |
| 751 | 0.00002 | 0.000169 | 0.894471 | 0.10534 | 0 | 0 | 0 | 0 | 0 |
| 752 | 0.00002 | 0.00012 | 0.891885 | 0.107976 | 0 | 0 | 0 | 0 | 0 |
| 753 | 0.00004 | 0.000101 | 0.893717 | 0.106142 | 0 | 0 | 0 | 0 | 0 |
| 754 | 0.00003 | 0.000229 | 0.890616 | 0.109125 | 0 | 0 | 0 | 0 | 0 |
| 755 | 0 | 0.000209 | 0.891053 | 0.108738 | 0 | 0 | 0 | 0 | 0 |
| 756 | 0.00001 | 0.000141 | 0.889788 | 0.110061 | 0 | 0 | 0 | 0 | 0 |
| 757 | 0.00004 | 0.000249 | 0.892169 | 0.107542 | 0 | 0 | 0 | 0 | 0 |
| 758 | 0.00001 | 0.000221 | 0.890602 | 0.109167 | 0 | 0 | 0 | 0 | 0 |
| 759 | 0.00002 | 0.000179 | 0.891516 | 0.108286 | 0 | 0 | 0 | 0 | 0 |
| 760 | 0.00001 | 0.00026 | 0.892743 | 0.106987 | 0 | 0 | 0 | 0 | 0 |
| 761 | 0.00004 | 0.000189 | 0.893031 | 0.10674 | 0 | 0 | 0 | 0 | 0 |
| 762 | 0.00002 | 0.0002 | 0.8942 | 0.10558 | 0 | 0 | 0 | 0 | 0 |
| 763 | 0.00002 | 0.000159 | 0.890644 | 0.109176 | 0 | 0 | 0 | 0 | 0 |
| 764 | 0.00001 | 0.00012 | 0.897748 | 0.102122 | 0 | 0 | 0 | 0 | 0 |
| 765 | 0 | 0.0002 | 0.890809 | 0.108991 | 0 | 0 | 0 | 0 | 0 |
| 766 | 0 | 0.000139 | 0.889426 | 0.110435 | 0 | 0 | 0 | 0 | 0 |
| 767 | 0.00001 | 0.000212 | 0.893428 | 0.106349 | 0 | 0 | 0 | 0 | 0 |
| 768 | 0 | 0.00024 | 0.890978 | 0.108782 | 0 | 0 | 0 | 0 | 0 |
| 769 | 0 | 0.00022 | 0.891323 | 0.108457 | 0 | 0 | 0 | 0 | 0 |
| 770 | 0.00001 | 0.000243 | 0.892211 | 0.107536 | 0 | 0 | 0 | 0 | 0 |
| 771 | 0.00003 | 0.00011 | 0.891786 | 0.108044 | 0 | 0 | 0 | 0 | 0 |
| 772 | 0.00001 | 0.000171 | 0.888954 | 0.110866 | 0 | 0 | 0 | 0 | 0 |
| 773 | 0.00002 | 0.00028 | 0.887005 | 0.112695 | 0 | 0 | 0 | 0 | 0 |
| 774 | 0.00003 | 0.00014 | 0.892266 | 0.107564 | 0 | 0 | 0 | 0 | 0 |
| 775 | 0.00001 | 0.000181 | 0.893299 | 0.10651 | 0 | 0 | 0 | 0 | 0 |
| 776 | 0.00001 | 0.00022 | 0.890765 | 0.109005 | 0 | 0 | 0 | 0 | 0 |
| 777 | 0.00002 | 0.000199 | 0.893943 | 0.105837 | 0 | 0 | 0 | 0 | 0 |
| 778 | 0 | 0.000189 | 0.895369 | 0.104441 | 0 | 0 | 0 | 0 | 0 |
| 779 | 0.00002 | 0.000191 | 0.897334 | 0.102455 | 0 | 0 | 0 | 0 | 0 |
| 780 | 0.00001 | 0.00017 | 0.891013 | 0.108807 | 0 | 0 | 0 | 0 | 0 |
| 781 | 0 | 0.0002 | 0.892063 | 0.107738 | 0 | 0 | 0 | 0 | 0 |
| 782 | 0 | 0.000182 | 0.896486 | 0.103333 | 0 | 0 | 0 | 0 | 0 |
| 783 | 0 | 0.000199 | 0.893064 | 0.106737 | 0 | 0 | 0 | 0 | 0 |
| 784 | 0.00001 | 0.000182 | 0.892154 | 0.107655 | 0 | 0 | 0 | 0 | 0 |
| 785 | 0.00003 | 0.00011 | 0.894585 | 0.105275 | 0 | 0 | 0 | 0 | 0 |
| 786 | 0.00001 | 0.000219 | 0.891442 | 0.108329 | 0 | 0 | 0 | 0 | 0 |
| 787 | 0 | 0.00013 | 0.892996 | 0.106874 | 0 | 0 | 0 | 0 | 0 |
| 788 | 0 | 0.00017 | 0.896343 | 0.103487 | 0 | 0 | 0 | 0 | 0 |
| 789 | 0.00001 | 0.00017 | 0.888768 | 0.111052 | 0 | 0 | 0 | 0 | 0 |
| 790 | 0.00001 | 0.00011 | 0.893499 | 0.106381 | 0 | 0 | 0 | 0 | 0 |
| 791 | 0 | 0.00008 | 0.894569 | 0.105351 | 0 | 0 | 0 | 0 | 0 |
| 792 | 0.00004 | 0.000222 | 0.893935 | 0.105803 | 0 | 0 | 0 | 0 | 0 |
| 793 | 0 | 0.000179 | 0.889347 | 0.110474 | 0 | 0 | 0 | 0 | 0 |
| 794 | 0.00001 | 0.000161 | 0.894049 | 0.10578 | 0 | 0 | 0 | 0 | 0 |
| 795 | 0.00001 | 0.00015 | 0.890076 | 0.109764 | 0 | 0 | 0 | 0 | 0 |
| 796 | 0.00001 | 0.00024 | 0.893391 | 0.106359 | 0 | 0 | 0 | 0 | 0 |
| 797 | 0.00001 | 0.00009 | 0.891353 | 0.108547 | 0 | 0 | 0 | 0 | 0 |
| 798 | 0 | 0.00014 | 0.891152 | 0.108698 | 0 | 0 | 0 | 0 | 0 |
| 799 | 0.00001 | 0.000189 | 0.893385 | 0.106416 | 0 | 0 | 0 | 0 | 0 |
| 800 | 0.00002 | 0.000159 | 0.889097 | 0.110724 | 0 | 0 | 0 | 0 | 0 |
| 801 | 0.00003 | 0.00017 | 0.898146 | 0.101654 | 0 | 0 | 0 | 0 | 0 |
| 802 | 0.00002 | 0.00014 | 0.890337 | 0.109503 | 0 | 0 | 0 | 0 | 0 |
| 803 | 0.00001 | 0.000199 | 0.895497 | 0.104294 | 0 | 0 | 0 | 0 | 0 |
| 804 | 0.00002 | 0.000299 | 0.891041 | 0.10864 | 0 | 0 | 0 | 0 | 0 |
| 805 | 0 | 0.000139 | 0.895256 | 0.104604 | 0 | 0 | 0 | 0 | 0 |
| 806 | 0 | 0.000201 | 0.890192 | 0.109607 | 0 | 0 | 0 | 0 | 0 |
| 807 | 0.00002 | 0.000169 | 0.893666 | 0.106145 | 0 | 0 | 0 | 0 | 0 |
| 808 | 0.00006 | 0.00011 | 0.891106 | 0.108724 | 0 | 0 | 0 | 0 | 0 |
| 809 | 0 | 0.000139 | 0.891717 | 0.108144 | 0 | 0 | 0 | 0 | 0 |
| 810 | 0 | 0.000221 | 0.891806 | 0.107972 | 0 | 0 | 0 | 0 | 0 |
| 811 | 0.00002 | 0.0002 | 0.891872 | 0.107908 | 0 | 0 | 0 | 0 | 0 |
| 812 | 0.00002 | 0.00007 | 0.895102 | 0.104808 | 0 | 0 | 0 | 0 | 0 |
| 813 | 0.00002 | 0.00021 | 0.894773 | 0.104997 | 0 | 0 | 0 | 0 | 0 |
| 814 | 0.00001 | 0.000389 | 0.895595 | 0.104006 | 0 | 0 | 0 | 0 | 0 |
| 815 | 0.00004 | 0.00008 | 0.889967 | 0.109914 | 0 | 0 | 0 | 0 | 0 |
| 816 | 0.00002 | 0.000219 | 0.894308 | 0.105453 | 0 | 0 | 0 | 0 | 0 |
| 817 | 0.00001 | 0.000221 | 0.896179 | 0.10359 | 0 | 0 | 0 | 0 | 0 |
| 818 | 0.00001 | 0.00015 | 0.890727 | 0.109113 | 0 | 0 | 0 | 0 | 0 |
| 819 | 0.00001 | 0.00014 | 0.892094 | 0.107756 | 0 | 0 | 0 | 0 | 0 |
| 820 | 0 | 0.00017 | 0.892631 | 0.107199 | 0 | 0 | 0 | 0 | 0 |
| 821 | 0.00002 | 0.00017 | 0.895908 | 0.103902 | 0 | 0 | 0 | 0 | 0 |
| 822 | 0.00001 | 0.000241 | 0.893974 | 0.105776 | 0 | 0 | 0 | 0 | 0 |
| 823 | 0.00002 | 0.000171 | 0.889934 | 0.109875 | 0 | 0 | 0 | 0 | 0 |
| 824 | 0.00001 | 0.000191 | 0.896431 | 0.103368 | 0 | 0 | 0 | 0 | 0 |
| 825 | 0.00001 | 0.00014 | 0.895698 | 0.104151 | 0 | 0 | 0 | 0 | 0 |
| 826 | 0.00003 | 0.000218 | 0.893277 | 0.106475 | 0 | 0 | 0 | 0 | 0 |
| 827 | 0 | 0.000242 | 0.891172 | 0.108586 | 0 | 0 | 0 | 0 | 0 |
| 828 | 0 | 0.00012 | 0.89387 | 0.10601 | 0 | 0 | 0 | 0 | 0 |
| 829 | 0.00001 | 0.00019 | 0.891089 | 0.10871 | 0 | 0 | 0 | 0 | 0 |
| 830 | 0 | 0.000171 | 0.894276 | 0.105553 | 0 | 0 | 0 | 0 | 0 |
| 831 | 0.00001 | 0.00029 | 0.888514 | 0.111186 | 0 | 0 | 0 | 0 | 0 |
| 832 | 0.00001 | 0.000191 | 0.891723 | 0.108077 | 0 | 0 | 0 | 0 | 0 |
| 833 | 0.00002 | 0.00021 | 0.892228 | 0.107543 | 0 | 0 | 0 | 0 | 0 |
| 834 | 0.00001 | 0.000188 | 0.890455 | 0.109347 | 0 | 0 | 0 | 0 | 0 |
| 835 | 0.00001 | 0.00017 | 0.889732 | 0.110088 | 0 | 0 | 0 | 0 | 0 |
| 836 | 0 | 0.000131 | 0.891201 | 0.108668 | 0 | 0 | 0 | 0 | 0 |
| 837 | 0.00001 | 0.00016 | 0.896608 | 0.103222 | 0 | 0 | 0 | 0 | 0 |
| 838 | 0.00002 | 0.000171 | 0.891706 | 0.108103 | 0 | 0 | 0 | 0 | 0 |
| 839 | 0 | 0.00017 | 0.891045 | 0.108785 | 0 | 0 | 0 | 0 | 0 |
| 840 | 0.00001 | 0.00012 | 0.892802 | 0.107068 | 0 | 0 | 0 | 0 | 0 |
| 841 | 0.00003 | 0.000272 | 0.894683 | 0.105015 | 0 | 0 | 0 | 0 | 0 |
| 842 | 0.00001 | 0.000109 | 0.890434 | 0.109447 | 0 | 0 | 0 | 0 | 0 |
| 843 | 0.00004 | 0.000158 | 0.895751 | 0.104051 | 0 | 0 | 0 | 0 | 0 |
| 844 | 0.00001 | 0.000139 | 0.894542 | 0.105309 | 0 | 0 | 0 | 0 | 0 |
| 845 | 0 | 0.00017 | 0.889429 | 0.110401 | 0 | 0 | 0 | 0 | 0 |
| 846 | 0.00003 | 0.00009 | 0.890497 | 0.109383 | 0 | 0 | 0 | 0 | 0 |
| 847 | 0.00002 | 0.000191 | 0.890693 | 0.109096 | 0 | 0 | 0 | 0 | 0 |
| 848 | 0 | 0.00013 | 0.895288 | 0.104582 | 0 | 0 | 0 | 0 | 0 |
| 849 | 0.00002 | 0.00014 | 0.897566 | 0.102275 | 0 | 0 | 0 | 0 | 0 |
| 850 | 0.00002 | 0.0001 | 0.892349 | 0.107531 | 0 | 0 | 0 | 0 | 0 |
| 851 | 0 | 0.000201 | 0.891667 | 0.108132 | 0 | 0 | 0 | 0 | 0 |
| 852 | 0.00001 | 0.00012 | 0.890196 | 0.109673 | 0 | 0 | 0 | 0 | 0 |
| 853 | 0.00001 | 0.00021 | 0.889078 | 0.110703 | 0 | 0 | 0 | 0 | 0 |
| 854 | 0 | 0.00017 | 0.890294 | 0.109536 | 0 | 0 | 0 | 0 | 0 |
| 855 | 0.00003 | 0.00022 | 0.891894 | 0.107856 | 0 | 0 | 0 | 0 | 0 |
| 856 | 0.00001 | 0.00016 | 0.886972 | 0.112858 | 0 | 0 | 0 | 0 | 0 |
| 857 | 0.00002 | 0.00013 | 0.889373 | 0.110477 | 0 | 0 | 0 | 0 | 0 |
| 858 | 0.00001 | 0.00023 | 0.891749 | 0.108011 | 0 | 0 | 0 | 0 | 0 |
| 859 | 0.00002 | 0.000251 | 0.893588 | 0.10614 | 0 | 0 | 0 | 0 | 0 |
| 860 | 0.00002 | 0.00031 | 0.890425 | 0.109245 | 0 | 0 | 0 | 0 | 0 |
| 861 | 0.00001 | 0.00024 | 0.896035 | 0.103704 | 0 | 0 | 0 | 0 | 0 |
| 862 | 0 | 0.000198 | 0.891053 | 0.108748 | 0 | 0 | 0 | 0 | 0 |
| 863 | 0.00001 | 0.00018 | 0.891077 | 0.108733 | 0 | 0 | 0 | 0 | 0 |
| 864 | 0 | 0.000179 | 0.892434 | 0.107387 | 0 | 0 | 0 | 0 | 0 |
| 865 | 0.00002 | 0.000221 | 0.892654 | 0.107105 | 0 | 0 | 0 | 0 | 0 |
| 866 | 0.00002 | 0.00015 | 0.892353 | 0.107478 | 0 | 0 | 0 | 0 | 0 |
| 867 | 0.00003 | 0.000159 | 0.891408 | 0.108403 | 0 | 0 | 0 | 0 | 0 |
| 868 | 0 | 0.00018 | 0.886399 | 0.113421 | 0 | 0 | 0 | 0 | 0 |
| 869 | 0.00001 | 0.00007 | 0.891636 | 0.108284 | 0 | 0 | 0 | 0 | 0 |
| 870 | 0.00001 | 0.0001 | 0.89253 | 0.10736 | 0 | 0 | 0 | 0 | 0 |
| 871 | 0.00002 | 0.00014 | 0.897327 | 0.102513 | 0 | 0 | 0 | 0 | 0 |
| 872 | 0.00001 | 0.000171 | 0.891835 | 0.107984 | 0 | 0 | 0 | 0 | 0 |
| 873 | 0.00002 | 0.000211 | 0.890224 | 0.109545 | 0 | 0 | 0 | 0 | 0 |
| 874 | 0.00003 | 0.000179 | 0.891539 | 0.108252 | 0 | 0 | 0 | 0 | 0 |
| 875 | 0.00005 | 0.000161 | 0.89358 | 0.106209 | 0 | 0 | 0 | 0 | 0 |
| 876 | 0.00001 | 0.000247 | 0.891912 | 0.10783 | 0 | 0 | 0 | 0 | 0 |
| 877 | 0.00001 | 0.00018 | 0.894154 | 0.105656 | 0 | 0 | 0 | 0 | 0 |
| 878 | 0 | 0.000231 | 0.894625 | 0.105144 | 0 | 0 | 0 | 0 | 0 |
| 879 | 0 | 0.0002 | 0.891164 | 0.108636 | 0 | 0 | 0 | 0 | 0 |
| 880 | 0.00003 | 0.000161 | 0.892984 | 0.106825 | 0 | 0 | 0 | 0 | 0 |
| 881 | 0.00002 | 0.000141 | 0.889736 | 0.110104 | 0 | 0 | 0 | 0 | 0 |
| 882 | 0.00001 | 0.00017 | 0.89064 | 0.10918 | 0 | 0 | 0 | 0 | 0 |
| 883 | 0.00002 | 0.00018 | 0.89297 | 0.10683 | 0 | 0 | 0 | 0 | 0 |
| 884 | 0.00002 | 0.00018 | 0.890152 | 0.109648 | 0 | 0 | 0 | 0 | 0 |
| 885 | 0.00001 | 0.00017 | 0.896336 | 0.103484 | 0 | 0 | 0 | 0 | 0 |
| 886 | 0.00001 | 0.000179 | 0.894412 | 0.105399 | 0 | 0 | 0 | 0 | 0 |
| 887 | 0.00001 | 0.000149 | 0.893359 | 0.106482 | 0 | 0 | 0 | 0 | 0 |
| 888 | 0.00001 | 0.00018 | 0.886086 | 0.113724 | 0 | 0 | 0 | 0 | 0 |
| 889 | 0.00001 | 0.000252 | 0.883491 | 0.116248 | 0 | 0 | 0 | 0 | 0 |
| 890 | 0 | 0.00026 | 0.894926 | 0.104813 | 0 | 0 | 0 | 0 | 0 |
| 891 | 0 | 0.00017 | 0.890393 | 0.109436 | 0 | 0 | 0 | 0 | 0 |
| 892 | 0 | 0.000169 | 0.892672 | 0.107159 | 0 | 0 | 0 | 0 | 0 |
| 893 | 0.00001 | 0.00026 | 0.891313 | 0.108417 | 0 | 0 | 0 | 0 | 0 |
| 894 | 0.00002 | 0.000252 | 0.891024 | 0.108704 | 0 | 0 | 0 | 0 | 0 |
| 895 | 0.00001 | 0.00022 | 0.897223 | 0.102547 | 0 | 0 | 0 | 0 | 0 |
| 896 | 0 | 0.00013 | 0.892155 | 0.107715 | 0 | 0 | 0 | 0 | 0 |
| 897 | 0 | 0.000169 | 0.893833 | 0.105998 | 0 | 0 | 0 | 0 | 0 |
| 898 | 0.00002 | 0.00007 | 0.891909 | 0.108001 | 0 | 0 | 0 | 0 | 0 |
| 899 | 0.00002 | 0.000202 | 0.894452 | 0.105326 | 0 | 0 | 0 | 0 | 0 |
| 900 | 0.00003 | 0.000192 | 0.890439 | 0.109339 | 0 | 0 | 0 | 0 | 0 |
| 901 | 0.00002 | 0.00021 | 0.898131 | 0.101639 | 0 | 0 | 0 | 0 | 0 |
| 902 | 0.00001 | 0.00014 | 0.892358 | 0.107492 | 0 | 0 | 0 | 0 | 0 |
| 903 | 0.00001 | 0.0002 | 0.892703 | 0.107087 | 0 | 0 | 0 | 0 | 0 |
| 904 | 0 | 0.000199 | 0.89496 | 0.104841 | 0 | 0 | 0 | 0 | 0 |
| 905 | 0.00001 | 0.00022 | 0.894557 | 0.105213 | 0 | 0 | 0 | 0 | 0 |
| 906 | 0 | 0.00021 | 0.89223 | 0.10756 | 0 | 0 | 0 | 0 | 0 |
| 907 | 0.00001 | 0.000136 | 0.898493 | 0.101361 | 0 | 0 | 0 | 0 | 0 |
| 908 | 0 | 0.000308 | 0.895338 | 0.104354 | 0 | 0 | 0 | 0 | 0 |
| 909 | 0 | 0.00022 | 0.892036 | 0.107744 | 0 | 0 | 0 | 0 | 0 |
| 910 | 0 | 0.000161 | 0.892091 | 0.107747 | 0 | 0 | 0 | 0 | 0 |
| 911 | 0 | 0.000101 | 0.895678 | 0.104221 | 0 | 0 | 0 | 0 | 0 |
| 912 | 0.00001 | 0.00021 | 0.889431 | 0.110349 | 0 | 0 | 0 | 0 | 0 |
| 913 | 0.00001 | 0.000309 | 0.894958 | 0.104723 | 0 | 0 | 0 | 0 | 0 |
| 914 | 0 | 0.00009 | 0.895576 | 0.104334 | 0 | 0 | 0 | 0 | 0 |
| 915 | 0.00001 | 0.0001 | 0.888423 | 0.111467 | 0 | 0 | 0 | 0 | 0 |
| 916 | 0.00002 | 0.00024 | 0.893707 | 0.106033 | 0 | 0 | 0 | 0 | 0 |
| 917 | 0.00003 | 0.000229 | 0.89234 | 0.107401 | 0 | 0 | 0 | 0 | 0 |
| 918 | 0 | 0.00013 | 0.894668 | 0.105202 | 0 | 0 | 0 | 0 | 0 |
| 919 | 0.00005 | 0.000271 | 0.891521 | 0.108158 | 0 | 0 | 0 | 0 | 0 |
| 920 | 0.00002 | 0.00012 | 0.89776 | 0.102101 | 0 | 0 | 0 | 0 | 0 |
| 921 | 0.00001 | 0.000159 | 0.892623 | 0.107207 | 0 | 0 | 0 | 0 | 0 |
| 922 | 0.00003 | 0.000141 | 0.893361 | 0.106469 | 0 | 0 | 0 | 0 | 0 |
| 923 | 0.00003 | 0.00016 | 0.893458 | 0.106352 | 0 | 0 | 0 | 0 | 0 |
| 924 | 0 | 0.000099 | 0.893123 | 0.106778 | 0 | 0 | 0 | 0 | 0 |
| 925 | 0.00002 | 0.000181 | 0.892379 | 0.10742 | 0 | 0 | 0 | 0 | 0 |
| 926 | 0.00003 | 0.000249 | 0.894352 | 0.105369 | 0 | 0 | 0 | 0 | 0 |
| 927 | 0.00001 | 0.000191 | 0.890701 | 0.109098 | 0 | 0 | 0 | 0 | 0 |
| 928 | 0 | 0.000149 | 0.891472 | 0.108379 | 0 | 0 | 0 | 0 | 0 |
| 929 | 0.00001 | 0.00021 | 0.889946 | 0.109834 | 0 | 0 | 0 | 0 | 0 |
| 930 | 0.00001 | 0.000239 | 0.89461 | 0.10514 | 0 | 0 | 0 | 0 | 0 |
| 931 | 0.00001 | 0.000201 | 0.897146 | 0.102643 | 0 | 0 | 0 | 0 | 0 |
| 932 | 0 | 0.000159 | 0.893665 | 0.106176 | 0 | 0 | 0 | 0 | 0 |
| 933 | 0.00002 | 0.000161 | 0.895639 | 0.10418 | 0 | 0 | 0 | 0 | 0 |
| 934 | 0.00002 | 0.00016 | 0.888412 | 0.111408 | 0 | 0 | 0 | 0 | 0 |
| 935 | 0.00001 | 0.0002 | 0.891758 | 0.108032 | 0 | 0 | 0 | 0 | 0 |
| 936 | 0 | 0.000191 | 0.895536 | 0.104273 | 0 | 0 | 0 | 0 | 0 |
| 937 | 0.00001 | 0.00016 | 0.896377 | 0.103452 | 0 | 0 | 0 | 0 | 0 |
| 938 | 0.00001 | 0.00012 | 0.890491 | 0.109379 | 0 | 0 | 0 | 0 | 0 |
| 939 | 0 | 0.000131 | 0.895336 | 0.104534 | 0 | 0 | 0 | 0 | 0 |
| 940 | 0.00001 | 0.00013 | 0.889697 | 0.110163 | 0 | 0 | 0 | 0 | 0 |
| 941 | 0.00002 | 0.00011 | 0.890877 | 0.108992 | 0 | 0 | 0 | 0 | 0 |
| 942 | 0.00004 | 0.000209 | 0.895298 | 0.104453 | 0 | 0 | 0 | 0 | 0 |
| 943 | 0.00001 | 0.00016 | 0.895832 | 0.103998 | 0 | 0 | 0 | 0 | 0 |
| 944 | 0.00001 | 0.00013 | 0.89096 | 0.108899 | 0 | 0 | 0 | 0 | 0 |
| 945 | 0.00001 | 0.000121 | 0.88817 | 0.1117 | 0 | 0 | 0 | 0 | 0 |
| 946 | 0 | 0.000101 | 0.893153 | 0.106746 | 0 | 0 | 0 | 0 | 0 |
| 947 | 0 | 0.00014 | 0.889095 | 0.110765 | 0 | 0 | 0 | 0 | 0 |
| 948 | 0.00002 | 0.00018 | 0.891599 | 0.108202 | 0 | 0 | 0 | 0 | 0 |
| 949 | 0.00003 | 0.0001 | 0.897217 | 0.102654 | 0 | 0 | 0 | 0 | 0 |
| 950 | 0.00001 | 0.000159 | 0.895848 | 0.103982 | 0 | 0 | 0 | 0 | 0 |
| 951 | 0.00001 | 0.000239 | 0.895562 | 0.104189 | 0 | 0 | 0 | 0 | 0 |
| 952 | 0.00002 | 0.00016 | 0.893483 | 0.106337 | 0 | 0 | 0 | 0 | 0 |
| 953 | 0 | 0.00011 | 0.892948 | 0.106942 | 0 | 0 | 0 | 0 | 0 |
| 954 | 0 | 0.00019 | 0.889193 | 0.110617 | 0 | 0 | 0 | 0 | 0 |
| 955 | 0 | 0.000181 | 0.894026 | 0.105793 | 0 | 0 | 0 | 0 | 0 |
| 956 | 0.00003 | 0.00026 | 0.894856 | 0.104854 | 0 | 0 | 0 | 0 | 0 |
| 957 | 0 | 0.00023 | 0.888926 | 0.110844 | 0 | 0 | 0 | 0 | 0 |
| 958 | 0.00002 | 0.00007 | 0.89316 | 0.10675 | 0 | 0 | 0 | 0 | 0 |
| 959 | 0.00003 | 0.00023 | 0.889293 | 0.110447 | 0 | 0 | 0 | 0 | 0 |
| 960 | 0 | 0.000191 | 0.894347 | 0.105463 | 0 | 0 | 0 | 0 | 0 |
| 961 | 0.00001 | 0.00018 | 0.891652 | 0.108159 | 0 | 0 | 0 | 0 | 0 |
| 962 | 0 | 0.000151 | 0.89266 | 0.10719 | 0 | 0 | 0 | 0 | 0 |
| 963 | 0.00001 | 0.0001 | 0.893573 | 0.106317 | 0 | 0 | 0 | 0 | 0 |
| 964 | 0.00001 | 0.00011 | 0.89383 | 0.10605 | 0 | 0 | 0 | 0 | 0 |
| 965 | 0.00004 | 0.000151 | 0.893688 | 0.106121 | 0 | 0 | 0 | 0 | 0 |
| 966 | 0 | 0.000259 | 0.894351 | 0.105389 | 0 | 0 | 0 | 0 | 0 |
| 967 | 0.00001 | 0.000141 | 0.894921 | 0.104929 | 0 | 0 | 0 | 0 | 0 |
| 968 | 0.00002 | 0.000229 | 0.892044 | 0.107708 | 0 | 0 | 0 | 0 | 0 |
| 969 | 0.00004 | 0.000241 | 0.892804 | 0.106914 | 0 | 0 | 0 | 0 | 0 |
| 970 | 0.00001 | 0.000161 | 0.891898 | 0.10793 | 0 | 0 | 0 | 0 | 0 |
| 971 | 0.00004 | 0.000201 | 0.895817 | 0.103941 | 0 | 0 | 0 | 0 | 0 |
| 972 | 0.00001 | 0.00022 | 0.888615 | 0.111154 | 0 | 0 | 0 | 0 | 0 |
| 973 | 0 | 0.000171 | 0.891504 | 0.108325 | 0 | 0 | 0 | 0 | 0 |
| 974 | 0.00002 | 0.0001 | 0.891802 | 0.108078 | 0 | 0 | 0 | 0 | 0 |
| 975 | 0.00001 | 0.000219 | 0.892895 | 0.106876 | 0 | 0 | 0 | 0 | 0 |
| 976 | 0.00001 | 0.00015 | 0.894661 | 0.10518 | 0 | 0 | 0 | 0 | 0 |
| 977 | 0.00003 | 0.00019 | 0.890389 | 0.109392 | 0 | 0 | 0 | 0 | 0 |
| 978 | 0.00002 | 0.00015 | 0.892844 | 0.106986 | 0 | 0 | 0 | 0 | 0 |
| 979 | 0.00001 | 0.000191 | 0.892967 | 0.106832 | 0 | 0 | 0 | 0 | 0 |
| 980 | 0.00002 | 0.000169 | 0.894 | 0.105812 | 0 | 0 | 0 | 0 | 0 |
| 981 | 0 | 0.00011 | 0.893944 | 0.105946 | 0 | 0 | 0 | 0 | 0 |
| 982 | 0.00004 | 0.000151 | 0.889893 | 0.109916 | 0 | 0 | 0 | 0 | 0 |
| 983 | 0 | 0.00019 | 0.887751 | 0.112059 | 0 | 0 | 0 | 0 | 0 |
| 984 | 0.00001 | 0.000151 | 0.893092 | 0.106747 | 0 | 0 | 0 | 0 | 0 |
| 985 | 0.00001 | 0.00018 | 0.893862 | 0.105948 | 0 | 0 | 0 | 0 | 0 |
| 986 | 0 | 0.00019 | 0.896744 | 0.103066 | 0 | 0 | 0 | 0 | 0 |
| 987 | 0 | 0.00008 | 0.896869 | 0.103051 | 0 | 0 | 0 | 0 | 0 |
| 988 | 0.00001 | 0.000229 | 0.892097 | 0.107664 | 0 | 0 | 0 | 0 | 0 |
| 989 | 0 | 0.00017 | 0.887801 | 0.112029 | 0 | 0 | 0 | 0 | 0 |
| 990 | 0 | 0.00025 | 0.891208 | 0.108542 | 0 | 0 | 0 | 0 | 0 |
| 991 | 0.00001 | 0.00031 | 0.893653 | 0.106027 | 0 | 0 | 0 | 0 | 0 |
| 992 | 0.00001 | 0.000169 | 0.892734 | 0.107087 | 0 | 0 | 0 | 0 | 0 |
| 993 | 0.00002 | 0.00019 | 0.896433 | 0.103358 | 0 | 0 | 0 | 0 | 0 |
| 994 | 0.00002 | 0.00018 | 0.897651 | 0.102149 | 0 | 0 | 0 | 0 | 0 |
| 995 | 0 | 0.0002 | 0.893549 | 0.10625 | 0 | 0 | 0 | 0 | 0 |
| 996 | 0.00002 | 0.000151 | 0.896971 | 0.102859 | 0 | 0 | 0 | 0 | 0 |
| 997 | 0.00001 | 0.00024 | 0.890053 | 0.109696 | 0 | 0 | 0 | 0 | 0 |
| 998 | 0.00003 | 0.00008 | 0.892544 | 0.107345 | 0 | 0 | 0 | 0 | 0 |
| 999 | 0 | 0.000229 | 0.895042 | 0.104729 | 0 | 0 | 0 | 0 | 0 |
| 1000 | 0.00001 | 0.000139 | 0.891591 | 0.10826 | 0 | 0 | 0 | 0 | 0 |

| Year 8 | Race |  |  |  |  |  |  |  |  |
| --- | --- | --- | --- | --- | --- | --- | --- | --- | --- |
| Trial | 003.0 | 005.0 | 007.0 | 037.1 | 043.0 | 303.0 | 003.2 | 403.0 | 003.4 |
| 1 | 0 | 0.00011 | 0.90112 | 0.09877 | 0 | 0 | 0 | 0 | 0 |
| 2 | 0.00002 | 0.00007 | 0.897529 | 0.10238 | 0 | 0 | 0 | 0 | 0 |
| 3 | 0.00002 | 0.00005 | 0.903699 | 0.096231 | 0 | 0 | 0 | 0 | 0 |
| 4 | 0.00002 | 0.00008 | 0.903125 | 0.096775 | 0 | 0 | 0 | 0 | 0 |
| 5 | 0.00002 | 0.000071 | 0.90102 | 0.09889 | 0 | 0 | 0 | 0 | 0 |
| 6 | 0.00001 | 0.00005 | 0.901463 | 0.098478 | 0 | 0 | 0 | 0 | 0 |
| 7 | 0.00001 | 0.0001 | 0.901866 | 0.098024 | 0 | 0 | 0 | 0 | 0 |
| 8 | 0 | 0.00004 | 0.902348 | 0.097612 | 0 | 0 | 0 | 0 | 0 |
| 9 | 0.00002 | 0.00007 | 0.898438 | 0.101472 | 0 | 0 | 0 | 0 | 0 |
| 10 | 0.00001 | 0.000149 | 0.898398 | 0.101443 | 0 | 0 | 0 | 0 | 0 |
| 11 | 0.00001 | 0.00006 | 0.90164 | 0.098291 | 0 | 0 | 0 | 0 | 0 |
| 12 | 0.00001 | 0.00005 | 0.898385 | 0.101554 | 0 | 0 | 0 | 0 | 0 |
| 13 | 0 | 0.000189 | 0.900427 | 0.099384 | 0 | 0 | 0 | 0 | 0 |
| 14 | 0 | 0.00015 | 0.895565 | 0.104285 | 0 | 0 | 0 | 0 | 0 |
| 15 | 0.00001 | 0.00007 | 0.896498 | 0.103422 | 0 | 0 | 0 | 0 | 0 |
| 16 | 0 | 0.00003 | 0.901521 | 0.098449 | 0 | 0 | 0 | 0 | 0 |
| 17 | 0.00001 | 0.000179 | 0.900499 | 0.099312 | 0 | 0 | 0 | 0 | 0 |
| 18 | 0.00002 | 0.000131 | 0.900599 | 0.099251 | 0 | 0 | 0 | 0 | 0 |
| 19 | 0.00002 | 0.000079 | 0.901644 | 0.098257 | 0 | 0 | 0 | 0 | 0 |
| 20 | 0.00001 | 0.00006 | 0.899439 | 0.100491 | 0 | 0 | 0 | 0 | 0 |
| 21 | 0.00002 | 0.00005 | 0.900984 | 0.098946 | 0 | 0 | 0 | 0 | 0 |
| 22 | 0.00002 | 0.00014 | 0.89806 | 0.10178 | 0 | 0 | 0 | 0 | 0 |
| 23 | 0.00003 | 0.000119 | 0.903398 | 0.096453 | 0 | 0 | 0 | 0 | 0 |
| 24 | 0 | 0.00005 | 0.900137 | 0.099813 | 0 | 0 | 0 | 0 | 0 |
| 25 | 0.00002 | 0.00003 | 0.903571 | 0.096379 | 0 | 0 | 0 | 0 | 0 |
| 26 | 0 | 0.00006 | 0.90294 | 0.097 | 0 | 0 | 0 | 0 | 0 |
| 27 | 0.00001 | 0.00007 | 0.901784 | 0.098136 | 0 | 0 | 0 | 0 | 0 |
| 28 | 0 | 0.00001 | 0.901454 | 0.098476 | 6E-05 | 0 | 0 | 0 | 0 |
| 29 | 0.00001 | 0.00002 | 0.903044 | 0.096926 | 0 | 0 | 0 | 0 | 0 |
| 30 | 0 | 0.00012 | 0.90023 | 0.09965 | 0 | 0 | 0 | 0 | 0 |
| 31 | 0.00001 | 0.00006 | 0.902899 | 0.097031 | 0 | 0 | 0 | 0 | 0 |
| 32 | 0.00003 | 0.0001 | 0.902998 | 0.096872 | 0 | 0 | 0 | 0 | 0 |
| 33 | 0.00001 | 0.00004 | 0.901925 | 0.098026 | 0 | 0 | 0 | 0 | 0 |
| 34 | 0 | 0.00007 | 0.901253 | 0.098676 | 0 | 0 | 0 | 0 | 0 |
| 35 | 0.00001 | 0.00004 | 0.895428 | 0.104522 | 0 | 0 | 0 | 0 | 0 |
| 36 | 0.00003 | 0.00006 | 0.898255 | 0.101656 | 0 | 0 | 0 | 0 | 0 |
| 37 | 0.00002 | 0.00004 | 0.904783 | 0.095157 | 0 | 0 | 0 | 0 | 0 |
| 38 | 0 | 0.0001 | 0.902698 | 0.097202 | 0 | 0 | 0 | 0 | 0 |
| 39 | 0 | 0.00003 | 0.899994 | 0.099976 | 0 | 0 | 0 | 0 | 0 |
| 40 | 0 | 0.00009 | 0.89619 | 0.10372 | 0 | 0 | 0 | 0 | 0 |
| 41 | 0.00002 | 0.00007 | 0.900539 | 0.099372 | 0 | 0 | 0 | 0 | 0 |
| 42 | 0.00002 | 0.00013 | 0.901537 | 0.098313 | 0 | 0 | 0 | 0 | 0 |
| 43 | 0 | 0.00001 | 0.899988 | 0.100002 | 0 | 0 | 0 | 0 | 0 |
| 44 | 0 | 0.00007 | 0.901231 | 0.098699 | 0 | 0 | 0 | 0 | 0 |
| 45 | 0.00001 | 0.000101 | 0.898986 | 0.100903 | 0 | 0 | 0 | 0 | 0 |
| 46 | 0.00002 | 0 | 0.897344 | 0.102636 | 0 | 0 | 0 | 0 | 0 |
| 47 | 0 | 0.00004 | 0.900602 | 0.099358 | 0 | 0 | 0 | 0 | 0 |
| 48 | 0.00001 | 0.00008 | 0.900325 | 0.099586 | 0 | 0 | 0 | 0 | 0 |
| 49 | 0 | 0.00011 | 0.899032 | 0.100858 | 0 | 0 | 0 | 0 | 0 |
| 50 | 0 | 0.00003 | 0.902994 | 0.096976 | 0 | 0 | 0 | 0 | 0 |
| 51 | 0.00001 | 0.00009 | 0.899713 | 0.100186 | 0 | 0 | 0 | 0 | 0 |
| 52 | 0 | 0.00006 | 0.903173 | 0.096767 | 0 | 0 | 0 | 0 | 0 |
| 53 | 0 | 0.00002 | 0.902413 | 0.097567 | 0 | 0 | 0 | 0 | 0 |
| 54 | 0 | 0.00007 | 0.901028 | 0.098902 | 0 | 0 | 0 | 0 | 0 |
| 55 | 0.00003 | 0.00009 | 0.901644 | 0.098237 | 0 | 0 | 0 | 0 | 0 |
| 56 | 0.00001 | 0.00006 | 0.901401 | 0.09853 | 0 | 0 | 0 | 0 | 0 |
| 57 | 0.00001 | 0.00009 | 0.90168 | 0.09822 | 0 | 0 | 0 | 0 | 0 |
| 58 | 0.00001 | 0.00006 | 0.901272 | 0.098659 | 0 | 0 | 0 | 0 | 0 |
| 59 | 0.00002 | 0.00006 | 0.902609 | 0.09731 | 0 | 0 | 0 | 0 | 0 |
| 60 | 0.00001 | 0.00008 | 0.902465 | 0.097445 | 0 | 0 | 0 | 0 | 0 |
| 61 | 0.00001 | 0.00005 | 0.899722 | 0.100218 | 0 | 0 | 0 | 0 | 0 |
| 62 | 0 | 0.00006 | 0.901962 | 0.097979 | 0 | 0 | 0 | 0 | 0 |
| 63 | 0.00002 | 0.00003 | 0.89983 | 0.10012 | 0 | 0 | 0 | 0 | 0 |
| 64 | 0.00001 | 0.00005 | 0.897504 | 0.102436 | 0 | 0 | 0 | 0 | 0 |
| 65 | 0 | 0.000079 | 0.901348 | 0.098573 | 0 | 0 | 0 | 0 | 0 |
| 66 | 0.00002 | 0.00008 | 0.903773 | 0.096126 | 0 | 0 | 0 | 0 | 0 |
| 67 | 0.00002 | 0.00004 | 0.903151 | 0.096789 | 0 | 0 | 0 | 0 | 0 |
| 68 | 0.00001 | 0.000089 | 0.900627 | 0.099274 | 0 | 0 | 0 | 0 | 0 |
| 69 | 0.00003 | 0.00006 | 0.899829 | 0.100081 | 0 | 0 | 0 | 0 | 0 |
| 70 | 0.00002 | 0.00008 | 0.901567 | 0.098333 | 0 | 0 | 0 | 0 | 0 |
| 71 | 0.00001 | 0.00008 | 0.898643 | 0.101117 | 2E-04 | 0 | 0 | 0 | 0 |
| 72 | 0 | 0.0001 | 0.903611 | 0.096289 | 0 | 0 | 0 | 0 | 0 |
| 73 | 0 | 0.000109 | 0.900759 | 0.099131 | 0 | 0 | 0 | 0 | 0 |
| 74 | 0.00001 | 0.00012 | 0.904768 | 0.095102 | 0 | 0 | 0 | 0 | 0 |
| 75 | 0.00001 | 0.00012 | 0.900944 | 0.098926 | 0 | 0 | 0 | 0 | 0 |
| 76 | 0.00001 | 0.00008 | 0.901847 | 0.098064 | 0 | 0 | 0 | 0 | 0 |
| 77 | 0.00001 | 0.000061 | 0.900904 | 0.099025 | 0 | 0 | 0 | 0 | 0 |
| 78 | 0.00001 | 0.00004 | 0.902218 | 0.097732 | 0 | 0 | 0 | 0 | 0 |
| 79 | 0.00001 | 0.00005 | 0.901449 | 0.09849 | 0 | 0 | 0 | 0 | 0 |
| 80 | 0.00001 | 0.00006 | 0.90104 | 0.09889 | 0 | 0 | 0 | 0 | 0 |
| 81 | 0.00001 | 0.0001 | 0.898126 | 0.101763 | 0 | 0 | 0 | 0 | 0 |
| 82 | 0.00001 | 0.00009 | 0.896857 | 0.103043 | 0 | 0 | 0 | 0 | 0 |
| 83 | 0.00002 | 0.00006 | 0.904755 | 0.095164 | 0 | 0 | 0 | 0 | 0 |
| 84 | 0.00001 | 0.00006 | 0.904793 | 0.095137 | 0 | 0 | 0 | 0 | 0 |
| 85 | 0.00001 | 0.000071 | 0.89907 | 0.100849 | 0 | 0 | 0 | 0 | 0 |
| 86 | 0.00001 | 0.00004 | 0.909099 | 0.090851 | 0 | 0 | 0 | 0 | 0 |
| 87 | 0.00001 | 0.000081 | 0.899747 | 0.100162 | 0 | 0 | 0 | 0 | 0 |
| 88 | 0.00002 | 0.00007 | 0.900278 | 0.099632 | 0 | 0 | 0 | 0 | 0 |
| 89 | 0.00001 | 0.000111 | 0.896953 | 0.102927 | 0 | 0 | 0 | 0 | 0 |
| 90 | 0.00002 | 0.00005 | 0.902283 | 0.097647 | 0 | 0 | 0 | 0 | 0 |
| 91 | 0 | 0.00006 | 0.901874 | 0.098067 | 0 | 0 | 0 | 0 | 0 |
| 92 | 0.00002 | 0.000121 | 0.899848 | 0.100011 | 0 | 0 | 0 | 0 | 0 |
| 93 | 0.00001 | 0.000108 | 0.904523 | 0.09536 | 0 | 0 | 0 | 0 | 0 |
| 94 | 0.00001 | 0.000101 | 0.902273 | 0.097617 | 0 | 0 | 0 | 0 | 0 |
| 95 | 0 | 0.0001 | 0.899736 | 0.100164 | 0 | 0 | 0 | 0 | 0 |
| 96 | 0.00001 | 0.00009 | 0.901648 | 0.098252 | 0 | 0 | 0 | 0 | 0 |
| 97 | 0 | 0.00002 | 0.897697 | 0.102283 | 0 | 0 | 0 | 0 | 0 |
| 98 | 0.00003 | 0.00008 | 0.900611 | 0.099279 | 0 | 0 | 0 | 0 | 0 |
| 99 | 0.00001 | 0.00004 | 0.896588 | 0.103362 | 0 | 0 | 0 | 0 | 0 |
| 100 | 0.00001 | 0.00003 | 0.903481 | 0.096479 | 0 | 0 | 0 | 0 | 0 |
| 101 | 0.00001 | 0.00007 | 0.901496 | 0.098424 | 0 | 0 | 0 | 0 | 0 |
| 102 | 0.00001 | 0.00009 | 0.904552 | 0.095348 | 0 | 0 | 0 | 0 | 0 |
| 103 | 0 | 0.00004 | 0.900201 | 0.099759 | 0 | 0 | 0 | 0 | 0 |
| 104 | 0.00002 | 0.00008 | 0.90532 | 0.09458 | 0 | 0 | 0 | 0 | 0 |
| 105 | 0.00001 | 0.00009 | 0.900539 | 0.099361 | 0 | 0 | 0 | 0 | 0 |
| 106 | 0.00001 | 0.00008 | 0.901365 | 0.098545 | 0 | 0 | 0 | 0 | 0 |
| 107 | 0.00001 | 0.000129 | 0.902567 | 0.097174 | 1E-04 | 0 | 0 | 0 | 0 |
| 108 | 0.00001 | 0.00006 | 0.89799 | 0.101939 | 0 | 0 | 0 | 0 | 0 |
| 109 | 0 | 0.000081 | 0.901792 | 0.098128 | 0 | 0 | 0 | 0 | 0 |
| 110 | 0.00001 | 0.00005 | 0.901539 | 0.098401 | 0 | 0 | 0 | 0 | 0 |
| 111 | 0.00001 | 0.000089 | 0.90504 | 0.094861 | 0 | 0 | 0 | 0 | 0 |
| 112 | 0.00002 | 0.00011 | 0.902118 | 0.097752 | 0 | 0 | 0 | 0 | 0 |
| 113 | 0.00004 | 0.00008 | 0.900292 | 0.099588 | 0 | 0 | 0 | 0 | 0 |
| 114 | 0.00002 | 0.00006 | 0.902256 | 0.097664 | 0 | 0 | 0 | 0 | 0 |
| 115 | 0.00005 | 0.00006 | 0.904069 | 0.095821 | 0 | 0 | 0 | 0 | 0 |
| 116 | 0.00004 | 0.00006 | 0.900743 | 0.099157 | 0 | 0 | 0 | 0 | 0 |
| 117 | 0.00003 | 0.00008 | 0.900822 | 0.099068 | 0 | 0 | 0 | 0 | 0 |
| 118 | 0 | 0.00004 | 0.903168 | 0.096792 | 0 | 0 | 0 | 0 | 0 |
| 119 | 0.00004 | 0.00004 | 0.900975 | 0.098944 | 0 | 0 | 0 | 0 | 0 |
| 120 | 0.00002 | 0.00006 | 0.901019 | 0.0989 | 0 | 0 | 0 | 0 | 0 |
| 121 | 0 | 0.00005 | 0.900702 | 0.099248 | 0 | 0 | 0 | 0 | 0 |
| 122 | 0.00002 | 0.00009 | 0.902691 | 0.097199 | 0 | 0 | 0 | 0 | 0 |
| 123 | 0 | 0.00003 | 0.902505 | 0.097465 | 0 | 0 | 0 | 0 | 0 |
| 124 | 0.00001 | 0.00007 | 0.904256 | 0.095663 | 0 | 0 | 0 | 0 | 0 |
| 125 | 0 | 0.00006 | 0.899828 | 0.100112 | 0 | 0 | 0 | 0 | 0 |
| 126 | 0.00002 | 0.00002 | 0.898949 | 0.101011 | 0 | 0 | 0 | 0 | 0 |
| 127 | 0 | 0.00002 | 0.900864 | 0.099116 | 0 | 0 | 0 | 0 | 0 |
| 128 | 0 | 0.00003 | 0.900528 | 0.099442 | 0 | 0 | 0 | 0 | 0 |
| 129 | 0.00001 | 0.00007 | 0.899325 | 0.100595 | 0 | 0 | 0 | 0 | 0 |
| 130 | 0.00002 | 0.00004 | 0.902055 | 0.097884 | 0 | 0 | 0 | 0 | 0 |
| 131 | 0 | 0.00008 | 0.902181 | 0.097739 | 0 | 0 | 0 | 0 | 0 |
| 132 | 0.00003 | 0.00004 | 0.904112 | 0.095818 | 0 | 0 | 0 | 0 | 0 |
| 133 | 0.00001 | 0.00004 | 0.901061 | 0.098889 | 0 | 0 | 0 | 0 | 0 |
| 134 | 0 | 0.00003 | 0.903619 | 0.096351 | 0 | 0 | 0 | 0 | 0 |
| 135 | 0.00001 | 0.000061 | 0.895371 | 0.104558 | 0 | 0 | 0 | 0 | 0 |
| 136 | 0.00001 | 0.00007 | 0.904726 | 0.095194 | 0 | 0 | 0 | 0 | 0 |
| 137 | 0.00001 | 0.00001 | 0.900955 | 0.099025 | 0 | 0 | 0 | 0 | 0 |
| 138 | 0 | 0.00004 | 0.895706 | 0.104254 | 0 | 0 | 0 | 0 | 0 |
| 139 | 0.00002 | 0.00005 | 0.90315 | 0.09678 | 0 | 0 | 0 | 0 | 0 |
| 140 | 0.00001 | 0.00005 | 0.897412 | 0.102528 | 0 | 0 | 0 | 0 | 0 |
| 141 | 0 | 0.00007 | 0.899323 | 0.100607 | 0 | 0 | 0 | 0 | 0 |
| 142 | 0.00004 | 0.00005 | 0.895143 | 0.104768 | 0 | 0 | 0 | 0 | 0 |
| 143 | 0.00004 | 0.00005 | 0.89942 | 0.100489 | 0 | 0 | 0 | 0 | 0 |
| 144 | 0 | 0.000159 | 0.898599 | 0.101242 | 0 | 0 | 0 | 0 | 0 |
| 145 | 0.00003 | 0.00007 | 0.902796 | 0.097103 | 0 | 0 | 0 | 0 | 0 |
| 146 | 0.00002 | 0.0001 | 0.903913 | 0.095966 | 0 | 0 | 0 | 0 | 0 |
| 147 | 0 | 0.00004 | 0.899687 | 0.100273 | 0 | 0 | 0 | 0 | 0 |
| 148 | 0.00001 | 0.00004 | 0.900803 | 0.099146 | 0 | 0 | 0 | 0 | 0 |
| 149 | 0.00002 | 0.000061 | 0.900801 | 0.099119 | 0 | 0 | 0 | 0 | 0 |
| 150 | 0 | 0.000095 | 0.905544 | 0.094361 | 0 | 0 | 0 | 0 | 0 |
| 151 | 0 | 0.00012 | 0.900751 | 0.099129 | 0 | 0 | 0 | 0 | 0 |
| 152 | 0.00001 | 0.00004 | 0.898185 | 0.101765 | 0 | 0 | 0 | 0 | 0 |
| 153 | 0.00001 | 0.00006 | 0.904565 | 0.095365 | 0 | 0 | 0 | 0 | 0 |
| 154 | 0.00001 | 0.00009 | 0.898866 | 0.101035 | 0 | 0 | 0 | 0 | 0 |
| 155 | 0.00001 | 0.00004 | 0.899178 | 0.100772 | 0 | 0 | 0 | 0 | 0 |
| 156 | 0.00002 | 0.00007 | 0.899103 | 0.100807 | 0 | 0 | 0 | 0 | 0 |
| 157 | 0.00002 | 0.00009 | 0.89888 | 0.101009 | 0 | 0 | 0 | 0 | 0 |
| 158 | 0.00002 | 0.00011 | 0.900532 | 0.099337 | 0 | 0 | 0 | 0 | 0 |
| 159 | 0.00002 | 0.00001 | 0.899886 | 0.100084 | 0 | 0 | 0 | 0 | 0 |
| 160 | 0.00001 | 0.000111 | 0.903169 | 0.09671 | 0 | 0 | 0 | 0 | 0 |
| 161 | 0.00001 | 0.00004 | 0.901505 | 0.098445 | 0 | 0 | 0 | 0 | 0 |
| 162 | 0.00002 | 0.00007 | 0.900894 | 0.099016 | 0 | 0 | 0 | 0 | 0 |
| 163 | 0.00001 | 0.00004 | 0.899945 | 0.100005 | 0 | 0 | 0 | 0 | 0 |
| 164 | 0.00001 | 0.00007 | 0.896664 | 0.103256 | 0 | 0 | 0 | 0 | 0 |
| 165 | 0 | 0.00007 | 0.90664 | 0.09329 | 0 | 0 | 0 | 0 | 0 |
| 166 | 0.00001 | 0.00007 | 0.901296 | 0.098624 | 0 | 0 | 0 | 0 | 0 |
| 167 | 0 | 0.00013 | 0.900683 | 0.099187 | 0 | 0 | 0 | 0 | 0 |
| 168 | 0.00001 | 0.0001 | 0.899963 | 0.099927 | 0 | 0 | 0 | 0 | 0 |
| 169 | 0.00001 | 0.00011 | 0.898854 | 0.101026 | 0 | 0 | 0 | 0 | 0 |
| 170 | 0.00003 | 0.00005 | 0.900183 | 0.099736 | 0 | 0 | 0 | 0 | 0 |
| 171 | 0.00002 | 0.00007 | 0.901215 | 0.098695 | 0 | 0 | 0 | 0 | 0 |
| 172 | 0 | 0.00009 | 0.900126 | 0.099784 | 0 | 0 | 0 | 0 | 0 |
| 173 | 0 | 0.0001 | 0.90321 | 0.09669 | 0 | 0 | 0 | 0 | 0 |
| 174 | 0.00001 | 0.00006 | 0.898394 | 0.101536 | 0 | 0 | 0 | 0 | 0 |
| 175 | 0.00001 | 0.00006 | 0.898591 | 0.101339 | 0 | 0 | 0 | 0 | 0 |
| 176 | 0.00003 | 0.000101 | 0.905502 | 0.094367 | 0 | 0 | 0 | 0 | 0 |
| 177 | 0.00002 | 0.000099 | 0.90112 | 0.09876 | 0 | 0 | 0 | 0 | 0 |
| 178 | 0 | 0.00009 | 0.899488 | 0.100422 | 0 | 0 | 0 | 0 | 0 |
| 179 | 0 | 0.000169 | 0.900867 | 0.098964 | 0 | 0 | 0 | 0 | 0 |
| 180 | 0.00002 | 0.00011 | 0.901084 | 0.098785 | 0 | 0 | 0 | 0 | 0 |
| 181 | 0 | 0.00006 | 0.90245 | 0.09749 | 0 | 0 | 0 | 0 | 0 |
| 182 | 0 | 0.000091 | 0.899208 | 0.100701 | 0 | 0 | 0 | 0 | 0 |
| 183 | 0.00001 | 0.00006 | 0.90397 | 0.09596 | 0 | 0 | 0 | 0 | 0 |
| 184 | 0 | 0.000081 | 0.903792 | 0.096128 | 0 | 0 | 0 | 0 | 0 |
| 185 | 0.00001 | 0.0001 | 0.902888 | 0.097001 | 0 | 0 | 0 | 0 | 0 |
| 186 | 0.00001 | 0.00007 | 0.899917 | 0.100003 | 0 | 0 | 0 | 0 | 0 |
| 187 | 0 | 0.00007 | 0.905183 | 0.094747 | 0 | 0 | 0 | 0 | 0 |
| 188 | 0.00002 | 0.00006 | 0.899261 | 0.100659 | 0 | 0 | 0 | 0 | 0 |
| 189 | 0.00001 | 0.00008 | 0.901027 | 0.098883 | 0 | 0 | 0 | 0 | 0 |
| 190 | 0.00002 | 0.00006 | 0.900887 | 0.099033 | 0 | 0 | 0 | 0 | 0 |
| 191 | 0.00001 | 0.00012 | 0.900635 | 0.099234 | 0 | 0 | 0 | 0 | 0 |
| 192 | 0.00001 | 0.00004 | 0.900462 | 0.099488 | 0 | 0 | 0 | 0 | 0 |
| 193 | 0.00001 | 0.00007 | 0.898269 | 0.101652 | 0 | 0 | 0 | 0 | 0 |
| 194 | 0.00002 | 0.000149 | 0.897959 | 0.101873 | 0 | 0 | 0 | 0 | 0 |
| 195 | 0.00002 | 0.00006 | 0.89872 | 0.1012 | 0 | 0 | 0 | 0 | 0 |
| 196 | 0 | 0.000139 | 0.900446 | 0.099415 | 0 | 0 | 0 | 0 | 0 |
| 197 | 0.00002 | 0.00005 | 0.905599 | 0.094331 | 0 | 0 | 0 | 0 | 0 |
| 198 | 0 | 0.0001 | 0.900498 | 0.099261 | 1E-04 | 0 | 0 | 0 | 0 |
| 199 | 0.00001 | 0.00006 | 0.904337 | 0.095593 | 0 | 0 | 0 | 0 | 0 |
| 200 | 0 | 0.000111 | 0.896928 | 0.102961 | 0 | 0 | 0 | 0 | 0 |
| 201 | 0.00001 | 0.00011 | 0.90075 | 0.099131 | 0 | 0 | 0 | 0 | 0 |
| 202 | 0.00001 | 0.00009 | 0.903054 | 0.096846 | 0 | 0 | 0 | 0 | 0 |
| 203 | 0.00002 | 0.00005 | 0.904948 | 0.094982 | 0 | 0 | 0 | 0 | 0 |
| 204 | 0.00002 | 0.000079 | 0.902459 | 0.097441 | 0 | 0 | 0 | 0 | 0 |
| 205 | 0.00001 | 0.000059 | 0.896438 | 0.103492 | 0 | 0 | 0 | 0 | 0 |
| 206 | 0.00001 | 0.00006 | 0.897459 | 0.102471 | 0 | 0 | 0 | 0 | 0 |
| 207 | 0.00002 | 0.00008 | 0.903935 | 0.095965 | 0 | 0 | 0 | 0 | 0 |
| 208 | 0.00002 | 0.00009 | 0.904481 | 0.095409 | 0 | 0 | 0 | 0 | 0 |
| 209 | 0.00002 | 0.00004 | 0.900015 | 0.099925 | 0 | 0 | 0 | 0 | 0 |
| 210 | 0.00002 | 0.00008 | 0.901739 | 0.098161 | 0 | 0 | 0 | 0 | 0 |
| 211 | 0 | 0.00005 | 0.897519 | 0.102431 | 0 | 0 | 0 | 0 | 0 |
| 212 | 0 | 0.00007 | 0.899812 | 0.100118 | 0 | 0 | 0 | 0 | 0 |
| 213 | 0.00001 | 0.00011 | 0.90155 | 0.09833 | 0 | 0 | 0 | 0 | 0 |
| 214 | 0.00003 | 0.00007 | 0.900961 | 0.098938 | 0 | 0 | 0 | 0 | 0 |
| 215 | 0.00002 | 0.00008 | 0.900897 | 0.099003 | 0 | 0 | 0 | 0 | 0 |
| 216 | 0.00001 | 0.0001 | 0.902842 | 0.097048 | 0 | 0 | 0 | 0 | 0 |
| 217 | 0 | 0.00005 | 0.900082 | 0.099868 | 0 | 0 | 0 | 0 | 0 |
| 218 | 0 | 0.00006 | 0.902527 | 0.097413 | 0 | 0 | 0 | 0 | 0 |
| 219 | 0.00002 | 0.00005 | 0.908186 | 0.091744 | 0 | 0 | 0 | 0 | 0 |
| 220 | 0.00001 | 0.00012 | 0.900725 | 0.099146 | 0 | 0 | 0 | 0 | 0 |
| 221 | 0.00003 | 0.00008 | 0.900125 | 0.099764 | 0 | 0 | 0 | 0 | 0 |
| 222 | 0 | 0.00007 | 0.900304 | 0.099626 | 0 | 0 | 0 | 0 | 0 |
| 223 | 0.00002 | 0.00013 | 0.900134 | 0.099716 | 0 | 0 | 0 | 0 | 0 |
| 224 | 0.00002 | 0.00008 | 0.899999 | 0.099901 | 0 | 0 | 0 | 0 | 0 |
| 225 | 0.00001 | 0.00008 | 0.904528 | 0.095382 | 0 | 0 | 0 | 0 | 0 |
| 226 | 0.00001 | 0.00001 | 0.901757 | 0.098223 | 0 | 0 | 0 | 0 | 0 |
| 227 | 0.00001 | 0.000091 | 0.900254 | 0.099646 | 0 | 0 | 0 | 0 | 0 |
| 228 | 0.00001 | 0.00007 | 0.904669 | 0.09525 | 0 | 0 | 0 | 0 | 0 |
| 229 | 0.00002 | 0.00013 | 0.902649 | 0.0972 | 0 | 0 | 0 | 0 | 0 |
| 230 | 0 | 0.000059 | 0.89984 | 0.100101 | 0 | 0 | 0 | 0 | 0 |
| 231 | 0 | 0.000211 | 0.902459 | 0.09733 | 0 | 0 | 0 | 0 | 0 |
| 232 | 0 | 0.00005 | 0.900568 | 0.099382 | 0 | 0 | 0 | 0 | 0 |
| 233 | 0.00001 | 0.0001 | 0.903044 | 0.096846 | 0 | 0 | 0 | 0 | 0 |
| 234 | 0.00001 | 0.00001 | 0.899467 | 0.100513 | 0 | 0 | 0 | 0 | 0 |
| 235 | 0.00001 | 0.00004 | 0.906099 | 0.093851 | 0 | 0 | 0 | 0 | 0 |
| 236 | 0.00001 | 0.00001 | 0.897801 | 0.102179 | 0 | 0 | 0 | 0 | 0 |
| 237 | 0.00002 | 0.00003 | 0.900297 | 0.099653 | 0 | 0 | 0 | 0 | 0 |
| 238 | 0.00002 | 0.00008 | 0.89806 | 0.10184 | 0 | 0 | 0 | 0 | 0 |
| 239 | 0.00001 | 0.00006 | 0.897911 | 0.102019 | 0 | 0 | 0 | 0 | 0 |
| 240 | 0.00001 | 0.00006 | 0.901909 | 0.098021 | 0 | 0 | 0 | 0 | 0 |
| 241 | 0.00003 | 0.00006 | 0.902049 | 0.097861 | 0 | 0 | 0 | 0 | 0 |
| 242 | 0.00001 | 0.00009 | 0.900275 | 0.099625 | 0 | 0 | 0 | 0 | 0 |
| 243 | 0.00004 | 0.00005 | 0.906322 | 0.093588 | 0 | 0 | 0 | 0 | 0 |
| 244 | 0 | 0.00006 | 0.899032 | 0.100908 | 0 | 0 | 0 | 0 | 0 |
| 245 | 0.00003 | 0.000141 | 0.899234 | 0.100544 | 5E-05 | 0 | 0 | 0 | 0 |
| 246 | 0.00002 | 0.000141 | 0.897863 | 0.101976 | 0 | 0 | 0 | 0 | 0 |
| 247 | 0.00001 | 0.00008 | 0.8981 | 0.10181 | 0 | 0 | 0 | 0 | 0 |
| 248 | 0.00001 | 0.000139 | 0.902555 | 0.097295 | 0 | 0 | 0 | 0 | 0 |
| 249 | 0.00001 | 0.00005 | 0.902404 | 0.097536 | 0 | 0 | 0 | 0 | 0 |
| 250 | 0.00001 | 0.00007 | 0.898102 | 0.101819 | 0 | 0 | 0 | 0 | 0 |
| 251 | 0.00002 | 0.00007 | 0.898164 | 0.101746 | 0 | 0 | 0 | 0 | 0 |
| 252 | 0.00001 | 0.00004 | 0.903028 | 0.096922 | 0 | 0 | 0 | 0 | 0 |
| 253 | 0.00003 | 0.00005 | 0.901678 | 0.098243 | 0 | 0 | 0 | 0 | 0 |
| 254 | 0 | 0.00003 | 0.898234 | 0.101736 | 0 | 0 | 0 | 0 | 0 |
| 255 | 0.00004 | 0.00005 | 0.900605 | 0.099305 | 0 | 0 | 0 | 0 | 0 |
| 256 | 0.00005 | 0.000109 | 0.900995 | 0.098846 | 0 | 0 | 0 | 0 | 0 |
| 257 | 0 | 0.00013 | 0.899972 | 0.099898 | 0 | 0 | 0 | 0 | 0 |
| 258 | 0.00002 | 0.00006 | 0.897779 | 0.102141 | 0 | 0 | 0 | 0 | 0 |
| 259 | 0 | 0.000139 | 0.899136 | 0.100725 | 0 | 0 | 0 | 0 | 0 |
| 260 | 0.00002 | 0.00005 | 0.903006 | 0.096924 | 0 | 0 | 0 | 0 | 0 |
| 261 | 0 | 0.00002 | 0.904591 | 0.095389 | 0 | 0 | 0 | 0 | 0 |
| 262 | 0.00001 | 0.000111 | 0.9007 | 0.099179 | 0 | 0 | 0 | 0 | 0 |
| 263 | 0 | 0.000091 | 0.89919 | 0.10072 | 0 | 0 | 0 | 0 | 0 |
| 264 | 0.00001 | 0.00003 | 0.897386 | 0.102574 | 0 | 0 | 0 | 0 | 0 |
| 265 | 0 | 0.00005 | 0.898607 | 0.101343 | 0 | 0 | 0 | 0 | 0 |
| 266 | 0.00003 | 0.000099 | 0.903309 | 0.096562 | 0 | 0 | 0 | 0 | 0 |
| 267 | 0.00002 | 0.00002 | 0.900963 | 0.098997 | 0 | 0 | 0 | 0 | 0 |
| 268 | 0.00001 | 0.00004 | 0.903538 | 0.096412 | 0 | 0 | 0 | 0 | 0 |
| 269 | 0.00002 | 0.00003 | 0.903353 | 0.096598 | 0 | 0 | 0 | 0 | 0 |
| 270 | 0.00002 | 0.00012 | 0.902933 | 0.096928 | 0 | 0 | 0 | 0 | 0 |
| 271 | 0.00002 | 0.00004 | 0.899966 | 0.099974 | 0 | 0 | 0 | 0 | 0 |
| 272 | 0.00001 | 0.00006 | 0.899116 | 0.100813 | 0 | 0 | 0 | 0 | 0 |
| 273 | 0 | 0.00004 | 0.904875 | 0.095085 | 0 | 0 | 0 | 0 | 0 |
| 274 | 0.00001 | 0.000149 | 0.90478 | 0.095061 | 0 | 0 | 0 | 0 | 0 |
| 275 | 0.00001 | 0.000099 | 0.902096 | 0.097794 | 0 | 0 | 0 | 0 | 0 |
| 276 | 0 | 0.00003 | 0.901032 | 0.098938 | 0 | 0 | 0 | 0 | 0 |
| 277 | 0.00002 | 0.000091 | 0.895662 | 0.104227 | 0 | 0 | 0 | 0 | 0 |
| 278 | 0 | 0.00006 | 0.901759 | 0.098182 | 0 | 0 | 0 | 0 | 0 |
| 279 | 0 | 0.00007 | 0.899167 | 0.100763 | 0 | 0 | 0 | 0 | 0 |
| 280 | 0.00001 | 0.0001 | 0.897733 | 0.102157 | 0 | 0 | 0 | 0 | 0 |
| 281 | 0.00003 | 0.00005 | 0.898574 | 0.101346 | 0 | 0 | 0 | 0 | 0 |
| 282 | 0.00002 | 0.0001 | 0.903167 | 0.096713 | 0 | 0 | 0 | 0 | 0 |
| 283 | 0.00001 | 0.0001 | 0.902984 | 0.096906 | 0 | 0 | 0 | 0 | 0 |
| 284 | 0.00001 | 0.00003 | 0.906719 | 0.093241 | 0 | 0 | 0 | 0 | 0 |
| 285 | 0.00001 | 0.00006 | 0.897428 | 0.102502 | 0 | 0 | 0 | 0 | 0 |
| 286 | 0.00002 | 0.00006 | 0.904089 | 0.095831 | 0 | 0 | 0 | 0 | 0 |
| 287 | 0.00003 | 0.00006 | 0.902637 | 0.097273 | 0 | 0 | 0 | 0 | 0 |
| 288 | 0.00002 | 0.0001 | 0.900014 | 0.099866 | 0 | 0 | 0 | 0 | 0 |
| 289 | 0.00003 | 0.00009 | 0.903225 | 0.096654 | 0 | 0 | 0 | 0 | 0 |
| 290 | 0 | 0.00008 | 0.899127 | 0.100792 | 0 | 0 | 0 | 0 | 0 |
| 291 | 0.00001 | 0.00004 | 0.9003 | 0.09965 | 0 | 0 | 0 | 0 | 0 |
| 292 | 0.00002 | 0.00002 | 0.898804 | 0.101156 | 0 | 0 | 0 | 0 | 0 |
| 293 | 0.00002 | 0.00007 | 0.896238 | 0.103672 | 0 | 0 | 0 | 0 | 0 |
| 294 | 0.00001 | 0.00011 | 0.899406 | 0.100474 | 0 | 0 | 0 | 0 | 0 |
| 295 | 0 | 0.00002 | 0.902997 | 0.096983 | 0 | 0 | 0 | 0 | 0 |
| 296 | 0.00002 | 0.00001 | 0.901617 | 0.098353 | 0 | 0 | 0 | 0 | 0 |
| 297 | 0 | 0.00007 | 0.898736 | 0.101194 | 0 | 0 | 0 | 0 | 0 |
| 298 | 0 | 0.00008 | 0.901493 | 0.098427 | 0 | 0 | 0 | 0 | 0 |
| 299 | 0 | 0.00001 | 0.896063 | 0.103927 | 0 | 0 | 0 | 0 | 0 |
| 300 | 0 | 0.000089 | 0.899319 | 0.100592 | 0 | 0 | 0 | 0 | 0 |
| 301 | 0.00001 | 0.00009 | 0.899888 | 0.100012 | 0 | 0 | 0 | 0 | 0 |
| 302 | 0.00001 | 0.00004 | 0.898651 | 0.101299 | 0 | 0 | 0 | 0 | 0 |
| 303 | 0 | 0.00008 | 0.899858 | 0.100062 | 0 | 0 | 0 | 0 | 0 |
| 304 | 0.00002 | 0.00007 | 0.89754 | 0.102369 | 0 | 0 | 0 | 0 | 0 |
| 305 | 0.00001 | 0.00009 | 0.902554 | 0.097346 | 0 | 0 | 0 | 0 | 0 |
| 306 | 0.00001 | 0.00009 | 0.902722 | 0.097178 | 0 | 0 | 0 | 0 | 0 |
| 307 | 0 | 0.000071 | 0.902439 | 0.09749 | 0 | 0 | 0 | 0 | 0 |
| 308 | 0.00002 | 0.000141 | 0.903054 | 0.096785 | 0 | 0 | 0 | 0 | 0 |
| 309 | 0.00003 | 0.000069 | 0.902124 | 0.097776 | 0 | 0 | 0 | 0 | 0 |
| 310 | 0 | 0.00007 | 0.899864 | 0.100066 | 0 | 0 | 0 | 0 | 0 |
| 311 | 0.00001 | 0.000119 | 0.899471 | 0.100399 | 0 | 0 | 0 | 0 | 0 |
| 312 | 0 | 0.00004 | 0.897729 | 0.102231 | 0 | 0 | 0 | 0 | 0 |
| 313 | 0 | 0.00012 | 0.900569 | 0.099311 | 0 | 0 | 0 | 0 | 0 |
| 314 | 0.00001 | 0.00003 | 0.901153 | 0.098807 | 0 | 0 | 0 | 0 | 0 |
| 315 | 0.00004 | 0.00009 | 0.899664 | 0.100206 | 0 | 0 | 0 | 0 | 0 |
| 316 | 0.00001 | 0.00003 | 0.899141 | 0.10082 | 0 | 0 | 0 | 0 | 0 |
| 317 | 0.00001 | 0.00011 | 0.901271 | 0.09861 | 0 | 0 | 0 | 0 | 0 |
| 318 | 0.00001 | 0.00002 | 0.895741 | 0.104209 | 2E-05 | 0 | 0 | 0 | 0 |
| 319 | 0.00001 | 0.00007 | 0.897837 | 0.102083 | 0 | 0 | 0 | 0 | 0 |
| 320 | 0.00001 | 0.00008 | 0.901198 | 0.098711 | 0 | 0 | 0 | 0 | 0 |
| 321 | 0.00001 | 0.00006 | 0.899539 | 0.100391 | 0 | 0 | 0 | 0 | 0 |
| 322 | 0.00002 | 0.00009 | 0.902183 | 0.097707 | 0 | 0 | 0 | 0 | 0 |
| 323 | 0.00001 | 0.00014 | 0.901828 | 0.098022 | 0 | 0 | 0 | 0 | 0 |
| 324 | 0.00002 | 0.00007 | 0.901166 | 0.098745 | 0 | 0 | 0 | 0 | 0 |
| 325 | 0 | 0.00005 | 0.902311 | 0.097639 | 0 | 0 | 0 | 0 | 0 |
| 326 | 0 | 0.00007 | 0.902272 | 0.097658 | 0 | 0 | 0 | 0 | 0 |
| 327 | 0 | 0.00013 | 0.900474 | 0.099395 | 0 | 0 | 0 | 0 | 0 |
| 328 | 0 | 0.00005 | 0.901192 | 0.098758 | 0 | 0 | 0 | 0 | 0 |
| 329 | 0 | 0.000099 | 0.899562 | 0.100338 | 0 | 0 | 0 | 0 | 0 |
| 330 | 0 | 0.00004 | 0.901251 | 0.098709 | 0 | 0 | 0 | 0 | 0 |
| 331 | 0.00001 | 0.00004 | 0.901968 | 0.097982 | 0 | 0 | 0 | 0 | 0 |
| 332 | 0 | 0.00006 | 0.900973 | 0.098967 | 0 | 0 | 0 | 0 | 0 |
| 333 | 0.00002 | 0.00008 | 0.896121 | 0.10378 | 0 | 0 | 0 | 0 | 0 |
| 334 | 0 | 0.00008 | 0.900889 | 0.099031 | 0 | 0 | 0 | 0 | 0 |
| 335 | 0.00001 | 0.00002 | 0.901211 | 0.09876 | 0 | 0 | 0 | 0 | 0 |
| 336 | 0.00004 | 0.000121 | 0.896414 | 0.103425 | 0 | 0 | 0 | 0 | 0 |
| 337 | 0.00001 | 0.00007 | 0.90323 | 0.09669 | 0 | 0 | 0 | 0 | 0 |
| 338 | 0.00003 | 0.00005 | 0.903989 | 0.095931 | 0 | 0 | 0 | 0 | 0 |
| 339 | 0.00002 | 0.000089 | 0.90121 | 0.09868 | 0 | 0 | 0 | 0 | 0 |
| 340 | 0 | 0.00008 | 0.898195 | 0.101725 | 0 | 0 | 0 | 0 | 0 |
| 341 | 0.00001 | 0.00003 | 0.898765 | 0.101195 | 0 | 0 | 0 | 0 | 0 |
| 342 | 0 | 0.00003 | 0.897748 | 0.102222 | 0 | 0 | 0 | 0 | 0 |
| 343 | 0 | 0.0001 | 0.898966 | 0.100934 | 0 | 0 | 0 | 0 | 0 |
| 344 | 0.00001 | 0.00005 | 0.902069 | 0.097871 | 0 | 0 | 0 | 0 | 0 |
| 345 | 0.00003 | 0.000111 | 0.903971 | 0.095888 | 0 | 0 | 0 | 0 | 0 |
| 346 | 0.00002 | 0.000089 | 0.905008 | 0.094883 | 0 | 0 | 0 | 0 | 0 |
| 347 | 0 | 0.00009 | 0.898648 | 0.101262 | 0 | 0 | 0 | 0 | 0 |
| 348 | 0 | 0.00007 | 0.900523 | 0.099407 | 0 | 0 | 0 | 0 | 0 |
| 349 | 0 | 0.00009 | 0.900269 | 0.099641 | 0 | 0 | 0 | 0 | 0 |
| 350 | 0.00002 | 0.00009 | 0.901664 | 0.098226 | 0 | 0 | 0 | 0 | 0 |
| 351 | 0 | 0.00004 | 0.899716 | 0.100244 | 0 | 0 | 0 | 0 | 0 |
| 352 | 0.00001 | 0.00002 | 0.902483 | 0.097487 | 0 | 0 | 0 | 0 | 0 |
| 353 | 0 | 0.000101 | 0.901591 | 0.098308 | 0 | 0 | 0 | 0 | 0 |
| 354 | 0.00001 | 0.00005 | 0.899491 | 0.100449 | 0 | 0 | 0 | 0 | 0 |
| 355 | 0.00002 | 0.00003 | 0.904854 | 0.095097 | 0 | 0 | 0 | 0 | 0 |
| 356 | 0.00001 | 0.00001 | 0.903192 | 0.096788 | 0 | 0 | 0 | 0 | 0 |
| 357 | 0.00002 | 0.00009 | 0.896215 | 0.103676 | 0 | 0 | 0 | 0 | 0 |
| 358 | 0 | 0.00008 | 0.901657 | 0.098263 | 0 | 0 | 0 | 0 | 0 |
| 359 | 0.00001 | 0.000052 | 0.898382 | 0.101557 | 0 | 0 | 0 | 0 | 0 |
| 360 | 0.00002 | 0.00009 | 0.902931 | 0.096959 | 0 | 0 | 0 | 0 | 0 |
| 361 | 0.00001 | 0.00004 | 0.899058 | 0.100892 | 0 | 0 | 0 | 0 | 0 |
| 362 | 0.00001 | 0.00006 | 0.901058 | 0.098873 | 0 | 0 | 0 | 0 | 0 |
| 363 | 0.00001 | 0.00008 | 0.902054 | 0.097857 | 0 | 0 | 0 | 0 | 0 |
| 364 | 0.00001 | 0.00008 | 0.903902 | 0.096008 | 0 | 0 | 0 | 0 | 0 |
| 365 | 0.00001 | 0.00009 | 0.901026 | 0.098874 | 0 | 0 | 0 | 0 | 0 |
| 366 | 0.00003 | 0.00003 | 0.901276 | 0.098665 | 0 | 0 | 0 | 0 | 0 |
| 367 | 0.00004 | 0.00007 | 0.898633 | 0.101256 | 0 | 0 | 0 | 0 | 0 |
| 368 | 0.00001 | 0.000091 | 0.900625 | 0.099274 | 0 | 0 | 0 | 0 | 0 |
| 369 | 0.00001 | 0.00005 | 0.899319 | 0.100621 | 0 | 0 | 0 | 0 | 0 |
| 370 | 0.00002 | 0.00009 | 0.900788 | 0.099102 | 0 | 0 | 0 | 0 | 0 |
| 371 | 0.00002 | 0.00007 | 0.901083 | 0.098787 | 4E-05 | 0 | 0 | 0 | 0 |
| 372 | 0.00001 | 0.0001 | 0.897687 | 0.102203 | 0 | 0 | 0 | 0 | 0 |
| 373 | 0 | 0.00003 | 0.896653 | 0.103317 | 0 | 0 | 0 | 0 | 0 |
| 374 | 0.00002 | 0.00006 | 0.903346 | 0.096574 | 0 | 0 | 0 | 0 | 0 |
| 375 | 0.00001 | 0.0001 | 0.897873 | 0.102018 | 0 | 0 | 0 | 0 | 0 |
| 376 | 0.00002 | 0.000113 | 0.897745 | 0.102122 | 0 | 0 | 0 | 0 | 0 |
| 377 | 0.00002 | 0.00006 | 0.897489 | 0.102431 | 0 | 0 | 0 | 0 | 0 |
| 378 | 0.00002 | 0.00008 | 0.902024 | 0.097876 | 0 | 0 | 0 | 0 | 0 |
| 379 | 0.00003 | 0.00002 | 0.903407 | 0.096544 | 0 | 0 | 0 | 0 | 0 |
| 380 | 0 | 0.00004 | 0.895712 | 0.104248 | 0 | 0 | 0 | 0 | 0 |
| 381 | 0 | 0.000059 | 0.902013 | 0.097927 | 0 | 0 | 0 | 0 | 0 |
| 382 | 0 | 0.00008 | 0.901273 | 0.098647 | 0 | 0 | 0 | 0 | 0 |
| 383 | 0.00001 | 0.00011 | 0.899053 | 0.100826 | 0 | 0 | 0 | 0 | 0 |
| 384 | 0 | 0.00007 | 0.896883 | 0.103047 | 0 | 0 | 0 | 0 | 0 |
| 385 | 0 | 0.00007 | 0.902881 | 0.097049 | 0 | 0 | 0 | 0 | 0 |
| 386 | 0.00001 | 0.00002 | 0.899175 | 0.100796 | 0 | 0 | 0 | 0 | 0 |
| 387 | 0.00001 | 0.00008 | 0.899791 | 0.100119 | 0 | 0 | 0 | 0 | 0 |
| 388 | 0.00003 | 0.00006 | 0.904342 | 0.095568 | 0 | 0 | 0 | 0 | 0 |
| 389 | 0.00002 | 0.000121 | 0.90255 | 0.097309 | 0 | 0 | 0 | 0 | 0 |
| 390 | 0 | 0.00014 | 0.900307 | 0.099553 | 0 | 0 | 0 | 0 | 0 |
| 391 | 0.00001 | 0.00007 | 0.894948 | 0.104972 | 0 | 0 | 0 | 0 | 0 |
| 392 | 0 | 0.00004 | 0.901909 | 0.098051 | 0 | 0 | 0 | 0 | 0 |
| 393 | 0 | 0.00005 | 0.897732 | 0.102217 | 0 | 0 | 0 | 0 | 0 |
| 394 | 0.00002 | 0.00009 | 0.903777 | 0.096114 | 0 | 0 | 0 | 0 | 0 |
| 395 | 0.00002 | 0.00004 | 0.904083 | 0.095856 | 0 | 0 | 0 | 0 | 0 |
| 396 | 0.00002 | 0.000061 | 0.901065 | 0.098854 | 0 | 0 | 0 | 0 | 0 |
| 397 | 0.00005 | 0.00011 | 0.901328 | 0.098513 | 0 | 0 | 0 | 0 | 0 |
| 398 | 0.00001 | 0.000129 | 0.90228 | 0.09758 | 0 | 0 | 0 | 0 | 0 |
| 399 | 0.00003 | 0.00004 | 0.900113 | 0.099817 | 0 | 0 | 0 | 0 | 0 |
| 400 | 0.00001 | 0.00008 | 0.904759 | 0.095151 | 0 | 0 | 0 | 0 | 0 |
| 401 | 0.00001 | 0.00007 | 0.899991 | 0.099929 | 0 | 0 | 0 | 0 | 0 |
| 402 | 0.00001 | 0.000091 | 0.900271 | 0.099628 | 0 | 0 | 0 | 0 | 0 |
| 403 | 0 | 0.00007 | 0.900939 | 0.098991 | 0 | 0 | 0 | 0 | 0 |
| 404 | 0.00002 | 0.00007 | 0.899977 | 0.099933 | 0 | 0 | 0 | 0 | 0 |
| 405 | 0.00003 | 0.00004 | 0.902352 | 0.097578 | 0 | 0 | 0 | 0 | 0 |
| 406 | 0 | 0.000111 | 0.900807 | 0.099082 | 0 | 0 | 0 | 0 | 0 |
| 407 | 0.00002 | 0.00012 | 0.898984 | 0.100876 | 0 | 0 | 0 | 0 | 0 |
| 408 | 0.00001 | 0.0001 | 0.898216 | 0.101674 | 0 | 0 | 0 | 0 | 0 |
| 409 | 0 | 0.00007 | 0.897068 | 0.102862 | 0 | 0 | 0 | 0 | 0 |
| 410 | 0.00001 | 0.00005 | 0.898646 | 0.101294 | 0 | 0 | 0 | 0 | 0 |
| 411 | 0 | 0.00007 | 0.899809 | 0.100121 | 0 | 0 | 0 | 0 | 0 |
| 412 | 0.00001 | 0.000111 | 0.899281 | 0.100599 | 0 | 0 | 0 | 0 | 0 |
| 413 | 0.00001 | 0.00008 | 0.898836 | 0.101073 | 0 | 0 | 0 | 0 | 0 |
| 414 | 0.00001 | 0.00005 | 0.900412 | 0.099528 | 0 | 0 | 0 | 0 | 0 |
| 415 | 0.00006 | 0.00007 | 0.898841 | 0.101029 | 0 | 0 | 0 | 0 | 0 |
| 416 | 0.00001 | 0.00015 | 0.899996 | 0.099844 | 0 | 0 | 0 | 0 | 0 |
| 417 | 0.00001 | 0.00007 | 0.901166 | 0.098753 | 0 | 0 | 0 | 0 | 0 |
| 418 | 0.00001 | 0.00005 | 0.904621 | 0.095318 | 0 | 0 | 0 | 0 | 0 |
| 419 | 0 | 0.00005 | 0.898128 | 0.101822 | 0 | 0 | 0 | 0 | 0 |
| 420 | 0.00003 | 0.000081 | 0.904416 | 0.095473 | 0 | 0 | 0 | 0 | 0 |
| 421 | 0 | 0.00004 | 0.90107 | 0.098891 | 0 | 0 | 0 | 0 | 0 |
| 422 | 0 | 0.00015 | 0.902904 | 0.096946 | 0 | 0 | 0 | 0 | 0 |
| 423 | 0 | 0.00007 | 0.904323 | 0.095607 | 0 | 0 | 0 | 0 | 0 |
| 424 | 0 | 0.00007 | 0.904301 | 0.095629 | 0 | 0 | 0 | 0 | 0 |
| 425 | 0.00005 | 0.00008 | 0.898781 | 0.101089 | 0 | 0 | 0 | 0 | 0 |
| 426 | 0 | 0.00007 | 0.898814 | 0.101116 | 0 | 0 | 0 | 0 | 0 |
| 427 | 0.00003 | 0.000121 | 0.901674 | 0.098176 | 0 | 0 | 0 | 0 | 0 |
| 428 | 0.00003 | 0.00003 | 0.903911 | 0.096029 | 0 | 0 | 0 | 0 | 0 |
| 429 | 0.00003 | 0.00007 | 0.903497 | 0.096403 | 0 | 0 | 0 | 0 | 0 |
| 430 | 0.00001 | 0.000091 | 0.900928 | 0.098971 | 0 | 0 | 0 | 0 | 0 |
| 431 | 0 | 0.00003 | 0.902099 | 0.097871 | 0 | 0 | 0 | 0 | 0 |
| 432 | 0.00001 | 0.0001 | 0.895345 | 0.104545 | 0 | 0 | 0 | 0 | 0 |
| 433 | 0 | 0.00005 | 0.901075 | 0.098875 | 0 | 0 | 0 | 0 | 0 |
| 434 | 0 | 0.00003 | 0.903202 | 0.096768 | 0 | 0 | 0 | 0 | 0 |
| 435 | 0.00001 | 0.00005 | 0.90056 | 0.09938 | 0 | 0 | 0 | 0 | 0 |
| 436 | 0 | 0.000021 | 0.892081 | 0.107898 | 0 | 0 | 0 | 0 | 0 |
| 437 | 0.00002 | 0.00008 | 0.900493 | 0.099407 | 0 | 0 | 0 | 0 | 0 |
| 438 | 0.00001 | 0.00009 | 0.900123 | 0.099777 | 0 | 0 | 0 | 0 | 0 |
| 439 | 0.00001 | 0.00007 | 0.901772 | 0.098148 | 0 | 0 | 0 | 0 | 0 |
| 440 | 0.00001 | 0.00001 | 0.902321 | 0.097659 | 0 | 0 | 0 | 0 | 0 |
| 441 | 0.00002 | 0.00007 | 0.898365 | 0.101545 | 0 | 0 | 0 | 0 | 0 |
| 442 | 0.00001 | 0.00005 | 0.899823 | 0.100117 | 0 | 0 | 0 | 0 | 0 |
| 443 | 0.00001 | 0.00005 | 0.903188 | 0.096752 | 0 | 0 | 0 | 0 | 0 |
| 444 | 0.00002 | 0.00008 | 0.896199 | 0.103701 | 0 | 0 | 0 | 0 | 0 |
| 445 | 0.00002 | 0.00009 | 0.897125 | 0.102766 | 0 | 0 | 0 | 0 | 0 |
| 446 | 0.00001 | 0.00001 | 0.902447 | 0.097533 | 0 | 0 | 0 | 0 | 0 |
| 447 | 0.00001 | 0.00005 | 0.903175 | 0.096765 | 0 | 0 | 0 | 0 | 0 |
| 448 | 0.00001 | 0.00014 | 0.899714 | 0.100136 | 0 | 0 | 0 | 0 | 0 |
| 449 | 0.00002 | 0.00003 | 0.903442 | 0.096508 | 0 | 0 | 0 | 0 | 0 |
| 450 | 0.00001 | 0.00002 | 0.901443 | 0.098528 | 0 | 0 | 0 | 0 | 0 |
| 451 | 0.00001 | 0.000089 | 0.905703 | 0.094198 | 0 | 0 | 0 | 0 | 0 |
| 452 | 0.00001 | 0.00004 | 0.899203 | 0.100747 | 0 | 0 | 0 | 0 | 0 |
| 453 | 0.00001 | 0.00008 | 0.900296 | 0.099614 | 0 | 0 | 0 | 0 | 0 |
| 454 | 0 | 0.00006 | 0.901782 | 0.098158 | 0 | 0 | 0 | 0 | 0 |
| 455 | 0 | 0.00006 | 0.905586 | 0.094354 | 0 | 0 | 0 | 0 | 0 |
| 456 | 0.00002 | 0.00006 | 0.899535 | 0.100385 | 0 | 0 | 0 | 0 | 0 |
| 457 | 0.00001 | 0.00005 | 0.900989 | 0.098951 | 0 | 0 | 0 | 0 | 0 |
| 458 | 0 | 0.00003 | 0.901564 | 0.098406 | 0 | 0 | 0 | 0 | 0 |
| 459 | 0 | 0.00003 | 0.896014 | 0.103956 | 0 | 0 | 0 | 0 | 0 |
| 460 | 0.00002 | 0.00007 | 0.896555 | 0.103354 | 0 | 0 | 0 | 0 | 0 |
| 461 | 0.00003 | 0.00003 | 0.900786 | 0.099155 | 0 | 0 | 0 | 0 | 0 |
| 462 | 0 | 0.00002 | 0.9078 | 0.09218 | 0 | 0 | 0 | 0 | 0 |
| 463 | 0 | 0.00005 | 0.899106 | 0.100844 | 0 | 0 | 0 | 0 | 0 |
| 464 | 0.00001 | 0.00003 | 0.902066 | 0.097725 | 2E-04 | 0 | 0 | 0 | 0 |
| 465 | 0 | 0.00009 | 0.901479 | 0.098432 | 0 | 0 | 0 | 0 | 0 |
| 466 | 0.00002 | 0.00007 | 0.897402 | 0.102507 | 0 | 0 | 0 | 0 | 0 |
| 467 | 0 | 0.00004 | 0.894881 | 0.105079 | 0 | 0 | 0 | 0 | 0 |
| 468 | 0.00001 | 0.00007 | 0.902575 | 0.097345 | 0 | 0 | 0 | 0 | 0 |
| 469 | 0 | 0.00006 | 0.899729 | 0.100211 | 0 | 0 | 0 | 0 | 0 |
| 470 | 0 | 0.00003 | 0.902741 | 0.097228 | 0 | 0 | 0 | 0 | 0 |
| 471 | 0.00002 | 0.000099 | 0.902913 | 0.096968 | 0 | 0 | 0 | 0 | 0 |
| 472 | 0 | 0.00004 | 0.901481 | 0.098479 | 0 | 0 | 0 | 0 | 0 |
| 473 | 0.00001 | 0.00009 | 0.900671 | 0.099229 | 0 | 0 | 0 | 0 | 0 |
| 474 | 0.00001 | 0.00005 | 0.900739 | 0.099201 | 0 | 0 | 0 | 0 | 0 |
| 475 | 0.00001 | 0.00004 | 0.900403 | 0.099547 | 0 | 0 | 0 | 0 | 0 |
| 476 | 0.00002 | 0.000081 | 0.894579 | 0.10532 | 0 | 0 | 0 | 0 | 0 |
| 477 | 0 | 0.00007 | 0.901669 | 0.098261 | 0 | 0 | 0 | 0 | 0 |
| 478 | 0 | 0.00006 | 0.900501 | 0.099439 | 0 | 0 | 0 | 0 | 0 |
| 479 | 0 | 0.00004 | 0.896475 | 0.103485 | 0 | 0 | 0 | 0 | 0 |
| 480 | 0 | 0.00014 | 0.897404 | 0.102457 | 0 | 0 | 0 | 0 | 0 |
| 481 | 0.00003 | 0.000071 | 0.905255 | 0.094644 | 0 | 0 | 0 | 0 | 0 |
| 482 | 0 | 0.00007 | 0.898781 | 0.101149 | 0 | 0 | 0 | 0 | 0 |
| 483 | 0 | 0.00003 | 0.901299 | 0.09867 | 0 | 0 | 0 | 0 | 0 |
| 484 | 0.00001 | 0.00003 | 0.89453 | 0.105429 | 0 | 0 | 0 | 0 | 0 |
| 485 | 0 | 0.00006 | 0.906686 | 0.093254 | 0 | 0 | 0 | 0 | 0 |
| 486 | 0.00002 | 0.00006 | 0.900944 | 0.098976 | 0 | 0 | 0 | 0 | 0 |
| 487 | 0 | 0.00004 | 0.901053 | 0.098907 | 0 | 0 | 0 | 0 | 0 |
| 488 | 0 | 0.00006 | 0.900241 | 0.099699 | 0 | 0 | 0 | 0 | 0 |
| 489 | 0.00001 | 0.00005 | 0.902807 | 0.097133 | 0 | 0 | 0 | 0 | 0 |
| 490 | 0.00001 | 0.00009 | 0.903995 | 0.095905 | 0 | 0 | 0 | 0 | 0 |
| 491 | 0.00003 | 0.000081 | 0.898262 | 0.101627 | 0 | 0 | 0 | 0 | 0 |
| 492 | 0.00002 | 0.00007 | 0.896747 | 0.103162 | 0 | 0 | 0 | 0 | 0 |
| 493 | 0.00001 | 0.00012 | 0.898432 | 0.101438 | 0 | 0 | 0 | 0 | 0 |
| 494 | 0.00002 | 0.00003 | 0.900101 | 0.099849 | 0 | 0 | 0 | 0 | 0 |
| 495 | 0.00003 | 0.00004 | 0.899335 | 0.100595 | 0 | 0 | 0 | 0 | 0 |
| 496 | 0 | 0.000089 | 0.898608 | 0.101263 | 4E-05 | 0 | 0 | 0 | 0 |
| 497 | 0.00001 | 0.00012 | 0.90099 | 0.09888 | 0 | 0 | 0 | 0 | 0 |
| 498 | 0.00001 | 0.00008 | 0.905352 | 0.094558 | 0 | 0 | 0 | 0 | 0 |
| 499 | 0 | 0.00002 | 0.900385 | 0.099595 | 0 | 0 | 0 | 0 | 0 |
| 500 | 0 | 0.000081 | 0.902779 | 0.097141 | 0 | 0 | 0 | 0 | 0 |
| 501 | 0.00001 | 0.00005 | 0.9021 | 0.09784 | 0 | 0 | 0 | 0 | 0 |
| 502 | 0.00002 | 0.00013 | 0.900376 | 0.099473 | 0 | 0 | 0 | 0 | 0 |
| 503 | 0.00001 | 0.00004 | 0.899021 | 0.100929 | 0 | 0 | 0 | 0 | 0 |
| 504 | 0.00004 | 0.00007 | 0.901827 | 0.098063 | 0 | 0 | 0 | 0 | 0 |
| 505 | 0.00001 | 0.00009 | 0.899345 | 0.100515 | 4E-05 | 0 | 0 | 0 | 0 |
| 506 | 0.00001 | 0.00004 | 0.901154 | 0.098795 | 0 | 0 | 0 | 0 | 0 |
| 507 | 0.00001 | 0.000121 | 0.90026 | 0.09961 | 0 | 0 | 0 | 0 | 0 |
| 508 | 0.00001 | 0.000109 | 0.900261 | 0.09962 | 0 | 0 | 0 | 0 | 0 |
| 509 | 0 | 0.00004 | 0.898786 | 0.101174 | 0 | 0 | 0 | 0 | 0 |
| 510 | 0 | 0.00007 | 0.901663 | 0.098268 | 0 | 0 | 0 | 0 | 0 |
| 511 | 0 | 0.00004 | 0.901036 | 0.098924 | 0 | 0 | 0 | 0 | 0 |
| 512 | 0.00002 | 0.00006 | 0.898799 | 0.101121 | 0 | 0 | 0 | 0 | 0 |
| 513 | 0 | 0.00007 | 0.901583 | 0.098347 | 0 | 0 | 0 | 0 | 0 |
| 514 | 0 | 0.00004 | 0.899596 | 0.100364 | 0 | 0 | 0 | 0 | 0 |
| 515 | 0.00002 | 0.00005 | 0.903005 | 0.096924 | 0 | 0 | 0 | 0 | 0 |
| 516 | 0.00001 | 0.00011 | 0.896107 | 0.103773 | 0 | 0 | 0 | 0 | 0 |
| 517 | 0.00001 | 0.00008 | 0.898458 | 0.101452 | 0 | 0 | 0 | 0 | 0 |
| 518 | 0.00002 | 0.00011 | 0.901954 | 0.097916 | 0 | 0 | 0 | 0 | 0 |
| 519 | 0.00001 | 0.00007 | 0.898729 | 0.101191 | 0 | 0 | 0 | 0 | 0 |
| 520 | 0 | 0.00001 | 0.902062 | 0.097928 | 0 | 0 | 0 | 0 | 0 |
| 521 | 0.00002 | 0.00007 | 0.898149 | 0.101761 | 0 | 0 | 0 | 0 | 0 |
| 522 | 0.00001 | 0.00012 | 0.902534 | 0.097336 | 0 | 0 | 0 | 0 | 0 |
| 523 | 0.00002 | 0.00004 | 0.898499 | 0.101441 | 0 | 0 | 0 | 0 | 0 |
| 524 | 0.00001 | 0.00004 | 0.897717 | 0.102232 | 0 | 0 | 0 | 0 | 0 |
| 525 | 0.00001 | 0.00009 | 0.896753 | 0.103147 | 0 | 0 | 0 | 0 | 0 |
| 526 | 0 | 0.00007 | 0.898814 | 0.101116 | 0 | 0 | 0 | 0 | 0 |
| 527 | 0 | 0.00004 | 0.901641 | 0.098318 | 0 | 0 | 0 | 0 | 0 |
| 528 | 0.00003 | 0.00007 | 0.898477 | 0.101424 | 0 | 0 | 0 | 0 | 0 |
| 529 | 0 | 0.00006 | 0.898655 | 0.101285 | 0 | 0 | 0 | 0 | 0 |
| 530 | 0.00001 | 0.00012 | 0.896871 | 0.102999 | 0 | 0 | 0 | 0 | 0 |
| 531 | 0.00002 | 0.00009 | 0.900491 | 0.099398 | 0 | 0 | 0 | 0 | 0 |
| 532 | 0.00001 | 0.00004 | 0.906503 | 0.093447 | 0 | 0 | 0 | 0 | 0 |
| 533 | 0.00001 | 0.000099 | 0.899227 | 0.100663 | 0 | 0 | 0 | 0 | 0 |
| 534 | 0 | 0.00007 | 0.897357 | 0.102573 | 0 | 0 | 0 | 0 | 0 |
| 535 | 0 | 0.000069 | 0.905066 | 0.094864 | 0 | 0 | 0 | 0 | 0 |
| 536 | 0.00002 | 0.00003 | 0.902871 | 0.097079 | 0 | 0 | 0 | 0 | 0 |
| 537 | 0.00001 | 0.00003 | 0.899806 | 0.100154 | 0 | 0 | 0 | 0 | 0 |
| 538 | 0.00001 | 0.00012 | 0.896309 | 0.103561 | 0 | 0 | 0 | 0 | 0 |
| 539 | 0.00003 | 0.00014 | 0.897388 | 0.102443 | 0 | 0 | 0 | 0 | 0 |
| 540 | 0.00002 | 0.00014 | 0.89818 | 0.101661 | 0 | 0 | 0 | 0 | 0 |
| 541 | 0.00001 | 0.00004 | 0.902617 | 0.097333 | 0 | 0 | 0 | 0 | 0 |
| 542 | 0.00001 | 0.000091 | 0.900607 | 0.099292 | 0 | 0 | 0 | 0 | 0 |
| 543 | 0.00001 | 0.000071 | 0.904302 | 0.095618 | 0 | 0 | 0 | 0 | 0 |
| 544 | 0 | 0.00003 | 0.904264 | 0.095706 | 0 | 0 | 0 | 0 | 0 |
| 545 | 0.00002 | 0.00008 | 0.894484 | 0.105416 | 0 | 0 | 0 | 0 | 0 |
| 546 | 0 | 0.00009 | 0.904715 | 0.095195 | 0 | 0 | 0 | 0 | 0 |
| 547 | 0.00001 | 0.00011 | 0.89817 | 0.10171 | 0 | 0 | 0 | 0 | 0 |
| 548 | 0.00001 | 0.00007 | 0.903383 | 0.096537 | 0 | 0 | 0 | 0 | 0 |
| 549 | 0.00002 | 0.00006 | 0.896804 | 0.103117 | 0 | 0 | 0 | 0 | 0 |
| 550 | 0.00001 | 0.00011 | 0.903012 | 0.096868 | 0 | 0 | 0 | 0 | 0 |
| 551 | 0.00001 | 0.00007 | 0.901395 | 0.098525 | 0 | 0 | 0 | 0 | 0 |
| 552 | 0.00001 | 0.00005 | 0.898728 | 0.101211 | 0 | 0 | 0 | 0 | 0 |
| 553 | 0.00001 | 0.00013 | 0.899852 | 0.100008 | 0 | 0 | 0 | 0 | 0 |
| 554 | 0.00002 | 0.0001 | 0.905928 | 0.093951 | 0 | 0 | 0 | 0 | 0 |
| 555 | 0.00002 | 0.00004 | 0.899318 | 0.100623 | 0 | 0 | 0 | 0 | 0 |
| 556 | 0.00001 | 0.00002 | 0.904798 | 0.095172 | 0 | 0 | 0 | 0 | 0 |
| 557 | 0 | 0.00008 | 0.899501 | 0.100419 | 0 | 0 | 0 | 0 | 0 |
| 558 | 0 | 0.00006 | 0.899521 | 0.10042 | 0 | 0 | 0 | 0 | 0 |
| 559 | 0.00001 | 0.00003 | 0.900401 | 0.099559 | 0 | 0 | 0 | 0 | 0 |
| 560 | 0 | 0.00006 | 0.902695 | 0.097244 | 0 | 0 | 0 | 0 | 0 |
| 561 | 0 | 0.000091 | 0.899381 | 0.100528 | 0 | 0 | 0 | 0 | 0 |
| 562 | 0.00001 | 0.00004 | 0.896358 | 0.103592 | 0 | 0 | 0 | 0 | 0 |
| 563 | 0.00001 | 0.000111 | 0.896042 | 0.103837 | 0 | 0 | 0 | 0 | 0 |
| 564 | 0.00001 | 0.00008 | 0.902788 | 0.097122 | 0 | 0 | 0 | 0 | 0 |
| 565 | 0 | 0.00012 | 0.896157 | 0.103722 | 0 | 0 | 0 | 0 | 0 |
| 566 | 0 | 0.0001 | 0.900523 | 0.099377 | 0 | 0 | 0 | 0 | 0 |
| 567 | 0.00001 | 0.00003 | 0.898881 | 0.101079 | 0 | 0 | 0 | 0 | 0 |
| 568 | 0 | 0.00004 | 0.900027 | 0.099933 | 0 | 0 | 0 | 0 | 0 |
| 569 | 0.00001 | 0.00008 | 0.903493 | 0.096417 | 0 | 0 | 0 | 0 | 0 |
| 570 | 0.00001 | 0.000091 | 0.895966 | 0.103933 | 0 | 0 | 0 | 0 | 0 |
| 571 | 0 | 0.0001 | 0.900886 | 0.099014 | 0 | 0 | 0 | 0 | 0 |
| 572 | 0.00002 | 0.000089 | 0.901081 | 0.098809 | 0 | 0 | 0 | 0 | 0 |
| 573 | 0 | 0.000111 | 0.900783 | 0.099106 | 0 | 0 | 0 | 0 | 0 |
| 574 | 0 | 0.00006 | 0.90142 | 0.09852 | 0 | 0 | 0 | 0 | 0 |
| 575 | 0 | 0.00005 | 0.901292 | 0.098658 | 0 | 0 | 0 | 0 | 0 |
| 576 | 0 | 0.00007 | 0.906249 | 0.09368 | 0 | 0 | 0 | 0 | 0 |
| 577 | 0.00002 | 0.00003 | 0.897497 | 0.102453 | 0 | 0 | 0 | 0 | 0 |
| 578 | 0 | 0.00005 | 0.901374 | 0.098576 | 0 | 0 | 0 | 0 | 0 |
| 579 | 0.00001 | 0.000069 | 0.898195 | 0.101726 | 0 | 0 | 0 | 0 | 0 |
| 580 | 0.00002 | 0.00005 | 0.903433 | 0.096496 | 0 | 0 | 0 | 0 | 0 |
| 581 | 0.00001 | 0.00011 | 0.897773 | 0.102107 | 0 | 0 | 0 | 0 | 0 |
| 582 | 0.00001 | 0.00008 | 0.900527 | 0.099383 | 0 | 0 | 0 | 0 | 0 |
| 583 | 0.00002 | 0.00011 | 0.897613 | 0.102256 | 0 | 0 | 0 | 0 | 0 |
| 584 | 0.00002 | 0.00005 | 0.897299 | 0.102631 | 0 | 0 | 0 | 0 | 0 |
| 585 | 0.00003 | 0.00009 | 0.897562 | 0.102318 | 0 | 0 | 0 | 0 | 0 |
| 586 | 0.00003 | 0.000099 | 0.899966 | 0.099905 | 0 | 0 | 0 | 0 | 0 |
| 587 | 0.00002 | 0.00005 | 0.900546 | 0.099384 | 0 | 0 | 0 | 0 | 0 |
| 588 | 0 | 0.00005 | 0.903164 | 0.096785 | 0 | 0 | 0 | 0 | 0 |
| 589 | 0 | 0.00005 | 0.901797 | 0.098153 | 0 | 0 | 0 | 0 | 0 |
| 590 | 0.00001 | 0.00007 | 0.898319 | 0.101601 | 0 | 0 | 0 | 0 | 0 |
| 591 | 0.00004 | 0.00005 | 0.899607 | 0.100303 | 0 | 0 | 0 | 0 | 0 |
| 592 | 0.00003 | 0.00013 | 0.899443 | 0.100397 | 0 | 0 | 0 | 0 | 0 |
| 593 | 0.00003 | 0.000091 | 0.904866 | 0.095013 | 0 | 0 | 0 | 0 | 0 |
| 594 | 0 | 0.00005 | 0.900398 | 0.099552 | 0 | 0 | 0 | 0 | 0 |
| 595 | 0 | 0.00013 | 0.900286 | 0.099584 | 0 | 0 | 0 | 0 | 0 |
| 596 | 0.00001 | 0.00008 | 0.902461 | 0.097449 | 0 | 0 | 0 | 0 | 0 |
| 597 | 0.00002 | 0.00003 | 0.898856 | 0.101095 | 0 | 0 | 0 | 0 | 0 |
| 598 | 0.00002 | 0.00013 | 0.899761 | 0.10009 | 0 | 0 | 0 | 0 | 0 |
| 599 | 0.00003 | 0.00004 | 0.902273 | 0.097657 | 0 | 0 | 0 | 0 | 0 |
| 600 | 0.00001 | 0.00005 | 0.898239 | 0.101701 | 0 | 0 | 0 | 0 | 0 |
| 601 | 0.00002 | 0.00003 | 0.90207 | 0.09788 | 0 | 0 | 0 | 0 | 0 |
| 602 | 0 | 0.00006 | 0.902879 | 0.097061 | 0 | 0 | 0 | 0 | 0 |
| 603 | 0 | 0.00009 | 0.901314 | 0.098596 | 0 | 0 | 0 | 0 | 0 |
| 604 | 0.00001 | 0.00005 | 0.901038 | 0.098902 | 0 | 0 | 0 | 0 | 0 |
| 605 | 0.00002 | 0.00009 | 0.901217 | 0.098673 | 0 | 0 | 0 | 0 | 0 |
| 606 | 0.00002 | 0.000151 | 0.903505 | 0.096324 | 0 | 0 | 0 | 0 | 0 |
| 607 | 0.00002 | 0.0001 | 0.904825 | 0.095055 | 0 | 0 | 0 | 0 | 0 |
| 608 | 0 | 0.000059 | 0.898353 | 0.101588 | 0 | 0 | 0 | 0 | 0 |
| 609 | 0.00002 | 0.00009 | 0.901837 | 0.098053 | 0 | 0 | 0 | 0 | 0 |
| 610 | 0.00003 | 0.00004 | 0.899851 | 0.100078 | 0 | 0 | 0 | 0 | 0 |
| 611 | 0.00002 | 0.00005 | 0.901819 | 0.098111 | 0 | 0 | 0 | 0 | 0 |
| 612 | 0.00001 | 0.00003 | 0.89796 | 0.102 | 0 | 0 | 0 | 0 | 0 |
| 613 | 0.00003 | 0.000131 | 0.896274 | 0.103565 | 0 | 0 | 0 | 0 | 0 |
| 614 | 0.00002 | 0.00003 | 0.905235 | 0.094715 | 0 | 0 | 0 | 0 | 0 |
| 615 | 0.00001 | 0.00005 | 0.90053 | 0.09941 | 0 | 0 | 0 | 0 | 0 |
| 616 | 0 | 0.000141 | 0.901137 | 0.098722 | 0 | 0 | 0 | 0 | 0 |
| 617 | 0 | 0.00006 | 0.900864 | 0.099076 | 0 | 0 | 0 | 0 | 0 |
| 618 | 0.00001 | 0.00004 | 0.895876 | 0.104074 | 0 | 0 | 0 | 0 | 0 |
| 619 | 0 | 0.000051 | 0.903669 | 0.09628 | 0 | 0 | 0 | 0 | 0 |
| 620 | 0.00001 | 0.00009 | 0.90038 | 0.09952 | 0 | 0 | 0 | 0 | 0 |
| 621 | 0.00004 | 0.00001 | 0.90212 | 0.09783 | 0 | 0 | 0 | 0 | 0 |
| 622 | 0.00003 | 0.0001 | 0.895674 | 0.104196 | 0 | 0 | 0 | 0 | 0 |
| 623 | 0.00001 | 0.00001 | 0.901421 | 0.098559 | 0 | 0 | 0 | 0 | 0 |
| 624 | 0.00003 | 0.00012 | 0.900243 | 0.099606 | 0 | 0 | 0 | 0 | 0 |
| 625 | 0.00002 | 0.000161 | 0.901073 | 0.098695 | 5E-05 | 0 | 0 | 0 | 0 |
| 626 | 0.00002 | 0.00007 | 0.898837 | 0.101073 | 0 | 0 | 0 | 0 | 0 |
| 627 | 0.00001 | 0.00003 | 0.90066 | 0.0993 | 0 | 0 | 0 | 0 | 0 |
| 628 | 0 | 0.000171 | 0.900732 | 0.099098 | 0 | 0 | 0 | 0 | 0 |
| 629 | 0 | 0.00006 | 0.900744 | 0.099196 | 0 | 0 | 0 | 0 | 0 |
| 630 | 0.00001 | 0.00004 | 0.898244 | 0.101705 | 0 | 0 | 0 | 0 | 0 |
| 631 | 0.00002 | 0.00006 | 0.900193 | 0.099727 | 0 | 0 | 0 | 0 | 0 |
| 632 | 0 | 0.00001 | 0.90013 | 0.09986 | 0 | 0 | 0 | 0 | 0 |
| 633 | 0.00005 | 0.00008 | 0.901299 | 0.098571 | 0 | 0 | 0 | 0 | 0 |
| 634 | 0.00002 | 0.00004 | 0.898389 | 0.10155 | 0 | 0 | 0 | 0 | 0 |
| 635 | 0 | 0.00011 | 0.904025 | 0.095865 | 0 | 0 | 0 | 0 | 0 |
| 636 | 0 | 0.00007 | 0.898998 | 0.100933 | 0 | 0 | 0 | 0 | 0 |
| 637 | 0.00001 | 0.00002 | 0.900537 | 0.099433 | 0 | 0 | 0 | 0 | 0 |
| 638 | 0.00003 | 0.000099 | 0.901309 | 0.098562 | 0 | 0 | 0 | 0 | 0 |
| 639 | 0.00001 | 0.00003 | 0.896119 | 0.10384 | 0 | 0 | 0 | 0 | 0 |
| 640 | 0 | 0.00009 | 0.895348 | 0.104562 | 0 | 0 | 0 | 0 | 0 |
| 641 | 0.00001 | 0.000091 | 0.901503 | 0.098396 | 0 | 0 | 0 | 0 | 0 |
| 642 | 0.00002 | 0.00006 | 0.902091 | 0.097829 | 0 | 0 | 0 | 0 | 0 |
| 643 | 0 | 0.00003 | 0.899868 | 0.100101 | 0 | 0 | 0 | 0 | 0 |
| 644 | 0.00001 | 0.000109 | 0.903316 | 0.096565 | 0 | 0 | 0 | 0 | 0 |
| 645 | 0.00003 | 0.00004 | 0.902114 | 0.097815 | 0 | 0 | 0 | 0 | 0 |
| 646 | 0.00001 | 0.00006 | 0.899531 | 0.100399 | 0 | 0 | 0 | 0 | 0 |
| 647 | 0.00002 | 0.00007 | 0.902169 | 0.097741 | 0 | 0 | 0 | 0 | 0 |
| 648 | 0.00002 | 0.00011 | 0.900967 | 0.098903 | 0 | 0 | 0 | 0 | 0 |
| 649 | 0.00003 | 0.00009 | 0.901608 | 0.098272 | 0 | 0 | 0 | 0 | 0 |
| 650 | 0 | 0.00003 | 0.89859 | 0.10138 | 0 | 0 | 0 | 0 | 0 |
| 651 | 0.00001 | 0.00003 | 0.900938 | 0.099023 | 0 | 0 | 0 | 0 | 0 |
| 652 | 0.00003 | 0.00009 | 0.901288 | 0.098593 | 0 | 0 | 0 | 0 | 0 |
| 653 | 0.00001 | 0.000111 | 0.902803 | 0.097076 | 0 | 0 | 0 | 0 | 0 |
| 654 | 0.00001 | 0.00003 | 0.901162 | 0.098798 | 0 | 0 | 0 | 0 | 0 |
| 655 | 0.00002 | 0.00007 | 0.897138 | 0.102771 | 0 | 0 | 0 | 0 | 0 |
| 656 | 0 | 0.00003 | 0.907078 | 0.092892 | 0 | 0 | 0 | 0 | 0 |
| 657 | 0 | 0.00006 | 0.900219 | 0.099721 | 0 | 0 | 0 | 0 | 0 |
| 658 | 0 | 0.00009 | 0.899661 | 0.100249 | 0 | 0 | 0 | 0 | 0 |
| 659 | 0.00003 | 0.000071 | 0.897299 | 0.1026 | 0 | 0 | 0 | 0 | 0 |
| 660 | 0.00003 | 0.00007 | 0.905747 | 0.094154 | 0 | 0 | 0 | 0 | 0 |
| 661 | 0 | 0.0001 | 0.901383 | 0.098517 | 0 | 0 | 0 | 0 | 0 |
| 662 | 0.00001 | 0.00005 | 0.898151 | 0.101789 | 0 | 0 | 0 | 0 | 0 |
| 663 | 0.00006 | 0.00003 | 0.902066 | 0.097844 | 0 | 0 | 0 | 0 | 0 |
| 664 | 0 | 0.00007 | 0.906322 | 0.093609 | 0 | 0 | 0 | 0 | 0 |
| 665 | 0.00002 | 0.00006 | 0.896691 | 0.103228 | 0 | 0 | 0 | 0 | 0 |
| 666 | 0 | 0.00008 | 0.898599 | 0.101321 | 0 | 0 | 0 | 0 | 0 |
| 667 | 0.00003 | 0.00007 | 0.897062 | 0.102838 | 0 | 0 | 0 | 0 | 0 |
| 668 | 0 | 0.000079 | 0.897086 | 0.102835 | 0 | 0 | 0 | 0 | 0 |
| 669 | 0.00001 | 0.00006 | 0.897685 | 0.102245 | 0 | 0 | 0 | 0 | 0 |
| 670 | 0 | 0.00004 | 0.898734 | 0.101226 | 0 | 0 | 0 | 0 | 0 |
| 671 | 0.00001 | 0.00013 | 0.900066 | 0.099794 | 0 | 0 | 0 | 0 | 0 |
| 672 | 0.00002 | 0.00004 | 0.900649 | 0.099291 | 0 | 0 | 0 | 0 | 0 |
| 673 | 0.00003 | 0.00004 | 0.899196 | 0.100734 | 0 | 0 | 0 | 0 | 0 |
| 674 | 0.00002 | 0.00003 | 0.900492 | 0.099457 | 0 | 0 | 0 | 0 | 0 |
| 675 | 0.00002 | 0.00008 | 0.902876 | 0.097024 | 0 | 0 | 0 | 0 | 0 |
| 676 | 0.00002 | 0.000101 | 0.901463 | 0.098416 | 0 | 0 | 0 | 0 | 0 |
| 677 | 0.00002 | 0.00004 | 0.899732 | 0.100208 | 0 | 0 | 0 | 0 | 0 |
| 678 | 0.00001 | 0.0001 | 0.897769 | 0.102121 | 0 | 0 | 0 | 0 | 0 |
| 679 | 0 | 0.00007 | 0.903127 | 0.096803 | 0 | 0 | 0 | 0 | 0 |
| 680 | 0.00001 | 0.000111 | 0.899265 | 0.100614 | 0 | 0 | 0 | 0 | 0 |
| 681 | 0.00001 | 0.000079 | 0.897192 | 0.102718 | 0 | 0 | 0 | 0 | 0 |
| 682 | 0 | 0.00007 | 0.898439 | 0.101491 | 0 | 0 | 0 | 0 | 0 |
| 683 | 0.00002 | 0.00011 | 0.90166 | 0.09821 | 0 | 0 | 0 | 0 | 0 |
| 684 | 0.00004 | 0.00005 | 0.900701 | 0.099209 | 0 | 0 | 0 | 0 | 0 |
| 685 | 0.00001 | 0.00004 | 0.903415 | 0.096535 | 0 | 0 | 0 | 0 | 0 |
| 686 | 0 | 0.00014 | 0.895132 | 0.104727 | 0 | 0 | 0 | 0 | 0 |
| 687 | 0 | 0.00004 | 0.900075 | 0.099885 | 0 | 0 | 0 | 0 | 0 |
| 688 | 0.00002 | 0.00004 | 0.900591 | 0.099349 | 0 | 0 | 0 | 0 | 0 |
| 689 | 0.00001 | 0.000081 | 0.897922 | 0.101987 | 0 | 0 | 0 | 0 | 0 |
| 690 | 0.00004 | 0.000089 | 0.900746 | 0.099125 | 0 | 0 | 0 | 0 | 0 |
| 691 | 0 | 0.00006 | 0.900935 | 0.099005 | 0 | 0 | 0 | 0 | 0 |
| 692 | 0 | 0.00008 | 0.900373 | 0.099547 | 0 | 0 | 0 | 0 | 0 |
| 693 | 0 | 0.0001 | 0.898078 | 0.101822 | 0 | 0 | 0 | 0 | 0 |
| 694 | 0.00003 | 0.0001 | 0.903232 | 0.096637 | 0 | 0 | 0 | 0 | 0 |
| 695 | 0.00001 | 0.00005 | 0.901668 | 0.098272 | 0 | 0 | 0 | 0 | 0 |
| 696 | 0.00002 | 0.00009 | 0.90454 | 0.09535 | 0 | 0 | 0 | 0 | 0 |
| 697 | 0 | 0.00004 | 0.903913 | 0.096047 | 0 | 0 | 0 | 0 | 0 |
| 698 | 0.00001 | 0.00004 | 0.898541 | 0.101409 | 0 | 0 | 0 | 0 | 0 |
| 699 | 0 | 0.00006 | 0.902037 | 0.097903 | 0 | 0 | 0 | 0 | 0 |
| 700 | 0.00001 | 0.00007 | 0.898601 | 0.101319 | 0 | 0 | 0 | 0 | 0 |
| 701 | 0.00001 | 0.00008 | 0.908653 | 0.091257 | 0 | 0 | 0 | 0 | 0 |
| 702 | 0.00002 | 0.00013 | 0.904977 | 0.094873 | 0 | 0 | 0 | 0 | 0 |
| 703 | 0.00001 | 0.00005 | 0.899499 | 0.10044 | 0 | 0 | 0 | 0 | 0 |
| 704 | 0.00002 | 0.00012 | 0.899361 | 0.1005 | 0 | 0 | 0 | 0 | 0 |
| 705 | 0.00003 | 0.00006 | 0.899559 | 0.100351 | 0 | 0 | 0 | 0 | 0 |
| 706 | 0 | 0.00007 | 0.89943 | 0.100499 | 0 | 0 | 0 | 0 | 0 |
| 707 | 0.00001 | 0.00008 | 0.896899 | 0.103011 | 0 | 0 | 0 | 0 | 0 |
| 708 | 0.00001 | 0.00013 | 0.898428 | 0.101432 | 0 | 0 | 0 | 0 | 0 |
| 709 | 0.00001 | 0.00009 | 0.903377 | 0.096522 | 0 | 0 | 0 | 0 | 0 |
| 710 | 0 | 0.00016 | 0.90157 | 0.09827 | 0 | 0 | 0 | 0 | 0 |
| 711 | 0.00003 | 0.00004 | 0.903635 | 0.096295 | 0 | 0 | 0 | 0 | 0 |
| 712 | 0.00001 | 0.00005 | 0.906501 | 0.093439 | 0 | 0 | 0 | 0 | 0 |
| 713 | 0.00001 | 0.00005 | 0.897622 | 0.102318 | 0 | 0 | 0 | 0 | 0 |
| 714 | 0.00001 | 0.00006 | 0.900009 | 0.099921 | 0 | 0 | 0 | 0 | 0 |
| 715 | 0.00001 | 0.00007 | 0.896059 | 0.10386 | 0 | 0 | 0 | 0 | 0 |
| 716 | 0.00001 | 0.000129 | 0.895205 | 0.104656 | 0 | 0 | 0 | 0 | 0 |
| 717 | 0.00001 | 0.000089 | 0.901174 | 0.098727 | 0 | 0 | 0 | 0 | 0 |
| 718 | 0.00002 | 0.000181 | 0.901013 | 0.098786 | 0 | 0 | 0 | 0 | 0 |
| 719 | 0.00001 | 0.00006 | 0.903772 | 0.096158 | 0 | 0 | 0 | 0 | 0 |
| 720 | 0 | 0.00005 | 0.899271 | 0.100679 | 0 | 0 | 0 | 0 | 0 |
| 721 | 0.00003 | 0.00003 | 0.898093 | 0.101847 | 0 | 0 | 0 | 0 | 0 |
| 722 | 0.00001 | 0.000099 | 0.902931 | 0.096959 | 0 | 0 | 0 | 0 | 0 |
| 723 | 0.00002 | 0.00009 | 0.897831 | 0.102058 | 0 | 0 | 0 | 0 | 0 |
| 724 | 0 | 0.0001 | 0.903224 | 0.096676 | 0 | 0 | 0 | 0 | 0 |
| 725 | 0.00001 | 0.00001 | 0.898793 | 0.101187 | 0 | 0 | 0 | 0 | 0 |
| 726 | 0.00001 | 0.000131 | 0.901408 | 0.098452 | 0 | 0 | 0 | 0 | 0 |
| 727 | 0 | 0.00008 | 0.897946 | 0.101974 | 0 | 0 | 0 | 0 | 0 |
| 728 | 0.00001 | 0.00016 | 0.900205 | 0.099624 | 0 | 0 | 0 | 0 | 0 |
| 729 | 0.00001 | 0.00007 | 0.898869 | 0.101051 | 0 | 0 | 0 | 0 | 0 |
| 730 | 0.00002 | 0.00005 | 0.898161 | 0.10177 | 0 | 0 | 0 | 0 | 0 |
| 731 | 0 | 0.00008 | 0.902342 | 0.097578 | 0 | 0 | 0 | 0 | 0 |
| 732 | 0.00002 | 0.00005 | 0.903215 | 0.096715 | 0 | 0 | 0 | 0 | 0 |
| 733 | 0.00001 | 0.00007 | 0.899055 | 0.100865 | 0 | 0 | 0 | 0 | 0 |
| 734 | 0.00001 | 0.000071 | 0.900842 | 0.099077 | 0 | 0 | 0 | 0 | 0 |
| 735 | 0.00001 | 0.00012 | 0.902962 | 0.096908 | 0 | 0 | 0 | 0 | 0 |
| 736 | 0.00001 | 0.00003 | 0.903852 | 0.096108 | 0 | 0 | 0 | 0 | 0 |
| 737 | 0.00001 | 0.00011 | 0.900172 | 0.099708 | 0 | 0 | 0 | 0 | 0 |
| 738 | 0.00003 | 0.00007 | 0.897731 | 0.102169 | 0 | 0 | 0 | 0 | 0 |
| 739 | 0.000011 | 0.000066 | 0.890926 | 0.108997 | 0 | 0 | 0 | 0 | 0 |
| 740 | 0 | 0.00006 | 0.903739 | 0.096201 | 0 | 0 | 0 | 0 | 0 |
| 741 | 0.00002 | 0.00004 | 0.900815 | 0.099126 | 0 | 0 | 0 | 0 | 0 |
| 742 | 0.00004 | 0.00005 | 0.900426 | 0.099484 | 0 | 0 | 0 | 0 | 0 |
| 743 | 0.00002 | 0.00006 | 0.902312 | 0.097608 | 0 | 0 | 0 | 0 | 0 |
| 744 | 0.00002 | 0.000169 | 0.903421 | 0.096282 | 1E-04 | 0 | 0 | 0 | 0 |
| 745 | 0.00001 | 0.000089 | 0.903325 | 0.096576 | 0 | 0 | 0 | 0 | 0 |
| 746 | 0.00001 | 0.00007 | 0.895108 | 0.104812 | 0 | 0 | 0 | 0 | 0 |
| 747 | 0.00001 | 0.00004 | 0.899947 | 0.100003 | 0 | 0 | 0 | 0 | 0 |
| 748 | 0.00001 | 0.00008 | 0.902222 | 0.097688 | 0 | 0 | 0 | 0 | 0 |
| 749 | 0.00001 | 0.00013 | 0.9064 | 0.09346 | 0 | 0 | 0 | 0 | 0 |
| 750 | 0 | 0.00005 | 0.901602 | 0.098348 | 0 | 0 | 0 | 0 | 0 |
| 751 | 0.00001 | 0.00008 | 0.902543 | 0.097367 | 0 | 0 | 0 | 0 | 0 |
| 752 | 0.00002 | 0.00004 | 0.902847 | 0.097093 | 0 | 0 | 0 | 0 | 0 |
| 753 | 0.00001 | 0.00002 | 0.901963 | 0.098007 | 0 | 0 | 0 | 0 | 0 |
| 754 | 0.00002 | 0.000111 | 0.898929 | 0.10094 | 0 | 0 | 0 | 0 | 0 |
| 755 | 0.00002 | 0.00008 | 0.898867 | 0.101033 | 0 | 0 | 0 | 0 | 0 |
| 756 | 0.00004 | 0.00007 | 0.899391 | 0.100499 | 0 | 0 | 0 | 0 | 0 |
| 757 | 0.00001 | 0.00006 | 0.902422 | 0.097508 | 0 | 0 | 0 | 0 | 0 |
| 758 | 0.00003 | 0.00009 | 0.897896 | 0.101984 | 0 | 0 | 0 | 0 | 0 |
| 759 | 0 | 0.000091 | 0.898913 | 0.100997 | 0 | 0 | 0 | 0 | 0 |
| 760 | 0.00001 | 0.00011 | 0.902517 | 0.097364 | 0 | 0 | 0 | 0 | 0 |
| 761 | 0.00002 | 0.00007 | 0.90102 | 0.09889 | 0 | 0 | 0 | 0 | 0 |
| 762 | 0.00001 | 0.00009 | 0.904278 | 0.095623 | 0 | 0 | 0 | 0 | 0 |
| 763 | 0.00001 | 0.000079 | 0.899811 | 0.100099 | 0 | 0 | 0 | 0 | 0 |
| 764 | 0 | 0.00003 | 0.907596 | 0.092374 | 0 | 0 | 0 | 0 | 0 |
| 765 | 0.00002 | 0.00007 | 0.899264 | 0.100645 | 0 | 0 | 0 | 0 | 0 |
| 766 | 0.00002 | 0.00008 | 0.899186 | 0.100714 | 0 | 0 | 0 | 0 | 0 |
| 767 | 0 | 0.0001 | 0.903126 | 0.096773 | 0 | 0 | 0 | 0 | 0 |
| 768 | 0.00005 | 0.00008 | 0.898617 | 0.101253 | 0 | 0 | 0 | 0 | 0 |
| 769 | 0.00001 | 0.00003 | 0.900621 | 0.099339 | 0 | 0 | 0 | 0 | 0 |
| 770 | 0.00001 | 0.0001 | 0.901192 | 0.098698 | 0 | 0 | 0 | 0 | 0 |
| 771 | 0.00002 | 0.00004 | 0.89916 | 0.100701 | 8E-05 | 0 | 0 | 0 | 0 |
| 772 | 0.00001 | 0.00008 | 0.898009 | 0.101902 | 0 | 0 | 0 | 0 | 0 |
| 773 | 0.00001 | 0.00004 | 0.895039 | 0.10491 | 0 | 0 | 0 | 0 | 0 |
| 774 | 0.00001 | 0.00007 | 0.90224 | 0.097681 | 0 | 0 | 0 | 0 | 0 |
| 775 | 0 | 0.00007 | 0.902233 | 0.097697 | 0 | 0 | 0 | 0 | 0 |
| 776 | 0 | 0.00005 | 0.898182 | 0.101768 | 0 | 0 | 0 | 0 | 0 |
| 777 | 0.00003 | 0.00009 | 0.902764 | 0.097116 | 0 | 0 | 0 | 0 | 0 |
| 778 | 0 | 0.00007 | 0.904112 | 0.095818 | 0 | 0 | 0 | 0 | 0 |
| 779 | 0.00001 | 0.00006 | 0.906733 | 0.093198 | 0 | 0 | 0 | 0 | 0 |
| 780 | 0 | 0.00009 | 0.900735 | 0.099175 | 0 | 0 | 0 | 0 | 0 |
| 781 | 0 | 0.00009 | 0.900244 | 0.099666 | 0 | 0 | 0 | 0 | 0 |
| 782 | 0 | 0.00008 | 0.905107 | 0.094813 | 0 | 0 | 0 | 0 | 0 |
| 783 | 0.00001 | 0.0001 | 0.901189 | 0.098701 | 0 | 0 | 0 | 0 | 0 |
| 784 | 0.00001 | 0.00007 | 0.902019 | 0.097901 | 0 | 0 | 0 | 0 | 0 |
| 785 | 0.00005 | 0.00003 | 0.902409 | 0.09751 | 0 | 0 | 0 | 0 | 0 |
| 786 | 0 | 0.00007 | 0.899662 | 0.100268 | 0 | 0 | 0 | 0 | 0 |
| 787 | 0 | 0.000069 | 0.902076 | 0.097855 | 0 | 0 | 0 | 0 | 0 |
| 788 | 0 | 0.000079 | 0.90529 | 0.094631 | 0 | 0 | 0 | 0 | 0 |
| 789 | 0.00003 | 0.00003 | 0.896408 | 0.103532 | 0 | 0 | 0 | 0 | 0 |
| 790 | 0.00003 | 0.00005 | 0.901757 | 0.098162 | 0 | 0 | 0 | 0 | 0 |
| 791 | 0 | 0.00002 | 0.902704 | 0.097276 | 0 | 0 | 0 | 0 | 0 |
| 792 | 0.00001 | 0.00007 | 0.904311 | 0.095609 | 0 | 0 | 0 | 0 | 0 |
| 793 | 0.00001 | 0.00006 | 0.897696 | 0.102234 | 0 | 0 | 0 | 0 | 0 |
| 794 | 0.00001 | 0.00008 | 0.902056 | 0.097854 | 0 | 0 | 0 | 0 | 0 |
| 795 | 0.00001 | 0.00004 | 0.898962 | 0.100988 | 0 | 0 | 0 | 0 | 0 |
| 796 | 0.00002 | 0.00007 | 0.902898 | 0.097012 | 0 | 0 | 0 | 0 | 0 |
| 797 | 0.00002 | 0.00003 | 0.902011 | 0.097939 | 0 | 0 | 0 | 0 | 0 |
| 798 | 0.00001 | 0.00008 | 0.898719 | 0.10113 | 6E-05 | 0 | 0 | 0 | 0 |
| 799 | 0.00001 | 0.00014 | 0.901874 | 0.097976 | 0 | 0 | 0 | 0 | 0 |
| 800 | 0 | 0.00003 | 0.897228 | 0.102742 | 0 | 0 | 0 | 0 | 0 |
| 801 | 0.00002 | 0.00004 | 0.906347 | 0.093593 | 0 | 0 | 0 | 0 | 0 |
| 802 | 0.00001 | 0.00008 | 0.897449 | 0.10246 | 0 | 0 | 0 | 0 | 0 |
| 803 | 0.00001 | 0.00008 | 0.9037 | 0.09621 | 0 | 0 | 0 | 0 | 0 |
| 804 | 0.00002 | 0.00009 | 0.900261 | 0.099629 | 0 | 0 | 0 | 0 | 0 |
| 805 | 0 | 0.000059 | 0.904709 | 0.095232 | 0 | 0 | 0 | 0 | 0 |
| 806 | 0 | 0.00004 | 0.898879 | 0.101081 | 0 | 0 | 0 | 0 | 0 |
| 807 | 0.00001 | 0.00006 | 0.901588 | 0.098342 | 0 | 0 | 0 | 0 | 0 |
| 808 | 0 | 0.00003 | 0.897522 | 0.102448 | 0 | 0 | 0 | 0 | 0 |
| 809 | 0.00001 | 0.00004 | 0.90229 | 0.097661 | 0 | 0 | 0 | 0 | 0 |
| 810 | 0 | 0.000091 | 0.899532 | 0.100378 | 0 | 0 | 0 | 0 | 0 |
| 811 | 0.00002 | 0.00007 | 0.900155 | 0.099755 | 0 | 0 | 0 | 0 | 0 |
| 812 | 0.00003 | 0.00003 | 0.903102 | 0.096837 | 0 | 0 | 0 | 0 | 0 |
| 813 | 0.00003 | 0.00008 | 0.902843 | 0.097047 | 0 | 0 | 0 | 0 | 0 |
| 814 | 0.00001 | 0.00014 | 0.9025 | 0.09735 | 0 | 0 | 0 | 0 | 0 |
| 815 | 0.00001 | 0.00007 | 0.89893 | 0.10099 | 0 | 0 | 0 | 0 | 0 |
| 816 | 0.00003 | 0.00008 | 0.901905 | 0.097985 | 0 | 0 | 0 | 0 | 0 |
| 817 | 0.00001 | 0.00007 | 0.902994 | 0.096926 | 0 | 0 | 0 | 0 | 0 |
| 818 | 0.00001 | 0.00006 | 0.899673 | 0.100257 | 0 | 0 | 0 | 0 | 0 |
| 819 | 0 | 0.00008 | 0.900176 | 0.099744 | 0 | 0 | 0 | 0 | 0 |
| 820 | 0.00001 | 0.00007 | 0.898671 | 0.101249 | 0 | 0 | 0 | 0 | 0 |
| 821 | 0.00002 | 0.000081 | 0.902693 | 0.097206 | 0 | 0 | 0 | 0 | 0 |
| 822 | 0.00001 | 0.00006 | 0.90153 | 0.098401 | 0 | 0 | 0 | 0 | 0 |
| 823 | 0.00001 | 0.00006 | 0.89852 | 0.10141 | 0 | 0 | 0 | 0 | 0 |
| 824 | 0.00001 | 0.00005 | 0.906633 | 0.093307 | 0 | 0 | 0 | 0 | 0 |
| 825 | 0.00001 | 0.00003 | 0.902731 | 0.097229 | 0 | 0 | 0 | 0 | 0 |
| 826 | 0 | 0.00013 | 0.903946 | 0.095924 | 0 | 0 | 0 | 0 | 0 |
| 827 | 0.00002 | 0.000121 | 0.899932 | 0.099928 | 0 | 0 | 0 | 0 | 0 |
| 828 | 0 | 0.00002 | 0.901272 | 0.098708 | 0 | 0 | 0 | 0 | 0 |
| 829 | 0 | 0.000061 | 0.89903 | 0.100909 | 0 | 0 | 0 | 0 | 0 |
| 830 | 0 | 0.000089 | 0.901931 | 0.09798 | 0 | 0 | 0 | 0 | 0 |
| 831 | 0 | 0.00013 | 0.896295 | 0.103576 | 0 | 0 | 0 | 0 | 0 |
| 832 | 0 | 0.00008 | 0.899918 | 0.100002 | 0 | 0 | 0 | 0 | 0 |
| 833 | 0.00002 | 0.00009 | 0.90139 | 0.0985 | 0 | 0 | 0 | 0 | 0 |
| 834 | 0.00002 | 0.00005 | 0.900329 | 0.099601 | 0 | 0 | 0 | 0 | 0 |
| 835 | 0 | 0.00003 | 0.898443 | 0.101527 | 0 | 0 | 0 | 0 | 0 |
| 836 | 0 | 0.00009 | 0.899678 | 0.100232 | 0 | 0 | 0 | 0 | 0 |
| 837 | 0.00001 | 0.00008 | 0.903711 | 0.096199 | 0 | 0 | 0 | 0 | 0 |
| 838 | 0.00001 | 0.000089 | 0.899556 | 0.100345 | 0 | 0 | 0 | 0 | 0 |
| 839 | 0.00003 | 0.00003 | 0.897563 | 0.102377 | 0 | 0 | 0 | 0 | 0 |
| 840 | 0.00002 | 0.00003 | 0.901008 | 0.098942 | 0 | 0 | 0 | 0 | 0 |
| 841 | 0 | 0.00012 | 0.902315 | 0.097565 | 0 | 0 | 0 | 0 | 0 |
| 842 | 0 | 0.00007 | 0.898723 | 0.101207 | 0 | 0 | 0 | 0 | 0 |
| 843 | 0.00001 | 0.000071 | 0.902322 | 0.097597 | 0 | 0 | 0 | 0 | 0 |
| 844 | 0.00002 | 0.00006 | 0.901635 | 0.098285 | 0 | 0 | 0 | 0 | 0 |
| 845 | 0 | 0.00003 | 0.897407 | 0.102563 | 0 | 0 | 0 | 0 | 0 |
| 846 | 0 | 0.000008 | 0.961562 | 0.03843 | 0 | 0 | 0 | 0 | 0 |
| 847 | 0.00001 | 0.00008 | 0.897893 | 0.102017 | 0 | 0 | 0 | 0 | 0 |
| 848 | 0.00001 | 0.00002 | 0.903781 | 0.096189 | 0 | 0 | 0 | 0 | 0 |
| 849 | 0.00001 | 0.000069 | 0.905396 | 0.094525 | 0 | 0 | 0 | 0 | 0 |
| 850 | 0.00001 | 0.00001 | 0.900454 | 0.099526 | 0 | 0 | 0 | 0 | 0 |
| 851 | 0.00001 | 0.00005 | 0.902498 | 0.097442 | 0 | 0 | 0 | 0 | 0 |
| 852 | 0.00002 | 0.00004 | 0.897668 | 0.102272 | 0 | 0 | 0 | 0 | 0 |
| 853 | 0.00003 | 0.0001 | 0.898746 | 0.101124 | 0 | 0 | 0 | 0 | 0 |
| 854 | 0.00001 | 0.00006 | 0.89951 | 0.10042 | 0 | 0 | 0 | 0 | 0 |
| 855 | 0.00003 | 0.00006 | 0.901314 | 0.098596 | 0 | 0 | 0 | 0 | 0 |
| 856 | 0.00001 | 0.00005 | 0.897286 | 0.102654 | 0 | 0 | 0 | 0 | 0 |
| 857 | 0 | 0.00003 | 0.899763 | 0.100208 | 0 | 0 | 0 | 0 | 0 |
| 858 | 0.00001 | 0.00009 | 0.900542 | 0.099357 | 0 | 0 | 0 | 0 | 0 |
| 859 | 0.00001 | 0.00012 | 0.901833 | 0.098036 | 0 | 0 | 0 | 0 | 0 |
| 860 | 0.00002 | 0.000119 | 0.899649 | 0.100212 | 0 | 0 | 0 | 0 | 0 |
| 861 | 0.00001 | 0.00011 | 0.904569 | 0.095251 | 6E-05 | 0 | 0 | 0 | 0 |
| 862 | 0.00002 | 0.00007 | 0.900153 | 0.099757 | 0 | 0 | 0 | 0 | 0 |
| 863 | 0.00002 | 0.000071 | 0.900352 | 0.099557 | 0 | 0 | 0 | 0 | 0 |
| 864 | 0.00002 | 0.00009 | 0.901415 | 0.098476 | 0 | 0 | 0 | 0 | 0 |
| 865 | 0.00002 | 0.00007 | 0.900039 | 0.099871 | 0 | 0 | 0 | 0 | 0 |
| 866 | 0 | 0.00004 | 0.899551 | 0.100409 | 0 | 0 | 0 | 0 | 0 |
| 867 | 0.00001 | 0.00008 | 0.901753 | 0.098156 | 0 | 0 | 0 | 0 | 0 |
| 868 | 0 | 0.00009 | 0.895321 | 0.104589 | 0 | 0 | 0 | 0 | 0 |
| 869 | 0.00001 | 0.00001 | 0.8995 | 0.10048 | 0 | 0 | 0 | 0 | 0 |
| 870 | 0 | 0.00002 | 0.901379 | 0.098601 | 0 | 0 | 0 | 0 | 0 |
| 871 | 0.00001 | 0.00006 | 0.90523 | 0.0947 | 0 | 0 | 0 | 0 | 0 |
| 872 | 0.00001 | 0.00011 | 0.899997 | 0.099883 | 0 | 0 | 0 | 0 | 0 |
| 873 | 0.00002 | 0.00009 | 0.897962 | 0.101929 | 0 | 0 | 0 | 0 | 0 |
| 874 | 0.00003 | 0.00007 | 0.898619 | 0.101281 | 0 | 0 | 0 | 0 | 0 |
| 875 | 0.00001 | 0.00003 | 0.901884 | 0.098076 | 0 | 0 | 0 | 0 | 0 |
| 876 | 0 | 0.00009 | 0.901668 | 0.098242 | 0 | 0 | 0 | 0 | 0 |
| 877 | 0 | 0.00004 | 0.90199 | 0.09797 | 0 | 0 | 0 | 0 | 0 |
| 878 | 0.00001 | 0.00004 | 0.90318 | 0.09677 | 0 | 0 | 0 | 0 | 0 |
| 879 | 0.00002 | 0.00004 | 0.89942 | 0.10052 | 0 | 0 | 0 | 0 | 0 |
| 880 | 0.00001 | 0.000079 | 0.901885 | 0.098026 | 0 | 0 | 0 | 0 | 0 |
| 881 | 0 | 0.00001 | 0.899066 | 0.100924 | 0 | 0 | 0 | 0 | 0 |
| 882 | 0 | 0.00009 | 0.898239 | 0.101671 | 0 | 0 | 0 | 0 | 0 |
| 883 | 0 | 0.00009 | 0.899929 | 0.099981 | 0 | 0 | 0 | 0 | 0 |
| 884 | 0.00005 | 0.00007 | 0.898567 | 0.101312 | 0 | 0 | 0 | 0 | 0 |
| 885 | 0.00001 | 0.000079 | 0.905313 | 0.094598 | 0 | 0 | 0 | 0 | 0 |
| 886 | 0.00002 | 0.00003 | 0.902908 | 0.097042 | 0 | 0 | 0 | 0 | 0 |
| 887 | 0.00002 | 0.00005 | 0.900438 | 0.099491 | 0 | 0 | 0 | 0 | 0 |
| 888 | 0.00001 | 0.00005 | 0.896211 | 0.103729 | 0 | 0 | 0 | 0 | 0 |
| 889 | 0.00001 | 0.000121 | 0.892785 | 0.107084 | 0 | 0 | 0 | 0 | 0 |
| 890 | 0.00002 | 0.000081 | 0.903856 | 0.096043 | 0 | 0 | 0 | 0 | 0 |
| 891 | 0 | 0.000089 | 0.897616 | 0.102295 | 0 | 0 | 0 | 0 | 0 |
| 892 | 0.00003 | 0.00003 | 0.901753 | 0.098187 | 0 | 0 | 0 | 0 | 0 |
| 893 | 0.00002 | 0.00014 | 0.899852 | 0.099988 | 0 | 0 | 0 | 0 | 0 |
| 894 | 0.000051 | 0.000061 | 0.899983 | 0.099906 | 0 | 0 | 0 | 0 | 0 |
| 895 | 0.00001 | 0.00006 | 0.907602 | 0.092328 | 0 | 0 | 0 | 0 | 0 |
| 896 | 0.00003 | 0.00002 | 0.900326 | 0.099624 | 0 | 0 | 0 | 0 | 0 |
| 897 | 0.00001 | 0.00006 | 0.903323 | 0.096607 | 0 | 0 | 0 | 0 | 0 |
| 898 | 0.00001 | 0.00002 | 0.901833 | 0.098137 | 0 | 0 | 0 | 0 | 0 |
| 899 | 0.00004 | 0.00007 | 0.903408 | 0.096483 | 0 | 0 | 0 | 0 | 0 |
| 900 | 0.00001 | 0.00008 | 0.900636 | 0.099274 | 0 | 0 | 0 | 0 | 0 |
| 901 | 0.00003 | 0.00007 | 0.905108 | 0.094791 | 0 | 0 | 0 | 0 | 0 |
| 902 | 0 | 0.00003 | 0.899037 | 0.100933 | 0 | 0 | 0 | 0 | 0 |
| 903 | 0.00001 | 0.000129 | 0.900581 | 0.09928 | 0 | 0 | 0 | 0 | 0 |
| 904 | 0.00001 | 0.00006 | 0.903218 | 0.096712 | 0 | 0 | 0 | 0 | 0 |
| 905 | 0 | 0.00007 | 0.902911 | 0.097018 | 0 | 0 | 0 | 0 | 0 |
| 906 | 0.00001 | 0.000081 | 0.901113 | 0.098796 | 0 | 0 | 0 | 0 | 0 |
| 907 | 0.00003 | 0.00003 | 0.906864 | 0.093076 | 0 | 0 | 0 | 0 | 0 |
| 908 | 0.00001 | 0.0001 | 0.90204 | 0.097849 | 0 | 0 | 0 | 0 | 0 |
| 909 | 0.00003 | 0.00004 | 0.900124 | 0.099806 | 0 | 0 | 0 | 0 | 0 |
| 910 | 0.00002 | 0.00009 | 0.899861 | 0.100028 | 0 | 0 | 0 | 0 | 0 |
| 911 | 0.00002 | 0.00002 | 0.90456 | 0.0954 | 0 | 0 | 0 | 0 | 0 |
| 912 | 0.00002 | 0.00004 | 0.898343 | 0.101597 | 0 | 0 | 0 | 0 | 0 |
| 913 | 0.00003 | 0.0001 | 0.902542 | 0.097328 | 0 | 0 | 0 | 0 | 0 |
| 914 | 0.00002 | 0.00002 | 0.903232 | 0.096728 | 0 | 0 | 0 | 0 | 0 |
| 915 | 0.00002 | 0.00001 | 0.897242 | 0.102728 | 0 | 0 | 0 | 0 | 0 |
| 916 | 0.00002 | 0.00008 | 0.901908 | 0.097992 | 0 | 0 | 0 | 0 | 0 |
| 917 | 0.00002 | 0.00007 | 0.901357 | 0.098554 | 0 | 0 | 0 | 0 | 0 |
| 918 | 0 | 0.00006 | 0.904212 | 0.095728 | 0 | 0 | 0 | 0 | 0 |
| 919 | 0 | 0.00006 | 0.900197 | 0.099743 | 0 | 0 | 0 | 0 | 0 |
| 920 | 0 | 0.00009 | 0.905099 | 0.094811 | 0 | 0 | 0 | 0 | 0 |
| 921 | 0.00001 | 0.00008 | 0.902134 | 0.097775 | 0 | 0 | 0 | 0 | 0 |
| 922 | 0.00001 | 0.00006 | 0.902094 | 0.097837 | 0 | 0 | 0 | 0 | 0 |
| 923 | 0.00001 | 0.00003 | 0.901574 | 0.098386 | 0 | 0 | 0 | 0 | 0 |
| 924 | 0 | 0.00004 | 0.901579 | 0.098381 | 0 | 0 | 0 | 0 | 0 |
| 925 | 0.00001 | 0.000129 | 0.901192 | 0.098669 | 0 | 0 | 0 | 0 | 0 |
| 926 | 0 | 0.0001 | 0.903803 | 0.096098 | 0 | 0 | 0 | 0 | 0 |
| 927 | 0.00001 | 0.00006 | 0.89833 | 0.1016 | 0 | 0 | 0 | 0 | 0 |
| 928 | 0.00002 | 0.00008 | 0.899915 | 0.099985 | 0 | 0 | 0 | 0 | 0 |
| 929 | 0.00001 | 0.00015 | 0.896361 | 0.103479 | 0 | 0 | 0 | 0 | 0 |
| 930 | 0.00001 | 0.00005 | 0.901549 | 0.098391 | 0 | 0 | 0 | 0 | 0 |
| 931 | 0.00001 | 0.00005 | 0.905855 | 0.094086 | 0 | 0 | 0 | 0 | 0 |
| 932 | 0.00001 | 0.00006 | 0.903658 | 0.096272 | 0 | 0 | 0 | 0 | 0 |
| 933 | 0.00003 | 0.00005 | 0.905662 | 0.094257 | 0 | 0 | 0 | 0 | 0 |
| 934 | 0.00001 | 0.00003 | 0.898711 | 0.10125 | 0 | 0 | 0 | 0 | 0 |
| 935 | 0.00001 | 0.00005 | 0.901303 | 0.098637 | 0 | 0 | 0 | 0 | 0 |
| 936 | 0.00002 | 0.00006 | 0.903925 | 0.095995 | 0 | 0 | 0 | 0 | 0 |
| 937 | 0.00003 | 0.00007 | 0.903682 | 0.096218 | 0 | 0 | 0 | 0 | 0 |
| 938 | 0.00002 | 0.00004 | 0.90098 | 0.09896 | 0 | 0 | 0 | 0 | 0 |
| 939 | 0 | 0.00001 | 0.903652 | 0.096338 | 0 | 0 | 0 | 0 | 0 |
| 940 | 0.00001 | 0.00006 | 0.897962 | 0.101968 | 0 | 0 | 0 | 0 | 0 |
| 941 | 0.00001 | 0.00003 | 0.901698 | 0.098262 | 0 | 0 | 0 | 0 | 0 |
| 942 | 0.00003 | 0.00006 | 0.904075 | 0.095835 | 0 | 0 | 0 | 0 | 0 |
| 943 | 0.00003 | 0.00002 | 0.905734 | 0.094216 | 0 | 0 | 0 | 0 | 0 |
| 944 | 0.00001 | 0.00005 | 0.900336 | 0.099604 | 0 | 0 | 0 | 0 | 0 |
| 945 | 0.00001 | 0.00002 | 0.896578 | 0.103392 | 0 | 0 | 0 | 0 | 0 |
| 946 | 0.00002 | 0.00004 | 0.901521 | 0.098419 | 0 | 0 | 0 | 0 | 0 |
| 947 | 0.00001 | 0.00004 | 0.898078 | 0.101872 | 0 | 0 | 0 | 0 | 0 |
| 948 | 0.00004 | 0.00005 | 0.900036 | 0.099874 | 0 | 0 | 0 | 0 | 0 |
| 949 | 0 | 0.00005 | 0.904765 | 0.095184 | 0 | 0 | 0 | 0 | 0 |
| 950 | 0.00001 | 0.00004 | 0.90367 | 0.09628 | 0 | 0 | 0 | 0 | 0 |
| 951 | 0 | 0.000081 | 0.90437 | 0.095549 | 0 | 0 | 0 | 0 | 0 |
| 952 | 0 | 0.00009 | 0.902071 | 0.097839 | 0 | 0 | 0 | 0 | 0 |
| 953 | 0 | 0.00001 | 0.901595 | 0.098395 | 0 | 0 | 0 | 0 | 0 |
| 954 | 0 | 0.00006 | 0.897847 | 0.102093 | 0 | 0 | 0 | 0 | 0 |
| 955 | 0.00002 | 0.00009 | 0.901952 | 0.097938 | 0 | 0 | 0 | 0 | 0 |
| 956 | 0.00003 | 0.00008 | 0.903337 | 0.096553 | 0 | 0 | 0 | 0 | 0 |
| 957 | 0 | 0.00009 | 0.896351 | 0.103559 | 0 | 0 | 0 | 0 | 0 |
| 958 | 0.00002 | 0.00002 | 0.90219 | 0.09777 | 0 | 0 | 0 | 0 | 0 |
| 959 | 0 | 0.00006 | 0.89843 | 0.10151 | 0 | 0 | 0 | 0 | 0 |
| 960 | 0.00002 | 0.00005 | 0.902934 | 0.096997 | 0 | 0 | 0 | 0 | 0 |
| 961 | 0 | 0.00007 | 0.900573 | 0.099358 | 0 | 0 | 0 | 0 | 0 |
| 962 | 0.00001 | 0.00005 | 0.900781 | 0.099159 | 0 | 0 | 0 | 0 | 0 |
| 963 | 0.00001 | 0.00003 | 0.900705 | 0.099255 | 0 | 0 | 0 | 0 | 0 |
| 964 | 0.00002 | 0.00001 | 0.90166 | 0.09831 | 0 | 0 | 0 | 0 | 0 |
| 965 | 0 | 0.00003 | 0.903043 | 0.096928 | 0 | 0 | 0 | 0 | 0 |
| 966 | 0 | 0.00013 | 0.9038 | 0.09607 | 0 | 0 | 0 | 0 | 0 |
| 967 | 0.00001 | 0.00005 | 0.903088 | 0.096852 | 0 | 0 | 0 | 0 | 0 |
| 968 | 0.00003 | 0.000111 | 0.898376 | 0.101483 | 0 | 0 | 0 | 0 | 0 |
| 969 | 0 | 0.00015 | 0.901727 | 0.098122 | 0 | 0 | 0 | 0 | 0 |
| 970 | 0.00002 | 0.0001 | 0.900005 | 0.099875 | 0 | 0 | 0 | 0 | 0 |
| 971 | 0.00002 | 0.0001 | 0.903132 | 0.096748 | 0 | 0 | 0 | 0 | 0 |
| 972 | 0.00002 | 0.00008 | 0.897801 | 0.102099 | 0 | 0 | 0 | 0 | 0 |
| 973 | 0 | 0.00009 | 0.901329 | 0.098581 | 0 | 0 | 0 | 0 | 0 |
| 974 | 0.00002 | 0.00002 | 0.899795 | 0.100165 | 0 | 0 | 0 | 0 | 0 |
| 975 | 0 | 0.000109 | 0.902611 | 0.09728 | 0 | 0 | 0 | 0 | 0 |
| 976 | 0.00001 | 0.00003 | 0.902266 | 0.097694 | 0 | 0 | 0 | 0 | 0 |
| 977 | 0.00002 | 0.0001 | 0.900067 | 0.099814 | 0 | 0 | 0 | 0 | 0 |
| 978 | 0.00001 | 0.00004 | 0.900497 | 0.099453 | 0 | 0 | 0 | 0 | 0 |
| 979 | 0.00002 | 0.00006 | 0.901267 | 0.098652 | 0 | 0 | 0 | 0 | 0 |
| 980 | 0.00001 | 0.00008 | 0.902769 | 0.097141 | 0 | 0 | 0 | 0 | 0 |
| 981 | 0 | 0.00006 | 0.902522 | 0.097418 | 0 | 0 | 0 | 0 | 0 |
| 982 | 0 | 0.00002 | 0.89872 | 0.10126 | 0 | 0 | 0 | 0 | 0 |
| 983 | 0 | 0.00007 | 0.897296 | 0.102634 | 0 | 0 | 0 | 0 | 0 |
| 984 | 0.00001 | 0.00007 | 0.900468 | 0.099452 | 0 | 0 | 0 | 0 | 0 |
| 985 | 0.00001 | 0.00003 | 0.902221 | 0.097739 | 0 | 0 | 0 | 0 | 0 |
| 986 | 0.00003 | 0.000051 | 0.903771 | 0.096148 | 0 | 0 | 0 | 0 | 0 |
| 987 | 0 | 0.00003 | 0.906067 | 0.093903 | 0 | 0 | 0 | 0 | 0 |
| 988 | 0.00001 | 0.00003 | 0.901754 | 0.098206 | 0 | 0 | 0 | 0 | 0 |
| 989 | 0 | 0.00009 | 0.896433 | 0.103477 | 0 | 0 | 0 | 0 | 0 |
| 990 | 0.00002 | 0.0001 | 0.899283 | 0.100597 | 0 | 0 | 0 | 0 | 0 |
| 991 | 0.00003 | 0.00014 | 0.901241 | 0.09859 | 0 | 0 | 0 | 0 | 0 |
| 992 | 0 | 0.000109 | 0.901037 | 0.098854 | 0 | 0 | 0 | 0 | 0 |
| 993 | 0.00001 | 0.00009 | 0.905362 | 0.094538 | 0 | 0 | 0 | 0 | 0 |
| 994 | 0.00001 | 0.00011 | 0.905417 | 0.094463 | 0 | 0 | 0 | 0 | 0 |
| 995 | 0 | 0.00006 | 0.899473 | 0.100467 | 0 | 0 | 0 | 0 | 0 |
| 996 | 0.00002 | 0.000069 | 0.905986 | 0.093925 | 0 | 0 | 0 | 0 | 0 |
| 997 | 0 | 0.00006 | 0.899866 | 0.100074 | 0 | 0 | 0 | 0 | 0 |
| 998 | 0.00001 | 0.00003 | 0.902561 | 0.097399 | 0 | 0 | 0 | 0 | 0 |
| 999 | 0 | 0.00006 | 0.904331 | 0.095609 | 0 | 0 | 0 | 0 | 0 |
| 1000 | 0.00001 | 0.0001 | 0.898777 | 0.101113 | 0 | 0 | 0 | 0 | 0 |

| Year 9 | Race |  |  |  |  |  |  |  |  |
| --- | --- | --- | --- | --- | --- | --- | --- | --- | --- |
| Trial | 003.0 | 005.0 | 007.0 | 037.1 | 043.0 | 303.0 | 003.2 | 403.0 | 003.4 |
| 1 | 0 | 0.00003 | 0.909528 | 0.090442 | 0 | 0 | 0 | 0 | 0 |
| 2 | 0.00002 | 0.00002 | 0.90536 | 0.0946 | 0 | 0 | 0 | 0 | 0 |
| 3 | 0 | 0.00003 | 0.911056 | 0.088914 | 0 | 0 | 0 | 0 | 0 |
| 4 | 0.00004 | 0.00004 | 0.909456 | 0.090465 | 0 | 0 | 0 | 0 | 0 |
| 5 | 0.00003 | 0.00002 | 0.90844 | 0.09151 | 0 | 0 | 0 | 0 | 0 |
| 6 | 0 | 0.00001 | 0.908729 | 0.091261 | 0 | 0 | 0 | 0 | 0 |
| 7 | 0.00001 | 0.00002 | 0.908563 | 0.091407 | 0 | 0 | 0 | 0 | 0 |
| 8 | 0 | 0.00002 | 0.910656 | 0.089324 | 0 | 0 | 0 | 0 | 0 |
| 9 | 0.00001 | 0.00003 | 0.905182 | 0.094778 | 0 | 0 | 0 | 0 | 0 |
| 10 | 0 | 0.00008 | 0.90823 | 0.09169 | 0 | 0 | 0 | 0 | 0 |
| 11 | 0 | 0.00004 | 0.909986 | 0.089974 | 0 | 0 | 0 | 0 | 0 |
| 12 | 0 | 0.00004 | 0.904134 | 0.095826 | 0 | 0 | 0 | 0 | 0 |
| 13 | 0.00001 | 0.00009 | 0.908206 | 0.091694 | 0 | 0 | 0 | 0 | 0 |
| 14 | 0.00001 | 0.00002 | 0.903443 | 0.096527 | 0 | 0 | 0 | 0 | 0 |
| 15 | 0 | 0.00002 | 0.902923 | 0.097057 | 0 | 0 | 0 | 0 | 0 |
| 16 | 0.00001 | 0.00001 | 0.908411 | 0.091569 | 0 | 0 | 0 | 0 | 0 |
| 17 | 0 | 0.00006 | 0.908871 | 0.091069 | 0 | 0 | 0 | 0 | 0 |
| 18 | 0 | 0.00002 | 0.907862 | 0.092118 | 0 | 0 | 0 | 0 | 0 |
| 19 | 0.00001 | 0.00003 | 0.908769 | 0.091191 | 0 | 0 | 0 | 0 | 0 |
| 20 | 0.00001 | 0.00002 | 0.907799 | 0.092171 | 0 | 0 | 0 | 0 | 0 |
| 21 | 0.00001 | 0.00005 | 0.908858 | 0.091082 | 0 | 0 | 0 | 0 | 0 |
| 22 | 0 | 0.00007 | 0.90583 | 0.0941 | 0 | 0 | 0 | 0 | 0 |
| 23 | 0 | 0.00003 | 0.910018 | 0.089952 | 0 | 0 | 0 | 0 | 0 |
| 24 | 0.00002 | 0.00002 | 0.907295 | 0.092665 | 0 | 0 | 0 | 0 | 0 |
| 25 | 0.00003 | 0 | 0.908422 | 0.091548 | 0 | 0 | 0 | 0 | 0 |
| 26 | 0.00002 | 0.00004 | 0.911379 | 0.088561 | 0 | 0 | 0 | 0 | 0 |
| 27 | 0.00003 | 0 | 0.908744 | 0.091226 | 0 | 0 | 0 | 0 | 0 |
| 28 | 0.00001 | 0 | 0.910084 | 0.089566 | 3E-04 | 0 | 0 | 0 | 0 |
| 29 | 0 | 0.00001 | 0.911523 | 0.088467 | 0 | 0 | 0 | 0 | 0 |
| 30 | 0.00002 | 0.000041 | 0.906153 | 0.093786 | 0 | 0 | 0 | 0 | 0 |
| 31 | 0.00001 | 0.00001 | 0.911214 | 0.088766 | 0 | 0 | 0 | 0 | 0 |
| 32 | 0.00002 | 0.00003 | 0.909676 | 0.090274 | 0 | 0 | 0 | 0 | 0 |
| 33 | 0 | 0.00001 | 0.908809 | 0.091181 | 0 | 0 | 0 | 0 | 0 |
| 34 | 0.00001 | 0.00002 | 0.90916 | 0.09081 | 0 | 0 | 0 | 0 | 0 |
| 35 | 0 | 0.00001 | 0.904595 | 0.095395 | 0 | 0 | 0 | 0 | 0 |
| 36 | 0.00001 | 0.00004 | 0.906938 | 0.093013 | 0 | 0 | 0 | 0 | 0 |
| 37 | 0 | 0.00002 | 0.912728 | 0.087252 | 0 | 0 | 0 | 0 | 0 |
| 38 | 0.00003 | 0 | 0.910079 | 0.089891 | 0 | 0 | 0 | 0 | 0 |
| 39 | 0.00001 | 0.00001 | 0.909633 | 0.090347 | 0 | 0 | 0 | 0 | 0 |
| 40 | 0.00002 | 0.00004 | 0.902083 | 0.097857 | 0 | 0 | 0 | 0 | 0 |
| 41 | 0.00002 | 0.00003 | 0.905994 | 0.093956 | 0 | 0 | 0 | 0 | 0 |
| 42 | 0.00003 | 0.000069 | 0.911001 | 0.0889 | 0 | 0 | 0 | 0 | 0 |
| 43 | 0 | 0 | 0.906201 | 0.093799 | 0 | 0 | 0 | 0 | 0 |
| 44 | 0.00001 | 0.00002 | 0.910487 | 0.089483 | 0 | 0 | 0 | 0 | 0 |
| 45 | 0.00002 | 0.00003 | 0.906883 | 0.093067 | 0 | 0 | 0 | 0 | 0 |
| 46 | 0.00001 | 0 | 0.906959 | 0.093031 | 0 | 0 | 0 | 0 | 0 |
| 47 | 0 | 0.00002 | 0.907409 | 0.092571 | 0 | 0 | 0 | 0 | 0 |
| 48 | 0 | 0.00006 | 0.907998 | 0.091941 | 0 | 0 | 0 | 0 | 0 |
| 49 | 0.00001 | 0.00004 | 0.909366 | 0.090584 | 0 | 0 | 0 | 0 | 0 |
| 50 | 0 | 0.00004 | 0.910423 | 0.089537 | 0 | 0 | 0 | 0 | 0 |
| 51 | 0 | 0.00001 | 0.907275 | 0.092715 | 0 | 0 | 0 | 0 | 0 |
| 52 | 0.00001 | 0.00005 | 0.911485 | 0.088455 | 0 | 0 | 0 | 0 | 0 |
| 53 | 0.00002 | 0.00003 | 0.909703 | 0.090247 | 0 | 0 | 0 | 0 | 0 |
| 54 | 0.00001 | 0.00006 | 0.907114 | 0.092817 | 0 | 0 | 0 | 0 | 0 |
| 55 | 0.00002 | 0.00005 | 0.908672 | 0.091258 | 0 | 0 | 0 | 0 | 0 |
| 56 | 0.00002 | 0.00002 | 0.909101 | 0.090859 | 0 | 0 | 0 | 0 | 0 |
| 57 | 0.00006 | 0.00003 | 0.908953 | 0.090957 | 0 | 0 | 0 | 0 | 0 |
| 58 | 0.00002 | 0.000059 | 0.909021 | 0.0909 | 0 | 0 | 0 | 0 | 0 |
| 59 | 0 | 0.00002 | 0.912226 | 0.087754 | 0 | 0 | 0 | 0 | 0 |
| 60 | 0.00002 | 0.00003 | 0.909131 | 0.090819 | 0 | 0 | 0 | 0 | 0 |
| 61 | 0.00001 | 0.00003 | 0.906864 | 0.093096 | 0 | 0 | 0 | 0 | 0 |
| 62 | 0.00001 | 0.00001 | 0.909011 | 0.090969 | 0 | 0 | 0 | 0 | 0 |
| 63 | 0.00001 | 0.00001 | 0.907248 | 0.092732 | 0 | 0 | 0 | 0 | 0 |
| 64 | 0 | 0.00002 | 0.905383 | 0.094597 | 0 | 0 | 0 | 0 | 0 |
| 65 | 0.00001 | 0.00004 | 0.90939 | 0.09056 | 0 | 0 | 0 | 0 | 0 |
| 66 | 0.00001 | 0.00004 | 0.912063 | 0.087887 | 0 | 0 | 0 | 0 | 0 |
| 67 | 0.00003 | 0.00003 | 0.912746 | 0.087193 | 0 | 0 | 0 | 0 | 0 |
| 68 | 0.00004 | 0.00004 | 0.90769 | 0.092231 | 0 | 0 | 0 | 0 | 0 |
| 69 | 0 | 0.00001 | 0.907809 | 0.092181 | 0 | 0 | 0 | 0 | 0 |
| 70 | 0.00001 | 0.00002 | 0.909696 | 0.090274 | 0 | 0 | 0 | 0 | 0 |
| 71 | 0.00001 | 0.00008 | 0.906288 | 0.09278 | 8E-04 | 0 | 0 | 0 | 0 |
| 72 | 0.00002 | 0.00003 | 0.91007 | 0.089879 | 0 | 0 | 0 | 0 | 0 |
| 73 | 0.00001 | 0.00004 | 0.907449 | 0.092501 | 0 | 0 | 0 | 0 | 0 |
| 74 | 0.00001 | 0.00004 | 0.911896 | 0.088055 | 0 | 0 | 0 | 0 | 0 |
| 75 | 0 | 0.00005 | 0.909208 | 0.090742 | 0 | 0 | 0 | 0 | 0 |
| 76 | 0.00001 | 0.00002 | 0.90924 | 0.09073 | 0 | 0 | 0 | 0 | 0 |
| 77 | 0.00001 | 0.00002 | 0.907275 | 0.092695 | 0 | 0 | 0 | 0 | 0 |
| 78 | 0.00003 | 0.00001 | 0.908303 | 0.091657 | 0 | 0 | 0 | 0 | 0 |
| 79 | 0.00003 | 0.00001 | 0.910259 | 0.089701 | 0 | 0 | 0 | 0 | 0 |
| 80 | 0.00001 | 0.00001 | 0.907983 | 0.091998 | 0 | 0 | 0 | 0 | 0 |
| 81 | 0.00001 | 0.00007 | 0.906684 | 0.093236 | 0 | 0 | 0 | 0 | 0 |
| 82 | 0.00003 | 0.000039 | 0.903924 | 0.096007 | 0 | 0 | 0 | 0 | 0 |
| 83 | 0 | 0.00001 | 0.912089 | 0.087901 | 0 | 0 | 0 | 0 | 0 |
| 84 | 0.00001 | 0 | 0.910989 | 0.089001 | 0 | 0 | 0 | 0 | 0 |
| 85 | 0.00002 | 0.00003 | 0.907668 | 0.092283 | 0 | 0 | 0 | 0 | 0 |
| 86 | 0.00002 | 0 | 0.91688 | 0.0831 | 0 | 0 | 0 | 0 | 0 |
| 87 | 0.00003 | 0.00001 | 0.907728 | 0.092232 | 0 | 0 | 0 | 0 | 0 |
| 88 | 0 | 0 | 0.907918 | 0.092082 | 0 | 0 | 0 | 0 | 0 |
| 89 | 0 | 0.00004 | 0.905538 | 0.094422 | 0 | 0 | 0 | 0 | 0 |
| 90 | 0.00001 | 0 | 0.909825 | 0.090165 | 0 | 0 | 0 | 0 | 0 |
| 91 | 0.00001 | 0 | 0.908226 | 0.091764 | 0 | 0 | 0 | 0 | 0 |
| 92 | 0 | 0.00003 | 0.908392 | 0.091577 | 0 | 0 | 0 | 0 | 0 |
| 93 | 0.00001 | 0.00003 | 0.912581 | 0.087379 | 0 | 0 | 0 | 0 | 0 |
| 94 | 0.00001 | 0.00003 | 0.909788 | 0.090171 | 0 | 0 | 0 | 0 | 0 |
| 95 | 0 | 0.00004 | 0.908809 | 0.091151 | 0 | 0 | 0 | 0 | 0 |
| 96 | 0.00001 | 0.00004 | 0.908891 | 0.091059 | 0 | 0 | 0 | 0 | 0 |
| 97 | 0.00001 | 0.00003 | 0.906317 | 0.093643 | 0 | 0 | 0 | 0 | 0 |
| 98 | 0.00001 | 0.00003 | 0.909114 | 0.090846 | 0 | 0 | 0 | 0 | 0 |
| 99 | 0.00003 | 0.00001 | 0.905318 | 0.094642 | 0 | 0 | 0 | 0 | 0 |
| 100 | 0.00002 | 0 | 0.911319 | 0.088661 | 0 | 0 | 0 | 0 | 0 |
| 101 | 0.00001 | 0.00003 | 0.911305 | 0.088655 | 0 | 0 | 0 | 0 | 0 |
| 102 | 0.00001 | 0.00002 | 0.911637 | 0.088333 | 0 | 0 | 0 | 0 | 0 |
| 103 | 0 | 0.00003 | 0.908278 | 0.091693 | 0 | 0 | 0 | 0 | 0 |
| 104 | 0.00002 | 0.00004 | 0.912345 | 0.087594 | 0 | 0 | 0 | 0 | 0 |
| 105 | 0 | 0.00002 | 0.907004 | 0.092976 | 0 | 0 | 0 | 0 | 0 |
| 106 | 0.00001 | 0.00006 | 0.90875 | 0.09118 | 0 | 0 | 0 | 0 | 0 |
| 107 | 0 | 0.00007 | 0.909748 | 0.089742 | 4E-04 | 0 | 0 | 0 | 0 |
| 108 | 0 | 0.00004 | 0.903202 | 0.096757 | 0 | 0 | 0 | 0 | 0 |
| 109 | 0 | 0.00003 | 0.907989 | 0.091982 | 0 | 0 | 0 | 0 | 0 |
| 110 | 0 | 0.00004 | 0.909063 | 0.090897 | 0 | 0 | 0 | 0 | 0 |
| 111 | 0.00001 | 0.00005 | 0.912653 | 0.087287 | 0 | 0 | 0 | 0 | 0 |
| 112 | 0.00001 | 0.00004 | 0.909095 | 0.090854 | 0 | 0 | 0 | 0 | 0 |
| 113 | 0.00001 | 0.00002 | 0.90899 | 0.09098 | 0 | 0 | 0 | 0 | 0 |
| 114 | 0.00002 | 0.00003 | 0.909954 | 0.089996 | 0 | 0 | 0 | 0 | 0 |
| 115 | 0.00002 | 0.00002 | 0.912152 | 0.087808 | 0 | 0 | 0 | 0 | 0 |
| 116 | 0.00002 | 0.00005 | 0.908399 | 0.091531 | 0 | 0 | 0 | 0 | 0 |
| 117 | 0 | 0.00001 | 0.909706 | 0.090284 | 0 | 0 | 0 | 0 | 0 |
| 118 | 0 | 0.00002 | 0.910891 | 0.089089 | 0 | 0 | 0 | 0 | 0 |
| 119 | 0 | 0.00001 | 0.908058 | 0.091932 | 0 | 0 | 0 | 0 | 0 |
| 120 | 0.00001 | 0.00004 | 0.907633 | 0.092317 | 0 | 0 | 0 | 0 | 0 |
| 121 | 0.00002 | 0.00001 | 0.908578 | 0.091392 | 0 | 0 | 0 | 0 | 0 |
| 122 | 0.00002 | 0.00002 | 0.910296 | 0.089664 | 0 | 0 | 0 | 0 | 0 |
| 123 | 0 | 0.00001 | 0.911257 | 0.088733 | 0 | 0 | 0 | 0 | 0 |
| 124 | 0 | 0.00003 | 0.911686 | 0.088284 | 0 | 0 | 0 | 0 | 0 |
| 125 | 0 | 0.00001 | 0.908145 | 0.091845 | 0 | 0 | 0 | 0 | 0 |
| 126 | 0.00001 | 0 | 0.908663 | 0.091327 | 0 | 0 | 0 | 0 | 0 |
| 127 | 0.00002 | 0 | 0.907618 | 0.092362 | 0 | 0 | 0 | 0 | 0 |
| 128 | 0.00002 | 0.00002 | 0.908531 | 0.091429 | 0 | 0 | 0 | 0 | 0 |
| 129 | 0.00002 | 0.00001 | 0.907162 | 0.092808 | 0 | 0 | 0 | 0 | 0 |
| 130 | 0.00002 | 0.00001 | 0.909726 | 0.090244 | 0 | 0 | 0 | 0 | 0 |
| 131 | 0.00001 | 0.00001 | 0.908918 | 0.091062 | 0 | 0 | 0 | 0 | 0 |
| 132 | 0.00002 | 0 | 0.910276 | 0.089703 | 0 | 0 | 0 | 0 | 0 |
| 133 | 0.00002 | 0.00001 | 0.90667 | 0.093299 | 0 | 0 | 0 | 0 | 0 |
| 134 | 0.00003 | 0.00002 | 0.910275 | 0.089675 | 0 | 0 | 0 | 0 | 0 |
| 135 | 0.00002 | 0.00001 | 0.905771 | 0.0942 | 0 | 0 | 0 | 0 | 0 |
| 136 | 0.00002 | 0.00002 | 0.913392 | 0.086568 | 0 | 0 | 0 | 0 | 0 |
| 137 | 0.00001 | 0 | 0.909276 | 0.090714 | 0 | 0 | 0 | 0 | 0 |
| 138 | 0 | 0.00002 | 0.904952 | 0.095028 | 0 | 0 | 0 | 0 | 0 |
| 139 | 0.00001 | 0.00002 | 0.91047 | 0.0895 | 0 | 0 | 0 | 0 | 0 |
| 140 | 0.00001 | 0 | 0.905477 | 0.094513 | 0 | 0 | 0 | 0 | 0 |
| 141 | 0.00001 | 0.00004 | 0.906846 | 0.093104 | 0 | 0 | 0 | 0 | 0 |
| 142 | 0.00001 | 0.00002 | 0.903861 | 0.096108 | 0 | 0 | 0 | 0 | 0 |
| 143 | 0 | 0 | 0.907749 | 0.092251 | 0 | 0 | 0 | 0 | 0 |
| 144 | 0.00001 | 0.00007 | 0.905861 | 0.094059 | 0 | 0 | 0 | 0 | 0 |
| 145 | 0.00001 | 0.00002 | 0.910161 | 0.089809 | 0 | 0 | 0 | 0 | 0 |
| 146 | 0.00003 | 0.00004 | 0.912021 | 0.08791 | 0 | 0 | 0 | 0 | 0 |
| 147 | 0 | 0.00002 | 0.906861 | 0.093119 | 0 | 0 | 0 | 0 | 0 |
| 148 | 0 | 0.00002 | 0.90965 | 0.09033 | 0 | 0 | 0 | 0 | 0 |
| 149 | 0.00004 | 0.00001 | 0.908628 | 0.091322 | 0 | 0 | 0 | 0 | 0 |
| 150 | 0.00001 | 0.00005 | 0.914497 | 0.085442 | 0 | 0 | 0 | 0 | 0 |
| 151 | 0.00001 | 0.00002 | 0.90863 | 0.09134 | 0 | 0 | 0 | 0 | 0 |
| 152 | 0 | 0 | 0.904575 | 0.095425 | 0 | 0 | 0 | 0 | 0 |
| 153 | 0.00002 | 0.00002 | 0.91248 | 0.08748 | 0 | 0 | 0 | 0 | 0 |
| 154 | 0.00001 | 0.000081 | 0.906482 | 0.093428 | 0 | 0 | 0 | 0 | 0 |
| 155 | 0.00003 | 0.00005 | 0.907938 | 0.091982 | 0 | 0 | 0 | 0 | 0 |
| 156 | 0 | 0.00003 | 0.908084 | 0.091886 | 0 | 0 | 0 | 0 | 0 |
| 157 | 0.00001 | 0.00002 | 0.907329 | 0.092641 | 0 | 0 | 0 | 0 | 0 |
| 158 | 0.00002 | 0.00004 | 0.909143 | 0.090797 | 0 | 0 | 0 | 0 | 0 |
| 159 | 0 | 0 | 0.908122 | 0.091878 | 0 | 0 | 0 | 0 | 0 |
| 160 | 0.00001 | 0.00004 | 0.910075 | 0.089876 | 0 | 0 | 0 | 0 | 0 |
| 161 | 0.00001 | 0.00001 | 0.908731 | 0.091249 | 0 | 0 | 0 | 0 | 0 |
| 162 | 0.00001 | 0.00003 | 0.910283 | 0.089678 | 0 | 0 | 0 | 0 | 0 |
| 163 | 0 | 0 | 0.907883 | 0.092117 | 0 | 0 | 0 | 0 | 0 |
| 164 | 0 | 0.00002 | 0.903595 | 0.096385 | 0 | 0 | 0 | 0 | 0 |
| 165 | 0.00001 | 0.00001 | 0.915096 | 0.084884 | 0 | 0 | 0 | 0 | 0 |
| 166 | 0.00001 | 0.00003 | 0.909422 | 0.090538 | 0 | 0 | 0 | 0 | 0 |
| 167 | 0 | 0.00007 | 0.90978 | 0.090149 | 0 | 0 | 0 | 0 | 0 |
| 168 | 0.00001 | 0.00001 | 0.907114 | 0.092866 | 0 | 0 | 0 | 0 | 0 |
| 169 | 0.00003 | 0.00004 | 0.906426 | 0.093504 | 0 | 0 | 0 | 0 | 0 |
| 170 | 0.00004 | 0.00002 | 0.907609 | 0.092331 | 0 | 0 | 0 | 0 | 0 |
| 171 | 0.00002 | 0.00004 | 0.908888 | 0.091052 | 0 | 0 | 0 | 0 | 0 |
| 172 | 0.00001 | 0.00005 | 0.907812 | 0.092128 | 0 | 0 | 0 | 0 | 0 |
| 173 | 0.00002 | 0.00006 | 0.910577 | 0.089343 | 0 | 0 | 0 | 0 | 0 |
| 174 | 0.00004 | 0.00003 | 0.905234 | 0.094696 | 0 | 0 | 0 | 0 | 0 |
| 175 | 0.00003 | 0.00003 | 0.906033 | 0.093907 | 0 | 0 | 0 | 0 | 0 |
| 176 | 0 | 0.00003 | 0.913798 | 0.086172 | 0 | 0 | 0 | 0 | 0 |
| 177 | 0.00003 | 0.00001 | 0.909357 | 0.090603 | 0 | 0 | 0 | 0 | 0 |
| 178 | 0 | 0.00005 | 0.906349 | 0.093601 | 0 | 0 | 0 | 0 | 0 |
| 179 | 0 | 0.000069 | 0.907545 | 0.092386 | 0 | 0 | 0 | 0 | 0 |
| 180 | 0 | 0.00005 | 0.908163 | 0.091787 | 0 | 0 | 0 | 0 | 0 |
| 181 | 0 | 0.00001 | 0.910792 | 0.089198 | 0 | 0 | 0 | 0 | 0 |
| 182 | 0.00002 | 0.00005 | 0.907484 | 0.092447 | 0 | 0 | 0 | 0 | 0 |
| 183 | 0 | 0 | 0.91222 | 0.08778 | 0 | 0 | 0 | 0 | 0 |
| 184 | 0.00001 | 0.00002 | 0.910997 | 0.088974 | 0 | 0 | 0 | 0 | 0 |
| 185 | 0.00002 | 0.00009 | 0.909993 | 0.089897 | 0 | 0 | 0 | 0 | 0 |
| 186 | 0.00001 | 0.00001 | 0.908008 | 0.091972 | 0 | 0 | 0 | 0 | 0 |
| 187 | 0.00002 | 0.00004 | 0.914133 | 0.085807 | 0 | 0 | 0 | 0 | 0 |
| 188 | 0 | 0.00003 | 0.907701 | 0.092269 | 0 | 0 | 0 | 0 | 0 |
| 189 | 0 | 0.00005 | 0.907458 | 0.092492 | 0 | 0 | 0 | 0 | 0 |
| 190 | 0.00002 | 0.00005 | 0.908946 | 0.090984 | 0 | 0 | 0 | 0 | 0 |
| 191 | 0.00001 | 0.00006 | 0.908623 | 0.091308 | 0 | 0 | 0 | 0 | 0 |
| 192 | 0 | 0.00003 | 0.909063 | 0.090907 | 0 | 0 | 0 | 0 | 0 |
| 193 | 0.00001 | 0.00003 | 0.907304 | 0.092656 | 0 | 0 | 0 | 0 | 0 |
| 194 | 0.00001 | 0.000061 | 0.905303 | 0.094626 | 0 | 0 | 0 | 0 | 0 |
| 195 | 0.00004 | 0.00003 | 0.906211 | 0.09372 | 0 | 0 | 0 | 0 | 0 |
| 196 | 0.00001 | 0.00004 | 0.908675 | 0.091275 | 0 | 0 | 0 | 0 | 0 |
| 197 | 0.00003 | 0.00002 | 0.913338 | 0.086612 | 0 | 0 | 0 | 0 | 0 |
| 198 | 0.00001 | 0.00002 | 0.907992 | 0.091357 | 6E-04 | 0 | 0 | 0 | 0 |
| 199 | 0 | 0.00005 | 0.912809 | 0.087141 | 0 | 0 | 0 | 0 | 0 |
| 200 | 0.00001 | 0.00003 | 0.905357 | 0.094603 | 0 | 0 | 0 | 0 | 0 |
| 201 | 0.00001 | 0.00001 | 0.909993 | 0.089987 | 0 | 0 | 0 | 0 | 0 |
| 202 | 0 | 0.00005 | 0.911009 | 0.088941 | 0 | 0 | 0 | 0 | 0 |
| 203 | 0.00002 | 0.00001 | 0.910887 | 0.089083 | 0 | 0 | 0 | 0 | 0 |
| 204 | 0.00001 | 0.00005 | 0.910085 | 0.089855 | 0 | 0 | 0 | 0 | 0 |
| 205 | 0 | 0.00002 | 0.904596 | 0.095383 | 0 | 0 | 0 | 0 | 0 |
| 206 | 0.00001 | 0.00001 | 0.905866 | 0.094114 | 0 | 0 | 0 | 0 | 0 |
| 207 | 0 | 0.00004 | 0.911799 | 0.088161 | 0 | 0 | 0 | 0 | 0 |
| 208 | 0 | 0.00003 | 0.91232 | 0.087649 | 0 | 0 | 0 | 0 | 0 |
| 209 | 0.00001 | 0.00002 | 0.908457 | 0.091513 | 0 | 0 | 0 | 0 | 0 |
| 210 | 0.00002 | 0.00003 | 0.909945 | 0.090005 | 0 | 0 | 0 | 0 | 0 |
| 211 | 0 | 0 | 0.904997 | 0.095003 | 0 | 0 | 0 | 0 | 0 |
| 212 | 0.00001 | 0.00002 | 0.907732 | 0.092238 | 0 | 0 | 0 | 0 | 0 |
| 213 | 0.00001 | 0.00006 | 0.90918 | 0.090751 | 0 | 0 | 0 | 0 | 0 |
| 214 | 0 | 0.00003 | 0.909324 | 0.090646 | 0 | 0 | 0 | 0 | 0 |
| 215 | 0.00002 | 0.00003 | 0.908344 | 0.091606 | 0 | 0 | 0 | 0 | 0 |
| 216 | 0.00001 | 0.00004 | 0.912052 | 0.087898 | 0 | 0 | 0 | 0 | 0 |
| 217 | 0.00001 | 0.00003 | 0.908154 | 0.091806 | 0 | 0 | 0 | 0 | 0 |
| 218 | 0.00001 | 0.00002 | 0.91021 | 0.08976 | 0 | 0 | 0 | 0 | 0 |
| 219 | 0.00001 | 0 | 0.916335 | 0.083655 | 0 | 0 | 0 | 0 | 0 |
| 220 | 0.00001 | 0.000091 | 0.908318 | 0.091581 | 0 | 0 | 0 | 0 | 0 |
| 221 | 0.00003 | 0.00001 | 0.908505 | 0.091455 | 0 | 0 | 0 | 0 | 0 |
| 222 | 0.00001 | 0 | 0.907995 | 0.091995 | 0 | 0 | 0 | 0 | 0 |
| 223 | 0.00002 | 0.000069 | 0.907531 | 0.09238 | 0 | 0 | 0 | 0 | 0 |
| 224 | 0.00001 | 0.00003 | 0.907257 | 0.092703 | 0 | 0 | 0 | 0 | 0 |
| 225 | 0.00001 | 0 | 0.911524 | 0.088466 | 0 | 0 | 0 | 0 | 0 |
| 226 | 0.00001 | 0 | 0.907494 | 0.092496 | 0 | 0 | 0 | 0 | 0 |
| 227 | 0.00001 | 0.00003 | 0.909215 | 0.090745 | 0 | 0 | 0 | 0 | 0 |
| 228 | 0.00001 | 0.00004 | 0.913098 | 0.086852 | 0 | 0 | 0 | 0 | 0 |
| 229 | 0.00001 | 0.00009 | 0.908834 | 0.091066 | 0 | 0 | 0 | 0 | 0 |
| 230 | 0.00002 | 0.00002 | 0.908396 | 0.091564 | 0 | 0 | 0 | 0 | 0 |
| 231 | 0 | 0.00014 | 0.908193 | 0.091666 | 0 | 0 | 0 | 0 | 0 |
| 232 | 0.00002 | 0.00002 | 0.90919 | 0.09077 | 0 | 0 | 0 | 0 | 0 |
| 233 | 0.00002 | 0.00004 | 0.909752 | 0.090188 | 0 | 0 | 0 | 0 | 0 |
| 234 | 0.00002 | 0.00001 | 0.906788 | 0.093182 | 0 | 0 | 0 | 0 | 0 |
| 235 | 0.00001 | 0 | 0.913505 | 0.086485 | 0 | 0 | 0 | 0 | 0 |
| 236 | 0 | 0 | 0.905396 | 0.094604 | 0 | 0 | 0 | 0 | 0 |
| 237 | 0.00002 | 0.00002 | 0.908995 | 0.090965 | 0 | 0 | 0 | 0 | 0 |
| 238 | 0.00001 | 0.00006 | 0.905891 | 0.094039 | 0 | 0 | 0 | 0 | 0 |
| 239 | 0 | 0.00001 | 0.905394 | 0.094596 | 0 | 0 | 0 | 0 | 0 |
| 240 | 0 | 0 | 0.91003 | 0.08997 | 0 | 0 | 0 | 0 | 0 |
| 241 | 0.00001 | 0.00003 | 0.909693 | 0.090267 | 0 | 0 | 0 | 0 | 0 |
| 242 | 0.00001 | 0.00005 | 0.908667 | 0.091273 | 0 | 0 | 0 | 0 | 0 |
| 243 | 0.00001 | 0.00004 | 0.912787 | 0.087163 | 0 | 0 | 0 | 0 | 0 |
| 244 | 0 | 0.00002 | 0.907739 | 0.092241 | 0 | 0 | 0 | 0 | 0 |
| 245 | 0.00003 | 0.00005 | 0.906824 | 0.092837 | 3E-04 | 0 | 0 | 0 | 0 |
| 246 | 0.00001 | 0.00004 | 0.90508 | 0.09487 | 0 | 0 | 0 | 0 | 0 |
| 247 | 0 | 0.00003 | 0.907579 | 0.092391 | 0 | 0 | 0 | 0 | 0 |
| 248 | 0 | 0.00006 | 0.910016 | 0.089924 | 0 | 0 | 0 | 0 | 0 |
| 249 | 0.00003 | 0.00001 | 0.910858 | 0.089102 | 0 | 0 | 0 | 0 | 0 |
| 250 | 0.00001 | 0.00001 | 0.906762 | 0.093218 | 0 | 0 | 0 | 0 | 0 |
| 251 | 0.00002 | 0.00005 | 0.907295 | 0.092635 | 0 | 0 | 0 | 0 | 0 |
| 252 | 0 | 0.00002 | 0.910313 | 0.089667 | 0 | 0 | 0 | 0 | 0 |
| 253 | 0.00002 | 0.00002 | 0.910266 | 0.089694 | 0 | 0 | 0 | 0 | 0 |
| 254 | 0 | 0 | 0.907525 | 0.092475 | 0 | 0 | 0 | 0 | 0 |
| 255 | 0.00003 | 0.00001 | 0.908801 | 0.091159 | 0 | 0 | 0 | 0 | 0 |
| 256 | 0.00002 | 0.00004 | 0.907066 | 0.092874 | 0 | 0 | 0 | 0 | 0 |
| 257 | 0.00001 | 0.00004 | 0.908643 | 0.091307 | 0 | 0 | 0 | 0 | 0 |
| 258 | 0.00003 | 0.00004 | 0.905082 | 0.094849 | 0 | 0 | 0 | 0 | 0 |
| 259 | 0 | 0.00006 | 0.906298 | 0.093642 | 0 | 0 | 0 | 0 | 0 |
| 260 | 0.00004 | 0 | 0.911575 | 0.088384 | 0 | 0 | 0 | 0 | 0 |
| 261 | 0 | 0.00002 | 0.913104 | 0.086876 | 0 | 0 | 0 | 0 | 0 |
| 262 | 0.00001 | 0.00003 | 0.909702 | 0.090258 | 0 | 0 | 0 | 0 | 0 |
| 263 | 0 | 0.00004 | 0.908815 | 0.091145 | 0 | 0 | 0 | 0 | 0 |
| 264 | 0.00002 | 0.00001 | 0.90469 | 0.095281 | 0 | 0 | 0 | 0 | 0 |
| 265 | 0.00002 | 0.00003 | 0.907054 | 0.092896 | 0 | 0 | 0 | 0 | 0 |
| 266 | 0.00001 | 0.00004 | 0.912175 | 0.087775 | 0 | 0 | 0 | 0 | 0 |
| 267 | 0.00002 | 0 | 0.909217 | 0.090763 | 0 | 0 | 0 | 0 | 0 |
| 268 | 0 | 0.00003 | 0.911705 | 0.088265 | 0 | 0 | 0 | 0 | 0 |
| 269 | 0.00001 | 0 | 0.910832 | 0.089158 | 0 | 0 | 0 | 0 | 0 |
| 270 | 0.00001 | 0.00004 | 0.912164 | 0.087786 | 0 | 0 | 0 | 0 | 0 |
| 271 | 0.00002 | 0 | 0.907515 | 0.092465 | 0 | 0 | 0 | 0 | 0 |
| 272 | 0.00001 | 0.00001 | 0.908503 | 0.091478 | 0 | 0 | 0 | 0 | 0 |
| 273 | 0.00002 | 0 | 0.912375 | 0.087605 | 0 | 0 | 0 | 0 | 0 |
| 274 | 0.00001 | 0.00006 | 0.912115 | 0.087814 | 0 | 0 | 0 | 0 | 0 |
| 275 | 0.00001 | 0.00003 | 0.909629 | 0.090331 | 0 | 0 | 0 | 0 | 0 |
| 276 | 0.00001 | 0.00001 | 0.908114 | 0.091866 | 0 | 0 | 0 | 0 | 0 |
| 277 | 0.00001 | 0.00003 | 0.903349 | 0.096611 | 0 | 0 | 0 | 0 | 0 |
| 278 | 0.00001 | 0 | 0.909034 | 0.090956 | 0 | 0 | 0 | 0 | 0 |
| 279 | 0 | 0.00002 | 0.907204 | 0.092776 | 0 | 0 | 0 | 0 | 0 |
| 280 | 0.00001 | 0.00008 | 0.907571 | 0.092339 | 0 | 0 | 0 | 0 | 0 |
| 281 | 0.00001 | 0.00003 | 0.906142 | 0.093818 | 0 | 0 | 0 | 0 | 0 |
| 282 | 0.00002 | 0.00006 | 0.910244 | 0.089676 | 0 | 0 | 0 | 0 | 0 |
| 283 | 0 | 0.00005 | 0.909617 | 0.090333 | 0 | 0 | 0 | 0 | 0 |
| 284 | 0.00001 | 0.00003 | 0.913319 | 0.086641 | 0 | 0 | 0 | 0 | 0 |
| 285 | 0.00003 | 0.00002 | 0.906114 | 0.093837 | 0 | 0 | 0 | 0 | 0 |
| 286 | 0.00002 | 0.00002 | 0.91247 | 0.08749 | 0 | 0 | 0 | 0 | 0 |
| 287 | 0 | 0 | 0.911009 | 0.088991 | 0 | 0 | 0 | 0 | 0 |
| 288 | 0 | 0.00001 | 0.907538 | 0.092452 | 0 | 0 | 0 | 0 | 0 |
| 289 | 0.00001 | 0.00003 | 0.910252 | 0.089708 | 0 | 0 | 0 | 0 | 0 |
| 290 | 0.00001 | 0.00001 | 0.908029 | 0.09195 | 0 | 0 | 0 | 0 | 0 |
| 291 | 0 | 0.00001 | 0.907989 | 0.092001 | 0 | 0 | 0 | 0 | 0 |
| 292 | 0 | 0.00001 | 0.906788 | 0.093202 | 0 | 0 | 0 | 0 | 0 |
| 293 | 0.00003 | 0.00005 | 0.904072 | 0.095848 | 0 | 0 | 0 | 0 | 0 |
| 294 | 0.00001 | 0.00006 | 0.906912 | 0.093019 | 0 | 0 | 0 | 0 | 0 |
| 295 | 0.00001 | 0 | 0.912151 | 0.087839 | 0 | 0 | 0 | 0 | 0 |
| 296 | 0.00001 | 0 | 0.90987 | 0.09012 | 0 | 0 | 0 | 0 | 0 |
| 297 | 0.00001 | 0.00002 | 0.907788 | 0.092182 | 0 | 0 | 0 | 0 | 0 |
| 298 | 0 | 0.00005 | 0.908048 | 0.091902 | 0 | 0 | 0 | 0 | 0 |
| 299 | 0.00002 | 0 | 0.90408 | 0.0959 | 0 | 0 | 0 | 0 | 0 |
| 300 | 0.00002 | 0.00004 | 0.908235 | 0.091705 | 0 | 0 | 0 | 0 | 0 |
| 301 | 0.00001 | 0.00002 | 0.907697 | 0.092273 | 0 | 0 | 0 | 0 | 0 |
| 302 | 0.00001 | 0.00001 | 0.905392 | 0.094588 | 0 | 0 | 0 | 0 | 0 |
| 303 | 0.00001 | 0.00001 | 0.907559 | 0.092421 | 0 | 0 | 0 | 0 | 0 |
| 304 | 0 | 0.00003 | 0.90552 | 0.09445 | 0 | 0 | 0 | 0 | 0 |
| 305 | 0.00002 | 0.00001 | 0.908144 | 0.091826 | 0 | 0 | 0 | 0 | 0 |
| 306 | 0 | 0.00002 | 0.911123 | 0.088857 | 0 | 0 | 0 | 0 | 0 |
| 307 | 0 | 0.00004 | 0.90935 | 0.09061 | 0 | 0 | 0 | 0 | 0 |
| 308 | 0.00002 | 0.000069 | 0.913183 | 0.086728 | 0 | 0 | 0 | 0 | 0 |
| 309 | 0.00001 | 0.00001 | 0.909742 | 0.090238 | 0 | 0 | 0 | 0 | 0 |
| 310 | 0.00002 | 0.00001 | 0.908514 | 0.091456 | 0 | 0 | 0 | 0 | 0 |
| 311 | 0.00004 | 0.00005 | 0.909435 | 0.090475 | 0 | 0 | 0 | 0 | 0 |
| 312 | 0 | 0 | 0.904828 | 0.095172 | 0 | 0 | 0 | 0 | 0 |
| 313 | 0 | 0.00004 | 0.909677 | 0.090284 | 0 | 0 | 0 | 0 | 0 |
| 314 | 0.00003 | 0 | 0.909047 | 0.090923 | 0 | 0 | 0 | 0 | 0 |
| 315 | 0.00003 | 0.00004 | 0.907734 | 0.092196 | 0 | 0 | 0 | 0 | 0 |
| 316 | 0 | 0 | 0.90701 | 0.09299 | 0 | 0 | 0 | 0 | 0 |
| 317 | 0.00002 | 0.00007 | 0.909113 | 0.090797 | 0 | 0 | 0 | 0 | 0 |
| 318 | 0 | 0.00001 | 0.905317 | 0.094584 | 9E-05 | 0 | 0 | 0 | 0 |
| 319 | 0.00001 | 0 | 0.905624 | 0.094366 | 0 | 0 | 0 | 0 | 0 |
| 320 | 0.00001 | 0.00005 | 0.908447 | 0.091493 | 0 | 0 | 0 | 0 | 0 |
| 321 | 0.00002 | 0.00003 | 0.907442 | 0.092508 | 0 | 0 | 0 | 0 | 0 |
| 322 | 0 | 0.00005 | 0.908794 | 0.091156 | 0 | 0 | 0 | 0 | 0 |
| 323 | 0.00001 | 0.00001 | 0.910196 | 0.089784 | 0 | 0 | 0 | 0 | 0 |
| 324 | 0.00001 | 0 | 0.909259 | 0.090731 | 0 | 0 | 0 | 0 | 0 |
| 325 | 0.00002 | 0 | 0.910634 | 0.089346 | 0 | 0 | 0 | 0 | 0 |
| 326 | 0.00004 | 0.00005 | 0.911094 | 0.088817 | 0 | 0 | 0 | 0 | 0 |
| 327 | 0 | 0.00005 | 0.908605 | 0.091345 | 0 | 0 | 0 | 0 | 0 |
| 328 | 0.00001 | 0.00002 | 0.907025 | 0.092945 | 0 | 0 | 0 | 0 | 0 |
| 329 | 0 | 0.00006 | 0.906553 | 0.093387 | 0 | 0 | 0 | 0 | 0 |
| 330 | 0 | 0 | 0.908978 | 0.091022 | 0 | 0 | 0 | 0 | 0 |
| 331 | 0.00002 | 0.00003 | 0.908509 | 0.091442 | 0 | 0 | 0 | 0 | 0 |
| 332 | 0.00001 | 0.00002 | 0.908495 | 0.091475 | 0 | 0 | 0 | 0 | 0 |
| 333 | 0 | 0.00006 | 0.904398 | 0.095542 | 0 | 0 | 0 | 0 | 0 |
| 334 | 0 | 0.00002 | 0.909543 | 0.090437 | 0 | 0 | 0 | 0 | 0 |
| 335 | 0.00003 | 0 | 0.909027 | 0.090943 | 0 | 0 | 0 | 0 | 0 |
| 336 | 0.00004 | 0.00003 | 0.903078 | 0.096852 | 0 | 0 | 0 | 0 | 0 |
| 337 | 0.00001 | 0.00002 | 0.910884 | 0.089086 | 0 | 0 | 0 | 0 | 0 |
| 338 | 0.00001 | 0.00001 | 0.910273 | 0.089707 | 0 | 0 | 0 | 0 | 0 |
| 339 | 0.00002 | 0.00005 | 0.907869 | 0.092061 | 0 | 0 | 0 | 0 | 0 |
| 340 | 0.00001 | 0.00002 | 0.907405 | 0.092566 | 0 | 0 | 0 | 0 | 0 |
| 341 | 0.00001 | 0.00001 | 0.907232 | 0.092748 | 0 | 0 | 0 | 0 | 0 |
| 342 | 0.00001 | 0 | 0.906589 | 0.093401 | 0 | 0 | 0 | 0 | 0 |
| 343 | 0.00001 | 0.00005 | 0.907766 | 0.092174 | 0 | 0 | 0 | 0 | 0 |
| 344 | 0.00002 | 0.00001 | 0.908565 | 0.091405 | 0 | 0 | 0 | 0 | 0 |
| 345 | 0.00005 | 0.00001 | 0.912687 | 0.087253 | 0 | 0 | 0 | 0 | 0 |
| 346 | 0.00001 | 0.00002 | 0.913141 | 0.08683 | 0 | 0 | 0 | 0 | 0 |
| 347 | 0.00001 | 0.00003 | 0.907284 | 0.092676 | 0 | 0 | 0 | 0 | 0 |
| 348 | 0.00001 | 0.00004 | 0.907801 | 0.092149 | 0 | 0 | 0 | 0 | 0 |
| 349 | 0 | 0.00001 | 0.908533 | 0.091457 | 0 | 0 | 0 | 0 | 0 |
| 350 | 0.00001 | 0.00002 | 0.909503 | 0.090467 | 0 | 0 | 0 | 0 | 0 |
| 351 | 0.00001 | 0.00001 | 0.907607 | 0.092373 | 0 | 0 | 0 | 0 | 0 |
| 352 | 0 | 0 | 0.91222 | 0.08778 | 0 | 0 | 0 | 0 | 0 |
| 353 | 0.00002 | 0.00005 | 0.908639 | 0.09129 | 0 | 0 | 0 | 0 | 0 |
| 354 | 0.00002 | 0.00002 | 0.907249 | 0.092711 | 0 | 0 | 0 | 0 | 0 |
| 355 | 0.00001 | 0.00003 | 0.910837 | 0.089123 | 0 | 0 | 0 | 0 | 0 |
| 356 | 0.00002 | 0 | 0.910228 | 0.089752 | 0 | 0 | 0 | 0 | 0 |
| 357 | 0.00001 | 0.00004 | 0.903688 | 0.096262 | 0 | 0 | 0 | 0 | 0 |
| 358 | 0.00003 | 0.00003 | 0.908558 | 0.091381 | 0 | 0 | 0 | 0 | 0 |
| 359 | 0.00002 | 0.00003 | 0.908226 | 0.091724 | 0 | 0 | 0 | 0 | 0 |
| 360 | 0.00002 | 0.00003 | 0.908181 | 0.091769 | 0 | 0 | 0 | 0 | 0 |
| 361 | 0 | 0.00001 | 0.906724 | 0.093266 | 0 | 0 | 0 | 0 | 0 |
| 362 | 0 | 0.00001 | 0.91033 | 0.08966 | 0 | 0 | 0 | 0 | 0 |
| 363 | 0.00001 | 0.00006 | 0.910433 | 0.089497 | 0 | 0 | 0 | 0 | 0 |
| 364 | 0 | 0.00005 | 0.912803 | 0.087147 | 0 | 0 | 0 | 0 | 0 |
| 365 | 0.00001 | 0.00004 | 0.907886 | 0.092064 | 0 | 0 | 0 | 0 | 0 |
| 366 | 0 | 0 | 0.907908 | 0.092092 | 0 | 0 | 0 | 0 | 0 |
| 367 | 0 | 0.00003 | 0.906586 | 0.093384 | 0 | 0 | 0 | 0 | 0 |
| 368 | 0.00002 | 0.00005 | 0.908837 | 0.091093 | 0 | 0 | 0 | 0 | 0 |
| 369 | 0 | 0.00003 | 0.906882 | 0.093088 | 0 | 0 | 0 | 0 | 0 |
| 370 | 0.00001 | 0.00003 | 0.909008 | 0.090952 | 0 | 0 | 0 | 0 | 0 |
| 371 | 0.00001 | 0.00002 | 0.909747 | 0.089993 | 2E-04 | 0 | 0 | 0 | 0 |
| 372 | 0.00001 | 0.00006 | 0.904778 | 0.095152 | 0 | 0 | 0 | 0 | 0 |
| 373 | 0 | 0.00002 | 0.905019 | 0.094961 | 0 | 0 | 0 | 0 | 0 |
| 374 | 0.00001 | 0.00002 | 0.910974 | 0.088996 | 0 | 0 | 0 | 0 | 0 |
| 375 | 0.00001 | 0.00003 | 0.906136 | 0.093824 | 0 | 0 | 0 | 0 | 0 |
| 376 | 0 | 0.00007 | 0.905724 | 0.094207 | 0 | 0 | 0 | 0 | 0 |
| 377 | 0.00002 | 0.00002 | 0.90616 | 0.0938 | 0 | 0 | 0 | 0 | 0 |
| 378 | 0.00001 | 0.00003 | 0.909166 | 0.090793 | 0 | 0 | 0 | 0 | 0 |
| 379 | 0.00001 | 0.00001 | 0.911048 | 0.088932 | 0 | 0 | 0 | 0 | 0 |
| 380 | 0.00001 | 0.00003 | 0.903094 | 0.096866 | 0 | 0 | 0 | 0 | 0 |
| 381 | 0.00002 | 0.00001 | 0.910589 | 0.089381 | 0 | 0 | 0 | 0 | 0 |
| 382 | 0.00002 | 0.00003 | 0.90952 | 0.09043 | 0 | 0 | 0 | 0 | 0 |
| 383 | 0.00001 | 0.00004 | 0.908443 | 0.091507 | 0 | 0 | 0 | 0 | 0 |
| 384 | 0.00001 | 0.00004 | 0.903977 | 0.095973 | 0 | 0 | 0 | 0 | 0 |
| 385 | 0.00002 | 0.00002 | 0.910867 | 0.089093 | 0 | 0 | 0 | 0 | 0 |
| 386 | 0.00003 | 0.00001 | 0.907551 | 0.092409 | 0 | 0 | 0 | 0 | 0 |
| 387 | 0 | 0.00005 | 0.907008 | 0.092941 | 0 | 0 | 0 | 0 | 0 |
| 388 | 0.00002 | 0.00002 | 0.911213 | 0.088747 | 0 | 0 | 0 | 0 | 0 |
| 389 | 0.00002 | 0.000049 | 0.909565 | 0.090366 | 0 | 0 | 0 | 0 | 0 |
| 390 | 0.00001 | 0.00004 | 0.908963 | 0.090988 | 0 | 0 | 0 | 0 | 0 |
| 391 | 0.00001 | 0.00002 | 0.903492 | 0.096478 | 0 | 0 | 0 | 0 | 0 |
| 392 | 0.00001 | 0.00003 | 0.909471 | 0.090488 | 0 | 0 | 0 | 0 | 0 |
| 393 | 0 | 0.00001 | 0.905724 | 0.094267 | 0 | 0 | 0 | 0 | 0 |
| 394 | 0.00001 | 0.00002 | 0.91233 | 0.087639 | 0 | 0 | 0 | 0 | 0 |
| 395 | 0 | 0.00002 | 0.912639 | 0.087341 | 0 | 0 | 0 | 0 | 0 |
| 396 | 0 | 0.00002 | 0.908888 | 0.091092 | 0 | 0 | 0 | 0 | 0 |
| 397 | 0.00002 | 0.00003 | 0.908594 | 0.091356 | 0 | 0 | 0 | 0 | 0 |
| 398 | 0.00003 | 0.00006 | 0.911256 | 0.088654 | 0 | 0 | 0 | 0 | 0 |
| 399 | 0.00001 | 0.00002 | 0.908152 | 0.091818 | 0 | 0 | 0 | 0 | 0 |
| 400 | 0 | 0.00005 | 0.912647 | 0.087303 | 0 | 0 | 0 | 0 | 0 |
| 401 | 0 | 0.00005 | 0.907478 | 0.092473 | 0 | 0 | 0 | 0 | 0 |
| 402 | 0 | 0.00007 | 0.908355 | 0.091575 | 0 | 0 | 0 | 0 | 0 |
| 403 | 0 | 0.00007 | 0.908247 | 0.091683 | 0 | 0 | 0 | 0 | 0 |
| 404 | 0.00001 | 0.00002 | 0.908365 | 0.091605 | 0 | 0 | 0 | 0 | 0 |
| 405 | 0.00001 | 0.00001 | 0.910141 | 0.089839 | 0 | 0 | 0 | 0 | 0 |
| 406 | 0.00002 | 0.00005 | 0.908099 | 0.091831 | 0 | 0 | 0 | 0 | 0 |
| 407 | 0.00001 | 0.00003 | 0.905939 | 0.094021 | 0 | 0 | 0 | 0 | 0 |
| 408 | 0.00002 | 0.00004 | 0.904202 | 0.095738 | 0 | 0 | 0 | 0 | 0 |
| 409 | 0 | 0.00001 | 0.904483 | 0.095507 | 0 | 0 | 0 | 0 | 0 |
| 410 | 0 | 0.00002 | 0.904743 | 0.095237 | 0 | 0 | 0 | 0 | 0 |
| 411 | 0 | 0.00001 | 0.906821 | 0.093169 | 0 | 0 | 0 | 0 | 0 |
| 412 | 0.00002 | 0.00008 | 0.907093 | 0.092806 | 0 | 0 | 0 | 0 | 0 |
| 413 | 0 | 0.000061 | 0.905164 | 0.094775 | 0 | 0 | 0 | 0 | 0 |
| 414 | 0 | 0 | 0.908829 | 0.091171 | 0 | 0 | 0 | 0 | 0 |
| 415 | 0.00003 | 0.00001 | 0.906153 | 0.093807 | 0 | 0 | 0 | 0 | 0 |
| 416 | 0.00001 | 0.00007 | 0.90816 | 0.09176 | 0 | 0 | 0 | 0 | 0 |
| 417 | 0 | 0.00005 | 0.911501 | 0.088449 | 0 | 0 | 0 | 0 | 0 |
| 418 | 0 | 0 | 0.910194 | 0.089806 | 0 | 0 | 0 | 0 | 0 |
| 419 | 0.00001 | 0.00002 | 0.906243 | 0.093727 | 0 | 0 | 0 | 0 | 0 |
| 420 | 0.00002 | 0.00003 | 0.914092 | 0.085857 | 0 | 0 | 0 | 0 | 0 |
| 421 | 0.00001 | 0.00001 | 0.908387 | 0.091593 | 0 | 0 | 0 | 0 | 0 |
| 422 | 0.00002 | 0.00007 | 0.910182 | 0.089727 | 0 | 0 | 0 | 0 | 0 |
| 423 | 0.00003 | 0.00002 | 0.910986 | 0.088964 | 0 | 0 | 0 | 0 | 0 |
| 424 | 0 | 0.00003 | 0.911805 | 0.088165 | 0 | 0 | 0 | 0 | 0 |
| 425 | 0.00002 | 0.00005 | 0.90657 | 0.09336 | 0 | 0 | 0 | 0 | 0 |
| 426 | 0 | 0.00003 | 0.907281 | 0.092689 | 0 | 0 | 0 | 0 | 0 |
| 427 | 0.00004 | 0.00006 | 0.909196 | 0.090703 | 0 | 0 | 0 | 0 | 0 |
| 428 | 0 | 0.00002 | 0.9125 | 0.08748 | 0 | 0 | 0 | 0 | 0 |
| 429 | 0 | 0.00003 | 0.910347 | 0.089623 | 0 | 0 | 0 | 0 | 0 |
| 430 | 0.00002 | 0.00005 | 0.908603 | 0.091327 | 0 | 0 | 0 | 0 | 0 |
| 431 | 0 | 0.00002 | 0.910743 | 0.089237 | 0 | 0 | 0 | 0 | 0 |
| 432 | 0.00004 | 0.00005 | 0.904203 | 0.095708 | 0 | 0 | 0 | 0 | 0 |
| 433 | 0 | 0.00001 | 0.908371 | 0.091619 | 0 | 0 | 0 | 0 | 0 |
| 434 | 0.00003 | 0.00001 | 0.908928 | 0.091031 | 0 | 0 | 0 | 0 | 0 |
| 435 | 0.00002 | 0.00002 | 0.907317 | 0.092643 | 0 | 0 | 0 | 0 | 0 |
| 436 | 0.00001 | 0 | 0.899866 | 0.100124 | 0 | 0 | 0 | 0 | 0 |
| 437 | 0.00001 | 0.00002 | 0.908816 | 0.091154 | 0 | 0 | 0 | 0 | 0 |
| 438 | 0.00003 | 0.00004 | 0.908918 | 0.091012 | 0 | 0 | 0 | 0 | 0 |
| 439 | 0 | 0.00004 | 0.909508 | 0.090452 | 0 | 0 | 0 | 0 | 0 |
| 440 | 0.00002 | 0 | 0.90892 | 0.09106 | 0 | 0 | 0 | 0 | 0 |
| 441 | 0.00001 | 0.00006 | 0.906742 | 0.093188 | 0 | 0 | 0 | 0 | 0 |
| 442 | 0.00001 | 0.00001 | 0.908441 | 0.091539 | 0 | 0 | 0 | 0 | 0 |
| 443 | 0.00001 | 0.00002 | 0.911749 | 0.088221 | 0 | 0 | 0 | 0 | 0 |
| 444 | 0.00002 | 0.00001 | 0.906237 | 0.093733 | 0 | 0 | 0 | 0 | 0 |
| 445 | 0.00001 | 0 | 0.905914 | 0.094076 | 0 | 0 | 0 | 0 | 0 |
| 446 | 0.00001 | 0 | 0.909741 | 0.090249 | 0 | 0 | 0 | 0 | 0 |
| 447 | 0.00001 | 0.00002 | 0.91015 | 0.08982 | 0 | 0 | 0 | 0 | 0 |
| 448 | 0.00002 | 0.00006 | 0.907868 | 0.092053 | 0 | 0 | 0 | 0 | 0 |
| 449 | 0 | 0.00004 | 0.909407 | 0.090553 | 0 | 0 | 0 | 0 | 0 |
| 450 | 0.00001 | 0 | 0.908545 | 0.091445 | 0 | 0 | 0 | 0 | 0 |
| 451 | 0.00003 | 0.00003 | 0.914418 | 0.085522 | 0 | 0 | 0 | 0 | 0 |
| 452 | 0.00003 | 0.00001 | 0.9101 | 0.08986 | 0 | 0 | 0 | 0 | 0 |
| 453 | 0 | 0.00001 | 0.908311 | 0.09168 | 0 | 0 | 0 | 0 | 0 |
| 454 | 0.00001 | 0.00002 | 0.909347 | 0.090623 | 0 | 0 | 0 | 0 | 0 |
| 455 | 0 | 0.00001 | 0.915329 | 0.084661 | 0 | 0 | 0 | 0 | 0 |
| 456 | 0.00002 | 0.00002 | 0.906954 | 0.093006 | 0 | 0 | 0 | 0 | 0 |
| 457 | 0.00003 | 0.00002 | 0.909052 | 0.090898 | 0 | 0 | 0 | 0 | 0 |
| 458 | 0.00003 | 0.00002 | 0.909988 | 0.089962 | 0 | 0 | 0 | 0 | 0 |
| 459 | 0.00001 | 0.00002 | 0.903827 | 0.096143 | 0 | 0 | 0 | 0 | 0 |
| 460 | 0.00003 | 0.00005 | 0.903956 | 0.095965 | 0 | 0 | 0 | 0 | 0 |
| 461 | 0.00001 | 0 | 0.910314 | 0.089676 | 0 | 0 | 0 | 0 | 0 |
| 462 | 0.00001 | 0.00001 | 0.914044 | 0.085936 | 0 | 0 | 0 | 0 | 0 |
| 463 | 0.00002 | 0.00001 | 0.904926 | 0.095044 | 0 | 0 | 0 | 0 | 0 |
| 464 | 0 | 0 | 0.907509 | 0.091714 | 8E-04 | 0 | 0 | 0 | 0 |
| 465 | 0.00001 | 0.00004 | 0.910352 | 0.089599 | 0 | 0 | 0 | 0 | 0 |
| 466 | 0.00001 | 0.00002 | 0.905491 | 0.094479 | 0 | 0 | 0 | 0 | 0 |
| 467 | 0.00001 | 0.00002 | 0.904393 | 0.095577 | 0 | 0 | 0 | 0 | 0 |
| 468 | 0 | 0 | 0.911586 | 0.088414 | 0 | 0 | 0 | 0 | 0 |
| 469 | 0.00001 | 0.00003 | 0.906944 | 0.093016 | 0 | 0 | 0 | 0 | 0 |
| 470 | 0.00003 | 0.00003 | 0.909086 | 0.090854 | 0 | 0 | 0 | 0 | 0 |
| 471 | 0 | 0.00005 | 0.909304 | 0.090646 | 0 | 0 | 0 | 0 | 0 |
| 472 | 0 | 0 | 0.908174 | 0.091826 | 0 | 0 | 0 | 0 | 0 |
| 473 | 0.00001 | 0.00002 | 0.908851 | 0.091119 | 0 | 0 | 0 | 0 | 0 |
| 474 | 0.00001 | 0.00001 | 0.908765 | 0.091215 | 0 | 0 | 0 | 0 | 0 |
| 475 | 0.00001 | 0 | 0.90732 | 0.09267 | 0 | 0 | 0 | 0 | 0 |
| 476 | 0.00001 | 0.00004 | 0.901427 | 0.098522 | 0 | 0 | 0 | 0 | 0 |
| 477 | 0.00001 | 0.00002 | 0.909242 | 0.090728 | 0 | 0 | 0 | 0 | 0 |
| 478 | 0.00001 | 0.00004 | 0.906996 | 0.092954 | 0 | 0 | 0 | 0 | 0 |
| 479 | 0.00002 | 0.00001 | 0.904305 | 0.095665 | 0 | 0 | 0 | 0 | 0 |
| 480 | 0.00003 | 0.000059 | 0.905707 | 0.094204 | 0 | 0 | 0 | 0 | 0 |
| 481 | 0 | 0.00003 | 0.912826 | 0.087144 | 0 | 0 | 0 | 0 | 0 |
| 482 | 0 | 0.00001 | 0.90528 | 0.09471 | 0 | 0 | 0 | 0 | 0 |
| 483 | 0 | 0.00002 | 0.910922 | 0.089058 | 0 | 0 | 0 | 0 | 0 |
| 484 | 0 | 0.00001 | 0.904566 | 0.095424 | 0 | 0 | 0 | 0 | 0 |
| 485 | 0.00001 | 0.00004 | 0.913633 | 0.086317 | 0 | 0 | 0 | 0 | 0 |
| 486 | 0.00002 | 0.00005 | 0.908375 | 0.091554 | 0 | 0 | 0 | 0 | 0 |
| 487 | 0.00004 | 0.00002 | 0.908231 | 0.091709 | 0 | 0 | 0 | 0 | 0 |
| 488 | 0.00002 | 0.00006 | 0.907948 | 0.091972 | 0 | 0 | 0 | 0 | 0 |
| 489 | 0 | 0 | 0.908739 | 0.091261 | 0 | 0 | 0 | 0 | 0 |
| 490 | 0.00001 | 0.00004 | 0.911097 | 0.088853 | 0 | 0 | 0 | 0 | 0 |
| 491 | 0.00002 | 0.00001 | 0.908523 | 0.091447 | 0 | 0 | 0 | 0 | 0 |
| 492 | 0.00003 | 0.00001 | 0.906218 | 0.093742 | 0 | 0 | 0 | 0 | 0 |
| 493 | 0.00003 | 0.00002 | 0.906872 | 0.093078 | 0 | 0 | 0 | 0 | 0 |
| 494 | 0.00002 | 0.00001 | 0.906266 | 0.093704 | 0 | 0 | 0 | 0 | 0 |
| 495 | 0 | 0 | 0.908709 | 0.091291 | 0 | 0 | 0 | 0 | 0 |
| 496 | 0 | 0.00002 | 0.905769 | 0.094121 | 9E-05 | 0 | 0 | 0 | 0 |
| 497 | 0.00002 | 0.00005 | 0.908011 | 0.091919 | 0 | 0 | 0 | 0 | 0 |
| 498 | 0 | 0.000049 | 0.913146 | 0.086805 | 0 | 0 | 0 | 0 | 0 |
| 499 | 0.00001 | 0 | 0.906752 | 0.093238 | 0 | 0 | 0 | 0 | 0 |
| 500 | 0 | 0.00003 | 0.910633 | 0.089337 | 0 | 0 | 0 | 0 | 0 |
| 501 | 0.00001 | 0.00004 | 0.907905 | 0.092045 | 0 | 0 | 0 | 0 | 0 |
| 502 | 0 | 0.000059 | 0.908734 | 0.091207 | 0 | 0 | 0 | 0 | 0 |
| 503 | 0 | 0.00002 | 0.907614 | 0.092366 | 0 | 0 | 0 | 0 | 0 |
| 504 | 0.00001 | 0.00006 | 0.910461 | 0.089469 | 0 | 0 | 0 | 0 | 0 |
| 505 | 0.00001 | 0.00002 | 0.905309 | 0.094581 | 8E-05 | 0 | 0 | 0 | 0 |
| 506 | 0 | 0.00002 | 0.908357 | 0.091623 | 0 | 0 | 0 | 0 | 0 |
| 507 | 0 | 0.00003 | 0.906957 | 0.093013 | 0 | 0 | 0 | 0 | 0 |
| 508 | 0 | 0.00004 | 0.906335 | 0.093625 | 0 | 0 | 0 | 0 | 0 |
| 509 | 0.00003 | 0.00002 | 0.906495 | 0.093454 | 0 | 0 | 0 | 0 | 0 |
| 510 | 0.00001 | 0.000039 | 0.909304 | 0.090647 | 0 | 0 | 0 | 0 | 0 |
| 511 | 0.00001 | 0.00001 | 0.907408 | 0.092572 | 0 | 0 | 0 | 0 | 0 |
| 512 | 0.00001 | 0.00002 | 0.907528 | 0.092443 | 0 | 0 | 0 | 0 | 0 |
| 513 | 0.00003 | 0.00005 | 0.909957 | 0.089964 | 0 | 0 | 0 | 0 | 0 |
| 514 | 0.00002 | 0.00001 | 0.908153 | 0.091817 | 0 | 0 | 0 | 0 | 0 |
| 515 | 0 | 0.00001 | 0.91172 | 0.08827 | 0 | 0 | 0 | 0 | 0 |
| 516 | 0 | 0.00002 | 0.903861 | 0.096119 | 0 | 0 | 0 | 0 | 0 |
| 517 | 0.00003 | 0.00001 | 0.906172 | 0.093788 | 0 | 0 | 0 | 0 | 0 |
| 518 | 0.00001 | 0.000061 | 0.908623 | 0.091306 | 0 | 0 | 0 | 0 | 0 |
| 519 | 0 | 0.00003 | 0.907205 | 0.092765 | 0 | 0 | 0 | 0 | 0 |
| 520 | 0.00002 | 0.00001 | 0.910088 | 0.089882 | 0 | 0 | 0 | 0 | 0 |
| 521 | 0.00001 | 0 | 0.907736 | 0.092254 | 0 | 0 | 0 | 0 | 0 |
| 522 | 0 | 0.00005 | 0.910283 | 0.089667 | 0 | 0 | 0 | 0 | 0 |
| 523 | 0.00003 | 0.00003 | 0.905573 | 0.094367 | 0 | 0 | 0 | 0 | 0 |
| 524 | 0.00002 | 0.00001 | 0.906587 | 0.093383 | 0 | 0 | 0 | 0 | 0 |
| 525 | 0.00001 | 0.00004 | 0.905112 | 0.094838 | 0 | 0 | 0 | 0 | 0 |
| 526 | 0.00002 | 0.00004 | 0.907123 | 0.092817 | 0 | 0 | 0 | 0 | 0 |
| 527 | 0 | 0.00001 | 0.910014 | 0.089976 | 0 | 0 | 0 | 0 | 0 |
| 528 | 0 | 0.00003 | 0.904976 | 0.094994 | 0 | 0 | 0 | 0 | 0 |
| 529 | 0 | 0.00005 | 0.907371 | 0.092579 | 0 | 0 | 0 | 0 | 0 |
| 530 | 0 | 0.00003 | 0.904234 | 0.095736 | 0 | 0 | 0 | 0 | 0 |
| 531 | 0.00002 | 0.00002 | 0.908912 | 0.091048 | 0 | 0 | 0 | 0 | 0 |
| 532 | 0.00001 | 0.00002 | 0.914867 | 0.085104 | 0 | 0 | 0 | 0 | 0 |
| 533 | 0.00003 | 0.00005 | 0.907592 | 0.092328 | 0 | 0 | 0 | 0 | 0 |
| 534 | 0 | 0.00006 | 0.906357 | 0.093583 | 0 | 0 | 0 | 0 | 0 |
| 535 | 0.00001 | 0.00004 | 0.912125 | 0.087825 | 0 | 0 | 0 | 0 | 0 |
| 536 | 0.00002 | 0.00001 | 0.91158 | 0.08839 | 0 | 0 | 0 | 0 | 0 |
| 537 | 0.00002 | 0 | 0.906884 | 0.093096 | 0 | 0 | 0 | 0 | 0 |
| 538 | 0.00001 | 0.00005 | 0.904919 | 0.095022 | 0 | 0 | 0 | 0 | 0 |
| 539 | 0.00003 | 0.00002 | 0.903788 | 0.096162 | 0 | 0 | 0 | 0 | 0 |
| 540 | 0.00004 | 0.00005 | 0.906641 | 0.09327 | 0 | 0 | 0 | 0 | 0 |
| 541 | 0.00001 | 0.00002 | 0.909231 | 0.090738 | 0 | 0 | 0 | 0 | 0 |
| 542 | 0 | 0.00004 | 0.907387 | 0.092573 | 0 | 0 | 0 | 0 | 0 |
| 543 | 0 | 0.00003 | 0.911593 | 0.088377 | 0 | 0 | 0 | 0 | 0 |
| 544 | 0.00001 | 0 | 0.912303 | 0.087687 | 0 | 0 | 0 | 0 | 0 |
| 545 | 0.00001 | 0.00005 | 0.902541 | 0.097399 | 0 | 0 | 0 | 0 | 0 |
| 546 | 0 | 0.00005 | 0.911344 | 0.088606 | 0 | 0 | 0 | 0 | 0 |
| 547 | 0 | 0.00002 | 0.907407 | 0.092573 | 0 | 0 | 0 | 0 | 0 |
| 548 | 0.00002 | 0.00003 | 0.911678 | 0.088272 | 0 | 0 | 0 | 0 | 0 |
| 549 | 0.00003 | 0.00001 | 0.905325 | 0.094635 | 0 | 0 | 0 | 0 | 0 |
| 550 | 0 | 0.00002 | 0.909004 | 0.090976 | 0 | 0 | 0 | 0 | 0 |
| 551 | 0 | 0.00004 | 0.907676 | 0.092284 | 0 | 0 | 0 | 0 | 0 |
| 552 | 0.00001 | 0.00003 | 0.907458 | 0.092502 | 0 | 0 | 0 | 0 | 0 |
| 553 | 0.00001 | 0.00004 | 0.907817 | 0.092133 | 0 | 0 | 0 | 0 | 0 |
| 554 | 0.00002 | 0.00005 | 0.914468 | 0.085462 | 0 | 0 | 0 | 0 | 0 |
| 555 | 0.00003 | 0.00001 | 0.906343 | 0.093617 | 0 | 0 | 0 | 0 | 0 |
| 556 | 0.00001 | 0.00001 | 0.912765 | 0.087215 | 0 | 0 | 0 | 0 | 0 |
| 557 | 0 | 0.00004 | 0.90745 | 0.09251 | 0 | 0 | 0 | 0 | 0 |
| 558 | 0.00001 | 0.00002 | 0.909338 | 0.090632 | 0 | 0 | 0 | 0 | 0 |
| 559 | 0.00003 | 0.00001 | 0.910286 | 0.089674 | 0 | 0 | 0 | 0 | 0 |
| 560 | 0 | 0.00001 | 0.908988 | 0.091002 | 0 | 0 | 0 | 0 | 0 |
| 561 | 0.00001 | 0.00001 | 0.906109 | 0.093871 | 0 | 0 | 0 | 0 | 0 |
| 562 | 0.00002 | 0 | 0.904266 | 0.095714 | 0 | 0 | 0 | 0 | 0 |
| 563 | 0.00001 | 0.00006 | 0.903703 | 0.096227 | 0 | 0 | 0 | 0 | 0 |
| 564 | 0.00001 | 0.00005 | 0.911205 | 0.088736 | 0 | 0 | 0 | 0 | 0 |
| 565 | 0.00002 | 0.00003 | 0.90365 | 0.0963 | 0 | 0 | 0 | 0 | 0 |
| 566 | 0.00001 | 0.00007 | 0.909475 | 0.090445 | 0 | 0 | 0 | 0 | 0 |
| 567 | 0 | 0.00001 | 0.907663 | 0.092327 | 0 | 0 | 0 | 0 | 0 |
| 568 | 0.00001 | 0 | 0.905819 | 0.094171 | 0 | 0 | 0 | 0 | 0 |
| 569 | 0 | 0.00004 | 0.911774 | 0.088186 | 0 | 0 | 0 | 0 | 0 |
| 570 | 0.00001 | 0.00005 | 0.904313 | 0.095626 | 0 | 0 | 0 | 0 | 0 |
| 571 | 0.00001 | 0.00002 | 0.908127 | 0.091843 | 0 | 0 | 0 | 0 | 0 |
| 572 | 0 | 0.00003 | 0.909494 | 0.090477 | 0 | 0 | 0 | 0 | 0 |
| 573 | 0 | 0.00006 | 0.909411 | 0.090529 | 0 | 0 | 0 | 0 | 0 |
| 574 | 0.00001 | 0 | 0.908499 | 0.091491 | 0 | 0 | 0 | 0 | 0 |
| 575 | 0 | 0.00001 | 0.908528 | 0.091462 | 0 | 0 | 0 | 0 | 0 |
| 576 | 0.00001 | 0.00001 | 0.915068 | 0.084912 | 0 | 0 | 0 | 0 | 0 |
| 577 | 0.00001 | 0 | 0.905226 | 0.094764 | 0 | 0 | 0 | 0 | 0 |
| 578 | 0.00001 | 0.00002 | 0.908821 | 0.091149 | 0 | 0 | 0 | 0 | 0 |
| 579 | 0.00001 | 0.00004 | 0.906313 | 0.093638 | 0 | 0 | 0 | 0 | 0 |
| 580 | 0.00001 | 0.00003 | 0.910261 | 0.089699 | 0 | 0 | 0 | 0 | 0 |
| 581 | 0.00002 | 0.00003 | 0.905068 | 0.094882 | 0 | 0 | 0 | 0 | 0 |
| 582 | 0.00002 | 0.00002 | 0.907753 | 0.092207 | 0 | 0 | 0 | 0 | 0 |
| 583 | 0.00003 | 0.00004 | 0.90496 | 0.094969 | 0 | 0 | 0 | 0 | 0 |
| 584 | 0.00002 | 0.00001 | 0.904006 | 0.095964 | 0 | 0 | 0 | 0 | 0 |
| 585 | 0.00002 | 0.00002 | 0.90557 | 0.09439 | 0 | 0 | 0 | 0 | 0 |
| 586 | 0.00002 | 0.00005 | 0.909773 | 0.090157 | 0 | 0 | 0 | 0 | 0 |
| 587 | 0 | 0 | 0.909884 | 0.090116 | 0 | 0 | 0 | 0 | 0 |
| 588 | 0.00001 | 0.00003 | 0.912566 | 0.087394 | 0 | 0 | 0 | 0 | 0 |
| 589 | 0 | 0.00006 | 0.908215 | 0.091726 | 0 | 0 | 0 | 0 | 0 |
| 590 | 0 | 0.00001 | 0.904741 | 0.09525 | 0 | 0 | 0 | 0 | 0 |
| 591 | 0 | 0.00004 | 0.906359 | 0.093601 | 0 | 0 | 0 | 0 | 0 |
| 592 | 0.00003 | 0.00004 | 0.908039 | 0.09189 | 0 | 0 | 0 | 0 | 0 |
| 593 | 0.00001 | 0.00002 | 0.911603 | 0.088368 | 0 | 0 | 0 | 0 | 0 |
| 594 | 0.00002 | 0 | 0.906349 | 0.093631 | 0 | 0 | 0 | 0 | 0 |
| 595 | 0.00001 | 0.000099 | 0.90581 | 0.09408 | 0 | 0 | 0 | 0 | 0 |
| 596 | 0.00001 | 0.00002 | 0.910409 | 0.089561 | 0 | 0 | 0 | 0 | 0 |
| 597 | 0.00001 | 0.00001 | 0.907674 | 0.092306 | 0 | 0 | 0 | 0 | 0 |
| 598 | 0.00001 | 0.00004 | 0.907459 | 0.092491 | 0 | 0 | 0 | 0 | 0 |
| 599 | 0.00001 | 0.00004 | 0.908146 | 0.091804 | 0 | 0 | 0 | 0 | 0 |
| 600 | 0.00001 | 0.00002 | 0.906039 | 0.093931 | 0 | 0 | 0 | 0 | 0 |
| 601 | 0.00002 | 0.00002 | 0.910006 | 0.089954 | 0 | 0 | 0 | 0 | 0 |
| 602 | 0.00001 | 0.00005 | 0.910863 | 0.089077 | 0 | 0 | 0 | 0 | 0 |
| 603 | 0.00001 | 0.00003 | 0.909889 | 0.090071 | 0 | 0 | 0 | 0 | 0 |
| 604 | 0 | 0.00001 | 0.909222 | 0.090768 | 0 | 0 | 0 | 0 | 0 |
| 605 | 0.00002 | 0.00001 | 0.90937 | 0.0906 | 0 | 0 | 0 | 0 | 0 |
| 606 | 0 | 0.00003 | 0.912204 | 0.087766 | 0 | 0 | 0 | 0 | 0 |
| 607 | 0 | 0.00002 | 0.912248 | 0.087732 | 0 | 0 | 0 | 0 | 0 |
| 608 | 0 | 0.00005 | 0.905969 | 0.093982 | 0 | 0 | 0 | 0 | 0 |
| 609 | 0.00002 | 0.00004 | 0.910625 | 0.089315 | 0 | 0 | 0 | 0 | 0 |
| 610 | 0.00001 | 0.00002 | 0.907225 | 0.092745 | 0 | 0 | 0 | 0 | 0 |
| 611 | 0.00003 | 0.00002 | 0.909475 | 0.090475 | 0 | 0 | 0 | 0 | 0 |
| 612 | 0.00001 | 0.00002 | 0.906357 | 0.093613 | 0 | 0 | 0 | 0 | 0 |
| 613 | 0 | 0.00004 | 0.903396 | 0.096564 | 0 | 0 | 0 | 0 | 0 |
| 614 | 0.00001 | 0.00003 | 0.911952 | 0.088007 | 0 | 0 | 0 | 0 | 0 |
| 615 | 0.00001 | 0.00002 | 0.907452 | 0.092517 | 0 | 0 | 0 | 0 | 0 |
| 616 | 0.00002 | 0.00008 | 0.907575 | 0.092325 | 0 | 0 | 0 | 0 | 0 |
| 617 | 0.00001 | 0.00002 | 0.909099 | 0.090871 | 0 | 0 | 0 | 0 | 0 |
| 618 | 0.00001 | 0.00001 | 0.904762 | 0.095218 | 0 | 0 | 0 | 0 | 0 |
| 619 | 0.00001 | 0.00001 | 0.911803 | 0.088177 | 0 | 0 | 0 | 0 | 0 |
| 620 | 0.00002 | 0.00004 | 0.907404 | 0.092536 | 0 | 0 | 0 | 0 | 0 |
| 621 | 0.00001 | 0 | 0.910162 | 0.089828 | 0 | 0 | 0 | 0 | 0 |
| 622 | 0.00001 | 0.00004 | 0.904684 | 0.095266 | 0 | 0 | 0 | 0 | 0 |
| 623 | 0.00001 | 0.00001 | 0.9086 | 0.09138 | 0 | 0 | 0 | 0 | 0 |
| 624 | 0 | 0.00005 | 0.908189 | 0.091761 | 0 | 0 | 0 | 0 | 0 |
| 625 | 0.00002 | 0.00008 | 0.906834 | 0.092797 | 3E-04 | 0 | 0 | 0 | 0 |
| 626 | 0.00001 | 0.00004 | 0.904784 | 0.095165 | 0 | 0 | 0 | 0 | 0 |
| 627 | 0 | 0.00002 | 0.911205 | 0.088775 | 0 | 0 | 0 | 0 | 0 |
| 628 | 0.00004 | 0.000099 | 0.908164 | 0.091697 | 0 | 0 | 0 | 0 | 0 |
| 629 | 0.00001 | 0.00003 | 0.9086 | 0.09136 | 0 | 0 | 0 | 0 | 0 |
| 630 | 0.00002 | 0 | 0.908715 | 0.091265 | 0 | 0 | 0 | 0 | 0 |
| 631 | 0.00001 | 0.00002 | 0.907147 | 0.092823 | 0 | 0 | 0 | 0 | 0 |
| 632 | 0.00001 | 0 | 0.909649 | 0.090341 | 0 | 0 | 0 | 0 | 0 |
| 633 | 0.00001 | 0.00003 | 0.908922 | 0.091038 | 0 | 0 | 0 | 0 | 0 |
| 634 | 0.00002 | 0 | 0.905143 | 0.094837 | 0 | 0 | 0 | 0 | 0 |
| 635 | 0.00001 | 0.00004 | 0.912402 | 0.087548 | 0 | 0 | 0 | 0 | 0 |
| 636 | 0.00001 | 0.00003 | 0.906203 | 0.093757 | 0 | 0 | 0 | 0 | 0 |
| 637 | 0 | 0 | 0.907135 | 0.092865 | 0 | 0 | 0 | 0 | 0 |
| 638 | 0.00003 | 0.00004 | 0.909278 | 0.090652 | 0 | 0 | 0 | 0 | 0 |
| 639 | 0.00002 | 0.00001 | 0.904694 | 0.095277 | 0 | 0 | 0 | 0 | 0 |
| 640 | 0.00001 | 0.00005 | 0.903199 | 0.096742 | 0 | 0 | 0 | 0 | 0 |
| 641 | 0.00001 | 0.00005 | 0.909211 | 0.090729 | 0 | 0 | 0 | 0 | 0 |
| 642 | 0.00001 | 0.00003 | 0.909981 | 0.089979 | 0 | 0 | 0 | 0 | 0 |
| 643 | 0 | 0.00002 | 0.906947 | 0.093033 | 0 | 0 | 0 | 0 | 0 |
| 644 | 0.00002 | 0.000061 | 0.911144 | 0.088775 | 0 | 0 | 0 | 0 | 0 |
| 645 | 0.00002 | 0.00003 | 0.908909 | 0.091041 | 0 | 0 | 0 | 0 | 0 |
| 646 | 0.00003 | 0.00002 | 0.906628 | 0.093322 | 0 | 0 | 0 | 0 | 0 |
| 647 | 0.00001 | 0.00003 | 0.907986 | 0.091975 | 0 | 0 | 0 | 0 | 0 |
| 648 | 0 | 0.00002 | 0.909101 | 0.090879 | 0 | 0 | 0 | 0 | 0 |
| 649 | 0 | 0 | 0.908352 | 0.091648 | 0 | 0 | 0 | 0 | 0 |
| 650 | 0.00004 | 0.00001 | 0.907712 | 0.092238 | 0 | 0 | 0 | 0 | 0 |
| 651 | 0 | 0.00003 | 0.907651 | 0.092319 | 0 | 0 | 0 | 0 | 0 |
| 652 | 0.00001 | 0.00005 | 0.909148 | 0.090792 | 0 | 0 | 0 | 0 | 0 |
| 653 | 0.00001 | 0.00003 | 0.911172 | 0.088788 | 0 | 0 | 0 | 0 | 0 |
| 654 | 0.00001 | 0.00002 | 0.908233 | 0.091737 | 0 | 0 | 0 | 0 | 0 |
| 655 | 0.00001 | 0.00003 | 0.906141 | 0.093819 | 0 | 0 | 0 | 0 | 0 |
| 656 | 0 | 0 | 0.915352 | 0.084648 | 0 | 0 | 0 | 0 | 0 |
| 657 | 0.00001 | 0.00002 | 0.908781 | 0.091189 | 0 | 0 | 0 | 0 | 0 |
| 658 | 0 | 0.00004 | 0.907231 | 0.092729 | 0 | 0 | 0 | 0 | 0 |
| 659 | 0 | 0.00003 | 0.906489 | 0.093481 | 0 | 0 | 0 | 0 | 0 |
| 660 | 0.00001 | 0.00003 | 0.912865 | 0.087095 | 0 | 0 | 0 | 0 | 0 |
| 661 | 0 | 0.00004 | 0.909527 | 0.090433 | 0 | 0 | 0 | 0 | 0 |
| 662 | 0.00001 | 0.00003 | 0.907049 | 0.092911 | 0 | 0 | 0 | 0 | 0 |
| 663 | 0.00001 | 0 | 0.908965 | 0.091025 | 0 | 0 | 0 | 0 | 0 |
| 664 | 0.00001 | 0.00006 | 0.912999 | 0.086931 | 0 | 0 | 0 | 0 | 0 |
| 665 | 0.00001 | 0.00001 | 0.904905 | 0.095075 | 0 | 0 | 0 | 0 | 0 |
| 666 | 0.00002 | 0.00001 | 0.908842 | 0.091129 | 0 | 0 | 0 | 0 | 0 |
| 667 | 0.00003 | 0.00001 | 0.905296 | 0.094664 | 0 | 0 | 0 | 0 | 0 |
| 668 | 0 | 0.00001 | 0.904549 | 0.095441 | 0 | 0 | 0 | 0 | 0 |
| 669 | 0.00001 | 0.00002 | 0.905049 | 0.094921 | 0 | 0 | 0 | 0 | 0 |
| 670 | 0 | 0.00003 | 0.907805 | 0.092165 | 0 | 0 | 0 | 0 | 0 |
| 671 | 0.00001 | 0.00002 | 0.909042 | 0.090928 | 0 | 0 | 0 | 0 | 0 |
| 672 | 0.00001 | 0.00002 | 0.909365 | 0.090605 | 0 | 0 | 0 | 0 | 0 |
| 673 | 0.00001 | 0.00003 | 0.907023 | 0.092937 | 0 | 0 | 0 | 0 | 0 |
| 674 | 0.00002 | 0.00001 | 0.907796 | 0.092174 | 0 | 0 | 0 | 0 | 0 |
| 675 | 0.00002 | 0.00003 | 0.910402 | 0.089548 | 0 | 0 | 0 | 0 | 0 |
| 676 | 0.00003 | 0.00005 | 0.911115 | 0.088805 | 0 | 0 | 0 | 0 | 0 |
| 677 | 0.00001 | 0.00003 | 0.90861 | 0.09135 | 0 | 0 | 0 | 0 | 0 |
| 678 | 0 | 0 | 0.906519 | 0.093481 | 0 | 0 | 0 | 0 | 0 |
| 679 | 0 | 0 | 0.910406 | 0.089594 | 0 | 0 | 0 | 0 | 0 |
| 680 | 0.00004 | 0.00004 | 0.907 | 0.09292 | 0 | 0 | 0 | 0 | 0 |
| 681 | 0 | 0 | 0.905084 | 0.094916 | 0 | 0 | 0 | 0 | 0 |
| 682 | 0.00001 | 0.00001 | 0.907052 | 0.092928 | 0 | 0 | 0 | 0 | 0 |
| 683 | 0 | 0.00007 | 0.910218 | 0.089712 | 0 | 0 | 0 | 0 | 0 |
| 684 | 0.00002 | 0.00004 | 0.908489 | 0.091451 | 0 | 0 | 0 | 0 | 0 |
| 685 | 0.00002 | 0.00002 | 0.910189 | 0.089771 | 0 | 0 | 0 | 0 | 0 |
| 686 | 0.00002 | 0.00002 | 0.903066 | 0.096894 | 0 | 0 | 0 | 0 | 0 |
| 687 | 0 | 0.00002 | 0.907856 | 0.092124 | 0 | 0 | 0 | 0 | 0 |
| 688 | 0.00002 | 0.00003 | 0.908691 | 0.09126 | 0 | 0 | 0 | 0 | 0 |
| 689 | 0.00004 | 0.00002 | 0.904901 | 0.095039 | 0 | 0 | 0 | 0 | 0 |
| 690 | 0.00002 | 0.00002 | 0.907876 | 0.092084 | 0 | 0 | 0 | 0 | 0 |
| 691 | 0 | 0.00004 | 0.909989 | 0.089971 | 0 | 0 | 0 | 0 | 0 |
| 692 | 0.00001 | 0.00005 | 0.90759 | 0.09235 | 0 | 0 | 0 | 0 | 0 |
| 693 | 0.00001 | 0.00001 | 0.905758 | 0.094222 | 0 | 0 | 0 | 0 | 0 |
| 694 | 0 | 0.00006 | 0.910366 | 0.089574 | 0 | 0 | 0 | 0 | 0 |
| 695 | 0.00002 | 0.00002 | 0.909147 | 0.090813 | 0 | 0 | 0 | 0 | 0 |
| 696 | 0.00001 | 0.00004 | 0.912565 | 0.087385 | 0 | 0 | 0 | 0 | 0 |
| 697 | 0.00001 | 0 | 0.910295 | 0.089695 | 0 | 0 | 0 | 0 | 0 |
| 698 | 0.00003 | 0.00004 | 0.907819 | 0.092112 | 0 | 0 | 0 | 0 | 0 |
| 699 | 0.00003 | 0.00003 | 0.90906 | 0.09088 | 0 | 0 | 0 | 0 | 0 |
| 700 | 0.00002 | 0.00002 | 0.906407 | 0.093553 | 0 | 0 | 0 | 0 | 0 |
| 701 | 0.00001 | 0.00004 | 0.914734 | 0.085216 | 0 | 0 | 0 | 0 | 0 |
| 702 | 0.00002 | 0.00006 | 0.912175 | 0.087746 | 0 | 0 | 0 | 0 | 0 |
| 703 | 0.00003 | 0.00001 | 0.906363 | 0.093597 | 0 | 0 | 0 | 0 | 0 |
| 704 | 0 | 0.00004 | 0.908638 | 0.091322 | 0 | 0 | 0 | 0 | 0 |
| 705 | 0.00002 | 0.00003 | 0.90778 | 0.092171 | 0 | 0 | 0 | 0 | 0 |
| 706 | 0 | 0.00003 | 0.908189 | 0.091781 | 0 | 0 | 0 | 0 | 0 |
| 707 | 0 | 0.00002 | 0.904752 | 0.095228 | 0 | 0 | 0 | 0 | 0 |
| 708 | 0.00003 | 0.00004 | 0.907889 | 0.092041 | 0 | 0 | 0 | 0 | 0 |
| 709 | 0.00002 | 0.00005 | 0.911985 | 0.087945 | 0 | 0 | 0 | 0 | 0 |
| 710 | 0.00001 | 0.00006 | 0.908738 | 0.091193 | 0 | 0 | 0 | 0 | 0 |
| 711 | 0 | 0.00001 | 0.910089 | 0.089901 | 0 | 0 | 0 | 0 | 0 |
| 712 | 0.00002 | 0 | 0.912998 | 0.086982 | 0 | 0 | 0 | 0 | 0 |
| 713 | 0.00002 | 0 | 0.906302 | 0.093678 | 0 | 0 | 0 | 0 | 0 |
| 714 | 0 | 0.00001 | 0.906596 | 0.093394 | 0 | 0 | 0 | 0 | 0 |
| 715 | 0 | 0.00002 | 0.905066 | 0.094914 | 0 | 0 | 0 | 0 | 0 |
| 716 | 0.00001 | 0.00004 | 0.90455 | 0.0954 | 0 | 0 | 0 | 0 | 0 |
| 717 | 0 | 0.00002 | 0.907696 | 0.092284 | 0 | 0 | 0 | 0 | 0 |
| 718 | 0.00001 | 0.00003 | 0.908042 | 0.091918 | 0 | 0 | 0 | 0 | 0 |
| 719 | 0 | 0.00002 | 0.911563 | 0.088417 | 0 | 0 | 0 | 0 | 0 |
| 720 | 0.00001 | 0.00003 | 0.907363 | 0.092597 | 0 | 0 | 0 | 0 | 0 |
| 721 | 0.00002 | 0 | 0.906332 | 0.093648 | 0 | 0 | 0 | 0 | 0 |
| 722 | 0.00002 | 0.00002 | 0.910789 | 0.08917 | 0 | 0 | 0 | 0 | 0 |
| 723 | 0.00003 | 0.00004 | 0.905217 | 0.094712 | 0 | 0 | 0 | 0 | 0 |
| 724 | 0 | 0.00002 | 0.909488 | 0.090492 | 0 | 0 | 0 | 0 | 0 |
| 725 | 0.00002 | 0.00001 | 0.908387 | 0.091584 | 0 | 0 | 0 | 0 | 0 |
| 726 | 0 | 0.00004 | 0.908273 | 0.091686 | 0 | 0 | 0 | 0 | 0 |
| 727 | 0.00001 | 0.00005 | 0.906818 | 0.093121 | 0 | 0 | 0 | 0 | 0 |
| 728 | 0 | 0.00011 | 0.90805 | 0.09184 | 0 | 0 | 0 | 0 | 0 |
| 729 | 0.00001 | 0.00002 | 0.90657 | 0.0934 | 0 | 0 | 0 | 0 | 0 |
| 730 | 0.00001 | 0.00003 | 0.90697 | 0.092991 | 0 | 0 | 0 | 0 | 0 |
| 731 | 0.00003 | 0 | 0.911082 | 0.088888 | 0 | 0 | 0 | 0 | 0 |
| 732 | 0.00001 | 0.00004 | 0.911126 | 0.088824 | 0 | 0 | 0 | 0 | 0 |
| 733 | 0.00002 | 0.00001 | 0.906552 | 0.093418 | 0 | 0 | 0 | 0 | 0 |
| 734 | 0.00001 | 0.00002 | 0.90982 | 0.09015 | 0 | 0 | 0 | 0 | 0 |
| 735 | 0.00003 | 0.00005 | 0.910875 | 0.089045 | 0 | 0 | 0 | 0 | 0 |
| 736 | 0 | 0.00001 | 0.91205 | 0.08794 | 0 | 0 | 0 | 0 | 0 |
| 737 | 0.00001 | 0.00005 | 0.907908 | 0.092032 | 0 | 0 | 0 | 0 | 0 |
| 738 | 0.00001 | 0.00003 | 0.905049 | 0.094911 | 0 | 0 | 0 | 0 | 0 |
| 739 | 0.00001 | 0.00005 | 0.900103 | 0.099836 | 0 | 0 | 0 | 0 | 0 |
| 740 | 0.00001 | 0.00002 | 0.910984 | 0.088986 | 0 | 0 | 0 | 0 | 0 |
| 741 | 0.00002 | 0.00003 | 0.907667 | 0.092283 | 0 | 0 | 0 | 0 | 0 |
| 742 | 0.00001 | 0.00001 | 0.908932 | 0.091048 | 0 | 0 | 0 | 0 | 0 |
| 743 | 0 | 0.00003 | 0.909751 | 0.090219 | 0 | 0 | 0 | 0 | 0 |
| 744 | 0.00003 | 0.00003 | 0.910547 | 0.088846 | 5E-04 | 0 | 0 | 0 | 0 |
| 745 | 0.00003 | 0.00005 | 0.910994 | 0.088927 | 0 | 0 | 0 | 0 | 0 |
| 746 | 0.00003 | 0.00004 | 0.901741 | 0.098189 | 0 | 0 | 0 | 0 | 0 |
| 747 | 0 | 0.00002 | 0.907058 | 0.092922 | 0 | 0 | 0 | 0 | 0 |
| 748 | 0.00001 | 0.00002 | 0.910183 | 0.089787 | 0 | 0 | 0 | 0 | 0 |
| 749 | 0.00001 | 0.00006 | 0.914016 | 0.085914 | 0 | 0 | 0 | 0 | 0 |
| 750 | 0.00001 | 0 | 0.910706 | 0.089284 | 0 | 0 | 0 | 0 | 0 |
| 751 | 0.00001 | 0.00003 | 0.910556 | 0.089404 | 0 | 0 | 0 | 0 | 0 |
| 752 | 0 | 0 | 0.91146 | 0.08854 | 0 | 0 | 0 | 0 | 0 |
| 753 | 0 | 0 | 0.909832 | 0.090168 | 0 | 0 | 0 | 0 | 0 |
| 754 | 0.00001 | 0.00007 | 0.906653 | 0.093268 | 0 | 0 | 0 | 0 | 0 |
| 755 | 0.00002 | 0.00002 | 0.906708 | 0.093252 | 0 | 0 | 0 | 0 | 0 |
| 756 | 0.00002 | 0.00002 | 0.908441 | 0.09152 | 0 | 0 | 0 | 0 | 0 |
| 757 | 0.00001 | 0.00004 | 0.910497 | 0.089453 | 0 | 0 | 0 | 0 | 0 |
| 758 | 0.00001 | 0.00003 | 0.906251 | 0.09371 | 0 | 0 | 0 | 0 | 0 |
| 759 | 0.00002 | 0.00006 | 0.908999 | 0.090921 | 0 | 0 | 0 | 0 | 0 |
| 760 | 0 | 0.00004 | 0.90979 | 0.090171 | 0 | 0 | 0 | 0 | 0 |
| 761 | 0 | 0.00001 | 0.909192 | 0.090798 | 0 | 0 | 0 | 0 | 0 |
| 762 | 0.00002 | 0.00001 | 0.909874 | 0.090096 | 0 | 0 | 0 | 0 | 0 |
| 763 | 0.00002 | 0.00001 | 0.908299 | 0.091671 | 0 | 0 | 0 | 0 | 0 |
| 764 | 0.00004 | 0.00001 | 0.91614 | 0.08381 | 0 | 0 | 0 | 0 | 0 |
| 765 | 0.00002 | 0.00003 | 0.906111 | 0.093839 | 0 | 0 | 0 | 0 | 0 |
| 766 | 0.00002 | 0.000051 | 0.904567 | 0.095362 | 0 | 0 | 0 | 0 | 0 |
| 767 | 0 | 0.00005 | 0.909754 | 0.090195 | 0 | 0 | 0 | 0 | 0 |
| 768 | 0.00001 | 0.00002 | 0.906542 | 0.093428 | 0 | 0 | 0 | 0 | 0 |
| 769 | 0 | 0.00001 | 0.909922 | 0.090068 | 0 | 0 | 0 | 0 | 0 |
| 770 | 0.00001 | 0.00002 | 0.909042 | 0.090927 | 0 | 0 | 0 | 0 | 0 |
| 771 | 0.00002 | 0 | 0.907024 | 0.092593 | 4E-04 | 0 | 0 | 0 | 0 |
| 772 | 0.00001 | 0.00004 | 0.90556 | 0.094389 | 0 | 0 | 0 | 0 | 0 |
| 773 | 0.00003 | 0.00001 | 0.903542 | 0.096418 | 0 | 0 | 0 | 0 | 0 |
| 774 | 0.00001 | 0.00004 | 0.909426 | 0.090525 | 0 | 0 | 0 | 0 | 0 |
| 775 | 0.00003 | 0.00002 | 0.909769 | 0.090181 | 0 | 0 | 0 | 0 | 0 |
| 776 | 0 | 0.00005 | 0.904402 | 0.095548 | 0 | 0 | 0 | 0 | 0 |
| 777 | 0.00001 | 0.00004 | 0.909913 | 0.090038 | 0 | 0 | 0 | 0 | 0 |
| 778 | 0.00002 | 0.00001 | 0.911121 | 0.088849 | 0 | 0 | 0 | 0 | 0 |
| 779 | 0 | 0.00002 | 0.914624 | 0.085356 | 0 | 0 | 0 | 0 | 0 |
| 780 | 0.00002 | 0.00003 | 0.907629 | 0.09232 | 0 | 0 | 0 | 0 | 0 |
| 781 | 0.00003 | 0.00001 | 0.907703 | 0.092257 | 0 | 0 | 0 | 0 | 0 |
| 782 | 0 | 0.00001 | 0.912064 | 0.087926 | 0 | 0 | 0 | 0 | 0 |
| 783 | 0 | 0.00004 | 0.908277 | 0.091683 | 0 | 0 | 0 | 0 | 0 |
| 784 | 0 | 0.00003 | 0.908944 | 0.091026 | 0 | 0 | 0 | 0 | 0 |
| 785 | 0.00001 | 0.00001 | 0.910462 | 0.089518 | 0 | 0 | 0 | 0 | 0 |
| 786 | 0.00002 | 0.00004 | 0.9057 | 0.094241 | 0 | 0 | 0 | 0 | 0 |
| 787 | 0.00001 | 0.00004 | 0.907243 | 0.092707 | 0 | 0 | 0 | 0 | 0 |
| 788 | 0 | 0.00003 | 0.913674 | 0.086296 | 0 | 0 | 0 | 0 | 0 |
| 789 | 0.00003 | 0.00002 | 0.90595 | 0.094 | 0 | 0 | 0 | 0 | 0 |
| 790 | 0.00001 | 0.00002 | 0.908963 | 0.091007 | 0 | 0 | 0 | 0 | 0 |
| 791 | 0.00001 | 0.00003 | 0.911384 | 0.088576 | 0 | 0 | 0 | 0 | 0 |
| 792 | 0.00001 | 0.00003 | 0.912684 | 0.087275 | 0 | 0 | 0 | 0 | 0 |
| 793 | 0.00002 | 0.00001 | 0.907447 | 0.092523 | 0 | 0 | 0 | 0 | 0 |
| 794 | 0 | 0 | 0.908709 | 0.091291 | 0 | 0 | 0 | 0 | 0 |
| 795 | 0.00002 | 0.00003 | 0.907431 | 0.092519 | 0 | 0 | 0 | 0 | 0 |
| 796 | 0.00002 | 0.00004 | 0.910808 | 0.089132 | 0 | 0 | 0 | 0 | 0 |
| 797 | 0.00001 | 0 | 0.911287 | 0.088703 | 0 | 0 | 0 | 0 | 0 |
| 798 | 0.00001 | 0.00003 | 0.906147 | 0.093604 | 2E-04 | 0 | 0 | 0 | 0 |
| 799 | 0.00002 | 0.00005 | 0.910288 | 0.089642 | 0 | 0 | 0 | 0 | 0 |
| 800 | 0.00001 | 0.00001 | 0.903912 | 0.096068 | 0 | 0 | 0 | 0 | 0 |
| 801 | 0.00001 | 0.00002 | 0.914142 | 0.085828 | 0 | 0 | 0 | 0 | 0 |
| 802 | 0.00001 | 0.00001 | 0.904895 | 0.095085 | 0 | 0 | 0 | 0 | 0 |
| 803 | 0.00004 | 0.00003 | 0.911812 | 0.088118 | 0 | 0 | 0 | 0 | 0 |
| 804 | 0.00002 | 0.00003 | 0.908954 | 0.090996 | 0 | 0 | 0 | 0 | 0 |
| 805 | 0 | 0.00004 | 0.911696 | 0.088265 | 0 | 0 | 0 | 0 | 0 |
| 806 | 0 | 0.00001 | 0.906128 | 0.093862 | 0 | 0 | 0 | 0 | 0 |
| 807 | 0.00001 | 0 | 0.910682 | 0.089308 | 0 | 0 | 0 | 0 | 0 |
| 808 | 0.00003 | 0.00002 | 0.906385 | 0.093564 | 0 | 0 | 0 | 0 | 0 |
| 809 | 0 | 0.00003 | 0.910126 | 0.089844 | 0 | 0 | 0 | 0 | 0 |
| 810 | 0.00001 | 0.00003 | 0.907991 | 0.091969 | 0 | 0 | 0 | 0 | 0 |
| 811 | 0.00001 | 0.00003 | 0.908794 | 0.091166 | 0 | 0 | 0 | 0 | 0 |
| 812 | 0.00001 | 0.00001 | 0.909009 | 0.090971 | 0 | 0 | 0 | 0 | 0 |
| 813 | 0 | 0.00005 | 0.910767 | 0.089183 | 0 | 0 | 0 | 0 | 0 |
| 814 | 0.00001 | 0.00004 | 0.908841 | 0.091109 | 0 | 0 | 0 | 0 | 0 |
| 815 | 0 | 0 | 0.906492 | 0.093508 | 0 | 0 | 0 | 0 | 0 |
| 816 | 0.00003 | 0.00004 | 0.910545 | 0.089385 | 0 | 0 | 0 | 0 | 0 |
| 817 | 0.00001 | 0.00003 | 0.911575 | 0.088385 | 0 | 0 | 0 | 0 | 0 |
| 818 | 0.00001 | 0.00001 | 0.905856 | 0.094124 | 0 | 0 | 0 | 0 | 0 |
| 819 | 0.00002 | 0.00001 | 0.907118 | 0.092851 | 0 | 0 | 0 | 0 | 0 |
| 820 | 0 | 0 | 0.906411 | 0.093589 | 0 | 0 | 0 | 0 | 0 |
| 821 | 0.00002 | 0.00003 | 0.910718 | 0.089232 | 0 | 0 | 0 | 0 | 0 |
| 822 | 0.00002 | 0.00001 | 0.909742 | 0.090228 | 0 | 0 | 0 | 0 | 0 |
| 823 | 0 | 0.00001 | 0.907017 | 0.092973 | 0 | 0 | 0 | 0 | 0 |
| 824 | 0.00002 | 0.00001 | 0.915847 | 0.084123 | 0 | 0 | 0 | 0 | 0 |
| 825 | 0.00002 | 0.00001 | 0.911476 | 0.088494 | 0 | 0 | 0 | 0 | 0 |
| 826 | 0 | 0.00007 | 0.911286 | 0.088644 | 0 | 0 | 0 | 0 | 0 |
| 827 | 0 | 0.00005 | 0.906249 | 0.093701 | 0 | 0 | 0 | 0 | 0 |
| 828 | 0 | 0 | 0.908159 | 0.091841 | 0 | 0 | 0 | 0 | 0 |
| 829 | 0 | 0.00002 | 0.906587 | 0.093393 | 0 | 0 | 0 | 0 | 0 |
| 830 | 0 | 0.00005 | 0.909606 | 0.090344 | 0 | 0 | 0 | 0 | 0 |
| 831 | 0.00003 | 0.00006 | 0.904937 | 0.094973 | 0 | 0 | 0 | 0 | 0 |
| 832 | 0.00002 | 0 | 0.907943 | 0.092037 | 0 | 0 | 0 | 0 | 0 |
| 833 | 0 | 0.00003 | 0.9098 | 0.09017 | 0 | 0 | 0 | 0 | 0 |
| 834 | 0.00003 | 0.00006 | 0.906934 | 0.092977 | 0 | 0 | 0 | 0 | 0 |
| 835 | 0.00001 | 0.00002 | 0.90636 | 0.09361 | 0 | 0 | 0 | 0 | 0 |
| 836 | 0 | 0.00003 | 0.908009 | 0.091961 | 0 | 0 | 0 | 0 | 0 |
| 837 | 0.00003 | 0.00004 | 0.910966 | 0.088964 | 0 | 0 | 0 | 0 | 0 |
| 838 | 0.00001 | 0.00004 | 0.906697 | 0.093252 | 0 | 0 | 0 | 0 | 0 |
| 839 | 0.00002 | 0 | 0.905414 | 0.094566 | 0 | 0 | 0 | 0 | 0 |
| 840 | 0.00002 | 0 | 0.907648 | 0.092332 | 0 | 0 | 0 | 0 | 0 |
| 841 | 0.00003 | 0.00005 | 0.908096 | 0.091824 | 0 | 0 | 0 | 0 | 0 |
| 842 | 0 | 0.00003 | 0.906757 | 0.093213 | 0 | 0 | 0 | 0 | 0 |
| 843 | 0.00001 | 0 | 0.910739 | 0.089251 | 0 | 0 | 0 | 0 | 0 |
| 844 | 0.00001 | 0.00002 | 0.908215 | 0.091755 | 0 | 0 | 0 | 0 | 0 |
| 845 | 0.00002 | 0 | 0.904148 | 0.095832 | 0 | 0 | 0 | 0 | 0 |
| 846 | 0.00002 | 0.00001 | 0.96513 | 0.03484 | 0 | 0 | 0 | 0 | 0 |
| 847 | 0 | 0.00003 | 0.904705 | 0.095265 | 0 | 0 | 0 | 0 | 0 |
| 848 | 0 | 0.00001 | 0.912328 | 0.087662 | 0 | 0 | 0 | 0 | 0 |
| 849 | 0.00002 | 0.00003 | 0.915116 | 0.084834 | 0 | 0 | 0 | 0 | 0 |
| 850 | 0 | 0 | 0.90978 | 0.09022 | 0 | 0 | 0 | 0 | 0 |
| 851 | 0.00001 | 0.00001 | 0.908791 | 0.091189 | 0 | 0 | 0 | 0 | 0 |
| 852 | 0.00003 | 0.00002 | 0.90565 | 0.0943 | 0 | 0 | 0 | 0 | 0 |
| 853 | 0.00001 | 0.00003 | 0.906453 | 0.093506 | 0 | 0 | 0 | 0 | 0 |
| 854 | 0.00001 | 0.00005 | 0.908756 | 0.091184 | 0 | 0 | 0 | 0 | 0 |
| 855 | 0 | 0.00002 | 0.906945 | 0.093035 | 0 | 0 | 0 | 0 | 0 |
| 856 | 0.00001 | 0.00003 | 0.9059 | 0.09406 | 0 | 0 | 0 | 0 | 0 |
| 857 | 0.00001 | 0.00003 | 0.906892 | 0.093067 | 0 | 0 | 0 | 0 | 0 |
| 858 | 0.00001 | 0.00005 | 0.908464 | 0.091476 | 0 | 0 | 0 | 0 | 0 |
| 859 | 0.00001 | 0.00007 | 0.909697 | 0.090223 | 0 | 0 | 0 | 0 | 0 |
| 860 | 0.00002 | 0.00004 | 0.905519 | 0.09442 | 0 | 0 | 0 | 0 | 0 |
| 861 | 0.00002 | 0.00003 | 0.911221 | 0.088488 | 2E-04 | 0 | 0 | 0 | 0 |
| 862 | 0 | 0.00002 | 0.907172 | 0.092808 | 0 | 0 | 0 | 0 | 0 |
| 863 | 0.00001 | 0.00001 | 0.908261 | 0.091719 | 0 | 0 | 0 | 0 | 0 |
| 864 | 0 | 0.00001 | 0.909005 | 0.090985 | 0 | 0 | 0 | 0 | 0 |
| 865 | 0.00001 | 0.00005 | 0.910053 | 0.089888 | 0 | 0 | 0 | 0 | 0 |
| 866 | 0 | 0.00002 | 0.907077 | 0.092903 | 0 | 0 | 0 | 0 | 0 |
| 867 | 0.00001 | 0.00003 | 0.909007 | 0.090953 | 0 | 0 | 0 | 0 | 0 |
| 868 | 0 | 0.000099 | 0.903325 | 0.096576 | 0 | 0 | 0 | 0 | 0 |
| 869 | 0.00001 | 0 | 0.906623 | 0.093367 | 0 | 0 | 0 | 0 | 0 |
| 870 | 0.00001 | 0.00003 | 0.909152 | 0.090808 | 0 | 0 | 0 | 0 | 0 |
| 871 | 0.00002 | 0.00002 | 0.912891 | 0.087069 | 0 | 0 | 0 | 0 | 0 |
| 872 | 0.00002 | 0.00005 | 0.907871 | 0.092059 | 0 | 0 | 0 | 0 | 0 |
| 873 | 0.00001 | 0.00003 | 0.905291 | 0.094669 | 0 | 0 | 0 | 0 | 0 |
| 874 | 0.00001 | 0.00003 | 0.905964 | 0.093996 | 0 | 0 | 0 | 0 | 0 |
| 875 | 0.00001 | 0.00002 | 0.909323 | 0.090647 | 0 | 0 | 0 | 0 | 0 |
| 876 | 0.00001 | 0.00001 | 0.908686 | 0.091294 | 0 | 0 | 0 | 0 | 0 |
| 877 | 0 | 0 | 0.909618 | 0.090382 | 0 | 0 | 0 | 0 | 0 |
| 878 | 0.00002 | 0 | 0.911111 | 0.088869 | 0 | 0 | 0 | 0 | 0 |
| 879 | 0.00002 | 0.00003 | 0.906804 | 0.093146 | 0 | 0 | 0 | 0 | 0 |
| 880 | 0.00002 | 0.00005 | 0.908922 | 0.091009 | 0 | 0 | 0 | 0 | 0 |
| 881 | 0.00003 | 0.00001 | 0.906619 | 0.093341 | 0 | 0 | 0 | 0 | 0 |
| 882 | 0 | 0.00001 | 0.906982 | 0.093008 | 0 | 0 | 0 | 0 | 0 |
| 883 | 0.00001 | 0.00005 | 0.908347 | 0.091593 | 0 | 0 | 0 | 0 | 0 |
| 884 | 0.00002 | 0.00002 | 0.906734 | 0.093226 | 0 | 0 | 0 | 0 | 0 |
| 885 | 0.00002 | 0.00003 | 0.912161 | 0.087788 | 0 | 0 | 0 | 0 | 0 |
| 886 | 0.00001 | 0.00002 | 0.908156 | 0.091814 | 0 | 0 | 0 | 0 | 0 |
| 887 | 0.00003 | 0.00003 | 0.90898 | 0.09096 | 0 | 0 | 0 | 0 | 0 |
| 888 | 0 | 0.00002 | 0.903574 | 0.096406 | 0 | 0 | 0 | 0 | 0 |
| 889 | 0.00003 | 0.00006 | 0.901442 | 0.098468 | 0 | 0 | 0 | 0 | 0 |
| 890 | 0 | 0.00005 | 0.911276 | 0.088674 | 0 | 0 | 0 | 0 | 0 |
| 891 | 0.00003 | 0.00003 | 0.904936 | 0.095004 | 0 | 0 | 0 | 0 | 0 |
| 892 | 0.00001 | 0.00002 | 0.910759 | 0.089211 | 0 | 0 | 0 | 0 | 0 |
| 893 | 0 | 0.00005 | 0.907151 | 0.092799 | 0 | 0 | 0 | 0 | 0 |
| 894 | 0.00001 | 0.00003 | 0.907142 | 0.092818 | 0 | 0 | 0 | 0 | 0 |
| 895 | 0.00001 | 0.00001 | 0.914701 | 0.085279 | 0 | 0 | 0 | 0 | 0 |
| 896 | 0.00002 | 0 | 0.90835 | 0.09163 | 0 | 0 | 0 | 0 | 0 |
| 897 | 0 | 0.00002 | 0.911307 | 0.088672 | 0 | 0 | 0 | 0 | 0 |
| 898 | 0 | 0 | 0.910869 | 0.089131 | 0 | 0 | 0 | 0 | 0 |
| 899 | 0 | 0.00003 | 0.910918 | 0.089052 | 0 | 0 | 0 | 0 | 0 |
| 900 | 0 | 0.00004 | 0.909511 | 0.09045 | 0 | 0 | 0 | 0 | 0 |
| 901 | 0.00002 | 0.00003 | 0.913902 | 0.086049 | 0 | 0 | 0 | 0 | 0 |
| 902 | 0.00001 | 0.00003 | 0.90585 | 0.09411 | 0 | 0 | 0 | 0 | 0 |
| 903 | 0.00001 | 0.00005 | 0.908727 | 0.091214 | 0 | 0 | 0 | 0 | 0 |
| 904 | 0.00002 | 0.00004 | 0.910428 | 0.089512 | 0 | 0 | 0 | 0 | 0 |
| 905 | 0.00001 | 0.00001 | 0.910443 | 0.089537 | 0 | 0 | 0 | 0 | 0 |
| 906 | 0.00001 | 0.00001 | 0.908935 | 0.091045 | 0 | 0 | 0 | 0 | 0 |
| 907 | 0 | 0 | 0.915328 | 0.084672 | 0 | 0 | 0 | 0 | 0 |
| 908 | 0.00001 | 0.00008 | 0.909398 | 0.090512 | 0 | 0 | 0 | 0 | 0 |
| 909 | 0.00003 | 0 | 0.909016 | 0.090954 | 0 | 0 | 0 | 0 | 0 |
| 910 | 0.00001 | 0.00002 | 0.907351 | 0.092619 | 0 | 0 | 0 | 0 | 0 |
| 911 | 0.00001 | 0 | 0.910786 | 0.089204 | 0 | 0 | 0 | 0 | 0 |
| 912 | 0 | 0 | 0.905447 | 0.094553 | 0 | 0 | 0 | 0 | 0 |
| 913 | 0 | 0.00003 | 0.908379 | 0.091591 | 0 | 0 | 0 | 0 | 0 |
| 914 | 0 | 0.00002 | 0.910773 | 0.089207 | 0 | 0 | 0 | 0 | 0 |
| 915 | 0.00003 | 0 | 0.904573 | 0.095398 | 0 | 0 | 0 | 0 | 0 |
| 916 | 0.00001 | 0.00003 | 0.908477 | 0.091483 | 0 | 0 | 0 | 0 | 0 |
| 917 | 0.00003 | 0.00002 | 0.90895 | 0.091 | 0 | 0 | 0 | 0 | 0 |
| 918 | 0.00001 | 0.00002 | 0.91103 | 0.08894 | 0 | 0 | 0 | 0 | 0 |
| 919 | 0.00003 | 0.00003 | 0.907149 | 0.09279 | 0 | 0 | 0 | 0 | 0 |
| 920 | 0.00002 | 0.00003 | 0.914048 | 0.085902 | 0 | 0 | 0 | 0 | 0 |
| 921 | 0 | 0.00004 | 0.910323 | 0.089637 | 0 | 0 | 0 | 0 | 0 |
| 922 | 0.00002 | 0.00002 | 0.910735 | 0.089225 | 0 | 0 | 0 | 0 | 0 |
| 923 | 0 | 0.00001 | 0.908707 | 0.091283 | 0 | 0 | 0 | 0 | 0 |
| 924 | 0.00002 | 0.00003 | 0.909467 | 0.090483 | 0 | 0 | 0 | 0 | 0 |
| 925 | 0.00002 | 0.00003 | 0.908191 | 0.091759 | 0 | 0 | 0 | 0 | 0 |
| 926 | 0.00001 | 0.00005 | 0.912865 | 0.087075 | 0 | 0 | 0 | 0 | 0 |
| 927 | 0 | 0.00001 | 0.90682 | 0.09317 | 0 | 0 | 0 | 0 | 0 |
| 928 | 0.00002 | 0.00004 | 0.909397 | 0.090543 | 0 | 0 | 0 | 0 | 0 |
| 929 | 0.00002 | 0.00005 | 0.904481 | 0.095449 | 0 | 0 | 0 | 0 | 0 |
| 930 | 0 | 0.00001 | 0.907868 | 0.092122 | 0 | 0 | 0 | 0 | 0 |
| 931 | 0 | 0.00002 | 0.911777 | 0.088203 | 0 | 0 | 0 | 0 | 0 |
| 932 | 0 | 0.00002 | 0.911008 | 0.088972 | 0 | 0 | 0 | 0 | 0 |
| 933 | 0.00001 | 0 | 0.913553 | 0.086437 | 0 | 0 | 0 | 0 | 0 |
| 934 | 0.00001 | 0.00001 | 0.906254 | 0.093726 | 0 | 0 | 0 | 0 | 0 |
| 935 | 0 | 0.00001 | 0.909523 | 0.090467 | 0 | 0 | 0 | 0 | 0 |
| 936 | 0.00001 | 0.00002 | 0.911922 | 0.088048 | 0 | 0 | 0 | 0 | 0 |
| 937 | 0 | 0.00003 | 0.912058 | 0.087912 | 0 | 0 | 0 | 0 | 0 |
| 938 | 0 | 0.00001 | 0.909834 | 0.090156 | 0 | 0 | 0 | 0 | 0 |
| 939 | 0.00002 | 0 | 0.91114 | 0.08884 | 0 | 0 | 0 | 0 | 0 |
| 940 | 0.00001 | 0.00002 | 0.905436 | 0.094534 | 0 | 0 | 0 | 0 | 0 |
| 941 | 0.00003 | 0.00001 | 0.909198 | 0.090762 | 0 | 0 | 0 | 0 | 0 |
| 942 | 0 | 0 | 0.911392 | 0.088608 | 0 | 0 | 0 | 0 | 0 |
| 943 | 0 | 0 | 0.912775 | 0.087225 | 0 | 0 | 0 | 0 | 0 |
| 944 | 0.00003 | 0.00003 | 0.909107 | 0.090832 | 0 | 0 | 0 | 0 | 0 |
| 945 | 0.00001 | 0.00001 | 0.90511 | 0.09487 | 0 | 0 | 0 | 0 | 0 |
| 946 | 0 | 0.00002 | 0.908856 | 0.091124 | 0 | 0 | 0 | 0 | 0 |
| 947 | 0 | 0.00001 | 0.905232 | 0.094757 | 0 | 0 | 0 | 0 | 0 |
| 948 | 0 | 0.00001 | 0.908106 | 0.091884 | 0 | 0 | 0 | 0 | 0 |
| 949 | 0.00001 | 0.00001 | 0.912621 | 0.087359 | 0 | 0 | 0 | 0 | 0 |
| 950 | 0 | 0.00001 | 0.911416 | 0.088574 | 0 | 0 | 0 | 0 | 0 |
| 951 | 0 | 0.00003 | 0.91255 | 0.08742 | 0 | 0 | 0 | 0 | 0 |
| 952 | 0 | 0.00003 | 0.910267 | 0.089702 | 0 | 0 | 0 | 0 | 0 |
| 953 | 0 | 0 | 0.909985 | 0.090015 | 0 | 0 | 0 | 0 | 0 |
| 954 | 0.00003 | 0.00002 | 0.906375 | 0.093575 | 0 | 0 | 0 | 0 | 0 |
| 955 | 0.00002 | 0.00002 | 0.911834 | 0.088126 | 0 | 0 | 0 | 0 | 0 |
| 956 | 0.00001 | 0.00002 | 0.912504 | 0.087466 | 0 | 0 | 0 | 0 | 0 |
| 957 | 0 | 0 | 0.904673 | 0.095327 | 0 | 0 | 0 | 0 | 0 |
| 958 | 0.00002 | 0.00001 | 0.909982 | 0.089988 | 0 | 0 | 0 | 0 | 0 |
| 959 | 0.00002 | 0.00002 | 0.906396 | 0.093564 | 0 | 0 | 0 | 0 | 0 |
| 960 | 0.00001 | 0.00002 | 0.911933 | 0.088037 | 0 | 0 | 0 | 0 | 0 |
| 961 | 0 | 0.000049 | 0.910513 | 0.089438 | 0 | 0 | 0 | 0 | 0 |
| 962 | 0.00002 | 0.00001 | 0.909306 | 0.090664 | 0 | 0 | 0 | 0 | 0 |
| 963 | 0 | 0.00001 | 0.908305 | 0.091685 | 0 | 0 | 0 | 0 | 0 |
| 964 | 0 | 0 | 0.90929 | 0.09071 | 0 | 0 | 0 | 0 | 0 |
| 965 | 0.00001 | 0.00001 | 0.911662 | 0.088318 | 0 | 0 | 0 | 0 | 0 |
| 966 | 0.00001 | 0.00004 | 0.911216 | 0.088734 | 0 | 0 | 0 | 0 | 0 |
| 967 | 0 | 0.00003 | 0.911395 | 0.088575 | 0 | 0 | 0 | 0 | 0 |
| 968 | 0.00003 | 0.00006 | 0.906902 | 0.093008 | 0 | 0 | 0 | 0 | 0 |
| 969 | 0 | 0.00007 | 0.908523 | 0.091407 | 0 | 0 | 0 | 0 | 0 |
| 970 | 0 | 0.00005 | 0.907279 | 0.092671 | 0 | 0 | 0 | 0 | 0 |
| 971 | 0.00002 | 0.000059 | 0.910576 | 0.089345 | 0 | 0 | 0 | 0 | 0 |
| 972 | 0.00002 | 0.00003 | 0.906103 | 0.093847 | 0 | 0 | 0 | 0 | 0 |
| 973 | 0 | 0.00006 | 0.909705 | 0.090235 | 0 | 0 | 0 | 0 | 0 |
| 974 | 0.00001 | 0 | 0.90595 | 0.09404 | 0 | 0 | 0 | 0 | 0 |
| 975 | 0 | 0.00006 | 0.912112 | 0.087829 | 0 | 0 | 0 | 0 | 0 |
| 976 | 0.00002 | 0.00001 | 0.909073 | 0.090897 | 0 | 0 | 0 | 0 | 0 |
| 977 | 0.00001 | 0.00003 | 0.908159 | 0.091801 | 0 | 0 | 0 | 0 | 0 |
| 978 | 0 | 0.00002 | 0.908806 | 0.091174 | 0 | 0 | 0 | 0 | 0 |
| 979 | 0.00004 | 0 | 0.907162 | 0.092798 | 0 | 0 | 0 | 0 | 0 |
| 980 | 0.00001 | 0.00005 | 0.908985 | 0.090955 | 0 | 0 | 0 | 0 | 0 |
| 981 | 0.00002 | 0.00001 | 0.910263 | 0.089707 | 0 | 0 | 0 | 0 | 0 |
| 982 | 0.00002 | 0.00002 | 0.905649 | 0.094311 | 0 | 0 | 0 | 0 | 0 |
| 983 | 0 | 0 | 0.906639 | 0.093361 | 0 | 0 | 0 | 0 | 0 |
| 984 | 0 | 0.00004 | 0.908953 | 0.091007 | 0 | 0 | 0 | 0 | 0 |
| 985 | 0.00001 | 0 | 0.910119 | 0.089871 | 0 | 0 | 0 | 0 | 0 |
| 986 | 0.00002 | 0 | 0.912048 | 0.087932 | 0 | 0 | 0 | 0 | 0 |
| 987 | 0.00001 | 0.00004 | 0.91289 | 0.08706 | 0 | 0 | 0 | 0 | 0 |
| 988 | 0 | 0.00002 | 0.909993 | 0.089987 | 0 | 0 | 0 | 0 | 0 |
| 989 | 0.00001 | 0.00005 | 0.905583 | 0.094357 | 0 | 0 | 0 | 0 | 0 |
| 990 | 0 | 0.00004 | 0.907263 | 0.092697 | 0 | 0 | 0 | 0 | 0 |
| 991 | 0 | 0.00003 | 0.909728 | 0.090242 | 0 | 0 | 0 | 0 | 0 |
| 992 | 0.00002 | 0.00004 | 0.911537 | 0.088403 | 0 | 0 | 0 | 0 | 0 |
| 993 | 0.00003 | 0.00002 | 0.913298 | 0.086652 | 0 | 0 | 0 | 0 | 0 |
| 994 | 0.00002 | 0.00008 | 0.911992 | 0.087907 | 0 | 0 | 0 | 0 | 0 |
| 995 | 0.00001 | 0.00004 | 0.906748 | 0.093202 | 0 | 0 | 0 | 0 | 0 |
| 996 | 0.00001 | 0.00001 | 0.913446 | 0.086534 | 0 | 0 | 0 | 0 | 0 |
| 997 | 0 | 0.00001 | 0.905701 | 0.094289 | 0 | 0 | 0 | 0 | 0 |
| 998 | 0 | 0 | 0.908847 | 0.091153 | 0 | 0 | 0 | 0 | 0 |
| 999 | 0 | 0.00005 | 0.911394 | 0.088556 | 0 | 0 | 0 | 0 | 0 |
| 1000 | 0 | 0.00007 | 0.907226 | 0.092704 | 0 | 0 | 0 | 0 | 0 |

| Year 10 | Race |  |  |  |  |  |  |  |  |
| --- | --- | --- | --- | --- | --- | --- | --- | --- | --- |
| Trial | 003.0 | 005.0 | 007.0 | 037.1 | 043.0 | 303.0 | 003.2 | 403.0 | 003.4 |
| 1 | 0 | 0.00001 | 0.916224 | 0.083766 | 0 | 0 | 0 | 0 | 0 |
| 2 | 0.00005 | 0 | 0.911648 | 0.088302 | 0 | 0 | 0 | 0 | 0 |
| 3 | 0.00004 | 0.00001 | 0.919286 | 0.080664 | 0 | 0 | 0 | 0 | 0 |
| 4 | 0.00001 | 0.00004 | 0.916889 | 0.083062 | 0 | 0 | 0 | 0 | 0 |
| 5 | 0 | 0.00002 | 0.913587 | 0.086393 | 0 | 0 | 0 | 0 | 0 |
| 6 | 0.00002 | 0 | 0.917052 | 0.082928 | 0 | 0 | 0 | 0 | 0 |
| 7 | 0.00002 | 0 | 0.914944 | 0.085036 | 0 | 0 | 0 | 0 | 0 |
| 8 | 0.00002 | 0 | 0.918354 | 0.081626 | 0 | 0 | 0 | 0 | 0 |
| 9 | 0.00001 | 0.00001 | 0.913122 | 0.086858 | 0 | 0 | 0 | 0 | 0 |
| 10 | 0 | 0.00004 | 0.915512 | 0.084448 | 0 | 0 | 0 | 0 | 0 |
| 11 | 0.00003 | 0.00001 | 0.918899 | 0.081061 | 0 | 0 | 0 | 0 | 0 |
| 12 | 0.00002 | 0.00002 | 0.91206 | 0.0879 | 0 | 0 | 0 | 0 | 0 |
| 13 | 0.00001 | 0.00002 | 0.916481 | 0.083489 | 0 | 0 | 0 | 0 | 0 |
| 14 | 0.00001 | 0.00003 | 0.912282 | 0.087678 | 0 | 0 | 0 | 0 | 0 |
| 15 | 0 | 0.00002 | 0.91098 | 0.089 | 0 | 0 | 0 | 0 | 0 |
| 16 | 0.00002 | 0 | 0.916044 | 0.083936 | 0 | 0 | 0 | 0 | 0 |
| 17 | 0 | 0.00002 | 0.915505 | 0.084475 | 0 | 0 | 0 | 0 | 0 |
| 18 | 0 | 0.00001 | 0.915901 | 0.084089 | 0 | 0 | 0 | 0 | 0 |
| 19 | 0.00001 | 0.00001 | 0.916798 | 0.083182 | 0 | 0 | 0 | 0 | 0 |
| 20 | 0.00001 | 0 | 0.913843 | 0.086147 | 0 | 0 | 0 | 0 | 0 |
| 21 | 0.00001 | 0.00001 | 0.915773 | 0.084207 | 0 | 0 | 0 | 0 | 0 |
| 22 | 0.00001 | 0.00003 | 0.912696 | 0.087265 | 0 | 0 | 0 | 0 | 0 |
| 23 | 0 | 0.00002 | 0.917621 | 0.082359 | 0 | 0 | 0 | 0 | 0 |
| 24 | 0 | 0 | 0.91605 | 0.08395 | 0 | 0 | 0 | 0 | 0 |
| 25 | 0.00001 | 0 | 0.917038 | 0.082952 | 0 | 0 | 0 | 0 | 0 |
| 26 | 0.00002 | 0.00002 | 0.919836 | 0.080124 | 0 | 0 | 0 | 0 | 0 |
| 27 | 0.00003 | 0 | 0.915351 | 0.084619 | 0 | 0 | 0 | 0 | 0 |
| 28 | 0.00001 | 0 | 0.91557 | 0.083007 | 0 | 0 | 0 | 0 | 0 |
| 29 | 0.00001 | 0.00001 | 0.918443 | 0.081537 | 0 | 0 | 0 | 0 | 0 |
| 30 | 0.00001 | 0.00002 | 0.914217 | 0.085753 | 0 | 0 | 0 | 0 | 0 |
| 31 | 0 | 0.00003 | 0.917975 | 0.081995 | 0 | 0 | 0 | 0 | 0 |
| 32 | 0.00001 | 0.00002 | 0.917034 | 0.082936 | 0 | 0 | 0 | 0 | 0 |
| 33 | 0.00001 | 0 | 0.915145 | 0.084845 | 0 | 0 | 0 | 0 | 0 |
| 34 | 0 | 0.00001 | 0.916788 | 0.083202 | 0 | 0 | 0 | 0 | 0 |
| 35 | 0.00002 | 0 | 0.912038 | 0.087942 | 0 | 0 | 0 | 0 | 0 |
| 36 | 0.00003 | 0.00002 | 0.913307 | 0.086644 | 0 | 0 | 0 | 0 | 0 |
| 37 | 0.00001 | 0 | 0.921387 | 0.078603 | 0 | 0 | 0 | 0 | 0 |
| 38 | 0 | 0 | 0.916831 | 0.083169 | 0 | 0 | 0 | 0 | 0 |
| 39 | 0 | 0 | 0.916313 | 0.083687 | 0 | 0 | 0 | 0 | 0 |
| 40 | 0.00003 | 0.00002 | 0.910977 | 0.088974 | 0 | 0 | 0 | 0 | 0 |
| 41 | 0.00002 | 0.00001 | 0.912959 | 0.08701 | 0 | 0 | 0 | 0 | 0 |
| 42 | 0.00002 | 0.00001 | 0.91865 | 0.08132 | 0 | 0 | 0 | 0 | 0 |
| 43 | 0 | 0 | 0.913003 | 0.086997 | 0 | 0 | 0 | 0 | 0 |
| 44 | 0.00001 | 0.00002 | 0.917278 | 0.082692 | 0 | 0 | 0 | 0 | 0 |
| 45 | 0.00001 | 0.00003 | 0.916206 | 0.083754 | 0 | 0 | 0 | 0 | 0 |
| 46 | 0.00001 | 0 | 0.912836 | 0.087154 | 0 | 0 | 0 | 0 | 0 |
| 47 | 0 | 0 | 0.916138 | 0.083862 | 0 | 0 | 0 | 0 | 0 |
| 48 | 0.00003 | 0 | 0.916232 | 0.083738 | 0 | 0 | 0 | 0 | 0 |
| 49 | 0.00001 | 0.00003 | 0.917344 | 0.082616 | 0 | 0 | 0 | 0 | 0 |
| 50 | 0 | 0.00003 | 0.918572 | 0.081398 | 0 | 0 | 0 | 0 | 0 |
| 51 | 0.00002 | 0.00002 | 0.913274 | 0.086685 | 0 | 0 | 0 | 0 | 0 |
| 52 | 0.00003 | 0.00002 | 0.917993 | 0.081957 | 0 | 0 | 0 | 0 | 0 |
| 53 | 0.00001 | 0 | 0.917196 | 0.082794 | 0 | 0 | 0 | 0 | 0 |
| 54 | 0.00002 | 0.00003 | 0.916337 | 0.083613 | 0 | 0 | 0 | 0 | 0 |
| 55 | 0.00001 | 0.00002 | 0.914712 | 0.085258 | 0 | 0 | 0 | 0 | 0 |
| 56 | 0.00004 | 0 | 0.915226 | 0.084734 | 0 | 0 | 0 | 0 | 0 |
| 57 | 0.00001 | 0.00001 | 0.91671 | 0.08327 | 0 | 0 | 0 | 0 | 0 |
| 58 | 0.00002 | 0.00002 | 0.915399 | 0.084561 | 0 | 0 | 0 | 0 | 0 |
| 59 | 0 | 0 | 0.919789 | 0.080211 | 0 | 0 | 0 | 0 | 0 |
| 60 | 0.00001 | 0 | 0.915781 | 0.084209 | 0 | 0 | 0 | 0 | 0 |
| 61 | 0.00003 | 0 | 0.912821 | 0.087149 | 0 | 0 | 0 | 0 | 0 |
| 62 | 0.00001 | 0 | 0.916152 | 0.083838 | 0 | 0 | 0 | 0 | 0 |
| 63 | 0.00001 | 0.00002 | 0.914994 | 0.084976 | 0 | 0 | 0 | 0 | 0 |
| 64 | 0.00001 | 0 | 0.913037 | 0.086953 | 0 | 0 | 0 | 0 | 0 |
| 65 | 0 | 0.00001 | 0.917345 | 0.082645 | 0 | 0 | 0 | 0 | 0 |
| 66 | 0.00001 | 0.00001 | 0.919156 | 0.080824 | 0 | 0 | 0 | 0 | 0 |
| 67 | 0 | 0.00002 | 0.918158 | 0.081822 | 0 | 0 | 0 | 0 | 0 |
| 68 | 0.00001 | 0.00002 | 0.914369 | 0.085601 | 0 | 0 | 0 | 0 | 0 |
| 69 | 0 | 0 | 0.91702 | 0.08298 | 0 | 0 | 0 | 0 | 0 |
| 70 | 0 | 0 | 0.916475 | 0.083525 | 0 | 0 | 0 | 0 | 0 |
| 71 | 0.00002 | 0.00005 | 0.91062 | 0.085673 | 0 | 0 | 0 | 0 | 0 |
| 72 | 0 | 0.00001 | 0.919086 | 0.080904 | 0 | 0 | 0 | 0 | 0 |
| 73 | 0 | 0.00001 | 0.91376 | 0.08623 | 0 | 0 | 0 | 0 | 0 |
| 74 | 0.00002 | 0.00004 | 0.919679 | 0.080261 | 0 | 0 | 0 | 0 | 0 |
| 75 | 0.00002 | 0.00005 | 0.916723 | 0.083207 | 0 | 0 | 0 | 0 | 0 |
| 76 | 0 | 0.00002 | 0.917124 | 0.082856 | 0 | 0 | 0 | 0 | 0 |
| 77 | 0.00002 | 0 | 0.91395 | 0.08603 | 0 | 0 | 0 | 0 | 0 |
| 78 | 0.00002 | 0 | 0.915805 | 0.084175 | 0 | 0 | 0 | 0 | 0 |
| 79 | 0.00001 | 0.00001 | 0.917814 | 0.082166 | 0 | 0 | 0 | 0 | 0 |
| 80 | 0 | 0 | 0.916387 | 0.083613 | 0 | 0 | 0 | 0 | 0 |
| 81 | 0.00001 | 0.00002 | 0.915086 | 0.084884 | 0 | 0 | 0 | 0 | 0 |
| 82 | 0.00001 | 0 | 0.910102 | 0.089888 | 0 | 0 | 0 | 0 | 0 |
| 83 | 0.00002 | 0.00001 | 0.918439 | 0.081531 | 0 | 0 | 0 | 0 | 0 |
| 84 | 0.00003 | 0 | 0.917834 | 0.082136 | 0 | 0 | 0 | 0 | 0 |
| 85 | 0.00003 | 0 | 0.913995 | 0.085975 | 0 | 0 | 0 | 0 | 0 |
| 86 | 0.00001 | 0 | 0.924562 | 0.075428 | 0 | 0 | 0 | 0 | 0 |
| 87 | 0.00004 | 0 | 0.914528 | 0.085432 | 0 | 0 | 0 | 0 | 0 |
| 88 | 0.00002 | 0 | 0.915269 | 0.084711 | 0 | 0 | 0 | 0 | 0 |
| 89 | 0.00001 | 0 | 0.914039 | 0.085951 | 0 | 0 | 0 | 0 | 0 |
| 90 | 0.00003 | 0 | 0.915455 | 0.084515 | 0 | 0 | 0 | 0 | 0 |
| 91 | 0.00001 | 0 | 0.915529 | 0.084461 | 0 | 0 | 0 | 0 | 0 |
| 92 | 0.00003 | 0 | 0.916391 | 0.083579 | 0 | 0 | 0 | 0 | 0 |
| 93 | 0 | 0.00001 | 0.919853 | 0.080137 | 0 | 0 | 0 | 0 | 0 |
| 94 | 0 | 0.00001 | 0.917996 | 0.081994 | 0 | 0 | 0 | 0 | 0 |
| 95 | 0.00002 | 0.00003 | 0.916849 | 0.083101 | 0 | 0 | 0 | 0 | 0 |
| 96 | 0.00001 | 0.00001 | 0.916553 | 0.083427 | 0 | 0 | 0 | 0 | 0 |
| 97 | 0.00003 | 0.00001 | 0.914862 | 0.085098 | 0 | 0 | 0 | 0 | 0 |
| 98 | 0.00003 | 0.00001 | 0.913289 | 0.086671 | 0 | 0 | 0 | 0 | 0 |
| 99 | 0.00004 | 0.00001 | 0.911052 | 0.088898 | 0 | 0 | 0 | 0 | 0 |
| 100 | 0.00001 | 0 | 0.918841 | 0.081149 | 0 | 0 | 0 | 0 | 0 |
| 101 | 0 | 0.00001 | 0.918698 | 0.081292 | 0 | 0 | 0 | 0 | 0 |
| 102 | 0.00001 | 0.00003 | 0.918879 | 0.081081 | 0 | 0 | 0 | 0 | 0 |
| 103 | 0 | 0.00002 | 0.917008 | 0.082972 | 0 | 0 | 0 | 0 | 0 |
| 104 | 0.00002 | 0.00001 | 0.919025 | 0.080944 | 0 | 0 | 0 | 0 | 0 |
| 105 | 0.00002 | 0.00001 | 0.914456 | 0.085514 | 0 | 0 | 0 | 0 | 0 |
| 106 | 0 | 0.00003 | 0.916358 | 0.083612 | 0 | 0 | 0 | 0 | 0 |
| 107 | 0.00002 | 0.00004 | 0.915544 | 0.082419 | 0 | 0 | 0 | 0 | 0 |
| 108 | 0 | 0 | 0.909066 | 0.090934 | 0 | 0 | 0 | 0 | 0 |
| 109 | 0.00002 | 0.00002 | 0.916057 | 0.083903 | 0 | 0 | 0 | 0 | 0 |
| 110 | 0.00001 | 0.00001 | 0.915863 | 0.084117 | 0 | 0 | 0 | 0 | 0 |
| 111 | 0 | 0.00002 | 0.918484 | 0.081496 | 0 | 0 | 0 | 0 | 0 |
| 112 | 0.00002 | 0.00001 | 0.915726 | 0.084244 | 0 | 0 | 0 | 0 | 0 |
| 113 | 0.00001 | 0.00001 | 0.916177 | 0.083803 | 0 | 0 | 0 | 0 | 0 |
| 114 | 0.00002 | 0.00001 | 0.915978 | 0.083992 | 0 | 0 | 0 | 0 | 0 |
| 115 | 0.00004 | 0 | 0.919116 | 0.080844 | 0 | 0 | 0 | 0 | 0 |
| 116 | 0.00001 | 0.00003 | 0.916329 | 0.083632 | 0 | 0 | 0 | 0 | 0 |
| 117 | 0.00002 | 0 | 0.915951 | 0.084029 | 0 | 0 | 0 | 0 | 0 |
| 118 | 0 | 0 | 0.917808 | 0.082192 | 0 | 0 | 0 | 0 | 0 |
| 119 | 0.00002 | 0 | 0.91501 | 0.08497 | 0 | 0 | 0 | 0 | 0 |
| 120 | 0.00001 | 0.00001 | 0.915377 | 0.084603 | 0 | 0 | 0 | 0 | 0 |
| 121 | 0 | 0.00001 | 0.91681 | 0.083181 | 0 | 0 | 0 | 0 | 0 |
| 122 | 0.00002 | 0.00001 | 0.919409 | 0.080562 | 0 | 0 | 0 | 0 | 0 |
| 123 | 0.00001 | 0 | 0.918556 | 0.081434 | 0 | 0 | 0 | 0 | 0 |
| 124 | 0.00001 | 0 | 0.918511 | 0.081479 | 0 | 0 | 0 | 0 | 0 |
| 125 | 0.00001 | 0 | 0.915991 | 0.084 | 0 | 0 | 0 | 0 | 0 |
| 126 | 0.00001 | 0 | 0.91578 | 0.08421 | 0 | 0 | 0 | 0 | 0 |
| 127 | 0.00002 | 0 | 0.914608 | 0.085372 | 0 | 0 | 0 | 0 | 0 |
| 128 | 0.00001 | 0 | 0.916088 | 0.083902 | 0 | 0 | 0 | 0 | 0 |
| 129 | 0 | 0 | 0.91431 | 0.08569 | 0 | 0 | 0 | 0 | 0 |
| 130 | 0 | 0.00001 | 0.917127 | 0.082863 | 0 | 0 | 0 | 0 | 0 |
| 131 | 0 | 0 | 0.915823 | 0.084177 | 0 | 0 | 0 | 0 | 0 |
| 132 | 0.00001 | 0 | 0.916717 | 0.083273 | 0 | 0 | 0 | 0 | 0 |
| 133 | 0.00001 | 0 | 0.913247 | 0.086743 | 0 | 0 | 0 | 0 | 0 |
| 134 | 0.00001 | 0.00002 | 0.917694 | 0.082276 | 0 | 0 | 0 | 0 | 0 |
| 135 | 0.00001 | 0 | 0.91216 | 0.08783 | 0 | 0 | 0 | 0 | 0 |
| 136 | 0.00002 | 0.00001 | 0.919705 | 0.080264 | 0 | 0 | 0 | 0 | 0 |
| 137 | 0.00001 | 0 | 0.916301 | 0.083689 | 0 | 0 | 0 | 0 | 0 |
| 138 | 0.00002 | 0.00001 | 0.913658 | 0.086312 | 0 | 0 | 0 | 0 | 0 |
| 139 | 0.00003 | 0 | 0.917513 | 0.082457 | 0 | 0 | 0 | 0 | 0 |
| 140 | 0 | 0 | 0.91505 | 0.08495 | 0 | 0 | 0 | 0 | 0 |
| 141 | 0 | 0.00001 | 0.913141 | 0.086849 | 0 | 0 | 0 | 0 | 0 |
| 142 | 0 | 0.00001 | 0.91117 | 0.088819 | 0 | 0 | 0 | 0 | 0 |
| 143 | 0.00001 | 0 | 0.914167 | 0.085823 | 0 | 0 | 0 | 0 | 0 |
| 144 | 0 | 0.00003 | 0.912997 | 0.086973 | 0 | 0 | 0 | 0 | 0 |
| 145 | 0.00001 | 0.00001 | 0.917321 | 0.082659 | 0 | 0 | 0 | 0 | 0 |
| 146 | 0.00001 | 0.00002 | 0.919629 | 0.080341 | 0 | 0 | 0 | 0 | 0 |
| 147 | 0 | 0 | 0.914771 | 0.085229 | 0 | 0 | 0 | 0 | 0 |
| 148 | 0.00002 | 0.00001 | 0.917507 | 0.082464 | 0 | 0 | 0 | 0 | 0 |
| 149 | 0.00001 | 0 | 0.917017 | 0.082973 | 0 | 0 | 0 | 0 | 0 |
| 150 | 0 | 0.00001 | 0.920753 | 0.079237 | 0 | 0 | 0 | 0 | 0 |
| 151 | 0.00001 | 0.00003 | 0.916397 | 0.083563 | 0 | 0 | 0 | 0 | 0 |
| 152 | 0.00001 | 0 | 0.913133 | 0.086857 | 0 | 0 | 0 | 0 | 0 |
| 153 | 0.00001 | 0.00002 | 0.920143 | 0.079827 | 0 | 0 | 0 | 0 | 0 |
| 154 | 0.00002 | 0.00004 | 0.915521 | 0.084419 | 0 | 0 | 0 | 0 | 0 |
| 155 | 0.00002 | 0.00001 | 0.915536 | 0.084434 | 0 | 0 | 0 | 0 | 0 |
| 156 | 0.00001 | 0 | 0.914782 | 0.085208 | 0 | 0 | 0 | 0 | 0 |
| 157 | 0 | 0 | 0.913213 | 0.086787 | 0 | 0 | 0 | 0 | 0 |
| 158 | 0.00002 | 0.00001 | 0.916773 | 0.083197 | 0 | 0 | 0 | 0 | 0 |
| 159 | 0.00002 | 0 | 0.9145 | 0.08548 | 0 | 0 | 0 | 0 | 0 |
| 160 | 0 | 0 | 0.917796 | 0.082204 | 0 | 0 | 0 | 0 | 0 |
| 161 | 0 | 0 | 0.914873 | 0.085127 | 0 | 0 | 0 | 0 | 0 |
| 162 | 0.00001 | 0 | 0.91705 | 0.08294 | 0 | 0 | 0 | 0 | 0 |
| 163 | 0.00003 | 0 | 0.91493 | 0.08504 | 0 | 0 | 0 | 0 | 0 |
| 164 | 0 | 0.00001 | 0.908884 | 0.091106 | 0 | 0 | 0 | 0 | 0 |
| 165 | 0.00003 | 0.00001 | 0.922115 | 0.077845 | 0 | 0 | 0 | 0 | 0 |
| 166 | 0.00001 | 0 | 0.916097 | 0.083893 | 0 | 0 | 0 | 0 | 0 |
| 167 | 0 | 0.00002 | 0.917012 | 0.082968 | 0 | 0 | 0 | 0 | 0 |
| 168 | 0.00002 | 0 | 0.914961 | 0.085019 | 0 | 0 | 0 | 0 | 0 |
| 169 | 0.00004 | 0.00002 | 0.912733 | 0.087207 | 0 | 0 | 0 | 0 | 0 |
| 170 | 0.00005 | 0.00001 | 0.91637 | 0.08357 | 0 | 0 | 0 | 0 | 0 |
| 171 | 0 | 0.00001 | 0.916024 | 0.083966 | 0 | 0 | 0 | 0 | 0 |
| 172 | 0.00001 | 0.00001 | 0.914158 | 0.085822 | 0 | 0 | 0 | 0 | 0 |
| 173 | 0.00004 | 0.00005 | 0.917895 | 0.082015 | 0 | 0 | 0 | 0 | 0 |
| 174 | 0.00001 | 0.00001 | 0.9133 | 0.08668 | 0 | 0 | 0 | 0 | 0 |
| 175 | 0.00002 | 0 | 0.91258 | 0.0874 | 0 | 0 | 0 | 0 | 0 |
| 176 | 0.00002 | 0.00001 | 0.919794 | 0.080176 | 0 | 0 | 0 | 0 | 0 |
| 177 | 0 | 0 | 0.917588 | 0.082412 | 0 | 0 | 0 | 0 | 0 |
| 178 | 0 | 0.00003 | 0.914597 | 0.085373 | 0 | 0 | 0 | 0 | 0 |
| 179 | 0 | 0.00003 | 0.914804 | 0.085166 | 0 | 0 | 0 | 0 | 0 |
| 180 | 0.00002 | 0.00001 | 0.915712 | 0.084258 | 0 | 0 | 0 | 0 | 0 |
| 181 | 0.00001 | 0 | 0.917476 | 0.082514 | 0 | 0 | 0 | 0 | 0 |
| 182 | 0 | 0.00004 | 0.914253 | 0.085707 | 0 | 0 | 0 | 0 | 0 |
| 183 | 0.00001 | 0 | 0.920491 | 0.079499 | 0 | 0 | 0 | 0 | 0 |
| 184 | 0.00002 | 0 | 0.917958 | 0.082022 | 0 | 0 | 0 | 0 | 0 |
| 185 | 0.00002 | 0.00003 | 0.917566 | 0.082384 | 0 | 0 | 0 | 0 | 0 |
| 186 | 0 | 0 | 0.915914 | 0.084086 | 0 | 0 | 0 | 0 | 0 |
| 187 | 0.00003 | 0.00001 | 0.920739 | 0.079221 | 0 | 0 | 0 | 0 | 0 |
| 188 | 0.00001 | 0.00001 | 0.915588 | 0.084392 | 0 | 0 | 0 | 0 | 0 |
| 189 | 0.00002 | 0.00003 | 0.914008 | 0.085942 | 0 | 0 | 0 | 0 | 0 |
| 190 | 0.00002 | 0.00003 | 0.917139 | 0.082811 | 0 | 0 | 0 | 0 | 0 |
| 191 | 0.00001 | 0.00004 | 0.916311 | 0.083639 | 0 | 0 | 0 | 0 | 0 |
| 192 | 0.00001 | 0.00003 | 0.91543 | 0.08453 | 0 | 0 | 0 | 0 | 0 |
| 193 | 0.00001 | 0.00001 | 0.912676 | 0.087304 | 0 | 0 | 0 | 0 | 0 |
| 194 | 0.00001 | 0.00001 | 0.910828 | 0.089151 | 0 | 0 | 0 | 0 | 0 |
| 195 | 0.00002 | 0.00001 | 0.91288 | 0.08709 | 0 | 0 | 0 | 0 | 0 |
| 196 | 0.00002 | 0.00002 | 0.915782 | 0.084177 | 0 | 0 | 0 | 0 | 0 |
| 197 | 0 | 0 | 0.920821 | 0.079179 | 0 | 0 | 0 | 0 | 0 |
| 198 | 0 | 0 | 0.914117 | 0.082946 | 0 | 0 | 0 | 0 | 0 |
| 199 | 0 | 0.00004 | 0.919824 | 0.080136 | 0 | 0 | 0 | 0 | 0 |
| 200 | 0 | 0 | 0.911016 | 0.088984 | 0 | 0 | 0 | 0 | 0 |
| 201 | 0 | 0 | 0.916827 | 0.083173 | 0 | 0 | 0 | 0 | 0 |
| 202 | 0 | 0.00002 | 0.916688 | 0.083292 | 0 | 0 | 0 | 0 | 0 |
| 203 | 0 | 0 | 0.917923 | 0.082077 | 0 | 0 | 0 | 0 | 0 |
| 204 | 0 | 0.00002 | 0.916426 | 0.083554 | 0 | 0 | 0 | 0 | 0 |
| 205 | 0.00001 | 0 | 0.913249 | 0.086741 | 0 | 0 | 0 | 0 | 0 |
| 206 | 0 | 0 | 0.913507 | 0.086493 | 0 | 0 | 0 | 0 | 0 |
| 207 | 0.00001 | 0.00003 | 0.919907 | 0.080053 | 0 | 0 | 0 | 0 | 0 |
| 208 | 0.00002 | 0.00002 | 0.920008 | 0.079952 | 0 | 0 | 0 | 0 | 0 |
| 209 | 0 | 0 | 0.914947 | 0.085053 | 0 | 0 | 0 | 0 | 0 |
| 210 | 0.00002 | 0.00002 | 0.918032 | 0.081927 | 0 | 0 | 0 | 0 | 0 |
| 211 | 0 | 0 | 0.913683 | 0.086317 | 0 | 0 | 0 | 0 | 0 |
| 212 | 0 | 0.00001 | 0.91513 | 0.084859 | 0 | 0 | 0 | 0 | 0 |
| 213 | 0 | 0.00001 | 0.916821 | 0.083169 | 0 | 0 | 0 | 0 | 0 |
| 214 | 0 | 0.00003 | 0.916969 | 0.083001 | 0 | 0 | 0 | 0 | 0 |
| 215 | 0 | 0 | 0.916247 | 0.083753 | 0 | 0 | 0 | 0 | 0 |
| 216 | 0 | 0.00002 | 0.919854 | 0.080126 | 0 | 0 | 0 | 0 | 0 |
| 217 | 0.00001 | 0.00002 | 0.914937 | 0.085033 | 0 | 0 | 0 | 0 | 0 |
| 218 | 0.00002 | 0 | 0.91772 | 0.08226 | 0 | 0 | 0 | 0 | 0 |
| 219 | 0.00001 | 0 | 0.923777 | 0.076213 | 0 | 0 | 0 | 0 | 0 |
| 220 | 0.00003 | 0.00006 | 0.915327 | 0.084582 | 0 | 0 | 0 | 0 | 0 |
| 221 | 0.00001 | 0 | 0.915564 | 0.084426 | 0 | 0 | 0 | 0 | 0 |
| 222 | 0.00002 | 0 | 0.915217 | 0.084763 | 0 | 0 | 0 | 0 | 0 |
| 223 | 0.00002 | 0.00001 | 0.915707 | 0.084263 | 0 | 0 | 0 | 0 | 0 |
| 224 | 0.00001 | 0.00001 | 0.914623 | 0.085357 | 0 | 0 | 0 | 0 | 0 |
| 225 | 0.00001 | 0 | 0.916711 | 0.083279 | 0 | 0 | 0 | 0 | 0 |
| 226 | 0 | 0 | 0.913455 | 0.086545 | 0 | 0 | 0 | 0 | 0 |
| 227 | 0 | 0 | 0.91671 | 0.08329 | 0 | 0 | 0 | 0 | 0 |
| 228 | 0 | 0.00001 | 0.919989 | 0.080001 | 0 | 0 | 0 | 0 | 0 |
| 229 | 0.00001 | 0.00003 | 0.918329 | 0.081631 | 0 | 0 | 0 | 0 | 0 |
| 230 | 0.00005 | 0.00002 | 0.916554 | 0.083376 | 0 | 0 | 0 | 0 | 0 |
| 231 | 0.00002 | 0.00006 | 0.917208 | 0.082712 | 0 | 0 | 0 | 0 | 0 |
| 232 | 0 | 0.00003 | 0.916655 | 0.083315 | 0 | 0 | 0 | 0 | 0 |
| 233 | 0 | 0.00002 | 0.915665 | 0.084315 | 0 | 0 | 0 | 0 | 0 |
| 234 | 0.00001 | 0 | 0.915675 | 0.084315 | 0 | 0 | 0 | 0 | 0 |
| 235 | 0.00002 | 0 | 0.921166 | 0.078814 | 0 | 0 | 0 | 0 | 0 |
| 236 | 0.00002 | 0 | 0.911013 | 0.088967 | 0 | 0 | 0 | 0 | 0 |
| 237 | 0 | 0 | 0.916262 | 0.083738 | 0 | 0 | 0 | 0 | 0 |
| 238 | 0.00002 | 0.00005 | 0.912838 | 0.087093 | 0 | 0 | 0 | 0 | 0 |
| 239 | 0.00001 | 0 | 0.910914 | 0.089076 | 0 | 0 | 0 | 0 | 0 |
| 240 | 0.00003 | 0 | 0.919031 | 0.080939 | 0 | 0 | 0 | 0 | 0 |
| 241 | 0.00003 | 0.00002 | 0.918069 | 0.081881 | 0 | 0 | 0 | 0 | 0 |
| 242 | 0 | 0.00001 | 0.915879 | 0.084111 | 0 | 0 | 0 | 0 | 0 |
| 243 | 0.00002 | 0.00001 | 0.920246 | 0.079724 | 0 | 0 | 0 | 0 | 0 |
| 244 | 0.00001 | 0 | 0.915495 | 0.084495 | 0 | 0 | 0 | 0 | 0 |
| 245 | 0.00001 | 0.00001 | 0.913396 | 0.085564 | 0 | 0 | 0 | 0 | 0 |
| 246 | 0.00001 | 0.00004 | 0.912704 | 0.087246 | 0 | 0 | 0 | 0 | 0 |
| 247 | 0.00001 | 0 | 0.914871 | 0.085119 | 0 | 0 | 0 | 0 | 0 |
| 248 | 0.00001 | 0 | 0.916781 | 0.083209 | 0 | 0 | 0 | 0 | 0 |
| 249 | 0.00001 | 0.00001 | 0.918723 | 0.081257 | 0 | 0 | 0 | 0 | 0 |
| 250 | 0.00003 | 0 | 0.91316 | 0.08681 | 0 | 0 | 0 | 0 | 0 |
| 251 | 0.00001 | 0.00002 | 0.914795 | 0.085175 | 0 | 0 | 0 | 0 | 0 |
| 252 | 0 | 0.00002 | 0.917595 | 0.082385 | 0 | 0 | 0 | 0 | 0 |
| 253 | 0 | 0.00001 | 0.916793 | 0.083197 | 0 | 0 | 0 | 0 | 0 |
| 254 | 0.00001 | 0 | 0.91562 | 0.08437 | 0 | 0 | 0 | 0 | 0 |
| 255 | 0.00002 | 0 | 0.916365 | 0.083615 | 0 | 0 | 0 | 0 | 0 |
| 256 | 0.00005 | 0.00001 | 0.91378 | 0.086159 | 0 | 0 | 0 | 0 | 0 |
| 257 | 0 | 0 | 0.915924 | 0.084076 | 0 | 0 | 0 | 0 | 0 |
| 258 | 0.00002 | 0.00002 | 0.912331 | 0.087629 | 0 | 0 | 0 | 0 | 0 |
| 259 | 0.00003 | 0.00003 | 0.914488 | 0.085451 | 0 | 0 | 0 | 0 | 0 |
| 260 | 0.00004 | 0 | 0.918416 | 0.081543 | 0 | 0 | 0 | 0 | 0 |
| 261 | 0.00002 | 0 | 0.920671 | 0.079309 | 0 | 0 | 0 | 0 | 0 |
| 262 | 0.00002 | 0.00001 | 0.915914 | 0.084056 | 0 | 0 | 0 | 0 | 0 |
| 263 | 0.00001 | 0.00001 | 0.914977 | 0.085002 | 0 | 0 | 0 | 0 | 0 |
| 264 | 0.00001 | 0 | 0.91262 | 0.08737 | 0 | 0 | 0 | 0 | 0 |
| 265 | 0 | 0.00001 | 0.915971 | 0.084019 | 0 | 0 | 0 | 0 | 0 |
| 266 | 0.00001 | 0.00001 | 0.918565 | 0.081415 | 0 | 0 | 0 | 0 | 0 |
| 267 | 0.00004 | 0 | 0.917438 | 0.082522 | 0 | 0 | 0 | 0 | 0 |
| 268 | 0.00001 | 0.00001 | 0.919551 | 0.080429 | 0 | 0 | 0 | 0 | 0 |
| 269 | 0.00003 | 0 | 0.916265 | 0.083705 | 0 | 0 | 0 | 0 | 0 |
| 270 | 0.00003 | 0.00003 | 0.917986 | 0.081954 | 0 | 0 | 0 | 0 | 0 |
| 271 | 0.00002 | 0 | 0.915978 | 0.084002 | 0 | 0 | 0 | 0 | 0 |
| 272 | 0 | 0.00001 | 0.91785 | 0.08214 | 0 | 0 | 0 | 0 | 0 |
| 273 | 0 | 0 | 0.918886 | 0.081114 | 0 | 0 | 0 | 0 | 0 |
| 274 | 0.00002 | 0.00003 | 0.919471 | 0.080479 | 0 | 0 | 0 | 0 | 0 |
| 275 | 0.00001 | 0 | 0.917434 | 0.082556 | 0 | 0 | 0 | 0 | 0 |
| 276 | 0 | 0 | 0.913869 | 0.086131 | 0 | 0 | 0 | 0 | 0 |
| 277 | 0 | 0.00002 | 0.910997 | 0.088983 | 0 | 0 | 0 | 0 | 0 |
| 278 | 0.00003 | 0 | 0.916592 | 0.083378 | 0 | 0 | 0 | 0 | 0 |
| 279 | 0.00001 | 0 | 0.914251 | 0.085739 | 0 | 0 | 0 | 0 | 0 |
| 280 | 0.00002 | 0.00004 | 0.91488 | 0.08506 | 0 | 0 | 0 | 0 | 0 |
| 281 | 0.00002 | 0.00001 | 0.91316 | 0.08681 | 0 | 0 | 0 | 0 | 0 |
| 282 | 0.00002 | 0 | 0.918208 | 0.081772 | 0 | 0 | 0 | 0 | 0 |
| 283 | 0.00003 | 0.00003 | 0.918368 | 0.081572 | 0 | 0 | 0 | 0 | 0 |
| 284 | 0 | 0.00001 | 0.920186 | 0.079804 | 0 | 0 | 0 | 0 | 0 |
| 285 | 0.00002 | 0 | 0.913694 | 0.086286 | 0 | 0 | 0 | 0 | 0 |
| 286 | 0 | 0.00001 | 0.920517 | 0.079473 | 0 | 0 | 0 | 0 | 0 |
| 287 | 0.00002 | 0 | 0.91712 | 0.08286 | 0 | 0 | 0 | 0 | 0 |
| 288 | 0 | 0 | 0.914582 | 0.085418 | 0 | 0 | 0 | 0 | 0 |
| 289 | 0 | 0 | 0.917483 | 0.082517 | 0 | 0 | 0 | 0 | 0 |
| 290 | 0.00003 | 0.00002 | 0.914766 | 0.085184 | 0 | 0 | 0 | 0 | 0 |
| 291 | 0.00001 | 0.00001 | 0.914297 | 0.085683 | 0 | 0 | 0 | 0 | 0 |
| 292 | 0.00001 | 0.00001 | 0.914115 | 0.085865 | 0 | 0 | 0 | 0 | 0 |
| 293 | 0.00002 | 0.00001 | 0.912607 | 0.087363 | 0 | 0 | 0 | 0 | 0 |
| 294 | 0 | 0.00002 | 0.913549 | 0.086431 | 0 | 0 | 0 | 0 | 0 |
| 295 | 0.00002 | 0 | 0.919437 | 0.080543 | 0 | 0 | 0 | 0 | 0 |
| 296 | 0.00001 | 0 | 0.916191 | 0.083799 | 0 | 0 | 0 | 0 | 0 |
| 297 | 0 | 0.00002 | 0.914667 | 0.085313 | 0 | 0 | 0 | 0 | 0 |
| 298 | 0.00001 | 0 | 0.915818 | 0.084172 | 0 | 0 | 0 | 0 | 0 |
| 299 | 0 | 0 | 0.91025 | 0.08975 | 0 | 0 | 0 | 0 | 0 |
| 300 | 0.00002 | 0.00002 | 0.917265 | 0.082695 | 0 | 0 | 0 | 0 | 0 |
| 301 | 0.00001 | 0 | 0.914481 | 0.085509 | 0 | 0 | 0 | 0 | 0 |
| 302 | 0.00003 | 0 | 0.912299 | 0.087671 | 0 | 0 | 0 | 0 | 0 |
| 303 | 0 | 0 | 0.914516 | 0.085484 | 0 | 0 | 0 | 0 | 0 |
| 304 | 0.00002 | 0.00002 | 0.913872 | 0.086087 | 0 | 0 | 0 | 0 | 0 |
| 305 | 0.00001 | 0 | 0.916328 | 0.083662 | 0 | 0 | 0 | 0 | 0 |
| 306 | 0.00003 | 0.00002 | 0.917572 | 0.082378 | 0 | 0 | 0 | 0 | 0 |
| 307 | 0 | 0.00001 | 0.917109 | 0.082881 | 0 | 0 | 0 | 0 | 0 |
| 308 | 0.00002 | 0.00002 | 0.921063 | 0.078897 | 0 | 0 | 0 | 0 | 0 |
| 309 | 0.00003 | 0 | 0.918588 | 0.081382 | 0 | 0 | 0 | 0 | 0 |
| 310 | 0.00002 | 0.00002 | 0.91456 | 0.0854 | 0 | 0 | 0 | 0 | 0 |
| 311 | 0 | 0 | 0.915753 | 0.084247 | 0 | 0 | 0 | 0 | 0 |
| 312 | 0.00001 | 0 | 0.912528 | 0.087462 | 0 | 0 | 0 | 0 | 0 |
| 313 | 0.00001 | 0.00001 | 0.916654 | 0.083326 | 0 | 0 | 0 | 0 | 0 |
| 314 | 0.00001 | 0 | 0.916196 | 0.083794 | 0 | 0 | 0 | 0 | 0 |
| 315 | 0 | 0.00001 | 0.914871 | 0.085119 | 0 | 0 | 0 | 0 | 0 |
| 316 | 0.00001 | 0 | 0.915865 | 0.084125 | 0 | 0 | 0 | 0 | 0 |
| 317 | 0.00003 | 0.00004 | 0.915331 | 0.084599 | 0 | 0 | 0 | 0 | 0 |
| 318 | 0 | 0.00002 | 0.912485 | 0.087043 | 0 | 0 | 0 | 0 | 0 |
| 319 | 0.00003 | 0 | 0.913692 | 0.086278 | 0 | 0 | 0 | 0 | 0 |
| 320 | 0.00001 | 0.00002 | 0.916702 | 0.083268 | 0 | 0 | 0 | 0 | 0 |
| 321 | 0.00001 | 0 | 0.91468 | 0.08531 | 0 | 0 | 0 | 0 | 0 |
| 322 | 0.00001 | 0.00002 | 0.916202 | 0.083768 | 0 | 0 | 0 | 0 | 0 |
| 323 | 0 | 0 | 0.91659 | 0.08341 | 0 | 0 | 0 | 0 | 0 |
| 324 | 0 | 0 | 0.917687 | 0.082313 | 0 | 0 | 0 | 0 | 0 |
| 325 | 0.00003 | 0 | 0.917972 | 0.081998 | 0 | 0 | 0 | 0 | 0 |
| 326 | 0.00003 | 0.00002 | 0.918017 | 0.081933 | 0 | 0 | 0 | 0 | 0 |
| 327 | 0 | 0.00001 | 0.915041 | 0.084949 | 0 | 0 | 0 | 0 | 0 |
| 328 | 0 | 0.00003 | 0.915216 | 0.084753 | 0 | 0 | 0 | 0 | 0 |
| 329 | 0 | 0.00003 | 0.914083 | 0.085887 | 0 | 0 | 0 | 0 | 0 |
| 330 | 0 | 0 | 0.916165 | 0.083835 | 0 | 0 | 0 | 0 | 0 |
| 331 | 0.00003 | 0.00002 | 0.91547 | 0.08448 | 0 | 0 | 0 | 0 | 0 |
| 332 | 0.00001 | 0 | 0.915847 | 0.084143 | 0 | 0 | 0 | 0 | 0 |
| 333 | 0.00001 | 0 | 0.912584 | 0.087406 | 0 | 0 | 0 | 0 | 0 |
| 334 | 0.00002 | 0.00002 | 0.917004 | 0.082956 | 0 | 0 | 0 | 0 | 0 |
| 335 | 0.00001 | 0 | 0.916744 | 0.083246 | 0 | 0 | 0 | 0 | 0 |
| 336 | 0.00002 | 0.00001 | 0.910204 | 0.089766 | 0 | 0 | 0 | 0 | 0 |
| 337 | 0.00001 | 0 | 0.917354 | 0.082636 | 0 | 0 | 0 | 0 | 0 |
| 338 | 0.00003 | 0 | 0.918021 | 0.081948 | 0 | 0 | 0 | 0 | 0 |
| 339 | 0.00002 | 0.00002 | 0.914848 | 0.085112 | 0 | 0 | 0 | 0 | 0 |
| 340 | 0.00002 | 0.00004 | 0.915068 | 0.084872 | 0 | 0 | 0 | 0 | 0 |
| 341 | 0.00001 | 0 | 0.913902 | 0.086088 | 0 | 0 | 0 | 0 | 0 |
| 342 | 0.00001 | 0 | 0.913919 | 0.086071 | 0 | 0 | 0 | 0 | 0 |
| 343 | 0.00001 | 0.00007 | 0.915162 | 0.084758 | 0 | 0 | 0 | 0 | 0 |
| 344 | 0.00001 | 0 | 0.914574 | 0.085416 | 0 | 0 | 0 | 0 | 0 |
| 345 | 0.00004 | 0 | 0.920204 | 0.079756 | 0 | 0 | 0 | 0 | 0 |
| 346 | 0.00002 | 0.00002 | 0.920609 | 0.079351 | 0 | 0 | 0 | 0 | 0 |
| 347 | 0 | 0.00002 | 0.912994 | 0.086986 | 0 | 0 | 0 | 0 | 0 |
| 348 | 0.00002 | 0.00001 | 0.914681 | 0.085289 | 0 | 0 | 0 | 0 | 0 |
| 349 | 0 | 0 | 0.916073 | 0.083927 | 0 | 0 | 0 | 0 | 0 |
| 350 | 0.00004 | 0.00001 | 0.917607 | 0.082343 | 0 | 0 | 0 | 0 | 0 |
| 351 | 0.00001 | 0.00001 | 0.914355 | 0.085625 | 0 | 0 | 0 | 0 | 0 |
| 352 | 0.00001 | 0 | 0.919158 | 0.080832 | 0 | 0 | 0 | 0 | 0 |
| 353 | 0 | 0 | 0.917185 | 0.082815 | 0 | 0 | 0 | 0 | 0 |
| 354 | 0.00004 | 0 | 0.915074 | 0.084886 | 0 | 0 | 0 | 0 | 0 |
| 355 | 0 | 0.00002 | 0.918911 | 0.081069 | 0 | 0 | 0 | 0 | 0 |
| 356 | 0 | 0 | 0.916052 | 0.083948 | 0 | 0 | 0 | 0 | 0 |
| 357 | 0.00002 | 0.00001 | 0.911474 | 0.088496 | 0 | 0 | 0 | 0 | 0 |
| 358 | 0 | 0.00001 | 0.916435 | 0.083555 | 0 | 0 | 0 | 0 | 0 |
| 359 | 0.00001 | 0.00001 | 0.914019 | 0.085961 | 0 | 0 | 0 | 0 | 0 |
| 360 | 0.00001 | 0 | 0.915048 | 0.084942 | 0 | 0 | 0 | 0 | 0 |
| 361 | 0 | 0.00001 | 0.914081 | 0.085909 | 0 | 0 | 0 | 0 | 0 |
| 362 | 0.00003 | 0.00002 | 0.917825 | 0.082125 | 0 | 0 | 0 | 0 | 0 |
| 363 | 0.00002 | 0.00003 | 0.917099 | 0.082851 | 0 | 0 | 0 | 0 | 0 |
| 364 | 0.00002 | 0.00002 | 0.918172 | 0.081787 | 0 | 0 | 0 | 0 | 0 |
| 365 | 0 | 0.00001 | 0.91546 | 0.08453 | 0 | 0 | 0 | 0 | 0 |
| 366 | 0 | 0 | 0.916112 | 0.083888 | 0 | 0 | 0 | 0 | 0 |
| 367 | 0.00001 | 0.00001 | 0.914192 | 0.085788 | 0 | 0 | 0 | 0 | 0 |
| 368 | 0.00002 | 0.00002 | 0.918404 | 0.081556 | 0 | 0 | 0 | 0 | 0 |
| 369 | 0.00002 | 0 | 0.913046 | 0.086934 | 0 | 0 | 0 | 0 | 0 |
| 370 | 0.00002 | 0 | 0.916175 | 0.083805 | 0 | 0 | 0 | 0 | 0 |
| 371 | 0.00002 | 0.00002 | 0.915887 | 0.083104 | 0 | 0 | 0 | 0 | 0 |
| 372 | 0 | 0.00001 | 0.911753 | 0.088237 | 0 | 0 | 0 | 0 | 0 |
| 373 | 0.00001 | 0.00003 | 0.91292 | 0.08704 | 0 | 0 | 0 | 0 | 0 |
| 374 | 0 | 0.00001 | 0.917545 | 0.082445 | 0 | 0 | 0 | 0 | 0 |
| 375 | 0.00001 | 0 | 0.913097 | 0.086893 | 0 | 0 | 0 | 0 | 0 |
| 376 | 0.00002 | 0.00002 | 0.912246 | 0.087714 | 0 | 0 | 0 | 0 | 0 |
| 377 | 0 | 0.00001 | 0.912276 | 0.087714 | 0 | 0 | 0 | 0 | 0 |
| 378 | 0.00004 | 0.00001 | 0.917431 | 0.082519 | 0 | 0 | 0 | 0 | 0 |
| 379 | 0.00001 | 0 | 0.917646 | 0.082344 | 0 | 0 | 0 | 0 | 0 |
| 380 | 0.00002 | 0.00001 | 0.910799 | 0.089171 | 0 | 0 | 0 | 0 | 0 |
| 381 | 0.00001 | 0.00001 | 0.917402 | 0.082578 | 0 | 0 | 0 | 0 | 0 |
| 382 | 0.00001 | 0.00001 | 0.91634 | 0.08364 | 0 | 0 | 0 | 0 | 0 |
| 383 | 0.00003 | 0.00003 | 0.914351 | 0.085589 | 0 | 0 | 0 | 0 | 0 |
| 384 | 0.00001 | 0 | 0.910928 | 0.089062 | 0 | 0 | 0 | 0 | 0 |
| 385 | 0.00002 | 0.00002 | 0.916911 | 0.083049 | 0 | 0 | 0 | 0 | 0 |
| 386 | 0.00001 | 0.00001 | 0.914946 | 0.085034 | 0 | 0 | 0 | 0 | 0 |
| 387 | 0.00001 | 0.000049 | 0.915234 | 0.084706 | 0 | 0 | 0 | 0 | 0 |
| 388 | 0 | 0 | 0.918877 | 0.081123 | 0 | 0 | 0 | 0 | 0 |
| 389 | 0.00001 | 0.00002 | 0.916138 | 0.083832 | 0 | 0 | 0 | 0 | 0 |
| 390 | 0 | 0.00003 | 0.916446 | 0.083524 | 0 | 0 | 0 | 0 | 0 |
| 391 | 0.00002 | 0.00001 | 0.909428 | 0.090542 | 0 | 0 | 0 | 0 | 0 |
| 392 | 0.00002 | 0 | 0.916958 | 0.083022 | 0 | 0 | 0 | 0 | 0 |
| 393 | 0 | 0.00001 | 0.913523 | 0.086467 | 0 | 0 | 0 | 0 | 0 |
| 394 | 0 | 0.00001 | 0.919107 | 0.080883 | 0 | 0 | 0 | 0 | 0 |
| 395 | 0.00001 | 0.00001 | 0.919309 | 0.080671 | 0 | 0 | 0 | 0 | 0 |
| 396 | 0 | 0 | 0.916728 | 0.083272 | 0 | 0 | 0 | 0 | 0 |
| 397 | 0 | 0.00001 | 0.915886 | 0.084104 | 0 | 0 | 0 | 0 | 0 |
| 398 | 0.00001 | 0 | 0.919357 | 0.080633 | 0 | 0 | 0 | 0 | 0 |
| 399 | 0.00001 | 0.00001 | 0.916559 | 0.083421 | 0 | 0 | 0 | 0 | 0 |
| 400 | 0 | 0.00003 | 0.920256 | 0.079714 | 0 | 0 | 0 | 0 | 0 |
| 401 | 0 | 0.00001 | 0.914865 | 0.085125 | 0 | 0 | 0 | 0 | 0 |
| 402 | 0.00001 | 0.00004 | 0.91625 | 0.083701 | 0 | 0 | 0 | 0 | 0 |
| 403 | 0.00001 | 0.00002 | 0.915998 | 0.083973 | 0 | 0 | 0 | 0 | 0 |
| 404 | 0.00001 | 0.00001 | 0.915687 | 0.084293 | 0 | 0 | 0 | 0 | 0 |
| 405 | 0.00002 | 0 | 0.91731 | 0.08267 | 0 | 0 | 0 | 0 | 0 |
| 406 | 0.00002 | 0.00001 | 0.914918 | 0.085052 | 0 | 0 | 0 | 0 | 0 |
| 407 | 0.00002 | 0 | 0.912575 | 0.087405 | 0 | 0 | 0 | 0 | 0 |
| 408 | 0.00002 | 0.00004 | 0.911917 | 0.088023 | 0 | 0 | 0 | 0 | 0 |
| 409 | 0.00001 | 0.00001 | 0.912723 | 0.087257 | 0 | 0 | 0 | 0 | 0 |
| 410 | 0.00004 | 0.00001 | 0.911731 | 0.08822 | 0 | 0 | 0 | 0 | 0 |
| 411 | 0 | 0.00001 | 0.914997 | 0.084993 | 0 | 0 | 0 | 0 | 0 |
| 412 | 0 | 0.00002 | 0.914431 | 0.085549 | 0 | 0 | 0 | 0 | 0 |
| 413 | 0.00001 | 0.00004 | 0.912442 | 0.087508 | 0 | 0 | 0 | 0 | 0 |
| 414 | 0 | 0 | 0.915905 | 0.084095 | 0 | 0 | 0 | 0 | 0 |
| 415 | 0.00001 | 0 | 0.912685 | 0.087305 | 0 | 0 | 0 | 0 | 0 |
| 416 | 0 | 0.00004 | 0.916814 | 0.083146 | 0 | 0 | 0 | 0 | 0 |
| 417 | 0 | 0.00003 | 0.91825 | 0.08172 | 0 | 0 | 0 | 0 | 0 |
| 418 | 0.00001 | 0 | 0.916796 | 0.083194 | 0 | 0 | 0 | 0 | 0 |
| 419 | 0.00002 | 0 | 0.913028 | 0.086952 | 0 | 0 | 0 | 0 | 0 |
| 420 | 0.00001 | 0.00003 | 0.921922 | 0.078039 | 0 | 0 | 0 | 0 | 0 |
| 421 | 0.00001 | 0 | 0.915338 | 0.084652 | 0 | 0 | 0 | 0 | 0 |
| 422 | 0.00002 | 0.00002 | 0.918439 | 0.081521 | 0 | 0 | 0 | 0 | 0 |
| 423 | 0.00001 | 0 | 0.918702 | 0.081288 | 0 | 0 | 0 | 0 | 0 |
| 424 | 0.00005 | 0.00001 | 0.919106 | 0.080834 | 0 | 0 | 0 | 0 | 0 |
| 425 | 0.00004 | 0.00002 | 0.913362 | 0.086578 | 0 | 0 | 0 | 0 | 0 |
| 426 | 0.00001 | 0.00002 | 0.914519 | 0.085451 | 0 | 0 | 0 | 0 | 0 |
| 427 | 0.00001 | 0.00005 | 0.915976 | 0.083964 | 0 | 0 | 0 | 0 | 0 |
| 428 | 0.00002 | 0.00001 | 0.921036 | 0.078934 | 0 | 0 | 0 | 0 | 0 |
| 429 | 0 | 0 | 0.918999 | 0.081001 | 0 | 0 | 0 | 0 | 0 |
| 430 | 0.00001 | 0.00002 | 0.914469 | 0.085501 | 0 | 0 | 0 | 0 | 0 |
| 431 | 0.00002 | 0 | 0.919187 | 0.080793 | 0 | 0 | 0 | 0 | 0 |
| 432 | 0 | 0.00004 | 0.912253 | 0.087707 | 0 | 0 | 0 | 0 | 0 |
| 433 | 0 | 0 | 0.916897 | 0.083103 | 0 | 0 | 0 | 0 | 0 |
| 434 | 0.00002 | 0.00001 | 0.916187 | 0.083783 | 0 | 0 | 0 | 0 | 0 |
| 435 | 0.00001 | 0 | 0.915063 | 0.084927 | 0 | 0 | 0 | 0 | 0 |
| 436 | 0.00004 | 0 | 0.909748 | 0.090212 | 0 | 0 | 0 | 0 | 0 |
| 437 | 0.00001 | 0.00001 | 0.914477 | 0.085503 | 0 | 0 | 0 | 0 | 0 |
| 438 | 0.00001 | 0.00002 | 0.917551 | 0.082419 | 0 | 0 | 0 | 0 | 0 |
| 439 | 0 | 0.00001 | 0.917025 | 0.082965 | 0 | 0 | 0 | 0 | 0 |
| 440 | 0 | 0 | 0.916361 | 0.083639 | 0 | 0 | 0 | 0 | 0 |
| 441 | 0 | 0.00004 | 0.91484 | 0.08512 | 0 | 0 | 0 | 0 | 0 |
| 442 | 0.00001 | 0 | 0.914794 | 0.085196 | 0 | 0 | 0 | 0 | 0 |
| 443 | 0.00001 | 0.00004 | 0.919577 | 0.080373 | 0 | 0 | 0 | 0 | 0 |
| 444 | 0 | 0.00001 | 0.912977 | 0.087013 | 0 | 0 | 0 | 0 | 0 |
| 445 | 0.00001 | 0 | 0.914049 | 0.085941 | 0 | 0 | 0 | 0 | 0 |
| 446 | 0.00001 | 0 | 0.91597 | 0.08402 | 0 | 0 | 0 | 0 | 0 |
| 447 | 0.00001 | 0.00001 | 0.918144 | 0.081836 | 0 | 0 | 0 | 0 | 0 |
| 448 | 0.00003 | 0.00003 | 0.915284 | 0.084656 | 0 | 0 | 0 | 0 | 0 |
| 449 | 0.00002 | 0 | 0.916922 | 0.083058 | 0 | 0 | 0 | 0 | 0 |
| 450 | 0.00001 | 0 | 0.915692 | 0.084298 | 0 | 0 | 0 | 0 | 0 |
| 451 | 0.00002 | 0 | 0.920752 | 0.079228 | 0 | 0 | 0 | 0 | 0 |
| 452 | 0.00002 | 0 | 0.91713 | 0.08285 | 0 | 0 | 0 | 0 | 0 |
| 453 | 0.00001 | 0 | 0.916844 | 0.083146 | 0 | 0 | 0 | 0 | 0 |
| 454 | 0.00001 | 0 | 0.914697 | 0.085292 | 0 | 0 | 0 | 0 | 0 |
| 455 | 0 | 0 | 0.921374 | 0.078626 | 0 | 0 | 0 | 0 | 0 |
| 456 | 0.00004 | 0.00001 | 0.913793 | 0.086157 | 0 | 0 | 0 | 0 | 0 |
| 457 | 0.00002 | 0.00001 | 0.91548 | 0.08449 | 0 | 0 | 0 | 0 | 0 |
| 458 | 0.00001 | 0 | 0.917785 | 0.082205 | 0 | 0 | 0 | 0 | 0 |
| 459 | 0.00001 | 0 | 0.90998 | 0.09001 | 0 | 0 | 0 | 0 | 0 |
| 460 | 0.00001 | 0.00001 | 0.911108 | 0.088872 | 0 | 0 | 0 | 0 | 0 |
| 461 | 0.00002 | 0 | 0.917591 | 0.082389 | 0 | 0 | 0 | 0 | 0 |
| 462 | 0 | 0 | 0.921584 | 0.078416 | 0 | 0 | 0 | 0 | 0 |
| 463 | 0.00002 | 0 | 0.913603 | 0.086377 | 0 | 0 | 0 | 0 | 0 |
| 464 | 0.00001 | 0 | 0.909653 | 0.086687 | 0 | 0 | 0 | 0 | 0 |
| 465 | 0.00001 | 0 | 0.919812 | 0.080178 | 0 | 0 | 0 | 0 | 0 |
| 466 | 0.00003 | 0.00001 | 0.912182 | 0.087778 | 0 | 0 | 0 | 0 | 0 |
| 467 | 0.00001 | 0.00001 | 0.912184 | 0.087796 | 0 | 0 | 0 | 0 | 0 |
| 468 | 0.00002 | 0 | 0.917989 | 0.081991 | 0 | 0 | 0 | 0 | 0 |
| 469 | 0.00001 | 0.00003 | 0.915015 | 0.084945 | 0 | 0 | 0 | 0 | 0 |
| 470 | 0.00001 | 0.00001 | 0.916352 | 0.083628 | 0 | 0 | 0 | 0 | 0 |
| 471 | 0.00002 | 0 | 0.915314 | 0.084666 | 0 | 0 | 0 | 0 | 0 |
| 472 | 0.00001 | 0 | 0.915023 | 0.084967 | 0 | 0 | 0 | 0 | 0 |
| 473 | 0 | 0.00001 | 0.916556 | 0.083434 | 0 | 0 | 0 | 0 | 0 |
| 474 | 0.00002 | 0.00001 | 0.917712 | 0.082258 | 0 | 0 | 0 | 0 | 0 |
| 475 | 0 | 0 | 0.914924 | 0.085076 | 0 | 0 | 0 | 0 | 0 |
| 476 | 0.00001 | 0.00001 | 0.909118 | 0.090862 | 0 | 0 | 0 | 0 | 0 |
| 477 | 0.00001 | 0 | 0.916808 | 0.083182 | 0 | 0 | 0 | 0 | 0 |
| 478 | 0 | 0.00001 | 0.915579 | 0.084411 | 0 | 0 | 0 | 0 | 0 |
| 479 | 0.00003 | 0 | 0.912017 | 0.087953 | 0 | 0 | 0 | 0 | 0 |
| 480 | 0.00001 | 0 | 0.91326 | 0.08673 | 0 | 0 | 0 | 0 | 0 |
| 481 | 0.00003 | 0.00001 | 0.919958 | 0.080002 | 0 | 0 | 0 | 0 | 0 |
| 482 | 0.00002 | 0.00002 | 0.913244 | 0.086717 | 0 | 0 | 0 | 0 | 0 |
| 483 | 0.00001 | 0.00001 | 0.916751 | 0.083229 | 0 | 0 | 0 | 0 | 0 |
| 484 | 0.00001 | 0 | 0.9123 | 0.08769 | 0 | 0 | 0 | 0 | 0 |
| 485 | 0 | 0.00001 | 0.920671 | 0.079319 | 0 | 0 | 0 | 0 | 0 |
| 486 | 0.00002 | 0 | 0.916245 | 0.083734 | 0 | 0 | 0 | 0 | 0 |
| 487 | 0.00001 | 0 | 0.915449 | 0.084541 | 0 | 0 | 0 | 0 | 0 |
| 488 | 0.00002 | 0.00001 | 0.914932 | 0.085038 | 0 | 0 | 0 | 0 | 0 |
| 489 | 0 | 0 | 0.91551 | 0.08449 | 0 | 0 | 0 | 0 | 0 |
| 490 | 0 | 0.00001 | 0.919597 | 0.080393 | 0 | 0 | 0 | 0 | 0 |
| 491 | 0 | 0.00001 | 0.915378 | 0.084612 | 0 | 0 | 0 | 0 | 0 |
| 492 | 0.00001 | 0.00002 | 0.914254 | 0.085716 | 0 | 0 | 0 | 0 | 0 |
| 493 | 0.00004 | 0.00001 | 0.914428 | 0.085522 | 0 | 0 | 0 | 0 | 0 |
| 494 | 0.00001 | 0 | 0.915392 | 0.084598 | 0 | 0 | 0 | 0 | 0 |
| 495 | 0 | 0 | 0.915011 | 0.084989 | 0 | 0 | 0 | 0 | 0 |
| 496 | 0 | 0 | 0.912793 | 0.08688 | 0 | 0 | 0 | 0 | 0 |
| 497 | 0.00002 | 0.00001 | 0.91495 | 0.08502 | 0 | 0 | 0 | 0 | 0 |
| 498 | 0.00003 | 0.00005 | 0.921037 | 0.078884 | 0 | 0 | 0 | 0 | 0 |
| 499 | 0.00001 | 0 | 0.913296 | 0.086694 | 0 | 0 | 0 | 0 | 0 |
| 500 | 0 | 0.00001 | 0.918656 | 0.081334 | 0 | 0 | 0 | 0 | 0 |
| 501 | 0.00002 | 0 | 0.915184 | 0.084796 | 0 | 0 | 0 | 0 | 0 |
| 502 | 0.00001 | 0.00003 | 0.916276 | 0.083684 | 0 | 0 | 0 | 0 | 0 |
| 503 | 0 | 0.00001 | 0.913111 | 0.086879 | 0 | 0 | 0 | 0 | 0 |
| 504 | 0.00001 | 0.00004 | 0.91726 | 0.08269 | 0 | 0 | 0 | 0 | 0 |
| 505 | 0.00001 | 0 | 0.91297 | 0.086541 | 0 | 0 | 0 | 0 | 0 |
| 506 | 0.00001 | 0.00001 | 0.914094 | 0.085886 | 0 | 0 | 0 | 0 | 0 |
| 507 | 0 | 0.00001 | 0.914745 | 0.085245 | 0 | 0 | 0 | 0 | 0 |
| 508 | 0.00002 | 0.00002 | 0.913711 | 0.086249 | 0 | 0 | 0 | 0 | 0 |
| 509 | 0.00002 | 0 | 0.911886 | 0.088094 | 0 | 0 | 0 | 0 | 0 |
| 510 | 0.00001 | 0.00002 | 0.917945 | 0.082025 | 0 | 0 | 0 | 0 | 0 |
| 511 | 0 | 0 | 0.91468 | 0.08532 | 0 | 0 | 0 | 0 | 0 |
| 512 | 0 | 0.00001 | 0.914088 | 0.085902 | 0 | 0 | 0 | 0 | 0 |
| 513 | 0.00002 | 0.00001 | 0.916544 | 0.083426 | 0 | 0 | 0 | 0 | 0 |
| 514 | 0.00002 | 0 | 0.915666 | 0.084314 | 0 | 0 | 0 | 0 | 0 |
| 515 | 0 | 0 | 0.918592 | 0.081408 | 0 | 0 | 0 | 0 | 0 |
| 516 | 0.00001 | 0 | 0.909917 | 0.090073 | 0 | 0 | 0 | 0 | 0 |
| 517 | 0.00003 | 0 | 0.913343 | 0.086627 | 0 | 0 | 0 | 0 | 0 |
| 518 | 0 | 0 | 0.915627 | 0.084373 | 0 | 0 | 0 | 0 | 0 |
| 519 | 0.00006 | 0.00002 | 0.914506 | 0.085415 | 0 | 0 | 0 | 0 | 0 |
| 520 | 0.00001 | 0 | 0.916213 | 0.083777 | 0 | 0 | 0 | 0 | 0 |
| 521 | 0 | 0 | 0.9146 | 0.0854 | 0 | 0 | 0 | 0 | 0 |
| 522 | 0 | 0.00001 | 0.918489 | 0.081501 | 0 | 0 | 0 | 0 | 0 |
| 523 | 0 | 0.00002 | 0.914155 | 0.085825 | 0 | 0 | 0 | 0 | 0 |
| 524 | 0 | 0.00001 | 0.914719 | 0.085271 | 0 | 0 | 0 | 0 | 0 |
| 525 | 0 | 0 | 0.91311 | 0.08689 | 0 | 0 | 0 | 0 | 0 |
| 526 | 0.00001 | 0.00001 | 0.915897 | 0.084083 | 0 | 0 | 0 | 0 | 0 |
| 527 | 0.00002 | 0 | 0.918007 | 0.081973 | 0 | 0 | 0 | 0 | 0 |
| 528 | 0 | 0 | 0.913076 | 0.086924 | 0 | 0 | 0 | 0 | 0 |
| 529 | 0 | 0.00001 | 0.915659 | 0.084331 | 0 | 0 | 0 | 0 | 0 |
| 530 | 0.00001 | 0 | 0.912984 | 0.087006 | 0 | 0 | 0 | 0 | 0 |
| 531 | 0.00001 | 0.00001 | 0.915604 | 0.084376 | 0 | 0 | 0 | 0 | 0 |
| 532 | 0 | 0 | 0.921759 | 0.078241 | 0 | 0 | 0 | 0 | 0 |
| 533 | 0.00003 | 0.00001 | 0.915023 | 0.084937 | 0 | 0 | 0 | 0 | 0 |
| 534 | 0.00002 | 0.00004 | 0.913632 | 0.086309 | 0 | 0 | 0 | 0 | 0 |
| 535 | 0 | 0.00001 | 0.919235 | 0.080755 | 0 | 0 | 0 | 0 | 0 |
| 536 | 0 | 0 | 0.917945 | 0.082055 | 0 | 0 | 0 | 0 | 0 |
| 537 | 0.00003 | 0 | 0.914646 | 0.085324 | 0 | 0 | 0 | 0 | 0 |
| 538 | 0.00001 | 0.00001 | 0.912673 | 0.087307 | 0 | 0 | 0 | 0 | 0 |
| 539 | 0 | 0 | 0.911064 | 0.088936 | 0 | 0 | 0 | 0 | 0 |
| 540 | 0.00002 | 0 | 0.913737 | 0.086243 | 0 | 0 | 0 | 0 | 0 |
| 541 | 0.00001 | 0.00001 | 0.916938 | 0.083042 | 0 | 0 | 0 | 0 | 0 |
| 542 | 0 | 0.00002 | 0.915456 | 0.084524 | 0 | 0 | 0 | 0 | 0 |
| 543 | 0 | 0.00001 | 0.920598 | 0.079392 | 0 | 0 | 0 | 0 | 0 |
| 544 | 0.00001 | 0 | 0.91883 | 0.08116 | 0 | 0 | 0 | 0 | 0 |
| 545 | 0.00003 | 0.00001 | 0.909623 | 0.090336 | 0 | 0 | 0 | 0 | 0 |
| 546 | 0.00002 | 0.00002 | 0.918756 | 0.081205 | 0 | 0 | 0 | 0 | 0 |
| 547 | 0.00002 | 0.00001 | 0.915096 | 0.084874 | 0 | 0 | 0 | 0 | 0 |
| 548 | 0.00001 | 0.00002 | 0.918937 | 0.081033 | 0 | 0 | 0 | 0 | 0 |
| 549 | 0 | 0.00001 | 0.910854 | 0.089136 | 0 | 0 | 0 | 0 | 0 |
| 550 | 0.00001 | 0 | 0.915535 | 0.084455 | 0 | 0 | 0 | 0 | 0 |
| 551 | 0 | 0.00001 | 0.916594 | 0.083397 | 0 | 0 | 0 | 0 | 0 |
| 552 | 0.00001 | 0.00002 | 0.915141 | 0.084829 | 0 | 0 | 0 | 0 | 0 |
| 553 | 0.00004 | 0 | 0.913834 | 0.086126 | 0 | 0 | 0 | 0 | 0 |
| 554 | 0.00001 | 0.00002 | 0.921451 | 0.078519 | 0 | 0 | 0 | 0 | 0 |
| 555 | 0.00004 | 0.00001 | 0.912686 | 0.087264 | 0 | 0 | 0 | 0 | 0 |
| 556 | 0.00002 | 0 | 0.919314 | 0.080666 | 0 | 0 | 0 | 0 | 0 |
| 557 | 0.00001 | 0 | 0.914879 | 0.085111 | 0 | 0 | 0 | 0 | 0 |
| 558 | 0.00001 | 0.00001 | 0.916829 | 0.083151 | 0 | 0 | 0 | 0 | 0 |
| 559 | 0.00001 | 0 | 0.91863 | 0.08136 | 0 | 0 | 0 | 0 | 0 |
| 560 | 0 | 0.00001 | 0.917058 | 0.082932 | 0 | 0 | 0 | 0 | 0 |
| 561 | 0.00003 | 0.00001 | 0.913306 | 0.086653 | 0 | 0 | 0 | 0 | 0 |
| 562 | 0 | 0 | 0.912525 | 0.087475 | 0 | 0 | 0 | 0 | 0 |
| 563 | 0 | 0.00004 | 0.910963 | 0.088997 | 0 | 0 | 0 | 0 | 0 |
| 564 | 0.00001 | 0.00002 | 0.91758 | 0.08239 | 0 | 0 | 0 | 0 | 0 |
| 565 | 0.00001 | 0 | 0.912254 | 0.087736 | 0 | 0 | 0 | 0 | 0 |
| 566 | 0.00002 | 0.00003 | 0.91752 | 0.08243 | 0 | 0 | 0 | 0 | 0 |
| 567 | 0.00001 | 0.00002 | 0.915414 | 0.084556 | 0 | 0 | 0 | 0 | 0 |
| 568 | 0.00001 | 0 | 0.912497 | 0.087493 | 0 | 0 | 0 | 0 | 0 |
| 569 | 0.00001 | 0 | 0.918327 | 0.081663 | 0 | 0 | 0 | 0 | 0 |
| 570 | 0.00001 | 0 | 0.910483 | 0.089507 | 0 | 0 | 0 | 0 | 0 |
| 571 | 0.00003 | 0.00001 | 0.914663 | 0.085297 | 0 | 0 | 0 | 0 | 0 |
| 572 | 0.00001 | 0 | 0.916953 | 0.083037 | 0 | 0 | 0 | 0 | 0 |
| 573 | 0.00001 | 0.00003 | 0.917275 | 0.082685 | 0 | 0 | 0 | 0 | 0 |
| 574 | 0.00002 | 0 | 0.915695 | 0.084285 | 0 | 0 | 0 | 0 | 0 |
| 575 | 0 | 0.00001 | 0.915471 | 0.084518 | 0 | 0 | 0 | 0 | 0 |
| 576 | 0.00002 | 0.00001 | 0.9214 | 0.07857 | 0 | 0 | 0 | 0 | 0 |
| 577 | 0.00001 | 0 | 0.913922 | 0.086068 | 0 | 0 | 0 | 0 | 0 |
| 578 | 0.00003 | 0 | 0.917223 | 0.082747 | 0 | 0 | 0 | 0 | 0 |
| 579 | 0 | 0.00002 | 0.912276 | 0.087704 | 0 | 0 | 0 | 0 | 0 |
| 580 | 0 | 0.00001 | 0.917599 | 0.082391 | 0 | 0 | 0 | 0 | 0 |
| 581 | 0.00003 | 0.00001 | 0.912491 | 0.087469 | 0 | 0 | 0 | 0 | 0 |
| 582 | 0.00002 | 0 | 0.915017 | 0.084963 | 0 | 0 | 0 | 0 | 0 |
| 583 | 0.00001 | 0.00003 | 0.912321 | 0.087639 | 0 | 0 | 0 | 0 | 0 |
| 584 | 0.00001 | 0 | 0.910158 | 0.089832 | 0 | 0 | 0 | 0 | 0 |
| 585 | 0 | 0.00001 | 0.912169 | 0.087821 | 0 | 0 | 0 | 0 | 0 |
| 586 | 0.00001 | 0.00002 | 0.916856 | 0.083114 | 0 | 0 | 0 | 0 | 0 |
| 587 | 0 | 0 | 0.916446 | 0.083554 | 0 | 0 | 0 | 0 | 0 |
| 588 | 0.00002 | 0 | 0.919756 | 0.080224 | 0 | 0 | 0 | 0 | 0 |
| 589 | 0.00001 | 0.00006 | 0.916181 | 0.083749 | 0 | 0 | 0 | 0 | 0 |
| 590 | 0.00001 | 0.00001 | 0.912125 | 0.087855 | 0 | 0 | 0 | 0 | 0 |
| 591 | 0.00001 | 0.00001 | 0.913128 | 0.086852 | 0 | 0 | 0 | 0 | 0 |
| 592 | 0.00003 | 0.00004 | 0.915248 | 0.084681 | 0 | 0 | 0 | 0 | 0 |
| 593 | 0.00003 | 0 | 0.917894 | 0.082076 | 0 | 0 | 0 | 0 | 0 |
| 594 | 0.00002 | 0 | 0.913647 | 0.086333 | 0 | 0 | 0 | 0 | 0 |
| 595 | 0.00002 | 0.00004 | 0.913451 | 0.08649 | 0 | 0 | 0 | 0 | 0 |
| 596 | 0 | 0.00001 | 0.917969 | 0.082021 | 0 | 0 | 0 | 0 | 0 |
| 597 | 0.00001 | 0.00002 | 0.915814 | 0.084156 | 0 | 0 | 0 | 0 | 0 |
| 598 | 0.00002 | 0 | 0.915502 | 0.084478 | 0 | 0 | 0 | 0 | 0 |
| 599 | 0.00003 | 0.00002 | 0.915205 | 0.084745 | 0 | 0 | 0 | 0 | 0 |
| 600 | 0.00001 | 0.00004 | 0.911323 | 0.088628 | 0 | 0 | 0 | 0 | 0 |
| 601 | 0.00001 | 0 | 0.9169 | 0.08309 | 0 | 0 | 0 | 0 | 0 |
| 602 | 0.00002 | 0 | 0.919514 | 0.080466 | 0 | 0 | 0 | 0 | 0 |
| 603 | 0 | 0 | 0.918145 | 0.081855 | 0 | 0 | 0 | 0 | 0 |
| 604 | 0 | 0.00001 | 0.914505 | 0.085485 | 0 | 0 | 0 | 0 | 0 |
| 605 | 0.00002 | 0 | 0.916111 | 0.083869 | 0 | 0 | 0 | 0 | 0 |
| 606 | 0.00002 | 0 | 0.918974 | 0.081006 | 0 | 0 | 0 | 0 | 0 |
| 607 | 0 | 0 | 0.919284 | 0.080716 | 0 | 0 | 0 | 0 | 0 |
| 608 | 0.00001 | 0 | 0.915889 | 0.084101 | 0 | 0 | 0 | 0 | 0 |
| 609 | 0 | 0 | 0.917628 | 0.082372 | 0 | 0 | 0 | 0 | 0 |
| 610 | 0 | 0 | 0.913471 | 0.086529 | 0 | 0 | 0 | 0 | 0 |
| 611 | 0.00001 | 0.00002 | 0.916644 | 0.083326 | 0 | 0 | 0 | 0 | 0 |
| 612 | 0.00002 | 0.00001 | 0.91512 | 0.08485 | 0 | 0 | 0 | 0 | 0 |
| 613 | 0.00002 | 0.00001 | 0.909794 | 0.090176 | 0 | 0 | 0 | 0 | 0 |
| 614 | 0.00001 | 0.00001 | 0.918372 | 0.081608 | 0 | 0 | 0 | 0 | 0 |
| 615 | 0.00003 | 0.00001 | 0.915561 | 0.084399 | 0 | 0 | 0 | 0 | 0 |
| 616 | 0 | 0.00002 | 0.914521 | 0.085459 | 0 | 0 | 0 | 0 | 0 |
| 617 | 0 | 0 | 0.91563 | 0.08437 | 0 | 0 | 0 | 0 | 0 |
| 618 | 0.00001 | 0.00001 | 0.912147 | 0.087833 | 0 | 0 | 0 | 0 | 0 |
| 619 | 0.00002 | 0.00002 | 0.919186 | 0.080774 | 0 | 0 | 0 | 0 | 0 |
| 620 | 0.00002 | 0.00003 | 0.914751 | 0.085199 | 0 | 0 | 0 | 0 | 0 |
| 621 | 0 | 0 | 0.917726 | 0.082274 | 0 | 0 | 0 | 0 | 0 |
| 622 | 0 | 0.00002 | 0.911026 | 0.088954 | 0 | 0 | 0 | 0 | 0 |
| 623 | 0 | 0 | 0.915258 | 0.084742 | 0 | 0 | 0 | 0 | 0 |
| 624 | 0.00002 | 0.00001 | 0.914595 | 0.085376 | 0 | 0 | 0 | 0 | 0 |
| 625 | 0 | 0.00002 | 0.913767 | 0.085083 | 0 | 0 | 0 | 0 | 0 |
| 626 | 0.00001 | 0.00004 | 0.912374 | 0.087576 | 0 | 0 | 0 | 0 | 0 |
| 627 | 0.00001 | 0 | 0.918542 | 0.081448 | 0 | 0 | 0 | 0 | 0 |
| 628 | 0.00002 | 0.00005 | 0.914906 | 0.085024 | 0 | 0 | 0 | 0 | 0 |
| 629 | 0.00002 | 0 | 0.915204 | 0.084776 | 0 | 0 | 0 | 0 | 0 |
| 630 | 0.00002 | 0 | 0.916772 | 0.083208 | 0 | 0 | 0 | 0 | 0 |
| 631 | 0.00002 | 0.00001 | 0.914093 | 0.085877 | 0 | 0 | 0 | 0 | 0 |
| 632 | 0 | 0 | 0.916812 | 0.083188 | 0 | 0 | 0 | 0 | 0 |
| 633 | 0 | 0.00001 | 0.916016 | 0.083974 | 0 | 0 | 0 | 0 | 0 |
| 634 | 0 | 0 | 0.914018 | 0.085982 | 0 | 0 | 0 | 0 | 0 |
| 635 | 0.00001 | 0.00004 | 0.919483 | 0.080467 | 0 | 0 | 0 | 0 | 0 |
| 636 | 0 | 0.00001 | 0.913988 | 0.086002 | 0 | 0 | 0 | 0 | 0 |
| 637 | 0.00001 | 0 | 0.913812 | 0.086178 | 0 | 0 | 0 | 0 | 0 |
| 638 | 0.00002 | 0 | 0.914698 | 0.085282 | 0 | 0 | 0 | 0 | 0 |
| 639 | 0 | 0 | 0.912613 | 0.087387 | 0 | 0 | 0 | 0 | 0 |
| 640 | 0.00001 | 0.00003 | 0.909689 | 0.090272 | 0 | 0 | 0 | 0 | 0 |
| 641 | 0.00001 | 0 | 0.916577 | 0.083413 | 0 | 0 | 0 | 0 | 0 |
| 642 | 0 | 0.00001 | 0.917105 | 0.082885 | 0 | 0 | 0 | 0 | 0 |
| 643 | 0.00001 | 0.00001 | 0.914014 | 0.085966 | 0 | 0 | 0 | 0 | 0 |
| 644 | 0.00002 | 0.00005 | 0.916663 | 0.083267 | 0 | 0 | 0 | 0 | 0 |
| 645 | 0.00001 | 0.00003 | 0.916024 | 0.083936 | 0 | 0 | 0 | 0 | 0 |
| 646 | 0.00004 | 0.00001 | 0.91504 | 0.084909 | 0 | 0 | 0 | 0 | 0 |
| 647 | 0.00001 | 0.00002 | 0.915251 | 0.084719 | 0 | 0 | 0 | 0 | 0 |
| 648 | 0.00001 | 0 | 0.918203 | 0.081787 | 0 | 0 | 0 | 0 | 0 |
| 649 | 0.00002 | 0 | 0.913823 | 0.086157 | 0 | 0 | 0 | 0 | 0 |
| 650 | 0 | 0 | 0.915373 | 0.084627 | 0 | 0 | 0 | 0 | 0 |
| 651 | 0.00001 | 0.00001 | 0.915408 | 0.084572 | 0 | 0 | 0 | 0 | 0 |
| 652 | 0.00002 | 0.00003 | 0.915583 | 0.084367 | 0 | 0 | 0 | 0 | 0 |
| 653 | 0.00001 | 0.00001 | 0.918878 | 0.081102 | 0 | 0 | 0 | 0 | 0 |
| 654 | 0.00001 | 0.00002 | 0.91565 | 0.08432 | 0 | 0 | 0 | 0 | 0 |
| 655 | 0 | 0.00001 | 0.913618 | 0.086372 | 0 | 0 | 0 | 0 | 0 |
| 656 | 0.00002 | 0 | 0.922338 | 0.077642 | 0 | 0 | 0 | 0 | 0 |
| 657 | 0.00002 | 0.00002 | 0.915701 | 0.084259 | 0 | 0 | 0 | 0 | 0 |
| 658 | 0.00001 | 0.00002 | 0.915547 | 0.084423 | 0 | 0 | 0 | 0 | 0 |
| 659 | 0 | 0.00001 | 0.912 | 0.08799 | 0 | 0 | 0 | 0 | 0 |
| 660 | 0.00001 | 0 | 0.91997 | 0.08002 | 0 | 0 | 0 | 0 | 0 |
| 661 | 0.00005 | 0.00001 | 0.9173 | 0.08264 | 0 | 0 | 0 | 0 | 0 |
| 662 | 0.00001 | 0 | 0.913434 | 0.086556 | 0 | 0 | 0 | 0 | 0 |
| 663 | 0.00001 | 0 | 0.916291 | 0.083699 | 0 | 0 | 0 | 0 | 0 |
| 664 | 0.00001 | 0.00004 | 0.919112 | 0.080838 | 0 | 0 | 0 | 0 | 0 |
| 665 | 0 | 0.00001 | 0.914448 | 0.085542 | 0 | 0 | 0 | 0 | 0 |
| 666 | 0.00001 | 0 | 0.915989 | 0.084 | 0 | 0 | 0 | 0 | 0 |
| 667 | 0 | 0 | 0.911885 | 0.088115 | 0 | 0 | 0 | 0 | 0 |
| 668 | 0 | 0.00001 | 0.912888 | 0.087102 | 0 | 0 | 0 | 0 | 0 |
| 669 | 0.00001 | 0.00002 | 0.913149 | 0.086821 | 0 | 0 | 0 | 0 | 0 |
| 670 | 0.00002 | 0 | 0.915274 | 0.084706 | 0 | 0 | 0 | 0 | 0 |
| 671 | 0 | 0 | 0.916594 | 0.083406 | 0 | 0 | 0 | 0 | 0 |
| 672 | 0.00001 | 0.00003 | 0.915297 | 0.084663 | 0 | 0 | 0 | 0 | 0 |
| 673 | 0.00003 | 0.00001 | 0.913782 | 0.086178 | 0 | 0 | 0 | 0 | 0 |
| 674 | 0.00003 | 0 | 0.915448 | 0.084522 | 0 | 0 | 0 | 0 | 0 |
| 675 | 0.00003 | 0 | 0.915788 | 0.084182 | 0 | 0 | 0 | 0 | 0 |
| 676 | 0.00004 | 0.00004 | 0.919397 | 0.080523 | 0 | 0 | 0 | 0 | 0 |
| 677 | 0.00002 | 0 | 0.915913 | 0.084067 | 0 | 0 | 0 | 0 | 0 |
| 678 | 0.00002 | 0 | 0.914983 | 0.084997 | 0 | 0 | 0 | 0 | 0 |
| 679 | 0.00002 | 0 | 0.918116 | 0.081864 | 0 | 0 | 0 | 0 | 0 |
| 680 | 0.00005 | 0.00001 | 0.913713 | 0.086227 | 0 | 0 | 0 | 0 | 0 |
| 681 | 0 | 0 | 0.912801 | 0.087199 | 0 | 0 | 0 | 0 | 0 |
| 682 | 0 | 0 | 0.913833 | 0.086167 | 0 | 0 | 0 | 0 | 0 |
| 683 | 0.00002 | 0.00003 | 0.916688 | 0.083261 | 0 | 0 | 0 | 0 | 0 |
| 684 | 0.00002 | 0 | 0.91718 | 0.0828 | 0 | 0 | 0 | 0 | 0 |
| 685 | 0.00001 | 0 | 0.915416 | 0.084574 | 0 | 0 | 0 | 0 | 0 |
| 686 | 0.00001 | 0 | 0.910207 | 0.089783 | 0 | 0 | 0 | 0 | 0 |
| 687 | 0.00004 | 0.00001 | 0.917345 | 0.082605 | 0 | 0 | 0 | 0 | 0 |
| 688 | 0.00003 | 0 | 0.917719 | 0.082251 | 0 | 0 | 0 | 0 | 0 |
| 689 | 0.00001 | 0.00001 | 0.912554 | 0.087426 | 0 | 0 | 0 | 0 | 0 |
| 690 | 0.00001 | 0.00001 | 0.913995 | 0.085985 | 0 | 0 | 0 | 0 | 0 |
| 691 | 0 | 0.00004 | 0.917589 | 0.082371 | 0 | 0 | 0 | 0 | 0 |
| 692 | 0 | 0 | 0.914354 | 0.085646 | 0 | 0 | 0 | 0 | 0 |
| 693 | 0.00002 | 0 | 0.912628 | 0.087352 | 0 | 0 | 0 | 0 | 0 |
| 694 | 0 | 0.00002 | 0.91867 | 0.08131 | 0 | 0 | 0 | 0 | 0 |
| 695 | 0.00002 | 0 | 0.915586 | 0.084394 | 0 | 0 | 0 | 0 | 0 |
| 696 | 0.00003 | 0.00003 | 0.920212 | 0.079728 | 0 | 0 | 0 | 0 | 0 |
| 697 | 0 | 0 | 0.917545 | 0.082455 | 0 | 0 | 0 | 0 | 0 |
| 698 | 0.00002 | 0.00001 | 0.914774 | 0.085196 | 0 | 0 | 0 | 0 | 0 |
| 699 | 0.00001 | 0.00001 | 0.916863 | 0.083117 | 0 | 0 | 0 | 0 | 0 |
| 700 | 0.00003 | 0 | 0.914544 | 0.085426 | 0 | 0 | 0 | 0 | 0 |
| 701 | 0.00003 | 0.00003 | 0.920553 | 0.079387 | 0 | 0 | 0 | 0 | 0 |
| 702 | 0.00001 | 0.00005 | 0.918252 | 0.081688 | 0 | 0 | 0 | 0 | 0 |
| 703 | 0.00002 | 0.00001 | 0.914084 | 0.085886 | 0 | 0 | 0 | 0 | 0 |
| 704 | 0.00001 | 0 | 0.915413 | 0.084577 | 0 | 0 | 0 | 0 | 0 |
| 705 | 0.00001 | 0 | 0.915215 | 0.084775 | 0 | 0 | 0 | 0 | 0 |
| 706 | 0.00001 | 0 | 0.914981 | 0.085009 | 0 | 0 | 0 | 0 | 0 |
| 707 | 0.00001 | 0 | 0.913703 | 0.086287 | 0 | 0 | 0 | 0 | 0 |
| 708 | 0.00002 | 0.00001 | 0.914455 | 0.085515 | 0 | 0 | 0 | 0 | 0 |
| 709 | 0 | 0.00002 | 0.919997 | 0.079983 | 0 | 0 | 0 | 0 | 0 |
| 710 | 0 | 0.00001 | 0.915092 | 0.084898 | 0 | 0 | 0 | 0 | 0 |
| 711 | 0 | 0.00001 | 0.916229 | 0.083761 | 0 | 0 | 0 | 0 | 0 |
| 712 | 0 | 0 | 0.920278 | 0.079722 | 0 | 0 | 0 | 0 | 0 |
| 713 | 0.00003 | 0 | 0.911621 | 0.088349 | 0 | 0 | 0 | 0 | 0 |
| 714 | 0.00001 | 0 | 0.913256 | 0.086734 | 0 | 0 | 0 | 0 | 0 |
| 715 | 0.00003 | 0.00001 | 0.911998 | 0.087961 | 0 | 0 | 0 | 0 | 0 |
| 716 | 0 | 0.00001 | 0.914087 | 0.085903 | 0 | 0 | 0 | 0 | 0 |
| 717 | 0.00001 | 0.00001 | 0.915539 | 0.084441 | 0 | 0 | 0 | 0 | 0 |
| 718 | 0.00003 | 0.00003 | 0.915256 | 0.084684 | 0 | 0 | 0 | 0 | 0 |
| 719 | 0 | 0.00001 | 0.919409 | 0.080581 | 0 | 0 | 0 | 0 | 0 |
| 720 | 0.00002 | 0.00004 | 0.914534 | 0.085406 | 0 | 0 | 0 | 0 | 0 |
| 721 | 0 | 0 | 0.913288 | 0.086712 | 0 | 0 | 0 | 0 | 0 |
| 722 | 0 | 0 | 0.916233 | 0.083767 | 0 | 0 | 0 | 0 | 0 |
| 723 | 0.00001 | 0.00002 | 0.912884 | 0.087086 | 0 | 0 | 0 | 0 | 0 |
| 724 | 0 | 0 | 0.917213 | 0.082787 | 0 | 0 | 0 | 0 | 0 |
| 725 | 0.00002 | 0 | 0.91444 | 0.085541 | 0 | 0 | 0 | 0 | 0 |
| 726 | 0 | 0.00003 | 0.916256 | 0.083714 | 0 | 0 | 0 | 0 | 0 |
| 727 | 0 | 0.00003 | 0.914176 | 0.085794 | 0 | 0 | 0 | 0 | 0 |
| 728 | 0.00002 | 0.00007 | 0.915494 | 0.084416 | 0 | 0 | 0 | 0 | 0 |
| 729 | 0.00001 | 0.00002 | 0.912829 | 0.087141 | 0 | 0 | 0 | 0 | 0 |
| 730 | 0.00001 | 0.00002 | 0.914832 | 0.085138 | 0 | 0 | 0 | 0 | 0 |
| 731 | 0.00001 | 0 | 0.918218 | 0.081772 | 0 | 0 | 0 | 0 | 0 |
| 732 | 0.00002 | 0 | 0.91787 | 0.08211 | 0 | 0 | 0 | 0 | 0 |
| 733 | 0.00002 | 0.00001 | 0.913965 | 0.086005 | 0 | 0 | 0 | 0 | 0 |
| 734 | 0.00001 | 0.00002 | 0.917119 | 0.082851 | 0 | 0 | 0 | 0 | 0 |
| 735 | 0 | 0.00003 | 0.919683 | 0.080287 | 0 | 0 | 0 | 0 | 0 |
| 736 | 0 | 0.00001 | 0.919222 | 0.080768 | 0 | 0 | 0 | 0 | 0 |
| 737 | 0 | 0.00003 | 0.915127 | 0.084843 | 0 | 0 | 0 | 0 | 0 |
| 738 | 0.00002 | 0.00001 | 0.911344 | 0.088625 | 0 | 0 | 0 | 0 | 0 |
| 739 | 0.00001 | 0 | 0.90798 | 0.09201 | 0 | 0 | 0 | 0 | 0 |
| 740 | 0 | 0 | 0.91781 | 0.08219 | 0 | 0 | 0 | 0 | 0 |
| 741 | 0.00002 | 0.00001 | 0.914707 | 0.085263 | 0 | 0 | 0 | 0 | 0 |
| 742 | 0.00001 | 0 | 0.91479 | 0.0852 | 0 | 0 | 0 | 0 | 0 |
| 743 | 0 | 0.00001 | 0.916273 | 0.083717 | 0 | 0 | 0 | 0 | 0 |
| 744 | 0.00004 | 0.00001 | 0.915934 | 0.081586 | 0 | 0 | 0 | 0 | 0 |
| 745 | 0 | 0.00002 | 0.919055 | 0.080925 | 0 | 0 | 0 | 0 | 0 |
| 746 | 0.00001 | 0.00002 | 0.909671 | 0.090299 | 0 | 0 | 0 | 0 | 0 |
| 747 | 0.00003 | 0 | 0.915174 | 0.084796 | 0 | 0 | 0 | 0 | 0 |
| 748 | 0.00002 | 0.00001 | 0.918157 | 0.081813 | 0 | 0 | 0 | 0 | 0 |
| 749 | 0 | 0 | 0.922114 | 0.077886 | 0 | 0 | 0 | 0 | 0 |
| 750 | 0 | 0 | 0.916868 | 0.083132 | 0 | 0 | 0 | 0 | 0 |
| 751 | 0.00001 | 0 | 0.919014 | 0.080976 | 0 | 0 | 0 | 0 | 0 |
| 752 | 0.00001 | 0 | 0.918081 | 0.081909 | 0 | 0 | 0 | 0 | 0 |
| 753 | 0 | 0 | 0.915743 | 0.084257 | 0 | 0 | 0 | 0 | 0 |
| 754 | 0.00001 | 0.00003 | 0.914254 | 0.085705 | 0 | 0 | 0 | 0 | 0 |
| 755 | 0.00003 | 0.00002 | 0.914733 | 0.085217 | 0 | 0 | 0 | 0 | 0 |
| 756 | 0.00002 | 0 | 0.913352 | 0.086628 | 0 | 0 | 0 | 0 | 0 |
| 757 | 0 | 0.00002 | 0.918216 | 0.081764 | 0 | 0 | 0 | 0 | 0 |
| 758 | 0.00001 | 0.00002 | 0.913614 | 0.086356 | 0 | 0 | 0 | 0 | 0 |
| 759 | 0.00001 | 0.00002 | 0.917026 | 0.082943 | 0 | 0 | 0 | 0 | 0 |
| 760 | 0.00001 | 0.00001 | 0.918145 | 0.081835 | 0 | 0 | 0 | 0 | 0 |
| 761 | 0.00001 | 0 | 0.914461 | 0.085529 | 0 | 0 | 0 | 0 | 0 |
| 762 | 0.00003 | 0.00001 | 0.915956 | 0.084004 | 0 | 0 | 0 | 0 | 0 |
| 763 | 0 | 0 | 0.917052 | 0.082948 | 0 | 0 | 0 | 0 | 0 |
| 764 | 0.00001 | 0 | 0.923485 | 0.076505 | 0 | 0 | 0 | 0 | 0 |
| 765 | 0 | 0.00001 | 0.913981 | 0.086009 | 0 | 0 | 0 | 0 | 0 |
| 766 | 0 | 0.00002 | 0.911792 | 0.088188 | 0 | 0 | 0 | 0 | 0 |
| 767 | 0.00001 | 0.00001 | 0.916254 | 0.083726 | 0 | 0 | 0 | 0 | 0 |
| 768 | 0.00001 | 0.00001 | 0.912809 | 0.087171 | 0 | 0 | 0 | 0 | 0 |
| 769 | 0.00002 | 0 | 0.915773 | 0.084207 | 0 | 0 | 0 | 0 | 0 |
| 770 | 0.00001 | 0.00001 | 0.915089 | 0.084891 | 0 | 0 | 0 | 0 | 0 |
| 771 | 0.00001 | 0 | 0.91315 | 0.085065 | 0 | 0 | 0 | 0 | 0 |
| 772 | 0.00001 | 0.00003 | 0.912795 | 0.087165 | 0 | 0 | 0 | 0 | 0 |
| 773 | 0.00001 | 0 | 0.910468 | 0.089522 | 0 | 0 | 0 | 0 | 0 |
| 774 | 0 | 0 | 0.917256 | 0.082744 | 0 | 0 | 0 | 0 | 0 |
| 775 | 0.00001 | 0 | 0.916334 | 0.083656 | 0 | 0 | 0 | 0 | 0 |
| 776 | 0 | 0 | 0.912047 | 0.087953 | 0 | 0 | 0 | 0 | 0 |
| 777 | 0 | 0.00002 | 0.917286 | 0.082694 | 0 | 0 | 0 | 0 | 0 |
| 778 | 0.00002 | 0.00001 | 0.917541 | 0.082429 | 0 | 0 | 0 | 0 | 0 |
| 779 | 0 | 0.00001 | 0.923115 | 0.076875 | 0 | 0 | 0 | 0 | 0 |
| 780 | 0.00004 | 0.00001 | 0.915097 | 0.084853 | 0 | 0 | 0 | 0 | 0 |
| 781 | 0.00002 | 0.00002 | 0.914584 | 0.085376 | 0 | 0 | 0 | 0 | 0 |
| 782 | 0.00001 | 0 | 0.920091 | 0.079899 | 0 | 0 | 0 | 0 | 0 |
| 783 | 0 | 0.00002 | 0.91622 | 0.08376 | 0 | 0 | 0 | 0 | 0 |
| 784 | 0.00002 | 0 | 0.916188 | 0.083792 | 0 | 0 | 0 | 0 | 0 |
| 785 | 0.00002 | 0.00001 | 0.917577 | 0.082393 | 0 | 0 | 0 | 0 | 0 |
| 786 | 0.00003 | 0.00004 | 0.914546 | 0.085384 | 0 | 0 | 0 | 0 | 0 |
| 787 | 0 | 0.00001 | 0.91483 | 0.08516 | 0 | 0 | 0 | 0 | 0 |
| 788 | 0.00001 | 0.00001 | 0.921329 | 0.078651 | 0 | 0 | 0 | 0 | 0 |
| 789 | 0.00001 | 0 | 0.91256 | 0.08743 | 0 | 0 | 0 | 0 | 0 |
| 790 | 0.00002 | 0 | 0.916631 | 0.083349 | 0 | 0 | 0 | 0 | 0 |
| 791 | 0 | 0 | 0.9182 | 0.0818 | 0 | 0 | 0 | 0 | 0 |
| 792 | 0.00001 | 0.00002 | 0.918442 | 0.081528 | 0 | 0 | 0 | 0 | 0 |
| 793 | 0.00001 | 0 | 0.914887 | 0.085103 | 0 | 0 | 0 | 0 | 0 |
| 794 | 0 | 0 | 0.917223 | 0.082777 | 0 | 0 | 0 | 0 | 0 |
| 795 | 0.00002 | 0 | 0.914302 | 0.085677 | 0 | 0 | 0 | 0 | 0 |
| 796 | 0 | 0 | 0.918493 | 0.081507 | 0 | 0 | 0 | 0 | 0 |
| 797 | 0.00003 | 0 | 0.917017 | 0.082952 | 0 | 0 | 0 | 0 | 0 |
| 798 | 0.00001 | 0.00002 | 0.912362 | 0.086649 | 0 | 0 | 0 | 0 | 0 |
| 799 | 0.00002 | 0.00003 | 0.917084 | 0.082865 | 0 | 0 | 0 | 0 | 0 |
| 800 | 0 | 0.00001 | 0.911709 | 0.088281 | 0 | 0 | 0 | 0 | 0 |
| 801 | 0.00001 | 0 | 0.921022 | 0.078968 | 0 | 0 | 0 | 0 | 0 |
| 802 | 0.00001 | 0 | 0.911844 | 0.088146 | 0 | 0 | 0 | 0 | 0 |
| 803 | 0 | 0.00001 | 0.919078 | 0.080912 | 0 | 0 | 0 | 0 | 0 |
| 804 | 0.00001 | 0.00003 | 0.916136 | 0.083824 | 0 | 0 | 0 | 0 | 0 |
| 805 | 0.00002 | 0 | 0.918803 | 0.081177 | 0 | 0 | 0 | 0 | 0 |
| 806 | 0.00001 | 0 | 0.913587 | 0.086403 | 0 | 0 | 0 | 0 | 0 |
| 807 | 0.00001 | 0 | 0.919421 | 0.080569 | 0 | 0 | 0 | 0 | 0 |
| 808 | 0.00001 | 0.00001 | 0.914339 | 0.085641 | 0 | 0 | 0 | 0 | 0 |
| 809 | 0.00001 | 0 | 0.916068 | 0.083922 | 0 | 0 | 0 | 0 | 0 |
| 810 | 0.00002 | 0 | 0.915535 | 0.084445 | 0 | 0 | 0 | 0 | 0 |
| 811 | 0.00002 | 0.00002 | 0.916121 | 0.083839 | 0 | 0 | 0 | 0 | 0 |
| 812 | 0 | 0 | 0.916481 | 0.083519 | 0 | 0 | 0 | 0 | 0 |
| 813 | 0.00001 | 0.00003 | 0.917319 | 0.082641 | 0 | 0 | 0 | 0 | 0 |
| 814 | 0.00001 | 0.00001 | 0.915866 | 0.084114 | 0 | 0 | 0 | 0 | 0 |
| 815 | 0.00001 | 0 | 0.913985 | 0.086005 | 0 | 0 | 0 | 0 | 0 |
| 816 | 0.00001 | 0 | 0.916251 | 0.083739 | 0 | 0 | 0 | 0 | 0 |
| 817 | 0.00002 | 0 | 0.91809 | 0.08189 | 0 | 0 | 0 | 0 | 0 |
| 818 | 0 | 0 | 0.911808 | 0.088192 | 0 | 0 | 0 | 0 | 0 |
| 819 | 0.00002 | 0 | 0.91382 | 0.08616 | 0 | 0 | 0 | 0 | 0 |
| 820 | 0.00001 | 0 | 0.915286 | 0.084704 | 0 | 0 | 0 | 0 | 0 |
| 821 | 0.00001 | 0.00001 | 0.918265 | 0.081714 | 0 | 0 | 0 | 0 | 0 |
| 822 | 0.00001 | 0 | 0.917638 | 0.082352 | 0 | 0 | 0 | 0 | 0 |
| 823 | 0.00001 | 0 | 0.916313 | 0.083677 | 0 | 0 | 0 | 0 | 0 |
| 824 | 0.00004 | 0.00002 | 0.922548 | 0.077392 | 0 | 0 | 0 | 0 | 0 |
| 825 | 0 | 0 | 0.916912 | 0.083088 | 0 | 0 | 0 | 0 | 0 |
| 826 | 0.00002 | 0.00001 | 0.919332 | 0.080638 | 0 | 0 | 0 | 0 | 0 |
| 827 | 0.00001 | 0 | 0.91501 | 0.08498 | 0 | 0 | 0 | 0 | 0 |
| 828 | 0.00001 | 0 | 0.915022 | 0.084968 | 0 | 0 | 0 | 0 | 0 |
| 829 | 0.00001 | 0.00001 | 0.913221 | 0.086759 | 0 | 0 | 0 | 0 | 0 |
| 830 | 0.00003 | 0.00002 | 0.91705 | 0.0829 | 0 | 0 | 0 | 0 | 0 |
| 831 | 0 | 0.00003 | 0.912683 | 0.087287 | 0 | 0 | 0 | 0 | 0 |
| 832 | 0.00002 | 0 | 0.915156 | 0.084824 | 0 | 0 | 0 | 0 | 0 |
| 833 | 0.00001 | 0.00003 | 0.916115 | 0.083845 | 0 | 0 | 0 | 0 | 0 |
| 834 | 0 | 0.00003 | 0.914285 | 0.085685 | 0 | 0 | 0 | 0 | 0 |
| 835 | 0.00001 | 0.00001 | 0.914684 | 0.085296 | 0 | 0 | 0 | 0 | 0 |
| 836 | 0.00001 | 0 | 0.915702 | 0.084288 | 0 | 0 | 0 | 0 | 0 |
| 837 | 0.00002 | 0.00001 | 0.917476 | 0.082493 | 0 | 0 | 0 | 0 | 0 |
| 838 | 0.00001 | 0.00001 | 0.915314 | 0.084666 | 0 | 0 | 0 | 0 | 0 |
| 839 | 0.00001 | 0 | 0.912967 | 0.087023 | 0 | 0 | 0 | 0 | 0 |
| 840 | 0.00001 | 0 | 0.913051 | 0.086939 | 0 | 0 | 0 | 0 | 0 |
| 841 | 0.00003 | 0.00002 | 0.915705 | 0.084244 | 0 | 0 | 0 | 0 | 0 |
| 842 | 0.00002 | 0 | 0.913512 | 0.086468 | 0 | 0 | 0 | 0 | 0 |
| 843 | 0 | 0 | 0.916722 | 0.083278 | 0 | 0 | 0 | 0 | 0 |
| 844 | 0.00001 | 0 | 0.913889 | 0.086101 | 0 | 0 | 0 | 0 | 0 |
| 845 | 0.00001 | 0 | 0.912453 | 0.087537 | 0 | 0 | 0 | 0 | 0 |
| 846 | 0.00001 | 0 | 0.968245 | 0.031745 | 0 | 0 | 0 | 0 | 0 |
| 847 | 0 | 0.00001 | 0.912362 | 0.087628 | 0 | 0 | 0 | 0 | 0 |
| 848 | 0 | 0 | 0.919629 | 0.080371 | 0 | 0 | 0 | 0 | 0 |
| 849 | 0.00003 | 0.00005 | 0.921647 | 0.078274 | 0 | 0 | 0 | 0 | 0 |
| 850 | 0 | 0 | 0.916136 | 0.083864 | 0 | 0 | 0 | 0 | 0 |
| 851 | 0 | 0 | 0.915375 | 0.084625 | 0 | 0 | 0 | 0 | 0 |
| 852 | 0 | 0 | 0.91382 | 0.08618 | 0 | 0 | 0 | 0 | 0 |
| 853 | 0.00001 | 0.00002 | 0.912574 | 0.087396 | 0 | 0 | 0 | 0 | 0 |
| 854 | 0 | 0.00001 | 0.916203 | 0.083787 | 0 | 0 | 0 | 0 | 0 |
| 855 | 0.00001 | 0.00001 | 0.913912 | 0.086068 | 0 | 0 | 0 | 0 | 0 |
| 856 | 0.00002 | 0.00002 | 0.913901 | 0.086059 | 0 | 0 | 0 | 0 | 0 |
| 857 | 0.00002 | 0.00002 | 0.916136 | 0.083824 | 0 | 0 | 0 | 0 | 0 |
| 858 | 0.00002 | 0 | 0.916608 | 0.083372 | 0 | 0 | 0 | 0 | 0 |
| 859 | 0 | 0.00002 | 0.916355 | 0.083625 | 0 | 0 | 0 | 0 | 0 |
| 860 | 0 | 0.00002 | 0.913265 | 0.086715 | 0 | 0 | 0 | 0 | 0 |
| 861 | 0.00001 | 0.00002 | 0.916606 | 0.082292 | 0 | 0 | 0 | 0 | 0 |
| 862 | 0.00002 | 0 | 0.91476 | 0.08522 | 0 | 0 | 0 | 0 | 0 |
| 863 | 0.00003 | 0 | 0.913698 | 0.086272 | 0 | 0 | 0 | 0 | 0 |
| 864 | 0 | 0 | 0.917225 | 0.082775 | 0 | 0 | 0 | 0 | 0 |
| 865 | 0.00001 | 0.00001 | 0.916178 | 0.083802 | 0 | 0 | 0 | 0 | 0 |
| 866 | 0 | 0 | 0.914952 | 0.085048 | 0 | 0 | 0 | 0 | 0 |
| 867 | 0.00001 | 0 | 0.916935 | 0.083055 | 0 | 0 | 0 | 0 | 0 |
| 868 | 0.00001 | 0.00005 | 0.912515 | 0.087425 | 0 | 0 | 0 | 0 | 0 |
| 869 | 0.00002 | 0 | 0.91491 | 0.08507 | 0 | 0 | 0 | 0 | 0 |
| 870 | 0 | 0.00001 | 0.916013 | 0.083977 | 0 | 0 | 0 | 0 | 0 |
| 871 | 0.00004 | 0.00001 | 0.921892 | 0.078058 | 0 | 0 | 0 | 0 | 0 |
| 872 | 0.00002 | 0.00002 | 0.913526 | 0.086434 | 0 | 0 | 0 | 0 | 0 |
| 873 | 0 | 0.00001 | 0.912198 | 0.087792 | 0 | 0 | 0 | 0 | 0 |
| 874 | 0.00001 | 0.00002 | 0.914625 | 0.085346 | 0 | 0 | 0 | 0 | 0 |
| 875 | 0.00003 | 0.00001 | 0.916461 | 0.083498 | 0 | 0 | 0 | 0 | 0 |
| 876 | 0.00001 | 0.00001 | 0.91446 | 0.08552 | 0 | 0 | 0 | 0 | 0 |
| 877 | 0.00002 | 0 | 0.918751 | 0.081229 | 0 | 0 | 0 | 0 | 0 |
| 878 | 0.00001 | 0 | 0.919705 | 0.080285 | 0 | 0 | 0 | 0 | 0 |
| 879 | 0.00001 | 0.00001 | 0.914383 | 0.085597 | 0 | 0 | 0 | 0 | 0 |
| 880 | 0.00001 | 0.00002 | 0.916687 | 0.083284 | 0 | 0 | 0 | 0 | 0 |
| 881 | 0.00001 | 0.00001 | 0.915263 | 0.084717 | 0 | 0 | 0 | 0 | 0 |
| 882 | 0 | 0 | 0.914312 | 0.085688 | 0 | 0 | 0 | 0 | 0 |
| 883 | 0.00001 | 0.00001 | 0.915503 | 0.084477 | 0 | 0 | 0 | 0 | 0 |
| 884 | 0.00002 | 0 | 0.915018 | 0.084962 | 0 | 0 | 0 | 0 | 0 |
| 885 | 0.00001 | 0 | 0.919762 | 0.080228 | 0 | 0 | 0 | 0 | 0 |
| 886 | 0.00001 | 0 | 0.914956 | 0.085034 | 0 | 0 | 0 | 0 | 0 |
| 887 | 0.00001 | 0.00002 | 0.915869 | 0.084101 | 0 | 0 | 0 | 0 | 0 |
| 888 | 0 | 0 | 0.911966 | 0.088034 | 0 | 0 | 0 | 0 | 0 |
| 889 | 0.00002 | 0.00006 | 0.908771 | 0.09115 | 0 | 0 | 0 | 0 | 0 |
| 890 | 0 | 0 | 0.919405 | 0.080595 | 0 | 0 | 0 | 0 | 0 |
| 891 | 0 | 0.00003 | 0.910933 | 0.089037 | 0 | 0 | 0 | 0 | 0 |
| 892 | 0.00001 | 0.00002 | 0.916646 | 0.083324 | 0 | 0 | 0 | 0 | 0 |
| 893 | 0 | 0.00002 | 0.91402 | 0.08596 | 0 | 0 | 0 | 0 | 0 |
| 894 | 0.00001 | 0.00001 | 0.913013 | 0.086967 | 0 | 0 | 0 | 0 | 0 |
| 895 | 0 | 0.00001 | 0.921907 | 0.078083 | 0 | 0 | 0 | 0 | 0 |
| 896 | 0.00001 | 0 | 0.915053 | 0.084937 | 0 | 0 | 0 | 0 | 0 |
| 897 | 0 | 0 | 0.918819 | 0.081181 | 0 | 0 | 0 | 0 | 0 |
| 898 | 0.00001 | 0 | 0.918088 | 0.081902 | 0 | 0 | 0 | 0 | 0 |
| 899 | 0 | 0.00001 | 0.920066 | 0.079924 | 0 | 0 | 0 | 0 | 0 |
| 900 | 0 | 0 | 0.916241 | 0.083759 | 0 | 0 | 0 | 0 | 0 |
| 901 | 0.00002 | 0.00001 | 0.920308 | 0.079662 | 0 | 0 | 0 | 0 | 0 |
| 902 | 0 | 0 | 0.912179 | 0.087821 | 0 | 0 | 0 | 0 | 0 |
| 903 | 0.00003 | 0.00003 | 0.915598 | 0.084342 | 0 | 0 | 0 | 0 | 0 |
| 904 | 0.00002 | 0.00001 | 0.91898 | 0.08099 | 0 | 0 | 0 | 0 | 0 |
| 905 | 0 | 0.00001 | 0.91666 | 0.08333 | 0 | 0 | 0 | 0 | 0 |
| 906 | 0.00001 | 0 | 0.916034 | 0.083956 | 0 | 0 | 0 | 0 | 0 |
| 907 | 0.00002 | 0 | 0.922258 | 0.077722 | 0 | 0 | 0 | 0 | 0 |
| 908 | 0.00002 | 0.00001 | 0.915258 | 0.084712 | 0 | 0 | 0 | 0 | 0 |
| 909 | 0.00002 | 0 | 0.916385 | 0.083595 | 0 | 0 | 0 | 0 | 0 |
| 910 | 0.00001 | 0 | 0.914383 | 0.085607 | 0 | 0 | 0 | 0 | 0 |
| 911 | 0.00002 | 0 | 0.918342 | 0.081638 | 0 | 0 | 0 | 0 | 0 |
| 912 | 0 | 0 | 0.911164 | 0.088836 | 0 | 0 | 0 | 0 | 0 |
| 913 | 0.00001 | 0.00005 | 0.915407 | 0.084533 | 0 | 0 | 0 | 0 | 0 |
| 914 | 0 | 0.00002 | 0.91876 | 0.08122 | 0 | 0 | 0 | 0 | 0 |
| 915 | 0.00001 | 0 | 0.909942 | 0.090048 | 0 | 0 | 0 | 0 | 0 |
| 916 | 0.00001 | 0.00002 | 0.916955 | 0.083015 | 0 | 0 | 0 | 0 | 0 |
| 917 | 0.00001 | 0.00001 | 0.917289 | 0.082691 | 0 | 0 | 0 | 0 | 0 |
| 918 | 0.00002 | 0.00001 | 0.918878 | 0.081092 | 0 | 0 | 0 | 0 | 0 |
| 919 | 0.00002 | 0.00001 | 0.913652 | 0.086318 | 0 | 0 | 0 | 0 | 0 |
| 920 | 0.00001 | 0.00003 | 0.921043 | 0.078916 | 0 | 0 | 0 | 0 | 0 |
| 921 | 0 | 0.00002 | 0.918297 | 0.081683 | 0 | 0 | 0 | 0 | 0 |
| 922 | 0.00002 | 0.00001 | 0.916775 | 0.083195 | 0 | 0 | 0 | 0 | 0 |
| 923 | 0.00002 | 0 | 0.915981 | 0.083999 | 0 | 0 | 0 | 0 | 0 |
| 924 | 0 | 0.00001 | 0.916373 | 0.083617 | 0 | 0 | 0 | 0 | 0 |
| 925 | 0.00003 | 0.00002 | 0.915638 | 0.084311 | 0 | 0 | 0 | 0 | 0 |
| 926 | 0.00002 | 0 | 0.919041 | 0.080938 | 0 | 0 | 0 | 0 | 0 |
| 927 | 0.00001 | 0 | 0.913486 | 0.086504 | 0 | 0 | 0 | 0 | 0 |
| 928 | 0 | 0 | 0.916775 | 0.083225 | 0 | 0 | 0 | 0 | 0 |
| 929 | 0 | 0.00002 | 0.911658 | 0.088322 | 0 | 0 | 0 | 0 | 0 |
| 930 | 0 | 0 | 0.9172 | 0.0828 | 0 | 0 | 0 | 0 | 0 |
| 931 | 0 | 0.00001 | 0.919407 | 0.080583 | 0 | 0 | 0 | 0 | 0 |
| 932 | 0.00001 | 0.00001 | 0.918922 | 0.081058 | 0 | 0 | 0 | 0 | 0 |
| 933 | 0.00001 | 0 | 0.920124 | 0.079866 | 0 | 0 | 0 | 0 | 0 |
| 934 | 0.00001 | 0 | 0.91349 | 0.0865 | 0 | 0 | 0 | 0 | 0 |
| 935 | 0.00001 | 0 | 0.917777 | 0.082213 | 0 | 0 | 0 | 0 | 0 |
| 936 | 0.00001 | 0.00002 | 0.917432 | 0.082538 | 0 | 0 | 0 | 0 | 0 |
| 937 | 0 | 0 | 0.919946 | 0.080054 | 0 | 0 | 0 | 0 | 0 |
| 938 | 0 | 0.00001 | 0.917822 | 0.082168 | 0 | 0 | 0 | 0 | 0 |
| 939 | 0 | 0 | 0.919368 | 0.080632 | 0 | 0 | 0 | 0 | 0 |
| 940 | 0.00002 | 0 | 0.91258 | 0.0874 | 0 | 0 | 0 | 0 | 0 |
| 941 | 0.00003 | 0.00001 | 0.917339 | 0.082621 | 0 | 0 | 0 | 0 | 0 |
| 942 | 0.00002 | 0 | 0.918023 | 0.081957 | 0 | 0 | 0 | 0 | 0 |
| 943 | 0.00002 | 0 | 0.918689 | 0.081291 | 0 | 0 | 0 | 0 | 0 |
| 944 | 0 | 0 | 0.916897 | 0.083103 | 0 | 0 | 0 | 0 | 0 |
| 945 | 0.00002 | 0 | 0.911653 | 0.088327 | 0 | 0 | 0 | 0 | 0 |
| 946 | 0 | 0 | 0.915391 | 0.084609 | 0 | 0 | 0 | 0 | 0 |
| 947 | 0.00001 | 0 | 0.914267 | 0.085723 | 0 | 0 | 0 | 0 | 0 |
| 948 | 0.00003 | 0 | 0.915782 | 0.084188 | 0 | 0 | 0 | 0 | 0 |
| 949 | 0.00001 | 0 | 0.920146 | 0.079844 | 0 | 0 | 0 | 0 | 0 |
| 950 | 0 | 0 | 0.91908 | 0.08092 | 0 | 0 | 0 | 0 | 0 |
| 951 | 0.00001 | 0.00002 | 0.919358 | 0.080612 | 0 | 0 | 0 | 0 | 0 |
| 952 | 0 | 0.00001 | 0.918025 | 0.081965 | 0 | 0 | 0 | 0 | 0 |
| 953 | 0.00001 | 0 | 0.915875 | 0.084115 | 0 | 0 | 0 | 0 | 0 |
| 954 | 0.00001 | 0.00001 | 0.912491 | 0.087489 | 0 | 0 | 0 | 0 | 0 |
| 955 | 0.00003 | 0 | 0.919882 | 0.080088 | 0 | 0 | 0 | 0 | 0 |
| 956 | 0.00001 | 0.00002 | 0.919311 | 0.080659 | 0 | 0 | 0 | 0 | 0 |
| 957 | 0.00001 | 0 | 0.91042 | 0.08957 | 0 | 0 | 0 | 0 | 0 |
| 958 | 0.00001 | 0 | 0.916279 | 0.083711 | 0 | 0 | 0 | 0 | 0 |
| 959 | 0 | 0.00001 | 0.914183 | 0.085807 | 0 | 0 | 0 | 0 | 0 |
| 960 | 0.00002 | 0.00001 | 0.919707 | 0.080263 | 0 | 0 | 0 | 0 | 0 |
| 961 | 0.00001 | 0.00002 | 0.915941 | 0.08403 | 0 | 0 | 0 | 0 | 0 |
| 962 | 0.00001 | 0 | 0.917094 | 0.082896 | 0 | 0 | 0 | 0 | 0 |
| 963 | 0.00001 | 0 | 0.915211 | 0.084779 | 0 | 0 | 0 | 0 | 0 |
| 964 | 0.00002 | 0 | 0.917535 | 0.082445 | 0 | 0 | 0 | 0 | 0 |
| 965 | 0.00001 | 0 | 0.919268 | 0.080722 | 0 | 0 | 0 | 0 | 0 |
| 966 | 0.00001 | 0 | 0.918794 | 0.081196 | 0 | 0 | 0 | 0 | 0 |
| 967 | 0 | 0.00002 | 0.920453 | 0.079527 | 0 | 0 | 0 | 0 | 0 |
| 968 | 0.00001 | 0 | 0.915096 | 0.084894 | 0 | 0 | 0 | 0 | 0 |
| 969 | 0.00001 | 0.00007 | 0.914521 | 0.085399 | 0 | 0 | 0 | 0 | 0 |
| 970 | 0.00004 | 0.00001 | 0.913171 | 0.086779 | 0 | 0 | 0 | 0 | 0 |
| 971 | 0.00004 | 0.00002 | 0.919184 | 0.080756 | 0 | 0 | 0 | 0 | 0 |
| 972 | 0 | 0 | 0.914166 | 0.085834 | 0 | 0 | 0 | 0 | 0 |
| 973 | 0 | 0.00002 | 0.916211 | 0.083769 | 0 | 0 | 0 | 0 | 0 |
| 974 | 0.00001 | 0 | 0.914624 | 0.085366 | 0 | 0 | 0 | 0 | 0 |
| 975 | 0 | 0.00002 | 0.919782 | 0.080198 | 0 | 0 | 0 | 0 | 0 |
| 976 | 0 | 0.00002 | 0.916944 | 0.083036 | 0 | 0 | 0 | 0 | 0 |
| 977 | 0.00001 | 0.00001 | 0.914971 | 0.085009 | 0 | 0 | 0 | 0 | 0 |
| 978 | 0.00001 | 0.00001 | 0.91659 | 0.08339 | 0 | 0 | 0 | 0 | 0 |
| 979 | 0.00001 | 0 | 0.914994 | 0.084996 | 0 | 0 | 0 | 0 | 0 |
| 980 | 0.00001 | 0.00002 | 0.916013 | 0.083957 | 0 | 0 | 0 | 0 | 0 |
| 981 | 0 | 0 | 0.918233 | 0.081767 | 0 | 0 | 0 | 0 | 0 |
| 982 | 0.00001 | 0.00002 | 0.913041 | 0.086929 | 0 | 0 | 0 | 0 | 0 |
| 983 | 0 | 0 | 0.913023 | 0.086977 | 0 | 0 | 0 | 0 | 0 |
| 984 | 0 | 0.00001 | 0.916482 | 0.083508 | 0 | 0 | 0 | 0 | 0 |
| 985 | 0.00001 | 0 | 0.916606 | 0.083384 | 0 | 0 | 0 | 0 | 0 |
| 986 | 0.00002 | 0 | 0.918701 | 0.081279 | 0 | 0 | 0 | 0 | 0 |
| 987 | 0 | 0.00001 | 0.919431 | 0.080559 | 0 | 0 | 0 | 0 | 0 |
| 988 | 0.00002 | 0 | 0.91762 | 0.08236 | 0 | 0 | 0 | 0 | 0 |
| 989 | 0.00003 | 0.00003 | 0.913667 | 0.086273 | 0 | 0 | 0 | 0 | 0 |
| 990 | 0 | 0.00006 | 0.912825 | 0.087115 | 0 | 0 | 0 | 0 | 0 |
| 991 | 0 | 0.00001 | 0.918221 | 0.081769 | 0 | 0 | 0 | 0 | 0 |
| 992 | 0.00001 | 0.00002 | 0.919516 | 0.080454 | 0 | 0 | 0 | 0 | 0 |
| 993 | 0.00001 | 0 | 0.919965 | 0.080025 | 0 | 0 | 0 | 0 | 0 |
| 994 | 0 | 0.00002 | 0.919423 | 0.080557 | 0 | 0 | 0 | 0 | 0 |
| 995 | 0 | 0.00001 | 0.914063 | 0.085927 | 0 | 0 | 0 | 0 | 0 |
| 996 | 0.00001 | 0 | 0.919757 | 0.080233 | 0 | 0 | 0 | 0 | 0 |
| 997 | 0 | 0 | 0.912576 | 0.087424 | 0 | 0 | 0 | 0 | 0 |
| 998 | 0.00002 | 0 | 0.916307 | 0.083673 | 0 | 0 | 0 | 0 | 0 |
| 999 | 0.00003 | 0.00002 | 0.920372 | 0.079578 | 0 | 0 | 0 | 0 | 0 |
| 1000 | 0 | 0.00003 | 0.914564 | 0.085406 | 0 | 0 | 0 | 0 | 0 |

| Year 11 |  |  |  |  |  |  |  |  |  |
| --- | --- | --- | --- | --- | --- | --- | --- | --- | --- |
| Trial/  Race | 003.0 | 005.0 | 007.0 | 037.1 | 043.0 | 303.0 | 003.2 | 403.0 | 003.4 |
| 1 | 0.00001 | 0.00001 | 0.922724 | 0.077256 | 0 | 0 | 0 | 0 | 0 |
| 2 | 0.00002 | 0 | 0.918387 | 0.081593 | 0 | 0 | 0 | 0 | 0 |
| 3 | 0 | 0 | 0.925558 | 0.074442 | 0 | 0 | 0 | 0 | 0 |
| 4 | 0 | 0.00001 | 0.922639 | 0.077351 | 0 | 0 | 0 | 0 | 0 |
| 5 | 0 | 0 | 0.920774 | 0.079226 | 0 | 0 | 0 | 0 | 0 |
| 6 | 0 | 0 | 0.923065 | 0.076935 | 0 | 0 | 0 | 0 | 0 |
| 7 | 0.00002 | 0 | 0.921829 | 0.078151 | 0 | 0 | 0 | 0 | 0 |
| 8 | 0 | 0 | 0.924546 | 0.075454 | 0 | 0 | 0 | 0 | 0 |
| 9 | 0 | 0 | 0.919431 | 0.080569 | 0 | 0 | 0 | 0 | 0 |
| 10 | 0.00003 | 0.00003 | 0.92185 | 0.07809 | 0 | 0 | 0 | 0 | 0 |
| 11 | 0.00001 | 0 | 0.925117 | 0.074873 | 0 | 0 | 0 | 0 | 0 |
| 12 | 0 | 0 | 0.919573 | 0.080427 | 0 | 0 | 0 | 0 | 0 |
| 13 | 0 | 0 | 0.922994 | 0.077006 | 0 | 0 | 0 | 0 | 0 |
| 14 | 0.00001 | 0 | 0.920731 | 0.079259 | 0 | 0 | 0 | 0 | 0 |
| 15 | 0.00003 | 0.00001 | 0.917956 | 0.082004 | 0 | 0 | 0 | 0 | 0 |
| 16 | 0.00002 | 0 | 0.92211 | 0.07787 | 0 | 0 | 0 | 0 | 0 |
| 17 | 0.00001 | 0 | 0.923255 | 0.076735 | 0 | 0 | 0 | 0 | 0 |
| 18 | 0 | 0 | 0.922021 | 0.077979 | 0 | 0 | 0 | 0 | 0 |
| 19 | 0.00002 | 0.00001 | 0.922284 | 0.077686 | 0 | 0 | 0 | 0 | 0 |
| 20 | 0.00002 | 0 | 0.921992 | 0.077988 | 0 | 0 | 0 | 0 | 0 |
| 21 | 0.00001 | 0.00001 | 0.923762 | 0.076218 | 0 | 0 | 0 | 0 | 0 |
| 22 | 0.00001 | 0.00001 | 0.92014 | 0.07984 | 0 | 0 | 0 | 0 | 0 |
| 23 | 0 | 0.00002 | 0.924195 | 0.075785 | 0 | 0 | 0 | 0 | 0 |
| 24 | 0.00001 | 0 | 0.923409 | 0.076581 | 0 | 0 | 0 | 0 | 0 |
| 25 | 0.00001 | 0 | 0.921529 | 0.078461 | 0 | 0 | 0 | 0 | 0 |
| 26 | 0 | 0 | 0.925542 | 0.074458 | 0 | 0 | 0 | 0 | 0 |
| 27 | 0 | 0 | 0.921447 | 0.078553 | 0 | 0 | 0 | 0 | 0 |
| 28 | 0.00001 | 0 | 0.924342 | 0.075429 | 0 | 0 | 0 | 0 | 0 |
| 29 | 0.00001 | 0.00002 | 0.92443 | 0.07554 | 0 | 0 | 0 | 0 | 0 |
| 30 | 0.00001 | 0.00001 | 0.920478 | 0.079502 | 0 | 0 | 0 | 0 | 0 |
| 31 | 0.00003 | 0.00003 | 0.924989 | 0.074951 | 0 | 0 | 0 | 0 | 0 |
| 32 | 0.00004 | 0 | 0.923759 | 0.076201 | 0 | 0 | 0 | 0 | 0 |
| 33 | 0.00001 | 0 | 0.920739 | 0.079251 | 0 | 0 | 0 | 0 | 0 |
| 34 | 0.00002 | 0 | 0.923458 | 0.076522 | 0 | 0 | 0 | 0 | 0 |
| 35 | 0.00001 | 0 | 0.918292 | 0.081698 | 0 | 0 | 0 | 0 | 0 |
| 36 | 0.00002 | 0 | 0.920353 | 0.079627 | 0 | 0 | 0 | 0 | 0 |
| 37 | 0 | 0 | 0.92806 | 0.07194 | 0 | 0 | 0 | 0 | 0 |
| 38 | 0.00001 | 0 | 0.923999 | 0.075991 | 0 | 0 | 0 | 0 | 0 |
| 39 | 0.00002 | 0 | 0.922607 | 0.077373 | 0 | 0 | 0 | 0 | 0 |
| 40 | 0 | 0 | 0.919134 | 0.080866 | 0 | 0 | 0 | 0 | 0 |
| 41 | 0.00001 | 0.00001 | 0.919852 | 0.080128 | 0 | 0 | 0 | 0 | 0 |
| 42 | 0 | 0.00002 | 0.9245 | 0.07548 | 0 | 0 | 0 | 0 | 0 |
| 43 | 0.00002 | 0 | 0.920984 | 0.078996 | 0 | 0 | 0 | 0 | 0 |
| 44 | 0 | 0.00001 | 0.923173 | 0.076817 | 0 | 0 | 0 | 0 | 0 |
| 45 | 0.00001 | 0 | 0.92242 | 0.07757 | 0 | 0 | 0 | 0 | 0 |
| 46 | 0 | 0 | 0.9194 | 0.0806 | 0 | 0 | 0 | 0 | 0 |
| 47 | 0.00001 | 0 | 0.923189 | 0.076801 | 0 | 0 | 0 | 0 | 0 |
| 48 | 0 | 0 | 0.921047 | 0.078953 | 0 | 0 | 0 | 0 | 0 |
| 49 | 0.00003 | 0.00003 | 0.92448 | 0.07546 | 0 | 0 | 0 | 0 | 0 |
| 50 | 0 | 0.00004 | 0.925408 | 0.074552 | 0 | 0 | 0 | 0 | 0 |
| 51 | 0.00004 | 0.00002 | 0.920858 | 0.079082 | 0 | 0 | 0 | 0 | 0 |
| 52 | 0.00002 | 0.00002 | 0.924811 | 0.075149 | 0 | 0 | 0 | 0 | 0 |
| 53 | 0.00002 | 0 | 0.922857 | 0.077123 | 0 | 0 | 0 | 0 | 0 |
| 54 | 0.00002 | 0 | 0.921374 | 0.078606 | 0 | 0 | 0 | 0 | 0 |
| 55 | 0.00001 | 0.00002 | 0.919542 | 0.080427 | 0 | 0 | 0 | 0 | 0 |
| 56 | 0.00001 | 0 | 0.921984 | 0.078006 | 0 | 0 | 0 | 0 | 0 |
| 57 | 0.00003 | 0.00001 | 0.923176 | 0.076784 | 0 | 0 | 0 | 0 | 0 |
| 58 | 0.00001 | 0 | 0.920495 | 0.079495 | 0 | 0 | 0 | 0 | 0 |
| 59 | 0.00001 | 0 | 0.926853 | 0.073137 | 0 | 0 | 0 | 0 | 0 |
| 60 | 0.00001 | 0 | 0.923416 | 0.076574 | 0 | 0 | 0 | 0 | 0 |
| 61 | 0.00001 | 0 | 0.91805 | 0.08194 | 0 | 0 | 0 | 0 | 0 |
| 62 | 0.00002 | 0 | 0.921188 | 0.078793 | 0 | 0 | 0 | 0 | 0 |
| 63 | 0.00003 | 0 | 0.921074 | 0.078896 | 0 | 0 | 0 | 0 | 0 |
| 64 | 0.00001 | 0 | 0.919433 | 0.080557 | 0 | 0 | 0 | 0 | 0 |
| 65 | 0.00002 | 0.00001 | 0.924648 | 0.075322 | 0 | 0 | 0 | 0 | 0 |
| 66 | 0.00004 | 0 | 0.926784 | 0.073176 | 0 | 0 | 0 | 0 | 0 |
| 67 | 0 | 0 | 0.925314 | 0.074686 | 0 | 0 | 0 | 0 | 0 |
| 68 | 0.00001 | 0 | 0.920693 | 0.079297 | 0 | 0 | 0 | 0 | 0 |
| 69 | 0 | 0 | 0.923205 | 0.076795 | 0 | 0 | 0 | 0 | 0 |
| 70 | 0.00001 | 0 | 0.923534 | 0.076456 | 0 | 0 | 0 | 0 | 0 |
| 71 | 0 | 0.00002 | 0.92028 | 0.079074 | 0 | 0 | 0 | 0 | 0 |
| 72 | 0.00002 | 0.00001 | 0.925881 | 0.074089 | 0 | 0 | 0 | 0 | 0 |
| 73 | 0 | 0 | 0.920131 | 0.079869 | 0 | 0 | 0 | 0 | 0 |
| 74 | 0.00002 | 0.00002 | 0.926483 | 0.073478 | 0 | 0 | 0 | 0 | 0 |
| 75 | 0.00003 | 0.00003 | 0.921339 | 0.078601 | 0 | 0 | 0 | 0 | 0 |
| 76 | 0 | 0 | 0.925441 | 0.074559 | 0 | 0 | 0 | 0 | 0 |
| 77 | 0.00002 | 0 | 0.922416 | 0.077564 | 0 | 0 | 0 | 0 | 0 |
| 78 | 0.00001 | 0 | 0.922184 | 0.077806 | 0 | 0 | 0 | 0 | 0 |
| 79 | 0.00003 | 0 | 0.924444 | 0.075526 | 0 | 0 | 0 | 0 | 0 |
| 80 | 0.00001 | 0 | 0.923573 | 0.076417 | 0 | 0 | 0 | 0 | 0 |
| 81 | 0.00001 | 0.00002 | 0.921545 | 0.078425 | 0 | 0 | 0 | 0 | 0 |
| 82 | 0 | 0 | 0.917218 | 0.082782 | 0 | 0 | 0 | 0 | 0 |
| 83 | 0.00004 | 0 | 0.925706 | 0.074254 | 0 | 0 | 0 | 0 | 0 |
| 84 | 0.00001 | 0 | 0.923614 | 0.076376 | 0 | 0 | 0 | 0 | 0 |
| 85 | 0.00001 | 0 | 0.920582 | 0.079408 | 0 | 0 | 0 | 0 | 0 |
| 86 | 0.00001 | 0 | 0.930898 | 0.069092 | 0 | 0 | 0 | 0 | 0 |
| 87 | 0.00004 | 0 | 0.922256 | 0.077704 | 0 | 0 | 0 | 0 | 0 |
| 88 | 0.00001 | 0 | 0.92145 | 0.07854 | 0 | 0 | 0 | 0 | 0 |
| 89 | 0.00002 | 0 | 0.921144 | 0.078836 | 0 | 0 | 0 | 0 | 0 |
| 90 | 0.00002 | 0 | 0.920915 | 0.079065 | 0 | 0 | 0 | 0 | 0 |
| 91 | 0 | 0 | 0.92109 | 0.07891 | 0 | 0 | 0 | 0 | 0 |
| 92 | 0.00002 | 0 | 0.923055 | 0.076925 | 0 | 0 | 0 | 0 | 0 |
| 93 | 0.00001 | 0.00001 | 0.925893 | 0.074087 | 0 | 0 | 0 | 0 | 0 |
| 94 | 0 | 0.00002 | 0.924266 | 0.075714 | 0 | 0 | 0 | 0 | 0 |
| 95 | 0.00002 | 0.00001 | 0.922229 | 0.077742 | 0 | 0 | 0 | 0 | 0 |
| 96 | 0 | 0 | 0.923853 | 0.076147 | 0 | 0 | 0 | 0 | 0 |
| 97 | 0.00003 | 0 | 0.921412 | 0.078558 | 0 | 0 | 0 | 0 | 0 |
| 98 | 0.00001 | 0 | 0.92017 | 0.07982 | 0 | 0 | 0 | 0 | 0 |
| 99 | 0.00003 | 0 | 0.91828 | 0.08169 | 0 | 0 | 0 | 0 | 0 |
| 100 | 0.00001 | 0 | 0.925212 | 0.074778 | 0 | 0 | 0 | 0 | 0 |
| 101 | 0 | 0.00001 | 0.924496 | 0.075494 | 0 | 0 | 0 | 0 | 0 |
| 102 | 0 | 0 | 0.924914 | 0.075086 | 0 | 0 | 0 | 0 | 0 |
| 103 | 0.00001 | 0.00001 | 0.92275 | 0.07723 | 0 | 0 | 0 | 0 | 0 |
| 104 | 0.00001 | 0 | 0.925843 | 0.074147 | 0 | 0 | 0 | 0 | 0 |
| 105 | 0.00002 | 0 | 0.922231 | 0.077749 | 0 | 0 | 0 | 0 | 0 |
| 106 | 0.00001 | 0.00002 | 0.922146 | 0.077824 | 0 | 0 | 0 | 0 | 0 |
| 107 | 0.00002 | 0.00001 | 0.92511 | 0.074541 | 0 | 0 | 0 | 0 | 0 |
| 108 | 0 | 0 | 0.916901 | 0.083099 | 0 | 0 | 0 | 0 | 0 |
| 109 | 0.00002 | 0.00004 | 0.923689 | 0.076251 | 0 | 0 | 0 | 0 | 0 |
| 110 | 0.00002 | 0 | 0.921875 | 0.078105 | 0 | 0 | 0 | 0 | 0 |
| 111 | 0.00001 | 0.00001 | 0.924794 | 0.075186 | 0 | 0 | 0 | 0 | 0 |
| 112 | 0.00002 | 0 | 0.921302 | 0.078678 | 0 | 0 | 0 | 0 | 0 |
| 113 | 0 | 0 | 0.922927 | 0.077073 | 0 | 0 | 0 | 0 | 0 |
| 114 | 0.00001 | 0 | 0.922474 | 0.077516 | 0 | 0 | 0 | 0 | 0 |
| 115 | 0.00002 | 0 | 0.925628 | 0.074352 | 0 | 0 | 0 | 0 | 0 |
| 116 | 0.00002 | 0.00002 | 0.921631 | 0.078329 | 0 | 0 | 0 | 0 | 0 |
| 117 | 0 | 0 | 0.922061 | 0.077939 | 0 | 0 | 0 | 0 | 0 |
| 118 | 0 | 0 | 0.92378 | 0.07622 | 0 | 0 | 0 | 0 | 0 |
| 119 | 0 | 0 | 0.920608 | 0.079392 | 0 | 0 | 0 | 0 | 0 |
| 120 | 0 | 0 | 0.920904 | 0.079096 | 0 | 0 | 0 | 0 | 0 |
| 121 | 0 | 0 | 0.923201 | 0.076799 | 0 | 0 | 0 | 0 | 0 |
| 122 | 0.00001 | 0 | 0.926131 | 0.073859 | 0 | 0 | 0 | 0 | 0 |
| 123 | 0.00003 | 0 | 0.924023 | 0.075947 | 0 | 0 | 0 | 0 | 0 |
| 124 | 0.00001 | 0 | 0.924804 | 0.075186 | 0 | 0 | 0 | 0 | 0 |
| 125 | 0.00002 | 0 | 0.922287 | 0.077693 | 0 | 0 | 0 | 0 | 0 |
| 126 | 0.00002 | 0 | 0.922222 | 0.077758 | 0 | 0 | 0 | 0 | 0 |
| 127 | 0.00003 | 0 | 0.922189 | 0.077781 | 0 | 0 | 0 | 0 | 0 |
| 128 | 0.00002 | 0 | 0.922517 | 0.077463 | 0 | 0 | 0 | 0 | 0 |
| 129 | 0 | 0 | 0.921819 | 0.078181 | 0 | 0 | 0 | 0 | 0 |
| 130 | 0 | 0 | 0.923739 | 0.076261 | 0 | 0 | 0 | 0 | 0 |
| 131 | 0 | 0 | 0.92266 | 0.07734 | 0 | 0 | 0 | 0 | 0 |
| 132 | 0.00001 | 0 | 0.922283 | 0.077707 | 0 | 0 | 0 | 0 | 0 |
| 133 | 0 | 0 | 0.920198 | 0.079802 | 0 | 0 | 0 | 0 | 0 |
| 134 | 0.00001 | 0.00001 | 0.924748 | 0.075232 | 0 | 0 | 0 | 0 | 0 |
| 135 | 0 | 0 | 0.918427 | 0.081573 | 0 | 0 | 0 | 0 | 0 |
| 136 | 0.00003 | 0.00002 | 0.925296 | 0.074654 | 0 | 0 | 0 | 0 | 0 |
| 137 | 0.00001 | 0 | 0.922228 | 0.077762 | 0 | 0 | 0 | 0 | 0 |
| 138 | 0.00002 | 0.00001 | 0.920811 | 0.079159 | 0 | 0 | 0 | 0 | 0 |
| 139 | 0.00002 | 0 | 0.9248 | 0.07518 | 0 | 0 | 0 | 0 | 0 |
| 140 | 0.00001 | 0 | 0.920572 | 0.079418 | 0 | 0 | 0 | 0 | 0 |
| 141 | 0.00001 | 0 | 0.920906 | 0.079084 | 0 | 0 | 0 | 0 | 0 |
| 142 | 0 | 0 | 0.918542 | 0.081458 | 0 | 0 | 0 | 0 | 0 |
| 143 | 0.00001 | 0 | 0.92192 | 0.07807 | 0 | 0 | 0 | 0 | 0 |
| 144 | 0.00001 | 0.00001 | 0.920277 | 0.079703 | 0 | 0 | 0 | 0 | 0 |
| 145 | 0.00001 | 0 | 0.923793 | 0.076197 | 0 | 0 | 0 | 0 | 0 |
| 146 | 0.00002 | 0 | 0.927697 | 0.072284 | 0 | 0 | 0 | 0 | 0 |
| 147 | 0.00001 | 0 | 0.920861 | 0.079129 | 0 | 0 | 0 | 0 | 0 |
| 148 | 0 | 0.00002 | 0.922729 | 0.07725 | 0 | 0 | 0 | 0 | 0 |
| 149 | 0 | 0 | 0.924527 | 0.075473 | 0 | 0 | 0 | 0 | 0 |
| 150 | 0.00003 | 0 | 0.926597 | 0.073373 | 0 | 0 | 0 | 0 | 0 |
| 151 | 0.00001 | 0 | 0.923163 | 0.076827 | 0 | 0 | 0 | 0 | 0 |
| 152 | 0 | 0 | 0.920122 | 0.079878 | 0 | 0 | 0 | 0 | 0 |
| 153 | 0.00001 | 0 | 0.925565 | 0.074425 | 0 | 0 | 0 | 0 | 0 |
| 154 | 0.00001 | 0 | 0.922164 | 0.077825 | 0 | 0 | 0 | 0 | 0 |
| 155 | 0.00001 | 0 | 0.922706 | 0.077284 | 0 | 0 | 0 | 0 | 0 |
| 156 | 0.00002 | 0 | 0.924074 | 0.075906 | 0 | 0 | 0 | 0 | 0 |
| 157 | 0.00003 | 0 | 0.920526 | 0.079445 | 0 | 0 | 0 | 0 | 0 |
| 158 | 0.00002 | 0 | 0.92112 | 0.07886 | 0 | 0 | 0 | 0 | 0 |
| 159 | 0 | 0 | 0.921222 | 0.078778 | 0 | 0 | 0 | 0 | 0 |
| 160 | 0.00001 | 0 | 0.925128 | 0.074862 | 0 | 0 | 0 | 0 | 0 |
| 161 | 0.00003 | 0 | 0.921362 | 0.078608 | 0 | 0 | 0 | 0 | 0 |
| 162 | 0 | 0 | 0.922849 | 0.077151 | 0 | 0 | 0 | 0 | 0 |
| 163 | 0 | 0 | 0.921313 | 0.078687 | 0 | 0 | 0 | 0 | 0 |
| 164 | 0.00002 | 0 | 0.914841 | 0.085139 | 0 | 0 | 0 | 0 | 0 |
| 165 | 0.00003 | 0 | 0.928114 | 0.071857 | 0 | 0 | 0 | 0 | 0 |
| 166 | 0.00001 | 0 | 0.923726 | 0.076264 | 0 | 0 | 0 | 0 | 0 |
| 167 | 0.00001 | 0 | 0.921632 | 0.078358 | 0 | 0 | 0 | 0 | 0 |
| 168 | 0.00002 | 0 | 0.921389 | 0.078591 | 0 | 0 | 0 | 0 | 0 |
| 169 | 0.00002 | 0 | 0.918514 | 0.081466 | 0 | 0 | 0 | 0 | 0 |
| 170 | 0.00002 | 0 | 0.923248 | 0.076732 | 0 | 0 | 0 | 0 | 0 |
| 171 | 0.00001 | 0.00002 | 0.923499 | 0.076471 | 0 | 0 | 0 | 0 | 0 |
| 172 | 0.00001 | 0.00001 | 0.92038 | 0.0796 | 0 | 0 | 0 | 0 | 0 |
| 173 | 0.00002 | 0.00002 | 0.924526 | 0.075434 | 0 | 0 | 0 | 0 | 0 |
| 174 | 0.00001 | 0 | 0.919562 | 0.080428 | 0 | 0 | 0 | 0 | 0 |
| 175 | 0.00001 | 0 | 0.920184 | 0.079806 | 0 | 0 | 0 | 0 | 0 |
| 176 | 0.00005 | 0 | 0.926975 | 0.072975 | 0 | 0 | 0 | 0 | 0 |
| 177 | 0.00001 | 0 | 0.924318 | 0.075672 | 0 | 0 | 0 | 0 | 0 |
| 178 | 0.00002 | 0.00003 | 0.923385 | 0.076565 | 0 | 0 | 0 | 0 | 0 |
| 179 | 0 | 0 | 0.92265 | 0.07735 | 0 | 0 | 0 | 0 | 0 |
| 180 | 0.00001 | 0 | 0.922173 | 0.077817 | 0 | 0 | 0 | 0 | 0 |
| 181 | 0 | 0 | 0.925496 | 0.074504 | 0 | 0 | 0 | 0 | 0 |
| 182 | 0.00002 | 0.00004 | 0.920553 | 0.079388 | 0 | 0 | 0 | 0 | 0 |
| 183 | 0.00001 | 0 | 0.925993 | 0.073997 | 0 | 0 | 0 | 0 | 0 |
| 184 | 0.00001 | 0 | 0.923811 | 0.076179 | 0 | 0 | 0 | 0 | 0 |
| 185 | 0 | 0 | 0.922675 | 0.077325 | 0 | 0 | 0 | 0 | 0 |
| 186 | 0.00001 | 0 | 0.924066 | 0.075924 | 0 | 0 | 0 | 0 | 0 |
| 187 | 0.00001 | 0 | 0.929033 | 0.070958 | 0 | 0 | 0 | 0 | 0 |
| 188 | 0.00003 | 0 | 0.922346 | 0.077625 | 0 | 0 | 0 | 0 | 0 |
| 189 | 0.00001 | 0.00003 | 0.920437 | 0.079523 | 0 | 0 | 0 | 0 | 0 |
| 190 | 0.00002 | 0.00002 | 0.924201 | 0.075759 | 0 | 0 | 0 | 0 | 0 |
| 191 | 0.00002 | 0.00001 | 0.922848 | 0.077122 | 0 | 0 | 0 | 0 | 0 |
| 192 | 0.00001 | 0.00001 | 0.923567 | 0.076413 | 0 | 0 | 0 | 0 | 0 |
| 193 | 0.00001 | 0 | 0.917809 | 0.082181 | 0 | 0 | 0 | 0 | 0 |
| 194 | 0 | 0.00002 | 0.917321 | 0.082659 | 0 | 0 | 0 | 0 | 0 |
| 195 | 0.00001 | 0 | 0.921328 | 0.078662 | 0 | 0 | 0 | 0 | 0 |
| 196 | 0 | 0 | 0.922954 | 0.077046 | 0 | 0 | 0 | 0 | 0 |
| 197 | 0.00002 | 0 | 0.927357 | 0.072623 | 0 | 0 | 0 | 0 | 0 |
| 198 | 0 | 0 | 0.92342 | 0.076149 | 0 | 0 | 0 | 0 | 0 |
| 199 | 0.00001 | 0 | 0.92635 | 0.07364 | 0 | 0 | 0 | 0 | 0 |
| 200 | 0 | 0 | 0.919641 | 0.080359 | 0 | 0 | 0 | 0 | 0 |
| 201 | 0.00002 | 0 | 0.923592 | 0.076388 | 0 | 0 | 0 | 0 | 0 |
| 202 | 0.00002 | 0.00002 | 0.924712 | 0.075248 | 0 | 0 | 0 | 0 | 0 |
| 203 | 0 | 0 | 0.925158 | 0.074842 | 0 | 0 | 0 | 0 | 0 |
| 204 | 0.00002 | 0.00002 | 0.922636 | 0.077324 | 0 | 0 | 0 | 0 | 0 |
| 205 | 0.00003 | 0 | 0.920006 | 0.079965 | 0 | 0 | 0 | 0 | 0 |
| 206 | 0 | 0 | 0.917723 | 0.082277 | 0 | 0 | 0 | 0 | 0 |
| 207 | 0.00001 | 0 | 0.926282 | 0.073708 | 0 | 0 | 0 | 0 | 0 |
| 208 | 0.00002 | 0 | 0.925954 | 0.074026 | 0 | 0 | 0 | 0 | 0 |
| 209 | 0 | 0 | 0.921814 | 0.078186 | 0 | 0 | 0 | 0 | 0 |
| 210 | 0.00003 | 0.00001 | 0.924238 | 0.075722 | 0 | 0 | 0 | 0 | 0 |
| 211 | 0 | 0 | 0.921394 | 0.078606 | 0 | 0 | 0 | 0 | 0 |
| 212 | 0.00001 | 0 | 0.920257 | 0.079733 | 0 | 0 | 0 | 0 | 0 |
| 213 | 0 | 0 | 0.923094 | 0.076906 | 0 | 0 | 0 | 0 | 0 |
| 214 | 0.00001 | 0.00005 | 0.922844 | 0.077096 | 0 | 0 | 0 | 0 | 0 |
| 215 | 0 | 0 | 0.921663 | 0.078337 | 0 | 0 | 0 | 0 | 0 |
| 216 | 0 | 0.00001 | 0.926484 | 0.073506 | 0 | 0 | 0 | 0 | 0 |
| 217 | 0.00003 | 0.00001 | 0.921064 | 0.078896 | 0 | 0 | 0 | 0 | 0 |
| 218 | 0 | 0 | 0.925568 | 0.074432 | 0 | 0 | 0 | 0 | 0 |
| 219 | 0 | 0 | 0.928736 | 0.071264 | 0 | 0 | 0 | 0 | 0 |
| 220 | 0.00002 | 0.00003 | 0.922594 | 0.077356 | 0 | 0 | 0 | 0 | 0 |
| 221 | 0.00004 | 0 | 0.923906 | 0.076054 | 0 | 0 | 0 | 0 | 0 |
| 222 | 0.00002 | 0 | 0.921985 | 0.077995 | 0 | 0 | 0 | 0 | 0 |
| 223 | 0.00001 | 0 | 0.922085 | 0.077905 | 0 | 0 | 0 | 0 | 0 |
| 224 | 0.00001 | 0.00001 | 0.921347 | 0.078633 | 0 | 0 | 0 | 0 | 0 |
| 225 | 0.00002 | 0 | 0.923532 | 0.076448 | 0 | 0 | 0 | 0 | 0 |
| 226 | 0.00002 | 0 | 0.919913 | 0.080067 | 0 | 0 | 0 | 0 | 0 |
| 227 | 0.00004 | 0 | 0.923618 | 0.076342 | 0 | 0 | 0 | 0 | 0 |
| 228 | 0 | 0.00001 | 0.924419 | 0.075571 | 0 | 0 | 0 | 0 | 0 |
| 229 | 0 | 0.00001 | 0.923698 | 0.076292 | 0 | 0 | 0 | 0 | 0 |
| 230 | 0.00002 | 0 | 0.921766 | 0.078214 | 0 | 0 | 0 | 0 | 0 |
| 231 | 0.00004 | 0.00003 | 0.924222 | 0.075708 | 0 | 0 | 0 | 0 | 0 |
| 232 | 0 | 0.00001 | 0.921951 | 0.078039 | 0 | 0 | 0 | 0 | 0 |
| 233 | 0.00001 | 0 | 0.921565 | 0.078426 | 0 | 0 | 0 | 0 | 0 |
| 234 | 0 | 0 | 0.922992 | 0.077008 | 0 | 0 | 0 | 0 | 0 |
| 235 | 0.00002 | 0 | 0.928348 | 0.071632 | 0 | 0 | 0 | 0 | 0 |
| 236 | 0.00004 | 0 | 0.917562 | 0.082397 | 0 | 0 | 0 | 0 | 0 |
| 237 | 0.00001 | 0 | 0.923207 | 0.076784 | 0 | 0 | 0 | 0 | 0 |
| 238 | 0 | 0 | 0.917062 | 0.082938 | 0 | 0 | 0 | 0 | 0 |
| 239 | 0.00002 | 0 | 0.918848 | 0.081132 | 0 | 0 | 0 | 0 | 0 |
| 240 | 0 | 0 | 0.92641 | 0.07359 | 0 | 0 | 0 | 0 | 0 |
| 241 | 0.00002 | 0.00002 | 0.925116 | 0.074843 | 0 | 0 | 0 | 0 | 0 |
| 242 | 0 | 0 | 0.9241 | 0.0759 | 0 | 0 | 0 | 0 | 0 |
| 243 | 0.00001 | 0.00001 | 0.927081 | 0.072899 | 0 | 0 | 0 | 0 | 0 |
| 244 | 0.00002 | 0 | 0.92229 | 0.07769 | 0 | 0 | 0 | 0 | 0 |
| 245 | 0.00001 | 0 | 0.920595 | 0.079255 | 0 | 0 | 0 | 0 | 0 |
| 246 | 0.00002 | 0.00002 | 0.919767 | 0.080193 | 0 | 0 | 0 | 0 | 0 |
| 247 | 0 | 0 | 0.921861 | 0.078139 | 0 | 0 | 0 | 0 | 0 |
| 248 | 0.00001 | 0 | 0.923674 | 0.076316 | 0 | 0 | 0 | 0 | 0 |
| 249 | 0.00001 | 0 | 0.926206 | 0.073784 | 0 | 0 | 0 | 0 | 0 |
| 250 | 0 | 0 | 0.919674 | 0.080326 | 0 | 0 | 0 | 0 | 0 |
| 251 | 0.00001 | 0.00003 | 0.920683 | 0.079278 | 0 | 0 | 0 | 0 | 0 |
| 252 | 0.00003 | 0 | 0.925343 | 0.074627 | 0 | 0 | 0 | 0 | 0 |
| 253 | 0.00002 | 0 | 0.924624 | 0.075356 | 0 | 0 | 0 | 0 | 0 |
| 254 | 0.00002 | 0 | 0.921806 | 0.078174 | 0 | 0 | 0 | 0 | 0 |
| 255 | 0 | 0 | 0.922683 | 0.077317 | 0 | 0 | 0 | 0 | 0 |
| 256 | 0.00001 | 0.00001 | 0.921054 | 0.078926 | 0 | 0 | 0 | 0 | 0 |
| 257 | 0 | 0 | 0.923244 | 0.076756 | 0 | 0 | 0 | 0 | 0 |
| 258 | 0 | 0 | 0.917183 | 0.082817 | 0 | 0 | 0 | 0 | 0 |
| 259 | 0 | 0 | 0.920562 | 0.079438 | 0 | 0 | 0 | 0 | 0 |
| 260 | 0 | 0 | 0.925965 | 0.074035 | 0 | 0 | 0 | 0 | 0 |
| 261 | 0.00003 | 0 | 0.926499 | 0.073471 | 0 | 0 | 0 | 0 | 0 |
| 262 | 0.00002 | 0.00001 | 0.922301 | 0.077669 | 0 | 0 | 0 | 0 | 0 |
| 263 | 0.00001 | 0 | 0.922064 | 0.077926 | 0 | 0 | 0 | 0 | 0 |
| 264 | 0.00002 | 0 | 0.919184 | 0.080796 | 0 | 0 | 0 | 0 | 0 |
| 265 | 0.00001 | 0 | 0.922739 | 0.077251 | 0 | 0 | 0 | 0 | 0 |
| 266 | 0.00001 | 0.00002 | 0.926521 | 0.073449 | 0 | 0 | 0 | 0 | 0 |
| 267 | 0.00002 | 0 | 0.924939 | 0.075041 | 0 | 0 | 0 | 0 | 0 |
| 268 | 0 | 0.00001 | 0.924571 | 0.075419 | 0 | 0 | 0 | 0 | 0 |
| 269 | 0 | 0 | 0.922923 | 0.077077 | 0 | 0 | 0 | 0 | 0 |
| 270 | 0 | 0 | 0.924108 | 0.075892 | 0 | 0 | 0 | 0 | 0 |
| 271 | 0 | 0 | 0.922451 | 0.077549 | 0 | 0 | 0 | 0 | 0 |
| 272 | 0 | 0 | 0.925465 | 0.074535 | 0 | 0 | 0 | 0 | 0 |
| 273 | 0 | 0 | 0.925297 | 0.074703 | 0 | 0 | 0 | 0 | 0 |
| 274 | 0 | 0 | 0.926863 | 0.073137 | 0 | 0 | 0 | 0 | 0 |
| 275 | 0 | 0 | 0.924461 | 0.075539 | 0 | 0 | 0 | 0 | 0 |
| 276 | 0.00003 | 0 | 0.922252 | 0.077718 | 0 | 0 | 0 | 0 | 0 |
| 277 | 0 | 0.00001 | 0.917236 | 0.082754 | 0 | 0 | 0 | 0 | 0 |
| 278 | 0 | 0 | 0.924445 | 0.075555 | 0 | 0 | 0 | 0 | 0 |
| 279 | 0.00002 | 0 | 0.922157 | 0.077823 | 0 | 0 | 0 | 0 | 0 |
| 280 | 0 | 0 | 0.921685 | 0.078315 | 0 | 0 | 0 | 0 | 0 |
| 281 | 0.00001 | 0.00001 | 0.919889 | 0.080091 | 0 | 0 | 0 | 0 | 0 |
| 282 | 0.00002 | 0 | 0.924242 | 0.075738 | 0 | 0 | 0 | 0 | 0 |
| 283 | 0.00003 | 0.00001 | 0.925179 | 0.07478 | 0 | 0 | 0 | 0 | 0 |
| 284 | 0.00001 | 0 | 0.925379 | 0.074611 | 0 | 0 | 0 | 0 | 0 |
| 285 | 0.00001 | 0 | 0.920063 | 0.079927 | 0 | 0 | 0 | 0 | 0 |
| 286 | 0.00001 | 0 | 0.926789 | 0.073201 | 0 | 0 | 0 | 0 | 0 |
| 287 | 0.00002 | 0 | 0.922143 | 0.077837 | 0 | 0 | 0 | 0 | 0 |
| 288 | 0 | 0 | 0.921079 | 0.078921 | 0 | 0 | 0 | 0 | 0 |
| 289 | 0.00001 | 0 | 0.923959 | 0.076031 | 0 | 0 | 0 | 0 | 0 |
| 290 | 0 | 0 | 0.920204 | 0.079796 | 0 | 0 | 0 | 0 | 0 |
| 291 | 0.00001 | 0.00001 | 0.920686 | 0.079294 | 0 | 0 | 0 | 0 | 0 |
| 292 | 0.00002 | 0 | 0.91922 | 0.08076 | 0 | 0 | 0 | 0 | 0 |
| 293 | 0.00001 | 0 | 0.919868 | 0.080122 | 0 | 0 | 0 | 0 | 0 |
| 294 | 0 | 0.00002 | 0.919218 | 0.080762 | 0 | 0 | 0 | 0 | 0 |
| 295 | 0 | 0 | 0.925656 | 0.074344 | 0 | 0 | 0 | 0 | 0 |
| 296 | 0 | 0 | 0.92315 | 0.07685 | 0 | 0 | 0 | 0 | 0 |
| 297 | 0.00002 | 0.00001 | 0.921101 | 0.078869 | 0 | 0 | 0 | 0 | 0 |
| 298 | 0 | 0 | 0.922965 | 0.077035 | 0 | 0 | 0 | 0 | 0 |
| 299 | 0.00001 | 0 | 0.917125 | 0.082865 | 0 | 0 | 0 | 0 | 0 |
| 300 | 0.00002 | 0 | 0.923119 | 0.076861 | 0 | 0 | 0 | 0 | 0 |
| 301 | 0.00002 | 0 | 0.921287 | 0.078693 | 0 | 0 | 0 | 0 | 0 |
| 302 | 0.00001 | 0 | 0.918889 | 0.081101 | 0 | 0 | 0 | 0 | 0 |
| 303 | 0.00002 | 0 | 0.922084 | 0.077896 | 0 | 0 | 0 | 0 | 0 |
| 304 | 0 | 0 | 0.920696 | 0.079304 | 0 | 0 | 0 | 0 | 0 |
| 305 | 0.00001 | 0 | 0.923987 | 0.076003 | 0 | 0 | 0 | 0 | 0 |
| 306 | 0 | 0 | 0.926187 | 0.073813 | 0 | 0 | 0 | 0 | 0 |
| 307 | 0 | 0.00001 | 0.924653 | 0.075337 | 0 | 0 | 0 | 0 | 0 |
| 308 | 0.00001 | 0.00001 | 0.9271 | 0.07288 | 0 | 0 | 0 | 0 | 0 |
| 309 | 0 | 0 | 0.925668 | 0.074332 | 0 | 0 | 0 | 0 | 0 |
| 310 | 0.00002 | 0.00002 | 0.921864 | 0.078097 | 0 | 0 | 0 | 0 | 0 |
| 311 | 0 | 0 | 0.922588 | 0.077412 | 0 | 0 | 0 | 0 | 0 |
| 312 | 0 | 0 | 0.921176 | 0.078824 | 0 | 0 | 0 | 0 | 0 |
| 313 | 0.00002 | 0 | 0.923549 | 0.076431 | 0 | 0 | 0 | 0 | 0 |
| 314 | 0 | 0 | 0.92319 | 0.07681 | 0 | 0 | 0 | 0 | 0 |
| 315 | 0.00002 | 0 | 0.921761 | 0.078219 | 0 | 0 | 0 | 0 | 0 |
| 316 | 0.00002 | 0 | 0.920772 | 0.079208 | 0 | 0 | 0 | 0 | 0 |
| 317 | 0.00003 | 0.00001 | 0.920909 | 0.079051 | 0 | 0 | 0 | 0 | 0 |
| 318 | 0.00001 | 0.00001 | 0.919621 | 0.080329 | 0 | 0 | 0 | 0 | 0 |
| 319 | 0.00002 | 0 | 0.92121 | 0.07877 | 0 | 0 | 0 | 0 | 0 |
| 320 | 0.00001 | 0.00001 | 0.923943 | 0.076037 | 0 | 0 | 0 | 0 | 0 |
| 321 | 0.00002 | 0 | 0.920681 | 0.079299 | 0 | 0 | 0 | 0 | 0 |
| 322 | 0.00002 | 0 | 0.922815 | 0.077165 | 0 | 0 | 0 | 0 | 0 |
| 323 | 0 | 0 | 0.922897 | 0.077103 | 0 | 0 | 0 | 0 | 0 |
| 324 | 0.00002 | 0 | 0.924986 | 0.074994 | 0 | 0 | 0 | 0 | 0 |
| 325 | 0.00001 | 0 | 0.924465 | 0.075525 | 0 | 0 | 0 | 0 | 0 |
| 326 | 0.00003 | 0.00002 | 0.923209 | 0.07674 | 0 | 0 | 0 | 0 | 0 |
| 327 | 0.00001 | 0 | 0.921507 | 0.078483 | 0 | 0 | 0 | 0 | 0 |
| 328 | 0.00003 | 0.00001 | 0.922491 | 0.077469 | 0 | 0 | 0 | 0 | 0 |
| 329 | 0.00001 | 0.00001 | 0.922292 | 0.077688 | 0 | 0 | 0 | 0 | 0 |
| 330 | 0.00001 | 0 | 0.923857 | 0.076133 | 0 | 0 | 0 | 0 | 0 |
| 331 | 0.00003 | 0.00001 | 0.922036 | 0.077924 | 0 | 0 | 0 | 0 | 0 |
| 332 | 0.00001 | 0 | 0.92229 | 0.0777 | 0 | 0 | 0 | 0 | 0 |
| 333 | 0 | 0 | 0.919245 | 0.080755 | 0 | 0 | 0 | 0 | 0 |
| 334 | 0.00002 | 0.00004 | 0.923559 | 0.076381 | 0 | 0 | 0 | 0 | 0 |
| 335 | 0.00001 | 0 | 0.923434 | 0.076556 | 0 | 0 | 0 | 0 | 0 |
| 336 | 0.00003 | 0 | 0.918675 | 0.081295 | 0 | 0 | 0 | 0 | 0 |
| 337 | 0.00001 | 0 | 0.924161 | 0.075829 | 0 | 0 | 0 | 0 | 0 |
| 338 | 0.00001 | 0 | 0.925413 | 0.074577 | 0 | 0 | 0 | 0 | 0 |
| 339 | 0.00001 | 0 | 0.920109 | 0.079881 | 0 | 0 | 0 | 0 | 0 |
| 340 | 0.00002 | 0.00002 | 0.920691 | 0.079269 | 0 | 0 | 0 | 0 | 0 |
| 341 | 0.00001 | 0 | 0.919306 | 0.080684 | 0 | 0 | 0 | 0 | 0 |
| 342 | 0 | 0 | 0.920095 | 0.079905 | 0 | 0 | 0 | 0 | 0 |
| 343 | 0.00002 | 0.00002 | 0.921197 | 0.078764 | 0 | 0 | 0 | 0 | 0 |
| 344 | 0.00003 | 0 | 0.921842 | 0.078128 | 0 | 0 | 0 | 0 | 0 |
| 345 | 0.00001 | 0 | 0.925662 | 0.074328 | 0 | 0 | 0 | 0 | 0 |
| 346 | 0.00002 | 0 | 0.927013 | 0.072967 | 0 | 0 | 0 | 0 | 0 |
| 347 | 0.00002 | 0.00002 | 0.918173 | 0.081787 | 0 | 0 | 0 | 0 | 0 |
| 348 | 0.00003 | 0 | 0.920892 | 0.079078 | 0 | 0 | 0 | 0 | 0 |
| 349 | 0.00003 | 0 | 0.923414 | 0.076556 | 0 | 0 | 0 | 0 | 0 |
| 350 | 0.00001 | 0.00001 | 0.923971 | 0.076009 | 0 | 0 | 0 | 0 | 0 |
| 351 | 0.00002 | 0 | 0.923194 | 0.076786 | 0 | 0 | 0 | 0 | 0 |
| 352 | 0.00001 | 0 | 0.924078 | 0.075912 | 0 | 0 | 0 | 0 | 0 |
| 353 | 0.00001 | 0 | 0.924769 | 0.075221 | 0 | 0 | 0 | 0 | 0 |
| 354 | 0.00002 | 0 | 0.921532 | 0.078448 | 0 | 0 | 0 | 0 | 0 |
| 355 | 0.00002 | 0 | 0.926185 | 0.073796 | 0 | 0 | 0 | 0 | 0 |
| 356 | 0.00001 | 0 | 0.922284 | 0.077706 | 0 | 0 | 0 | 0 | 0 |
| 357 | 0.00003 | 0 | 0.918368 | 0.081602 | 0 | 0 | 0 | 0 | 0 |
| 358 | 0.00002 | 0.00001 | 0.923509 | 0.076461 | 0 | 0 | 0 | 0 | 0 |
| 359 | 0.00002 | 0 | 0.921551 | 0.078429 | 0 | 0 | 0 | 0 | 0 |
| 360 | 0.00001 | 0 | 0.921635 | 0.078355 | 0 | 0 | 0 | 0 | 0 |
| 361 | 0 | 0 | 0.921729 | 0.078271 | 0 | 0 | 0 | 0 | 0 |
| 362 | 0 | 0 | 0.925699 | 0.074301 | 0 | 0 | 0 | 0 | 0 |
| 363 | 0.00001 | 0.00002 | 0.924058 | 0.075912 | 0 | 0 | 0 | 0 | 0 |
| 364 | 0.00001 | 0.00001 | 0.924706 | 0.075274 | 0 | 0 | 0 | 0 | 0 |
| 365 | 0.00001 | 0 | 0.922263 | 0.077727 | 0 | 0 | 0 | 0 | 0 |
| 366 | 0.00001 | 0 | 0.922801 | 0.077189 | 0 | 0 | 0 | 0 | 0 |
| 367 | 0.00002 | 0 | 0.920838 | 0.079142 | 0 | 0 | 0 | 0 | 0 |
| 368 | 0.00003 | 0.00002 | 0.925622 | 0.074329 | 0 | 0 | 0 | 0 | 0 |
| 369 | 0.00001 | 0 | 0.919574 | 0.080416 | 0 | 0 | 0 | 0 | 0 |
| 370 | 0.00001 | 0 | 0.923242 | 0.076749 | 0 | 0 | 0 | 0 | 0 |
| 371 | 0.00002 | 0.00001 | 0.922555 | 0.077266 | 0 | 0 | 0 | 0 | 0 |
| 372 | 0.00001 | 0 | 0.919369 | 0.080621 | 0 | 0 | 0 | 0 | 0 |
| 373 | 0 | 0.00002 | 0.919135 | 0.080845 | 0 | 0 | 0 | 0 | 0 |
| 374 | 0 | 0 | 0.923491 | 0.076509 | 0 | 0 | 0 | 0 | 0 |
| 375 | 0.00003 | 0 | 0.922231 | 0.077739 | 0 | 0 | 0 | 0 | 0 |
| 376 | 0.00002 | 0.00002 | 0.91812 | 0.08184 | 0 | 0 | 0 | 0 | 0 |
| 377 | 0 | 0 | 0.920209 | 0.079791 | 0 | 0 | 0 | 0 | 0 |
| 378 | 0.00002 | 0 | 0.925324 | 0.074656 | 0 | 0 | 0 | 0 | 0 |
| 379 | 0.00002 | 0 | 0.924429 | 0.075551 | 0 | 0 | 0 | 0 | 0 |
| 380 | 0.00001 | 0 | 0.9178 | 0.08219 | 0 | 0 | 0 | 0 | 0 |
| 381 | 0.00002 | 0.00002 | 0.922275 | 0.077685 | 0 | 0 | 0 | 0 | 0 |
| 382 | 0.00001 | 0 | 0.923858 | 0.076132 | 0 | 0 | 0 | 0 | 0 |
| 383 | 0.00004 | 0 | 0.92123 | 0.07873 | 0 | 0 | 0 | 0 | 0 |
| 384 | 0.00001 | 0 | 0.916871 | 0.083119 | 0 | 0 | 0 | 0 | 0 |
| 385 | 0 | 0.00002 | 0.921899 | 0.078081 | 0 | 0 | 0 | 0 | 0 |
| 386 | 0.00003 | 0.00001 | 0.921497 | 0.078463 | 0 | 0 | 0 | 0 | 0 |
| 387 | 0 | 0 | 0.922084 | 0.077916 | 0 | 0 | 0 | 0 | 0 |
| 388 | 0 | 0 | 0.925906 | 0.074094 | 0 | 0 | 0 | 0 | 0 |
| 389 | 0 | 0.00002 | 0.923375 | 0.076605 | 0 | 0 | 0 | 0 | 0 |
| 390 | 0.00003 | 0.00003 | 0.924173 | 0.075767 | 0 | 0 | 0 | 0 | 0 |
| 391 | 0.00001 | 0 | 0.918722 | 0.081268 | 0 | 0 | 0 | 0 | 0 |
| 392 | 0 | 0 | 0.922475 | 0.077525 | 0 | 0 | 0 | 0 | 0 |
| 393 | 0.00002 | 0 | 0.919248 | 0.080732 | 0 | 0 | 0 | 0 | 0 |
| 394 | 0 | 0 | 0.924588 | 0.075412 | 0 | 0 | 0 | 0 | 0 |
| 395 | 0.00002 | 0 | 0.924994 | 0.074986 | 0 | 0 | 0 | 0 | 0 |
| 396 | 0.00001 | 0 | 0.924721 | 0.075269 | 0 | 0 | 0 | 0 | 0 |
| 397 | 0 | 0 | 0.922848 | 0.077152 | 0 | 0 | 0 | 0 | 0 |
| 398 | 0.00003 | 0 | 0.926533 | 0.073437 | 0 | 0 | 0 | 0 | 0 |
| 399 | 0 | 0 | 0.924606 | 0.075394 | 0 | 0 | 0 | 0 | 0 |
| 400 | 0 | 0.00004 | 0.924921 | 0.075039 | 0 | 0 | 0 | 0 | 0 |
| 401 | 0.00004 | 0 | 0.921535 | 0.078425 | 0 | 0 | 0 | 0 | 0 |
| 402 | 0.00002 | 0 | 0.922525 | 0.077455 | 0 | 0 | 0 | 0 | 0 |
| 403 | 0.00001 | 0 | 0.923346 | 0.076644 | 0 | 0 | 0 | 0 | 0 |
| 404 | 0.00003 | 0 | 0.920477 | 0.079493 | 0 | 0 | 0 | 0 | 0 |
| 405 | 0 | 0 | 0.925147 | 0.074853 | 0 | 0 | 0 | 0 | 0 |
| 406 | 0 | 0.00001 | 0.920065 | 0.079925 | 0 | 0 | 0 | 0 | 0 |
| 407 | 0.00001 | 0 | 0.918836 | 0.081154 | 0 | 0 | 0 | 0 | 0 |
| 408 | 0.00001 | 0.00003 | 0.917908 | 0.082052 | 0 | 0 | 0 | 0 | 0 |
| 409 | 0.00003 | 0 | 0.918025 | 0.081945 | 0 | 0 | 0 | 0 | 0 |
| 410 | 0 | 0.00001 | 0.917772 | 0.082218 | 0 | 0 | 0 | 0 | 0 |
| 411 | 0.00003 | 0.00001 | 0.92147 | 0.078491 | 0 | 0 | 0 | 0 | 0 |
| 412 | 0 | 0.00002 | 0.921806 | 0.078174 | 0 | 0 | 0 | 0 | 0 |
| 413 | 0 | 0 | 0.919512 | 0.080488 | 0 | 0 | 0 | 0 | 0 |
| 414 | 0.00001 | 0 | 0.92386 | 0.07613 | 0 | 0 | 0 | 0 | 0 |
| 415 | 0.00001 | 0 | 0.920375 | 0.079615 | 0 | 0 | 0 | 0 | 0 |
| 416 | 0 | 0.00001 | 0.923624 | 0.076366 | 0 | 0 | 0 | 0 | 0 |
| 417 | 0.00001 | 0 | 0.926308 | 0.073682 | 0 | 0 | 0 | 0 | 0 |
| 418 | 0.00001 | 0 | 0.923712 | 0.076278 | 0 | 0 | 0 | 0 | 0 |
| 419 | 0.00001 | 0 | 0.919976 | 0.080014 | 0 | 0 | 0 | 0 | 0 |
| 420 | 0.00001 | 0.00001 | 0.929196 | 0.070783 | 0 | 0 | 0 | 0 | 0 |
| 421 | 0.00001 | 0 | 0.921966 | 0.078024 | 0 | 0 | 0 | 0 | 0 |
| 422 | 0.00002 | 0.00001 | 0.923839 | 0.076131 | 0 | 0 | 0 | 0 | 0 |
| 423 | 0.00001 | 0 | 0.924989 | 0.075001 | 0 | 0 | 0 | 0 | 0 |
| 424 | 0 | 0.00001 | 0.926589 | 0.073401 | 0 | 0 | 0 | 0 | 0 |
| 425 | 0 | 0.00002 | 0.920315 | 0.079665 | 0 | 0 | 0 | 0 | 0 |
| 426 | 0.00001 | 0.00001 | 0.922011 | 0.077969 | 0 | 0 | 0 | 0 | 0 |
| 427 | 0 | 0.00001 | 0.92319 | 0.0768 | 0 | 0 | 0 | 0 | 0 |
| 428 | 0 | 0 | 0.926191 | 0.073809 | 0 | 0 | 0 | 0 | 0 |
| 429 | 0 | 0 | 0.924419 | 0.075581 | 0 | 0 | 0 | 0 | 0 |
| 430 | 0 | 0 | 0.921084 | 0.078916 | 0 | 0 | 0 | 0 | 0 |
| 431 | 0.00003 | 0 | 0.925024 | 0.074946 | 0 | 0 | 0 | 0 | 0 |
| 432 | 0.00001 | 0.00002 | 0.918596 | 0.081374 | 0 | 0 | 0 | 0 | 0 |
| 433 | 0.00002 | 0 | 0.923501 | 0.076479 | 0 | 0 | 0 | 0 | 0 |
| 434 | 0 | 0.00001 | 0.923407 | 0.076583 | 0 | 0 | 0 | 0 | 0 |
| 435 | 0.00001 | 0 | 0.921025 | 0.078965 | 0 | 0 | 0 | 0 | 0 |
| 436 | 0 | 0 | 0.915467 | 0.084533 | 0 | 0 | 0 | 0 | 0 |
| 437 | 0 | 0 | 0.920732 | 0.079268 | 0 | 0 | 0 | 0 | 0 |
| 438 | 0.00001 | 0 | 0.92421 | 0.07578 | 0 | 0 | 0 | 0 | 0 |
| 439 | 0 | 0 | 0.925479 | 0.074521 | 0 | 0 | 0 | 0 | 0 |
| 440 | 0 | 0 | 0.923783 | 0.076217 | 0 | 0 | 0 | 0 | 0 |
| 441 | 0.00001 | 0.00003 | 0.920481 | 0.079479 | 0 | 0 | 0 | 0 | 0 |
| 442 | 0.00001 | 0 | 0.922629 | 0.077361 | 0 | 0 | 0 | 0 | 0 |
| 443 | 0.00001 | 0.00003 | 0.92712 | 0.07284 | 0 | 0 | 0 | 0 | 0 |
| 444 | 0 | 0.00001 | 0.919976 | 0.080014 | 0 | 0 | 0 | 0 | 0 |
| 445 | 0.00001 | 0 | 0.921895 | 0.078095 | 0 | 0 | 0 | 0 | 0 |
| 446 | 0.00002 | 0 | 0.921806 | 0.078174 | 0 | 0 | 0 | 0 | 0 |
| 447 | 0 | 0 | 0.925388 | 0.074612 | 0 | 0 | 0 | 0 | 0 |
| 448 | 0.00002 | 0.00001 | 0.922556 | 0.077414 | 0 | 0 | 0 | 0 | 0 |
| 449 | 0.00001 | 0 | 0.923318 | 0.076672 | 0 | 0 | 0 | 0 | 0 |
| 450 | 0.00001 | 0 | 0.92264 | 0.07735 | 0 | 0 | 0 | 0 | 0 |
| 451 | 0.00001 | 0 | 0.92601 | 0.07398 | 0 | 0 | 0 | 0 | 0 |
| 452 | 0 | 0 | 0.924397 | 0.075603 | 0 | 0 | 0 | 0 | 0 |
| 453 | 0 | 0 | 0.921689 | 0.078311 | 0 | 0 | 0 | 0 | 0 |
| 454 | 0.00001 | 0 | 0.922092 | 0.077898 | 0 | 0 | 0 | 0 | 0 |
| 455 | 0.00001 | 0 | 0.927635 | 0.072355 | 0 | 0 | 0 | 0 | 0 |
| 456 | 0.00001 | 0.00002 | 0.921253 | 0.078717 | 0 | 0 | 0 | 0 | 0 |
| 457 | 0.00002 | 0.00002 | 0.922259 | 0.077701 | 0 | 0 | 0 | 0 | 0 |
| 458 | 0.00001 | 0 | 0.92397 | 0.07602 | 0 | 0 | 0 | 0 | 0 |
| 459 | 0.00001 | 0 | 0.915171 | 0.084819 | 0 | 0 | 0 | 0 | 0 |
| 460 | 0.00001 | 0.00001 | 0.91848 | 0.0815 | 0 | 0 | 0 | 0 | 0 |
| 461 | 0.00003 | 0 | 0.925774 | 0.074196 | 0 | 0 | 0 | 0 | 0 |
| 462 | 0 | 0 | 0.926528 | 0.073472 | 0 | 0 | 0 | 0 | 0 |
| 463 | 0.00002 | 0 | 0.920504 | 0.079476 | 0 | 0 | 0 | 0 | 0 |
| 464 | 0 | 0 | 0.920105 | 0.079399 | 0 | 0 | 0 | 0 | 0 |
| 465 | 0.00001 | 0 | 0.925929 | 0.074061 | 0 | 0 | 0 | 0 | 0 |
| 466 | 0.00002 | 0 | 0.919493 | 0.080487 | 0 | 0 | 0 | 0 | 0 |
| 467 | 0 | 0 | 0.918943 | 0.081057 | 0 | 0 | 0 | 0 | 0 |
| 468 | 0.00002 | 0 | 0.924692 | 0.075288 | 0 | 0 | 0 | 0 | 0 |
| 469 | 0.00002 | 0 | 0.921199 | 0.078781 | 0 | 0 | 0 | 0 | 0 |
| 470 | 0 | 0 | 0.924513 | 0.075487 | 0 | 0 | 0 | 0 | 0 |
| 471 | 0.00003 | 0 | 0.921873 | 0.078097 | 0 | 0 | 0 | 0 | 0 |
| 472 | 0.00001 | 0 | 0.921767 | 0.078222 | 0 | 0 | 0 | 0 | 0 |
| 473 | 0.00003 | 0 | 0.923173 | 0.076797 | 0 | 0 | 0 | 0 | 0 |
| 474 | 0.00002 | 0 | 0.923458 | 0.076522 | 0 | 0 | 0 | 0 | 0 |
| 475 | 0.00001 | 0 | 0.922833 | 0.077157 | 0 | 0 | 0 | 0 | 0 |
| 476 | 0 | 0.00001 | 0.915355 | 0.084635 | 0 | 0 | 0 | 0 | 0 |
| 477 | 0.00003 | 0 | 0.923696 | 0.076274 | 0 | 0 | 0 | 0 | 0 |
| 478 | 0.00001 | 0.00001 | 0.921642 | 0.078338 | 0 | 0 | 0 | 0 | 0 |
| 479 | 0 | 0 | 0.919684 | 0.080316 | 0 | 0 | 0 | 0 | 0 |
| 480 | 0.00002 | 0 | 0.919888 | 0.080091 | 0 | 0 | 0 | 0 | 0 |
| 481 | 0 | 0.00001 | 0.926753 | 0.073237 | 0 | 0 | 0 | 0 | 0 |
| 482 | 0 | 0.00002 | 0.919242 | 0.080738 | 0 | 0 | 0 | 0 | 0 |
| 483 | 0.00001 | 0 | 0.924243 | 0.075747 | 0 | 0 | 0 | 0 | 0 |
| 484 | 0 | 0 | 0.918534 | 0.081466 | 0 | 0 | 0 | 0 | 0 |
| 485 | 0.00001 | 0.00001 | 0.927127 | 0.072853 | 0 | 0 | 0 | 0 | 0 |
| 486 | 0.00002 | 0 | 0.921721 | 0.078259 | 0 | 0 | 0 | 0 | 0 |
| 487 | 0.00001 | 0 | 0.921845 | 0.078145 | 0 | 0 | 0 | 0 | 0 |
| 488 | 0 | 0 | 0.919891 | 0.080109 | 0 | 0 | 0 | 0 | 0 |
| 489 | 0.00001 | 0 | 0.921761 | 0.078229 | 0 | 0 | 0 | 0 | 0 |
| 490 | 0 | 0 | 0.926531 | 0.073469 | 0 | 0 | 0 | 0 | 0 |
| 491 | 0.00002 | 0 | 0.921684 | 0.078296 | 0 | 0 | 0 | 0 | 0 |
| 492 | 0.00003 | 0.00002 | 0.92184 | 0.07811 | 0 | 0 | 0 | 0 | 0 |
| 493 | 0.00004 | 0 | 0.922506 | 0.077455 | 0 | 0 | 0 | 0 | 0 |
| 494 | 0.00001 | 0 | 0.922801 | 0.077189 | 0 | 0 | 0 | 0 | 0 |
| 495 | 0.00001 | 0 | 0.922027 | 0.077963 | 0 | 0 | 0 | 0 | 0 |
| 496 | 0.00003 | 0 | 0.920232 | 0.079677 | 0 | 0 | 0 | 0 | 0 |
| 497 | 0.00002 | 0 | 0.923329 | 0.076651 | 0 | 0 | 0 | 0 | 0 |
| 498 | 0 | 0 | 0.92704 | 0.07296 | 0 | 0 | 0 | 0 | 0 |
| 499 | 0.00002 | 0 | 0.921922 | 0.078058 | 0 | 0 | 0 | 0 | 0 |
| 500 | 0.00001 | 0 | 0.925169 | 0.074821 | 0 | 0 | 0 | 0 | 0 |
| 501 | 0 | 0 | 0.921941 | 0.078059 | 0 | 0 | 0 | 0 | 0 |
| 502 | 0.00002 | 0 | 0.923323 | 0.076657 | 0 | 0 | 0 | 0 | 0 |
| 503 | 0.00001 | 0.00002 | 0.920066 | 0.079904 | 0 | 0 | 0 | 0 | 0 |
| 504 | 0 | 0.00001 | 0.924683 | 0.075307 | 0 | 0 | 0 | 0 | 0 |
| 505 | 0.00004 | 0 | 0.920651 | 0.079279 | 0 | 0 | 0 | 0 | 0 |
| 506 | 0.00002 | 0 | 0.919186 | 0.080794 | 0 | 0 | 0 | 0 | 0 |
| 507 | 0.00005 | 0.00001 | 0.922107 | 0.077833 | 0 | 0 | 0 | 0 | 0 |
| 508 | 0.00003 | 0.00002 | 0.920541 | 0.079409 | 0 | 0 | 0 | 0 | 0 |
| 509 | 0.00001 | 0 | 0.91963 | 0.08036 | 0 | 0 | 0 | 0 | 0 |
| 510 | 0.00001 | 0.00002 | 0.923918 | 0.076052 | 0 | 0 | 0 | 0 | 0 |
| 511 | 0.00002 | 0 | 0.920975 | 0.079005 | 0 | 0 | 0 | 0 | 0 |
| 512 | 0 | 0.00001 | 0.91974 | 0.08025 | 0 | 0 | 0 | 0 | 0 |
| 513 | 0.00001 | 0 | 0.921615 | 0.078375 | 0 | 0 | 0 | 0 | 0 |
| 514 | 0 | 0 | 0.922565 | 0.077435 | 0 | 0 | 0 | 0 | 0 |
| 515 | 0.00001 | 0 | 0.925569 | 0.074421 | 0 | 0 | 0 | 0 | 0 |
| 516 | 0.00001 | 0 | 0.916914 | 0.083076 | 0 | 0 | 0 | 0 | 0 |
| 517 | 0.00002 | 0 | 0.920474 | 0.079506 | 0 | 0 | 0 | 0 | 0 |
| 518 | 0 | 0 | 0.92323 | 0.07677 | 0 | 0 | 0 | 0 | 0 |
| 519 | 0.00002 | 0.00001 | 0.921013 | 0.078957 | 0 | 0 | 0 | 0 | 0 |
| 520 | 0 | 0 | 0.923528 | 0.076472 | 0 | 0 | 0 | 0 | 0 |
| 521 | 0 | 0 | 0.920522 | 0.079478 | 0 | 0 | 0 | 0 | 0 |
| 522 | 0 | 0 | 0.926467 | 0.073533 | 0 | 0 | 0 | 0 | 0 |
| 523 | 0 | 0 | 0.921411 | 0.078589 | 0 | 0 | 0 | 0 | 0 |
| 524 | 0.00001 | 0.00001 | 0.920813 | 0.079167 | 0 | 0 | 0 | 0 | 0 |
| 525 | 0.00001 | 0 | 0.92059 | 0.0794 | 0 | 0 | 0 | 0 | 0 |
| 526 | 0 | 0.00003 | 0.921674 | 0.078296 | 0 | 0 | 0 | 0 | 0 |
| 527 | 0.00002 | 0 | 0.921738 | 0.078242 | 0 | 0 | 0 | 0 | 0 |
| 528 | 0 | 0 | 0.92127 | 0.07873 | 0 | 0 | 0 | 0 | 0 |
| 529 | 0.00001 | 0 | 0.923332 | 0.076658 | 0 | 0 | 0 | 0 | 0 |
| 530 | 0.00003 | 0 | 0.920352 | 0.079618 | 0 | 0 | 0 | 0 | 0 |
| 531 | 0.00003 | 0 | 0.921922 | 0.078048 | 0 | 0 | 0 | 0 | 0 |
| 532 | 0.00001 | 0 | 0.928711 | 0.071279 | 0 | 0 | 0 | 0 | 0 |
| 533 | 0.00003 | 0 | 0.923227 | 0.076743 | 0 | 0 | 0 | 0 | 0 |
| 534 | 0 | 0 | 0.919636 | 0.080364 | 0 | 0 | 0 | 0 | 0 |
| 535 | 0.00001 | 0.00001 | 0.92436 | 0.07562 | 0 | 0 | 0 | 0 | 0 |
| 536 | 0 | 0 | 0.926476 | 0.073524 | 0 | 0 | 0 | 0 | 0 |
| 537 | 0.00003 | 0 | 0.920554 | 0.079416 | 0 | 0 | 0 | 0 | 0 |
| 538 | 0.00001 | 0 | 0.919809 | 0.080181 | 0 | 0 | 0 | 0 | 0 |
| 539 | 0 | 0 | 0.918671 | 0.081329 | 0 | 0 | 0 | 0 | 0 |
| 540 | 0 | 0 | 0.920872 | 0.079128 | 0 | 0 | 0 | 0 | 0 |
| 541 | 0.00001 | 0.00001 | 0.925007 | 0.074973 | 0 | 0 | 0 | 0 | 0 |
| 542 | 0.00002 | 0 | 0.923376 | 0.076604 | 0 | 0 | 0 | 0 | 0 |
| 543 | 0.00003 | 0 | 0.926696 | 0.073274 | 0 | 0 | 0 | 0 | 0 |
| 544 | 0 | 0 | 0.925839 | 0.074161 | 0 | 0 | 0 | 0 | 0 |
| 545 | 0.00001 | 0 | 0.917646 | 0.082344 | 0 | 0 | 0 | 0 | 0 |
| 546 | 0.00002 | 0.00001 | 0.926771 | 0.073199 | 0 | 0 | 0 | 0 | 0 |
| 547 | 0.00002 | 0 | 0.920261 | 0.079719 | 0 | 0 | 0 | 0 | 0 |
| 548 | 0.00001 | 0 | 0.925744 | 0.074246 | 0 | 0 | 0 | 0 | 0 |
| 549 | 0.00003 | 0 | 0.91907 | 0.0809 | 0 | 0 | 0 | 0 | 0 |
| 550 | 0.00001 | 0 | 0.923021 | 0.076969 | 0 | 0 | 0 | 0 | 0 |
| 551 | 0 | 0 | 0.924338 | 0.075662 | 0 | 0 | 0 | 0 | 0 |
| 552 | 0 | 0.00003 | 0.92117 | 0.0788 | 0 | 0 | 0 | 0 | 0 |
| 553 | 0.00002 | 0 | 0.921674 | 0.078306 | 0 | 0 | 0 | 0 | 0 |
| 554 | 0.00003 | 0 | 0.928272 | 0.071698 | 0 | 0 | 0 | 0 | 0 |
| 555 | 0.00003 | 0 | 0.91939 | 0.08058 | 0 | 0 | 0 | 0 | 0 |
| 556 | 0 | 0 | 0.926667 | 0.073333 | 0 | 0 | 0 | 0 | 0 |
| 557 | 0.00003 | 0 | 0.922304 | 0.077666 | 0 | 0 | 0 | 0 | 0 |
| 558 | 0.00002 | 0 | 0.922633 | 0.077347 | 0 | 0 | 0 | 0 | 0 |
| 559 | 0 | 0 | 0.925321 | 0.074679 | 0 | 0 | 0 | 0 | 0 |
| 560 | 0.00001 | 0.00001 | 0.923516 | 0.076464 | 0 | 0 | 0 | 0 | 0 |
| 561 | 0.00003 | 0.00001 | 0.919043 | 0.080917 | 0 | 0 | 0 | 0 | 0 |
| 562 | 0 | 0 | 0.919024 | 0.080976 | 0 | 0 | 0 | 0 | 0 |
| 563 | 0.00001 | 0 | 0.91909 | 0.080899 | 0 | 0 | 0 | 0 | 0 |
| 564 | 0 | 0 | 0.923422 | 0.076578 | 0 | 0 | 0 | 0 | 0 |
| 565 | 0.00003 | 0 | 0.919794 | 0.080176 | 0 | 0 | 0 | 0 | 0 |
| 566 | 0 | 0 | 0.924051 | 0.075949 | 0 | 0 | 0 | 0 | 0 |
| 567 | 0.00001 | 0 | 0.921262 | 0.078728 | 0 | 0 | 0 | 0 | 0 |
| 568 | 0.00002 | 0 | 0.918638 | 0.081342 | 0 | 0 | 0 | 0 | 0 |
| 569 | 0.00001 | 0 | 0.924328 | 0.075662 | 0 | 0 | 0 | 0 | 0 |
| 570 | 0.00001 | 0 | 0.917586 | 0.082404 | 0 | 0 | 0 | 0 | 0 |
| 571 | 0.00001 | 0 | 0.920902 | 0.079088 | 0 | 0 | 0 | 0 | 0 |
| 572 | 0.00001 | 0 | 0.92415 | 0.07584 | 0 | 0 | 0 | 0 | 0 |
| 573 | 0.00001 | 0.00001 | 0.924762 | 0.075218 | 0 | 0 | 0 | 0 | 0 |
| 574 | 0.00002 | 0 | 0.921954 | 0.078027 | 0 | 0 | 0 | 0 | 0 |
| 575 | 0.00002 | 0 | 0.921607 | 0.078373 | 0 | 0 | 0 | 0 | 0 |
| 576 | 0.00001 | 0 | 0.926974 | 0.073016 | 0 | 0 | 0 | 0 | 0 |
| 577 | 0.00001 | 0 | 0.920464 | 0.079526 | 0 | 0 | 0 | 0 | 0 |
| 578 | 0 | 0 | 0.925448 | 0.074552 | 0 | 0 | 0 | 0 | 0 |
| 579 | 0.00002 | 0.00001 | 0.919327 | 0.080643 | 0 | 0 | 0 | 0 | 0 |
| 580 | 0.00001 | 0.00001 | 0.924116 | 0.075864 | 0 | 0 | 0 | 0 | 0 |
| 581 | 0 | 0 | 0.919358 | 0.080642 | 0 | 0 | 0 | 0 | 0 |
| 582 | 0.00001 | 0 | 0.921977 | 0.078013 | 0 | 0 | 0 | 0 | 0 |
| 583 | 0 | 0.00003 | 0.919359 | 0.080611 | 0 | 0 | 0 | 0 | 0 |
| 584 | 0 | 0 | 0.917939 | 0.082061 | 0 | 0 | 0 | 0 | 0 |
| 585 | 0.00001 | 0 | 0.920103 | 0.079887 | 0 | 0 | 0 | 0 | 0 |
| 586 | 0 | 0.00001 | 0.922627 | 0.077363 | 0 | 0 | 0 | 0 | 0 |
| 587 | 0.00001 | 0 | 0.923167 | 0.076823 | 0 | 0 | 0 | 0 | 0 |
| 588 | 0.00001 | 0 | 0.925388 | 0.074602 | 0 | 0 | 0 | 0 | 0 |
| 589 | 0.00001 | 0.00004 | 0.922291 | 0.077659 | 0 | 0 | 0 | 0 | 0 |
| 590 | 0 | 0.00001 | 0.918779 | 0.081211 | 0 | 0 | 0 | 0 | 0 |
| 591 | 0.00002 | 0.00001 | 0.919185 | 0.080785 | 0 | 0 | 0 | 0 | 0 |
| 592 | 0.00002 | 0.00002 | 0.920658 | 0.079301 | 0 | 0 | 0 | 0 | 0 |
| 593 | 0.00002 | 0 | 0.924333 | 0.075647 | 0 | 0 | 0 | 0 | 0 |
| 594 | 0.00001 | 0 | 0.919982 | 0.080008 | 0 | 0 | 0 | 0 | 0 |
| 595 | 0.00002 | 0.00001 | 0.920921 | 0.079049 | 0 | 0 | 0 | 0 | 0 |
| 596 | 0.00001 | 0 | 0.923701 | 0.07629 | 0 | 0 | 0 | 0 | 0 |
| 597 | 0 | 0.00001 | 0.923651 | 0.076339 | 0 | 0 | 0 | 0 | 0 |
| 598 | 0 | 0 | 0.92304 | 0.07696 | 0 | 0 | 0 | 0 | 0 |
| 599 | 0.00003 | 0.00002 | 0.923061 | 0.076889 | 0 | 0 | 0 | 0 | 0 |
| 600 | 0.00001 | 0.00003 | 0.918612 | 0.081348 | 0 | 0 | 0 | 0 | 0 |
| 601 | 0 | 0 | 0.920375 | 0.079625 | 0 | 0 | 0 | 0 | 0 |
| 602 | 0.00002 | 0 | 0.926065 | 0.073915 | 0 | 0 | 0 | 0 | 0 |
| 603 | 0.00002 | 0 | 0.923401 | 0.076579 | 0 | 0 | 0 | 0 | 0 |
| 604 | 0.00001 | 0 | 0.921772 | 0.078218 | 0 | 0 | 0 | 0 | 0 |
| 605 | 0.00001 | 0 | 0.921717 | 0.078273 | 0 | 0 | 0 | 0 | 0 |
| 606 | 0.00001 | 0 | 0.924637 | 0.075353 | 0 | 0 | 0 | 0 | 0 |
| 607 | 0 | 0 | 0.926897 | 0.073103 | 0 | 0 | 0 | 0 | 0 |
| 608 | 0.00001 | 0 | 0.924682 | 0.075308 | 0 | 0 | 0 | 0 | 0 |
| 609 | 0.00001 | 0 | 0.92464 | 0.07535 | 0 | 0 | 0 | 0 | 0 |
| 610 | 0.00001 | 0 | 0.919178 | 0.080812 | 0 | 0 | 0 | 0 | 0 |
| 611 | 0.00001 | 0.00004 | 0.923067 | 0.076883 | 0 | 0 | 0 | 0 | 0 |
| 612 | 0.00002 | 0.00002 | 0.921589 | 0.078371 | 0 | 0 | 0 | 0 | 0 |
| 613 | 0 | 0 | 0.917235 | 0.082765 | 0 | 0 | 0 | 0 | 0 |
| 614 | 0.00001 | 0.00001 | 0.924924 | 0.075056 | 0 | 0 | 0 | 0 | 0 |
| 615 | 0.00001 | 0.00001 | 0.923657 | 0.076323 | 0 | 0 | 0 | 0 | 0 |
| 616 | 0.00001 | 0 | 0.923718 | 0.076272 | 0 | 0 | 0 | 0 | 0 |
| 617 | 0.00001 | 0 | 0.922856 | 0.077134 | 0 | 0 | 0 | 0 | 0 |
| 618 | 0 | 0 | 0.918604 | 0.081396 | 0 | 0 | 0 | 0 | 0 |
| 619 | 0.00003 | 0.00001 | 0.925251 | 0.074709 | 0 | 0 | 0 | 0 | 0 |
| 620 | 0.00001 | 0.00003 | 0.921353 | 0.078607 | 0 | 0 | 0 | 0 | 0 |
| 621 | 0.00001 | 0 | 0.924645 | 0.075345 | 0 | 0 | 0 | 0 | 0 |
| 622 | 0 | 0 | 0.918451 | 0.081549 | 0 | 0 | 0 | 0 | 0 |
| 623 | 0.00003 | 0 | 0.921885 | 0.078086 | 0 | 0 | 0 | 0 | 0 |
| 624 | 0.00006 | 0 | 0.920223 | 0.079717 | 0 | 0 | 0 | 0 | 0 |
| 625 | 0 | 0.00001 | 0.922059 | 0.077802 | 0 | 0 | 0 | 0 | 0 |
| 626 | 0.00002 | 0.00001 | 0.918939 | 0.081031 | 0 | 0 | 0 | 0 | 0 |
| 627 | 0 | 0 | 0.924409 | 0.075591 | 0 | 0 | 0 | 0 | 0 |
| 628 | 0.00002 | 0.00002 | 0.922593 | 0.077367 | 0 | 0 | 0 | 0 | 0 |
| 629 | 0.00001 | 0 | 0.920743 | 0.079247 | 0 | 0 | 0 | 0 | 0 |
| 630 | 0.00001 | 0 | 0.922892 | 0.077098 | 0 | 0 | 0 | 0 | 0 |
| 631 | 0 | 0 | 0.919632 | 0.080368 | 0 | 0 | 0 | 0 | 0 |
| 632 | 0 | 0 | 0.924192 | 0.075808 | 0 | 0 | 0 | 0 | 0 |
| 633 | 0 | 0.00001 | 0.923069 | 0.076921 | 0 | 0 | 0 | 0 | 0 |
| 634 | 0 | 0 | 0.91947 | 0.08053 | 0 | 0 | 0 | 0 | 0 |
| 635 | 0.00002 | 0 | 0.927662 | 0.072318 | 0 | 0 | 0 | 0 | 0 |
| 636 | 0 | 0 | 0.920533 | 0.079467 | 0 | 0 | 0 | 0 | 0 |
| 637 | 0.00001 | 0 | 0.919766 | 0.080224 | 0 | 0 | 0 | 0 | 0 |
| 638 | 0 | 0 | 0.922367 | 0.077633 | 0 | 0 | 0 | 0 | 0 |
| 639 | 0.00001 | 0 | 0.919131 | 0.080859 | 0 | 0 | 0 | 0 | 0 |
| 640 | 0.00001 | 0 | 0.917759 | 0.082231 | 0 | 0 | 0 | 0 | 0 |
| 641 | 0 | 0 | 0.92421 | 0.07579 | 0 | 0 | 0 | 0 | 0 |
| 642 | 0.00001 | 0.00001 | 0.92378 | 0.0762 | 0 | 0 | 0 | 0 | 0 |
| 643 | 0.00001 | 0 | 0.920748 | 0.079242 | 0 | 0 | 0 | 0 | 0 |
| 644 | 0.00002 | 0 | 0.921478 | 0.078502 | 0 | 0 | 0 | 0 | 0 |
| 645 | 0.00002 | 0.00002 | 0.923559 | 0.076401 | 0 | 0 | 0 | 0 | 0 |
| 646 | 0.00001 | 0 | 0.922346 | 0.077643 | 0 | 0 | 0 | 0 | 0 |
| 647 | 0.00001 | 0 | 0.920935 | 0.079055 | 0 | 0 | 0 | 0 | 0 |
| 648 | 0.00001 | 0 | 0.924586 | 0.075404 | 0 | 0 | 0 | 0 | 0 |
| 649 | 0 | 0 | 0.920641 | 0.079359 | 0 | 0 | 0 | 0 | 0 |
| 650 | 0.00001 | 0 | 0.922179 | 0.077811 | 0 | 0 | 0 | 0 | 0 |
| 651 | 0.00001 | 0 | 0.922423 | 0.077567 | 0 | 0 | 0 | 0 | 0 |
| 652 | 0.00002 | 0.00001 | 0.922049 | 0.077921 | 0 | 0 | 0 | 0 | 0 |
| 653 | 0.00003 | 0 | 0.925726 | 0.074244 | 0 | 0 | 0 | 0 | 0 |
| 654 | 0.00001 | 0 | 0.922114 | 0.077876 | 0 | 0 | 0 | 0 | 0 |
| 655 | 0 | 0.00002 | 0.920687 | 0.079293 | 0 | 0 | 0 | 0 | 0 |
| 656 | 0.00002 | 0 | 0.928787 | 0.071193 | 0 | 0 | 0 | 0 | 0 |
| 657 | 0.00001 | 0.00001 | 0.920013 | 0.079967 | 0 | 0 | 0 | 0 | 0 |
| 658 | 0 | 0 | 0.923518 | 0.076482 | 0 | 0 | 0 | 0 | 0 |
| 659 | 0.00001 | 0 | 0.91807 | 0.08192 | 0 | 0 | 0 | 0 | 0 |
| 660 | 0.00001 | 0 | 0.92579 | 0.0742 | 0 | 0 | 0 | 0 | 0 |
| 661 | 0 | 0 | 0.924459 | 0.075541 | 0 | 0 | 0 | 0 | 0 |
| 662 | 0.00004 | 0 | 0.919819 | 0.080142 | 0 | 0 | 0 | 0 | 0 |
| 663 | 0.00003 | 0 | 0.923005 | 0.076965 | 0 | 0 | 0 | 0 | 0 |
| 664 | 0.00002 | 0.00002 | 0.926869 | 0.073091 | 0 | 0 | 0 | 0 | 0 |
| 665 | 0 | 0 | 0.921945 | 0.078055 | 0 | 0 | 0 | 0 | 0 |
| 666 | 0 | 0 | 0.923085 | 0.076915 | 0 | 0 | 0 | 0 | 0 |
| 667 | 0.00001 | 0 | 0.917648 | 0.082342 | 0 | 0 | 0 | 0 | 0 |
| 668 | 0.00003 | 0 | 0.920616 | 0.079354 | 0 | 0 | 0 | 0 | 0 |
| 669 | 0.00001 | 0.00001 | 0.919845 | 0.080135 | 0 | 0 | 0 | 0 | 0 |
| 670 | 0.00001 | 0 | 0.921801 | 0.078188 | 0 | 0 | 0 | 0 | 0 |
| 671 | 0.00003 | 0 | 0.92301 | 0.07696 | 0 | 0 | 0 | 0 | 0 |
| 672 | 0 | 0.00002 | 0.923316 | 0.076664 | 0 | 0 | 0 | 0 | 0 |
| 673 | 0 | 0 | 0.920541 | 0.079459 | 0 | 0 | 0 | 0 | 0 |
| 674 | 0.00001 | 0 | 0.921253 | 0.078737 | 0 | 0 | 0 | 0 | 0 |
| 675 | 0.00002 | 0 | 0.923369 | 0.076611 | 0 | 0 | 0 | 0 | 0 |
| 676 | 0 | 0.00001 | 0.925543 | 0.074447 | 0 | 0 | 0 | 0 | 0 |
| 677 | 0.00003 | 0 | 0.922147 | 0.077823 | 0 | 0 | 0 | 0 | 0 |
| 678 | 0 | 0 | 0.923185 | 0.076815 | 0 | 0 | 0 | 0 | 0 |
| 679 | 0.00001 | 0 | 0.925786 | 0.074204 | 0 | 0 | 0 | 0 | 0 |
| 680 | 0.00001 | 0 | 0.920633 | 0.079357 | 0 | 0 | 0 | 0 | 0 |
| 681 | 0 | 0 | 0.920077 | 0.079923 | 0 | 0 | 0 | 0 | 0 |
| 682 | 0.00002 | 0 | 0.921047 | 0.078933 | 0 | 0 | 0 | 0 | 0 |
| 683 | 0.00003 | 0 | 0.921866 | 0.078104 | 0 | 0 | 0 | 0 | 0 |
| 684 | 0 | 0 | 0.925157 | 0.074843 | 0 | 0 | 0 | 0 | 0 |
| 685 | 0 | 0 | 0.922412 | 0.077588 | 0 | 0 | 0 | 0 | 0 |
| 686 | 0.00001 | 0 | 0.917913 | 0.082077 | 0 | 0 | 0 | 0 | 0 |
| 687 | 0 | 0.00001 | 0.92457 | 0.07542 | 0 | 0 | 0 | 0 | 0 |
| 688 | 0.00001 | 0 | 0.923119 | 0.076871 | 0 | 0 | 0 | 0 | 0 |
| 689 | 0 | 0 | 0.919314 | 0.080686 | 0 | 0 | 0 | 0 | 0 |
| 690 | 0.00003 | 0 | 0.921413 | 0.078557 | 0 | 0 | 0 | 0 | 0 |
| 691 | 0 | 0.00001 | 0.924821 | 0.075169 | 0 | 0 | 0 | 0 | 0 |
| 692 | 0.00001 | 0 | 0.920274 | 0.079716 | 0 | 0 | 0 | 0 | 0 |
| 693 | 0.00003 | 0 | 0.918915 | 0.081055 | 0 | 0 | 0 | 0 | 0 |
| 694 | 0.00001 | 0.00002 | 0.925146 | 0.074824 | 0 | 0 | 0 | 0 | 0 |
| 695 | 0.00001 | 0 | 0.92146 | 0.07853 | 0 | 0 | 0 | 0 | 0 |
| 696 | 0 | 0 | 0.926604 | 0.073396 | 0 | 0 | 0 | 0 | 0 |
| 697 | 0.00003 | 0 | 0.924326 | 0.075644 | 0 | 0 | 0 | 0 | 0 |
| 698 | 0 | 0.00001 | 0.922835 | 0.077155 | 0 | 0 | 0 | 0 | 0 |
| 699 | 0.00001 | 0 | 0.924032 | 0.075957 | 0 | 0 | 0 | 0 | 0 |
| 700 | 0.00002 | 0 | 0.9223 | 0.07768 | 0 | 0 | 0 | 0 | 0 |
| 701 | 0.00001 | 0.00003 | 0.928929 | 0.071032 | 0 | 0 | 0 | 0 | 0 |
| 702 | 0 | 0.00004 | 0.926024 | 0.073936 | 0 | 0 | 0 | 0 | 0 |
| 703 | 0.00001 | 0 | 0.921415 | 0.078575 | 0 | 0 | 0 | 0 | 0 |
| 704 | 0 | 0 | 0.92183 | 0.07817 | 0 | 0 | 0 | 0 | 0 |
| 705 | 0 | 0 | 0.920392 | 0.079608 | 0 | 0 | 0 | 0 | 0 |
| 706 | 0 | 0 | 0.92022 | 0.07978 | 0 | 0 | 0 | 0 | 0 |
| 707 | 0.00001 | 0 | 0.920395 | 0.079595 | 0 | 0 | 0 | 0 | 0 |
| 708 | 0 | 0 | 0.919958 | 0.080042 | 0 | 0 | 0 | 0 | 0 |
| 709 | 0.00002 | 0 | 0.925763 | 0.074217 | 0 | 0 | 0 | 0 | 0 |
| 710 | 0 | 0 | 0.923028 | 0.076972 | 0 | 0 | 0 | 0 | 0 |
| 711 | 0 | 0.00001 | 0.923844 | 0.076146 | 0 | 0 | 0 | 0 | 0 |
| 712 | 0.00001 | 0 | 0.926605 | 0.073385 | 0 | 0 | 0 | 0 | 0 |
| 713 | 0.00003 | 0 | 0.918439 | 0.081531 | 0 | 0 | 0 | 0 | 0 |
| 714 | 0 | 0 | 0.919857 | 0.080143 | 0 | 0 | 0 | 0 | 0 |
| 715 | 0.00001 | 0.00001 | 0.919639 | 0.080341 | 0 | 0 | 0 | 0 | 0 |
| 716 | 0.00001 | 0 | 0.922274 | 0.077716 | 0 | 0 | 0 | 0 | 0 |
| 717 | 0 | 0 | 0.922124 | 0.077876 | 0 | 0 | 0 | 0 | 0 |
| 718 | 0.00001 | 0 | 0.921019 | 0.078971 | 0 | 0 | 0 | 0 | 0 |
| 719 | 0 | 0 | 0.926288 | 0.073712 | 0 | 0 | 0 | 0 | 0 |
| 720 | 0.00004 | 0 | 0.920082 | 0.079879 | 0 | 0 | 0 | 0 | 0 |
| 721 | 0.00001 | 0 | 0.920748 | 0.079242 | 0 | 0 | 0 | 0 | 0 |
| 722 | 0 | 0 | 0.924391 | 0.075609 | 0 | 0 | 0 | 0 | 0 |
| 723 | 0.00001 | 0.00001 | 0.921139 | 0.078841 | 0 | 0 | 0 | 0 | 0 |
| 724 | 0.00001 | 0 | 0.923609 | 0.076381 | 0 | 0 | 0 | 0 | 0 |
| 725 | 0 | 0 | 0.921424 | 0.078576 | 0 | 0 | 0 | 0 | 0 |
| 726 | 0.00003 | 0.00001 | 0.922148 | 0.077812 | 0 | 0 | 0 | 0 | 0 |
| 727 | 0.00001 | 0 | 0.920635 | 0.079355 | 0 | 0 | 0 | 0 | 0 |
| 728 | 0 | 0.00001 | 0.920976 | 0.079014 | 0 | 0 | 0 | 0 | 0 |
| 729 | 0 | 0.00001 | 0.919878 | 0.080112 | 0 | 0 | 0 | 0 | 0 |
| 730 | 0.00002 | 0.00001 | 0.921151 | 0.07882 | 0 | 0 | 0 | 0 | 0 |
| 731 | 0.00001 | 0 | 0.9254 | 0.07459 | 0 | 0 | 0 | 0 | 0 |
| 732 | 0.00001 | 0 | 0.924056 | 0.075934 | 0 | 0 | 0 | 0 | 0 |
| 733 | 0.00002 | 0 | 0.918726 | 0.081254 | 0 | 0 | 0 | 0 | 0 |
| 734 | 0 | 0.00001 | 0.9243 | 0.075691 | 0 | 0 | 0 | 0 | 0 |
| 735 | 0.00001 | 0 | 0.926602 | 0.073388 | 0 | 0 | 0 | 0 | 0 |
| 736 | 0.00003 | 0 | 0.926123 | 0.073847 | 0 | 0 | 0 | 0 | 0 |
| 737 | 0.00001 | 0.00001 | 0.923559 | 0.076421 | 0 | 0 | 0 | 0 | 0 |
| 738 | 0 | 0.00001 | 0.919148 | 0.080842 | 0 | 0 | 0 | 0 | 0 |
| 739 | 0.00002 | 0 | 0.913431 | 0.086549 | 0 | 0 | 0 | 0 | 0 |
| 740 | 0 | 0 | 0.923684 | 0.076316 | 0 | 0 | 0 | 0 | 0 |
| 741 | 0.00001 | 0 | 0.921703 | 0.078287 | 0 | 0 | 0 | 0 | 0 |
| 742 | 0.00001 | 0 | 0.922349 | 0.077641 | 0 | 0 | 0 | 0 | 0 |
| 743 | 0.00001 | 0 | 0.923682 | 0.076308 | 0 | 0 | 0 | 0 | 0 |
| 744 | 0.00004 | 0 | 0.92485 | 0.074763 | 0 | 0 | 0 | 0 | 0 |
| 745 | 0.00001 | 0.00001 | 0.924795 | 0.075185 | 0 | 0 | 0 | 0 | 0 |
| 746 | 0.00001 | 0.00001 | 0.916577 | 0.083403 | 0 | 0 | 0 | 0 | 0 |
| 747 | 0.00001 | 0 | 0.920895 | 0.079095 | 0 | 0 | 0 | 0 | 0 |
| 748 | 0.00005 | 0 | 0.923662 | 0.076288 | 0 | 0 | 0 | 0 | 0 |
| 749 | 0 | 0 | 0.928685 | 0.071315 | 0 | 0 | 0 | 0 | 0 |
| 750 | 0.00003 | 0 | 0.923366 | 0.076604 | 0 | 0 | 0 | 0 | 0 |
| 751 | 0.00001 | 0 | 0.926386 | 0.073605 | 0 | 0 | 0 | 0 | 0 |
| 752 | 0.00001 | 0 | 0.924501 | 0.075489 | 0 | 0 | 0 | 0 | 0 |
| 753 | 0.00004 | 0 | 0.92222 | 0.07774 | 0 | 0 | 0 | 0 | 0 |
| 754 | 0.00003 | 0.00001 | 0.92045 | 0.07951 | 0 | 0 | 0 | 0 | 0 |
| 755 | 0.00002 | 0.00002 | 0.921507 | 0.078453 | 0 | 0 | 0 | 0 | 0 |
| 756 | 0.00001 | 0 | 0.920853 | 0.079137 | 0 | 0 | 0 | 0 | 0 |
| 757 | 0 | 0 | 0.923574 | 0.076426 | 0 | 0 | 0 | 0 | 0 |
| 758 | 0.00002 | 0 | 0.92048 | 0.0795 | 0 | 0 | 0 | 0 | 0 |
| 759 | 0 | 0.00002 | 0.925695 | 0.074285 | 0 | 0 | 0 | 0 | 0 |
| 760 | 0.00002 | 0.00004 | 0.925528 | 0.074412 | 0 | 0 | 0 | 0 | 0 |
| 761 | 0.00001 | 0 | 0.920867 | 0.079123 | 0 | 0 | 0 | 0 | 0 |
| 762 | 0.00001 | 0 | 0.922939 | 0.077051 | 0 | 0 | 0 | 0 | 0 |
| 763 | 0 | 0 | 0.924514 | 0.075486 | 0 | 0 | 0 | 0 | 0 |
| 764 | 0 | 0 | 0.928698 | 0.071302 | 0 | 0 | 0 | 0 | 0 |
| 765 | 0.00001 | 0.00001 | 0.920044 | 0.079936 | 0 | 0 | 0 | 0 | 0 |
| 766 | 0 | 0.00001 | 0.918534 | 0.081456 | 0 | 0 | 0 | 0 | 0 |
| 767 | 0.00002 | 0.00001 | 0.923606 | 0.076364 | 0 | 0 | 0 | 0 | 0 |
| 768 | 0 | 0 | 0.921815 | 0.078185 | 0 | 0 | 0 | 0 | 0 |
| 769 | 0.00001 | 0 | 0.921642 | 0.078348 | 0 | 0 | 0 | 0 | 0 |
| 770 | 0.00003 | 0 | 0.922448 | 0.077523 | 0 | 0 | 0 | 0 | 0 |
| 771 | 0.00003 | 0 | 0.921898 | 0.077792 | 0 | 0 | 0 | 0 | 0 |
| 772 | 0.00002 | 0.00001 | 0.919807 | 0.080163 | 0 | 0 | 0 | 0 | 0 |
| 773 | 0 | 0 | 0.917643 | 0.082357 | 0 | 0 | 0 | 0 | 0 |
| 774 | 0.00002 | 0 | 0.923786 | 0.076194 | 0 | 0 | 0 | 0 | 0 |
| 775 | 0 | 0 | 0.922689 | 0.077311 | 0 | 0 | 0 | 0 | 0 |
| 776 | 0.00001 | 0 | 0.919759 | 0.080231 | 0 | 0 | 0 | 0 | 0 |
| 777 | 0 | 0 | 0.924301 | 0.075699 | 0 | 0 | 0 | 0 | 0 |
| 778 | 0 | 0 | 0.923386 | 0.076614 | 0 | 0 | 0 | 0 | 0 |
| 779 | 0.00002 | 0.00001 | 0.928589 | 0.071382 | 0 | 0 | 0 | 0 | 0 |
| 780 | 0.00001 | 0 | 0.921164 | 0.078826 | 0 | 0 | 0 | 0 | 0 |
| 781 | 0.00001 | 0.00002 | 0.920675 | 0.079295 | 0 | 0 | 0 | 0 | 0 |
| 782 | 0 | 0 | 0.926014 | 0.073986 | 0 | 0 | 0 | 0 | 0 |
| 783 | 0.00001 | 0.00001 | 0.920764 | 0.079216 | 0 | 0 | 0 | 0 | 0 |
| 784 | 0 | 0 | 0.921267 | 0.078733 | 0 | 0 | 0 | 0 | 0 |
| 785 | 0.00002 | 0.00002 | 0.924297 | 0.075663 | 0 | 0 | 0 | 0 | 0 |
| 786 | 0 | 0.00001 | 0.921253 | 0.078737 | 0 | 0 | 0 | 0 | 0 |
| 787 | 0 | 0 | 0.92193 | 0.07807 | 0 | 0 | 0 | 0 | 0 |
| 788 | 0 | 0 | 0.926394 | 0.073606 | 0 | 0 | 0 | 0 | 0 |
| 789 | 0.00002 | 0 | 0.919363 | 0.080617 | 0 | 0 | 0 | 0 | 0 |
| 790 | 0.00002 | 0 | 0.923976 | 0.076004 | 0 | 0 | 0 | 0 | 0 |
| 791 | 0.00001 | 0 | 0.923839 | 0.076151 | 0 | 0 | 0 | 0 | 0 |
| 792 | 0.00002 | 0.00002 | 0.925215 | 0.074745 | 0 | 0 | 0 | 0 | 0 |
| 793 | 0.00001 | 0 | 0.922193 | 0.077797 | 0 | 0 | 0 | 0 | 0 |
| 794 | 0.00001 | 0 | 0.924209 | 0.075781 | 0 | 0 | 0 | 0 | 0 |
| 795 | 0.00002 | 0 | 0.920322 | 0.079658 | 0 | 0 | 0 | 0 | 0 |
| 796 | 0 | 0 | 0.924762 | 0.075238 | 0 | 0 | 0 | 0 | 0 |
| 797 | 0.00001 | 0 | 0.924085 | 0.075905 | 0 | 0 | 0 | 0 | 0 |
| 798 | 0.00001 | 0.00002 | 0.919628 | 0.080282 | 0 | 0 | 0 | 0 | 0 |
| 799 | 0 | 0.00001 | 0.923068 | 0.076922 | 0 | 0 | 0 | 0 | 0 |
| 800 | 0.00002 | 0.00001 | 0.918343 | 0.081627 | 0 | 0 | 0 | 0 | 0 |
| 801 | 0 | 0 | 0.926805 | 0.073195 | 0 | 0 | 0 | 0 | 0 |
| 802 | 0 | 0 | 0.918507 | 0.081493 | 0 | 0 | 0 | 0 | 0 |
| 803 | 0.00003 | 0.00001 | 0.925513 | 0.074447 | 0 | 0 | 0 | 0 | 0 |
| 804 | 0.00002 | 0.00001 | 0.922267 | 0.077703 | 0 | 0 | 0 | 0 | 0 |
| 805 | 0.00001 | 0 | 0.926292 | 0.073698 | 0 | 0 | 0 | 0 | 0 |
| 806 | 0.00002 | 0 | 0.920144 | 0.079836 | 0 | 0 | 0 | 0 | 0 |
| 807 | 0.00001 | 0 | 0.925527 | 0.074463 | 0 | 0 | 0 | 0 | 0 |
| 808 | 0 | 0.00001 | 0.922071 | 0.077919 | 0 | 0 | 0 | 0 | 0 |
| 809 | 0.00002 | 0 | 0.923503 | 0.076477 | 0 | 0 | 0 | 0 | 0 |
| 810 | 0.00002 | 0 | 0.921034 | 0.078946 | 0 | 0 | 0 | 0 | 0 |
| 811 | 0.00003 | 0.00002 | 0.922496 | 0.077454 | 0 | 0 | 0 | 0 | 0 |
| 812 | 0.00002 | 0 | 0.922726 | 0.077254 | 0 | 0 | 0 | 0 | 0 |
| 813 | 0 | 0.00003 | 0.92389 | 0.076081 | 0 | 0 | 0 | 0 | 0 |
| 814 | 0.00001 | 0 | 0.922736 | 0.077254 | 0 | 0 | 0 | 0 | 0 |
| 815 | 0.00001 | 0 | 0.921432 | 0.078558 | 0 | 0 | 0 | 0 | 0 |
| 816 | 0 | 0 | 0.92165 | 0.07835 | 0 | 0 | 0 | 0 | 0 |
| 817 | 0 | 0 | 0.924657 | 0.075343 | 0 | 0 | 0 | 0 | 0 |
| 818 | 0 | 0 | 0.918115 | 0.081885 | 0 | 0 | 0 | 0 | 0 |
| 819 | 0.00001 | 0 | 0.91856 | 0.08143 | 0 | 0 | 0 | 0 | 0 |
| 820 | 0 | 0 | 0.922777 | 0.077223 | 0 | 0 | 0 | 0 | 0 |
| 821 | 0 | 0.00001 | 0.924775 | 0.075215 | 0 | 0 | 0 | 0 | 0 |
| 822 | 0.00003 | 0 | 0.92319 | 0.07678 | 0 | 0 | 0 | 0 | 0 |
| 823 | 0.00002 | 0 | 0.922741 | 0.077239 | 0 | 0 | 0 | 0 | 0 |
| 824 | 0.00001 | 0.00001 | 0.928846 | 0.071134 | 0 | 0 | 0 | 0 | 0 |
| 825 | 0.00001 | 0 | 0.924643 | 0.075347 | 0 | 0 | 0 | 0 | 0 |
| 826 | 0.00001 | 0 | 0.925537 | 0.074453 | 0 | 0 | 0 | 0 | 0 |
| 827 | 0.00001 | 0 | 0.921948 | 0.078042 | 0 | 0 | 0 | 0 | 0 |
| 828 | 0.00002 | 0 | 0.921437 | 0.078543 | 0 | 0 | 0 | 0 | 0 |
| 829 | 0.00004 | 0 | 0.920275 | 0.079685 | 0 | 0 | 0 | 0 | 0 |
| 830 | 0.00001 | 0 | 0.92502 | 0.07497 | 0 | 0 | 0 | 0 | 0 |
| 831 | 0.00001 | 0 | 0.918263 | 0.081727 | 0 | 0 | 0 | 0 | 0 |
| 832 | 0.00004 | 0 | 0.923237 | 0.076723 | 0 | 0 | 0 | 0 | 0 |
| 833 | 0.00005 | 0.00001 | 0.922377 | 0.077563 | 0 | 0 | 0 | 0 | 0 |
| 834 | 0 | 0.00001 | 0.919422 | 0.080568 | 0 | 0 | 0 | 0 | 0 |
| 835 | 0.00002 | 0.00001 | 0.922917 | 0.077053 | 0 | 0 | 0 | 0 | 0 |
| 836 | 0 | 0 | 0.923748 | 0.076252 | 0 | 0 | 0 | 0 | 0 |
| 837 | 0 | 0 | 0.923749 | 0.076251 | 0 | 0 | 0 | 0 | 0 |
| 838 | 0.00001 | 0.00001 | 0.920196 | 0.079784 | 0 | 0 | 0 | 0 | 0 |
| 839 | 0.00002 | 0 | 0.919293 | 0.080687 | 0 | 0 | 0 | 0 | 0 |
| 840 | 0.00002 | 0 | 0.921635 | 0.078345 | 0 | 0 | 0 | 0 | 0 |
| 841 | 0 | 0 | 0.921588 | 0.078412 | 0 | 0 | 0 | 0 | 0 |
| 842 | 0 | 0 | 0.920543 | 0.079457 | 0 | 0 | 0 | 0 | 0 |
| 843 | 0.00002 | 0 | 0.92346 | 0.07652 | 0 | 0 | 0 | 0 | 0 |
| 844 | 0 | 0 | 0.922057 | 0.077943 | 0 | 0 | 0 | 0 | 0 |
| 845 | 0.00001 | 0 | 0.920103 | 0.079887 | 0 | 0 | 0 | 0 | 0 |
| 846 | 0 | 0 | 0.971426 | 0.028574 | 0 | 0 | 0 | 0 | 0 |
| 847 | 0 | 0 | 0.918301 | 0.081699 | 0 | 0 | 0 | 0 | 0 |
| 848 | 0 | 0 | 0.926334 | 0.073666 | 0 | 0 | 0 | 0 | 0 |
| 849 | 0.00001 | 0.00002 | 0.928236 | 0.071734 | 0 | 0 | 0 | 0 | 0 |
| 850 | 0 | 0 | 0.922384 | 0.077616 | 0 | 0 | 0 | 0 | 0 |
| 851 | 0 | 0 | 0.922819 | 0.077181 | 0 | 0 | 0 | 0 | 0 |
| 852 | 0.00001 | 0 | 0.920535 | 0.079455 | 0 | 0 | 0 | 0 | 0 |
| 853 | 0.00001 | 0.00001 | 0.919104 | 0.080876 | 0 | 0 | 0 | 0 | 0 |
| 854 | 0.00002 | 0 | 0.921861 | 0.078119 | 0 | 0 | 0 | 0 | 0 |
| 855 | 0.00001 | 0 | 0.919714 | 0.080276 | 0 | 0 | 0 | 0 | 0 |
| 856 | 0.00003 | 0 | 0.920964 | 0.079006 | 0 | 0 | 0 | 0 | 0 |
| 857 | 0.00001 | 0.00001 | 0.924002 | 0.075978 | 0 | 0 | 0 | 0 | 0 |
| 858 | 0 | 0 | 0.922841 | 0.077159 | 0 | 0 | 0 | 0 | 0 |
| 859 | 0.00001 | 0.00001 | 0.923143 | 0.076837 | 0 | 0 | 0 | 0 | 0 |
| 860 | 0 | 0 | 0.919322 | 0.080678 | 0 | 0 | 0 | 0 | 0 |
| 861 | 0.00001 | 0 | 0.923413 | 0.076356 | 0 | 0 | 0 | 0 | 0 |
| 862 | 0.00001 | 0 | 0.922182 | 0.077808 | 0 | 0 | 0 | 0 | 0 |
| 863 | 0.00001 | 0 | 0.920666 | 0.079324 | 0 | 0 | 0 | 0 | 0 |
| 864 | 0.00001 | 0 | 0.92561 | 0.07438 | 0 | 0 | 0 | 0 | 0 |
| 865 | 0 | 0 | 0.922055 | 0.077945 | 0 | 0 | 0 | 0 | 0 |
| 866 | 0.00001 | 0 | 0.922189 | 0.077801 | 0 | 0 | 0 | 0 | 0 |
| 867 | 0.00001 | 0 | 0.924011 | 0.075979 | 0 | 0 | 0 | 0 | 0 |
| 868 | 0.00002 | 0.00003 | 0.918773 | 0.081177 | 0 | 0 | 0 | 0 | 0 |
| 869 | 0.00004 | 0 | 0.921028 | 0.078932 | 0 | 0 | 0 | 0 | 0 |
| 870 | 0.00002 | 0 | 0.92362 | 0.07636 | 0 | 0 | 0 | 0 | 0 |
| 871 | 0.00001 | 0.00004 | 0.928653 | 0.071297 | 0 | 0 | 0 | 0 | 0 |
| 872 | 0.00003 | 0 | 0.921256 | 0.078713 | 0 | 0 | 0 | 0 | 0 |
| 873 | 0 | 0 | 0.919065 | 0.080935 | 0 | 0 | 0 | 0 | 0 |
| 874 | 0.00002 | 0 | 0.922018 | 0.077962 | 0 | 0 | 0 | 0 | 0 |
| 875 | 0.00003 | 0 | 0.924516 | 0.075454 | 0 | 0 | 0 | 0 | 0 |
| 876 | 0.00001 | 0 | 0.922295 | 0.077695 | 0 | 0 | 0 | 0 | 0 |
| 877 | 0.00004 | 0 | 0.926065 | 0.073895 | 0 | 0 | 0 | 0 | 0 |
| 878 | 0 | 0 | 0.925911 | 0.074089 | 0 | 0 | 0 | 0 | 0 |
| 879 | 0 | 0 | 0.921608 | 0.078392 | 0 | 0 | 0 | 0 | 0 |
| 880 | 0.00002 | 0 | 0.924057 | 0.075923 | 0 | 0 | 0 | 0 | 0 |
| 881 | 0 | 0 | 0.92104 | 0.07896 | 0 | 0 | 0 | 0 | 0 |
| 882 | 0.00001 | 0 | 0.921366 | 0.078624 | 0 | 0 | 0 | 0 | 0 |
| 883 | 0.00002 | 0.00001 | 0.921502 | 0.078468 | 0 | 0 | 0 | 0 | 0 |
| 884 | 0.00001 | 0 | 0.921998 | 0.077992 | 0 | 0 | 0 | 0 | 0 |
| 885 | 0.00001 | 0 | 0.927544 | 0.072446 | 0 | 0 | 0 | 0 | 0 |
| 886 | 0.00003 | 0 | 0.92164 | 0.07833 | 0 | 0 | 0 | 0 | 0 |
| 887 | 0.00002 | 0.00001 | 0.923046 | 0.076925 | 0 | 0 | 0 | 0 | 0 |
| 888 | 0.00003 | 0 | 0.918828 | 0.081141 | 0 | 0 | 0 | 0 | 0 |
| 889 | 0 | 0.00002 | 0.91601 | 0.08397 | 0 | 0 | 0 | 0 | 0 |
| 890 | 0.00002 | 0 | 0.926564 | 0.073416 | 0 | 0 | 0 | 0 | 0 |
| 891 | 0.00002 | 0.00002 | 0.9181 | 0.08186 | 0 | 0 | 0 | 0 | 0 |
| 892 | 0.00001 | 0 | 0.922675 | 0.077315 | 0 | 0 | 0 | 0 | 0 |
| 893 | 0 | 0.00001 | 0.921265 | 0.078725 | 0 | 0 | 0 | 0 | 0 |
| 894 | 0.00001 | 0 | 0.919654 | 0.080336 | 0 | 0 | 0 | 0 | 0 |
| 895 | 0.00001 | 0.00001 | 0.927991 | 0.071989 | 0 | 0 | 0 | 0 | 0 |
| 896 | 0 | 0 | 0.921232 | 0.078768 | 0 | 0 | 0 | 0 | 0 |
| 897 | 0.00002 | 0 | 0.925245 | 0.074735 | 0 | 0 | 0 | 0 | 0 |
| 898 | 0.00001 | 0 | 0.924228 | 0.075762 | 0 | 0 | 0 | 0 | 0 |
| 899 | 0.00003 | 0 | 0.924998 | 0.074972 | 0 | 0 | 0 | 0 | 0 |
| 900 | 0 | 0 | 0.923197 | 0.076803 | 0 | 0 | 0 | 0 | 0 |
| 901 | 0.00001 | 0.00001 | 0.926659 | 0.073321 | 0 | 0 | 0 | 0 | 0 |
| 902 | 0.00001 | 0 | 0.919541 | 0.080449 | 0 | 0 | 0 | 0 | 0 |
| 903 | 0 | 0 | 0.92138 | 0.07862 | 0 | 0 | 0 | 0 | 0 |
| 904 | 0 | 0 | 0.927189 | 0.072811 | 0 | 0 | 0 | 0 | 0 |
| 905 | 0.00001 | 0.00001 | 0.924191 | 0.075789 | 0 | 0 | 0 | 0 | 0 |
| 906 | 0 | 0 | 0.92332 | 0.07668 | 0 | 0 | 0 | 0 | 0 |
| 907 | 0.00001 | 0 | 0.928937 | 0.071053 | 0 | 0 | 0 | 0 | 0 |
| 908 | 0.00002 | 0 | 0.92206 | 0.07792 | 0 | 0 | 0 | 0 | 0 |
| 909 | 0.00001 | 0 | 0.923093 | 0.076897 | 0 | 0 | 0 | 0 | 0 |
| 910 | 0 | 0 | 0.921978 | 0.078022 | 0 | 0 | 0 | 0 | 0 |
| 911 | 0.00003 | 0 | 0.926121 | 0.073849 | 0 | 0 | 0 | 0 | 0 |
| 912 | 0.00001 | 0 | 0.919208 | 0.080782 | 0 | 0 | 0 | 0 | 0 |
| 913 | 0.00002 | 0.00002 | 0.922366 | 0.077594 | 0 | 0 | 0 | 0 | 0 |
| 914 | 0.00001 | 0.00001 | 0.926682 | 0.073298 | 0 | 0 | 0 | 0 | 0 |
| 915 | 0.00002 | 0 | 0.9184 | 0.081579 | 0 | 0 | 0 | 0 | 0 |
| 916 | 0 | 0.00001 | 0.922152 | 0.077838 | 0 | 0 | 0 | 0 | 0 |
| 917 | 0.00001 | 0.00002 | 0.92243 | 0.07754 | 0 | 0 | 0 | 0 | 0 |
| 918 | 0.00002 | 0 | 0.92619 | 0.07379 | 0 | 0 | 0 | 0 | 0 |
| 919 | 0.00004 | 0.00001 | 0.9187 | 0.081251 | 0 | 0 | 0 | 0 | 0 |
| 920 | 0.00001 | 0 | 0.927275 | 0.072714 | 0 | 0 | 0 | 0 | 0 |
| 921 | 0 | 0.00004 | 0.924153 | 0.075807 | 0 | 0 | 0 | 0 | 0 |
| 922 | 0.00001 | 0.00001 | 0.923351 | 0.076629 | 0 | 0 | 0 | 0 | 0 |
| 923 | 0.00002 | 0 | 0.922166 | 0.077814 | 0 | 0 | 0 | 0 | 0 |
| 924 | 0 | 0.00001 | 0.923817 | 0.076173 | 0 | 0 | 0 | 0 | 0 |
| 925 | 0.00001 | 0.00001 | 0.922054 | 0.077926 | 0 | 0 | 0 | 0 | 0 |
| 926 | 0.00001 | 0 | 0.92483 | 0.07516 | 0 | 0 | 0 | 0 | 0 |
| 927 | 0.00003 | 0 | 0.921119 | 0.078851 | 0 | 0 | 0 | 0 | 0 |
| 928 | 0 | 0 | 0.92268 | 0.07732 | 0 | 0 | 0 | 0 | 0 |
| 929 | 0.00001 | 0.00002 | 0.917747 | 0.082223 | 0 | 0 | 0 | 0 | 0 |
| 930 | 0.00001 | 0 | 0.92344 | 0.07655 | 0 | 0 | 0 | 0 | 0 |
| 931 | 0 | 0 | 0.92543 | 0.07457 | 0 | 0 | 0 | 0 | 0 |
| 932 | 0.00003 | 0 | 0.924908 | 0.075062 | 0 | 0 | 0 | 0 | 0 |
| 933 | 0.00002 | 0 | 0.92713 | 0.07285 | 0 | 0 | 0 | 0 | 0 |
| 934 | 0.00002 | 0 | 0.919951 | 0.080029 | 0 | 0 | 0 | 0 | 0 |
| 935 | 0.00002 | 0 | 0.922945 | 0.077035 | 0 | 0 | 0 | 0 | 0 |
| 936 | 0 | 0 | 0.92395 | 0.07605 | 0 | 0 | 0 | 0 | 0 |
| 937 | 0 | 0 | 0.924465 | 0.075535 | 0 | 0 | 0 | 0 | 0 |
| 938 | 0.00002 | 0 | 0.925537 | 0.074443 | 0 | 0 | 0 | 0 | 0 |
| 939 | 0.00001 | 0 | 0.925634 | 0.074356 | 0 | 0 | 0 | 0 | 0 |
| 940 | 0.00001 | 0 | 0.91935 | 0.08064 | 0 | 0 | 0 | 0 | 0 |
| 941 | 0.00001 | 0 | 0.924755 | 0.075235 | 0 | 0 | 0 | 0 | 0 |
| 942 | 0.00002 | 0 | 0.923556 | 0.076424 | 0 | 0 | 0 | 0 | 0 |
| 943 | 0 | 0 | 0.925258 | 0.074742 | 0 | 0 | 0 | 0 | 0 |
| 944 | 0.00003 | 0 | 0.923464 | 0.076507 | 0 | 0 | 0 | 0 | 0 |
| 945 | 0 | 0 | 0.918872 | 0.081128 | 0 | 0 | 0 | 0 | 0 |
| 946 | 0.00003 | 0 | 0.922205 | 0.077765 | 0 | 0 | 0 | 0 | 0 |
| 947 | 0.00005 | 0 | 0.920796 | 0.079154 | 0 | 0 | 0 | 0 | 0 |
| 948 | 0 | 0 | 0.922186 | 0.077814 | 0 | 0 | 0 | 0 | 0 |
| 949 | 0.00002 | 0 | 0.926078 | 0.073902 | 0 | 0 | 0 | 0 | 0 |
| 950 | 0.00002 | 0 | 0.925143 | 0.074837 | 0 | 0 | 0 | 0 | 0 |
| 951 | 0.00001 | 0.00002 | 0.926322 | 0.073648 | 0 | 0 | 0 | 0 | 0 |
| 952 | 0 | 0 | 0.924558 | 0.075442 | 0 | 0 | 0 | 0 | 0 |
| 953 | 0 | 0 | 0.922533 | 0.077467 | 0 | 0 | 0 | 0 | 0 |
| 954 | 0.00001 | 0.00001 | 0.91794 | 0.08204 | 0 | 0 | 0 | 0 | 0 |
| 955 | 0 | 0 | 0.925689 | 0.074311 | 0 | 0 | 0 | 0 | 0 |
| 956 | 0.00001 | 0 | 0.926952 | 0.073038 | 0 | 0 | 0 | 0 | 0 |
| 957 | 0.00001 | 0 | 0.917792 | 0.082198 | 0 | 0 | 0 | 0 | 0 |
| 958 | 0 | 0 | 0.924741 | 0.075259 | 0 | 0 | 0 | 0 | 0 |
| 959 | 0 | 0 | 0.921154 | 0.078846 | 0 | 0 | 0 | 0 | 0 |
| 960 | 0 | 0.00001 | 0.92616 | 0.07383 | 0 | 0 | 0 | 0 | 0 |
| 961 | 0.00001 | 0 | 0.920757 | 0.079233 | 0 | 0 | 0 | 0 | 0 |
| 962 | 0.00001 | 0 | 0.922974 | 0.077016 | 0 | 0 | 0 | 0 | 0 |
| 963 | 0.00001 | 0 | 0.922728 | 0.077262 | 0 | 0 | 0 | 0 | 0 |
| 964 | 0.00001 | 0 | 0.924009 | 0.075981 | 0 | 0 | 0 | 0 | 0 |
| 965 | 0.00001 | 0 | 0.926271 | 0.073719 | 0 | 0 | 0 | 0 | 0 |
| 966 | 0.00002 | 0 | 0.925023 | 0.074957 | 0 | 0 | 0 | 0 | 0 |
| 967 | 0 | 0.00002 | 0.926752 | 0.073228 | 0 | 0 | 0 | 0 | 0 |
| 968 | 0.00002 | 0 | 0.920493 | 0.079486 | 0 | 0 | 0 | 0 | 0 |
| 969 | 0 | 0.00002 | 0.920271 | 0.079709 | 0 | 0 | 0 | 0 | 0 |
| 970 | 0 | 0 | 0.919669 | 0.080331 | 0 | 0 | 0 | 0 | 0 |
| 971 | 0 | 0.00003 | 0.925418 | 0.074552 | 0 | 0 | 0 | 0 | 0 |
| 972 | 0.00002 | 0 | 0.921143 | 0.078837 | 0 | 0 | 0 | 0 | 0 |
| 973 | 0.00001 | 0.00003 | 0.923789 | 0.076171 | 0 | 0 | 0 | 0 | 0 |
| 974 | 0.00002 | 0 | 0.92007 | 0.07991 | 0 | 0 | 0 | 0 | 0 |
| 975 | 0.00001 | 0 | 0.926397 | 0.073593 | 0 | 0 | 0 | 0 | 0 |
| 976 | 0.00001 | 0.00001 | 0.922653 | 0.077327 | 0 | 0 | 0 | 0 | 0 |
| 977 | 0.00001 | 0 | 0.92098 | 0.07901 | 0 | 0 | 0 | 0 | 0 |
| 978 | 0.00001 | 0 | 0.922471 | 0.077519 | 0 | 0 | 0 | 0 | 0 |
| 979 | 0.00002 | 0 | 0.921286 | 0.078695 | 0 | 0 | 0 | 0 | 0 |
| 980 | 0.00001 | 0 | 0.922513 | 0.077477 | 0 | 0 | 0 | 0 | 0 |
| 981 | 0.00001 | 0 | 0.924399 | 0.075591 | 0 | 0 | 0 | 0 | 0 |
| 982 | 0 | 0 | 0.917525 | 0.082475 | 0 | 0 | 0 | 0 | 0 |
| 983 | 0 | 0 | 0.92054 | 0.07946 | 0 | 0 | 0 | 0 | 0 |
| 984 | 0 | 0 | 0.922672 | 0.077328 | 0 | 0 | 0 | 0 | 0 |
| 985 | 0.00004 | 0 | 0.921233 | 0.078727 | 0 | 0 | 0 | 0 | 0 |
| 986 | 0 | 0 | 0.926151 | 0.073849 | 0 | 0 | 0 | 0 | 0 |
| 987 | 0.00001 | 0 | 0.925492 | 0.074498 | 0 | 0 | 0 | 0 | 0 |
| 988 | 0 | 0 | 0.924522 | 0.075478 | 0 | 0 | 0 | 0 | 0 |
| 989 | 0.00002 | 0 | 0.920511 | 0.079469 | 0 | 0 | 0 | 0 | 0 |
| 990 | 0 | 0.00001 | 0.918634 | 0.081356 | 0 | 0 | 0 | 0 | 0 |
| 991 | 0.00001 | 0 | 0.923587 | 0.076403 | 0 | 0 | 0 | 0 | 0 |
| 992 | 0.00002 | 0.00001 | 0.924617 | 0.075353 | 0 | 0 | 0 | 0 | 0 |
| 993 | 0.00002 | 0 | 0.925967 | 0.074013 | 0 | 0 | 0 | 0 | 0 |
| 994 | 0.00002 | 0.00002 | 0.926915 | 0.073045 | 0 | 0 | 0 | 0 | 0 |
| 995 | 0 | 0.00001 | 0.920421 | 0.079569 | 0 | 0 | 0 | 0 | 0 |
| 996 | 0.00001 | 0 | 0.925457 | 0.074533 | 0 | 0 | 0 | 0 | 0 |
| 997 | 0.00002 | 0 | 0.919408 | 0.080572 | 0 | 0 | 0 | 0 | 0 |
| 998 | 0.00003 | 0 | 0.921496 | 0.078474 | 0 | 0 | 0 | 0 | 0 |
| 999 | 0.00002 | 0 | 0.926686 | 0.073294 | 0 | 0 | 0 | 0 | 0 |
| 1000 | 0.00001 | 0.00001 | 0.921221 | 0.078759 | 0 | 0 | 0 | 0 | 0 |

| Year 12 | Race |  |  |  |  |  |  |  |  |
| --- | --- | --- | --- | --- | --- | --- | --- | --- | --- |
| Trial | 003.0 | 005.0 | 007.0 | 037.1 | 043.0 | 303.0 | 003.2 | 403.0 | 003.4 |
| 1 | 0.00001 | 0 | 0.928207 | 0.071783 | 0 | 0 | 0 | 0 | 0 |
| 2 | 0 | 0 | 0.924363 | 0.075637 | 0 | 0 | 0 | 0 | 0 |
| 3 | 0 | 0 | 0.932257 | 0.067743 | 0 | 0 | 0 | 0 | 0 |
| 4 | 0.00001 | 0 | 0.929189 | 0.070801 | 0 | 0 | 0 | 0 | 0 |
| 5 | 0.00001 | 0 | 0.927022 | 0.072968 | 0 | 0 | 0 | 0 | 0 |
| 6 | 0 | 0 | 0.92954 | 0.07046 | 0 | 0 | 0 | 0 | 0 |
| 7 | 0.00002 | 0 | 0.927997 | 0.071983 | 0 | 0 | 0 | 0 | 0 |
| 8 | 0.00003 | 0 | 0.930711 | 0.06926 | 0 | 0 | 0 | 0 | 0 |
| 9 | 0.00001 | 0 | 0.925991 | 0.073999 | 0 | 0 | 0 | 0 | 0 |
| 10 | 0.00001 | 0.00002 | 0.928437 | 0.071533 | 0 | 0 | 0 | 0 | 0 |
| 11 | 0.00001 | 0 | 0.93211 | 0.06788 | 0 | 0 | 0 | 0 | 0 |
| 12 | 0.00001 | 0 | 0.92614 | 0.07385 | 0 | 0 | 0 | 0 | 0 |
| 13 | 0.00002 | 0 | 0.930458 | 0.069522 | 0 | 0 | 0 | 0 | 0 |
| 14 | 0.00001 | 0 | 0.92489 | 0.0751 | 0 | 0 | 0 | 0 | 0 |
| 15 | 0 | 0 | 0.925332 | 0.074668 | 0 | 0 | 0 | 0 | 0 |
| 16 | 0 | 0 | 0.927867 | 0.072133 | 0 | 0 | 0 | 0 | 0 |
| 17 | 0 | 0 | 0.928829 | 0.071171 | 0 | 0 | 0 | 0 | 0 |
| 18 | 0.00002 | 0 | 0.928433 | 0.071547 | 0 | 0 | 0 | 0 | 0 |
| 19 | 0.00002 | 0 | 0.927303 | 0.072677 | 0 | 0 | 0 | 0 | 0 |
| 20 | 0.00001 | 0 | 0.929636 | 0.070354 | 0 | 0 | 0 | 0 | 0 |
| 21 | 0.00002 | 0 | 0.928619 | 0.071361 | 0 | 0 | 0 | 0 | 0 |
| 22 | 0 | 0 | 0.927447 | 0.072553 | 0 | 0 | 0 | 0 | 0 |
| 23 | 0.00001 | 0 | 0.931168 | 0.068822 | 0 | 0 | 0 | 0 | 0 |
| 24 | 0.00003 | 0 | 0.929393 | 0.070577 | 0 | 0 | 0 | 0 | 0 |
| 25 | 0 | 0 | 0.926948 | 0.073052 | 0 | 0 | 0 | 0 | 0 |
| 26 | 0.00001 | 0 | 0.930866 | 0.069124 | 0 | 0 | 0 | 0 | 0 |
| 27 | 0.00001 | 0 | 0.927723 | 0.072268 | 0 | 0 | 0 | 0 | 0 |
| 28 | 0.00003 | 0 | 0.932406 | 0.067544 | 0 | 0 | 0 | 0 | 0 |
| 29 | 0.00001 | 0 | 0.930443 | 0.069547 | 0 | 0 | 0 | 0 | 0 |
| 30 | 0.00002 | 0.00001 | 0.926085 | 0.073885 | 0 | 0 | 0 | 0 | 0 |
| 31 | 0.00003 | 0 | 0.931384 | 0.068586 | 0 | 0 | 0 | 0 | 0 |
| 32 | 0.00004 | 0 | 0.929596 | 0.070364 | 0 | 0 | 0 | 0 | 0 |
| 33 | 0 | 0 | 0.928075 | 0.071925 | 0 | 0 | 0 | 0 | 0 |
| 34 | 0 | 0 | 0.929384 | 0.070616 | 0 | 0 | 0 | 0 | 0 |
| 35 | 0.00003 | 0 | 0.925353 | 0.074617 | 0 | 0 | 0 | 0 | 0 |
| 36 | 0.00001 | 0 | 0.927147 | 0.072843 | 0 | 0 | 0 | 0 | 0 |
| 37 | 0.00001 | 0 | 0.933785 | 0.066205 | 0 | 0 | 0 | 0 | 0 |
| 38 | 0.00001 | 0 | 0.930055 | 0.069935 | 0 | 0 | 0 | 0 | 0 |
| 39 | 0.00003 | 0 | 0.927769 | 0.072201 | 0 | 0 | 0 | 0 | 0 |
| 40 | 0.00002 | 0 | 0.92544 | 0.07454 | 0 | 0 | 0 | 0 | 0 |
| 41 | 0.00001 | 0 | 0.924079 | 0.075911 | 0 | 0 | 0 | 0 | 0 |
| 42 | 0 | 0 | 0.929376 | 0.070624 | 0 | 0 | 0 | 0 | 0 |
| 43 | 0.00003 | 0 | 0.929044 | 0.070926 | 0 | 0 | 0 | 0 | 0 |
| 44 | 0 | 0.00001 | 0.927961 | 0.072029 | 0 | 0 | 0 | 0 | 0 |
| 45 | 0 | 0 | 0.929997 | 0.070003 | 0 | 0 | 0 | 0 | 0 |
| 46 | 0.00002 | 0 | 0.92758 | 0.0724 | 0 | 0 | 0 | 0 | 0 |
| 47 | 0.00001 | 0 | 0.929511 | 0.070479 | 0 | 0 | 0 | 0 | 0 |
| 48 | 0.00001 | 0 | 0.926253 | 0.073737 | 0 | 0 | 0 | 0 | 0 |
| 49 | 0 | 0.00003 | 0.930484 | 0.069486 | 0 | 0 | 0 | 0 | 0 |
| 50 | 0.00001 | 0.00002 | 0.930535 | 0.069435 | 0 | 0 | 0 | 0 | 0 |
| 51 | 0.00003 | 0 | 0.928156 | 0.071814 | 0 | 0 | 0 | 0 | 0 |
| 52 | 0.00002 | 0.00002 | 0.932194 | 0.067766 | 0 | 0 | 0 | 0 | 0 |
| 53 | 0.00001 | 0 | 0.928096 | 0.071894 | 0 | 0 | 0 | 0 | 0 |
| 54 | 0.00001 | 0 | 0.928082 | 0.071908 | 0 | 0 | 0 | 0 | 0 |
| 55 | 0.00001 | 0 | 0.924866 | 0.075124 | 0 | 0 | 0 | 0 | 0 |
| 56 | 0 | 0 | 0.929994 | 0.070006 | 0 | 0 | 0 | 0 | 0 |
| 57 | 0.00002 | 0 | 0.930408 | 0.069572 | 0 | 0 | 0 | 0 | 0 |
| 58 | 0.00001 | 0 | 0.926591 | 0.073399 | 0 | 0 | 0 | 0 | 0 |
| 59 | 0.00002 | 0 | 0.932087 | 0.067893 | 0 | 0 | 0 | 0 | 0 |
| 60 | 0 | 0 | 0.929125 | 0.070875 | 0 | 0 | 0 | 0 | 0 |
| 61 | 0.00003 | 0 | 0.924688 | 0.075282 | 0 | 0 | 0 | 0 | 0 |
| 62 | 0.00003 | 0 | 0.927182 | 0.072788 | 0 | 0 | 0 | 0 | 0 |
| 63 | 0.00001 | 0 | 0.927303 | 0.072687 | 0 | 0 | 0 | 0 | 0 |
| 64 | 0.00001 | 0 | 0.925686 | 0.074304 | 0 | 0 | 0 | 0 | 0 |
| 65 | 0 | 0 | 0.930658 | 0.069342 | 0 | 0 | 0 | 0 | 0 |
| 66 | 0 | 0 | 0.932774 | 0.067226 | 0 | 0 | 0 | 0 | 0 |
| 67 | 0.00001 | 0 | 0.92992 | 0.07007 | 0 | 0 | 0 | 0 | 0 |
| 68 | 0.00003 | 0 | 0.928228 | 0.071742 | 0 | 0 | 0 | 0 | 0 |
| 69 | 0 | 0 | 0.927477 | 0.072523 | 0 | 0 | 0 | 0 | 0 |
| 70 | 0.00001 | 0 | 0.929202 | 0.070788 | 0 | 0 | 0 | 0 | 0 |
| 71 | 0.00001 | 0 | 0.926076 | 0.073834 | 0 | 0 | 0 | 0 | 0 |
| 72 | 0.00002 | 0 | 0.932086 | 0.067894 | 0 | 0 | 0 | 0 | 0 |
| 73 | 0 | 0 | 0.927063 | 0.072937 | 0 | 0 | 0 | 0 | 0 |
| 74 | 0.00002 | 0 | 0.931065 | 0.068915 | 0 | 0 | 0 | 0 | 0 |
| 75 | 0.00003 | 0.00002 | 0.927256 | 0.072694 | 0 | 0 | 0 | 0 | 0 |
| 76 | 0.00001 | 0 | 0.930915 | 0.069075 | 0 | 0 | 0 | 0 | 0 |
| 77 | 0 | 0 | 0.928238 | 0.071762 | 0 | 0 | 0 | 0 | 0 |
| 78 | 0 | 0 | 0.929022 | 0.070978 | 0 | 0 | 0 | 0 | 0 |
| 79 | 0.00004 | 0 | 0.9303 | 0.06966 | 0 | 0 | 0 | 0 | 0 |
| 80 | 0 | 0 | 0.927278 | 0.072722 | 0 | 0 | 0 | 0 | 0 |
| 81 | 0.00001 | 0.00001 | 0.928091 | 0.071889 | 0 | 0 | 0 | 0 | 0 |
| 82 | 0 | 0 | 0.92282 | 0.07718 | 0 | 0 | 0 | 0 | 0 |
| 83 | 0 | 0 | 0.933054 | 0.066946 | 0 | 0 | 0 | 0 | 0 |
| 84 | 0.00002 | 0 | 0.929289 | 0.070692 | 0 | 0 | 0 | 0 | 0 |
| 85 | 0.00001 | 0 | 0.926766 | 0.073224 | 0 | 0 | 0 | 0 | 0 |
| 86 | 0.00002 | 0 | 0.936627 | 0.063353 | 0 | 0 | 0 | 0 | 0 |
| 87 | 0.00001 | 0 | 0.929308 | 0.070682 | 0 | 0 | 0 | 0 | 0 |
| 88 | 0.00003 | 0 | 0.927913 | 0.072058 | 0 | 0 | 0 | 0 | 0 |
| 89 | 0.00001 | 0 | 0.926166 | 0.073824 | 0 | 0 | 0 | 0 | 0 |
| 90 | 0.00001 | 0 | 0.927691 | 0.072299 | 0 | 0 | 0 | 0 | 0 |
| 91 | 0.00001 | 0 | 0.928279 | 0.071711 | 0 | 0 | 0 | 0 | 0 |
| 92 | 0.00002 | 0 | 0.929109 | 0.070871 | 0 | 0 | 0 | 0 | 0 |
| 93 | 0.00002 | 0 | 0.932189 | 0.067791 | 0 | 0 | 0 | 0 | 0 |
| 94 | 0 | 0.00001 | 0.931001 | 0.068989 | 0 | 0 | 0 | 0 | 0 |
| 95 | 0 | 0 | 0.92771 | 0.07229 | 0 | 0 | 0 | 0 | 0 |
| 96 | 0.00002 | 0 | 0.930681 | 0.069299 | 0 | 0 | 0 | 0 | 0 |
| 97 | 0.00002 | 0 | 0.927423 | 0.072557 | 0 | 0 | 0 | 0 | 0 |
| 98 | 0 | 0 | 0.9265 | 0.0735 | 0 | 0 | 0 | 0 | 0 |
| 99 | 0.00002 | 0 | 0.923544 | 0.076436 | 0 | 0 | 0 | 0 | 0 |
| 100 | 0.00001 | 0 | 0.93177 | 0.06822 | 0 | 0 | 0 | 0 | 0 |
| 101 | 0.00002 | 0 | 0.931032 | 0.068948 | 0 | 0 | 0 | 0 | 0 |
| 102 | 0.00001 | 0 | 0.930771 | 0.069219 | 0 | 0 | 0 | 0 | 0 |
| 103 | 0.00003 | 0.00001 | 0.928992 | 0.070968 | 0 | 0 | 0 | 0 | 0 |
| 104 | 0.00001 | 0 | 0.930038 | 0.069952 | 0 | 0 | 0 | 0 | 0 |
| 105 | 0.00001 | 0 | 0.929725 | 0.070265 | 0 | 0 | 0 | 0 | 0 |
| 106 | 0 | 0.00002 | 0.928985 | 0.070995 | 0 | 0 | 0 | 0 | 0 |
| 107 | 0 | 0.00001 | 0.932036 | 0.067924 | 0 | 0 | 0 | 0 | 0 |
| 108 | 0 | 0 | 0.923636 | 0.076364 | 0 | 0 | 0 | 0 | 0 |
| 109 | 0 | 0.00002 | 0.930883 | 0.069097 | 0 | 0 | 0 | 0 | 0 |
| 110 | 0.00002 | 0 | 0.927888 | 0.072092 | 0 | 0 | 0 | 0 | 0 |
| 111 | 0.00001 | 0 | 0.930482 | 0.069508 | 0 | 0 | 0 | 0 | 0 |
| 112 | 0 | 0 | 0.927748 | 0.072252 | 0 | 0 | 0 | 0 | 0 |
| 113 | 0.00001 | 0 | 0.927931 | 0.072059 | 0 | 0 | 0 | 0 | 0 |
| 114 | 0.00001 | 0 | 0.927447 | 0.072543 | 0 | 0 | 0 | 0 | 0 |
| 115 | 0.00003 | 0 | 0.93238 | 0.06759 | 0 | 0 | 0 | 0 | 0 |
| 116 | 0.00002 | 0 | 0.927092 | 0.072888 | 0 | 0 | 0 | 0 | 0 |
| 117 | 0.00004 | 0 | 0.928629 | 0.071331 | 0 | 0 | 0 | 0 | 0 |
| 118 | 0 | 0 | 0.929668 | 0.070332 | 0 | 0 | 0 | 0 | 0 |
| 119 | 0.00002 | 0 | 0.926303 | 0.073677 | 0 | 0 | 0 | 0 | 0 |
| 120 | 0 | 0 | 0.927166 | 0.072834 | 0 | 0 | 0 | 0 | 0 |
| 121 | 0.00001 | 0 | 0.929132 | 0.070858 | 0 | 0 | 0 | 0 | 0 |
| 122 | 0 | 0 | 0.932027 | 0.067973 | 0 | 0 | 0 | 0 | 0 |
| 123 | 0.00001 | 0 | 0.930278 | 0.069712 | 0 | 0 | 0 | 0 | 0 |
| 124 | 0 | 0 | 0.929245 | 0.070755 | 0 | 0 | 0 | 0 | 0 |
| 125 | 0.00002 | 0 | 0.92777 | 0.07221 | 0 | 0 | 0 | 0 | 0 |
| 126 | 0 | 0 | 0.92952 | 0.07048 | 0 | 0 | 0 | 0 | 0 |
| 127 | 0.00001 | 0 | 0.929849 | 0.070141 | 0 | 0 | 0 | 0 | 0 |
| 128 | 0 | 0 | 0.927646 | 0.072354 | 0 | 0 | 0 | 0 | 0 |
| 129 | 0 | 0 | 0.928521 | 0.071479 | 0 | 0 | 0 | 0 | 0 |
| 130 | 0.00001 | 0 | 0.929795 | 0.070195 | 0 | 0 | 0 | 0 | 0 |
| 131 | 0.00001 | 0 | 0.929928 | 0.070062 | 0 | 0 | 0 | 0 | 0 |
| 132 | 0 | 0 | 0.928636 | 0.071364 | 0 | 0 | 0 | 0 | 0 |
| 133 | 0 | 0 | 0.926256 | 0.073744 | 0 | 0 | 0 | 0 | 0 |
| 134 | 0.00002 | 0 | 0.930809 | 0.069171 | 0 | 0 | 0 | 0 | 0 |
| 135 | 0.00001 | 0 | 0.925287 | 0.074703 | 0 | 0 | 0 | 0 | 0 |
| 136 | 0.00004 | 0.00001 | 0.930209 | 0.069741 | 0 | 0 | 0 | 0 | 0 |
| 137 | 0.00003 | 0 | 0.929168 | 0.070802 | 0 | 0 | 0 | 0 | 0 |
| 138 | 0.00001 | 0.00001 | 0.926461 | 0.073519 | 0 | 0 | 0 | 0 | 0 |
| 139 | 0.00006 | 0 | 0.93069 | 0.06925 | 0 | 0 | 0 | 0 | 0 |
| 140 | 0.00001 | 0 | 0.926406 | 0.073584 | 0 | 0 | 0 | 0 | 0 |
| 141 | 0.00003 | 0 | 0.92737 | 0.0726 | 0 | 0 | 0 | 0 | 0 |
| 142 | 0.00001 | 0 | 0.924938 | 0.075052 | 0 | 0 | 0 | 0 | 0 |
| 143 | 0 | 0 | 0.929127 | 0.070873 | 0 | 0 | 0 | 0 | 0 |
| 144 | 0.00002 | 0 | 0.926643 | 0.073336 | 0 | 0 | 0 | 0 | 0 |
| 145 | 0 | 0 | 0.93031 | 0.06969 | 0 | 0 | 0 | 0 | 0 |
| 146 | 0 | 0 | 0.933203 | 0.066797 | 0 | 0 | 0 | 0 | 0 |
| 147 | 0.00002 | 0 | 0.927469 | 0.072511 | 0 | 0 | 0 | 0 | 0 |
| 148 | 0.00001 | 0 | 0.928967 | 0.071023 | 0 | 0 | 0 | 0 | 0 |
| 149 | 0.00001 | 0 | 0.930464 | 0.069526 | 0 | 0 | 0 | 0 | 0 |
| 150 | 0.00002 | 0 | 0.932635 | 0.067345 | 0 | 0 | 0 | 0 | 0 |
| 151 | 0.00002 | 0 | 0.93046 | 0.06952 | 0 | 0 | 0 | 0 | 0 |
| 152 | 0 | 0 | 0.926647 | 0.073353 | 0 | 0 | 0 | 0 | 0 |
| 153 | 0.00001 | 0 | 0.931607 | 0.068383 | 0 | 0 | 0 | 0 | 0 |
| 154 | 0.00001 | 0 | 0.92871 | 0.07128 | 0 | 0 | 0 | 0 | 0 |
| 155 | 0 | 0 | 0.92833 | 0.07167 | 0 | 0 | 0 | 0 | 0 |
| 156 | 0.00001 | 0 | 0.930684 | 0.069306 | 0 | 0 | 0 | 0 | 0 |
| 157 | 0.00002 | 0 | 0.925627 | 0.074353 | 0 | 0 | 0 | 0 | 0 |
| 158 | 0 | 0 | 0.927027 | 0.072973 | 0 | 0 | 0 | 0 | 0 |
| 159 | 0.00001 | 0 | 0.927438 | 0.072552 | 0 | 0 | 0 | 0 | 0 |
| 160 | 0.00003 | 0 | 0.93024 | 0.06973 | 0 | 0 | 0 | 0 | 0 |
| 161 | 0.00002 | 0 | 0.926828 | 0.073152 | 0 | 0 | 0 | 0 | 0 |
| 162 | 0 | 0 | 0.928845 | 0.071155 | 0 | 0 | 0 | 0 | 0 |
| 163 | 0 | 0 | 0.928071 | 0.071929 | 0 | 0 | 0 | 0 | 0 |
| 164 | 0.00001 | 0 | 0.922879 | 0.077111 | 0 | 0 | 0 | 0 | 0 |
| 165 | 0.00002 | 0 | 0.935062 | 0.064918 | 0 | 0 | 0 | 0 | 0 |
| 166 | 0.00001 | 0 | 0.92949 | 0.0705 | 0 | 0 | 0 | 0 | 0 |
| 167 | 0.00002 | 0 | 0.929595 | 0.070385 | 0 | 0 | 0 | 0 | 0 |
| 168 | 0.00002 | 0 | 0.926044 | 0.073936 | 0 | 0 | 0 | 0 | 0 |
| 169 | 0.00002 | 0 | 0.924977 | 0.075003 | 0 | 0 | 0 | 0 | 0 |
| 170 | 0.00002 | 0 | 0.929765 | 0.070215 | 0 | 0 | 0 | 0 | 0 |
| 171 | 0 | 0.00001 | 0.929942 | 0.070048 | 0 | 0 | 0 | 0 | 0 |
| 172 | 0.00002 | 0 | 0.925847 | 0.074133 | 0 | 0 | 0 | 0 | 0 |
| 173 | 0.00001 | 0 | 0.931342 | 0.068648 | 0 | 0 | 0 | 0 | 0 |
| 174 | 0 | 0 | 0.926289 | 0.073711 | 0 | 0 | 0 | 0 | 0 |
| 175 | 0.00001 | 0 | 0.927186 | 0.072804 | 0 | 0 | 0 | 0 | 0 |
| 176 | 0 | 0 | 0.932276 | 0.067724 | 0 | 0 | 0 | 0 | 0 |
| 177 | 0 | 0 | 0.931185 | 0.068815 | 0 | 0 | 0 | 0 | 0 |
| 178 | 0.00002 | 0 | 0.929597 | 0.070383 | 0 | 0 | 0 | 0 | 0 |
| 179 | 0.00001 | 0 | 0.92911 | 0.07088 | 0 | 0 | 0 | 0 | 0 |
| 180 | 0.00003 | 0 | 0.928617 | 0.071353 | 0 | 0 | 0 | 0 | 0 |
| 181 | 0.00001 | 0 | 0.931096 | 0.068894 | 0 | 0 | 0 | 0 | 0 |
| 182 | 0 | 0.00002 | 0.927319 | 0.072661 | 0 | 0 | 0 | 0 | 0 |
| 183 | 0 | 0 | 0.930628 | 0.069372 | 0 | 0 | 0 | 0 | 0 |
| 184 | 0.00002 | 0 | 0.9304 | 0.06958 | 0 | 0 | 0 | 0 | 0 |
| 185 | 0 | 0 | 0.93009 | 0.06991 | 0 | 0 | 0 | 0 | 0 |
| 186 | 0 | 0 | 0.930095 | 0.069905 | 0 | 0 | 0 | 0 | 0 |
| 187 | 0 | 0 | 0.932522 | 0.067478 | 0 | 0 | 0 | 0 | 0 |
| 188 | 0.00001 | 0 | 0.928298 | 0.071692 | 0 | 0 | 0 | 0 | 0 |
| 189 | 0 | 0.00001 | 0.927704 | 0.072286 | 0 | 0 | 0 | 0 | 0 |
| 190 | 0.00001 | 0 | 0.929721 | 0.070269 | 0 | 0 | 0 | 0 | 0 |
| 191 | 0.00003 | 0 | 0.929394 | 0.070576 | 0 | 0 | 0 | 0 | 0 |
| 192 | 0 | 0 | 0.930104 | 0.069896 | 0 | 0 | 0 | 0 | 0 |
| 193 | 0.00001 | 0 | 0.924066 | 0.075924 | 0 | 0 | 0 | 0 | 0 |
| 194 | 0 | 0.00001 | 0.923483 | 0.076507 | 0 | 0 | 0 | 0 | 0 |
| 195 | 0.00004 | 0 | 0.926636 | 0.073324 | 0 | 0 | 0 | 0 | 0 |
| 196 | 0.00002 | 0 | 0.927957 | 0.072023 | 0 | 0 | 0 | 0 | 0 |
| 197 | 0.00001 | 0 | 0.934089 | 0.065901 | 0 | 0 | 0 | 0 | 0 |
| 198 | 0.00001 | 0 | 0.928814 | 0.071107 | 0 | 0 | 0 | 0 | 0 |
| 199 | 0.00002 | 0 | 0.932083 | 0.067897 | 0 | 0 | 0 | 0 | 0 |
| 200 | 0 | 0 | 0.927679 | 0.072321 | 0 | 0 | 0 | 0 | 0 |
| 201 | 0.00001 | 0 | 0.929981 | 0.070009 | 0 | 0 | 0 | 0 | 0 |
| 202 | 0.00001 | 0.00003 | 0.930035 | 0.069925 | 0 | 0 | 0 | 0 | 0 |
| 203 | 0 | 0 | 0.931027 | 0.068973 | 0 | 0 | 0 | 0 | 0 |
| 204 | 0.00002 | 0.00001 | 0.929287 | 0.070682 | 0 | 0 | 0 | 0 | 0 |
| 205 | 0 | 0 | 0.927348 | 0.072652 | 0 | 0 | 0 | 0 | 0 |
| 206 | 0 | 0 | 0.924204 | 0.075796 | 0 | 0 | 0 | 0 | 0 |
| 207 | 0.00001 | 0 | 0.931521 | 0.068469 | 0 | 0 | 0 | 0 | 0 |
| 208 | 0 | 0 | 0.932261 | 0.067739 | 0 | 0 | 0 | 0 | 0 |
| 209 | 0.00001 | 0 | 0.927622 | 0.072368 | 0 | 0 | 0 | 0 | 0 |
| 210 | 0 | 0.00001 | 0.930993 | 0.068997 | 0 | 0 | 0 | 0 | 0 |
| 211 | 0.00003 | 0 | 0.927665 | 0.072305 | 0 | 0 | 0 | 0 | 0 |
| 212 | 0.00001 | 0 | 0.925798 | 0.074192 | 0 | 0 | 0 | 0 | 0 |
| 213 | 0.00001 | 0 | 0.928513 | 0.071477 | 0 | 0 | 0 | 0 | 0 |
| 214 | 0 | 0 | 0.929303 | 0.070697 | 0 | 0 | 0 | 0 | 0 |
| 215 | 0.00002 | 0 | 0.928178 | 0.071802 | 0 | 0 | 0 | 0 | 0 |
| 216 | 0 | 0 | 0.932129 | 0.067871 | 0 | 0 | 0 | 0 | 0 |
| 217 | 0 | 0.00001 | 0.927464 | 0.072526 | 0 | 0 | 0 | 0 | 0 |
| 218 | 0 | 0 | 0.93165 | 0.06835 | 0 | 0 | 0 | 0 | 0 |
| 219 | 0 | 0 | 0.935047 | 0.064953 | 0 | 0 | 0 | 0 | 0 |
| 220 | 0.00001 | 0 | 0.929294 | 0.070696 | 0 | 0 | 0 | 0 | 0 |
| 221 | 0.00001 | 0 | 0.930433 | 0.069557 | 0 | 0 | 0 | 0 | 0 |
| 222 | 0 | 0 | 0.928744 | 0.071256 | 0 | 0 | 0 | 0 | 0 |
| 223 | 0.00001 | 0 | 0.929681 | 0.070309 | 0 | 0 | 0 | 0 | 0 |
| 224 | 0.00003 | 0 | 0.926151 | 0.073819 | 0 | 0 | 0 | 0 | 0 |
| 225 | 0.00001 | 0 | 0.928854 | 0.071136 | 0 | 0 | 0 | 0 | 0 |
| 226 | 0.00001 | 0 | 0.928035 | 0.071955 | 0 | 0 | 0 | 0 | 0 |
| 227 | 0.00003 | 0 | 0.930757 | 0.069213 | 0 | 0 | 0 | 0 | 0 |
| 228 | 0 | 0 | 0.929413 | 0.070587 | 0 | 0 | 0 | 0 | 0 |
| 229 | 0.00002 | 0 | 0.929678 | 0.070302 | 0 | 0 | 0 | 0 | 0 |
| 230 | 0.00002 | 0 | 0.92945 | 0.07053 | 0 | 0 | 0 | 0 | 0 |
| 231 | 0.00003 | 0.00001 | 0.931895 | 0.068065 | 0 | 0 | 0 | 0 | 0 |
| 232 | 0.00001 | 0 | 0.927236 | 0.072754 | 0 | 0 | 0 | 0 | 0 |
| 233 | 0.00004 | 0 | 0.92754 | 0.07242 | 0 | 0 | 0 | 0 | 0 |
| 234 | 0.00004 | 0 | 0.928843 | 0.071117 | 0 | 0 | 0 | 0 | 0 |
| 235 | 0 | 0 | 0.934592 | 0.065408 | 0 | 0 | 0 | 0 | 0 |
| 236 | 0.00004 | 0 | 0.925082 | 0.074878 | 0 | 0 | 0 | 0 | 0 |
| 237 | 0.00003 | 0 | 0.9279 | 0.07207 | 0 | 0 | 0 | 0 | 0 |
| 238 | 0.00002 | 0 | 0.921872 | 0.078108 | 0 | 0 | 0 | 0 | 0 |
| 239 | 0.00001 | 0 | 0.924428 | 0.075562 | 0 | 0 | 0 | 0 | 0 |
| 240 | 0.00001 | 0 | 0.932471 | 0.067519 | 0 | 0 | 0 | 0 | 0 |
| 241 | 0.00001 | 0.00001 | 0.931925 | 0.068055 | 0 | 0 | 0 | 0 | 0 |
| 242 | 0 | 0 | 0.930356 | 0.069644 | 0 | 0 | 0 | 0 | 0 |
| 243 | 0.00001 | 0 | 0.931878 | 0.068112 | 0 | 0 | 0 | 0 | 0 |
| 244 | 0.00002 | 0 | 0.928231 | 0.071749 | 0 | 0 | 0 | 0 | 0 |
| 245 | 0 | 0 | 0.928231 | 0.071749 | 0 | 0 | 0 | 0 | 0 |
| 246 | 0 | 0.00001 | 0.926756 | 0.073234 | 0 | 0 | 0 | 0 | 0 |
| 247 | 0 | 0 | 0.928151 | 0.071849 | 0 | 0 | 0 | 0 | 0 |
| 248 | 0 | 0 | 0.929496 | 0.070504 | 0 | 0 | 0 | 0 | 0 |
| 249 | 0.00001 | 0 | 0.932299 | 0.067691 | 0 | 0 | 0 | 0 | 0 |
| 250 | 0 | 0 | 0.927558 | 0.072442 | 0 | 0 | 0 | 0 | 0 |
| 251 | 0.00002 | 0.00003 | 0.927911 | 0.072039 | 0 | 0 | 0 | 0 | 0 |
| 252 | 0.00004 | 0 | 0.931711 | 0.068249 | 0 | 0 | 0 | 0 | 0 |
| 253 | 0.00002 | 0 | 0.929886 | 0.070094 | 0 | 0 | 0 | 0 | 0 |
| 254 | 0.00003 | 0 | 0.927722 | 0.072248 | 0 | 0 | 0 | 0 | 0 |
| 255 | 0 | 0 | 0.929515 | 0.070485 | 0 | 0 | 0 | 0 | 0 |
| 256 | 0.00002 | 0.00001 | 0.925832 | 0.074138 | 0 | 0 | 0 | 0 | 0 |
| 257 | 0 | 0 | 0.929636 | 0.070364 | 0 | 0 | 0 | 0 | 0 |
| 258 | 0.00002 | 0 | 0.92465 | 0.07533 | 0 | 0 | 0 | 0 | 0 |
| 259 | 0.00001 | 0 | 0.926133 | 0.073857 | 0 | 0 | 0 | 0 | 0 |
| 260 | 0 | 0 | 0.931741 | 0.068259 | 0 | 0 | 0 | 0 | 0 |
| 261 | 0 | 0 | 0.932559 | 0.067441 | 0 | 0 | 0 | 0 | 0 |
| 262 | 0 | 0.00002 | 0.927666 | 0.072314 | 0 | 0 | 0 | 0 | 0 |
| 263 | 0.00002 | 0 | 0.927737 | 0.072242 | 0 | 0 | 0 | 0 | 0 |
| 264 | 0.00001 | 0 | 0.922705 | 0.077285 | 0 | 0 | 0 | 0 | 0 |
| 265 | 0.00001 | 0 | 0.929288 | 0.070702 | 0 | 0 | 0 | 0 | 0 |
| 266 | 0.00002 | 0.00002 | 0.931714 | 0.068246 | 0 | 0 | 0 | 0 | 0 |
| 267 | 0.00002 | 0 | 0.929965 | 0.070015 | 0 | 0 | 0 | 0 | 0 |
| 268 | 0.00001 | 0 | 0.932773 | 0.067217 | 0 | 0 | 0 | 0 | 0 |
| 269 | 0.00002 | 0 | 0.930155 | 0.069825 | 0 | 0 | 0 | 0 | 0 |
| 270 | 0.00001 | 0 | 0.930252 | 0.069738 | 0 | 0 | 0 | 0 | 0 |
| 271 | 0 | 0 | 0.929968 | 0.070032 | 0 | 0 | 0 | 0 | 0 |
| 272 | 0 | 0 | 0.932161 | 0.067839 | 0 | 0 | 0 | 0 | 0 |
| 273 | 0.00001 | 0 | 0.931133 | 0.068857 | 0 | 0 | 0 | 0 | 0 |
| 274 | 0.00001 | 0 | 0.932558 | 0.067432 | 0 | 0 | 0 | 0 | 0 |
| 275 | 0.00001 | 0 | 0.930519 | 0.069471 | 0 | 0 | 0 | 0 | 0 |
| 276 | 0.00002 | 0 | 0.928635 | 0.071346 | 0 | 0 | 0 | 0 | 0 |
| 277 | 0.00001 | 0.00001 | 0.923691 | 0.076289 | 0 | 0 | 0 | 0 | 0 |
| 278 | 0 | 0 | 0.931348 | 0.068652 | 0 | 0 | 0 | 0 | 0 |
| 279 | 0.00001 | 0 | 0.929632 | 0.070358 | 0 | 0 | 0 | 0 | 0 |
| 280 | 0 | 0 | 0.928173 | 0.071827 | 0 | 0 | 0 | 0 | 0 |
| 281 | 0.00003 | 0 | 0.925254 | 0.074716 | 0 | 0 | 0 | 0 | 0 |
| 282 | 0.00001 | 0 | 0.929207 | 0.070783 | 0 | 0 | 0 | 0 | 0 |
| 283 | 0.00004 | 0 | 0.930255 | 0.069705 | 0 | 0 | 0 | 0 | 0 |
| 284 | 0.00001 | 0 | 0.931688 | 0.068303 | 0 | 0 | 0 | 0 | 0 |
| 285 | 0.00003 | 0 | 0.926902 | 0.073068 | 0 | 0 | 0 | 0 | 0 |
| 286 | 0.00001 | 0 | 0.934089 | 0.065901 | 0 | 0 | 0 | 0 | 0 |
| 287 | 0 | 0 | 0.927158 | 0.072842 | 0 | 0 | 0 | 0 | 0 |
| 288 | 0.00002 | 0 | 0.927168 | 0.072812 | 0 | 0 | 0 | 0 | 0 |
| 289 | 0.00001 | 0 | 0.931663 | 0.068327 | 0 | 0 | 0 | 0 | 0 |
| 290 | 0.00001 | 0 | 0.928688 | 0.071302 | 0 | 0 | 0 | 0 | 0 |
| 291 | 0.00001 | 0 | 0.927765 | 0.072225 | 0 | 0 | 0 | 0 | 0 |
| 292 | 0.00003 | 0 | 0.925917 | 0.074053 | 0 | 0 | 0 | 0 | 0 |
| 293 | 0.00001 | 0 | 0.926229 | 0.073761 | 0 | 0 | 0 | 0 | 0 |
| 294 | 0.00001 | 0 | 0.925326 | 0.074664 | 0 | 0 | 0 | 0 | 0 |
| 295 | 0 | 0 | 0.9325 | 0.0675 | 0 | 0 | 0 | 0 | 0 |
| 296 | 0.00002 | 0 | 0.928557 | 0.071423 | 0 | 0 | 0 | 0 | 0 |
| 297 | 0 | 0.00001 | 0.92686 | 0.07313 | 0 | 0 | 0 | 0 | 0 |
| 298 | 0.00002 | 0 | 0.92916 | 0.07082 | 0 | 0 | 0 | 0 | 0 |
| 299 | 0.00003 | 0 | 0.923405 | 0.076565 | 0 | 0 | 0 | 0 | 0 |
| 300 | 0 | 0 | 0.928694 | 0.071306 | 0 | 0 | 0 | 0 | 0 |
| 301 | 0.00004 | 0 | 0.929294 | 0.070666 | 0 | 0 | 0 | 0 | 0 |
| 302 | 0.00001 | 0 | 0.925836 | 0.074154 | 0 | 0 | 0 | 0 | 0 |
| 303 | 0.00001 | 0 | 0.928413 | 0.071577 | 0 | 0 | 0 | 0 | 0 |
| 304 | 0 | 0 | 0.926324 | 0.073676 | 0 | 0 | 0 | 0 | 0 |
| 305 | 0.00003 | 0 | 0.929495 | 0.070475 | 0 | 0 | 0 | 0 | 0 |
| 306 | 0 | 0 | 0.932263 | 0.067737 | 0 | 0 | 0 | 0 | 0 |
| 307 | 0 | 0.00001 | 0.93007 | 0.06992 | 0 | 0 | 0 | 0 | 0 |
| 308 | 0 | 0 | 0.932706 | 0.067294 | 0 | 0 | 0 | 0 | 0 |
| 309 | 0.00002 | 0 | 0.93085 | 0.06913 | 0 | 0 | 0 | 0 | 0 |
| 310 | 0.00001 | 0 | 0.928218 | 0.071772 | 0 | 0 | 0 | 0 | 0 |
| 311 | 0.00001 | 0 | 0.930464 | 0.069526 | 0 | 0 | 0 | 0 | 0 |
| 312 | 0 | 0 | 0.927586 | 0.072414 | 0 | 0 | 0 | 0 | 0 |
| 313 | 0 | 0 | 0.930598 | 0.069402 | 0 | 0 | 0 | 0 | 0 |
| 314 | 0.00001 | 0 | 0.930355 | 0.069635 | 0 | 0 | 0 | 0 | 0 |
| 315 | 0 | 0 | 0.927604 | 0.072396 | 0 | 0 | 0 | 0 | 0 |
| 316 | 0.00001 | 0 | 0.928291 | 0.071699 | 0 | 0 | 0 | 0 | 0 |
| 317 | 0.00001 | 0.00001 | 0.926326 | 0.073654 | 0 | 0 | 0 | 0 | 0 |
| 318 | 0.00001 | 0.00002 | 0.925367 | 0.074583 | 0 | 0 | 0 | 0 | 0 |
| 319 | 0.00002 | 0 | 0.92799 | 0.07199 | 0 | 0 | 0 | 0 | 0 |
| 320 | 0.00001 | 0 | 0.929451 | 0.070539 | 0 | 0 | 0 | 0 | 0 |
| 321 | 0.00002 | 0 | 0.927667 | 0.072313 | 0 | 0 | 0 | 0 | 0 |
| 322 | 0 | 0 | 0.928585 | 0.071415 | 0 | 0 | 0 | 0 | 0 |
| 323 | 0 | 0 | 0.930416 | 0.069584 | 0 | 0 | 0 | 0 | 0 |
| 324 | 0 | 0 | 0.931238 | 0.068762 | 0 | 0 | 0 | 0 | 0 |
| 325 | 0 | 0 | 0.930503 | 0.069497 | 0 | 0 | 0 | 0 | 0 |
| 326 | 0.00003 | 0.00001 | 0.928768 | 0.071192 | 0 | 0 | 0 | 0 | 0 |
| 327 | 0.00001 | 0 | 0.928534 | 0.071456 | 0 | 0 | 0 | 0 | 0 |
| 328 | 0.00001 | 0 | 0.927893 | 0.072097 | 0 | 0 | 0 | 0 | 0 |
| 329 | 0.00001 | 0.00001 | 0.928913 | 0.071068 | 0 | 0 | 0 | 0 | 0 |
| 330 | 0.00004 | 0 | 0.929812 | 0.070147 | 0 | 0 | 0 | 0 | 0 |
| 331 | 0.00001 | 0.00001 | 0.927491 | 0.072489 | 0 | 0 | 0 | 0 | 0 |
| 332 | 0.00003 | 0 | 0.928348 | 0.071622 | 0 | 0 | 0 | 0 | 0 |
| 333 | 0.00002 | 0 | 0.92462 | 0.07536 | 0 | 0 | 0 | 0 | 0 |
| 334 | 0.00001 | 0.00004 | 0.931012 | 0.068938 | 0 | 0 | 0 | 0 | 0 |
| 335 | 0.00001 | 0 | 0.929769 | 0.070221 | 0 | 0 | 0 | 0 | 0 |
| 336 | 0.00001 | 0 | 0.926596 | 0.073394 | 0 | 0 | 0 | 0 | 0 |
| 337 | 0 | 0 | 0.929665 | 0.070335 | 0 | 0 | 0 | 0 | 0 |
| 338 | 0.00004 | 0 | 0.931391 | 0.068569 | 0 | 0 | 0 | 0 | 0 |
| 339 | 0 | 0 | 0.926389 | 0.073611 | 0 | 0 | 0 | 0 | 0 |
| 340 | 0.00003 | 0 | 0.926713 | 0.073256 | 0 | 0 | 0 | 0 | 0 |
| 341 | 0.00001 | 0 | 0.92697 | 0.07302 | 0 | 0 | 0 | 0 | 0 |
| 342 | 0.00003 | 0 | 0.925646 | 0.074324 | 0 | 0 | 0 | 0 | 0 |
| 343 | 0.00001 | 0 | 0.927222 | 0.072767 | 0 | 0 | 0 | 0 | 0 |
| 344 | 0.00001 | 0 | 0.927049 | 0.072941 | 0 | 0 | 0 | 0 | 0 |
| 345 | 0.00003 | 0 | 0.931428 | 0.068542 | 0 | 0 | 0 | 0 | 0 |
| 346 | 0.00001 | 0 | 0.933915 | 0.066075 | 0 | 0 | 0 | 0 | 0 |
| 347 | 0 | 0.00001 | 0.925748 | 0.074242 | 0 | 0 | 0 | 0 | 0 |
| 348 | 0.00004 | 0 | 0.927002 | 0.072958 | 0 | 0 | 0 | 0 | 0 |
| 349 | 0.00001 | 0 | 0.929554 | 0.070436 | 0 | 0 | 0 | 0 | 0 |
| 350 | 0 | 0 | 0.930332 | 0.069668 | 0 | 0 | 0 | 0 | 0 |
| 351 | 0 | 0 | 0.930855 | 0.069145 | 0 | 0 | 0 | 0 | 0 |
| 352 | 0.00003 | 0 | 0.931553 | 0.068417 | 0 | 0 | 0 | 0 | 0 |
| 353 | 0.00001 | 0 | 0.929797 | 0.070193 | 0 | 0 | 0 | 0 | 0 |
| 354 | 0.00002 | 0 | 0.928489 | 0.071491 | 0 | 0 | 0 | 0 | 0 |
| 355 | 0 | 0 | 0.930735 | 0.069265 | 0 | 0 | 0 | 0 | 0 |
| 356 | 0.00001 | 0 | 0.927789 | 0.072201 | 0 | 0 | 0 | 0 | 0 |
| 357 | 0.00003 | 0 | 0.924547 | 0.075423 | 0 | 0 | 0 | 0 | 0 |
| 358 | 0.00002 | 0.00001 | 0.928576 | 0.071394 | 0 | 0 | 0 | 0 | 0 |
| 359 | 0.00001 | 0 | 0.928256 | 0.071734 | 0 | 0 | 0 | 0 | 0 |
| 360 | 0.00001 | 0 | 0.929461 | 0.070529 | 0 | 0 | 0 | 0 | 0 |
| 361 | 0 | 0 | 0.927725 | 0.072275 | 0 | 0 | 0 | 0 | 0 |
| 362 | 0 | 0 | 0.932191 | 0.067809 | 0 | 0 | 0 | 0 | 0 |
| 363 | 0.00004 | 0.00002 | 0.930851 | 0.06909 | 0 | 0 | 0 | 0 | 0 |
| 364 | 0.00003 | 0.00001 | 0.930469 | 0.069491 | 0 | 0 | 0 | 0 | 0 |
| 365 | 0.00001 | 0 | 0.928389 | 0.071601 | 0 | 0 | 0 | 0 | 0 |
| 366 | 0.00002 | 0 | 0.929091 | 0.070889 | 0 | 0 | 0 | 0 | 0 |
| 367 | 0.00001 | 0 | 0.927569 | 0.072421 | 0 | 0 | 0 | 0 | 0 |
| 368 | 0 | 0 | 0.931261 | 0.068739 | 0 | 0 | 0 | 0 | 0 |
| 369 | 0 | 0 | 0.926134 | 0.073866 | 0 | 0 | 0 | 0 | 0 |
| 370 | 0.00002 | 0 | 0.929964 | 0.070016 | 0 | 0 | 0 | 0 | 0 |
| 371 | 0.00003 | 0 | 0.930581 | 0.069349 | 0 | 0 | 0 | 0 | 0 |
| 372 | 0.00001 | 0 | 0.923925 | 0.076065 | 0 | 0 | 0 | 0 | 0 |
| 373 | 0.00002 | 0 | 0.925649 | 0.074331 | 0 | 0 | 0 | 0 | 0 |
| 374 | 0.00002 | 0 | 0.930647 | 0.069333 | 0 | 0 | 0 | 0 | 0 |
| 375 | 0.00001 | 0 | 0.927611 | 0.072379 | 0 | 0 | 0 | 0 | 0 |
| 376 | 0.00003 | 0.00002 | 0.92395 | 0.076 | 0 | 0 | 0 | 0 | 0 |
| 377 | 0.00002 | 0 | 0.92429 | 0.07569 | 0 | 0 | 0 | 0 | 0 |
| 378 | 0.00002 | 0 | 0.932181 | 0.067799 | 0 | 0 | 0 | 0 | 0 |
| 379 | 0.00001 | 0 | 0.930281 | 0.069709 | 0 | 0 | 0 | 0 | 0 |
| 380 | 0 | 0 | 0.923884 | 0.076116 | 0 | 0 | 0 | 0 | 0 |
| 381 | 0.00001 | 0.00003 | 0.928771 | 0.07119 | 0 | 0 | 0 | 0 | 0 |
| 382 | 0.00001 | 0 | 0.92952 | 0.070469 | 0 | 0 | 0 | 0 | 0 |
| 383 | 0.00004 | 0 | 0.926344 | 0.073616 | 0 | 0 | 0 | 0 | 0 |
| 384 | 0.00002 | 0 | 0.922538 | 0.077442 | 0 | 0 | 0 | 0 | 0 |
| 385 | 0 | 0.00001 | 0.928794 | 0.071196 | 0 | 0 | 0 | 0 | 0 |
| 386 | 0 | 0.00001 | 0.929325 | 0.070665 | 0 | 0 | 0 | 0 | 0 |
| 387 | 0.00003 | 0 | 0.92973 | 0.07024 | 0 | 0 | 0 | 0 | 0 |
| 388 | 0.00001 | 0 | 0.931034 | 0.068956 | 0 | 0 | 0 | 0 | 0 |
| 389 | 0 | 0.00001 | 0.93156 | 0.06843 | 0 | 0 | 0 | 0 | 0 |
| 390 | 0.00005 | 0.00003 | 0.930016 | 0.069904 | 0 | 0 | 0 | 0 | 0 |
| 391 | 0.00002 | 0 | 0.925645 | 0.074335 | 0 | 0 | 0 | 0 | 0 |
| 392 | 0 | 0 | 0.928612 | 0.071388 | 0 | 0 | 0 | 0 | 0 |
| 393 | 0 | 0 | 0.925724 | 0.074276 | 0 | 0 | 0 | 0 | 0 |
| 394 | 0.00003 | 0 | 0.931452 | 0.068518 | 0 | 0 | 0 | 0 | 0 |
| 395 | 0.00001 | 0 | 0.929877 | 0.070113 | 0 | 0 | 0 | 0 | 0 |
| 396 | 0.00001 | 0 | 0.929545 | 0.070445 | 0 | 0 | 0 | 0 | 0 |
| 397 | 0.00001 | 0 | 0.927952 | 0.072038 | 0 | 0 | 0 | 0 | 0 |
| 398 | 0 | 0 | 0.933225 | 0.066775 | 0 | 0 | 0 | 0 | 0 |
| 399 | 0.00001 | 0 | 0.931982 | 0.068008 | 0 | 0 | 0 | 0 | 0 |
| 400 | 0.00002 | 0.00002 | 0.931286 | 0.068674 | 0 | 0 | 0 | 0 | 0 |
| 401 | 0.00005 | 0 | 0.927239 | 0.072711 | 0 | 0 | 0 | 0 | 0 |
| 402 | 0.00001 | 0 | 0.929783 | 0.070207 | 0 | 0 | 0 | 0 | 0 |
| 403 | 0.00001 | 0 | 0.92969 | 0.0703 | 0 | 0 | 0 | 0 | 0 |
| 404 | 0.00002 | 0 | 0.925628 | 0.074352 | 0 | 0 | 0 | 0 | 0 |
| 405 | 0.00001 | 0 | 0.93166 | 0.06833 | 0 | 0 | 0 | 0 | 0 |
| 406 | 0.00002 | 0 | 0.928442 | 0.071538 | 0 | 0 | 0 | 0 | 0 |
| 407 | 0 | 0 | 0.924429 | 0.075571 | 0 | 0 | 0 | 0 | 0 |
| 408 | 0.00001 | 0.00004 | 0.92615 | 0.073801 | 0 | 0 | 0 | 0 | 0 |
| 409 | 0 | 0 | 0.925005 | 0.074995 | 0 | 0 | 0 | 0 | 0 |
| 410 | 0.00002 | 0 | 0.924709 | 0.075271 | 0 | 0 | 0 | 0 | 0 |
| 411 | 0.00002 | 0 | 0.928652 | 0.071328 | 0 | 0 | 0 | 0 | 0 |
| 412 | 0.00001 | 0.00001 | 0.927297 | 0.072683 | 0 | 0 | 0 | 0 | 0 |
| 413 | 0.00001 | 0 | 0.925905 | 0.074085 | 0 | 0 | 0 | 0 | 0 |
| 414 | 0 | 0 | 0.930731 | 0.069269 | 0 | 0 | 0 | 0 | 0 |
| 415 | 0.00003 | 0 | 0.926971 | 0.072999 | 0 | 0 | 0 | 0 | 0 |
| 416 | 0.00001 | 0 | 0.92911 | 0.07088 | 0 | 0 | 0 | 0 | 0 |
| 417 | 0 | 0 | 0.931564 | 0.068436 | 0 | 0 | 0 | 0 | 0 |
| 418 | 0 | 0 | 0.929497 | 0.070503 | 0 | 0 | 0 | 0 | 0 |
| 419 | 0.00004 | 0 | 0.926321 | 0.073639 | 0 | 0 | 0 | 0 | 0 |
| 420 | 0.00001 | 0 | 0.936853 | 0.063137 | 0 | 0 | 0 | 0 | 0 |
| 421 | 0.00001 | 0 | 0.926468 | 0.073522 | 0 | 0 | 0 | 0 | 0 |
| 422 | 0.00001 | 0.00001 | 0.927705 | 0.072275 | 0 | 0 | 0 | 0 | 0 |
| 423 | 0.00001 | 0 | 0.931386 | 0.068604 | 0 | 0 | 0 | 0 | 0 |
| 424 | 0 | 0.00001 | 0.933231 | 0.066759 | 0 | 0 | 0 | 0 | 0 |
| 425 | 0 | 0.00001 | 0.92759 | 0.0724 | 0 | 0 | 0 | 0 | 0 |
| 426 | 0.00004 | 0 | 0.927969 | 0.071991 | 0 | 0 | 0 | 0 | 0 |
| 427 | 0 | 0 | 0.929329 | 0.070671 | 0 | 0 | 0 | 0 | 0 |
| 428 | 0.00001 | 0 | 0.93133 | 0.068661 | 0 | 0 | 0 | 0 | 0 |
| 429 | 0 | 0 | 0.930837 | 0.069163 | 0 | 0 | 0 | 0 | 0 |
| 430 | 0.00001 | 0 | 0.928306 | 0.071684 | 0 | 0 | 0 | 0 | 0 |
| 431 | 0.00001 | 0 | 0.93202 | 0.06797 | 0 | 0 | 0 | 0 | 0 |
| 432 | 0 | 0.00002 | 0.924902 | 0.075078 | 0 | 0 | 0 | 0 | 0 |
| 433 | 0.00001 | 0 | 0.931589 | 0.068401 | 0 | 0 | 0 | 0 | 0 |
| 434 | 0 | 0.00003 | 0.930667 | 0.069303 | 0 | 0 | 0 | 0 | 0 |
| 435 | 0.00003 | 0 | 0.927844 | 0.072126 | 0 | 0 | 0 | 0 | 0 |
| 436 | 0.00001 | 0 | 0.922423 | 0.077567 | 0 | 0 | 0 | 0 | 0 |
| 437 | 0 | 0 | 0.927285 | 0.072715 | 0 | 0 | 0 | 0 | 0 |
| 438 | 0.00002 | 0 | 0.931683 | 0.068297 | 0 | 0 | 0 | 0 | 0 |
| 439 | 0.00002 | 0 | 0.931758 | 0.068222 | 0 | 0 | 0 | 0 | 0 |
| 440 | 0.00001 | 0 | 0.929424 | 0.070566 | 0 | 0 | 0 | 0 | 0 |
| 441 | 0.00001 | 0.00002 | 0.92791 | 0.07206 | 0 | 0 | 0 | 0 | 0 |
| 442 | 0.00001 | 0 | 0.928272 | 0.071718 | 0 | 0 | 0 | 0 | 0 |
| 443 | 0.00001 | 0.00001 | 0.932965 | 0.067015 | 0 | 0 | 0 | 0 | 0 |
| 444 | 0.00002 | 0.00001 | 0.926849 | 0.073121 | 0 | 0 | 0 | 0 | 0 |
| 445 | 0.00002 | 0 | 0.927377 | 0.072603 | 0 | 0 | 0 | 0 | 0 |
| 446 | 0 | 0 | 0.926957 | 0.073043 | 0 | 0 | 0 | 0 | 0 |
| 447 | 0.00001 | 0 | 0.931501 | 0.068489 | 0 | 0 | 0 | 0 | 0 |
| 448 | 0.00001 | 0 | 0.929244 | 0.070746 | 0 | 0 | 0 | 0 | 0 |
| 449 | 0 | 0 | 0.929746 | 0.070254 | 0 | 0 | 0 | 0 | 0 |
| 450 | 0 | 0 | 0.928259 | 0.071741 | 0 | 0 | 0 | 0 | 0 |
| 451 | 0.00002 | 0 | 0.93141 | 0.06857 | 0 | 0 | 0 | 0 | 0 |
| 452 | 0.00001 | 0 | 0.930432 | 0.069558 | 0 | 0 | 0 | 0 | 0 |
| 453 | 0 | 0 | 0.928028 | 0.071972 | 0 | 0 | 0 | 0 | 0 |
| 454 | 0 | 0 | 0.926045 | 0.073955 | 0 | 0 | 0 | 0 | 0 |
| 455 | 0 | 0 | 0.931736 | 0.068264 | 0 | 0 | 0 | 0 | 0 |
| 456 | 0.00002 | 0.00001 | 0.927095 | 0.072876 | 0 | 0 | 0 | 0 | 0 |
| 457 | 0.00001 | 0.00002 | 0.927262 | 0.072708 | 0 | 0 | 0 | 0 | 0 |
| 458 | 0.00002 | 0 | 0.930839 | 0.069141 | 0 | 0 | 0 | 0 | 0 |
| 459 | 0.00001 | 0 | 0.920853 | 0.079137 | 0 | 0 | 0 | 0 | 0 |
| 460 | 0.00002 | 0 | 0.924766 | 0.075214 | 0 | 0 | 0 | 0 | 0 |
| 461 | 0.00001 | 0 | 0.932622 | 0.067368 | 0 | 0 | 0 | 0 | 0 |
| 462 | 0.00003 | 0 | 0.93347 | 0.0665 | 0 | 0 | 0 | 0 | 0 |
| 463 | 0.00004 | 0 | 0.924833 | 0.075127 | 0 | 0 | 0 | 0 | 0 |
| 464 | 0.00001 | 0 | 0.926138 | 0.073743 | 0 | 0 | 0 | 0 | 0 |
| 465 | 0.00001 | 0 | 0.931019 | 0.068971 | 0 | 0 | 0 | 0 | 0 |
| 466 | 0 | 0 | 0.926919 | 0.073081 | 0 | 0 | 0 | 0 | 0 |
| 467 | 0 | 0 | 0.925574 | 0.074426 | 0 | 0 | 0 | 0 | 0 |
| 468 | 0 | 0 | 0.930902 | 0.069098 | 0 | 0 | 0 | 0 | 0 |
| 469 | 0 | 0 | 0.927931 | 0.072069 | 0 | 0 | 0 | 0 | 0 |
| 470 | 0 | 0 | 0.930659 | 0.069341 | 0 | 0 | 0 | 0 | 0 |
| 471 | 0.00003 | 0 | 0.927614 | 0.072356 | 0 | 0 | 0 | 0 | 0 |
| 472 | 0.00001 | 0 | 0.927883 | 0.072107 | 0 | 0 | 0 | 0 | 0 |
| 473 | 0.00001 | 0 | 0.931312 | 0.068678 | 0 | 0 | 0 | 0 | 0 |
| 474 | 0 | 0 | 0.929456 | 0.070544 | 0 | 0 | 0 | 0 | 0 |
| 475 | 0.00002 | 0 | 0.930423 | 0.069556 | 0 | 0 | 0 | 0 | 0 |
| 476 | 0.00001 | 0.00001 | 0.921468 | 0.078512 | 0 | 0 | 0 | 0 | 0 |
| 477 | 0.00001 | 0 | 0.930062 | 0.069928 | 0 | 0 | 0 | 0 | 0 |
| 478 | 0.00001 | 0 | 0.927332 | 0.072658 | 0 | 0 | 0 | 0 | 0 |
| 479 | 0.00003 | 0 | 0.927345 | 0.072625 | 0 | 0 | 0 | 0 | 0 |
| 480 | 0.00002 | 0 | 0.925616 | 0.074364 | 0 | 0 | 0 | 0 | 0 |
| 481 | 0.00003 | 0.00001 | 0.93369 | 0.06627 | 0 | 0 | 0 | 0 | 0 |
| 482 | 0.00001 | 0 | 0.926269 | 0.073721 | 0 | 0 | 0 | 0 | 0 |
| 483 | 0.00002 | 0 | 0.928979 | 0.071001 | 0 | 0 | 0 | 0 | 0 |
| 484 | 0 | 0 | 0.92623 | 0.07377 | 0 | 0 | 0 | 0 | 0 |
| 485 | 0 | 0.00001 | 0.932071 | 0.067919 | 0 | 0 | 0 | 0 | 0 |
| 486 | 0.00003 | 0 | 0.928591 | 0.071378 | 0 | 0 | 0 | 0 | 0 |
| 487 | 0.00003 | 0 | 0.927743 | 0.072227 | 0 | 0 | 0 | 0 | 0 |
| 488 | 0.00001 | 0 | 0.926519 | 0.073471 | 0 | 0 | 0 | 0 | 0 |
| 489 | 0.00001 | 0 | 0.92793 | 0.07206 | 0 | 0 | 0 | 0 | 0 |
| 490 | 0 | 0 | 0.933517 | 0.066483 | 0 | 0 | 0 | 0 | 0 |
| 491 | 0 | 0 | 0.928009 | 0.071991 | 0 | 0 | 0 | 0 | 0 |
| 492 | 0.00004 | 0 | 0.927222 | 0.072738 | 0 | 0 | 0 | 0 | 0 |
| 493 | 0.00001 | 0 | 0.928228 | 0.071762 | 0 | 0 | 0 | 0 | 0 |
| 494 | 0 | 0 | 0.929717 | 0.070283 | 0 | 0 | 0 | 0 | 0 |
| 495 | 0 | 0 | 0.927807 | 0.072193 | 0 | 0 | 0 | 0 | 0 |
| 496 | 0.00002 | 0 | 0.925678 | 0.074293 | 0 | 0 | 0 | 0 | 0 |
| 497 | 0 | 0 | 0.929434 | 0.070566 | 0 | 0 | 0 | 0 | 0 |
| 498 | 0 | 0 | 0.933933 | 0.066067 | 0 | 0 | 0 | 0 | 0 |
| 499 | 0.00001 | 0 | 0.927913 | 0.072077 | 0 | 0 | 0 | 0 | 0 |
| 500 | 0.00001 | 0 | 0.929509 | 0.070481 | 0 | 0 | 0 | 0 | 0 |
| 501 | 0.00003 | 0 | 0.927682 | 0.072288 | 0 | 0 | 0 | 0 | 0 |
| 502 | 0 | 0 | 0.92893 | 0.07107 | 0 | 0 | 0 | 0 | 0 |
| 503 | 0 | 0 | 0.925349 | 0.074651 | 0 | 0 | 0 | 0 | 0 |
| 504 | 0.00002 | 0 | 0.930045 | 0.069935 | 0 | 0 | 0 | 0 | 0 |
| 505 | 0.00001 | 0 | 0.927372 | 0.072608 | 0 | 0 | 0 | 0 | 0 |
| 506 | 0 | 0 | 0.925766 | 0.074234 | 0 | 0 | 0 | 0 | 0 |
| 507 | 0.00002 | 0 | 0.927828 | 0.072152 | 0 | 0 | 0 | 0 | 0 |
| 508 | 0.00001 | 0.00001 | 0.92687 | 0.07311 | 0 | 0 | 0 | 0 | 0 |
| 509 | 0 | 0 | 0.925559 | 0.074441 | 0 | 0 | 0 | 0 | 0 |
| 510 | 0 | 0 | 0.928401 | 0.071599 | 0 | 0 | 0 | 0 | 0 |
| 511 | 0 | 0 | 0.928363 | 0.071637 | 0 | 0 | 0 | 0 | 0 |
| 512 | 0.00003 | 0 | 0.926924 | 0.073046 | 0 | 0 | 0 | 0 | 0 |
| 513 | 0 | 0 | 0.926758 | 0.073242 | 0 | 0 | 0 | 0 | 0 |
| 514 | 0.00002 | 0 | 0.929425 | 0.070555 | 0 | 0 | 0 | 0 | 0 |
| 515 | 0.00001 | 0 | 0.930784 | 0.069206 | 0 | 0 | 0 | 0 | 0 |
| 516 | 0.00003 | 0 | 0.923519 | 0.076451 | 0 | 0 | 0 | 0 | 0 |
| 517 | 0 | 0 | 0.92787 | 0.07213 | 0 | 0 | 0 | 0 | 0 |
| 518 | 0 | 0 | 0.928885 | 0.071115 | 0 | 0 | 0 | 0 | 0 |
| 519 | 0.00001 | 0.00001 | 0.926978 | 0.073002 | 0 | 0 | 0 | 0 | 0 |
| 520 | 0.00001 | 0 | 0.929341 | 0.070649 | 0 | 0 | 0 | 0 | 0 |
| 521 | 0.00005 | 0 | 0.926082 | 0.073868 | 0 | 0 | 0 | 0 | 0 |
| 522 | 0.00001 | 0 | 0.93224 | 0.06775 | 0 | 0 | 0 | 0 | 0 |
| 523 | 0.00001 | 0 | 0.927944 | 0.072046 | 0 | 0 | 0 | 0 | 0 |
| 524 | 0.00001 | 0 | 0.927754 | 0.072236 | 0 | 0 | 0 | 0 | 0 |
| 525 | 0 | 0 | 0.926645 | 0.073355 | 0 | 0 | 0 | 0 | 0 |
| 526 | 0.00002 | 0.00002 | 0.928194 | 0.071766 | 0 | 0 | 0 | 0 | 0 |
| 527 | 0.00002 | 0 | 0.928231 | 0.071749 | 0 | 0 | 0 | 0 | 0 |
| 528 | 0.00002 | 0 | 0.92714 | 0.07284 | 0 | 0 | 0 | 0 | 0 |
| 529 | 0 | 0 | 0.928445 | 0.071555 | 0 | 0 | 0 | 0 | 0 |
| 530 | 0.00001 | 0 | 0.927569 | 0.072421 | 0 | 0 | 0 | 0 | 0 |
| 531 | 0.00002 | 0 | 0.927299 | 0.072681 | 0 | 0 | 0 | 0 | 0 |
| 532 | 0 | 0 | 0.933788 | 0.066212 | 0 | 0 | 0 | 0 | 0 |
| 533 | 0.00002 | 0 | 0.927842 | 0.072138 | 0 | 0 | 0 | 0 | 0 |
| 534 | 0.00001 | 0 | 0.926763 | 0.073227 | 0 | 0 | 0 | 0 | 0 |
| 535 | 0.00002 | 0 | 0.931535 | 0.068445 | 0 | 0 | 0 | 0 | 0 |
| 536 | 0.00003 | 0 | 0.931423 | 0.068546 | 0 | 0 | 0 | 0 | 0 |
| 537 | 0.00002 | 0 | 0.927217 | 0.072763 | 0 | 0 | 0 | 0 | 0 |
| 538 | 0.00001 | 0 | 0.927075 | 0.072915 | 0 | 0 | 0 | 0 | 0 |
| 539 | 0 | 0 | 0.924305 | 0.075695 | 0 | 0 | 0 | 0 | 0 |
| 540 | 0 | 0 | 0.926551 | 0.073449 | 0 | 0 | 0 | 0 | 0 |
| 541 | 0.00003 | 0.00001 | 0.931141 | 0.068819 | 0 | 0 | 0 | 0 | 0 |
| 542 | 0.00002 | 0 | 0.928581 | 0.071399 | 0 | 0 | 0 | 0 | 0 |
| 543 | 0.00001 | 0 | 0.932406 | 0.067584 | 0 | 0 | 0 | 0 | 0 |
| 544 | 0 | 0 | 0.932212 | 0.067788 | 0 | 0 | 0 | 0 | 0 |
| 545 | 0.00001 | 0 | 0.92377 | 0.07622 | 0 | 0 | 0 | 0 | 0 |
| 546 | 0.00001 | 0.00001 | 0.933511 | 0.066469 | 0 | 0 | 0 | 0 | 0 |
| 547 | 0 | 0 | 0.92801 | 0.07199 | 0 | 0 | 0 | 0 | 0 |
| 548 | 0 | 0 | 0.930331 | 0.069669 | 0 | 0 | 0 | 0 | 0 |
| 549 | 0.00001 | 0 | 0.92522 | 0.07477 | 0 | 0 | 0 | 0 | 0 |
| 550 | 0.00001 | 0 | 0.928946 | 0.071044 | 0 | 0 | 0 | 0 | 0 |
| 551 | 0.00001 | 0 | 0.930118 | 0.069872 | 0 | 0 | 0 | 0 | 0 |
| 552 | 0 | 0.00001 | 0.926606 | 0.073384 | 0 | 0 | 0 | 0 | 0 |
| 553 | 0.00002 | 0 | 0.927045 | 0.072935 | 0 | 0 | 0 | 0 | 0 |
| 554 | 0 | 0 | 0.93524 | 0.06476 | 0 | 0 | 0 | 0 | 0 |
| 555 | 0.00001 | 0 | 0.925045 | 0.074945 | 0 | 0 | 0 | 0 | 0 |
| 556 | 0.00002 | 0 | 0.932495 | 0.067485 | 0 | 0 | 0 | 0 | 0 |
| 557 | 0 | 0 | 0.928211 | 0.071789 | 0 | 0 | 0 | 0 | 0 |
| 558 | 0.00001 | 0 | 0.929044 | 0.070946 | 0 | 0 | 0 | 0 | 0 |
| 559 | 0.00003 | 0 | 0.930898 | 0.069072 | 0 | 0 | 0 | 0 | 0 |
| 560 | 0 | 0 | 0.930397 | 0.069603 | 0 | 0 | 0 | 0 | 0 |
| 561 | 0 | 0 | 0.925387 | 0.074613 | 0 | 0 | 0 | 0 | 0 |
| 562 | 0 | 0 | 0.925831 | 0.074169 | 0 | 0 | 0 | 0 | 0 |
| 563 | 0.00002 | 0 | 0.926544 | 0.073436 | 0 | 0 | 0 | 0 | 0 |
| 564 | 0 | 0 | 0.929384 | 0.070616 | 0 | 0 | 0 | 0 | 0 |
| 565 | 0 | 0 | 0.925714 | 0.074286 | 0 | 0 | 0 | 0 | 0 |
| 566 | 0.00003 | 0 | 0.931479 | 0.068491 | 0 | 0 | 0 | 0 | 0 |
| 567 | 0.00003 | 0 | 0.928149 | 0.071821 | 0 | 0 | 0 | 0 | 0 |
| 568 | 0.00003 | 0 | 0.924951 | 0.075019 | 0 | 0 | 0 | 0 | 0 |
| 569 | 0 | 0 | 0.930526 | 0.069474 | 0 | 0 | 0 | 0 | 0 |
| 570 | 0.00001 | 0 | 0.925104 | 0.074886 | 0 | 0 | 0 | 0 | 0 |
| 571 | 0.00003 | 0 | 0.926114 | 0.073856 | 0 | 0 | 0 | 0 | 0 |
| 572 | 0.00001 | 0 | 0.930887 | 0.069103 | 0 | 0 | 0 | 0 | 0 |
| 573 | 0.00002 | 0 | 0.931414 | 0.068565 | 0 | 0 | 0 | 0 | 0 |
| 574 | 0.00001 | 0 | 0.92797 | 0.07202 | 0 | 0 | 0 | 0 | 0 |
| 575 | 0.00002 | 0 | 0.927163 | 0.072817 | 0 | 0 | 0 | 0 | 0 |
| 576 | 0.00002 | 0 | 0.934276 | 0.065704 | 0 | 0 | 0 | 0 | 0 |
| 577 | 0.00002 | 0 | 0.926982 | 0.072998 | 0 | 0 | 0 | 0 | 0 |
| 578 | 0 | 0 | 0.931251 | 0.068749 | 0 | 0 | 0 | 0 | 0 |
| 579 | 0.00002 | 0 | 0.925222 | 0.074758 | 0 | 0 | 0 | 0 | 0 |
| 580 | 0.00001 | 0.00001 | 0.929644 | 0.070336 | 0 | 0 | 0 | 0 | 0 |
| 581 | 0.00001 | 0 | 0.926868 | 0.073122 | 0 | 0 | 0 | 0 | 0 |
| 582 | 0 | 0 | 0.927695 | 0.072305 | 0 | 0 | 0 | 0 | 0 |
| 583 | 0.00001 | 0.00005 | 0.925565 | 0.074375 | 0 | 0 | 0 | 0 | 0 |
| 584 | 0.00002 | 0 | 0.925033 | 0.074947 | 0 | 0 | 0 | 0 | 0 |
| 585 | 0.00001 | 0 | 0.926283 | 0.073707 | 0 | 0 | 0 | 0 | 0 |
| 586 | 0.00001 | 0 | 0.926858 | 0.073132 | 0 | 0 | 0 | 0 | 0 |
| 587 | 0.00001 | 0 | 0.927708 | 0.072282 | 0 | 0 | 0 | 0 | 0 |
| 588 | 0.00001 | 0 | 0.932038 | 0.067952 | 0 | 0 | 0 | 0 | 0 |
| 589 | 0.00001 | 0.00001 | 0.928251 | 0.071729 | 0 | 0 | 0 | 0 | 0 |
| 590 | 0.00001 | 0.00001 | 0.924878 | 0.075102 | 0 | 0 | 0 | 0 | 0 |
| 591 | 0.00004 | 0.00001 | 0.923299 | 0.07665 | 0 | 0 | 0 | 0 | 0 |
| 592 | 0.00002 | 0 | 0.92592 | 0.07406 | 0 | 0 | 0 | 0 | 0 |
| 593 | 0.00001 | 0 | 0.929139 | 0.070851 | 0 | 0 | 0 | 0 | 0 |
| 594 | 0.00002 | 0 | 0.925762 | 0.074218 | 0 | 0 | 0 | 0 | 0 |
| 595 | 0 | 0 | 0.92803 | 0.07197 | 0 | 0 | 0 | 0 | 0 |
| 596 | 0.00002 | 0 | 0.930527 | 0.069453 | 0 | 0 | 0 | 0 | 0 |
| 597 | 0 | 0 | 0.931352 | 0.068648 | 0 | 0 | 0 | 0 | 0 |
| 598 | 0 | 0 | 0.929456 | 0.070544 | 0 | 0 | 0 | 0 | 0 |
| 599 | 0 | 0.00001 | 0.929968 | 0.070022 | 0 | 0 | 0 | 0 | 0 |
| 600 | 0.00001 | 0 | 0.925384 | 0.074606 | 0 | 0 | 0 | 0 | 0 |
| 601 | 0 | 0 | 0.927848 | 0.072152 | 0 | 0 | 0 | 0 | 0 |
| 602 | 0.00001 | 0 | 0.931626 | 0.068364 | 0 | 0 | 0 | 0 | 0 |
| 603 | 0.00001 | 0 | 0.928441 | 0.071549 | 0 | 0 | 0 | 0 | 0 |
| 604 | 0.00001 | 0 | 0.92781 | 0.07218 | 0 | 0 | 0 | 0 | 0 |
| 605 | 0.00001 | 0 | 0.92794 | 0.07205 | 0 | 0 | 0 | 0 | 0 |
| 606 | 0.00003 | 0 | 0.929976 | 0.069994 | 0 | 0 | 0 | 0 | 0 |
| 607 | 0.00001 | 0 | 0.932585 | 0.067405 | 0 | 0 | 0 | 0 | 0 |
| 608 | 0.00002 | 0 | 0.929389 | 0.070591 | 0 | 0 | 0 | 0 | 0 |
| 609 | 0.00002 | 0 | 0.930098 | 0.069882 | 0 | 0 | 0 | 0 | 0 |
| 610 | 0 | 0 | 0.925264 | 0.074736 | 0 | 0 | 0 | 0 | 0 |
| 611 | 0.00001 | 0.00001 | 0.927512 | 0.072468 | 0 | 0 | 0 | 0 | 0 |
| 612 | 0.00002 | 0.00001 | 0.928776 | 0.071194 | 0 | 0 | 0 | 0 | 0 |
| 613 | 0.00001 | 0 | 0.923778 | 0.076212 | 0 | 0 | 0 | 0 | 0 |
| 614 | 0.00002 | 0 | 0.930068 | 0.069912 | 0 | 0 | 0 | 0 | 0 |
| 615 | 0.00001 | 0.00002 | 0.930031 | 0.069939 | 0 | 0 | 0 | 0 | 0 |
| 616 | 0 | 0 | 0.928941 | 0.071059 | 0 | 0 | 0 | 0 | 0 |
| 617 | 0.00001 | 0 | 0.929279 | 0.070711 | 0 | 0 | 0 | 0 | 0 |
| 618 | 0.00001 | 0 | 0.924192 | 0.075798 | 0 | 0 | 0 | 0 | 0 |
| 619 | 0.00002 | 0 | 0.931267 | 0.068712 | 0 | 0 | 0 | 0 | 0 |
| 620 | 0 | 0.00001 | 0.928326 | 0.071664 | 0 | 0 | 0 | 0 | 0 |
| 621 | 0.00002 | 0 | 0.930991 | 0.068989 | 0 | 0 | 0 | 0 | 0 |
| 622 | 0 | 0 | 0.92435 | 0.07565 | 0 | 0 | 0 | 0 | 0 |
| 623 | 0.00001 | 0 | 0.92872 | 0.07127 | 0 | 0 | 0 | 0 | 0 |
| 624 | 0 | 0 | 0.927131 | 0.072869 | 0 | 0 | 0 | 0 | 0 |
| 625 | 0.00003 | 0 | 0.929253 | 0.070697 | 0 | 0 | 0 | 0 | 0 |
| 626 | 0.00002 | 0 | 0.924924 | 0.075056 | 0 | 0 | 0 | 0 | 0 |
| 627 | 0 | 0 | 0.929599 | 0.070401 | 0 | 0 | 0 | 0 | 0 |
| 628 | 0.00001 | 0.00001 | 0.928709 | 0.071271 | 0 | 0 | 0 | 0 | 0 |
| 629 | 0 | 0 | 0.92667 | 0.07333 | 0 | 0 | 0 | 0 | 0 |
| 630 | 0.00001 | 0 | 0.928183 | 0.071807 | 0 | 0 | 0 | 0 | 0 |
| 631 | 0.00001 | 0 | 0.925864 | 0.074126 | 0 | 0 | 0 | 0 | 0 |
| 632 | 0 | 0 | 0.928747 | 0.071253 | 0 | 0 | 0 | 0 | 0 |
| 633 | 0.00002 | 0.00001 | 0.928469 | 0.071501 | 0 | 0 | 0 | 0 | 0 |
| 634 | 0 | 0 | 0.926072 | 0.073928 | 0 | 0 | 0 | 0 | 0 |
| 635 | 0 | 0 | 0.934648 | 0.065352 | 0 | 0 | 0 | 0 | 0 |
| 636 | 0 | 0 | 0.926754 | 0.073246 | 0 | 0 | 0 | 0 | 0 |
| 637 | 0.00002 | 0 | 0.925215 | 0.074765 | 0 | 0 | 0 | 0 | 0 |
| 638 | 0 | 0 | 0.927906 | 0.072094 | 0 | 0 | 0 | 0 | 0 |
| 639 | 0.00002 | 0 | 0.926428 | 0.073552 | 0 | 0 | 0 | 0 | 0 |
| 640 | 0.00001 | 0 | 0.923811 | 0.076179 | 0 | 0 | 0 | 0 | 0 |
| 641 | 0 | 0 | 0.929189 | 0.070811 | 0 | 0 | 0 | 0 | 0 |
| 642 | 0 | 0 | 0.929996 | 0.070004 | 0 | 0 | 0 | 0 | 0 |
| 643 | 0.00002 | 0 | 0.927302 | 0.072678 | 0 | 0 | 0 | 0 | 0 |
| 644 | 0 | 0 | 0.926242 | 0.073758 | 0 | 0 | 0 | 0 | 0 |
| 645 | 0.00002 | 0.00001 | 0.929664 | 0.070306 | 0 | 0 | 0 | 0 | 0 |
| 646 | 0.00001 | 0 | 0.927208 | 0.072782 | 0 | 0 | 0 | 0 | 0 |
| 647 | 0.00001 | 0 | 0.926827 | 0.073163 | 0 | 0 | 0 | 0 | 0 |
| 648 | 0.00001 | 0 | 0.93142 | 0.06857 | 0 | 0 | 0 | 0 | 0 |
| 649 | 0.00001 | 0 | 0.929013 | 0.070977 | 0 | 0 | 0 | 0 | 0 |
| 650 | 0 | 0 | 0.929362 | 0.070638 | 0 | 0 | 0 | 0 | 0 |
| 651 | 0 | 0 | 0.929682 | 0.070318 | 0 | 0 | 0 | 0 | 0 |
| 652 | 0 | 0.00001 | 0.927522 | 0.072468 | 0 | 0 | 0 | 0 | 0 |
| 653 | 0.00004 | 0 | 0.931483 | 0.068477 | 0 | 0 | 0 | 0 | 0 |
| 654 | 0.00003 | 0 | 0.928906 | 0.071064 | 0 | 0 | 0 | 0 | 0 |
| 655 | 0.00002 | 0 | 0.926017 | 0.073963 | 0 | 0 | 0 | 0 | 0 |
| 656 | 0.00001 | 0 | 0.935464 | 0.064526 | 0 | 0 | 0 | 0 | 0 |
| 657 | 0.00001 | 0 | 0.927533 | 0.072457 | 0 | 0 | 0 | 0 | 0 |
| 658 | 0 | 0 | 0.929503 | 0.070497 | 0 | 0 | 0 | 0 | 0 |
| 659 | 0 | 0 | 0.925497 | 0.074503 | 0 | 0 | 0 | 0 | 0 |
| 660 | 0.00001 | 0 | 0.932595 | 0.067395 | 0 | 0 | 0 | 0 | 0 |
| 661 | 0 | 0 | 0.92897 | 0.07103 | 0 | 0 | 0 | 0 | 0 |
| 662 | 0.00002 | 0 | 0.928103 | 0.071877 | 0 | 0 | 0 | 0 | 0 |
| 663 | 0.00003 | 0 | 0.928783 | 0.071187 | 0 | 0 | 0 | 0 | 0 |
| 664 | 0.00001 | 0 | 0.933124 | 0.066867 | 0 | 0 | 0 | 0 | 0 |
| 665 | 0.00002 | 0 | 0.928258 | 0.071722 | 0 | 0 | 0 | 0 | 0 |
| 666 | 0.00004 | 0 | 0.929 | 0.07096 | 0 | 0 | 0 | 0 | 0 |
| 667 | 0.00002 | 0 | 0.924135 | 0.075845 | 0 | 0 | 0 | 0 | 0 |
| 668 | 0.00001 | 0 | 0.927397 | 0.072593 | 0 | 0 | 0 | 0 | 0 |
| 669 | 0.00001 | 0.00001 | 0.925335 | 0.074645 | 0 | 0 | 0 | 0 | 0 |
| 670 | 0 | 0 | 0.929301 | 0.070699 | 0 | 0 | 0 | 0 | 0 |
| 671 | 0.00001 | 0 | 0.929254 | 0.070735 | 0 | 0 | 0 | 0 | 0 |
| 672 | 0.00001 | 0.00001 | 0.928381 | 0.071599 | 0 | 0 | 0 | 0 | 0 |
| 673 | 0.00001 | 0 | 0.925609 | 0.074381 | 0 | 0 | 0 | 0 | 0 |
| 674 | 0.00001 | 0 | 0.928382 | 0.071608 | 0 | 0 | 0 | 0 | 0 |
| 675 | 0 | 0 | 0.929218 | 0.070782 | 0 | 0 | 0 | 0 | 0 |
| 676 | 0 | 0 | 0.931244 | 0.068756 | 0 | 0 | 0 | 0 | 0 |
| 677 | 0.00001 | 0 | 0.929142 | 0.070848 | 0 | 0 | 0 | 0 | 0 |
| 678 | 0.00002 | 0 | 0.929206 | 0.070774 | 0 | 0 | 0 | 0 | 0 |
| 679 | 0 | 0 | 0.93164 | 0.06836 | 0 | 0 | 0 | 0 | 0 |
| 680 | 0.00002 | 0 | 0.926483 | 0.073497 | 0 | 0 | 0 | 0 | 0 |
| 681 | 0 | 0 | 0.927383 | 0.072617 | 0 | 0 | 0 | 0 | 0 |
| 682 | 0.00003 | 0 | 0.927895 | 0.072075 | 0 | 0 | 0 | 0 | 0 |
| 683 | 0.00001 | 0 | 0.928391 | 0.071599 | 0 | 0 | 0 | 0 | 0 |
| 684 | 0.00001 | 0 | 0.93067 | 0.06932 | 0 | 0 | 0 | 0 | 0 |
| 685 | 0.00001 | 0 | 0.929653 | 0.070337 | 0 | 0 | 0 | 0 | 0 |
| 686 | 0.00003 | 0 | 0.925458 | 0.074512 | 0 | 0 | 0 | 0 | 0 |
| 687 | 0 | 0.00001 | 0.930924 | 0.069066 | 0 | 0 | 0 | 0 | 0 |
| 688 | 0.00003 | 0 | 0.929641 | 0.070329 | 0 | 0 | 0 | 0 | 0 |
| 689 | 0.00001 | 0 | 0.924715 | 0.075275 | 0 | 0 | 0 | 0 | 0 |
| 690 | 0.00002 | 0 | 0.928964 | 0.071016 | 0 | 0 | 0 | 0 | 0 |
| 691 | 0.00002 | 0 | 0.930836 | 0.069144 | 0 | 0 | 0 | 0 | 0 |
| 692 | 0.00001 | 0 | 0.926592 | 0.073398 | 0 | 0 | 0 | 0 | 0 |
| 693 | 0.00001 | 0 | 0.92451 | 0.07548 | 0 | 0 | 0 | 0 | 0 |
| 694 | 0.00001 | 0.00002 | 0.93237 | 0.0676 | 0 | 0 | 0 | 0 | 0 |
| 695 | 0.00003 | 0 | 0.929075 | 0.070895 | 0 | 0 | 0 | 0 | 0 |
| 696 | 0.00002 | 0 | 0.932574 | 0.067406 | 0 | 0 | 0 | 0 | 0 |
| 697 | 0.00003 | 0 | 0.929692 | 0.070278 | 0 | 0 | 0 | 0 | 0 |
| 698 | 0 | 0.00002 | 0.930157 | 0.069823 | 0 | 0 | 0 | 0 | 0 |
| 699 | 0 | 0 | 0.929027 | 0.070973 | 0 | 0 | 0 | 0 | 0 |
| 700 | 0.00004 | 0 | 0.926916 | 0.073044 | 0 | 0 | 0 | 0 | 0 |
| 701 | 0.00001 | 0.00002 | 0.933731 | 0.066239 | 0 | 0 | 0 | 0 | 0 |
| 702 | 0 | 0.00002 | 0.93151 | 0.06847 | 0 | 0 | 0 | 0 | 0 |
| 703 | 0.00001 | 0 | 0.927153 | 0.072837 | 0 | 0 | 0 | 0 | 0 |
| 704 | 0.00001 | 0 | 0.928548 | 0.071442 | 0 | 0 | 0 | 0 | 0 |
| 705 | 0.00005 | 0 | 0.927503 | 0.072447 | 0 | 0 | 0 | 0 | 0 |
| 706 | 0.00001 | 0 | 0.925808 | 0.074182 | 0 | 0 | 0 | 0 | 0 |
| 707 | 0 | 0 | 0.927902 | 0.072098 | 0 | 0 | 0 | 0 | 0 |
| 708 | 0 | 0 | 0.925468 | 0.074532 | 0 | 0 | 0 | 0 | 0 |
| 709 | 0.00001 | 0 | 0.930967 | 0.069023 | 0 | 0 | 0 | 0 | 0 |
| 710 | 0.00001 | 0 | 0.928884 | 0.071106 | 0 | 0 | 0 | 0 | 0 |
| 711 | 0.00001 | 0 | 0.929584 | 0.070406 | 0 | 0 | 0 | 0 | 0 |
| 712 | 0.00004 | 0 | 0.933006 | 0.066954 | 0 | 0 | 0 | 0 | 0 |
| 713 | 0.00001 | 0 | 0.925238 | 0.074752 | 0 | 0 | 0 | 0 | 0 |
| 714 | 0 | 0 | 0.92541 | 0.07459 | 0 | 0 | 0 | 0 | 0 |
| 715 | 0.00001 | 0.00001 | 0.925852 | 0.074128 | 0 | 0 | 0 | 0 | 0 |
| 716 | 0.00001 | 0 | 0.927046 | 0.072944 | 0 | 0 | 0 | 0 | 0 |
| 717 | 0.00001 | 0 | 0.929683 | 0.070307 | 0 | 0 | 0 | 0 | 0 |
| 718 | 0 | 0 | 0.927252 | 0.072748 | 0 | 0 | 0 | 0 | 0 |
| 719 | 0.00001 | 0 | 0.93275 | 0.06724 | 0 | 0 | 0 | 0 | 0 |
| 720 | 0.00003 | 0 | 0.926962 | 0.073008 | 0 | 0 | 0 | 0 | 0 |
| 721 | 0.00002 | 0 | 0.927123 | 0.072857 | 0 | 0 | 0 | 0 | 0 |
| 722 | 0.00002 | 0 | 0.930249 | 0.069731 | 0 | 0 | 0 | 0 | 0 |
| 723 | 0.00004 | 0 | 0.92803 | 0.07193 | 0 | 0 | 0 | 0 | 0 |
| 724 | 0 | 0 | 0.930688 | 0.069312 | 0 | 0 | 0 | 0 | 0 |
| 725 | 0.00003 | 0 | 0.927833 | 0.072137 | 0 | 0 | 0 | 0 | 0 |
| 726 | 0.00001 | 0 | 0.928372 | 0.071618 | 0 | 0 | 0 | 0 | 0 |
| 727 | 0.00002 | 0 | 0.926388 | 0.073592 | 0 | 0 | 0 | 0 | 0 |
| 728 | 0.00001 | 0 | 0.926211 | 0.073779 | 0 | 0 | 0 | 0 | 0 |
| 729 | 0.00002 | 0.00001 | 0.927578 | 0.072392 | 0 | 0 | 0 | 0 | 0 |
| 730 | 0.00002 | 0.00001 | 0.927677 | 0.072293 | 0 | 0 | 0 | 0 | 0 |
| 731 | 0 | 0 | 0.931966 | 0.068034 | 0 | 0 | 0 | 0 | 0 |
| 732 | 0.00002 | 0 | 0.928963 | 0.071017 | 0 | 0 | 0 | 0 | 0 |
| 733 | 0.00001 | 0 | 0.926543 | 0.073447 | 0 | 0 | 0 | 0 | 0 |
| 734 | 0 | 0.00001 | 0.930451 | 0.069539 | 0 | 0 | 0 | 0 | 0 |
| 735 | 0 | 0 | 0.931582 | 0.068418 | 0 | 0 | 0 | 0 | 0 |
| 736 | 0 | 0 | 0.93188 | 0.06812 | 0 | 0 | 0 | 0 | 0 |
| 737 | 0.00002 | 0.00002 | 0.929412 | 0.070548 | 0 | 0 | 0 | 0 | 0 |
| 738 | 0 | 0 | 0.926677 | 0.073323 | 0 | 0 | 0 | 0 | 0 |
| 739 | 0.00002 | 0 | 0.920234 | 0.079746 | 0 | 0 | 0 | 0 | 0 |
| 740 | 0.00001 | 0 | 0.929126 | 0.070864 | 0 | 0 | 0 | 0 | 0 |
| 741 | 0.00001 | 0 | 0.928096 | 0.071894 | 0 | 0 | 0 | 0 | 0 |
| 742 | 0.00001 | 0 | 0.929019 | 0.070971 | 0 | 0 | 0 | 0 | 0 |
| 743 | 0.00001 | 0 | 0.929569 | 0.070421 | 0 | 0 | 0 | 0 | 0 |
| 744 | 0.00002 | 0 | 0.930908 | 0.069032 | 0 | 0 | 0 | 0 | 0 |
| 745 | 0.00001 | 0 | 0.93074 | 0.06925 | 0 | 0 | 0 | 0 | 0 |
| 746 | 0.00002 | 0.00001 | 0.924016 | 0.075954 | 0 | 0 | 0 | 0 | 0 |
| 747 | 0.00001 | 0 | 0.925687 | 0.074303 | 0 | 0 | 0 | 0 | 0 |
| 748 | 0.00002 | 0 | 0.930753 | 0.069227 | 0 | 0 | 0 | 0 | 0 |
| 749 | 0.00003 | 0 | 0.934292 | 0.065678 | 0 | 0 | 0 | 0 | 0 |
| 750 | 0 | 0 | 0.930534 | 0.069466 | 0 | 0 | 0 | 0 | 0 |
| 751 | 0.00003 | 0 | 0.933817 | 0.066153 | 0 | 0 | 0 | 0 | 0 |
| 752 | 0.00002 | 0 | 0.929855 | 0.070125 | 0 | 0 | 0 | 0 | 0 |
| 753 | 0.00002 | 0 | 0.929812 | 0.070168 | 0 | 0 | 0 | 0 | 0 |
| 754 | 0.00002 | 0 | 0.927086 | 0.072894 | 0 | 0 | 0 | 0 | 0 |
| 755 | 0.00001 | 0 | 0.928068 | 0.071922 | 0 | 0 | 0 | 0 | 0 |
| 756 | 0.00001 | 0 | 0.925943 | 0.074047 | 0 | 0 | 0 | 0 | 0 |
| 757 | 0 | 0 | 0.930099 | 0.069901 | 0 | 0 | 0 | 0 | 0 |
| 758 | 0.00001 | 0 | 0.926893 | 0.073097 | 0 | 0 | 0 | 0 | 0 |
| 759 | 0 | 0.00002 | 0.932178 | 0.067802 | 0 | 0 | 0 | 0 | 0 |
| 760 | 0.00001 | 0 | 0.932571 | 0.067419 | 0 | 0 | 0 | 0 | 0 |
| 761 | 0 | 0 | 0.926285 | 0.073715 | 0 | 0 | 0 | 0 | 0 |
| 762 | 0.00001 | 0 | 0.929318 | 0.070672 | 0 | 0 | 0 | 0 | 0 |
| 763 | 0 | 0 | 0.930254 | 0.069746 | 0 | 0 | 0 | 0 | 0 |
| 764 | 0.00002 | 0 | 0.933436 | 0.066544 | 0 | 0 | 0 | 0 | 0 |
| 765 | 0.00002 | 0 | 0.925096 | 0.074884 | 0 | 0 | 0 | 0 | 0 |
| 766 | 0 | 0 | 0.925045 | 0.074955 | 0 | 0 | 0 | 0 | 0 |
| 767 | 0.00001 | 0 | 0.93079 | 0.0692 | 0 | 0 | 0 | 0 | 0 |
| 768 | 0.00003 | 0 | 0.929617 | 0.070353 | 0 | 0 | 0 | 0 | 0 |
| 769 | 0.00003 | 0 | 0.927839 | 0.072131 | 0 | 0 | 0 | 0 | 0 |
| 770 | 0.00002 | 0 | 0.929048 | 0.070932 | 0 | 0 | 0 | 0 | 0 |
| 771 | 0 | 0 | 0.928278 | 0.071682 | 0 | 0 | 0 | 0 | 0 |
| 772 | 0.00001 | 0 | 0.926022 | 0.073968 | 0 | 0 | 0 | 0 | 0 |
| 773 | 0 | 0 | 0.922799 | 0.077201 | 0 | 0 | 0 | 0 | 0 |
| 774 | 0.00001 | 0 | 0.930165 | 0.069825 | 0 | 0 | 0 | 0 | 0 |
| 775 | 0 | 0 | 0.928496 | 0.071504 | 0 | 0 | 0 | 0 | 0 |
| 776 | 0.00002 | 0 | 0.926725 | 0.073255 | 0 | 0 | 0 | 0 | 0 |
| 777 | 0 | 0 | 0.929143 | 0.070857 | 0 | 0 | 0 | 0 | 0 |
| 778 | 0.00002 | 0 | 0.929943 | 0.070037 | 0 | 0 | 0 | 0 | 0 |
| 779 | 0.00001 | 0.00002 | 0.934116 | 0.065855 | 0 | 0 | 0 | 0 | 0 |
| 780 | 0.00001 | 0 | 0.926784 | 0.073206 | 0 | 0 | 0 | 0 | 0 |
| 781 | 0 | 0.00002 | 0.927203 | 0.072777 | 0 | 0 | 0 | 0 | 0 |
| 782 | 0.00003 | 0 | 0.932849 | 0.067121 | 0 | 0 | 0 | 0 | 0 |
| 783 | 0.00002 | 0.00001 | 0.927196 | 0.072774 | 0 | 0 | 0 | 0 | 0 |
| 784 | 0.00003 | 0 | 0.92785 | 0.072119 | 0 | 0 | 0 | 0 | 0 |
| 785 | 0.00002 | 0.00002 | 0.930926 | 0.069035 | 0 | 0 | 0 | 0 | 0 |
| 786 | 0.00002 | 0 | 0.927669 | 0.07231 | 0 | 0 | 0 | 0 | 0 |
| 787 | 0 | 0 | 0.926799 | 0.073201 | 0 | 0 | 0 | 0 | 0 |
| 788 | 0.00001 | 0 | 0.932196 | 0.067794 | 0 | 0 | 0 | 0 | 0 |
| 789 | 0 | 0 | 0.927485 | 0.072515 | 0 | 0 | 0 | 0 | 0 |
| 790 | 0.00001 | 0 | 0.92822 | 0.07177 | 0 | 0 | 0 | 0 | 0 |
| 791 | 0.00001 | 0 | 0.929858 | 0.070132 | 0 | 0 | 0 | 0 | 0 |
| 792 | 0.00001 | 0 | 0.930377 | 0.069613 | 0 | 0 | 0 | 0 | 0 |
| 793 | 0 | 0 | 0.927446 | 0.072554 | 0 | 0 | 0 | 0 | 0 |
| 794 | 0 | 0 | 0.930099 | 0.069901 | 0 | 0 | 0 | 0 | 0 |
| 795 | 0.00001 | 0 | 0.926999 | 0.072991 | 0 | 0 | 0 | 0 | 0 |
| 796 | 0 | 0 | 0.931826 | 0.068174 | 0 | 0 | 0 | 0 | 0 |
| 797 | 0.00003 | 0 | 0.930915 | 0.069055 | 0 | 0 | 0 | 0 | 0 |
| 798 | 0 | 0.00001 | 0.926701 | 0.073259 | 0 | 0 | 0 | 0 | 0 |
| 799 | 0 | 0 | 0.930676 | 0.069324 | 0 | 0 | 0 | 0 | 0 |
| 800 | 0 | 0 | 0.924485 | 0.075515 | 0 | 0 | 0 | 0 | 0 |
| 801 | 0.00002 | 0 | 0.93246 | 0.06752 | 0 | 0 | 0 | 0 | 0 |
| 802 | 0 | 0 | 0.924961 | 0.075039 | 0 | 0 | 0 | 0 | 0 |
| 803 | 0.00004 | 0.00001 | 0.931669 | 0.068281 | 0 | 0 | 0 | 0 | 0 |
| 804 | 0 | 0.00001 | 0.9287 | 0.07129 | 0 | 0 | 0 | 0 | 0 |
| 805 | 0 | 0 | 0.930422 | 0.069578 | 0 | 0 | 0 | 0 | 0 |
| 806 | 0 | 0 | 0.927416 | 0.072584 | 0 | 0 | 0 | 0 | 0 |
| 807 | 0.00001 | 0 | 0.932219 | 0.06777 | 0 | 0 | 0 | 0 | 0 |
| 808 | 0 | 0 | 0.927988 | 0.072012 | 0 | 0 | 0 | 0 | 0 |
| 809 | 0.00002 | 0 | 0.930322 | 0.069658 | 0 | 0 | 0 | 0 | 0 |
| 810 | 0.00001 | 0 | 0.926653 | 0.073337 | 0 | 0 | 0 | 0 | 0 |
| 811 | 0.00001 | 0 | 0.929531 | 0.070459 | 0 | 0 | 0 | 0 | 0 |
| 812 | 0.00002 | 0 | 0.929483 | 0.070497 | 0 | 0 | 0 | 0 | 0 |
| 813 | 0 | 0.00002 | 0.928525 | 0.071455 | 0 | 0 | 0 | 0 | 0 |
| 814 | 0.00002 | 0 | 0.927535 | 0.072445 | 0 | 0 | 0 | 0 | 0 |
| 815 | 0 | 0 | 0.927851 | 0.072149 | 0 | 0 | 0 | 0 | 0 |
| 816 | 0.00001 | 0 | 0.92968 | 0.07031 | 0 | 0 | 0 | 0 | 0 |
| 817 | 0 | 0 | 0.930861 | 0.069139 | 0 | 0 | 0 | 0 | 0 |
| 818 | 0 | 0 | 0.922819 | 0.077181 | 0 | 0 | 0 | 0 | 0 |
| 819 | 0 | 0 | 0.92704 | 0.07296 | 0 | 0 | 0 | 0 | 0 |
| 820 | 0 | 0 | 0.927751 | 0.072249 | 0 | 0 | 0 | 0 | 0 |
| 821 | 0.00001 | 0.00001 | 0.931046 | 0.068934 | 0 | 0 | 0 | 0 | 0 |
| 822 | 0.00004 | 0 | 0.930126 | 0.069834 | 0 | 0 | 0 | 0 | 0 |
| 823 | 0.00001 | 0 | 0.927803 | 0.072187 | 0 | 0 | 0 | 0 | 0 |
| 824 | 0 | 0 | 0.935136 | 0.064864 | 0 | 0 | 0 | 0 | 0 |
| 825 | 0.00001 | 0 | 0.930945 | 0.069045 | 0 | 0 | 0 | 0 | 0 |
| 826 | 0.00002 | 0 | 0.931265 | 0.068715 | 0 | 0 | 0 | 0 | 0 |
| 827 | 0 | 0 | 0.929352 | 0.070648 | 0 | 0 | 0 | 0 | 0 |
| 828 | 0.00001 | 0 | 0.927423 | 0.072567 | 0 | 0 | 0 | 0 | 0 |
| 829 | 0 | 0 | 0.925419 | 0.074581 | 0 | 0 | 0 | 0 | 0 |
| 830 | 0.00002 | 0 | 0.931702 | 0.068279 | 0 | 0 | 0 | 0 | 0 |
| 831 | 0 | 0 | 0.923373 | 0.076627 | 0 | 0 | 0 | 0 | 0 |
| 832 | 0.00001 | 0 | 0.929077 | 0.070913 | 0 | 0 | 0 | 0 | 0 |
| 833 | 0.00002 | 0 | 0.928513 | 0.071467 | 0 | 0 | 0 | 0 | 0 |
| 834 | 0 | 0 | 0.926434 | 0.073566 | 0 | 0 | 0 | 0 | 0 |
| 835 | 0 | 0 | 0.928869 | 0.071131 | 0 | 0 | 0 | 0 | 0 |
| 836 | 0 | 0 | 0.930422 | 0.069578 | 0 | 0 | 0 | 0 | 0 |
| 837 | 0 | 0 | 0.930501 | 0.069499 | 0 | 0 | 0 | 0 | 0 |
| 838 | 0.00003 | 0 | 0.928057 | 0.071913 | 0 | 0 | 0 | 0 | 0 |
| 839 | 0 | 0 | 0.926237 | 0.073763 | 0 | 0 | 0 | 0 | 0 |
| 840 | 0 | 0 | 0.927941 | 0.072059 | 0 | 0 | 0 | 0 | 0 |
| 841 | 0.00001 | 0 | 0.929388 | 0.070602 | 0 | 0 | 0 | 0 | 0 |
| 842 | 0.00002 | 0 | 0.926917 | 0.073063 | 0 | 0 | 0 | 0 | 0 |
| 843 | 0.00001 | 0 | 0.929058 | 0.070932 | 0 | 0 | 0 | 0 | 0 |
| 844 | 0 | 0 | 0.9283 | 0.0717 | 0 | 0 | 0 | 0 | 0 |
| 845 | 0.00001 | 0 | 0.926077 | 0.073913 | 0 | 0 | 0 | 0 | 0 |
| 846 | 0.00002 | 0 | 0.973869 | 0.026111 | 0 | 0 | 0 | 0 | 0 |
| 847 | 0.00003 | 0 | 0.925349 | 0.074621 | 0 | 0 | 0 | 0 | 0 |
| 848 | 0.00002 | 0 | 0.93139 | 0.06859 | 0 | 0 | 0 | 0 | 0 |
| 849 | 0.00002 | 0 | 0.934495 | 0.065485 | 0 | 0 | 0 | 0 | 0 |
| 850 | 0.00002 | 0 | 0.930076 | 0.069904 | 0 | 0 | 0 | 0 | 0 |
| 851 | 0 | 0 | 0.92957 | 0.07043 | 0 | 0 | 0 | 0 | 0 |
| 852 | 0 | 0 | 0.927312 | 0.072688 | 0 | 0 | 0 | 0 | 0 |
| 853 | 0.00001 | 0 | 0.926294 | 0.073696 | 0 | 0 | 0 | 0 | 0 |
| 854 | 0.00001 | 0 | 0.927823 | 0.072167 | 0 | 0 | 0 | 0 | 0 |
| 855 | 0 | 0 | 0.9252 | 0.0748 | 0 | 0 | 0 | 0 | 0 |
| 856 | 0.00002 | 0 | 0.926677 | 0.073303 | 0 | 0 | 0 | 0 | 0 |
| 857 | 0.00001 | 0 | 0.92965 | 0.07034 | 0 | 0 | 0 | 0 | 0 |
| 858 | 0.00001 | 0 | 0.930727 | 0.069263 | 0 | 0 | 0 | 0 | 0 |
| 859 | 0.00003 | 0 | 0.931014 | 0.068956 | 0 | 0 | 0 | 0 | 0 |
| 860 | 0.00001 | 0 | 0.925002 | 0.074988 | 0 | 0 | 0 | 0 | 0 |
| 861 | 0.00002 | 0 | 0.930316 | 0.069614 | 0 | 0 | 0 | 0 | 0 |
| 862 | 0.00001 | 0 | 0.928735 | 0.071255 | 0 | 0 | 0 | 0 | 0 |
| 863 | 0 | 0 | 0.9259 | 0.0741 | 0 | 0 | 0 | 0 | 0 |
| 864 | 0.00003 | 0 | 0.93042 | 0.06955 | 0 | 0 | 0 | 0 | 0 |
| 865 | 0.00001 | 0 | 0.927728 | 0.072262 | 0 | 0 | 0 | 0 | 0 |
| 866 | 0.00001 | 0 | 0.928694 | 0.071296 | 0 | 0 | 0 | 0 | 0 |
| 867 | 0.00004 | 0 | 0.929631 | 0.070329 | 0 | 0 | 0 | 0 | 0 |
| 868 | 0.00002 | 0.00001 | 0.926109 | 0.073861 | 0 | 0 | 0 | 0 | 0 |
| 869 | 0.00004 | 0 | 0.927468 | 0.072492 | 0 | 0 | 0 | 0 | 0 |
| 870 | 0.00001 | 0 | 0.930059 | 0.069931 | 0 | 0 | 0 | 0 | 0 |
| 871 | 0.00002 | 0.00002 | 0.934574 | 0.065386 | 0 | 0 | 0 | 0 | 0 |
| 872 | 0 | 0 | 0.927627 | 0.072373 | 0 | 0 | 0 | 0 | 0 |
| 873 | 0.00001 | 0 | 0.925208 | 0.074782 | 0 | 0 | 0 | 0 | 0 |
| 874 | 0 | 0 | 0.928231 | 0.071769 | 0 | 0 | 0 | 0 | 0 |
| 875 | 0.00001 | 0 | 0.931846 | 0.068144 | 0 | 0 | 0 | 0 | 0 |
| 876 | 0 | 0 | 0.929184 | 0.070816 | 0 | 0 | 0 | 0 | 0 |
| 877 | 0.00004 | 0 | 0.933499 | 0.066461 | 0 | 0 | 0 | 0 | 0 |
| 878 | 0.00002 | 0 | 0.931504 | 0.068477 | 0 | 0 | 0 | 0 | 0 |
| 879 | 0.00001 | 0 | 0.927149 | 0.072841 | 0 | 0 | 0 | 0 | 0 |
| 880 | 0 | 0 | 0.928879 | 0.071121 | 0 | 0 | 0 | 0 | 0 |
| 881 | 0.00002 | 0 | 0.927099 | 0.072881 | 0 | 0 | 0 | 0 | 0 |
| 882 | 0.00002 | 0 | 0.926321 | 0.073659 | 0 | 0 | 0 | 0 | 0 |
| 883 | 0 | 0 | 0.928013 | 0.071987 | 0 | 0 | 0 | 0 | 0 |
| 884 | 0.00003 | 0 | 0.926665 | 0.073305 | 0 | 0 | 0 | 0 | 0 |
| 885 | 0 | 0 | 0.933929 | 0.066071 | 0 | 0 | 0 | 0 | 0 |
| 886 | 0.00001 | 0 | 0.926965 | 0.073025 | 0 | 0 | 0 | 0 | 0 |
| 887 | 0 | 0 | 0.929412 | 0.070588 | 0 | 0 | 0 | 0 | 0 |
| 888 | 0.00001 | 0 | 0.925141 | 0.074849 | 0 | 0 | 0 | 0 | 0 |
| 889 | 0.00002 | 0 | 0.922183 | 0.077797 | 0 | 0 | 0 | 0 | 0 |
| 890 | 0.00002 | 0 | 0.931388 | 0.068592 | 0 | 0 | 0 | 0 | 0 |
| 891 | 0.00001 | 0.00001 | 0.924946 | 0.075034 | 0 | 0 | 0 | 0 | 0 |
| 892 | 0.00001 | 0 | 0.929166 | 0.070824 | 0 | 0 | 0 | 0 | 0 |
| 893 | 0 | 0 | 0.929129 | 0.070871 | 0 | 0 | 0 | 0 | 0 |
| 894 | 0.00001 | 0 | 0.92443 | 0.07556 | 0 | 0 | 0 | 0 | 0 |
| 895 | 0 | 0.00001 | 0.934121 | 0.065869 | 0 | 0 | 0 | 0 | 0 |
| 896 | 0.00002 | 0 | 0.92688 | 0.0731 | 0 | 0 | 0 | 0 | 0 |
| 897 | 0 | 0 | 0.930951 | 0.069049 | 0 | 0 | 0 | 0 | 0 |
| 898 | 0.00004 | 0 | 0.92999 | 0.06997 | 0 | 0 | 0 | 0 | 0 |
| 899 | 0.00001 | 0 | 0.930892 | 0.069098 | 0 | 0 | 0 | 0 | 0 |
| 900 | 0.00002 | 0 | 0.930294 | 0.069686 | 0 | 0 | 0 | 0 | 0 |
| 901 | 0 | 0 | 0.931691 | 0.068309 | 0 | 0 | 0 | 0 | 0 |
| 902 | 0.00001 | 0 | 0.925972 | 0.074018 | 0 | 0 | 0 | 0 | 0 |
| 903 | 0 | 0 | 0.928173 | 0.071827 | 0 | 0 | 0 | 0 | 0 |
| 904 | 0.00001 | 0 | 0.932985 | 0.067005 | 0 | 0 | 0 | 0 | 0 |
| 905 | 0 | 0 | 0.93028 | 0.06972 | 0 | 0 | 0 | 0 | 0 |
| 906 | 0 | 0 | 0.928971 | 0.071029 | 0 | 0 | 0 | 0 | 0 |
| 907 | 0.00001 | 0 | 0.935153 | 0.064837 | 0 | 0 | 0 | 0 | 0 |
| 908 | 0.00001 | 0 | 0.927669 | 0.072321 | 0 | 0 | 0 | 0 | 0 |
| 909 | 0.00001 | 0 | 0.928939 | 0.071051 | 0 | 0 | 0 | 0 | 0 |
| 910 | 0 | 0 | 0.928581 | 0.071419 | 0 | 0 | 0 | 0 | 0 |
| 911 | 0.00001 | 0 | 0.932408 | 0.067582 | 0 | 0 | 0 | 0 | 0 |
| 912 | 0 | 0 | 0.927207 | 0.072793 | 0 | 0 | 0 | 0 | 0 |
| 913 | 0 | 0.00001 | 0.92999 | 0.069999 | 0 | 0 | 0 | 0 | 0 |
| 914 | 0.00003 | 0 | 0.931581 | 0.068389 | 0 | 0 | 0 | 0 | 0 |
| 915 | 0 | 0 | 0.925073 | 0.074927 | 0 | 0 | 0 | 0 | 0 |
| 916 | 0.00001 | 0 | 0.928384 | 0.071606 | 0 | 0 | 0 | 0 | 0 |
| 917 | 0.00002 | 0.00001 | 0.92959 | 0.07038 | 0 | 0 | 0 | 0 | 0 |
| 918 | 0.00001 | 0 | 0.931864 | 0.068126 | 0 | 0 | 0 | 0 | 0 |
| 919 | 0.00001 | 0 | 0.926144 | 0.073846 | 0 | 0 | 0 | 0 | 0 |
| 920 | 0.00001 | 0 | 0.933525 | 0.066465 | 0 | 0 | 0 | 0 | 0 |
| 921 | 0 | 0.00002 | 0.930238 | 0.069742 | 0 | 0 | 0 | 0 | 0 |
| 922 | 0.00001 | 0 | 0.92926 | 0.07073 | 0 | 0 | 0 | 0 | 0 |
| 923 | 0.00004 | 0 | 0.928007 | 0.071953 | 0 | 0 | 0 | 0 | 0 |
| 924 | 0.00001 | 0 | 0.928538 | 0.071452 | 0 | 0 | 0 | 0 | 0 |
| 925 | 0.00001 | 0 | 0.927447 | 0.072543 | 0 | 0 | 0 | 0 | 0 |
| 926 | 0.00002 | 0 | 0.931431 | 0.068549 | 0 | 0 | 0 | 0 | 0 |
| 927 | 0.00001 | 0 | 0.927032 | 0.072958 | 0 | 0 | 0 | 0 | 0 |
| 928 | 0.00001 | 0 | 0.928834 | 0.071156 | 0 | 0 | 0 | 0 | 0 |
| 929 | 0.00001 | 0 | 0.922931 | 0.077059 | 0 | 0 | 0 | 0 | 0 |
| 930 | 0 | 0 | 0.92936 | 0.07064 | 0 | 0 | 0 | 0 | 0 |
| 931 | 0.00004 | 0 | 0.932102 | 0.067858 | 0 | 0 | 0 | 0 | 0 |
| 932 | 0.00001 | 0 | 0.930401 | 0.069589 | 0 | 0 | 0 | 0 | 0 |
| 933 | 0.00002 | 0 | 0.934645 | 0.065335 | 0 | 0 | 0 | 0 | 0 |
| 934 | 0.00002 | 0 | 0.925214 | 0.074766 | 0 | 0 | 0 | 0 | 0 |
| 935 | 0.00001 | 0 | 0.928931 | 0.071059 | 0 | 0 | 0 | 0 | 0 |
| 936 | 0 | 0 | 0.930605 | 0.069395 | 0 | 0 | 0 | 0 | 0 |
| 937 | 0.00002 | 0 | 0.929152 | 0.070827 | 0 | 0 | 0 | 0 | 0 |
| 938 | 0.00002 | 0 | 0.931927 | 0.068053 | 0 | 0 | 0 | 0 | 0 |
| 939 | 0 | 0 | 0.932162 | 0.067838 | 0 | 0 | 0 | 0 | 0 |
| 940 | 0 | 0 | 0.924905 | 0.075095 | 0 | 0 | 0 | 0 | 0 |
| 941 | 0.00001 | 0 | 0.929417 | 0.070573 | 0 | 0 | 0 | 0 | 0 |
| 942 | 0 | 0 | 0.928781 | 0.071219 | 0 | 0 | 0 | 0 | 0 |
| 943 | 0.00002 | 0 | 0.930495 | 0.069485 | 0 | 0 | 0 | 0 | 0 |
| 944 | 0 | 0 | 0.930344 | 0.069656 | 0 | 0 | 0 | 0 | 0 |
| 945 | 0.00002 | 0 | 0.924195 | 0.075785 | 0 | 0 | 0 | 0 | 0 |
| 946 | 0.00002 | 0 | 0.927415 | 0.072566 | 0 | 0 | 0 | 0 | 0 |
| 947 | 0.00001 | 0 | 0.927572 | 0.072418 | 0 | 0 | 0 | 0 | 0 |
| 948 | 0.00002 | 0 | 0.928868 | 0.071112 | 0 | 0 | 0 | 0 | 0 |
| 949 | 0.00001 | 0 | 0.931444 | 0.068546 | 0 | 0 | 0 | 0 | 0 |
| 950 | 0.00003 | 0 | 0.931404 | 0.068566 | 0 | 0 | 0 | 0 | 0 |
| 951 | 0.00001 | 0.00003 | 0.932392 | 0.067568 | 0 | 0 | 0 | 0 | 0 |
| 952 | 0 | 0 | 0.931667 | 0.068333 | 0 | 0 | 0 | 0 | 0 |
| 953 | 0.00003 | 0 | 0.927814 | 0.072156 | 0 | 0 | 0 | 0 | 0 |
| 954 | 0 | 0 | 0.925184 | 0.074816 | 0 | 0 | 0 | 0 | 0 |
| 955 | 0.00002 | 0 | 0.930977 | 0.069003 | 0 | 0 | 0 | 0 | 0 |
| 956 | 0.00002 | 0 | 0.931935 | 0.068045 | 0 | 0 | 0 | 0 | 0 |
| 957 | 0.00001 | 0 | 0.923767 | 0.076223 | 0 | 0 | 0 | 0 | 0 |
| 958 | 0.00002 | 0 | 0.930674 | 0.069306 | 0 | 0 | 0 | 0 | 0 |
| 959 | 0 | 0 | 0.927704 | 0.072296 | 0 | 0 | 0 | 0 | 0 |
| 960 | 0.00001 | 0 | 0.932759 | 0.067231 | 0 | 0 | 0 | 0 | 0 |
| 961 | 0.00001 | 0 | 0.926447 | 0.073543 | 0 | 0 | 0 | 0 | 0 |
| 962 | 0.00002 | 0 | 0.929068 | 0.070912 | 0 | 0 | 0 | 0 | 0 |
| 963 | 0.00001 | 0 | 0.927648 | 0.072342 | 0 | 0 | 0 | 0 | 0 |
| 964 | 0.00002 | 0 | 0.931068 | 0.068912 | 0 | 0 | 0 | 0 | 0 |
| 965 | 0.00002 | 0 | 0.932488 | 0.067492 | 0 | 0 | 0 | 0 | 0 |
| 966 | 0.00006 | 0 | 0.932093 | 0.067847 | 0 | 0 | 0 | 0 | 0 |
| 967 | 0 | 0.00001 | 0.933642 | 0.066348 | 0 | 0 | 0 | 0 | 0 |
| 968 | 0.00003 | 0 | 0.926781 | 0.073189 | 0 | 0 | 0 | 0 | 0 |
| 969 | 0 | 0.00003 | 0.926201 | 0.073769 | 0 | 0 | 0 | 0 | 0 |
| 970 | 0 | 0 | 0.926635 | 0.073365 | 0 | 0 | 0 | 0 | 0 |
| 971 | 0 | 0 | 0.930514 | 0.069486 | 0 | 0 | 0 | 0 | 0 |
| 972 | 0.00001 | 0 | 0.926012 | 0.073978 | 0 | 0 | 0 | 0 | 0 |
| 973 | 0.00001 | 0 | 0.930077 | 0.069913 | 0 | 0 | 0 | 0 | 0 |
| 974 | 0.00001 | 0 | 0.925463 | 0.074527 | 0 | 0 | 0 | 0 | 0 |
| 975 | 0.00003 | 0 | 0.933281 | 0.066689 | 0 | 0 | 0 | 0 | 0 |
| 976 | 0.00001 | 0 | 0.929616 | 0.070374 | 0 | 0 | 0 | 0 | 0 |
| 977 | 0.00001 | 0 | 0.928121 | 0.071869 | 0 | 0 | 0 | 0 | 0 |
| 978 | 0 | 0 | 0.929738 | 0.070262 | 0 | 0 | 0 | 0 | 0 |
| 979 | 0 | 0 | 0.926553 | 0.073447 | 0 | 0 | 0 | 0 | 0 |
| 980 | 0.00002 | 0 | 0.930219 | 0.069761 | 0 | 0 | 0 | 0 | 0 |
| 981 | 0.00002 | 0 | 0.931733 | 0.068247 | 0 | 0 | 0 | 0 | 0 |
| 982 | 0.00003 | 0 | 0.92438 | 0.07559 | 0 | 0 | 0 | 0 | 0 |
| 983 | 0 | 0 | 0.927088 | 0.072912 | 0 | 0 | 0 | 0 | 0 |
| 984 | 0.00001 | 0 | 0.929092 | 0.070898 | 0 | 0 | 0 | 0 | 0 |
| 985 | 0.00001 | 0 | 0.92829 | 0.0717 | 0 | 0 | 0 | 0 | 0 |
| 986 | 0 | 0 | 0.93234 | 0.06766 | 0 | 0 | 0 | 0 | 0 |
| 987 | 0.00002 | 0 | 0.931326 | 0.068654 | 0 | 0 | 0 | 0 | 0 |
| 988 | 0.00001 | 0 | 0.931178 | 0.068812 | 0 | 0 | 0 | 0 | 0 |
| 989 | 0.00002 | 0 | 0.927301 | 0.072679 | 0 | 0 | 0 | 0 | 0 |
| 990 | 0.00003 | 0.00001 | 0.925413 | 0.074547 | 0 | 0 | 0 | 0 | 0 |
| 991 | 0 | 0 | 0.929587 | 0.070413 | 0 | 0 | 0 | 0 | 0 |
| 992 | 0.00002 | 0 | 0.930248 | 0.069732 | 0 | 0 | 0 | 0 | 0 |
| 993 | 0.00001 | 0 | 0.932524 | 0.067466 | 0 | 0 | 0 | 0 | 0 |
| 994 | 0 | 0.00001 | 0.933664 | 0.066326 | 0 | 0 | 0 | 0 | 0 |
| 995 | 0.00001 | 0 | 0.926121 | 0.073869 | 0 | 0 | 0 | 0 | 0 |
| 996 | 0.00002 | 0 | 0.932844 | 0.067136 | 0 | 0 | 0 | 0 | 0 |
| 997 | 0.00002 | 0 | 0.925155 | 0.074825 | 0 | 0 | 0 | 0 | 0 |
| 998 | 0.00001 | 0 | 0.927641 | 0.072349 | 0 | 0 | 0 | 0 | 0 |
| 999 | 0 | 0 | 0.933711 | 0.066289 | 0 | 0 | 0 | 0 | 0 |
| 1000 | 0.00001 | 0 | 0.925751 | 0.074239 | 0 | 0 | 0 | 0 | 0 |

| Year 13 | Race |  |  |  |  |  |  |  |  |
| --- | --- | --- | --- | --- | --- | --- | --- | --- | --- |
| Trial | 003.0 | 005.0 | 007.0 | 037.1 | 043.0 | 303.0 | 003.2 | 403.0 | 003.4 |
| 1 | 0.00002 | 0 | 0.933067 | 0.066913 | 0 | 0 | 0 | 0 | 0 |
| 2 | 0 | 0 | 0.930954 | 0.069046 | 0 | 0 | 0 | 0 | 0 |
| 3 | 0 | 0 | 0.936183 | 0.063817 | 0 | 0 | 0 | 0 | 0 |
| 4 | 0 | 0 | 0.934575 | 0.065425 | 0 | 0 | 0 | 0 | 0 |
| 5 | 0.00001 | 0 | 0.932698 | 0.067292 | 0 | 0 | 0 | 0 | 0 |
| 6 | 0.00001 | 0 | 0.934942 | 0.065048 | 0 | 0 | 0 | 0 | 0 |
| 7 | 0.00002 | 0 | 0.934077 | 0.065903 | 0 | 0 | 0 | 0 | 0 |
| 8 | 0.00001 | 0 | 0.936511 | 0.063479 | 0 | 0 | 0 | 0 | 0 |
| 9 | 0.00001 | 0 | 0.931338 | 0.068652 | 0 | 0 | 0 | 0 | 0 |
| 10 | 0.00001 | 0.00001 | 0.933185 | 0.066795 | 0 | 0 | 0 | 0 | 0 |
| 11 | 0 | 0 | 0.938078 | 0.061922 | 0 | 0 | 0 | 0 | 0 |
| 12 | 0.00002 | 0 | 0.930981 | 0.068999 | 0 | 0 | 0 | 0 | 0 |
| 13 | 0.00001 | 0 | 0.934287 | 0.065703 | 0 | 0 | 0 | 0 | 0 |
| 14 | 0.00001 | 0 | 0.930954 | 0.069036 | 0 | 0 | 0 | 0 | 0 |
| 15 | 0.00001 | 0 | 0.930991 | 0.068999 | 0 | 0 | 0 | 0 | 0 |
| 16 | 0.00001 | 0 | 0.932772 | 0.067218 | 0 | 0 | 0 | 0 | 0 |
| 17 | 0 | 0 | 0.934684 | 0.065316 | 0 | 0 | 0 | 0 | 0 |
| 18 | 0.00001 | 0 | 0.93421 | 0.06578 | 0 | 0 | 0 | 0 | 0 |
| 19 | 0.00002 | 0 | 0.932973 | 0.067007 | 0 | 0 | 0 | 0 | 0 |
| 20 | 0 | 0 | 0.935845 | 0.064155 | 0 | 0 | 0 | 0 | 0 |
| 21 | 0.00001 | 0 | 0.933643 | 0.066347 | 0 | 0 | 0 | 0 | 0 |
| 22 | 0.00001 | 0 | 0.933007 | 0.066983 | 0 | 0 | 0 | 0 | 0 |
| 23 | 0.00001 | 0 | 0.936293 | 0.063697 | 0 | 0 | 0 | 0 | 0 |
| 24 | 0 | 0 | 0.93557 | 0.06443 | 0 | 0 | 0 | 0 | 0 |
| 25 | 0 | 0 | 0.933128 | 0.066872 | 0 | 0 | 0 | 0 | 0 |
| 26 | 0.00003 | 0 | 0.936218 | 0.063752 | 0 | 0 | 0 | 0 | 0 |
| 27 | 0.00002 | 0 | 0.933645 | 0.066335 | 0 | 0 | 0 | 0 | 0 |
| 28 | 0.00001 | 0 | 0.938189 | 0.061731 | 0 | 0 | 0 | 0 | 0 |
| 29 | 0.00004 | 0 | 0.936388 | 0.063572 | 0 | 0 | 0 | 0 | 0 |
| 30 | 0.00001 | 0 | 0.931276 | 0.068714 | 0 | 0 | 0 | 0 | 0 |
| 31 | 0.00002 | 0 | 0.936044 | 0.063936 | 0 | 0 | 0 | 0 | 0 |
| 32 | 0.00002 | 0 | 0.936451 | 0.063529 | 0 | 0 | 0 | 0 | 0 |
| 33 | 0 | 0 | 0.933374 | 0.066626 | 0 | 0 | 0 | 0 | 0 |
| 34 | 0.00002 | 0 | 0.93503 | 0.06495 | 0 | 0 | 0 | 0 | 0 |
| 35 | 0.00004 | 0 | 0.930386 | 0.069574 | 0 | 0 | 0 | 0 | 0 |
| 36 | 0.00002 | 0 | 0.931543 | 0.068437 | 0 | 0 | 0 | 0 | 0 |
| 37 | 0 | 0 | 0.939707 | 0.060293 | 0 | 0 | 0 | 0 | 0 |
| 38 | 0.00002 | 0 | 0.935742 | 0.064238 | 0 | 0 | 0 | 0 | 0 |
| 39 | 0.00002 | 0 | 0.932834 | 0.067146 | 0 | 0 | 0 | 0 | 0 |
| 40 | 0.00002 | 0 | 0.933226 | 0.066754 | 0 | 0 | 0 | 0 | 0 |
| 41 | 0 | 0 | 0.928946 | 0.071054 | 0 | 0 | 0 | 0 | 0 |
| 42 | 0.00001 | 0 | 0.934636 | 0.065354 | 0 | 0 | 0 | 0 | 0 |
| 43 | 0.00002 | 0 | 0.933601 | 0.066379 | 0 | 0 | 0 | 0 | 0 |
| 44 | 0.00003 | 0.00001 | 0.93256 | 0.0674 | 0 | 0 | 0 | 0 | 0 |
| 45 | 0.00002 | 0 | 0.935997 | 0.063983 | 0 | 0 | 0 | 0 | 0 |
| 46 | 0.00002 | 0 | 0.93327 | 0.066709 | 0 | 0 | 0 | 0 | 0 |
| 47 | 0.00003 | 0 | 0.935112 | 0.064858 | 0 | 0 | 0 | 0 | 0 |
| 48 | 0.00002 | 0 | 0.932728 | 0.067252 | 0 | 0 | 0 | 0 | 0 |
| 49 | 0.00001 | 0.00001 | 0.935787 | 0.064193 | 0 | 0 | 0 | 0 | 0 |
| 50 | 0.00001 | 0 | 0.936386 | 0.063604 | 0 | 0 | 0 | 0 | 0 |
| 51 | 0.00002 | 0 | 0.933549 | 0.066431 | 0 | 0 | 0 | 0 | 0 |
| 52 | 0.00002 | 0.00002 | 0.937947 | 0.062013 | 0 | 0 | 0 | 0 | 0 |
| 53 | 0.00005 | 0 | 0.93413 | 0.06582 | 0 | 0 | 0 | 0 | 0 |
| 54 | 0.00001 | 0 | 0.931803 | 0.068187 | 0 | 0 | 0 | 0 | 0 |
| 55 | 0.00002 | 0 | 0.931325 | 0.068655 | 0 | 0 | 0 | 0 | 0 |
| 56 | 0.00001 | 0 | 0.934443 | 0.065547 | 0 | 0 | 0 | 0 | 0 |
| 57 | 0.00003 | 0 | 0.937634 | 0.062336 | 0 | 0 | 0 | 0 | 0 |
| 58 | 0.00001 | 0 | 0.933231 | 0.066759 | 0 | 0 | 0 | 0 | 0 |
| 59 | 0.00001 | 0 | 0.937823 | 0.062167 | 0 | 0 | 0 | 0 | 0 |
| 60 | 0.00005 | 0 | 0.934559 | 0.065391 | 0 | 0 | 0 | 0 | 0 |
| 61 | 0.00001 | 0 | 0.928891 | 0.071099 | 0 | 0 | 0 | 0 | 0 |
| 62 | 0.00002 | 0 | 0.932417 | 0.067563 | 0 | 0 | 0 | 0 | 0 |
| 63 | 0.00002 | 0 | 0.932674 | 0.067306 | 0 | 0 | 0 | 0 | 0 |
| 64 | 0.00002 | 0 | 0.93279 | 0.06719 | 0 | 0 | 0 | 0 | 0 |
| 65 | 0.00001 | 0 | 0.936293 | 0.063697 | 0 | 0 | 0 | 0 | 0 |
| 66 | 0.00002 | 0 | 0.938564 | 0.061416 | 0 | 0 | 0 | 0 | 0 |
| 67 | 0 | 0 | 0.935377 | 0.064623 | 0 | 0 | 0 | 0 | 0 |
| 68 | 0 | 0 | 0.935425 | 0.064575 | 0 | 0 | 0 | 0 | 0 |
| 69 | 0.00001 | 0 | 0.932682 | 0.067307 | 0 | 0 | 0 | 0 | 0 |
| 70 | 0.00003 | 0 | 0.935222 | 0.064748 | 0 | 0 | 0 | 0 | 0 |
| 71 | 0.00001 | 0 | 0.932315 | 0.067338 | 0 | 0 | 0 | 0 | 0 |
| 72 | 0.00002 | 0 | 0.938981 | 0.060999 | 0 | 0 | 0 | 0 | 0 |
| 73 | 0.00001 | 0 | 0.933119 | 0.066871 | 0 | 0 | 0 | 0 | 0 |
| 74 | 0.00002 | 0 | 0.937588 | 0.062392 | 0 | 0 | 0 | 0 | 0 |
| 75 | 0.00003 | 0 | 0.933948 | 0.066022 | 0 | 0 | 0 | 0 | 0 |
| 76 | 0.00002 | 0 | 0.936288 | 0.063692 | 0 | 0 | 0 | 0 | 0 |
| 77 | 0 | 0 | 0.934716 | 0.065284 | 0 | 0 | 0 | 0 | 0 |
| 78 | 0.00002 | 0 | 0.934461 | 0.065519 | 0 | 0 | 0 | 0 | 0 |
| 79 | 0.00002 | 0 | 0.934376 | 0.065604 | 0 | 0 | 0 | 0 | 0 |
| 80 | 0.00003 | 0 | 0.933453 | 0.066518 | 0 | 0 | 0 | 0 | 0 |
| 81 | 0 | 0.00001 | 0.935595 | 0.064395 | 0 | 0 | 0 | 0 | 0 |
| 82 | 0.00002 | 0 | 0.927719 | 0.072261 | 0 | 0 | 0 | 0 | 0 |
| 83 | 0.00001 | 0 | 0.939578 | 0.060412 | 0 | 0 | 0 | 0 | 0 |
| 84 | 0.00002 | 0 | 0.934873 | 0.065107 | 0 | 0 | 0 | 0 | 0 |
| 85 | 0.00002 | 0 | 0.932558 | 0.067422 | 0 | 0 | 0 | 0 | 0 |
| 86 | 0.00001 | 0 | 0.941696 | 0.058294 | 0 | 0 | 0 | 0 | 0 |
| 87 | 0.00001 | 0 | 0.933085 | 0.066905 | 0 | 0 | 0 | 0 | 0 |
| 88 | 0.00001 | 0 | 0.934143 | 0.065847 | 0 | 0 | 0 | 0 | 0 |
| 89 | 0.00002 | 0 | 0.930722 | 0.069257 | 0 | 0 | 0 | 0 | 0 |
| 90 | 0.00002 | 0 | 0.93467 | 0.06531 | 0 | 0 | 0 | 0 | 0 |
| 91 | 0.00003 | 0 | 0.933598 | 0.066372 | 0 | 0 | 0 | 0 | 0 |
| 92 | 0.00004 | 0 | 0.934607 | 0.065353 | 0 | 0 | 0 | 0 | 0 |
| 93 | 0 | 0 | 0.937388 | 0.062612 | 0 | 0 | 0 | 0 | 0 |
| 94 | 0 | 0 | 0.937385 | 0.062615 | 0 | 0 | 0 | 0 | 0 |
| 95 | 0.00002 | 0 | 0.933254 | 0.066726 | 0 | 0 | 0 | 0 | 0 |
| 96 | 0 | 0 | 0.936831 | 0.063169 | 0 | 0 | 0 | 0 | 0 |
| 97 | 0.00002 | 0 | 0.932901 | 0.067079 | 0 | 0 | 0 | 0 | 0 |
| 98 | 0 | 0 | 0.933167 | 0.066833 | 0 | 0 | 0 | 0 | 0 |
| 99 | 0.00002 | 0 | 0.930906 | 0.069074 | 0 | 0 | 0 | 0 | 0 |
| 100 | 0 | 0 | 0.936898 | 0.063102 | 0 | 0 | 0 | 0 | 0 |
| 101 | 0.00001 | 0 | 0.936664 | 0.063326 | 0 | 0 | 0 | 0 | 0 |
| 102 | 0.00002 | 0 | 0.935705 | 0.064276 | 0 | 0 | 0 | 0 | 0 |
| 103 | 0.00003 | 0 | 0.934899 | 0.065071 | 0 | 0 | 0 | 0 | 0 |
| 104 | 0.00001 | 0 | 0.93416 | 0.06583 | 0 | 0 | 0 | 0 | 0 |
| 105 | 0 | 0 | 0.935381 | 0.064619 | 0 | 0 | 0 | 0 | 0 |
| 106 | 0 | 0.00001 | 0.935395 | 0.064595 | 0 | 0 | 0 | 0 | 0 |
| 107 | 0.00002 | 0 | 0.936635 | 0.063194 | 0 | 0 | 0 | 0 | 0 |
| 108 | 0 | 0 | 0.930145 | 0.069855 | 0 | 0 | 0 | 0 | 0 |
| 109 | 0.00004 | 0 | 0.936738 | 0.063222 | 0 | 0 | 0 | 0 | 0 |
| 110 | 0 | 0 | 0.933712 | 0.066288 | 0 | 0 | 0 | 0 | 0 |
| 111 | 0 | 0 | 0.93628 | 0.06372 | 0 | 0 | 0 | 0 | 0 |
| 112 | 0 | 0 | 0.933454 | 0.066546 | 0 | 0 | 0 | 0 | 0 |
| 113 | 0.00002 | 0 | 0.932618 | 0.067362 | 0 | 0 | 0 | 0 | 0 |
| 114 | 0 | 0 | 0.933886 | 0.066114 | 0 | 0 | 0 | 0 | 0 |
| 115 | 0.00003 | 0 | 0.937623 | 0.062347 | 0 | 0 | 0 | 0 | 0 |
| 116 | 0.00003 | 0 | 0.933237 | 0.066733 | 0 | 0 | 0 | 0 | 0 |
| 117 | 0.00001 | 0 | 0.934182 | 0.065807 | 0 | 0 | 0 | 0 | 0 |
| 118 | 0 | 0 | 0.935571 | 0.064429 | 0 | 0 | 0 | 0 | 0 |
| 119 | 0.00001 | 0 | 0.932762 | 0.067228 | 0 | 0 | 0 | 0 | 0 |
| 120 | 0 | 0 | 0.933231 | 0.066769 | 0 | 0 | 0 | 0 | 0 |
| 121 | 0 | 0 | 0.934898 | 0.065102 | 0 | 0 | 0 | 0 | 0 |
| 122 | 0.00001 | 0 | 0.937585 | 0.062405 | 0 | 0 | 0 | 0 | 0 |
| 123 | 0.00002 | 0 | 0.936516 | 0.063464 | 0 | 0 | 0 | 0 | 0 |
| 124 | 0 | 0 | 0.937276 | 0.062724 | 0 | 0 | 0 | 0 | 0 |
| 125 | 0.00002 | 0 | 0.933648 | 0.066332 | 0 | 0 | 0 | 0 | 0 |
| 126 | 0.00002 | 0 | 0.935532 | 0.064448 | 0 | 0 | 0 | 0 | 0 |
| 127 | 0 | 0 | 0.934919 | 0.065081 | 0 | 0 | 0 | 0 | 0 |
| 128 | 0 | 0 | 0.932335 | 0.067665 | 0 | 0 | 0 | 0 | 0 |
| 129 | 0 | 0 | 0.934988 | 0.065012 | 0 | 0 | 0 | 0 | 0 |
| 130 | 0.00001 | 0 | 0.935452 | 0.064538 | 0 | 0 | 0 | 0 | 0 |
| 131 | 0.00002 | 0 | 0.935871 | 0.064109 | 0 | 0 | 0 | 0 | 0 |
| 132 | 0 | 0 | 0.934681 | 0.065319 | 0 | 0 | 0 | 0 | 0 |
| 133 | 0.00003 | 0 | 0.932551 | 0.067419 | 0 | 0 | 0 | 0 | 0 |
| 134 | 0.00003 | 0 | 0.936395 | 0.063575 | 0 | 0 | 0 | 0 | 0 |
| 135 | 0.00003 | 0 | 0.930782 | 0.069188 | 0 | 0 | 0 | 0 | 0 |
| 136 | 0.00002 | 0 | 0.935435 | 0.064545 | 0 | 0 | 0 | 0 | 0 |
| 137 | 0.00003 | 0 | 0.935122 | 0.064848 | 0 | 0 | 0 | 0 | 0 |
| 138 | 0.00002 | 0 | 0.93216 | 0.06782 | 0 | 0 | 0 | 0 | 0 |
| 139 | 0.00002 | 0 | 0.936703 | 0.063277 | 0 | 0 | 0 | 0 | 0 |
| 140 | 0 | 0 | 0.932556 | 0.067444 | 0 | 0 | 0 | 0 | 0 |
| 141 | 0.00001 | 0 | 0.932614 | 0.067376 | 0 | 0 | 0 | 0 | 0 |
| 142 | 0.00002 | 0 | 0.931018 | 0.068962 | 0 | 0 | 0 | 0 | 0 |
| 143 | 0 | 0 | 0.934515 | 0.065485 | 0 | 0 | 0 | 0 | 0 |
| 144 | 0.00001 | 0 | 0.932158 | 0.067832 | 0 | 0 | 0 | 0 | 0 |
| 145 | 0 | 0 | 0.936109 | 0.063891 | 0 | 0 | 0 | 0 | 0 |
| 146 | 0.00001 | 0 | 0.93751 | 0.06248 | 0 | 0 | 0 | 0 | 0 |
| 147 | 0.00003 | 0 | 0.934615 | 0.065355 | 0 | 0 | 0 | 0 | 0 |
| 148 | 0.00001 | 0 | 0.93465 | 0.06534 | 0 | 0 | 0 | 0 | 0 |
| 149 | 0.00003 | 0 | 0.934938 | 0.065032 | 0 | 0 | 0 | 0 | 0 |
| 150 | 0 | 0 | 0.937839 | 0.062161 | 0 | 0 | 0 | 0 | 0 |
| 151 | 0.00002 | 0 | 0.935648 | 0.064332 | 0 | 0 | 0 | 0 | 0 |
| 152 | 0.00001 | 0 | 0.931862 | 0.068128 | 0 | 0 | 0 | 0 | 0 |
| 153 | 0 | 0 | 0.937799 | 0.062201 | 0 | 0 | 0 | 0 | 0 |
| 154 | 0.00002 | 0 | 0.935868 | 0.064112 | 0 | 0 | 0 | 0 | 0 |
| 155 | 0 | 0 | 0.935047 | 0.064953 | 0 | 0 | 0 | 0 | 0 |
| 156 | 0.00003 | 0 | 0.936184 | 0.063785 | 0 | 0 | 0 | 0 | 0 |
| 157 | 0.00001 | 0 | 0.932561 | 0.067429 | 0 | 0 | 0 | 0 | 0 |
| 158 | 0 | 0 | 0.93112 | 0.06888 | 0 | 0 | 0 | 0 | 0 |
| 159 | 0.00001 | 0 | 0.934145 | 0.065845 | 0 | 0 | 0 | 0 | 0 |
| 160 | 0.00003 | 0 | 0.936264 | 0.063706 | 0 | 0 | 0 | 0 | 0 |
| 161 | 0 | 0 | 0.931323 | 0.068677 | 0 | 0 | 0 | 0 | 0 |
| 162 | 0.00002 | 0 | 0.934445 | 0.065535 | 0 | 0 | 0 | 0 | 0 |
| 163 | 0.00001 | 0 | 0.933756 | 0.066234 | 0 | 0 | 0 | 0 | 0 |
| 164 | 0.00002 | 0 | 0.929449 | 0.070531 | 0 | 0 | 0 | 0 | 0 |
| 165 | 0.00002 | 0 | 0.939337 | 0.060643 | 0 | 0 | 0 | 0 | 0 |
| 166 | 0 | 0 | 0.934177 | 0.065823 | 0 | 0 | 0 | 0 | 0 |
| 167 | 0 | 0 | 0.935956 | 0.064044 | 0 | 0 | 0 | 0 | 0 |
| 168 | 0.00002 | 0 | 0.932611 | 0.067369 | 0 | 0 | 0 | 0 | 0 |
| 169 | 0.00002 | 0 | 0.930673 | 0.069307 | 0 | 0 | 0 | 0 | 0 |
| 170 | 0.00003 | 0 | 0.934158 | 0.065812 | 0 | 0 | 0 | 0 | 0 |
| 171 | 0.00001 | 0 | 0.936147 | 0.063843 | 0 | 0 | 0 | 0 | 0 |
| 172 | 0.00001 | 0 | 0.931916 | 0.068074 | 0 | 0 | 0 | 0 | 0 |
| 173 | 0 | 0 | 0.935476 | 0.064524 | 0 | 0 | 0 | 0 | 0 |
| 174 | 0.00002 | 0 | 0.932989 | 0.066991 | 0 | 0 | 0 | 0 | 0 |
| 175 | 0.00003 | 0 | 0.931717 | 0.068253 | 0 | 0 | 0 | 0 | 0 |
| 176 | 0.00001 | 0 | 0.937056 | 0.062934 | 0 | 0 | 0 | 0 | 0 |
| 177 | 0 | 0 | 0.937078 | 0.062922 | 0 | 0 | 0 | 0 | 0 |
| 178 | 0.00001 | 0 | 0.935029 | 0.064961 | 0 | 0 | 0 | 0 | 0 |
| 179 | 0.00002 | 0 | 0.934255 | 0.065725 | 0 | 0 | 0 | 0 | 0 |
| 180 | 0.00001 | 0 | 0.934913 | 0.065077 | 0 | 0 | 0 | 0 | 0 |
| 181 | 0.00002 | 0 | 0.934699 | 0.065281 | 0 | 0 | 0 | 0 | 0 |
| 182 | 0.00002 | 0 | 0.931537 | 0.068443 | 0 | 0 | 0 | 0 | 0 |
| 183 | 0.00001 | 0 | 0.936818 | 0.063172 | 0 | 0 | 0 | 0 | 0 |
| 184 | 0.00001 | 0 | 0.936599 | 0.063391 | 0 | 0 | 0 | 0 | 0 |
| 185 | 0 | 0 | 0.935573 | 0.064427 | 0 | 0 | 0 | 0 | 0 |
| 186 | 0 | 0 | 0.936395 | 0.063605 | 0 | 0 | 0 | 0 | 0 |
| 187 | 0 | 0 | 0.939163 | 0.060837 | 0 | 0 | 0 | 0 | 0 |
| 188 | 0 | 0 | 0.934775 | 0.065225 | 0 | 0 | 0 | 0 | 0 |
| 189 | 0 | 0 | 0.934029 | 0.065971 | 0 | 0 | 0 | 0 | 0 |
| 190 | 0.00002 | 0 | 0.935608 | 0.064372 | 0 | 0 | 0 | 0 | 0 |
| 191 | 0 | 0 | 0.935529 | 0.064471 | 0 | 0 | 0 | 0 | 0 |
| 192 | 0 | 0 | 0.936071 | 0.063929 | 0 | 0 | 0 | 0 | 0 |
| 193 | 0.00002 | 0 | 0.930021 | 0.069959 | 0 | 0 | 0 | 0 | 0 |
| 194 | 0.00002 | 0 | 0.929806 | 0.070174 | 0 | 0 | 0 | 0 | 0 |
| 195 | 0.00001 | 0 | 0.932858 | 0.067132 | 0 | 0 | 0 | 0 | 0 |
| 196 | 0.00001 | 0 | 0.931622 | 0.068367 | 0 | 0 | 0 | 0 | 0 |
| 197 | 0.00001 | 0 | 0.938212 | 0.061778 | 0 | 0 | 0 | 0 | 0 |
| 198 | 0.00001 | 0 | 0.93529 | 0.064353 | 0 | 0 | 0 | 0 | 0 |
| 199 | 0.00004 | 0 | 0.937486 | 0.062474 | 0 | 0 | 0 | 0 | 0 |
| 200 | 0 | 0 | 0.93156 | 0.06844 | 0 | 0 | 0 | 0 | 0 |
| 201 | 0.00002 | 0 | 0.934715 | 0.065265 | 0 | 0 | 0 | 0 | 0 |
| 202 | 0.00001 | 0.00003 | 0.93544 | 0.064519 | 0 | 0 | 0 | 0 | 0 |
| 203 | 0.00003 | 0 | 0.936164 | 0.063806 | 0 | 0 | 0 | 0 | 0 |
| 204 | 0 | 0 | 0.935456 | 0.064544 | 0 | 0 | 0 | 0 | 0 |
| 205 | 0.00003 | 0 | 0.930829 | 0.069141 | 0 | 0 | 0 | 0 | 0 |
| 206 | 0.00001 | 0 | 0.92928 | 0.07071 | 0 | 0 | 0 | 0 | 0 |
| 207 | 0.00001 | 0 | 0.935798 | 0.064192 | 0 | 0 | 0 | 0 | 0 |
| 208 | 0.00001 | 0 | 0.938266 | 0.061724 | 0 | 0 | 0 | 0 | 0 |
| 209 | 0 | 0 | 0.932283 | 0.067717 | 0 | 0 | 0 | 0 | 0 |
| 210 | 0.00001 | 0 | 0.935024 | 0.064966 | 0 | 0 | 0 | 0 | 0 |
| 211 | 0.00005 | 0 | 0.934619 | 0.065331 | 0 | 0 | 0 | 0 | 0 |
| 212 | 0 | 0 | 0.931025 | 0.068975 | 0 | 0 | 0 | 0 | 0 |
| 213 | 0 | 0 | 0.935537 | 0.064463 | 0 | 0 | 0 | 0 | 0 |
| 214 | 0.00002 | 0 | 0.935494 | 0.064487 | 0 | 0 | 0 | 0 | 0 |
| 215 | 0.00001 | 0 | 0.932232 | 0.067758 | 0 | 0 | 0 | 0 | 0 |
| 216 | 0 | 0 | 0.937134 | 0.062866 | 0 | 0 | 0 | 0 | 0 |
| 217 | 0.00002 | 0.00001 | 0.934333 | 0.065638 | 0 | 0 | 0 | 0 | 0 |
| 218 | 0 | 0 | 0.937925 | 0.062075 | 0 | 0 | 0 | 0 | 0 |
| 219 | 0.00004 | 0 | 0.940661 | 0.059299 | 0 | 0 | 0 | 0 | 0 |
| 220 | 0 | 0 | 0.934247 | 0.065753 | 0 | 0 | 0 | 0 | 0 |
| 221 | 0 | 0 | 0.934649 | 0.065351 | 0 | 0 | 0 | 0 | 0 |
| 222 | 0 | 0 | 0.935226 | 0.064774 | 0 | 0 | 0 | 0 | 0 |
| 223 | 0.00001 | 0 | 0.934453 | 0.065537 | 0 | 0 | 0 | 0 | 0 |
| 224 | 0 | 0 | 0.933383 | 0.066617 | 0 | 0 | 0 | 0 | 0 |
| 225 | 0.00001 | 0 | 0.935524 | 0.064466 | 0 | 0 | 0 | 0 | 0 |
| 226 | 0.00003 | 0 | 0.933405 | 0.066564 | 0 | 0 | 0 | 0 | 0 |
| 227 | 0 | 0 | 0.936748 | 0.063252 | 0 | 0 | 0 | 0 | 0 |
| 228 | 0 | 0 | 0.936254 | 0.063746 | 0 | 0 | 0 | 0 | 0 |
| 229 | 0.00001 | 0 | 0.934346 | 0.065644 | 0 | 0 | 0 | 0 | 0 |
| 230 | 0.00001 | 0 | 0.935157 | 0.064833 | 0 | 0 | 0 | 0 | 0 |
| 231 | 0.00001 | 0 | 0.938078 | 0.061912 | 0 | 0 | 0 | 0 | 0 |
| 232 | 0.00003 | 0 | 0.932458 | 0.067512 | 0 | 0 | 0 | 0 | 0 |
| 233 | 0.00003 | 0 | 0.932891 | 0.067079 | 0 | 0 | 0 | 0 | 0 |
| 234 | 0.00002 | 0 | 0.933896 | 0.066084 | 0 | 0 | 0 | 0 | 0 |
| 235 | 0.00001 | 0 | 0.940675 | 0.059315 | 0 | 0 | 0 | 0 | 0 |
| 236 | 0.00002 | 0 | 0.930864 | 0.069116 | 0 | 0 | 0 | 0 | 0 |
| 237 | 0 | 0 | 0.93386 | 0.06614 | 0 | 0 | 0 | 0 | 0 |
| 238 | 0.00002 | 0 | 0.927055 | 0.072925 | 0 | 0 | 0 | 0 | 0 |
| 239 | 0.00002 | 0 | 0.929583 | 0.070397 | 0 | 0 | 0 | 0 | 0 |
| 240 | 0.00001 | 0 | 0.93678 | 0.06321 | 0 | 0 | 0 | 0 | 0 |
| 241 | 0 | 0 | 0.937376 | 0.062624 | 0 | 0 | 0 | 0 | 0 |
| 242 | 0.00001 | 0 | 0.936123 | 0.063867 | 0 | 0 | 0 | 0 | 0 |
| 243 | 0.00001 | 0 | 0.936525 | 0.063465 | 0 | 0 | 0 | 0 | 0 |
| 244 | 0 | 0 | 0.934451 | 0.065549 | 0 | 0 | 0 | 0 | 0 |
| 245 | 0.00001 | 0 | 0.933822 | 0.066068 | 0 | 0 | 0 | 0 | 0 |
| 246 | 0.00002 | 0 | 0.931786 | 0.068194 | 0 | 0 | 0 | 0 | 0 |
| 247 | 0 | 0 | 0.933118 | 0.066882 | 0 | 0 | 0 | 0 | 0 |
| 248 | 0 | 0 | 0.935385 | 0.064615 | 0 | 0 | 0 | 0 | 0 |
| 249 | 0.00002 | 0 | 0.939467 | 0.060513 | 0 | 0 | 0 | 0 | 0 |
| 250 | 0.00004 | 0 | 0.933789 | 0.066171 | 0 | 0 | 0 | 0 | 0 |
| 251 | 0.00002 | 0.00002 | 0.934597 | 0.065363 | 0 | 0 | 0 | 0 | 0 |
| 252 | 0.00001 | 0 | 0.936291 | 0.063699 | 0 | 0 | 0 | 0 | 0 |
| 253 | 0.00002 | 0 | 0.936434 | 0.063546 | 0 | 0 | 0 | 0 | 0 |
| 254 | 0.00002 | 0 | 0.934523 | 0.065458 | 0 | 0 | 0 | 0 | 0 |
| 255 | 0.00003 | 0 | 0.935573 | 0.064397 | 0 | 0 | 0 | 0 | 0 |
| 256 | 0.00002 | 0 | 0.931555 | 0.068425 | 0 | 0 | 0 | 0 | 0 |
| 257 | 0.00001 | 0 | 0.934478 | 0.065512 | 0 | 0 | 0 | 0 | 0 |
| 258 | 0 | 0 | 0.932121 | 0.067879 | 0 | 0 | 0 | 0 | 0 |
| 259 | 0.00003 | 0 | 0.931629 | 0.068341 | 0 | 0 | 0 | 0 | 0 |
| 260 | 0.00002 | 0 | 0.937062 | 0.062918 | 0 | 0 | 0 | 0 | 0 |
| 261 | 0.00001 | 0 | 0.937093 | 0.062897 | 0 | 0 | 0 | 0 | 0 |
| 262 | 0.00001 | 0 | 0.934231 | 0.065759 | 0 | 0 | 0 | 0 | 0 |
| 263 | 0.00002 | 0 | 0.934035 | 0.065945 | 0 | 0 | 0 | 0 | 0 |
| 264 | 0 | 0 | 0.929526 | 0.070474 | 0 | 0 | 0 | 0 | 0 |
| 265 | 0 | 0 | 0.933957 | 0.066043 | 0 | 0 | 0 | 0 | 0 |
| 266 | 0.00001 | 0.00001 | 0.936779 | 0.063201 | 0 | 0 | 0 | 0 | 0 |
| 267 | 0 | 0 | 0.935295 | 0.064705 | 0 | 0 | 0 | 0 | 0 |
| 268 | 0 | 0 | 0.938187 | 0.061813 | 0 | 0 | 0 | 0 | 0 |
| 269 | 0.00001 | 0 | 0.936075 | 0.063915 | 0 | 0 | 0 | 0 | 0 |
| 270 | 0.00001 | 0 | 0.936112 | 0.063878 | 0 | 0 | 0 | 0 | 0 |
| 271 | 0.00002 | 0 | 0.935302 | 0.064678 | 0 | 0 | 0 | 0 | 0 |
| 272 | 0 | 0 | 0.938251 | 0.061749 | 0 | 0 | 0 | 0 | 0 |
| 273 | 0 | 0 | 0.936557 | 0.063443 | 0 | 0 | 0 | 0 | 0 |
| 274 | 0.00003 | 0 | 0.93894 | 0.06103 | 0 | 0 | 0 | 0 | 0 |
| 275 | 0.00002 | 0 | 0.935328 | 0.064652 | 0 | 0 | 0 | 0 | 0 |
| 276 | 0.00004 | 0 | 0.933344 | 0.066616 | 0 | 0 | 0 | 0 | 0 |
| 277 | 0.00001 | 0 | 0.929373 | 0.070617 | 0 | 0 | 0 | 0 | 0 |
| 278 | 0 | 0 | 0.936312 | 0.063688 | 0 | 0 | 0 | 0 | 0 |
| 279 | 0 | 0 | 0.935071 | 0.064929 | 0 | 0 | 0 | 0 | 0 |
| 280 | 0.00002 | 0 | 0.933402 | 0.066578 | 0 | 0 | 0 | 0 | 0 |
| 281 | 0.00002 | 0 | 0.932944 | 0.067036 | 0 | 0 | 0 | 0 | 0 |
| 282 | 0.00001 | 0 | 0.934591 | 0.065399 | 0 | 0 | 0 | 0 | 0 |
| 283 | 0.00002 | 0 | 0.936484 | 0.063496 | 0 | 0 | 0 | 0 | 0 |
| 284 | 0 | 0 | 0.93768 | 0.06232 | 0 | 0 | 0 | 0 | 0 |
| 285 | 0.00001 | 0 | 0.933552 | 0.066438 | 0 | 0 | 0 | 0 | 0 |
| 286 | 0.00001 | 0 | 0.940003 | 0.059987 | 0 | 0 | 0 | 0 | 0 |
| 287 | 0 | 0 | 0.931504 | 0.068496 | 0 | 0 | 0 | 0 | 0 |
| 288 | 0.00002 | 0 | 0.93283 | 0.06715 | 0 | 0 | 0 | 0 | 0 |
| 289 | 0 | 0 | 0.93745 | 0.06255 | 0 | 0 | 0 | 0 | 0 |
| 290 | 0.00002 | 0 | 0.93501 | 0.06497 | 0 | 0 | 0 | 0 | 0 |
| 291 | 0.00004 | 0 | 0.932741 | 0.067219 | 0 | 0 | 0 | 0 | 0 |
| 292 | 0.00001 | 0 | 0.93178 | 0.06821 | 0 | 0 | 0 | 0 | 0 |
| 293 | 0 | 0 | 0.932537 | 0.067463 | 0 | 0 | 0 | 0 | 0 |
| 294 | 0.00002 | 0 | 0.931984 | 0.067996 | 0 | 0 | 0 | 0 | 0 |
| 295 | 0.00003 | 0 | 0.937292 | 0.062678 | 0 | 0 | 0 | 0 | 0 |
| 296 | 0 | 0 | 0.93587 | 0.06413 | 0 | 0 | 0 | 0 | 0 |
| 297 | 0 | 0.00001 | 0.933344 | 0.066646 | 0 | 0 | 0 | 0 | 0 |
| 298 | 0.00001 | 0 | 0.934386 | 0.065604 | 0 | 0 | 0 | 0 | 0 |
| 299 | 0 | 0 | 0.929403 | 0.070597 | 0 | 0 | 0 | 0 | 0 |
| 300 | 0.00001 | 0 | 0.934971 | 0.065019 | 0 | 0 | 0 | 0 | 0 |
| 301 | 0.00001 | 0 | 0.933648 | 0.066342 | 0 | 0 | 0 | 0 | 0 |
| 302 | 0 | 0 | 0.932362 | 0.067638 | 0 | 0 | 0 | 0 | 0 |
| 303 | 0 | 0 | 0.935436 | 0.064564 | 0 | 0 | 0 | 0 | 0 |
| 304 | 0.00001 | 0 | 0.933116 | 0.066874 | 0 | 0 | 0 | 0 | 0 |
| 305 | 0.00001 | 0 | 0.93508 | 0.06491 | 0 | 0 | 0 | 0 | 0 |
| 306 | 0 | 0 | 0.937118 | 0.062882 | 0 | 0 | 0 | 0 | 0 |
| 307 | 0.00002 | 0 | 0.935789 | 0.064191 | 0 | 0 | 0 | 0 | 0 |
| 308 | 0.00003 | 0 | 0.938346 | 0.061624 | 0 | 0 | 0 | 0 | 0 |
| 309 | 0.00001 | 0 | 0.937848 | 0.062142 | 0 | 0 | 0 | 0 | 0 |
| 310 | 0.00002 | 0 | 0.932681 | 0.067299 | 0 | 0 | 0 | 0 | 0 |
| 311 | 0 | 0 | 0.936611 | 0.063389 | 0 | 0 | 0 | 0 | 0 |
| 312 | 0.00002 | 0 | 0.933665 | 0.066315 | 0 | 0 | 0 | 0 | 0 |
| 313 | 0 | 0 | 0.936603 | 0.063397 | 0 | 0 | 0 | 0 | 0 |
| 314 | 0 | 0 | 0.936266 | 0.063734 | 0 | 0 | 0 | 0 | 0 |
| 315 | 0.00004 | 0 | 0.932462 | 0.067498 | 0 | 0 | 0 | 0 | 0 |
| 316 | 0 | 0 | 0.932571 | 0.067429 | 0 | 0 | 0 | 0 | 0 |
| 317 | 0.00001 | 0 | 0.932135 | 0.067855 | 0 | 0 | 0 | 0 | 0 |
| 318 | 0 | 0 | 0.931839 | 0.068011 | 0 | 0 | 0 | 0 | 0 |
| 319 | 0.00005 | 0 | 0.935566 | 0.064384 | 0 | 0 | 0 | 0 | 0 |
| 320 | 0 | 0 | 0.935891 | 0.064109 | 0 | 0 | 0 | 0 | 0 |
| 321 | 0.00003 | 0 | 0.934121 | 0.065849 | 0 | 0 | 0 | 0 | 0 |
| 322 | 0 | 0 | 0.93272 | 0.06728 | 0 | 0 | 0 | 0 | 0 |
| 323 | 0 | 0 | 0.935782 | 0.064218 | 0 | 0 | 0 | 0 | 0 |
| 324 | 0 | 0 | 0.937188 | 0.062812 | 0 | 0 | 0 | 0 | 0 |
| 325 | 0 | 0 | 0.935115 | 0.064885 | 0 | 0 | 0 | 0 | 0 |
| 326 | 0 | 0.00002 | 0.935096 | 0.064884 | 0 | 0 | 0 | 0 | 0 |
| 327 | 0.00002 | 0 | 0.93387 | 0.06611 | 0 | 0 | 0 | 0 | 0 |
| 328 | 0 | 0 | 0.932796 | 0.067204 | 0 | 0 | 0 | 0 | 0 |
| 329 | 0 | 0.00002 | 0.93461 | 0.06537 | 0 | 0 | 0 | 0 | 0 |
| 330 | 0.00001 | 0 | 0.935383 | 0.064607 | 0 | 0 | 0 | 0 | 0 |
| 331 | 0.00001 | 0.00001 | 0.933383 | 0.066597 | 0 | 0 | 0 | 0 | 0 |
| 332 | 0.00002 | 0 | 0.934875 | 0.065105 | 0 | 0 | 0 | 0 | 0 |
| 333 | 0.00005 | 0 | 0.931634 | 0.068316 | 0 | 0 | 0 | 0 | 0 |
| 334 | 0.00002 | 0.00002 | 0.935875 | 0.064085 | 0 | 0 | 0 | 0 | 0 |
| 335 | 0.00001 | 0 | 0.935967 | 0.064023 | 0 | 0 | 0 | 0 | 0 |
| 336 | 0 | 0 | 0.931534 | 0.068466 | 0 | 0 | 0 | 0 | 0 |
| 337 | 0.00002 | 0 | 0.935165 | 0.064815 | 0 | 0 | 0 | 0 | 0 |
| 338 | 0 | 0 | 0.935835 | 0.064165 | 0 | 0 | 0 | 0 | 0 |
| 339 | 0.00002 | 0 | 0.93224 | 0.06774 | 0 | 0 | 0 | 0 | 0 |
| 340 | 0.00002 | 0 | 0.932056 | 0.067924 | 0 | 0 | 0 | 0 | 0 |
| 341 | 0 | 0 | 0.932513 | 0.067487 | 0 | 0 | 0 | 0 | 0 |
| 342 | 0.00003 | 0 | 0.93207 | 0.0679 | 0 | 0 | 0 | 0 | 0 |
| 343 | 0.00003 | 0 | 0.93271 | 0.06726 | 0 | 0 | 0 | 0 | 0 |
| 344 | 0.00002 | 0 | 0.93225 | 0.06773 | 0 | 0 | 0 | 0 | 0 |
| 345 | 0 | 0 | 0.936905 | 0.063095 | 0 | 0 | 0 | 0 | 0 |
| 346 | 0 | 0 | 0.940547 | 0.059453 | 0 | 0 | 0 | 0 | 0 |
| 347 | 0.00003 | 0 | 0.930486 | 0.069484 | 0 | 0 | 0 | 0 | 0 |
| 348 | 0.00002 | 0 | 0.93316 | 0.066819 | 0 | 0 | 0 | 0 | 0 |
| 349 | 0.00003 | 0 | 0.935059 | 0.064911 | 0 | 0 | 0 | 0 | 0 |
| 350 | 0 | 0 | 0.935988 | 0.064012 | 0 | 0 | 0 | 0 | 0 |
| 351 | 0.00001 | 0 | 0.936516 | 0.063474 | 0 | 0 | 0 | 0 | 0 |
| 352 | 0.00002 | 0 | 0.937697 | 0.062283 | 0 | 0 | 0 | 0 | 0 |
| 353 | 0.00002 | 0 | 0.935765 | 0.064215 | 0 | 0 | 0 | 0 | 0 |
| 354 | 0.00001 | 0 | 0.934617 | 0.065373 | 0 | 0 | 0 | 0 | 0 |
| 355 | 0 | 0 | 0.936927 | 0.063073 | 0 | 0 | 0 | 0 | 0 |
| 356 | 0.00003 | 0 | 0.933351 | 0.066618 | 0 | 0 | 0 | 0 | 0 |
| 357 | 0.00002 | 0 | 0.93125 | 0.06873 | 0 | 0 | 0 | 0 | 0 |
| 358 | 0.00002 | 0 | 0.934036 | 0.065943 | 0 | 0 | 0 | 0 | 0 |
| 359 | 0.00001 | 0 | 0.933285 | 0.066705 | 0 | 0 | 0 | 0 | 0 |
| 360 | 0 | 0 | 0.93563 | 0.06437 | 0 | 0 | 0 | 0 | 0 |
| 361 | 0.00001 | 0 | 0.932702 | 0.067288 | 0 | 0 | 0 | 0 | 0 |
| 362 | 0 | 0 | 0.938184 | 0.061816 | 0 | 0 | 0 | 0 | 0 |
| 363 | 0.00002 | 0.00002 | 0.935844 | 0.064116 | 0 | 0 | 0 | 0 | 0 |
| 364 | 0 | 0.00001 | 0.935626 | 0.064364 | 0 | 0 | 0 | 0 | 0 |
| 365 | 0 | 0 | 0.934119 | 0.065881 | 0 | 0 | 0 | 0 | 0 |
| 366 | 0.00002 | 0 | 0.935083 | 0.064897 | 0 | 0 | 0 | 0 | 0 |
| 367 | 0.00001 | 0 | 0.933702 | 0.066288 | 0 | 0 | 0 | 0 | 0 |
| 368 | 0 | 0 | 0.936488 | 0.063512 | 0 | 0 | 0 | 0 | 0 |
| 369 | 0.00002 | 0 | 0.931631 | 0.068349 | 0 | 0 | 0 | 0 | 0 |
| 370 | 0.00002 | 0 | 0.936005 | 0.063974 | 0 | 0 | 0 | 0 | 0 |
| 371 | 0.00001 | 0 | 0.935739 | 0.064012 | 0 | 0 | 0 | 0 | 0 |
| 372 | 0 | 0 | 0.929954 | 0.070046 | 0 | 0 | 0 | 0 | 0 |
| 373 | 0.00003 | 0 | 0.93062 | 0.06935 | 0 | 0 | 0 | 0 | 0 |
| 374 | 0.00001 | 0 | 0.938281 | 0.061709 | 0 | 0 | 0 | 0 | 0 |
| 375 | 0.00001 | 0 | 0.933424 | 0.066566 | 0 | 0 | 0 | 0 | 0 |
| 376 | 0.00001 | 0.00001 | 0.93069 | 0.06929 | 0 | 0 | 0 | 0 | 0 |
| 377 | 0.00002 | 0 | 0.930388 | 0.069592 | 0 | 0 | 0 | 0 | 0 |
| 378 | 0.00001 | 0 | 0.937544 | 0.062446 | 0 | 0 | 0 | 0 | 0 |
| 379 | 0.00001 | 0 | 0.934941 | 0.065049 | 0 | 0 | 0 | 0 | 0 |
| 380 | 0 | 0 | 0.92953 | 0.07047 | 0 | 0 | 0 | 0 | 0 |
| 381 | 0.00002 | 0.00001 | 0.934084 | 0.065886 | 0 | 0 | 0 | 0 | 0 |
| 382 | 0 | 0 | 0.933357 | 0.066643 | 0 | 0 | 0 | 0 | 0 |
| 383 | 0.00002 | 0 | 0.932712 | 0.067268 | 0 | 0 | 0 | 0 | 0 |
| 384 | 0 | 0 | 0.928285 | 0.071715 | 0 | 0 | 0 | 0 | 0 |
| 385 | 0.00002 | 0 | 0.934852 | 0.065128 | 0 | 0 | 0 | 0 | 0 |
| 386 | 0.00001 | 0 | 0.935199 | 0.064791 | 0 | 0 | 0 | 0 | 0 |
| 387 | 0.00002 | 0 | 0.935601 | 0.064379 | 0 | 0 | 0 | 0 | 0 |
| 388 | 0.00002 | 0 | 0.935246 | 0.064734 | 0 | 0 | 0 | 0 | 0 |
| 389 | 0.00001 | 0.00001 | 0.936917 | 0.063064 | 0 | 0 | 0 | 0 | 0 |
| 390 | 0.00001 | 0.00001 | 0.93653 | 0.06345 | 0 | 0 | 0 | 0 | 0 |
| 391 | 0 | 0 | 0.932731 | 0.067269 | 0 | 0 | 0 | 0 | 0 |
| 392 | 0.00001 | 0 | 0.934798 | 0.065192 | 0 | 0 | 0 | 0 | 0 |
| 393 | 0.00002 | 0 | 0.93146 | 0.06852 | 0 | 0 | 0 | 0 | 0 |
| 394 | 0.00005 | 0 | 0.937619 | 0.062331 | 0 | 0 | 0 | 0 | 0 |
| 395 | 0.00001 | 0 | 0.935731 | 0.064259 | 0 | 0 | 0 | 0 | 0 |
| 396 | 0.00001 | 0 | 0.934197 | 0.065793 | 0 | 0 | 0 | 0 | 0 |
| 397 | 0.00001 | 0 | 0.933337 | 0.066653 | 0 | 0 | 0 | 0 | 0 |
| 398 | 0.00001 | 0 | 0.940193 | 0.059797 | 0 | 0 | 0 | 0 | 0 |
| 399 | 0.00001 | 0 | 0.937195 | 0.062795 | 0 | 0 | 0 | 0 | 0 |
| 400 | 0.00001 | 0.00001 | 0.937225 | 0.062755 | 0 | 0 | 0 | 0 | 0 |
| 401 | 0.00001 | 0 | 0.935017 | 0.064973 | 0 | 0 | 0 | 0 | 0 |
| 402 | 0.00001 | 0 | 0.936075 | 0.063915 | 0 | 0 | 0 | 0 | 0 |
| 403 | 0 | 0 | 0.934507 | 0.065493 | 0 | 0 | 0 | 0 | 0 |
| 404 | 0.00002 | 0 | 0.933822 | 0.066158 | 0 | 0 | 0 | 0 | 0 |
| 405 | 0 | 0 | 0.936993 | 0.063007 | 0 | 0 | 0 | 0 | 0 |
| 406 | 0.00002 | 0 | 0.935259 | 0.064721 | 0 | 0 | 0 | 0 | 0 |
| 407 | 0 | 0 | 0.93116 | 0.06884 | 0 | 0 | 0 | 0 | 0 |
| 408 | 0.00001 | 0.00002 | 0.932862 | 0.067108 | 0 | 0 | 0 | 0 | 0 |
| 409 | 0 | 0 | 0.930308 | 0.069692 | 0 | 0 | 0 | 0 | 0 |
| 410 | 0.00001 | 0 | 0.929888 | 0.070102 | 0 | 0 | 0 | 0 | 0 |
| 411 | 0.00001 | 0 | 0.93507 | 0.06492 | 0 | 0 | 0 | 0 | 0 |
| 412 | 0 | 0 | 0.934294 | 0.065706 | 0 | 0 | 0 | 0 | 0 |
| 413 | 0 | 0 | 0.931322 | 0.068678 | 0 | 0 | 0 | 0 | 0 |
| 414 | 0 | 0 | 0.936566 | 0.063434 | 0 | 0 | 0 | 0 | 0 |
| 415 | 0.00003 | 0 | 0.932854 | 0.067116 | 0 | 0 | 0 | 0 | 0 |
| 416 | 0.00001 | 0 | 0.934331 | 0.065659 | 0 | 0 | 0 | 0 | 0 |
| 417 | 0 | 0 | 0.937627 | 0.062373 | 0 | 0 | 0 | 0 | 0 |
| 418 | 0 | 0 | 0.935081 | 0.064919 | 0 | 0 | 0 | 0 | 0 |
| 419 | 0.00003 | 0 | 0.932294 | 0.067676 | 0 | 0 | 0 | 0 | 0 |
| 420 | 0 | 0 | 0.941541 | 0.058459 | 0 | 0 | 0 | 0 | 0 |
| 421 | 0.00002 | 0 | 0.93215 | 0.06783 | 0 | 0 | 0 | 0 | 0 |
| 422 | 0 | 0.00004 | 0.932946 | 0.067014 | 0 | 0 | 0 | 0 | 0 |
| 423 | 0.00003 | 0 | 0.937717 | 0.062253 | 0 | 0 | 0 | 0 | 0 |
| 424 | 0.00001 | 0.00001 | 0.937909 | 0.06207 | 0 | 0 | 0 | 0 | 0 |
| 425 | 0.00002 | 0 | 0.932954 | 0.067026 | 0 | 0 | 0 | 0 | 0 |
| 426 | 0 | 0 | 0.933517 | 0.066483 | 0 | 0 | 0 | 0 | 0 |
| 427 | 0.00002 | 0 | 0.93558 | 0.0644 | 0 | 0 | 0 | 0 | 0 |
| 428 | 0.00001 | 0 | 0.936654 | 0.063336 | 0 | 0 | 0 | 0 | 0 |
| 429 | 0.00002 | 0 | 0.937198 | 0.062782 | 0 | 0 | 0 | 0 | 0 |
| 430 | 0.00003 | 0 | 0.933318 | 0.066652 | 0 | 0 | 0 | 0 | 0 |
| 431 | 0.00003 | 0 | 0.937913 | 0.062057 | 0 | 0 | 0 | 0 | 0 |
| 432 | 0 | 0.00002 | 0.932398 | 0.067582 | 0 | 0 | 0 | 0 | 0 |
| 433 | 0.00001 | 0 | 0.93659 | 0.0634 | 0 | 0 | 0 | 0 | 0 |
| 434 | 0 | 0.00002 | 0.937205 | 0.062775 | 0 | 0 | 0 | 0 | 0 |
| 435 | 0 | 0 | 0.934631 | 0.065369 | 0 | 0 | 0 | 0 | 0 |
| 436 | 0.00003 | 0 | 0.927615 | 0.072355 | 0 | 0 | 0 | 0 | 0 |
| 437 | 0.00001 | 0 | 0.933727 | 0.066263 | 0 | 0 | 0 | 0 | 0 |
| 438 | 0.00002 | 0 | 0.936378 | 0.063602 | 0 | 0 | 0 | 0 | 0 |
| 439 | 0.00001 | 0 | 0.936762 | 0.063228 | 0 | 0 | 0 | 0 | 0 |
| 440 | 0.00002 | 0 | 0.93498 | 0.065 | 0 | 0 | 0 | 0 | 0 |
| 441 | 0.00001 | 0.00001 | 0.932827 | 0.067153 | 0 | 0 | 0 | 0 | 0 |
| 442 | 0.00001 | 0 | 0.933914 | 0.066076 | 0 | 0 | 0 | 0 | 0 |
| 443 | 0.00002 | 0 | 0.938416 | 0.061564 | 0 | 0 | 0 | 0 | 0 |
| 444 | 0 | 0.00001 | 0.933587 | 0.066403 | 0 | 0 | 0 | 0 | 0 |
| 445 | 0 | 0 | 0.933861 | 0.066139 | 0 | 0 | 0 | 0 | 0 |
| 446 | 0.00002 | 0 | 0.932074 | 0.067906 | 0 | 0 | 0 | 0 | 0 |
| 447 | 0.00002 | 0 | 0.937213 | 0.062767 | 0 | 0 | 0 | 0 | 0 |
| 448 | 0.00002 | 0 | 0.93626 | 0.06372 | 0 | 0 | 0 | 0 | 0 |
| 449 | 0 | 0 | 0.934918 | 0.065082 | 0 | 0 | 0 | 0 | 0 |
| 450 | 0.00002 | 0 | 0.934573 | 0.065407 | 0 | 0 | 0 | 0 | 0 |
| 451 | 0.00001 | 0 | 0.937068 | 0.062922 | 0 | 0 | 0 | 0 | 0 |
| 452 | 0.00003 | 0 | 0.936345 | 0.063625 | 0 | 0 | 0 | 0 | 0 |
| 453 | 0.00001 | 0 | 0.933886 | 0.066104 | 0 | 0 | 0 | 0 | 0 |
| 454 | 0 | 0 | 0.932319 | 0.067681 | 0 | 0 | 0 | 0 | 0 |
| 455 | 0 | 0 | 0.937285 | 0.062715 | 0 | 0 | 0 | 0 | 0 |
| 456 | 0.00001 | 0 | 0.934103 | 0.065887 | 0 | 0 | 0 | 0 | 0 |
| 457 | 0 | 0.00001 | 0.93222 | 0.06777 | 0 | 0 | 0 | 0 | 0 |
| 458 | 0 | 0 | 0.936166 | 0.063834 | 0 | 0 | 0 | 0 | 0 |
| 459 | 0.00002 | 0 | 0.927516 | 0.072464 | 0 | 0 | 0 | 0 | 0 |
| 460 | 0 | 0 | 0.929116 | 0.070884 | 0 | 0 | 0 | 0 | 0 |
| 461 | 0.00002 | 0 | 0.938095 | 0.061884 | 0 | 0 | 0 | 0 | 0 |
| 462 | 0 | 0 | 0.93929 | 0.06071 | 0 | 0 | 0 | 0 | 0 |
| 463 | 0.00001 | 0 | 0.930875 | 0.069115 | 0 | 0 | 0 | 0 | 0 |
| 464 | 0.00002 | 0 | 0.931262 | 0.068168 | 0 | 0 | 0 | 0 | 0 |
| 465 | 0 | 0 | 0.936446 | 0.063554 | 0 | 0 | 0 | 0 | 0 |
| 466 | 0 | 0 | 0.933227 | 0.066773 | 0 | 0 | 0 | 0 | 0 |
| 467 | 0.00003 | 0 | 0.930916 | 0.069054 | 0 | 0 | 0 | 0 | 0 |
| 468 | 0.00003 | 0 | 0.937086 | 0.062884 | 0 | 0 | 0 | 0 | 0 |
| 469 | 0.00001 | 0 | 0.93322 | 0.06677 | 0 | 0 | 0 | 0 | 0 |
| 470 | 0 | 0 | 0.935957 | 0.064043 | 0 | 0 | 0 | 0 | 0 |
| 471 | 0 | 0 | 0.933339 | 0.066661 | 0 | 0 | 0 | 0 | 0 |
| 472 | 0 | 0 | 0.932348 | 0.067652 | 0 | 0 | 0 | 0 | 0 |
| 473 | 0.00001 | 0 | 0.936904 | 0.063086 | 0 | 0 | 0 | 0 | 0 |
| 474 | 0.00001 | 0 | 0.93664 | 0.06335 | 0 | 0 | 0 | 0 | 0 |
| 475 | 0.00002 | 0 | 0.937217 | 0.062763 | 0 | 0 | 0 | 0 | 0 |
| 476 | 0 | 0.00002 | 0.926763 | 0.073217 | 0 | 0 | 0 | 0 | 0 |
| 477 | 0.00003 | 0 | 0.936255 | 0.063715 | 0 | 0 | 0 | 0 | 0 |
| 478 | 0 | 0 | 0.932855 | 0.067145 | 0 | 0 | 0 | 0 | 0 |
| 479 | 0.00001 | 0 | 0.932946 | 0.067044 | 0 | 0 | 0 | 0 | 0 |
| 480 | 0.00002 | 0 | 0.930854 | 0.069126 | 0 | 0 | 0 | 0 | 0 |
| 481 | 0.00004 | 0 | 0.938444 | 0.061516 | 0 | 0 | 0 | 0 | 0 |
| 482 | 0.00001 | 0 | 0.930681 | 0.069309 | 0 | 0 | 0 | 0 | 0 |
| 483 | 0.00002 | 0 | 0.935883 | 0.064097 | 0 | 0 | 0 | 0 | 0 |
| 484 | 0.00001 | 0 | 0.933471 | 0.066519 | 0 | 0 | 0 | 0 | 0 |
| 485 | 0 | 0 | 0.937306 | 0.062694 | 0 | 0 | 0 | 0 | 0 |
| 486 | 0.00001 | 0 | 0.932751 | 0.067239 | 0 | 0 | 0 | 0 | 0 |
| 487 | 0.00001 | 0 | 0.932239 | 0.067751 | 0 | 0 | 0 | 0 | 0 |
| 488 | 0.00004 | 0 | 0.931466 | 0.068494 | 0 | 0 | 0 | 0 | 0 |
| 489 | 0.00002 | 0 | 0.934103 | 0.065877 | 0 | 0 | 0 | 0 | 0 |
| 490 | 0.00001 | 0 | 0.937393 | 0.062597 | 0 | 0 | 0 | 0 | 0 |
| 491 | 0.00001 | 0 | 0.934397 | 0.065593 | 0 | 0 | 0 | 0 | 0 |
| 492 | 0.00001 | 0 | 0.93376 | 0.06623 | 0 | 0 | 0 | 0 | 0 |
| 493 | 0.00001 | 0 | 0.933741 | 0.066249 | 0 | 0 | 0 | 0 | 0 |
| 494 | 0 | 0 | 0.934772 | 0.065228 | 0 | 0 | 0 | 0 | 0 |
| 495 | 0.00001 | 0 | 0.933454 | 0.066536 | 0 | 0 | 0 | 0 | 0 |
| 496 | 0 | 0 | 0.933069 | 0.066862 | 0 | 0 | 0 | 0 | 0 |
| 497 | 0.00001 | 0 | 0.934336 | 0.065654 | 0 | 0 | 0 | 0 | 0 |
| 498 | 0.00001 | 0 | 0.938679 | 0.061311 | 0 | 0 | 0 | 0 | 0 |
| 499 | 0.00001 | 0 | 0.932126 | 0.067864 | 0 | 0 | 0 | 0 | 0 |
| 500 | 0.00002 | 0 | 0.935189 | 0.064792 | 0 | 0 | 0 | 0 | 0 |
| 501 | 0.00003 | 0 | 0.933167 | 0.066803 | 0 | 0 | 0 | 0 | 0 |
| 502 | 0.00002 | 0 | 0.935518 | 0.064462 | 0 | 0 | 0 | 0 | 0 |
| 503 | 0 | 0 | 0.929647 | 0.070353 | 0 | 0 | 0 | 0 | 0 |
| 504 | 0.00001 | 0 | 0.934919 | 0.065071 | 0 | 0 | 0 | 0 | 0 |
| 505 | 0.00001 | 0 | 0.932385 | 0.067575 | 0 | 0 | 0 | 0 | 0 |
| 506 | 0.00001 | 0 | 0.931661 | 0.068329 | 0 | 0 | 0 | 0 | 0 |
| 507 | 0.00003 | 0 | 0.934968 | 0.065003 | 0 | 0 | 0 | 0 | 0 |
| 508 | 0.00002 | 0 | 0.932226 | 0.067753 | 0 | 0 | 0 | 0 | 0 |
| 509 | 0.00001 | 0 | 0.929448 | 0.070542 | 0 | 0 | 0 | 0 | 0 |
| 510 | 0.00001 | 0 | 0.935291 | 0.064699 | 0 | 0 | 0 | 0 | 0 |
| 511 | 0 | 0 | 0.934413 | 0.065587 | 0 | 0 | 0 | 0 | 0 |
| 512 | 0 | 0 | 0.934641 | 0.065359 | 0 | 0 | 0 | 0 | 0 |
| 513 | 0.00002 | 0 | 0.933109 | 0.066871 | 0 | 0 | 0 | 0 | 0 |
| 514 | 0.00001 | 0 | 0.934411 | 0.065579 | 0 | 0 | 0 | 0 | 0 |
| 515 | 0 | 0 | 0.936276 | 0.063724 | 0 | 0 | 0 | 0 | 0 |
| 516 | 0.00002 | 0 | 0.929866 | 0.070114 | 0 | 0 | 0 | 0 | 0 |
| 517 | 0.00002 | 0 | 0.933101 | 0.066879 | 0 | 0 | 0 | 0 | 0 |
| 518 | 0.00001 | 0 | 0.934233 | 0.065757 | 0 | 0 | 0 | 0 | 0 |
| 519 | 0.000041 | 0 | 0.931882 | 0.068078 | 0 | 0 | 0 | 0 | 0 |
| 520 | 0.00001 | 0 | 0.934611 | 0.065379 | 0 | 0 | 0 | 0 | 0 |
| 521 | 0.00003 | 0 | 0.932785 | 0.067185 | 0 | 0 | 0 | 0 | 0 |
| 522 | 0.00002 | 0 | 0.937126 | 0.062854 | 0 | 0 | 0 | 0 | 0 |
| 523 | 0.00001 | 0 | 0.932762 | 0.067228 | 0 | 0 | 0 | 0 | 0 |
| 524 | 0.00001 | 0 | 0.933425 | 0.066565 | 0 | 0 | 0 | 0 | 0 |
| 525 | 0.00002 | 0 | 0.932471 | 0.067509 | 0 | 0 | 0 | 0 | 0 |
| 526 | 0 | 0.00002 | 0.933037 | 0.066943 | 0 | 0 | 0 | 0 | 0 |
| 527 | 0.00001 | 0 | 0.933325 | 0.066665 | 0 | 0 | 0 | 0 | 0 |
| 528 | 0 | 0 | 0.933156 | 0.066844 | 0 | 0 | 0 | 0 | 0 |
| 529 | 0.00001 | 0 | 0.934706 | 0.065284 | 0 | 0 | 0 | 0 | 0 |
| 530 | 0.00002 | 0 | 0.932085 | 0.067895 | 0 | 0 | 0 | 0 | 0 |
| 531 | 0.00002 | 0 | 0.934087 | 0.065894 | 0 | 0 | 0 | 0 | 0 |
| 532 | 0.00005 | 0 | 0.939458 | 0.060492 | 0 | 0 | 0 | 0 | 0 |
| 533 | 0 | 0 | 0.933419 | 0.066581 | 0 | 0 | 0 | 0 | 0 |
| 534 | 0.00001 | 0 | 0.931854 | 0.068137 | 0 | 0 | 0 | 0 | 0 |
| 535 | 0.00001 | 0 | 0.937331 | 0.062659 | 0 | 0 | 0 | 0 | 0 |
| 536 | 0.00001 | 0 | 0.937729 | 0.062261 | 0 | 0 | 0 | 0 | 0 |
| 537 | 0.00003 | 0 | 0.933896 | 0.066074 | 0 | 0 | 0 | 0 | 0 |
| 538 | 0.00001 | 0 | 0.93241 | 0.06758 | 0 | 0 | 0 | 0 | 0 |
| 539 | 0.00001 | 0 | 0.929277 | 0.070713 | 0 | 0 | 0 | 0 | 0 |
| 540 | 0 | 0 | 0.932004 | 0.067996 | 0 | 0 | 0 | 0 | 0 |
| 541 | 0 | 0.00001 | 0.936514 | 0.063476 | 0 | 0 | 0 | 0 | 0 |
| 542 | 0 | 0 | 0.934422 | 0.065578 | 0 | 0 | 0 | 0 | 0 |
| 543 | 0 | 0 | 0.93641 | 0.06359 | 0 | 0 | 0 | 0 | 0 |
| 544 | 0.00001 | 0 | 0.938099 | 0.061891 | 0 | 0 | 0 | 0 | 0 |
| 545 | 0 | 0 | 0.930362 | 0.069638 | 0 | 0 | 0 | 0 | 0 |
| 546 | 0.00002 | 0 | 0.939041 | 0.060939 | 0 | 0 | 0 | 0 | 0 |
| 547 | 0.00001 | 0 | 0.933567 | 0.066423 | 0 | 0 | 0 | 0 | 0 |
| 548 | 0 | 0 | 0.936716 | 0.063284 | 0 | 0 | 0 | 0 | 0 |
| 549 | 0.00001 | 0 | 0.930588 | 0.069402 | 0 | 0 | 0 | 0 | 0 |
| 550 | 0.00001 | 0 | 0.932671 | 0.067319 | 0 | 0 | 0 | 0 | 0 |
| 551 | 0.00002 | 0 | 0.935205 | 0.064775 | 0 | 0 | 0 | 0 | 0 |
| 552 | 0.00001 | 0.00001 | 0.933721 | 0.066259 | 0 | 0 | 0 | 0 | 0 |
| 553 | 0.00002 | 0 | 0.93313 | 0.06685 | 0 | 0 | 0 | 0 | 0 |
| 554 | 0.00002 | 0 | 0.939327 | 0.060653 | 0 | 0 | 0 | 0 | 0 |
| 555 | 0 | 0 | 0.930498 | 0.069502 | 0 | 0 | 0 | 0 | 0 |
| 556 | 0.00001 | 0 | 0.936368 | 0.063622 | 0 | 0 | 0 | 0 | 0 |
| 557 | 0 | 0 | 0.933327 | 0.066673 | 0 | 0 | 0 | 0 | 0 |
| 558 | 0 | 0 | 0.934878 | 0.065122 | 0 | 0 | 0 | 0 | 0 |
| 559 | 0 | 0 | 0.937321 | 0.062679 | 0 | 0 | 0 | 0 | 0 |
| 560 | 0.00001 | 0 | 0.937049 | 0.062941 | 0 | 0 | 0 | 0 | 0 |
| 561 | 0.00002 | 0 | 0.932603 | 0.067377 | 0 | 0 | 0 | 0 | 0 |
| 562 | 0 | 0 | 0.932395 | 0.067605 | 0 | 0 | 0 | 0 | 0 |
| 563 | 0.00003 | 0 | 0.932156 | 0.067815 | 0 | 0 | 0 | 0 | 0 |
| 564 | 0 | 0 | 0.934219 | 0.065781 | 0 | 0 | 0 | 0 | 0 |
| 565 | 0.00001 | 0 | 0.931756 | 0.068234 | 0 | 0 | 0 | 0 | 0 |
| 566 | 0.00001 | 0 | 0.937034 | 0.062956 | 0 | 0 | 0 | 0 | 0 |
| 567 | 0.00006 | 0 | 0.934426 | 0.065514 | 0 | 0 | 0 | 0 | 0 |
| 568 | 0.00001 | 0 | 0.931363 | 0.068627 | 0 | 0 | 0 | 0 | 0 |
| 569 | 0.00003 | 0 | 0.936951 | 0.063019 | 0 | 0 | 0 | 0 | 0 |
| 570 | 0.00001 | 0 | 0.931103 | 0.068887 | 0 | 0 | 0 | 0 | 0 |
| 571 | 0.00003 | 0 | 0.931682 | 0.068287 | 0 | 0 | 0 | 0 | 0 |
| 572 | 0.00002 | 0 | 0.937573 | 0.062407 | 0 | 0 | 0 | 0 | 0 |
| 573 | 0.00001 | 0 | 0.937884 | 0.062106 | 0 | 0 | 0 | 0 | 0 |
| 574 | 0.00002 | 0 | 0.933535 | 0.066445 | 0 | 0 | 0 | 0 | 0 |
| 575 | 0.00002 | 0 | 0.933533 | 0.066447 | 0 | 0 | 0 | 0 | 0 |
| 576 | 0.00002 | 0 | 0.939511 | 0.060469 | 0 | 0 | 0 | 0 | 0 |
| 577 | 0 | 0 | 0.932592 | 0.067408 | 0 | 0 | 0 | 0 | 0 |
| 578 | 0.00002 | 0 | 0.935947 | 0.064033 | 0 | 0 | 0 | 0 | 0 |
| 579 | 0.00003 | 0 | 0.930991 | 0.068979 | 0 | 0 | 0 | 0 | 0 |
| 580 | 0.00001 | 0 | 0.933335 | 0.066655 | 0 | 0 | 0 | 0 | 0 |
| 581 | 0 | 0 | 0.932247 | 0.067753 | 0 | 0 | 0 | 0 | 0 |
| 582 | 0.00003 | 0 | 0.933243 | 0.066727 | 0 | 0 | 0 | 0 | 0 |
| 583 | 0 | 0.00003 | 0.931058 | 0.068912 | 0 | 0 | 0 | 0 | 0 |
| 584 | 0 | 0 | 0.931093 | 0.068907 | 0 | 0 | 0 | 0 | 0 |
| 585 | 0.00001 | 0 | 0.931759 | 0.068231 | 0 | 0 | 0 | 0 | 0 |
| 586 | 0.00002 | 0 | 0.932552 | 0.067428 | 0 | 0 | 0 | 0 | 0 |
| 587 | 0.00004 | 0 | 0.934048 | 0.065912 | 0 | 0 | 0 | 0 | 0 |
| 588 | 0.00001 | 0 | 0.937984 | 0.062006 | 0 | 0 | 0 | 0 | 0 |
| 589 | 0.00001 | 0.00002 | 0.934117 | 0.065853 | 0 | 0 | 0 | 0 | 0 |
| 590 | 0 | 0 | 0.929739 | 0.070261 | 0 | 0 | 0 | 0 | 0 |
| 591 | 0 | 0 | 0.92832 | 0.07168 | 0 | 0 | 0 | 0 | 0 |
| 592 | 0.00001 | 0 | 0.932349 | 0.067641 | 0 | 0 | 0 | 0 | 0 |
| 593 | 0 | 0 | 0.93511 | 0.06489 | 0 | 0 | 0 | 0 | 0 |
| 594 | 0.00001 | 0 | 0.931035 | 0.068955 | 0 | 0 | 0 | 0 | 0 |
| 595 | 0 | 0 | 0.933514 | 0.066486 | 0 | 0 | 0 | 0 | 0 |
| 596 | 0.00002 | 0 | 0.93473 | 0.06525 | 0 | 0 | 0 | 0 | 0 |
| 597 | 0 | 0 | 0.93599 | 0.06401 | 0 | 0 | 0 | 0 | 0 |
| 598 | 0.00002 | 0 | 0.93602 | 0.06396 | 0 | 0 | 0 | 0 | 0 |
| 599 | 0.00001 | 0 | 0.935035 | 0.064955 | 0 | 0 | 0 | 0 | 0 |
| 600 | 0.00001 | 0 | 0.93153 | 0.06846 | 0 | 0 | 0 | 0 | 0 |
| 601 | 0 | 0 | 0.933156 | 0.066844 | 0 | 0 | 0 | 0 | 0 |
| 602 | 0 | 0 | 0.936927 | 0.063073 | 0 | 0 | 0 | 0 | 0 |
| 603 | 0 | 0 | 0.933725 | 0.066275 | 0 | 0 | 0 | 0 | 0 |
| 604 | 0.00001 | 0 | 0.934737 | 0.065253 | 0 | 0 | 0 | 0 | 0 |
| 605 | 0.00001 | 0 | 0.935358 | 0.064632 | 0 | 0 | 0 | 0 | 0 |
| 606 | 0.00002 | 0 | 0.936886 | 0.063094 | 0 | 0 | 0 | 0 | 0 |
| 607 | 0 | 0 | 0.939382 | 0.060618 | 0 | 0 | 0 | 0 | 0 |
| 608 | 0.00004 | 0 | 0.936103 | 0.063857 | 0 | 0 | 0 | 0 | 0 |
| 609 | 0.00002 | 0 | 0.935551 | 0.064429 | 0 | 0 | 0 | 0 | 0 |
| 610 | 0 | 0 | 0.932044 | 0.067956 | 0 | 0 | 0 | 0 | 0 |
| 611 | 0.00001 | 0 | 0.933413 | 0.066577 | 0 | 0 | 0 | 0 | 0 |
| 612 | 0 | 0 | 0.934377 | 0.065623 | 0 | 0 | 0 | 0 | 0 |
| 613 | 0.00001 | 0 | 0.930061 | 0.069929 | 0 | 0 | 0 | 0 | 0 |
| 614 | 0.00002 | 0 | 0.93584 | 0.064141 | 0 | 0 | 0 | 0 | 0 |
| 615 | 0 | 0.00001 | 0.937684 | 0.062307 | 0 | 0 | 0 | 0 | 0 |
| 616 | 0 | 0 | 0.93535 | 0.06465 | 0 | 0 | 0 | 0 | 0 |
| 617 | 0.00001 | 0 | 0.93515 | 0.06484 | 0 | 0 | 0 | 0 | 0 |
| 618 | 0.00002 | 0 | 0.931238 | 0.068742 | 0 | 0 | 0 | 0 | 0 |
| 619 | 0.00001 | 0 | 0.937987 | 0.062003 | 0 | 0 | 0 | 0 | 0 |
| 620 | 0.00001 | 0.00001 | 0.933882 | 0.066098 | 0 | 0 | 0 | 0 | 0 |
| 621 | 0.00003 | 0 | 0.934359 | 0.065611 | 0 | 0 | 0 | 0 | 0 |
| 622 | 0.00001 | 0 | 0.92824 | 0.07175 | 0 | 0 | 0 | 0 | 0 |
| 623 | 0.00001 | 0 | 0.933964 | 0.066026 | 0 | 0 | 0 | 0 | 0 |
| 624 | 0.00001 | 0 | 0.932727 | 0.067263 | 0 | 0 | 0 | 0 | 0 |
| 625 | 0.00002 | 0 | 0.93586 | 0.06405 | 0 | 0 | 0 | 0 | 0 |
| 626 | 0.00001 | 0 | 0.93016 | 0.06983 | 0 | 0 | 0 | 0 | 0 |
| 627 | 0.00002 | 0 | 0.933966 | 0.066014 | 0 | 0 | 0 | 0 | 0 |
| 628 | 0.00002 | 0.00002 | 0.93365 | 0.06631 | 0 | 0 | 0 | 0 | 0 |
| 629 | 0.00001 | 0 | 0.932001 | 0.067989 | 0 | 0 | 0 | 0 | 0 |
| 630 | 0 | 0 | 0.932663 | 0.067337 | 0 | 0 | 0 | 0 | 0 |
| 631 | 0.00001 | 0 | 0.932828 | 0.067162 | 0 | 0 | 0 | 0 | 0 |
| 632 | 0.00001 | 0 | 0.934548 | 0.065442 | 0 | 0 | 0 | 0 | 0 |
| 633 | 0.00003 | 0 | 0.935096 | 0.064874 | 0 | 0 | 0 | 0 | 0 |
| 634 | 0 | 0 | 0.933184 | 0.066816 | 0 | 0 | 0 | 0 | 0 |
| 635 | 0 | 0 | 0.940132 | 0.059868 | 0 | 0 | 0 | 0 | 0 |
| 636 | 0 | 0 | 0.932735 | 0.067265 | 0 | 0 | 0 | 0 | 0 |
| 637 | 0 | 0 | 0.93195 | 0.06805 | 0 | 0 | 0 | 0 | 0 |
| 638 | 0.00003 | 0 | 0.934081 | 0.065889 | 0 | 0 | 0 | 0 | 0 |
| 639 | 0 | 0 | 0.931254 | 0.068746 | 0 | 0 | 0 | 0 | 0 |
| 640 | 0.00001 | 0 | 0.930228 | 0.069762 | 0 | 0 | 0 | 0 | 0 |
| 641 | 0.00001 | 0 | 0.935213 | 0.064777 | 0 | 0 | 0 | 0 | 0 |
| 642 | 0.00002 | 0 | 0.937129 | 0.062851 | 0 | 0 | 0 | 0 | 0 |
| 643 | 0.00001 | 0 | 0.933068 | 0.066922 | 0 | 0 | 0 | 0 | 0 |
| 644 | 0.00003 | 0 | 0.932515 | 0.067454 | 0 | 0 | 0 | 0 | 0 |
| 645 | 0.00001 | 0.00001 | 0.936052 | 0.063928 | 0 | 0 | 0 | 0 | 0 |
| 646 | 0 | 0 | 0.931625 | 0.068375 | 0 | 0 | 0 | 0 | 0 |
| 647 | 0.00001 | 0 | 0.931923 | 0.068066 | 0 | 0 | 0 | 0 | 0 |
| 648 | 0.00001 | 0 | 0.936515 | 0.063475 | 0 | 0 | 0 | 0 | 0 |
| 649 | 0.00002 | 0 | 0.933655 | 0.066325 | 0 | 0 | 0 | 0 | 0 |
| 650 | 0.00001 | 0 | 0.933136 | 0.066854 | 0 | 0 | 0 | 0 | 0 |
| 651 | 0 | 0 | 0.934948 | 0.065052 | 0 | 0 | 0 | 0 | 0 |
| 652 | 0.00001 | 0 | 0.932318 | 0.067672 | 0 | 0 | 0 | 0 | 0 |
| 653 | 0.00002 | 0 | 0.937409 | 0.062571 | 0 | 0 | 0 | 0 | 0 |
| 654 | 0 | 0 | 0.934018 | 0.065982 | 0 | 0 | 0 | 0 | 0 |
| 655 | 0 | 0 | 0.932155 | 0.067845 | 0 | 0 | 0 | 0 | 0 |
| 656 | 0.00001 | 0 | 0.939568 | 0.060422 | 0 | 0 | 0 | 0 | 0 |
| 657 | 0.00002 | 0 | 0.933491 | 0.066489 | 0 | 0 | 0 | 0 | 0 |
| 658 | 0.00002 | 0 | 0.935758 | 0.064222 | 0 | 0 | 0 | 0 | 0 |
| 659 | 0.00001 | 0 | 0.930936 | 0.069054 | 0 | 0 | 0 | 0 | 0 |
| 660 | 0.00002 | 0 | 0.937965 | 0.062015 | 0 | 0 | 0 | 0 | 0 |
| 661 | 0.00002 | 0 | 0.934471 | 0.065508 | 0 | 0 | 0 | 0 | 0 |
| 662 | 0 | 0 | 0.934234 | 0.065766 | 0 | 0 | 0 | 0 | 0 |
| 663 | 0.00004 | 0 | 0.934553 | 0.065407 | 0 | 0 | 0 | 0 | 0 |
| 664 | 0.00002 | 0 | 0.938091 | 0.061889 | 0 | 0 | 0 | 0 | 0 |
| 665 | 0.00001 | 0 | 0.931977 | 0.068013 | 0 | 0 | 0 | 0 | 0 |
| 666 | 0.00002 | 0 | 0.935382 | 0.064598 | 0 | 0 | 0 | 0 | 0 |
| 667 | 0.00001 | 0 | 0.930924 | 0.069066 | 0 | 0 | 0 | 0 | 0 |
| 668 | 0 | 0 | 0.933765 | 0.066235 | 0 | 0 | 0 | 0 | 0 |
| 669 | 0 | 0.00001 | 0.930753 | 0.069237 | 0 | 0 | 0 | 0 | 0 |
| 670 | 0.00001 | 0 | 0.935769 | 0.064221 | 0 | 0 | 0 | 0 | 0 |
| 671 | 0.00001 | 0 | 0.935111 | 0.064879 | 0 | 0 | 0 | 0 | 0 |
| 672 | 0.00002 | 0 | 0.934237 | 0.065743 | 0 | 0 | 0 | 0 | 0 |
| 673 | 0.00001 | 0 | 0.930322 | 0.069668 | 0 | 0 | 0 | 0 | 0 |
| 674 | 0.00001 | 0 | 0.933814 | 0.066176 | 0 | 0 | 0 | 0 | 0 |
| 675 | 0.00002 | 0 | 0.935791 | 0.064189 | 0 | 0 | 0 | 0 | 0 |
| 676 | 0 | 0 | 0.93604 | 0.06396 | 0 | 0 | 0 | 0 | 0 |
| 677 | 0.00002 | 0 | 0.935078 | 0.064902 | 0 | 0 | 0 | 0 | 0 |
| 678 | 0 | 0 | 0.935971 | 0.064029 | 0 | 0 | 0 | 0 | 0 |
| 679 | 0.00001 | 0 | 0.937517 | 0.062473 | 0 | 0 | 0 | 0 | 0 |
| 680 | 0 | 0 | 0.932179 | 0.067821 | 0 | 0 | 0 | 0 | 0 |
| 681 | 0.00002 | 0 | 0.93347 | 0.06651 | 0 | 0 | 0 | 0 | 0 |
| 682 | 0.00005 | 0 | 0.933577 | 0.066373 | 0 | 0 | 0 | 0 | 0 |
| 683 | 0 | 0 | 0.934643 | 0.065357 | 0 | 0 | 0 | 0 | 0 |
| 684 | 0.00001 | 0 | 0.936685 | 0.063305 | 0 | 0 | 0 | 0 | 0 |
| 685 | 0 | 0 | 0.935274 | 0.064726 | 0 | 0 | 0 | 0 | 0 |
| 686 | 0.00001 | 0 | 0.930716 | 0.069274 | 0 | 0 | 0 | 0 | 0 |
| 687 | 0 | 0 | 0.936542 | 0.063458 | 0 | 0 | 0 | 0 | 0 |
| 688 | 0.00003 | 0 | 0.936283 | 0.063687 | 0 | 0 | 0 | 0 | 0 |
| 689 | 0.00003 | 0 | 0.929653 | 0.070317 | 0 | 0 | 0 | 0 | 0 |
| 690 | 0.00001 | 0 | 0.937005 | 0.062985 | 0 | 0 | 0 | 0 | 0 |
| 691 | 0.00002 | 0 | 0.936734 | 0.063246 | 0 | 0 | 0 | 0 | 0 |
| 692 | 0 | 0 | 0.931875 | 0.068125 | 0 | 0 | 0 | 0 | 0 |
| 693 | 0.00002 | 0 | 0.931047 | 0.068933 | 0 | 0 | 0 | 0 | 0 |
| 694 | 0.00002 | 0.00001 | 0.9365 | 0.06347 | 0 | 0 | 0 | 0 | 0 |
| 695 | 0 | 0 | 0.934373 | 0.065627 | 0 | 0 | 0 | 0 | 0 |
| 696 | 0 | 0 | 0.939032 | 0.060968 | 0 | 0 | 0 | 0 | 0 |
| 697 | 0.00002 | 0 | 0.935946 | 0.064035 | 0 | 0 | 0 | 0 | 0 |
| 698 | 0 | 0 | 0.936042 | 0.063958 | 0 | 0 | 0 | 0 | 0 |
| 699 | 0.00001 | 0 | 0.933577 | 0.066413 | 0 | 0 | 0 | 0 | 0 |
| 700 | 0 | 0 | 0.931859 | 0.068141 | 0 | 0 | 0 | 0 | 0 |
| 701 | 0 | 0.00001 | 0.938873 | 0.061117 | 0 | 0 | 0 | 0 | 0 |
| 702 | 0 | 0.00001 | 0.937448 | 0.062542 | 0 | 0 | 0 | 0 | 0 |
| 703 | 0 | 0 | 0.93359 | 0.06641 | 0 | 0 | 0 | 0 | 0 |
| 704 | 0.00003 | 0 | 0.935262 | 0.064708 | 0 | 0 | 0 | 0 | 0 |
| 705 | 0.00002 | 0 | 0.93139 | 0.06859 | 0 | 0 | 0 | 0 | 0 |
| 706 | 0.00005 | 0 | 0.932014 | 0.067936 | 0 | 0 | 0 | 0 | 0 |
| 707 | 0.00001 | 0 | 0.933347 | 0.066643 | 0 | 0 | 0 | 0 | 0 |
| 708 | 0.00001 | 0 | 0.930512 | 0.069478 | 0 | 0 | 0 | 0 | 0 |
| 709 | 0.00001 | 0 | 0.938274 | 0.061716 | 0 | 0 | 0 | 0 | 0 |
| 710 | 0.00002 | 0 | 0.934607 | 0.065373 | 0 | 0 | 0 | 0 | 0 |
| 711 | 0.00002 | 0 | 0.934983 | 0.064997 | 0 | 0 | 0 | 0 | 0 |
| 712 | 0 | 0 | 0.93828 | 0.06172 | 0 | 0 | 0 | 0 | 0 |
| 713 | 0.00001 | 0 | 0.930625 | 0.069365 | 0 | 0 | 0 | 0 | 0 |
| 714 | 0.00002 | 0 | 0.931677 | 0.068303 | 0 | 0 | 0 | 0 | 0 |
| 715 | 0 | 0 | 0.931313 | 0.068687 | 0 | 0 | 0 | 0 | 0 |
| 716 | 0.00001 | 0 | 0.9331 | 0.06689 | 0 | 0 | 0 | 0 | 0 |
| 717 | 0 | 0 | 0.935044 | 0.064956 | 0 | 0 | 0 | 0 | 0 |
| 718 | 0.00003 | 0 | 0.933719 | 0.066251 | 0 | 0 | 0 | 0 | 0 |
| 719 | 0.00002 | 0 | 0.938753 | 0.061227 | 0 | 0 | 0 | 0 | 0 |
| 720 | 0.00002 | 0 | 0.933403 | 0.066577 | 0 | 0 | 0 | 0 | 0 |
| 721 | 0.00001 | 0 | 0.931796 | 0.068194 | 0 | 0 | 0 | 0 | 0 |
| 722 | 0 | 0 | 0.936449 | 0.063551 | 0 | 0 | 0 | 0 | 0 |
| 723 | 0.00002 | 0 | 0.934697 | 0.065282 | 0 | 0 | 0 | 0 | 0 |
| 724 | 0 | 0 | 0.936012 | 0.063988 | 0 | 0 | 0 | 0 | 0 |
| 725 | 0.00002 | 0 | 0.933901 | 0.066079 | 0 | 0 | 0 | 0 | 0 |
| 726 | 0 | 0 | 0.933431 | 0.066569 | 0 | 0 | 0 | 0 | 0 |
| 727 | 0 | 0 | 0.930712 | 0.069288 | 0 | 0 | 0 | 0 | 0 |
| 728 | 0.00002 | 0 | 0.932118 | 0.067862 | 0 | 0 | 0 | 0 | 0 |
| 729 | 0 | 0.00001 | 0.932366 | 0.067624 | 0 | 0 | 0 | 0 | 0 |
| 730 | 0.00001 | 0 | 0.934372 | 0.065618 | 0 | 0 | 0 | 0 | 0 |
| 731 | 0.00001 | 0 | 0.937841 | 0.062149 | 0 | 0 | 0 | 0 | 0 |
| 732 | 0.00001 | 0 | 0.934532 | 0.065458 | 0 | 0 | 0 | 0 | 0 |
| 733 | 0.00002 | 0 | 0.931845 | 0.068135 | 0 | 0 | 0 | 0 | 0 |
| 734 | 0.00003 | 0 | 0.937313 | 0.062657 | 0 | 0 | 0 | 0 | 0 |
| 735 | 0.00001 | 0 | 0.935689 | 0.064301 | 0 | 0 | 0 | 0 | 0 |
| 736 | 0 | 0 | 0.938146 | 0.061854 | 0 | 0 | 0 | 0 | 0 |
| 737 | 0.00001 | 0.00001 | 0.93484 | 0.06514 | 0 | 0 | 0 | 0 | 0 |
| 738 | 0.00001 | 0 | 0.9328 | 0.06719 | 0 | 0 | 0 | 0 | 0 |
| 739 | 0 | 0 | 0.925295 | 0.074705 | 0 | 0 | 0 | 0 | 0 |
| 740 | 0.00001 | 0 | 0.934897 | 0.065093 | 0 | 0 | 0 | 0 | 0 |
| 741 | 0 | 0 | 0.935846 | 0.064154 | 0 | 0 | 0 | 0 | 0 |
| 742 | 0.00002 | 0 | 0.935523 | 0.064457 | 0 | 0 | 0 | 0 | 0 |
| 743 | 0.00003 | 0 | 0.935301 | 0.064669 | 0 | 0 | 0 | 0 | 0 |
| 744 | 0 | 0 | 0.936279 | 0.063541 | 0 | 0 | 0 | 0 | 0 |
| 745 | 0.00001 | 0 | 0.936735 | 0.063255 | 0 | 0 | 0 | 0 | 0 |
| 746 | 0.00001 | 0 | 0.929741 | 0.070249 | 0 | 0 | 0 | 0 | 0 |
| 747 | 0 | 0 | 0.931649 | 0.068351 | 0 | 0 | 0 | 0 | 0 |
| 748 | 0.00002 | 0 | 0.935494 | 0.064486 | 0 | 0 | 0 | 0 | 0 |
| 749 | 0 | 0 | 0.939061 | 0.060939 | 0 | 0 | 0 | 0 | 0 |
| 750 | 0 | 0 | 0.935512 | 0.064488 | 0 | 0 | 0 | 0 | 0 |
| 751 | 0.00001 | 0 | 0.940261 | 0.059729 | 0 | 0 | 0 | 0 | 0 |
| 752 | 0.00002 | 0 | 0.936258 | 0.063722 | 0 | 0 | 0 | 0 | 0 |
| 753 | 0 | 0 | 0.934711 | 0.065289 | 0 | 0 | 0 | 0 | 0 |
| 754 | 0.00001 | 0 | 0.932482 | 0.067508 | 0 | 0 | 0 | 0 | 0 |
| 755 | 0 | 0 | 0.935158 | 0.064842 | 0 | 0 | 0 | 0 | 0 |
| 756 | 0.00001 | 0 | 0.93183 | 0.06816 | 0 | 0 | 0 | 0 | 0 |
| 757 | 0.00001 | 0 | 0.936476 | 0.063514 | 0 | 0 | 0 | 0 | 0 |
| 758 | 0.00002 | 0 | 0.932202 | 0.067778 | 0 | 0 | 0 | 0 | 0 |
| 759 | 0.00002 | 0.00001 | 0.937003 | 0.062967 | 0 | 0 | 0 | 0 | 0 |
| 760 | 0.00001 | 0 | 0.938102 | 0.061888 | 0 | 0 | 0 | 0 | 0 |
| 761 | 0.00001 | 0 | 0.932899 | 0.067091 | 0 | 0 | 0 | 0 | 0 |
| 762 | 0.00001 | 0 | 0.935474 | 0.064516 | 0 | 0 | 0 | 0 | 0 |
| 763 | 0.00001 | 0 | 0.937298 | 0.062692 | 0 | 0 | 0 | 0 | 0 |
| 764 | 0.00002 | 0 | 0.939191 | 0.06079 | 0 | 0 | 0 | 0 | 0 |
| 765 | 0.00002 | 0 | 0.931527 | 0.068453 | 0 | 0 | 0 | 0 | 0 |
| 766 | 0.00001 | 0 | 0.929022 | 0.070968 | 0 | 0 | 0 | 0 | 0 |
| 767 | 0.00001 | 0 | 0.937389 | 0.062601 | 0 | 0 | 0 | 0 | 0 |
| 768 | 0.00001 | 0 | 0.933729 | 0.066261 | 0 | 0 | 0 | 0 | 0 |
| 769 | 0 | 0 | 0.93341 | 0.06659 | 0 | 0 | 0 | 0 | 0 |
| 770 | 0.00001 | 0 | 0.934122 | 0.065868 | 0 | 0 | 0 | 0 | 0 |
| 771 | 0.00003 | 0 | 0.933847 | 0.065932 | 0 | 0 | 0 | 0 | 0 |
| 772 | 0 | 0 | 0.934296 | 0.065704 | 0 | 0 | 0 | 0 | 0 |
| 773 | 0.00002 | 0 | 0.928196 | 0.071784 | 0 | 0 | 0 | 0 | 0 |
| 774 | 0.00001 | 0 | 0.936058 | 0.063932 | 0 | 0 | 0 | 0 | 0 |
| 775 | 0.00001 | 0 | 0.934663 | 0.065327 | 0 | 0 | 0 | 0 | 0 |
| 776 | 0.00002 | 0 | 0.933243 | 0.066737 | 0 | 0 | 0 | 0 | 0 |
| 777 | 0.00001 | 0 | 0.934179 | 0.065811 | 0 | 0 | 0 | 0 | 0 |
| 778 | 0.00001 | 0 | 0.934566 | 0.065424 | 0 | 0 | 0 | 0 | 0 |
| 779 | 0.00001 | 0.00001 | 0.940098 | 0.059882 | 0 | 0 | 0 | 0 | 0 |
| 780 | 0 | 0 | 0.932483 | 0.067517 | 0 | 0 | 0 | 0 | 0 |
| 781 | 0.00002 | 0.00001 | 0.933285 | 0.066685 | 0 | 0 | 0 | 0 | 0 |
| 782 | 0.00002 | 0 | 0.937715 | 0.062265 | 0 | 0 | 0 | 0 | 0 |
| 783 | 0.00002 | 0 | 0.933335 | 0.066645 | 0 | 0 | 0 | 0 | 0 |
| 784 | 0.00001 | 0 | 0.93247 | 0.06752 | 0 | 0 | 0 | 0 | 0 |
| 785 | 0.00002 | 0 | 0.935826 | 0.064154 | 0 | 0 | 0 | 0 | 0 |
| 786 | 0.00004 | 0 | 0.933859 | 0.066101 | 0 | 0 | 0 | 0 | 0 |
| 787 | 0.00003 | 0 | 0.932329 | 0.067641 | 0 | 0 | 0 | 0 | 0 |
| 788 | 0.00002 | 0 | 0.938221 | 0.061759 | 0 | 0 | 0 | 0 | 0 |
| 789 | 0.00002 | 0 | 0.933409 | 0.066571 | 0 | 0 | 0 | 0 | 0 |
| 790 | 0.00001 | 0 | 0.932952 | 0.067038 | 0 | 0 | 0 | 0 | 0 |
| 791 | 0 | 0 | 0.934638 | 0.065362 | 0 | 0 | 0 | 0 | 0 |
| 792 | 0.00004 | 0 | 0.936303 | 0.063658 | 0 | 0 | 0 | 0 | 0 |
| 793 | 0.00002 | 0 | 0.933634 | 0.066346 | 0 | 0 | 0 | 0 | 0 |
| 794 | 0.00001 | 0 | 0.936125 | 0.063865 | 0 | 0 | 0 | 0 | 0 |
| 795 | 0.00001 | 0 | 0.93223 | 0.06776 | 0 | 0 | 0 | 0 | 0 |
| 796 | 0.00001 | 0 | 0.938439 | 0.061551 | 0 | 0 | 0 | 0 | 0 |
| 797 | 0.00002 | 0 | 0.936692 | 0.063288 | 0 | 0 | 0 | 0 | 0 |
| 798 | 0.00001 | 0 | 0.931481 | 0.068379 | 0 | 0 | 0 | 0 | 0 |
| 799 | 0.00001 | 0 | 0.936167 | 0.063823 | 0 | 0 | 0 | 0 | 0 |
| 800 | 0 | 0 | 0.931495 | 0.068505 | 0 | 0 | 0 | 0 | 0 |
| 801 | 0.00003 | 0 | 0.937388 | 0.062582 | 0 | 0 | 0 | 0 | 0 |
| 802 | 0 | 0 | 0.930342 | 0.069658 | 0 | 0 | 0 | 0 | 0 |
| 803 | 0.00003 | 0 | 0.936824 | 0.063146 | 0 | 0 | 0 | 0 | 0 |
| 804 | 0 | 0 | 0.93364 | 0.06636 | 0 | 0 | 0 | 0 | 0 |
| 805 | 0.00002 | 0 | 0.936795 | 0.063185 | 0 | 0 | 0 | 0 | 0 |
| 806 | 0.00002 | 0 | 0.932503 | 0.067477 | 0 | 0 | 0 | 0 | 0 |
| 807 | 0.00001 | 0 | 0.938763 | 0.061227 | 0 | 0 | 0 | 0 | 0 |
| 808 | 0 | 0 | 0.933429 | 0.066571 | 0 | 0 | 0 | 0 | 0 |
| 809 | 0.00001 | 0 | 0.935417 | 0.064573 | 0 | 0 | 0 | 0 | 0 |
| 810 | 0.00001 | 0 | 0.932462 | 0.067528 | 0 | 0 | 0 | 0 | 0 |
| 811 | 0.00001 | 0 | 0.935327 | 0.064663 | 0 | 0 | 0 | 0 | 0 |
| 812 | 0.00004 | 0 | 0.935299 | 0.064661 | 0 | 0 | 0 | 0 | 0 |
| 813 | 0.00001 | 0.00001 | 0.933974 | 0.066006 | 0 | 0 | 0 | 0 | 0 |
| 814 | 0.00004 | 0 | 0.933433 | 0.066527 | 0 | 0 | 0 | 0 | 0 |
| 815 | 0.00001 | 0 | 0.933752 | 0.066238 | 0 | 0 | 0 | 0 | 0 |
| 816 | 0.00001 | 0 | 0.934693 | 0.065297 | 0 | 0 | 0 | 0 | 0 |
| 817 | 0.00002 | 0 | 0.936521 | 0.063459 | 0 | 0 | 0 | 0 | 0 |
| 818 | 0.00002 | 0 | 0.930162 | 0.069818 | 0 | 0 | 0 | 0 | 0 |
| 819 | 0 | 0 | 0.932855 | 0.067145 | 0 | 0 | 0 | 0 | 0 |
| 820 | 0 | 0 | 0.933367 | 0.066633 | 0 | 0 | 0 | 0 | 0 |
| 821 | 0.00001 | 0 | 0.937257 | 0.062733 | 0 | 0 | 0 | 0 | 0 |
| 822 | 0.00001 | 0 | 0.935668 | 0.064322 | 0 | 0 | 0 | 0 | 0 |
| 823 | 0 | 0 | 0.934207 | 0.065793 | 0 | 0 | 0 | 0 | 0 |
| 824 | 0.00001 | 0 | 0.939954 | 0.060036 | 0 | 0 | 0 | 0 | 0 |
| 825 | 0.00001 | 0 | 0.937545 | 0.062445 | 0 | 0 | 0 | 0 | 0 |
| 826 | 0 | 0 | 0.936872 | 0.063128 | 0 | 0 | 0 | 0 | 0 |
| 827 | 0 | 0 | 0.934659 | 0.065341 | 0 | 0 | 0 | 0 | 0 |
| 828 | 0.00001 | 0 | 0.934368 | 0.065622 | 0 | 0 | 0 | 0 | 0 |
| 829 | 0 | 0 | 0.930748 | 0.069252 | 0 | 0 | 0 | 0 | 0 |
| 830 | 0.00002 | 0 | 0.935932 | 0.064048 | 0 | 0 | 0 | 0 | 0 |
| 831 | 0.00001 | 0 | 0.9298 | 0.07019 | 0 | 0 | 0 | 0 | 0 |
| 832 | 0.00001 | 0 | 0.935962 | 0.064028 | 0 | 0 | 0 | 0 | 0 |
| 833 | 0.00003 | 0 | 0.935138 | 0.064832 | 0 | 0 | 0 | 0 | 0 |
| 834 | 0.00002 | 0 | 0.932646 | 0.067334 | 0 | 0 | 0 | 0 | 0 |
| 835 | 0.00001 | 0 | 0.935666 | 0.064324 | 0 | 0 | 0 | 0 | 0 |
| 836 | 0.00001 | 0 | 0.937203 | 0.062787 | 0 | 0 | 0 | 0 | 0 |
| 837 | 0.00003 | 0 | 0.935753 | 0.064217 | 0 | 0 | 0 | 0 | 0 |
| 838 | 0.00002 | 0 | 0.934194 | 0.065786 | 0 | 0 | 0 | 0 | 0 |
| 839 | 0.00003 | 0 | 0.932535 | 0.067435 | 0 | 0 | 0 | 0 | 0 |
| 840 | 0.00003 | 0 | 0.933155 | 0.066815 | 0 | 0 | 0 | 0 | 0 |
| 841 | 0.00002 | 0 | 0.935546 | 0.064434 | 0 | 0 | 0 | 0 | 0 |
| 842 | 0.00002 | 0 | 0.932596 | 0.067384 | 0 | 0 | 0 | 0 | 0 |
| 843 | 0.00001 | 0 | 0.9347 | 0.06529 | 0 | 0 | 0 | 0 | 0 |
| 844 | 0 | 0 | 0.934448 | 0.065552 | 0 | 0 | 0 | 0 | 0 |
| 845 | 0.00002 | 0 | 0.933068 | 0.066912 | 0 | 0 | 0 | 0 | 0 |
| 846 | 0.00004 | 0 | 0.976385 | 0.023575 | 0 | 0 | 0 | 0 | 0 |
| 847 | 0.00001 | 0 | 0.93164 | 0.06835 | 0 | 0 | 0 | 0 | 0 |
| 848 | 0.00001 | 0 | 0.935857 | 0.064133 | 0 | 0 | 0 | 0 | 0 |
| 849 | 0 | 0 | 0.939543 | 0.060457 | 0 | 0 | 0 | 0 | 0 |
| 850 | 0.00001 | 0 | 0.935503 | 0.064487 | 0 | 0 | 0 | 0 | 0 |
| 851 | 0 | 0 | 0.936173 | 0.063827 | 0 | 0 | 0 | 0 | 0 |
| 852 | 0.00002 | 0 | 0.932141 | 0.067838 | 0 | 0 | 0 | 0 | 0 |
| 853 | 0.00001 | 0 | 0.931568 | 0.068423 | 0 | 0 | 0 | 0 | 0 |
| 854 | 0 | 0 | 0.933514 | 0.066486 | 0 | 0 | 0 | 0 | 0 |
| 855 | 0 | 0 | 0.93201 | 0.06799 | 0 | 0 | 0 | 0 | 0 |
| 856 | 0.00002 | 0 | 0.931607 | 0.068373 | 0 | 0 | 0 | 0 | 0 |
| 857 | 0.00001 | 0 | 0.934816 | 0.065174 | 0 | 0 | 0 | 0 | 0 |
| 858 | 0.00001 | 0 | 0.938053 | 0.061937 | 0 | 0 | 0 | 0 | 0 |
| 859 | 0 | 0 | 0.936686 | 0.063314 | 0 | 0 | 0 | 0 | 0 |
| 860 | 0.00003 | 0 | 0.931609 | 0.068361 | 0 | 0 | 0 | 0 | 0 |
| 861 | 0.00001 | 0 | 0.935152 | 0.064537 | 0 | 0 | 0 | 0 | 0 |
| 862 | 0.00001 | 0 | 0.934897 | 0.065093 | 0 | 0 | 0 | 0 | 0 |
| 863 | 0.00001 | 0 | 0.93077 | 0.06922 | 0 | 0 | 0 | 0 | 0 |
| 864 | 0.00001 | 0 | 0.936313 | 0.063677 | 0 | 0 | 0 | 0 | 0 |
| 865 | 0.00002 | 0 | 0.933166 | 0.066814 | 0 | 0 | 0 | 0 | 0 |
| 866 | 0.00002 | 0 | 0.934271 | 0.065709 | 0 | 0 | 0 | 0 | 0 |
| 867 | 0 | 0 | 0.935323 | 0.064677 | 0 | 0 | 0 | 0 | 0 |
| 868 | 0 | 0 | 0.931743 | 0.068257 | 0 | 0 | 0 | 0 | 0 |
| 869 | 0.00004 | 0 | 0.935098 | 0.064862 | 0 | 0 | 0 | 0 | 0 |
| 870 | 0.00003 | 0 | 0.935109 | 0.064861 | 0 | 0 | 0 | 0 | 0 |
| 871 | 0.00001 | 0.00001 | 0.939377 | 0.060603 | 0 | 0 | 0 | 0 | 0 |
| 872 | 0.00002 | 0 | 0.933065 | 0.066915 | 0 | 0 | 0 | 0 | 0 |
| 873 | 0.00002 | 0 | 0.931754 | 0.068226 | 0 | 0 | 0 | 0 | 0 |
| 874 | 0.00001 | 0 | 0.934546 | 0.065444 | 0 | 0 | 0 | 0 | 0 |
| 875 | 0.00001 | 0 | 0.936716 | 0.063274 | 0 | 0 | 0 | 0 | 0 |
| 876 | 0.00001 | 0 | 0.934549 | 0.065441 | 0 | 0 | 0 | 0 | 0 |
| 877 | 0 | 0 | 0.938829 | 0.061171 | 0 | 0 | 0 | 0 | 0 |
| 878 | 0.00001 | 0 | 0.937177 | 0.062813 | 0 | 0 | 0 | 0 | 0 |
| 879 | 0 | 0 | 0.93418 | 0.06582 | 0 | 0 | 0 | 0 | 0 |
| 880 | 0 | 0 | 0.934259 | 0.065741 | 0 | 0 | 0 | 0 | 0 |
| 881 | 0.00001 | 0 | 0.93435 | 0.06564 | 0 | 0 | 0 | 0 | 0 |
| 882 | 0 | 0 | 0.932134 | 0.067866 | 0 | 0 | 0 | 0 | 0 |
| 883 | 0.00004 | 0 | 0.933843 | 0.066117 | 0 | 0 | 0 | 0 | 0 |
| 884 | 0.00003 | 0 | 0.932346 | 0.067624 | 0 | 0 | 0 | 0 | 0 |
| 885 | 0.00001 | 0 | 0.939354 | 0.060636 | 0 | 0 | 0 | 0 | 0 |
| 886 | 0.00001 | 0 | 0.932834 | 0.067156 | 0 | 0 | 0 | 0 | 0 |
| 887 | 0.00001 | 0 | 0.935212 | 0.064778 | 0 | 0 | 0 | 0 | 0 |
| 888 | 0 | 0 | 0.930455 | 0.069545 | 0 | 0 | 0 | 0 | 0 |
| 889 | 0.00002 | 0 | 0.927778 | 0.072202 | 0 | 0 | 0 | 0 | 0 |
| 890 | 0 | 0 | 0.936082 | 0.063918 | 0 | 0 | 0 | 0 | 0 |
| 891 | 0.00003 | 0 | 0.932071 | 0.067899 | 0 | 0 | 0 | 0 | 0 |
| 892 | 0 | 0 | 0.934569 | 0.065431 | 0 | 0 | 0 | 0 | 0 |
| 893 | 0.00001 | 0 | 0.932758 | 0.067232 | 0 | 0 | 0 | 0 | 0 |
| 894 | 0 | 0 | 0.931269 | 0.068731 | 0 | 0 | 0 | 0 | 0 |
| 895 | 0.00002 | 0 | 0.940244 | 0.059736 | 0 | 0 | 0 | 0 | 0 |
| 896 | 0.00002 | 0 | 0.933192 | 0.066788 | 0 | 0 | 0 | 0 | 0 |
| 897 | 0 | 0 | 0.937281 | 0.062719 | 0 | 0 | 0 | 0 | 0 |
| 898 | 0.00002 | 0 | 0.935294 | 0.064686 | 0 | 0 | 0 | 0 | 0 |
| 899 | 0.00001 | 0 | 0.936736 | 0.063254 | 0 | 0 | 0 | 0 | 0 |
| 900 | 0.00001 | 0 | 0.935329 | 0.064661 | 0 | 0 | 0 | 0 | 0 |
| 901 | 0.00002 | 0 | 0.938669 | 0.061311 | 0 | 0 | 0 | 0 | 0 |
| 902 | 0 | 0 | 0.932623 | 0.067377 | 0 | 0 | 0 | 0 | 0 |
| 903 | 0.00001 | 0 | 0.933413 | 0.066577 | 0 | 0 | 0 | 0 | 0 |
| 904 | 0 | 0 | 0.938464 | 0.061536 | 0 | 0 | 0 | 0 | 0 |
| 905 | 0.00002 | 0 | 0.935719 | 0.064261 | 0 | 0 | 0 | 0 | 0 |
| 906 | 0.00002 | 0 | 0.933546 | 0.066434 | 0 | 0 | 0 | 0 | 0 |
| 907 | 0.00001 | 0 | 0.940814 | 0.059177 | 0 | 0 | 0 | 0 | 0 |
| 908 | 0 | 0 | 0.934804 | 0.065196 | 0 | 0 | 0 | 0 | 0 |
| 909 | 0.00002 | 0 | 0.934967 | 0.065013 | 0 | 0 | 0 | 0 | 0 |
| 910 | 0.00001 | 0 | 0.933908 | 0.066082 | 0 | 0 | 0 | 0 | 0 |
| 911 | 0.00001 | 0 | 0.937007 | 0.062983 | 0 | 0 | 0 | 0 | 0 |
| 912 | 0 | 0 | 0.933398 | 0.066602 | 0 | 0 | 0 | 0 | 0 |
| 913 | 0.00001 | 0.00002 | 0.934108 | 0.065862 | 0 | 0 | 0 | 0 | 0 |
| 914 | 0 | 0 | 0.938027 | 0.061973 | 0 | 0 | 0 | 0 | 0 |
| 915 | 0.00001 | 0 | 0.931564 | 0.068426 | 0 | 0 | 0 | 0 | 0 |
| 916 | 0.00002 | 0 | 0.932363 | 0.067617 | 0 | 0 | 0 | 0 | 0 |
| 917 | 0.00003 | 0 | 0.935107 | 0.064863 | 0 | 0 | 0 | 0 | 0 |
| 918 | 0.00002 | 0 | 0.938951 | 0.061029 | 0 | 0 | 0 | 0 | 0 |
| 919 | 0.00001 | 0 | 0.931683 | 0.068307 | 0 | 0 | 0 | 0 | 0 |
| 920 | 0.00001 | 0 | 0.938003 | 0.061986 | 0 | 0 | 0 | 0 | 0 |
| 921 | 0.00002 | 0.00001 | 0.935324 | 0.064646 | 0 | 0 | 0 | 0 | 0 |
| 922 | 0 | 0 | 0.935234 | 0.064766 | 0 | 0 | 0 | 0 | 0 |
| 923 | 0.00002 | 0 | 0.933173 | 0.066807 | 0 | 0 | 0 | 0 | 0 |
| 924 | 0 | 0 | 0.935668 | 0.064332 | 0 | 0 | 0 | 0 | 0 |
| 925 | 0 | 0 | 0.935039 | 0.064961 | 0 | 0 | 0 | 0 | 0 |
| 926 | 0.00002 | 0 | 0.937141 | 0.062839 | 0 | 0 | 0 | 0 | 0 |
| 927 | 0 | 0 | 0.932008 | 0.067992 | 0 | 0 | 0 | 0 | 0 |
| 928 | 0.00001 | 0 | 0.933799 | 0.066191 | 0 | 0 | 0 | 0 | 0 |
| 929 | 0.00001 | 0 | 0.928471 | 0.071519 | 0 | 0 | 0 | 0 | 0 |
| 930 | 0 | 0 | 0.935267 | 0.064733 | 0 | 0 | 0 | 0 | 0 |
| 931 | 0.00005 | 0 | 0.938555 | 0.061395 | 0 | 0 | 0 | 0 | 0 |
| 932 | 0.00002 | 0 | 0.936045 | 0.063935 | 0 | 0 | 0 | 0 | 0 |
| 933 | 0.00002 | 0 | 0.939312 | 0.060668 | 0 | 0 | 0 | 0 | 0 |
| 934 | 0.00001 | 0 | 0.929568 | 0.070422 | 0 | 0 | 0 | 0 | 0 |
| 935 | 0 | 0 | 0.934997 | 0.065003 | 0 | 0 | 0 | 0 | 0 |
| 936 | 0 | 0 | 0.935818 | 0.064182 | 0 | 0 | 0 | 0 | 0 |
| 937 | 0.00002 | 0 | 0.935934 | 0.064045 | 0 | 0 | 0 | 0 | 0 |
| 938 | 0.00002 | 0 | 0.93827 | 0.06171 | 0 | 0 | 0 | 0 | 0 |
| 939 | 0 | 0 | 0.938005 | 0.061995 | 0 | 0 | 0 | 0 | 0 |
| 940 | 0.00001 | 0 | 0.932025 | 0.067965 | 0 | 0 | 0 | 0 | 0 |
| 941 | 0.00002 | 0 | 0.933476 | 0.066504 | 0 | 0 | 0 | 0 | 0 |
| 942 | 0.00002 | 0 | 0.934808 | 0.065172 | 0 | 0 | 0 | 0 | 0 |
| 943 | 0.00002 | 0 | 0.936536 | 0.063444 | 0 | 0 | 0 | 0 | 0 |
| 944 | 0 | 0 | 0.937272 | 0.062728 | 0 | 0 | 0 | 0 | 0 |
| 945 | 0 | 0 | 0.932019 | 0.067981 | 0 | 0 | 0 | 0 | 0 |
| 946 | 0 | 0 | 0.932492 | 0.067508 | 0 | 0 | 0 | 0 | 0 |
| 947 | 0.00001 | 0 | 0.934858 | 0.065132 | 0 | 0 | 0 | 0 | 0 |
| 948 | 0.00001 | 0 | 0.934215 | 0.065775 | 0 | 0 | 0 | 0 | 0 |
| 949 | 0.00001 | 0 | 0.936185 | 0.063805 | 0 | 0 | 0 | 0 | 0 |
| 950 | 0.00002 | 0 | 0.938625 | 0.061355 | 0 | 0 | 0 | 0 | 0 |
| 951 | 0.00002 | 0.00003 | 0.93932 | 0.06063 | 0 | 0 | 0 | 0 | 0 |
| 952 | 0 | 0 | 0.936926 | 0.063074 | 0 | 0 | 0 | 0 | 0 |
| 953 | 0.00002 | 0 | 0.934836 | 0.065144 | 0 | 0 | 0 | 0 | 0 |
| 954 | 0 | 0 | 0.931279 | 0.068721 | 0 | 0 | 0 | 0 | 0 |
| 955 | 0.00001 | 0 | 0.936408 | 0.063582 | 0 | 0 | 0 | 0 | 0 |
| 956 | 0 | 0 | 0.937885 | 0.062115 | 0 | 0 | 0 | 0 | 0 |
| 957 | 0 | 0 | 0.930355 | 0.069645 | 0 | 0 | 0 | 0 | 0 |
| 958 | 0 | 0 | 0.937292 | 0.062708 | 0 | 0 | 0 | 0 | 0 |
| 959 | 0.00001 | 0 | 0.933123 | 0.066867 | 0 | 0 | 0 | 0 | 0 |
| 960 | 0.00003 | 0 | 0.937428 | 0.062542 | 0 | 0 | 0 | 0 | 0 |
| 961 | 0.00002 | 0 | 0.932543 | 0.067437 | 0 | 0 | 0 | 0 | 0 |
| 962 | 0.00001 | 0 | 0.935694 | 0.064296 | 0 | 0 | 0 | 0 | 0 |
| 963 | 0 | 0 | 0.932905 | 0.067095 | 0 | 0 | 0 | 0 | 0 |
| 964 | 0.00001 | 0 | 0.937926 | 0.062064 | 0 | 0 | 0 | 0 | 0 |
| 965 | 0.00001 | 0 | 0.937277 | 0.062714 | 0 | 0 | 0 | 0 | 0 |
| 966 | 0.00002 | 0 | 0.936828 | 0.063152 | 0 | 0 | 0 | 0 | 0 |
| 967 | 0.00001 | 0 | 0.939309 | 0.060681 | 0 | 0 | 0 | 0 | 0 |
| 968 | 0.00004 | 0 | 0.933327 | 0.066633 | 0 | 0 | 0 | 0 | 0 |
| 969 | 0.00001 | 0.00001 | 0.932527 | 0.067453 | 0 | 0 | 0 | 0 | 0 |
| 970 | 0.00003 | 0 | 0.931448 | 0.068522 | 0 | 0 | 0 | 0 | 0 |
| 971 | 0.00001 | 0 | 0.936163 | 0.063827 | 0 | 0 | 0 | 0 | 0 |
| 972 | 0.00001 | 0 | 0.933388 | 0.066602 | 0 | 0 | 0 | 0 | 0 |
| 973 | 0 | 0 | 0.936735 | 0.063265 | 0 | 0 | 0 | 0 | 0 |
| 974 | 0.00002 | 0 | 0.931543 | 0.068437 | 0 | 0 | 0 | 0 | 0 |
| 975 | 0.00002 | 0 | 0.940169 | 0.059811 | 0 | 0 | 0 | 0 | 0 |
| 976 | 0 | 0 | 0.936121 | 0.063879 | 0 | 0 | 0 | 0 | 0 |
| 977 | 0.00003 | 0 | 0.934746 | 0.065223 | 0 | 0 | 0 | 0 | 0 |
| 978 | 0.00002 | 0 | 0.93599 | 0.06399 | 0 | 0 | 0 | 0 | 0 |
| 979 | 0.00001 | 0 | 0.93396 | 0.06603 | 0 | 0 | 0 | 0 | 0 |
| 980 | 0 | 0 | 0.935401 | 0.064599 | 0 | 0 | 0 | 0 | 0 |
| 981 | 0.00001 | 0 | 0.939035 | 0.060955 | 0 | 0 | 0 | 0 | 0 |
| 982 | 0.00002 | 0 | 0.930078 | 0.069901 | 0 | 0 | 0 | 0 | 0 |
| 983 | 0.00002 | 0 | 0.931587 | 0.068393 | 0 | 0 | 0 | 0 | 0 |
| 984 | 0.00002 | 0 | 0.934915 | 0.065065 | 0 | 0 | 0 | 0 | 0 |
| 985 | 0.00001 | 0 | 0.932943 | 0.067047 | 0 | 0 | 0 | 0 | 0 |
| 986 | 0 | 0 | 0.937766 | 0.062234 | 0 | 0 | 0 | 0 | 0 |
| 987 | 0.00001 | 0 | 0.935189 | 0.064801 | 0 | 0 | 0 | 0 | 0 |
| 988 | 0 | 0 | 0.934285 | 0.065715 | 0 | 0 | 0 | 0 | 0 |
| 989 | 0.00002 | 0 | 0.932694 | 0.067286 | 0 | 0 | 0 | 0 | 0 |
| 990 | 0.00001 | 0.00001 | 0.931298 | 0.068682 | 0 | 0 | 0 | 0 | 0 |
| 991 | 0.00001 | 0 | 0.93534 | 0.06465 | 0 | 0 | 0 | 0 | 0 |
| 992 | 0.00001 | 0 | 0.935708 | 0.064282 | 0 | 0 | 0 | 0 | 0 |
| 993 | 0 | 0 | 0.939008 | 0.060992 | 0 | 0 | 0 | 0 | 0 |
| 994 | 0 | 0 | 0.939424 | 0.060576 | 0 | 0 | 0 | 0 | 0 |
| 995 | 0 | 0 | 0.930535 | 0.069465 | 0 | 0 | 0 | 0 | 0 |
| 996 | 0.00004 | 0 | 0.937967 | 0.061993 | 0 | 0 | 0 | 0 | 0 |
| 997 | 0.00001 | 0 | 0.930107 | 0.069883 | 0 | 0 | 0 | 0 | 0 |
| 998 | 0 | 0 | 0.934261 | 0.065739 | 0 | 0 | 0 | 0 | 0 |
| 999 | 0 | 0 | 0.938095 | 0.061905 | 0 | 0 | 0 | 0 | 0 |
| 1000 | 0.00003 | 0 | 0.932379 | 0.067591 | 0 | 0 | 0 | 0 | 0 |

| Year 14 | Race |  |  |  |  |  |  |  |  |
| --- | --- | --- | --- | --- | --- | --- | --- | --- | --- |
| Trial | 003.0 | 005.0 | 007.0 | 037.1 | 043.0 | 303.0 | 003.2 | 403.0 | 003.4 |
| 1 | 0.00001 | 0 | 0.939366 | 0.060624 | 0 | 0 | 0 | 0 | 0 |
| 2 | 0.00002 | 0 | 0.936608 | 0.063372 | 0 | 0 | 0 | 0 | 0 |
| 3 | 0.00001 | 0 | 0.942126 | 0.057864 | 0 | 0 | 0 | 0 | 0 |
| 4 | 0 | 0 | 0.938803 | 0.061197 | 0 | 0 | 0 | 0 | 0 |
| 5 | 0.00002 | 0 | 0.938732 | 0.061248 | 0 | 0 | 0 | 0 | 0 |
| 6 | 0.00001 | 0 | 0.939871 | 0.060119 | 0 | 0 | 0 | 0 | 0 |
| 7 | 0.00001 | 0 | 0.938489 | 0.061501 | 0 | 0 | 0 | 0 | 0 |
| 8 | 0.00001 | 0 | 0.942638 | 0.057352 | 0 | 0 | 0 | 0 | 0 |
| 9 | 0 | 0 | 0.936358 | 0.063642 | 0 | 0 | 0 | 0 | 0 |
| 10 | 0 | 0.00001 | 0.93923 | 0.06076 | 0 | 0 | 0 | 0 | 0 |
| 11 | 0.00002 | 0 | 0.944389 | 0.055591 | 0 | 0 | 0 | 0 | 0 |
| 12 | 0.00001 | 0 | 0.93709 | 0.0629 | 0 | 0 | 0 | 0 | 0 |
| 13 | 0.00001 | 0 | 0.939376 | 0.060614 | 0 | 0 | 0 | 0 | 0 |
| 14 | 0.00002 | 0 | 0.936565 | 0.063415 | 0 | 0 | 0 | 0 | 0 |
| 15 | 0 | 0 | 0.936506 | 0.063494 | 0 | 0 | 0 | 0 | 0 |
| 16 | 0.00001 | 0 | 0.939092 | 0.060898 | 0 | 0 | 0 | 0 | 0 |
| 17 | 0.00003 | 0 | 0.939927 | 0.060043 | 0 | 0 | 0 | 0 | 0 |
| 18 | 0 | 0 | 0.939234 | 0.060766 | 0 | 0 | 0 | 0 | 0 |
| 19 | 0 | 0 | 0.938224 | 0.061776 | 0 | 0 | 0 | 0 | 0 |
| 20 | 0.00001 | 0 | 0.941437 | 0.058553 | 0 | 0 | 0 | 0 | 0 |
| 21 | 0.00001 | 0 | 0.937504 | 0.062485 | 0 | 0 | 0 | 0 | 0 |
| 22 | 0 | 0 | 0.93848 | 0.06152 | 0 | 0 | 0 | 0 | 0 |
| 23 | 0.00003 | 0 | 0.941844 | 0.058127 | 0 | 0 | 0 | 0 | 0 |
| 24 | 0 | 0 | 0.941329 | 0.058671 | 0 | 0 | 0 | 0 | 0 |
| 25 | 0.00002 | 0 | 0.939266 | 0.060714 | 0 | 0 | 0 | 0 | 0 |
| 26 | 0.00001 | 0 | 0.940746 | 0.059244 | 0 | 0 | 0 | 0 | 0 |
| 27 | 0 | 0 | 0.938292 | 0.061708 | 0 | 0 | 0 | 0 | 0 |
| 28 | 0 | 0 | 0.94267 | 0.05698 | 0 | 0 | 0 | 0 | 0 |
| 29 | 0.00002 | 0 | 0.941444 | 0.058536 | 0 | 0 | 0 | 0 | 0 |
| 30 | 0 | 0 | 0.936363 | 0.063637 | 0 | 0 | 0 | 0 | 0 |
| 31 | 0.00001 | 0 | 0.94169 | 0.0583 | 0 | 0 | 0 | 0 | 0 |
| 32 | 0.00002 | 0 | 0.939972 | 0.060009 | 0 | 0 | 0 | 0 | 0 |
| 33 | 0.00001 | 0 | 0.938823 | 0.061167 | 0 | 0 | 0 | 0 | 0 |
| 34 | 0.00001 | 0 | 0.94104 | 0.05895 | 0 | 0 | 0 | 0 | 0 |
| 35 | 0.00001 | 0 | 0.936712 | 0.063278 | 0 | 0 | 0 | 0 | 0 |
| 36 | 0 | 0 | 0.936724 | 0.063276 | 0 | 0 | 0 | 0 | 0 |
| 37 | 0.00001 | 0 | 0.945003 | 0.054987 | 0 | 0 | 0 | 0 | 0 |
| 38 | 0.00001 | 0 | 0.939948 | 0.060042 | 0 | 0 | 0 | 0 | 0 |
| 39 | 0.00001 | 0 | 0.939199 | 0.060791 | 0 | 0 | 0 | 0 | 0 |
| 40 | 0.00002 | 0 | 0.939178 | 0.060802 | 0 | 0 | 0 | 0 | 0 |
| 41 | 0.00001 | 0 | 0.93548 | 0.06451 | 0 | 0 | 0 | 0 | 0 |
| 42 | 0.00001 | 0 | 0.939894 | 0.060096 | 0 | 0 | 0 | 0 | 0 |
| 43 | 0 | 0 | 0.940001 | 0.059999 | 0 | 0 | 0 | 0 | 0 |
| 44 | 0 | 0 | 0.938466 | 0.061534 | 0 | 0 | 0 | 0 | 0 |
| 45 | 0 | 0 | 0.941125 | 0.058875 | 0 | 0 | 0 | 0 | 0 |
| 46 | 0 | 0 | 0.938923 | 0.061077 | 0 | 0 | 0 | 0 | 0 |
| 47 | 0.00004 | 0 | 0.940166 | 0.059794 | 0 | 0 | 0 | 0 | 0 |
| 48 | 0.00002 | 0 | 0.938179 | 0.0618 | 0 | 0 | 0 | 0 | 0 |
| 49 | 0 | 0 | 0.942672 | 0.057328 | 0 | 0 | 0 | 0 | 0 |
| 50 | 0.00001 | 0 | 0.940546 | 0.059444 | 0 | 0 | 0 | 0 | 0 |
| 51 | 0.00004 | 0 | 0.938786 | 0.061174 | 0 | 0 | 0 | 0 | 0 |
| 52 | 0 | 0 | 0.942173 | 0.057827 | 0 | 0 | 0 | 0 | 0 |
| 53 | 0.00001 | 0 | 0.940171 | 0.059819 | 0 | 0 | 0 | 0 | 0 |
| 54 | 0.00001 | 0 | 0.936797 | 0.063193 | 0 | 0 | 0 | 0 | 0 |
| 55 | 0.00002 | 0 | 0.936624 | 0.063356 | 0 | 0 | 0 | 0 | 0 |
| 56 | 0.00002 | 0 | 0.938877 | 0.061103 | 0 | 0 | 0 | 0 | 0 |
| 57 | 0.00002 | 0 | 0.944148 | 0.055832 | 0 | 0 | 0 | 0 | 0 |
| 58 | 0 | 0 | 0.93906 | 0.06094 | 0 | 0 | 0 | 0 | 0 |
| 59 | 0 | 0 | 0.941937 | 0.058063 | 0 | 0 | 0 | 0 | 0 |
| 60 | 0.00005 | 0 | 0.93986 | 0.06009 | 0 | 0 | 0 | 0 | 0 |
| 61 | 0.00002 | 0 | 0.932751 | 0.067229 | 0 | 0 | 0 | 0 | 0 |
| 62 | 0.00001 | 0 | 0.938675 | 0.061315 | 0 | 0 | 0 | 0 | 0 |
| 63 | 0.00001 | 0 | 0.937221 | 0.062769 | 0 | 0 | 0 | 0 | 0 |
| 64 | 0.00001 | 0 | 0.93876 | 0.06123 | 0 | 0 | 0 | 0 | 0 |
| 65 | 0.00002 | 0 | 0.942682 | 0.057298 | 0 | 0 | 0 | 0 | 0 |
| 66 | 0 | 0 | 0.943336 | 0.056664 | 0 | 0 | 0 | 0 | 0 |
| 67 | 0.00002 | 0 | 0.940656 | 0.059324 | 0 | 0 | 0 | 0 | 0 |
| 68 | 0 | 0 | 0.94108 | 0.05892 | 0 | 0 | 0 | 0 | 0 |
| 69 | 0.00001 | 0 | 0.939429 | 0.060561 | 0 | 0 | 0 | 0 | 0 |
| 70 | 0.00002 | 0 | 0.940783 | 0.059197 | 0 | 0 | 0 | 0 | 0 |
| 71 | 0 | 0 | 0.938471 | 0.0601 | 0 | 0 | 0 | 0 | 0 |
| 72 | 0 | 0 | 0.943214 | 0.056786 | 0 | 0 | 0 | 0 | 0 |
| 73 | 0.00001 | 0 | 0.939343 | 0.060647 | 0 | 0 | 0 | 0 | 0 |
| 74 | 0 | 0 | 0.942984 | 0.057016 | 0 | 0 | 0 | 0 | 0 |
| 75 | 0.00001 | 0 | 0.939253 | 0.060737 | 0 | 0 | 0 | 0 | 0 |
| 76 | 0.00001 | 0 | 0.942013 | 0.057977 | 0 | 0 | 0 | 0 | 0 |
| 77 | 0 | 0 | 0.939562 | 0.060438 | 0 | 0 | 0 | 0 | 0 |
| 78 | 0 | 0 | 0.940643 | 0.059357 | 0 | 0 | 0 | 0 | 0 |
| 79 | 0 | 0 | 0.940529 | 0.059471 | 0 | 0 | 0 | 0 | 0 |
| 80 | 0 | 0 | 0.938454 | 0.061546 | 0 | 0 | 0 | 0 | 0 |
| 81 | 0.00001 | 0 | 0.94187 | 0.05812 | 0 | 0 | 0 | 0 | 0 |
| 82 | 0 | 0 | 0.934384 | 0.065616 | 0 | 0 | 0 | 0 | 0 |
| 83 | 0 | 0 | 0.943291 | 0.056709 | 0 | 0 | 0 | 0 | 0 |
| 84 | 0 | 0 | 0.940517 | 0.059483 | 0 | 0 | 0 | 0 | 0 |
| 85 | 0 | 0 | 0.939399 | 0.060601 | 0 | 0 | 0 | 0 | 0 |
| 86 | 0.00002 | 0 | 0.94694 | 0.053039 | 0 | 0 | 0 | 0 | 0 |
| 87 | 0 | 0 | 0.938565 | 0.061435 | 0 | 0 | 0 | 0 | 0 |
| 88 | 0.00004 | 0 | 0.939145 | 0.060815 | 0 | 0 | 0 | 0 | 0 |
| 89 | 0.00003 | 0 | 0.936327 | 0.063643 | 0 | 0 | 0 | 0 | 0 |
| 90 | 0 | 0 | 0.940795 | 0.059205 | 0 | 0 | 0 | 0 | 0 |
| 91 | 0.00002 | 0 | 0.938248 | 0.061732 | 0 | 0 | 0 | 0 | 0 |
| 92 | 0.00001 | 0 | 0.940509 | 0.059481 | 0 | 0 | 0 | 0 | 0 |
| 93 | 0.00001 | 0 | 0.942331 | 0.057659 | 0 | 0 | 0 | 0 | 0 |
| 94 | 0.00001 | 0 | 0.942653 | 0.057338 | 0 | 0 | 0 | 0 | 0 |
| 95 | 0.00004 | 0 | 0.939416 | 0.060544 | 0 | 0 | 0 | 0 | 0 |
| 96 | 0.00001 | 0 | 0.941527 | 0.058463 | 0 | 0 | 0 | 0 | 0 |
| 97 | 0.00002 | 0 | 0.939295 | 0.060685 | 0 | 0 | 0 | 0 | 0 |
| 98 | 0 | 0 | 0.938454 | 0.061546 | 0 | 0 | 0 | 0 | 0 |
| 99 | 0.00001 | 0 | 0.936936 | 0.063054 | 0 | 0 | 0 | 0 | 0 |
| 100 | 0.00002 | 0 | 0.94167 | 0.05831 | 0 | 0 | 0 | 0 | 0 |
| 101 | 0 | 0 | 0.943167 | 0.056833 | 0 | 0 | 0 | 0 | 0 |
| 102 | 0 | 0 | 0.940717 | 0.059283 | 0 | 0 | 0 | 0 | 0 |
| 103 | 0.00001 | 0 | 0.939209 | 0.060781 | 0 | 0 | 0 | 0 | 0 |
| 104 | 0.00001 | 0 | 0.939236 | 0.060754 | 0 | 0 | 0 | 0 | 0 |
| 105 | 0.00001 | 0 | 0.940559 | 0.059431 | 0 | 0 | 0 | 0 | 0 |
| 106 | 0 | 0 | 0.940841 | 0.059159 | 0 | 0 | 0 | 0 | 0 |
| 107 | 0 | 0 | 0.941141 | 0.058331 | 0 | 0 | 0 | 0 | 0 |
| 108 | 0.00001 | 0 | 0.935855 | 0.064135 | 0 | 0 | 0 | 0 | 0 |
| 109 | 0.00001 | 0 | 0.941358 | 0.058632 | 0 | 0 | 0 | 0 | 0 |
| 110 | 0.00001 | 0 | 0.939856 | 0.060134 | 0 | 0 | 0 | 0 | 0 |
| 111 | 0 | 0 | 0.9412 | 0.0588 | 0 | 0 | 0 | 0 | 0 |
| 112 | 0.00007 | 0 | 0.938013 | 0.061917 | 0 | 0 | 0 | 0 | 0 |
| 113 | 0.00001 | 0 | 0.938645 | 0.061345 | 0 | 0 | 0 | 0 | 0 |
| 114 | 0.00001 | 0 | 0.940732 | 0.059258 | 0 | 0 | 0 | 0 | 0 |
| 115 | 0.00001 | 0 | 0.942569 | 0.057421 | 0 | 0 | 0 | 0 | 0 |
| 116 | 0 | 0 | 0.938644 | 0.061356 | 0 | 0 | 0 | 0 | 0 |
| 117 | 0.00003 | 0 | 0.939502 | 0.060468 | 0 | 0 | 0 | 0 | 0 |
| 118 | 0 | 0 | 0.941195 | 0.058805 | 0 | 0 | 0 | 0 | 0 |
| 119 | 0.00001 | 0 | 0.940456 | 0.059535 | 0 | 0 | 0 | 0 | 0 |
| 120 | 0.00001 | 0 | 0.938553 | 0.061437 | 0 | 0 | 0 | 0 | 0 |
| 121 | 0 | 0 | 0.93905 | 0.06095 | 0 | 0 | 0 | 0 | 0 |
| 122 | 0.00001 | 0 | 0.942894 | 0.057096 | 0 | 0 | 0 | 0 | 0 |
| 123 | 0.00003 | 0 | 0.940774 | 0.059196 | 0 | 0 | 0 | 0 | 0 |
| 124 | 0.00001 | 0 | 0.942463 | 0.057527 | 0 | 0 | 0 | 0 | 0 |
| 125 | 0.00001 | 0 | 0.9391 | 0.06089 | 0 | 0 | 0 | 0 | 0 |
| 126 | 0.00001 | 0 | 0.942036 | 0.057954 | 0 | 0 | 0 | 0 | 0 |
| 127 | 0.00001 | 0 | 0.939456 | 0.060534 | 0 | 0 | 0 | 0 | 0 |
| 128 | 0.00002 | 0 | 0.93669 | 0.06329 | 0 | 0 | 0 | 0 | 0 |
| 129 | 0 | 0 | 0.939877 | 0.060123 | 0 | 0 | 0 | 0 | 0 |
| 130 | 0.00001 | 0 | 0.940875 | 0.059115 | 0 | 0 | 0 | 0 | 0 |
| 131 | 0 | 0 | 0.941284 | 0.058716 | 0 | 0 | 0 | 0 | 0 |
| 132 | 0.00001 | 0 | 0.941107 | 0.058883 | 0 | 0 | 0 | 0 | 0 |
| 133 | 0.00003 | 0 | 0.937535 | 0.062435 | 0 | 0 | 0 | 0 | 0 |
| 134 | 0.00002 | 0 | 0.942067 | 0.057913 | 0 | 0 | 0 | 0 | 0 |
| 135 | 0 | 0 | 0.936806 | 0.063194 | 0 | 0 | 0 | 0 | 0 |
| 136 | 0.00001 | 0 | 0.940287 | 0.059703 | 0 | 0 | 0 | 0 | 0 |
| 137 | 0 | 0 | 0.940771 | 0.059229 | 0 | 0 | 0 | 0 | 0 |
| 138 | 0.00002 | 0 | 0.93691 | 0.06307 | 0 | 0 | 0 | 0 | 0 |
| 139 | 0.00001 | 0 | 0.94159 | 0.0584 | 0 | 0 | 0 | 0 | 0 |
| 140 | 0 | 0 | 0.938098 | 0.061902 | 0 | 0 | 0 | 0 | 0 |
| 141 | 0.00001 | 0 | 0.938797 | 0.061193 | 0 | 0 | 0 | 0 | 0 |
| 142 | 0 | 0 | 0.935473 | 0.064527 | 0 | 0 | 0 | 0 | 0 |
| 143 | 0 | 0 | 0.939898 | 0.060102 | 0 | 0 | 0 | 0 | 0 |
| 144 | 0.00003 | 0 | 0.937502 | 0.062468 | 0 | 0 | 0 | 0 | 0 |
| 145 | 0.00001 | 0 | 0.940831 | 0.059159 | 0 | 0 | 0 | 0 | 0 |
| 146 | 0 | 0 | 0.943289 | 0.056711 | 0 | 0 | 0 | 0 | 0 |
| 147 | 0.00001 | 0 | 0.939128 | 0.060862 | 0 | 0 | 0 | 0 | 0 |
| 148 | 0 | 0 | 0.940618 | 0.059382 | 0 | 0 | 0 | 0 | 0 |
| 149 | 0.00002 | 0 | 0.938816 | 0.061164 | 0 | 0 | 0 | 0 | 0 |
| 150 | 0.00003 | 0 | 0.943005 | 0.056965 | 0 | 0 | 0 | 0 | 0 |
| 151 | 0 | 0 | 0.941135 | 0.058865 | 0 | 0 | 0 | 0 | 0 |
| 152 | 0.00001 | 0 | 0.93637 | 0.06362 | 0 | 0 | 0 | 0 | 0 |
| 153 | 0.00001 | 0 | 0.942775 | 0.057215 | 0 | 0 | 0 | 0 | 0 |
| 154 | 0 | 0 | 0.941057 | 0.058943 | 0 | 0 | 0 | 0 | 0 |
| 155 | 0.00001 | 0 | 0.939791 | 0.060199 | 0 | 0 | 0 | 0 | 0 |
| 156 | 0.00005 | 0 | 0.940382 | 0.059569 | 0 | 0 | 0 | 0 | 0 |
| 157 | 0.00002 | 0 | 0.938354 | 0.061626 | 0 | 0 | 0 | 0 | 0 |
| 158 | 0.00001 | 0 | 0.936102 | 0.063888 | 0 | 0 | 0 | 0 | 0 |
| 159 | 0.00003 | 0 | 0.938508 | 0.061462 | 0 | 0 | 0 | 0 | 0 |
| 160 | 0.00001 | 0 | 0.939421 | 0.060569 | 0 | 0 | 0 | 0 | 0 |
| 161 | 0.00001 | 0 | 0.935831 | 0.064159 | 0 | 0 | 0 | 0 | 0 |
| 162 | 0.00001 | 0 | 0.939737 | 0.060253 | 0 | 0 | 0 | 0 | 0 |
| 163 | 0.00001 | 0 | 0.938792 | 0.061198 | 0 | 0 | 0 | 0 | 0 |
| 164 | 0.00001 | 0 | 0.933997 | 0.065993 | 0 | 0 | 0 | 0 | 0 |
| 165 | 0 | 0 | 0.944912 | 0.055088 | 0 | 0 | 0 | 0 | 0 |
| 166 | 0 | 0 | 0.940091 | 0.059909 | 0 | 0 | 0 | 0 | 0 |
| 167 | 0 | 0 | 0.939925 | 0.060075 | 0 | 0 | 0 | 0 | 0 |
| 168 | 0.00001 | 0 | 0.937864 | 0.062126 | 0 | 0 | 0 | 0 | 0 |
| 169 | 0.00003 | 0 | 0.935468 | 0.064502 | 0 | 0 | 0 | 0 | 0 |
| 170 | 0.00002 | 0 | 0.939034 | 0.060946 | 0 | 0 | 0 | 0 | 0 |
| 171 | 0.00002 | 0 | 0.942765 | 0.057215 | 0 | 0 | 0 | 0 | 0 |
| 172 | 0.00001 | 0 | 0.93778 | 0.06221 | 0 | 0 | 0 | 0 | 0 |
| 173 | 0 | 0 | 0.94018 | 0.05982 | 0 | 0 | 0 | 0 | 0 |
| 174 | 0 | 0 | 0.93742 | 0.06258 | 0 | 0 | 0 | 0 | 0 |
| 175 | 0.00001 | 0 | 0.937311 | 0.062679 | 0 | 0 | 0 | 0 | 0 |
| 176 | 0 | 0 | 0.941266 | 0.058734 | 0 | 0 | 0 | 0 | 0 |
| 177 | 0 | 0 | 0.9418 | 0.0582 | 0 | 0 | 0 | 0 | 0 |
| 178 | 0 | 0 | 0.940432 | 0.059568 | 0 | 0 | 0 | 0 | 0 |
| 179 | 0 | 0 | 0.93924 | 0.06076 | 0 | 0 | 0 | 0 | 0 |
| 180 | 0.00001 | 0 | 0.940552 | 0.059438 | 0 | 0 | 0 | 0 | 0 |
| 181 | 0.00002 | 0 | 0.9402 | 0.05978 | 0 | 0 | 0 | 0 | 0 |
| 182 | 0 | 0 | 0.936279 | 0.063721 | 0 | 0 | 0 | 0 | 0 |
| 183 | 0.00001 | 0 | 0.943363 | 0.056628 | 0 | 0 | 0 | 0 | 0 |
| 184 | 0.00002 | 0 | 0.942114 | 0.057866 | 0 | 0 | 0 | 0 | 0 |
| 185 | 0.00001 | 0 | 0.940971 | 0.059019 | 0 | 0 | 0 | 0 | 0 |
| 186 | 0.00003 | 0 | 0.941137 | 0.058833 | 0 | 0 | 0 | 0 | 0 |
| 187 | 0 | 0 | 0.944987 | 0.055013 | 0 | 0 | 0 | 0 | 0 |
| 188 | 0 | 0 | 0.940194 | 0.059806 | 0 | 0 | 0 | 0 | 0 |
| 189 | 0.00002 | 0 | 0.938762 | 0.061218 | 0 | 0 | 0 | 0 | 0 |
| 190 | 0.00001 | 0 | 0.94073 | 0.05926 | 0 | 0 | 0 | 0 | 0 |
| 191 | 0.00001 | 0 | 0.941122 | 0.058868 | 0 | 0 | 0 | 0 | 0 |
| 192 | 0.00002 | 0 | 0.941606 | 0.058374 | 0 | 0 | 0 | 0 | 0 |
| 193 | 0.00002 | 0 | 0.935862 | 0.064117 | 0 | 0 | 0 | 0 | 0 |
| 194 | 0.00001 | 0 | 0.93607 | 0.06392 | 0 | 0 | 0 | 0 | 0 |
| 195 | 0.00003 | 0 | 0.937441 | 0.062529 | 0 | 0 | 0 | 0 | 0 |
| 196 | 0.00003 | 0 | 0.935955 | 0.064015 | 0 | 0 | 0 | 0 | 0 |
| 197 | 0.00001 | 0 | 0.944888 | 0.055102 | 0 | 0 | 0 | 0 | 0 |
| 198 | 0.00001 | 0 | 0.939437 | 0.059005 | 0 | 0 | 0 | 0 | 0 |
| 199 | 0 | 0 | 0.942181 | 0.057819 | 0 | 0 | 0 | 0 | 0 |
| 200 | 0 | 0 | 0.936622 | 0.063378 | 0 | 0 | 0 | 0 | 0 |
| 201 | 0.00001 | 0 | 0.939725 | 0.060265 | 0 | 0 | 0 | 0 | 0 |
| 202 | 0.00001 | 0 | 0.940721 | 0.059269 | 0 | 0 | 0 | 0 | 0 |
| 203 | 0.00001 | 0 | 0.941527 | 0.058463 | 0 | 0 | 0 | 0 | 0 |
| 204 | 0.00001 | 0 | 0.940569 | 0.059421 | 0 | 0 | 0 | 0 | 0 |
| 205 | 0.00003 | 0 | 0.935184 | 0.064786 | 0 | 0 | 0 | 0 | 0 |
| 206 | 0.00001 | 0 | 0.93308 | 0.06691 | 0 | 0 | 0 | 0 | 0 |
| 207 | 0 | 0 | 0.941879 | 0.058121 | 0 | 0 | 0 | 0 | 0 |
| 208 | 0 | 0 | 0.943582 | 0.056418 | 0 | 0 | 0 | 0 | 0 |
| 209 | 0 | 0 | 0.936652 | 0.063348 | 0 | 0 | 0 | 0 | 0 |
| 210 | 0 | 0 | 0.941128 | 0.058872 | 0 | 0 | 0 | 0 | 0 |
| 211 | 0.00003 | 0 | 0.939731 | 0.060239 | 0 | 0 | 0 | 0 | 0 |
| 212 | 0 | 0 | 0.936061 | 0.063939 | 0 | 0 | 0 | 0 | 0 |
| 213 | 0.00001 | 0 | 0.941114 | 0.058876 | 0 | 0 | 0 | 0 | 0 |
| 214 | 0.00002 | 0 | 0.93984 | 0.06014 | 0 | 0 | 0 | 0 | 0 |
| 215 | 0 | 0 | 0.938537 | 0.061463 | 0 | 0 | 0 | 0 | 0 |
| 216 | 0 | 0 | 0.941823 | 0.058177 | 0 | 0 | 0 | 0 | 0 |
| 217 | 0.00001 | 0 | 0.93985 | 0.06014 | 0 | 0 | 0 | 0 | 0 |
| 218 | 0.00001 | 0 | 0.943945 | 0.056045 | 0 | 0 | 0 | 0 | 0 |
| 219 | 0.00003 | 0 | 0.945104 | 0.054866 | 0 | 0 | 0 | 0 | 0 |
| 220 | 0 | 0 | 0.938808 | 0.061192 | 0 | 0 | 0 | 0 | 0 |
| 221 | 0 | 0 | 0.939434 | 0.060566 | 0 | 0 | 0 | 0 | 0 |
| 222 | 0.00003 | 0 | 0.939649 | 0.060321 | 0 | 0 | 0 | 0 | 0 |
| 223 | 0 | 0 | 0.940796 | 0.059204 | 0 | 0 | 0 | 0 | 0 |
| 224 | 0.00001 | 0 | 0.939401 | 0.060589 | 0 | 0 | 0 | 0 | 0 |
| 225 | 0.00001 | 0 | 0.941172 | 0.058818 | 0 | 0 | 0 | 0 | 0 |
| 226 | 0.00003 | 0 | 0.938404 | 0.061566 | 0 | 0 | 0 | 0 | 0 |
| 227 | 0.00001 | 0 | 0.942054 | 0.057936 | 0 | 0 | 0 | 0 | 0 |
| 228 | 0.00002 | 0 | 0.940428 | 0.059552 | 0 | 0 | 0 | 0 | 0 |
| 229 | 0.00001 | 0 | 0.939138 | 0.060852 | 0 | 0 | 0 | 0 | 0 |
| 230 | 0 | 0 | 0.940844 | 0.059156 | 0 | 0 | 0 | 0 | 0 |
| 231 | 0.00001 | 0 | 0.944221 | 0.055769 | 0 | 0 | 0 | 0 | 0 |
| 232 | 0.00002 | 0 | 0.937096 | 0.062884 | 0 | 0 | 0 | 0 | 0 |
| 233 | 0.00002 | 0 | 0.938648 | 0.061332 | 0 | 0 | 0 | 0 | 0 |
| 234 | 0.00001 | 0 | 0.939739 | 0.060251 | 0 | 0 | 0 | 0 | 0 |
| 235 | 0.00001 | 0 | 0.944178 | 0.055812 | 0 | 0 | 0 | 0 | 0 |
| 236 | 0.00002 | 0 | 0.935682 | 0.064298 | 0 | 0 | 0 | 0 | 0 |
| 237 | 0 | 0 | 0.940189 | 0.059811 | 0 | 0 | 0 | 0 | 0 |
| 238 | 0 | 0 | 0.933124 | 0.066876 | 0 | 0 | 0 | 0 | 0 |
| 239 | 0.00001 | 0 | 0.936237 | 0.063753 | 0 | 0 | 0 | 0 | 0 |
| 240 | 0.00001 | 0 | 0.941109 | 0.058881 | 0 | 0 | 0 | 0 | 0 |
| 241 | 0 | 0 | 0.941861 | 0.058139 | 0 | 0 | 0 | 0 | 0 |
| 242 | 0.00001 | 0 | 0.942616 | 0.057374 | 0 | 0 | 0 | 0 | 0 |
| 243 | 0.00001 | 0 | 0.940886 | 0.059103 | 0 | 0 | 0 | 0 | 0 |
| 244 | 0 | 0 | 0.93983 | 0.06017 | 0 | 0 | 0 | 0 | 0 |
| 245 | 0 | 0 | 0.940333 | 0.059359 | 0 | 0 | 0 | 0 | 0 |
| 246 | 0 | 0 | 0.935338 | 0.064662 | 0 | 0 | 0 | 0 | 0 |
| 247 | 0.00002 | 0 | 0.939038 | 0.060942 | 0 | 0 | 0 | 0 | 0 |
| 248 | 0 | 0 | 0.941188 | 0.058812 | 0 | 0 | 0 | 0 | 0 |
| 249 | 0.00001 | 0 | 0.943537 | 0.056453 | 0 | 0 | 0 | 0 | 0 |
| 250 | 0.00001 | 0 | 0.938202 | 0.061788 | 0 | 0 | 0 | 0 | 0 |
| 251 | 0.00001 | 0 | 0.940575 | 0.059415 | 0 | 0 | 0 | 0 | 0 |
| 252 | 0.00002 | 0 | 0.940303 | 0.059677 | 0 | 0 | 0 | 0 | 0 |
| 253 | 0.00001 | 0 | 0.941044 | 0.058946 | 0 | 0 | 0 | 0 | 0 |
| 254 | 0.00003 | 0 | 0.938765 | 0.061205 | 0 | 0 | 0 | 0 | 0 |
| 255 | 0.00001 | 0 | 0.93941 | 0.06058 | 0 | 0 | 0 | 0 | 0 |
| 256 | 0.00002 | 0 | 0.937388 | 0.062592 | 0 | 0 | 0 | 0 | 0 |
| 257 | 0.00001 | 0 | 0.939663 | 0.060327 | 0 | 0 | 0 | 0 | 0 |
| 258 | 0.00001 | 0 | 0.937051 | 0.062939 | 0 | 0 | 0 | 0 | 0 |
| 259 | 0.00003 | 0 | 0.93754 | 0.06243 | 0 | 0 | 0 | 0 | 0 |
| 260 | 0.00001 | 0 | 0.942947 | 0.057043 | 0 | 0 | 0 | 0 | 0 |
| 261 | 0.00001 | 0 | 0.94191 | 0.05808 | 0 | 0 | 0 | 0 | 0 |
| 262 | 0.00002 | 0 | 0.940304 | 0.059676 | 0 | 0 | 0 | 0 | 0 |
| 263 | 0.00001 | 0 | 0.940526 | 0.059464 | 0 | 0 | 0 | 0 | 0 |
| 264 | 0.00001 | 0 | 0.935358 | 0.064632 | 0 | 0 | 0 | 0 | 0 |
| 265 | 0.00002 | 0 | 0.93918 | 0.0608 | 0 | 0 | 0 | 0 | 0 |
| 266 | 0 | 0 | 0.942269 | 0.057731 | 0 | 0 | 0 | 0 | 0 |
| 267 | 0.00002 | 0 | 0.939331 | 0.060649 | 0 | 0 | 0 | 0 | 0 |
| 268 | 0.00002 | 0 | 0.94368 | 0.0563 | 0 | 0 | 0 | 0 | 0 |
| 269 | 0 | 0 | 0.941519 | 0.058481 | 0 | 0 | 0 | 0 | 0 |
| 270 | 0 | 0 | 0.940726 | 0.059274 | 0 | 0 | 0 | 0 | 0 |
| 271 | 0.00003 | 0 | 0.940225 | 0.059745 | 0 | 0 | 0 | 0 | 0 |
| 272 | 0.00004 | 0 | 0.943039 | 0.056921 | 0 | 0 | 0 | 0 | 0 |
| 273 | 0.00001 | 0 | 0.942185 | 0.057805 | 0 | 0 | 0 | 0 | 0 |
| 274 | 0.00001 | 0 | 0.945506 | 0.054484 | 0 | 0 | 0 | 0 | 0 |
| 275 | 0.00002 | 0 | 0.940467 | 0.059513 | 0 | 0 | 0 | 0 | 0 |
| 276 | 0.00001 | 0 | 0.938277 | 0.061713 | 0 | 0 | 0 | 0 | 0 |
| 277 | 0 | 0 | 0.935383 | 0.064617 | 0 | 0 | 0 | 0 | 0 |
| 278 | 0.00001 | 0 | 0.941304 | 0.058686 | 0 | 0 | 0 | 0 | 0 |
| 279 | 0.00001 | 0 | 0.941026 | 0.058964 | 0 | 0 | 0 | 0 | 0 |
| 280 | 0 | 0 | 0.93835 | 0.06165 | 0 | 0 | 0 | 0 | 0 |
| 281 | 0.00001 | 0 | 0.938676 | 0.061314 | 0 | 0 | 0 | 0 | 0 |
| 282 | 0.00003 | 0 | 0.939847 | 0.060123 | 0 | 0 | 0 | 0 | 0 |
| 283 | 0.00001 | 0 | 0.94168 | 0.05831 | 0 | 0 | 0 | 0 | 0 |
| 284 | 0.00001 | 0 | 0.942679 | 0.057311 | 0 | 0 | 0 | 0 | 0 |
| 285 | 0 | 0 | 0.939992 | 0.060008 | 0 | 0 | 0 | 0 | 0 |
| 286 | 0.00003 | 0 | 0.944538 | 0.055432 | 0 | 0 | 0 | 0 | 0 |
| 287 | 0.00006 | 0 | 0.937857 | 0.062083 | 0 | 0 | 0 | 0 | 0 |
| 288 | 0 | 0 | 0.938871 | 0.061129 | 0 | 0 | 0 | 0 | 0 |
| 289 | 0.00001 | 0 | 0.941762 | 0.058228 | 0 | 0 | 0 | 0 | 0 |
| 290 | 0 | 0 | 0.941099 | 0.058901 | 0 | 0 | 0 | 0 | 0 |
| 291 | 0 | 0 | 0.938314 | 0.061686 | 0 | 0 | 0 | 0 | 0 |
| 292 | 0.00001 | 0 | 0.93812 | 0.06187 | 0 | 0 | 0 | 0 | 0 |
| 293 | 0.00001 | 0 | 0.938395 | 0.061595 | 0 | 0 | 0 | 0 | 0 |
| 294 | 0.00002 | 0 | 0.93869 | 0.06129 | 0 | 0 | 0 | 0 | 0 |
| 295 | 0.00001 | 0 | 0.942138 | 0.057852 | 0 | 0 | 0 | 0 | 0 |
| 296 | 0 | 0 | 0.940905 | 0.059095 | 0 | 0 | 0 | 0 | 0 |
| 297 | 0 | 0 | 0.93839 | 0.06161 | 0 | 0 | 0 | 0 | 0 |
| 298 | 0.00001 | 0 | 0.940118 | 0.059872 | 0 | 0 | 0 | 0 | 0 |
| 299 | 0.00003 | 0 | 0.935047 | 0.064923 | 0 | 0 | 0 | 0 | 0 |
| 300 | 0.00001 | 0 | 0.94121 | 0.05878 | 0 | 0 | 0 | 0 | 0 |
| 301 | 0.00003 | 0 | 0.939988 | 0.059982 | 0 | 0 | 0 | 0 | 0 |
| 302 | 0 | 0 | 0.938296 | 0.061704 | 0 | 0 | 0 | 0 | 0 |
| 303 | 0.00002 | 0 | 0.940157 | 0.059823 | 0 | 0 | 0 | 0 | 0 |
| 304 | 0 | 0 | 0.938499 | 0.061501 | 0 | 0 | 0 | 0 | 0 |
| 305 | 0.00003 | 0 | 0.941087 | 0.058883 | 0 | 0 | 0 | 0 | 0 |
| 306 | 0 | 0 | 0.942879 | 0.057121 | 0 | 0 | 0 | 0 | 0 |
| 307 | 0.00002 | 0 | 0.941181 | 0.058799 | 0 | 0 | 0 | 0 | 0 |
| 308 | 0.00001 | 0 | 0.945067 | 0.054923 | 0 | 0 | 0 | 0 | 0 |
| 309 | 0.00002 | 0 | 0.943074 | 0.056906 | 0 | 0 | 0 | 0 | 0 |
| 310 | 0 | 0 | 0.937677 | 0.062323 | 0 | 0 | 0 | 0 | 0 |
| 311 | 0 | 0 | 0.941148 | 0.058852 | 0 | 0 | 0 | 0 | 0 |
| 312 | 0.00001 | 0 | 0.93767 | 0.06232 | 0 | 0 | 0 | 0 | 0 |
| 313 | 0 | 0 | 0.940529 | 0.059471 | 0 | 0 | 0 | 0 | 0 |
| 314 | 0.00002 | 0 | 0.940998 | 0.058982 | 0 | 0 | 0 | 0 | 0 |
| 315 | 0.00002 | 0 | 0.938208 | 0.061772 | 0 | 0 | 0 | 0 | 0 |
| 316 | 0.00002 | 0 | 0.937388 | 0.062592 | 0 | 0 | 0 | 0 | 0 |
| 317 | 0.00001 | 0 | 0.937676 | 0.062314 | 0 | 0 | 0 | 0 | 0 |
| 318 | 0.00003 | 0 | 0.937425 | 0.061934 | 0 | 0 | 0 | 0 | 0 |
| 319 | 0.00003 | 0 | 0.941192 | 0.058778 | 0 | 0 | 0 | 0 | 0 |
| 320 | 0.00001 | 0 | 0.940725 | 0.059265 | 0 | 0 | 0 | 0 | 0 |
| 321 | 0 | 0 | 0.939716 | 0.060284 | 0 | 0 | 0 | 0 | 0 |
| 322 | 0.00001 | 0 | 0.937501 | 0.062489 | 0 | 0 | 0 | 0 | 0 |
| 323 | 0.00001 | 0 | 0.941766 | 0.058224 | 0 | 0 | 0 | 0 | 0 |
| 324 | 0.00001 | 0 | 0.942939 | 0.057051 | 0 | 0 | 0 | 0 | 0 |
| 325 | 0.00003 | 0 | 0.940579 | 0.059391 | 0 | 0 | 0 | 0 | 0 |
| 326 | 0.00001 | 0.00001 | 0.941253 | 0.058727 | 0 | 0 | 0 | 0 | 0 |
| 327 | 0.00001 | 0 | 0.938796 | 0.061194 | 0 | 0 | 0 | 0 | 0 |
| 328 | 0.00001 | 0 | 0.938637 | 0.061353 | 0 | 0 | 0 | 0 | 0 |
| 329 | 0 | 0.00002 | 0.939365 | 0.060615 | 0 | 0 | 0 | 0 | 0 |
| 330 | 0 | 0 | 0.941786 | 0.058214 | 0 | 0 | 0 | 0 | 0 |
| 331 | 0.00001 | 0 | 0.939532 | 0.060458 | 0 | 0 | 0 | 0 | 0 |
| 332 | 0.00002 | 0 | 0.939997 | 0.059983 | 0 | 0 | 0 | 0 | 0 |
| 333 | 0.00002 | 0 | 0.937057 | 0.062923 | 0 | 0 | 0 | 0 | 0 |
| 334 | 0.00002 | 0.00001 | 0.941448 | 0.058522 | 0 | 0 | 0 | 0 | 0 |
| 335 | 0 | 0 | 0.941504 | 0.058496 | 0 | 0 | 0 | 0 | 0 |
| 336 | 0.00001 | 0 | 0.935876 | 0.064114 | 0 | 0 | 0 | 0 | 0 |
| 337 | 0 | 0 | 0.942032 | 0.057968 | 0 | 0 | 0 | 0 | 0 |
| 338 | 0.00001 | 0 | 0.940421 | 0.059569 | 0 | 0 | 0 | 0 | 0 |
| 339 | 0.00002 | 0 | 0.937793 | 0.062187 | 0 | 0 | 0 | 0 | 0 |
| 340 | 0.00002 | 0 | 0.937686 | 0.062294 | 0 | 0 | 0 | 0 | 0 |
| 341 | 0.00002 | 0 | 0.937716 | 0.062264 | 0 | 0 | 0 | 0 | 0 |
| 342 | 0.00001 | 0 | 0.936452 | 0.063538 | 0 | 0 | 0 | 0 | 0 |
| 343 | 0 | 0 | 0.937974 | 0.062026 | 0 | 0 | 0 | 0 | 0 |
| 344 | 0 | 0 | 0.937246 | 0.062754 | 0 | 0 | 0 | 0 | 0 |
| 345 | 0.00001 | 0 | 0.942586 | 0.057404 | 0 | 0 | 0 | 0 | 0 |
| 346 | 0.00001 | 0 | 0.945614 | 0.054376 | 0 | 0 | 0 | 0 | 0 |
| 347 | 0.00002 | 0 | 0.93391 | 0.06607 | 0 | 0 | 0 | 0 | 0 |
| 348 | 0.00001 | 0 | 0.939667 | 0.060323 | 0 | 0 | 0 | 0 | 0 |
| 349 | 0.00001 | 0 | 0.940835 | 0.059155 | 0 | 0 | 0 | 0 | 0 |
| 350 | 0.00001 | 0 | 0.941622 | 0.058368 | 0 | 0 | 0 | 0 | 0 |
| 351 | 0.00001 | 0 | 0.941488 | 0.058502 | 0 | 0 | 0 | 0 | 0 |
| 352 | 0.00003 | 0 | 0.943011 | 0.05696 | 0 | 0 | 0 | 0 | 0 |
| 353 | 0 | 0 | 0.940446 | 0.059554 | 0 | 0 | 0 | 0 | 0 |
| 354 | 0 | 0 | 0.940458 | 0.059542 | 0 | 0 | 0 | 0 | 0 |
| 355 | 0 | 0 | 0.941531 | 0.058469 | 0 | 0 | 0 | 0 | 0 |
| 356 | 0.00001 | 0 | 0.939428 | 0.060562 | 0 | 0 | 0 | 0 | 0 |
| 357 | 0 | 0 | 0.936726 | 0.063274 | 0 | 0 | 0 | 0 | 0 |
| 358 | 0.00001 | 0 | 0.938991 | 0.060999 | 0 | 0 | 0 | 0 | 0 |
| 359 | 0.00003 | 0 | 0.938796 | 0.061174 | 0 | 0 | 0 | 0 | 0 |
| 360 | 0.00001 | 0 | 0.939291 | 0.060699 | 0 | 0 | 0 | 0 | 0 |
| 361 | 0 | 0 | 0.938699 | 0.061301 | 0 | 0 | 0 | 0 | 0 |
| 362 | 0 | 0 | 0.944109 | 0.055891 | 0 | 0 | 0 | 0 | 0 |
| 363 | 0.00001 | 0.00002 | 0.941575 | 0.058395 | 0 | 0 | 0 | 0 | 0 |
| 364 | 0 | 0 | 0.939645 | 0.060355 | 0 | 0 | 0 | 0 | 0 |
| 365 | 0.00001 | 0 | 0.941076 | 0.058914 | 0 | 0 | 0 | 0 | 0 |
| 366 | 0 | 0 | 0.939243 | 0.060757 | 0 | 0 | 0 | 0 | 0 |
| 367 | 0.00001 | 0 | 0.939616 | 0.060374 | 0 | 0 | 0 | 0 | 0 |
| 368 | 0.00002 | 0 | 0.942135 | 0.057845 | 0 | 0 | 0 | 0 | 0 |
| 369 | 0.00002 | 0 | 0.93754 | 0.06244 | 0 | 0 | 0 | 0 | 0 |
| 370 | 0.00002 | 0 | 0.939679 | 0.060301 | 0 | 0 | 0 | 0 | 0 |
| 371 | 0 | 0 | 0.939752 | 0.059225 | 0 | 0 | 0 | 0 | 0 |
| 372 | 0.00001 | 0 | 0.935915 | 0.064075 | 0 | 0 | 0 | 0 | 0 |
| 373 | 0 | 0 | 0.935736 | 0.064264 | 0 | 0 | 0 | 0 | 0 |
| 374 | 0.00001 | 0 | 0.942656 | 0.057334 | 0 | 0 | 0 | 0 | 0 |
| 375 | 0.00001 | 0 | 0.936819 | 0.063171 | 0 | 0 | 0 | 0 | 0 |
| 376 | 0.00003 | 0.00002 | 0.936366 | 0.063583 | 0 | 0 | 0 | 0 | 0 |
| 377 | 0.00001 | 0 | 0.935085 | 0.064905 | 0 | 0 | 0 | 0 | 0 |
| 378 | 0.00001 | 0 | 0.942338 | 0.057652 | 0 | 0 | 0 | 0 | 0 |
| 379 | 0 | 0 | 0.941697 | 0.058303 | 0 | 0 | 0 | 0 | 0 |
| 380 | 0.00001 | 0 | 0.934292 | 0.065698 | 0 | 0 | 0 | 0 | 0 |
| 381 | 0.00001 | 0 | 0.939525 | 0.060465 | 0 | 0 | 0 | 0 | 0 |
| 382 | 0 | 0 | 0.937151 | 0.062849 | 0 | 0 | 0 | 0 | 0 |
| 383 | 0.00003 | 0 | 0.937033 | 0.062938 | 0 | 0 | 0 | 0 | 0 |
| 384 | 0.00002 | 0 | 0.934346 | 0.065634 | 0 | 0 | 0 | 0 | 0 |
| 385 | 0 | 0 | 0.940836 | 0.059164 | 0 | 0 | 0 | 0 | 0 |
| 386 | 0 | 0 | 0.941162 | 0.058838 | 0 | 0 | 0 | 0 | 0 |
| 387 | 0.00002 | 0 | 0.941343 | 0.058637 | 0 | 0 | 0 | 0 | 0 |
| 388 | 0.00002 | 0 | 0.940469 | 0.059511 | 0 | 0 | 0 | 0 | 0 |
| 389 | 0 | 0.00001 | 0.941171 | 0.058819 | 0 | 0 | 0 | 0 | 0 |
| 390 | 0 | 0 | 0.940173 | 0.059827 | 0 | 0 | 0 | 0 | 0 |
| 391 | 0.00001 | 0 | 0.938858 | 0.061132 | 0 | 0 | 0 | 0 | 0 |
| 392 | 0 | 0 | 0.939987 | 0.060013 | 0 | 0 | 0 | 0 | 0 |
| 393 | 0.00001 | 0 | 0.937778 | 0.062212 | 0 | 0 | 0 | 0 | 0 |
| 394 | 0 | 0 | 0.94288 | 0.05712 | 0 | 0 | 0 | 0 | 0 |
| 395 | 0.00001 | 0 | 0.942838 | 0.057152 | 0 | 0 | 0 | 0 | 0 |
| 396 | 0.00001 | 0 | 0.939561 | 0.060429 | 0 | 0 | 0 | 0 | 0 |
| 397 | 0.00001 | 0 | 0.939509 | 0.060481 | 0 | 0 | 0 | 0 | 0 |
| 398 | 0.00001 | 0 | 0.944439 | 0.055551 | 0 | 0 | 0 | 0 | 0 |
| 399 | 0.00003 | 0 | 0.942169 | 0.057801 | 0 | 0 | 0 | 0 | 0 |
| 400 | 0.00001 | 0.00002 | 0.942196 | 0.057774 | 0 | 0 | 0 | 0 | 0 |
| 401 | 0.00001 | 0 | 0.939481 | 0.060509 | 0 | 0 | 0 | 0 | 0 |
| 402 | 0 | 0 | 0.941135 | 0.058865 | 0 | 0 | 0 | 0 | 0 |
| 403 | 0.00003 | 0 | 0.940204 | 0.059766 | 0 | 0 | 0 | 0 | 0 |
| 404 | 0.00002 | 0 | 0.939023 | 0.060957 | 0 | 0 | 0 | 0 | 0 |
| 405 | 0 | 0 | 0.941317 | 0.058683 | 0 | 0 | 0 | 0 | 0 |
| 406 | 0 | 0 | 0.940419 | 0.059581 | 0 | 0 | 0 | 0 | 0 |
| 407 | 0 | 0 | 0.938162 | 0.061838 | 0 | 0 | 0 | 0 | 0 |
| 408 | 0.00001 | 0.00001 | 0.939299 | 0.060681 | 0 | 0 | 0 | 0 | 0 |
| 409 | 0.00001 | 0 | 0.937312 | 0.062678 | 0 | 0 | 0 | 0 | 0 |
| 410 | 0 | 0 | 0.934607 | 0.065393 | 0 | 0 | 0 | 0 | 0 |
| 411 | 0.00001 | 0 | 0.940475 | 0.059515 | 0 | 0 | 0 | 0 | 0 |
| 412 | 0.00001 | 0 | 0.940677 | 0.059313 | 0 | 0 | 0 | 0 | 0 |
| 413 | 0.00002 | 0 | 0.935836 | 0.064144 | 0 | 0 | 0 | 0 | 0 |
| 414 | 0.00002 | 0 | 0.94274 | 0.05724 | 0 | 0 | 0 | 0 | 0 |
| 415 | 0 | 0 | 0.937615 | 0.062385 | 0 | 0 | 0 | 0 | 0 |
| 416 | 0 | 0 | 0.941073 | 0.058927 | 0 | 0 | 0 | 0 | 0 |
| 417 | 0.000041 | 0 | 0.942426 | 0.057534 | 0 | 0 | 0 | 0 | 0 |
| 418 | 0 | 0 | 0.94026 | 0.05974 | 0 | 0 | 0 | 0 | 0 |
| 419 | 0 | 0 | 0.939151 | 0.060849 | 0 | 0 | 0 | 0 | 0 |
| 420 | 0 | 0 | 0.945404 | 0.054596 | 0 | 0 | 0 | 0 | 0 |
| 421 | 0.00001 | 0 | 0.937016 | 0.062974 | 0 | 0 | 0 | 0 | 0 |
| 422 | 0 | 0.00001 | 0.938507 | 0.061483 | 0 | 0 | 0 | 0 | 0 |
| 423 | 0 | 0 | 0.942283 | 0.057717 | 0 | 0 | 0 | 0 | 0 |
| 424 | 0.00001 | 0.00002 | 0.943324 | 0.056647 | 0 | 0 | 0 | 0 | 0 |
| 425 | 0.00001 | 0 | 0.938735 | 0.061255 | 0 | 0 | 0 | 0 | 0 |
| 426 | 0.00001 | 0 | 0.93948 | 0.06051 | 0 | 0 | 0 | 0 | 0 |
| 427 | 0.00003 | 0 | 0.941487 | 0.058483 | 0 | 0 | 0 | 0 | 0 |
| 428 | 0 | 0 | 0.942049 | 0.057951 | 0 | 0 | 0 | 0 | 0 |
| 429 | 0.00002 | 0 | 0.941515 | 0.058465 | 0 | 0 | 0 | 0 | 0 |
| 430 | 0.00003 | 0 | 0.938102 | 0.061868 | 0 | 0 | 0 | 0 | 0 |
| 431 | 0.00002 | 0 | 0.942642 | 0.057338 | 0 | 0 | 0 | 0 | 0 |
| 432 | 0.00001 | 0.00002 | 0.937988 | 0.061982 | 0 | 0 | 0 | 0 | 0 |
| 433 | 0.00001 | 0 | 0.942126 | 0.057864 | 0 | 0 | 0 | 0 | 0 |
| 434 | 0 | 0.00001 | 0.941587 | 0.058403 | 0 | 0 | 0 | 0 | 0 |
| 435 | 0 | 0 | 0.941326 | 0.058674 | 0 | 0 | 0 | 0 | 0 |
| 436 | 0.00003 | 0 | 0.93478 | 0.06519 | 0 | 0 | 0 | 0 | 0 |
| 437 | 0.00002 | 0 | 0.939763 | 0.060217 | 0 | 0 | 0 | 0 | 0 |
| 438 | 0.00001 | 0 | 0.941333 | 0.058657 | 0 | 0 | 0 | 0 | 0 |
| 439 | 0.00001 | 0 | 0.941572 | 0.058418 | 0 | 0 | 0 | 0 | 0 |
| 440 | 0.00002 | 0 | 0.941018 | 0.058962 | 0 | 0 | 0 | 0 | 0 |
| 441 | 0.00004 | 0.00001 | 0.93987 | 0.06008 | 0 | 0 | 0 | 0 | 0 |
| 442 | 0 | 0 | 0.939799 | 0.060201 | 0 | 0 | 0 | 0 | 0 |
| 443 | 0.00001 | 0 | 0.943282 | 0.056708 | 0 | 0 | 0 | 0 | 0 |
| 444 | 0 | 0 | 0.93998 | 0.06002 | 0 | 0 | 0 | 0 | 0 |
| 445 | 0.00001 | 0 | 0.939253 | 0.060737 | 0 | 0 | 0 | 0 | 0 |
| 446 | 0.00001 | 0 | 0.937762 | 0.062228 | 0 | 0 | 0 | 0 | 0 |
| 447 | 0.00002 | 0 | 0.942195 | 0.057785 | 0 | 0 | 0 | 0 | 0 |
| 448 | 0 | 0 | 0.940691 | 0.059309 | 0 | 0 | 0 | 0 | 0 |
| 449 | 0.00001 | 0 | 0.93988 | 0.06011 | 0 | 0 | 0 | 0 | 0 |
| 450 | 0.00002 | 0 | 0.939399 | 0.060581 | 0 | 0 | 0 | 0 | 0 |
| 451 | 0.00001 | 0 | 0.942967 | 0.057023 | 0 | 0 | 0 | 0 | 0 |
| 452 | 0.00002 | 0 | 0.939517 | 0.060463 | 0 | 0 | 0 | 0 | 0 |
| 453 | 0.00002 | 0 | 0.939971 | 0.060008 | 0 | 0 | 0 | 0 | 0 |
| 454 | 0.00001 | 0 | 0.938829 | 0.061161 | 0 | 0 | 0 | 0 | 0 |
| 455 | 0.00001 | 0 | 0.942405 | 0.057585 | 0 | 0 | 0 | 0 | 0 |
| 456 | 0 | 0 | 0.939803 | 0.060197 | 0 | 0 | 0 | 0 | 0 |
| 457 | 0.00003 | 0 | 0.936564 | 0.063406 | 0 | 0 | 0 | 0 | 0 |
| 458 | 0 | 0 | 0.941337 | 0.058663 | 0 | 0 | 0 | 0 | 0 |
| 459 | 0.00002 | 0 | 0.934524 | 0.065456 | 0 | 0 | 0 | 0 | 0 |
| 460 | 0 | 0 | 0.934754 | 0.065246 | 0 | 0 | 0 | 0 | 0 |
| 461 | 0.00001 | 0 | 0.943744 | 0.056246 | 0 | 0 | 0 | 0 | 0 |
| 462 | 0 | 0 | 0.94367 | 0.05633 | 0 | 0 | 0 | 0 | 0 |
| 463 | 0.00002 | 0 | 0.935466 | 0.064514 | 0 | 0 | 0 | 0 | 0 |
| 464 | 0.00001 | 0 | 0.935097 | 0.062462 | 0 | 0 | 0 | 0 | 0 |
| 465 | 0 | 0 | 0.941741 | 0.058259 | 0 | 0 | 0 | 0 | 0 |
| 466 | 0.00001 | 0 | 0.938299 | 0.061691 | 0 | 0 | 0 | 0 | 0 |
| 467 | 0 | 0 | 0.935889 | 0.064111 | 0 | 0 | 0 | 0 | 0 |
| 468 | 0.00002 | 0 | 0.94213 | 0.05785 | 0 | 0 | 0 | 0 | 0 |
| 469 | 0 | 0 | 0.938308 | 0.061692 | 0 | 0 | 0 | 0 | 0 |
| 470 | 0.00001 | 0 | 0.940965 | 0.059025 | 0 | 0 | 0 | 0 | 0 |
| 471 | 0.00002 | 0 | 0.937322 | 0.062658 | 0 | 0 | 0 | 0 | 0 |
| 472 | 0.00001 | 0 | 0.937841 | 0.062149 | 0 | 0 | 0 | 0 | 0 |
| 473 | 0.00003 | 0 | 0.943171 | 0.056799 | 0 | 0 | 0 | 0 | 0 |
| 474 | 0.00001 | 0 | 0.941596 | 0.058394 | 0 | 0 | 0 | 0 | 0 |
| 475 | 0.00002 | 0 | 0.942494 | 0.057486 | 0 | 0 | 0 | 0 | 0 |
| 476 | 0.00001 | 0.00001 | 0.932095 | 0.067885 | 0 | 0 | 0 | 0 | 0 |
| 477 | 0 | 0 | 0.941104 | 0.058896 | 0 | 0 | 0 | 0 | 0 |
| 478 | 0 | 0 | 0.938988 | 0.061012 | 0 | 0 | 0 | 0 | 0 |
| 479 | 0.00001 | 0 | 0.938714 | 0.061276 | 0 | 0 | 0 | 0 | 0 |
| 480 | 0.00001 | 0 | 0.936084 | 0.063906 | 0 | 0 | 0 | 0 | 0 |
| 481 | 0.00003 | 0 | 0.942996 | 0.056974 | 0 | 0 | 0 | 0 | 0 |
| 482 | 0.00002 | 0 | 0.936582 | 0.063398 | 0 | 0 | 0 | 0 | 0 |
| 483 | 0 | 0 | 0.940885 | 0.059115 | 0 | 0 | 0 | 0 | 0 |
| 484 | 0.00001 | 0 | 0.937815 | 0.062175 | 0 | 0 | 0 | 0 | 0 |
| 485 | 0.00001 | 0 | 0.943389 | 0.056601 | 0 | 0 | 0 | 0 | 0 |
| 486 | 0.00002 | 0 | 0.937001 | 0.062979 | 0 | 0 | 0 | 0 | 0 |
| 487 | 0 | 0 | 0.937593 | 0.062407 | 0 | 0 | 0 | 0 | 0 |
| 488 | 0.00001 | 0 | 0.937443 | 0.062547 | 0 | 0 | 0 | 0 | 0 |
| 489 | 0.00001 | 0 | 0.939322 | 0.060668 | 0 | 0 | 0 | 0 | 0 |
| 490 | 0.00002 | 0 | 0.942311 | 0.057669 | 0 | 0 | 0 | 0 | 0 |
| 491 | 0.00001 | 0 | 0.940005 | 0.059985 | 0 | 0 | 0 | 0 | 0 |
| 492 | 0 | 0 | 0.939419 | 0.060581 | 0 | 0 | 0 | 0 | 0 |
| 493 | 0.00002 | 0 | 0.938472 | 0.061508 | 0 | 0 | 0 | 0 | 0 |
| 494 | 0 | 0 | 0.940041 | 0.059959 | 0 | 0 | 0 | 0 | 0 |
| 495 | 0.00001 | 0 | 0.938649 | 0.061341 | 0 | 0 | 0 | 0 | 0 |
| 496 | 0.00003 | 0 | 0.937818 | 0.061822 | 0 | 0 | 0 | 0 | 0 |
| 497 | 0.00001 | 0 | 0.942205 | 0.057785 | 0 | 0 | 0 | 0 | 0 |
| 498 | 0 | 0 | 0.944118 | 0.055882 | 0 | 0 | 0 | 0 | 0 |
| 499 | 0.00001 | 0 | 0.937187 | 0.062803 | 0 | 0 | 0 | 0 | 0 |
| 500 | 0 | 0 | 0.940501 | 0.059499 | 0 | 0 | 0 | 0 | 0 |
| 501 | 0.00001 | 0 | 0.938712 | 0.061278 | 0 | 0 | 0 | 0 | 0 |
| 502 | 0.00002 | 0 | 0.939603 | 0.060377 | 0 | 0 | 0 | 0 | 0 |
| 503 | 0 | 0 | 0.935925 | 0.064075 | 0 | 0 | 0 | 0 | 0 |
| 504 | 0.00001 | 0 | 0.941982 | 0.058008 | 0 | 0 | 0 | 0 | 0 |
| 505 | 0.00001 | 0 | 0.937164 | 0.062696 | 0 | 0 | 0 | 0 | 0 |
| 506 | 0.00003 | 0 | 0.937255 | 0.062715 | 0 | 0 | 0 | 0 | 0 |
| 507 | 0.00001 | 0 | 0.940198 | 0.059792 | 0 | 0 | 0 | 0 | 0 |
| 508 | 0.00003 | 0 | 0.938449 | 0.061521 | 0 | 0 | 0 | 0 | 0 |
| 509 | 0.00001 | 0 | 0.934706 | 0.065284 | 0 | 0 | 0 | 0 | 0 |
| 510 | 0.00002 | 0 | 0.93963 | 0.06035 | 0 | 0 | 0 | 0 | 0 |
| 511 | 0.00001 | 0 | 0.939924 | 0.060066 | 0 | 0 | 0 | 0 | 0 |
| 512 | 0.00001 | 0 | 0.941093 | 0.058898 | 0 | 0 | 0 | 0 | 0 |
| 513 | 0.00001 | 0 | 0.937568 | 0.062422 | 0 | 0 | 0 | 0 | 0 |
| 514 | 0.00001 | 0 | 0.938748 | 0.061242 | 0 | 0 | 0 | 0 | 0 |
| 515 | 0.00001 | 0 | 0.939839 | 0.060151 | 0 | 0 | 0 | 0 | 0 |
| 516 | 0 | 0 | 0.936683 | 0.063317 | 0 | 0 | 0 | 0 | 0 |
| 517 | 0 | 0 | 0.938867 | 0.061133 | 0 | 0 | 0 | 0 | 0 |
| 518 | 0 | 0 | 0.939383 | 0.060617 | 0 | 0 | 0 | 0 | 0 |
| 519 | 0.00001 | 0 | 0.937442 | 0.062548 | 0 | 0 | 0 | 0 | 0 |
| 520 | 0.00005 | 0 | 0.93961 | 0.06034 | 0 | 0 | 0 | 0 | 0 |
| 521 | 0.00003 | 0 | 0.939525 | 0.060445 | 0 | 0 | 0 | 0 | 0 |
| 522 | 0.00001 | 0 | 0.942249 | 0.057741 | 0 | 0 | 0 | 0 | 0 |
| 523 | 0 | 0 | 0.938673 | 0.061327 | 0 | 0 | 0 | 0 | 0 |
| 524 | 0.00002 | 0 | 0.939273 | 0.060707 | 0 | 0 | 0 | 0 | 0 |
| 525 | 0 | 0 | 0.937837 | 0.062163 | 0 | 0 | 0 | 0 | 0 |
| 526 | 0 | 0.00001 | 0.93788 | 0.06211 | 0 | 0 | 0 | 0 | 0 |
| 527 | 0.00001 | 0 | 0.938613 | 0.061377 | 0 | 0 | 0 | 0 | 0 |
| 528 | 0 | 0 | 0.938779 | 0.061221 | 0 | 0 | 0 | 0 | 0 |
| 529 | 0.00001 | 0 | 0.939093 | 0.060897 | 0 | 0 | 0 | 0 | 0 |
| 530 | 0.00001 | 0 | 0.937393 | 0.062597 | 0 | 0 | 0 | 0 | 0 |
| 531 | 0 | 0 | 0.93788 | 0.06212 | 0 | 0 | 0 | 0 | 0 |
| 532 | 0.00003 | 0 | 0.944742 | 0.055228 | 0 | 0 | 0 | 0 | 0 |
| 533 | 0.00001 | 0 | 0.937558 | 0.062432 | 0 | 0 | 0 | 0 | 0 |
| 534 | 0.00002 | 0 | 0.936779 | 0.063201 | 0 | 0 | 0 | 0 | 0 |
| 535 | 0.00002 | 0 | 0.941755 | 0.058225 | 0 | 0 | 0 | 0 | 0 |
| 536 | 0 | 0 | 0.942863 | 0.057137 | 0 | 0 | 0 | 0 | 0 |
| 537 | 0.00001 | 0 | 0.937817 | 0.062173 | 0 | 0 | 0 | 0 | 0 |
| 538 | 0 | 0 | 0.938415 | 0.061585 | 0 | 0 | 0 | 0 | 0 |
| 539 | 0.00001 | 0 | 0.935644 | 0.064346 | 0 | 0 | 0 | 0 | 0 |
| 540 | 0 | 0 | 0.936538 | 0.063462 | 0 | 0 | 0 | 0 | 0 |
| 541 | 0.00001 | 0.00001 | 0.941474 | 0.058506 | 0 | 0 | 0 | 0 | 0 |
| 542 | 0.00002 | 0 | 0.939663 | 0.060317 | 0 | 0 | 0 | 0 | 0 |
| 543 | 0 | 0 | 0.94221 | 0.05779 | 0 | 0 | 0 | 0 | 0 |
| 544 | 0.00001 | 0 | 0.944356 | 0.055634 | 0 | 0 | 0 | 0 | 0 |
| 545 | 0.00001 | 0 | 0.937769 | 0.062221 | 0 | 0 | 0 | 0 | 0 |
| 546 | 0.00002 | 0 | 0.944153 | 0.055828 | 0 | 0 | 0 | 0 | 0 |
| 547 | 0.00001 | 0 | 0.939051 | 0.060939 | 0 | 0 | 0 | 0 | 0 |
| 548 | 0.00001 | 0 | 0.941832 | 0.058158 | 0 | 0 | 0 | 0 | 0 |
| 549 | 0 | 0 | 0.935903 | 0.064097 | 0 | 0 | 0 | 0 | 0 |
| 550 | 0.00002 | 0 | 0.937778 | 0.062202 | 0 | 0 | 0 | 0 | 0 |
| 551 | 0.00001 | 0 | 0.94079 | 0.0592 | 0 | 0 | 0 | 0 | 0 |
| 552 | 0 | 0 | 0.93938 | 0.06062 | 0 | 0 | 0 | 0 | 0 |
| 553 | 0.00002 | 0 | 0.937273 | 0.062707 | 0 | 0 | 0 | 0 | 0 |
| 554 | 0.00002 | 0 | 0.94328 | 0.0567 | 0 | 0 | 0 | 0 | 0 |
| 555 | 0.00001 | 0 | 0.937247 | 0.062743 | 0 | 0 | 0 | 0 | 0 |
| 556 | 0.00002 | 0 | 0.941481 | 0.058499 | 0 | 0 | 0 | 0 | 0 |
| 557 | 0.00001 | 0 | 0.938515 | 0.061475 | 0 | 0 | 0 | 0 | 0 |
| 558 | 0 | 0 | 0.939355 | 0.060645 | 0 | 0 | 0 | 0 | 0 |
| 559 | 0 | 0 | 0.943067 | 0.056933 | 0 | 0 | 0 | 0 | 0 |
| 560 | 0.00002 | 0 | 0.943904 | 0.056076 | 0 | 0 | 0 | 0 | 0 |
| 561 | 0.00001 | 0 | 0.936848 | 0.063142 | 0 | 0 | 0 | 0 | 0 |
| 562 | 0.00001 | 0 | 0.937237 | 0.062753 | 0 | 0 | 0 | 0 | 0 |
| 563 | 0 | 0 | 0.938176 | 0.061824 | 0 | 0 | 0 | 0 | 0 |
| 564 | 0 | 0 | 0.939899 | 0.060101 | 0 | 0 | 0 | 0 | 0 |
| 565 | 0.00001 | 0 | 0.937774 | 0.062216 | 0 | 0 | 0 | 0 | 0 |
| 566 | 0.00002 | 0 | 0.941436 | 0.058544 | 0 | 0 | 0 | 0 | 0 |
| 567 | 0.00001 | 0 | 0.940926 | 0.059064 | 0 | 0 | 0 | 0 | 0 |
| 568 | 0.00001 | 0 | 0.937382 | 0.062608 | 0 | 0 | 0 | 0 | 0 |
| 569 | 0.00002 | 0 | 0.941818 | 0.058162 | 0 | 0 | 0 | 0 | 0 |
| 570 | 0.00001 | 0 | 0.935858 | 0.064132 | 0 | 0 | 0 | 0 | 0 |
| 571 | 0.00001 | 0 | 0.936745 | 0.063245 | 0 | 0 | 0 | 0 | 0 |
| 572 | 0.00003 | 0 | 0.942633 | 0.057337 | 0 | 0 | 0 | 0 | 0 |
| 573 | 0.00001 | 0 | 0.943153 | 0.056837 | 0 | 0 | 0 | 0 | 0 |
| 574 | 0 | 0 | 0.940295 | 0.059705 | 0 | 0 | 0 | 0 | 0 |
| 575 | 0.00001 | 0 | 0.939572 | 0.060418 | 0 | 0 | 0 | 0 | 0 |
| 576 | 0.00001 | 0 | 0.943951 | 0.056039 | 0 | 0 | 0 | 0 | 0 |
| 577 | 0.00001 | 0 | 0.938705 | 0.061285 | 0 | 0 | 0 | 0 | 0 |
| 578 | 0.00001 | 0 | 0.941836 | 0.058154 | 0 | 0 | 0 | 0 | 0 |
| 579 | 0.00005 | 0 | 0.936874 | 0.063076 | 0 | 0 | 0 | 0 | 0 |
| 580 | 0.00001 | 0 | 0.9394 | 0.06059 | 0 | 0 | 0 | 0 | 0 |
| 581 | 0.00001 | 0 | 0.937426 | 0.062564 | 0 | 0 | 0 | 0 | 0 |
| 582 | 0.00001 | 0 | 0.939313 | 0.060677 | 0 | 0 | 0 | 0 | 0 |
| 583 | 0.00001 | 0.00001 | 0.935508 | 0.064472 | 0 | 0 | 0 | 0 | 0 |
| 584 | 0.00001 | 0 | 0.937358 | 0.062632 | 0 | 0 | 0 | 0 | 0 |
| 585 | 0 | 0 | 0.93699 | 0.06301 | 0 | 0 | 0 | 0 | 0 |
| 586 | 0 | 0 | 0.938715 | 0.061285 | 0 | 0 | 0 | 0 | 0 |
| 587 | 0.00004 | 0 | 0.940243 | 0.059717 | 0 | 0 | 0 | 0 | 0 |
| 588 | 0.00001 | 0 | 0.942763 | 0.057227 | 0 | 0 | 0 | 0 | 0 |
| 589 | 0.00001 | 0 | 0.940536 | 0.059454 | 0 | 0 | 0 | 0 | 0 |
| 590 | 0.00002 | 0 | 0.936079 | 0.063901 | 0 | 0 | 0 | 0 | 0 |
| 591 | 0 | 0 | 0.93473 | 0.06527 | 0 | 0 | 0 | 0 | 0 |
| 592 | 0 | 0 | 0.938089 | 0.061911 | 0 | 0 | 0 | 0 | 0 |
| 593 | 0 | 0 | 0.940609 | 0.059391 | 0 | 0 | 0 | 0 | 0 |
| 594 | 0.00002 | 0 | 0.93613 | 0.06385 | 0 | 0 | 0 | 0 | 0 |
| 595 | 0.00001 | 0 | 0.940277 | 0.059713 | 0 | 0 | 0 | 0 | 0 |
| 596 | 0.00003 | 0 | 0.939608 | 0.060362 | 0 | 0 | 0 | 0 | 0 |
| 597 | 0.00001 | 0 | 0.941213 | 0.058777 | 0 | 0 | 0 | 0 | 0 |
| 598 | 0.00001 | 0 | 0.941681 | 0.058309 | 0 | 0 | 0 | 0 | 0 |
| 599 | 0.00002 | 0 | 0.940161 | 0.059819 | 0 | 0 | 0 | 0 | 0 |
| 600 | 0.00001 | 0 | 0.936437 | 0.063553 | 0 | 0 | 0 | 0 | 0 |
| 601 | 0.00001 | 0 | 0.938521 | 0.061469 | 0 | 0 | 0 | 0 | 0 |
| 602 | 0.00001 | 0 | 0.942656 | 0.057334 | 0 | 0 | 0 | 0 | 0 |
| 603 | 0.00001 | 0 | 0.940124 | 0.059866 | 0 | 0 | 0 | 0 | 0 |
| 604 | 0 | 0 | 0.940155 | 0.059845 | 0 | 0 | 0 | 0 | 0 |
| 605 | 0.00001 | 0 | 0.93963 | 0.06036 | 0 | 0 | 0 | 0 | 0 |
| 606 | 0.00004 | 0 | 0.94149 | 0.05847 | 0 | 0 | 0 | 0 | 0 |
| 607 | 0.00004 | 0 | 0.9443 | 0.05566 | 0 | 0 | 0 | 0 | 0 |
| 608 | 0.00001 | 0 | 0.941126 | 0.058864 | 0 | 0 | 0 | 0 | 0 |
| 609 | 0.00002 | 0 | 0.941121 | 0.058859 | 0 | 0 | 0 | 0 | 0 |
| 610 | 0.00001 | 0 | 0.938124 | 0.061866 | 0 | 0 | 0 | 0 | 0 |
| 611 | 0 | 0 | 0.940012 | 0.059988 | 0 | 0 | 0 | 0 | 0 |
| 612 | 0 | 0 | 0.940333 | 0.059667 | 0 | 0 | 0 | 0 | 0 |
| 613 | 0.00002 | 0 | 0.936133 | 0.063847 | 0 | 0 | 0 | 0 | 0 |
| 614 | 0 | 0 | 0.941616 | 0.058384 | 0 | 0 | 0 | 0 | 0 |
| 615 | 0 | 0 | 0.944259 | 0.055741 | 0 | 0 | 0 | 0 | 0 |
| 616 | 0 | 0 | 0.940596 | 0.059404 | 0 | 0 | 0 | 0 | 0 |
| 617 | 0.00001 | 0 | 0.93954 | 0.06045 | 0 | 0 | 0 | 0 | 0 |
| 618 | 0 | 0 | 0.936863 | 0.063137 | 0 | 0 | 0 | 0 | 0 |
| 619 | 0.00001 | 0 | 0.94361 | 0.05638 | 0 | 0 | 0 | 0 | 0 |
| 620 | 0.00003 | 0 | 0.939577 | 0.060393 | 0 | 0 | 0 | 0 | 0 |
| 621 | 0.00003 | 0 | 0.940315 | 0.059655 | 0 | 0 | 0 | 0 | 0 |
| 622 | 0.00001 | 0 | 0.934124 | 0.065866 | 0 | 0 | 0 | 0 | 0 |
| 623 | 0.00001 | 0 | 0.938634 | 0.061356 | 0 | 0 | 0 | 0 | 0 |
| 624 | 0.00002 | 0 | 0.93922 | 0.060761 | 0 | 0 | 0 | 0 | 0 |
| 625 | 0.00001 | 0 | 0.940275 | 0.059405 | 0 | 0 | 0 | 0 | 0 |
| 626 | 0 | 0 | 0.935772 | 0.064228 | 0 | 0 | 0 | 0 | 0 |
| 627 | 0.00001 | 0 | 0.938748 | 0.061242 | 0 | 0 | 0 | 0 | 0 |
| 628 | 0.00003 | 0 | 0.939243 | 0.060727 | 0 | 0 | 0 | 0 | 0 |
| 629 | 0.00003 | 0 | 0.938037 | 0.061933 | 0 | 0 | 0 | 0 | 0 |
| 630 | 0 | 0 | 0.938174 | 0.061826 | 0 | 0 | 0 | 0 | 0 |
| 631 | 0.00003 | 0 | 0.937723 | 0.062247 | 0 | 0 | 0 | 0 | 0 |
| 632 | 0 | 0 | 0.939204 | 0.060796 | 0 | 0 | 0 | 0 | 0 |
| 633 | 0.00003 | 0 | 0.940375 | 0.059595 | 0 | 0 | 0 | 0 | 0 |
| 634 | 0.00001 | 0 | 0.938948 | 0.061042 | 0 | 0 | 0 | 0 | 0 |
| 635 | 0.00002 | 0 | 0.945168 | 0.054813 | 0 | 0 | 0 | 0 | 0 |
| 636 | 0 | 0 | 0.938315 | 0.061685 | 0 | 0 | 0 | 0 | 0 |
| 637 | 0.00002 | 0 | 0.937532 | 0.062448 | 0 | 0 | 0 | 0 | 0 |
| 638 | 0.00002 | 0 | 0.938099 | 0.061881 | 0 | 0 | 0 | 0 | 0 |
| 639 | 0.00001 | 0 | 0.93449 | 0.0655 | 0 | 0 | 0 | 0 | 0 |
| 640 | 0.00003 | 0 | 0.937486 | 0.062484 | 0 | 0 | 0 | 0 | 0 |
| 641 | 0.00002 | 0 | 0.940181 | 0.059799 | 0 | 0 | 0 | 0 | 0 |
| 642 | 0 | 0 | 0.941741 | 0.058259 | 0 | 0 | 0 | 0 | 0 |
| 643 | 0.00001 | 0 | 0.939136 | 0.060854 | 0 | 0 | 0 | 0 | 0 |
| 644 | 0.00002 | 0 | 0.938319 | 0.061661 | 0 | 0 | 0 | 0 | 0 |
| 645 | 0 | 0 | 0.942547 | 0.057453 | 0 | 0 | 0 | 0 | 0 |
| 646 | 0 | 0 | 0.936354 | 0.063646 | 0 | 0 | 0 | 0 | 0 |
| 647 | 0 | 0 | 0.93754 | 0.06246 | 0 | 0 | 0 | 0 | 0 |
| 648 | 0 | 0 | 0.943169 | 0.056831 | 0 | 0 | 0 | 0 | 0 |
| 649 | 0.00001 | 0 | 0.93871 | 0.06128 | 0 | 0 | 0 | 0 | 0 |
| 650 | 0.00003 | 0 | 0.936961 | 0.063009 | 0 | 0 | 0 | 0 | 0 |
| 651 | 0 | 0 | 0.939996 | 0.060004 | 0 | 0 | 0 | 0 | 0 |
| 652 | 0.00001 | 0 | 0.938239 | 0.061751 | 0 | 0 | 0 | 0 | 0 |
| 653 | 0.00001 | 0 | 0.942215 | 0.057775 | 0 | 0 | 0 | 0 | 0 |
| 654 | 0.00002 | 0 | 0.940165 | 0.059815 | 0 | 0 | 0 | 0 | 0 |
| 655 | 0.00002 | 0 | 0.93623 | 0.06375 | 0 | 0 | 0 | 0 | 0 |
| 656 | 0.00003 | 0 | 0.945186 | 0.054784 | 0 | 0 | 0 | 0 | 0 |
| 657 | 0.00002 | 0 | 0.937561 | 0.062419 | 0 | 0 | 0 | 0 | 0 |
| 658 | 0.00002 | 0 | 0.941076 | 0.058904 | 0 | 0 | 0 | 0 | 0 |
| 659 | 0.00002 | 0 | 0.937135 | 0.062845 | 0 | 0 | 0 | 0 | 0 |
| 660 | 0.00001 | 0 | 0.942947 | 0.057043 | 0 | 0 | 0 | 0 | 0 |
| 661 | 0 | 0 | 0.94061 | 0.05939 | 0 | 0 | 0 | 0 | 0 |
| 662 | 0.00001 | 0 | 0.940173 | 0.059817 | 0 | 0 | 0 | 0 | 0 |
| 663 | 0.00002 | 0 | 0.939928 | 0.060052 | 0 | 0 | 0 | 0 | 0 |
| 664 | 0 | 0 | 0.944318 | 0.055682 | 0 | 0 | 0 | 0 | 0 |
| 665 | 0.00001 | 0 | 0.937598 | 0.062392 | 0 | 0 | 0 | 0 | 0 |
| 666 | 0.00001 | 0 | 0.939802 | 0.060188 | 0 | 0 | 0 | 0 | 0 |
| 667 | 0.00003 | 0 | 0.935941 | 0.064029 | 0 | 0 | 0 | 0 | 0 |
| 668 | 0.00001 | 0 | 0.938768 | 0.061222 | 0 | 0 | 0 | 0 | 0 |
| 669 | 0.00003 | 0 | 0.935825 | 0.064145 | 0 | 0 | 0 | 0 | 0 |
| 670 | 0.00002 | 0 | 0.941088 | 0.058892 | 0 | 0 | 0 | 0 | 0 |
| 671 | 0 | 0 | 0.940418 | 0.059582 | 0 | 0 | 0 | 0 | 0 |
| 672 | 0.00003 | 0 | 0.94006 | 0.05991 | 0 | 0 | 0 | 0 | 0 |
| 673 | 0 | 0 | 0.936206 | 0.063794 | 0 | 0 | 0 | 0 | 0 |
| 674 | 0.00002 | 0 | 0.939104 | 0.060876 | 0 | 0 | 0 | 0 | 0 |
| 675 | 0 | 0 | 0.942211 | 0.057789 | 0 | 0 | 0 | 0 | 0 |
| 676 | 0.00003 | 0 | 0.940854 | 0.059116 | 0 | 0 | 0 | 0 | 0 |
| 677 | 0.00002 | 0 | 0.940047 | 0.059933 | 0 | 0 | 0 | 0 | 0 |
| 678 | 0 | 0 | 0.940733 | 0.059267 | 0 | 0 | 0 | 0 | 0 |
| 679 | 0.00001 | 0 | 0.94224 | 0.05775 | 0 | 0 | 0 | 0 | 0 |
| 680 | 0 | 0 | 0.93799 | 0.06201 | 0 | 0 | 0 | 0 | 0 |
| 681 | 0 | 0 | 0.938502 | 0.061498 | 0 | 0 | 0 | 0 | 0 |
| 682 | 0.00002 | 0 | 0.938086 | 0.061893 | 0 | 0 | 0 | 0 | 0 |
| 683 | 0.00001 | 0 | 0.941138 | 0.058852 | 0 | 0 | 0 | 0 | 0 |
| 684 | 0.00001 | 0 | 0.941305 | 0.058685 | 0 | 0 | 0 | 0 | 0 |
| 685 | 0.00001 | 0 | 0.941732 | 0.058258 | 0 | 0 | 0 | 0 | 0 |
| 686 | 0.00002 | 0 | 0.936458 | 0.063522 | 0 | 0 | 0 | 0 | 0 |
| 687 | 0.00002 | 0 | 0.941391 | 0.058589 | 0 | 0 | 0 | 0 | 0 |
| 688 | 0 | 0 | 0.942463 | 0.057537 | 0 | 0 | 0 | 0 | 0 |
| 689 | 0.00001 | 0 | 0.935158 | 0.064832 | 0 | 0 | 0 | 0 | 0 |
| 690 | 0.00002 | 0 | 0.940959 | 0.059021 | 0 | 0 | 0 | 0 | 0 |
| 691 | 0.00002 | 0 | 0.941051 | 0.058929 | 0 | 0 | 0 | 0 | 0 |
| 692 | 0 | 0 | 0.937011 | 0.062989 | 0 | 0 | 0 | 0 | 0 |
| 693 | 0.00001 | 0 | 0.936082 | 0.063908 | 0 | 0 | 0 | 0 | 0 |
| 694 | 0 | 0 | 0.941834 | 0.058166 | 0 | 0 | 0 | 0 | 0 |
| 695 | 0.00001 | 0 | 0.939187 | 0.060803 | 0 | 0 | 0 | 0 | 0 |
| 696 | 0.00001 | 0 | 0.943393 | 0.056597 | 0 | 0 | 0 | 0 | 0 |
| 697 | 0.00001 | 0 | 0.940447 | 0.059543 | 0 | 0 | 0 | 0 | 0 |
| 698 | 0.00001 | 0 | 0.938924 | 0.061066 | 0 | 0 | 0 | 0 | 0 |
| 699 | 0.00002 | 0 | 0.939636 | 0.060344 | 0 | 0 | 0 | 0 | 0 |
| 700 | 0.00001 | 0 | 0.939488 | 0.060502 | 0 | 0 | 0 | 0 | 0 |
| 701 | 0.00002 | 0 | 0.943758 | 0.056222 | 0 | 0 | 0 | 0 | 0 |
| 702 | 0.00001 | 0 | 0.941474 | 0.058516 | 0 | 0 | 0 | 0 | 0 |
| 703 | 0 | 0 | 0.939227 | 0.060773 | 0 | 0 | 0 | 0 | 0 |
| 704 | 0.00001 | 0 | 0.94 | 0.05999 | 0 | 0 | 0 | 0 | 0 |
| 705 | 0 | 0 | 0.936579 | 0.063421 | 0 | 0 | 0 | 0 | 0 |
| 706 | 0.00002 | 0 | 0.937296 | 0.062685 | 0 | 0 | 0 | 0 | 0 |
| 707 | 0.00001 | 0 | 0.939074 | 0.060916 | 0 | 0 | 0 | 0 | 0 |
| 708 | 0.00002 | 0 | 0.936182 | 0.063798 | 0 | 0 | 0 | 0 | 0 |
| 709 | 0.00001 | 0 | 0.943845 | 0.056145 | 0 | 0 | 0 | 0 | 0 |
| 710 | 0.00002 | 0 | 0.939662 | 0.060318 | 0 | 0 | 0 | 0 | 0 |
| 711 | 0.00001 | 0 | 0.940628 | 0.059362 | 0 | 0 | 0 | 0 | 0 |
| 712 | 0.00004 | 0 | 0.943741 | 0.056219 | 0 | 0 | 0 | 0 | 0 |
| 713 | 0.00002 | 0 | 0.93597 | 0.06401 | 0 | 0 | 0 | 0 | 0 |
| 714 | 0 | 0 | 0.937497 | 0.062503 | 0 | 0 | 0 | 0 | 0 |
| 715 | 0.00003 | 0 | 0.937546 | 0.062424 | 0 | 0 | 0 | 0 | 0 |
| 716 | 0.00001 | 0 | 0.938324 | 0.061666 | 0 | 0 | 0 | 0 | 0 |
| 717 | 0.00003 | 0 | 0.93956 | 0.06041 | 0 | 0 | 0 | 0 | 0 |
| 718 | 0 | 0 | 0.937672 | 0.062328 | 0 | 0 | 0 | 0 | 0 |
| 719 | 0.00002 | 0 | 0.944479 | 0.055501 | 0 | 0 | 0 | 0 | 0 |
| 720 | 0.00003 | 0 | 0.937799 | 0.062171 | 0 | 0 | 0 | 0 | 0 |
| 721 | 0.00001 | 0 | 0.936791 | 0.063199 | 0 | 0 | 0 | 0 | 0 |
| 722 | 0.00001 | 0 | 0.940912 | 0.059078 | 0 | 0 | 0 | 0 | 0 |
| 723 | 0.00002 | 0 | 0.940367 | 0.059613 | 0 | 0 | 0 | 0 | 0 |
| 724 | 0 | 0 | 0.940722 | 0.059278 | 0 | 0 | 0 | 0 | 0 |
| 725 | 0 | 0 | 0.939417 | 0.060583 | 0 | 0 | 0 | 0 | 0 |
| 726 | 0 | 0 | 0.938891 | 0.061109 | 0 | 0 | 0 | 0 | 0 |
| 727 | 0.00003 | 0 | 0.937907 | 0.062063 | 0 | 0 | 0 | 0 | 0 |
| 728 | 0.00002 | 0 | 0.937065 | 0.062915 | 0 | 0 | 0 | 0 | 0 |
| 729 | 0.00001 | 0 | 0.937671 | 0.062319 | 0 | 0 | 0 | 0 | 0 |
| 730 | 0 | 0 | 0.939458 | 0.060542 | 0 | 0 | 0 | 0 | 0 |
| 731 | 0.00002 | 0 | 0.943082 | 0.056898 | 0 | 0 | 0 | 0 | 0 |
| 732 | 0 | 0 | 0.941309 | 0.058691 | 0 | 0 | 0 | 0 | 0 |
| 733 | 0 | 0 | 0.938271 | 0.061729 | 0 | 0 | 0 | 0 | 0 |
| 734 | 0.00001 | 0 | 0.943709 | 0.056281 | 0 | 0 | 0 | 0 | 0 |
| 735 | 0.00002 | 0 | 0.941886 | 0.058094 | 0 | 0 | 0 | 0 | 0 |
| 736 | 0.00001 | 0 | 0.943037 | 0.056953 | 0 | 0 | 0 | 0 | 0 |
| 737 | 0 | 0 | 0.940243 | 0.059757 | 0 | 0 | 0 | 0 | 0 |
| 738 | 0.00001 | 0 | 0.940844 | 0.059146 | 0 | 0 | 0 | 0 | 0 |
| 739 | 0.00001 | 0 | 0.932508 | 0.067482 | 0 | 0 | 0 | 0 | 0 |
| 740 | 0 | 0 | 0.938948 | 0.061052 | 0 | 0 | 0 | 0 | 0 |
| 741 | 0.00001 | 0 | 0.941378 | 0.058612 | 0 | 0 | 0 | 0 | 0 |
| 742 | 0.00002 | 0 | 0.941182 | 0.058797 | 0 | 0 | 0 | 0 | 0 |
| 743 | 0 | 0 | 0.941519 | 0.058481 | 0 | 0 | 0 | 0 | 0 |
| 744 | 0 | 0 | 0.939416 | 0.059672 | 0 | 0 | 0 | 0 | 0 |
| 745 | 0 | 0 | 0.942712 | 0.057288 | 0 | 0 | 0 | 0 | 0 |
| 746 | 0.00001 | 0 | 0.935577 | 0.064414 | 0 | 0 | 0 | 0 | 0 |
| 747 | 0.00001 | 0 | 0.937515 | 0.062475 | 0 | 0 | 0 | 0 | 0 |
| 748 | 0.00004 | 0 | 0.939822 | 0.060137 | 0 | 0 | 0 | 0 | 0 |
| 749 | 0.00001 | 0 | 0.942506 | 0.057484 | 0 | 0 | 0 | 0 | 0 |
| 750 | 0.00004 | 0 | 0.940735 | 0.059225 | 0 | 0 | 0 | 0 | 0 |
| 751 | 0 | 0 | 0.945161 | 0.054839 | 0 | 0 | 0 | 0 | 0 |
| 752 | 0.00005 | 0 | 0.941606 | 0.058344 | 0 | 0 | 0 | 0 | 0 |
| 753 | 0 | 0 | 0.939924 | 0.060076 | 0 | 0 | 0 | 0 | 0 |
| 754 | 0.00001 | 0 | 0.93813 | 0.06186 | 0 | 0 | 0 | 0 | 0 |
| 755 | 0.00001 | 0 | 0.940722 | 0.059268 | 0 | 0 | 0 | 0 | 0 |
| 756 | 0 | 0 | 0.937433 | 0.062567 | 0 | 0 | 0 | 0 | 0 |
| 757 | 0.00002 | 0 | 0.942774 | 0.057206 | 0 | 0 | 0 | 0 | 0 |
| 758 | 0.00001 | 0 | 0.939136 | 0.060854 | 0 | 0 | 0 | 0 | 0 |
| 759 | 0.00001 | 0 | 0.94213 | 0.05786 | 0 | 0 | 0 | 0 | 0 |
| 760 | 0.00002 | 0 | 0.94283 | 0.057149 | 0 | 0 | 0 | 0 | 0 |
| 761 | 0.00001 | 0 | 0.938388 | 0.061602 | 0 | 0 | 0 | 0 | 0 |
| 762 | 0.00002 | 0 | 0.940234 | 0.059746 | 0 | 0 | 0 | 0 | 0 |
| 763 | 0.00001 | 0 | 0.942367 | 0.057623 | 0 | 0 | 0 | 0 | 0 |
| 764 | 0.00003 | 0 | 0.943178 | 0.056792 | 0 | 0 | 0 | 0 | 0 |
| 765 | 0.00002 | 0 | 0.936774 | 0.063206 | 0 | 0 | 0 | 0 | 0 |
| 766 | 0.00001 | 0 | 0.935429 | 0.064561 | 0 | 0 | 0 | 0 | 0 |
| 767 | 0.00001 | 0 | 0.943014 | 0.056976 | 0 | 0 | 0 | 0 | 0 |
| 768 | 0.00001 | 0 | 0.937997 | 0.061994 | 0 | 0 | 0 | 0 | 0 |
| 769 | 0.00003 | 0 | 0.939403 | 0.060567 | 0 | 0 | 0 | 0 | 0 |
| 770 | 0 | 0 | 0.941404 | 0.058596 | 0 | 0 | 0 | 0 | 0 |
| 771 | 0 | 0 | 0.939208 | 0.059692 | 0 | 0 | 0 | 0 | 0 |
| 772 | 0 | 0 | 0.939912 | 0.060088 | 0 | 0 | 0 | 0 | 0 |
| 773 | 0.00002 | 0 | 0.935271 | 0.064709 | 0 | 0 | 0 | 0 | 0 |
| 774 | 0.00001 | 0 | 0.940655 | 0.059335 | 0 | 0 | 0 | 0 | 0 |
| 775 | 0.00002 | 0 | 0.940731 | 0.059248 | 0 | 0 | 0 | 0 | 0 |
| 776 | 0 | 0 | 0.940775 | 0.059225 | 0 | 0 | 0 | 0 | 0 |
| 777 | 0 | 0 | 0.939133 | 0.060867 | 0 | 0 | 0 | 0 | 0 |
| 778 | 0 | 0 | 0.939648 | 0.060352 | 0 | 0 | 0 | 0 | 0 |
| 779 | 0.00001 | 0 | 0.945459 | 0.054531 | 0 | 0 | 0 | 0 | 0 |
| 780 | 0 | 0 | 0.938111 | 0.061889 | 0 | 0 | 0 | 0 | 0 |
| 781 | 0.00003 | 0 | 0.938314 | 0.061656 | 0 | 0 | 0 | 0 | 0 |
| 782 | 0.00001 | 0 | 0.940266 | 0.059724 | 0 | 0 | 0 | 0 | 0 |
| 783 | 0.00003 | 0 | 0.938194 | 0.061776 | 0 | 0 | 0 | 0 | 0 |
| 784 | 0.00002 | 0 | 0.938567 | 0.061413 | 0 | 0 | 0 | 0 | 0 |
| 785 | 0.00003 | 0 | 0.942337 | 0.057633 | 0 | 0 | 0 | 0 | 0 |
| 786 | 0 | 0 | 0.93994 | 0.06006 | 0 | 0 | 0 | 0 | 0 |
| 787 | 0.00001 | 0 | 0.938851 | 0.061139 | 0 | 0 | 0 | 0 | 0 |
| 788 | 0.00001 | 0 | 0.942216 | 0.057774 | 0 | 0 | 0 | 0 | 0 |
| 789 | 0.00003 | 0 | 0.936811 | 0.063159 | 0 | 0 | 0 | 0 | 0 |
| 790 | 0.00002 | 0 | 0.938401 | 0.061579 | 0 | 0 | 0 | 0 | 0 |
| 791 | 0 | 0 | 0.94107 | 0.05893 | 0 | 0 | 0 | 0 | 0 |
| 792 | 0.00001 | 0 | 0.942248 | 0.057742 | 0 | 0 | 0 | 0 | 0 |
| 793 | 0 | 0 | 0.938927 | 0.061073 | 0 | 0 | 0 | 0 | 0 |
| 794 | 0.00001 | 0 | 0.941387 | 0.058602 | 0 | 0 | 0 | 0 | 0 |
| 795 | 0.00001 | 0 | 0.937106 | 0.062884 | 0 | 0 | 0 | 0 | 0 |
| 796 | 0.00002 | 0 | 0.943462 | 0.056519 | 0 | 0 | 0 | 0 | 0 |
| 797 | 0.00001 | 0 | 0.942914 | 0.057076 | 0 | 0 | 0 | 0 | 0 |
| 798 | 0.00001 | 0 | 0.937191 | 0.062095 | 0 | 0 | 0 | 0 | 0 |
| 799 | 0.00002 | 0 | 0.941309 | 0.058671 | 0 | 0 | 0 | 0 | 0 |
| 800 | 0 | 0 | 0.936884 | 0.063116 | 0 | 0 | 0 | 0 | 0 |
| 801 | 0.00001 | 0 | 0.942759 | 0.057231 | 0 | 0 | 0 | 0 | 0 |
| 802 | 0.00001 | 0 | 0.935278 | 0.064712 | 0 | 0 | 0 | 0 | 0 |
| 803 | 0.00001 | 0 | 0.941801 | 0.058189 | 0 | 0 | 0 | 0 | 0 |
| 804 | 0 | 0 | 0.939005 | 0.060995 | 0 | 0 | 0 | 0 | 0 |
| 805 | 0.00002 | 0 | 0.941392 | 0.058588 | 0 | 0 | 0 | 0 | 0 |
| 806 | 0.00001 | 0 | 0.939505 | 0.060485 | 0 | 0 | 0 | 0 | 0 |
| 807 | 0.00001 | 0 | 0.943449 | 0.056541 | 0 | 0 | 0 | 0 | 0 |
| 808 | 0.00001 | 0 | 0.939139 | 0.060851 | 0 | 0 | 0 | 0 | 0 |
| 809 | 0.00001 | 0 | 0.940852 | 0.059138 | 0 | 0 | 0 | 0 | 0 |
| 810 | 0.00001 | 0 | 0.937598 | 0.062392 | 0 | 0 | 0 | 0 | 0 |
| 811 | 0.00002 | 0 | 0.940466 | 0.059514 | 0 | 0 | 0 | 0 | 0 |
| 812 | 0.00002 | 0 | 0.940051 | 0.059929 | 0 | 0 | 0 | 0 | 0 |
| 813 | 0.00002 | 0 | 0.940684 | 0.059296 | 0 | 0 | 0 | 0 | 0 |
| 814 | 0.00003 | 0 | 0.938635 | 0.061335 | 0 | 0 | 0 | 0 | 0 |
| 815 | 0.00003 | 0 | 0.939724 | 0.060246 | 0 | 0 | 0 | 0 | 0 |
| 816 | 0.00001 | 0 | 0.938973 | 0.061017 | 0 | 0 | 0 | 0 | 0 |
| 817 | 0 | 0 | 0.943412 | 0.056588 | 0 | 0 | 0 | 0 | 0 |
| 818 | 0.00002 | 0 | 0.93553 | 0.06445 | 0 | 0 | 0 | 0 | 0 |
| 819 | 0 | 0 | 0.93805 | 0.06195 | 0 | 0 | 0 | 0 | 0 |
| 820 | 0 | 0 | 0.938962 | 0.061038 | 0 | 0 | 0 | 0 | 0 |
| 821 | 0 | 0 | 0.942438 | 0.057562 | 0 | 0 | 0 | 0 | 0 |
| 822 | 0.00001 | 0 | 0.940494 | 0.059496 | 0 | 0 | 0 | 0 | 0 |
| 823 | 0.00001 | 0 | 0.938378 | 0.061612 | 0 | 0 | 0 | 0 | 0 |
| 824 | 0.00002 | 0 | 0.945281 | 0.054699 | 0 | 0 | 0 | 0 | 0 |
| 825 | 0.00003 | 0 | 0.94245 | 0.05752 | 0 | 0 | 0 | 0 | 0 |
| 826 | 0.00001 | 0 | 0.941455 | 0.058535 | 0 | 0 | 0 | 0 | 0 |
| 827 | 0.00001 | 0 | 0.940078 | 0.059912 | 0 | 0 | 0 | 0 | 0 |
| 828 | 0.00001 | 0 | 0.940276 | 0.059714 | 0 | 0 | 0 | 0 | 0 |
| 829 | 0.00002 | 0 | 0.935626 | 0.064354 | 0 | 0 | 0 | 0 | 0 |
| 830 | 0.00001 | 0 | 0.940502 | 0.059488 | 0 | 0 | 0 | 0 | 0 |
| 831 | 0.00002 | 0 | 0.935784 | 0.064196 | 0 | 0 | 0 | 0 | 0 |
| 832 | 0 | 0 | 0.94272 | 0.05728 | 0 | 0 | 0 | 0 | 0 |
| 833 | 0.00001 | 0 | 0.941233 | 0.058757 | 0 | 0 | 0 | 0 | 0 |
| 834 | 0.00003 | 0 | 0.936994 | 0.062976 | 0 | 0 | 0 | 0 | 0 |
| 835 | 0.00002 | 0 | 0.942023 | 0.057957 | 0 | 0 | 0 | 0 | 0 |
| 836 | 0.00001 | 0 | 0.94341 | 0.05658 | 0 | 0 | 0 | 0 | 0 |
| 837 | 0.00003 | 0 | 0.940732 | 0.059238 | 0 | 0 | 0 | 0 | 0 |
| 838 | 0 | 0 | 0.93965 | 0.06035 | 0 | 0 | 0 | 0 | 0 |
| 839 | 0.00002 | 0 | 0.938378 | 0.061602 | 0 | 0 | 0 | 0 | 0 |
| 840 | 0.00002 | 0 | 0.938338 | 0.061642 | 0 | 0 | 0 | 0 | 0 |
| 841 | 0.00002 | 0 | 0.940087 | 0.059893 | 0 | 0 | 0 | 0 | 0 |
| 842 | 0.00001 | 0 | 0.93848 | 0.06151 | 0 | 0 | 0 | 0 | 0 |
| 843 | 0.00001 | 0 | 0.940477 | 0.059513 | 0 | 0 | 0 | 0 | 0 |
| 844 | 0.00001 | 0 | 0.939392 | 0.060598 | 0 | 0 | 0 | 0 | 0 |
| 845 | 0.00003 | 0 | 0.937161 | 0.062809 | 0 | 0 | 0 | 0 | 0 |
| 846 | 0.00001 | 0 | 0.979333 | 0.020657 | 0 | 0 | 0 | 0 | 0 |
| 847 | 0 | 0 | 0.937085 | 0.062915 | 0 | 0 | 0 | 0 | 0 |
| 848 | 0 | 0 | 0.941438 | 0.058562 | 0 | 0 | 0 | 0 | 0 |
| 849 | 0.00003 | 0 | 0.945094 | 0.054876 | 0 | 0 | 0 | 0 | 0 |
| 850 | 0 | 0 | 0.939336 | 0.060664 | 0 | 0 | 0 | 0 | 0 |
| 851 | 0.00001 | 0 | 0.941347 | 0.058643 | 0 | 0 | 0 | 0 | 0 |
| 852 | 0 | 0 | 0.93708 | 0.06292 | 0 | 0 | 0 | 0 | 0 |
| 853 | 0.00001 | 0 | 0.937544 | 0.062446 | 0 | 0 | 0 | 0 | 0 |
| 854 | 0.00001 | 0 | 0.938117 | 0.061873 | 0 | 0 | 0 | 0 | 0 |
| 855 | 0.00002 | 0 | 0.936383 | 0.063597 | 0 | 0 | 0 | 0 | 0 |
| 856 | 0.00001 | 0 | 0.936791 | 0.063199 | 0 | 0 | 0 | 0 | 0 |
| 857 | 0.00001 | 0 | 0.940313 | 0.059677 | 0 | 0 | 0 | 0 | 0 |
| 858 | 0.00002 | 0 | 0.943844 | 0.056136 | 0 | 0 | 0 | 0 | 0 |
| 859 | 0.00001 | 0 | 0.940616 | 0.059374 | 0 | 0 | 0 | 0 | 0 |
| 860 | 0 | 0 | 0.936248 | 0.063752 | 0 | 0 | 0 | 0 | 0 |
| 861 | 0 | 0 | 0.940304 | 0.058304 | 0 | 0 | 0 | 0 | 0 |
| 862 | 0.00001 | 0 | 0.941034 | 0.058956 | 0 | 0 | 0 | 0 | 0 |
| 863 | 0.00002 | 0 | 0.93588 | 0.064101 | 0 | 0 | 0 | 0 | 0 |
| 864 | 0.00004 | 0 | 0.943006 | 0.056955 | 0 | 0 | 0 | 0 | 0 |
| 865 | 0 | 0 | 0.938274 | 0.061726 | 0 | 0 | 0 | 0 | 0 |
| 866 | 0.00003 | 0 | 0.939298 | 0.060672 | 0 | 0 | 0 | 0 | 0 |
| 867 | 0.00001 | 0 | 0.940888 | 0.059102 | 0 | 0 | 0 | 0 | 0 |
| 868 | 0 | 0 | 0.937233 | 0.062767 | 0 | 0 | 0 | 0 | 0 |
| 869 | 0.00003 | 0 | 0.940324 | 0.059646 | 0 | 0 | 0 | 0 | 0 |
| 870 | 0 | 0 | 0.939749 | 0.060251 | 0 | 0 | 0 | 0 | 0 |
| 871 | 0 | 0 | 0.943549 | 0.056451 | 0 | 0 | 0 | 0 | 0 |
| 872 | 0.00001 | 0 | 0.937713 | 0.062277 | 0 | 0 | 0 | 0 | 0 |
| 873 | 0.00005 | 0 | 0.937456 | 0.062494 | 0 | 0 | 0 | 0 | 0 |
| 874 | 0.00001 | 0 | 0.94035 | 0.05964 | 0 | 0 | 0 | 0 | 0 |
| 875 | 0.00002 | 0 | 0.942176 | 0.057804 | 0 | 0 | 0 | 0 | 0 |
| 876 | 0.00001 | 0 | 0.939229 | 0.060761 | 0 | 0 | 0 | 0 | 0 |
| 877 | 0.00002 | 0 | 0.943407 | 0.056573 | 0 | 0 | 0 | 0 | 0 |
| 878 | 0.00003 | 0 | 0.942604 | 0.057366 | 0 | 0 | 0 | 0 | 0 |
| 879 | 0 | 0 | 0.938486 | 0.061514 | 0 | 0 | 0 | 0 | 0 |
| 880 | 0.00001 | 0 | 0.938967 | 0.061023 | 0 | 0 | 0 | 0 | 0 |
| 881 | 0.00002 | 0 | 0.938805 | 0.061175 | 0 | 0 | 0 | 0 | 0 |
| 882 | 0.00001 | 0 | 0.936066 | 0.063924 | 0 | 0 | 0 | 0 | 0 |
| 883 | 0 | 0 | 0.940317 | 0.059683 | 0 | 0 | 0 | 0 | 0 |
| 884 | 0 | 0 | 0.937272 | 0.062728 | 0 | 0 | 0 | 0 | 0 |
| 885 | 0.00003 | 0 | 0.94407 | 0.0559 | 0 | 0 | 0 | 0 | 0 |
| 886 | 0 | 0 | 0.938461 | 0.061539 | 0 | 0 | 0 | 0 | 0 |
| 887 | 0 | 0 | 0.940544 | 0.059456 | 0 | 0 | 0 | 0 | 0 |
| 888 | 0.00001 | 0 | 0.935411 | 0.064579 | 0 | 0 | 0 | 0 | 0 |
| 889 | 0.00001 | 0 | 0.934595 | 0.065395 | 0 | 0 | 0 | 0 | 0 |
| 890 | 0 | 0 | 0.940366 | 0.059634 | 0 | 0 | 0 | 0 | 0 |
| 891 | 0 | 0 | 0.938453 | 0.061547 | 0 | 0 | 0 | 0 | 0 |
| 892 | 0 | 0 | 0.942142 | 0.057858 | 0 | 0 | 0 | 0 | 0 |
| 893 | 0.00003 | 0 | 0.939674 | 0.060296 | 0 | 0 | 0 | 0 | 0 |
| 894 | 0 | 0 | 0.934419 | 0.065581 | 0 | 0 | 0 | 0 | 0 |
| 895 | 0.00004 | 0 | 0.94523 | 0.05473 | 0 | 0 | 0 | 0 | 0 |
| 896 | 0.00001 | 0 | 0.938143 | 0.061847 | 0 | 0 | 0 | 0 | 0 |
| 897 | 0.00002 | 0 | 0.943334 | 0.056646 | 0 | 0 | 0 | 0 | 0 |
| 898 | 0.00001 | 0 | 0.940625 | 0.059365 | 0 | 0 | 0 | 0 | 0 |
| 899 | 0.00002 | 0 | 0.943389 | 0.056591 | 0 | 0 | 0 | 0 | 0 |
| 900 | 0.00002 | 0 | 0.939909 | 0.060071 | 0 | 0 | 0 | 0 | 0 |
| 901 | 0.00002 | 0 | 0.943846 | 0.056134 | 0 | 0 | 0 | 0 | 0 |
| 902 | 0.00001 | 0 | 0.937567 | 0.062423 | 0 | 0 | 0 | 0 | 0 |
| 903 | 0.00001 | 0 | 0.939397 | 0.060593 | 0 | 0 | 0 | 0 | 0 |
| 904 | 0 | 0 | 0.943758 | 0.056242 | 0 | 0 | 0 | 0 | 0 |
| 905 | 0.00002 | 0 | 0.940366 | 0.059614 | 0 | 0 | 0 | 0 | 0 |
| 906 | 0 | 0 | 0.939504 | 0.060496 | 0 | 0 | 0 | 0 | 0 |
| 907 | 0 | 0 | 0.945855 | 0.054145 | 0 | 0 | 0 | 0 | 0 |
| 908 | 0 | 0 | 0.939274 | 0.060726 | 0 | 0 | 0 | 0 | 0 |
| 909 | 0.00004 | 0 | 0.938465 | 0.061495 | 0 | 0 | 0 | 0 | 0 |
| 910 | 0.00001 | 0 | 0.939916 | 0.060074 | 0 | 0 | 0 | 0 | 0 |
| 911 | 0 | 0 | 0.940865 | 0.059135 | 0 | 0 | 0 | 0 | 0 |
| 912 | 0 | 0 | 0.938363 | 0.061637 | 0 | 0 | 0 | 0 | 0 |
| 913 | 0.00001 | 0.00001 | 0.938045 | 0.061935 | 0 | 0 | 0 | 0 | 0 |
| 914 | 0.00003 | 0 | 0.942682 | 0.057288 | 0 | 0 | 0 | 0 | 0 |
| 915 | 0.00003 | 0 | 0.936116 | 0.063854 | 0 | 0 | 0 | 0 | 0 |
| 916 | 0 | 0 | 0.93895 | 0.06105 | 0 | 0 | 0 | 0 | 0 |
| 917 | 0.00002 | 0 | 0.940316 | 0.059664 | 0 | 0 | 0 | 0 | 0 |
| 918 | 0.00001 | 0 | 0.944235 | 0.055755 | 0 | 0 | 0 | 0 | 0 |
| 919 | 0 | 0 | 0.937667 | 0.062333 | 0 | 0 | 0 | 0 | 0 |
| 920 | 0.00001 | 0 | 0.944139 | 0.055851 | 0 | 0 | 0 | 0 | 0 |
| 921 | 0 | 0.00001 | 0.939803 | 0.060187 | 0 | 0 | 0 | 0 | 0 |
| 922 | 0.00003 | 0 | 0.940969 | 0.059001 | 0 | 0 | 0 | 0 | 0 |
| 923 | 0.00002 | 0 | 0.939319 | 0.060661 | 0 | 0 | 0 | 0 | 0 |
| 924 | 0 | 0 | 0.942212 | 0.057788 | 0 | 0 | 0 | 0 | 0 |
| 925 | 0 | 0 | 0.941208 | 0.058792 | 0 | 0 | 0 | 0 | 0 |
| 926 | 0.00001 | 0 | 0.941557 | 0.058433 | 0 | 0 | 0 | 0 | 0 |
| 927 | 0.00003 | 0 | 0.937295 | 0.062675 | 0 | 0 | 0 | 0 | 0 |
| 928 | 0.00001 | 0 | 0.940671 | 0.059319 | 0 | 0 | 0 | 0 | 0 |
| 929 | 0 | 0 | 0.933154 | 0.066846 | 0 | 0 | 0 | 0 | 0 |
| 930 | 0.00001 | 0 | 0.941431 | 0.058559 | 0 | 0 | 0 | 0 | 0 |
| 931 | 0 | 0 | 0.944188 | 0.055812 | 0 | 0 | 0 | 0 | 0 |
| 932 | 0.00002 | 0 | 0.943129 | 0.056851 | 0 | 0 | 0 | 0 | 0 |
| 933 | 0.00001 | 0 | 0.944281 | 0.055709 | 0 | 0 | 0 | 0 | 0 |
| 934 | 0.00002 | 0 | 0.935036 | 0.064944 | 0 | 0 | 0 | 0 | 0 |
| 935 | 0.00001 | 0 | 0.942678 | 0.057312 | 0 | 0 | 0 | 0 | 0 |
| 936 | 0.00001 | 0 | 0.941402 | 0.058588 | 0 | 0 | 0 | 0 | 0 |
| 937 | 0 | 0 | 0.939954 | 0.060046 | 0 | 0 | 0 | 0 | 0 |
| 938 | 0.00005 | 0 | 0.945428 | 0.054522 | 0 | 0 | 0 | 0 | 0 |
| 939 | 0.00001 | 0 | 0.943562 | 0.056428 | 0 | 0 | 0 | 0 | 0 |
| 940 | 0.00001 | 0 | 0.937713 | 0.062277 | 0 | 0 | 0 | 0 | 0 |
| 941 | 0.00001 | 0 | 0.938557 | 0.061433 | 0 | 0 | 0 | 0 | 0 |
| 942 | 0.00002 | 0 | 0.939959 | 0.060021 | 0 | 0 | 0 | 0 | 0 |
| 943 | 0.00002 | 0 | 0.941618 | 0.058362 | 0 | 0 | 0 | 0 | 0 |
| 944 | 0.00001 | 0 | 0.942462 | 0.057528 | 0 | 0 | 0 | 0 | 0 |
| 945 | 0.00001 | 0 | 0.937835 | 0.062155 | 0 | 0 | 0 | 0 | 0 |
| 946 | 0 | 0 | 0.939063 | 0.060937 | 0 | 0 | 0 | 0 | 0 |
| 947 | 0 | 0 | 0.939846 | 0.060154 | 0 | 0 | 0 | 0 | 0 |
| 948 | 0.00002 | 0 | 0.938138 | 0.061842 | 0 | 0 | 0 | 0 | 0 |
| 949 | 0 | 0 | 0.940678 | 0.059322 | 0 | 0 | 0 | 0 | 0 |
| 950 | 0.00002 | 0 | 0.942463 | 0.057517 | 0 | 0 | 0 | 0 | 0 |
| 951 | 0.00003 | 0.00001 | 0.944825 | 0.055135 | 0 | 0 | 0 | 0 | 0 |
| 952 | 0 | 0 | 0.942335 | 0.057665 | 0 | 0 | 0 | 0 | 0 |
| 953 | 0 | 0 | 0.940418 | 0.059582 | 0 | 0 | 0 | 0 | 0 |
| 954 | 0 | 0 | 0.937092 | 0.062908 | 0 | 0 | 0 | 0 | 0 |
| 955 | 0.00001 | 0 | 0.940812 | 0.059178 | 0 | 0 | 0 | 0 | 0 |
| 956 | 0.00002 | 0 | 0.943805 | 0.056175 | 0 | 0 | 0 | 0 | 0 |
| 957 | 0.00001 | 0 | 0.936738 | 0.063252 | 0 | 0 | 0 | 0 | 0 |
| 958 | 0.00002 | 0 | 0.943102 | 0.056878 | 0 | 0 | 0 | 0 | 0 |
| 959 | 0.00002 | 0 | 0.937601 | 0.062379 | 0 | 0 | 0 | 0 | 0 |
| 960 | 0.00002 | 0 | 0.941909 | 0.058071 | 0 | 0 | 0 | 0 | 0 |
| 961 | 0.00002 | 0 | 0.938429 | 0.061551 | 0 | 0 | 0 | 0 | 0 |
| 962 | 0 | 0 | 0.941101 | 0.058899 | 0 | 0 | 0 | 0 | 0 |
| 963 | 0 | 0 | 0.937323 | 0.062677 | 0 | 0 | 0 | 0 | 0 |
| 964 | 0.00001 | 0 | 0.94196 | 0.05803 | 0 | 0 | 0 | 0 | 0 |
| 965 | 0.00001 | 0 | 0.943972 | 0.056018 | 0 | 0 | 0 | 0 | 0 |
| 966 | 0.00001 | 0 | 0.942434 | 0.057556 | 0 | 0 | 0 | 0 | 0 |
| 967 | 0 | 0 | 0.945295 | 0.054705 | 0 | 0 | 0 | 0 | 0 |
| 968 | 0.00002 | 0 | 0.938597 | 0.061383 | 0 | 0 | 0 | 0 | 0 |
| 969 | 0.00002 | 0 | 0.938632 | 0.061348 | 0 | 0 | 0 | 0 | 0 |
| 970 | 0 | 0 | 0.93688 | 0.06312 | 0 | 0 | 0 | 0 | 0 |
| 971 | 0.00002 | 0 | 0.941396 | 0.058584 | 0 | 0 | 0 | 0 | 0 |
| 972 | 0.00001 | 0 | 0.93901 | 0.06098 | 0 | 0 | 0 | 0 | 0 |
| 973 | 0 | 0 | 0.941024 | 0.058976 | 0 | 0 | 0 | 0 | 0 |
| 974 | 0 | 0 | 0.936603 | 0.063397 | 0 | 0 | 0 | 0 | 0 |
| 975 | 0.00001 | 0 | 0.945137 | 0.054853 | 0 | 0 | 0 | 0 | 0 |
| 976 | 0 | 0 | 0.940672 | 0.059328 | 0 | 0 | 0 | 0 | 0 |
| 977 | 0.00001 | 0 | 0.939928 | 0.060062 | 0 | 0 | 0 | 0 | 0 |
| 978 | 0.00004 | 0 | 0.941008 | 0.058952 | 0 | 0 | 0 | 0 | 0 |
| 979 | 0 | 0 | 0.938327 | 0.061673 | 0 | 0 | 0 | 0 | 0 |
| 980 | 0.00002 | 0 | 0.941109 | 0.058871 | 0 | 0 | 0 | 0 | 0 |
| 981 | 0.00002 | 0 | 0.943401 | 0.056579 | 0 | 0 | 0 | 0 | 0 |
| 982 | 0.00002 | 0 | 0.935903 | 0.064077 | 0 | 0 | 0 | 0 | 0 |
| 983 | 0.00001 | 0 | 0.936339 | 0.063651 | 0 | 0 | 0 | 0 | 0 |
| 984 | 0.00004 | 0 | 0.940566 | 0.059394 | 0 | 0 | 0 | 0 | 0 |
| 985 | 0.00001 | 0 | 0.938597 | 0.061393 | 0 | 0 | 0 | 0 | 0 |
| 986 | 0.00002 | 0 | 0.941861 | 0.058119 | 0 | 0 | 0 | 0 | 0 |
| 987 | 0.00002 | 0 | 0.940579 | 0.059401 | 0 | 0 | 0 | 0 | 0 |
| 988 | 0.00001 | 0 | 0.939135 | 0.060855 | 0 | 0 | 0 | 0 | 0 |
| 989 | 0.00001 | 0 | 0.938232 | 0.061758 | 0 | 0 | 0 | 0 | 0 |
| 990 | 0.00001 | 0 | 0.937748 | 0.062242 | 0 | 0 | 0 | 0 | 0 |
| 991 | 0.00001 | 0 | 0.941073 | 0.058917 | 0 | 0 | 0 | 0 | 0 |
| 992 | 0.00001 | 0 | 0.94004 | 0.05995 | 0 | 0 | 0 | 0 | 0 |
| 993 | 0 | 0 | 0.944023 | 0.055977 | 0 | 0 | 0 | 0 | 0 |
| 994 | 0 | 0 | 0.945016 | 0.054984 | 0 | 0 | 0 | 0 | 0 |
| 995 | 0.00001 | 0 | 0.937059 | 0.062931 | 0 | 0 | 0 | 0 | 0 |
| 996 | 0.00003 | 0 | 0.942378 | 0.057592 | 0 | 0 | 0 | 0 | 0 |
| 997 | 0.00002 | 0 | 0.933863 | 0.066117 | 0 | 0 | 0 | 0 | 0 |
| 998 | 0.00002 | 0 | 0.939961 | 0.060019 | 0 | 0 | 0 | 0 | 0 |
| 999 | 0 | 0 | 0.943806 | 0.056194 | 0 | 0 | 0 | 0 | 0 |
| 1000 | 0.00001 | 0 | 0.938076 | 0.061914 | 0 | 0 | 0 | 0 | 0 |

| Year 15 | Race |  |  |  |  |  |  |  |  |
| --- | --- | --- | --- | --- | --- | --- | --- | --- | --- |
| Trial | 003.0 | 005.0 | 007.0 | 037.1 | 043.0 | 303.0 | 003.2 | 403.0 | 003.4 |
| 1 | 0.00003 | 0 | 0.944533 | 0.055437 | 0 | 0 | 0 | 0 | 0 |
| 2 | 0.00001 | 0 | 0.941651 | 0.058339 | 0 | 0 | 0 | 0 | 0 |
| 3 | 0 | 0 | 0.946117 | 0.053883 | 0 | 0 | 0 | 0 | 0 |
| 4 | 0 | 0 | 0.944319 | 0.055681 | 0 | 0 | 0 | 0 | 0 |
| 5 | 0 | 0 | 0.943877 | 0.056123 | 0 | 0 | 0 | 0 | 0 |
| 6 | 0.00001 | 0 | 0.945173 | 0.054817 | 0 | 0 | 0 | 0 | 0 |
| 7 | 0 | 0 | 0.944731 | 0.055269 | 0 | 0 | 0 | 0 | 0 |
| 8 | 0.00002 | 0 | 0.947083 | 0.052897 | 0 | 0 | 0 | 0 | 0 |
| 9 | 0 | 0 | 0.941108 | 0.058892 | 0 | 0 | 0 | 0 | 0 |
| 10 | 0 | 0 | 0.944452 | 0.055548 | 0 | 0 | 0 | 0 | 0 |
| 11 | 0.00005 | 0 | 0.948783 | 0.051167 | 0 | 0 | 0 | 0 | 0 |
| 12 | 0.00002 | 0 | 0.940776 | 0.059204 | 0 | 0 | 0 | 0 | 0 |
| 13 | 0.00002 | 0 | 0.944611 | 0.055369 | 0 | 0 | 0 | 0 | 0 |
| 14 | 0.00003 | 0 | 0.941443 | 0.058527 | 0 | 0 | 0 | 0 | 0 |
| 15 | 0 | 0 | 0.941923 | 0.058077 | 0 | 0 | 0 | 0 | 0 |
| 16 | 0.00001 | 0 | 0.9443 | 0.05569 | 0 | 0 | 0 | 0 | 0 |
| 17 | 0.00004 | 0 | 0.944693 | 0.055267 | 0 | 0 | 0 | 0 | 0 |
| 18 | 0.00001 | 0 | 0.945349 | 0.054641 | 0 | 0 | 0 | 0 | 0 |
| 19 | 0 | 0 | 0.942985 | 0.057015 | 0 | 0 | 0 | 0 | 0 |
| 20 | 0.00002 | 0 | 0.946735 | 0.053246 | 0 | 0 | 0 | 0 | 0 |
| 21 | 0.00001 | 0 | 0.941928 | 0.058062 | 0 | 0 | 0 | 0 | 0 |
| 22 | 0 | 0 | 0.94306 | 0.05694 | 0 | 0 | 0 | 0 | 0 |
| 23 | 0.00001 | 0 | 0.946502 | 0.053488 | 0 | 0 | 0 | 0 | 0 |
| 24 | 0.00001 | 0 | 0.946931 | 0.053059 | 0 | 0 | 0 | 0 | 0 |
| 25 | 0.00002 | 0 | 0.943843 | 0.056136 | 0 | 0 | 0 | 0 | 0 |
| 26 | 0.00001 | 0 | 0.945876 | 0.054114 | 0 | 0 | 0 | 0 | 0 |
| 27 | 0 | 0 | 0.943274 | 0.056726 | 0 | 0 | 0 | 0 | 0 |
| 28 | 0.00002 | 0 | 0.947075 | 0.051266 | 0.00164 | 0 | 0 | 0 | 0 |
| 29 | 0 | 0 | 0.946719 | 0.053281 | 0 | 0 | 0 | 0 | 0 |
| 30 | 0.00001 | 0 | 0.940672 | 0.059318 | 0 | 0 | 0 | 0 | 0 |
| 31 | 0.00001 | 0 | 0.947157 | 0.052833 | 0 | 0 | 0 | 0 | 0 |
| 32 | 0.00001 | 0 | 0.945066 | 0.054924 | 0 | 0 | 0 | 0 | 0 |
| 33 | 0.00002 | 0 | 0.943827 | 0.056152 | 0 | 0 | 0 | 0 | 0 |
| 34 | 0 | 0 | 0.945734 | 0.054266 | 0 | 0 | 0 | 0 | 0 |
| 35 | 0.00002 | 0 | 0.942296 | 0.057684 | 0 | 0 | 0 | 0 | 0 |
| 36 | 0.00001 | 0 | 0.942036 | 0.057954 | 0 | 0 | 0 | 0 | 0 |
| 37 | 0.00002 | 0 | 0.948684 | 0.051296 | 0 | 0 | 0 | 0 | 0 |
| 38 | 0.00003 | 0 | 0.944942 | 0.055028 | 0 | 0 | 0 | 0 | 0 |
| 39 | 0.00001 | 0 | 0.943235 | 0.056755 | 0 | 0 | 0 | 0 | 0 |
| 40 | 0.00001 | 0 | 0.943752 | 0.056238 | 0 | 0 | 0 | 0 | 0 |
| 41 | 0.00004 | 0 | 0.941468 | 0.058492 | 0 | 0 | 0 | 0 | 0 |
| 42 | 0 | 0 | 0.945323 | 0.054677 | 0 | 0 | 0 | 0 | 0 |
| 43 | 0.00004 | 0 | 0.944846 | 0.055114 | 0 | 0 | 0 | 0 | 0 |
| 44 | 0.00001 | 0 | 0.943386 | 0.056604 | 0 | 0 | 0 | 0 | 0 |
| 45 | 0 | 0 | 0.945552 | 0.054448 | 0 | 0 | 0 | 0 | 0 |
| 46 | 0 | 0 | 0.942967 | 0.057033 | 0 | 0 | 0 | 0 | 0 |
| 47 | 0.00001 | 0 | 0.945701 | 0.054289 | 0 | 0 | 0 | 0 | 0 |
| 48 | 0 | 0 | 0.94253 | 0.05747 | 0 | 0 | 0 | 0 | 0 |
| 49 | 0.00001 | 0 | 0.947799 | 0.052191 | 0 | 0 | 0 | 0 | 0 |
| 50 | 0.00001 | 0 | 0.944356 | 0.055635 | 0 | 0 | 0 | 0 | 0 |
| 51 | 0.00001 | 0 | 0.943894 | 0.056096 | 0 | 0 | 0 | 0 | 0 |
| 52 | 0.00002 | 0 | 0.946235 | 0.053745 | 0 | 0 | 0 | 0 | 0 |
| 53 | 0.00001 | 0 | 0.945243 | 0.054747 | 0 | 0 | 0 | 0 | 0 |
| 54 | 0.00001 | 0 | 0.943073 | 0.056917 | 0 | 0 | 0 | 0 | 0 |
| 55 | 0.00001 | 0 | 0.941684 | 0.058306 | 0 | 0 | 0 | 0 | 0 |
| 56 | 0.00001 | 0 | 0.943853 | 0.056137 | 0 | 0 | 0 | 0 | 0 |
| 57 | 0.00001 | 0 | 0.948685 | 0.051305 | 0 | 0 | 0 | 0 | 0 |
| 58 | 0.00002 | 0 | 0.944263 | 0.055717 | 0 | 0 | 0 | 0 | 0 |
| 59 | 0.00002 | 0 | 0.94561 | 0.05437 | 0 | 0 | 0 | 0 | 0 |
| 60 | 0.00002 | 0 | 0.943517 | 0.056463 | 0 | 0 | 0 | 0 | 0 |
| 61 | 0.00001 | 0 | 0.93848 | 0.06151 | 0 | 0 | 0 | 0 | 0 |
| 62 | 0 | 0 | 0.943667 | 0.056333 | 0 | 0 | 0 | 0 | 0 |
| 63 | 0 | 0 | 0.943334 | 0.056666 | 0 | 0 | 0 | 0 | 0 |
| 64 | 0 | 0 | 0.942878 | 0.057122 | 0 | 0 | 0 | 0 | 0 |
| 65 | 0.00002 | 0 | 0.947455 | 0.052524 | 0 | 0 | 0 | 0 | 0 |
| 66 | 0.00003 | 0 | 0.94776 | 0.05221 | 0 | 0 | 0 | 0 | 0 |
| 67 | 0.00001 | 0 | 0.944748 | 0.055242 | 0 | 0 | 0 | 0 | 0 |
| 68 | 0 | 0 | 0.947083 | 0.052917 | 0 | 0 | 0 | 0 | 0 |
| 69 | 0.00001 | 0 | 0.944803 | 0.055187 | 0 | 0 | 0 | 0 | 0 |
| 70 | 0.00002 | 0 | 0.94403 | 0.05595 | 0 | 0 | 0 | 0 | 0 |
| 71 | 0.00002 | 0 | 0.938941 | 0.054402 | 0.006637 | 0 | 0 | 0 | 0 |
| 72 | 0.00001 | 0 | 0.945822 | 0.054168 | 0 | 0 | 0 | 0 | 0 |
| 73 | 0.00001 | 0 | 0.944844 | 0.055146 | 0 | 0 | 0 | 0 | 0 |
| 74 | 0.00001 | 0 | 0.947672 | 0.052318 | 0 | 0 | 0 | 0 | 0 |
| 75 | 0 | 0 | 0.944322 | 0.055678 | 0 | 0 | 0 | 0 | 0 |
| 76 | 0.00001 | 0 | 0.946727 | 0.053263 | 0 | 0 | 0 | 0 | 0 |
| 77 | 0.00001 | 0 | 0.94513 | 0.05486 | 0 | 0 | 0 | 0 | 0 |
| 78 | 0.00003 | 0 | 0.945471 | 0.054499 | 0 | 0 | 0 | 0 | 0 |
| 79 | 0 | 0 | 0.945502 | 0.054498 | 0 | 0 | 0 | 0 | 0 |
| 80 | 0.00002 | 0 | 0.944144 | 0.055836 | 0 | 0 | 0 | 0 | 0 |
| 81 | 0.00001 | 0 | 0.947624 | 0.052366 | 0 | 0 | 0 | 0 | 0 |
| 82 | 0 | 0 | 0.938954 | 0.061046 | 0 | 0 | 0 | 0 | 0 |
| 83 | 0.00002 | 0 | 0.948179 | 0.051801 | 0 | 0 | 0 | 0 | 0 |
| 84 | 0 | 0 | 0.945054 | 0.054946 | 0 | 0 | 0 | 0 | 0 |
| 85 | 0.00001 | 0 | 0.942995 | 0.056995 | 0 | 0 | 0 | 0 | 0 |
| 86 | 0.00001 | 0 | 0.950453 | 0.049537 | 0 | 0 | 0 | 0 | 0 |
| 87 | 0 | 0 | 0.942821 | 0.057179 | 0 | 0 | 0 | 0 | 0 |
| 88 | 0.00004 | 0 | 0.944025 | 0.055935 | 0 | 0 | 0 | 0 | 0 |
| 89 | 0.00001 | 0 | 0.943457 | 0.056533 | 0 | 0 | 0 | 0 | 0 |
| 90 | 0 | 0 | 0.945069 | 0.054931 | 0 | 0 | 0 | 0 | 0 |
| 91 | 0.00002 | 0 | 0.944924 | 0.055056 | 0 | 0 | 0 | 0 | 0 |
| 92 | 0.00002 | 0 | 0.945238 | 0.054742 | 0 | 0 | 0 | 0 | 0 |
| 93 | 0 | 0 | 0.94708 | 0.05292 | 0 | 0 | 0 | 0 | 0 |
| 94 | 0 | 0 | 0.947615 | 0.052385 | 0 | 0 | 0 | 0 | 0 |
| 95 | 0.00002 | 0 | 0.944575 | 0.055405 | 0 | 0 | 0 | 0 | 0 |
| 96 | 0.00002 | 0 | 0.945312 | 0.054668 | 0 | 0 | 0 | 0 | 0 |
| 97 | 0.00002 | 0 | 0.94359 | 0.056389 | 0 | 0 | 0 | 0 | 0 |
| 98 | 0.00002 | 0 | 0.94389 | 0.05609 | 0 | 0 | 0 | 0 | 0 |
| 99 | 0.00002 | 0 | 0.941479 | 0.058501 | 0 | 0 | 0 | 0 | 0 |
| 100 | 0.00001 | 0 | 0.948382 | 0.051609 | 0 | 0 | 0 | 0 | 0 |
| 101 | 0.00002 | 0 | 0.947479 | 0.052501 | 0 | 0 | 0 | 0 | 0 |
| 102 | 0.00002 | 0 | 0.946237 | 0.053743 | 0 | 0 | 0 | 0 | 0 |
| 103 | 0 | 0 | 0.943899 | 0.056101 | 0 | 0 | 0 | 0 | 0 |
| 104 | 0.00001 | 0 | 0.944766 | 0.055224 | 0 | 0 | 0 | 0 | 0 |
| 105 | 0.00002 | 0 | 0.94642 | 0.05356 | 0 | 0 | 0 | 0 | 0 |
| 106 | 0 | 0 | 0.946224 | 0.053776 | 0 | 0 | 0 | 0 | 0 |
| 107 | 0 | 0 | 0.946135 | 0.051423 | 0.002442 | 0 | 0 | 0 | 0 |
| 108 | 0.00002 | 0 | 0.940884 | 0.059096 | 0 | 0 | 0 | 0 | 0 |
| 109 | 0 | 0 | 0.94615 | 0.05385 | 0 | 0 | 0 | 0 | 0 |
| 110 | 0.00002 | 0 | 0.943329 | 0.056651 | 0 | 0 | 0 | 0 | 0 |
| 111 | 0.00001 | 0 | 0.945285 | 0.054705 | 0 | 0 | 0 | 0 | 0 |
| 112 | 0.00004 | 0 | 0.942793 | 0.057167 | 0 | 0 | 0 | 0 | 0 |
| 113 | 0.00002 | 0 | 0.943446 | 0.056534 | 0 | 0 | 0 | 0 | 0 |
| 114 | 0.00002 | 0 | 0.945612 | 0.054368 | 0 | 0 | 0 | 0 | 0 |
| 115 | 0.00001 | 0 | 0.94786 | 0.05213 | 0 | 0 | 0 | 0 | 0 |
| 116 | 0.00002 | 0 | 0.943631 | 0.056349 | 0 | 0 | 0 | 0 | 0 |
| 117 | 0 | 0 | 0.944388 | 0.055612 | 0 | 0 | 0 | 0 | 0 |
| 118 | 0 | 0 | 0.946381 | 0.053619 | 0 | 0 | 0 | 0 | 0 |
| 119 | 0.00001 | 0 | 0.945589 | 0.054401 | 0 | 0 | 0 | 0 | 0 |
| 120 | 0.00001 | 0 | 0.943346 | 0.056644 | 0 | 0 | 0 | 0 | 0 |
| 121 | 0.00002 | 0 | 0.943475 | 0.056505 | 0 | 0 | 0 | 0 | 0 |
| 122 | 0.00001 | 0 | 0.9491 | 0.05089 | 0 | 0 | 0 | 0 | 0 |
| 123 | 0.00001 | 0 | 0.945241 | 0.054749 | 0 | 0 | 0 | 0 | 0 |
| 124 | 0.00001 | 0 | 0.946608 | 0.053382 | 0 | 0 | 0 | 0 | 0 |
| 125 | 0.00001 | 0 | 0.943718 | 0.056272 | 0 | 0 | 0 | 0 | 0 |
| 126 | 0.00002 | 0 | 0.946578 | 0.053402 | 0 | 0 | 0 | 0 | 0 |
| 127 | 0.00001 | 0 | 0.944383 | 0.055607 | 0 | 0 | 0 | 0 | 0 |
| 128 | 0.00001 | 0 | 0.941675 | 0.058315 | 0 | 0 | 0 | 0 | 0 |
| 129 | 0.00002 | 0 | 0.945105 | 0.054875 | 0 | 0 | 0 | 0 | 0 |
| 130 | 0.00002 | 0 | 0.946107 | 0.053873 | 0 | 0 | 0 | 0 | 0 |
| 131 | 0 | 0 | 0.946019 | 0.053981 | 0 | 0 | 0 | 0 | 0 |
| 132 | 0 | 0 | 0.947123 | 0.052877 | 0 | 0 | 0 | 0 | 0 |
| 133 | 0.00001 | 0 | 0.942676 | 0.057314 | 0 | 0 | 0 | 0 | 0 |
| 134 | 0.00002 | 0 | 0.946671 | 0.053309 | 0 | 0 | 0 | 0 | 0 |
| 135 | 0.00002 | 0 | 0.942896 | 0.057084 | 0 | 0 | 0 | 0 | 0 |
| 136 | 0.00002 | 0 | 0.94375 | 0.05623 | 0 | 0 | 0 | 0 | 0 |
| 137 | 0 | 0 | 0.946713 | 0.053287 | 0 | 0 | 0 | 0 | 0 |
| 138 | 0.00002 | 0 | 0.941567 | 0.058413 | 0 | 0 | 0 | 0 | 0 |
| 139 | 0.00002 | 0 | 0.94644 | 0.05354 | 0 | 0 | 0 | 0 | 0 |
| 140 | 0.00002 | 0 | 0.942472 | 0.057508 | 0 | 0 | 0 | 0 | 0 |
| 141 | 0.00001 | 0 | 0.944127 | 0.055863 | 0 | 0 | 0 | 0 | 0 |
| 142 | 0 | 0 | 0.941104 | 0.058896 | 0 | 0 | 0 | 0 | 0 |
| 143 | 0.00001 | 0 | 0.944119 | 0.055871 | 0 | 0 | 0 | 0 | 0 |
| 144 | 0.00002 | 0 | 0.943399 | 0.05658 | 0 | 0 | 0 | 0 | 0 |
| 145 | 0.00001 | 0 | 0.945794 | 0.054196 | 0 | 0 | 0 | 0 | 0 |
| 146 | 0.00001 | 0 | 0.948616 | 0.051374 | 0 | 0 | 0 | 0 | 0 |
| 147 | 0.00001 | 0 | 0.943961 | 0.056029 | 0 | 0 | 0 | 0 | 0 |
| 148 | 0.00001 | 0 | 0.945316 | 0.054674 | 0 | 0 | 0 | 0 | 0 |
| 149 | 0 | 0 | 0.942938 | 0.057062 | 0 | 0 | 0 | 0 | 0 |
| 150 | 0.00004 | 0 | 0.94755 | 0.05241 | 0 | 0 | 0 | 0 | 0 |
| 151 | 0 | 0 | 0.946262 | 0.053738 | 0 | 0 | 0 | 0 | 0 |
| 152 | 0 | 0 | 0.940295 | 0.059705 | 0 | 0 | 0 | 0 | 0 |
| 153 | 0.00001 | 0 | 0.94813 | 0.05186 | 0 | 0 | 0 | 0 | 0 |
| 154 | 0.00001 | 0 | 0.946184 | 0.053806 | 0 | 0 | 0 | 0 | 0 |
| 155 | 0.00002 | 0 | 0.9441 | 0.05588 | 0 | 0 | 0 | 0 | 0 |
| 156 | 0.00002 | 0 | 0.946004 | 0.053976 | 0 | 0 | 0 | 0 | 0 |
| 157 | 0.00001 | 0 | 0.942791 | 0.057199 | 0 | 0 | 0 | 0 | 0 |
| 158 | 0 | 0 | 0.940244 | 0.059756 | 0 | 0 | 0 | 0 | 0 |
| 159 | 0.00002 | 0 | 0.944289 | 0.055691 | 0 | 0 | 0 | 0 | 0 |
| 160 | 0.00002 | 0 | 0.944346 | 0.055634 | 0 | 0 | 0 | 0 | 0 |
| 161 | 0 | 0 | 0.941242 | 0.058758 | 0 | 0 | 0 | 0 | 0 |
| 162 | 0.00001 | 0 | 0.944853 | 0.055137 | 0 | 0 | 0 | 0 | 0 |
| 163 | 0 | 0 | 0.942781 | 0.057219 | 0 | 0 | 0 | 0 | 0 |
| 164 | 0 | 0 | 0.938901 | 0.061099 | 0 | 0 | 0 | 0 | 0 |
| 165 | 0 | 0 | 0.949729 | 0.050271 | 0 | 0 | 0 | 0 | 0 |
| 166 | 0.00001 | 0 | 0.944564 | 0.055426 | 0 | 0 | 0 | 0 | 0 |
| 167 | 0 | 0 | 0.943702 | 0.056298 | 0 | 0 | 0 | 0 | 0 |
| 168 | 0.00002 | 0 | 0.943419 | 0.056561 | 0 | 0 | 0 | 0 | 0 |
| 169 | 0 | 0 | 0.941008 | 0.058992 | 0 | 0 | 0 | 0 | 0 |
| 170 | 0.00003 | 0 | 0.943223 | 0.056747 | 0 | 0 | 0 | 0 | 0 |
| 171 | 0.00001 | 0 | 0.946447 | 0.053543 | 0 | 0 | 0 | 0 | 0 |
| 172 | 0 | 0 | 0.943072 | 0.056928 | 0 | 0 | 0 | 0 | 0 |
| 173 | 0 | 0 | 0.944853 | 0.055147 | 0 | 0 | 0 | 0 | 0 |
| 174 | 0.00001 | 0 | 0.942157 | 0.057833 | 0 | 0 | 0 | 0 | 0 |
| 175 | 0 | 0 | 0.941757 | 0.058243 | 0 | 0 | 0 | 0 | 0 |
| 176 | 0 | 0 | 0.946073 | 0.053927 | 0 | 0 | 0 | 0 | 0 |
| 177 | 0.00001 | 0 | 0.947882 | 0.052108 | 0 | 0 | 0 | 0 | 0 |
| 178 | 0.00002 | 0 | 0.946805 | 0.053176 | 0 | 0 | 0 | 0 | 0 |
| 179 | 0.00002 | 0 | 0.94608 | 0.0539 | 0 | 0 | 0 | 0 | 0 |
| 180 | 0.00001 | 0 | 0.946357 | 0.053633 | 0 | 0 | 0 | 0 | 0 |
| 181 | 0.00003 | 0 | 0.945848 | 0.054122 | 0 | 0 | 0 | 0 | 0 |
| 182 | 0 | 0 | 0.941047 | 0.058953 | 0 | 0 | 0 | 0 | 0 |
| 183 | 0 | 0 | 0.948511 | 0.051489 | 0 | 0 | 0 | 0 | 0 |
| 184 | 0.00003 | 0 | 0.946394 | 0.053576 | 0 | 0 | 0 | 0 | 0 |
| 185 | 0.00002 | 0 | 0.947005 | 0.052975 | 0 | 0 | 0 | 0 | 0 |
| 186 | 0 | 0 | 0.94668 | 0.05332 | 0 | 0 | 0 | 0 | 0 |
| 187 | 0 | 0 | 0.950267 | 0.049733 | 0 | 0 | 0 | 0 | 0 |
| 188 | 0.00003 | 0 | 0.94497 | 0.055 | 0 | 0 | 0 | 0 | 0 |
| 189 | 0 | 0 | 0.942695 | 0.057305 | 0 | 0 | 0 | 0 | 0 |
| 190 | 0.00002 | 0 | 0.944306 | 0.055675 | 0 | 0 | 0 | 0 | 0 |
| 191 | 0 | 0 | 0.947485 | 0.052515 | 0 | 0 | 0 | 0 | 0 |
| 192 | 0.00001 | 0 | 0.946239 | 0.053751 | 0 | 0 | 0 | 0 | 0 |
| 193 | 0.00001 | 0 | 0.941361 | 0.058629 | 0 | 0 | 0 | 0 | 0 |
| 194 | 0.00003 | 0 | 0.941103 | 0.058867 | 0 | 0 | 0 | 0 | 0 |
| 195 | 0.00001 | 0 | 0.94142 | 0.05857 | 0 | 0 | 0 | 0 | 0 |
| 196 | 0.00001 | 0 | 0.941475 | 0.058515 | 0 | 0 | 0 | 0 | 0 |
| 197 | 0.00001 | 0 | 0.949971 | 0.050019 | 0 | 0 | 0 | 0 | 0 |
| 198 | 0.00001 | 0 | 0.940194 | 0.053116 | 0.00668 | 0 | 0 | 0 | 0 |
| 199 | 0.00001 | 0 | 0.946152 | 0.053838 | 0 | 0 | 0 | 0 | 0 |
| 200 | 0.00001 | 0 | 0.941595 | 0.058395 | 0 | 0 | 0 | 0 | 0 |
| 201 | 0.00002 | 0 | 0.944183 | 0.055797 | 0 | 0 | 0 | 0 | 0 |
| 202 | 0 | 0 | 0.945609 | 0.054391 | 0 | 0 | 0 | 0 | 0 |
| 203 | 0 | 0 | 0.946542 | 0.053458 | 0 | 0 | 0 | 0 | 0 |
| 204 | 0.00002 | 0 | 0.945276 | 0.054704 | 0 | 0 | 0 | 0 | 0 |
| 205 | 0 | 0 | 0.940864 | 0.059136 | 0 | 0 | 0 | 0 | 0 |
| 206 | 0.00002 | 0 | 0.938927 | 0.061053 | 0 | 0 | 0 | 0 | 0 |
| 207 | 0.00002 | 0 | 0.947892 | 0.052088 | 0 | 0 | 0 | 0 | 0 |
| 208 | 0.00002 | 0 | 0.946644 | 0.053336 | 0 | 0 | 0 | 0 | 0 |
| 209 | 0.00003 | 0 | 0.943329 | 0.056641 | 0 | 0 | 0 | 0 | 0 |
| 210 | 0 | 0 | 0.946689 | 0.053311 | 0 | 0 | 0 | 0 | 0 |
| 211 | 0 | 0 | 0.943959 | 0.056041 | 0 | 0 | 0 | 0 | 0 |
| 212 | 0.00001 | 0 | 0.94093 | 0.05906 | 0 | 0 | 0 | 0 | 0 |
| 213 | 0.00001 | 0 | 0.946038 | 0.053952 | 0 | 0 | 0 | 0 | 0 |
| 214 | 0.00001 | 0 | 0.944135 | 0.055855 | 0 | 0 | 0 | 0 | 0 |
| 215 | 0.00001 | 0 | 0.944762 | 0.055228 | 0 | 0 | 0 | 0 | 0 |
| 216 | 0.00001 | 0 | 0.947071 | 0.052919 | 0 | 0 | 0 | 0 | 0 |
| 217 | 0 | 0 | 0.945704 | 0.054296 | 0 | 0 | 0 | 0 | 0 |
| 218 | 0.00002 | 0 | 0.948689 | 0.051291 | 0 | 0 | 0 | 0 | 0 |
| 219 | 0.00001 | 0 | 0.949835 | 0.050155 | 0 | 0 | 0 | 0 | 0 |
| 220 | 0 | 0 | 0.943606 | 0.056394 | 0 | 0 | 0 | 0 | 0 |
| 221 | 0.00003 | 0 | 0.945522 | 0.054448 | 0 | 0 | 0 | 0 | 0 |
| 222 | 0.00001 | 0 | 0.944492 | 0.055498 | 0 | 0 | 0 | 0 | 0 |
| 223 | 0.00001 | 0 | 0.944815 | 0.055175 | 0 | 0 | 0 | 0 | 0 |
| 224 | 0 | 0 | 0.94458 | 0.05542 | 0 | 0 | 0 | 0 | 0 |
| 225 | 0.00001 | 0 | 0.947089 | 0.052901 | 0 | 0 | 0 | 0 | 0 |
| 226 | 0 | 0 | 0.942124 | 0.057876 | 0 | 0 | 0 | 0 | 0 |
| 227 | 0.00001 | 0 | 0.946857 | 0.053133 | 0 | 0 | 0 | 0 | 0 |
| 228 | 0.00002 | 0 | 0.943357 | 0.056623 | 0 | 0 | 0 | 0 | 0 |
| 229 | 0 | 0 | 0.943693 | 0.056307 | 0 | 0 | 0 | 0 | 0 |
| 230 | 0.00002 | 0 | 0.94527 | 0.05471 | 0 | 0 | 0 | 0 | 0 |
| 231 | 0.00001 | 0 | 0.949077 | 0.050913 | 0 | 0 | 0 | 0 | 0 |
| 232 | 0.00001 | 0 | 0.942452 | 0.057538 | 0 | 0 | 0 | 0 | 0 |
| 233 | 0 | 0 | 0.943317 | 0.056683 | 0 | 0 | 0 | 0 | 0 |
| 234 | 0 | 0 | 0.944836 | 0.055164 | 0 | 0 | 0 | 0 | 0 |
| 235 | 0 | 0 | 0.94707 | 0.05293 | 0 | 0 | 0 | 0 | 0 |
| 236 | 0.00002 | 0 | 0.941058 | 0.058922 | 0 | 0 | 0 | 0 | 0 |
| 237 | 0.00001 | 0 | 0.944816 | 0.055174 | 0 | 0 | 0 | 0 | 0 |
| 238 | 0.00002 | 0 | 0.939037 | 0.060943 | 0 | 0 | 0 | 0 | 0 |
| 239 | 0.00001 | 0 | 0.940944 | 0.059046 | 0 | 0 | 0 | 0 | 0 |
| 240 | 0.00001 | 0 | 0.946799 | 0.053191 | 0 | 0 | 0 | 0 | 0 |
| 241 | 0.00001 | 0 | 0.946878 | 0.053112 | 0 | 0 | 0 | 0 | 0 |
| 242 | 0 | 0 | 0.947466 | 0.052534 | 0 | 0 | 0 | 0 | 0 |
| 243 | 0.00002 | 0 | 0.946295 | 0.053685 | 0 | 0 | 0 | 0 | 0 |
| 244 | 0 | 0 | 0.945049 | 0.054951 | 0 | 0 | 0 | 0 | 0 |
| 245 | 0 | 0 | 0.944273 | 0.054312 | 0.001415 | 0 | 0 | 0 | 0 |
| 246 | 0 | 0 | 0.940019 | 0.059981 | 0 | 0 | 0 | 0 | 0 |
| 247 | 0 | 0 | 0.943918 | 0.056082 | 0 | 0 | 0 | 0 | 0 |
| 248 | 0 | 0 | 0.94591 | 0.05409 | 0 | 0 | 0 | 0 | 0 |
| 249 | 0.00001 | 0 | 0.947884 | 0.052106 | 0 | 0 | 0 | 0 | 0 |
| 250 | 0.00001 | 0 | 0.943772 | 0.056218 | 0 | 0 | 0 | 0 | 0 |
| 251 | 0.00002 | 0 | 0.946604 | 0.053376 | 0 | 0 | 0 | 0 | 0 |
| 252 | 0 | 0 | 0.94635 | 0.05365 | 0 | 0 | 0 | 0 | 0 |
| 253 | 0 | 0 | 0.945796 | 0.054204 | 0 | 0 | 0 | 0 | 0 |
| 254 | 0 | 0 | 0.945457 | 0.054543 | 0 | 0 | 0 | 0 | 0 |
| 255 | 0.00002 | 0 | 0.94489 | 0.05509 | 0 | 0 | 0 | 0 | 0 |
| 256 | 0 | 0 | 0.941803 | 0.058197 | 0 | 0 | 0 | 0 | 0 |
| 257 | 0.00002 | 0 | 0.944572 | 0.055408 | 0 | 0 | 0 | 0 | 0 |
| 258 | 0.00003 | 0 | 0.941546 | 0.058424 | 0 | 0 | 0 | 0 | 0 |
| 259 | 0.00002 | 0 | 0.943055 | 0.056925 | 0 | 0 | 0 | 0 | 0 |
| 260 | 0.00001 | 0 | 0.947868 | 0.052122 | 0 | 0 | 0 | 0 | 0 |
| 261 | 0.00002 | 0 | 0.94664 | 0.053341 | 0 | 0 | 0 | 0 | 0 |
| 262 | 0.00002 | 0 | 0.945016 | 0.054964 | 0 | 0 | 0 | 0 | 0 |
| 263 | 0.00001 | 0 | 0.945023 | 0.054967 | 0 | 0 | 0 | 0 | 0 |
| 264 | 0 | 0 | 0.939555 | 0.060445 | 0 | 0 | 0 | 0 | 0 |
| 265 | 0.00001 | 0 | 0.94305 | 0.05694 | 0 | 0 | 0 | 0 | 0 |
| 266 | 0.00003 | 0 | 0.946355 | 0.053615 | 0 | 0 | 0 | 0 | 0 |
| 267 | 0 | 0 | 0.944894 | 0.055106 | 0 | 0 | 0 | 0 | 0 |
| 268 | 0 | 0 | 0.948401 | 0.051599 | 0 | 0 | 0 | 0 | 0 |
| 269 | 0.00001 | 0 | 0.946643 | 0.053347 | 0 | 0 | 0 | 0 | 0 |
| 270 | 0.00001 | 0 | 0.946153 | 0.053838 | 0 | 0 | 0 | 0 | 0 |
| 271 | 0.00001 | 0 | 0.945429 | 0.054561 | 0 | 0 | 0 | 0 | 0 |
| 272 | 0.00001 | 0 | 0.94736 | 0.05263 | 0 | 0 | 0 | 0 | 0 |
| 273 | 0.00001 | 0 | 0.946924 | 0.053066 | 0 | 0 | 0 | 0 | 0 |
| 274 | 0 | 0 | 0.950347 | 0.049653 | 0 | 0 | 0 | 0 | 0 |
| 275 | 0 | 0 | 0.946815 | 0.053185 | 0 | 0 | 0 | 0 | 0 |
| 276 | 0 | 0 | 0.942893 | 0.057107 | 0 | 0 | 0 | 0 | 0 |
| 277 | 0.00001 | 0 | 0.939555 | 0.060435 | 0 | 0 | 0 | 0 | 0 |
| 278 | 0.00001 | 0 | 0.946316 | 0.053674 | 0 | 0 | 0 | 0 | 0 |
| 279 | 0 | 0 | 0.946117 | 0.053883 | 0 | 0 | 0 | 0 | 0 |
| 280 | 0 | 0 | 0.943295 | 0.056705 | 0 | 0 | 0 | 0 | 0 |
| 281 | 0 | 0 | 0.943399 | 0.056601 | 0 | 0 | 0 | 0 | 0 |
| 282 | 0.00001 | 0 | 0.944591 | 0.055399 | 0 | 0 | 0 | 0 | 0 |
| 283 | 0.00002 | 0 | 0.94729 | 0.05269 | 0 | 0 | 0 | 0 | 0 |
| 284 | 0 | 0 | 0.947129 | 0.052871 | 0 | 0 | 0 | 0 | 0 |
| 285 | 0.00001 | 0 | 0.943952 | 0.056038 | 0 | 0 | 0 | 0 | 0 |
| 286 | 0.00002 | 0 | 0.949476 | 0.050505 | 0 | 0 | 0 | 0 | 0 |
| 287 | 0.00003 | 0 | 0.942845 | 0.057124 | 0 | 0 | 0 | 0 | 0 |
| 288 | 0 | 0 | 0.942982 | 0.057018 | 0 | 0 | 0 | 0 | 0 |
| 289 | 0 | 0 | 0.946513 | 0.053487 | 0 | 0 | 0 | 0 | 0 |
| 290 | 0 | 0 | 0.946325 | 0.053675 | 0 | 0 | 0 | 0 | 0 |
| 291 | 0.00002 | 0 | 0.944089 | 0.055891 | 0 | 0 | 0 | 0 | 0 |
| 292 | 0.00001 | 0 | 0.943553 | 0.056437 | 0 | 0 | 0 | 0 | 0 |
| 293 | 0 | 0 | 0.944239 | 0.055761 | 0 | 0 | 0 | 0 | 0 |
| 294 | 0 | 0 | 0.942488 | 0.057512 | 0 | 0 | 0 | 0 | 0 |
| 295 | 0 | 0 | 0.947726 | 0.052274 | 0 | 0 | 0 | 0 | 0 |
| 296 | 0.00004 | 0 | 0.945035 | 0.054925 | 0 | 0 | 0 | 0 | 0 |
| 297 | 0 | 0 | 0.943519 | 0.056481 | 0 | 0 | 0 | 0 | 0 |
| 298 | 0 | 0 | 0.945573 | 0.054427 | 0 | 0 | 0 | 0 | 0 |
| 299 | 0.00001 | 0 | 0.940911 | 0.059079 | 0 | 0 | 0 | 0 | 0 |
| 300 | 0.00004 | 0 | 0.946434 | 0.053525 | 0 | 0 | 0 | 0 | 0 |
| 301 | 0.00002 | 0 | 0.945298 | 0.054682 | 0 | 0 | 0 | 0 | 0 |
| 302 | 0 | 0 | 0.942456 | 0.057544 | 0 | 0 | 0 | 0 | 0 |
| 303 | 0.00001 | 0 | 0.947059 | 0.052931 | 0 | 0 | 0 | 0 | 0 |
| 304 | 0 | 0 | 0.942756 | 0.057244 | 0 | 0 | 0 | 0 | 0 |
| 305 | 0.00002 | 0 | 0.946291 | 0.053689 | 0 | 0 | 0 | 0 | 0 |
| 306 | 0 | 0 | 0.947644 | 0.052356 | 0 | 0 | 0 | 0 | 0 |
| 307 | 0.00002 | 0 | 0.946267 | 0.053712 | 0 | 0 | 0 | 0 | 0 |
| 308 | 0.00001 | 0 | 0.949458 | 0.050532 | 0 | 0 | 0 | 0 | 0 |
| 309 | 0.00002 | 0 | 0.947617 | 0.052363 | 0 | 0 | 0 | 0 | 0 |
| 310 | 0.00001 | 0 | 0.942664 | 0.057326 | 0 | 0 | 0 | 0 | 0 |
| 311 | 0.00002 | 0 | 0.94592 | 0.05406 | 0 | 0 | 0 | 0 | 0 |
| 312 | 0 | 0 | 0.942722 | 0.057278 | 0 | 0 | 0 | 0 | 0 |
| 313 | 0 | 0 | 0.946301 | 0.053699 | 0 | 0 | 0 | 0 | 0 |
| 314 | 0.00004 | 0 | 0.94502 | 0.05494 | 0 | 0 | 0 | 0 | 0 |
| 315 | 0 | 0 | 0.944403 | 0.055597 | 0 | 0 | 0 | 0 | 0 |
| 316 | 0.00001 | 0 | 0.94395 | 0.05604 | 0 | 0 | 0 | 0 | 0 |
| 317 | 0.00001 | 0 | 0.941993 | 0.057997 | 0 | 0 | 0 | 0 | 0 |
| 318 | 0.00001 | 0 | 0.940865 | 0.056435 | 0.00269 | 0 | 0 | 0 | 0 |
| 319 | 0.00002 | 0 | 0.945311 | 0.054669 | 0 | 0 | 0 | 0 | 0 |
| 320 | 0.00001 | 0 | 0.946369 | 0.053621 | 0 | 0 | 0 | 0 | 0 |
| 321 | 0 | 0 | 0.945724 | 0.054276 | 0 | 0 | 0 | 0 | 0 |
| 322 | 0.00004 | 0 | 0.942427 | 0.057533 | 0 | 0 | 0 | 0 | 0 |
| 323 | 0.00001 | 0 | 0.946554 | 0.053436 | 0 | 0 | 0 | 0 | 0 |
| 324 | 0.00001 | 0 | 0.94758 | 0.05241 | 0 | 0 | 0 | 0 | 0 |
| 325 | 0.00004 | 0 | 0.945834 | 0.054126 | 0 | 0 | 0 | 0 | 0 |
| 326 | 0 | 0 | 0.94586 | 0.05414 | 0 | 0 | 0 | 0 | 0 |
| 327 | 0 | 0 | 0.94473 | 0.05527 | 0 | 0 | 0 | 0 | 0 |
| 328 | 0.00001 | 0 | 0.944184 | 0.055806 | 0 | 0 | 0 | 0 | 0 |
| 329 | 0.00001 | 0 | 0.943295 | 0.056695 | 0 | 0 | 0 | 0 | 0 |
| 330 | 0.00002 | 0 | 0.94547 | 0.05451 | 0 | 0 | 0 | 0 | 0 |
| 331 | 0 | 0 | 0.944139 | 0.055861 | 0 | 0 | 0 | 0 | 0 |
| 332 | 0.00004 | 0 | 0.944746 | 0.055214 | 0 | 0 | 0 | 0 | 0 |
| 333 | 0.00001 | 0 | 0.940921 | 0.059069 | 0 | 0 | 0 | 0 | 0 |
| 334 | 0.00001 | 0 | 0.945266 | 0.054724 | 0 | 0 | 0 | 0 | 0 |
| 335 | 0.00001 | 0 | 0.946186 | 0.053804 | 0 | 0 | 0 | 0 | 0 |
| 336 | 0.00001 | 0 | 0.938838 | 0.061152 | 0 | 0 | 0 | 0 | 0 |
| 337 | 0.00001 | 0 | 0.946209 | 0.053781 | 0 | 0 | 0 | 0 | 0 |
| 338 | 0 | 0 | 0.945669 | 0.054331 | 0 | 0 | 0 | 0 | 0 |
| 339 | 0.00001 | 0 | 0.942981 | 0.057008 | 0 | 0 | 0 | 0 | 0 |
| 340 | 0 | 0 | 0.942642 | 0.057358 | 0 | 0 | 0 | 0 | 0 |
| 341 | 0.00004 | 0 | 0.943034 | 0.056926 | 0 | 0 | 0 | 0 | 0 |
| 342 | 0 | 0 | 0.942759 | 0.057241 | 0 | 0 | 0 | 0 | 0 |
| 343 | 0.00001 | 0 | 0.943513 | 0.056477 | 0 | 0 | 0 | 0 | 0 |
| 344 | 0.00001 | 0 | 0.941238 | 0.058752 | 0 | 0 | 0 | 0 | 0 |
| 345 | 0.00001 | 0 | 0.947653 | 0.052337 | 0 | 0 | 0 | 0 | 0 |
| 346 | 0 | 0 | 0.950572 | 0.049428 | 0 | 0 | 0 | 0 | 0 |
| 347 | 0 | 0 | 0.940138 | 0.059862 | 0 | 0 | 0 | 0 | 0 |
| 348 | 0.00001 | 0 | 0.944489 | 0.055501 | 0 | 0 | 0 | 0 | 0 |
| 349 | 0.00003 | 0 | 0.94516 | 0.05481 | 0 | 0 | 0 | 0 | 0 |
| 350 | 0.00002 | 0 | 0.946381 | 0.053599 | 0 | 0 | 0 | 0 | 0 |
| 351 | 0.00003 | 0 | 0.946822 | 0.053148 | 0 | 0 | 0 | 0 | 0 |
| 352 | 0.00001 | 0 | 0.946895 | 0.053095 | 0 | 0 | 0 | 0 | 0 |
| 353 | 0 | 0 | 0.94469 | 0.05531 | 0 | 0 | 0 | 0 | 0 |
| 354 | 0 | 0 | 0.94463 | 0.05537 | 0 | 0 | 0 | 0 | 0 |
| 355 | 0 | 0 | 0.945861 | 0.054139 | 0 | 0 | 0 | 0 | 0 |
| 356 | 0.00001 | 0 | 0.945596 | 0.054394 | 0 | 0 | 0 | 0 | 0 |
| 357 | 0.00001 | 0 | 0.941216 | 0.058774 | 0 | 0 | 0 | 0 | 0 |
| 358 | 0 | 0 | 0.942679 | 0.057321 | 0 | 0 | 0 | 0 | 0 |
| 359 | 0.00001 | 0 | 0.943342 | 0.056648 | 0 | 0 | 0 | 0 | 0 |
| 360 | 0 | 0 | 0.943286 | 0.056714 | 0 | 0 | 0 | 0 | 0 |
| 361 | 0.00001 | 0 | 0.943414 | 0.056576 | 0 | 0 | 0 | 0 | 0 |
| 362 | 0.00001 | 0 | 0.948895 | 0.051095 | 0 | 0 | 0 | 0 | 0 |
| 363 | 0 | 0.00001 | 0.946947 | 0.053043 | 0 | 0 | 0 | 0 | 0 |
| 364 | 0 | 0 | 0.945096 | 0.054904 | 0 | 0 | 0 | 0 | 0 |
| 365 | 0.00001 | 0 | 0.946429 | 0.053561 | 0 | 0 | 0 | 0 | 0 |
| 366 | 0 | 0 | 0.945134 | 0.054866 | 0 | 0 | 0 | 0 | 0 |
| 367 | 0 | 0 | 0.946 | 0.054 | 0 | 0 | 0 | 0 | 0 |
| 368 | 0.00001 | 0 | 0.946862 | 0.053128 | 0 | 0 | 0 | 0 | 0 |
| 369 | 0 | 0 | 0.943152 | 0.056848 | 0 | 0 | 0 | 0 | 0 |
| 370 | 0 | 0 | 0.943641 | 0.056359 | 0 | 0 | 0 | 0 | 0 |
| 371 | 0 | 0 | 0.940885 | 0.054663 | 0.004451 | 0 | 0 | 0 | 0 |
| 372 | 0.00002 | 0 | 0.94076 | 0.059221 | 0 | 0 | 0 | 0 | 0 |
| 373 | 0.00001 | 0 | 0.939746 | 0.060244 | 0 | 0 | 0 | 0 | 0 |
| 374 | 0 | 0 | 0.948845 | 0.051155 | 0 | 0 | 0 | 0 | 0 |
| 375 | 0.00001 | 0 | 0.942041 | 0.057949 | 0 | 0 | 0 | 0 | 0 |
| 376 | 0.00002 | 0.00001 | 0.94086 | 0.05911 | 0 | 0 | 0 | 0 | 0 |
| 377 | 0.00002 | 0 | 0.938994 | 0.060986 | 0 | 0 | 0 | 0 | 0 |
| 378 | 0.00001 | 0 | 0.947657 | 0.052333 | 0 | 0 | 0 | 0 | 0 |
| 379 | 0.00002 | 0 | 0.9467 | 0.05328 | 0 | 0 | 0 | 0 | 0 |
| 380 | 0 | 0 | 0.939601 | 0.060399 | 0 | 0 | 0 | 0 | 0 |
| 381 | 0.00002 | 0 | 0.943807 | 0.056173 | 0 | 0 | 0 | 0 | 0 |
| 382 | 0 | 0 | 0.943179 | 0.056821 | 0 | 0 | 0 | 0 | 0 |
| 383 | 0 | 0 | 0.941184 | 0.058816 | 0 | 0 | 0 | 0 | 0 |
| 384 | 0.00001 | 0 | 0.941501 | 0.058489 | 0 | 0 | 0 | 0 | 0 |
| 385 | 0 | 0 | 0.945112 | 0.054888 | 0 | 0 | 0 | 0 | 0 |
| 386 | 0.00002 | 0 | 0.945691 | 0.054289 | 0 | 0 | 0 | 0 | 0 |
| 387 | 0.00003 | 0 | 0.946028 | 0.053942 | 0 | 0 | 0 | 0 | 0 |
| 388 | 0.00002 | 0 | 0.944413 | 0.055567 | 0 | 0 | 0 | 0 | 0 |
| 389 | 0 | 0 | 0.94588 | 0.05412 | 0 | 0 | 0 | 0 | 0 |
| 390 | 0 | 0 | 0.945397 | 0.054603 | 0 | 0 | 0 | 0 | 0 |
| 391 | 0.00001 | 0 | 0.944001 | 0.055989 | 0 | 0 | 0 | 0 | 0 |
| 392 | 0 | 0 | 0.945167 | 0.054833 | 0 | 0 | 0 | 0 | 0 |
| 393 | 0.00003 | 0 | 0.941661 | 0.058309 | 0 | 0 | 0 | 0 | 0 |
| 394 | 0 | 0 | 0.947738 | 0.052262 | 0 | 0 | 0 | 0 | 0 |
| 395 | 0.00002 | 0 | 0.94743 | 0.05255 | 0 | 0 | 0 | 0 | 0 |
| 396 | 0.00002 | 0 | 0.944327 | 0.055653 | 0 | 0 | 0 | 0 | 0 |
| 397 | 0.00002 | 0 | 0.94422 | 0.05576 | 0 | 0 | 0 | 0 | 0 |
| 398 | 0 | 0 | 0.94853 | 0.05147 | 0 | 0 | 0 | 0 | 0 |
| 399 | 0 | 0 | 0.945647 | 0.054353 | 0 | 0 | 0 | 0 | 0 |
| 400 | 0 | 0 | 0.947719 | 0.052281 | 0 | 0 | 0 | 0 | 0 |
| 401 | 0 | 0 | 0.943604 | 0.056396 | 0 | 0 | 0 | 0 | 0 |
| 402 | 0 | 0 | 0.94527 | 0.05473 | 0 | 0 | 0 | 0 | 0 |
| 403 | 0.00001 | 0 | 0.94546 | 0.05453 | 0 | 0 | 0 | 0 | 0 |
| 404 | 0.00003 | 0 | 0.94396 | 0.05601 | 0 | 0 | 0 | 0 | 0 |
| 405 | 0.00001 | 0 | 0.947041 | 0.052949 | 0 | 0 | 0 | 0 | 0 |
| 406 | 0.00001 | 0 | 0.94548 | 0.05451 | 0 | 0 | 0 | 0 | 0 |
| 407 | 0.00002 | 0 | 0.942789 | 0.057191 | 0 | 0 | 0 | 0 | 0 |
| 408 | 0.00001 | 0.00001 | 0.942461 | 0.057518 | 0 | 0 | 0 | 0 | 0 |
| 409 | 0 | 0 | 0.943011 | 0.056989 | 0 | 0 | 0 | 0 | 0 |
| 410 | 0.00001 | 0 | 0.938338 | 0.061652 | 0 | 0 | 0 | 0 | 0 |
| 411 | 0.00002 | 0 | 0.946066 | 0.053914 | 0 | 0 | 0 | 0 | 0 |
| 412 | 0.00003 | 0 | 0.94527 | 0.054699 | 0 | 0 | 0 | 0 | 0 |
| 413 | 0 | 0 | 0.940089 | 0.059911 | 0 | 0 | 0 | 0 | 0 |
| 414 | 0 | 0 | 0.947281 | 0.052719 | 0 | 0 | 0 | 0 | 0 |
| 415 | 0 | 0 | 0.942231 | 0.057769 | 0 | 0 | 0 | 0 | 0 |
| 416 | 0.00002 | 0 | 0.945819 | 0.054161 | 0 | 0 | 0 | 0 | 0 |
| 417 | 0.00003 | 0 | 0.946452 | 0.053518 | 0 | 0 | 0 | 0 | 0 |
| 418 | 0.00001 | 0 | 0.943835 | 0.056155 | 0 | 0 | 0 | 0 | 0 |
| 419 | 0 | 0 | 0.944131 | 0.055869 | 0 | 0 | 0 | 0 | 0 |
| 420 | 0 | 0 | 0.949436 | 0.050564 | 0 | 0 | 0 | 0 | 0 |
| 421 | 0 | 0 | 0.941514 | 0.058486 | 0 | 0 | 0 | 0 | 0 |
| 422 | 0.00001 | 0.00001 | 0.943462 | 0.056518 | 0 | 0 | 0 | 0 | 0 |
| 423 | 0.00001 | 0 | 0.945927 | 0.054063 | 0 | 0 | 0 | 0 | 0 |
| 424 | 0 | 0 | 0.948012 | 0.051988 | 0 | 0 | 0 | 0 | 0 |
| 425 | 0 | 0 | 0.94559 | 0.05441 | 0 | 0 | 0 | 0 | 0 |
| 426 | 0 | 0 | 0.944089 | 0.055911 | 0 | 0 | 0 | 0 | 0 |
| 427 | 0.00001 | 0 | 0.946072 | 0.053918 | 0 | 0 | 0 | 0 | 0 |
| 428 | 0.00001 | 0 | 0.947367 | 0.052623 | 0 | 0 | 0 | 0 | 0 |
| 429 | 0.00002 | 0 | 0.945565 | 0.054415 | 0 | 0 | 0 | 0 | 0 |
| 430 | 0.00002 | 0 | 0.943632 | 0.056348 | 0 | 0 | 0 | 0 | 0 |
| 431 | 0.00001 | 0 | 0.947056 | 0.052934 | 0 | 0 | 0 | 0 | 0 |
| 432 | 0 | 0 | 0.942939 | 0.057061 | 0 | 0 | 0 | 0 | 0 |
| 433 | 0.00002 | 0 | 0.947298 | 0.052682 | 0 | 0 | 0 | 0 | 0 |
| 434 | 0 | 0 | 0.946539 | 0.053461 | 0 | 0 | 0 | 0 | 0 |
| 435 | 0.00001 | 0 | 0.945379 | 0.054611 | 0 | 0 | 0 | 0 | 0 |
| 436 | 0.00001 | 0 | 0.941169 | 0.058821 | 0 | 0 | 0 | 0 | 0 |
| 437 | 0 | 0 | 0.945327 | 0.054673 | 0 | 0 | 0 | 0 | 0 |
| 438 | 0.00001 | 0 | 0.946118 | 0.053872 | 0 | 0 | 0 | 0 | 0 |
| 439 | 0.00001 | 0 | 0.946046 | 0.053944 | 0 | 0 | 0 | 0 | 0 |
| 440 | 0 | 0 | 0.946855 | 0.053145 | 0 | 0 | 0 | 0 | 0 |
| 441 | 0.00003 | 0 | 0.944781 | 0.055189 | 0 | 0 | 0 | 0 | 0 |
| 442 | 0.00001 | 0 | 0.944791 | 0.055199 | 0 | 0 | 0 | 0 | 0 |
| 443 | 0.00004 | 0 | 0.948536 | 0.051424 | 0 | 0 | 0 | 0 | 0 |
| 444 | 0.00001 | 0 | 0.945933 | 0.054057 | 0 | 0 | 0 | 0 | 0 |
| 445 | 0.00001 | 0 | 0.943369 | 0.056621 | 0 | 0 | 0 | 0 | 0 |
| 446 | 0.00001 | 0 | 0.942494 | 0.057495 | 0 | 0 | 0 | 0 | 0 |
| 447 | 0 | 0 | 0.945939 | 0.054061 | 0 | 0 | 0 | 0 | 0 |
| 448 | 0.00001 | 0 | 0.944605 | 0.055385 | 0 | 0 | 0 | 0 | 0 |
| 449 | 0.00001 | 0 | 0.94566 | 0.05433 | 0 | 0 | 0 | 0 | 0 |
| 450 | 0 | 0 | 0.943994 | 0.056006 | 0 | 0 | 0 | 0 | 0 |
| 451 | 0 | 0 | 0.947641 | 0.052359 | 0 | 0 | 0 | 0 | 0 |
| 452 | 0.00001 | 0 | 0.944832 | 0.055158 | 0 | 0 | 0 | 0 | 0 |
| 453 | 0.00003 | 0 | 0.944091 | 0.055879 | 0 | 0 | 0 | 0 | 0 |
| 454 | 0 | 0 | 0.943597 | 0.056403 | 0 | 0 | 0 | 0 | 0 |
| 455 | 0 | 0 | 0.946347 | 0.053653 | 0 | 0 | 0 | 0 | 0 |
| 456 | 0.00002 | 0 | 0.944946 | 0.055034 | 0 | 0 | 0 | 0 | 0 |
| 457 | 0 | 0 | 0.94163 | 0.05837 | 0 | 0 | 0 | 0 | 0 |
| 458 | 0.00003 | 0 | 0.946792 | 0.053178 | 0 | 0 | 0 | 0 | 0 |
| 459 | 0.00001 | 0 | 0.93994 | 0.06005 | 0 | 0 | 0 | 0 | 0 |
| 460 | 0.00001 | 0 | 0.940014 | 0.059976 | 0 | 0 | 0 | 0 | 0 |
| 461 | 0.00001 | 0 | 0.949132 | 0.050858 | 0 | 0 | 0 | 0 | 0 |
| 462 | 0.00001 | 0 | 0.948139 | 0.051851 | 0 | 0 | 0 | 0 | 0 |
| 463 | 0.00001 | 0 | 0.941038 | 0.058952 | 0 | 0 | 0 | 0 | 0 |
| 464 | 0.00001 | 0 | 0.931986 | 0.057108 | 0.010896 | 0 | 0 | 0 | 0 |
| 465 | 0.00001 | 0 | 0.945949 | 0.054041 | 0 | 0 | 0 | 0 | 0 |
| 466 | 0.00001 | 0 | 0.943213 | 0.056777 | 0 | 0 | 0 | 0 | 0 |
| 467 | 0 | 0 | 0.938842 | 0.061158 | 0 | 0 | 0 | 0 | 0 |
| 468 | 0.00001 | 0 | 0.946812 | 0.053178 | 0 | 0 | 0 | 0 | 0 |
| 469 | 0.00002 | 0 | 0.943398 | 0.056582 | 0 | 0 | 0 | 0 | 0 |
| 470 | 0.00004 | 0 | 0.946195 | 0.053765 | 0 | 0 | 0 | 0 | 0 |
| 471 | 0.00001 | 0 | 0.942455 | 0.057535 | 0 | 0 | 0 | 0 | 0 |
| 472 | 0.00001 | 0 | 0.942658 | 0.057332 | 0 | 0 | 0 | 0 | 0 |
| 473 | 0.00001 | 0 | 0.948098 | 0.051892 | 0 | 0 | 0 | 0 | 0 |
| 474 | 0.00001 | 0 | 0.946647 | 0.053343 | 0 | 0 | 0 | 0 | 0 |
| 475 | 0.00002 | 0 | 0.948504 | 0.051476 | 0 | 0 | 0 | 0 | 0 |
| 476 | 0.00001 | 0.00001 | 0.937138 | 0.062842 | 0 | 0 | 0 | 0 | 0 |
| 477 | 0 | 0 | 0.946608 | 0.053392 | 0 | 0 | 0 | 0 | 0 |
| 478 | 0 | 0 | 0.942707 | 0.057293 | 0 | 0 | 0 | 0 | 0 |
| 479 | 0 | 0 | 0.943938 | 0.056062 | 0 | 0 | 0 | 0 | 0 |
| 480 | 0.00001 | 0 | 0.941278 | 0.058712 | 0 | 0 | 0 | 0 | 0 |
| 481 | 0.00001 | 0 | 0.946406 | 0.053584 | 0 | 0 | 0 | 0 | 0 |
| 482 | 0.00001 | 0 | 0.942071 | 0.057919 | 0 | 0 | 0 | 0 | 0 |
| 483 | 0.00001 | 0 | 0.946259 | 0.053731 | 0 | 0 | 0 | 0 | 0 |
| 484 | 0 | 0 | 0.942108 | 0.057892 | 0 | 0 | 0 | 0 | 0 |
| 485 | 0.00002 | 0 | 0.949738 | 0.050243 | 0 | 0 | 0 | 0 | 0 |
| 486 | 0.00001 | 0 | 0.941658 | 0.058332 | 0 | 0 | 0 | 0 | 0 |
| 487 | 0.00001 | 0 | 0.941921 | 0.058069 | 0 | 0 | 0 | 0 | 0 |
| 488 | 0 | 0 | 0.943076 | 0.056924 | 0 | 0 | 0 | 0 | 0 |
| 489 | 0 | 0 | 0.945191 | 0.054809 | 0 | 0 | 0 | 0 | 0 |
| 490 | 0.00001 | 0 | 0.946842 | 0.053148 | 0 | 0 | 0 | 0 | 0 |
| 491 | 0 | 0 | 0.94499 | 0.05501 | 0 | 0 | 0 | 0 | 0 |
| 492 | 0.00001 | 0 | 0.944437 | 0.055553 | 0 | 0 | 0 | 0 | 0 |
| 493 | 0 | 0 | 0.942363 | 0.057637 | 0 | 0 | 0 | 0 | 0 |
| 494 | 0.00001 | 0 | 0.944999 | 0.054991 | 0 | 0 | 0 | 0 | 0 |
| 495 | 0 | 0 | 0.943669 | 0.056331 | 0 | 0 | 0 | 0 | 0 |
| 496 | 0 | 0 | 0.941593 | 0.056895 | 0.001512 | 0 | 0 | 0 | 0 |
| 497 | 0.00002 | 0 | 0.947125 | 0.052855 | 0 | 0 | 0 | 0 | 0 |
| 498 | 0.00001 | 0 | 0.949614 | 0.050376 | 0 | 0 | 0 | 0 | 0 |
| 499 | 0.00001 | 0 | 0.942844 | 0.057146 | 0 | 0 | 0 | 0 | 0 |
| 500 | 0.00001 | 0 | 0.944389 | 0.055601 | 0 | 0 | 0 | 0 | 0 |
| 501 | 0.00002 | 0 | 0.943444 | 0.056536 | 0 | 0 | 0 | 0 | 0 |
| 502 | 0.00001 | 0 | 0.94482 | 0.05517 | 0 | 0 | 0 | 0 | 0 |
| 503 | 0.00001 | 0 | 0.939955 | 0.060035 | 0 | 0 | 0 | 0 | 0 |
| 504 | 0.00001 | 0 | 0.947185 | 0.052805 | 0 | 0 | 0 | 0 | 0 |
| 505 | 0.00001 | 0 | 0.941174 | 0.058227 | 0.000589 | 0 | 0 | 0 | 0 |
| 506 | 0.00002 | 0 | 0.942733 | 0.057247 | 0 | 0 | 0 | 0 | 0 |
| 507 | 0.00001 | 0 | 0.944721 | 0.055269 | 0 | 0 | 0 | 0 | 0 |
| 508 | 0.00003 | 0 | 0.943378 | 0.056592 | 0 | 0 | 0 | 0 | 0 |
| 509 | 0.00001 | 0 | 0.94039 | 0.0596 | 0 | 0 | 0 | 0 | 0 |
| 510 | 0 | 0 | 0.945109 | 0.054891 | 0 | 0 | 0 | 0 | 0 |
| 511 | 0 | 0 | 0.945177 | 0.054823 | 0 | 0 | 0 | 0 | 0 |
| 512 | 0 | 0 | 0.947092 | 0.052908 | 0 | 0 | 0 | 0 | 0 |
| 513 | 0 | 0 | 0.942481 | 0.057519 | 0 | 0 | 0 | 0 | 0 |
| 514 | 0.00001 | 0 | 0.943527 | 0.056463 | 0 | 0 | 0 | 0 | 0 |
| 515 | 0.00001 | 0 | 0.944522 | 0.055468 | 0 | 0 | 0 | 0 | 0 |
| 516 | 0.00001 | 0 | 0.941121 | 0.058869 | 0 | 0 | 0 | 0 | 0 |
| 517 | 0 | 0 | 0.944374 | 0.055626 | 0 | 0 | 0 | 0 | 0 |
| 518 | 0 | 0 | 0.943384 | 0.056616 | 0 | 0 | 0 | 0 | 0 |
| 519 | 0.00001 | 0 | 0.9419 | 0.05809 | 0 | 0 | 0 | 0 | 0 |
| 520 | 0.00001 | 0 | 0.944841 | 0.055149 | 0 | 0 | 0 | 0 | 0 |
| 521 | 0.00002 | 0 | 0.94543 | 0.05455 | 0 | 0 | 0 | 0 | 0 |
| 522 | 0.00001 | 0 | 0.948155 | 0.051835 | 0 | 0 | 0 | 0 | 0 |
| 523 | 0.00001 | 0 | 0.944367 | 0.055623 | 0 | 0 | 0 | 0 | 0 |
| 524 | 0.00001 | 0 | 0.943536 | 0.056454 | 0 | 0 | 0 | 0 | 0 |
| 525 | 0.00003 | 0 | 0.942506 | 0.057464 | 0 | 0 | 0 | 0 | 0 |
| 526 | 0 | 0 | 0.942769 | 0.057231 | 0 | 0 | 0 | 0 | 0 |
| 527 | 0.00002 | 0 | 0.943047 | 0.056933 | 0 | 0 | 0 | 0 | 0 |
| 528 | 0 | 0 | 0.942432 | 0.057568 | 0 | 0 | 0 | 0 | 0 |
| 529 | 0.00001 | 0 | 0.944167 | 0.055823 | 0 | 0 | 0 | 0 | 0 |
| 530 | 0.00002 | 0 | 0.942258 | 0.057722 | 0 | 0 | 0 | 0 | 0 |
| 531 | 0 | 0 | 0.943247 | 0.056753 | 0 | 0 | 0 | 0 | 0 |
| 532 | 0.00003 | 0 | 0.949494 | 0.050477 | 0 | 0 | 0 | 0 | 0 |
| 533 | 0 | 0 | 0.943921 | 0.056079 | 0 | 0 | 0 | 0 | 0 |
| 534 | 0.00003 | 0 | 0.943512 | 0.056458 | 0 | 0 | 0 | 0 | 0 |
| 535 | 0 | 0 | 0.946857 | 0.053143 | 0 | 0 | 0 | 0 | 0 |
| 536 | 0.000059 | 0 | 0.94868 | 0.05126 | 0 | 0 | 0 | 0 | 0 |
| 537 | 0.00001 | 0 | 0.94357 | 0.05642 | 0 | 0 | 0 | 0 | 0 |
| 538 | 0.00002 | 0 | 0.942181 | 0.057799 | 0 | 0 | 0 | 0 | 0 |
| 539 | 0 | 0 | 0.94029 | 0.05971 | 0 | 0 | 0 | 0 | 0 |
| 540 | 0 | 0 | 0.940816 | 0.059184 | 0 | 0 | 0 | 0 | 0 |
| 541 | 0.00003 | 0 | 0.945841 | 0.054129 | 0 | 0 | 0 | 0 | 0 |
| 542 | 0.00004 | 0 | 0.94468 | 0.05528 | 0 | 0 | 0 | 0 | 0 |
| 543 | 0.00001 | 0 | 0.946793 | 0.053197 | 0 | 0 | 0 | 0 | 0 |
| 544 | 0.00001 | 0 | 0.948707 | 0.051283 | 0 | 0 | 0 | 0 | 0 |
| 545 | 0.00001 | 0 | 0.943338 | 0.056652 | 0 | 0 | 0 | 0 | 0 |
| 546 | 0 | 0 | 0.948737 | 0.051263 | 0 | 0 | 0 | 0 | 0 |
| 547 | 0.00001 | 0 | 0.943813 | 0.056177 | 0 | 0 | 0 | 0 | 0 |
| 548 | 0.00001 | 0 | 0.945892 | 0.054098 | 0 | 0 | 0 | 0 | 0 |
| 549 | 0 | 0 | 0.940766 | 0.059234 | 0 | 0 | 0 | 0 | 0 |
| 550 | 0 | 0 | 0.943465 | 0.056535 | 0 | 0 | 0 | 0 | 0 |
| 551 | 0.00001 | 0 | 0.947286 | 0.052704 | 0 | 0 | 0 | 0 | 0 |
| 552 | 0.00001 | 0 | 0.945005 | 0.054985 | 0 | 0 | 0 | 0 | 0 |
| 553 | 0.00002 | 0 | 0.942498 | 0.057482 | 0 | 0 | 0 | 0 | 0 |
| 554 | 0 | 0 | 0.948236 | 0.051764 | 0 | 0 | 0 | 0 | 0 |
| 555 | 0.00001 | 0 | 0.942769 | 0.057221 | 0 | 0 | 0 | 0 | 0 |
| 556 | 0.00001 | 0 | 0.94595 | 0.05404 | 0 | 0 | 0 | 0 | 0 |
| 557 | 0.00001 | 0 | 0.943289 | 0.056701 | 0 | 0 | 0 | 0 | 0 |
| 558 | 0.00001 | 0 | 0.944072 | 0.055918 | 0 | 0 | 0 | 0 | 0 |
| 559 | 0.00002 | 0 | 0.947907 | 0.052074 | 0 | 0 | 0 | 0 | 0 |
| 560 | 0 | 0 | 0.948438 | 0.051562 | 0 | 0 | 0 | 0 | 0 |
| 561 | 0.00002 | 0 | 0.942492 | 0.057488 | 0 | 0 | 0 | 0 | 0 |
| 562 | 0.00002 | 0 | 0.941484 | 0.058496 | 0 | 0 | 0 | 0 | 0 |
| 563 | 0.00003 | 0 | 0.942088 | 0.057882 | 0 | 0 | 0 | 0 | 0 |
| 564 | 0.00001 | 0 | 0.944002 | 0.055988 | 0 | 0 | 0 | 0 | 0 |
| 565 | 0.00001 | 0 | 0.942683 | 0.057307 | 0 | 0 | 0 | 0 | 0 |
| 566 | 0.00002 | 0 | 0.946843 | 0.053137 | 0 | 0 | 0 | 0 | 0 |
| 567 | 0.00001 | 0 | 0.945122 | 0.054868 | 0 | 0 | 0 | 0 | 0 |
| 568 | 0 | 0 | 0.943047 | 0.056953 | 0 | 0 | 0 | 0 | 0 |
| 569 | 0.00001 | 0 | 0.94697 | 0.05302 | 0 | 0 | 0 | 0 | 0 |
| 570 | 0.00002 | 0 | 0.941099 | 0.058881 | 0 | 0 | 0 | 0 | 0 |
| 571 | 0 | 0 | 0.941341 | 0.058659 | 0 | 0 | 0 | 0 | 0 |
| 572 | 0.00001 | 0 | 0.949141 | 0.050849 | 0 | 0 | 0 | 0 | 0 |
| 573 | 0.00001 | 0 | 0.948005 | 0.051985 | 0 | 0 | 0 | 0 | 0 |
| 574 | 0.00002 | 0 | 0.945169 | 0.054811 | 0 | 0 | 0 | 0 | 0 |
| 575 | 0.00001 | 0 | 0.9441 | 0.05589 | 0 | 0 | 0 | 0 | 0 |
| 576 | 0.00001 | 0 | 0.948619 | 0.051371 | 0 | 0 | 0 | 0 | 0 |
| 577 | 0.00003 | 0 | 0.943844 | 0.056126 | 0 | 0 | 0 | 0 | 0 |
| 578 | 0.00002 | 0 | 0.945756 | 0.054224 | 0 | 0 | 0 | 0 | 0 |
| 579 | 0.00001 | 0 | 0.942101 | 0.057889 | 0 | 0 | 0 | 0 | 0 |
| 580 | 0 | 0 | 0.943264 | 0.056736 | 0 | 0 | 0 | 0 | 0 |
| 581 | 0.00002 | 0 | 0.941793 | 0.058187 | 0 | 0 | 0 | 0 | 0 |
| 582 | 0.00001 | 0 | 0.943116 | 0.056874 | 0 | 0 | 0 | 0 | 0 |
| 583 | 0.00003 | 0.00001 | 0.940401 | 0.059559 | 0 | 0 | 0 | 0 | 0 |
| 584 | 0.00002 | 0 | 0.941076 | 0.058904 | 0 | 0 | 0 | 0 | 0 |
| 585 | 0 | 0 | 0.943534 | 0.056466 | 0 | 0 | 0 | 0 | 0 |
| 586 | 0 | 0 | 0.942782 | 0.057218 | 0 | 0 | 0 | 0 | 0 |
| 587 | 0.00005 | 0 | 0.944722 | 0.055228 | 0 | 0 | 0 | 0 | 0 |
| 588 | 0 | 0 | 0.948175 | 0.051825 | 0 | 0 | 0 | 0 | 0 |
| 589 | 0.00003 | 0 | 0.945883 | 0.054087 | 0 | 0 | 0 | 0 | 0 |
| 590 | 0.00001 | 0 | 0.940284 | 0.059706 | 0 | 0 | 0 | 0 | 0 |
| 591 | 0.00001 | 0 | 0.940719 | 0.059271 | 0 | 0 | 0 | 0 | 0 |
| 592 | 0.00002 | 0 | 0.942715 | 0.057265 | 0 | 0 | 0 | 0 | 0 |
| 593 | 0.00001 | 0 | 0.945901 | 0.054089 | 0 | 0 | 0 | 0 | 0 |
| 594 | 0 | 0 | 0.941325 | 0.058675 | 0 | 0 | 0 | 0 | 0 |
| 595 | 0.00002 | 0 | 0.945153 | 0.054827 | 0 | 0 | 0 | 0 | 0 |
| 596 | 0.00003 | 0 | 0.944088 | 0.055882 | 0 | 0 | 0 | 0 | 0 |
| 597 | 0.00001 | 0 | 0.945138 | 0.054852 | 0 | 0 | 0 | 0 | 0 |
| 598 | 0 | 0 | 0.947013 | 0.052987 | 0 | 0 | 0 | 0 | 0 |
| 599 | 0 | 0 | 0.944811 | 0.055189 | 0 | 0 | 0 | 0 | 0 |
| 600 | 0 | 0 | 0.941496 | 0.058504 | 0 | 0 | 0 | 0 | 0 |
| 601 | 0 | 0 | 0.943855 | 0.056145 | 0 | 0 | 0 | 0 | 0 |
| 602 | 0.00002 | 0 | 0.947197 | 0.052783 | 0 | 0 | 0 | 0 | 0 |
| 603 | 0.00001 | 0 | 0.944732 | 0.055258 | 0 | 0 | 0 | 0 | 0 |
| 604 | 0.00001 | 0 | 0.944667 | 0.055323 | 0 | 0 | 0 | 0 | 0 |
| 605 | 0 | 0 | 0.94576 | 0.05424 | 0 | 0 | 0 | 0 | 0 |
| 606 | 0 | 0 | 0.945833 | 0.054167 | 0 | 0 | 0 | 0 | 0 |
| 607 | 0.00003 | 0 | 0.94869 | 0.051279 | 0 | 0 | 0 | 0 | 0 |
| 608 | 0.00002 | 0 | 0.946788 | 0.053191 | 0 | 0 | 0 | 0 | 0 |
| 609 | 0.00004 | 0 | 0.946686 | 0.053274 | 0 | 0 | 0 | 0 | 0 |
| 610 | 0 | 0 | 0.943941 | 0.056059 | 0 | 0 | 0 | 0 | 0 |
| 611 | 0.00001 | 0 | 0.943953 | 0.056037 | 0 | 0 | 0 | 0 | 0 |
| 612 | 0.00001 | 0 | 0.944762 | 0.055228 | 0 | 0 | 0 | 0 | 0 |
| 613 | 0.00001 | 0 | 0.940772 | 0.059218 | 0 | 0 | 0 | 0 | 0 |
| 614 | 0.00001 | 0 | 0.945142 | 0.054848 | 0 | 0 | 0 | 0 | 0 |
| 615 | 0.00002 | 0 | 0.949841 | 0.050139 | 0 | 0 | 0 | 0 | 0 |
| 616 | 0.00002 | 0 | 0.944694 | 0.055286 | 0 | 0 | 0 | 0 | 0 |
| 617 | 0 | 0 | 0.944832 | 0.055168 | 0 | 0 | 0 | 0 | 0 |
| 618 | 0.00001 | 0 | 0.940624 | 0.059366 | 0 | 0 | 0 | 0 | 0 |
| 619 | 0.00002 | 0 | 0.948719 | 0.051261 | 0 | 0 | 0 | 0 | 0 |
| 620 | 0 | 0 | 0.94397 | 0.05603 | 0 | 0 | 0 | 0 | 0 |
| 621 | 0.00002 | 0 | 0.945306 | 0.054674 | 0 | 0 | 0 | 0 | 0 |
| 622 | 0.00001 | 0 | 0.939473 | 0.060517 | 0 | 0 | 0 | 0 | 0 |
| 623 | 0.00004 | 0 | 0.944054 | 0.055905 | 0 | 0 | 0 | 0 | 0 |
| 624 | 0.00001 | 0 | 0.944317 | 0.055673 | 0 | 0 | 0 | 0 | 0 |
| 625 | 0 | 0 | 0.94465 | 0.053956 | 0.001395 | 0 | 0 | 0 | 0 |
| 626 | 0.00001 | 0 | 0.939997 | 0.059993 | 0 | 0 | 0 | 0 | 0 |
| 627 | 0.00001 | 0 | 0.943779 | 0.056211 | 0 | 0 | 0 | 0 | 0 |
| 628 | 0.00001 | 0 | 0.945076 | 0.054914 | 0 | 0 | 0 | 0 | 0 |
| 629 | 0.00001 | 0 | 0.943436 | 0.056554 | 0 | 0 | 0 | 0 | 0 |
| 630 | 0.00002 | 0 | 0.942412 | 0.057568 | 0 | 0 | 0 | 0 | 0 |
| 631 | 0.00003 | 0 | 0.942086 | 0.057883 | 0 | 0 | 0 | 0 | 0 |
| 632 | 0.00001 | 0 | 0.944983 | 0.055007 | 0 | 0 | 0 | 0 | 0 |
| 633 | 0 | 0 | 0.944821 | 0.055179 | 0 | 0 | 0 | 0 | 0 |
| 634 | 0.00002 | 0 | 0.944497 | 0.055483 | 0 | 0 | 0 | 0 | 0 |
| 635 | 0.00001 | 0 | 0.949547 | 0.050443 | 0 | 0 | 0 | 0 | 0 |
| 636 | 0.00001 | 0 | 0.943553 | 0.056437 | 0 | 0 | 0 | 0 | 0 |
| 637 | 0.00001 | 0 | 0.941521 | 0.058469 | 0 | 0 | 0 | 0 | 0 |
| 638 | 0.00001 | 0 | 0.9427 | 0.05729 | 0 | 0 | 0 | 0 | 0 |
| 639 | 0.00002 | 0 | 0.93984 | 0.06014 | 0 | 0 | 0 | 0 | 0 |
| 640 | 0 | 0 | 0.943127 | 0.056873 | 0 | 0 | 0 | 0 | 0 |
| 641 | 0 | 0 | 0.944854 | 0.055146 | 0 | 0 | 0 | 0 | 0 |
| 642 | 0.00001 | 0 | 0.945882 | 0.054108 | 0 | 0 | 0 | 0 | 0 |
| 643 | 0 | 0 | 0.943426 | 0.056574 | 0 | 0 | 0 | 0 | 0 |
| 644 | 0.00004 | 0 | 0.943095 | 0.056865 | 0 | 0 | 0 | 0 | 0 |
| 645 | 0 | 0 | 0.947246 | 0.052754 | 0 | 0 | 0 | 0 | 0 |
| 646 | 0.00001 | 0 | 0.940688 | 0.059302 | 0 | 0 | 0 | 0 | 0 |
| 647 | 0 | 0 | 0.941555 | 0.058445 | 0 | 0 | 0 | 0 | 0 |
| 648 | 0 | 0 | 0.948066 | 0.051934 | 0 | 0 | 0 | 0 | 0 |
| 649 | 0.00001 | 0 | 0.944392 | 0.055598 | 0 | 0 | 0 | 0 | 0 |
| 650 | 0.00001 | 0 | 0.941111 | 0.058879 | 0 | 0 | 0 | 0 | 0 |
| 651 | 0 | 0 | 0.944981 | 0.055019 | 0 | 0 | 0 | 0 | 0 |
| 652 | 0.00001 | 0 | 0.943089 | 0.056901 | 0 | 0 | 0 | 0 | 0 |
| 653 | 0.00001 | 0 | 0.94782 | 0.05217 | 0 | 0 | 0 | 0 | 0 |
| 654 | 0 | 0 | 0.944759 | 0.055241 | 0 | 0 | 0 | 0 | 0 |
| 655 | 0 | 0 | 0.941287 | 0.058713 | 0 | 0 | 0 | 0 | 0 |
| 656 | 0.00001 | 0 | 0.950534 | 0.049456 | 0 | 0 | 0 | 0 | 0 |
| 657 | 0 | 0 | 0.943698 | 0.056302 | 0 | 0 | 0 | 0 | 0 |
| 658 | 0.00001 | 0 | 0.945048 | 0.054942 | 0 | 0 | 0 | 0 | 0 |
| 659 | 0.00001 | 0 | 0.941585 | 0.058405 | 0 | 0 | 0 | 0 | 0 |
| 660 | 0.00002 | 0 | 0.947235 | 0.052745 | 0 | 0 | 0 | 0 | 0 |
| 661 | 0.00001 | 0 | 0.944486 | 0.055504 | 0 | 0 | 0 | 0 | 0 |
| 662 | 0.00001 | 0 | 0.944466 | 0.055524 | 0 | 0 | 0 | 0 | 0 |
| 663 | 0.00001 | 0 | 0.945424 | 0.054566 | 0 | 0 | 0 | 0 | 0 |
| 664 | 0.00002 | 0 | 0.948623 | 0.051357 | 0 | 0 | 0 | 0 | 0 |
| 665 | 0 | 0 | 0.943229 | 0.056771 | 0 | 0 | 0 | 0 | 0 |
| 666 | 0.00002 | 0 | 0.945174 | 0.054806 | 0 | 0 | 0 | 0 | 0 |
| 667 | 0.00003 | 0 | 0.941844 | 0.058126 | 0 | 0 | 0 | 0 | 0 |
| 668 | 0.00001 | 0 | 0.944186 | 0.055804 | 0 | 0 | 0 | 0 | 0 |
| 669 | 0.00001 | 0 | 0.939417 | 0.060573 | 0 | 0 | 0 | 0 | 0 |
| 670 | 0.00005 | 0 | 0.944735 | 0.055214 | 0 | 0 | 0 | 0 | 0 |
| 671 | 0.00001 | 0 | 0.945259 | 0.054731 | 0 | 0 | 0 | 0 | 0 |
| 672 | 0.00002 | 0 | 0.945663 | 0.054317 | 0 | 0 | 0 | 0 | 0 |
| 673 | 0 | 0 | 0.941353 | 0.058647 | 0 | 0 | 0 | 0 | 0 |
| 674 | 0.00002 | 0 | 0.943454 | 0.056526 | 0 | 0 | 0 | 0 | 0 |
| 675 | 0.00001 | 0 | 0.947701 | 0.052289 | 0 | 0 | 0 | 0 | 0 |
| 676 | 0.00002 | 0 | 0.946297 | 0.053683 | 0 | 0 | 0 | 0 | 0 |
| 677 | 0.00002 | 0 | 0.945631 | 0.05435 | 0 | 0 | 0 | 0 | 0 |
| 678 | 0.00001 | 0 | 0.94656 | 0.05343 | 0 | 0 | 0 | 0 | 0 |
| 679 | 0 | 0 | 0.947317 | 0.052683 | 0 | 0 | 0 | 0 | 0 |
| 680 | 0 | 0 | 0.942909 | 0.057091 | 0 | 0 | 0 | 0 | 0 |
| 681 | 0 | 0 | 0.94297 | 0.05703 | 0 | 0 | 0 | 0 | 0 |
| 682 | 0.00003 | 0 | 0.944813 | 0.055157 | 0 | 0 | 0 | 0 | 0 |
| 683 | 0.00001 | 0 | 0.94641 | 0.05358 | 0 | 0 | 0 | 0 | 0 |
| 684 | 0.00003 | 0 | 0.947031 | 0.052939 | 0 | 0 | 0 | 0 | 0 |
| 685 | 0.00001 | 0 | 0.946737 | 0.053253 | 0 | 0 | 0 | 0 | 0 |
| 686 | 0.00003 | 0 | 0.941174 | 0.058796 | 0 | 0 | 0 | 0 | 0 |
| 687 | 0.00002 | 0 | 0.946871 | 0.053109 | 0 | 0 | 0 | 0 | 0 |
| 688 | 0.00002 | 0 | 0.947385 | 0.052596 | 0 | 0 | 0 | 0 | 0 |
| 689 | 0 | 0 | 0.938354 | 0.061646 | 0 | 0 | 0 | 0 | 0 |
| 690 | 0.00004 | 0 | 0.945674 | 0.054286 | 0 | 0 | 0 | 0 | 0 |
| 691 | 0.00001 | 0 | 0.94398 | 0.05601 | 0 | 0 | 0 | 0 | 0 |
| 692 | 0.00003 | 0 | 0.941355 | 0.058615 | 0 | 0 | 0 | 0 | 0 |
| 693 | 0 | 0 | 0.940313 | 0.059687 | 0 | 0 | 0 | 0 | 0 |
| 694 | 0 | 0 | 0.946525 | 0.053475 | 0 | 0 | 0 | 0 | 0 |
| 695 | 0.00002 | 0 | 0.945733 | 0.054247 | 0 | 0 | 0 | 0 | 0 |
| 696 | 0.00002 | 0 | 0.947531 | 0.052449 | 0 | 0 | 0 | 0 | 0 |
| 697 | 0 | 0 | 0.945599 | 0.054401 | 0 | 0 | 0 | 0 | 0 |
| 698 | 0.00002 | 0 | 0.944204 | 0.055776 | 0 | 0 | 0 | 0 | 0 |
| 699 | 0.00001 | 0 | 0.947453 | 0.052537 | 0 | 0 | 0 | 0 | 0 |
| 700 | 0.00001 | 0 | 0.944181 | 0.055809 | 0 | 0 | 0 | 0 | 0 |
| 701 | 0 | 0 | 0.94856 | 0.05144 | 0 | 0 | 0 | 0 | 0 |
| 702 | 0.00001 | 0 | 0.946015 | 0.053975 | 0 | 0 | 0 | 0 | 0 |
| 703 | 0.00002 | 0 | 0.943617 | 0.056363 | 0 | 0 | 0 | 0 | 0 |
| 704 | 0 | 0 | 0.944781 | 0.055219 | 0 | 0 | 0 | 0 | 0 |
| 705 | 0 | 0 | 0.941304 | 0.058696 | 0 | 0 | 0 | 0 | 0 |
| 706 | 0.00001 | 0 | 0.943141 | 0.056849 | 0 | 0 | 0 | 0 | 0 |
| 707 | 0.00001 | 0 | 0.945245 | 0.054745 | 0 | 0 | 0 | 0 | 0 |
| 708 | 0.00001 | 0 | 0.940646 | 0.059344 | 0 | 0 | 0 | 0 | 0 |
| 709 | 0.00001 | 0 | 0.94897 | 0.051021 | 0 | 0 | 0 | 0 | 0 |
| 710 | 0 | 0 | 0.943549 | 0.056451 | 0 | 0 | 0 | 0 | 0 |
| 711 | 0.00001 | 0 | 0.945101 | 0.054889 | 0 | 0 | 0 | 0 | 0 |
| 712 | 0.00002 | 0 | 0.947494 | 0.052486 | 0 | 0 | 0 | 0 | 0 |
| 713 | 0 | 0 | 0.939972 | 0.060028 | 0 | 0 | 0 | 0 | 0 |
| 714 | 0.00003 | 0 | 0.942473 | 0.057497 | 0 | 0 | 0 | 0 | 0 |
| 715 | 0.00001 | 0 | 0.943057 | 0.056934 | 0 | 0 | 0 | 0 | 0 |
| 716 | 0.00003 | 0 | 0.944039 | 0.055931 | 0 | 0 | 0 | 0 | 0 |
| 717 | 0.00002 | 0 | 0.945216 | 0.054764 | 0 | 0 | 0 | 0 | 0 |
| 718 | 0 | 0 | 0.942807 | 0.057193 | 0 | 0 | 0 | 0 | 0 |
| 719 | 0 | 0 | 0.949872 | 0.050128 | 0 | 0 | 0 | 0 | 0 |
| 720 | 0 | 0 | 0.943396 | 0.056604 | 0 | 0 | 0 | 0 | 0 |
| 721 | 0.00001 | 0 | 0.942594 | 0.057396 | 0 | 0 | 0 | 0 | 0 |
| 722 | 0.00002 | 0 | 0.945431 | 0.054549 | 0 | 0 | 0 | 0 | 0 |
| 723 | 0.00003 | 0 | 0.945796 | 0.054174 | 0 | 0 | 0 | 0 | 0 |
| 724 | 0 | 0 | 0.946029 | 0.053971 | 0 | 0 | 0 | 0 | 0 |
| 725 | 0.00002 | 0 | 0.943604 | 0.056376 | 0 | 0 | 0 | 0 | 0 |
| 726 | 0 | 0 | 0.944379 | 0.055621 | 0 | 0 | 0 | 0 | 0 |
| 727 | 0.00002 | 0 | 0.942892 | 0.057088 | 0 | 0 | 0 | 0 | 0 |
| 728 | 0 | 0 | 0.941469 | 0.058531 | 0 | 0 | 0 | 0 | 0 |
| 729 | 0.00003 | 0 | 0.943292 | 0.056678 | 0 | 0 | 0 | 0 | 0 |
| 730 | 0 | 0 | 0.944832 | 0.055168 | 0 | 0 | 0 | 0 | 0 |
| 731 | 0.00002 | 0 | 0.947666 | 0.052314 | 0 | 0 | 0 | 0 | 0 |
| 732 | 0 | 0 | 0.945118 | 0.054882 | 0 | 0 | 0 | 0 | 0 |
| 733 | 0.00001 | 0 | 0.941931 | 0.058059 | 0 | 0 | 0 | 0 | 0 |
| 734 | 0.00001 | 0 | 0.948646 | 0.051344 | 0 | 0 | 0 | 0 | 0 |
| 735 | 0.00002 | 0 | 0.946162 | 0.053818 | 0 | 0 | 0 | 0 | 0 |
| 736 | 0.00002 | 0 | 0.948861 | 0.051119 | 0 | 0 | 0 | 0 | 0 |
| 737 | 0.00002 | 0 | 0.945274 | 0.054706 | 0 | 0 | 0 | 0 | 0 |
| 738 | 0.00001 | 0 | 0.945734 | 0.054256 | 0 | 0 | 0 | 0 | 0 |
| 739 | 0.00003 | 0 | 0.938054 | 0.061916 | 0 | 0 | 0 | 0 | 0 |
| 740 | 0.00002 | 0 | 0.942942 | 0.057038 | 0 | 0 | 0 | 0 | 0 |
| 741 | 0.00001 | 0 | 0.947069 | 0.052921 | 0 | 0 | 0 | 0 | 0 |
| 742 | 0.00002 | 0 | 0.945974 | 0.054006 | 0 | 0 | 0 | 0 | 0 |
| 743 | 0 | 0 | 0.945482 | 0.054518 | 0 | 0 | 0 | 0 | 0 |
| 744 | 0 | 0 | 0.941068 | 0.055099 | 0.003833 | 0 | 0 | 0 | 0 |
| 745 | 0 | 0 | 0.947401 | 0.052599 | 0 | 0 | 0 | 0 | 0 |
| 746 | 0.00001 | 0 | 0.942338 | 0.057652 | 0 | 0 | 0 | 0 | 0 |
| 747 | 0 | 0 | 0.942122 | 0.057878 | 0 | 0 | 0 | 0 | 0 |
| 748 | 0.00003 | 0 | 0.944495 | 0.055475 | 0 | 0 | 0 | 0 | 0 |
| 749 | 0.00001 | 0 | 0.946792 | 0.053198 | 0 | 0 | 0 | 0 | 0 |
| 750 | 0 | 0 | 0.945053 | 0.054947 | 0 | 0 | 0 | 0 | 0 |
| 751 | 0.00002 | 0 | 0.948886 | 0.051094 | 0 | 0 | 0 | 0 | 0 |
| 752 | 0.00001 | 0 | 0.944476 | 0.055514 | 0 | 0 | 0 | 0 | 0 |
| 753 | 0.00001 | 0 | 0.944928 | 0.055062 | 0 | 0 | 0 | 0 | 0 |
| 754 | 0.00001 | 0 | 0.943558 | 0.056432 | 0 | 0 | 0 | 0 | 0 |
| 755 | 0.00002 | 0 | 0.945438 | 0.054542 | 0 | 0 | 0 | 0 | 0 |
| 756 | 0.00001 | 0 | 0.941723 | 0.058267 | 0 | 0 | 0 | 0 | 0 |
| 757 | 0.00002 | 0 | 0.947258 | 0.052722 | 0 | 0 | 0 | 0 | 0 |
| 758 | 0.00001 | 0 | 0.944032 | 0.055958 | 0 | 0 | 0 | 0 | 0 |
| 759 | 0.00002 | 0 | 0.946675 | 0.053305 | 0 | 0 | 0 | 0 | 0 |
| 760 | 0.00001 | 0 | 0.947623 | 0.052367 | 0 | 0 | 0 | 0 | 0 |
| 761 | 0.00003 | 0 | 0.943698 | 0.056272 | 0 | 0 | 0 | 0 | 0 |
| 762 | 0.00004 | 0 | 0.945955 | 0.054005 | 0 | 0 | 0 | 0 | 0 |
| 763 | 0 | 0 | 0.946865 | 0.053135 | 0 | 0 | 0 | 0 | 0 |
| 764 | 0.00001 | 0 | 0.947721 | 0.052269 | 0 | 0 | 0 | 0 | 0 |
| 765 | 0.00002 | 0 | 0.942229 | 0.057751 | 0 | 0 | 0 | 0 | 0 |
| 766 | 0.00003 | 0 | 0.940039 | 0.059931 | 0 | 0 | 0 | 0 | 0 |
| 767 | 0.00004 | 0 | 0.947689 | 0.052271 | 0 | 0 | 0 | 0 | 0 |
| 768 | 0 | 0 | 0.9431 | 0.0569 | 0 | 0 | 0 | 0 | 0 |
| 769 | 0.00001 | 0 | 0.942992 | 0.056998 | 0 | 0 | 0 | 0 | 0 |
| 770 | 0.00001 | 0 | 0.944601 | 0.055389 | 0 | 0 | 0 | 0 | 0 |
| 771 | 0.00003 | 0 | 0.940431 | 0.054586 | 0.004953 | 0 | 0 | 0 | 0 |
| 772 | 0.00001 | 0 | 0.944068 | 0.055922 | 0 | 0 | 0 | 0 | 0 |
| 773 | 0.00001 | 0 | 0.940651 | 0.059339 | 0 | 0 | 0 | 0 | 0 |
| 774 | 0.00003 | 0 | 0.945612 | 0.054358 | 0 | 0 | 0 | 0 | 0 |
| 775 | 0 | 0 | 0.945777 | 0.054223 | 0 | 0 | 0 | 0 | 0 |
| 776 | 0.00001 | 0 | 0.94552 | 0.05447 | 0 | 0 | 0 | 0 | 0 |
| 777 | 0.00001 | 0 | 0.94399 | 0.056 | 0 | 0 | 0 | 0 | 0 |
| 778 | 0 | 0 | 0.943841 | 0.056159 | 0 | 0 | 0 | 0 | 0 |
| 779 | 0.00001 | 0 | 0.949784 | 0.050206 | 0 | 0 | 0 | 0 | 0 |
| 780 | 0.00001 | 0 | 0.942305 | 0.057685 | 0 | 0 | 0 | 0 | 0 |
| 781 | 0 | 0 | 0.943247 | 0.056753 | 0 | 0 | 0 | 0 | 0 |
| 782 | 0.00001 | 0 | 0.945187 | 0.054803 | 0 | 0 | 0 | 0 | 0 |
| 783 | 0.00002 | 0 | 0.942223 | 0.057757 | 0 | 0 | 0 | 0 | 0 |
| 784 | 0.00002 | 0 | 0.942629 | 0.057351 | 0 | 0 | 0 | 0 | 0 |
| 785 | 0.00001 | 0 | 0.945782 | 0.054208 | 0 | 0 | 0 | 0 | 0 |
| 786 | 0.00003 | 0 | 0.944885 | 0.055085 | 0 | 0 | 0 | 0 | 0 |
| 787 | 0 | 0 | 0.944059 | 0.055941 | 0 | 0 | 0 | 0 | 0 |
| 788 | 0.00002 | 0 | 0.946968 | 0.053012 | 0 | 0 | 0 | 0 | 0 |
| 789 | 0.00001 | 0 | 0.943261 | 0.056729 | 0 | 0 | 0 | 0 | 0 |
| 790 | 0.00002 | 0 | 0.942393 | 0.057587 | 0 | 0 | 0 | 0 | 0 |
| 791 | 0.00001 | 0 | 0.946751 | 0.053239 | 0 | 0 | 0 | 0 | 0 |
| 792 | 0.00001 | 0 | 0.946695 | 0.053295 | 0 | 0 | 0 | 0 | 0 |
| 793 | 0.00002 | 0 | 0.945188 | 0.054792 | 0 | 0 | 0 | 0 | 0 |
| 794 | 0.00001 | 0 | 0.946066 | 0.053924 | 0 | 0 | 0 | 0 | 0 |
| 795 | 0.00001 | 0 | 0.942608 | 0.057382 | 0 | 0 | 0 | 0 | 0 |
| 796 | 0.00001 | 0 | 0.94824 | 0.05175 | 0 | 0 | 0 | 0 | 0 |
| 797 | 0 | 0 | 0.946802 | 0.053198 | 0 | 0 | 0 | 0 | 0 |
| 798 | 0.00001 | 0 | 0.938753 | 0.058064 | 0.003173 | 0 | 0 | 0 | 0 |
| 799 | 0.00001 | 0 | 0.946326 | 0.053664 | 0 | 0 | 0 | 0 | 0 |
| 800 | 0.00001 | 0 | 0.942199 | 0.057791 | 0 | 0 | 0 | 0 | 0 |
| 801 | 0.00002 | 0 | 0.948301 | 0.051679 | 0 | 0 | 0 | 0 | 0 |
| 802 | 0.00004 | 0 | 0.94066 | 0.0593 | 0 | 0 | 0 | 0 | 0 |
| 803 | 0.00002 | 0 | 0.945917 | 0.054063 | 0 | 0 | 0 | 0 | 0 |
| 804 | 0 | 0 | 0.943957 | 0.056043 | 0 | 0 | 0 | 0 | 0 |
| 805 | 0.00003 | 0 | 0.946348 | 0.053623 | 0 | 0 | 0 | 0 | 0 |
| 806 | 0.00001 | 0 | 0.945835 | 0.054155 | 0 | 0 | 0 | 0 | 0 |
| 807 | 0 | 0 | 0.94772 | 0.05228 | 0 | 0 | 0 | 0 | 0 |
| 808 | 0 | 0 | 0.943463 | 0.056537 | 0 | 0 | 0 | 0 | 0 |
| 809 | 0 | 0 | 0.945722 | 0.054278 | 0 | 0 | 0 | 0 | 0 |
| 810 | 0.00001 | 0 | 0.942156 | 0.057834 | 0 | 0 | 0 | 0 | 0 |
| 811 | 0 | 0 | 0.944948 | 0.055052 | 0 | 0 | 0 | 0 | 0 |
| 812 | 0.00003 | 0 | 0.944888 | 0.055081 | 0 | 0 | 0 | 0 | 0 |
| 813 | 0.00002 | 0 | 0.945319 | 0.054661 | 0 | 0 | 0 | 0 | 0 |
| 814 | 0.00002 | 0 | 0.943283 | 0.056697 | 0 | 0 | 0 | 0 | 0 |
| 815 | 0 | 0 | 0.944952 | 0.055048 | 0 | 0 | 0 | 0 | 0 |
| 816 | 0 | 0 | 0.944084 | 0.055916 | 0 | 0 | 0 | 0 | 0 |
| 817 | 0.00001 | 0 | 0.947391 | 0.052599 | 0 | 0 | 0 | 0 | 0 |
| 818 | 0.00001 | 0 | 0.940433 | 0.059557 | 0 | 0 | 0 | 0 | 0 |
| 819 | 0.00002 | 0 | 0.94252 | 0.05746 | 0 | 0 | 0 | 0 | 0 |
| 820 | 0.00001 | 0 | 0.943031 | 0.056959 | 0 | 0 | 0 | 0 | 0 |
| 821 | 0.00001 | 0 | 0.946864 | 0.053127 | 0 | 0 | 0 | 0 | 0 |
| 822 | 0.00001 | 0 | 0.945662 | 0.054328 | 0 | 0 | 0 | 0 | 0 |
| 823 | 0.00001 | 0 | 0.943315 | 0.056675 | 0 | 0 | 0 | 0 | 0 |
| 824 | 0.00001 | 0 | 0.948955 | 0.051035 | 0 | 0 | 0 | 0 | 0 |
| 825 | 0.00003 | 0 | 0.947072 | 0.052898 | 0 | 0 | 0 | 0 | 0 |
| 826 | 0.00001 | 0 | 0.945996 | 0.053994 | 0 | 0 | 0 | 0 | 0 |
| 827 | 0.00002 | 0 | 0.943638 | 0.056342 | 0 | 0 | 0 | 0 | 0 |
| 828 | 0 | 0 | 0.944578 | 0.055422 | 0 | 0 | 0 | 0 | 0 |
| 829 | 0.00002 | 0 | 0.940494 | 0.059486 | 0 | 0 | 0 | 0 | 0 |
| 830 | 0.00002 | 0 | 0.945249 | 0.054731 | 0 | 0 | 0 | 0 | 0 |
| 831 | 0 | 0 | 0.940545 | 0.059455 | 0 | 0 | 0 | 0 | 0 |
| 832 | 0.00001 | 0 | 0.946916 | 0.053074 | 0 | 0 | 0 | 0 | 0 |
| 833 | 0 | 0 | 0.947079 | 0.052921 | 0 | 0 | 0 | 0 | 0 |
| 834 | 0 | 0 | 0.942555 | 0.057445 | 0 | 0 | 0 | 0 | 0 |
| 835 | 0.00002 | 0 | 0.947573 | 0.052407 | 0 | 0 | 0 | 0 | 0 |
| 836 | 0.00001 | 0 | 0.949321 | 0.050669 | 0 | 0 | 0 | 0 | 0 |
| 837 | 0.00002 | 0 | 0.944851 | 0.055129 | 0 | 0 | 0 | 0 | 0 |
| 838 | 0 | 0 | 0.944859 | 0.055141 | 0 | 0 | 0 | 0 | 0 |
| 839 | 0.00002 | 0 | 0.943729 | 0.056251 | 0 | 0 | 0 | 0 | 0 |
| 840 | 0.00001 | 0 | 0.943537 | 0.056453 | 0 | 0 | 0 | 0 | 0 |
| 841 | 0.00001 | 0 | 0.945175 | 0.054815 | 0 | 0 | 0 | 0 | 0 |
| 842 | 0.00004 | 0 | 0.945126 | 0.054834 | 0 | 0 | 0 | 0 | 0 |
| 843 | 0.00001 | 0 | 0.943772 | 0.056218 | 0 | 0 | 0 | 0 | 0 |
| 844 | 0.00001 | 0 | 0.944865 | 0.055126 | 0 | 0 | 0 | 0 | 0 |
| 845 | 0 | 0 | 0.943104 | 0.056896 | 0 | 0 | 0 | 0 | 0 |
| 846 | 0.00001 | 0 | 0.980744 | 0.019246 | 0 | 0 | 0 | 0 | 0 |
| 847 | 0 | 0 | 0.941765 | 0.058235 | 0 | 0 | 0 | 0 | 0 |
| 848 | 0.00001 | 0 | 0.945777 | 0.054213 | 0 | 0 | 0 | 0 | 0 |
| 849 | 0.00002 | 0 | 0.949129 | 0.050851 | 0 | 0 | 0 | 0 | 0 |
| 850 | 0 | 0 | 0.94499 | 0.05501 | 0 | 0 | 0 | 0 | 0 |
| 851 | 0.00003 | 0 | 0.946275 | 0.053695 | 0 | 0 | 0 | 0 | 0 |
| 852 | 0.00002 | 0 | 0.94293 | 0.05705 | 0 | 0 | 0 | 0 | 0 |
| 853 | 0.00001 | 0 | 0.942266 | 0.057724 | 0 | 0 | 0 | 0 | 0 |
| 854 | 0 | 0 | 0.943323 | 0.056677 | 0 | 0 | 0 | 0 | 0 |
| 855 | 0.00003 | 0 | 0.942366 | 0.057604 | 0 | 0 | 0 | 0 | 0 |
| 856 | 0.00004 | 0 | 0.94232 | 0.05764 | 0 | 0 | 0 | 0 | 0 |
| 857 | 0 | 0 | 0.944353 | 0.055647 | 0 | 0 | 0 | 0 | 0 |
| 858 | 0.00001 | 0 | 0.948344 | 0.051646 | 0 | 0 | 0 | 0 | 0 |
| 859 | 0 | 0 | 0.945137 | 0.054863 | 0 | 0 | 0 | 0 | 0 |
| 860 | 0.00001 | 0 | 0.941967 | 0.058023 | 0 | 0 | 0 | 0 | 0 |
| 861 | 0 | 0 | 0.939579 | 0.054149 | 0.006271 | 0 | 0 | 0 | 0 |
| 862 | 0.00001 | 0 | 0.946387 | 0.053603 | 0 | 0 | 0 | 0 | 0 |
| 863 | 0.00002 | 0 | 0.941412 | 0.058568 | 0 | 0 | 0 | 0 | 0 |
| 864 | 0.00003 | 0 | 0.948622 | 0.051348 | 0 | 0 | 0 | 0 | 0 |
| 865 | 0.00002 | 0 | 0.943421 | 0.056559 | 0 | 0 | 0 | 0 | 0 |
| 866 | 0.00003 | 0 | 0.944936 | 0.055034 | 0 | 0 | 0 | 0 | 0 |
| 867 | 0 | 0 | 0.945722 | 0.054278 | 0 | 0 | 0 | 0 | 0 |
| 868 | 0.00001 | 0 | 0.940775 | 0.059215 | 0 | 0 | 0 | 0 | 0 |
| 869 | 0 | 0 | 0.944873 | 0.055127 | 0 | 0 | 0 | 0 | 0 |
| 870 | 0.00002 | 0 | 0.943911 | 0.056069 | 0 | 0 | 0 | 0 | 0 |
| 871 | 0.00001 | 0 | 0.947889 | 0.052101 | 0 | 0 | 0 | 0 | 0 |
| 872 | 0.00001 | 0 | 0.944176 | 0.055814 | 0 | 0 | 0 | 0 | 0 |
| 873 | 0.00001 | 0 | 0.94237 | 0.05762 | 0 | 0 | 0 | 0 | 0 |
| 874 | 0.00001 | 0 | 0.945487 | 0.054503 | 0 | 0 | 0 | 0 | 0 |
| 875 | 0 | 0 | 0.946503 | 0.053497 | 0 | 0 | 0 | 0 | 0 |
| 876 | 0.00002 | 0 | 0.945197 | 0.054783 | 0 | 0 | 0 | 0 | 0 |
| 877 | 0.00001 | 0 | 0.949203 | 0.050786 | 0 | 0 | 0 | 0 | 0 |
| 878 | 0 | 0 | 0.948023 | 0.051977 | 0 | 0 | 0 | 0 | 0 |
| 879 | 0 | 0 | 0.943589 | 0.056411 | 0 | 0 | 0 | 0 | 0 |
| 880 | 0 | 0 | 0.94185 | 0.05815 | 0 | 0 | 0 | 0 | 0 |
| 881 | 0 | 0 | 0.943596 | 0.056404 | 0 | 0 | 0 | 0 | 0 |
| 882 | 0 | 0 | 0.940845 | 0.059155 | 0 | 0 | 0 | 0 | 0 |
| 883 | 0.00001 | 0 | 0.946221 | 0.053768 | 0 | 0 | 0 | 0 | 0 |
| 884 | 0.00001 | 0 | 0.94314 | 0.05685 | 0 | 0 | 0 | 0 | 0 |
| 885 | 0.00001 | 0 | 0.948173 | 0.051817 | 0 | 0 | 0 | 0 | 0 |
| 886 | 0 | 0 | 0.942889 | 0.057111 | 0 | 0 | 0 | 0 | 0 |
| 887 | 0 | 0 | 0.944642 | 0.055358 | 0 | 0 | 0 | 0 | 0 |
| 888 | 0.00003 | 0 | 0.941015 | 0.058955 | 0 | 0 | 0 | 0 | 0 |
| 889 | 0.00001 | 0 | 0.939501 | 0.060489 | 0 | 0 | 0 | 0 | 0 |
| 890 | 0 | 0 | 0.945085 | 0.054915 | 0 | 0 | 0 | 0 | 0 |
| 891 | 0.00001 | 0 | 0.943428 | 0.056562 | 0 | 0 | 0 | 0 | 0 |
| 892 | 0.00003 | 0 | 0.946682 | 0.053288 | 0 | 0 | 0 | 0 | 0 |
| 893 | 0.00001 | 0 | 0.944321 | 0.055669 | 0 | 0 | 0 | 0 | 0 |
| 894 | 0.00002 | 0 | 0.939363 | 0.060617 | 0 | 0 | 0 | 0 | 0 |
| 895 | 0.00003 | 0 | 0.948934 | 0.051035 | 0 | 0 | 0 | 0 | 0 |
| 896 | 0.00002 | 0 | 0.94381 | 0.056171 | 0 | 0 | 0 | 0 | 0 |
| 897 | 0.00001 | 0 | 0.947638 | 0.052352 | 0 | 0 | 0 | 0 | 0 |
| 898 | 0.00001 | 0 | 0.945768 | 0.054222 | 0 | 0 | 0 | 0 | 0 |
| 899 | 0 | 0 | 0.948151 | 0.051849 | 0 | 0 | 0 | 0 | 0 |
| 900 | 0 | 0 | 0.94435 | 0.05565 | 0 | 0 | 0 | 0 | 0 |
| 901 | 0 | 0 | 0.948093 | 0.051907 | 0 | 0 | 0 | 0 | 0 |
| 902 | 0 | 0 | 0.943824 | 0.056176 | 0 | 0 | 0 | 0 | 0 |
| 903 | 0 | 0 | 0.944005 | 0.055995 | 0 | 0 | 0 | 0 | 0 |
| 904 | 0.00001 | 0 | 0.947942 | 0.052048 | 0 | 0 | 0 | 0 | 0 |
| 905 | 0.00001 | 0 | 0.945027 | 0.054962 | 0 | 0 | 0 | 0 | 0 |
| 906 | 0.00001 | 0 | 0.943732 | 0.056258 | 0 | 0 | 0 | 0 | 0 |
| 907 | 0.00001 | 0 | 0.950124 | 0.049866 | 0 | 0 | 0 | 0 | 0 |
| 908 | 0.00003 | 0 | 0.943448 | 0.056522 | 0 | 0 | 0 | 0 | 0 |
| 909 | 0 | 0 | 0.942386 | 0.057614 | 0 | 0 | 0 | 0 | 0 |
| 910 | 0 | 0 | 0.94501 | 0.05499 | 0 | 0 | 0 | 0 | 0 |
| 911 | 0.00001 | 0 | 0.945061 | 0.054929 | 0 | 0 | 0 | 0 | 0 |
| 912 | 0.00003 | 0 | 0.942091 | 0.057879 | 0 | 0 | 0 | 0 | 0 |
| 913 | 0.00001 | 0 | 0.942433 | 0.057557 | 0 | 0 | 0 | 0 | 0 |
| 914 | 0.00002 | 0 | 0.946399 | 0.053581 | 0 | 0 | 0 | 0 | 0 |
| 915 | 0.00002 | 0 | 0.940693 | 0.059287 | 0 | 0 | 0 | 0 | 0 |
| 916 | 0 | 0 | 0.943338 | 0.056662 | 0 | 0 | 0 | 0 | 0 |
| 917 | 0 | 0 | 0.945002 | 0.054998 | 0 | 0 | 0 | 0 | 0 |
| 918 | 0.00002 | 0 | 0.948162 | 0.051818 | 0 | 0 | 0 | 0 | 0 |
| 919 | 0 | 0 | 0.942931 | 0.057069 | 0 | 0 | 0 | 0 | 0 |
| 920 | 0.00001 | 0 | 0.948621 | 0.051369 | 0 | 0 | 0 | 0 | 0 |
| 921 | 0.00002 | 0 | 0.944841 | 0.055139 | 0 | 0 | 0 | 0 | 0 |
| 922 | 0.00001 | 0 | 0.946388 | 0.053602 | 0 | 0 | 0 | 0 | 0 |
| 923 | 0 | 0 | 0.945082 | 0.054918 | 0 | 0 | 0 | 0 | 0 |
| 924 | 0.00001 | 0 | 0.946925 | 0.053065 | 0 | 0 | 0 | 0 | 0 |
| 925 | 0.00001 | 0 | 0.945318 | 0.054672 | 0 | 0 | 0 | 0 | 0 |
| 926 | 0.00001 | 0 | 0.947054 | 0.052936 | 0 | 0 | 0 | 0 | 0 |
| 927 | 0.00002 | 0 | 0.942488 | 0.057492 | 0 | 0 | 0 | 0 | 0 |
| 928 | 0.00001 | 0 | 0.944158 | 0.055832 | 0 | 0 | 0 | 0 | 0 |
| 929 | 0.00001 | 0 | 0.937692 | 0.062298 | 0 | 0 | 0 | 0 | 0 |
| 930 | 0.00003 | 0 | 0.947097 | 0.052873 | 0 | 0 | 0 | 0 | 0 |
| 931 | 0.00001 | 0 | 0.94813 | 0.05186 | 0 | 0 | 0 | 0 | 0 |
| 932 | 0.00002 | 0 | 0.948082 | 0.051898 | 0 | 0 | 0 | 0 | 0 |
| 933 | 0.00002 | 0 | 0.948615 | 0.051365 | 0 | 0 | 0 | 0 | 0 |
| 934 | 0.00001 | 0 | 0.940208 | 0.059782 | 0 | 0 | 0 | 0 | 0 |
| 935 | 0.00001 | 0 | 0.947544 | 0.052446 | 0 | 0 | 0 | 0 | 0 |
| 936 | 0.00003 | 0 | 0.946181 | 0.053789 | 0 | 0 | 0 | 0 | 0 |
| 937 | 0.00002 | 0 | 0.944899 | 0.055081 | 0 | 0 | 0 | 0 | 0 |
| 938 | 0.00001 | 0 | 0.948898 | 0.051092 | 0 | 0 | 0 | 0 | 0 |
| 939 | 0.00002 | 0 | 0.948584 | 0.051396 | 0 | 0 | 0 | 0 | 0 |
| 940 | 0.00004 | 0 | 0.944257 | 0.055703 | 0 | 0 | 0 | 0 | 0 |
| 941 | 0 | 0 | 0.943338 | 0.056662 | 0 | 0 | 0 | 0 | 0 |
| 942 | 0.00001 | 0 | 0.944519 | 0.055471 | 0 | 0 | 0 | 0 | 0 |
| 943 | 0.00002 | 0 | 0.946457 | 0.053523 | 0 | 0 | 0 | 0 | 0 |
| 944 | 0.00003 | 0 | 0.94617 | 0.0538 | 0 | 0 | 0 | 0 | 0 |
| 945 | 0 | 0 | 0.941787 | 0.058213 | 0 | 0 | 0 | 0 | 0 |
| 946 | 0.00001 | 0 | 0.942629 | 0.057361 | 0 | 0 | 0 | 0 | 0 |
| 947 | 0.00001 | 0 | 0.944322 | 0.055668 | 0 | 0 | 0 | 0 | 0 |
| 948 | 0.00001 | 0 | 0.943949 | 0.056041 | 0 | 0 | 0 | 0 | 0 |
| 949 | 0.00001 | 0 | 0.946205 | 0.053785 | 0 | 0 | 0 | 0 | 0 |
| 950 | 0.00002 | 0 | 0.948595 | 0.051385 | 0 | 0 | 0 | 0 | 0 |
| 951 | 0.00001 | 0 | 0.949852 | 0.050138 | 0 | 0 | 0 | 0 | 0 |
| 952 | 0 | 0 | 0.946942 | 0.053058 | 0 | 0 | 0 | 0 | 0 |
| 953 | 0.00002 | 0 | 0.9447 | 0.05528 | 0 | 0 | 0 | 0 | 0 |
| 954 | 0.00001 | 0 | 0.941444 | 0.058546 | 0 | 0 | 0 | 0 | 0 |
| 955 | 0.00001 | 0 | 0.946448 | 0.053542 | 0 | 0 | 0 | 0 | 0 |
| 956 | 0 | 0 | 0.948136 | 0.051864 | 0 | 0 | 0 | 0 | 0 |
| 957 | 0.00003 | 0 | 0.943075 | 0.056895 | 0 | 0 | 0 | 0 | 0 |
| 958 | 0 | 0 | 0.947526 | 0.052474 | 0 | 0 | 0 | 0 | 0 |
| 959 | 0 | 0 | 0.941439 | 0.058561 | 0 | 0 | 0 | 0 | 0 |
| 960 | 0 | 0 | 0.946562 | 0.053438 | 0 | 0 | 0 | 0 | 0 |
| 961 | 0.00002 | 0 | 0.943131 | 0.056849 | 0 | 0 | 0 | 0 | 0 |
| 962 | 0.00001 | 0 | 0.946524 | 0.053466 | 0 | 0 | 0 | 0 | 0 |
| 963 | 0.00002 | 0 | 0.942136 | 0.057844 | 0 | 0 | 0 | 0 | 0 |
| 964 | 0 | 0 | 0.945918 | 0.054082 | 0 | 0 | 0 | 0 | 0 |
| 965 | 0.00003 | 0 | 0.948893 | 0.051078 | 0 | 0 | 0 | 0 | 0 |
| 966 | 0.00002 | 0 | 0.94621 | 0.05377 | 0 | 0 | 0 | 0 | 0 |
| 967 | 0 | 0 | 0.949631 | 0.050369 | 0 | 0 | 0 | 0 | 0 |
| 968 | 0.00002 | 0 | 0.944054 | 0.055926 | 0 | 0 | 0 | 0 | 0 |
| 969 | 0.00001 | 0 | 0.942509 | 0.057481 | 0 | 0 | 0 | 0 | 0 |
| 970 | 0 | 0 | 0.943016 | 0.056984 | 0 | 0 | 0 | 0 | 0 |
| 971 | 0 | 0 | 0.94509 | 0.05491 | 0 | 0 | 0 | 0 | 0 |
| 972 | 0 | 0 | 0.944868 | 0.055132 | 0 | 0 | 0 | 0 | 0 |
| 973 | 0.00001 | 0 | 0.946303 | 0.053687 | 0 | 0 | 0 | 0 | 0 |
| 974 | 0 | 0 | 0.941586 | 0.058414 | 0 | 0 | 0 | 0 | 0 |
| 975 | 0.00003 | 0 | 0.949894 | 0.050076 | 0 | 0 | 0 | 0 | 0 |
| 976 | 0.00002 | 0 | 0.945688 | 0.054292 | 0 | 0 | 0 | 0 | 0 |
| 977 | 0.00002 | 0 | 0.945079 | 0.054901 | 0 | 0 | 0 | 0 | 0 |
| 978 | 0.00001 | 0 | 0.946132 | 0.053859 | 0 | 0 | 0 | 0 | 0 |
| 979 | 0.00002 | 0 | 0.943793 | 0.056187 | 0 | 0 | 0 | 0 | 0 |
| 980 | 0.00001 | 0 | 0.946165 | 0.053825 | 0 | 0 | 0 | 0 | 0 |
| 981 | 0.00002 | 0 | 0.948331 | 0.051649 | 0 | 0 | 0 | 0 | 0 |
| 982 | 0.00001 | 0 | 0.940237 | 0.059753 | 0 | 0 | 0 | 0 | 0 |
| 983 | 0 | 0 | 0.941133 | 0.058867 | 0 | 0 | 0 | 0 | 0 |
| 984 | 0.00002 | 0 | 0.945677 | 0.054303 | 0 | 0 | 0 | 0 | 0 |
| 985 | 0 | 0 | 0.943855 | 0.056145 | 0 | 0 | 0 | 0 | 0 |
| 986 | 0.00002 | 0 | 0.947769 | 0.052211 | 0 | 0 | 0 | 0 | 0 |
| 987 | 0.00005 | 0 | 0.946079 | 0.053871 | 0 | 0 | 0 | 0 | 0 |
| 988 | 0.00001 | 0 | 0.944386 | 0.055604 | 0 | 0 | 0 | 0 | 0 |
| 989 | 0.00003 | 0 | 0.944379 | 0.055591 | 0 | 0 | 0 | 0 | 0 |
| 990 | 0.00001 | 0 | 0.942182 | 0.057808 | 0 | 0 | 0 | 0 | 0 |
| 991 | 0.00002 | 0 | 0.946211 | 0.053769 | 0 | 0 | 0 | 0 | 0 |
| 992 | 0.00001 | 0 | 0.945116 | 0.054874 | 0 | 0 | 0 | 0 | 0 |
| 993 | 0.00001 | 0 | 0.948317 | 0.051673 | 0 | 0 | 0 | 0 | 0 |
| 994 | 0.00001 | 0 | 0.949355 | 0.050635 | 0 | 0 | 0 | 0 | 0 |
| 995 | 0.00002 | 0 | 0.942395 | 0.057585 | 0 | 0 | 0 | 0 | 0 |
| 996 | 0.00001 | 0 | 0.947623 | 0.052367 | 0 | 0 | 0 | 0 | 0 |
| 997 | 0.00002 | 0 | 0.93986 | 0.06012 | 0 | 0 | 0 | 0 | 0 |
| 998 | 0.00001 | 0 | 0.943384 | 0.056606 | 0 | 0 | 0 | 0 | 0 |
| 999 | 0.00001 | 0 | 0.948563 | 0.051427 | 0 | 0 | 0 | 0 | 0 |
| 1000 | 0.00001 | 0 | 0.942794 | 0.057196 | 0 | 0 | 0 | 0 | 0 |
